# Supplementary material for: Potential Targeted Therapies in Ovarian Cancer
Source: Pharmaceuticals (Basel). 2022 Oct 26;15(11):1324. doi: 10.3390/ph15111324 (PMC9697427; doi:10.3390/ph15111324)
Supplement: Supplementary file 1 [file pharmaceuticals-15-01324-s001.zip › pharmaceuticals-1921877-supplementary.pdf]

Section S1. IonTorrent® Oncomine^{TM} Reports used to identify druggable targets

Druggable targets identified in *ATM, BRCA1/2, CDK12, CHEK1, EGFR, FGFR3* and *PIK3CA.*

Clinical trials and grading of evidence for the mutations are available in the reports.

The reports are from 25th November 2021.

Sample Cancer Type: Unknown Primary Origin

Table of Contents

Table of Contents	Page
Relevant Therapy Details	2
Clinical Trials	7
Relevant Therapy Summary	77
Clinical Trials Summary	83

Report Highlights

6 Relevant Biomarkers
1 Therapies Available
62 Clinical Trials

Relevant Biomarkers

Genomic Alteration	Relevant Therapies (In this cancer type)	Relevant Therapies (In other cancer type)	Clinical Trials
ATM mutation<br>**Prognostic significance:** None<br>**Diagnostic significance:** None	None	**olaparib** [1]	61
ATM splice site mutation<br>**Prognostic significance:** None<br>**Diagnostic significance:** None	None	**olaparib** [1]	61
ATM truncating mutation<br>**Prognostic significance:** None<br>**Diagnostic significance:** None	None	**olaparib** [1]	61
ATM deletion<br>**Prognostic significance:** None<br>**Diagnostic significance:** None	None	None	24
ATM fusion<br>**Prognostic significance:** None<br>**Diagnostic significance:** None	None	None	12
ATM aberration<br>**Prognostic significance:** None<br>**Diagnostic significance:** None	None	None	11

Public data sources included in relevant therapies: FDA[1], NCCN, **EMA**[2], ESMO
Public data sources included in prognostic and diagnostic significance: NCCN, ESMO

Disclaimer: The data presented here is from a curated knowledgebase of publicly available information, but may not be exhaustive. The data version is 2021.11(003).The content of this report has not been evaluated or approved by the FDA or other regulatory agencies.

Relevant Therapy Details

Current FDA Information

● In this cancer type ○ In other cancer type ◑ In this cancer type and other cancer types

FDA information is current as of 2021-10-13. For the most up-to-date information, search www.fda.gov.

ATM mutation

○ olaparib

Cancer type: Castration-Resistant Prostate Cancer **Label as of:** 2021-03-11 **Variant class:** ATM mutation

Indications and usage:

LYNPARZA® is a poly (ADP-ribose) polymerase (PARP) inhibitor indicated:

Ovarian cancer

- for the maintenance treatment of adult patients with deleterious or suspected deleterious germline or somatic BRCA-mutated advanced epithelial ovarian, fallopian tube or primary peritoneal cancer who are in complete or partial response to first-line platinum-based chemotherapy. Select patients for therapy based on an FDA-approved companion diagnostic for LYNPARZA®.
- in combination with bevacizumab for the maintenance treatment of adult patients with advanced epithelial ovarian, fallopian tube or primary peritoneal cancer who are in complete or partial response to first-line platinum-based chemotherapy and whose cancer is associated with homologous recombination deficiency (HRD)-positive status defined by either:
  - a deleterious or suspected deleterious BRCA mutation, and/or
  - genomic instability.

Select patients for therapy based on an FDA-approved companion diagnostic for LYNPARZA®.

- for the maintenance treatment of adult patients with recurrent epithelial ovarian, fallopian tube or primary peritoneal cancer, who are in complete or partial response to platinum-based chemotherapy.
- for the treatment of adult patients with deleterious or suspected deleterious germline BRCA-mutated (gBRCAm) advanced ovarian cancer who have been treated with three or more prior lines of chemotherapy. Select patients for therapy based on an FDA-approved companion diagnostic for LYNPARZA®.

Breast cancer

- for the treatment of adult patients with deleterious or suspected deleterious gBRCAm, HER2-negative metastatic breast cancer who have been treated with chemotherapy in the neoadjuvant, adjuvant or metastatic setting. Patients with hormone receptor (HR)-positive breast cancer should have been treated with a prior endocrine therapy or be considered inappropriate for endocrine therapy. Select patients for therapy based on an FDA-approved companion diagnostic for LYNPARZA®.

Pancreatic cancer

- for the maintenance treatment of adult patients with deleterious or suspected deleterious gBRCAm metastatic pancreatic adenocarcinoma whose disease has not progressed on at least 16 weeks of a first-line platinum-based chemotherapy regimen. Select patients for therapy based on an FDA-approved companion diagnostic for LYNPARZA®.

Prostate cancer

- for the treatment of adult patients with deleterious or suspected deleterious germline or somatic homologous recombination repair (HRR) gene-mutated metastatic castration-resistant prostate cancer (mCRPC) who have progressed following prior treatment with enzalutamide or abiraterone. Select patients for therapy based on an FDA-approved companion diagnostic for LYNPARZA®.

Reference:

https://www.accessdata.fda.gov/drugsatfda_docs/label/2021/208558s019s020lbl.pdf

Disclaimer: The data presented here is from a curated knowledgebase of publicly available information, but may not be exhaustive. The data version is 2021.11(003).

ATM splice site mutation

○ olaparib

Cancer type: Castration-Resistant Prostate Cancer **Label as of:** 2021-03-11 **Variant class:** ATM mutation

Indications and usage:

LYNPARZA® is a poly (ADP-ribose) polymerase (PARP) inhibitor indicated:

Ovarian cancer

- for the maintenance treatment of adult patients with deleterious or suspected deleterious germline or somatic BRCA-mutated advanced epithelial ovarian, fallopian tube or primary peritoneal cancer who are in complete or partial response to first-line platinum-based chemotherapy. Select patients for therapy based on an FDA-approved companion diagnostic for LYNPARZA®.
- in combination with bevacizumab for the maintenance treatment of adult patients with advanced epithelial ovarian, fallopian tube or primary peritoneal cancer who are in complete or partial response to first-line platinum-based chemotherapy and whose cancer is associated with homologous recombination deficiency (HRD)-positive status defined by either:
  - a deleterious or suspected deleterious BRCA mutation, and/or
  - genomic instability.

Select patients for therapy based on an FDA-approved companion diagnostic for LYNPARZA®.

- for the maintenance treatment of adult patients with recurrent epithelial ovarian, fallopian tube or primary peritoneal cancer, who are in complete or partial response to platinum-based chemotherapy.
- for the treatment of adult patients with deleterious or suspected deleterious germline BRCA-mutated (gBRCAm) advanced ovarian cancer who have been treated with three or more prior lines of chemotherapy. Select patients for therapy based on an FDA-approved companion diagnostic for LYNPARZA®.

Breast cancer

- for the treatment of adult patients with deleterious or suspected deleterious gBRCAm, HER2-negative metastatic breast cancer who have been treated with chemotherapy in the neoadjuvant, adjuvant or metastatic setting. Patients with hormone receptor (HR)-positive breast cancer should have been treated with a prior endocrine therapy or be considered inappropriate for endocrine therapy. Select patients for therapy based on an FDA-approved companion diagnostic for LYNPARZA®.

Pancreatic cancer

- for the maintenance treatment of adult patients with deleterious or suspected deleterious gBRCAm metastatic pancreatic adenocarcinoma whose disease has not progressed on at least 16 weeks of a first-line platinum-based chemotherapy regimen. Select patients for therapy based on an FDA-approved companion diagnostic for LYNPARZA®.

Prostate cancer

- for the treatment of adult patients with deleterious or suspected deleterious germline or somatic homologous recombination repair (HRR) gene-mutated metastatic castration-resistant prostate cancer (mCRPC) who have progressed following prior treatment with enzalutamide or abiraterone. Select patients for therapy based on an FDA-approved companion diagnostic for LYNPARZA®.

Reference:

https://www.accessdata.fda.gov/drugsatfda_docs/label/2021/208558s019s020lbl.pdf

Disclaimer: The data presented here is from a curated knowledgebase of publicly available information, but may not be exhaustive. The data version is 2021.11(003).

ATM truncating mutation

○ olaparib

Cancer type: Castration-Resistant Prostate Cancer **Label as of:** 2021-03-11 **Variant class:** ATM mutation

Indications and usage:

LYNPARZA® is a poly (ADP-ribose) polymerase (PARP) inhibitor indicated:

Ovarian cancer

- for the maintenance treatment of adult patients with deleterious or suspected deleterious germline or somatic BRCA-mutated advanced epithelial ovarian, fallopian tube or primary peritoneal cancer who are in complete or partial response to first-line platinum-based chemotherapy. Select patients for therapy based on an FDA-approved companion diagnostic for LYNPARZA®.
- in combination with bevacizumab for the maintenance treatment of adult patients with advanced epithelial ovarian, fallopian tube or primary peritoneal cancer who are in complete or partial response to first-line platinum-based chemotherapy and whose cancer is associated with homologous recombination deficiency (HRD)-positive status defined by either:
  - a deleterious or suspected deleterious BRCA mutation, and/or
  - genomic instability.

Select patients for therapy based on an FDA-approved companion diagnostic for LYNPARZA®.

- for the maintenance treatment of adult patients with recurrent epithelial ovarian, fallopian tube or primary peritoneal cancer, who are in complete or partial response to platinum-based chemotherapy.
- for the treatment of adult patients with deleterious or suspected deleterious germline BRCA-mutated (gBRCAm) advanced ovarian cancer who have been treated with three or more prior lines of chemotherapy. Select patients for therapy based on an FDA-approved companion diagnostic for LYNPARZA®.

Breast cancer

- for the treatment of adult patients with deleterious or suspected deleterious gBRCAm, HER2-negative metastatic breast cancer who have been treated with chemotherapy in the neoadjuvant, adjuvant or metastatic setting. Patients with hormone receptor (HR)-positive breast cancer should have been treated with a prior endocrine therapy or be considered inappropriate for endocrine therapy. Select patients for therapy based on an FDA-approved companion diagnostic for LYNPARZA®.

Pancreatic cancer

- for the maintenance treatment of adult patients with deleterious or suspected deleterious gBRCAm metastatic pancreatic adenocarcinoma whose disease has not progressed on at least 16 weeks of a first-line platinum-based chemotherapy regimen. Select patients for therapy based on an FDA-approved companion diagnostic for LYNPARZA®.

Prostate cancer

- for the treatment of adult patients with deleterious or suspected deleterious germline or somatic homologous recombination repair (HRR) gene-mutated metastatic castration-resistant prostate cancer (mCRPC) who have progressed following prior treatment with enzalutamide or abiraterone. Select patients for therapy based on an FDA-approved companion diagnostic for LYNPARZA®.

Reference:

https://www.accessdata.fda.gov/drugsatfda_docs/label/2021/208558s019s020lbl.pdf

Current NCCN Information

● In this cancer type ○ In other cancer type ◐ In this cancer type and other cancer types

NCCN information is current as of 2021-10-01. For the most up-to-date information, search www.nccn.org.
For NCCN International Adaptations & Translations, search www.nccn.org/global/international_adaptations.aspx.

ATM mutation

○ olaparib

Cancer type: Castration-Resistant Prostate Cancer **Variant class:** ATM mutation

NCCN Recommendation category: 1

Population segment (Line of therapy):
- Adenocarcinoma; Metastatic (Subsequent therapy); Useful in certain circumstances

Reference: NCCN Guidelines® - NCCN-Prostate Cancer [Version 1.2022]

○ olaparib

Cancer type: Castration-Resistant Prostate Cancer **Variant class:** ATM mutation

NCCN Recommendation category: 2B

Population segment (Line of therapy):
- Adenocarcinoma; Visceral Metastases (Subsequent therapy); Useful in certain circumstances

Reference: NCCN Guidelines® - NCCN-Prostate Cancer [Version 1.2022]

ATM splice site mutation

○ olaparib

Cancer type: Castration-Resistant Prostate Cancer **Variant class:** ATM mutation

NCCN Recommendation category: 1

Population segment (Line of therapy):
- Adenocarcinoma; Metastatic (Subsequent therapy); Useful in certain circumstances

Reference: NCCN Guidelines® - NCCN-Prostate Cancer [Version 1.2022]

○ olaparib

Cancer type: Castration-Resistant Prostate Cancer **Variant class:** ATM mutation

NCCN Recommendation category: 2B

Population segment (Line of therapy):
- Adenocarcinoma; Visceral Metastases (Subsequent therapy); Useful in certain circumstances

Reference: NCCN Guidelines® - NCCN-Prostate Cancer [Version 1.2022]

Disclaimer: The data presented here is from a curated knowledgebase of publicly available information, but may not be exhaustive. The data version is 2021.11(003).

ATM truncating mutation

○ olaparib

Cancer type: Castration-Resistant Prostate Cancer **Variant class:** ATM mutation

NCCN Recommendation category: 1

Population segment (Line of therapy):
- Adenocarcinoma; Metastatic (Subsequent therapy); Useful in certain circumstances

Reference: NCCN Guidelines® - NCCN-Prostate Cancer [Version 1.2022]

○ olaparib

Cancer type: Castration-Resistant Prostate Cancer **Variant class:** ATM mutation

NCCN Recommendation category: 2B

Population segment (Line of therapy):
- Adenocarcinoma; Visceral Metastases (Subsequent therapy); Useful in certain circumstances

Reference: NCCN Guidelines® - NCCN-Prostate Cancer [Version 1.2022]

Disclaimer: The data presented here is from a curated knowledgebase of publicly available information, but may not be exhaustive. The data version is 2021.11(003).

Current Clinical Trials Information

Clinical Trials information is current as of 2021-10-01. For the most up-to-date information regarding a particular trial, search www.clinicaltrials.gov by NCT ID or search local clinical trials authority website by local identifier listed in 'Other identifiers'.

ATM mutation + ATM splice site mutation + ATM truncating mutation + ATM deletion

NCT04276376

A Multicenter, Open Label, Phase II Basket Trial Exploring the Efficacy And Safety of The Combination of Rucaparib (PARP Inhibitor) And Atezolizumab (Anti-PD-L1 Antibody) In Patients With DNA Repair-Deficient or Platinum-Sensitive Solid Tumors

Cancer type: Bladder Urothelial Carcinoma, Non-Small Cell Lung Cancer, Prostate Cancer, Unspecified Solid Tumor

Variant classes: ATM deletion & ATM mutation

Other identifiers: 2018/2727, ARIANES, EudraCT Number: 2018-001744-62

Population segments: Second line, Stage III, Stage IV

Exclusion criteria variant classes: ATM deletion (Breast Cancer, Ovarian Cancer), ATM mutation (Breast Cancer, Ovarian Cancer), BARD1 deletion (Breast Cancer, Ovarian Cancer), BARD1 mutation (Breast Cancer, Ovarian Cancer), BRCA1 germline deletion (Breast Cancer, Ovarian Cancer), BRCA1 germline mutation (Breast Cancer, Ovarian Cancer), BRCA2 germline deletion (Breast Cancer, Ovarian Cancer), BRCA2 germline mutation (Breast Cancer, Ovarian Cancer), BRIP1 deletion (Breast Cancer, Ovarian Cancer), BRIP1 mutation (Breast Cancer, Ovarian Cancer), CDK12 deletion (Breast Cancer, Ovarian Cancer), CDK12 mutation (Breast Cancer, Ovarian Cancer), CHEK2 deletion (Breast Cancer, Ovarian Cancer), CHEK2 mutation (Breast Cancer, Ovarian Cancer), FANCA deletion (Breast Cancer, Ovarian Cancer), FANCA mutation (Breast Cancer, Ovarian Cancer), NBN deletion (Breast Cancer, Ovarian Cancer), NBN mutation (Breast Cancer, Ovarian Cancer), PALB2 deletion (Breast Cancer, Ovarian Cancer), PALB2 mutation (Breast Cancer, Ovarian Cancer), RAD51 deletion (Breast Cancer, Ovarian Cancer), RAD51 mutation (Breast Cancer, Ovarian Cancer), RAD51C deletion (Breast Cancer, Ovarian Cancer), RAD51C mutation (Breast Cancer, Ovarian Cancer), RAD51D deletion (Breast Cancer, Ovarian Cancer), RAD51D mutation (Breast Cancer, Ovarian Cancer), RAD54L deletion (Breast Cancer, Ovarian Cancer), RAD54L mutation (Breast Cancer, Ovarian Cancer)

Phase: II

Therapies: rucaparib, atezolizumab

Location: France

NCT04095273

A Multicenter, Non-randomized, Open-label Phase Ib Study to Determine the Maximum Tolerated and Recommended Phase II Dose of the ATR Inhibitor BAY1895344 in Combination With Pembrolizumab and to Characterize Its Safety, Tolerability, Pharmacokinetics and Preliminary Anti-tumor Activity in Patients With Advanced Solid Tumors

Cancer type: Breast Cancer, Castration-Resistant Prostate Cancer, Colorectal Cancer, Gastric Cancer, Gastroesophageal Junction Adenocarcinoma, Non-Small Cell Lung Cancer, Pancreatic Cancer

Variant classes: ATM deletion & ATM mutation

Other inclusion criteria: ERBB2 negative, Hormone receptor positive

Other identifiers: 19741, EudraCT Number: 2018-003420-36, Keynote 919, NCI-2019-06734

Population segments: HER2 negative, Hormone refractory, Second line, Stage III, Stage IV

Phase: I

Therapies: BAY-1895344, pembrolizumab

Locations: Germany, Spain, Switzerland, United States

US States: CA, CT, MA, MD, NC, NY, OH, TX

Contact: Bayer Clinical Trials Contact [clinical-trials-contact@bayer.com]

Disclaimer: The data presented here is from a curated knowledgebase of publicly available information, but may not be exhaustive. The data version is 2021.11(003).

ATM mutation + ATM splice site mutation + ATM truncating mutation + ATM deletion (continued)

NCT04126070
A Phase II Multicohort Study of Nivolumab in Combination With Docetaxel and Androgen Deprivation Therapy in Metastatic Hormone Sensitive Prostate Cancer Patients With DNA Damage Repair Defects or Inflamed Tumors

Cancer type: Prostate Cancer

Variant classes: DNA repair deletion & DNA repair mutation

Other identifiers: 19-384, NCI-2020-02149

Population segments: First line, Stage IV

Phase: II

Therapies: nivolumab, chemotherapy, hormone therapy

Location: United States

US State: MA

Contact: Dr. Xiao X. Wei [617-632-4524; xiaox_wei@dfci.harvard.edu]

ATM mutation

NCT03682289
Phase II Trial of AZD6738 Alone and in Combination With Olaparib in Patients With Selected Solid Tumor Malignancies

Cancer type: Prostate Cancer, Unspecified Solid Tumor

Variant class: ATM mutation

Other inclusion criteria: ATM underexpression

Other identifiers: 18-25513, 189510, NCI-2018-01648

Population segments: Second line, Stage III, Stage IV

Phase: II

Therapy: ceralasertib

Location: United States

US State: CA

Contact: Erika Zigman [877-837-3222; cancertrials@ucsf.edu]

NCT04564027
A Modular Phase IIa Multicentre Open-Label Study to Investigate DNA-damage Response Agents (or Combinations) in Patients With Advanced Cancer Whose Tumours Contain Molecular Alterations (PLANETTE)

Cancer type: Castration-Resistant Prostate Cancer, Unspecified Solid Tumor

Variant class: ATM mutation

Other identifiers: D5339C00001, EudraCT Number: 2020-002529-27, PLANETTE

Population segments: Hormone refractory, Second line, Stage III, Stage IV

Exclusion criteria variant class: ATM mutation (Non-Small Cell Lung Cancer)

Phase: II

Therapy: ceralasertib

Location: United States

US States: CA, FL, IN, MI, NV, PA, SC

Contact: AstraZeneca Clinical Study Information Center [877-240-9479; information.center@astrazeneca.com]

Disclaimer: The data presented here is from a curated knowledgebase of publicly available information, but may not be exhaustive. The data version is 2021.11(003).

ATM mutation (continued)

NCT02029001
A Two-period, Multicenter, Randomized, Open-label, Phase II Study Evaluating the Clinical Benefit of a Maintenance Treatment Targeting Tumor Molecular Alterations in Patients with Progressive Locally-advanced or Metastatic Solid Tumors.

Cancer type: Unspecified Solid Tumor

Variant class: ATM mutation

Other identifiers: ET12-081, EudraCT Number: 2012-004510-34, MOST, ProfiLER

Population segments: Maintenance/Consolidation, Second line, Stage III, Stage IV, Third line

Exclusion criteria variant classes: ATM mutation (Gastric Cancer), BRCA1 germline mutation (Gastric Cancer), BRCA1 mutation (Gastric Cancer), BRCA2 germline mutation (Gastric Cancer), BRCA2 mutation (Gastric Cancer), HRR mutation (Gastric Cancer), PALB2 mutation (Gastric Cancer), RAD51 mutation (Gastric Cancer)

Phase: II

Therapy: olaparib

Location: France

NCT03742895
A Phase II Study of Olaparib Monotherapy in Participants With Previously Treated, Homologous Recombination Repair Mutation (HRRm) or Homologous Recombination Deficiency (HRD) Positive Advanced Cancer

Cancer type: Unspecified Solid Tumor

Variant class: ATM mutation

Other identifiers: (MK-7339-002 / LYNK-002), 19-139, 194694, 2018-01891, 7339-002, 7339-002-00, EudraCT Number: 2018-003007-19, IRAS ID: 255158, JapicCTI-194694, LYNK002, LYNK-002, MK7339-002, MK-7339-002, MK7339-002-00, NCI-2018-03519, PER-046-18, SNCTP000003157

Population segments: BRCA, Fourth line or greater, Second line, Stage III, Stage IV, Third line

Exclusion criteria variant classes: BRCA germline mutation (Breast Cancer, Ovarian Cancer), BRCA mutation (Ovarian Cancer)

Phase: II

Therapy: olaparib

Locations: Argentina, Australia, Canada, Colombia, Denmark, France, Guatemala, Ireland, Israel, Italy, Mexico, Peru, Republic of Korea, Romania, Russian Federation, Spain, Switzerland, Turkey, United Kingdom, United States

US States: AZ, CA, CO, GA, KY, MA, MD, MI, NE, NJ, NY, SD, UT, WA

Contact: Toll Free Number [888-577-8839; Trialsites@merck.com]

NCT03967938
Efficacy of Olaparib in Advanced Cancers Occurring in Patients With Germline Mutations or Somatic Tumor Mutations in Homologous Recombination Genes

Cancer type: Unspecified Solid Tumor

Variant class: ATM mutation

Other identifiers: 1-2018 BSMO, 1-2018BSMO, EudraCT Number: 2018-002966-37, Precision 2 - Olaparib

Population segments: Line of therapy N/A, Stage III, Stage IV

Exclusion criteria variant classes: BRCA1 germline mutation (Breast Cancer, Pancreatic Cancer, Prostate Cancer), BRCA2 germline mutation (Breast Cancer, Pancreatic Cancer, Prostate Cancer)

Phase: II

Therapy: olaparib

Location: Belgium

ATM mutation (continued)

NCT02693535
Targeted Agent and Profiling Utilization Registry (TAPUR) Study.

Cancer type: Unspecified Solid Tumor

Variant class: ATM mutation

Other identifiers: 20170529, NCI-2017-00510, Pro00014171, TAPUR

Population segments: Aggressive, BRAF, Diffuse large B-cell lymphoma (DLBCL), Extranodal marginal zone B-cell lymphoma (MALT), FGFR, First line, Follicular lymphoma (FL), Fourth line or greater, Indolent, Lymphoblastic lymphoma (LBL), Mantle cell lymphoma (MCL), Other subtype, Second line, Small lymphocytic lymphoma (SLL), Stage III, Stage IV, Third line, Waldenstrom's macroglobulinemia (WM)

Phase: II

Therapies: olaparib, ipilimumab + nivolumab, atezolizumab + talazoparib

Location: United States

US States: AL, AZ, CA, FL, GA, HI, IL, IN, ME, MI, NC, ND, NE, NH, OH, OR, PA, SD, TX, UT, VA, WA

Contact: Pam Mangat [pam.mangat@asco.org]

NCT02286687
Phase II Study of the PARP Inhibitor Talazoparib in Advanced Cancer Patients With Somatic Alterations in BRCA1/2, Mutations/Deletions in Other BRCA Pathway Genes and Germline Mutation in BRCA1/2 (Not Breast or Ovarian Cancer)

Cancer type: Unspecified Cancer

Variant class: ATM mutation

Other identifiers: 2013-0961, NCI-2014-02494

Population segments: Fourth line or greater, Stage III, Stage IV

Exclusion criteria variant classes: BRCA1 germline mutation (Breast Cancer, Ovarian Cancer), BRCA2 germline mutation (Breast Cancer, Ovarian Cancer)

Phase: II

Therapy: talazoparib

Location: United States

US State: TX

Contact: Dr. Sarina Piha-Paul [713-563-1930; spihapau@mdanderson.org]

NCT02401347
A Phase II Clinical Trial of the PARP Inhibitor Talazoparib in BRCA1 and BRCA2 Wild Type Patients With Advanced Triple Negative Breast Cancer and Homologous Recombination Deficiency or Advanced HER2 Negative Breast Cancer or Other Solid Tumors With a Mutation in Homologous Recombination Pathway Genes Talazoparib Beyond BRCA (TBB) Trial

Cancer type: Breast Cancer, Unspecified Solid Tumor

Variant class: ATM mutation

Other inclusion criteria: ERBB2 negative, Hormone receptor positive

Other identifiers: BRS0050, NCI-2015-00036, TBB

Population segments: Estrogen receptor positive, HER2 negative, Progesterone receptor positive, Second line, Stage III, Stage IV, Triple receptor negative

Exclusion criteria variant classes: BRCA1 germline mutation (Breast Cancer, Triple Negative Breast Cancer, Unspecified Solid Tumor), BRCA1 mutation (Breast Cancer, Triple Negative Breast Cancer, Unspecified Solid Tumor), BRCA2 germline mutation (Breast Cancer, Triple Negative Breast Cancer, Unspecified Solid Tumor), BRCA2 mutation (Breast Cancer, Triple Negative Breast Cancer, Unspecified Solid Tumor), ERBB2 positive (Breast Cancer)

Phase: II

Therapy: talazoparib

Location: United States

US State: CA

Contact: Amanda Selin [650-721-6977; aselin@stanford.edu]

Disclaimer: The data presented here is from a curated knowledgebase of publicly available information, but may not be exhaustive. The data version is 2021.11(003).

ATM mutation (continued)

NCT04550494
A Pharmacodynamics-Driven Trial of Talazoparib, an Oral PARP Inhibitor, in Patients With Advanced Solid Tumors and Aberrations in Genes Involved in DNA Damage Response

Cancer type: Unspecified Solid Tumor

Variant class: ATM mutation

Other identifiers: 000246, 000264-C, 10000264, 10371, NCI-2020-06906

Population segments: BRCA, Fourth line or greater, HER2 positive, Hormone refractory, Second line, Stage II, Stage III, Stage IV, Third line

Phase: II

Therapy: talazoparib

Location: United States

US State: MD

Contact: Multiple contacts: See www.clinicaltrials.gov for complete list of contacts.

NCT03611868
A Phase Ib/II Study of APG-115 in Combination With Pembrolizumab in Patients With Unresectable or Metastatic Melanomas or Advanced Solid Tumors

Cancer type: Unspecified Solid Tumor

Variant class: ATM mutation

Other inclusion criteria: TP53 wild type

Other identifiers: 2020-0136, APG-115-US-002, Keynote MK-3475-B66, NCI-2019-08846

Population segments: Adenocarcinoma, Fourth line or greater, STK11, Second line, Stage III, Stage IV, Third line

Exclusion criteria variant class: FGFR fusion (Bladder Urothelial Carcinoma)

Phase: I/II

Therapies: alrizomadlin, pembrolizumab

Locations: Australia, United States

US States: AR, AZ, CA, DC, FL, MO, NY, PA, TN, TX, VA

Contact: Kathryn Shantz [301-802-3414; kate.shantz@ascentage.com]

NCT03842228
A Phase Ib Biomarker-Driven Combination Trial of Copanlisib, Olaparib, and Durvalumab (MEDI4736) in Patients With Advanced Solid Tumors

Cancer type: Unspecified Solid Tumor

Variant class: ATM mutation

Other identifiers: 10217, 19-628, NCI10217, NCI-2019-00601

Population segments: Line of therapy N/A, Stage III, Stage IV

Phase: I

Therapies: copanlisib, olaparib, durvalumab

Location: United States

US States: MA, TX

Contact: Multiple contacts: See www.clinicaltrials.gov for complete list of contacts.

Disclaimer: The data presented here is from a curated knowledgebase of publicly available information, but may not be exhaustive. The data version is 2021.11(003).

ATM mutation (continued)

NCT04586335

Open Label, Phase Ib Study to Evaluate the Safety, Tolerability, Pharmacokinetics and Clinical Activity of CYH33, an Oral a-specific PI3K Inhibitor in Combination With Olaparib, an Oral PARP Inhibitor in Patients With Advanced Solid Tumors.

Cancer type: Breast Cancer, Endometrial Carcinoma, Ovarian Cancer, Prostate Cancer, Unspecified Solid Tumor

Variant class: ATM mutation

Other identifiers: CTR20202339, CYH33-G102

Population segments: BRCA, Second line, Stage III, Stage IV

Phase: I

Therapies: HH-CYH33, olaparib

Locations: Australia, United States

US State: TX

Contact: Dr. Jason Sudia [908-380-1329; jason.sudia@haihepharma.com]

NCT04123366

A Phase II Study of Olaparib in Combination With Pembrolizumab in Participants With Previously Treated, Homologous Recombination Repair Mutation (HRRm) and/or Homologous Recombination Deficiency (HRD)-Positive Advanced Cancer

Cancer type: Unspecified Solid Tumor

Variant class: HRR mutation

Other identifiers: 7339-007, EudraCT Number: 2019-001745-40, jRCT2011200025, KEYLYNK-007, MK-7339-007, NCI-2019-08758, PER-029-19

Population segments: BRCA, Second line, Stage III, Stage IV

Exclusion criteria variant classes: BRCA germline mutation (Breast Cancer, Ovarian Cancer), BRCA mutation (Breast Cancer, Ovarian Cancer)

Phase: II

Therapies: olaparib, pembrolizumab

Locations: Argentina, Australia, Canada, Colombia, France, Germany, Guatemala, Israel, Italy, Japan, Latvia, Mexico, Peru, Poland, Puerto Rico, Republic of Korea, Romania, South Africa, Spain, Sweden, Turkey, Ukraine, United States

US States: AL, AZ, CA, CO, FL, GA, KY, NJ, NY, OH, TX, UT, VA

Contact: Toll Free Number [888-577-8839; Trialsites@merck.com]

NCT04497116

Phase I/IIa Study of the Safety, Pharmacokinetics, Pharmacodynamics and Preliminary Clinical Activity of RP-3500 Alone or in Combination With Talazoparib in Advanced Solid Tumors With ATR Inhibitor Sensitizing Mutations (TRESR Study)

Cancer type: Unspecified Solid Tumor

Variant class: ATM aberration

Other identifiers: 20-521, EudraCT Number: 2020-000301-87, IRAS ID: 286609, NCI-2020-06869, RP-3500-01, TRESR, TRESR Study

Population segments: Second line, Stage III, Stage IV

Phase: I/II

Therapies: RP-3500, talazoparib

Locations: Canada, Denmark, United Kingdom, United States

US States: MA, NC, NY, RI, TN, TX

Contact: Dr. Peter Manley [857-322-5553; pmanley@reparerx.com]

ATM mutation (continued)

NCT03415659
A Phase I, Open-label, Single-center, Single/Multiple-dose, Dose-escalation/ Dose-expansion Clinical Study on Tolerance and Pharmacokinetics of HWH340 Tablet in Patients With Advanced Solid Tumors

Cancer type: Unspecified Solid Tumor

Variant class: HRR mutation

Other inclusion criteria: BRCA1 germline mutation negative, BRCA1 mutation negative, BRCA2 germline mutation negative, BRCA2 mutation negative

Other identifiers: CTR20171649, HWH340-RFPA 20170821, RFPA 20170821

Population segments: BRCA, Fourth line or greater, HER2 negative, Second line, Stage III, Stage IV, Third line

Phase: I

Therapy: HWH-340

Location: China

NCT03767075
Basket of Baskets: A Modular, Open-label, Phase II, Multicentre Study To Evaluate Targeted Agents in Molecularly Selected Populations With Advanced Solid Tumours

Cancer type: Unspecified Solid Tumor

Variant class: DNA repair mutation

Other identifiers: (Basket of Baskets) (BoB), EudraCT number: 2017-005108-89, MO39164, NL65937.031.18, VHIO17002

Population segments: Second line, Stage III, Stage IV

Phase: II

Therapy: atezolizumab

Locations: France, Germany, Netherlands, Spain, Sweden, United Kingdom

---

NCT04905914
A Phase I/IIa, Open-Label, Safety, Pharmacokinetic, And Preliminary Efficacy Study Of Oral ATRN-119 In Patients With Advanced Solid Tumors

Cancer type: Unspecified Solid Tumor

Variant class: DNA repair mutation

Other identifier: AR-276-01

Population segments: Line of therapy N/A, Stage III, Stage IV

Phase: I/II

Therapy: ATRN-119

Location: United States

US State: MA

Contact: Robert Hasson [619-540-6253; rhasson@pacificlinkconsulting.com]

ATM mutation (continued)

NCT04901702
A Randomized Phase I/II Study of Talazoparib or Temozolomide in Combination With Onivyde in Children With Recurrent Solid Malignancies and Ewing Sarcoma

Cancer type: Unspecified Solid Tumor

Variant class: HRR pathway

Other identifiers: EudraCT Number: 2021-003570-31, ONITT

Population segments: Second line, Stage IV

Exclusion criteria variant classes: HRR germline pathway (Central Nervous System Neoplasm, Ewing Sarcoma), HRR pathway (Central Nervous System Neoplasm, Ewing Sarcoma)

Phase: I/II

Therapies: talazoparib, chemotherapy

Locations: Canada, United States

US State: TN

Contact: Dr. Sara Federico [866-278-5833; referralinfo@stjude.org]

NCT03155620
NCI-COG Pediatric MATCH (Molecular Analysis for Therapy Choice) Screening Protocol

Cancer type: Unspecified Solid Tumor

Variant class: DNA repair pathway

Other identifiers: 17-729, 2017-0625, 20170841, APEC1621SC, COGAPEC1621, COGAPEC1621SC, N 55017 SC, NCI-2017-01251, NCI-COG Pediatric MATCH, Pediatric MATCH

Population segments: ALK, Aggressive, BRAF, FGFR, HRAS, Indolent, KRAS, NRAS, Pediatric or Adolescent, Second line, Stage III, Stage IV

Phase: II

Therapy: olaparib

Locations: Puerto Rico, United States

US States: AK, AL, AR, AZ, CA, CO, CT, DC, DE, FL, GA, HI, IA, ID, IL, IN, KY, LA, MA, MD, ME, MI, MN, MO, MS, NC, NE, NH, NJ, NV, NY, OH, OK, OR, PA, RI, SC, SD, TN, TX, UT, VA, VT, WA, WI

Contact: Multiple contacts: See www.clinicaltrials.gov for complete list of contacts.

NCT03233204
NCI-COG Pediatric MATCH (Molecular Analysis for Therapy Choice)- Phase 2 Subprotocol of Olaparib in Patients With Tumors Harboring Defects in DNA Damage Repair Genes

Cancer type: Unspecified Solid Tumor

Variant class: DNA repair pathway

Other identifiers: 20170841, APEC1621H, NCI-2017-00766

Population segments: Aggressive, Indolent, Pediatric or Adolescent, Second line, Stage III, Stage IV

Phase: II

Therapy: olaparib

Locations: Puerto Rico, United States

US States: AK, AL, AR, AZ, CA, CO, DC, DE, FL, GA, IA, ID, IL, IN, KY, LA, MD, ME, MI, MN, MO, MS, NC, ND, NE, NJ, NY, OH, OK, OR, PA, SC, SD, TN, TX, UT, VA, VT, WA, WI

Contact: Multiple contacts: See www.clinicaltrials.gov for complete list of contacts.

ATM mutation (continued)

NCT04267939
An Open-label Phase Ib Study to Determine the Maximum Tolerated and/or Recommended Phase II Dose of the ATR Inhibitor BAY 1895344 in Combination With PARP Inhibitor Niraparib, in Patients With Recurrent Advanced Solid Tumors and Ovarian Cancer

Cancer type: Ovarian Cancer, Unspecified Solid Tumor

Variant class: DNA repair pathway

Other identifiers: 18595, 19-810, 2019-0471, 20-222, EudraCT Number: 2018-003930-34, NCI-2020-01405

Population segments: Maintenance/Consolidation, Second line, Stage III, Stage IV

Exclusion criteria variant class: DNA repair pathway (Prostate Cancer)

Phase: I

Therapies: BAY-1895344, niraparib

Location: United States

US States: MA, NY, TX

Contact: Bayer Clinical Trials Contact [888-842-2937; clinical-trials-contact@bayer.com]

NCT04693468
Modular Phase IB Hypothesis-Testing, Biomarker-Driven, Talazoparib Combination Trial (TalaCom)

Cancer type: Unspecified Solid Tumor

Variant class: DNA repair pathway

Other identifiers: 2020-0436, NCI-2020-06041, TalaCom

Population segments: Hormone refractory, Second line, Stage III, Stage IV

Phase: I

Therapies: talazoparib, palbociclib, axitinib, crizotinib

Location: United States

US State: TX

Contact: Timothy A. Yap [713-563-1784; tyap@mdanderson.org]

NCT02975934
TRITON3: A Multicenter, Randomized, Open Label Phase III Study of Rucaparib Versus Physician's Choice of Therapy for Patients With Metastatic Castration Resistant Prostate Cancer Associated With Homologous Recombination Deficiency

Cancer type: Castration-Resistant Prostate Cancer

Variant class: ATM mutation

Other identifiers: 16238, 1723GCCC, 18-146, 18-300, 20171185, 2018IS040, CO-338-063, CSET 2620, EudraCT Number: 2016-003163-20, GU 146, IGUP17020, IRAS ID: 222298, NCI-2017-01110, S16-02260, STAR Trial, TRITON 3_CO-338-063, TRITON3

Population segments: BRCA, First line, Hormone refractory, Stage IV

Phase: III

Therapies: rucaparib, hormone therapy

Locations: Australia, Belgium, Canada, Denmark, France, Germany, Ireland, Israel, Italy, Spain, United Kingdom, United States

US States: AZ, CA, CO, CT, DE, FL, HI, MD, MN, NC, NY, OH, OR, TN, TX, VA, WA

Contact: Clovis Oncology For North America [844-258-7662; medinfo@clovisoncology.com]

ATM mutation (continued)

NCT03810105
A Phase II Study of Olaparib and Durvalumab in Men With Castration Sensitive Biochemically Recurrent Non-Metastatic Prostate Cancer Harboring Mutations in DNA Damage Repair

Cancer type: Prostate Cancer

Variant class: ATM mutation

Other identifiers: 18-480, NCI-2019-01627

Population segments: Adjuvant, Stage I, Stage II, Stage III

Phase: II

Therapies: durvalumab, olaparib

Location: United States

US States: CA, IL, MI, NJ, NY

Contact: Dr. Karen Autio [646-422-4632; autiok@mskcc.org]

NCT04336943
Durvalumab (MEDI4736) and Olaparib (AZD2281) for Treatment of Biochemically Recurrent Prostate Cancer in Men Predicted to Have a High Neoantigen Load: A Pilot Study

Cancer type: Prostate Adenocarcinoma

Variant class: ATM mutation

Other identifiers: 10509, NCI-2020-01860, RG1007001

Population segments: Line of therapy N/A, Stage I, Stage II, Stage III

Phase: II

Therapies: durvalumab, olaparib

Location: United States

US State: WA

Contact: Michael Schweizer [206-606-6252; schweize@uw.edu]

NCT04053322
An International Multicenter Phase II Trial of Durvalumab (MEDI4736) Plus OLAparib Plus Fulvestrant in Metastatic or Locally Advanced ER-positive, HER2-negative Breast Cancer Patients Selected Using Criteria That Predict Sensitivity to Olaparib

Cancer type: Breast Cancer

Variant class: ATM mutation

Other inclusion criteria: ERBB2 negative, ER positive

Other identifiers: CADUSEIME08, DOLAF, EudraCT Number: 2018-003832-57, UC-0140/1812

Population segments: BRCA, Estrogen receptor positive, FGFR, HER2 negative, Progesterone receptor positive, Second line, Stage III, Stage IV

Phase: II

Therapies: durvalumab, olaparib, hormone therapy

Location: France

Disclaimer: The data presented here is from a curated knowledgebase of publicly available information, but may not be exhaustive. The data version is 2021.11(003).

ATM mutation (continued)

NCT05011383
High-dose Testosterone in Men With Metastatic Castration-resistant Prostate Cancer and ATM or CDK12 Deficiency

Cancer type: Castration-Resistant Prostate Cancer

Variant class: ATM mutation

Other identifiers: SPLP-003-20F, VA-BAT

Population segments: Hormone refractory, Second line, Stage IV

Phase: II

Therapy: hormone therapy

Location: United States

US State: WA

Contact: Dr. Robert B. Montgomery [206-277-6878; Robert.Montgomery@va.gov]

NCT03012321
BRCAAway: A Randomized Phase II Trial of Abiraterone, Olaparib, or Abiraterone + Olaparib in Patients With Metastatic Castration-Resistant Prostate Cancer With DNA Repair Defects

Cancer type: Castration-Resistant Prostate Cancer

Variant class: ATM mutation

Other identifiers: 19137, BRCAAway, NCI-2016-01834, NU 16U05, NU_16U05, PCCTC #: c16-168, PCCTC c16-168, STU00203960

Population segments: First line, Hormone refractory, Stage IV

Phase: II

Therapies: hormone therapy, olaparib, steroid

Location: United States

US States: CA, FL, IL, IN, MD, MI, MN, MO, NC, NY, UT, VA

Contact: Study Coordinator [312-695-1301; cancertrials@northwestern.edu]

NCT03553004
Niraparib in Metastatic Pancreatic Cancer After Previous Chemotherapy (NIRA-PANC): A Phase II Trial

Cancer type: Pancreatic Cancer

Variant class: ATM mutation

Other identifiers: IIT-2017-NIRA-PANC, NIRA-PANC, STUDY00141862

Population segments: BRCA, Second line, Stage IV, Third line

Phase: II

Therapy: niraparib

Location: United States

US States: KS, MO

Contact: Clinical Trials Nurse Navigator [913-588-3671; navigation@kumc.edu]

NCT03601923
Phase II Proof-of-Concept Trial Testing the PARP Inhibitor Niraparib in Patients With Pancreatic Cancer Harboring Deficiencies in Homologous Recombination DNA Repair

Cancer type: Pancreatic Cancer

Variant class: ATM mutation

Other identifiers: 18-207, NCI-2018-02395

Population segments: Second line, Stage IV

Phase: II

Therapy: niraparib

Location: United States

US State: MA

Contact: Dr. James Clreary [617-632-5960; JCLEARY@PARTNERS.ORG]

Disclaimer: The data presented here is from a curated knowledgebase of publicly available information, but may not be exhaustive. The data version is 2021.11(003).

ATM mutation (continued)

NCT03840967
A Phase II Study Evaluating Safety and Efficacy of Niraparib in Patients With Previously Treated Homologous Recombination (HR) Defective or Loss of Heterozygosity (LOH) High Metastatic Esophageal/Gastroesophageal Junction/ Proximal Gastric Adenocarcinoma

Cancer type: Esophageal Cancer, Gastric Cancer, Gastroesophageal Junction Adenocarcinoma

Variant class: ATM mutation

Other identifiers: 20166, 71618, HCRN ESO17-325, NCI-2019-07395

Population segments: Second line, Stage III, Stage IV

Phase: II

Therapy: niraparib

Location: United States

US States: FL, IN, MI, NY

Contact: Dr. Shadia Jalal [317-274-3658; sjalal@iu.edu]

NCT03925350
A Phase II Study of Niraparib in Patients With Advanced Melanoma With Genetic Homologous Recombination (HR) Mutation / Alteration

Cancer type: Melanoma

Variant class: ATM mutation

Other identifier: CPMC17-MEL01

Population segments: Second line, Stage III, Stage IV

Phase: II

Therapy: niraparib

Location: United States

US State: CA

Contact: Laurel Brechtel [415-600-1654; BrechtLA@sutterhealth.org]

NCT04030559
Phase II Trial of PARP Inhibitor Niraparib for Men With High Risk Prostate Cancer and DNA Damage Response Defects

Cancer type: Prostate Cancer

Variant class: ATM mutation

Other identifiers: 1412676, NCI-2019-04365, UCDCC#279

Population segments: First line, Neoadjuvant, Stage I, Stage II, Stage IV

Phase: II

Therapy: niraparib

Location: United States

US State: CA

Contact: Marc Dall'Era [916-734-3771; mdallera@ucdavis.edu]

NCT04187833
Phase II Trial of Nivolumab in Combination with Talazoparib in Patients with Unresectable or Metastatic Melanoma and Mutations in BRCA or BRCA-ness Genes.

Cancer type: Melanoma

Variant class: ATM mutation

Other identifiers: CASE2619, NCI-2020-00416

Population segments: Second line, Stage III, Stage IV

Phase: II

Therapies: nivolumab, talazoparib

Location: United States

US State: OH

Contact: Dr. Pauline Funchain [866-223-8100; TaussigResearch@ccf.org]

Disclaimer: The data presented here is from a curated knowledgebase of publicly available information, but may not be exhaustive. The data version is 2021.11(003).

ATM mutation (continued)

NCT03344965
A Phase II Study of Olaparib Monotherapy in Metastatic Breast Cancer Patients With Germline or Somatic Mutations in DNA Repair Genes (Olaparib Expanded)

Cancer type: Breast Cancer

Variant class: ATM mutation

Other identifiers: 17-428, 18-414, NCI-2018-00778, TBCRC 048, TBCRC048

Population segments: BRCA, Estrogen receptor positive, HER2 negative, HER2 positive, Progesterone receptor positive, Second line, Stage IV

Exclusion criteria variant classes: BRCA1 germline mutation (Breast Cancer), BRCA2 germline mutation (Breast Cancer)

Phase: II

Therapy: olaparib

Location: United States

US States: AL, CA, IL, MA, MD, NC, NJ, NY, PA, WA

Contact: Dr. Nadine Tung [617-667-1962; ntung@bidmc.harvard.edu]

NCT03375307
A Phase II Study of Olaparib (AZD2281) in Patients With Metastatic/Advanced Urothelial Carcinoma With DNA-Repair Defects

Cancer type: Bladder Urothelial Carcinoma

Variant class: ATM mutation

Other identifiers: 10144, 19C0023, NCI-2017-02296, P162941

Population segments: BRCA, STK11, Second line, Stage III, Stage IV

Phase: II

Therapy: olaparib

Location: United States

US States: CA, CO, KY, MD, NY, OK

Contact: Multiple contacts: See www.clinicaltrials.gov for complete list of contacts.

NCT03786796
Phase II Study of Olaparib in Metastatic Renal Cell Carcinoma Patients Harboring a BAP-1 or Other DNA Repair Gene Mutations (ORCHID)

Cancer type: Kidney Cancer

Variant class: ATM mutation

Other identifiers: CRMS-70750, J18166, NCI-2019-06309, ORCHID

Population segments: Fourth line or greater, Second line, Stage IV, Third line

Phase: II

Therapy: olaparib

Location: United States

US State: MD

Contact: Irina Rifkind [410-502-2043; irifkin1@jhmi.edu]

Disclaimer: The data presented here is from a curated knowledgebase of publicly available information, but may not be exhaustive. The data version is 2021.11(003).

ATM mutation (continued)

NCT04042831
A Phase II Study of Olaparib in Patients With Advanced Biliary Tract Cancer With Aberrant DNA Repair Gene Mutations

Cancer type: Biliary Tract Carcinoma

Variant class: ATM mutation

Other identifiers: ACCRU-ICRN-1702, NCI-2019-04434

Population segments: Second line, Stage IV

Phase: II

Therapy: olaparib

Location: United States

US States: AZ, MN, NY, TX

Contact: Multiple contacts: See www.clinicaltrials.gov for complete list of contacts.

NCT04515836
Phase II Trial of Olaparib in Homologous Recombination Deficient (HRD) Malignant Mesothelioma

Cancer type: Mesothelioma

Variant class: ATM mutation

Population segments: (N/A), Second line

Phase: II

Therapy: olaparib

Location: United States

US State: IL

Contact: Dr. Hedy L. Kindler [773-702-0360; hkindler@medicine.bsd.uchicago.edu]

NCT04633902
Phase II Study of Olaparib in Combination With Pembrolizumab in Patients With Advanced Melanoma With Homologous Recombination (HR) Pathway Gene Mutation

Cancer type: Melanoma

Variant class: ATM mutation

Other identifier: CPMC19-MEL01

Population segments: BRAF, Second line, Stage III, Stage IV

Phase: II

Therapies: olaparib, pembrolizumab

Location: United States

US State: CA

Contact: Dr. Kevin B. Kim [415-885-8600; ClinicalResearch@sutterhealth.org]

NCT03248570
Phase II Open Label Study of Pembrolizumab in Patients With Metastatic Castration Resistant Prostate Cancer (mCRPC) With or Without DNA Damage Repair Defects

Cancer type: Castration-Resistant Prostate Cancer

Variant class: ATM mutation

Other identifiers: 16557, 17-22165, CC 16557, NCI-2017-02408

Population segments: BRCA, Hormone refractory, Line of therapy N/A, Stage IV

Phase: II

Therapy: pembrolizumab

Location: United States

US State: CA

Contact: Patricia Li [415-476-5975; GUTrials@ucsf.edu]

Disclaimer: The data presented here is from a curated knowledgebase of publicly available information, but may not be exhaustive. The data version is 2021.11(003).

ATM mutation (continued)

NCT03506997
PERSEUS1: Phase II Trial of the Immune Checkpoint Inhibitor Pembrolizumab for Patients Suffering From Metastatic Prostate Cancer

Cancer type: Castration-Resistant Prostate Cancer

Variant class: ATM mutation

Other identifiers: CCR4559, EudraCT Number: 2017-000931-15, ICR-CTSU/2016/10060, IRAS ID 208952, PERSEUS1

Population segments: Hormone refractory, Second line, Stage IV

Phase: II

Therapy: pembrolizumab

Location: United Kingdom

NCT03533946
A Phase II Study of Rucaparib Monotherapy in Nonmetastatic, Hormone-Sensitive Prostate Cancer Demonstrating "BRCAness" Genotype (ROAR)

Cancer type: Prostate Cancer

Variant class: ATM mutation

Other identifiers: HCI111833, NCI-2018-01754, ROAR

Population segments: First line, Stage I, Stage II, Stage III

Phase: II

Therapy: rucaparib

Location: United States

US State: UT

Contact: Jill Broghammer [801-213-6232; jill.broghammer@hci.utah.edu]

NCT04892693
Talazoparib in Advanced Breast Cancer Patients With Homologous Recombinant Deficiency: A Phase II Clinical and Exploratory Biomarker Study of Talazoparib

Cancer type: Breast Cancer

Variant class: ATM mutation

Other identifier: H-2009-079-1157

Population segments: BRCA, Line of therapy N/A, Stage III, Stage IV

Phase: II

Therapy: talazoparib

Location: Republic of Korea

NCT04740190
Combination Talazoparib - Carboplatin for Recurrent High-grade Glioma With DNA Damage Repair Deficiency (DDRd)

Cancer type: Anaplastic Astrocytoma, Anaplastic Oligoastrocytoma, Anaplastic Oligodendroglioma, Anaplastic Pleomorphic Xanthoastrocytoma, Diffuse Midline Glioma, Glioblastoma

Variant class: ATM mutation

Other identifiers: TAC-GReD, UW-20396

Population segments: (N/A), BRCA, Second line

Phase: II

Therapies: talazoparib, chemotherapy

Location: Hong Kong

Disclaimer: The data presented here is from a curated knowledgebase of publicly available information, but may not be exhaustive. The data version is 2021.11(003).

ATM mutation (continued)

NCT04253262
A Phase Ib/II Study of Copanlisib Combined With Rucaparib in Patients With Metastatic Castration-resistant Prostate Cancer

Cancer type: Castration-Resistant Prostate Cancer

Variant class: ATM mutation

Other identifiers: BrUOG 360, BrUOG360

Population segments: Hormone refractory, Second line, Stage IV

Phase: I/II

Therapies: copanlisib, rucaparib

Location: United States

US State: RI

Contact: BrUOG [401-863-3000; BrUOG@brown.edu]

NCT04095273
A Multicenter, Non-randomized, Open-label Phase Ib Study to Determine the Maximum Tolerated and Recommended Phase II Dose of the ATR Inhibitor BAY1895344 in Combination With Pembrolizumab and to Characterize Its Safety, Tolerability, Pharmacokinetics and Preliminary Anti-tumor Activity in Patients With Advanced Solid Tumors

Cancer type: Breast Cancer

Variant class: ATM mutation

Other inclusion criteria: ERBB2 negative, Hormone receptor positive

Other identifiers: 19741, EudraCT Number: 2018-003420-36, Keynote 919, NCI-2019-06734

Population segments: HER2 negative, Hormone refractory, Second line, Stage III, Stage IV

Phase: I

Therapies: BAY-1895344, pembrolizumab

Locations: Germany, Spain, Switzerland, United States

US States: CA, CT, MA, MD, NC, NY, OH, TX

Contact: Bayer Clinical Trials Contact [clinical-trials-contact@bayer.com]

NCT04169841
Precision Medicine Phase II Study Evaluating the Efficacy of a Double Immunotherapy by Durvalumab and Tremelimumab Combined With Olaparib in Patients With Solid Cancers and Carriers of Homologous Recombination Repair Genes Mutation in Response or Stable After Olaparib Treatment

Cancer type: Breast Cancer, Endometrial Carcinoma, Head and Neck Cancer, Kidney Cancer, Non-Small Cell Lung Cancer, Ovarian Cancer, Pancreatic Cancer, Prostate Cancer

Variant class: HRR mutation

Other identifier: GUIDE2REPAIR

Population segments: Fourth line or greater, Hormone refractory, Maintenance/ Consolidation, Second line, Stage III, Stage IV, Third line

Exclusion criteria variant classes: ALK fusion (Lung Cancer), BRAF mutation (Lung Cancer), EGFR mutation (Lung Cancer), ROS1 mutation (Lung Cancer)

Phase: II

Therapies: durvalumab, tremelimumab, olaparib

Location: France

Disclaimer: The data presented here is from a curated knowledgebase of publicly available information, but may not be exhaustive. The data version is 2021.11(003).

ATM mutation (continued)

NCT04940637

A Phase II, Open-Label, Single Arm, Prospective, Multicenter Study of Niraparib Plus Dostarlimab in Patients With Advanced NSCLC and/or MPM, and Positive for PD-L1 Expression and Germline or Somatic Mutations in the HRR Genes

Cancer type: Mesothelioma, Non-Small Cell Lung Cancer

Variant class: HRR mutation

Other inclusion criteria: ALK fusion negative, CD274 expression, EGFRi sensitizing mutation negative, ROS1 fusion negative or CD274 expression

Other identifiers: EudraCT Number: 2020-000109-10, UNITO-001, UNITO-001/2020

Population segments: Second line, Stage III, Stage IV

Exclusion criteria variant classes: ALK fusion (Non-Small Cell Lung Cancer), EGFRi sensitizing mutation (Non-Small Cell Lung Cancer), ROS1 fusion (Non-Small Cell Lung Cancer)

Phase: II

Therapies: niraparib, dostarlimab

Location: Italy

NCT04939662

Phase II, Single-arm Study of Olaparib and Bevacizumab Combination Therapy in Relapsed Small Cell Lung Cancer Subjects With DNA Damage Response and the Repair Pathway Alteration, ATM Deficiency, SLFN11 Positive, or POU2F3 Positive

Cancer type: Small Cell Lung Cancer

Variant class: HRR mutation

Other identifier: SUKSES-B2

Population segments: (N/A), BRCA, Pulmonary, Second line, Third line

Phase: II

Therapies: olaparib, bevacizumab

Location: Republic of Korea

NCT04666740

A Phase II Trial to Evaluate the Safety and Antitumor Activity of Pembrolizumab and OLApaRib (POLAR) Maintenance for Patients With Metastatic Pancreatic Ductal Adenocarcinoma and Homologous Recombination Deficiency and/or Exceptional Treatment Response to Platinum-Based Therapy

Cancer type: Pancreatic Cancer

Variant class: ATM aberration

Other identifier: 20-481

Population segments: BRCA, Maintenance/Consolidation, Stage IV

Phase: II

Therapies: pembrolizumab, olaparib

Location: United States

US States: NJ, NY

Contact: Dr. Wungki Park [646-888-4543; yaqubiea@mskcc.org]

Disclaimer: The data presented here is from a curated knowledgebase of publicly available information, but may not be exhaustive. The data version is 2021.11(003).

ATM mutation (continued)

NCT02264678

A Modular Phase I, Open-Label, Multicentre Study to Assess the Safety, Tolerability, Pharmacokinetics and Preliminary Anti-tumour Activity of Ceralasertib in Combination With Cytotoxic Chemotherapy and/or DNA Damage Repair/Novel Anti-cancer Agents in Patients With Advanced Solid Malignancies

Cancer type: Triple Negative Breast Cancer

Variant class: HRR mutation

Other inclusion criteria: BRCA germline mutation negative, BRCA mutation negative

Other identifiers: 0C-14-9, 16-031, 18-342, D5330C00004, EudraCT Number: 2014-002233-66, IRAS ID 160200, NCI-2015-00687, Study 4

Population segments: Adenocarcinoma, BRCA, HER2 negative, Pulmonary, Second line, Stage III, Stage IV, Third line, Triple receptor negative

Exclusion criteria variant class: ERBB2 positive (Breast Cancer)

Phase: I

Therapies: ceralasertib, olaparib

Locations: France, Republic of Korea, United Kingdom, United States

US States: CA, MA, NY

Contact: AstraZeneca Clinical Study Information Center [877-240-9479; information.center@astrazeneca.com]

NCT04890613

Phase Ib Expansion Study of CX-5461 in Patients With Solid Tumours and BRCA2 and/or PALB2 Mutation

Cancer type: Ovarian Cancer

Variant class: HRR mutation

Other identifier: CX-5461-04

Population segments: BRCA, Second line, Stage II, Stage III, Stage IV

Phase: I

Therapy: pidnarulex

Location: Canada

NCT05002868

A Multi-center, Open-label, Phase I/Ib Study to Assess the Safety, Pharmacokinetics and Anti-tumor Activity of RP12146, a Poly (ADP-ribose) Polymerase (PARP) Inhibitor, in Patients With Locally Advanced or Metastatic Solid Tumors

Cancer type: Biliary Tract Carcinoma, Breast Cancer, Colorectal Cancer, Endometrial Carcinoma, Gastric Cancer, Ovarian Cancer, Pancreatic Cancer, Prostate Cancer, Small Cell Lung Cancer

Variant class: HRR mutation

Other identifier: RP12146-2101

Population segments: Extensive, Pulmonary, Second line, Stage III, Stage IV

Exclusion criteria variant class: ERBB2 positive (Breast Cancer)

Phase: I

Therapy: RP12146

Location: Poland

Disclaimer: The data presented here is from a curated knowledgebase of publicly available information, but may not be exhaustive. The data version is 2021.11(003).

ATM mutation (continued)

NCT03395197

A Phase III, Randomized, Double-blind, Placebo-controlled Study Of Talazoparib With Enzalutamide In Metastatic Castration-resistant Prostate Cancer

Cancer type: Castration-Resistant Prostate Cancer

Variant class: DNA repair mutation

Other identifiers: BASIC-ROYAL / HIGH-ROYAL, C3441021, CSET 2938, CTR20191443, CTR20191444, EudraCT Number: 2017-003295-31, GU 170 - C3441021, HCC 19-054, JapicCTI-194665, LH19.022, MOH_2019-05-16_007127, NCI-2018-01651, Pfizer C3441021, R19062M, RECF3951, RWF_C3441021 TALAPRO-2, TALAPRO-2, TX228665, UCI-18-47, VICCURO17115

Population segments: First line, Hormone refractory, Second line, Stage III, Stage IV

Phase: III

Therapies: talazoparib, hormone therapy

Locations: Argentina, Australia, Belgium, Brazil, Canada, Chile, China, Czech Republic, Finland, France, Germany, Hungary, Israel, Italy, Japan, New Zealand, Norway, Peru, Poland, Portugal, Republic of Korea, South Africa, Spain, Sweden, United Kingdom, United States

US States: AZ, CA, CO, FL, GA, IL, IN, KS, MO, NE, NJ, NY, OH, OK, OR, PA, SC, TN, TX, UT, VA, WI

Contact: Pfizer CT.gov Call Center [800-718-1021; ClinicalTrials.gov_Inquiries@pfizer.com]

NCT04821622

TALAPRO-3: A Phase III, Randomized, Double-blind, Study of Talazoparib With Enzalutamide Versus Placebo With Enzalutamide in Men With DDR Gene Mutated Metastatic Castration-sensitive Prostate Cancer

Cancer type: Prostate Adenocarcinoma

Variant class: DNA repair mutation

Other identifiers: C3441052, CTR20212191, EudraCT Number: 2021-000248-23, jRCT2031210098, TALAPRO-3

Population segments: Second line, Stage IV

Phase: III

Therapies: talazoparib, hormone therapy

Locations: Japan, Republic of Korea, United States

US States: CA, DC, FL, GA, IL, MI, NJ, NY, PA, SC, TX, UT

Contact: Pfizer CT.gov Call Center [800-718-1021; ClinicalTrials.gov_Inquiries@pfizer.com]

NCT04038502

An Open-label, Multicenter Phase II Study to Compare the Efficacy of Carboplatin as First-line Followed by Second-line Olaparib Versus Olaparib as First-line Followed by Second-line Carboplatin in the Treatment of Patients With Castration Resistant Prostate Cancer Containing Homologous Recombination Deficiency

Cancer type: Castration-Resistant Prostate Cancer

Variant class: DNA repair mutation

Other identifiers: 01781, 01783, 19-08, BRACeD, CX001963-01, ONCA-009-18F

Population segments: First line, Hormone refractory, Second line, Stage IV

Phase: II

Therapies: olaparib, chemotherapy

Location: United States

US States: CA, DC, FL, IL, MI, NC, NY, PA, WA

Contact: Dr. Robert B. Montgomery [206-277-6878; rbmontgo@u.washington.edu]

ATM mutation (continued)

No NCT ID - see other identifier(s)
Single-arm Exploratory Study for Sintilimab Second-line or Later-line Treatment of Advanced Non-small Cell Lung Cancer with DDR Pathway Gene Mutation

Cancer type: Non-Small Cell Lung Cancer

Variant class: DNA repair mutation

Other identifier: ChiCTR1900023234

Population segments: Fourth line or greater, Second line, Stage III, Stage IV, Third line

Exclusion criteria variant classes: ALK fusion (Non-Small Cell Lung Cancer), EGFRi sensitizing mutation (Non-Small Cell Lung Cancer), ROS1 fusion (Non-Small Cell Lung Cancer)

Phase: I/II

Therapy: sintilimab

Location: China

NCT04849364
A Phase II Circulating Tumor DNA Enriched, Genomically Directed Post-neoadjuvant Trial for Patients With Residual Triple Negative Breast Cancer (PERSEVERE)

Cancer type: Triple Negative Breast Cancer

Variant class: DNA repair pathway

Other identifiers: HCRN BRE18-334, PERSEVERE

Population segments: Adjuvant, HER2 negative, Stage I, Stage II, Stage III, Triple receptor negative

Phase: II

Therapies: talazoparib, chemotherapy

Location: United States

US State: IN

Contact: Dr. Bryan P. Schneider [317-948-3885; bpschnei@iu.edu]

ATM splice site mutation

NCT03682289
Phase II Trial of AZD6738 Alone and in Combination With Olaparib in Patients With Selected Solid Tumor Malignancies

Cancer type: Prostate Cancer, Unspecified Solid Tumor

Variant class: ATM mutation

Other inclusion criteria: ATM underexpression

Other identifiers: 18-25513, 189510, NCI-2018-01648

Population segments: Second line, Stage III, Stage IV

Phase: II

Therapy: ceralasertib

Location: United States

US State: CA

Contact: Erika Zigman [877-837-3222; cancertrials@ucsf.edu]

Disclaimer: The data presented here is from a curated knowledgebase of publicly available information, but may not be exhaustive. The data version is 2021.11(003).

ATM splice site mutation (continued)

NCT04564027

A Modular Phase IIa Multicentre Open-Label Study to Investigate DNA-damage Response Agents (or Combinations) in Patients With Advanced Cancer Whose Tumours Contain Molecular Alterations (PLANETTE)

Cancer type: Castration-Resistant Prostate Cancer, Unspecified Solid Tumor

Variant class: ATM mutation

Other identifiers: D5339C00001, EudraCT Number: 2020-002529-27, PLANETTE

Population segments: Hormone refractory, Second line, Stage III, Stage IV

Exclusion criteria variant class: ATM mutation (Non-Small Cell Lung Cancer)

Phase: II

Therapy: ceralasertib

Location: United States

US States: CA, FL, IN, MI, NV, PA, SC

Contact: AstraZeneca Clinical Study Information Center [877-240-9479; information.center@astrazeneca.com]

NCT02029001

A Two-period, Multicenter, Randomized, Open-label, Phase II Study Evaluating the Clinical Benefit of a Maintenance Treatment Targeting Tumor Molecular Alterations in Patients with Progressive Locally-advanced or Metastatic Solid Tumors.

Cancer type: Unspecified Solid Tumor

Variant class: ATM mutation

Other identifiers: ET12-081, EudraCT Number: 2012-004510-34, MOST, ProfiLER

Population segments: Maintenance/Consolidation, Second line, Stage III, Stage IV, Third line

Exclusion criteria variant classes: ATM mutation (Gastric Cancer), BRCA1 germline mutation (Gastric Cancer), BRCA1 mutation (Gastric Cancer), BRCA2 germline mutation (Gastric Cancer), BRCA2 mutation (Gastric Cancer), HRR mutation (Gastric Cancer), PALB2 mutation (Gastric Cancer), RAD51 mutation (Gastric Cancer)

Phase: II

Therapy: olaparib

Location: France

NCT03742895

A Phase II Study of Olaparib Monotherapy in Participants With Previously Treated, Homologous Recombination Repair Mutation (HRRm) or Homologous Recombination Deficiency (HRD) Positive Advanced Cancer

Cancer type: Unspecified Solid Tumor

Variant class: ATM mutation

Other identifiers: (MK-7339-002 / LYNK-002), 19-139, 194694, 2018-01891, 7339-002, 7339-002-00, EudraCT Number: 2018-003007-19, IRAS ID: 255158, JapicCTI-194694, LYNK002, LYNK-002, MK7339-002, MK-7339-002, MK7339-002-00, NCI-2018-03519, PER-046-18, SNCTP000003157

Population segments: BRCA, Fourth line or greater, Second line, Stage III, Stage IV, Third line

Exclusion criteria variant classes: BRCA germline mutation (Breast Cancer, Ovarian Cancer), BRCA mutation (Ovarian Cancer)

Phase: II

Therapy: olaparib

Locations: Argentina, Australia, Canada, Colombia, Denmark, France, Guatemala, Ireland, Israel, Italy, Mexico, Peru, Republic of Korea, Romania, Russian Federation, Spain, Switzerland, Turkey, United Kingdom, United States

US States: AZ, CA, CO, GA, KY, MA, MD, MI, NE, NJ, NY, SD, UT, WA

Contact: Toll Free Number [888-577-8839; Trialsites@merck.com]

ATM splice site mutation (continued)

NCT03967938
Efficacy of Olaparib in Advanced Cancers Occurring in Patients With Germline Mutations or Somatic Tumor Mutations in Homologous Recombination Genes

Cancer type: Unspecified Solid Tumor

Variant class: ATM mutation

Other identifiers: 1-2018 BSMO, 1-2018BSMO, EudraCT Number: 2018-002966-37, Precision 2 - Olaparib

Population segments: Line of therapy N/A, Stage III, Stage IV

Exclusion criteria variant classes: BRCA1 germline mutation (Breast Cancer, Pancreatic Cancer, Prostate Cancer), BRCA2 germline mutation (Breast Cancer, Pancreatic Cancer, Prostate Cancer)

Phase: II

Therapy: olaparib

Location: Belgium

NCT02693535
Targeted Agent and Profiling Utilization Registry (TAPUR) Study.

Cancer type: Unspecified Solid Tumor

Variant class: ATM mutation

Other identifiers: 20170529, NCI-2017-00510, Pro00014171, TAPUR

Population segments: Aggressive, BRAF, Diffuse large B-cell lymphoma (DLBCL), Extranodal marginal zone B-cell lymphoma (MALT), FGFR, First line, Follicular lymphoma (FL), Fourth line or greater, Indolent, Lymphoblastic lymphoma (LBL), Mantle cell lymphoma (MCL), Other subtype, Second line, Small lymphocytic lymphoma (SLL), Stage III, Stage IV, Third line, Waldenstrom's macroglobulinemia (WM)

Phase: II

Therapies: olaparib, ipilimumab + nivolumab, atezolizumab + talazoparib

Location: United States

US States: AL, AZ, CA, FL, GA, HI, IL, IN, ME, MI, NC, ND, NE, NH, OH, OR, PA, SD, TX, UT, VA, WA

Contact: Pam Mangat [pam.mangat@asco.org]

NCT02286687
Phase II Study of the PARP Inhibitor Talazoparib in Advanced Cancer Patients With Somatic Alterations in BRCA1/2, Mutations/Deletions in Other BRCA Pathway Genes and Germline Mutation in BRCA1/2 (Not Breast or Ovarian Cancer)

Cancer type: Unspecified Cancer

Variant class: ATM mutation

Other identifiers: 2013-0961, NCI-2014-02494

Population segments: Fourth line or greater, Stage III, Stage IV

Exclusion criteria variant classes: BRCA1 germline mutation (Breast Cancer, Ovarian Cancer), BRCA2 germline mutation (Breast Cancer, Ovarian Cancer)

Phase: II

Therapy: talazoparib

Location: United States

US State: TX

Contact: Dr. Sarina Piha-Paul [713-563-1930; spihapau@mdanderson.org]

Disclaimer: The data presented here is from a curated knowledgebase of publicly available information, but may not be exhaustive. The data version is 2021.11(003).

ATM splice site mutation (continued)

NCT02401347
A Phase II Clinical Trial of the PARP Inhibitor Talazoparib in BRCA1 and BRCA2 Wild Type Patients With Advanced Triple Negative Breast Cancer and Homologous Recombination Deficiency or Advanced HER2 Negative Breast Cancer or Other Solid Tumors With a Mutation in Homologous Recombination Pathway Genes Talazoparib Beyond BRCA (TBB) Trial

Cancer type: Breast Cancer, Unspecified Solid Tumor

Variant class: ATM mutation

Other inclusion criteria: ERBB2 negative, Hormone receptor positive

Other identifiers: BRS0050, NCI-2015-00036, TBB

Population segments: Estrogen receptor positive, HER2 negative, Progesterone receptor positive, Second line, Stage III, Stage IV, Triple receptor negative

Exclusion criteria variant classes: BRCA1 germline mutation (Breast Cancer, Triple Negative Breast Cancer, Unspecified Solid Tumor), BRCA1 mutation (Breast Cancer, Triple Negative Breast Cancer, Unspecified Solid Tumor), BRCA2 germline mutation (Breast Cancer, Triple Negative Breast Cancer, Unspecified Solid Tumor), BRCA2 mutation (Breast Cancer, Triple Negative Breast Cancer, Unspecified Solid Tumor), ERBB2 positive (Breast Cancer)

Phase: II

Therapy: talazoparib

Location: United States

US State: CA

Contact: Amanda Selin [650-721-6977; aselin@stanford.edu]

NCT04550494
A Pharmacodynamics-Driven Trial of Talazoparib, an Oral PARP Inhibitor, in Patients With Advanced Solid Tumors and Aberrations in Genes Involved in DNA Damage Response

Cancer type: Unspecified Solid Tumor

Variant class: ATM mutation

Other identifiers: 000246, 000264-C, 10000264, 10371, NCI-2020-06906

Population segments: BRCA, Fourth line or greater, HER2 positive, Hormone refractory, Second line, Stage II, Stage III, Stage IV, Third line

Phase: II

Therapy: talazoparib

Location: United States

US State: MD

Contact: Multiple contacts: See www.clinicaltrials.gov for complete list of contacts.

NCT03611868
A Phase Ib/II Study of APG-115 in Combination With Pembrolizumab in Patients With Unresectable or Metastatic Melanomas or Advanced Solid Tumors

Cancer type: Unspecified Solid Tumor

Variant class: ATM mutation

Other inclusion criteria: TP53 wild type

Other identifiers: 2020-0136, APG-115-US-002, Keynote MK-3475-B66, NCI-2019-08846

Population segments: Adenocarcinoma, Fourth line or greater, STK11, Second line, Stage III, Stage IV, Third line

Exclusion criteria variant class: FGFR fusion (Bladder Urothelial Carcinoma)

Phase: I/II

Therapies: alrizomadlin, pembrolizumab

Locations: Australia, United States

US States: AR, AZ, CA, DC, FL, MO, NY, PA, TN, TX, VA

Contact: Kathryn Shantz [301-802-3414; kate.shantz@ascentage.com]

Disclaimer: The data presented here is from a curated knowledgebase of publicly available information, but may not be exhaustive. The data version is 2021.11(003).

ATM splice site mutation (continued)

NCT03842228
A Phase Ib Biomarker-Driven Combination Trial of Copanlisib, Olaparib, and Durvalumab (MEDI4736) in Patients With Advanced Solid Tumors

Cancer type: Unspecified Solid Tumor

Variant class: ATM mutation

Other identifiers: 10217, 19-628, NCI10217, NCI-2019-00601

Population segments: Line of therapy N/A, Stage III, Stage IV

Phase: I

Therapies: copanlisib, olaparib, durvalumab

Location: United States

US States: MA, TX

Contact: Multiple contacts: See www.clinicaltrials.gov for complete list of contacts.

NCT04586335
Open Label, Phase Ib Study to Evaluate the Safety, Tolerability, Pharmacokinetics and Clinical Activity of CYH33, an Oral a-specific PI3K Inhibitor in Combination With Olaparib, an Oral PARP Inhibitor in Patients With Advanced Solid Tumors.

Cancer type: Breast Cancer, Endometrial Carcinoma, Ovarian Cancer, Prostate Cancer, Unspecified Solid Tumor

Variant class: ATM mutation

Other identifiers: CTR20202339, CYH33-G102

Population segments: BRCA, Second line, Stage III, Stage IV

Phase: I

Therapies: HH-CYH33, olaparib

Locations: Australia, United States

US State: TX

Contact: Dr. Jason Sudia [908-380-1329; jason.sudia@haihepharma.com]

NCT04123366
A Phase II Study of Olaparib in Combination With Pembrolizumab in Participants With Previously Treated, Homologous Recombination Repair Mutation (HRRm) and/or Homologous Recombination Deficiency (HRD)-Positive Advanced Cancer

Cancer type: Unspecified Solid Tumor

Variant class: HRR mutation

Other identifiers: 7339-007, EudraCT Number: 2019-001745-40, jRCT2011200025, KEYLYNK-007, MK-7339-007, NCI-2019-08758, PER-029-19

Population segments: BRCA, Second line, Stage III, Stage IV

Exclusion criteria variant classes: BRCA germline mutation (Breast Cancer, Ovarian Cancer), BRCA mutation (Breast Cancer, Ovarian Cancer)

Phase: II

Therapies: olaparib, pembrolizumab

Locations: Argentina, Australia, Canada, Colombia, France, Germany, Guatemala, Israel, Italy, Japan, Latvia, Mexico, Peru, Poland, Puerto Rico, Republic of Korea, Romania, South Africa, Spain, Sweden, Turkey, Ukraine, United States

US States: AL, AZ, CA, CO, FL, GA, KY, NJ, NY, OH, TX, UT, VA

Contact: Toll Free Number [888-577-8839; Trialsites@merck.com]

Disclaimer: The data presented here is from a curated knowledgebase of publicly available information, but may not be exhaustive. The data version is 2021.11(003).

ATM splice site mutation (continued)

NCT04497116
Phase I/IIa Study of the Safety, Pharmacokinetics, Pharmacodynamics and Preliminary Clinical Activity of RP-3500 Alone or in Combination With Talazoparib in Advanced Solid Tumors With ATR Inhibitor Sensitizing Mutations (TRESR Study)

Cancer type: Unspecified Solid Tumor

Variant class: ATM aberration

Other identifiers: 20-521, EudraCT Number: 2020-000301-87, IRAS ID: 286609, NCI-2020-06869, RP-3500-01, TRESR, TRESR Study

Population segments: Second line, Stage III, Stage IV

Phase: I/II

Therapies: RP-3500, talazoparib

Locations: Canada, Denmark, United Kingdom, United States

US States: MA, NC, NY, RI, TN, TX

Contact: Dr. Peter Manley [857-322-5553; pmanley@reparerx.com]

NCT03415659
A Phase I, Open-label, Single-center, Single/Multiple-dose, Dose-escalation/ Dose-expansion Clinical Study on Tolerance and Pharmacokinetics of HWH340 Tablet in Patients With Advanced Solid Tumors

Cancer type: Unspecified Solid Tumor

Variant class: HRR mutation

Other inclusion criteria: BRCA1 germline mutation negative, BRCA1 mutation negative, BRCA2 germline mutation negative, BRCA2 mutation negative

Other identifiers: CTR20171649, HWH340-RFPA 20170821, RFPA 20170821

Population segments: BRCA, Fourth line or greater, HER2 negative, Second line, Stage III, Stage IV, Third line

Phase: I

Therapy: HWH-340

Location: China

NCT03767075
Basket of Baskets: A Modular, Open-label, Phase II, Multicentre Study To Evaluate Targeted Agents in Molecularly Selected Populations With Advanced Solid Tumours

Cancer type: Unspecified Solid Tumor

Variant class: DNA repair mutation

Other identifiers: (Basket of Baskets) (BoB), EudraCT number: 2017-005108-89, MO39164, NL65937.031.18, VHIO17002

Population segments: Second line, Stage III, Stage IV

Phase: II

Therapy: atezolizumab

Locations: France, Germany, Netherlands, Spain, Sweden, United Kingdom

---

NCT04905914
A Phase I/IIa, Open-Label, Safety, Pharmacokinetic, And Preliminary Efficacy Study Of Oral ATRN-119 In Patients With Advanced Solid Tumors

Cancer type: Unspecified Solid Tumor

Variant class: DNA repair mutation

Other identifier: AR-276-01

Population segments: Line of therapy N/A, Stage III, Stage IV

Phase: I/II

Therapy: ATRN-119

Location: United States

US State: MA

Contact: Robert Hasson [619-540-6253; rhasson@pacificlinkconsulting.com]

Disclaimer: The data presented here is from a curated knowledgebase of publicly available information, but may not be exhaustive. The data version is 2021.11(003).

ATM splice site mutation (continued)

NCT04901702

A Randomized Phase I/II Study of Talazoparib or Temozolomide in Combination With Onivyde in Children With Recurrent Solid Malignancies and Ewing Sarcoma

Cancer type: Unspecified Solid Tumor

Variant class: HRR pathway

Other identifiers: EudraCT Number: 2021-003570-31, ONITT

Population segments: Second line, Stage IV

Exclusion criteria variant classes: HRR germline pathway (Central Nervous System Neoplasm, Ewing Sarcoma), HRR pathway (Central Nervous System Neoplasm, Ewing Sarcoma)

Phase: I/II

Therapies: talazoparib, chemotherapy

Locations: Canada, United States

US State: TN

Contact: Dr. Sara Federico [866-278-5833; referralinfo@stjude.org]

NCT03155620

NCI-COG Pediatric MATCH (Molecular Analysis for Therapy Choice) Screening Protocol

Cancer type: Unspecified Solid Tumor

Variant class: DNA repair pathway

Other identifiers: 17-729, 2017-0625, 20170841, APEC1621SC, COGAPEC1621, COGAPEC1621SC, N 55017 SC, NCI-2017-01251, NCI-COG Pediatric MATCH, Pediatric MATCH

Population segments: ALK, Aggressive, BRAF, FGFR, HRAS, Indolent, KRAS, NRAS, Pediatric or Adolescent, Second line, Stage III, Stage IV

Phase: II

Therapy: olaparib

Locations: Puerto Rico, United States

US States: AK, AL, AR, AZ, CA, CO, CT, DC, DE, FL, GA, HI, IA, ID, IL, IN, KY, LA, MA, MD, ME, MI, MN, MO, MS, NC, NE, NH, NJ, NV, NY, OH, OK, OR, PA, RI, SC, SD, TN, TX, UT, VA, VT, WA, WI

Contact: Multiple contacts: See www.clinicaltrials.gov for complete list of contacts.

NCT03233204

NCI-COG Pediatric MATCH (Molecular Analysis for Therapy Choice)- Phase 2 Subprotocol of Olaparib in Patients With Tumors Harboring Defects in DNA Damage Repair Genes

Cancer type: Unspecified Solid Tumor

Variant class: DNA repair pathway

Other identifiers: 20170841, APEC1621H, NCI-2017-00766

Population segments: Aggressive, Indolent, Pediatric or Adolescent, Second line, Stage III, Stage IV

Phase: II

Therapy: olaparib

Locations: Puerto Rico, United States

US States: AK, AL, AR, AZ, CA, CO, DC, DE, FL, GA, IA, ID, IL, IN, KY, LA, MD, ME, MI, MN, MO, MS, NC, ND, NE, NJ, NY, OH, OK, OR, PA, SC, SD, TN, TX, UT, VA, VT, WA, WI

Contact: Multiple contacts: See www.clinicaltrials.gov for complete list of contacts.

Disclaimer: The data presented here is from a curated knowledgebase of publicly available information, but may not be exhaustive. The data version is 2021.11(003).

ATM splice site mutation (continued)

NCT04267939
An Open-label Phase Ib Study to Determine the Maximum Tolerated and/or Recommended Phase II Dose of the ATR Inhibitor BAY 1895344 in Combination With PARP Inhibitor Niraparib, in Patients With Recurrent Advanced Solid Tumors and Ovarian Cancer

Cancer type: Ovarian Cancer, Unspecified Solid Tumor

Variant class: DNA repair pathway

Other identifiers: 18595, 19-810, 2019-0471, 20-222, EudraCT Number: 2018-003930-34, NCI-2020-01405

Population segments: Maintenance/Consolidation, Second line, Stage III, Stage IV

Exclusion criteria variant class: DNA repair pathway (Prostate Cancer)

Phase: I

Therapies: BAY-1895344, niraparib

Location: United States

US States: MA, NY, TX

Contact: Bayer Clinical Trials Contact [888-842-2937; clinical-trials-contact@bayer.com]

NCT04693468
Modular Phase IB Hypothesis-Testing, Biomarker-Driven, Talazoparib Combination Trial (TalaCom)

Cancer type: Unspecified Solid Tumor

Variant class: DNA repair pathway

Other identifiers: 2020-0436, NCI-2020-06041, TalaCom

Population segments: Hormone refractory, Second line, Stage III, Stage IV

Phase: I

Therapies: talazoparib, palbociclib, axitinib, crizotinib

Location: United States

US State: TX

Contact: Timothy A. Yap [713-563-1784; tyap@mdanderson.org]

NCT03810105
A Phase II Study of Olaparib and Durvalumab in Men With Castration Sensitive Biochemically Recurrent Non-Metastatic Prostate Cancer Harboring Mutations in DNA Damage Repair

Cancer type: Prostate Cancer

Variant class: ATM splice site mutation or DNA repair splice site mutation

Other identifiers: 18-480, NCI-2019-01627

Population segments: Adjuvant, Stage I, Stage II, Stage III

Phase: II

Therapies: durvalumab, olaparib

Location: United States

US States: CA, IL, MI, NJ, NY

Contact: Dr. Karen Autio [646-422-4632; autiok@mskcc.org]

Disclaimer: The data presented here is from a curated knowledgebase of publicly available information, but may not be exhaustive. The data version is 2021.11(003).

ATM splice site mutation (continued)

NCT02975934
TRITON3: A Multicenter, Randomized, Open Label Phase III Study of Rucaparib Versus Physician's Choice of Therapy for Patients With Metastatic Castration Resistant Prostate Cancer Associated With Homologous Recombination Deficiency

Cancer type: Castration-Resistant Prostate Cancer

Variant class: ATM mutation

Other identifiers: 16238, 1723GCCC, 18-146, 18-300, 20171185, 2018IS040, CO-338-063, CSET 2620, EudraCT Number: 2016-003163-20, GU 146, IGUP17020, IRAS ID: 222298, NCI-2017-01110, S16-02260, STAR Trial, TRITON 3_CO-338-063, TRITON3

Population segments: BRCA, First line, Hormone refractory, Stage IV

Phase: III

Therapies: rucaparib, hormone therapy

Locations: Australia, Belgium, Canada, Denmark, France, Germany, Ireland, Israel, Italy, Spain, United Kingdom, United States

US States: AZ, CA, CO, CT, DE, FL, HI, MD, MN, NC, NY, OH, OR, TN, TX, VA, WA

Contact: Clovis Oncology For North America [844-258-7662; medinfo@clovisoncology.com]

NCT04336943
Durvalumab (MEDI4736) and Olaparib (AZD2281) for Treatment of Biochemically Recurrent Prostate Cancer in Men Predicted to Have a High Neoantigen Load: A Pilot Study

Cancer type: Prostate Adenocarcinoma

Variant class: ATM mutation

Other identifiers: 10509, NCI-2020-01860, RG1007001

Population segments: Line of therapy N/A, Stage I, Stage II, Stage III

Phase: II

Therapies: durvalumab, olaparib

Location: United States

US State: WA

Contact: Michael Schweizer [206-606-6252; schweize@uw.edu]

NCT04053322
An International Multicenter Phase II Trial of Durvalumab (MEDI4736) Plus OLAparib Plus Fulvestrant in Metastatic or Locally Advanced ER-positive, HER2-negative Breast Cancer Patients Selected Using Criteria That Predict Sensitivity to Olaparib

Cancer type: Breast Cancer

Variant class: ATM mutation

Other inclusion criteria: ERBB2 negative, ER positive

Other identifiers: CADUSEIME08, DOLAF, EudraCT Number: 2018-003832-57, UC-0140/1812

Population segments: BRCA, Estrogen receptor positive, FGFR, HER2 negative, Progesterone receptor positive, Second line, Stage III, Stage IV

Phase: II

Therapies: durvalumab, olaparib, hormone therapy

Location: France

ATM splice site mutation (continued)

NCT05011383
High-dose Testosterone in Men With Metastatic Castration-resistant Prostate Cancer and ATM or CDK12 Deficiency

Cancer type: Castration-Resistant Prostate Cancer

Variant class: ATM mutation

Other identifiers: SPLP-003-20F, VA-BAT

Population segments: Hormone refractory, Second line, Stage IV

Phase: II

Therapy: hormone therapy

Location: United States

US State: WA

Contact: Dr. Robert B. Montgomery [206-277-6878; Robert.Montgomery@va.gov]

NCT03012321
BRCAAway: A Randomized Phase II Trial of Abiraterone, Olaparib, or Abiraterone + Olaparib in Patients With Metastatic Castration-Resistant Prostate Cancer With DNA Repair Defects

Cancer type: Castration-Resistant Prostate Cancer

Variant class: ATM mutation

Other identifiers: 19137, BRCAAway, NCI-2016-01834, NU 16U05, NU_16U05, PCCTC #: c16-168, PCCTC c16-168, STU00203960

Population segments: First line, Hormone refractory, Stage IV

Phase: II

Therapies: hormone therapy, olaparib, steroid

Location: United States

US States: CA, FL, IL, IN, MD, MI, MN, MO, NC, NY, UT, VA

Contact: Study Coordinator [312-695-1301; cancertrials@northwestern.edu]

NCT03553004
Niraparib in Metastatic Pancreatic Cancer After Previous Chemotherapy (NIRA-PANC): A Phase II Trial

Cancer type: Pancreatic Cancer

Variant class: ATM mutation

Other identifiers: IIT-2017-NIRA-PANC, NIRA-PANC, STUDY00141862

Population segments: BRCA, Second line, Stage IV, Third line

Phase: II

Therapy: niraparib

Location: United States

US States: KS, MO

Contact: Clinical Trials Nurse Navigator [913-588-3671; navigation@kumc.edu]

NCT03601923
Phase II Proof-of-Concept Trial Testing the PARP Inhibitor Niraparib in Patients With Pancreatic Cancer Harboring Deficiencies in Homologous Recombination DNA Repair

Cancer type: Pancreatic Cancer

Variant class: ATM mutation

Other identifiers: 18-207, NCI-2018-02395

Population segments: Second line, Stage IV

Phase: II

Therapy: niraparib

Location: United States

US State: MA

Contact: Dr. James Clreary [617-632-5960; JCLEARY@PARTNERS.ORG]

Disclaimer: The data presented here is from a curated knowledgebase of publicly available information, but may not be exhaustive. The data version is 2021.11(003).

ATM splice site mutation (continued)

NCT03840967
A Phase II Study Evaluating Safety and Efficacy of Niraparib in Patients With Previously Treated Homologous Recombination (HR) Defective or Loss of Heterozygosity (LOH) High Metastatic Esophageal/Gastroesophageal Junction/ Proximal Gastric Adenocarcinoma

Cancer type: Esophageal Cancer, Gastric Cancer, Gastroesophageal Junction Adenocarcinoma

Variant class: ATM mutation

Other identifiers: 20166, 71618, HCRN ESO17-325, NCI-2019-07395

Population segments: Second line, Stage III, Stage IV

Phase: II

Therapy: niraparib

Location: United States

US States: FL, IN, MI, NY

Contact: Dr. Shadia Jalal [317-274-3658; sjalal@iu.edu]

NCT03925350
A Phase II Study of Niraparib in Patients With Advanced Melanoma With Genetic Homologous Recombination (HR) Mutation / Alteration

Cancer type: Melanoma

Variant class: ATM mutation

Other identifier: CPMC17-MEL01

Population segments: Second line, Stage III, Stage IV

Phase: II

Therapy: niraparib

Location: United States

US State: CA

Contact: Laurel Brechtel [415-600-1654; BrechtLA@sutterhealth.org]

NCT04030559
Phase II Trial of PARP Inhibitor Niraparib for Men With High Risk Prostate Cancer and DNA Damage Response Defects

Cancer type: Prostate Cancer

Variant class: ATM mutation

Other identifiers: 1412676, NCI-2019-04365, UCDCC#279

Population segments: First line, Neoadjuvant, Stage I, Stage II, Stage IV

Phase: II

Therapy: niraparib

Location: United States

US State: CA

Contact: Marc Dall'Era [916-734-3771; mdallera@ucdavis.edu]

NCT04187833
Phase II Trial of Nivolumab in Combination with Talazoparib in Patients with Unresectable or Metastatic Melanoma and Mutations in BRCA or BRCA-ness Genes.

Cancer type: Melanoma

Variant class: ATM mutation

Other identifiers: CASE2619, NCI-2020-00416

Population segments: Second line, Stage III, Stage IV

Phase: II

Therapies: nivolumab, talazoparib

Location: United States

US State: OH

Contact: Dr. Pauline Funchain [866-223-8100; TaussigResearch@ccf.org]

Disclaimer: The data presented here is from a curated knowledgebase of publicly available information, but may not be exhaustive. The data version is 2021.11(003).

ATM splice site mutation (continued)

NCT03344965
A Phase II Study of Olaparib Monotherapy in Metastatic Breast Cancer Patients With Germline or Somatic Mutations in DNA Repair Genes (Olaparib Expanded)

Cancer type: Breast Cancer

Variant class: ATM mutation

Other identifiers: 17-428, 18-414, NCI-2018-00778, TBCRC 048, TBCRC048

Population segments: BRCA, Estrogen receptor positive, HER2 negative, HER2 positive, Progesterone receptor positive, Second line, Stage IV

Exclusion criteria variant classes: BRCA1 germline mutation (Breast Cancer), BRCA2 germline mutation (Breast Cancer)

Phase: II

Therapy: olaparib

Location: United States

US States: AL, CA, IL, MA, MD, NC, NJ, NY, PA, WA

Contact: Dr. Nadine Tung [617-667-1962; ntung@bidmc.harvard.edu]

NCT03375307
A Phase II Study of Olaparib (AZD2281) in Patients With Metastatic/Advanced Urothelial Carcinoma With DNA-Repair Defects

Cancer type: Bladder Urothelial Carcinoma

Variant class: ATM mutation

Other identifiers: 10144, 19C0023, NCI-2017-02296, P162941

Population segments: BRCA, STK11, Second line, Stage III, Stage IV

Phase: II

Therapy: olaparib

Location: United States

US States: CA, CO, KY, MD, NY, OK

Contact: Multiple contacts: See www.clinicaltrials.gov for complete list of contacts.

NCT03786796
Phase II Study of Olaparib in Metastatic Renal Cell Carcinoma Patients Harboring a BAP-1 or Other DNA Repair Gene Mutations (ORCHID)

Cancer type: Kidney Cancer

Variant class: ATM mutation

Other identifiers: CRMS-70750, J18166, NCI-2019-06309, ORCHID

Population segments: Fourth line or greater, Second line, Stage IV, Third line

Phase: II

Therapy: olaparib

Location: United States

US State: MD

Contact: Irina Rifkind [410-502-2043; irifkin1@jhmi.edu]

Disclaimer: The data presented here is from a curated knowledgebase of publicly available information, but may not be exhaustive. The data version is 2021.11(003).

ATM splice site mutation (continued)

NCT04042831
A Phase II Study of Olaparib in Patients With Advanced Biliary Tract Cancer With Aberrant DNA Repair Gene Mutations

Cancer type: Biliary Tract Carcinoma

Variant class: ATM mutation

Other identifiers: ACCRU-ICRN-1702, NCI-2019-04434

Population segments: Second line, Stage IV

Phase: II

Therapy: olaparib

Location: United States

US States: AZ, MN, NY, TX

Contact: Multiple contacts: See www.clinicaltrials.gov for complete list of contacts.

NCT04515836
Phase II Trial of Olaparib in Homologous Recombination Deficient (HRD) Malignant Mesothelioma

Cancer type: Mesothelioma

Variant class: ATM mutation

Population segments: (N/A), Second line

Phase: II

Therapy: olaparib

Location: United States

US State: IL

Contact: Dr. Hedy L. Kindler [773-702-0360; hkindler@medicine.bsd.uchicago.edu]

NCT04633902
Phase II Study of Olaparib in Combination With Pembrolizumab in Patients With Advanced Melanoma With Homologous Recombination (HR) Pathway Gene Mutation

Cancer type: Melanoma

Variant class: ATM mutation

Other identifier: CPMC19-MEL01

Population segments: BRAF, Second line, Stage III, Stage IV

Phase: II

Therapies: olaparib, pembrolizumab

Location: United States

US State: CA

Contact: Dr. Kevin B. Kim [415-885-8600; ClinicalResearch@sutterhealth.org]

NCT03248570
Phase II Open Label Study of Pembrolizumab in Patients With Metastatic Castration Resistant Prostate Cancer (mCRPC) With or Without DNA Damage Repair Defects

Cancer type: Castration-Resistant Prostate Cancer

Variant class: ATM mutation

Other identifiers: 16557, 17-22165, CC 16557, NCI-2017-02408

Population segments: BRCA, Hormone refractory, Line of therapy N/A, Stage IV

Phase: II

Therapy: pembrolizumab

Location: United States

US State: CA

Contact: Patricia Li [415-476-5975; GUTrials@ucsf.edu]

Disclaimer: The data presented here is from a curated knowledgebase of publicly available information, but may not be exhaustive. The data version is 2021.11(003).

ATM splice site mutation (continued)

NCT03506997

PERSEUS1: Phase II Trial of the Immune Checkpoint Inhibitor Pembrolizumab for Patients Suffering From Metastatic Prostate Cancer

Cancer type: Castration-Resistant Prostate Cancer

Variant class: ATM mutation

Other identifiers: CCR4559, EudraCT Number: 2017-000931-15, ICR-CTSU/2016/10060, IRAS ID 208952, PERSEUS1

Population segments: Hormone refractory, Second line, Stage IV

Phase: II

Therapy: pembrolizumab

Location: United Kingdom

NCT03533946

A Phase II Study of Rucaparib Monotherapy in Nonmetastatic, Hormone-Sensitive Prostate Cancer Demonstrating "BRCAness" Genotype (ROAR)

Cancer type: Prostate Cancer

Variant class: ATM mutation

Other identifiers: HCI111833, NCI-2018-01754, ROAR

Population segments: First line, Stage I, Stage II, Stage III

Phase: II

Therapy: rucaparib

Location: United States

US State: UT

Contact: Jill Broghammer [801-213-6232; jill.broghammer@hci.utah.edu]

NCT04892693

Talazoparib in Advanced Breast Cancer Patients With Homologous Recombinant Deficiency: A Phase II Clinical and Exploratory Biomarker Study of Talazoparib

Cancer type: Breast Cancer

Variant class: ATM mutation

Other identifier: H-2009-079-1157

Population segments: BRCA, Line of therapy N/A, Stage III, Stage IV

Phase: II

Therapy: talazoparib

Location: Republic of Korea

NCT04740190

Combination Talazoparib - Carboplatin for Recurrent High-grade Glioma With DNA Damage Repair Deficiency (DDRd)

Cancer type: Anaplastic Astrocytoma, Anaplastic Oligoastrocytoma, Anaplastic Oligodendroglioma, Anaplastic Pleomorphic Xanthoastrocytoma, Diffuse Midline Glioma, Glioblastoma

Variant class: ATM mutation

Other identifiers: TAC-GReD, UW-20396

Population segments: (N/A), BRCA, Second line

Phase: II

Therapies: talazoparib, chemotherapy

Location: Hong Kong

Disclaimer: The data presented here is from a curated knowledgebase of publicly available information, but may not be exhaustive. The data version is 2021.11(003).

ATM splice site mutation (continued)

NCT04253262
A Phase Ib/II Study of Copanlisib Combined With Rucaparib in Patients With Metastatic Castration-resistant Prostate Cancer

Cancer type: Castration-Resistant Prostate Cancer

Variant class: ATM mutation

Other identifiers: BrUOG 360, BrUOG360

Population segments: Hormone refractory, Second line, Stage IV

Phase: I/II

Therapies: copanlisib, rucaparib

Location: United States

US State: RI

Contact: BrUOG [401-863-3000; BrUOG@brown.edu]

NCT04095273
A Multicenter, Non-randomized, Open-label Phase Ib Study to Determine the Maximum Tolerated and Recommended Phase II Dose of the ATR Inhibitor BAY1895344 in Combination With Pembrolizumab and to Characterize Its Safety, Tolerability, Pharmacokinetics and Preliminary Anti-tumor Activity in Patients With Advanced Solid Tumors

Cancer type: Breast Cancer

Variant class: ATM mutation

Other inclusion criteria: ERBB2 negative, Hormone receptor positive

Other identifiers: 19741, EudraCT Number: 2018-003420-36, Keynote 919, NCI-2019-06734

Population segments: HER2 negative, Hormone refractory, Second line, Stage III, Stage IV

Phase: I

Therapies: BAY-1895344, pembrolizumab

Locations: Germany, Spain, Switzerland, United States

US States: CA, CT, MA, MD, NC, NY, OH, TX

Contact: Bayer Clinical Trials Contact [clinical-trials-contact@bayer.com]

NCT04169841
Precision Medicine Phase II Study Evaluating the Efficacy of a Double Immunotherapy by Durvalumab and Tremelimumab Combined With Olaparib in Patients With Solid Cancers and Carriers of Homologous Recombination Repair Genes Mutation in Response or Stable After Olaparib Treatment

Cancer type: Breast Cancer, Endometrial Carcinoma, Head and Neck Cancer, Kidney Cancer, Non-Small Cell Lung Cancer, Ovarian Cancer, Pancreatic Cancer, Prostate Cancer

Variant class: HRR mutation

Other identifier: GUIDE2REPAIR

Population segments: Fourth line or greater, Hormone refractory, Maintenance/ Consolidation, Second line, Stage III, Stage IV, Third line

Exclusion criteria variant classes: ALK fusion (Lung Cancer), BRAF mutation (Lung Cancer), EGFR mutation (Lung Cancer), ROS1 mutation (Lung Cancer)

Phase: II

Therapies: durvalumab, tremelimumab, olaparib

Location: France

ATM splice site mutation (continued)

NCT04940637
A Phase II, Open-Label, Single Arm, Prospective, Multicenter Study of Niraparib Plus Dostarlimab in Patients With Advanced NSCLC and/or MPM, and Positive for PD-L1 Expression and Germline or Somatic Mutations in the HRR Genes

Cancer type: Mesothelioma, Non-Small Cell Lung Cancer

Variant class: HRR mutation

Other inclusion criteria: ALK fusion negative, CD274 expression, EGFRi sensitizing mutation negative, ROS1 fusion negative or CD274 expression

Other identifiers: EudraCT Number: 2020-000109-10, UNITO-001, UNITO-001/2020

Population segments: Second line, Stage III, Stage IV

Exclusion criteria variant classes: ALK fusion (Non-Small Cell Lung Cancer), EGFRi sensitizing mutation (Non-Small Cell Lung Cancer), ROS1 fusion (Non-Small Cell Lung Cancer)

Phase: II

Therapies: niraparib, dostarlimab

Location: Italy

NCT04939662
Phase II, Single-arm Study of Olaparib and Bevacizumab Combination Therapy in Relapsed Small Cell Lung Cancer Subjects With DNA Damage Response and the Repair Pathway Alteration, ATM Deficiency, SLFN11 Positive, or POU2F3 Positive

Cancer type: Small Cell Lung Cancer

Variant class: HRR mutation

Other identifier: SUKSES-B2

Population segments: (N/A), BRCA, Pulmonary, Second line, Third line

Phase: II

Therapies: olaparib, bevacizumab

Location: Republic of Korea

NCT04666740
A Phase II Trial to Evaluate the Safety and Antitumor Activity of Pembrolizumab and OLApaRib (POLAR) Maintenance for Patients With Metastatic Pancreatic Ductal Adenocarcinoma and Homologous Recombination Deficiency and/or Exceptional Treatment Response to Platinum-Based Therapy

Cancer type: Pancreatic Cancer

Variant class: ATM aberration

Other identifier: 20-481

Population segments: BRCA, Maintenance/Consolidation, Stage IV

Phase: II

Therapies: pembrolizumab, olaparib

Location: United States

US States: NJ, NY

Contact: Dr. Wungki Park [646-888-4543; yaqubiea@mskcc.org]

Disclaimer: The data presented here is from a curated knowledgebase of publicly available information, but may not be exhaustive. The data version is 2021.11(003).

ATM splice site mutation (continued)

NCT02264678

A Modular Phase I, Open-Label, Multicentre Study to Assess the Safety, Tolerability, Pharmacokinetics and Preliminary Anti-tumour Activity of Ceralasertib in Combination With Cytotoxic Chemotherapy and/or DNA Damage Repair/Novel Anti-cancer Agents in Patients With Advanced Solid Malignancies

Cancer type: Triple Negative Breast Cancer

Variant class: HRR mutation

Other inclusion criteria: BRCA germline mutation negative, BRCA mutation negative

Other identifiers: 0C-14-9, 16-031, 18-342, D5330C00004, EudraCT Number: 2014-002233-66, IRAS ID 160200, NCI-2015-00687, Study 4

Population segments: Adenocarcinoma, BRCA, HER2 negative, Pulmonary, Second line, Stage III, Stage IV, Third line, Triple receptor negative

Exclusion criteria variant class: ERBB2 positive (Breast Cancer)

Phase: I

Therapies: ceralasertib, olaparib

Locations: France, Republic of Korea, United Kingdom, United States

US States: CA, MA, NY

Contact: AstraZeneca Clinical Study Information Center [877-240-9479; information.center@astrazeneca.com]

NCT04890613

Phase Ib Expansion Study of CX-5461 in Patients With Solid Tumours and BRCA2 and/or PALB2 Mutation

Cancer type: Ovarian Cancer

Variant class: HRR mutation

Other identifier: CX-5461-04

Population segments: BRCA, Second line, Stage II, Stage III, Stage IV

Phase: I

Therapy: pidnarulex

Location: Canada

NCT05002868

A Multi-center, Open-label, Phase I/Ib Study to Assess the Safety, Pharmacokinetics and Anti-tumor Activity of RP12146, a Poly (ADP-ribose) Polymerase (PARP) Inhibitor, in Patients With Locally Advanced or Metastatic Solid Tumors

Cancer type: Biliary Tract Carcinoma, Breast Cancer, Colorectal Cancer, Endometrial Carcinoma, Gastric Cancer, Ovarian Cancer, Pancreatic Cancer, Prostate Cancer, Small Cell Lung Cancer

Variant class: HRR mutation

Other identifier: RP12146-2101

Population segments: Extensive, Pulmonary, Second line, Stage III, Stage IV

Exclusion criteria variant class: ERBB2 positive (Breast Cancer)

Phase: I

Therapy: RP12146

Location: Poland

ATM splice site mutation (continued)

NCT03395197

A Phase III, Randomized, Double-blind, Placebo-controlled Study Of Talazoparib With Enzalutamide In Metastatic Castration-resistant Prostate Cancer

Cancer type: Castration-Resistant Prostate Cancer

Variant class: DNA repair mutation

Other identifiers: BASIC-ROYAL / HIGH-ROYAL, C3441021, CSET 2938, CTR20191443, CTR20191444, EudraCT Number: 2017-003295-31, GU 170 - C3441021, HCC 19-054, JapicCTI-194665, LH19.022, MOH_2019-05-16_007127, NCI-2018-01651, Pfizer C3441021, R19062M, RECF3951, RWF_C3441021 TALAPRO-2, TALAPRO-2, TX228665, UCI-18-47, VICCURO17115

Population segments: First line, Hormone refractory, Second line, Stage III, Stage IV

Phase: III

Therapies: talazoparib, hormone therapy

Locations: Argentina, Australia, Belgium, Brazil, Canada, Chile, China, Czech Republic, Finland, France, Germany, Hungary, Israel, Italy, Japan, New Zealand, Norway, Peru, Poland, Portugal, Republic of Korea, South Africa, Spain, Sweden, United Kingdom, United States

US States: AZ, CA, CO, FL, GA, IL, IN, KS, MO, NE, NJ, NY, OH, OK, OR, PA, SC, TN, TX, UT, VA, WI

Contact: Pfizer CT.gov Call Center [800-718-1021; ClinicalTrials.gov_Inquiries@pfizer.com]

NCT04821622

TALAPRO-3: A Phase III, Randomized, Double-blind, Study of Talazoparib With Enzalutamide Versus Placebo With Enzalutamide in Men With DDR Gene Mutated Metastatic Castration-sensitive Prostate Cancer

Cancer type: Prostate Adenocarcinoma

Variant class: DNA repair mutation

Other identifiers: C3441052, CTR20212191, EudraCT Number: 2021-000248-23, jRCT2031210098, TALAPRO-3

Population segments: Second line, Stage IV

Phase: III

Therapies: talazoparib, hormone therapy

Locations: Japan, Republic of Korea, United States

US States: CA, DC, FL, GA, IL, MI, NJ, NY, PA, SC, TX, UT

Contact: Pfizer CT.gov Call Center [800-718-1021; ClinicalTrials.gov_Inquiries@pfizer.com]

NCT04038502

An Open-label, Multicenter Phase II Study to Compare the Efficacy of Carboplatin as First-line Followed by Second-line Olaparib Versus Olaparib as First-line Followed by Second-line Carboplatin in the Treatment of Patients With Castration Resistant Prostate Cancer Containing Homologous Recombination Deficiency

Cancer type: Castration-Resistant Prostate Cancer

Variant class: DNA repair mutation

Other identifiers: 01781, 01783, 19-08, BRACeD, CX001963-01, ONCA-009-18F

Population segments: First line, Hormone refractory, Second line, Stage IV

Phase: II

Therapies: olaparib, chemotherapy

Location: United States

US States: CA, DC, FL, IL, MI, NC, NY, PA, WA

Contact: Dr. Robert B. Montgomery [206-277-6878; rbmontgo@u.washington.edu]

Disclaimer: The data presented here is from a curated knowledgebase of publicly available information, but may not be exhaustive. The data version is 2021.11(003).

ATM splice site mutation (continued)

No NCT ID - see other identifier(s)
Single-arm Exploratory Study for Sintilimab Second-line or Later-line Treatment of Advanced Non-small Cell Lung Cancer with DDR Pathway Gene Mutation

Cancer type: Non-Small Cell Lung Cancer

Variant class: DNA repair mutation

Other identifier: ChiCTR1900023234

Population segments: Fourth line or greater, Second line, Stage III, Stage IV, Third line

Exclusion criteria variant classes: ALK fusion (Non-Small Cell Lung Cancer), EGFRi sensitizing mutation (Non-Small Cell Lung Cancer), ROS1 fusion (Non-Small Cell Lung Cancer)

Phase: I/II

Therapy: sintilimab

Location: China

NCT04849364
A Phase II Circulating Tumor DNA Enriched, Genomically Directed Post-neoadjuvant Trial for Patients With Residual Triple Negative Breast Cancer (PERSEVERE)

Cancer type: Triple Negative Breast Cancer

Variant class: DNA repair pathway

Other identifiers: HCRN BRE18-334, PERSEVERE

Population segments: Adjuvant, HER2 negative, Stage I, Stage II, Stage III, Triple receptor negative

Phase: II

Therapies: talazoparib, chemotherapy

Location: United States

US State: IN

Contact: Dr. Bryan P. Schneider [317-948-3885; bpschnei@iu.edu]

ATM truncating mutation

NCT04550494
A Pharmacodynamics-Driven Trial of Talazoparib, an Oral PARP Inhibitor, in Patients With Advanced Solid Tumors and Aberrations in Genes Involved in DNA Damage Response

Cancer type: Unspecified Solid Tumor

Variant class: ATM truncating mutation

Other identifiers: 000246, 000264-C, 10000264, 10371, NCI-2020-06906

Population segments: BRCA, Fourth line or greater, HER2 positive, Hormone refractory, Second line, Stage II, Stage III, Stage IV, Third line

Phase: II

Therapy: talazoparib

Location: United States

US State: MD

Contact: Multiple contacts: See www.clinicaltrials.gov for complete list of contacts.

Disclaimer: The data presented here is from a curated knowledgebase of publicly available information, but may not be exhaustive. The data version is 2021.11(003).

ATM truncating mutation (continued)

NCT03682289
Phase II Trial of AZD6738 Alone and in Combination With Olaparib in Patients With Selected Solid Tumor Malignancies

Cancer type: Prostate Cancer, Unspecified Solid Tumor

Variant class: ATM mutation

Other inclusion criteria: ATM underexpression

Other identifiers: 18-25513, 189510, NCI-2018-01648

Population segments: Second line, Stage III, Stage IV

Phase: II

Therapy: ceralasertib

Location: United States

US State: CA

Contact: Erika Zigman [877-837-3222; cancertrials@ucsf.edu]

NCT04564027
A Modular Phase IIa Multicentre Open-Label Study to Investigate DNA-damage Response Agents (or Combinations) in Patients With Advanced Cancer Whose Tumours Contain Molecular Alterations (PLANETTE)

Cancer type: Castration-Resistant Prostate Cancer, Unspecified Solid Tumor

Variant class: ATM mutation

Other identifiers: D5339C00001, EudraCT Number: 2020-002529-27, PLANETTE

Population segments: Hormone refractory, Second line, Stage III, Stage IV

Exclusion criteria variant class: ATM mutation (Non-Small Cell Lung Cancer)

Phase: II

Therapy: ceralasertib

Location: United States

US States: CA, FL, IN, MI, NV, PA, SC

Contact: AstraZeneca Clinical Study Information Center [877-240-9479; information.center@astrazeneca.com]

NCT02029001
A Two-period, Multicenter, Randomized, Open-label, Phase II Study Evaluating the Clinical Benefit of a Maintenance Treatment Targeting Tumor Molecular Alterations in Patients with Progressive Locally-advanced or Metastatic Solid Tumors.

Cancer type: Unspecified Solid Tumor

Variant class: ATM mutation

Other identifiers: ET12-081, EudraCT Number: 2012-004510-34, MOST, ProfiLER

Population segments: Maintenance/Consolidation, Second line, Stage III, Stage IV, Third line

Exclusion criteria variant classes: ATM mutation (Gastric Cancer), BRCA1 germline mutation (Gastric Cancer), BRCA1 mutation (Gastric Cancer), BRCA2 germline mutation (Gastric Cancer), BRCA2 mutation (Gastric Cancer), HRR mutation (Gastric Cancer), PALB2 mutation (Gastric Cancer), RAD51 mutation (Gastric Cancer)

Phase: II

Therapy: olaparib

Location: France

ATM truncating mutation (continued)

NCT03742895

A Phase II Study of Olaparib Monotherapy in Participants With Previously Treated, Homologous Recombination Repair Mutation (HRRm) or Homologous Recombination Deficiency (HRD) Positive Advanced Cancer

Cancer type: Unspecified Solid Tumor

Variant class: ATM mutation

Other identifiers: (MK-7339-002 / LYNK-002), 19-139, 194694, 2018-01891, 7339-002, 7339-002-00, EudraCT Number: 2018-003007-19, IRAS ID: 255158, JapicCTI-194694, LYNK002, LYNK-002, MK7339-002, MK-7339-002, MK7339-002-00, NCI-2018-03519, PER-046-18, SNCTP000003157

Population segments: BRCA, Fourth line or greater, Second line, Stage III, Stage IV, Third line

Exclusion criteria variant classes: BRCA germline mutation (Breast Cancer, Ovarian Cancer), BRCA mutation (Ovarian Cancer)

Phase: II

Therapy: olaparib

Locations: Argentina, Australia, Canada, Colombia, Denmark, France, Guatemala, Ireland, Israel, Italy, Mexico, Peru, Republic of Korea, Romania, Russian Federation, Spain, Switzerland, Turkey, United Kingdom, United States

US States: AZ, CA, CO, GA, KY, MA, MD, MI, NE, NJ, NY, SD, UT, WA

Contact: Toll Free Number [888-577-8839; Trialsites@merck.com]

NCT03967938

Efficacy of Olaparib in Advanced Cancers Occurring in Patients With Germline Mutations or Somatic Tumor Mutations in Homologous Recombination Genes

Cancer type: Unspecified Solid Tumor

Variant class: ATM mutation

Other identifiers: 1-2018 BSMO, 1-2018BSMO, EudraCT Number: 2018-002966-37, Precision 2 - Olaparib

Population segments: Line of therapy N/A, Stage III, Stage IV

Exclusion criteria variant classes: BRCA1 germline mutation (Breast Cancer, Pancreatic Cancer, Prostate Cancer), BRCA2 germline mutation (Breast Cancer, Pancreatic Cancer, Prostate Cancer)

Phase: II

Therapy: olaparib

Location: Belgium

NCT02693535

Targeted Agent and Profiling Utilization Registry (TAPUR) Study.

Cancer type: Unspecified Solid Tumor

Variant class: ATM mutation

Other identifiers: 20170529, NCI-2017-00510, Pro00014171, TAPUR

Population segments: Aggressive, BRAF, Diffuse large B-cell lymphoma (DLBCL), Extranodal marginal zone B-cell lymphoma (MALT), FGFR, First line, Follicular lymphoma (FL), Fourth line or greater, Indolent, Lymphoblastic lymphoma (LBL), Mantle cell lymphoma (MCL), Other subtype, Second line, Small lymphocytic lymphoma (SLL), Stage III, Stage IV, Third line, Waldenstrom's macroglobulinemia (WM)

Phase: II

Therapies: olaparib, ipilimumab + nivolumab, atezolizumab + talazoparib

Location: United States

US States: AL, AZ, CA, FL, GA, HI, IL, IN, ME, MI, NC, ND, NE, NH, OH, OR, PA, SD, TX, UT, VA, WA

Contact: Pam Mangat [pam.mangat@asco.org]

Disclaimer: The data presented here is from a curated knowledgebase of publicly available information, but may not be exhaustive. The data version is 2021.11(003).

ATM truncating mutation (continued)

NCT02286687
Phase II Study of the PARP Inhibitor Talazoparib in Advanced Cancer Patients With Somatic Alterations in BRCA1/2, Mutations/Deletions in Other BRCA Pathway Genes and Germline Mutation in BRCA1/2 (Not Breast or Ovarian Cancer)

Cancer type: Unspecified Cancer

Variant class: ATM mutation

Other identifiers: 2013-0961, NCI-2014-02494

Population segments: Fourth line or greater, Stage III, Stage IV

Exclusion criteria variant classes: BRCA1 germline mutation (Breast Cancer, Ovarian Cancer), BRCA2 germline mutation (Breast Cancer, Ovarian Cancer)

Phase: II

Therapy: talazoparib

Location: United States

US State: TX

Contact: Dr. Sarina Piha-Paul [713-563-1930; spihapau@mdanderson.org]

NCT02401347
A Phase II Clinical Trial of the PARP Inhibitor Talazoparib in BRCA1 and BRCA2 Wild Type Patients With Advanced Triple Negative Breast Cancer and Homologous Recombination Deficiency or Advanced HER2 Negative Breast Cancer or Other Solid Tumors With a Mutation in Homologous Recombination Pathway Genes Talazoparib Beyond BRCA (TBB) Trial

Cancer type: Breast Cancer, Unspecified Solid Tumor

Variant class: ATM mutation

Other inclusion criteria: ERBB2 negative, Hormone receptor positive

Other identifiers: BRS0050, NCI-2015-00036, TBB

Population segments: Estrogen receptor positive, HER2 negative, Progesterone receptor positive, Second line, Stage III, Stage IV, Triple receptor negative

Exclusion criteria variant classes: BRCA1 germline mutation (Breast Cancer, Triple Negative Breast Cancer, Unspecified Solid Tumor), BRCA1 mutation (Breast Cancer, Triple Negative Breast Cancer, Unspecified Solid Tumor), BRCA2 germline mutation (Breast Cancer, Triple Negative Breast Cancer, Unspecified Solid Tumor), BRCA2 mutation (Breast Cancer, Triple Negative Breast Cancer, Unspecified Solid Tumor), ERBB2 positive (Breast Cancer)

Phase: II

Therapy: talazoparib

Location: United States

US State: CA

Contact: Amanda Selin [650-721-6977; aselin@stanford.edu]

NCT03611868
A Phase Ib/II Study of APG-115 in Combination With Pembrolizumab in Patients With Unresectable or Metastatic Melanomas or Advanced Solid Tumors

Cancer type: Unspecified Solid Tumor

Variant class: ATM mutation

Other inclusion criteria: TP53 wild type

Other identifiers: 2020-0136, APG-115-US-002, Keynote MK-3475-B66, NCI-2019-08846

Population segments: Adenocarcinoma, Fourth line or greater, STK11, Second line, Stage III, Stage IV, Third line

Exclusion criteria variant class: FGFR fusion (Bladder Urothelial Carcinoma)

Phase: I/II

Therapies: alrizomadlin, pembrolizumab

Locations: Australia, United States

US States: AR, AZ, CA, DC, FL, MO, NY, PA, TN, TX, VA

Contact: Kathryn Shantz [301-802-3414; kate.shantz@ascentage.com]

Disclaimer: The data presented here is from a curated knowledgebase of publicly available information, but may not be exhaustive. The data version is 2021.11(003).

ATM truncating mutation (continued)

NCT03842228
A Phase Ib Biomarker-Driven Combination Trial of Copanlisib, Olaparib, and Durvalumab (MEDI4736) in Patients With Advanced Solid Tumors

Cancer type: Unspecified Solid Tumor

Variant class: ATM mutation

Other identifiers: 10217, 19-628, NCI10217, NCI-2019-00601

Population segments: Line of therapy N/A, Stage III, Stage IV

Phase: I

Therapies: copanlisib, olaparib, durvalumab

Location: United States

US States: MA, TX

Contact: Multiple contacts: See www.clinicaltrials.gov for complete list of contacts.

NCT04586335
Open Label, Phase Ib Study to Evaluate the Safety, Tolerability, Pharmacokinetics and Clinical Activity of CYH33, an Oral a-specific PI3K Inhibitor in Combination With Olaparib, an Oral PARP Inhibitor in Patients With Advanced Solid Tumors.

Cancer type: Breast Cancer, Endometrial Carcinoma, Ovarian Cancer, Prostate Cancer, Unspecified Solid Tumor

Variant class: ATM mutation

Other identifiers: CTR20202339, CYH33-G102

Population segments: BRCA, Second line, Stage III, Stage IV

Phase: I

Therapies: HH-CYH33, olaparib

Locations: Australia, United States

US State: TX

Contact: Dr. Jason Sudia [908-380-1329; jason.sudia@haihepharma.com]

NCT04123366
A Phase II Study of Olaparib in Combination With Pembrolizumab in Participants With Previously Treated, Homologous Recombination Repair Mutation (HRRm) and/or Homologous Recombination Deficiency (HRD)-Positive Advanced Cancer

Cancer type: Unspecified Solid Tumor

Variant class: HRR mutation

Other identifiers: 7339-007, EudraCT Number: 2019-001745-40, jRCT2011200025, KEYLYNK-007, MK-7339-007, NCI-2019-08758, PER-029-19

Population segments: BRCA, Second line, Stage III, Stage IV

Exclusion criteria variant classes: BRCA germline mutation (Breast Cancer, Ovarian Cancer), BRCA mutation (Breast Cancer, Ovarian Cancer)

Phase: II

Therapies: olaparib, pembrolizumab

Locations: Argentina, Australia, Canada, Colombia, France, Germany, Guatemala, Israel, Italy, Japan, Latvia, Mexico, Peru, Poland, Puerto Rico, Republic of Korea, Romania, South Africa, Spain, Sweden, Turkey, Ukraine, United States

US States: AL, AZ, CA, CO, FL, GA, KY, NJ, NY, OH, TX, UT, VA

Contact: Toll Free Number [888-577-8839; Trialsites@merck.com]

Disclaimer: The data presented here is from a curated knowledgebase of publicly available information, but may not be exhaustive. The data version is 2021.11(003).

ATM truncating mutation (continued)

NCT04497116

Phase I/IIa Study of the Safety, Pharmacokinetics, Pharmacodynamics and Preliminary Clinical Activity of RP-3500 Alone or in Combination With Talazoparib in Advanced Solid Tumors With ATR Inhibitor Sensitizing Mutations (TRESR Study)

Cancer type: Unspecified Solid Tumor

Variant class: ATM aberration

Other identifiers: 20-521, EudraCT Number: 2020-000301-87, IRAS ID: 286609, NCI-2020-06869, RP-3500-01, TRESR, TRESR Study

Population segments: Second line, Stage III, Stage IV

Phase: I/II

Therapies: RP-3500, talazoparib

Locations: Canada, Denmark, United Kingdom, United States

US States: MA, NC, NY, RI, TN, TX

Contact: Dr. Peter Manley [857-322-5553; pmanley@reparerx.com]

NCT03415659

A Phase I, Open-label, Single-center, Single/Multiple-dose, Dose-escalation/ Dose-expansion Clinical Study on Tolerance and Pharmacokinetics of HWH340 Tablet in Patients With Advanced Solid Tumors

Cancer type: Unspecified Solid Tumor

Variant class: HRR mutation

Other inclusion criteria: BRCA1 germline mutation negative, BRCA1 mutation negative, BRCA2 germline mutation negative, BRCA2 mutation negative

Other identifiers: CTR20171649, HWH340-RFPA 20170821, RFPA 20170821

Population segments: BRCA, Fourth line or greater, HER2 negative, Second line, Stage III, Stage IV, Third line

Phase: I

Therapy: HWH-340

Location: China

NCT03767075

Basket of Baskets: A Modular, Open-label, Phase II, Multicentre Study To Evaluate Targeted Agents in Molecularly Selected Populations With Advanced Solid Tumours

Cancer type: Unspecified Solid Tumor

Variant class: DNA repair mutation

Other identifiers: (Basket of Baskets) (BoB), EudraCT number: 2017-005108-89, MO39164, NL65937.031.18, VHIO17002

Population segments: Second line, Stage III, Stage IV

Phase: II

Therapy: atezolizumab

Locations: France, Germany, Netherlands, Spain, Sweden, United Kingdom

---

NCT04905914

A Phase I/IIa, Open-Label, Safety, Pharmacokinetic, And Preliminary Efficacy Study Of Oral ATRN-119 In Patients With Advanced Solid Tumors

Cancer type: Unspecified Solid Tumor

Variant class: DNA repair mutation

Other identifier: AR-276-01

Population segments: Line of therapy N/A, Stage III, Stage IV

Phase: I/II

Therapy: ATRN-119

Location: United States

US State: MA

Contact: Robert Hasson [619-540-6253; rhasson@pacificlinkconsulting.com]

Disclaimer: The data presented here is from a curated knowledgebase of publicly available information, but may not be exhaustive. The data version is 2021.11(003).

ATM truncating mutation (continued)

NCT04901702

A Randomized Phase I/II Study of Talazoparib or Temozolomide in Combination With Onivyde in Children With Recurrent Solid Malignancies and Ewing Sarcoma

Cancer type: Unspecified Solid Tumor

Variant class: HRR pathway

Other identifiers: EudraCT Number: 2021-003570-31, ONITT

Population segments: Second line, Stage IV

Exclusion criteria variant classes: HRR germline pathway (Central Nervous System Neoplasm, Ewing Sarcoma), HRR pathway (Central Nervous System Neoplasm, Ewing Sarcoma)

Phase: I/II

Therapies: talazoparib, chemotherapy

Locations: Canada, United States

US State: TN

Contact: Dr. Sara Federico [866-278-5833; referralinfo@stjude.org]

NCT03155620

NCI-COG Pediatric MATCH (Molecular Analysis for Therapy Choice) Screening Protocol

Cancer type: Unspecified Solid Tumor

Variant class: DNA repair pathway

Other identifiers: 17-729, 2017-0625, 20170841, APEC1621SC, COGAPEC1621, COGAPEC1621SC, N 55017 SC, NCI-2017-01251, NCI-COG Pediatric MATCH, Pediatric MATCH

Population segments: ALK, Aggressive, BRAF, FGFR, HRAS, Indolent, KRAS, NRAS, Pediatric or Adolescent, Second line, Stage III, Stage IV

Phase: II

Therapy: olaparib

Locations: Puerto Rico, United States

US States: AK, AL, AR, AZ, CA, CO, CT, DC, DE, FL, GA, HI, IA, ID, IL, IN, KY, LA, MA, MD, ME, MI, MN, MO, MS, NC, NE, NH, NJ, NV, NY, OH, OK, OR, PA, RI, SC, SD, TN, TX, UT, VA, VT, WA, WI

Contact: Multiple contacts: See www.clinicaltrials.gov for complete list of contacts.

NCT03233204

NCI-COG Pediatric MATCH (Molecular Analysis for Therapy Choice)- Phase 2 Subprotocol of Olaparib in Patients With Tumors Harboring Defects in DNA Damage Repair Genes

Cancer type: Unspecified Solid Tumor

Variant class: DNA repair pathway

Other identifiers: 20170841, APEC1621H, NCI-2017-00766

Population segments: Aggressive, Indolent, Pediatric or Adolescent, Second line, Stage III, Stage IV

Phase: II

Therapy: olaparib

Locations: Puerto Rico, United States

US States: AK, AL, AR, AZ, CA, CO, DC, DE, FL, GA, IA, ID, IL, IN, KY, LA, MD, ME, MI, MN, MO, MS, NC, ND, NE, NJ, NY, OH, OK, OR, PA, SC, SD, TN, TX, UT, VA, VT, WA, WI

Contact: Multiple contacts: See www.clinicaltrials.gov for complete list of contacts.

ATM truncating mutation (continued)

NCT04267939
An Open-label Phase Ib Study to Determine the Maximum Tolerated and/or Recommended Phase II Dose of the ATR Inhibitor BAY 1895344 in Combination With PARP Inhibitor Niraparib, in Patients With Recurrent Advanced Solid Tumors and Ovarian Cancer

Cancer type: Ovarian Cancer, Unspecified Solid Tumor

Variant class: DNA repair pathway

Other identifiers: 18595, 19-810, 2019-0471, 20-222, EudraCT Number: 2018-003930-34, NCI-2020-01405

Population segments: Maintenance/Consolidation, Second line, Stage III, Stage IV

Exclusion criteria variant class: DNA repair pathway (Prostate Cancer)

Phase: I

Therapies: BAY-1895344, niraparib

Location: United States

US States: MA, NY, TX

Contact: Bayer Clinical Trials Contact [888-842-2937; clinical-trials-contact@bayer.com]

NCT04693468
Modular Phase IB Hypothesis-Testing, Biomarker-Driven, Talazoparib Combination Trial (TalaCom)

Cancer type: Unspecified Solid Tumor

Variant class: DNA repair pathway

Other identifiers: 2020-0436, NCI-2020-06041, TalaCom

Population segments: Hormone refractory, Second line, Stage III, Stage IV

Phase: I

Therapies: talazoparib, palbociclib, axitinib, crizotinib

Location: United States

US State: TX

Contact: Timothy A. Yap [713-563-1784; tyap@mdanderson.org]

NCT03810105
A Phase II Study of Olaparib and Durvalumab in Men With Castration Sensitive Biochemically Recurrent Non-Metastatic Prostate Cancer Harboring Mutations in DNA Damage Repair

Cancer type: Prostate Cancer

Variant class: ATM truncating mutation or ATM mutation

Other identifiers: 18-480, NCI-2019-01627

Population segments: Adjuvant, Stage I, Stage II, Stage III

Phase: II

Therapies: durvalumab, olaparib

Location: United States

US States: CA, IL, MI, NJ, NY

Contact: Dr. Karen Autio [646-422-4632; autiok@mskcc.org]

Disclaimer: The data presented here is from a curated knowledgebase of publicly available information, but may not be exhaustive. The data version is 2021.11(003).

ATM truncating mutation (continued)

NCT03840967
A Phase II Study Evaluating Safety and Efficacy of Niraparib in Patients With Previously Treated Homologous Recombination (HR) Defective or Loss of Heterozygosity (LOH) High Metastatic Esophageal/Gastroesophageal Junction/Proximal Gastric Adenocarcinoma

Cancer type: Esophageal Cancer, Gastric Cancer, Gastroesophageal Junction Adenocarcinoma

Variant class: ATM truncating mutation

Other identifiers: 20166, 71618, HCRN ESO17-325, NCI-2019-07395

Population segments: Second line, Stage III, Stage IV

Phase: II

Therapy: niraparib

Location: United States

US States: FL, IN, MI, NY

Contact: Dr. Shadia Jalal [317-274-3658; sjalal@iu.edu]

NCT02975934
TRITON3: A Multicenter, Randomized, Open Label Phase III Study of Rucaparib Versus Physician's Choice of Therapy for Patients With Metastatic Castration Resistant Prostate Cancer Associated With Homologous Recombination Deficiency

Cancer type: Castration-Resistant Prostate Cancer

Variant class: ATM mutation

Other identifiers: 16238, 1723GCCC, 18-146, 18-300, 20171185, 2018IS040, CO-338-063, CSET 2620, EudraCT Number: 2016-003163-20, GU 146, IGUP17020, IRAS ID: 222298, NCI-2017-01110, S16-02260, STAR Trial, TRITON 3_CO-338-063, TRITON3

Population segments: BRCA, First line, Hormone refractory, Stage IV

Phase: III

Therapies: rucaparib, hormone therapy

Locations: Australia, Belgium, Canada, Denmark, France, Germany, Ireland, Israel, Italy, Spain, United Kingdom, United States

US States: AZ, CA, CO, CT, DE, FL, HI, MD, MN, NC, NY, OH, OR, TN, TX, VA, WA

Contact: Clovis Oncology For North America [844-258-7662; medinfo@clovisoncology.com]

NCT04336943
Durvalumab (MEDI4736) and Olaparib (AZD2281) for Treatment of Biochemically Recurrent Prostate Cancer in Men Predicted to Have a High Neoantigen Load: A Pilot Study

Cancer type: Prostate Adenocarcinoma

Variant class: ATM mutation

Other identifiers: 10509, NCI-2020-01860, RG1007001

Population segments: Line of therapy N/A, Stage I, Stage II, Stage III

Phase: II

Therapies: durvalumab, olaparib

Location: United States

US State: WA

Contact: Michael Schweizer [206-606-6252; schweize@uw.edu]

Disclaimer: The data presented here is from a curated knowledgebase of publicly available information, but may not be exhaustive. The data version is 2021.11(003).

ATM truncating mutation (continued)

NCT04053322
An International Multicenter Phase II Trial of Durvalumab (MEDI4736) Plus OLAparib Plus Fulvestrant in Metastatic or Locally Advanced ER-positive, HER2-negative Breast Cancer Patients Selected Using Criteria That Predict Sensitivity to Olaparib

Cancer type: Breast Cancer

Variant class: ATM mutation

Other inclusion criteria: ERBB2 negative, ER positive

Other identifiers: CADUSEIME08, DOLAF, EudraCT Number: 2018-003832-57, UC-0140/1812

Population segments: BRCA, Estrogen receptor positive, FGFR, HER2 negative, Progesterone receptor positive, Second line, Stage III, Stage IV

Phase: II

Therapies: durvalumab, olaparib, hormone therapy

Location: France

NCT05011383
High-dose Testosterone in Men With Metastatic Castration-resistant Prostate Cancer and ATM or CDK12 Deficiency

Cancer type: Castration-Resistant Prostate Cancer

Variant class: ATM mutation

Other identifiers: SPLP-003-20F, VA-BAT

Population segments: Hormone refractory, Second line, Stage IV

Phase: II

Therapy: hormone therapy

Location: United States

US State: WA

Contact: Dr. Robert B. Montgomery [206-277-6878; Robert.Montgomery@va.gov]

NCT03012321
BRCAAway: A Randomized Phase II Trial of Abiraterone, Olaparib, or Abiraterone + Olaparib in Patients With Metastatic Castration-Resistant Prostate Cancer With DNA Repair Defects

Cancer type: Castration-Resistant Prostate Cancer

Variant class: ATM mutation

Other identifiers: 19137, BRCAAway, NCI-2016-01834, NU 16U05, NU_16U05, PCCTC #: c16-168, PCCTC c16-168, STU00203960

Population segments: First line, Hormone refractory, Stage IV

Phase: II

Therapies: hormone therapy, olaparib, steroid

Location: United States

US States: CA, FL, IL, IN, MD, MI, MN, MO, NC, NY, UT, VA

Contact: Study Coordinator [312-695-1301; cancertrials@northwestern.edu]

Disclaimer: The data presented here is from a curated knowledgebase of publicly available information, but may not be exhaustive. The data version is 2021.11(003).

ATM truncating mutation (continued)

NCT03553004
Niraparib in Metastatic Pancreatic Cancer After Previous Chemotherapy (NIRA-PANC): A Phase II Trial

Cancer type: Pancreatic Cancer

Variant class: ATM mutation

Other identifiers: IIT-2017-NIRA-PANC, NIRA-PANC, STUDY00141862

Population segments: BRCA, Second line, Stage IV, Third line

Phase: II

Therapy: niraparib

Location: United States

US States: KS, MO

Contact: Clinical Trials Nurse Navigator [913-588-3671; navigation@kumc.edu]

NCT03601923
Phase II Proof-of-Concept Trial Testing the PARP Inhibitor Niraparib in Patients With Pancreatic Cancer Harboring Deficiencies in Homologous Recombination DNA Repair

Cancer type: Pancreatic Cancer

Variant class: ATM mutation

Other identifiers: 18-207, NCI-2018-02395

Population segments: Second line, Stage IV

Phase: II

Therapy: niraparib

Location: United States

US State: MA

Contact: Dr. James Clreary [617-632-5960; JCLEARY@PARTNERS.ORG]

NCT03925350
A Phase II Study of Niraparib in Patients With Advanced Melanoma With Genetic Homologous Recombination (HR) Mutation / Alteration

Cancer type: Melanoma

Variant class: ATM mutation

Other identifier: CPMC17-MEL01

Population segments: Second line, Stage III, Stage IV

Phase: II

Therapy: niraparib

Location: United States

US State: CA

Contact: Laurel Brechtel [415-600-1654; BrechtLA@sutterhealth.org]

NCT04030559
Phase II Trial of PARP Inhibitor Niraparib for Men With High Risk Prostate Cancer and DNA Damage Response Defects

Cancer type: Prostate Cancer

Variant class: ATM mutation

Other identifiers: 1412676, NCI-2019-04365, UCDCC#279

Population segments: First line, Neoadjuvant, Stage I, Stage II, Stage IV

Phase: II

Therapy: niraparib

Location: United States

US State: CA

Contact: Marc Dall'Era [916-734-3771; mdallera@ucdavis.edu]

Disclaimer: The data presented here is from a curated knowledgebase of publicly available information, but may not be exhaustive. The data version is 2021.11(003).

ATM truncating mutation (continued)

NCT04187833
Phase II Trial of Nivolumab in Combination with Talazoparib in Patients with Unresectable or Metastatic Melanoma and Mutations in BRCA or BRCA-ness Genes.

Cancer type: Melanoma

Variant class: ATM mutation

Other identifiers: CASE2619, NCI-2020-00416

Population segments: Second line, Stage III, Stage IV

Phase: II

Therapies: nivolumab, talazoparib

Location: United States

US State: OH

Contact: Dr. Pauline Funchain [866-223-8100; TaussigResearch@ccf.org]

NCT03344965
A Phase II Study of Olaparib Monotherapy in Metastatic Breast Cancer Patients With Germline or Somatic Mutations in DNA Repair Genes (Olaparib Expanded)

Cancer type: Breast Cancer

Variant class: ATM mutation

Other identifiers: 17-428, 18-414, NCI-2018-00778, TBCRC 048, TBCRC048

Population segments: BRCA, Estrogen receptor positive, HER2 negative, HER2 positive, Progesterone receptor positive, Second line, Stage IV

Exclusion criteria variant classes: BRCA1 germline mutation (Breast Cancer), BRCA2 germline mutation (Breast Cancer)

Phase: II

Therapy: olaparib

Location: United States

US States: AL, CA, IL, MA, MD, NC, NJ, NY, PA, WA

Contact: Dr. Nadine Tung [617-667-1962; ntung@bidmc.harvard.edu]

NCT03375307
A Phase II Study of Olaparib (AZD2281) in Patients With Metastatic/Advanced Urothelial Carcinoma With DNA-Repair Defects

Cancer type: Bladder Urothelial Carcinoma

Variant class: ATM mutation

Other identifiers: 10144, 19C0023, NCI-2017-02296, P162941

Population segments: BRCA, STK11, Second line, Stage III, Stage IV

Phase: II

Therapy: olaparib

Location: United States

US States: CA, CO, KY, MD, NY, OK

Contact: Multiple contacts: See www.clinicaltrials.gov for complete list of contacts.

Disclaimer: The data presented here is from a curated knowledgebase of publicly available information, but may not be exhaustive. The data version is 2021.11(003).

ATM truncating mutation (continued)

NCT03786796
Phase II Study of Olaparib in Metastatic Renal Cell Carcinoma Patients Harboring a BAP-1 or Other DNA Repair Gene Mutations (ORCHID)

Cancer type: Kidney Cancer

Variant class: ATM mutation

Other identifiers: CRMS-70750, J18166, NCI-2019-06309, ORCHID

Population segments: Fourth line or greater, Second line, Stage IV, Third line

Phase: II

Therapy: olaparib

Location: United States

US State: MD

Contact: Irina Rifkind [410-502-2043; irifkin1@jhmi.edu]

NCT04042831
A Phase II Study of Olaparib in Patients With Advanced Biliary Tract Cancer With Aberrant DNA Repair Gene Mutations

Cancer type: Biliary Tract Carcinoma

Variant class: ATM mutation

Other identifiers: ACCRU-ICRN-1702, NCI-2019-04434

Population segments: Second line, Stage IV

Phase: II

Therapy: olaparib

Location: United States

US States: AZ, MN, NY, TX

Contact: Multiple contacts: See www.clinicaltrials.gov for complete list of contacts.

NCT04515836
Phase II Trial of Olaparib in Homologous Recombination Deficient (HRD) Malignant Mesothelioma

Cancer type: Mesothelioma

Variant class: ATM mutation

Population segments: (N/A), Second line

Phase: II

Therapy: olaparib

Location: United States

US State: IL

Contact: Dr. Hedy L. Kindler [773-702-0360; hkindler@medicine.bsd.uchicago.edu]

NCT04633902
Phase II Study of Olaparib in Combination With Pembrolizumab in Patients With Advanced Melanoma With Homologous Recombination (HR) Pathway Gene Mutation

Cancer type: Melanoma

Variant class: ATM mutation

Other identifier: CPMC19-MEL01

Population segments: BRAF, Second line, Stage III, Stage IV

Phase: II

Therapies: olaparib, pembrolizumab

Location: United States

US State: CA

Contact: Dr. Kevin B. Kim [415-885-8600; ClinicalResearch@sutterhealth.org]

Disclaimer: The data presented here is from a curated knowledgebase of publicly available information, but may not be exhaustive. The data version is 2021.11(003).

ATM truncating mutation (continued)

NCT03248570
Phase II Open Label Study of Pembrolizumab in Patients With Metastatic Castration Resistant Prostate Cancer (mCRPC) With or Without DNA Damage Repair Defects

Cancer type: Castration-Resistant Prostate Cancer

Variant class: ATM mutation

Other identifiers: 16557, 17-22165, CC 16557, NCI-2017-02408

Population segments: BRCA, Hormone refractory, Line of therapy N/A, Stage IV

Phase: II

Therapy: pembrolizumab

Location: United States

US State: CA

Contact: Patricia Li [415-476-5975; GUTrials@ucsf.edu]

NCT03506997
PERSEUS1: Phase II Trial of the Immune Checkpoint Inhibitor Pembrolizumab for Patients Suffering From Metastatic Prostate Cancer

Cancer type: Castration-Resistant Prostate Cancer

Variant class: ATM mutation

Other identifiers: CCR4559, EudraCT Number: 2017-000931-15, ICR-CTSU/2016/10060, IRAS ID 208952, PERSEUS1

Population segments: Hormone refractory, Second line, Stage IV

Phase: II

Therapy: pembrolizumab

Location: United Kingdom

NCT03533946
A Phase II Study of Rucaparib Monotherapy in Nonmetastatic, Hormone-Sensitive Prostate Cancer Demonstrating "BRCAness" Genotype (ROAR)

Cancer type: Prostate Cancer

Variant class: ATM mutation

Other identifiers: HCI111833, NCI-2018-01754, ROAR

Population segments: First line, Stage I, Stage II, Stage III

Phase: II

Therapy: rucaparib

Location: United States

US State: UT

Contact: Jill Broghammer [801-213-6232; jill.broghammer@hci.utah.edu]

NCT04892693
Talazoparib in Advanced Breast Cancer Patients With Homologous Recombinant Deficiency: A Phase II Clinical and Exploratory Biomarker Study of Talazoparib

Cancer type: Breast Cancer

Variant class: ATM mutation

Other identifier: H-2009-079-1157

Population segments: BRCA, Line of therapy N/A, Stage III, Stage IV

Phase: II

Therapy: talazoparib

Location: Republic of Korea

Disclaimer: The data presented here is from a curated knowledgebase of publicly available information, but may not be exhaustive. The data version is 2021.11(003).

ATM truncating mutation (continued)

NCT04740190
Combination Talazoparib - Carboplatin for Recurrent High-grade Glioma With DNA Damage Repair Deficiency (DDRd)

Cancer type: Anaplastic Astrocytoma, Anaplastic Oligoastrocytoma, Anaplastic Oligodendroglioma, Anaplastic Pleomorphic Xanthoastrocytoma, Diffuse Midline Glioma, Glioblastoma

Variant class: ATM mutation

Other identifiers: TAC-GReD, UW-20396

Population segments: (N/A), BRCA, Second line

Phase: II

Therapies: talazoparib, chemotherapy

Location: Hong Kong

NCT04253262
A Phase Ib/II Study of Copanlisib Combined With Rucaparib in Patients With Metastatic Castration-resistant Prostate Cancer

Cancer type: Castration-Resistant Prostate Cancer

Variant class: ATM mutation

Other identifiers: BrUOG 360, BrUOG360

Population segments: Hormone refractory, Second line, Stage IV

Phase: I/II

Therapies: copanlisib, rucaparib

Location: United States

US State: RI

Contact: BrUOG [401-863-3000; BrUOG@brown.edu]

NCT04095273
A Multicenter, Non-randomized, Open-label Phase Ib Study to Determine the Maximum Tolerated and Recommended Phase II Dose of the ATR Inhibitor BAY1895344 in Combination With Pembrolizumab and to Characterize Its Safety, Tolerability, Pharmacokinetics and Preliminary Anti-tumor Activity in Patients With Advanced Solid Tumors

Cancer type: Breast Cancer

Variant class: ATM mutation

Other inclusion criteria: ERBB2 negative, Hormone receptor positive

Other identifiers: 19741, EudraCT Number: 2018-003420-36, Keynote 919, NCI-2019-06734

Population segments: HER2 negative, Hormone refractory, Second line, Stage III, Stage IV

Phase: I

Therapies: BAY-1895344, pembrolizumab

Locations: Germany, Spain, Switzerland, United States

US States: CA, CT, MA, MD, NC, NY, OH, TX

Contact: Bayer Clinical Trials Contact [clinical-trials-contact@bayer.com]

Disclaimer: The data presented here is from a curated knowledgebase of publicly available information, but may not be exhaustive. The data version is 2021.11(003).

ATM truncating mutation (continued)

NCT04169841

Precision Medicine Phase II Study Evaluating the Efficacy of a Double Immunotherapy by Durvalumab and Tremelimumab Combined With Olaparib in Patients With Solid Cancers and Carriers of Homologous Recombination Repair Genes Mutation in Response or Stable After Olaparib Treatment

Cancer type: Breast Cancer, Endometrial Carcinoma, Head and Neck Cancer, Kidney Cancer, Non-Small Cell Lung Cancer, Ovarian Cancer, Pancreatic Cancer, Prostate Cancer

Variant class: HRR mutation

Other identifier: GUIDE2REPAIR

Population segments: Fourth line or greater, Hormone refractory, Maintenance/ Consolidation, Second line, Stage III, Stage IV, Third line

Exclusion criteria variant classes: ALK fusion (Lung Cancer), BRAF mutation (Lung Cancer), EGFR mutation (Lung Cancer), ROS1 mutation (Lung Cancer)

Phase: II

Therapies: durvalumab, tremelimumab, olaparib

Location: France

NCT04940637

A Phase II, Open-Label, Single Arm, Prospective, Multicenter Study of Niraparib Plus Dostarlimab in Patients With Advanced NSCLC and/or MPM, and Positive for PD-L1 Expression and Germline or Somatic Mutations in the HRR Genes

Cancer type: Mesothelioma, Non-Small Cell Lung Cancer

Variant class: HRR mutation

Other inclusion criteria: ALK fusion negative, CD274 expression, EGFRi sensitizing mutation negative, ROS1 fusion negative or CD274 expression

Other identifiers: EudraCT Number: 2020-000109-10, UNITO-001, UNITO-001/2020

Population segments: Second line, Stage III, Stage IV

Exclusion criteria variant classes: ALK fusion (Non-Small Cell Lung Cancer), EGFRi sensitizing mutation (Non-Small Cell Lung Cancer), ROS1 fusion (Non-Small Cell Lung Cancer)

Phase: II

Therapies: niraparib, dostarlimab

Location: Italy

NCT04939662

Phase II, Single-arm Study of Olaparib and Bevacizumab Combination Therapy in Relapsed Small Cell Lung Cancer Subjects With DNA Damage Response and the Repair Pathway Alteration, ATM Deficiency, SLFN11 Positive, or POU2F3 Positive

Cancer type: Small Cell Lung Cancer

Variant class: HRR mutation

Other identifier: SUKSES-B2

Population segments: (N/A), BRCA, Pulmonary, Second line, Third line

Phase: II

Therapies: olaparib, bevacizumab

Location: Republic of Korea

ATM truncating mutation (continued)

NCT04666740

A Phase II Trial to Evaluate the Safety and Antitumor Activity of Pembrolizumab and OLApaRib (POLAR) Maintenance for Patients With Metastatic Pancreatic Ductal Adenocarcinoma and Homologous Recombination Deficiency and/or Exceptional Treatment Response to Platinum-Based Therapy

Cancer type: Pancreatic Cancer

Variant class: ATM aberration

Other identifier: 20-481

Population segments: BRCA, Maintenance/Consolidation, Stage IV

Phase: II

Therapies: pembrolizumab, olaparib

Location: United States

US States: NJ, NY

Contact: Dr. Wungki Park [646-888-4543; yaqubiea@mskcc.org]

NCT02264678

A Modular Phase I, Open-Label, Multicentre Study to Assess the Safety, Tolerability, Pharmacokinetics and Preliminary Anti-tumour Activity of Ceralasertib in Combination With Cytotoxic Chemotherapy and/or DNA Damage Repair/Novel Anti-cancer Agents in Patients With Advanced Solid Malignancies

Cancer type: Triple Negative Breast Cancer

Variant class: HRR mutation

Other inclusion criteria: BRCA germline mutation negative, BRCA mutation negative

Other identifiers: 0C-14-9, 16-031, 18-342, D5330C00004, EudraCT Number: 2014-002233-66, IRAS ID 160200, NCI-2015-00687, Study 4

Population segments: Adenocarcinoma, BRCA, HER2 negative, Pulmonary, Second line, Stage III, Stage IV, Third line, Triple receptor negative

Exclusion criteria variant class: ERBB2 positive (Breast Cancer)

Phase: I

Therapies: ceralasertib, olaparib

Locations: France, Republic of Korea, United Kingdom, United States

US States: CA, MA, NY

Contact: AstraZeneca Clinical Study Information Center [877-240-9479; information.center@astrazeneca.com]

NCT04890613

Phase Ib Expansion Study of CX-5461 in Patients With Solid Tumours and BRCA2 and/or PALB2 Mutation

Cancer type: Ovarian Cancer

Variant class: HRR mutation

Other identifier: CX-5461-04

Population segments: BRCA, Second line, Stage II, Stage III, Stage IV

Phase: I

Therapy: pidnarulex

Location: Canada

Disclaimer: The data presented here is from a curated knowledgebase of publicly available information, but may not be exhaustive. The data version is 2021.11(003).

ATM truncating mutation (continued)

NCT05002868

A Multi-center, Open-label, Phase I/Ib Study to Assess the Safety, Pharmacokinetics and Anti-tumor Activity of RP12146, a Poly (ADP-ribose) Polymerase (PARP) Inhibitor, in Patients With Locally Advanced or Metastatic Solid Tumors

Cancer type: Biliary Tract Carcinoma, Breast Cancer, Colorectal Cancer, Endometrial Carcinoma, Gastric Cancer, Ovarian Cancer, Pancreatic Cancer, Prostate Cancer, Small Cell Lung Cancer

Variant class: HRR mutation

Other identifier: RP12146-2101

Population segments: Extensive, Pulmonary, Second line, Stage III, Stage IV

Exclusion criteria variant class: ERBB2 positive (Breast Cancer)

Phase: I

Therapy: RP12146

Location: Poland

NCT03395197

A Phase III, Randomized, Double-blind, Placebo-controlled Study Of Talazoparib With Enzalutamide In Metastatic Castration-resistant Prostate Cancer

Cancer type: Castration-Resistant Prostate Cancer

Variant class: DNA repair mutation

Other identifiers: BASIC-ROYAL / HIGH-ROYAL, C3441021, CSET 2938, CTR20191443, CTR20191444, EudraCT Number: 2017-003295-31, GU 170 - C3441021, HCC 19-054, JapicCTI-194665, LH19.022, MOH_2019-05-16_007127, NCI-2018-01651, Pfizer C3441021, R19062M, RECF3951, RWF_C3441021 TALAPRO-2, TALAPRO-2, TX228665, UCI-18-47, VICCURO17115

Population segments: First line, Hormone refractory, Second line, Stage III, Stage IV

Phase: III

Therapies: talazoparib, hormone therapy

Locations: Argentina, Australia, Belgium, Brazil, Canada, Chile, China, Czech Republic, Finland, France, Germany, Hungary, Israel, Italy, Japan, New Zealand, Norway, Peru, Poland, Portugal, Republic of Korea, South Africa, Spain, Sweden, United Kingdom, United States

US States: AZ, CA, CO, FL, GA, IL, IN, KS, MO, NE, NJ, NY, OH, OK, OR, PA, SC, TN, TX, UT, VA, WI

Contact: Pfizer CT.gov Call Center [800-718-1021; ClinicalTrials.gov_Inquiries@pfizer.com]

NCT04821622

TALAPRO-3: A Phase III, Randomized, Double-blind, Study of Talazoparib With Enzalutamide Versus Placebo With Enzalutamide in Men With DDR Gene Mutated Metastatic Castration-sensitive Prostate Cancer

Cancer type: Prostate Adenocarcinoma

Variant class: DNA repair mutation

Other identifiers: C3441052, CTR20212191, EudraCT Number: 2021-000248-23, jRCT2031210098, TALAPRO-3

Population segments: Second line, Stage IV

Phase: III

Therapies: talazoparib, hormone therapy

Locations: Japan, Republic of Korea, United States

US States: CA, DC, FL, GA, IL, MI, NJ, NY, PA, SC, TX, UT

Contact: Pfizer CT.gov Call Center [800-718-1021; ClinicalTrials.gov_Inquiries@pfizer.com]

Disclaimer: The data presented here is from a curated knowledgebase of publicly available information, but may not be exhaustive. The data version is 2021.11(003).

ATM truncating mutation (continued)

NCT04038502
An Open-label, Multicenter Phase II Study to Compare the Efficacy of Carboplatin as First-line Followed by Second-line Olaparib Versus Olaparib as First-line Followed by Second-line Carboplatin in the Treatment of Patients With Castration Resistant Prostate Cancer Containing Homologous Recombination Deficiency

Cancer type: Castration-Resistant Prostate Cancer

Variant class: DNA repair mutation

Other identifiers: 01781, 01783, 19-08, BRACeD, CX001963-01, ONCA-009-18F

Population segments: First line, Hormone refractory, Second line, Stage IV

Phase: II

Therapies: olaparib, chemotherapy

Location: United States

US States: CA, DC, FL, IL, MI, NC, NY, PA, WA

Contact: Dr. Robert B. Montgomery [206-277-6878; rbmontgo@u.washington.edu]

No NCT ID - see other identifier(s)
Single-arm Exploratory Study for Sintilimab Second-line or Later-line Treatment of Advanced Non-small Cell Lung Cancer with DDR Pathway Gene Mutation

Cancer type: Non-Small Cell Lung Cancer

Variant class: DNA repair mutation

Other identifier: ChiCTR1900023234

Population segments: Fourth line or greater, Second line, Stage III, Stage IV, Third line

Exclusion criteria variant classes: ALK fusion (Non-Small Cell Lung Cancer), EGFRi sensitizing mutation (Non-Small Cell Lung Cancer), ROS1 fusion (Non-Small Cell Lung Cancer)

Phase: I/II

Therapy: sintilimab

Location: China

NCT04849364
A Phase II Circulating Tumor DNA Enriched, Genomically Directed Post-neoadjuvant Trial for Patients With Residual Triple Negative Breast Cancer (PERSEVERE)

Cancer type: Triple Negative Breast Cancer

Variant class: DNA repair pathway

Other identifiers: HCRN BRE18-334, PERSEVERE

Population segments: Adjuvant, HER2 negative, Stage I, Stage II, Stage III, Triple receptor negative

Phase: II

Therapies: talazoparib, chemotherapy

Location: United States

US State: IN

Contact: Dr. Bryan P. Schneider [317-948-3885; bpschnei@iu.edu]

Disclaimer: The data presented here is from a curated knowledgebase of publicly available information, but may not be exhaustive. The data version is 2021.11(003).

ATM deletion

NCT02693535
Targeted Agent and Profiling Utilization Registry (TAPUR) Study.

Cancer type: Unspecified Solid Tumor

Variant class: ATM deletion

Other identifiers: 20170529, NCI-2017-00510, Pro00014171, TAPUR

Population segments: Aggressive, BRAF, Diffuse large B-cell lymphoma (DLBCL), Extranodal marginal zone B-cell lymphoma (MALT), FGFR, First line, Follicular lymphoma (FL), Fourth line or greater, Indolent, Lymphoblastic lymphoma (LBL), Mantle cell lymphoma (MCL), Other subtype, Second line, Small lymphocytic lymphoma (SLL), Stage III, Stage IV, Third line, Waldenstrom's macroglobulinemia (WM)

Phase: II

Therapy: olaparib

Location: United States

US States: AL, AZ, CA, FL, GA, HI, IL, IN, ME, MI, NC, ND, NE, NH, OH, OR, PA, SD, TX, UT, VA, WA

Contact: Pam Mangat [pam.mangat@asco.org]

NCT02286687
Phase II Study of the PARP Inhibitor Talazoparib in Advanced Cancer Patients With Somatic Alterations in BRCA1/2, Mutations/Deletions in Other BRCA Pathway Genes and Germline Mutation in BRCA1/2 (Not Breast or Ovarian Cancer)

Cancer type: Unspecified Cancer

Variant class: ATM deletion

Other identifiers: 2013-0961, NCI-2014-02494

Population segments: Fourth line or greater, Stage III, Stage IV

Exclusion criteria variant classes: BRCA1 germline mutation (Breast Cancer, Ovarian Cancer), BRCA2 germline mutation (Breast Cancer, Ovarian Cancer)

Phase: II

Therapy: talazoparib

Location: United States

US State: TX

Contact: Dr. Sarina Piha-Paul [713-563-1930; spihapau@mdanderson.org]

NCT04497116
Phase I/IIa Study of the Safety, Pharmacokinetics, Pharmacodynamics and Preliminary Clinical Activity of RP-3500 Alone or in Combination With Talazoparib in Advanced Solid Tumors With ATR Inhibitor Sensitizing Mutations (TRESR Study)

Cancer type: Unspecified Solid Tumor

Variant class: ATM aberration

Other identifiers: 20-521, EudraCT Number: 2020-000301-87, IRAS ID: 286609, NCI-2020-06869, RP-3500-01, TRESR, TRESR Study

Population segments: Second line, Stage III, Stage IV

Phase: I/II

Therapies: RP-3500, talazoparib

Locations: Canada, Denmark, United Kingdom, United States

US States: MA, NC, NY, RI, TN, TX

Contact: Dr. Peter Manley [857-322-5553; pmanley@reparerx.com]

ATM deletion (continued)

NCT04901702
A Randomized Phase I/II Study of Talazoparib or Temozolomide in Combination With Onivyde in Children With Recurrent Solid Malignancies and Ewing Sarcoma

Cancer type: Unspecified Solid Tumor

Variant class: HRR pathway

Other identifiers: EudraCT Number: 2021-003570-31, ONITT

Population segments: Second line, Stage IV

Exclusion criteria variant classes: HRR germline pathway (Central Nervous System Neoplasm, Ewing Sarcoma), HRR pathway (Central Nervous System Neoplasm, Ewing Sarcoma)

Phase: I/II

Therapies: talazoparib, chemotherapy

Locations: Canada, United States

US State: TN

Contact: Dr. Sara Federico [866-278-5833; referralinfo@stjude.org]

NCT03155620
NCI-COG Pediatric MATCH (Molecular Analysis for Therapy Choice) Screening Protocol

Cancer type: Unspecified Solid Tumor

Variant class: DNA repair pathway

Other identifiers: 17-729, 2017-0625, 20170841, APEC1621SC, COGAPEC1621, COGAPEC1621SC, N 55017 SC, NCI-2017-01251, NCI-COG Pediatric MATCH, Pediatric MATCH

Population segments: ALK, Aggressive, BRAF, FGFR, HRAS, Indolent, KRAS, NRAS, Pediatric or Adolescent, Second line, Stage III, Stage IV

Phase: II

Therapy: olaparib

Locations: Puerto Rico, United States

US States: AK, AL, AR, AZ, CA, CO, CT, DC, DE, FL, GA, HI, IA, ID, IL, IN, KY, LA, MA, MD, ME, MI, MN, MO, MS, NC, NE, NH, NJ, NV, NY, OH, OK, OR, PA, RI, SC, SD, TN, TX, UT, VA, VT, WA, WI

Contact: Multiple contacts: See www.clinicaltrials.gov for complete list of contacts.

NCT03233204
NCI-COG Pediatric MATCH (Molecular Analysis for Therapy Choice)- Phase 2 Subprotocol of Olaparib in Patients With Tumors Harboring Defects in DNA Damage Repair Genes

Cancer type: Unspecified Solid Tumor

Variant class: DNA repair pathway

Other identifiers: 20170841, APEC1621H, NCI-2017-00766

Population segments: Aggressive, Indolent, Pediatric or Adolescent, Second line, Stage III, Stage IV

Phase: II

Therapy: olaparib

Locations: Puerto Rico, United States

US States: AK, AL, AR, AZ, CA, CO, DC, DE, FL, GA, IA, ID, IL, IN, KY, LA, MD, ME, MI, MN, MO, MS, NC, ND, NE, NJ, NY, OH, OK, OR, PA, SC, SD, TN, TX, UT, VA, VT, WA, WI

Contact: Multiple contacts: See www.clinicaltrials.gov for complete list of contacts.

Disclaimer: The data presented here is from a curated knowledgebase of publicly available information, but may not be exhaustive. The data version is 2021.11(003).

ATM deletion (continued)

NCT04267939
An Open-label Phase Ib Study to Determine the Maximum Tolerated and/or Recommended Phase II Dose of the ATR Inhibitor BAY 1895344 in Combination With PARP Inhibitor Niraparib, in Patients With Recurrent Advanced Solid Tumors and Ovarian Cancer

Cancer type: Ovarian Cancer, Unspecified Solid Tumor

Variant class: DNA repair pathway

Other identifiers: 18595, 19-810, 2019-0471, 20-222, EudraCT Number: 2018-003930-34, NCI-2020-01405

Population segments: Maintenance/Consolidation, Second line, Stage III, Stage IV

Exclusion criteria variant class: DNA repair pathway (Prostate Cancer)

Phase: I

Therapies: BAY-1895344, niraparib

Location: United States

US States: MA, NY, TX

Contact: Bayer Clinical Trials Contact [888-842-2937; clinical-trials-contact@bayer.com]

NCT04693468
Modular Phase IB Hypothesis-Testing, Biomarker-Driven, Talazoparib Combination Trial (TalaCom)

Cancer type: Unspecified Solid Tumor

Variant class: DNA repair pathway

Other identifiers: 2020-0436, NCI-2020-06041, TalaCom

Population segments: Hormone refractory, Second line, Stage III, Stage IV

Phase: I

Therapies: talazoparib, palbociclib, axitinib, crizotinib

Location: United States

US State: TX

Contact: Timothy A. Yap [713-563-1784; tyap@mdanderson.org]

NCT03810105
A Phase II Study of Olaparib and Durvalumab in Men With Castration Sensitive Biochemically Recurrent Non-Metastatic Prostate Cancer Harboring Mutations in DNA Damage Repair

Cancer type: Prostate Cancer

Variant class: ATM deletion

Other identifiers: 18-480, NCI-2019-01627

Population segments: Adjuvant, Stage I, Stage II, Stage III

Phase: II

Therapies: durvalumab, olaparib

Location: United States

US States: CA, IL, MI, NJ, NY

Contact: Dr. Karen Autio [646-422-4632; autiok@mskcc.org]

ATM deletion (continued)

NCT03012321
BRCAAway: A Randomized Phase II Trial of Abiraterone, Olaparib, or Abiraterone + Olaparib in Patients With Metastatic Castration-Resistant Prostate Cancer With DNA Repair Defects

Cancer type: Castration-Resistant Prostate Cancer

Variant class: ATM deletion

Other identifiers: 19137, BRCAAway, NCI-2016-01834, NU 16U05, NU_16U05, PCCTC #: c16-168, PCCTC c16-168, STU00203960

Population segments: First line, Hormone refractory, Stage IV

Phase: II

Therapies: hormone therapy, olaparib, steroid

Location: United States

US States: CA, FL, IL, IN, MD, MI, MN, MO, NC, NY, UT, VA

Contact: Study Coordinator [312-695-1301; cancertrials@northwestern.edu]

NCT03840967
A Phase II Study Evaluating Safety and Efficacy of Niraparib in Patients With Previously Treated Homologous Recombination (HR) Defective or Loss of Heterozygosity (LOH) High Metastatic Esophageal/Gastroesophageal Junction/ Proximal Gastric Adenocarcinoma

Cancer type: Esophageal Cancer, Gastric Cancer, Gastroesophageal Junction Adenocarcinoma

Variant class: ATM deletion

Other identifiers: 20166, 71618, HCRN ESO17-325, NCI-2019-07395

Population segments: Second line, Stage III, Stage IV

Phase: II

Therapy: niraparib

Location: United States

US States: FL, IN, MI, NY

Contact: Dr. Shadia Jalal [317-274-3658; sjalal@iu.edu]

NCT04030559
Phase II Trial of PARP Inhibitor Niraparib for Men With High Risk Prostate Cancer and DNA Damage Response Defects

Cancer type: Prostate Cancer

Variant class: ATM deletion

Other identifiers: 1412676, NCI-2019-04365, UCDCC#279

Population segments: First line, Neoadjuvant, Stage I, Stage II, Stage IV

Phase: II

Therapy: niraparib

Location: United States

US State: CA

Contact: Marc Dall'Era [916-734-3771; mdallera@ucdavis.edu]

Disclaimer: The data presented here is from a curated knowledgebase of publicly available information, but may not be exhaustive. The data version is 2021.11(003).

ATM deletion (continued)

NCT03344965
A Phase II Study of Olaparib Monotherapy in Metastatic Breast Cancer Patients With Germline or Somatic Mutations in DNA Repair Genes (Olaparib Expanded)

Cancer type: Breast Cancer

Variant class: ATM deletion

Other identifiers: 17-428, 18-414, NCI-2018-00778, TBCRC 048, TBCRC048

Population segments: BRCA, Estrogen receptor positive, HER2 negative, HER2 positive, Progesterone receptor positive, Second line, Stage IV

Exclusion criteria variant classes: BRCA1 germline mutation (Breast Cancer), BRCA2 germline mutation (Breast Cancer)

Phase: II

Therapy: olaparib

Location: United States

US States: AL, CA, IL, MA, MD, NC, NJ, NY, PA, WA

Contact: Dr. Nadine Tung [617-667-1962; ntung@bidmc.harvard.edu]

NCT04633902
Phase II Study of Olaparib in Combination With Pembrolizumab in Patients With Advanced Melanoma With Homologous Recombination (HR) Pathway Gene Mutation

Cancer type: Melanoma

Variant class: ATM deletion

Other identifier: CPMC19-MEL01

Population segments: BRAF, Second line, Stage III, Stage IV

Phase: II

Therapies: olaparib, pembrolizumab

Location: United States

US State: CA

Contact: Dr. Kevin B. Kim [415-885-8600; ClinicalResearch@sutterhealth.org]

NCT03248570
Phase II Open Label Study of Pembrolizumab in Patients With Metastatic Castration Resistant Prostate Cancer (mCRPC) With or Without DNA Damage Repair Defects

Cancer type: Castration-Resistant Prostate Cancer

Variant class: ATM deletion

Other identifiers: 16557, 17-22165, CC 16557, NCI-2017-02408

Population segments: BRCA, Hormone refractory, Line of therapy N/A, Stage IV

Phase: II

Therapy: pembrolizumab

Location: United States

US State: CA

Contact: Patricia Li [415-476-5975; GUTrials@ucsf.edu]

Disclaimer: The data presented here is from a curated knowledgebase of publicly available information, but may not be exhaustive. The data version is 2021.11(003).

ATM deletion (continued)

NCT04740190
Combination Talazoparib - Carboplatin for Recurrent High-grade Glioma With DNA Damage Repair Deficiency (DDRd)

Cancer type: Anaplastic Astrocytoma, Anaplastic Oligoastrocytoma, Anaplastic Oligodendroglioma, Anaplastic Pleomorphic Xanthoastrocytoma, Diffuse Midline Glioma, Glioblastoma

Variant class: ATM deletion

Other identifiers: TAC-GReD, UW-20396

Population segments: (N/A), BRCA, Second line

Phase: II

Therapies: talazoparib, chemotherapy

Location: Hong Kong

No NCT ID - see other identifier(s)
An Open-label, Single-arm Phase Ia/Ib Clinical Study: Phase Ia Dose Exploration Study to Evaluate the SC0245 Tablets in Patients with Advanced Malignant Solid Tumor, and Phase Ib Clinical Study in Gastric Cancer Patients with ATM Deficiency and Esophageal Carcinoma Patients with p53 Mutation

Cancer type: Gastric Cancer

Variant class: ATM deletion

Other identifiers: ChiCTR2100045406, CTR20210769, SC0245-001

Population segments: Second line, Stage III, Stage IV

Phase: I

Therapy: SC0245

Location: China

NCT04666740
A Phase II Trial to Evaluate the Safety and Antitumor Activity of Pembrolizumab and OLApaRib (POLAR) Maintenance for Patients With Metastatic Pancreatic Ductal Adenocarcinoma and Homologous Recombination Deficiency and/or Exceptional Treatment Response to Platinum-Based Therapy

Cancer type: Pancreatic Cancer

Variant class: ATM aberration

Other identifier: 20-481

Population segments: BRCA, Maintenance/Consolidation, Stage IV

Phase: II

Therapies: pembrolizumab, olaparib

Location: United States

US States: NJ, NY

Contact: Dr. Wungki Park [646-888-4543; yaqubiea@mskcc.org]

NCT03506997
PERSEUS1: Phase II Trial of the Immune Checkpoint Inhibitor Pembrolizumab for Patients Suffering From Metastatic Prostate Cancer

Cancer type: Castration-Resistant Prostate Cancer

Variant class: DNA repair pathway

Other inclusion criteria: MMR deficient

Other identifiers: CCR4559, EudraCT Number: 2017-000931-15, ICR-CTSU/2016/10060, IRAS ID 208952, PERSEUS1

Population segments: Hormone refractory, Second line, Stage IV

Phase: II

Therapy: pembrolizumab

Location: United Kingdom

Disclaimer: The data presented here is from a curated knowledgebase of publicly available information, but may not be exhaustive. The data version is 2021.11(003).

ATM deletion (continued)

NCT04849364
A Phase II Circulating Tumor DNA Enriched, Genomically Directed Post-neoadjuvant Trial for Patients With Residual Triple Negative Breast Cancer (PERSEVERE)

Cancer type: Triple Negative Breast Cancer

Variant class: DNA repair pathway

Other identifiers: HCRN BRE18-334, PERSEVERE

Population segments: Adjuvant, HER2 negative, Stage I, Stage II, Stage III, Triple receptor negative

Phase: II

Therapies: talazoparib, chemotherapy

Location: United States

US State: IN

Contact: Dr. Bryan P. Schneider [317-948-3885; bpschnei@iu.edu]

NCT05002868
A Multi-center, Open-label, Phase I/Ib Study to Assess the Safety, Pharmacokinetics and Anti-tumor Activity of RP12146, a Poly (ADP-ribose) Polymerase (PARP) Inhibitor, in Patients With Locally Advanced or Metastatic Solid Tumors

Cancer type: Biliary Tract Carcinoma, Breast Cancer, Colorectal Cancer, Endometrial Carcinoma, Gastric Cancer, Ovarian Cancer, Pancreatic Cancer, Prostate Cancer, Small Cell Lung Cancer

Variant class: DNA repair pathway

Other identifier: RP12146-2101

Population segments: Extensive, Pulmonary, Second line, Stage III, Stage IV

Exclusion criteria variant class: ERBB2 positive (Breast Cancer)

Phase: I

Therapy: RP12146

Location: Poland

ATM fusion

NCT04497116
Phase I/IIa Study of the Safety, Pharmacokinetics, Pharmacodynamics and Preliminary Clinical Activity of RP-3500 Alone or in Combination With Talazoparib in Advanced Solid Tumors With ATR Inhibitor Sensitizing Mutations (TRESR Study)

Cancer type: Unspecified Solid Tumor

Variant class: ATM aberration

Other identifiers: 20-521, EudraCT Number: 2020-000301-87, IRAS ID: 286609, NCI-2020-06869, RP-3500-01, TRESR, TRESR Study

Population segments: Second line, Stage III, Stage IV

Phase: I/II

Therapies: RP-3500, talazoparib

Locations: Canada, Denmark, United Kingdom, United States

US States: MA, NC, NY, RI, TN, TX

Contact: Dr. Peter Manley [857-322-5553; pmanley@reparerx.com]

Disclaimer: The data presented here is from a curated knowledgebase of publicly available information, but may not be exhaustive. The data version is 2021.11(003).

ATM fusion (continued)

NCT04901702
A Randomized Phase I/II Study of Talazoparib or Temozolomide in Combination With Onivyde in Children With Recurrent Solid Malignancies and Ewing Sarcoma

Cancer type: Unspecified Solid Tumor

Variant class: HRR pathway

Other identifiers: EudraCT Number: 2021-003570-31, ONITT

Population segments: Second line, Stage IV

Exclusion criteria variant classes: HRR germline pathway (Central Nervous System Neoplasm, Ewing Sarcoma), HRR pathway (Central Nervous System Neoplasm, Ewing Sarcoma)

Phase: I/II

Therapies: talazoparib, chemotherapy

Locations: Canada, United States

US State: TN

Contact: Dr. Sara Federico [866-278-5833; referralinfo@stjude.org]

NCT03155620
NCI-COG Pediatric MATCH (Molecular Analysis for Therapy Choice) Screening Protocol

Cancer type: Unspecified Solid Tumor

Variant class: DNA repair pathway

Other identifiers: 17-729, 2017-0625, 20170841, APEC1621SC, COGAPEC1621, COGAPEC1621SC, N 55017 SC, NCI-2017-01251, NCI-COG Pediatric MATCH, Pediatric MATCH

Population segments: ALK, Aggressive, BRAF, FGFR, HRAS, Indolent, KRAS, NRAS, Pediatric or Adolescent, Second line, Stage III, Stage IV

Phase: II

Therapy: olaparib

Locations: Puerto Rico, United States

US States: AK, AL, AR, AZ, CA, CO, CT, DC, DE, FL, GA, HI, IA, ID, IL, IN, KY, LA, MA, MD, ME, MI, MN, MO, MS, NC, NE, NH, NJ, NV, NY, OH, OK, OR, PA, RI, SC, SD, TN, TX, UT, VA, VT, WA, WI

Contact: Multiple contacts: See www.clinicaltrials.gov for complete list of contacts.

NCT03233204
NCI-COG Pediatric MATCH (Molecular Analysis for Therapy Choice)- Phase 2 Subprotocol of Olaparib in Patients With Tumors Harboring Defects in DNA Damage Repair Genes

Cancer type: Unspecified Solid Tumor

Variant class: DNA repair pathway

Other identifiers: 20170841, APEC1621H, NCI-2017-00766

Population segments: Aggressive, Indolent, Pediatric or Adolescent, Second line, Stage III, Stage IV

Phase: II

Therapy: olaparib

Locations: Puerto Rico, United States

US States: AK, AL, AR, AZ, CA, CO, DC, DE, FL, GA, IA, ID, IL, IN, KY, LA, MD, ME, MI, MN, MO, MS, NC, ND, NE, NJ, NY, OH, OK, OR, PA, SC, SD, TN, TX, UT, VA, VT, WA, WI

Contact: Multiple contacts: See www.clinicaltrials.gov for complete list of contacts.

Disclaimer: The data presented here is from a curated knowledgebase of publicly available information, but may not be exhaustive. The data version is 2021.11(003).

ATM fusion (continued)

NCT04267939
An Open-label Phase Ib Study to Determine the Maximum Tolerated and/or Recommended Phase II Dose of the ATR Inhibitor BAY 1895344 in Combination With PARP Inhibitor Niraparib, in Patients With Recurrent Advanced Solid Tumors and Ovarian Cancer

Cancer type: Ovarian Cancer, Unspecified Solid Tumor

Variant class: DNA repair pathway

Other identifiers: 18595, 19-810, 2019-0471, 20-222, EudraCT Number: 2018-003930-34, NCI-2020-01405

Population segments: Maintenance/Consolidation, Second line, Stage III, Stage IV

Exclusion criteria variant class: DNA repair pathway (Prostate Cancer)

Phase: I

Therapies: BAY-1895344, niraparib

Location: United States

US States: MA, NY, TX

Contact: Bayer Clinical Trials Contact [888-842-2937; clinical-trials-contact@bayer.com]

NCT04693468
Modular Phase IB Hypothesis-Testing, Biomarker-Driven, Talazoparib Combination Trial (TalaCom)

Cancer type: Unspecified Solid Tumor

Variant class: DNA repair pathway

Other identifiers: 2020-0436, NCI-2020-06041, TalaCom

Population segments: Hormone refractory, Second line, Stage III, Stage IV

Phase: I

Therapies: talazoparib, palbociclib, axitinib, crizotinib

Location: United States

US State: TX

Contact: Timothy A. Yap [713-563-1784; tyap@mdanderson.org]

NCT03840967
A Phase II Study Evaluating Safety and Efficacy of Niraparib in Patients With Previously Treated Homologous Recombination (HR) Defective or Loss of Heterozygosity (LOH) High Metastatic Esophageal/Gastroesophageal Junction/Proximal Gastric Adenocarcinoma

Cancer type: Esophageal Cancer, Gastric Cancer, Gastroesophageal Junction Adenocarcinoma

Variant class: ATM fusion

Other identifiers: 20166, 71618, HCRN ESO17-325, NCI-2019-07395

Population segments: Second line, Stage III, Stage IV

Phase: II

Therapy: niraparib

Location: United States

US States: FL, IN, MI, NY

Contact: Dr. Shadia Jalal [317-274-3658; sjalal@iu.edu]

Disclaimer: The data presented here is from a curated knowledgebase of publicly available information, but may not be exhaustive. The data version is 2021.11(003).

ATM fusion (continued)

NCT04666740

A Phase II Trial to Evaluate the Safety and Antitumor Activity of Pembrolizumab and OLApaRib (POLAR) Maintenance for Patients With Metastatic Pancreatic Ductal Adenocarcinoma and Homologous Recombination Deficiency and/or Exceptional Treatment Response to Platinum-Based Therapy

Cancer type: Pancreatic Cancer

Variant class: ATM aberration

Other identifier: 20-481

Population segments: BRCA, Maintenance/Consolidation, Stage IV

Phase: II

Therapies: pembrolizumab, olaparib

Location: United States

US States: NJ, NY

Contact: Dr. Wungki Park [646-888-4543; yaqubiea@mskcc.org]

NCT04633902

Phase II Study of Olaparib in Combination With Pembrolizumab in Patients With Advanced Melanoma With Homologous Recombination (HR) Pathway Gene Mutation

Cancer type: Melanoma

Variant class: HRR pathway

Other identifier: CPMC19-MEL01

Population segments: BRAF, Second line, Stage III, Stage IV

Phase: II

Therapies: olaparib, pembrolizumab

Location: United States

US State: CA

Contact: Dr. Kevin B. Kim [415-885-8600; ClinicalResearch@sutterhealth.org]

NCT03506997

PERSEUS1: Phase II Trial of the Immune Checkpoint Inhibitor Pembrolizumab for Patients Suffering From Metastatic Prostate Cancer

Cancer type: Castration-Resistant Prostate Cancer

Variant class: DNA repair pathway

Other inclusion criteria: MMR deficient

Other identifiers: CCR4559, EudraCT Number: 2017-000931-15, ICR-CTSU/2016/10060, IRAS ID 208952, PERSEUS1

Population segments: Hormone refractory, Second line, Stage IV

Phase: II

Therapy: pembrolizumab

Location: United Kingdom

NCT04849364

A Phase II Circulating Tumor DNA Enriched, Genomically Directed Post-neoadjuvant Trial for Patients With Residual Triple Negative Breast Cancer (PERSEVERE)

Cancer type: Triple Negative Breast Cancer

Variant class: DNA repair pathway

Other identifiers: HCRN BRE18-334, PERSEVERE

Population segments: Adjuvant, HER2 negative, Stage I, Stage II, Stage III, Triple receptor negative

Phase: II

Therapies: talazoparib, chemotherapy

Location: United States

US State: IN

Contact: Dr. Bryan P. Schneider [317-948-3885; bpschnei@iu.edu]

Disclaimer: The data presented here is from a curated knowledgebase of publicly available information, but may not be exhaustive. The data version is 2021.11(003).

ATM fusion (continued)

NCT05002868

A Multi-center, Open-label, Phase I/Ib Study to Assess the Safety, Pharmacokinetics and Anti-tumor Activity of RP12146, a Poly (ADP-ribose) Polymerase (PARP) Inhibitor, in Patients With Locally Advanced or Metastatic Solid Tumors

Cancer type: Biliary Tract Carcinoma, Breast Cancer, Colorectal Cancer, Endometrial Carcinoma, Gastric Cancer, Ovarian Cancer, Pancreatic Cancer, Prostate Cancer, Small Cell Lung Cancer

Variant class: DNA repair pathway

Other identifier: RP12146-2101

Population segments: Extensive, Pulmonary, Second line, Stage III, Stage IV

Exclusion criteria variant class: ERBB2 positive (Breast Cancer)

Phase: I

Therapy: RP12146

Location: Poland

ATM aberration

NCT04497116

Phase I/IIa Study of the Safety, Pharmacokinetics, Pharmacodynamics and Preliminary Clinical Activity of RP-3500 Alone or in Combination With Talazoparib in Advanced Solid Tumors With ATR Inhibitor Sensitizing Mutations (TRESR Study)

Cancer type: Unspecified Solid Tumor

Variant class: ATM aberration

Other identifiers: 20-521, EudraCT Number: 2020-000301-87, IRAS ID: 286609, NCI-2020-06869, RP-3500-01, TRESR, TRESR Study

Population segments: Second line, Stage III, Stage IV

Phase: I/II

Therapies: RP-3500, talazoparib

Locations: Canada, Denmark, United Kingdom, United States

US States: MA, NC, NY, RI, TN, TX

Contact: Dr. Peter Manley [857-322-5553; pmanley@reparerx.com]

NCT04901702

A Randomized Phase I/II Study of Talazoparib or Temozolomide in Combination With Onivyde in Children With Recurrent Solid Malignancies and Ewing Sarcoma

Cancer type: Unspecified Solid Tumor

Variant class: HRR pathway

Other identifiers: EudraCT Number: 2021-003570-31, ONITT

Population segments: Second line, Stage IV

Exclusion criteria variant classes: HRR germline pathway (Central Nervous System Neoplasm, Ewing Sarcoma), HRR pathway (Central Nervous System Neoplasm, Ewing Sarcoma)

Phase: I/II

Therapies: talazoparib, chemotherapy

Locations: Canada, United States

US State: TN

Contact: Dr. Sara Federico [866-278-5833; referralinfo@stjude.org]

ATM aberration (continued)

NCT03155620

NCI-COG Pediatric MATCH (Molecular Analysis for Therapy Choice) Screening Protocol

Cancer type: Unspecified Solid Tumor

Variant class: DNA repair pathway

Other identifiers: 17-729, 2017-0625, 20170841, APEC1621SC, COGAPEC1621, COGAPEC1621SC, N 55017 SC, NCI-2017-01251, NCI-COG Pediatric MATCH, Pediatric MATCH

Population segments: ALK, Aggressive, BRAF, FGFR, HRAS, Indolent, KRAS, NRAS, Pediatric or Adolescent, Second line, Stage III, Stage IV

Phase: II

Therapy: olaparib

Locations: Puerto Rico, United States

US States: AK, AL, AR, AZ, CA, CO, CT, DC, DE, FL, GA, HI, IA, ID, IL, IN, KY, LA, MA, MD, ME, MI, MN, MO, MS, NC, NE, NH, NJ, NV, NY, OH, OK, OR, PA, RI, SC, SD, TN, TX, UT, VA, VT, WA, WI

Contact: Multiple contacts: See www.clinicaltrials.gov for complete list of contacts.

NCT03233204

NCI-COG Pediatric MATCH (Molecular Analysis for Therapy Choice)- Phase 2 Subprotocol of Olaparib in Patients With Tumors Harboring Defects in DNA Damage Repair Genes

Cancer type: Unspecified Solid Tumor

Variant class: DNA repair pathway

Other identifiers: 20170841, APEC1621H, NCI-2017-00766

Population segments: Aggressive, Indolent, Pediatric or Adolescent, Second line, Stage III, Stage IV

Phase: II

Therapy: olaparib

Locations: Puerto Rico, United States

US States: AK, AL, AR, AZ, CA, CO, DC, DE, FL, GA, IA, ID, IL, IN, KY, LA, MD, ME, MI, MN, MO, MS, NC, ND, NE, NJ, NY, OH, OK, OR, PA, SC, SD, TN, TX, UT, VA, VT, WA, WI

Contact: Multiple contacts: See www.clinicaltrials.gov for complete list of contacts.

NCT04267939

An Open-label Phase Ib Study to Determine the Maximum Tolerated and/or Recommended Phase II Dose of the ATR Inhibitor BAY 1895344 in Combination With PARP Inhibitor Niraparib, in Patients With Recurrent Advanced Solid Tumors and Ovarian Cancer

Cancer type: Ovarian Cancer, Unspecified Solid Tumor

Variant class: DNA repair pathway

Other identifiers: 18595, 19-810, 2019-0471, 20-222, EudraCT Number: 2018-003930-34, NCI-2020-01405

Population segments: Maintenance/Consolidation, Second line, Stage III, Stage IV

Exclusion criteria variant class: DNA repair pathway (Prostate Cancer)

Phase: I

Therapies: BAY-1895344, niraparib

Location: United States

US States: MA, NY, TX

Contact: Bayer Clinical Trials Contact [888-842-2937; clinical-trials-contact@bayer.com]

Disclaimer: The data presented here is from a curated knowledgebase of publicly available information, but may not be exhaustive. The data version is 2021.11(003).

ATM aberration (continued)

NCT04693468
Modular Phase IB Hypothesis-Testing, Biomarker-Driven, Talazoparib Combination Trial (TalaCom)

Cancer type: Unspecified Solid Tumor

Variant class: DNA repair pathway

Other identifiers: 2020-0436, NCI-2020-06041, TalaCom

Population segments: Hormone refractory, Second line, Stage III, Stage IV

Phase: I

Therapies: talazoparib, palbociclib, axitinib, crizotinib

Location: United States

US State: TX

Contact: Timothy A. Yap [713-563-1784; tyap@mdanderson.org]

NCT04666740
A Phase II Trial to Evaluate the Safety and Antitumor Activity of Pembrolizumab and OLApaRib (POLAR) Maintenance for Patients With Metastatic Pancreatic Ductal Adenocarcinoma and Homologous Recombination Deficiency and/or Exceptional Treatment Response to Platinum-Based Therapy

Cancer type: Pancreatic Cancer

Variant class: ATM aberration

Other identifier: 20-481

Population segments: BRCA, Maintenance/Consolidation, Stage IV

Phase: II

Therapies: pembrolizumab, olaparib

Location: United States

US States: NJ, NY

Contact: Dr. Wungki Park [646-888-4543; yaqubiea@mskcc.org]

NCT04633902
Phase II Study of Olaparib in Combination With Pembrolizumab in Patients With Advanced Melanoma With Homologous Recombination (HR) Pathway Gene Mutation

Cancer type: Melanoma

Variant class: HRR pathway

Other identifier: CPMC19-MEL01

Population segments: BRAF, Second line, Stage III, Stage IV

Phase: II

Therapies: olaparib, pembrolizumab

Location: United States

US State: CA

Contact: Dr. Kevin B. Kim [415-885-8600; ClinicalResearch@sutterhealth.org]

NCT03506997
PERSEUS1: Phase II Trial of the Immune Checkpoint Inhibitor Pembrolizumab for Patients Suffering From Metastatic Prostate Cancer

Cancer type: Castration-Resistant Prostate Cancer

Variant class: DNA repair pathway

Other inclusion criteria: MMR deficient

Other identifiers: CCR4559, EudraCT Number: 2017-000931-15, ICR-CTSU/2016/10060, IRAS ID 208952, PERSEUS1

Population segments: Hormone refractory, Second line, Stage IV

Phase: II

Therapy: pembrolizumab

Location: United Kingdom

ATM aberration (continued)

NCT04849364
A Phase II Circulating Tumor DNA Enriched, Genomically Directed Post-neoadjuvant Trial for Patients With Residual Triple Negative Breast Cancer (PERSEVERE)

Cancer type: Triple Negative Breast Cancer

Variant class: DNA repair pathway

Other identifiers: HCRN BRE18-334, PERSEVERE

Population segments: Adjuvant, HER2 negative, Stage I, Stage II, Stage III, Triple receptor negative

Phase: II

Therapies: talazoparib, chemotherapy

Location: United States

US State: IN

Contact: Dr. Bryan P. Schneider [317-948-3885; bpschnei@iu.edu]

NCT05002868
A Multi-center, Open-label, Phase I/Ib Study to Assess the Safety, Pharmacokinetics and Anti-tumor Activity of RP12146, a Poly (ADP-ribose) Polymerase (PARP) Inhibitor, in Patients With Locally Advanced or Metastatic Solid Tumors

Cancer type: Biliary Tract Carcinoma, Breast Cancer, Colorectal Cancer, Endometrial Carcinoma, Gastric Cancer, Ovarian Cancer, Pancreatic Cancer, Prostate Cancer, Small Cell Lung Cancer

Variant class: DNA repair pathway

Other identifier: RP12146-2101

Population segments: Extensive, Pulmonary, Second line, Stage III, Stage IV

Exclusion criteria variant class: ERBB2 positive (Breast Cancer)

Phase: I

Therapy: RP12146

Location: Poland

Relevant Biomarkers

Genomic Alteration	Relevant Therapies (In this cancer type)	Relevant Therapies (In other cancer type)	Clinical Trials
ATM mutation<br>**Prognostic significance:** None<br>**Diagnostic significance:** None	None	**olaparib** [1]	61
ATM splice site mutation<br>**Prognostic significance:** None<br>**Diagnostic significance:** None	None	**olaparib** [1]	61
ATM truncating mutation<br>**Prognostic significance:** None<br>**Diagnostic significance:** None	None	**olaparib** [1]	61
ATM deletion<br>**Prognostic significance:** None<br>**Diagnostic significance:** None	None	None	24

Public data sources included in relevant therapies: FDA1, NCCN, **EMA**2, ESMO
Public data sources included in prognostic and diagnostic significance: NCCN, ESMO

Disclaimer: The data presented here is from a curated knowledgebase of publicly available information, but may not be exhaustive. The data version is 2021.11(003).

Relevant Biomarkers (continued)

Genomic Alteration	Relevant Therapies (In this cancer type)	Relevant Therapies (In other cancer type)	Clinical Trials
ATM fusion<br>**Prognostic significance:** None<br>**Diagnostic significance:** None	None	None	12
ATM aberration<br>**Prognostic significance:** None<br>**Diagnostic significance:** None	None	None	11

Public data sources included in relevant therapies: FDA1, NCCN, **EMA**2, ESMO
Public data sources included in prognostic and diagnostic significance: NCCN, ESMO

Relevant Therapy Summary

● In this cancer type ○ In other cancer type ◐ In this cancer type and other cancer types ✖ No evidence

ATM mutation

Relevant Therapy	FDA	NCCN	EMA	ESMO	Clinical Trials*
olaparib	○	○	✖	✖	◐ (II)
olaparib, pembrolizumab	✖	✖	✖	✖	◐ (II)
talazoparib	✖	✖	✖	✖	◐ (II)
talazoparib, chemotherapy	✖	✖	✖	✖	◐ (II)
atezolizumab	✖	✖	✖	✖	● (II)
ceralasertib	✖	✖	✖	✖	● (II)
olaparib, ipilimumab + nivolumab, atezolizumab + talazoparib	✖	✖	✖	✖	● (II)
rucaparib, atezolizumab	✖	✖	✖	✖	● (II)
alrizomadlin, pembrolizumab	✖	✖	✖	✖	● (I/II)
ATRN-119	✖	✖	✖	✖	● (I/II)
RP-3500, talazoparib	✖	✖	✖	✖	● (I/II)
BAY-1895344, niraparib	✖	✖	✖	✖	● (I)
copanlisib, olaparib, durvalumab	✖	✖	✖	✖	● (I)
HH-CYH33, olaparib	✖	✖	✖	✖	● (I)
HWH-340	✖	✖	✖	✖	● (I)
talazoparib, palbociclib, axitinib, crizotinib	✖	✖	✖	✖	● (I)
rucaparib, hormone therapy	✖	✖	✖	✖	○ (III)
talazoparib, hormone therapy	✖	✖	✖	✖	○ (III)
durvalumab, olaparib	✖	✖	✖	✖	○ (II)

* Most advanced phase (IV, III, II/III, II, I/II, I) is shown and multiple clinical trials may be available.

Disclaimer: The data presented here is from a curated knowledgebase of publicly available information, but may not be exhaustive. The data version is 2021.11(003).

Relevant Therapy Summary (continued)

● In this cancer type ○ In other cancer type ◑ In this cancer type and other cancer types ✖ No evidence

ATM mutation (continued)

Relevant Therapy	FDA	NCCN	EMA	ESMO	Clinical Trials*
durvalumab, olaparib, hormone therapy	✖	✖	✖	✖	○ (II)
durvalumab, tremelimumab, olaparib	✖	✖	✖	✖	○ (II)
hormone therapy	✖	✖	✖	✖	○ (II)
hormone therapy, olaparib, steroid	✖	✖	✖	✖	○ (II)
niraparib	✖	✖	✖	✖	○ (II)
niraparib, dostarlimab	✖	✖	✖	✖	○ (II)
nivolumab, chemotherapy, hormone therapy	✖	✖	✖	✖	○ (II)
nivolumab, talazoparib	✖	✖	✖	✖	○ (II)
olaparib, bevacizumab	✖	✖	✖	✖	○ (II)
olaparib, chemotherapy	✖	✖	✖	✖	○ (II)
pembrolizumab	✖	✖	✖	✖	○ (II)
pembrolizumab, olaparib	✖	✖	✖	✖	○ (II)
rucaparib	✖	✖	✖	✖	○ (II)
copanlisib, rucaparib	✖	✖	✖	✖	○ (I/II)
sintilimab	✖	✖	✖	✖	○ (I/II)
BAY-1895344, pembrolizumab	✖	✖	✖	✖	○ (I)
ceralasertib, olaparib	✖	✖	✖	✖	○ (I)
pidnarulex	✖	✖	✖	✖	○ (I)
RP12146	✖	✖	✖	✖	○ (I)

ATM splice site mutation

Relevant Therapy	FDA	NCCN	EMA	ESMO	Clinical Trials*
olaparib	○	○	✖	✖	◑ (II)
olaparib, pembrolizumab	✖	✖	✖	✖	◑ (II)
talazoparib	✖	✖	✖	✖	◑ (II)
talazoparib, chemotherapy	✖	✖	✖	✖	◑ (II)
atezolizumab	✖	✖	✖	✖	● (II)
ceralasertib	✖	✖	✖	✖	● (II)

* Most advanced phase (IV, III, II/III, II, I/II, I) is shown and multiple clinical trials may be available.

Disclaimer: The data presented here is from a curated knowledgebase of publicly available information, but may not be exhaustive. The data version is 2021.11(003).

Relevant Therapy Summary (continued)

● In this cancer type ○ In other cancer type ◐ In this cancer type and other cancer types ✖ No evidence

ATM splice site mutation (continued)

Relevant Therapy	FDA	NCCN	EMA	ESMO	Clinical Trials*
olaparib, ipilimumab + nivolumab, atezolizumab + talazoparib	✖	✖	✖	✖	● (II)
rucaparib, atezolizumab	✖	✖	✖	✖	● (II)
alrizomadlin, pembrolizumab	✖	✖	✖	✖	● (I/II)
ATRN-119	✖	✖	✖	✖	● (I/II)
RP-3500, talazoparib	✖	✖	✖	✖	● (I/II)
BAY-1895344, niraparib	✖	✖	✖	✖	● (I)
copanlisib, olaparib, durvalumab	✖	✖	✖	✖	● (I)
HH-CYH33, olaparib	✖	✖	✖	✖	● (I)
HWH-340	✖	✖	✖	✖	● (I)
talazoparib, palbociclib, axitinib, crizotinib	✖	✖	✖	✖	● (I)
rucaparib, hormone therapy	✖	✖	✖	✖	○ (III)
talazoparib, hormone therapy	✖	✖	✖	✖	○ (III)
durvalumab, olaparib	✖	✖	✖	✖	○ (II)
durvalumab, olaparib, hormone therapy	✖	✖	✖	✖	○ (II)
durvalumab, tremelimumab, olaparib	✖	✖	✖	✖	○ (II)
hormone therapy	✖	✖	✖	✖	○ (II)
hormone therapy, olaparib, steroid	✖	✖	✖	✖	○ (II)
niraparib	✖	✖	✖	✖	○ (II)
niraparib, dostarlimab	✖	✖	✖	✖	○ (II)
nivolumab, chemotherapy, hormone therapy	✖	✖	✖	✖	○ (II)
nivolumab, talazoparib	✖	✖	✖	✖	○ (II)
olaparib, bevacizumab	✖	✖	✖	✖	○ (II)
olaparib, chemotherapy	✖	✖	✖	✖	○ (II)
pembrolizumab	✖	✖	✖	✖	○ (II)
pembrolizumab, olaparib	✖	✖	✖	✖	○ (II)
rucaparib	✖	✖	✖	✖	○ (II)
copanlisib, rucaparib	✖	✖	✖	✖	○ (I/II)
sintilimab	✖	✖	✖	✖	○ (I/II)

* Most advanced phase (IV, III, II/III, II, I/II, I) is shown and multiple clinical trials may be available.

Disclaimer: The data presented here is from a curated knowledgebase of publicly available information, but may not be exhaustive. The data version is 2021.11(003).

Relevant Therapy Summary (continued)

● In this cancer type ○ In other cancer type ◐ In this cancer type and other cancer types ✖ No evidence

ATM splice site mutation (continued)

Relevant Therapy	FDA	NCCN	EMA	ESMO	Clinical Trials*
BAY-1895344, pembrolizumab	✖	✖	✖	✖	○ (I)
ceralasertib, olaparib	✖	✖	✖	✖	○ (I)
pidnarulex	✖	✖	✖	✖	○ (I)
RP12146	✖	✖	✖	✖	○ (I)

ATM truncating mutation

Relevant Therapy	FDA	NCCN	EMA	ESMO	Clinical Trials*
olaparib	○	○	✖	✖	◐ (II)
olaparib, pembrolizumab	✖	✖	✖	✖	◐ (II)
talazoparib	✖	✖	✖	✖	◐ (II)
talazoparib, chemotherapy	✖	✖	✖	✖	◐ (II)
atezolizumab	✖	✖	✖	✖	● (II)
ceralasertib	✖	✖	✖	✖	● (II)
olaparib, ipilimumab + nivolumab, atezolizumab + talazoparib	✖	✖	✖	✖	● (II)
rucaparib, atezolizumab	✖	✖	✖	✖	● (II)
alrizomadlin, pembrolizumab	✖	✖	✖	✖	● (I/II)
ATRN-119	✖	✖	✖	✖	● (I/II)
RP-3500, talazoparib	✖	✖	✖	✖	● (I/II)
BAY-1895344, niraparib	✖	✖	✖	✖	● (I)
copanlisib, olaparib, durvalumab	✖	✖	✖	✖	● (I)
HH-CYH33, olaparib	✖	✖	✖	✖	● (I)
HWH-340	✖	✖	✖	✖	● (I)
talazoparib, palbociclib, axitinib, crizotinib	✖	✖	✖	✖	● (I)
rucaparib, hormone therapy	✖	✖	✖	✖	○ (III)
talazoparib, hormone therapy	✖	✖	✖	✖	○ (III)
durvalumab, olaparib	✖	✖	✖	✖	○ (II)
durvalumab, olaparib, hormone therapy	✖	✖	✖	✖	○ (II)
durvalumab, tremelimumab, olaparib	✖	✖	✖	✖	○ (II)

* Most advanced phase (IV, III, II/III, II, I/II, I) is shown and multiple clinical trials may be available.

Disclaimer: The data presented here is from a curated knowledgebase of publicly available information, but may not be exhaustive. The data version is 2021.11(003).

Relevant Therapy Summary (continued)

● In this cancer type ○ In other cancer type ◐ In this cancer type and other cancer types ✖ No evidence

ATM truncating mutation (continued)

Relevant Therapy	FDA	NCCN	EMA	ESMO	Clinical Trials*
hormone therapy	✖	✖	✖	✖	○ (II)
hormone therapy, olaparib, steroid	✖	✖	✖	✖	○ (II)
niraparib	✖	✖	✖	✖	○ (II)
niraparib, dostarlimab	✖	✖	✖	✖	○ (II)
nivolumab, chemotherapy, hormone therapy	✖	✖	✖	✖	○ (II)
nivolumab, talazoparib	✖	✖	✖	✖	○ (II)
olaparib, bevacizumab	✖	✖	✖	✖	○ (II)
olaparib, chemotherapy	✖	✖	✖	✖	○ (II)
pembrolizumab	✖	✖	✖	✖	○ (II)
pembrolizumab, olaparib	✖	✖	✖	✖	○ (II)
rucaparib	✖	✖	✖	✖	○ (II)
copanlisib, rucaparib	✖	✖	✖	✖	○ (I/II)
sintilimab	✖	✖	✖	✖	○ (I/II)
BAY-1895344, pembrolizumab	✖	✖	✖	✖	○ (I)
ceralasertib, olaparib	✖	✖	✖	✖	○ (I)
pidnarulex	✖	✖	✖	✖	○ (I)
RP12146	✖	✖	✖	✖	○ (I)

ATM deletion

Relevant Therapy	FDA	NCCN	EMA	ESMO	Clinical Trials*
olaparib	✖	✖	✖	✖	◐ (II)
talazoparib, chemotherapy	✖	✖	✖	✖	◐ (II)
rucaparib, atezolizumab	✖	✖	✖	✖	● (II)
talazoparib	✖	✖	✖	✖	● (II)
RP-3500, talazoparib	✖	✖	✖	✖	● (I/II)
BAY-1895344, niraparib	✖	✖	✖	✖	● (I)
talazoparib, palbociclib, axitinib, crizotinib	✖	✖	✖	✖	● (I)
durvalumab, olaparib	✖	✖	✖	✖	○ (II)
hormone therapy, olaparib, steroid	✖	✖	✖	✖	○ (II)

* Most advanced phase (IV, III, II/III, II, I/II, I) is shown and multiple clinical trials may be available.

Disclaimer: The data presented here is from a curated knowledgebase of publicly available information, but may not be exhaustive. The data version is 2021.11(003).

Relevant Therapy Summary (continued)

● In this cancer type ○ In other cancer type ◐ In this cancer type and other cancer types ✖ No evidence

ATM deletion (continued)

Relevant Therapy	FDA	NCCN	EMA	ESMO	Clinical Trials*
niraparib	✖	✖	✖	✖	○ (II)
nivolumab, chemotherapy, hormone therapy	✖	✖	✖	✖	○ (II)
olaparib, pembrolizumab	✖	✖	✖	✖	○ (II)
pembrolizumab	✖	✖	✖	✖	○ (II)
pembrolizumab, olaparib	✖	✖	✖	✖	○ (II)
BAY-1895344, pembrolizumab	✖	✖	✖	✖	○ (I)
RP12146	✖	✖	✖	✖	○ (I)
SC0245	✖	✖	✖	✖	○ (I)

ATM fusion

Relevant Therapy	FDA	NCCN	EMA	ESMO	Clinical Trials*
talazoparib, chemotherapy	✖	✖	✖	✖	◐ (II)
olaparib	✖	✖	✖	✖	● (II)
RP-3500, talazoparib	✖	✖	✖	✖	● (I/II)
BAY-1895344, niraparib	✖	✖	✖	✖	● (I)
talazoparib, palbociclib, axitinib, crizotinib	✖	✖	✖	✖	● (I)
niraparib	✖	✖	✖	✖	○ (II)
olaparib, pembrolizumab	✖	✖	✖	✖	○ (II)
pembrolizumab	✖	✖	✖	✖	○ (II)
pembrolizumab, olaparib	✖	✖	✖	✖	○ (II)
RP12146	✖	✖	✖	✖	○ (I)

ATM aberration

Relevant Therapy	FDA	NCCN	EMA	ESMO	Clinical Trials*
talazoparib, chemotherapy	✖	✖	✖	✖	◐ (II)
olaparib	✖	✖	✖	✖	● (II)
RP-3500, talazoparib	✖	✖	✖	✖	● (I/II)
BAY-1895344, niraparib	✖	✖	✖	✖	● (I)
talazoparib, palbociclib, axitinib, crizotinib	✖	✖	✖	✖	● (I)

* Most advanced phase (IV, III, II/III, II, I/II, I) is shown and multiple clinical trials may be available.

Disclaimer: The data presented here is from a curated knowledgebase of publicly available information, but may not be exhaustive. The data version is 2021.11(003).

Relevant Therapy Summary (continued)

● In this cancer type ○ In other cancer type ◐ In this cancer type and other cancer types ✖ No evidence

ATM aberration (continued)

Relevant Therapy	FDA	NCCN	EMA	ESMO	Clinical Trials*
olaparib, pembrolizumab	✖	✖	✖	✖	○ (II)
pembrolizumab	✖	✖	✖	✖	○ (II)
pembrolizumab, olaparib	✖	✖	✖	✖	○ (II)
RP12146	✖	✖	✖	✖	○ (I)

* Most advanced phase (IV, III, II/III, II, I/II, I) is shown and multiple clinical trials may be available.

Relevant Biomarkers

Genomic Alteration	Relevant Therapies (In this cancer type)	Relevant Therapies (In other cancer type)	Clinical Trials
ATM mutation<br>**Prognostic significance:** None<br>**Diagnostic significance:** None	None	**olaparib** [1]	61
ATM splice site mutation<br>**Prognostic significance:** None<br>**Diagnostic significance:** None	None	**olaparib** [1]	61
ATM truncating mutation<br>**Prognostic significance:** None<br>**Diagnostic significance:** None	None	**olaparib** [1]	61
ATM deletion<br>**Prognostic significance:** None<br>**Diagnostic significance:** None	None	None	24
ATM fusion<br>**Prognostic significance:** None<br>**Diagnostic significance:** None	None	None	12
ATM aberration<br>**Prognostic significance:** None<br>**Diagnostic significance:** None	None	None	11

Public data sources included in relevant therapies: FDA1, NCCN, **EMA**2, ESMO
Public data sources included in prognostic and diagnostic significance: NCCN, ESMO

Clinical Trials Summary

ATM mutation + ATM splice site mutation + ATM truncating mutation + ATM deletion

NCT ID	Title	Phase
NCT04276376	A Multicenter, Open Label, Phase II Basket Trial Exploring the Efficacy And Safety of The Combination of Rucaparib (PARP Inhibitor) And Atezolizumab (Anti-PD-L1 Antibody) In Patients With DNA Repair-Deficient or Platinum-Sensitive Solid Tumors	II

Disclaimer: The data presented here is from a curated knowledgebase of publicly available information, but may not be exhaustive. The data version is 2021.11(003).

Clinical Trials Summary (continued)

ATM mutation + ATM splice site mutation + ATM truncating mutation + ATM deletion (continued)

NCT ID	Title	Phase
NCT04095273	A Multicenter, Non-randomized, Open-label Phase Ib Study to Determine the Maximum Tolerated and Recommended Phase II Dose of the ATR Inhibitor BAY1895344 in Combination With Pembrolizumab and to Characterize Its Safety, Tolerability, Pharmacokinetics and Preliminary Anti-tumor Activity in Patients With Advanced Solid Tumors	I
NCT04126070	A Phase II Multicohort Study of Nivolumab in Combination With Docetaxel and Androgen Deprivation Therapy in Metastatic Hormone Sensitive Prostate Cancer Patients With DNA Damage Repair Defects or Inflamed Tumors	II

ATM mutation

NCT ID	Title	Phase
NCT03682289	Phase II Trial of AZD6738 Alone and in Combination With Olaparib in Patients With Selected Solid Tumor Malignancies	II
NCT04564027	A Modular Phase IIa Multicentre Open-Label Study to Investigate DNA-damage Response Agents (or Combinations) in Patients With Advanced Cancer Whose Tumours Contain Molecular Alterations (PLANETTE)	II
NCT02029001	A Two-period, Multicenter, Randomized, Open-label, Phase II Study Evaluating the Clinical Benefit of a Maintenance Treatment Targeting Tumor Molecular Alterations in Patients with Progressive Locally-advanced or Metastatic Solid Tumors.	II
NCT03742895	A Phase II Study of Olaparib Monotherapy in Participants With Previously Treated, Homologous Recombination Repair Mutation (HRRm) or Homologous Recombination Deficiency (HRD) Positive Advanced Cancer	II
NCT03967938	Efficacy of Olaparib in Advanced Cancers Occurring in Patients With Germline Mutations or Somatic Tumor Mutations in Homologous Recombination Genes	II
NCT02693535	Targeted Agent and Profiling Utilization Registry (TAPUR) Study.	II
NCT02286687	Phase II Study of the PARP Inhibitor Talazoparib in Advanced Cancer Patients With Somatic Alterations in BRCA1/2, Mutations/Deletions in Other BRCA Pathway Genes and Germline Mutation in BRCA1/2 (Not Breast or Ovarian Cancer)	II
NCT02401347	A Phase II Clinical Trial of the PARP Inhibitor Talazoparib in BRCA1 and BRCA2 Wild Type Patients With Advanced Triple Negative Breast Cancer and Homologous Recombination Deficiency or Advanced HER2 Negative Breast Cancer or Other Solid Tumors With a Mutation in Homologous Recombination Pathway Genes Talazoparib Beyond BRCA (TBB) Trial	II
NCT04550494	A Pharmacodynamics-Driven Trial of Talazoparib, an Oral PARP Inhibitor, in Patients With Advanced Solid Tumors and Aberrations in Genes Involved in DNA Damage Response	II
NCT03611868	A Phase Ib/II Study of APG-115 in Combination With Pembrolizumab in Patients With Unresectable or Metastatic Melanomas or Advanced Solid Tumors	I/II
NCT03842228	A Phase Ib Biomarker-Driven Combination Trial of Copanlisib, Olaparib, and Durvalumab (MEDI4736) in Patients With Advanced Solid Tumors	I
NCT04586335	Open Label, Phase Ib Study to Evaluate the Safety, Tolerability, Pharmacokinetics and Clinical Activity of CYH33, an Oral a-specific PI3K Inhibitor in Combination With Olaparib, an Oral PARP Inhibitor in Patients With Advanced Solid Tumors.	I
NCT04123366	A Phase II Study of Olaparib in Combination With Pembrolizumab in Participants With Previously Treated, Homologous Recombination Repair Mutation (HRRm) and/or Homologous Recombination Deficiency (HRD)-Positive Advanced Cancer	II

Disclaimer: The data presented here is from a curated knowledgebase of publicly available information, but may not be exhaustive. The data version is 2021.11(003).

Clinical Trials Summary (continued)

ATM mutation (continued)

NCT ID	Title	Phase
NCT04497116	Phase I/IIa Study of the Safety, Pharmacokinetics, Pharmacodynamics and Preliminary Clinical Activity of RP-3500 Alone or in Combination With Talazoparib in Advanced Solid Tumors With ATR Inhibitor Sensitizing Mutations (TRESR Study)	I/II
NCT03415659	A Phase I, Open-label, Single-center, Single/Multiple-dose, Dose-escalation/Dose-expansion Clinical Study on Tolerance and Pharmacokinetics of HWH340 Tablet in Patients With Advanced Solid Tumors	I
NCT03767075	Basket of Baskets: A Modular, Open-label, Phase II, Multicentre Study To Evaluate Targeted Agents in Molecularly Selected Populations With Advanced Solid Tumours	II
NCT04905914	A Phase I/IIa, Open-Label, Safety, Pharmacokinetic, And Preliminary Efficacy Study Of Oral ATRN-119 In Patients With Advanced Solid Tumors	I/II
NCT04901702	A Randomized Phase I/II Study of Talazoparib or Temozolomide in Combination With Onivyde in Children With Recurrent Solid Malignancies and Ewing Sarcoma	I/II
NCT03155620	NCI-COG Pediatric MATCH (Molecular Analysis for Therapy Choice) Screening Protocol	II
NCT03233204	NCI-COG Pediatric MATCH (Molecular Analysis for Therapy Choice)- Phase 2 Subprotocol of Olaparib in Patients With Tumors Harboring Defects in DNA Damage Repair Genes	II
NCT04267939	An Open-label Phase Ib Study to Determine the Maximum Tolerated and/or Recommended Phase II Dose of the ATR Inhibitor BAY 1895344 in Combination With PARP Inhibitor Niraparib, in Patients With Recurrent Advanced Solid Tumors and Ovarian Cancer	I
NCT04693468	Modular Phase IB Hypothesis-Testing, Biomarker-Driven, Talazoparib Combination Trial (TalaCom)	I
NCT02975934	TRITON3: A Multicenter, Randomized, Open Label Phase III Study of Rucaparib Versus Physician's Choice of Therapy for Patients With Metastatic Castration Resistant Prostate Cancer Associated With Homologous Recombination Deficiency	III
NCT03810105	A Phase II Study of Olaparib and Durvalumab in Men With Castration Sensitive Biochemically Recurrent Non-Metastatic Prostate Cancer Harboring Mutations in DNA Damage Repair	II
NCT04336943	Durvalumab (MEDI4736) and Olaparib (AZD2281) for Treatment of Biochemically Recurrent Prostate Cancer in Men Predicted to Have a High Neoantigen Load: A Pilot Study	II
NCT04053322	An International Multicenter Phase II Trial of Durvalumab (MEDI4736) Plus OLAparib Plus Fulvestrant in Metastatic or Locally Advanced ER-positive, HER2-negative Breast Cancer Patients Selected Using Criteria That Predict Sensitivity to Olaparib	II
NCT05011383	High-dose Testosterone in Men With Metastatic Castration-resistant Prostate Cancer and ATM or CDK12 Deficiency	II
NCT03012321	BRCAAway: A Randomized Phase II Trial of Abiraterone, Olaparib, or Abiraterone + Olaparib in Patients With Metastatic Castration-Resistant Prostate Cancer With DNA Repair Defects	II
NCT03553004	Niraparib in Metastatic Pancreatic Cancer After Previous Chemotherapy (NIRA-PANC): A Phase II Trial	II
NCT03601923	Phase II Proof-of-Concept Trial Testing the PARP Inhibitor Niraparib in Patients With Pancreatic Cancer Harboring Deficiencies in Homologous Recombination DNA Repair	II
NCT03840967	A Phase II Study Evaluating Safety and Efficacy of Niraparib in Patients With Previously Treated Homologous Recombination (HR) Defective or Loss of Heterozygosity (LOH) High Metastatic Esophageal/Gastroesophageal Junction/Proximal Gastric Adenocarcinoma	II
NCT03925350	A Phase II Study of Niraparib in Patients With Advanced Melanoma With Genetic Homologous Recombination (HR) Mutation / Alteration	II

Clinical Trials Summary (continued)

ATM mutation (continued)

NCT ID	Title	Phase
NCT04030559	Phase II Trial of PARP Inhibitor Niraparib for Men With High Risk Prostate Cancer and DNA Damage Response Defects	II
NCT04187833	Phase II Trial of Nivolumab in Combination with Talazoparib in Patients with Unresectable or Metastatic Melanoma and Mutations in BRCA or BRCA-ness Genes.	II
NCT03344965	A Phase II Study of Olaparib Monotherapy in Metastatic Breast Cancer Patients With Germline or Somatic Mutations in DNA Repair Genes (Olaparib Expanded)	II
NCT03375307	A Phase II Study of Olaparib (AZD2281) in Patients With Metastatic/Advanced Urothelial Carcinoma With DNA-Repair Defects	II
NCT03786796	Phase II Study of Olaparib in Metastatic Renal Cell Carcinoma Patients Harboring a BAP-1 or Other DNA Repair Gene Mutations (ORCHID)	II
NCT04042831	A Phase II Study of Olaparib in Patients With Advanced Biliary Tract Cancer With Aberrant DNA Repair Gene Mutations	II
NCT04515836	Phase II Trial of Olaparib in Homologous Recombination Deficient (HRD) Malignant Mesothelioma	II
NCT04633902	Phase II Study of Olaparib in Combination With Pembrolizumab in Patients With Advanced Melanoma With Homologous Recombination (HR) Pathway Gene Mutation	II
NCT03248570	Phase II Open Label Study of Pembrolizumab in Patients With Metastatic Castration Resistant Prostate Cancer (mCRPC) With or Without DNA Damage Repair Defects	II
NCT03506997	PERSEUS1: Phase II Trial of the Immune Checkpoint Inhibitor Pembrolizumab for Patients Suffering From Metastatic Prostate Cancer	II
NCT03533946	A Phase II Study of Rucaparib Monotherapy in Nonmetastatic, Hormone-Sensitive Prostate Cancer Demonstrating "BRCAness" Genotype (ROAR)	II
NCT04892693	Talazoparib in Advanced Breast Cancer Patients With Homologous Recombinant Deficiency: A Phase II Clinical and Exploratory Biomarker Study of Talazoparib	II
NCT04740190	Combination Talazoparib - Carboplatin for Recurrent High-grade Glioma With DNA Damage Repair Deficiency (DDRd)	II
NCT04253262	A Phase Ib/II Study of Copanlisib Combined With Rucaparib in Patients With Metastatic Castration-resistant Prostate Cancer	I/II
NCT04169841	Precision Medicine Phase II Study Evaluating the Efficacy of a Double Immunotherapy by Durvalumab and Tremelimumab Combined With Olaparib in Patients With Solid Cancers and Carriers of Homologous Recombination Repair Genes Mutation in Response or Stable After Olaparib Treatment	II
NCT04940637	A Phase II, Open-Label, Single Arm, Prospective, Multicenter Study of Niraparib Plus Dostarlimab in Patients With Advanced NSCLC and/or MPM, and Positive for PD-L1 Expression and Germline or Somatic Mutations in the HRR Genes	II
NCT04939662	Phase II, Single-arm Study of Olaparib and Bevacizumab Combination Therapy in Relapsed Small Cell Lung Cancer Subjects With DNA Damage Response and the Repair Pathway Alteration, ATM Deficiency, SLFN11 Positive, or POU2F3 Positive	II
NCT04666740	A Phase II Trial to Evaluate the Safety and Antitumor Activity of Pembrolizumab and OLApaRib (POLAR) Maintenance for Patients With Metastatic Pancreatic Ductal Adenocarcinoma and Homologous Recombination Deficiency and/or Exceptional Treatment Response to Platinum-Based Therapy	II
NCT02264678	A Modular Phase I, Open-Label, Multicentre Study to Assess the Safety, Tolerability, Pharmacokinetics and Preliminary Anti-tumour Activity of Ceralasertib in Combination With Cytotoxic Chemotherapy and/or DNA Damage Repair/Novel Anti-cancer Agents in Patients With Advanced Solid Malignancies	I

Clinical Trials Summary (continued)

ATM mutation (continued)

NCT ID	Title	Phase
NCT04890613	Phase Ib Expansion Study of CX-5461 in Patients With Solid Tumours and BRCA2 and/or PALB2 Mutation	I
NCT05002868	A Multi-center, Open-label, Phase I/Ib Study to Assess the Safety, Pharmacokinetics and Anti-tumor Activity of RP12146, a Poly (ADP-ribose) Polymerase (PARP) Inhibitor, in Patients With Locally Advanced or Metastatic Solid Tumors	I
NCT03395197	A Phase III, Randomized, Double-blind, Placebo-controlled Study Of Talazoparib With Enzalutamide In Metastatic Castration-resistant Prostate Cancer	III
NCT04821622	TALAPRO-3: A Phase III, Randomized, Double-blind, Study of Talazoparib With Enzalutamide Versus Placebo With Enzalutamide in Men With DDR Gene Mutated Metastatic Castration-sensitive Prostate Cancer	III
NCT04038502	An Open-label, Multicenter Phase II Study to Compare the Efficacy of Carboplatin as First-line Followed by Second-line Olaparib Versus Olaparib as First-line Followed by Second-line Carboplatin in the Treatment of Patients With Castration Resistant Prostate Cancer Containing Homologous Recombination Deficiency	II
No NCT ID	Single-arm Exploratory Study for Sintilimab Second-line or Later-line Treatment of Advanced Non-small Cell Lung Cancer with DDR Pathway Gene Mutation	I/II
NCT04849364	A Phase II Circulating Tumor DNA Enriched, Genomically Directed Post-neoadjuvant Trial for Patients With Residual Triple Negative Breast Cancer (PERSEVERE)	II

ATM splice site mutation

NCT ID	Title	Phase
NCT03682289	Phase II Trial of AZD6738 Alone and in Combination With Olaparib in Patients With Selected Solid Tumor Malignancies	II
NCT04564027	A Modular Phase IIa Multicentre Open-Label Study to Investigate DNA-damage Response Agents (or Combinations) in Patients With Advanced Cancer Whose Tumours Contain Molecular Alterations (PLANETTE)	II
NCT02029001	A Two-period, Multicenter, Randomized, Open-label, Phase II Study Evaluating the Clinical Benefit of a Maintenance Treatment Targeting Tumor Molecular Alterations in Patients with Progressive Locally-advanced or Metastatic Solid Tumors.	II
NCT03742895	A Phase II Study of Olaparib Monotherapy in Participants With Previously Treated, Homologous Recombination Repair Mutation (HRRm) or Homologous Recombination Deficiency (HRD) Positive Advanced Cancer	II
NCT03967938	Efficacy of Olaparib in Advanced Cancers Occurring in Patients With Germline Mutations or Somatic Tumor Mutations in Homologous Recombination Genes	II
NCT02693535	Targeted Agent and Profiling Utilization Registry (TAPUR) Study.	II
NCT02286687	Phase II Study of the PARP Inhibitor Talazoparib in Advanced Cancer Patients With Somatic Alterations in BRCA1/2, Mutations/Deletions in Other BRCA Pathway Genes and Germline Mutation in BRCA1/2 (Not Breast or Ovarian Cancer)	II
NCT02401347	A Phase II Clinical Trial of the PARP Inhibitor Talazoparib in BRCA1 and BRCA2 Wild Type Patients With Advanced Triple Negative Breast Cancer and Homologous Recombination Deficiency or Advanced HER2 Negative Breast Cancer or Other Solid Tumors With a Mutation in Homologous Recombination Pathway Genes Talazoparib Beyond BRCA (TBB) Trial	II

Disclaimer: The data presented here is from a curated knowledgebase of publicly available information, but may not be exhaustive. The data version is 2021.11(003).

Clinical Trials Summary (continued)

ATM splice site mutation (continued)

NCT ID	Title	Phase
NCT04550494	A Pharmacodynamics-Driven Trial of Talazoparib, an Oral PARP Inhibitor, in Patients With Advanced Solid Tumors and Aberrations in Genes Involved in DNA Damage Response	II
NCT03611868	A Phase Ib/II Study of APG-115 in Combination With Pembrolizumab in Patients With Unresectable or Metastatic Melanomas or Advanced Solid Tumors	I/II
NCT03842228	A Phase Ib Biomarker-Driven Combination Trial of Copanlisib, Olaparib, and Durvalumab (MEDI4736) in Patients With Advanced Solid Tumors	I
NCT04586335	Open Label, Phase Ib Study to Evaluate the Safety, Tolerability, Pharmacokinetics and Clinical Activity of CYH33, an Oral a-specific PI3K Inhibitor in Combination With Olaparib, an Oral PARP Inhibitor in Patients With Advanced Solid Tumors.	I
NCT04123366	A Phase II Study of Olaparib in Combination With Pembrolizumab in Participants With Previously Treated, Homologous Recombination Repair Mutation (HRRm) and/or Homologous Recombination Deficiency (HRD)-Positive Advanced Cancer	II
NCT04497116	Phase I/IIa Study of the Safety, Pharmacokinetics, Pharmacodynamics and Preliminary Clinical Activity of RP-3500 Alone or in Combination With Talazoparib in Advanced Solid Tumors With ATR Inhibitor Sensitizing Mutations (TRESR Study)	I/II
NCT03415659	A Phase I, Open-label, Single-center, Single/Multiple-dose, Dose-escalation/Dose-expansion Clinical Study on Tolerance and Pharmacokinetics of HWH340 Tablet in Patients With Advanced Solid Tumors	I
NCT03767075	Basket of Baskets: A Modular, Open-label, Phase II, Multicentre Study To Evaluate Targeted Agents in Molecularly Selected Populations With Advanced Solid Tumours	II
NCT04905914	A Phase I/IIa, Open-Label, Safety, Pharmacokinetic, And Preliminary Efficacy Study Of Oral ATRN-119 In Patients With Advanced Solid Tumors	I/II
NCT04901702	A Randomized Phase I/II Study of Talazoparib or Temozolomide in Combination With Onivyde in Children With Recurrent Solid Malignancies and Ewing Sarcoma	I/II
NCT03155620	NCI-COG Pediatric MATCH (Molecular Analysis for Therapy Choice) Screening Protocol	II
NCT03233204	NCI-COG Pediatric MATCH (Molecular Analysis for Therapy Choice)- Phase 2 Subprotocol of Olaparib in Patients With Tumors Harboring Defects in DNA Damage Repair Genes	II
NCT04267939	An Open-label Phase Ib Study to Determine the Maximum Tolerated and/or Recommended Phase II Dose of the ATR Inhibitor BAY 1895344 in Combination With PARP Inhibitor Niraparib, in Patients With Recurrent Advanced Solid Tumors and Ovarian Cancer	I
NCT04693468	Modular Phase IB Hypothesis-Testing, Biomarker-Driven, Talazoparib Combination Trial (TalaCom)	I
NCT03810105	A Phase II Study of Olaparib and Durvalumab in Men With Castration Sensitive Biochemically Recurrent Non-Metastatic Prostate Cancer Harboring Mutations in DNA Damage Repair	II
NCT02975934	TRITON3: A Multicenter, Randomized, Open Label Phase III Study of Rucaparib Versus Physician's Choice of Therapy for Patients With Metastatic Castration Resistant Prostate Cancer Associated With Homologous Recombination Deficiency	III
NCT04336943	Durvalumab (MEDI4736) and Olaparib (AZD2281) for Treatment of Biochemically Recurrent Prostate Cancer in Men Predicted to Have a High Neoantigen Load: A Pilot Study	II
NCT04053322	An International Multicenter Phase II Trial of Durvalumab (MEDI4736) Plus OLAparib Plus Fulvestrant in Metastatic or Locally Advanced ER-positive, HER2-negative Breast Cancer Patients Selected Using Criteria That Predict Sensitivity to Olaparib	II
NCT05011383	High-dose Testosterone in Men With Metastatic Castration-resistant Prostate Cancer and ATM or CDK12 Deficiency	II

Clinical Trials Summary (continued)

ATM splice site mutation (continued)

NCT ID	Title	Phase
NCT03012321	BRCAAway: A Randomized Phase II Trial of Abiraterone, Olaparib, or Abiraterone + Olaparib in Patients With Metastatic Castration-Resistant Prostate Cancer With DNA Repair Defects	II
NCT03553004	Niraparib in Metastatic Pancreatic Cancer After Previous Chemotherapy (NIRA-PANC): A Phase II Trial	II
NCT03601923	Phase II Proof-of-Concept Trial Testing the PARP Inhibitor Niraparib in Patients With Pancreatic Cancer Harboring Deficiencies in Homologous Recombination DNA Repair	II
NCT03840967	A Phase II Study Evaluating Safety and Efficacy of Niraparib in Patients With Previously Treated Homologous Recombination (HR) Defective or Loss of Heterozygosity (LOH) High Metastatic Esophageal/Gastroesophageal Junction/Proximal Gastric Adenocarcinoma	II
NCT03925350	A Phase II Study of Niraparib in Patients With Advanced Melanoma With Genetic Homologous Recombination (HR) Mutation / Alteration	II
NCT04030559	Phase II Trial of PARP Inhibitor Niraparib for Men With High Risk Prostate Cancer and DNA Damage Response Defects	II
NCT04187833	Phase II Trial of Nivolumab in Combination with Talazoparib in Patients with Unresectable or Metastatic Melanoma and Mutations in BRCA or BRCA-ness Genes.	II
NCT03344965	A Phase II Study of Olaparib Monotherapy in Metastatic Breast Cancer Patients With Germline or Somatic Mutations in DNA Repair Genes (Olaparib Expanded)	II
NCT03375307	A Phase II Study of Olaparib (AZD2281) in Patients With Metastatic/Advanced Urothelial Carcinoma With DNA-Repair Defects	II
NCT03786796	Phase II Study of Olaparib in Metastatic Renal Cell Carcinoma Patients Harboring a BAP-1 or Other DNA Repair Gene Mutations (ORCHID)	II
NCT04042831	A Phase II Study of Olaparib in Patients With Advanced Biliary Tract Cancer With Aberrant DNA Repair Gene Mutations	II
NCT04515836	Phase II Trial of Olaparib in Homologous Recombination Deficient (HRD) Malignant Mesothelioma	II
NCT04633902	Phase II Study of Olaparib in Combination With Pembrolizumab in Patients With Advanced Melanoma With Homologous Recombination (HR) Pathway Gene Mutation	II
NCT03248570	Phase II Open Label Study of Pembrolizumab in Patients With Metastatic Castration Resistant Prostate Cancer (mCRPC) With or Without DNA Damage Repair Defects	II
NCT03506997	PERSEUS1: Phase II Trial of the Immune Checkpoint Inhibitor Pembrolizumab for Patients Suffering From Metastatic Prostate Cancer	II
NCT03533946	A Phase II Study of Rucaparib Monotherapy in Nonmetastatic, Hormone-Sensitive Prostate Cancer Demonstrating "BRCAness" Genotype (ROAR)	II
NCT04892693	Talazoparib in Advanced Breast Cancer Patients With Homologous Recombinant Deficiency: A Phase II Clinical and Exploratory Biomarker Study of Talazoparib	II
NCT04740190	Combination Talazoparib - Carboplatin for Recurrent High-grade Glioma With DNA Damage Repair Deficiency (DDRd)	II
NCT04253262	A Phase Ib/II Study of Copanlisib Combined With Rucaparib in Patients With Metastatic Castration-resistant Prostate Cancer	I/II
NCT04169841	Precision Medicine Phase II Study Evaluating the Efficacy of a Double Immunotherapy by Durvalumab and Tremelimumab Combined With Olaparib in Patients With Solid Cancers and Carriers of Homologous Recombination Repair Genes Mutation in Response or Stable After Olaparib Treatment	II

Disclaimer: The data presented here is from a curated knowledgebase of publicly available information, but may not be exhaustive. The data version is 2021.11(003).

Clinical Trials Summary (continued)

ATM splice site mutation (continued)

NCT ID	Title	Phase
NCT04940637	A Phase II, Open-Label, Single Arm, Prospective, Multicenter Study of Niraparib Plus Dostarlimab in Patients With Advanced NSCLC and/or MPM, and Positive for PD-L1 Expression and Germline or Somatic Mutations in the HRR Genes	II
NCT04939662	Phase II, Single-arm Study of Olaparib and Bevacizumab Combination Therapy in Relapsed Small Cell Lung Cancer Subjects With DNA Damage Response and the Repair Pathway Alteration, ATM Deficiency, SLFN11 Positive, or POU2F3 Positive	II
NCT04666740	A Phase II Trial to Evaluate the Safety and Antitumor Activity of Pembrolizumab and OLApaRib (POLAR) Maintenance for Patients With Metastatic Pancreatic Ductal Adenocarcinoma and Homologous Recombination Deficiency and/or Exceptional Treatment Response to Platinum-Based Therapy	II
NCT02264678	A Modular Phase I, Open-Label, Multicentre Study to Assess the Safety, Tolerability, Pharmacokinetics and Preliminary Anti-tumour Activity of Ceralasertib in Combination With Cytotoxic Chemotherapy and/or DNA Damage Repair/Novel Anti-cancer Agents in Patients With Advanced Solid Malignancies	I
NCT04890613	Phase Ib Expansion Study of CX-5461 in Patients With Solid Tumours and BRCA2 and/or PALB2 Mutation	I
NCT05002868	A Multi-center, Open-label, Phase I/Ib Study to Assess the Safety, Pharmacokinetics and Anti-tumor Activity of RP12146, a Poly (ADP-ribose) Polymerase (PARP) Inhibitor, in Patients With Locally Advanced or Metastatic Solid Tumors	I
NCT03395197	A Phase III, Randomized, Double-blind, Placebo-controlled Study Of Talazoparib With Enzalutamide In Metastatic Castration-resistant Prostate Cancer	III
NCT04821622	TALAPRO-3: A Phase III, Randomized, Double-blind, Study of Talazoparib With Enzalutamide Versus Placebo With Enzalutamide in Men With DDR Gene Mutated Metastatic Castration-sensitive Prostate Cancer	III
NCT04038502	An Open-label, Multicenter Phase II Study to Compare the Efficacy of Carboplatin as First-line Followed by Second-line Olaparib Versus Olaparib as First-line Followed by Second-line Carboplatin in the Treatment of Patients With Castration Resistant Prostate Cancer Containing Homologous Recombination Deficiency	II
No NCT ID	Single-arm Exploratory Study for Sintilimab Second-line or Later-line Treatment of Advanced Non-small Cell Lung Cancer with DDR Pathway Gene Mutation	I/II
NCT04849364	A Phase II Circulating Tumor DNA Enriched, Genomically Directed Post-neoadjuvant Trial for Patients With Residual Triple Negative Breast Cancer (PERSEVERE)	II

ATM truncating mutation

NCT ID	Title	Phase
NCT04550494	A Pharmacodynamics-Driven Trial of Talazoparib, an Oral PARP Inhibitor, in Patients With Advanced Solid Tumors and Aberrations in Genes Involved in DNA Damage Response	II
NCT03682289	Phase II Trial of AZD6738 Alone and in Combination With Olaparib in Patients With Selected Solid Tumor Malignancies	II
NCT04564027	A Modular Phase IIa Multicentre Open-Label Study to Investigate DNA-damage Response Agents (or Combinations) in Patients With Advanced Cancer Whose Tumours Contain Molecular Alterations (PLANETTE)	II
NCT02029001	A Two-period, Multicenter, Randomized, Open-label, Phase II Study Evaluating the Clinical Benefit of a Maintenance Treatment Targeting Tumor Molecular Alterations in Patients with Progressive Locally-advanced or Metastatic Solid Tumors.	II

Clinical Trials Summary (continued)

ATM truncating mutation (continued)

NCT ID	Title	Phase
NCT03742895	A Phase II Study of Olaparib Monotherapy in Participants With Previously Treated, Homologous Recombination Repair Mutation (HRRm) or Homologous Recombination Deficiency (HRD) Positive Advanced Cancer	II
NCT03967938	Efficacy of Olaparib in Advanced Cancers Occurring in Patients With Germline Mutations or Somatic Tumor Mutations in Homologous Recombination Genes	II
NCT02693535	Targeted Agent and Profiling Utilization Registry (TAPUR) Study.	II
NCT02286687	Phase II Study of the PARP Inhibitor Talazoparib in Advanced Cancer Patients With Somatic Alterations in BRCA1/2, Mutations/Deletions in Other BRCA Pathway Genes and Germline Mutation in BRCA1/2 (Not Breast or Ovarian Cancer)	II
NCT02401347	A Phase II Clinical Trial of the PARP Inhibitor Talazoparib in BRCA1 and BRCA2 Wild Type Patients With Advanced Triple Negative Breast Cancer and Homologous Recombination Deficiency or Advanced HER2 Negative Breast Cancer or Other Solid Tumors With a Mutation in Homologous Recombination Pathway Genes Talazoparib Beyond BRCA (TBB) Trial	II
NCT03611868	A Phase Ib/II Study of APG-115 in Combination With Pembrolizumab in Patients With Unresectable or Metastatic Melanomas or Advanced Solid Tumors	I/II
NCT03842228	A Phase Ib Biomarker-Driven Combination Trial of Copanlisib, Olaparib, and Durvalumab (MEDI4736) in Patients With Advanced Solid Tumors	I
NCT04586335	Open Label, Phase Ib Study to Evaluate the Safety, Tolerability, Pharmacokinetics and Clinical Activity of CYH33, an Oral a-specific PI3K Inhibitor in Combination With Olaparib, an Oral PARP Inhibitor in Patients With Advanced Solid Tumors.	I
NCT04123366	A Phase II Study of Olaparib in Combination With Pembrolizumab in Participants With Previously Treated, Homologous Recombination Repair Mutation (HRRm) and/or Homologous Recombination Deficiency (HRD)-Positive Advanced Cancer	II
NCT04497116	Phase I/IIa Study of the Safety, Pharmacokinetics, Pharmacodynamics and Preliminary Clinical Activity of RP-3500 Alone or in Combination With Talazoparib in Advanced Solid Tumors With ATR Inhibitor Sensitizing Mutations (TRESR Study)	I/II
NCT03415659	A Phase I, Open-label, Single-center, Single/Multiple-dose, Dose-escalation/Dose-expansion Clinical Study on Tolerance and Pharmacokinetics of HWH340 Tablet in Patients With Advanced Solid Tumors	I
NCT03767075	Basket of Baskets: A Modular, Open-label, Phase II, Multicentre Study To Evaluate Targeted Agents in Molecularly Selected Populations With Advanced Solid Tumours	II
NCT04905914	A Phase I/IIa, Open-Label, Safety, Pharmacokinetic, And Preliminary Efficacy Study Of Oral ATRN-119 In Patients With Advanced Solid Tumors	I/II
NCT04901702	A Randomized Phase I/II Study of Talazoparib or Temozolomide in Combination With Onivyde in Children With Recurrent Solid Malignancies and Ewing Sarcoma	I/II
NCT03155620	NCI-COG Pediatric MATCH (Molecular Analysis for Therapy Choice) Screening Protocol	II
NCT03233204	NCI-COG Pediatric MATCH (Molecular Analysis for Therapy Choice)- Phase 2 Subprotocol of Olaparib in Patients With Tumors Harboring Defects in DNA Damage Repair Genes	II
NCT04267939	An Open-label Phase Ib Study to Determine the Maximum Tolerated and/or Recommended Phase II Dose of the ATR Inhibitor BAY 1895344 in Combination With PARP Inhibitor Niraparib, in Patients With Recurrent Advanced Solid Tumors and Ovarian Cancer	I
NCT04693468	Modular Phase IB Hypothesis-Testing, Biomarker-Driven, Talazoparib Combination Trial (TalaCom)	I

Disclaimer: The data presented here is from a curated knowledgebase of publicly available information, but may not be exhaustive. The data version is 2021.11(003).

Clinical Trials Summary (continued)

ATM truncating mutation (continued)

NCT ID	Title	Phase
NCT03810105	A Phase II Study of Olaparib and Durvalumab in Men With Castration Sensitive Biochemically Recurrent Non-Metastatic Prostate Cancer Harboring Mutations in DNA Damage Repair	II
NCT03840967	A Phase II Study Evaluating Safety and Efficacy of Niraparib in Patients With Previously Treated Homologous Recombination (HR) Defective or Loss of Heterozygosity (LOH) High Metastatic Esophageal/Gastroesophageal Junction/Proximal Gastric Adenocarcinoma	II
NCT02975934	TRITON3: A Multicenter, Randomized, Open Label Phase III Study of Rucaparib Versus Physician's Choice of Therapy for Patients With Metastatic Castration Resistant Prostate Cancer Associated With Homologous Recombination Deficiency	III
NCT04336943	Durvalumab (MEDI4736) and Olaparib (AZD2281) for Treatment of Biochemically Recurrent Prostate Cancer in Men Predicted to Have a High Neoantigen Load: A Pilot Study	II
NCT04053322	An International Multicenter Phase II Trial of Durvalumab (MEDI4736) Plus OLAparib Plus Fulvestrant in Metastatic or Locally Advanced ER-positive, HER2-negative Breast Cancer Patients Selected Using Criteria That Predict Sensitivity to Olaparib	II
NCT05011383	High-dose Testosterone in Men With Metastatic Castration-resistant Prostate Cancer and ATM or CDK12 Deficiency	II
NCT03012321	BRCAAway: A Randomized Phase II Trial of Abiraterone, Olaparib, or Abiraterone + Olaparib in Patients With Metastatic Castration-Resistant Prostate Cancer With DNA Repair Defects	II
NCT03553004	Niraparib in Metastatic Pancreatic Cancer After Previous Chemotherapy (NIRA-PANC): A Phase II Trial	II
NCT03601923	Phase II Proof-of-Concept Trial Testing the PARP Inhibitor Niraparib in Patients With Pancreatic Cancer Harboring Deficiencies in Homologous Recombination DNA Repair	II
NCT03925350	A Phase II Study of Niraparib in Patients With Advanced Melanoma With Genetic Homologous Recombination (HR) Mutation / Alteration	II
NCT04030559	Phase II Trial of PARP Inhibitor Niraparib for Men With High Risk Prostate Cancer and DNA Damage Response Defects	II
NCT04187833	Phase II Trial of Nivolumab in Combination with Talazoparib in Patients with Unresectable or Metastatic Melanoma and Mutations in BRCA or BRCA-ness Genes.	II
NCT03344965	A Phase II Study of Olaparib Monotherapy in Metastatic Breast Cancer Patients With Germline or Somatic Mutations in DNA Repair Genes (Olaparib Expanded)	II
NCT03375307	A Phase II Study of Olaparib (AZD2281) in Patients With Metastatic/Advanced Urothelial Carcinoma With DNA-Repair Defects	II
NCT03786796	Phase II Study of Olaparib in Metastatic Renal Cell Carcinoma Patients Harboring a BAP-1 or Other DNA Repair Gene Mutations (ORCHID)	II
NCT04042831	A Phase II Study of Olaparib in Patients With Advanced Biliary Tract Cancer With Aberrant DNA Repair Gene Mutations	II
NCT04515836	Phase II Trial of Olaparib in Homologous Recombination Deficient (HRD) Malignant Mesothelioma	II
NCT04633902	Phase II Study of Olaparib in Combination With Pembrolizumab in Patients With Advanced Melanoma With Homologous Recombination (HR) Pathway Gene Mutation	II
NCT03248570	Phase II Open Label Study of Pembrolizumab in Patients With Metastatic Castration Resistant Prostate Cancer (mCRPC) With or Without DNA Damage Repair Defects	II
NCT03506997	PERSEUS1: Phase II Trial of the Immune Checkpoint Inhibitor Pembrolizumab for Patients Suffering From Metastatic Prostate Cancer	II

Clinical Trials Summary (continued)

ATM truncating mutation (continued)

NCT ID	Title	Phase
NCT03533946	A Phase II Study of Rucaparib Monotherapy in Nonmetastatic, Hormone-Sensitive Prostate Cancer Demonstrating "BRCAness" Genotype (ROAR)	II
NCT04892693	Talazoparib in Advanced Breast Cancer Patients With Homologous Recombinant Deficiency: A Phase II Clinical and Exploratory Biomarker Study of Talazoparib	II
NCT04740190	Combination Talazoparib - Carboplatin for Recurrent High-grade Glioma With DNA Damage Repair Deficiency (DDRd)	II
NCT04253262	A Phase Ib/II Study of Copanlisib Combined With Rucaparib in Patients With Metastatic Castration-resistant Prostate Cancer	I/II
NCT04169841	Precision Medicine Phase II Study Evaluating the Efficacy of a Double Immunotherapy by Durvalumab and Tremelimumab Combined With Olaparib in Patients With Solid Cancers and Carriers of Homologous Recombination Repair Genes Mutation in Response or Stable After Olaparib Treatment	II
NCT04940637	A Phase II, Open-Label, Single Arm, Prospective, Multicenter Study of Niraparib Plus Dostarlimab in Patients With Advanced NSCLC and/or MPM, and Positive for PD-L1 Expression and Germline or Somatic Mutations in the HRR Genes	II
NCT04939662	Phase II, Single-arm Study of Olaparib and Bevacizumab Combination Therapy in Relapsed Small Cell Lung Cancer Subjects With DNA Damage Response and the Repair Pathway Alteration, ATM Deficiency, SLFN11 Positive, or POU2F3 Positive	II
NCT04666740	A Phase II Trial to Evaluate the Safety and Antitumor Activity of Pembrolizumab and OLApaRib (POLAR) Maintenance for Patients With Metastatic Pancreatic Ductal Adenocarcinoma and Homologous Recombination Deficiency and/or Exceptional Treatment Response to Platinum-Based Therapy	II
NCT02264678	A Modular Phase I, Open-Label, Multicentre Study to Assess the Safety, Tolerability, Pharmacokinetics and Preliminary Anti-tumour Activity of Ceralasertib in Combination With Cytotoxic Chemotherapy and/or DNA Damage Repair/Novel Anti-cancer Agents in Patients With Advanced Solid Malignancies	I
NCT04890613	Phase Ib Expansion Study of CX-5461 in Patients With Solid Tumours and BRCA2 and/or PALB2 Mutation	I
NCT05002868	A Multi-center, Open-label, Phase I/Ib Study to Assess the Safety, Pharmacokinetics and Anti-tumor Activity of RP12146, a Poly (ADP-ribose) Polymerase (PARP) Inhibitor, in Patients With Locally Advanced or Metastatic Solid Tumors	I
NCT03395197	A Phase III, Randomized, Double-blind, Placebo-controlled Study Of Talazoparib With Enzalutamide In Metastatic Castration-resistant Prostate Cancer	III
NCT04821622	TALAPRO-3: A Phase III, Randomized, Double-blind, Study of Talazoparib With Enzalutamide Versus Placebo With Enzalutamide in Men With DDR Gene Mutated Metastatic Castration-sensitive Prostate Cancer	III
NCT04038502	An Open-label, Multicenter Phase II Study to Compare the Efficacy of Carboplatin as First-line Followed by Second-line Olaparib Versus Olaparib as First-line Followed by Second-line Carboplatin in the Treatment of Patients With Castration Resistant Prostate Cancer Containing Homologous Recombination Deficiency	II
No NCT ID	Single-arm Exploratory Study for Sintilimab Second-line or Later-line Treatment of Advanced Non-small Cell Lung Cancer with DDR Pathway Gene Mutation	I/II
NCT04849364	A Phase II Circulating Tumor DNA Enriched, Genomically Directed Post-neoadjuvant Trial for Patients With Residual Triple Negative Breast Cancer (PERSEVERE)	II

Disclaimer: The data presented here is from a curated knowledgebase of publicly available information, but may not be exhaustive. The data version is 2021.11(003).

Clinical Trials Summary (continued)

ATM deletion

NCT ID	Title	Phase
NCT02693535	Targeted Agent and Profiling Utilization Registry (TAPUR) Study.	II
NCT02286687	Phase II Study of the PARP Inhibitor Talazoparib in Advanced Cancer Patients With Somatic Alterations in BRCA1/2, Mutations/Deletions in Other BRCA Pathway Genes and Germline Mutation in BRCA1/2 (Not Breast or Ovarian Cancer)	II
NCT04497116	Phase I/IIa Study of the Safety, Pharmacokinetics, Pharmacodynamics and Preliminary Clinical Activity of RP-3500 Alone or in Combination With Talazoparib in Advanced Solid Tumors With ATR Inhibitor Sensitizing Mutations (TRESR Study)	I/II
NCT04901702	A Randomized Phase I/II Study of Talazoparib or Temozolomide in Combination With Onivyde in Children With Recurrent Solid Malignancies and Ewing Sarcoma	I/II
NCT03155620	NCI-COG Pediatric MATCH (Molecular Analysis for Therapy Choice) Screening Protocol	II
NCT03233204	NCI-COG Pediatric MATCH (Molecular Analysis for Therapy Choice)- Phase 2 Subprotocol of Olaparib in Patients With Tumors Harboring Defects in DNA Damage Repair Genes	II
NCT04267939	An Open-label Phase Ib Study to Determine the Maximum Tolerated and/or Recommended Phase II Dose of the ATR Inhibitor BAY 1895344 in Combination With PARP Inhibitor Niraparib, in Patients With Recurrent Advanced Solid Tumors and Ovarian Cancer	I
NCT04693468	Modular Phase IB Hypothesis-Testing, Biomarker-Driven, Talazoparib Combination Trial (TalaCom)	I
NCT03810105	A Phase II Study of Olaparib and Durvalumab in Men With Castration Sensitive Biochemically Recurrent Non-Metastatic Prostate Cancer Harboring Mutations in DNA Damage Repair	II
NCT03012321	BRCAAway: A Randomized Phase II Trial of Abiraterone, Olaparib, or Abiraterone + Olaparib in Patients With Metastatic Castration-Resistant Prostate Cancer With DNA Repair Defects	II
NCT03840967	A Phase II Study Evaluating Safety and Efficacy of Niraparib in Patients With Previously Treated Homologous Recombination (HR) Defective or Loss of Heterozygosity (LOH) High Metastatic Esophageal/Gastroesophageal Junction/Proximal Gastric Adenocarcinoma	II
NCT04030559	Phase II Trial of PARP Inhibitor Niraparib for Men With High Risk Prostate Cancer and DNA Damage Response Defects	II
NCT03344965	A Phase II Study of Olaparib Monotherapy in Metastatic Breast Cancer Patients With Germline or Somatic Mutations in DNA Repair Genes (Olaparib Expanded)	II
NCT04633902	Phase II Study of Olaparib in Combination With Pembrolizumab in Patients With Advanced Melanoma With Homologous Recombination (HR) Pathway Gene Mutation	II
NCT03248570	Phase II Open Label Study of Pembrolizumab in Patients With Metastatic Castration Resistant Prostate Cancer (mCRPC) With or Without DNA Damage Repair Defects	II
NCT04740190	Combination Talazoparib - Carboplatin for Recurrent High-grade Glioma With DNA Damage Repair Deficiency (DDRd)	II
No NCT ID	An Open-label, Single-arm Phase Ia/Ib Clinical Study: Phase Ia Dose Exploration Study to Evaluate the SC0245 Tablets in Patients with Advanced Malignant Solid Tumor, and Phase Ib Clinical Study in Gastric Cancer Patients with ATM Deficiency and Esophageal Carcinoma Patients with p53 Mutation	I
NCT04666740	A Phase II Trial to Evaluate the Safety and Antitumor Activity of Pembrolizumab and OLApaRib (POLAR) Maintenance for Patients With Metastatic Pancreatic Ductal Adenocarcinoma and Homologous Recombination Deficiency and/or Exceptional Treatment Response to Platinum-Based Therapy	II
NCT03506997	PERSEUS1: Phase II Trial of the Immune Checkpoint Inhibitor Pembrolizumab for Patients Suffering From Metastatic Prostate Cancer	II

Clinical Trials Summary (continued)

ATM deletion (continued)

NCT ID	Title	Phase
NCT04849364	A Phase II Circulating Tumor DNA Enriched, Genomically Directed Post-neoadjuvant Trial for Patients With Residual Triple Negative Breast Cancer (PERSEVERE)	II
NCT05002868	A Multi-center, Open-label, Phase I/Ib Study to Assess the Safety, Pharmacokinetics and Anti-tumor Activity of RP12146, a Poly (ADP-ribose) Polymerase (PARP) Inhibitor, in Patients With Locally Advanced or Metastatic Solid Tumors	I

ATM fusion

NCT ID	Title	Phase
NCT04497116	Phase I/IIa Study of the Safety, Pharmacokinetics, Pharmacodynamics and Preliminary Clinical Activity of RP-3500 Alone or in Combination With Talazoparib in Advanced Solid Tumors With ATR Inhibitor Sensitizing Mutations (TRESR Study)	I/II
NCT04901702	A Randomized Phase I/II Study of Talazoparib or Temozolomide in Combination With Onivyde in Children With Recurrent Solid Malignancies and Ewing Sarcoma	I/II
NCT03155620	NCI-COG Pediatric MATCH (Molecular Analysis for Therapy Choice) Screening Protocol	II
NCT03233204	NCI-COG Pediatric MATCH (Molecular Analysis for Therapy Choice)- Phase 2 Subprotocol of Olaparib in Patients With Tumors Harboring Defects in DNA Damage Repair Genes	II
NCT04267939	An Open-label Phase Ib Study to Determine the Maximum Tolerated and/or Recommended Phase II Dose of the ATR Inhibitor BAY 1895344 in Combination With PARP Inhibitor Niraparib, in Patients With Recurrent Advanced Solid Tumors and Ovarian Cancer	I
NCT04693468	Modular Phase IB Hypothesis-Testing, Biomarker-Driven, Talazoparib Combination Trial (TalaCom)	I
NCT03840967	A Phase II Study Evaluating Safety and Efficacy of Niraparib in Patients With Previously Treated Homologous Recombination (HR) Defective or Loss of Heterozygosity (LOH) High Metastatic Esophageal/Gastroesophageal Junction/Proximal Gastric Adenocarcinoma	II
NCT04666740	A Phase II Trial to Evaluate the Safety and Antitumor Activity of Pembrolizumab and OLApaRib (POLAR) Maintenance for Patients With Metastatic Pancreatic Ductal Adenocarcinoma and Homologous Recombination Deficiency and/or Exceptional Treatment Response to Platinum-Based Therapy	II
NCT04633902	Phase II Study of Olaparib in Combination With Pembrolizumab in Patients With Advanced Melanoma With Homologous Recombination (HR) Pathway Gene Mutation	II
NCT03506997	PERSEUS1: Phase II Trial of the Immune Checkpoint Inhibitor Pembrolizumab for Patients Suffering From Metastatic Prostate Cancer	II
NCT04849364	A Phase II Circulating Tumor DNA Enriched, Genomically Directed Post-neoadjuvant Trial for Patients With Residual Triple Negative Breast Cancer (PERSEVERE)	II
NCT05002868	A Multi-center, Open-label, Phase I/Ib Study to Assess the Safety, Pharmacokinetics and Anti-tumor Activity of RP12146, a Poly (ADP-ribose) Polymerase (PARP) Inhibitor, in Patients With Locally Advanced or Metastatic Solid Tumors	I

Disclaimer: The data presented here is from a curated knowledgebase of publicly available information, but may not be exhaustive. The data version is 2021.11(003).

Clinical Trials Summary (continued)

ATM aberration

NCT ID	Title	Phase
NCT04497116	Phase I/IIa Study of the Safety, Pharmacokinetics, Pharmacodynamics and Preliminary Clinical Activity of RP-3500 Alone or in Combination With Talazoparib in Advanced Solid Tumors With ATR Inhibitor Sensitizing Mutations (TRESR Study)	I/II
NCT04901702	A Randomized Phase I/II Study of Talazoparib or Temozolomide in Combination With Onivyde in Children With Recurrent Solid Malignancies and Ewing Sarcoma	I/II
NCT03155620	NCI-COG Pediatric MATCH (Molecular Analysis for Therapy Choice) Screening Protocol	II
NCT03233204	NCI-COG Pediatric MATCH (Molecular Analysis for Therapy Choice)- Phase 2 Subprotocol of Olaparib in Patients With Tumors Harboring Defects in DNA Damage Repair Genes	II
NCT04267939	An Open-label Phase Ib Study to Determine the Maximum Tolerated and/or Recommended Phase II Dose of the ATR Inhibitor BAY 1895344 in Combination With PARP Inhibitor Niraparib, in Patients With Recurrent Advanced Solid Tumors and Ovarian Cancer	I
NCT04693468	Modular Phase IB Hypothesis-Testing, Biomarker-Driven, Talazoparib Combination Trial (TalaCom)	I
NCT04666740	A Phase II Trial to Evaluate the Safety and Antitumor Activity of Pembrolizumab and OLApaRib (POLAR) Maintenance for Patients With Metastatic Pancreatic Ductal Adenocarcinoma and Homologous Recombination Deficiency and/or Exceptional Treatment Response to Platinum-Based Therapy	II
NCT04633902	Phase II Study of Olaparib in Combination With Pembrolizumab in Patients With Advanced Melanoma With Homologous Recombination (HR) Pathway Gene Mutation	II
NCT03506997	PERSEUS1: Phase II Trial of the Immune Checkpoint Inhibitor Pembrolizumab for Patients Suffering From Metastatic Prostate Cancer	II
NCT04849364	A Phase II Circulating Tumor DNA Enriched, Genomically Directed Post-neoadjuvant Trial for Patients With Residual Triple Negative Breast Cancer (PERSEVERE)	II
NCT05002868	A Multi-center, Open-label, Phase I/Ib Study to Assess the Safety, Pharmacokinetics and Anti-tumor Activity of RP12146, a Poly (ADP-ribose) Polymerase (PARP) Inhibitor, in Patients With Locally Advanced or Metastatic Solid Tumors	I

Sample Cancer Type: Unknown Primary Origin

Table of Contents

Table of Contents	Page
Relevant Therapy Details	2
Alert Details	26
Clinical Trials	27
Relevant Therapy Summary	112
Clinical Trials Summary	119

Report Highlights

6 Relevant Biomarkers
4 Therapies Available
79 Clinical Trials

Relevant Biomarkers

Genomic Alteration	Relevant Therapies (In this cancer type)	Relevant Therapies (In other cancer type)	Clinical Trials
BRCA1 mutation<br>**Prognostic significance:** None<br>**Diagnostic significance:** None	None	**bevacizumab + olaparib** [1, 2]<br>**niraparib** [1]<br>**olaparib** [1, 2]<br>**rucaparib** [1, 2]	77
BRCA1 splice site mutation<br>**Prognostic significance:** None<br>**Diagnostic significance:** None	None	**bevacizumab + olaparib** [1, 2]<br>**niraparib** [1]<br>**olaparib** [1, 2]<br>**rucaparib** [1, 2]	77
BRCA1 truncating mutation<br>**Prognostic significance:** None<br>**Diagnostic significance:** None	None	**bevacizumab + olaparib** [1, 2]<br>**niraparib** [1]<br>**olaparib** [1, 2]<br>**rucaparib** [1, 2]	77
BRCA1 deletion<br>**Prognostic significance:** None<br>**Diagnostic significance:** None	None	None	21
BRCA1 fusion<br>**Prognostic significance:** None<br>**Diagnostic significance:** None	None	None	16
BRCA1 aberration<br>**Prognostic significance:** None<br>**Diagnostic significance:** None	None	None	13

Public data sources included in relevant therapies: FDA1, NCCN, **EMA**2, ESMO
Public data sources included in prognostic and diagnostic significance: NCCN, ESMO

Disclaimer: The data presented here is from a curated knowledgebase of publicly available information, but may not be exhaustive. The data version is 2021.11(003).The content of this report has not been evaluated or approved by the FDA or other regulatory agencies.

Relevant Therapy Details

Current FDA Information

● In this cancer type ○ In other cancer type ◐ In this cancer type and other cancer types

FDA information is current as of 2021-10-13. For the most up-to-date information, search www.fda.gov.

BRCA1 mutation

○ niraparib

Cancer type: Ovarian Cancer **Label as of:** 2021-07-27 **Variant class:** BRCA1 mutation

Indications and usage:

ZEJULA® is a poly (ADP-ribose) polymerase (PARP) inhibitor indicated:

- for the maintenance treatment of adult patients with advanced epithelial ovarian, fallopian tube, or primary peritoneal cancer who are in a complete or partial response to first-line platinum-based chemotherapy.
- for the maintenance treatment of adult patients with recurrent epithelial ovarian, fallopian tube, or primary peritoneal cancer who are in a complete or partial response to platinum-based chemotherapy.
- for the treatment of adult patients with advanced ovarian, fallopian tube, or primary peritoneal cancer who have been treated with 3 or more prior chemotherapy regimens and whose cancer is associated with homologous recombination deficiency (HRD) positive status defined by either:
  - a deleterious or suspected deleterious BRCA mutation, or
  - genomic instability and who have progressed more than 6 months after response to the last platinum-based chemotherapy.

Select patients for therapy based on an FDA-approved companion diagnostic for ZEJULA®.

Reference:

https://www.accessdata.fda.gov/drugsatfda_docs/label/2021/208447s022s024lbl.pdf

BRCA1 mutation (continued)

○ olaparib, bevacizumab + olaparib

Cancer type: Castration-Resistant Prostate Cancer, Ovarian Cancer **Label as of:** 2021-03-11 **Variant class:** BRCA1 mutation

Indications and usage:

LYNPARZA® is a poly (ADP-ribose) polymerase (PARP) inhibitor indicated:

Ovarian cancer

- for the maintenance treatment of adult patients with deleterious or suspected deleterious germline or somatic BRCA-mutated advanced epithelial ovarian, fallopian tube or primary peritoneal cancer who are in complete or partial response to first-line platinum-based chemotherapy. Select patients for therapy based on an FDA-approved companion diagnostic for LYNPARZA®.
- in combination with bevacizumab for the maintenance treatment of adult patients with advanced epithelial ovarian, fallopian tube or primary peritoneal cancer who are in complete or partial response to first-line platinum-based chemotherapy and whose cancer is associated with homologous recombination deficiency (HRD)-positive status defined by either:
  - a deleterious or suspected deleterious BRCA mutation, and/or
  - genomic instability.

Select patients for therapy based on an FDA-approved companion diagnostic for LYNPARZA®.

- for the maintenance treatment of adult patients with recurrent epithelial ovarian, fallopian tube or primary peritoneal cancer, who are in complete or partial response to platinum-based chemotherapy.
- for the treatment of adult patients with deleterious or suspected deleterious germline BRCA-mutated (gBRCAm) advanced ovarian cancer who have been treated with three or more prior lines of chemotherapy. Select patients for therapy based on an FDA-approved companion diagnostic for LYNPARZA®.

Breast cancer

- for the treatment of adult patients with deleterious or suspected deleterious gBRCAm, HER2-negative metastatic breast cancer who have been treated with chemotherapy in the neoadjuvant, adjuvant or metastatic setting. Patients with hormone receptor (HR)-positive breast cancer should have been treated with a prior endocrine therapy or be considered inappropriate for endocrine therapy. Select patients for therapy based on an FDA-approved companion diagnostic for LYNPARZA®.

Pancreatic cancer

- for the maintenance treatment of adult patients with deleterious or suspected deleterious gBRCAm metastatic pancreatic adenocarcinoma whose disease has not progressed on at least 16 weeks of a first-line platinum-based chemotherapy regimen. Select patients for therapy based on an FDA-approved companion diagnostic for LYNPARZA®.

Prostate cancer

- for the treatment of adult patients with deleterious or suspected deleterious germline or somatic homologous recombination repair (HRR) gene-mutated metastatic castration-resistant prostate cancer (mCRPC) who have progressed following prior treatment with enzalutamide or abiraterone. Select patients for therapy based on an FDA-approved companion diagnostic for LYNPARZA®.

Reference:

https://www.accessdata.fda.gov/drugsatfda_docs/label/2021/208558s019s020lbl.pdf

Disclaimer: The data presented here is from a curated knowledgebase of publicly available information, but may not be exhaustive. The data version is 2021.11(003).

BRCA1 mutation (continued)

rucaparib

Cancer type: Castration-Resistant Prostate Cancer, Ovarian Cancer **Label as of:** 2021-09-30 **Variant class:** BRCA1 mutation

Indications and usage:

RUBRACA® is a poly (ADP-ribose) polymerase (PARP) inhibitor indicated:

Ovarian cancer

- for the maintenance treatment of adult patients with recurrent epithelial ovarian, fallopian tube, or primary peritoneal cancer who are in a complete or partial response to platinum-based chemotherapy.
- for the treatment of adult patients with a deleterious BRCA mutation (germline and/or somatic)-associated epithelial ovarian, fallopian tube, or primary peritoneal cancer who have been treated with two or more chemotherapies. Select patients for therapy based on an FDA-approved companion diagnostic for RUBRACA®.

Prostate cancer

- for the treatment of adult patients with a deleterious BRCA mutation (germline and/or somatic)-associated metastatic castration-resistant prostate cancer (mCRPC) who have been treated with androgen receptor-directed therapy and a taxane-based chemotherapy. Select patients for therapy based on an FDA-approved companion diagnostic for RUBRACA®.

This indication is approved under accelerated approval based on objective response rate and duration of response. Continued approval for this indication may be contingent upon verification and description of clinical benefit in confirmatory trials.

Reference:

https://www.accessdata.fda.gov/drugsatfda_docs/label/2021/209115s009lbl.pdf

BRCA1 splice site mutation

niraparib

Cancer type: Ovarian Cancer **Label as of:** 2021-07-27 **Variant class:** BRCA1 mutation

Indications and usage:

ZEJULA® is a poly (ADP-ribose) polymerase (PARP) inhibitor indicated:

- for the maintenance treatment of adult patients with advanced epithelial ovarian, fallopian tube, or primary peritoneal cancer who are in a complete or partial response to first-line platinum-based chemotherapy.
- for the maintenance treatment of adult patients with recurrent epithelial ovarian, fallopian tube, or primary peritoneal cancer who are in a complete or partial response to platinum-based chemotherapy.
- for the treatment of adult patients with advanced ovarian, fallopian tube, or primary peritoneal cancer who have been treated with 3 or more prior chemotherapy regimens and whose cancer is associated with homologous recombination deficiency (HRD) positive status defined by either:
  - a deleterious or suspected deleterious BRCA mutation, or
  - genomic instability and who have progressed more than 6 months after response to the last platinum-based chemotherapy.

Select patients for therapy based on an FDA-approved companion diagnostic for ZEJULA®.

Reference:

https://www.accessdata.fda.gov/drugsatfda_docs/label/2021/208447s022s024lbl.pdf

Disclaimer: The data presented here is from a curated knowledgebase of publicly available information, but may not be exhaustive. The data version is 2021.11(003).

BRCA1 splice site mutation (continued)

○ olaparib, bevacizumab + olaparib

Cancer type: Castration-Resistant Prostate Cancer, Ovarian Cancer **Label as of:** 2021-03-11 **Variant class:** BRCA1 mutation

Indications and usage:

LYNPARZA® is a poly (ADP-ribose) polymerase (PARP) inhibitor indicated:

Ovarian cancer

- for the maintenance treatment of adult patients with deleterious or suspected deleterious germline or somatic BRCA-mutated advanced epithelial ovarian, fallopian tube or primary peritoneal cancer who are in complete or partial response to first-line platinum-based chemotherapy. Select patients for therapy based on an FDA-approved companion diagnostic for LYNPARZA®.
- in combination with bevacizumab for the maintenance treatment of adult patients with advanced epithelial ovarian, fallopian tube or primary peritoneal cancer who are in complete or partial response to first-line platinum-based chemotherapy and whose cancer is associated with homologous recombination deficiency (HRD)-positive status defined by either:
  - a deleterious or suspected deleterious BRCA mutation, and/or
  - genomic instability.

Select patients for therapy based on an FDA-approved companion diagnostic for LYNPARZA®.

- for the maintenance treatment of adult patients with recurrent epithelial ovarian, fallopian tube or primary peritoneal cancer, who are in complete or partial response to platinum-based chemotherapy.
- for the treatment of adult patients with deleterious or suspected deleterious germline BRCA-mutated (gBRCAm) advanced ovarian cancer who have been treated with three or more prior lines of chemotherapy. Select patients for therapy based on an FDA-approved companion diagnostic for LYNPARZA®.

Breast cancer

- for the treatment of adult patients with deleterious or suspected deleterious gBRCAm, HER2-negative metastatic breast cancer who have been treated with chemotherapy in the neoadjuvant, adjuvant or metastatic setting. Patients with hormone receptor (HR)-positive breast cancer should have been treated with a prior endocrine therapy or be considered inappropriate for endocrine therapy. Select patients for therapy based on an FDA-approved companion diagnostic for LYNPARZA®.

Pancreatic cancer

- for the maintenance treatment of adult patients with deleterious or suspected deleterious gBRCAm metastatic pancreatic adenocarcinoma whose disease has not progressed on at least 16 weeks of a first-line platinum-based chemotherapy regimen. Select patients for therapy based on an FDA-approved companion diagnostic for LYNPARZA®.

Prostate cancer

- for the treatment of adult patients with deleterious or suspected deleterious germline or somatic homologous recombination repair (HRR) gene-mutated metastatic castration-resistant prostate cancer (mCRPC) who have progressed following prior treatment with enzalutamide or abiraterone. Select patients for therapy based on an FDA-approved companion diagnostic for LYNPARZA®.

Reference:

https://www.accessdata.fda.gov/drugsatfda_docs/label/2021/208558s019s020lbl.pdf

BRCA1 splice site mutation (continued)

rucaparib

Cancer type: Castration-Resistant Prostate Cancer, Ovarian Cancer **Label as of:** 2021-09-30 **Variant class:** BRCA1 mutation

Indications and usage:

RUBRACA® is a poly (ADP-ribose) polymerase (PARP) inhibitor indicated:

Ovarian cancer

- for the maintenance treatment of adult patients with recurrent epithelial ovarian, fallopian tube, or primary peritoneal cancer who are in a complete or partial response to platinum-based chemotherapy.
- for the treatment of adult patients with a deleterious BRCA mutation (germline and/or somatic)-associated epithelial ovarian, fallopian tube, or primary peritoneal cancer who have been treated with two or more chemotherapies. Select patients for therapy based on an FDA-approved companion diagnostic for RUBRACA®.

Prostate cancer

- for the treatment of adult patients with a deleterious BRCA mutation (germline and/or somatic)-associated metastatic castration-resistant prostate cancer (mCRPC) who have been treated with androgen receptor-directed therapy and a taxane-based chemotherapy. Select patients for therapy based on an FDA-approved companion diagnostic for RUBRACA®.

This indication is approved under accelerated approval based on objective response rate and duration of response. Continued approval for this indication may be contingent upon verification and description of clinical benefit in confirmatory trials.

Reference:

https://www.accessdata.fda.gov/drugsatfda_docs/label/2021/209115s009lbl.pdf

BRCA1 truncating mutation

niraparib

Cancer type: Ovarian Cancer **Label as of:** 2021-07-27 **Variant class:** BRCA1 mutation

Indications and usage:

ZEJULA® is a poly (ADP-ribose) polymerase (PARP) inhibitor indicated:

- for the maintenance treatment of adult patients with advanced epithelial ovarian, fallopian tube, or primary peritoneal cancer who are in a complete or partial response to first-line platinum-based chemotherapy.
- for the maintenance treatment of adult patients with recurrent epithelial ovarian, fallopian tube, or primary peritoneal cancer who are in a complete or partial response to platinum-based chemotherapy.
- for the treatment of adult patients with advanced ovarian, fallopian tube, or primary peritoneal cancer who have been treated with 3 or more prior chemotherapy regimens and whose cancer is associated with homologous recombination deficiency (HRD) positive status defined by either:
    - a deleterious or suspected deleterious BRCA mutation, or
    - genomic instability and who have progressed more than 6 months after response to the last platinum-based chemotherapy.

Select patients for therapy based on an FDA-approved companion diagnostic for ZEJULA®.

Reference:

https://www.accessdata.fda.gov/drugsatfda_docs/label/2021/208447s022s024lbl.pdf

Disclaimer: The data presented here is from a curated knowledgebase of publicly available information, but may not be exhaustive. The data version is 2021.11(003).

BRCA1 truncating mutation (continued)

○ olaparib, bevacizumab + olaparib

Cancer type: Castration-Resistant Prostate Cancer, Ovarian Cancer **Label as of:** 2021-03-11 **Variant class:** BRCA1 mutation

Indications and usage:

LYNPARZA® is a poly (ADP-ribose) polymerase (PARP) inhibitor indicated:

Ovarian cancer

- for the maintenance treatment of adult patients with deleterious or suspected deleterious germline or somatic BRCA-mutated advanced epithelial ovarian, fallopian tube or primary peritoneal cancer who are in complete or partial response to first-line platinum-based chemotherapy. Select patients for therapy based on an FDA-approved companion diagnostic for LYNPARZA®.
- in combination with bevacizumab for the maintenance treatment of adult patients with advanced epithelial ovarian, fallopian tube or primary peritoneal cancer who are in complete or partial response to first-line platinum-based chemotherapy and whose cancer is associated with homologous recombination deficiency (HRD)-positive status defined by either:
  - a deleterious or suspected deleterious BRCA mutation, and/or
  - genomic instability.

Select patients for therapy based on an FDA-approved companion diagnostic for LYNPARZA®.

- for the maintenance treatment of adult patients with recurrent epithelial ovarian, fallopian tube or primary peritoneal cancer, who are in complete or partial response to platinum-based chemotherapy.
- for the treatment of adult patients with deleterious or suspected deleterious germline BRCA-mutated (gBRCAm) advanced ovarian cancer who have been treated with three or more prior lines of chemotherapy. Select patients for therapy based on an FDA-approved companion diagnostic for LYNPARZA®.

Breast cancer

- for the treatment of adult patients with deleterious or suspected deleterious gBRCAm, HER2-negative metastatic breast cancer who have been treated with chemotherapy in the neoadjuvant, adjuvant or metastatic setting. Patients with hormone receptor (HR)-positive breast cancer should have been treated with a prior endocrine therapy or be considered inappropriate for endocrine therapy. Select patients for therapy based on an FDA-approved companion diagnostic for LYNPARZA®.

Pancreatic cancer

- for the maintenance treatment of adult patients with deleterious or suspected deleterious gBRCAm metastatic pancreatic adenocarcinoma whose disease has not progressed on at least 16 weeks of a first-line platinum-based chemotherapy regimen. Select patients for therapy based on an FDA-approved companion diagnostic for LYNPARZA®.

Prostate cancer

- for the treatment of adult patients with deleterious or suspected deleterious germline or somatic homologous recombination repair (HRR) gene-mutated metastatic castration-resistant prostate cancer (mCRPC) who have progressed following prior treatment with enzalutamide or abiraterone. Select patients for therapy based on an FDA-approved companion diagnostic for LYNPARZA®.

Reference:

https://www.accessdata.fda.gov/drugsatfda_docs/label/2021/208558s019s020lbl.pdf

BRCA1 truncating mutation (continued)

○ rucaparib

Cancer type: Castration-Resistant Prostate Cancer, Ovarian Cancer **Label as of:** 2021-09-30 **Variant class:** BRCA1 mutation

Indications and usage:

RUBRACA® is a poly (ADP-ribose) polymerase (PARP) inhibitor indicated:

Ovarian cancer

- for the maintenance treatment of adult patients with recurrent epithelial ovarian, fallopian tube, or primary peritoneal cancer who are in a complete or partial response to platinum-based chemotherapy.
- for the treatment of adult patients with a deleterious BRCA mutation (germline and/or somatic)-associated epithelial ovarian, fallopian tube, or primary peritoneal cancer who have been treated with two or more chemotherapies. Select patients for therapy based on an FDA-approved companion diagnostic for RUBRACA®.

Prostate cancer

- for the treatment of adult patients with a deleterious BRCA mutation (germline and/or somatic)-associated metastatic castration-resistant prostate cancer (mCRPC) who have been treated with androgen receptor-directed therapy and a taxane-based chemotherapy. Select patients for therapy based on an FDA-approved companion diagnostic for RUBRACA®.

This indication is approved under accelerated approval based on objective response rate and duration of response. Continued approval for this indication may be contingent upon verification and description of clinical benefit in confirmatory trials.

Reference:

https://www.accessdata.fda.gov/drugsatfda_docs/label/2021/209115s009lbl.pdf

Current NCCN Information

● In this cancer type ○ In other cancer type ◐ In this cancer type and other cancer types

NCCN information is current as of 2021-10-01. For the most up-to-date information, search www.nccn.org.
For NCCN International Adaptations & Translations, search www.nccn.org/global/international_adaptations.aspx.

BRCA1 mutation

○ bevacizumab + olaparib

Cancer type: Ovarian Cancer **Variant class:** BRCA1 mutation

NCCN Recommendation category: 1

Population segment (Line of therapy):
- Epithelial, Fallopian Tube, Primary Peritoneal; Stage II, Stage III, Stage IV; Partial response, Complete response (Maintenance therapy)

Reference: NCCN Guidelines® - NCCN-Ovarian Cancer [Version 3.2021]

○ niraparib

Cancer type: Ovarian Cancer **Variant class:** BRCA1 mutation

NCCN Recommendation category: 1

Population segment (Line of therapy):
- Epithelial, Fallopian Tube, Primary Peritoneal; Persistent, Recurrent, Partial response, Complete response (Maintenance therapy); Useful in certain circumstances

Reference: NCCN Guidelines® - NCCN-Ovarian Cancer [Version 3.2021]

○ niraparib

Cancer type: Ovarian Cancer **Variant class:** BRCA1 mutation

NCCN Recommendation category: 1

Population segment (Line of therapy):
- Epithelial, Fallopian Tube, Primary Peritoneal; Stage II, Stage III, Stage IV; Partial response, Complete response (Maintenance therapy)

Reference: NCCN Guidelines® - NCCN-Ovarian Cancer [Version 3.2021]

○ olaparib

Cancer type: Ovarian Cancer **Variant class:** BRCA1 mutation

NCCN Recommendation category: 1

Population segment (Line of therapy):
- Epithelial, Fallopian Tube, Primary Peritoneal; Persistent, Recurrent, Partial response, Complete response (Maintenance therapy); Useful in certain circumstances

Reference: NCCN Guidelines® - NCCN-Ovarian Cancer [Version 3.2021]

Disclaimer: The data presented here is from a curated knowledgebase of publicly available information, but may not be exhaustive. The data version is 2021.11(003).

BRCA1 mutation (continued)

olaparib

Cancer type: Ovarian Cancer **Variant class:** BRCA1 mutation

NCCN Recommendation category: 1

Population segment (Line of therapy):

- Epithelial, Fallopian Tube, Primary Peritoneal; Stage II, Stage III, Stage IV; Partial response, Complete response (Maintenance therapy)

Reference: NCCN Guidelines® - NCCN-Ovarian Cancer [Version 3.2021]

olaparib

Cancer type: Castration-Resistant Prostate Cancer **Variant class:** BRCA1 mutation

NCCN Recommendation category: 1

Population segment (Line of therapy):

- Adenocarcinoma; Metastatic (Subsequent therapy); Useful in certain circumstances

Reference: NCCN Guidelines® - NCCN-Prostate Cancer [Version 1.2022]

rucaparib

Cancer type: Ovarian Cancer **Variant class:** BRCA1 mutation

NCCN Recommendation category: 1

Population segment (Line of therapy):

- Epithelial, Fallopian Tube, Primary Peritoneal; Persistent, Recurrent, Partial response, Complete response (Maintenance therapy); Useful in certain circumstances

Reference: NCCN Guidelines® - NCCN-Ovarian Cancer [Version 3.2021]

niraparib

Cancer type: Ovarian Cancer **Variant class:** BRCA1 mutation

NCCN Recommendation category: 2A

Population segment (Line of therapy):

- Epithelial, Fallopian Tube, Primary Peritoneal; Stage II, Stage III, Stage IV; Partial response, Complete response (Maintenance therapy)

Reference: NCCN Guidelines® - NCCN-Ovarian Cancer [Version 3.2021]

Disclaimer: The data presented here is from a curated knowledgebase of publicly available information, but may not be exhaustive. The data version is 2021.11(003).

BRCA1 mutation (continued)

niraparib

Cancer type: Ovarian Cancer **Variant class:** BRCA1 mutation

NCCN Recommendation category: 2A

Population segment (Line of therapy):
- Epithelial, Less Common Ovarian Cancers, Fallopian Tube, Primary Peritoneal; Recurrent (Recurrence therapy); Preferred intervention

Reference: NCCN Guidelines® - NCCN-Ovarian Cancer [Version 3.2021]

olaparib

Cancer type: Ovarian Cancer **Variant class:** BRCA1 mutation

NCCN Recommendation category: 2A

Population segment (Line of therapy):
- Epithelial, Fallopian Tube, Primary Peritoneal; Stage II, Stage III, Stage IV; Partial response, Complete response (Maintenance therapy)

Reference: NCCN Guidelines® - NCCN-Ovarian Cancer [Version 3.2021]

rucaparib

Cancer type: Ovarian Cancer **Variant class:** BRCA1 mutation

NCCN Recommendation category: 2A

Population segment (Line of therapy):
- Epithelial, Less Common Ovarian Cancers, Fallopian Tube, Primary Peritoneal; Recurrent (Recurrence therapy); Preferred intervention

Reference: NCCN Guidelines® - NCCN-Ovarian Cancer [Version 3.2021]

rucaparib

Cancer type: Castration-Resistant Prostate Cancer **Variant class:** BRCA1 mutation

NCCN Recommendation category: 2A

Population segment (Line of therapy):
- Adenocarcinoma; Metastatic (Subsequent therapy); Useful in certain circumstances

Reference: NCCN Guidelines® - NCCN-Prostate Cancer [Version 1.2022]

Disclaimer: The data presented here is from a curated knowledgebase of publicly available information, but may not be exhaustive. The data version is 2021.11(003).

BRCA1 mutation (continued)

olaparib

Cancer type: Castration-Resistant Prostate Cancer **Variant class:** BRCA1 mutation

NCCN Recommendation category: 2B

Population segment (Line of therapy):
- Adenocarcinoma; Visceral Metastases (Subsequent therapy); Useful in certain circumstances

Reference: NCCN Guidelines® - NCCN-Prostate Cancer [Version 1.2022]

---

rucaparib

Cancer type: Castration-Resistant Prostate Cancer **Variant class:** BRCA1 mutation

NCCN Recommendation category: 2B

Population segment (Line of therapy):
- Adenocarcinoma; Visceral Metastases (Subsequent therapy); Useful in certain circumstances

Reference: NCCN Guidelines® - NCCN-Prostate Cancer [Version 1.2022]

BRCA1 splice site mutation

bevacizumab + olaparib

Cancer type: Ovarian Cancer **Variant class:** BRCA1 mutation

NCCN Recommendation category: 1

Population segment (Line of therapy):
- Epithelial, Fallopian Tube, Primary Peritoneal; Stage II, Stage III, Stage IV; Partial response, Complete response (Maintenance therapy)

Reference: NCCN Guidelines® - NCCN-Ovarian Cancer [Version 3.2021]

---

niraparib

Cancer type: Ovarian Cancer **Variant class:** BRCA1 mutation

NCCN Recommendation category: 1

Population segment (Line of therapy):
- Epithelial, Fallopian Tube, Primary Peritoneal; Persistent, Recurrent, Partial response, Complete response (Maintenance therapy); Useful in certain circumstances

Reference: NCCN Guidelines® - NCCN-Ovarian Cancer [Version 3.2021]

Disclaimer: The data presented here is from a curated knowledgebase of publicly available information, but may not be exhaustive. The data version is 2021.11(003).

BRCA1 splice site mutation (continued)

niraparib

Cancer type: Ovarian Cancer **Variant class:** BRCA1 mutation

NCCN Recommendation category: 1

Population segment (Line of therapy):
- Epithelial, Fallopian Tube, Primary Peritoneal; Stage II, Stage III, Stage IV; Partial response, Complete response (Maintenance therapy)

Reference: NCCN Guidelines® - NCCN-Ovarian Cancer [Version 3.2021]

olaparib

Cancer type: Ovarian Cancer **Variant class:** BRCA1 mutation

NCCN Recommendation category: 1

Population segment (Line of therapy):
- Epithelial, Fallopian Tube, Primary Peritoneal; Persistent, Recurrent, Partial response, Complete response (Maintenance therapy); Useful in certain circumstances

Reference: NCCN Guidelines® - NCCN-Ovarian Cancer [Version 3.2021]

olaparib

Cancer type: Ovarian Cancer **Variant class:** BRCA1 mutation

NCCN Recommendation category: 1

Population segment (Line of therapy):
- Epithelial, Fallopian Tube, Primary Peritoneal; Stage II, Stage III, Stage IV; Partial response, Complete response (Maintenance therapy)

Reference: NCCN Guidelines® - NCCN-Ovarian Cancer [Version 3.2021]

olaparib

Cancer type: Castration-Resistant Prostate Cancer **Variant class:** BRCA1 mutation

NCCN Recommendation category: 1

Population segment (Line of therapy):
- Adenocarcinoma; Metastatic (Subsequent therapy); Useful in certain circumstances

Reference: NCCN Guidelines® - NCCN-Prostate Cancer [Version 1.2022]

Disclaimer: The data presented here is from a curated knowledgebase of publicly available information, but may not be exhaustive. The data version is 2021.11(003).

BRCA1 splice site mutation (continued)

○ rucaparib

Cancer type: Ovarian Cancer **Variant class:** BRCA1 mutation

NCCN Recommendation category: 1

Population segment (Line of therapy):
- Epithelial, Fallopian Tube, Primary Peritoneal; Persistent, Recurrent, Partial response, Complete response (Maintenance therapy); Useful in certain circumstances

Reference: NCCN Guidelines® - NCCN-Ovarian Cancer [Version 3.2021]

○ niraparib

Cancer type: Ovarian Cancer **Variant class:** BRCA1 mutation

NCCN Recommendation category: 2A

Population segment (Line of therapy):
- Epithelial, Fallopian Tube, Primary Peritoneal; Stage II, Stage III, Stage IV; Partial response, Complete response (Maintenance therapy)

Reference: NCCN Guidelines® - NCCN-Ovarian Cancer [Version 3.2021]

○ niraparib

Cancer type: Ovarian Cancer **Variant class:** BRCA1 mutation

NCCN Recommendation category: 2A

Population segment (Line of therapy):
- Epithelial, Less Common Ovarian Cancers, Fallopian Tube, Primary Peritoneal; Recurrent (Recurrence therapy); Preferred intervention

Reference: NCCN Guidelines® - NCCN-Ovarian Cancer [Version 3.2021]

○ olaparib

Cancer type: Ovarian Cancer **Variant class:** BRCA1 mutation

NCCN Recommendation category: 2A

Population segment (Line of therapy):
- Epithelial, Fallopian Tube, Primary Peritoneal; Stage II, Stage III, Stage IV; Partial response, Complete response (Maintenance therapy)

Reference: NCCN Guidelines® - NCCN-Ovarian Cancer [Version 3.2021]

Disclaimer: The data presented here is from a curated knowledgebase of publicly available information, but may not be exhaustive. The data version is 2021.11(003).

BRCA1 splice site mutation (continued)

rucaparib

Cancer type: Ovarian Cancer **Variant class:** BRCA1 mutation

NCCN Recommendation category: 2A

Population segment (Line of therapy):
- Epithelial, Less Common Ovarian Cancers, Fallopian Tube, Primary Peritoneal; Recurrent (Recurrence therapy); Preferred intervention

Reference: NCCN Guidelines® - NCCN-Ovarian Cancer [Version 3.2021]

rucaparib

Cancer type: Castration-Resistant Prostate Cancer **Variant class:** BRCA1 mutation

NCCN Recommendation category: 2A

Population segment (Line of therapy):
- Adenocarcinoma; Metastatic (Subsequent therapy); Useful in certain circumstances

Reference: NCCN Guidelines® - NCCN-Prostate Cancer [Version 1.2022]

olaparib

Cancer type: Castration-Resistant Prostate Cancer **Variant class:** BRCA1 mutation

NCCN Recommendation category: 2B

Population segment (Line of therapy):
- Adenocarcinoma; Visceral Metastases (Subsequent therapy); Useful in certain circumstances

Reference: NCCN Guidelines® - NCCN-Prostate Cancer [Version 1.2022]

rucaparib

Cancer type: Castration-Resistant Prostate Cancer **Variant class:** BRCA1 mutation

NCCN Recommendation category: 2B

Population segment (Line of therapy):
- Adenocarcinoma; Visceral Metastases (Subsequent therapy); Useful in certain circumstances

Reference: NCCN Guidelines® - NCCN-Prostate Cancer [Version 1.2022]

Disclaimer: The data presented here is from a curated knowledgebase of publicly available information, but may not be exhaustive. The data version is 2021.11(003).

BRCA1 truncating mutation

bevacizumab + olaparib

Cancer type: Ovarian Cancer **Variant class:** BRCA1 mutation

NCCN Recommendation category: 1

Population segment (Line of therapy):
- Epithelial, Fallopian Tube, Primary Peritoneal; Stage II, Stage III, Stage IV; Partial response, Complete response (Maintenance therapy)

Reference: NCCN Guidelines® - NCCN-Ovarian Cancer [Version 3.2021]

niraparib

Cancer type: Ovarian Cancer **Variant class:** BRCA1 mutation

NCCN Recommendation category: 1

Population segment (Line of therapy):
- Epithelial, Fallopian Tube, Primary Peritoneal; Persistent, Recurrent, Partial response, Complete response (Maintenance therapy); Useful in certain circumstances

Reference: NCCN Guidelines® - NCCN-Ovarian Cancer [Version 3.2021]

niraparib

Cancer type: Ovarian Cancer **Variant class:** BRCA1 mutation

NCCN Recommendation category: 1

Population segment (Line of therapy):
- Epithelial, Fallopian Tube, Primary Peritoneal; Stage II, Stage III, Stage IV; Partial response, Complete response (Maintenance therapy)

Reference: NCCN Guidelines® - NCCN-Ovarian Cancer [Version 3.2021]

olaparib

Cancer type: Ovarian Cancer **Variant class:** BRCA1 mutation

NCCN Recommendation category: 1

Population segment (Line of therapy):
- Epithelial, Fallopian Tube, Primary Peritoneal; Persistent, Recurrent, Partial response, Complete response (Maintenance therapy); Useful in certain circumstances

Reference: NCCN Guidelines® - NCCN-Ovarian Cancer [Version 3.2021]

Disclaimer: The data presented here is from a curated knowledgebase of publicly available information, but may not be exhaustive. The data version is 2021.11(003).

BRCA1 truncating mutation (continued)

olaparib

Cancer type: Ovarian Cancer **Variant class:** BRCA1 mutation

NCCN Recommendation category: 1

Population segment (Line of therapy):

- Epithelial, Fallopian Tube, Primary Peritoneal; Stage II, Stage III, Stage IV; Partial response, Complete response (Maintenance therapy)

Reference: NCCN Guidelines® - NCCN-Ovarian Cancer [Version 3.2021]

olaparib

Cancer type: Castration-Resistant Prostate Cancer **Variant class:** BRCA1 mutation

NCCN Recommendation category: 1

Population segment (Line of therapy):

- Adenocarcinoma; Metastatic (Subsequent therapy); Useful in certain circumstances

Reference: NCCN Guidelines® - NCCN-Prostate Cancer [Version 1.2022]

rucaparib

Cancer type: Ovarian Cancer **Variant class:** BRCA1 mutation

NCCN Recommendation category: 1

Population segment (Line of therapy):

- Epithelial, Fallopian Tube, Primary Peritoneal; Persistent, Recurrent, Partial response, Complete response (Maintenance therapy); Useful in certain circumstances

Reference: NCCN Guidelines® - NCCN-Ovarian Cancer [Version 3.2021]

niraparib

Cancer type: Ovarian Cancer **Variant class:** BRCA1 mutation

NCCN Recommendation category: 2A

Population segment (Line of therapy):

- Epithelial, Fallopian Tube, Primary Peritoneal; Stage II, Stage III, Stage IV; Partial response, Complete response (Maintenance therapy)

Reference: NCCN Guidelines® - NCCN-Ovarian Cancer [Version 3.2021]

Disclaimer: The data presented here is from a curated knowledgebase of publicly available information, but may not be exhaustive. The data version is 2021.11(003).

BRCA1 truncating mutation (continued)

niraparib

Cancer type: Ovarian Cancer **Variant class:** BRCA1 mutation

NCCN Recommendation category: 2A

Population segment (Line of therapy):
- Epithelial, Less Common Ovarian Cancers, Fallopian Tube, Primary Peritoneal; Recurrent (Recurrence therapy); Preferred intervention

Reference: NCCN Guidelines® - NCCN-Ovarian Cancer [Version 3.2021]

olaparib

Cancer type: Ovarian Cancer **Variant class:** BRCA1 mutation

NCCN Recommendation category: 2A

Population segment (Line of therapy):
- Epithelial, Fallopian Tube, Primary Peritoneal; Stage II, Stage III, Stage IV; Partial response, Complete response (Maintenance therapy)

Reference: NCCN Guidelines® - NCCN-Ovarian Cancer [Version 3.2021]

rucaparib

Cancer type: Ovarian Cancer **Variant class:** BRCA1 mutation

NCCN Recommendation category: 2A

Population segment (Line of therapy):
- Epithelial, Less Common Ovarian Cancers, Fallopian Tube, Primary Peritoneal; Recurrent (Recurrence therapy); Preferred intervention

Reference: NCCN Guidelines® - NCCN-Ovarian Cancer [Version 3.2021]

rucaparib

Cancer type: Castration-Resistant Prostate Cancer **Variant class:** BRCA1 mutation

NCCN Recommendation category: 2A

Population segment (Line of therapy):
- Adenocarcinoma; Metastatic (Subsequent therapy); Useful in certain circumstances

Reference: NCCN Guidelines® - NCCN-Prostate Cancer [Version 1.2022]

Disclaimer: The data presented here is from a curated knowledgebase of publicly available information, but may not be exhaustive. The data version is 2021.11(003).

BRCA1 truncating mutation (continued)

olaparib

Cancer type: Castration-Resistant Prostate Cancer **Variant class:** BRCA1 mutation

NCCN Recommendation category: 2B

Population segment (Line of therapy):
- Adenocarcinoma; Visceral Metastases (Subsequent therapy); Useful in certain circumstances

Reference: NCCN Guidelines® - NCCN-Prostate Cancer [Version 1.2022]

rucaparib

Cancer type: Castration-Resistant Prostate Cancer **Variant class:** BRCA1 mutation

NCCN Recommendation category: 2B

Population segment (Line of therapy):
- Adenocarcinoma; Visceral Metastases (Subsequent therapy); Useful in certain circumstances

Reference: NCCN Guidelines® - NCCN-Prostate Cancer [Version 1.2022]

Disclaimer: The data presented here is from a curated knowledgebase of publicly available information, but may not be exhaustive. The data version is 2021.11(003).

Current EMA Information

● In this cancer type ○ In other cancer type ◐ In this cancer type and other cancer types

EMA information is current as of 2021-10-13. For the most up-to-date information, search www.ema.europa.eu/ema.

BRCA1 mutation

○ olaparib, bevacizumab + olaparib

Cancer type: Castration-Resistant Prostate Cancer, Ovarian Cancer **Label as of:** 2021-10-12 **Variant class:** BRCA1 mutation

Reference:

https://www.ema.europa.eu/en/documents/product-information/lynparza-epar-product-information_en.pdf

○ rucaparib

Cancer type: Ovarian Cancer **Label as of:** 2021-09-20 **Variant class:** BRCA1 mutation

Reference:

https://www.ema.europa.eu/en/documents/product-information/rubraca-epar-product-information_en.pdf

BRCA1 splice site mutation

○ olaparib, bevacizumab + olaparib

Cancer type: Castration-Resistant Prostate Cancer, Ovarian Cancer **Label as of:** 2021-10-12 **Variant class:** BRCA1 mutation

Reference:

https://www.ema.europa.eu/en/documents/product-information/lynparza-epar-product-information_en.pdf

○ rucaparib

Cancer type: Ovarian Cancer **Label as of:** 2021-09-20 **Variant class:** BRCA1 mutation

Reference:

https://www.ema.europa.eu/en/documents/product-information/rubraca-epar-product-information_en.pdf

BRCA1 truncating mutation

○ olaparib, bevacizumab + olaparib

Cancer type: Castration-Resistant Prostate Cancer, Ovarian Cancer **Label as of:** 2021-10-12 **Variant class:** BRCA1 mutation

Reference:

https://www.ema.europa.eu/en/documents/product-information/lynparza-epar-product-information_en.pdf

Disclaimer: The data presented here is from a curated knowledgebase of publicly available information, but may not be exhaustive. The data version is 2021.11(003).

BRCA1 truncating mutation (continued)

rucaparib

Cancer type: Ovarian Cancer **Label as of:** 2021-09-20 **Variant class:** BRCA1 mutation

Reference:

https://www.ema.europa.eu/en/documents/product-information/rubraca-epar-product-information_en.pdf

Current ESMO Information

● In this cancer type ○ In other cancer type ◐ In this cancer type and other cancer types

ESMO information is current as of 2021-10-01. For the most up-to-date information, search www.esmo.org.

BRCA1 mutation

○ olaparib

Cancer type: Castration-Resistant Prostate Cancer **Variant class:** BRCA1 mutation

ESMO Level of Evidence/Grade of Recommendation: I / B

Population segment (Line of therapy):
- Metastatic, Progression (Line of therapy not specified)

Reference: ESMO Clinical Practice Guidelines - ESMO-Cancer of the Prostate [Ann Oncol (2020)]

○ bevacizumab + olaparib

Cancer type: Ovarian Cancer **Variant class:** BRCA mutation

ESMO Level of Evidence/Grade of Recommendation: I / A

Population segment (Line of therapy):
- Epithelial; Relapsed (Maintenance therapy); ESMO-MCBS v1.1 score: 3

Reference: ESMO Clinical Practice Guidelines - ESMO-Newly Diagnosed and Relapsed Epithelial Ovarian Carcinoma [Ann Oncol 2013; 24 (Suppl 6): vi24-vi32. (eUpdate: 19 July 2021, 01 April 2020, 21 September 2016; Corrigendum: 03 October 2018)]

○ niraparib

Cancer type: Ovarian Cancer **Variant class:** BRCA mutation

ESMO Level of Evidence/Grade of Recommendation: I / A

Population segment (Line of therapy):
- Epithelial; Relapsed (Maintenance therapy); ESMO-MCBS v1.1 score: 3

Reference: ESMO Clinical Practice Guidelines - ESMO-Newly Diagnosed and Relapsed Epithelial Ovarian Carcinoma [Ann Oncol 2013; 24 (Suppl 6): vi24-vi32. (eUpdate: 19 July 2021, 01 April 2020, 21 September 2016; Corrigendum: 03 October 2018)]

○ olaparib

Cancer type: Ovarian Cancer **Variant class:** BRCA mutation

ESMO Level of Evidence/Grade of Recommendation: I / A

Population segment (Line of therapy):
- Epithelial; Relapsed (Maintenance therapy); ESMO-MCBS v1.1 score: 4

Reference: ESMO Clinical Practice Guidelines - ESMO-Newly Diagnosed and Relapsed Epithelial Ovarian Carcinoma [Ann Oncol 2013; 24 (Suppl 6): vi24-vi32. (eUpdate: 19 July 2021, 01 April 2020, 21 September 2016; Corrigendum: 03 October 2018)]

Disclaimer: The data presented here is from a curated knowledgebase of publicly available information, but may not be exhaustive. The data version is 2021.11(003).

BRCA1 mutation (continued)

rucaparib

Cancer type: Ovarian Cancer **Variant class:** BRCA mutation

ESMO Level of Evidence/Grade of Recommendation: III / A

Population segment (Line of therapy):
- Epithelial; Recurrent (Line of therapy not specified)

Reference: ESMO Clinical Practice Guidelines - ESMO-Newly Diagnosed and Relapsed Epithelial Ovarian Carcinoma [Ann Oncol 2013; 24 (Suppl 6): vi24-vi32. (eUpdate: 19 July 2021, 01 April 2020, 21 September 2016; Corrigendum: 03 October 2018)]

BRCA1 splice site mutation

olaparib

Cancer type: Castration-Resistant Prostate Cancer **Variant class:** BRCA1 mutation

ESMO Level of Evidence/Grade of Recommendation: I / B

Population segment (Line of therapy):
- Metastatic, Progression (Line of therapy not specified)

Reference: ESMO Clinical Practice Guidelines - ESMO-Cancer of the Prostate [Ann Oncol (2020)]

bevacizumab + olaparib

Cancer type: Ovarian Cancer **Variant class:** BRCA mutation

ESMO Level of Evidence/Grade of Recommendation: I / A

Population segment (Line of therapy):
- Epithelial; Relapsed (Maintenance therapy); ESMO-MCBS v1.1 score: 3

Reference: ESMO Clinical Practice Guidelines - ESMO-Newly Diagnosed and Relapsed Epithelial Ovarian Carcinoma [Ann Oncol 2013; 24 (Suppl 6): vi24-vi32. (eUpdate: 19 July 2021, 01 April 2020, 21 September 2016; Corrigendum: 03 October 2018)]

niraparib

Cancer type: Ovarian Cancer **Variant class:** BRCA mutation

ESMO Level of Evidence/Grade of Recommendation: I / A

Population segment (Line of therapy):
- Epithelial; Relapsed (Maintenance therapy); ESMO-MCBS v1.1 score: 3

Reference: ESMO Clinical Practice Guidelines - ESMO-Newly Diagnosed and Relapsed Epithelial Ovarian Carcinoma [Ann Oncol 2013; 24 (Suppl 6): vi24-vi32. (eUpdate: 19 July 2021, 01 April 2020, 21 September 2016; Corrigendum: 03 October 2018)]

BRCA1 splice site mutation (continued)

olaparib

Cancer type: Ovarian Cancer **Variant class:** BRCA mutation

ESMO Level of Evidence/Grade of Recommendation: I / A

Population segment (Line of therapy):
- Epithelial; Relapsed (Maintenance therapy); ESMO-MCBS v1.1 score: 4

Reference: ESMO Clinical Practice Guidelines - ESMO-Newly Diagnosed and Relapsed Epithelial Ovarian Carcinoma [Ann Oncol 2013; 24 (Suppl 6): vi24-vi32. (eUpdate: 19 July 2021, 01 April 2020, 21 September 2016; Corrigendum: 03 October 2018)]

rucaparib

Cancer type: Ovarian Cancer **Variant class:** BRCA mutation

ESMO Level of Evidence/Grade of Recommendation: III / A

Population segment (Line of therapy):
- Epithelial; Recurrent (Line of therapy not specified)

Reference: ESMO Clinical Practice Guidelines - ESMO-Newly Diagnosed and Relapsed Epithelial Ovarian Carcinoma [Ann Oncol 2013; 24 (Suppl 6): vi24-vi32. (eUpdate: 19 July 2021, 01 April 2020, 21 September 2016; Corrigendum: 03 October 2018)]

BRCA1 truncating mutation

olaparib

Cancer type: Castration-Resistant Prostate Cancer **Variant class:** BRCA1 mutation

ESMO Level of Evidence/Grade of Recommendation: I / B

Population segment (Line of therapy):
- Metastatic, Progression (Line of therapy not specified)

Reference: ESMO Clinical Practice Guidelines - ESMO-Cancer of the Prostate [Ann Oncol (2020)]

bevacizumab + olaparib

Cancer type: Ovarian Cancer **Variant class:** BRCA mutation

ESMO Level of Evidence/Grade of Recommendation: I / A

Population segment (Line of therapy):
- Epithelial; Relapsed (Maintenance therapy); ESMO-MCBS v1.1 score: 3

Reference: ESMO Clinical Practice Guidelines - ESMO-Newly Diagnosed and Relapsed Epithelial Ovarian Carcinoma [Ann Oncol 2013; 24 (Suppl 6): vi24-vi32. (eUpdate: 19 July 2021, 01 April 2020, 21 September 2016; Corrigendum: 03 October 2018)]

Disclaimer: The data presented here is from a curated knowledgebase of publicly available information, but may not be exhaustive. The data version is 2021.11(003).

BRCA1 truncating mutation (continued)

○ niraparib

Cancer type: Ovarian Cancer **Variant class:** BRCA mutation

ESMO Level of Evidence/Grade of Recommendation: I / A

Population segment (Line of therapy):
- Epithelial; Relapsed (Maintenance therapy); ESMO-MCBS v1.1 score: 3

Reference: ESMO Clinical Practice Guidelines - ESMO-Newly Diagnosed and Relapsed Epithelial Ovarian Carcinoma [Ann Oncol 2013; 24 (Suppl 6): vi24-vi32. (eUpdate: 19 July 2021, 01 April 2020, 21 September 2016; Corrigendum: 03 October 2018)]

○ olaparib

Cancer type: Ovarian Cancer **Variant class:** BRCA mutation

ESMO Level of Evidence/Grade of Recommendation: I / A

Population segment (Line of therapy):
- Epithelial; Relapsed (Maintenance therapy); ESMO-MCBS v1.1 score: 4

Reference: ESMO Clinical Practice Guidelines - ESMO-Newly Diagnosed and Relapsed Epithelial Ovarian Carcinoma [Ann Oncol 2013; 24 (Suppl 6): vi24-vi32. (eUpdate: 19 July 2021, 01 April 2020, 21 September 2016; Corrigendum: 03 October 2018)]

○ rucaparib

Cancer type: Ovarian Cancer **Variant class:** BRCA mutation

ESMO Level of Evidence/Grade of Recommendation: III / A

Population segment (Line of therapy):
- Epithelial; Recurrent (Line of therapy not specified)

Reference: ESMO Clinical Practice Guidelines - ESMO-Newly Diagnosed and Relapsed Epithelial Ovarian Carcinoma [Ann Oncol 2013; 24 (Suppl 6): vi24-vi32. (eUpdate: 19 July 2021, 01 April 2020, 21 September 2016; Corrigendum: 03 October 2018)]

Disclaimer: The data presented here is from a curated knowledgebase of publicly available information, but may not be exhaustive. The data version is 2021.11(003).

Alerts Informed By Public Data Sources

Current NCCN Information

Contraindicated   Not recommended   Resistance   Breakthrough   Fast Track

NCCN information is current as of 2021-10-01. For the most up-to-date information, search www.nccn.org.
For NCCN International Adaptations & Translations, search www.nccn.org/global/international_adaptations.aspx.

BRCA1 mutation

bevacizumab

Cancer type: Ovarian Cancer **Variant class:** BRCA1 mutation

Summary:

NCCN Guidelines® include the following supporting statement(s):

- "bevacizumab monotherapy is no longer recommended for patients with BRCA1/2 mutations"

Reference: NCCN Guidelines® - NCCN-Ovarian Cancer [Version 3.2021]

BRCA1 splice site mutation

bevacizumab

Cancer type: Ovarian Cancer **Variant class:** BRCA1 mutation

Summary:

NCCN Guidelines® include the following supporting statement(s):

- "bevacizumab monotherapy is no longer recommended for patients with BRCA1/2 mutations"

Reference: NCCN Guidelines® - NCCN-Ovarian Cancer [Version 3.2021]

BRCA1 truncating mutation

bevacizumab

Cancer type: Ovarian Cancer **Variant class:** BRCA1 mutation

Summary:

NCCN Guidelines® include the following supporting statement(s):

- "bevacizumab monotherapy is no longer recommended for patients with BRCA1/2 mutations"

Reference: NCCN Guidelines® - NCCN-Ovarian Cancer [Version 3.2021]

Disclaimer: The data presented here is from a curated knowledgebase of publicly available information, but may not be exhaustive. The data version is 2021.11(003).

Current Clinical Trials Information

Clinical Trials information is current as of 2021-10-01. For the most up-to-date information regarding a particular trial, search www.clinicaltrials.gov by NCT ID or search local clinical trials authority website by local identifier listed in 'Other identifiers'.

BRCA1 mutation + BRCA1 splice site mutation + BRCA1 truncating mutation + BRCA1 deletion

NCT04126070
A Phase II Multicohort Study of Nivolumab in Combination With Docetaxel and Androgen Deprivation Therapy in Metastatic Hormone Sensitive Prostate Cancer Patients With DNA Damage Repair Defects or Inflamed Tumors

Cancer type: Prostate Cancer

Variant classes: DNA repair deletion & DNA repair mutation

Other identifiers: 19-384, NCI-2020-02149

Population segments: First line, Stage IV

Phase: II

Therapies: nivolumab, chemotherapy, hormone therapy

Location: United States

US State: MA

Contact: Dr. Xiao X. Wei [617-632-4524; xiaox_wei@dfci.harvard.edu]

BRCA1 mutation

NCT02029001
A Two-period, Multicenter, Randomized, Open-label, Phase II Study Evaluating the Clinical Benefit of a Maintenance Treatment Targeting Tumor Molecular Alterations in Patients with Progressive Locally-advanced or Metastatic Solid Tumors.

Cancer type: Unspecified Solid Tumor

Variant class: BRCA1 mutation

Other identifiers: ET12-081, EudraCT Number: 2012-004510-34, MOST, ProfiLER

Population segments: Maintenance/Consolidation, Second line, Stage III, Stage IV, Third line

Exclusion criteria variant classes: ATM mutation (Gastric Cancer), BRCA1 germline mutation (Gastric Cancer), BRCA1 mutation (Gastric Cancer), BRCA2 germline mutation (Gastric Cancer), BRCA2 mutation (Gastric Cancer), HRR mutation (Gastric Cancer), PALB2 mutation (Gastric Cancer), RAD51 mutation (Gastric Cancer)

Phase: II

Therapy: olaparib

Location: France

NCT03967938
Efficacy of Olaparib in Advanced Cancers Occurring in Patients With Germline Mutations or Somatic Tumor Mutations in Homologous Recombination Genes

Cancer type: Unspecified Solid Tumor

Variant class: BRCA1 mutation

Other identifiers: 1-2018 BSMO, 1-2018BSMO, EudraCT Number: 2018-002966-37, Precision 2 - Olaparib

Population segments: Line of therapy N/A, Stage III, Stage IV

Exclusion criteria variant classes: BRCA1 germline mutation (Breast Cancer, Pancreatic Cancer, Prostate Cancer), BRCA2 germline mutation (Breast Cancer, Pancreatic Cancer, Prostate Cancer)

Phase: II

Therapy: olaparib

Location: Belgium

Disclaimer: The data presented here is from a curated knowledgebase of publicly available information, but may not be exhaustive. The data version is 2021.11(003).

BRCA1 mutation (continued)

NCT02693535
Targeted Agent and Profiling Utilization Registry (TAPUR) Study.

Cancer type: Unspecified Solid Tumor

Variant class: BRCA1 mutation

Other identifiers: 20170529, NCI-2017-00510, Pro00014171, TAPUR

Population segments: Aggressive, BRAF, Diffuse large B-cell lymphoma (DLBCL), Extranodal marginal zone B-cell lymphoma (MALT), FGFR, First line, Follicular lymphoma (FL), Fourth line or greater, Indolent, Lymphoblastic lymphoma (LBL), Mantle cell lymphoma (MCL), Other subtype, Second line, Small lymphocytic lymphoma (SLL), Stage III, Stage IV, Third line, Waldenstrom's macroglobulinemia (WM)

Phase: II

Therapies: olaparib, talazoparib, atezolizumab + talazoparib

Location: United States

US States: AL, AZ, CA, FL, GA, HI, IL, IN, ME, MI, NC, ND, NE, NH, OH, OR, PA, SD, TX, UT, VA, WA

Contact: Pam Mangat [pam.mangat@asco.org]

NCT04171700
A Phase II Multicenter, Open-label Study of Rucaparib as Treatment for Solid Tumors Associated With Deleterious Mutations in Homologous Recombination Repair Genes

Cancer type: Unspecified Solid Tumor

Variant class: BRCA1 mutation

Other identifiers: 19-756, 20619, AAAS6974, CO-338-100, LODESTAR, NCI-2019-08226

Population segments: BRCA, Second line, Stage III, Stage IV

Exclusion criteria variant classes: BRCA1 germline mutation (Breast Cancer, Ovarian Cancer, Prostate Cancer), BRCA2 germline mutation (Breast Cancer, Ovarian Cancer, Prostate Cancer)

Phase: II

Therapy: rucaparib

Location: United States

US States: CA, FL, IA, IL, MA, MN, NY, OH, OK, PA, TN, WA

Contact: Clovis Oncology For North America [844-258-7662; medinfo@clovisoncology.com]

NCT02286687
Phase II Study of the PARP Inhibitor Talazoparib in Advanced Cancer Patients With Somatic Alterations in BRCA1/2, Mutations/Deletions in Other BRCA Pathway Genes and Germline Mutation in BRCA1/2 (Not Breast or Ovarian Cancer)

Cancer type: Unspecified Cancer

Variant class: BRCA1 mutation

Other identifiers: 2013-0961, NCI-2014-02494

Population segments: Fourth line or greater, Stage III, Stage IV

Exclusion criteria variant classes: BRCA1 germline mutation (Breast Cancer, Ovarian Cancer), BRCA2 germline mutation (Breast Cancer, Ovarian Cancer)

Phase: II

Therapy: talazoparib

Location: United States

US State: TX

Contact: Dr. Sarina Piha-Paul [713-563-1930; spihapau@mdanderson.org]

BRCA1 mutation (continued)

NCT04550494
A Pharmacodynamics-Driven Trial of Talazoparib, an Oral PARP Inhibitor, in Patients With Advanced Solid Tumors and Aberrations in Genes Involved in DNA Damage Response

Cancer type: Unspecified Solid Tumor

Variant class: BRCA1 mutation

Other identifiers: 000246, 000264-C, 10000264, 10371, NCI-2020-06906

Population segments: BRCA, Fourth line or greater, HER2 positive, Hormone refractory, Second line, Stage II, Stage III, Stage IV, Third line

Phase: II

Therapy: talazoparib

Location: United States

US State: MD

Contact: Multiple contacts: See www.clinicaltrials.gov for complete list of contacts.

NCT03842228
A Phase Ib Biomarker-Driven Combination Trial of Copanlisib, Olaparib, and Durvalumab (MEDI4736) in Patients With Advanced Solid Tumors

Cancer type: Unspecified Solid Tumor

Variant class: BRCA1 mutation

Other identifiers: 10217, 19-628, NCI10217, NCI-2019-00601

Population segments: Line of therapy N/A, Stage III, Stage IV

Phase: I

Therapies: copanlisib, olaparib, durvalumab

Location: United States

US States: MA, TX

Contact: Multiple contacts: See www.clinicaltrials.gov for complete list of contacts.

NCT04586335
Open Label, Phase Ib Study to Evaluate the Safety, Tolerability, Pharmacokinetics and Clinical Activity of CYH33, an Oral a-specific PI3K Inhibitor in Combination With Olaparib, an Oral PARP Inhibitor in Patients With Advanced Solid Tumors.

Cancer type: Breast Cancer, Endometrial Carcinoma, Ovarian Cancer, Prostate Cancer, Unspecified Solid Tumor

Variant class: BRCA1 mutation

Other identifiers: CTR20202339, CYH33-G102

Population segments: BRCA, Second line, Stage III, Stage IV

Phase: I

Therapies: HH-CYH33, olaparib

Locations: Australia, United States

US State: TX

Contact: Dr. Jason Sudia [908-380-1329; jason.sudia@haihepharma.com]

NCT03415659
A Phase I, Open-label, Single-center, Single/Multiple-dose, Dose-escalation/ Dose-expansion Clinical Study on Tolerance and Pharmacokinetics of HWH340 Tablet in Patients With Advanced Solid Tumors

Cancer type: Unspecified Solid Tumor

Variant class: BRCA1 mutation

Other identifiers: CTR20171649, HWH340-RFPA 20170821, RFPA 20170821

Population segments: BRCA, Fourth line or greater, HER2 negative, Second line, Stage III, Stage IV, Third line

Phase: I

Therapy: HWH-340

Location: China

Disclaimer: The data presented here is from a curated knowledgebase of publicly available information, but may not be exhaustive. The data version is 2021.11(003).

BRCA1 mutation (continued)

NCT04197713
Phase I Sequential Trial of Agents Against DNA Repair (STAR)

Cancer type: Unspecified Solid Tumor

Variant class: BRCA1 mutation

Other identifiers: (STAR), 10329, 2020-0347, NCI-2019-08262, STAR Study

Population segments: Second line, Stage III, Stage IV

Phase: I

Therapies: olaparib, adavosertib

Location: United States

US State: TX

Contact: Site Public Contact [877-632-6789; askmdanderson@mdanderson.org]

NCT03297606
Canadian Profiling and Targeted Agent Utilization Trial (CAPTUR): A Phase II Basket Trial

Cancer type: Unspecified Solid Tumor

Variant class: BRCA1 aberration

Other identifiers: CA209-9DL, CAPTUR, ESR-17-12831, ML39800, PM1, WI233446

Population segments: Aggressive, Diffuse large B-cell lymphoma (DLBCL), Extranodal marginal zone B-cell lymphoma (MALT), First line, Follicular lymphoma (FL), Indolent, Lymphoblastic lymphoma (LBL), Mantle cell lymphoma (MCL), Other subtype, Second line, Stage III, Stage IV, Waldenstrom's macroglobulinemia (WM)

Phase: II

Therapy: olaparib

Location: Canada

NCT04497116
Phase I/IIa Study of the Safety, Pharmacokinetics, Pharmacodynamics and Preliminary Clinical Activity of RP-3500 Alone or in Combination With Talazoparib in Advanced Solid Tumors With ATR Inhibitor Sensitizing Mutations (TRESR Study)

Cancer type: Unspecified Solid Tumor

Variant class: BRCA1 aberration

Other identifiers: 20-521, EudraCT Number: 2020-000301-87, IRAS ID: 286609, NCI-2020-06869, RP-3500-01, TRESR, TRESR Study

Population segments: Second line, Stage III, Stage IV

Phase: I/II

Therapies: RP-3500, talazoparib

Locations: Canada, Denmark, United Kingdom, United States

US States: MA, NC, NY, RI, TN, TX

Contact: Dr. Peter Manley [857-322-5553; pmanley@reparerx.com]

Disclaimer: The data presented here is from a curated knowledgebase of publicly available information, but may not be exhaustive. The data version is 2021.11(003).

BRCA1 mutation (continued)

NCT04123366
A Phase II Study of Olaparib in Combination With Pembrolizumab in Participants With Previously Treated, Homologous Recombination Repair Mutation (HRRm) and/or Homologous Recombination Deficiency (HRD)-Positive Advanced Cancer

Cancer type: Unspecified Solid Tumor

Variant class: HRR mutation

Other identifiers: 7339-007, EudraCT Number: 2019-001745-40, jRCT2011200025, KEYLYNK-007, MK-7339-007, NCI-2019-08758, PER-029-19

Population segments: BRCA, Second line, Stage III, Stage IV

Exclusion criteria variant classes: BRCA germline mutation (Breast Cancer, Ovarian Cancer), BRCA mutation (Breast Cancer, Ovarian Cancer)

Phase: II

Therapies: olaparib, pembrolizumab

Locations: Argentina, Australia, Canada, Colombia, France, Germany, Guatemala, Israel, Italy, Japan, Latvia, Mexico, Peru, Poland, Puerto Rico, Republic of Korea, Romania, South Africa, Spain, Sweden, Turkey, Ukraine, United States

US States: AL, AZ, CA, CO, FL, GA, KY, NJ, NY, OH, TX, UT, VA

Contact: Toll Free Number [888-577-8839; Trialsites@merck.com]

NCT03767075
Basket of Baskets: A Modular, Open-label, Phase II, Multicentre Study To Evaluate Targeted Agents in Molecularly Selected Populations With Advanced Solid Tumours

Cancer type: Unspecified Solid Tumor

Variant class: DNA repair mutation

Other identifiers: (Basket of Baskets) (BoB), EudraCT number: 2017-005108-89, MO39164, NL65937.031.18, VHIO17002

Population segments: Second line, Stage III, Stage IV

Phase: II

Therapy: atezolizumab

Locations: France, Germany, Netherlands, Spain, Sweden, United Kingdom

---

NCT04905914
A Phase I/IIa, Open-Label, Safety, Pharmacokinetic, And Preliminary Efficacy Study Of Oral ATRN-119 In Patients With Advanced Solid Tumors

Cancer type: Unspecified Solid Tumor

Variant class: DNA repair mutation

Other identifier: AR-276-01

Population segments: Line of therapy N/A, Stage III, Stage IV

Phase: I/II

Therapy: ATRN-119

Location: United States

US State: MA

Contact: Robert Hasson [619-540-6253; rhasson@pacificlinkconsulting.com]

Disclaimer: The data presented here is from a curated knowledgebase of publicly available information, but may not be exhaustive. The data version is 2021.11(003).

BRCA1 mutation (continued)

NCT04901702
A Randomized Phase I/II Study of Talazoparib or Temozolomide in Combination With Onivyde in Children With Recurrent Solid Malignancies and Ewing Sarcoma

Cancer type: Unspecified Solid Tumor

Variant class: HRR pathway

Other identifiers: EudraCT Number: 2021-003570-31, ONITT

Population segments: Second line, Stage IV

Exclusion criteria variant classes: HRR germline pathway (Central Nervous System Neoplasm, Ewing Sarcoma), HRR pathway (Central Nervous System Neoplasm, Ewing Sarcoma)

Phase: I/II

Therapies: talazoparib, chemotherapy

Locations: Canada, United States

US State: TN

Contact: Dr. Sara Federico [866-278-5833; referralinfo@stjude.org]

NCT03155620
NCI-COG Pediatric MATCH (Molecular Analysis for Therapy Choice) Screening Protocol

Cancer type: Unspecified Solid Tumor

Variant class: DNA repair pathway

Other identifiers: 17-729, 2017-0625, 20170841, APEC1621SC, COGAPEC1621, COGAPEC1621SC, N 55017 SC, NCI-2017-01251, NCI-COG Pediatric MATCH, Pediatric MATCH

Population segments: ALK, Aggressive, BRAF, FGFR, HRAS, Indolent, KRAS, NRAS, Pediatric or Adolescent, Second line, Stage III, Stage IV

Phase: II

Therapy: olaparib

Locations: Puerto Rico, United States

US States: AK, AL, AR, AZ, CA, CO, CT, DC, DE, FL, GA, HI, IA, ID, IL, IN, KY, LA, MA, MD, ME, MI, MN, MO, MS, NC, NE, NH, NJ, NV, NY, OH, OK, OR, PA, RI, SC, SD, TN, TX, UT, VA, VT, WA, WI

Contact: Multiple contacts: See www.clinicaltrials.gov for complete list of contacts.

NCT03233204
NCI-COG Pediatric MATCH (Molecular Analysis for Therapy Choice)- Phase 2 Subprotocol of Olaparib in Patients With Tumors Harboring Defects in DNA Damage Repair Genes

Cancer type: Unspecified Solid Tumor

Variant class: DNA repair pathway

Other identifiers: 20170841, APEC1621H, NCI-2017-00766

Population segments: Aggressive, Indolent, Pediatric or Adolescent, Second line, Stage III, Stage IV

Phase: II

Therapy: olaparib

Locations: Puerto Rico, United States

US States: AK, AL, AR, AZ, CA, CO, DC, DE, FL, GA, IA, ID, IL, IN, KY, LA, MD, ME, MI, MN, MO, MS, NC, ND, NE, NJ, NY, OH, OK, OR, PA, SC, SD, TN, TX, UT, VA, VT, WA, WI

Contact: Multiple contacts: See www.clinicaltrials.gov for complete list of contacts.

BRCA1 mutation (continued)

NCT04267939

An Open-label Phase Ib Study to Determine the Maximum Tolerated and/or Recommended Phase II Dose of the ATR Inhibitor BAY 1895344 in Combination With PARP Inhibitor Niraparib, in Patients With Recurrent Advanced Solid Tumors and Ovarian Cancer

Cancer type: Ovarian Cancer, Unspecified Solid Tumor

Variant class: DNA repair pathway

Other identifiers: 18595, 19-810, 2019-0471, 20-222, EudraCT Number: 2018-003930-34, NCI-2020-01405

Population segments: Maintenance/Consolidation, Second line, Stage III, Stage IV

Exclusion criteria variant class: DNA repair pathway (Prostate Cancer)

Phase: I

Therapies: BAY-1895344, niraparib

Location: United States

US States: MA, NY, TX

Contact: Bayer Clinical Trials Contact [888-842-2937; clinical-trials-contact@bayer.com]

NCT04693468

Modular Phase IB Hypothesis-Testing, Biomarker-Driven, Talazoparib Combination Trial (TalaCom)

Cancer type: Unspecified Solid Tumor

Variant class: DNA repair pathway

Other identifiers: 2020-0436, NCI-2020-06041, TalaCom

Population segments: Hormone refractory, Second line, Stage III, Stage IV

Phase: I

Therapies: talazoparib, palbociclib, axitinib, crizotinib

Location: United States

US State: TX

Contact: Timothy A. Yap [713-563-1784; tyap@mdanderson.org]

NCT02975934

TRITON3: A Multicenter, Randomized, Open Label Phase III Study of Rucaparib Versus Physician's Choice of Therapy for Patients With Metastatic Castration Resistant Prostate Cancer Associated With Homologous Recombination Deficiency

Cancer type: Castration-Resistant Prostate Cancer

Variant class: BRCA1 mutation

Other identifiers: 16238, 1723GCCC, 18-146, 18-300, 20171185, 2018IS040, CO-338-063, CSET 2620, EudraCT Number: 2016-003163-20, GU 146, IGUP17020, IRAS ID: 222298, NCI-2017-01110, S16-02260, STAR Trial, TRITON 3_CO-338-063, TRITON3

Population segments: BRCA, First line, Hormone refractory, Stage IV

Phase: III

Therapies: rucaparib, hormone therapy

Locations: Australia, Belgium, Canada, Denmark, France, Germany, Ireland, Israel, Italy, Spain, United Kingdom, United States

US States: AZ, CA, CO, CT, DE, FL, HI, MD, MN, NC, NY, OH, OR, TN, TX, VA, WA

Contact: Clovis Oncology For North America [844-258-7662; medinfo@clovisoncology.com]

Disclaimer: The data presented here is from a curated knowledgebase of publicly available information, but may not be exhaustive. The data version is 2021.11(003).

BRCA1 mutation (continued)

NCT03810105
A Phase II Study of Olaparib and Durvalumab in Men With Castration Sensitive Biochemically Recurrent Non-Metastatic Prostate Cancer Harboring Mutations in DNA Damage Repair

Cancer type: Prostate Cancer

Variant class: BRCA1 mutation

Other identifiers: 18-480, NCI-2019-01627

Population segments: Adjuvant, Stage I, Stage II, Stage III

Phase: II

Therapies: durvalumab, olaparib

Location: United States

US States: CA, IL, MI, NJ, NY

Contact: Dr. Karen Autio [646-422-4632; autiok@mskcc.org]

NCT04336943
Durvalumab (MEDI4736) and Olaparib (AZD2281) for Treatment of Biochemically Recurrent Prostate Cancer in Men Predicted to Have a High Neoantigen Load: A Pilot Study

Cancer type: Prostate Adenocarcinoma

Variant class: BRCA1 mutation

Other identifiers: 10509, NCI-2020-01860, RG1007001

Population segments: Line of therapy N/A, Stage I, Stage II, Stage III

Phase: II

Therapies: durvalumab, olaparib

Location: United States

US State: WA

Contact: Michael Schweizer [206-606-6252; schweize@uw.edu]

NCT04053322
An International Multicenter Phase II Trial of Durvalumab (MEDI4736) Plus OLAparib Plus Fulvestrant in Metastatic or Locally Advanced ER-positive, HER2-negative Breast Cancer Patients Selected Using Criteria That Predict Sensitivity to Olaparib

Cancer type: Breast Cancer

Variant class: BRCA1 mutation

Other inclusion criteria: ERBB2 negative, ER positive

Other identifiers: CADUSEIME08, DOLAF, EudraCT Number: 2018-003832-57, UC-0140/1812

Population segments: BRCA, Estrogen receptor positive, FGFR, HER2 negative, Progesterone receptor positive, Second line, Stage III, Stage IV

Phase: II

Therapies: durvalumab, olaparib, hormone therapy

Location: France

BRCA1 mutation (continued)

NCT03012321
BRCAAway: A Randomized Phase II Trial of Abiraterone, Olaparib, or Abiraterone + Olaparib in Patients With Metastatic Castration-Resistant Prostate Cancer With DNA Repair Defects

Cancer type: Castration-Resistant Prostate Cancer

Variant class: BRCA1 mutation

Other identifiers: 19137, BRCAAway, NCI-2016-01834, NU 16U05, NU_16U05, PCCTC #: c16-168, PCCTC c16-168, STU00203960

Population segments: First line, Hormone refractory, Stage IV

Phase: II

Therapies: hormone therapy, olaparib, steroid

Location: United States

US States: CA, FL, IL, IN, MD, MI, MN, MO, NC, NY, UT, VA

Contact: Study Coordinator [312-695-1301; cancertrials@northwestern.edu]

No NCT ID - see other identifier(s)
Phase II Study To Evaluate The Efficacy And Safety Of Niraparib In Recurrent Or Persistent Rare Gynecologic Malignancies With Homologous Recombination Deficiency (JGOG2052)

Cancer type: Cervical Cancer, Endometrial Carcinoma, Ovarian Cancer, Uterine Leiomyosarcoma

Variant class: BRCA1 mutation

Other identifiers: (JGOG2052), jRCT2031210264

Population segments: BRCA, Second line, Stage III, Stage IV

Phase: II

Therapy: niraparib

Location: Japan

NCT03553004
Niraparib in Metastatic Pancreatic Cancer After Previous Chemotherapy (NIRA-PANC): A Phase II Trial

Cancer type: Pancreatic Cancer

Variant class: BRCA1 mutation

Other identifiers: IIT-2017-NIRA-PANC, NIRA-PANC, STUDY00141862

Population segments: BRCA, Second line, Stage IV, Third line

Phase: II

Therapy: niraparib

Location: United States

US States: KS, MO

Contact: Clinical Trials Nurse Navigator [913-588-3671; navigation@kumc.edu]

NCT03601923
Phase II Proof-of-Concept Trial Testing the PARP Inhibitor Niraparib in Patients With Pancreatic Cancer Harboring Deficiencies in Homologous Recombination DNA Repair

Cancer type: Pancreatic Cancer

Variant class: BRCA1 mutation

Other identifiers: 18-207, NCI-2018-02395

Population segments: Second line, Stage IV

Phase: II

Therapy: niraparib

Location: United States

US State: MA

Contact: Dr. James Clreary [617-632-5960; JCLEARY@PARTNERS.ORG]

Disclaimer: The data presented here is from a curated knowledgebase of publicly available information, but may not be exhaustive. The data version is 2021.11(003).

BRCA1 mutation (continued)

NCT03840967
A Phase II Study Evaluating Safety and Efficacy of Niraparib in Patients With Previously Treated Homologous Recombination (HR) Defective or Loss of Heterozygosity (LOH) High Metastatic Esophageal/Gastroesophageal Junction/ Proximal Gastric Adenocarcinoma

Cancer type: Esophageal Cancer, Gastric Cancer, Gastroesophageal Junction Adenocarcinoma

Variant class: BRCA1 mutation

Other identifiers: 20166, 71618, HCRN ESO17-325, NCI-2019-07395

Population segments: Second line, Stage III, Stage IV

Phase: II

Therapy: niraparib

Location: United States

US States: FL, IN, MI, NY

Contact: Dr. Shadia Jalal [317-274-3658; sjalal@iu.edu]

NCT03925350
A Phase II Study of Niraparib in Patients With Advanced Melanoma With Genetic Homologous Recombination (HR) Mutation / Alteration

Cancer type: Melanoma

Variant class: BRCA1 mutation

Other identifier: CPMC17-MEL01

Population segments: Second line, Stage III, Stage IV

Phase: II

Therapy: niraparib

Location: United States

US State: CA

Contact: Laurel Brechtel [415-600-1654; BrechtLA@sutterhealth.org]

NCT04493060
Phase II Study of Niraparib and TSR-042 in Patients With Germline or Somatic BRCA1/2 and PALB2-Related Pancreatic Cancer

Cancer type: Pancreatic Cancer

Variant class: BRCA1 mutation

Other identifiers: MC1841, NCI-2020-05352

Population segments: BRCA, Second line, Stage IV, Third line

Phase: II

Therapies: niraparib, dostarlimab

Location: United States

US States: AZ, FL, MN

Contact: Multiple contacts: See www.clinicaltrials.gov for complete list of contacts.

NCT04187833
Phase II Trial of Nivolumab in Combination with Talazoparib in Patients with Unresectable or Metastatic Melanoma and Mutations in BRCA or BRCA-ness Genes.

Cancer type: Melanoma

Variant class: BRCA1 mutation

Other identifiers: CASE2619, NCI-2020-00416

Population segments: Second line, Stage III, Stage IV

Phase: II

Therapies: nivolumab, talazoparib

Location: United States

US State: OH

Contact: Dr. Pauline Funchain [866-223-8100; TaussigResearch@ccf.org]

Disclaimer: The data presented here is from a curated knowledgebase of publicly available information, but may not be exhaustive. The data version is 2021.11(003).

BRCA1 mutation (continued)

NCT03344965
A Phase II Study of Olaparib Monotherapy in Metastatic Breast Cancer Patients With Germline or Somatic Mutations in DNA Repair Genes (Olaparib Expanded)

Cancer type: Breast Cancer

Variant class: BRCA1 mutation

Other identifiers: 17-428, 18-414, NCI-2018-00778, TBCRC 048, TBCRC048

Population segments: BRCA, Estrogen receptor positive, HER2 negative, HER2 positive, Progesterone receptor positive, Second line, Stage IV

Exclusion criteria variant classes: BRCA1 germline mutation (Breast Cancer), BRCA2 germline mutation (Breast Cancer)

Phase: II

Therapy: olaparib

Location: United States

US States: AL, CA, IL, MA, MD, NC, NJ, NY, PA, WA

Contact: Dr. Nadine Tung [617-667-1962; ntung@bidmc.harvard.edu]

NCT03375307
A Phase II Study of Olaparib (AZD2281) in Patients With Metastatic/Advanced Urothelial Carcinoma With DNA-Repair Defects

Cancer type: Bladder Urothelial Carcinoma

Variant class: BRCA1 mutation

Other identifiers: 10144, 19C0023, NCI-2017-02296, P162941

Population segments: BRCA, STK11, Second line, Stage III, Stage IV

Phase: II

Therapy: olaparib

Location: United States

US States: CA, CO, KY, MD, NY, OK

Contact: Multiple contacts: See www.clinicaltrials.gov for complete list of contacts.

NCT03742895
A Phase II Study of Olaparib Monotherapy in Participants With Previously Treated, Homologous Recombination Repair Mutation (HRRm) or Homologous Recombination Deficiency (HRD) Positive Advanced Cancer

Cancer type: Breast Cancer

Variant class: BRCA1 mutation

Other inclusion criteria: BRCA1 germline mutation negative, BRCA2 germline mutation negative

Other identifiers: (MK-7339-002 / LYNK-002), 19-139, 194694, 2018-01891, 7339-002, 7339-002-00, EudraCT Number: 2018-003007-19, IRAS ID: 255158, JapicCTI-194694, LYNK002, LYNK-002, MK7339-002, MK-7339-002, MK7339-002-00, NCI-2018-03519, PER-046-18, SNCTP000003157

Population segments: BRCA, Fourth line or greater, Second line, Stage III, Stage IV, Third line

Exclusion criteria variant classes: BRCA germline mutation (Breast Cancer, Ovarian Cancer), BRCA mutation (Ovarian Cancer)

Phase: II

Therapy: olaparib

Locations: Argentina, Australia, Canada, Colombia, Denmark, France, Guatemala, Ireland, Israel, Italy, Mexico, Peru, Republic of Korea, Romania, Russian Federation, Spain, Switzerland, Turkey, United Kingdom, United States

US States: AZ, CA, CO, GA, KY, MA, MD, MI, NE, NJ, NY, SD, UT, WA

Contact: Toll Free Number [888-577-8839; Trialsites@merck.com]

Disclaimer: The data presented here is from a curated knowledgebase of publicly available information, but may not be exhaustive. The data version is 2021.11(003).

BRCA1 mutation (continued)

NCT03786796
Phase II Study of Olaparib in Metastatic Renal Cell Carcinoma Patients Harboring a BAP-1 or Other DNA Repair Gene Mutations (ORCHID)

Cancer type: Kidney Cancer

Variant class: BRCA1 mutation

Other identifiers: CRMS-70750, J18166, NCI-2019-06309, ORCHID

Population segments: Fourth line or greater, Second line, Stage IV, Third line

Phase: II

Therapy: olaparib

Location: United States

US State: MD

Contact: Irina Rifkind [410-502-2043; irifkin1@jhmi.edu]

NCT04042831
A Phase II Study of Olaparib in Patients With Advanced Biliary Tract Cancer With Aberrant DNA Repair Gene Mutations

Cancer type: Biliary Tract Carcinoma

Variant class: BRCA1 mutation

Other identifiers: ACCRU-ICRN-1702, NCI-2019-04434

Population segments: Second line, Stage IV

Phase: II

Therapy: olaparib

Location: United States

US States: AZ, MN, NY, TX

Contact: Multiple contacts: See www.clinicaltrials.gov for complete list of contacts.

NCT04515836
Phase II Trial of Olaparib in Homologous Recombination Deficient (HRD) Malignant Mesothelioma

Cancer type: Mesothelioma

Variant class: BRCA1 mutation

Population segments: (N/A), Second line

Phase: II

Therapy: olaparib

Location: United States

US State: IL

Contact: Dr. Hedy L. Kindler [773-702-0360; hkindler@medicine.bsd.uchicago.edu]

NCT04858334
APOLLO: A Randomized Phase II Double-Blind Study of Olaparib Versus Placebo Following Adjuvant Chemotherapy in Patients With Resected Pancreatic Cancer and a Pathogenic BRCA1, BRCA2 or PALB2 Mutation

Cancer type: Pancreatic Cancer

Variant class: BRCA1 mutation

Other identifiers: APOLLO, CTSU/EA2192, EA2192, NCI-2020-05659

Population segments: Adjuvant, BRCA, Maintenance/Consolidation, Stage 0, Stage I, Stage II

Phase: II

Therapy: olaparib

Location: United States

US States: AZ, FL, IA, IL, IN, KS, MI, MO, NC, NJ, NM, NV, NY, OH, OR, PA, WA, WI

Contact: Multiple contacts: See www.clinicaltrials.gov for complete list of contacts.

Disclaimer: The data presented here is from a curated knowledgebase of publicly available information, but may not be exhaustive. The data version is 2021.11(003).

BRCA1 mutation (continued)

NCT02849496

A Phase II Open-Label, Randomized Study of PARP Inhibition (Olaparib) Either Alone or in Combination With Anti-PD-L1 Therapy (Atezolizumab; MPDL3280A) in Homologous DNA Repair (HDR) Deficient, Locally Advanced or Metastatic Non-HER2-Positive Breast Cancer.

Cancer type: Breast Cancer

Variant class: BRCA1 mutation

Other inclusion criteria: ERBB2 negative

Other identifiers: 10020, 109587 (10020), 1608018258, 17-709, 18979, 5UM1CA186709-04, Clinical Trial 18979, NCI-2016-01130, OSU-17011, PHII-146

Population segments: BRCA, HER2 negative, Second line, Stage III, Stage IV

Phase: II

Therapies: olaparib, atezolizumab

Location: United States

US States: CA, CO, CT, FL, IL, MA, MD, MI, MO, NC, NY, OH, PA, TN, UT

Contact: Multiple contacts: See www.clinicaltrials.gov for complete list of contacts.

NCT04939662

Phase II, Single-arm Study of Olaparib and Bevacizumab Combination Therapy in Relapsed Small Cell Lung Cancer Subjects With DNA Damage Response and the Repair Pathway Alteration, ATM Deficiency, SLFN11 Positive, or POU2F3 Positive

Cancer type: Small Cell Lung Cancer

Variant class: BRCA1 mutation

Other identifier: SUKSES-B2

Population segments: (N/A), BRCA, Pulmonary, Second line, Third line

Phase: II

Therapies: olaparib, bevacizumab

Location: Republic of Korea

NCT04261465

The NUVOLA TRIAL: Neoadjuvant Chemotherapy in Unresectable Ovarian Cancer With OLAparib and Weekly Carboplatin Plus Paclitaxel: A Phase II Open-label Multicentre Study

Cancer type: Ovarian Cancer

Variant class: BRCA1 mutation

Other identifiers: 2317, 2345, EudraCT Number: 2018-002687-65, NUVOLA, NUVOLA trial

Population segments: BRCA, Maintenance/Consolidation, Neoadjuvant, Stage III, Stage IV

Phase: II

Therapies: olaparib, chemotherapy

Location: Italy

NCT04633902

Phase II Study of Olaparib in Combination With Pembrolizumab in Patients With Advanced Melanoma With Homologous Recombination (HR) Pathway Gene Mutation

Cancer type: Melanoma

Variant class: BRCA1 mutation

Other identifier: CPMC19-MEL01

Population segments: BRAF, Second line, Stage III, Stage IV

Phase: II

Therapies: olaparib, pembrolizumab

Location: United States

US State: CA

Contact: Dr. Kevin B. Kim [415-885-8600; ClinicalResearch@sutterhealth.org]

Disclaimer: The data presented here is from a curated knowledgebase of publicly available information, but may not be exhaustive. The data version is 2021.11(003).

BRCA1 mutation (continued)

NCT03248570
Phase II Open Label Study of Pembrolizumab in Patients With Metastatic Castration Resistant Prostate Cancer (mCRPC) With or Without DNA Damage Repair Defects

Cancer type: Castration-Resistant Prostate Cancer

Variant class: BRCA1 mutation

Other identifiers: 16557, 17-22165, CC 16557, NCI-2017-02408

Population segments: BRCA, Hormone refractory, Line of therapy N/A, Stage IV

Phase: II

Therapy: pembrolizumab

Location: United States

US State: CA

Contact: Patricia Li [415-476-5975; GUTrials@ucsf.edu]

NCT03506997
PERSEUS1: Phase II Trial of the Immune Checkpoint Inhibitor Pembrolizumab for Patients Suffering From Metastatic Prostate Cancer

Cancer type: Castration-Resistant Prostate Cancer

Variant class: BRCA1 mutation

Other identifiers: CCR4559, EudraCT Number: 2017-000931-15, ICR-CTSU/2016/10060, IRAS ID 208952, PERSEUS1

Population segments: Hormone refractory, Second line, Stage IV

Phase: II

Therapy: pembrolizumab

Location: United Kingdom

NCT04089189
A Phase II Study to Evaluate the Efficacy, Safety, and Tolerability of IMP4297 Capsules in Subjects With Germline and/or Systemic BRCA1/2 Mutation Positive Advanced Ovarian Cancer With at Least 2 Prior Lines of Standard Systemic Therapy

Cancer type: Ovarian Cancer

Variant class: BRCA1 mutation

Other identifiers: CTR20190652, IMP4297-2018-CN01

Population segments: Stage III, Stage IV, Third line

Phase: II

Therapy: senaparib

Location: China

NCT03990896
Evaluation of Talazoparib, a PARP Inhibitor, in Patients With Somatic BRCA Mutant Metastatic Breast Cancer: Genotyping Based Clinical Trial

Cancer type: Breast Cancer, Triple Negative Breast Cancer

Variant class: BRCA1 mutation

Other inclusion criteria: ERBB2 negative, Hormone receptor positive

Other identifiers: 19-188, NCI-2019-07732

Population segments: BRCA, Estrogen receptor positive, Fourth line or greater, HER2 negative, Progesterone receptor positive, Second line, Stage IV, Third line, Triple receptor negative

Exclusion criteria variant classes: BRCA1 germline mutation (Breast Cancer, Triple Negative Breast Cancer), BRCA2 germline mutation (Breast Cancer, Triple Negative Breast Cancer)

Phase: II

Therapy: talazoparib

Location: United States

US States: MA, TX

Contact: Dr. Neelima Vidula [617-724-4000; nvidula@mgh.harvard.edu]

Disclaimer: The data presented here is from a curated knowledgebase of publicly available information, but may not be exhaustive. The data version is 2021.11(003).

BRCA1 mutation (continued)

NCT04892693
Talazoparib in Advanced Breast Cancer Patients With Homologous Recombinant Deficiency: A Phase II Clinical and Exploratory Biomarker Study of Talazoparib

Cancer type: Breast Cancer

Variant class: BRCA1 mutation

Other identifier: H-2009-079-1157

Population segments: BRCA, Line of therapy N/A, Stage III, Stage IV

Phase: II

Therapy: talazoparib

Location: Republic of Korea

NCT04740190
Combination Talazoparib - Carboplatin for Recurrent High-grade Glioma With DNA Damage Repair Deficiency (DDRd)

Cancer type: Anaplastic Astrocytoma, Anaplastic Oligoastrocytoma, Anaplastic Oligodendroglioma, Anaplastic Pleomorphic Xanthoastrocytoma, Diffuse Midline Glioma, Glioblastoma

Variant class: BRCA1 mutation

Other identifiers: TAC-GReD, UW-20396

Population segments: (N/A), BRCA, Second line

Phase: II

Therapies: talazoparib, chemotherapy

Location: Hong Kong

NCT04253262
A Phase Ib/II Study of Copanlisib Combined With Rucaparib in Patients With Metastatic Castration-resistant Prostate Cancer

Cancer type: Castration-Resistant Prostate Cancer

Variant class: BRCA1 mutation

Other identifiers: BrUOG 360, BrUOG360

Population segments: Hormone refractory, Second line, Stage IV

Phase: I/II

Therapies: copanlisib, rucaparib

Location: United States

US State: RI

Contact: BrUOG [401-863-3000; BrUOG@brown.edu]

NCT03685331
Harnessing Olaparib, Palbociclib and Endocrine Therapy: A Phase I/II Trial of Olaparib, Palbociclib and Fulvestrant in Patients With BRCA Mutation-associated, Hormone Receptor-positive, Human Epidermal Growth Factor Receptor 2 (HER2)-Negative Metastatic Breast Cancer (HOPE)

Cancer type: Breast Cancer

Variant class: BRCA1 mutation

Other inclusion criteria: ERBB2 negative, Hormone receptor positive

Other identifiers: (HOPE), NCI-2019-04210, UPCC 21118

Population segments: BRCA, Estrogen receptor positive, First line, HER2 negative, Progesterone receptor positive, Second line, Stage III, Stage IV, Third line

Phase: I/II

Therapies: olaparib, palbociclib, hormone therapy

Location: United States

US State: PA

Contact: Alexandra Torres [855-216-0098; PennCancerTrials@emergingmed.com]

Disclaimer: The data presented here is from a curated knowledgebase of publicly available information, but may not be exhaustive. The data version is 2021.11(003).

BRCA1 mutation (continued)

No NCT ID - see other identifier(s)
An Open-Label, Randomised, Phase I/II Trial Of Rucaparib Combined With Nivolumab + or - Ipilimumab To Augment Response In Homologous Repair Deficient Patients With Relapsed Ovarian, Primary Peritoneal And Fallopian Tube Cancer

Cancer type: Ovarian Cancer

Variant class: BRCA1 mutation

Other identifiers: CeNturIOn, CeNturIOn-2016, EudraCT Number: 2017-004780-13, ISRCTN10490346

Population segments: (N/A), BRCA, Fourth line or greater, Second line, Third line

Phase: I/II

Therapies: rucaparib, nivolumab, ipilimumab

Location: United Kingdom

NCT03964532
TALAVE: A Pilot Trial of Induction Talazoparib Followed by Combination of Talazoparib and Avelumab in Advanced Breast Cancer

Cancer type: Breast Cancer

Variant class: BRCA1 mutation

Other inclusion criteria: ERBB2 negative

Other identifiers: NCI-2019-06240, STUDY00000023, TALAVE

Population segments: Line of therapy N/A, Stage III, Stage IV

Phase: I/II

Therapies: talazoparib, avelumab

Location: United States

US States: DC, NC, UT

Contact: Dr. Julie Collins [202-444-2223; Julie.Collins@gunet.georgetown.edu]

NCT04673448
Phase IB Trial of Niraparib and TSR-042 in Patients With BRCA-Mutated Breast, Pancreas or Ovary Cancer

Cancer type: Breast Cancer, Ovarian Cancer, Pancreatic Cancer

Variant class: BRCA1 mutation

Other identifiers: 10020, NCI-2020-11636, RG1005140

Population segments: BRCA, Estrogen receptor positive, HER2 positive, Progesterone receptor positive, Second line, Stage III, Stage IV

Phase: I

Therapies: niraparib, dostarlimab

Location: United States

US State: WA

Contact: Elizabeth M. Swisher [206-543-3669; swishere@uw.edu]

NCT04890613
Phase Ib Expansion Study of CX-5461 in Patients With Solid Tumours and BRCA2 and/or PALB2 Mutation

Cancer type: Ovarian Cancer

Variant class: BRCA1 mutation

Other identifier: CX-5461-04

Population segments: BRCA, Second line, Stage II, Stage III, Stage IV

Phase: I

Therapy: pidnarulex

Location: Canada

Disclaimer: The data presented here is from a curated knowledgebase of publicly available information, but may not be exhaustive. The data version is 2021.11(003).

BRCA1 mutation (continued)

NCT04598321

BrUOG 390: Neoadjuvant Treatment With Talazoparib for Women With Newly Diagnosed, Advanced Ovarian Cancer Associated With a Mutation in BRCA1 or BRCA2 (mBRCA)

Cancer type: Ovarian Cancer

Variant class: BRCA1 mutation

Other identifier: BrUOG 390

Population segments: BRCA, Maintenance/Consolidation, Neoadjuvant, Stage II, Stage III, Stage IV

Phase: I

Therapy: talazoparib

Location: United States

US State: RI

Contact: BrUOG [401-863-3000; BrUOG@Brown.edu]

NCT03598270

A Phase III Randomized, Double-blinded Trial of Platinum-based Chemotherapy With or Without Atezolizumab Followed by Niraparib Maintenance With or Without Atezolizumab in Patients With Recurrent Ovarian, Tubal or Peritoneal Cancer and Platinum Treatment-free Interval (TFIp) >6 Months

Cancer type: Ovarian Cancer

Variant class: BRCA mutation

Other identifiers: AGO ovary 2.33, ANITA, BGOG-ov-41, ENGOT-ov41, ENGOT-OV41/GEICO 69-O/ANITA, ENGOT-ov41/GEICO/ANITA, ENGOT-Ov41/GEICO69-O/ANITA, EudraCT Number: 2018-000366-11, GEICO 69-O, GEICO-69-O

Population segments: (N/A), BRCA, Maintenance/Consolidation, Second line, Third line

Phase: III

Therapies: atezolizumab, chemotherapy, niraparib

Locations: Belgium, France, Germany, Italy, Spain

NCT03278717

International Phase III Randomised Study to Evaluate the Efficacy of Maintenance Therapy With Olaparib and Cediranib or Olaparib Alone in Patients With Relapsed Ovarian Cancer Following a Response to Platinum-based Chemotherapy

Cancer type: Ovarian Cancer

Variant class: BRCA mutation

Other identifiers: 36280, ACTRN12618000498291, ANZGOG 1415/2018, CRUK/15/074, CTC 0133, ENGOT-ov35, ENGOT-ov35/NCRI/ICON 9, EudraCT Number: 2017-000161-75, ICON9, NC174, OV26 (CRUK/UCL ICON9), UCL/14/0795

Population segments: BRCA, Maintenance/Consolidation, Second line, Stage III, Stage IV

Phase: III

Therapies: cediranib, olaparib

Locations: Australia, Canada, New Zealand, United Kingdom

Disclaimer: The data presented here is from a curated knowledgebase of publicly available information, but may not be exhaustive. The data version is 2021.11(003).

BRCA1 mutation (continued)

NCT04915755
A Randomized Phase III Double-Blinded Study Comparing the Efficacy and Safety of Niraparib to Placebo in Participants With Either HER2-Negative BRCA-Mutated or Triple-Negative Breast Cancer With Molecular Disease Based on Presence of Circulating Tumor DNA After Definitive Therapy (ZEST)

Cancer type: Breast Cancer

Variant class: BRCA mutation

Other inclusion criteria: ERBB2 negative

Other identifiers: 213831, EudraCT Number: 2020-003973-23, GSK 213831 ZEST, SNCTP000004543, ZEST

Population segments: BRCA, Estrogen receptor positive, HER2 negative, Progesterone receptor positive, Second line, Stage I, Stage II, Stage III, Triple receptor negative

Phase: III

Therapy: niraparib

Locations: Canada, France, Ireland, Italy, Netherlands, Norway, Poland, Russian Federation, Spain, United Kingdom, United States

US State: IL

Contact: US GSK Clinical Trials Call Center [877-379-3718; GSKClinicalSupportHD@gsk.com]

NCT03462342
Combination ATR and PARP Inhibitor (CAPRI) Trial With AZD 6738 and Olaparib in Recurrent Ovarian Cancer

Cancer type: Ovarian Cancer

Variant class: BRCA mutation

Other identifiers: 20-320, CAPRI, ESR-16-12311, NCI-2018-02375, UPCC 15817

Population segments: BRCA, Fourth line or greater, Second line, Stage III, Stage IV, Third line

Phase: II

Therapies: ceralasertib, olaparib

Location: United States

US States: MA, MD, PA

Contact: Diego Rodriguez [215-614-0234; diegorod@pennmedicine.upenn.edu]

No NCT ID - see other identifier(s)
An Investigator-Initiated Phase II Trial Of Niraparib For Patients With BRCA-Mutated Unresectable / Recurrent Biliary Tract, Pancreatic And Other Gastrointestinal Cancer (NIR-B)

Cancer type: Cholangiocarcinoma, Colon Cancer, Gastrointestinal Stromal Tumor, Pancreatic Cancer, Rectal Cancer

Variant class: BRCA mutation

Other identifiers: jRCT2011200023, NIR-B

Population segments: BRCA, Second line, Stage III, Stage IV, Third line

Phase: II

Therapy: niraparib

Location: Japan

NCT04716686
Niraparib Monotherapy as Maintain and Recurrent Treatment of Endometrial Serous Carcinoma: A Multi-center, Open-label, Prospective Clinical Study

Cancer type: Endometrial Serous Adenocarcinoma

Variant class: BRCA mutation

Other identifier: ZL-2306-914

Population segments: BRCA, Fourth line or greater, Maintenance/Consolidation, Second line, Stage III, Stage IV, Third line

Phase: II

Therapy: niraparib

Location: China

Disclaimer: The data presented here is from a curated knowledgebase of publicly available information, but may not be exhaustive. The data version is 2021.11(003).

BRCA1 mutation (continued)

NCT04348045
MAZEPPA: Phase II PRODIGE-GERCOR Study to Evaluate MAintenance Therapy With Olaparib or Selumetinib Plus Durvalumab According to BRCAness and KRAS Somatic Status Personalized in Metastatic Pancreatic Adenocarcinoma Patients

Cancer type: Pancreatic Cancer

Variant class: BRCA mutation

Other inclusion criteria: DPYD wild type

Other identifiers: D19-02, EudraCT Number: 2019-004366-18, MAZEPPA D19-02, MAZEPPA D19-02 PRODIGE 72, MAZEPPA_D19-02, PRODIGY 72 - MAZEPPA

Population segments: BRCA, KRAS, Maintenance/Consolidation, Stage IV

Phase: II

Therapy: olaparib

Location: France

NCT03025035
Open Label, Phase II Pilot Study of Immune Checkpoint Inhibition With Pembrolizumab in Combination With PARP Inhibition With Olaparib in Advanced BRCA-mutated or HDR-defect Breast Cancers

Cancer type: Breast Cancer

Variant class: BRCA mutation

Other identifier: IIT2015-18-Mita-MK3475

Population segments: BRCA, Estrogen receptor positive, HER2 negative, HER2 positive, Progesterone receptor positive, Second line, Stage III, Stage IV, Triple receptor negative

Phase: II

Therapies: pembrolizumab, olaparib

Location: United States

US State: CA

Contact: Parisa Mirzadehgan [310-967-4387; Parisa.Mirzadehgan@cshs.org]

NCT04666740
A Phase II Trial to Evaluate the Safety and Antitumor Activity of Pembrolizumab and OLApaRib (POLAR) Maintenance for Patients With Metastatic Pancreatic Ductal Adenocarcinoma and Homologous Recombination Deficiency and/or Exceptional Treatment Response to Platinum-Based Therapy

Cancer type: Pancreatic Cancer

Variant class: BRCA1 aberration

Other identifier: 20-481

Population segments: BRCA, Maintenance/Consolidation, Stage IV

Phase: II

Therapies: pembrolizumab, olaparib

Location: United States

US States: NJ, NY

Contact: Dr. Wungki Park [646-888-4543; yaqubiea@mskcc.org]

Disclaimer: The data presented here is from a curated knowledgebase of publicly available information, but may not be exhaustive. The data version is 2021.11(003).

BRCA1 mutation (continued)

NCT02264678
A Modular Phase I, Open-Label, Multicentre Study to Assess the Safety, Tolerability, Pharmacokinetics and Preliminary Anti-tumour Activity of Ceralasertib in Combination With Cytotoxic Chemotherapy and/or DNA Damage Repair/Novel Anti-cancer Agents in Patients With Advanced Solid Malignancies

Cancer type: Breast Cancer, Ovarian Cancer

Variant class: BRCA mutation

Other inclusion criteria: ERBB2 negative

Other identifiers: 0C-14-9, 16-031, 18-342, D5330C00004, EudraCT Number: 2014-002233-66, IRAS ID 160200, NCI-2015-00687, Study 4

Population segments: Adenocarcinoma, BRCA, HER2 negative, Pulmonary, Second line, Stage III, Stage IV, Third line, Triple receptor negative

Exclusion criteria variant class: ERBB2 positive (Breast Cancer)

Phase: I

Therapies: ceralasertib, olaparib

Locations: France, Republic of Korea, United Kingdom, United States

US States: CA, MA, NY

Contact: AstraZeneca Clinical Study Information Center [877-240-9479; information.center@astrazeneca.com]

NCT04169841
Precision Medicine Phase II Study Evaluating the Efficacy of a Double Immunotherapy by Durvalumab and Tremelimumab Combined With Olaparib in Patients With Solid Cancers and Carriers of Homologous Recombination Repair Genes Mutation in Response or Stable After Olaparib Treatment

Cancer type: Breast Cancer, Endometrial Carcinoma, Head and Neck Cancer, Kidney Cancer, Non-Small Cell Lung Cancer, Ovarian Cancer, Pancreatic Cancer, Prostate Cancer

Variant class: HRR mutation

Other identifier: GUIDE2REPAIR

Population segments: Fourth line or greater, Hormone refractory, Maintenance/ Consolidation, Second line, Stage III, Stage IV, Third line

Exclusion criteria variant classes: ALK fusion (Lung Cancer), BRAF mutation (Lung Cancer), EGFR mutation (Lung Cancer), ROS1 mutation (Lung Cancer)

Phase: II

Therapies: durvalumab, tremelimumab, olaparib

Location: France

NCT04030559
Phase II Trial of PARP Inhibitor Niraparib for Men With High Risk Prostate Cancer and DNA Damage Response Defects

Cancer type: Prostate Cancer

Variant class: HRR mutation

Other identifiers: 1412676, NCI-2019-04365, UCDCC#279

Population segments: First line, Neoadjuvant, Stage I, Stage II, Stage IV

Phase: II

Therapy: niraparib

Location: United States

US State: CA

Contact: Marc Dall'Era [916-734-3771; mdallera@ucdavis.edu]

BRCA1 mutation (continued)

NCT04940637

A Phase II, Open-Label, Single Arm, Prospective, Multicenter Study of Niraparib Plus Dostarlimab in Patients With Advanced NSCLC and/or MPM, and Positive for PD-L1 Expression and Germline or Somatic Mutations in the HRR Genes

Cancer type: Mesothelioma, Non-Small Cell Lung Cancer

Variant class: HRR mutation

Other inclusion criteria: ALK fusion negative, CD274 expression, EGFRi sensitizing mutation negative, ROS1 fusion negative or CD274 expression

Other identifiers: EudraCT Number: 2020-000109-10, UNITO-001, UNITO-001/2020

Population segments: Second line, Stage III, Stage IV

Exclusion criteria variant classes: ALK fusion (Non-Small Cell Lung Cancer), EGFRi sensitizing mutation (Non-Small Cell Lung Cancer), ROS1 fusion (Non-Small Cell Lung Cancer)

Phase: II

Therapies: niraparib, dostarlimab

Location: Italy

NCT05002868

A Multi-center, Open-label, Phase I/Ib Study to Assess the Safety, Pharmacokinetics and Anti-tumor Activity of RP12146, a Poly (ADP-ribose) Polymerase (PARP) Inhibitor, in Patients With Locally Advanced or Metastatic Solid Tumors

Cancer type: Biliary Tract Carcinoma, Breast Cancer, Colorectal Cancer, Endometrial Carcinoma, Gastric Cancer, Ovarian Cancer, Pancreatic Cancer, Prostate Cancer, Small Cell Lung Cancer

Variant class: HRR mutation

Other identifier: RP12146-2101

Population segments: Extensive, Pulmonary, Second line, Stage III, Stage IV

Exclusion criteria variant class: ERBB2 positive (Breast Cancer)

Phase: I

Therapy: RP12146

Location: Poland

NCT03395197

A Phase III, Randomized, Double-blind, Placebo-controlled Study Of Talazoparib With Enzalutamide In Metastatic Castration-resistant Prostate Cancer

Cancer type: Castration-Resistant Prostate Cancer

Variant class: DNA repair mutation

Other identifiers: BASIC-ROYAL / HIGH-ROYAL, C3441021, CSET 2938, CTR20191443, CTR20191444, EudraCT Number: 2017-003295-31, GU 170 - C3441021, HCC 19-054, JapicCTI-194665, LH19.022, MOH_2019-05-16_007127, NCI-2018-01651, Pfizer C3441021, R19062M, RECF3951, RWF_C3441021 TALAPRO-2, TALAPRO-2, TX228665, UCI-18-47, VICCURO17115

Population segments: First line, Hormone refractory, Second line, Stage III, Stage IV

Phase: III

Therapies: talazoparib, hormone therapy

Locations: Argentina, Australia, Belgium, Brazil, Canada, Chile, China, Czech Republic, Finland, France, Germany, Hungary, Israel, Italy, Japan, New Zealand, Norway, Peru, Poland, Portugal, Republic of Korea, South Africa, Spain, Sweden, United Kingdom, United States

US States: AZ, CA, CO, FL, GA, IL, IN, KS, MO, NE, NJ, NY, OH, OK, OR, PA, SC, TN, TX, UT, VA, WI

Contact: Pfizer CT.gov Call Center [800-718-1021; ClinicalTrials.gov_Inquiries@pfizer.com]

Disclaimer: The data presented here is from a curated knowledgebase of publicly available information, but may not be exhaustive. The data version is 2021.11(003).

BRCA1 mutation (continued)

NCT04821622

TALAPRO-3: A Phase III, Randomized, Double-blind, Study of Talazoparib With Enzalutamide Versus Placebo With Enzalutamide in Men With DDR Gene Mutated Metastatic Castration-sensitive Prostate Cancer

Cancer type: Prostate Adenocarcinoma

Variant class: DNA repair mutation

Other identifiers: C3441052, CTR20212191, EudraCT Number: 2021-000248-23, jRCT2031210098, TALAPRO-3

Population segments: Second line, Stage IV

Phase: III

Therapies: talazoparib, hormone therapy

Locations: Japan, Republic of Korea, United States

US States: CA, DC, FL, GA, IL, MI, NJ, NY, PA, SC, TX, UT

Contact: Pfizer CT.gov Call Center [800-718-1021; ClinicalTrials.gov_Inquiries@pfizer.com]

NCT04038502

An Open-label, Multicenter Phase II Study to Compare the Efficacy of Carboplatin as First-line Followed by Second-line Olaparib Versus Olaparib as First-line Followed by Second-line Carboplatin in the Treatment of Patients With Castration Resistant Prostate Cancer Containing Homologous Recombination Deficiency

Cancer type: Castration-Resistant Prostate Cancer

Variant class: DNA repair mutation

Other identifiers: 01781, 01783, 19-08, BRACeD, CX001963-01, ONCA-009-18F

Population segments: First line, Hormone refractory, Second line, Stage IV

Phase: II

Therapies: olaparib, chemotherapy

Location: United States

US States: CA, DC, FL, IL, MI, NC, NY, PA, WA

Contact: Dr. Robert B. Montgomery [206-277-6878; rbmontgo@u.washington.edu]

No NCT ID - see other identifier(s)

Single-arm Exploratory Study for Sintilimab Second-line or Later-line Treatment of Advanced Non-small Cell Lung Cancer with DDR Pathway Gene Mutation

Cancer type: Non-Small Cell Lung Cancer

Variant class: DNA repair mutation

Other identifier: ChiCTR1900023234

Population segments: Fourth line or greater, Second line, Stage III, Stage IV, Third line

Exclusion criteria variant classes: ALK fusion (Non-Small Cell Lung Cancer), EGFRi sensitizing mutation (Non-Small Cell Lung Cancer), ROS1 fusion (Non-Small Cell Lung Cancer)

Phase: I/II

Therapy: sintilimab

Location: China

Disclaimer: The data presented here is from a curated knowledgebase of publicly available information, but may not be exhaustive. The data version is 2021.11(003).

BRCA1 mutation (continued)

NCT04095273

A Multicenter, Non-randomized, Open-label Phase Ib Study to Determine the Maximum Tolerated and Recommended Phase II Dose of the ATR Inhibitor BAY1895344 in Combination With Pembrolizumab and to Characterize Its Safety, Tolerability, Pharmacokinetics and Preliminary Anti-tumor Activity in Patients With Advanced Solid Tumors

Cancer type: Breast Cancer, Castration-Resistant Prostate Cancer, Colorectal Cancer, Gastric Cancer, Gastroesophageal Junction Adenocarcinoma, Non-Small Cell Lung Cancer, Pancreatic Cancer

Variant class: DNA repair mutation

Other inclusion criteria: ATM mutation negative or ATM mutation negative, ERBB2 negative, Hormone receptor positive or ATM underexpression

Other identifiers: 19741, EudraCT Number: 2018-003420-36, Keynote 919, NCI-2019-06734

Population segments: HER2 negative, Hormone refractory, Second line, Stage III, Stage IV

Phase: I

Therapies: BAY-1895344, pembrolizumab

Locations: Germany, Spain, Switzerland, United States

US States: CA, CT, MA, MD, NC, NY, OH, TX

Contact: Bayer Clinical Trials Contact [clinical-trials-contact@bayer.com]

NCT04849364

A Phase II Circulating Tumor DNA Enriched, Genomically Directed Post-neoadjuvant Trial for Patients With Residual Triple Negative Breast Cancer (PERSEVERE)

Cancer type: Triple Negative Breast Cancer

Variant class: DNA repair pathway

Other identifiers: HCRN BRE18-334, PERSEVERE

Population segments: Adjuvant, HER2 negative, Stage I, Stage II, Stage III, Triple receptor negative

Phase: II

Therapies: talazoparib, chemotherapy

Location: United States

US State: IN

Contact: Dr. Bryan P. Schneider [317-948-3885; bpschnei@iu.edu]

Disclaimer: The data presented here is from a curated knowledgebase of publicly available information, but may not be exhaustive. The data version is 2021.11(003).

BRCA1 splice site mutation

NCT02029001

A Two-period, Multicenter, Randomized, Open-label, Phase II Study Evaluating the Clinical Benefit of a Maintenance Treatment Targeting Tumor Molecular Alterations in Patients with Progressive Locally-advanced or Metastatic Solid Tumors.

Cancer type: Unspecified Solid Tumor

Variant class: BRCA1 mutation

Other identifiers: ET12-081, EudraCT Number: 2012-004510-34, MOST, ProfiLER

Population segments: Maintenance/Consolidation, Second line, Stage III, Stage IV, Third line

Exclusion criteria variant classes: ATM mutation (Gastric Cancer), BRCA1 germline mutation (Gastric Cancer), BRCA1 mutation (Gastric Cancer), BRCA2 germline mutation (Gastric Cancer), BRCA2 mutation (Gastric Cancer), HRR mutation (Gastric Cancer), PALB2 mutation (Gastric Cancer), RAD51 mutation (Gastric Cancer)

Phase: II

Therapy: olaparib

Location: France

NCT03967938

Efficacy of Olaparib in Advanced Cancers Occurring in Patients With Germline Mutations or Somatic Tumor Mutations in Homologous Recombination Genes

Cancer type: Unspecified Solid Tumor

Variant class: BRCA1 mutation

Other identifiers: 1-2018 BSMO, 1-2018BSMO, EudraCT Number: 2018-002966-37, Precision 2 - Olaparib

Population segments: Line of therapy N/A, Stage III, Stage IV

Exclusion criteria variant classes: BRCA1 germline mutation (Breast Cancer, Pancreatic Cancer, Prostate Cancer), BRCA2 germline mutation (Breast Cancer, Pancreatic Cancer, Prostate Cancer)

Phase: II

Therapy: olaparib

Location: Belgium

NCT02693535

Targeted Agent and Profiling Utilization Registry (TAPUR) Study.

Cancer type: Unspecified Solid Tumor

Variant class: BRCA1 mutation

Other identifiers: 20170529, NCI-2017-00510, Pro00014171, TAPUR

Population segments: Aggressive, BRAF, Diffuse large B-cell lymphoma (DLBCL), Extranodal marginal zone B-cell lymphoma (MALT), FGFR, First line, Follicular lymphoma (FL), Fourth line or greater, Indolent, Lymphoblastic lymphoma (LBL), Mantle cell lymphoma (MCL), Other subtype, Second line, Small lymphocytic lymphoma (SLL), Stage III, Stage IV, Third line, Waldenstrom's macroglobulinemia (WM)

Phase: II

Therapies: olaparib, talazoparib, atezolizumab + talazoparib

Location: United States

US States: AL, AZ, CA, FL, GA, HI, IL, IN, ME, MI, NC, ND, NE, NH, OH, OR, PA, SD, TX, UT, VA, WA

Contact: Pam Mangat [pam.mangat@asco.org]

Disclaimer: The data presented here is from a curated knowledgebase of publicly available information, but may not be exhaustive. The data version is 2021.11(003).

BRCA1 splice site mutation (continued)

NCT04171700
A Phase II Multicenter, Open-label Study of Rucaparib as Treatment for Solid Tumors Associated With Deleterious Mutations in Homologous Recombination Repair Genes

Cancer type: Unspecified Solid Tumor

Variant class: BRCA1 mutation

Other identifiers: 19-756, 20619, AAAS6974, CO-338-100, LODESTAR, NCI-2019-08226

Population segments: BRCA, Second line, Stage III, Stage IV

Exclusion criteria variant classes: BRCA1 germline mutation (Breast Cancer, Ovarian Cancer, Prostate Cancer), BRCA2 germline mutation (Breast Cancer, Ovarian Cancer, Prostate Cancer)

Phase: II

Therapy: rucaparib

Location: United States

US States: CA, FL, IA, IL, MA, MN, NY, OH, OK, PA, TN, WA

Contact: Clovis Oncology For North America [844-258-7662; medinfo@clovisoncology.com]

NCT02286687
Phase II Study of the PARP Inhibitor Talazoparib in Advanced Cancer Patients With Somatic Alterations in BRCA1/2, Mutations/Deletions in Other BRCA Pathway Genes and Germline Mutation in BRCA1/2 (Not Breast or Ovarian Cancer)

Cancer type: Unspecified Cancer

Variant class: BRCA1 mutation

Other identifiers: 2013-0961, NCI-2014-02494

Population segments: Fourth line or greater, Stage III, Stage IV

Exclusion criteria variant classes: BRCA1 germline mutation (Breast Cancer, Ovarian Cancer), BRCA2 germline mutation (Breast Cancer, Ovarian Cancer)

Phase: II

Therapy: talazoparib

Location: United States

US State: TX

Contact: Dr. Sarina Piha-Paul [713-563-1930; spihapau@mdanderson.org]

NCT04550494
A Pharmacodynamics-Driven Trial of Talazoparib, an Oral PARP Inhibitor, in Patients With Advanced Solid Tumors and Aberrations in Genes Involved in DNA Damage Response

Cancer type: Unspecified Solid Tumor

Variant class: BRCA1 mutation

Other identifiers: 000246, 000264-C, 10000264, 10371, NCI-2020-06906

Population segments: BRCA, Fourth line or greater, HER2 positive, Hormone refractory, Second line, Stage II, Stage III, Stage IV, Third line

Phase: II

Therapy: talazoparib

Location: United States

US State: MD

Contact: Multiple contacts: See www.clinicaltrials.gov for complete list of contacts.

Disclaimer: The data presented here is from a curated knowledgebase of publicly available information, but may not be exhaustive. The data version is 2021.11(003).

BRCA1 splice site mutation (continued)

NCT03842228
A Phase Ib Biomarker-Driven Combination Trial of Copanlisib, Olaparib, and Durvalumab (MEDI4736) in Patients With Advanced Solid Tumors

Cancer type: Unspecified Solid Tumor

Variant class: BRCA1 mutation

Other identifiers: 10217, 19-628, NCI10217, NCI-2019-00601

Population segments: Line of therapy N/A, Stage III, Stage IV

Phase: I

Therapies: copanlisib, olaparib, durvalumab

Location: United States

US States: MA, TX

Contact: Multiple contacts: See www.clinicaltrials.gov for complete list of contacts.

NCT04586335
Open Label, Phase Ib Study to Evaluate the Safety, Tolerability, Pharmacokinetics and Clinical Activity of CYH33, an Oral a-specific PI3K Inhibitor in Combination With Olaparib, an Oral PARP Inhibitor in Patients With Advanced Solid Tumors.

Cancer type: Breast Cancer, Endometrial Carcinoma, Ovarian Cancer, Prostate Cancer, Unspecified Solid Tumor

Variant class: BRCA1 mutation

Other identifiers: CTR20202339, CYH33-G102

Population segments: BRCA, Second line, Stage III, Stage IV

Phase: I

Therapies: HH-CYH33, olaparib

Locations: Australia, United States

US State: TX

Contact: Dr. Jason Sudia [908-380-1329; jason.sudia@haihepharma.com]

NCT03415659
A Phase I, Open-label, Single-center, Single/Multiple-dose, Dose-escalation/ Dose-expansion Clinical Study on Tolerance and Pharmacokinetics of HWH340 Tablet in Patients With Advanced Solid Tumors

Cancer type: Unspecified Solid Tumor

Variant class: BRCA1 mutation

Other identifiers: CTR20171649, HWH340-RFPA 20170821, RFPA 20170821

Population segments: BRCA, Fourth line or greater, HER2 negative, Second line, Stage III, Stage IV, Third line

Phase: I

Therapy: HWH-340

Location: China

NCT04197713
Phase I Sequential Trial of Agents Against DNA Repair (STAR)

Cancer type: Unspecified Solid Tumor

Variant class: BRCA1 mutation

Other identifiers: (STAR), 10329, 2020-0347, NCI-2019-08262, STAR Study

Population segments: Second line, Stage III, Stage IV

Phase: I

Therapies: olaparib, adavosertib

Location: United States

US State: TX

Contact: Site Public Contact [877-632-6789; askmdanderson@mdanderson.org]

Disclaimer: The data presented here is from a curated knowledgebase of publicly available information, but may not be exhaustive. The data version is 2021.11(003).

BRCA1 splice site mutation (continued)

NCT03297606
Canadian Profiling and Targeted Agent Utilization Trial (CAPTUR): A Phase II Basket Trial

Cancer type: Unspecified Solid Tumor

Variant class: BRCA1 aberration

Other identifiers: CA209-9DL, CAPTUR, ESR-17-12831, ML39800, PM1, WI233446

Population segments: Aggressive, Diffuse large B-cell lymphoma (DLBCL), Extranodal marginal zone B-cell lymphoma (MALT), First line, Follicular lymphoma (FL), Indolent, Lymphoblastic lymphoma (LBL), Mantle cell lymphoma (MCL), Other subtype, Second line, Stage III, Stage IV, Waldenstrom's macroglobulinemia (WM)

Phase: II

Therapy: olaparib

Location: Canada

NCT04497116
Phase I/IIa Study of the Safety, Pharmacokinetics, Pharmacodynamics and Preliminary Clinical Activity of RP-3500 Alone or in Combination With Talazoparib in Advanced Solid Tumors With ATR Inhibitor Sensitizing Mutations (TRESR Study)

Cancer type: Unspecified Solid Tumor

Variant class: BRCA1 aberration

Other identifiers: 20-521, EudraCT Number: 2020-000301-87, IRAS ID: 286609, NCI-2020-06869, RP-3500-01, TRESR, TRESR Study

Population segments: Second line, Stage III, Stage IV

Phase: I/II

Therapies: RP-3500, talazoparib

Locations: Canada, Denmark, United Kingdom, United States

US States: MA, NC, NY, RI, TN, TX

Contact: Dr. Peter Manley [857-322-5553; pmanley@reparerx.com]

NCT04123366
A Phase II Study of Olaparib in Combination With Pembrolizumab in Participants With Previously Treated, Homologous Recombination Repair Mutation (HRRm) and/or Homologous Recombination Deficiency (HRD)-Positive Advanced Cancer

Cancer type: Unspecified Solid Tumor

Variant class: HRR mutation

Other identifiers: 7339-007, EudraCT Number: 2019-001745-40, jRCT2011200025, KEYLYNK-007, MK-7339-007, NCI-2019-08758, PER-029-19

Population segments: BRCA, Second line, Stage III, Stage IV

Exclusion criteria variant classes: BRCA germline mutation (Breast Cancer, Ovarian Cancer), BRCA mutation (Breast Cancer, Ovarian Cancer)

Phase: II

Therapies: olaparib, pembrolizumab

Locations: Argentina, Australia, Canada, Colombia, France, Germany, Guatemala, Israel, Italy, Japan, Latvia, Mexico, Peru, Poland, Puerto Rico, Republic of Korea, Romania, South Africa, Spain, Sweden, Turkey, Ukraine, United States

US States: AL, AZ, CA, CO, FL, GA, KY, NJ, NY, OH, TX, UT, VA

Contact: Toll Free Number [888-577-8839; Trialsites@merck.com]

Disclaimer: The data presented here is from a curated knowledgebase of publicly available information, but may not be exhaustive. The data version is 2021.11(003).

BRCA1 splice site mutation (continued)

NCT03767075
Basket of Baskets: A Modular, Open-label, Phase II, Multicentre Study To Evaluate Targeted Agents in Molecularly Selected Populations With Advanced Solid Tumours

Cancer type: Unspecified Solid Tumor

Variant class: DNA repair mutation

Other identifiers: (Basket of Baskets) (BoB), EudraCT number: 2017-005108-89, MO39164, NL65937.031.18, VHIO17002

Population segments: Second line, Stage III, Stage IV

Phase: II

Therapy: atezolizumab

Locations: France, Germany, Netherlands, Spain, Sweden, United Kingdom

---

NCT04905914
A Phase I/IIa, Open-Label, Safety, Pharmacokinetic, And Preliminary Efficacy Study Of Oral ATRN-119 In Patients With Advanced Solid Tumors

Cancer type: Unspecified Solid Tumor

Variant class: DNA repair mutation

Other identifier: AR-276-01

Population segments: Line of therapy N/A, Stage III, Stage IV

Phase: I/II

Therapy: ATRN-119

Location: United States

US State: MA

Contact: Robert Hasson [619-540-6253; rhasson@pacificlinkconsulting.com]

NCT04901702
A Randomized Phase I/II Study of Talazoparib or Temozolomide in Combination With Onivyde in Children With Recurrent Solid Malignancies and Ewing Sarcoma

Cancer type: Unspecified Solid Tumor

Variant class: HRR pathway

Other identifiers: EudraCT Number: 2021-003570-31, ONITT

Population segments: Second line, Stage IV

Exclusion criteria variant classes: HRR germline pathway (Central Nervous System Neoplasm, Ewing Sarcoma), HRR pathway (Central Nervous System Neoplasm, Ewing Sarcoma)

Phase: I/II

Therapies: talazoparib, chemotherapy

Locations: Canada, United States

US State: TN

Contact: Dr. Sara Federico [866-278-5833; referralinfo@stjude.org]

Disclaimer: The data presented here is from a curated knowledgebase of publicly available information, but may not be exhaustive. The data version is 2021.11(003).

BRCA1 splice site mutation (continued)

NCT03155620
NCI-COG Pediatric MATCH (Molecular Analysis for Therapy Choice) Screening Protocol

Cancer type: Unspecified Solid Tumor

Variant class: DNA repair pathway

Other identifiers: 17-729, 2017-0625, 20170841, APEC1621SC, COGAPEC1621, COGAPEC1621SC, N 55017 SC, NCI-2017-01251, NCI-COG Pediatric MATCH, Pediatric MATCH

Population segments: ALK, Aggressive, BRAF, FGFR, HRAS, Indolent, KRAS, NRAS, Pediatric or Adolescent, Second line, Stage III, Stage IV

Phase: II

Therapy: olaparib

Locations: Puerto Rico, United States

US States: AK, AL, AR, AZ, CA, CO, CT, DC, DE, FL, GA, HI, IA, ID, IL, IN, KY, LA, MA, MD, ME, MI, MN, MO, MS, NC, NE, NH, NJ, NV, NY, OH, OK, OR, PA, RI, SC, SD, TN, TX, UT, VA, VT, WA, WI

Contact: Multiple contacts: See www.clinicaltrials.gov for complete list of contacts.

NCT03233204
NCI-COG Pediatric MATCH (Molecular Analysis for Therapy Choice)- Phase 2 Subprotocol of Olaparib in Patients With Tumors Harboring Defects in DNA Damage Repair Genes

Cancer type: Unspecified Solid Tumor

Variant class: DNA repair pathway

Other identifiers: 20170841, APEC1621H, NCI-2017-00766

Population segments: Aggressive, Indolent, Pediatric or Adolescent, Second line, Stage III, Stage IV

Phase: II

Therapy: olaparib

Locations: Puerto Rico, United States

US States: AK, AL, AR, AZ, CA, CO, DC, DE, FL, GA, IA, ID, IL, IN, KY, LA, MD, ME, MI, MN, MO, MS, NC, ND, NE, NJ, NY, OH, OK, OR, PA, SC, SD, TN, TX, UT, VA, VT, WA, WI

Contact: Multiple contacts: See www.clinicaltrials.gov for complete list of contacts.

NCT04267939
An Open-label Phase Ib Study to Determine the Maximum Tolerated and/or Recommended Phase II Dose of the ATR Inhibitor BAY 1895344 in Combination With PARP Inhibitor Niraparib, in Patients With Recurrent Advanced Solid Tumors and Ovarian Cancer

Cancer type: Ovarian Cancer, Unspecified Solid Tumor

Variant class: DNA repair pathway

Other identifiers: 18595, 19-810, 2019-0471, 20-222, EudraCT Number: 2018-003930-34, NCI-2020-01405

Population segments: Maintenance/Consolidation, Second line, Stage III, Stage IV

Exclusion criteria variant class: DNA repair pathway (Prostate Cancer)

Phase: I

Therapies: BAY-1895344, niraparib

Location: United States

US States: MA, NY, TX

Contact: Bayer Clinical Trials Contact [888-842-2937; clinical-trials-contact@bayer.com]

Disclaimer: The data presented here is from a curated knowledgebase of publicly available information, but may not be exhaustive. The data version is 2021.11(003).

BRCA1 splice site mutation (continued)

NCT04693468
Modular Phase IB Hypothesis-Testing, Biomarker-Driven, Talazoparib Combination Trial (TalaCom)

Cancer type: Unspecified Solid Tumor

Variant class: DNA repair pathway

Other identifiers: 2020-0436, NCI-2020-06041, TalaCom

Population segments: Hormone refractory, Second line, Stage III, Stage IV

Phase: I

Therapies: talazoparib, palbociclib, axitinib, crizotinib

Location: United States

US State: TX

Contact: Timothy A. Yap [713-563-1784; tyap@mdanderson.org]

NCT03810105
A Phase II Study of Olaparib and Durvalumab in Men With Castration Sensitive Biochemically Recurrent Non-Metastatic Prostate Cancer Harboring Mutations in DNA Damage Repair

Cancer type: Prostate Cancer

Variant class: BRCA1 splice site mutation or DNA repair splice site mutation

Other identifiers: 18-480, NCI-2019-01627

Population segments: Adjuvant, Stage I, Stage II, Stage III

Phase: II

Therapies: durvalumab, olaparib

Location: United States

US States: CA, IL, MI, NJ, NY

Contact: Dr. Karen Autio [646-422-4632; autiok@mskcc.org]

NCT02975934
TRITON3: A Multicenter, Randomized, Open Label Phase III Study of Rucaparib Versus Physician's Choice of Therapy for Patients With Metastatic Castration Resistant Prostate Cancer Associated With Homologous Recombination Deficiency

Cancer type: Castration-Resistant Prostate Cancer

Variant class: BRCA1 mutation

Other identifiers: 16238, 1723GCCC, 18-146, 18-300, 20171185, 2018IS040, CO-338-063, CSET 2620, EudraCT Number: 2016-003163-20, GU 146, IGUP17020, IRAS ID: 222298, NCI-2017-01110, S16-02260, STAR Trial, TRITON 3_CO-338-063, TRITON3

Population segments: BRCA, First line, Hormone refractory, Stage IV

Phase: III

Therapies: rucaparib, hormone therapy

Locations: Australia, Belgium, Canada, Denmark, France, Germany, Ireland, Israel, Italy, Spain, United Kingdom, United States

US States: AZ, CA, CO, CT, DE, FL, HI, MD, MN, NC, NY, OH, OR, TN, TX, VA, WA

Contact: Clovis Oncology For North America [844-258-7662; medinfo@clovisoncology.com]

Disclaimer: The data presented here is from a curated knowledgebase of publicly available information, but may not be exhaustive. The data version is 2021.11(003).

BRCA1 splice site mutation (continued)

NCT04336943
Durvalumab (MEDI4736) and Olaparib (AZD2281) for Treatment of Biochemically Recurrent Prostate Cancer in Men Predicted to Have a High Neoantigen Load: A Pilot Study

Cancer type: Prostate Adenocarcinoma

Variant class: BRCA1 mutation

Other identifiers: 10509, NCI-2020-01860, RG1007001

Population segments: Line of therapy N/A, Stage I, Stage II, Stage III

Phase: II

Therapies: durvalumab, olaparib

Location: United States

US State: WA

Contact: Michael Schweizer [206-606-6252; schweize@uw.edu]

NCT04053322
An International Multicenter Phase II Trial of Durvalumab (MEDI4736) Plus OLAparib Plus Fulvestrant in Metastatic or Locally Advanced ER-positive, HER2-negative Breast Cancer Patients Selected Using Criteria That Predict Sensitivity to Olaparib

Cancer type: Breast Cancer

Variant class: BRCA1 mutation

Other inclusion criteria: ERBB2 negative, ER positive

Other identifiers: CADUSEIME08, DOLAF, EudraCT Number: 2018-003832-57, UC-0140/1812

Population segments: BRCA, Estrogen receptor positive, FGFR, HER2 negative, Progesterone receptor positive, Second line, Stage III, Stage IV

Phase: II

Therapies: durvalumab, olaparib, hormone therapy

Location: France

NCT03012321
BRCAAway: A Randomized Phase II Trial of Abiraterone, Olaparib, or Abiraterone + Olaparib in Patients With Metastatic Castration-Resistant Prostate Cancer With DNA Repair Defects

Cancer type: Castration-Resistant Prostate Cancer

Variant class: BRCA1 mutation

Other identifiers: 19137, BRCAAway, NCI-2016-01834, NU 16U05, NU_16U05, PCCTC #: c16-168, PCCTC c16-168, STU00203960

Population segments: First line, Hormone refractory, Stage IV

Phase: II

Therapies: hormone therapy, olaparib, steroid

Location: United States

US States: CA, FL, IL, IN, MD, MI, MN, MO, NC, NY, UT, VA

Contact: Study Coordinator [312-695-1301; cancertrials@northwestern.edu]

Disclaimer: The data presented here is from a curated knowledgebase of publicly available information, but may not be exhaustive. The data version is 2021.11(003).

BRCA1 splice site mutation (continued)

No NCT ID - see other identifier(s)
Phase II Study To Evaluate The Efficacy And Safety Of Niraparib In Recurrent Or Persistent Rare Gynecologic Malignancies With Homologous Recombination Deficiency (JGOG2052)

Cancer type: Cervical Cancer, Endometrial Carcinoma, Ovarian Cancer, Uterine Leiomyosarcoma

Variant class: BRCA1 mutation

Other identifiers: (JGOG2052), jRCT2031210264

Population segments: BRCA, Second line, Stage III, Stage IV

Phase: II

Therapy: niraparib

Location: Japan

NCT03553004
Niraparib in Metastatic Pancreatic Cancer After Previous Chemotherapy (NIRA-PANC): A Phase II Trial

Cancer type: Pancreatic Cancer

Variant class: BRCA1 mutation

Other identifiers: IIT-2017-NIRA-PANC, NIRA-PANC, STUDY00141862

Population segments: BRCA, Second line, Stage IV, Third line

Phase: II

Therapy: niraparib

Location: United States

US States: KS, MO

Contact: Clinical Trials Nurse Navigator [913-588-3671; navigation@kumc.edu]

NCT03601923
Phase II Proof-of-Concept Trial Testing the PARP Inhibitor Niraparib in Patients With Pancreatic Cancer Harboring Deficiencies in Homologous Recombination DNA Repair

Cancer type: Pancreatic Cancer

Variant class: BRCA1 mutation

Other identifiers: 18-207, NCI-2018-02395

Population segments: Second line, Stage IV

Phase: II

Therapy: niraparib

Location: United States

US State: MA

Contact: Dr. James Clreary [617-632-5960; JCLEARY@PARTNERS.ORG]

NCT03840967
A Phase II Study Evaluating Safety and Efficacy of Niraparib in Patients With Previously Treated Homologous Recombination (HR) Defective or Loss of Heterozygosity (LOH) High Metastatic Esophageal/Gastroesophageal Junction/Proximal Gastric Adenocarcinoma

Cancer type: Esophageal Cancer, Gastric Cancer, Gastroesophageal Junction Adenocarcinoma

Variant class: BRCA1 mutation

Other identifiers: 20166, 71618, HCRN ESO17-325, NCI-2019-07395

Population segments: Second line, Stage III, Stage IV

Phase: II

Therapy: niraparib

Location: United States

US States: FL, IN, MI, NY

Contact: Dr. Shadia Jalal [317-274-3658; sjalal@iu.edu]

Disclaimer: The data presented here is from a curated knowledgebase of publicly available information, but may not be exhaustive. The data version is 2021.11(003).

BRCA1 splice site mutation (continued)

NCT03925350

A Phase II Study of Niraparib in Patients With Advanced Melanoma With Genetic Homologous Recombination (HR) Mutation / Alteration

Cancer type: Melanoma

Variant class: BRCA1 mutation

Other identifier: CPMC17-MEL01

Population segments: Second line, Stage III, Stage IV

Phase: II

Therapy: niraparib

Location: United States

US State: CA

Contact: Laurel Brechtel [415-600-1654; BrechtLA@sutterhealth.org]

NCT04493060

Phase II Study of Niraparib and TSR-042 in Patients With Germline or Somatic BRCA1/2 and PALB2-Related Pancreatic Cancer

Cancer type: Pancreatic Cancer

Variant class: BRCA1 mutation

Other identifiers: MC1841, NCI-2020-05352

Population segments: BRCA, Second line, Stage IV, Third line

Phase: II

Therapies: niraparib, dostarlimab

Location: United States

US States: AZ, FL, MN

Contact: Multiple contacts: See www.clinicaltrials.gov for complete list of contacts.

NCT04187833

Phase II Trial of Nivolumab in Combination with Talazoparib in Patients with Unresectable or Metastatic Melanoma and Mutations in BRCA or BRCA-ness Genes.

Cancer type: Melanoma

Variant class: BRCA1 mutation

Other identifiers: CASE2619, NCI-2020-00416

Population segments: Second line, Stage III, Stage IV

Phase: II

Therapies: nivolumab, talazoparib

Location: United States

US State: OH

Contact: Dr. Pauline Funchain [866-223-8100; TaussigResearch@ccf.org]

Disclaimer: The data presented here is from a curated knowledgebase of publicly available information, but may not be exhaustive. The data version is 2021.11(003).

BRCA1 splice site mutation (continued)

NCT03344965
A Phase II Study of Olaparib Monotherapy in Metastatic Breast Cancer Patients With Germline or Somatic Mutations in DNA Repair Genes (Olaparib Expanded)

Cancer type: Breast Cancer

Variant class: BRCA1 mutation

Other identifiers: 17-428, 18-414, NCI-2018-00778, TBCRC 048, TBCRC048

Population segments: BRCA, Estrogen receptor positive, HER2 negative, HER2 positive, Progesterone receptor positive, Second line, Stage IV

Exclusion criteria variant classes: BRCA1 germline mutation (Breast Cancer), BRCA2 germline mutation (Breast Cancer)

Phase: II

Therapy: olaparib

Location: United States

US States: AL, CA, IL, MA, MD, NC, NJ, NY, PA, WA

Contact: Dr. Nadine Tung [617-667-1962; ntung@bidmc.harvard.edu]

NCT03375307
A Phase II Study of Olaparib (AZD2281) in Patients With Metastatic/Advanced Urothelial Carcinoma With DNA-Repair Defects

Cancer type: Bladder Urothelial Carcinoma

Variant class: BRCA1 mutation

Other identifiers: 10144, 19C0023, NCI-2017-02296, P162941

Population segments: BRCA, STK11, Second line, Stage III, Stage IV

Phase: II

Therapy: olaparib

Location: United States

US States: CA, CO, KY, MD, NY, OK

Contact: Multiple contacts: See www.clinicaltrials.gov for complete list of contacts.

NCT03742895
A Phase II Study of Olaparib Monotherapy in Participants With Previously Treated, Homologous Recombination Repair Mutation (HRRm) or Homologous Recombination Deficiency (HRD) Positive Advanced Cancer

Cancer type: Breast Cancer

Variant class: BRCA1 mutation

Other inclusion criteria: BRCA1 germline mutation negative, BRCA2 germline mutation negative

Other identifiers: (MK-7339-002 / LYNK-002), 19-139, 194694, 2018-01891, 7339-002, 7339-002-00, EudraCT Number: 2018-003007-19, IRAS ID: 255158, JapicCTI-194694, LYNK002, LYNK-002, MK7339-002, MK-7339-002, MK7339-002-00, NCI-2018-03519, PER-046-18, SNCTP000003157

Population segments: BRCA, Fourth line or greater, Second line, Stage III, Stage IV, Third line

Exclusion criteria variant classes: BRCA germline mutation (Breast Cancer, Ovarian Cancer), BRCA mutation (Ovarian Cancer)

Phase: II

Therapy: olaparib

Locations: Argentina, Australia, Canada, Colombia, Denmark, France, Guatemala, Ireland, Israel, Italy, Mexico, Peru, Republic of Korea, Romania, Russian Federation, Spain, Switzerland, Turkey, United Kingdom, United States

US States: AZ, CA, CO, GA, KY, MA, MD, MI, NE, NJ, NY, SD, UT, WA

Contact: Toll Free Number [888-577-8839; Trialsites@merck.com]

Disclaimer: The data presented here is from a curated knowledgebase of publicly available information, but may not be exhaustive. The data version is 2021.11(003).

BRCA1 splice site mutation (continued)

NCT03786796
Phase II Study of Olaparib in Metastatic Renal Cell Carcinoma Patients Harboring a BAP-1 or Other DNA Repair Gene Mutations (ORCHID)

Cancer type: Kidney Cancer

Variant class: BRCA1 mutation

Other identifiers: CRMS-70750, J18166, NCI-2019-06309, ORCHID

Population segments: Fourth line or greater, Second line, Stage IV, Third line

Phase: II

Therapy: olaparib

Location: United States

US State: MD

Contact: Irina Rifkind [410-502-2043; irifkin1@jhmi.edu]

NCT04042831
A Phase II Study of Olaparib in Patients With Advanced Biliary Tract Cancer With Aberrant DNA Repair Gene Mutations

Cancer type: Biliary Tract Carcinoma

Variant class: BRCA1 mutation

Other identifiers: ACCRU-ICRN-1702, NCI-2019-04434

Population segments: Second line, Stage IV

Phase: II

Therapy: olaparib

Location: United States

US States: AZ, MN, NY, TX

Contact: Multiple contacts: See www.clinicaltrials.gov for complete list of contacts.

NCT04515836
Phase II Trial of Olaparib in Homologous Recombination Deficient (HRD) Malignant Mesothelioma

Cancer type: Mesothelioma

Variant class: BRCA1 mutation

Population segments: (N/A), Second line

Phase: II

Therapy: olaparib

Location: United States

US State: IL

Contact: Dr. Hedy L. Kindler [773-702-0360; hkindler@medicine.bsd.uchicago.edu]

NCT04858334
APOLLO: A Randomized Phase II Double-Blind Study of Olaparib Versus Placebo Following Adjuvant Chemotherapy in Patients With Resected Pancreatic Cancer and a Pathogenic BRCA1, BRCA2 or PALB2 Mutation

Cancer type: Pancreatic Cancer

Variant class: BRCA1 mutation

Other identifiers: APOLLO, CTSU/EA2192, EA2192, NCI-2020-05659

Population segments: Adjuvant, BRCA, Maintenance/Consolidation, Stage 0, Stage I, Stage II

Phase: II

Therapy: olaparib

Location: United States

US States: AZ, FL, IA, IL, IN, KS, MI, MO, NC, NJ, NM, NV, NY, OH, OR, PA, WA, WI

Contact: Multiple contacts: See www.clinicaltrials.gov for complete list of contacts.

Disclaimer: The data presented here is from a curated knowledgebase of publicly available information, but may not be exhaustive. The data version is 2021.11(003).

BRCA1 splice site mutation (continued)

NCT02849496

A Phase II Open-Label, Randomized Study of PARP Inhibition (Olaparib) Either Alone or in Combination With Anti-PD-L1 Therapy (Atezolizumab; MPDL3280A) in Homologous DNA Repair (HDR) Deficient, Locally Advanced or Metastatic Non-HER2-Positive Breast Cancer.

Cancer type: Breast Cancer

Variant class: BRCA1 mutation

Other inclusion criteria: ERBB2 negative

Other identifiers: 10020, 109587 (10020), 1608018258, 17-709, 18979, 5UM1CA186709-04, Clinical Trial 18979, NCI-2016-01130, OSU-17011, PHII-146

Population segments: BRCA, HER2 negative, Second line, Stage III, Stage IV

Phase: II

Therapies: olaparib, atezolizumab

Location: United States

US States: CA, CO, CT, FL, IL, MA, MD, MI, MO, NC, NY, OH, PA, TN, UT

Contact: Multiple contacts: See www.clinicaltrials.gov for complete list of contacts.

NCT04939662

Phase II, Single-arm Study of Olaparib and Bevacizumab Combination Therapy in Relapsed Small Cell Lung Cancer Subjects With DNA Damage Response and the Repair Pathway Alteration, ATM Deficiency, SLFN11 Positive, or POU2F3 Positive

Cancer type: Small Cell Lung Cancer

Variant class: BRCA1 mutation

Other identifier: SUKSES-B2

Population segments: (N/A), BRCA, Pulmonary, Second line, Third line

Phase: II

Therapies: olaparib, bevacizumab

Location: Republic of Korea

NCT04261465

The NUVOLA TRIAL: Neoadjuvant Chemotherapy in Unresectable Ovarian Cancer With OLAparib and Weekly Carboplatin Plus Paclitaxel: A Phase II Open-label Multicentre Study

Cancer type: Ovarian Cancer

Variant class: BRCA1 mutation

Other identifiers: 2317, 2345, EudraCT Number: 2018-002687-65, NUVOLA, NUVOLA trial

Population segments: BRCA, Maintenance/Consolidation, Neoadjuvant, Stage III, Stage IV

Phase: II

Therapies: olaparib, chemotherapy

Location: Italy

NCT04633902

Phase II Study of Olaparib in Combination With Pembrolizumab in Patients With Advanced Melanoma With Homologous Recombination (HR) Pathway Gene Mutation

Cancer type: Melanoma

Variant class: BRCA1 mutation

Other identifier: CPMC19-MEL01

Population segments: BRAF, Second line, Stage III, Stage IV

Phase: II

Therapies: olaparib, pembrolizumab

Location: United States

US State: CA

Contact: Dr. Kevin B. Kim [415-885-8600; ClinicalResearch@sutterhealth.org]

Disclaimer: The data presented here is from a curated knowledgebase of publicly available information, but may not be exhaustive. The data version is 2021.11(003).

BRCA1 splice site mutation (continued)

NCT03248570
Phase II Open Label Study of Pembrolizumab in Patients With Metastatic Castration Resistant Prostate Cancer (mCRPC) With or Without DNA Damage Repair Defects

Cancer type: Castration-Resistant Prostate Cancer

Variant class: BRCA1 mutation

Other identifiers: 16557, 17-22165, CC 16557, NCI-2017-02408

Population segments: BRCA, Hormone refractory, Line of therapy N/A, Stage IV

Phase: II

Therapy: pembrolizumab

Location: United States

US State: CA

Contact: Patricia Li [415-476-5975; GUTrials@ucsf.edu]

NCT03506997
PERSEUS1: Phase II Trial of the Immune Checkpoint Inhibitor Pembrolizumab for Patients Suffering From Metastatic Prostate Cancer

Cancer type: Castration-Resistant Prostate Cancer

Variant class: BRCA1 mutation

Other identifiers: CCR4559, EudraCT Number: 2017-000931-15, ICR-CTSU/2016/10060, IRAS ID 208952, PERSEUS1

Population segments: Hormone refractory, Second line, Stage IV

Phase: II

Therapy: pembrolizumab

Location: United Kingdom

NCT04089189
A Phase II Study to Evaluate the Efficacy, Safety, and Tolerability of IMP4297 Capsules in Subjects With Germline and/or Systemic BRCA1/2 Mutation Positive Advanced Ovarian Cancer With at Least 2 Prior Lines of Standard Systemic Therapy

Cancer type: Ovarian Cancer

Variant class: BRCA1 mutation

Other identifiers: CTR20190652, IMP4297-2018-CN01

Population segments: Stage III, Stage IV, Third line

Phase: II

Therapy: senaparib

Location: China

NCT03990896
Evaluation of Talazoparib, a PARP Inhibitor, in Patients With Somatic BRCA Mutant Metastatic Breast Cancer: Genotyping Based Clinical Trial

Cancer type: Breast Cancer, Triple Negative Breast Cancer

Variant class: BRCA1 mutation

Other inclusion criteria: ERBB2 negative, Hormone receptor positive

Other identifiers: 19-188, NCI-2019-07732

Population segments: BRCA, Estrogen receptor positive, Fourth line or greater, HER2 negative, Progesterone receptor positive, Second line, Stage IV, Third line, Triple receptor negative

Exclusion criteria variant classes: BRCA1 germline mutation (Breast Cancer, Triple Negative Breast Cancer), BRCA2 germline mutation (Breast Cancer, Triple Negative Breast Cancer)

Phase: II

Therapy: talazoparib

Location: United States

US States: MA, TX

Contact: Dr. Neelima Vidula [617-724-4000; nvidula@mgh.harvard.edu]

BRCA1 splice site mutation (continued)

NCT04892693
Talazoparib in Advanced Breast Cancer Patients With Homologous Recombinant Deficiency: A Phase II Clinical and Exploratory Biomarker Study of Talazoparib

Cancer type: Breast Cancer

Variant class: BRCA1 mutation

Other identifier: H-2009-079-1157

Population segments: BRCA, Line of therapy N/A, Stage III, Stage IV

Phase: II

Therapy: talazoparib

Location: Republic of Korea

NCT04740190
Combination Talazoparib - Carboplatin for Recurrent High-grade Glioma With DNA Damage Repair Deficiency (DDRd)

Cancer type: Anaplastic Astrocytoma, Anaplastic Oligoastrocytoma, Anaplastic Oligodendroglioma, Anaplastic Pleomorphic Xanthoastrocytoma, Diffuse Midline Glioma, Glioblastoma

Variant class: BRCA1 mutation

Other identifiers: TAC-GReD, UW-20396

Population segments: (N/A), BRCA, Second line

Phase: II

Therapies: talazoparib, chemotherapy

Location: Hong Kong

NCT04253262
A Phase Ib/II Study of Copanlisib Combined With Rucaparib in Patients With Metastatic Castration-resistant Prostate Cancer

Cancer type: Castration-Resistant Prostate Cancer

Variant class: BRCA1 mutation

Other identifiers: BrUOG 360, BrUOG360

Population segments: Hormone refractory, Second line, Stage IV

Phase: I/II

Therapies: copanlisib, rucaparib

Location: United States

US State: RI

Contact: BrUOG [401-863-3000; BrUOG@brown.edu]

NCT03685331
Harnessing Olaparib, Palbociclib and Endocrine Therapy: A Phase I/II Trial of Olaparib, Palbociclib and Fulvestrant in Patients With BRCA Mutation-associated, Hormone Receptor-positive, Human Epidermal Growth Factor Receptor 2 (HER2)-Negative Metastatic Breast Cancer (HOPE)

Cancer type: Breast Cancer

Variant class: BRCA1 mutation

Other inclusion criteria: ERBB2 negative, Hormone receptor positive

Other identifiers: (HOPE), NCI-2019-04210, UPCC 21118

Population segments: BRCA, Estrogen receptor positive, First line, HER2 negative, Progesterone receptor positive, Second line, Stage III, Stage IV, Third line

Phase: I/II

Therapies: olaparib, palbociclib, hormone therapy

Location: United States

US State: PA

Contact: Alexandra Torres [855-216-0098; PennCancerTrials@emergingmed.com]

Disclaimer: The data presented here is from a curated knowledgebase of publicly available information, but may not be exhaustive. The data version is 2021.11(003).

BRCA1 splice site mutation (continued)

No NCT ID - see other identifier(s)
An Open-Label, Randomised, Phase I/II Trial Of Rucaparib Combined With Nivolumab + or - Ipilimumab To Augment Response In Homologous Repair Deficient Patients With Relapsed Ovarian, Primary Peritoneal And Fallopian Tube Cancer

Cancer type: Ovarian Cancer

Variant class: BRCA1 mutation

Other identifiers: CeNturIOn, CeNturIOn-2016, EudraCT Number: 2017-004780-13, ISRCTN10490346

Population segments: (N/A), BRCA, Fourth line or greater, Second line, Third line

Phase: I/II

Therapies: rucaparib, nivolumab, ipilimumab

Location: United Kingdom

NCT03964532
TALAVE: A Pilot Trial of Induction Talazoparib Followed by Combination of Talazoparib and Avelumab in Advanced Breast Cancer

Cancer type: Breast Cancer

Variant class: BRCA1 mutation

Other inclusion criteria: ERBB2 negative

Other identifiers: NCI-2019-06240, STUDY00000023, TALAVE

Population segments: Line of therapy N/A, Stage III, Stage IV

Phase: I/II

Therapies: talazoparib, avelumab

Location: United States

US States: DC, NC, UT

Contact: Dr. Julie Collins [202-444-2223; Julie.Collins@gunet.georgetown.edu]

NCT04673448
Phase IB Trial of Niraparib and TSR-042 in Patients With BRCA-Mutated Breast, Pancreas or Ovary Cancer

Cancer type: Breast Cancer, Ovarian Cancer, Pancreatic Cancer

Variant class: BRCA1 mutation

Other identifiers: 10020, NCI-2020-11636, RG1005140

Population segments: BRCA, Estrogen receptor positive, HER2 positive, Progesterone receptor positive, Second line, Stage III, Stage IV

Phase: I

Therapies: niraparib, dostarlimab

Location: United States

US State: WA

Contact: Elizabeth M. Swisher [206-543-3669; swishere@uw.edu]

NCT04890613
Phase Ib Expansion Study of CX-5461 in Patients With Solid Tumours and BRCA2 and/or PALB2 Mutation

Cancer type: Ovarian Cancer

Variant class: BRCA1 mutation

Other identifier: CX-5461-04

Population segments: BRCA, Second line, Stage II, Stage III, Stage IV

Phase: I

Therapy: pidnarulex

Location: Canada

Disclaimer: The data presented here is from a curated knowledgebase of publicly available information, but may not be exhaustive. The data version is 2021.11(003).

BRCA1 splice site mutation (continued)

NCT04598321

BrUOG 390: Neoadjuvant Treatment With Talazoparib for Women With Newly Diagnosed, Advanced Ovarian Cancer Associated With a Mutation in BRCA1 or BRCA2 (mBRCA)

Cancer type: Ovarian Cancer

Variant class: BRCA1 mutation

Other identifier: BrUOG 390

Population segments: BRCA, Maintenance/Consolidation, Neoadjuvant, Stage II, Stage III, Stage IV

Phase: I

Therapy: talazoparib

Location: United States

US State: RI

Contact: BrUOG [401-863-3000; BrUOG@Brown.edu]

NCT03598270

A Phase III Randomized, Double-blinded Trial of Platinum-based Chemotherapy With or Without Atezolizumab Followed by Niraparib Maintenance With or Without Atezolizumab in Patients With Recurrent Ovarian, Tubal or Peritoneal Cancer and Platinum Treatment-free Interval (TFIp) >6 Months

Cancer type: Ovarian Cancer

Variant class: BRCA mutation

Other identifiers: AGO ovary 2.33, ANITA, BGOG-ov-41, ENGOT-ov41, ENGOT-OV41/GEICO 69-O/ANITA, ENGOT-ov41/GEICO/ANITA, ENGOT-Ov41/GEICO69-O/ANITA, EudraCT Number: 2018-000366-11, GEICO 69-O, GEICO-69-O

Population segments: (N/A), BRCA, Maintenance/Consolidation, Second line, Third line

Phase: III

Therapies: atezolizumab, chemotherapy, niraparib

Locations: Belgium, France, Germany, Italy, Spain

NCT03278717

International Phase III Randomised Study to Evaluate the Efficacy of Maintenance Therapy With Olaparib and Cediranib or Olaparib Alone in Patients With Relapsed Ovarian Cancer Following a Response to Platinum-based Chemotherapy

Cancer type: Ovarian Cancer

Variant class: BRCA mutation

Other identifiers: 36280, ACTRN12618000498291, ANZGOG 1415/2018, CRUK/15/074, CTC 0133, ENGOT-ov35, ENGOT-ov35/NCRI/ICON 9, EudraCT Number: 2017-000161-75, ICON9, NC174, OV26 (CRUK/UCL ICON9), UCL/14/0795

Population segments: BRCA, Maintenance/Consolidation, Second line, Stage III, Stage IV

Phase: III

Therapies: cediranib, olaparib

Locations: Australia, Canada, New Zealand, United Kingdom

Disclaimer: The data presented here is from a curated knowledgebase of publicly available information, but may not be exhaustive. The data version is 2021.11(003).

BRCA1 splice site mutation (continued)

NCT04915755
A Randomized Phase III Double-Blinded Study Comparing the Efficacy and Safety of Niraparib to Placebo in Participants With Either HER2-Negative BRCA-Mutated or Triple-Negative Breast Cancer With Molecular Disease Based on Presence of Circulating Tumor DNA After Definitive Therapy (ZEST)

Cancer type: Breast Cancer

Variant class: BRCA mutation

Other inclusion criteria: ERBB2 negative

Other identifiers: 213831, EudraCT Number: 2020-003973-23, GSK 213831 ZEST, SNCTP000004543, ZEST

Population segments: BRCA, Estrogen receptor positive, HER2 negative, Progesterone receptor positive, Second line, Stage I, Stage II, Stage III, Triple receptor negative

Phase: III

Therapy: niraparib

Locations: Canada, France, Ireland, Italy, Netherlands, Norway, Poland, Russian Federation, Spain, United Kingdom, United States

US State: IL

Contact: US GSK Clinical Trials Call Center [877-379-3718; GSKClinicalSupportHD@gsk.com]

NCT03462342
Combination ATR and PARP Inhibitor (CAPRI) Trial With AZD 6738 and Olaparib in Recurrent Ovarian Cancer

Cancer type: Ovarian Cancer

Variant class: BRCA mutation

Other identifiers: 20-320, CAPRI, ESR-16-12311, NCI-2018-02375, UPCC 15817

Population segments: BRCA, Fourth line or greater, Second line, Stage III, Stage IV, Third line

Phase: II

Therapies: ceralasertib, olaparib

Location: United States

US States: MA, MD, PA

Contact: Diego Rodriguez [215-614-0234; diegorod@pennmedicine.upenn.edu]

No NCT ID - see other identifier(s)
An Investigator-Initiated Phase II Trial Of Niraparib For Patients With BRCA-Mutated Unresectable / Recurrent Biliary Tract, Pancreatic And Other Gastrointestinal Cancer (NIR-B)

Cancer type: Cholangiocarcinoma, Colon Cancer, Gastrointestinal Stromal Tumor, Pancreatic Cancer, Rectal Cancer

Variant class: BRCA mutation

Other identifiers: jRCT2011200023, NIR-B

Population segments: BRCA, Second line, Stage III, Stage IV, Third line

Phase: II

Therapy: niraparib

Location: Japan

NCT04716686
Niraparib Monotherapy as Maintain and Recurrent Treatment of Endometrial Serous Carcinoma: A Multi-center, Open-label, Prospective Clinical Study

Cancer type: Endometrial Serous Adenocarcinoma

Variant class: BRCA mutation

Other identifier: ZL-2306-914

Population segments: BRCA, Fourth line or greater, Maintenance/Consolidation, Second line, Stage III, Stage IV, Third line

Phase: II

Therapy: niraparib

Location: China

Disclaimer: The data presented here is from a curated knowledgebase of publicly available information, but may not be exhaustive. The data version is 2021.11(003).

BRCA1 splice site mutation (continued)

NCT04348045
MAZEPPA: Phase II PRODIGE-GERCOR Study to Evaluate MAintenance Therapy With Olaparib or Selumetinib Plus Durvalumab According to BRCAness and KRAS Somatic Status Personalized in Metastatic Pancreatic Adenocarcinoma Patients

Cancer type: Pancreatic Cancer

Variant class: BRCA mutation

Other inclusion criteria: DPYD wild type

Other identifiers: D19-02, EudraCT Number: 2019-004366-18, MAZEPPA D19-02, MAZEPPA D19-02 PRODIGE 72, MAZEPPA_D19-02, PRODIGY 72 - MAZEPPA

Population segments: BRCA, KRAS, Maintenance/Consolidation, Stage IV

Phase: II

Therapy: olaparib

Location: France

NCT03025035
Open Label, Phase II Pilot Study of Immune Checkpoint Inhibition With Pembrolizumab in Combination With PARP Inhibition With Olaparib in Advanced BRCA-mutated or HDR-defect Breast Cancers

Cancer type: Breast Cancer

Variant class: BRCA mutation

Other identifier: IIT2015-18-Mita-MK3475

Population segments: BRCA, Estrogen receptor positive, HER2 negative, HER2 positive, Progesterone receptor positive, Second line, Stage III, Stage IV, Triple receptor negative

Phase: II

Therapies: pembrolizumab, olaparib

Location: United States

US State: CA

Contact: Parisa Mirzadehgan [310-967-4387; Parisa.Mirzadehgan@cshs.org]

NCT04666740
A Phase II Trial to Evaluate the Safety and Antitumor Activity of Pembrolizumab and OLApaRib (POLAR) Maintenance for Patients With Metastatic Pancreatic Ductal Adenocarcinoma and Homologous Recombination Deficiency and/or Exceptional Treatment Response to Platinum-Based Therapy

Cancer type: Pancreatic Cancer

Variant class: BRCA1 aberration

Other identifier: 20-481

Population segments: BRCA, Maintenance/Consolidation, Stage IV

Phase: II

Therapies: pembrolizumab, olaparib

Location: United States

US States: NJ, NY

Contact: Dr. Wungki Park [646-888-4543; yaqubiea@mskcc.org]

Disclaimer: The data presented here is from a curated knowledgebase of publicly available information, but may not be exhaustive. The data version is 2021.11(003).

BRCA1 splice site mutation (continued)

NCT02264678

A Modular Phase I, Open-Label, Multicentre Study to Assess the Safety, Tolerability, Pharmacokinetics and Preliminary Anti-tumour Activity of Ceralasertib in Combination With Cytotoxic Chemotherapy and/or DNA Damage Repair/Novel Anti-cancer Agents in Patients With Advanced Solid Malignancies

Cancer type: Breast Cancer, Ovarian Cancer

Variant class: BRCA mutation

Other inclusion criteria: ERBB2 negative

Other identifiers: 0C-14-9, 16-031, 18-342, D5330C00004, EudraCT Number: 2014-002233-66, IRAS ID 160200, NCI-2015-00687, Study 4

Population segments: Adenocarcinoma, BRCA, HER2 negative, Pulmonary, Second line, Stage III, Stage IV, Third line, Triple receptor negative

Exclusion criteria variant class: ERBB2 positive (Breast Cancer)

Phase: I

Therapies: ceralasertib, olaparib

Locations: France, Republic of Korea, United Kingdom, United States

US States: CA, MA, NY

Contact: AstraZeneca Clinical Study Information Center [877-240-9479; information.center@astrazeneca.com]

NCT04169841

Precision Medicine Phase II Study Evaluating the Efficacy of a Double Immunotherapy by Durvalumab and Tremelimumab Combined With Olaparib in Patients With Solid Cancers and Carriers of Homologous Recombination Repair Genes Mutation in Response or Stable After Olaparib Treatment

Cancer type: Breast Cancer, Endometrial Carcinoma, Head and Neck Cancer, Kidney Cancer, Non-Small Cell Lung Cancer, Ovarian Cancer, Pancreatic Cancer, Prostate Cancer

Variant class: HRR mutation

Other identifier: GUIDE2REPAIR

Population segments: Fourth line or greater, Hormone refractory, Maintenance/ Consolidation, Second line, Stage III, Stage IV, Third line

Exclusion criteria variant classes: ALK fusion (Lung Cancer), BRAF mutation (Lung Cancer), EGFR mutation (Lung Cancer), ROS1 mutation (Lung Cancer)

Phase: II

Therapies: durvalumab, tremelimumab, olaparib

Location: France

NCT04030559

Phase II Trial of PARP Inhibitor Niraparib for Men With High Risk Prostate Cancer and DNA Damage Response Defects

Cancer type: Prostate Cancer

Variant class: HRR mutation

Other identifiers: 1412676, NCI-2019-04365, UCDCC#279

Population segments: First line, Neoadjuvant, Stage I, Stage II, Stage IV

Phase: II

Therapy: niraparib

Location: United States

US State: CA

Contact: Marc Dall'Era [916-734-3771; mdallera@ucdavis.edu]

Disclaimer: The data presented here is from a curated knowledgebase of publicly available information, but may not be exhaustive. The data version is 2021.11(003).

BRCA1 splice site mutation (continued)

NCT04940637

A Phase II, Open-Label, Single Arm, Prospective, Multicenter Study of Niraparib Plus Dostarlimab in Patients With Advanced NSCLC and/or MPM, and Positive for PD-L1 Expression and Germline or Somatic Mutations in the HRR Genes

Cancer type: Mesothelioma, Non-Small Cell Lung Cancer

Variant class: HRR mutation

Other inclusion criteria: ALK fusion negative, CD274 expression, EGFRi sensitizing mutation negative, ROS1 fusion negative or CD274 expression

Other identifiers: EudraCT Number: 2020-000109-10, UNITO-001, UNITO-001/2020

Population segments: Second line, Stage III, Stage IV

Exclusion criteria variant classes: ALK fusion (Non-Small Cell Lung Cancer), EGFRi sensitizing mutation (Non-Small Cell Lung Cancer), ROS1 fusion (Non-Small Cell Lung Cancer)

Phase: II

Therapies: niraparib, dostarlimab

Location: Italy

NCT05002868

A Multi-center, Open-label, Phase I/Ib Study to Assess the Safety, Pharmacokinetics and Anti-tumor Activity of RP12146, a Poly (ADP-ribose) Polymerase (PARP) Inhibitor, in Patients With Locally Advanced or Metastatic Solid Tumors

Cancer type: Biliary Tract Carcinoma, Breast Cancer, Colorectal Cancer, Endometrial Carcinoma, Gastric Cancer, Ovarian Cancer, Pancreatic Cancer, Prostate Cancer, Small Cell Lung Cancer

Variant class: HRR mutation

Other identifier: RP12146-2101

Population segments: Extensive, Pulmonary, Second line, Stage III, Stage IV

Exclusion criteria variant class: ERBB2 positive (Breast Cancer)

Phase: I

Therapy: RP12146

Location: Poland

NCT03395197

A Phase III, Randomized, Double-blind, Placebo-controlled Study Of Talazoparib With Enzalutamide In Metastatic Castration-resistant Prostate Cancer

Cancer type: Castration-Resistant Prostate Cancer

Variant class: DNA repair mutation

Other identifiers: BASIC-ROYAL / HIGH-ROYAL, C3441021, CSET 2938, CTR20191443, CTR20191444, EudraCT Number: 2017-003295-31, GU 170 - C3441021, HCC 19-054, JapicCTI-194665, LH19.022, MOH_2019-05-16_007127, NCI-2018-01651, Pfizer C3441021, R19062M, RECF3951, RWF_C3441021 TALAPRO-2, TALAPRO-2, TX228665, UCI-18-47, VICCURO17115

Population segments: First line, Hormone refractory, Second line, Stage III, Stage IV

Phase: III

Therapies: talazoparib, hormone therapy

Locations: Argentina, Australia, Belgium, Brazil, Canada, Chile, China, Czech Republic, Finland, France, Germany, Hungary, Israel, Italy, Japan, New Zealand, Norway, Peru, Poland, Portugal, Republic of Korea, South Africa, Spain, Sweden, United Kingdom, United States

US States: AZ, CA, CO, FL, GA, IL, IN, KS, MO, NE, NJ, NY, OH, OK, OR, PA, SC, TN, TX, UT, VA, WI

Contact: Pfizer CT.gov Call Center [800-718-1021; ClinicalTrials.gov_Inquiries@pfizer.com]

Disclaimer: The data presented here is from a curated knowledgebase of publicly available information, but may not be exhaustive. The data version is 2021.11(003).

BRCA1 splice site mutation (continued)

NCT04821622
TALAPRO-3: A Phase III, Randomized, Double-blind, Study of Talazoparib With Enzalutamide Versus Placebo With Enzalutamide in Men With DDR Gene Mutated Metastatic Castration-sensitive Prostate Cancer

Cancer type: Prostate Adenocarcinoma

Variant class: DNA repair mutation

Other identifiers: C3441052, CTR20212191, EudraCT Number: 2021-000248-23, jRCT2031210098, TALAPRO-3

Population segments: Second line, Stage IV

Phase: III

Therapies: talazoparib, hormone therapy

Locations: Japan, Republic of Korea, United States

US States: CA, DC, FL, GA, IL, MI, NJ, NY, PA, SC, TX, UT

Contact: Pfizer CT.gov Call Center [800-718-1021; ClinicalTrials.gov_Inquiries@pfizer.com]

NCT04038502
An Open-label, Multicenter Phase II Study to Compare the Efficacy of Carboplatin as First-line Followed by Second-line Olaparib Versus Olaparib as First-line Followed by Second-line Carboplatin in the Treatment of Patients With Castration Resistant Prostate Cancer Containing Homologous Recombination Deficiency

Cancer type: Castration-Resistant Prostate Cancer

Variant class: DNA repair mutation

Other identifiers: 01781, 01783, 19-08, BRACeD, CX001963-01, ONCA-009-18F

Population segments: First line, Hormone refractory, Second line, Stage IV

Phase: II

Therapies: olaparib, chemotherapy

Location: United States

US States: CA, DC, FL, IL, MI, NC, NY, PA, WA

Contact: Dr. Robert B. Montgomery [206-277-6878; rbmontgo@u.washington.edu]

No NCT ID - see other identifier(s)
Single-arm Exploratory Study for Sintilimab Second-line or Later-line Treatment of Advanced Non-small Cell Lung Cancer with DDR Pathway Gene Mutation

Cancer type: Non-Small Cell Lung Cancer

Variant class: DNA repair mutation

Other identifier: ChiCTR1900023234

Population segments: Fourth line or greater, Second line, Stage III, Stage IV, Third line

Exclusion criteria variant classes: ALK fusion (Non-Small Cell Lung Cancer), EGFRi sensitizing mutation (Non-Small Cell Lung Cancer), ROS1 fusion (Non-Small Cell Lung Cancer)

Phase: I/II

Therapy: sintilimab

Location: China

Disclaimer: The data presented here is from a curated knowledgebase of publicly available information, but may not be exhaustive. The data version is 2021.11(003).

BRCA1 splice site mutation (continued)

NCT04095273

A Multicenter, Non-randomized, Open-label Phase Ib Study to Determine the Maximum Tolerated and Recommended Phase II Dose of the ATR Inhibitor BAY1895344 in Combination With Pembrolizumab and to Characterize Its Safety, Tolerability, Pharmacokinetics and Preliminary Anti-tumor Activity in Patients With Advanced Solid Tumors

Cancer type: Breast Cancer, Castration-Resistant Prostate Cancer, Colorectal Cancer, Gastric Cancer, Gastroesophageal Junction Adenocarcinoma, Non-Small Cell Lung Cancer, Pancreatic Cancer

Variant class: DNA repair mutation

Other inclusion criteria: ATM mutation negative or ATM mutation negative, ERBB2 negative, Hormone receptor positive or ATM underexpression

Other identifiers: 19741, EudraCT Number: 2018-003420-36, Keynote 919, NCI-2019-06734

Population segments: HER2 negative, Hormone refractory, Second line, Stage III, Stage IV

Phase: I

Therapies: BAY-1895344, pembrolizumab

Locations: Germany, Spain, Switzerland, United States

US States: CA, CT, MA, MD, NC, NY, OH, TX

Contact: Bayer Clinical Trials Contact [clinical-trials-contact@bayer.com]

NCT04849364

A Phase II Circulating Tumor DNA Enriched, Genomically Directed Post-neoadjuvant Trial for Patients With Residual Triple Negative Breast Cancer (PERSEVERE)

Cancer type: Triple Negative Breast Cancer

Variant class: DNA repair pathway

Other identifiers: HCRN BRE18-334, PERSEVERE

Population segments: Adjuvant, HER2 negative, Stage I, Stage II, Stage III, Triple receptor negative

Phase: II

Therapies: talazoparib, chemotherapy

Location: United States

US State: IN

Contact: Dr. Bryan P. Schneider [317-948-3885; bpschnei@iu.edu]

Disclaimer: The data presented here is from a curated knowledgebase of publicly available information, but may not be exhaustive. The data version is 2021.11(003).

BRCA1 truncating mutation

NCT02029001

A Two-period, Multicenter, Randomized, Open-label, Phase II Study Evaluating the Clinical Benefit of a Maintenance Treatment Targeting Tumor Molecular Alterations in Patients with Progressive Locally-advanced or Metastatic Solid Tumors.

Cancer type: Unspecified Solid Tumor

Variant class: BRCA1 mutation

Other identifiers: ET12-081, EudraCT Number: 2012-004510-34, MOST, ProfiLER

Population segments: Maintenance/Consolidation, Second line, Stage III, Stage IV, Third line

Exclusion criteria variant classes: ATM mutation (Gastric Cancer), BRCA1 germline mutation (Gastric Cancer), BRCA1 mutation (Gastric Cancer), BRCA2 germline mutation (Gastric Cancer), BRCA2 mutation (Gastric Cancer), HRR mutation (Gastric Cancer), PALB2 mutation (Gastric Cancer), RAD51 mutation (Gastric Cancer)

Phase: II

Therapy: olaparib

Location: France

NCT03967938

Efficacy of Olaparib in Advanced Cancers Occurring in Patients With Germline Mutations or Somatic Tumor Mutations in Homologous Recombination Genes

Cancer type: Unspecified Solid Tumor

Variant class: BRCA1 mutation

Other identifiers: 1-2018 BSMO, 1-2018BSMO, EudraCT Number: 2018-002966-37, Precision 2 - Olaparib

Population segments: Line of therapy N/A, Stage III, Stage IV

Exclusion criteria variant classes: BRCA1 germline mutation (Breast Cancer, Pancreatic Cancer, Prostate Cancer), BRCA2 germline mutation (Breast Cancer, Pancreatic Cancer, Prostate Cancer)

Phase: II

Therapy: olaparib

Location: Belgium

NCT02693535

Targeted Agent and Profiling Utilization Registry (TAPUR) Study.

Cancer type: Unspecified Solid Tumor

Variant class: BRCA1 mutation

Other identifiers: 20170529, NCI-2017-00510, Pro00014171, TAPUR

Population segments: Aggressive, BRAF, Diffuse large B-cell lymphoma (DLBCL), Extranodal marginal zone B-cell lymphoma (MALT), FGFR, First line, Follicular lymphoma (FL), Fourth line or greater, Indolent, Lymphoblastic lymphoma (LBL), Mantle cell lymphoma (MCL), Other subtype, Second line, Small lymphocytic lymphoma (SLL), Stage III, Stage IV, Third line, Waldenstrom's macroglobulinemia (WM)

Phase: II

Therapies: olaparib, talazoparib, atezolizumab + talazoparib

Location: United States

US States: AL, AZ, CA, FL, GA, HI, IL, IN, ME, MI, NC, ND, NE, NH, OH, OR, PA, SD, TX, UT, VA, WA

Contact: Pam Mangat [pam.mangat@asco.org]

Disclaimer: The data presented here is from a curated knowledgebase of publicly available information, but may not be exhaustive. The data version is 2021.11(003).

BRCA1 truncating mutation (continued)

NCT04171700
A Phase II Multicenter, Open-label Study of Rucaparib as Treatment for Solid Tumors Associated With Deleterious Mutations in Homologous Recombination Repair Genes

Cancer type: Unspecified Solid Tumor

Variant class: BRCA1 mutation

Other identifiers: 19-756, 20619, AAAS6974, CO-338-100, LODESTAR, NCI-2019-08226

Population segments: BRCA, Second line, Stage III, Stage IV

Exclusion criteria variant classes: BRCA1 germline mutation (Breast Cancer, Ovarian Cancer, Prostate Cancer), BRCA2 germline mutation (Breast Cancer, Ovarian Cancer, Prostate Cancer)

Phase: II

Therapy: rucaparib

Location: United States

US States: CA, FL, IA, IL, MA, MN, NY, OH, OK, PA, TN, WA

Contact: Clovis Oncology For North America [844-258-7662; medinfo@clovisoncology.com]

NCT02286687
Phase II Study of the PARP Inhibitor Talazoparib in Advanced Cancer Patients With Somatic Alterations in BRCA1/2, Mutations/Deletions in Other BRCA Pathway Genes and Germline Mutation in BRCA1/2 (Not Breast or Ovarian Cancer)

Cancer type: Unspecified Cancer

Variant class: BRCA1 mutation

Other identifiers: 2013-0961, NCI-2014-02494

Population segments: Fourth line or greater, Stage III, Stage IV

Exclusion criteria variant classes: BRCA1 germline mutation (Breast Cancer, Ovarian Cancer), BRCA2 germline mutation (Breast Cancer, Ovarian Cancer)

Phase: II

Therapy: talazoparib

Location: United States

US State: TX

Contact: Dr. Sarina Piha-Paul [713-563-1930; spihapau@mdanderson.org]

NCT04550494
A Pharmacodynamics-Driven Trial of Talazoparib, an Oral PARP Inhibitor, in Patients With Advanced Solid Tumors and Aberrations in Genes Involved in DNA Damage Response

Cancer type: Unspecified Solid Tumor

Variant class: BRCA1 mutation

Other identifiers: 000246, 000264-C, 10000264, 10371, NCI-2020-06906

Population segments: BRCA, Fourth line or greater, HER2 positive, Hormone refractory, Second line, Stage II, Stage III, Stage IV, Third line

Phase: II

Therapy: talazoparib

Location: United States

US State: MD

Contact: Multiple contacts: See www.clinicaltrials.gov for complete list of contacts.

Disclaimer: The data presented here is from a curated knowledgebase of publicly available information, but may not be exhaustive. The data version is 2021.11(003).

BRCA1 truncating mutation (continued)

NCT03842228
A Phase Ib Biomarker-Driven Combination Trial of Copanlisib, Olaparib, and Durvalumab (MEDI4736) in Patients With Advanced Solid Tumors

Cancer type: Unspecified Solid Tumor

Variant class: BRCA1 mutation

Other identifiers: 10217, 19-628, NCI10217, NCI-2019-00601

Population segments: Line of therapy N/A, Stage III, Stage IV

Phase: I

Therapies: copanlisib, olaparib, durvalumab

Location: United States

US States: MA, TX

Contact: Multiple contacts: See www.clinicaltrials.gov for complete list of contacts.

NCT04586335
Open Label, Phase Ib Study to Evaluate the Safety, Tolerability, Pharmacokinetics and Clinical Activity of CYH33, an Oral a-specific PI3K Inhibitor in Combination With Olaparib, an Oral PARP Inhibitor in Patients With Advanced Solid Tumors.

Cancer type: Breast Cancer, Endometrial Carcinoma, Ovarian Cancer, Prostate Cancer, Unspecified Solid Tumor

Variant class: BRCA1 mutation

Other identifiers: CTR20202339, CYH33-G102

Population segments: BRCA, Second line, Stage III, Stage IV

Phase: I

Therapies: HH-CYH33, olaparib

Locations: Australia, United States

US State: TX

Contact: Dr. Jason Sudia [908-380-1329; jason.sudia@haihepharma.com]

NCT03415659
A Phase I, Open-label, Single-center, Single/Multiple-dose, Dose-escalation/ Dose-expansion Clinical Study on Tolerance and Pharmacokinetics of HWH340 Tablet in Patients With Advanced Solid Tumors

Cancer type: Unspecified Solid Tumor

Variant class: BRCA1 mutation

Other identifiers: CTR20171649, HWH340-RFPA 20170821, RFPA 20170821

Population segments: BRCA, Fourth line or greater, HER2 negative, Second line, Stage III, Stage IV, Third line

Phase: I

Therapy: HWH-340

Location: China

NCT04197713
Phase I Sequential Trial of Agents Against DNA Repair (STAR)

Cancer type: Unspecified Solid Tumor

Variant class: BRCA1 mutation

Other identifiers: (STAR), 10329, 2020-0347, NCI-2019-08262, STAR Study

Population segments: Second line, Stage III, Stage IV

Phase: I

Therapies: olaparib, adavosertib

Location: United States

US State: TX

Contact: Site Public Contact [877-632-6789; askmdanderson@mdanderson.org]

BRCA1 truncating mutation (continued)

NCT03297606
Canadian Profiling and Targeted Agent Utilization Trial (CAPTUR): A Phase II Basket Trial

Cancer type: Unspecified Solid Tumor

Variant class: BRCA1 aberration

Other identifiers: CA209-9DL, CAPTUR, ESR-17-12831, ML39800, PM1, WI233446

Population segments: Aggressive, Diffuse large B-cell lymphoma (DLBCL), Extranodal marginal zone B-cell lymphoma (MALT), First line, Follicular lymphoma (FL), Indolent, Lymphoblastic lymphoma (LBL), Mantle cell lymphoma (MCL), Other subtype, Second line, Stage III, Stage IV, Waldenstrom's macroglobulinemia (WM)

Phase: II

Therapy: olaparib

Location: Canada

NCT04497116
Phase I/IIa Study of the Safety, Pharmacokinetics, Pharmacodynamics and Preliminary Clinical Activity of RP-3500 Alone or in Combination With Talazoparib in Advanced Solid Tumors With ATR Inhibitor Sensitizing Mutations (TRESR Study)

Cancer type: Unspecified Solid Tumor

Variant class: BRCA1 aberration

Other identifiers: 20-521, EudraCT Number: 2020-000301-87, IRAS ID: 286609, NCI-2020-06869, RP-3500-01, TRESR, TRESR Study

Population segments: Second line, Stage III, Stage IV

Phase: I/II

Therapies: RP-3500, talazoparib

Locations: Canada, Denmark, United Kingdom, United States

US States: MA, NC, NY, RI, TN, TX

Contact: Dr. Peter Manley [857-322-5553; pmanley@reparerx.com]

NCT04123366
A Phase II Study of Olaparib in Combination With Pembrolizumab in Participants With Previously Treated, Homologous Recombination Repair Mutation (HRRm) and/or Homologous Recombination Deficiency (HRD)-Positive Advanced Cancer

Cancer type: Unspecified Solid Tumor

Variant class: HRR mutation

Other identifiers: 7339-007, EudraCT Number: 2019-001745-40, jRCT2011200025, KEYLYNK-007, MK-7339-007, NCI-2019-08758, PER-029-19

Population segments: BRCA, Second line, Stage III, Stage IV

Exclusion criteria variant classes: BRCA germline mutation (Breast Cancer, Ovarian Cancer), BRCA mutation (Breast Cancer, Ovarian Cancer)

Phase: II

Therapies: olaparib, pembrolizumab

Locations: Argentina, Australia, Canada, Colombia, France, Germany, Guatemala, Israel, Italy, Japan, Latvia, Mexico, Peru, Poland, Puerto Rico, Republic of Korea, Romania, South Africa, Spain, Sweden, Turkey, Ukraine, United States

US States: AL, AZ, CA, CO, FL, GA, KY, NJ, NY, OH, TX, UT, VA

Contact: Toll Free Number [888-577-8839; Trialsites@merck.com]

Disclaimer: The data presented here is from a curated knowledgebase of publicly available information, but may not be exhaustive. The data version is 2021.11(003).

BRCA1 truncating mutation (continued)

NCT03767075
Basket of Baskets: A Modular, Open-label, Phase II, Multicentre Study To Evaluate Targeted Agents in Molecularly Selected Populations With Advanced Solid Tumours

Cancer type: Unspecified Solid Tumor

Variant class: DNA repair mutation

Other identifiers: (Basket of Baskets) (BoB), EudraCT number: 2017-005108-89, MO39164, NL65937.031.18, VHIO17002

Population segments: Second line, Stage III, Stage IV

Phase: II

Therapy: atezolizumab

Locations: France, Germany, Netherlands, Spain, Sweden, United Kingdom

---

NCT04905914
A Phase I/IIa, Open-Label, Safety, Pharmacokinetic, And Preliminary Efficacy Study Of Oral ATRN-119 In Patients With Advanced Solid Tumors

Cancer type: Unspecified Solid Tumor

Variant class: DNA repair mutation

Other identifier: AR-276-01

Population segments: Line of therapy N/A, Stage III, Stage IV

Phase: I/II

Therapy: ATRN-119

Location: United States

US State: MA

Contact: Robert Hasson [619-540-6253; rhasson@pacificlinkconsulting.com]

NCT04901702
A Randomized Phase I/II Study of Talazoparib or Temozolomide in Combination With Onivyde in Children With Recurrent Solid Malignancies and Ewing Sarcoma

Cancer type: Unspecified Solid Tumor

Variant class: HRR pathway

Other identifiers: EudraCT Number: 2021-003570-31, ONITT

Population segments: Second line, Stage IV

Exclusion criteria variant classes: HRR germline pathway (Central Nervous System Neoplasm, Ewing Sarcoma), HRR pathway (Central Nervous System Neoplasm, Ewing Sarcoma)

Phase: I/II

Therapies: talazoparib, chemotherapy

Locations: Canada, United States

US State: TN

Contact: Dr. Sara Federico [866-278-5833; referralinfo@stjude.org]

BRCA1 truncating mutation (continued)

NCT03155620
NCI-COG Pediatric MATCH (Molecular Analysis for Therapy Choice) Screening Protocol

Cancer type: Unspecified Solid Tumor

Variant class: DNA repair pathway

Other identifiers: 17-729, 2017-0625, 20170841, APEC1621SC, COGAPEC1621, COGAPEC1621SC, N 55017 SC, NCI-2017-01251, NCI-COG Pediatric MATCH, Pediatric MATCH

Population segments: ALK, Aggressive, BRAF, FGFR, HRAS, Indolent, KRAS, NRAS, Pediatric or Adolescent, Second line, Stage III, Stage IV

Phase: II

Therapy: olaparib

Locations: Puerto Rico, United States

US States: AK, AL, AR, AZ, CA, CO, CT, DC, DE, FL, GA, HI, IA, ID, IL, IN, KY, LA, MA, MD, ME, MI, MN, MO, MS, NC, NE, NH, NJ, NV, NY, OH, OK, OR, PA, RI, SC, SD, TN, TX, UT, VA, VT, WA, WI

Contact: Multiple contacts: See www.clinicaltrials.gov for complete list of contacts.

NCT03233204
NCI-COG Pediatric MATCH (Molecular Analysis for Therapy Choice)- Phase 2 Subprotocol of Olaparib in Patients With Tumors Harboring Defects in DNA Damage Repair Genes

Cancer type: Unspecified Solid Tumor

Variant class: DNA repair pathway

Other identifiers: 20170841, APEC1621H, NCI-2017-00766

Population segments: Aggressive, Indolent, Pediatric or Adolescent, Second line, Stage III, Stage IV

Phase: II

Therapy: olaparib

Locations: Puerto Rico, United States

US States: AK, AL, AR, AZ, CA, CO, DC, DE, FL, GA, IA, ID, IL, IN, KY, LA, MD, ME, MI, MN, MO, MS, NC, ND, NE, NJ, NY, OH, OK, OR, PA, SC, SD, TN, TX, UT, VA, VT, WA, WI

Contact: Multiple contacts: See www.clinicaltrials.gov for complete list of contacts.

NCT04267939
An Open-label Phase Ib Study to Determine the Maximum Tolerated and/or Recommended Phase II Dose of the ATR Inhibitor BAY 1895344 in Combination With PARP Inhibitor Niraparib, in Patients With Recurrent Advanced Solid Tumors and Ovarian Cancer

Cancer type: Ovarian Cancer, Unspecified Solid Tumor

Variant class: DNA repair pathway

Other identifiers: 18595, 19-810, 2019-0471, 20-222, EudraCT Number: 2018-003930-34, NCI-2020-01405

Population segments: Maintenance/Consolidation, Second line, Stage III, Stage IV

Exclusion criteria variant class: DNA repair pathway (Prostate Cancer)

Phase: I

Therapies: BAY-1895344, niraparib

Location: United States

US States: MA, NY, TX

Contact: Bayer Clinical Trials Contact [888-842-2937; clinical-trials-contact@bayer.com]

Disclaimer: The data presented here is from a curated knowledgebase of publicly available information, but may not be exhaustive. The data version is 2021.11(003).

BRCA1 truncating mutation (continued)

NCT04693468
Modular Phase IB Hypothesis-Testing, Biomarker-Driven, Talazoparib Combination Trial (TalaCom)

Cancer type: Unspecified Solid Tumor

Variant class: DNA repair pathway

Other identifiers: 2020-0436, NCI-2020-06041, TalaCom

Population segments: Hormone refractory, Second line, Stage III, Stage IV

Phase: I

Therapies: talazoparib, palbociclib, axitinib, crizotinib

Location: United States

US State: TX

Contact: Timothy A. Yap [713-563-1784; tyap@mdanderson.org]

NCT03810105
A Phase II Study of Olaparib and Durvalumab in Men With Castration Sensitive Biochemically Recurrent Non-Metastatic Prostate Cancer Harboring Mutations in DNA Damage Repair

Cancer type: Prostate Cancer

Variant class: BRCA1 truncating mutation or BRCA1 mutation

Other identifiers: 18-480, NCI-2019-01627

Population segments: Adjuvant, Stage I, Stage II, Stage III

Phase: II

Therapies: durvalumab, olaparib

Location: United States

US States: CA, IL, MI, NJ, NY

Contact: Dr. Karen Autio [646-422-4632; autiok@mskcc.org]

NCT03840967
A Phase II Study Evaluating Safety and Efficacy of Niraparib in Patients With Previously Treated Homologous Recombination (HR) Defective or Loss of Heterozygosity (LOH) High Metastatic Esophageal/Gastroesophageal Junction/Proximal Gastric Adenocarcinoma

Cancer type: Esophageal Cancer, Gastric Cancer, Gastroesophageal Junction Adenocarcinoma

Variant class: BRCA1 truncating mutation

Other identifiers: 20166, 71618, HCRN ESO17-325, NCI-2019-07395

Population segments: Second line, Stage III, Stage IV

Phase: II

Therapy: niraparib

Location: United States

US States: FL, IN, MI, NY

Contact: Dr. Shadia Jalal [317-274-3658; sjalal@iu.edu]

Disclaimer: The data presented here is from a curated knowledgebase of publicly available information, but may not be exhaustive. The data version is 2021.11(003).

BRCA1 truncating mutation (continued)

NCT02975934
TRITON3: A Multicenter, Randomized, Open Label Phase III Study of Rucaparib Versus Physician's Choice of Therapy for Patients With Metastatic Castration Resistant Prostate Cancer Associated With Homologous Recombination Deficiency

Cancer type: Castration-Resistant Prostate Cancer

Variant class: BRCA1 mutation

Other identifiers: 16238, 1723GCCC, 18-146, 18-300, 20171185, 2018IS040, CO-338-063, CSET 2620, EudraCT Number: 2016-003163-20, GU 146, IGUP17020, IRAS ID: 222298, NCI-2017-01110, S16-02260, STAR Trial, TRITON 3_CO-338-063, TRITON3

Population segments: BRCA, First line, Hormone refractory, Stage IV

Phase: III

Therapies: rucaparib, hormone therapy

Locations: Australia, Belgium, Canada, Denmark, France, Germany, Ireland, Israel, Italy, Spain, United Kingdom, United States

US States: AZ, CA, CO, CT, DE, FL, HI, MD, MN, NC, NY, OH, OR, TN, TX, VA, WA

Contact: Clovis Oncology For North America [844-258-7662; medinfo@clovisoncology.com]

NCT04336943
Durvalumab (MEDI4736) and Olaparib (AZD2281) for Treatment of Biochemically Recurrent Prostate Cancer in Men Predicted to Have a High Neoantigen Load: A Pilot Study

Cancer type: Prostate Adenocarcinoma

Variant class: BRCA1 mutation

Other identifiers: 10509, NCI-2020-01860, RG1007001

Population segments: Line of therapy N/A, Stage I, Stage II, Stage III

Phase: II

Therapies: durvalumab, olaparib

Location: United States

US State: WA

Contact: Michael Schweizer [206-606-6252; schweize@uw.edu]

NCT04053322
An International Multicenter Phase II Trial of Durvalumab (MEDI4736) Plus OLAparib Plus Fulvestrant in Metastatic or Locally Advanced ER-positive, HER2-negative Breast Cancer Patients Selected Using Criteria That Predict Sensitivity to Olaparib

Cancer type: Breast Cancer

Variant class: BRCA1 mutation

Other inclusion criteria: ERBB2 negative, ER positive

Other identifiers: CADUSEIME08, DOLAF, EudraCT Number: 2018-003832-57, UC-0140/1812

Population segments: BRCA, Estrogen receptor positive, FGFR, HER2 negative, Progesterone receptor positive, Second line, Stage III, Stage IV

Phase: II

Therapies: durvalumab, olaparib, hormone therapy

Location: France

Disclaimer: The data presented here is from a curated knowledgebase of publicly available information, but may not be exhaustive. The data version is 2021.11(003).

BRCA1 truncating mutation (continued)

NCT03012321
BRCAAway: A Randomized Phase II Trial of Abiraterone, Olaparib, or Abiraterone + Olaparib in Patients With Metastatic Castration-Resistant Prostate Cancer With DNA Repair Defects

Cancer type: Castration-Resistant Prostate Cancer

Variant class: BRCA1 mutation

Other identifiers: 19137, BRCAAway, NCI-2016-01834, NU 16U05, NU_16U05, PCCTC #: c16-168, PCCTC c16-168, STU00203960

Population segments: First line, Hormone refractory, Stage IV

Phase: II

Therapies: hormone therapy, olaparib, steroid

Location: United States

US States: CA, FL, IL, IN, MD, MI, MN, MO, NC, NY, UT, VA

Contact: Study Coordinator [312-695-1301; cancertrials@northwestern.edu]

No NCT ID - see other identifier(s)
Phase II Study To Evaluate The Efficacy And Safety Of Niraparib In Recurrent Or Persistent Rare Gynecologic Malignancies With Homologous Recombination Deficiency (JGOG2052)

Cancer type: Cervical Cancer, Endometrial Carcinoma, Ovarian Cancer, Uterine Leiomyosarcoma

Variant class: BRCA1 mutation

Other identifiers: (JGOG2052), jRCT2031210264

Population segments: BRCA, Second line, Stage III, Stage IV

Phase: II

Therapy: niraparib

Location: Japan

NCT03553004
Niraparib in Metastatic Pancreatic Cancer After Previous Chemotherapy (NIRA-PANC): A Phase II Trial

Cancer type: Pancreatic Cancer

Variant class: BRCA1 mutation

Other identifiers: IIT-2017-NIRA-PANC, NIRA-PANC, STUDY00141862

Population segments: BRCA, Second line, Stage IV, Third line

Phase: II

Therapy: niraparib

Location: United States

US States: KS, MO

Contact: Clinical Trials Nurse Navigator [913-588-3671; navigation@kumc.edu]

NCT03601923
Phase II Proof-of-Concept Trial Testing the PARP Inhibitor Niraparib in Patients With Pancreatic Cancer Harboring Deficiencies in Homologous Recombination DNA Repair

Cancer type: Pancreatic Cancer

Variant class: BRCA1 mutation

Other identifiers: 18-207, NCI-2018-02395

Population segments: Second line, Stage IV

Phase: II

Therapy: niraparib

Location: United States

US State: MA

Contact: Dr. James Clreary [617-632-5960; JCLEARY@PARTNERS.ORG]

Disclaimer: The data presented here is from a curated knowledgebase of publicly available information, but may not be exhaustive. The data version is 2021.11(003).

BRCA1 truncating mutation (continued)

NCT03925350
A Phase II Study of Niraparib in Patients With Advanced Melanoma With Genetic Homologous Recombination (HR) Mutation / Alteration

Cancer type: Melanoma

Variant class: BRCA1 mutation

Other identifier: CPMC17-MEL01

Population segments: Second line, Stage III, Stage IV

Phase: II

Therapy: niraparib

Location: United States

US State: CA

Contact: Laurel Brechtel [415-600-1654; BrechtLA@sutterhealth.org]

NCT04493060
Phase II Study of Niraparib and TSR-042 in Patients With Germline or Somatic BRCA1/2 and PALB2-Related Pancreatic Cancer

Cancer type: Pancreatic Cancer

Variant class: BRCA1 mutation

Other identifiers: MC1841, NCI-2020-05352

Population segments: BRCA, Second line, Stage IV, Third line

Phase: II

Therapies: niraparib, dostarlimab

Location: United States

US States: AZ, FL, MN

Contact: Multiple contacts: See www.clinicaltrials.gov for complete list of contacts.

NCT04187833
Phase II Trial of Nivolumab in Combination with Talazoparib in Patients with Unresectable or Metastatic Melanoma and Mutations in BRCA or BRCA-ness Genes.

Cancer type: Melanoma

Variant class: BRCA1 mutation

Other identifiers: CASE2619, NCI-2020-00416

Population segments: Second line, Stage III, Stage IV

Phase: II

Therapies: nivolumab, talazoparib

Location: United States

US State: OH

Contact: Dr. Pauline Funchain [866-223-8100; TaussigResearch@ccf.org]

Disclaimer: The data presented here is from a curated knowledgebase of publicly available information, but may not be exhaustive. The data version is 2021.11(003).

BRCA1 truncating mutation (continued)

NCT03344965
A Phase II Study of Olaparib Monotherapy in Metastatic Breast Cancer Patients With Germline or Somatic Mutations in DNA Repair Genes (Olaparib Expanded)

Cancer type: Breast Cancer

Variant class: BRCA1 mutation

Other identifiers: 17-428, 18-414, NCI-2018-00778, TBCRC 048, TBCRC048

Population segments: BRCA, Estrogen receptor positive, HER2 negative, HER2 positive, Progesterone receptor positive, Second line, Stage IV

Exclusion criteria variant classes: BRCA1 germline mutation (Breast Cancer), BRCA2 germline mutation (Breast Cancer)

Phase: II

Therapy: olaparib

Location: United States

US States: AL, CA, IL, MA, MD, NC, NJ, NY, PA, WA

Contact: Dr. Nadine Tung [617-667-1962; ntung@bidmc.harvard.edu]

NCT03375307
A Phase II Study of Olaparib (AZD2281) in Patients With Metastatic/Advanced Urothelial Carcinoma With DNA-Repair Defects

Cancer type: Bladder Urothelial Carcinoma

Variant class: BRCA1 mutation

Other identifiers: 10144, 19C0023, NCI-2017-02296, P162941

Population segments: BRCA, STK11, Second line, Stage III, Stage IV

Phase: II

Therapy: olaparib

Location: United States

US States: CA, CO, KY, MD, NY, OK

Contact: Multiple contacts: See www.clinicaltrials.gov for complete list of contacts.

NCT03742895
A Phase II Study of Olaparib Monotherapy in Participants With Previously Treated, Homologous Recombination Repair Mutation (HRRm) or Homologous Recombination Deficiency (HRD) Positive Advanced Cancer

Cancer type: Breast Cancer

Variant class: BRCA1 mutation

Other inclusion criteria: BRCA1 germline mutation negative, BRCA2 germline mutation negative

Other identifiers: (MK-7339-002 / LYNK-002), 19-139, 194694, 2018-01891, 7339-002, 7339-002-00, EudraCT Number: 2018-003007-19, IRAS ID: 255158, JapicCTI-194694, LYNK002, LYNK-002, MK7339-002, MK-7339-002, MK7339-002-00, NCI-2018-03519, PER-046-18, SNCTP000003157

Population segments: BRCA, Fourth line or greater, Second line, Stage III, Stage IV, Third line

Exclusion criteria variant classes: BRCA germline mutation (Breast Cancer, Ovarian Cancer), BRCA mutation (Ovarian Cancer)

Phase: II

Therapy: olaparib

Locations: Argentina, Australia, Canada, Colombia, Denmark, France, Guatemala, Ireland, Israel, Italy, Mexico, Peru, Republic of Korea, Romania, Russian Federation, Spain, Switzerland, Turkey, United Kingdom, United States

US States: AZ, CA, CO, GA, KY, MA, MD, MI, NE, NJ, NY, SD, UT, WA

Contact: Toll Free Number [888-577-8839; Trialsites@merck.com]

Disclaimer: The data presented here is from a curated knowledgebase of publicly available information, but may not be exhaustive. The data version is 2021.11(003).

BRCA1 truncating mutation (continued)

NCT03786796
Phase II Study of Olaparib in Metastatic Renal Cell Carcinoma Patients Harboring a BAP-1 or Other DNA Repair Gene Mutations (ORCHID)

Cancer type: Kidney Cancer

Variant class: BRCA1 mutation

Other identifiers: CRMS-70750, J18166, NCI-2019-06309, ORCHID

Population segments: Fourth line or greater, Second line, Stage IV, Third line

Phase: II

Therapy: olaparib

Location: United States

US State: MD

Contact: Irina Rifkind [410-502-2043; irifkin1@jhmi.edu]

NCT04042831
A Phase II Study of Olaparib in Patients With Advanced Biliary Tract Cancer With Aberrant DNA Repair Gene Mutations

Cancer type: Biliary Tract Carcinoma

Variant class: BRCA1 mutation

Other identifiers: ACCRU-ICRN-1702, NCI-2019-04434

Population segments: Second line, Stage IV

Phase: II

Therapy: olaparib

Location: United States

US States: AZ, MN, NY, TX

Contact: Multiple contacts: See www.clinicaltrials.gov for complete list of contacts.

NCT04515836
Phase II Trial of Olaparib in Homologous Recombination Deficient (HRD) Malignant Mesothelioma

Cancer type: Mesothelioma

Variant class: BRCA1 mutation

Population segments: (N/A), Second line

Phase: II

Therapy: olaparib

Location: United States

US State: IL

Contact: Dr. Hedy L. Kindler [773-702-0360; hkindler@medicine.bsd.uchicago.edu]

NCT04858334
APOLLO: A Randomized Phase II Double-Blind Study of Olaparib Versus Placebo Following Adjuvant Chemotherapy in Patients With Resected Pancreatic Cancer and a Pathogenic BRCA1, BRCA2 or PALB2 Mutation

Cancer type: Pancreatic Cancer

Variant class: BRCA1 mutation

Other identifiers: APOLLO, CTSU/EA2192, EA2192, NCI-2020-05659

Population segments: Adjuvant, BRCA, Maintenance/Consolidation, Stage 0, Stage I, Stage II

Phase: II

Therapy: olaparib

Location: United States

US States: AZ, FL, IA, IL, IN, KS, MI, MO, NC, NJ, NM, NV, NY, OH, OR, PA, WA, WI

Contact: Multiple contacts: See www.clinicaltrials.gov for complete list of contacts.

Disclaimer: The data presented here is from a curated knowledgebase of publicly available information, but may not be exhaustive. The data version is 2021.11(003).

BRCA1 truncating mutation (continued)

NCT02849496

A Phase II Open-Label, Randomized Study of PARP Inhibition (Olaparib) Either Alone or in Combination With Anti-PD-L1 Therapy (Atezolizumab; MPDL3280A) in Homologous DNA Repair (HDR) Deficient, Locally Advanced or Metastatic Non-HER2-Positive Breast Cancer.

Cancer type: Breast Cancer

Variant class: BRCA1 mutation

Other inclusion criteria: ERBB2 negative

Other identifiers: 10020, 109587 (10020), 1608018258, 17-709, 18979, 5UM1CA186709-04, Clinical Trial 18979, NCI-2016-01130, OSU-17011, PHII-146

Population segments: BRCA, HER2 negative, Second line, Stage III, Stage IV

Phase: II

Therapies: olaparib, atezolizumab

Location: United States

US States: CA, CO, CT, FL, IL, MA, MD, MI, MO, NC, NY, OH, PA, TN, UT

Contact: Multiple contacts: See www.clinicaltrials.gov for complete list of contacts.

NCT04939662

Phase II, Single-arm Study of Olaparib and Bevacizumab Combination Therapy in Relapsed Small Cell Lung Cancer Subjects With DNA Damage Response and the Repair Pathway Alteration, ATM Deficiency, SLFN11 Positive, or POU2F3 Positive

Cancer type: Small Cell Lung Cancer

Variant class: BRCA1 mutation

Other identifier: SUKSES-B2

Population segments: (N/A), BRCA, Pulmonary, Second line, Third line

Phase: II

Therapies: olaparib, bevacizumab

Location: Republic of Korea

NCT04261465

The NUVOLA TRIAL: Neoadjuvant Chemotherapy in Unresectable Ovarian Cancer With OLAparib and Weekly Carboplatin Plus Paclitaxel: A Phase II Open-label Multicentre Study

Cancer type: Ovarian Cancer

Variant class: BRCA1 mutation

Other identifiers: 2317, 2345, EudraCT Number: 2018-002687-65, NUVOLA, NUVOLA trial

Population segments: BRCA, Maintenance/Consolidation, Neoadjuvant, Stage III, Stage IV

Phase: II

Therapies: olaparib, chemotherapy

Location: Italy

NCT04633902

Phase II Study of Olaparib in Combination With Pembrolizumab in Patients With Advanced Melanoma With Homologous Recombination (HR) Pathway Gene Mutation

Cancer type: Melanoma

Variant class: BRCA1 mutation

Other identifier: CPMC19-MEL01

Population segments: BRAF, Second line, Stage III, Stage IV

Phase: II

Therapies: olaparib, pembrolizumab

Location: United States

US State: CA

Contact: Dr. Kevin B. Kim [415-885-8600; ClinicalResearch@sutterhealth.org]

BRCA1 truncating mutation (continued)

NCT03248570
Phase II Open Label Study of Pembrolizumab in Patients With Metastatic Castration Resistant Prostate Cancer (mCRPC) With or Without DNA Damage Repair Defects

Cancer type: Castration-Resistant Prostate Cancer

Variant class: BRCA1 mutation

Other identifiers: 16557, 17-22165, CC 16557, NCI-2017-02408

Population segments: BRCA, Hormone refractory, Line of therapy N/A, Stage IV

Phase: II

Therapy: pembrolizumab

Location: United States

US State: CA

Contact: Patricia Li [415-476-5975; GUTrials@ucsf.edu]

NCT03506997
PERSEUS1: Phase II Trial of the Immune Checkpoint Inhibitor Pembrolizumab for Patients Suffering From Metastatic Prostate Cancer

Cancer type: Castration-Resistant Prostate Cancer

Variant class: BRCA1 mutation

Other identifiers: CCR4559, EudraCT Number: 2017-000931-15, ICR-CTSU/2016/10060, IRAS ID 208952, PERSEUS1

Population segments: Hormone refractory, Second line, Stage IV

Phase: II

Therapy: pembrolizumab

Location: United Kingdom

NCT04089189
A Phase II Study to Evaluate the Efficacy, Safety, and Tolerability of IMP4297 Capsules in Subjects With Germline and/or Systemic BRCA1/2 Mutation Positive Advanced Ovarian Cancer With at Least 2 Prior Lines of Standard Systemic Therapy

Cancer type: Ovarian Cancer

Variant class: BRCA1 mutation

Other identifiers: CTR20190652, IMP4297-2018-CN01

Population segments: Stage III, Stage IV, Third line

Phase: II

Therapy: senaparib

Location: China

NCT03990896
Evaluation of Talazoparib, a PARP Inhibitor, in Patients With Somatic BRCA Mutant Metastatic Breast Cancer: Genotyping Based Clinical Trial

Cancer type: Breast Cancer, Triple Negative Breast Cancer

Variant class: BRCA1 mutation

Other inclusion criteria: ERBB2 negative, Hormone receptor positive

Other identifiers: 19-188, NCI-2019-07732

Population segments: BRCA, Estrogen receptor positive, Fourth line or greater, HER2 negative, Progesterone receptor positive, Second line, Stage IV, Third line, Triple receptor negative

Exclusion criteria variant classes: BRCA1 germline mutation (Breast Cancer, Triple Negative Breast Cancer), BRCA2 germline mutation (Breast Cancer, Triple Negative Breast Cancer)

Phase: II

Therapy: talazoparib

Location: United States

US States: MA, TX

Contact: Dr. Neelima Vidula [617-724-4000; nvidula@mgh.harvard.edu]

Disclaimer: The data presented here is from a curated knowledgebase of publicly available information, but may not be exhaustive. The data version is 2021.11(003).

BRCA1 truncating mutation (continued)

NCT04892693
Talazoparib in Advanced Breast Cancer Patients With Homologous Recombinant Deficiency: A Phase II Clinical and Exploratory Biomarker Study of Talazoparib

Cancer type: Breast Cancer

Variant class: BRCA1 mutation

Other identifier: H-2009-079-1157

Population segments: BRCA, Line of therapy N/A, Stage III, Stage IV

Phase: II

Therapy: talazoparib

Location: Republic of Korea

NCT04740190
Combination Talazoparib - Carboplatin for Recurrent High-grade Glioma With DNA Damage Repair Deficiency (DDRd)

Cancer type: Anaplastic Astrocytoma, Anaplastic Oligoastrocytoma, Anaplastic Oligodendroglioma, Anaplastic Pleomorphic Xanthoastrocytoma, Diffuse Midline Glioma, Glioblastoma

Variant class: BRCA1 mutation

Other identifiers: TAC-GReD, UW-20396

Population segments: (N/A), BRCA, Second line

Phase: II

Therapies: talazoparib, chemotherapy

Location: Hong Kong

NCT04253262
A Phase Ib/II Study of Copanlisib Combined With Rucaparib in Patients With Metastatic Castration-resistant Prostate Cancer

Cancer type: Castration-Resistant Prostate Cancer

Variant class: BRCA1 mutation

Other identifiers: BrUOG 360, BrUOG360

Population segments: Hormone refractory, Second line, Stage IV

Phase: I/II

Therapies: copanlisib, rucaparib

Location: United States

US State: RI

Contact: BrUOG [401-863-3000; BrUOG@brown.edu]

NCT03685331
Harnessing Olaparib, Palbociclib and Endocrine Therapy: A Phase I/II Trial of Olaparib, Palbociclib and Fulvestrant in Patients With BRCA Mutation-associated, Hormone Receptor-positive, Human Epidermal Growth Factor Receptor 2 (HER2)-Negative Metastatic Breast Cancer (HOPE)

Cancer type: Breast Cancer

Variant class: BRCA1 mutation

Other inclusion criteria: ERBB2 negative, Hormone receptor positive

Other identifiers: (HOPE), NCI-2019-04210, UPCC 21118

Population segments: BRCA, Estrogen receptor positive, First line, HER2 negative, Progesterone receptor positive, Second line, Stage III, Stage IV, Third line

Phase: I/II

Therapies: olaparib, palbociclib, hormone therapy

Location: United States

US State: PA

Contact: Alexandra Torres [855-216-0098; PennCancerTrials@emergingmed.com]

Disclaimer: The data presented here is from a curated knowledgebase of publicly available information, but may not be exhaustive. The data version is 2021.11(003).

BRCA1 truncating mutation (continued)

No NCT ID - see other identifier(s)
An Open-Label, Randomised, Phase I/II Trial Of Rucaparib Combined With Nivolumab + or - Ipilimumab To Augment Response In Homologous Repair Deficient Patients With Relapsed Ovarian, Primary Peritoneal And Fallopian Tube Cancer

Cancer type: Ovarian Cancer

Variant class: BRCA1 mutation

Other identifiers: CeNturIOn, CeNturIOn-2016, EudraCT Number: 2017-004780-13, ISRCTN10490346

Population segments: (N/A), BRCA, Fourth line or greater, Second line, Third line

Phase: I/II

Therapies: rucaparib, nivolumab, ipilimumab

Location: United Kingdom

NCT03964532
TALAVE: A Pilot Trial of Induction Talazoparib Followed by Combination of Talazoparib and Avelumab in Advanced Breast Cancer

Cancer type: Breast Cancer

Variant class: BRCA1 mutation

Other inclusion criteria: ERBB2 negative

Other identifiers: NCI-2019-06240, STUDY00000023, TALAVE

Population segments: Line of therapy N/A, Stage III, Stage IV

Phase: I/II

Therapies: talazoparib, avelumab

Location: United States

US States: DC, NC, UT

Contact: Dr. Julie Collins [202-444-2223; Julie.Collins@gunet.georgetown.edu]

NCT04673448
Phase IB Trial of Niraparib and TSR-042 in Patients With BRCA-Mutated Breast, Pancreas or Ovary Cancer

Cancer type: Breast Cancer, Ovarian Cancer, Pancreatic Cancer

Variant class: BRCA1 mutation

Other identifiers: 10020, NCI-2020-11636, RG1005140

Population segments: BRCA, Estrogen receptor positive, HER2 positive, Progesterone receptor positive, Second line, Stage III, Stage IV

Phase: I

Therapies: niraparib, dostarlimab

Location: United States

US State: WA

Contact: Elizabeth M. Swisher [206-543-3669; swishere@uw.edu]

NCT04890613
Phase Ib Expansion Study of CX-5461 in Patients With Solid Tumours and BRCA2 and/or PALB2 Mutation

Cancer type: Ovarian Cancer

Variant class: BRCA1 mutation

Other identifier: CX-5461-04

Population segments: BRCA, Second line, Stage II, Stage III, Stage IV

Phase: I

Therapy: pidnarulex

Location: Canada

Disclaimer: The data presented here is from a curated knowledgebase of publicly available information, but may not be exhaustive. The data version is 2021.11(003).

BRCA1 truncating mutation (continued)

NCT04598321
BrUOG 390: Neoadjuvant Treatment With Talazoparib for Women With Newly Diagnosed, Advanced Ovarian Cancer Associated With a Mutation in BRCA1 or BRCA2 (mBRCA)

Cancer type: Ovarian Cancer

Variant class: BRCA1 mutation

Other identifier: BrUOG 390

Population segments: BRCA, Maintenance/Consolidation, Neoadjuvant, Stage II, Stage III, Stage IV

Phase: I

Therapy: talazoparib

Location: United States

US State: RI

Contact: BrUOG [401-863-3000; BrUOG@Brown.edu]

NCT03598270
A Phase III Randomized, Double-blinded Trial of Platinum-based Chemotherapy With or Without Atezolizumab Followed by Niraparib Maintenance With or Without Atezolizumab in Patients With Recurrent Ovarian, Tubal or Peritoneal Cancer and Platinum Treatment-free Interval (TFIp) >6 Months

Cancer type: Ovarian Cancer

Variant class: BRCA mutation

Other identifiers: AGO ovary 2.33, ANITA, BGOG-ov-41, ENGOT-ov41, ENGOT-OV41/GEICO 69-O/ANITA, ENGOT-ov41/GEICO/ANITA, ENGOT-Ov41/GEICO69-O/ANITA, EudraCT Number: 2018-000366-11, GEICO 69-O, GEICO-69-O

Population segments: (N/A), BRCA, Maintenance/Consolidation, Second line, Third line

Phase: III

Therapies: atezolizumab, chemotherapy, niraparib

Locations: Belgium, France, Germany, Italy, Spain

NCT03278717
International Phase III Randomised Study to Evaluate the Efficacy of Maintenance Therapy With Olaparib and Cediranib or Olaparib Alone in Patients With Relapsed Ovarian Cancer Following a Response to Platinum-based Chemotherapy

Cancer type: Ovarian Cancer

Variant class: BRCA mutation

Other identifiers: 36280, ACTRN12618000498291, ANZGOG 1415/2018, CRUK/15/074, CTC 0133, ENGOT-ov35, ENGOT-ov35/NCRI/ICON 9, EudraCT Number: 2017-000161-75, ICON9, NC174, OV26 (CRUK/UCL ICON9), UCL/14/0795

Population segments: BRCA, Maintenance/Consolidation, Second line, Stage III, Stage IV

Phase: III

Therapies: cediranib, olaparib

Locations: Australia, Canada, New Zealand, United Kingdom

Disclaimer: The data presented here is from a curated knowledgebase of publicly available information, but may not be exhaustive. The data version is 2021.11(003).

BRCA1 truncating mutation (continued)

NCT04915755
A Randomized Phase III Double-Blinded Study Comparing the Efficacy and Safety of Niraparib to Placebo in Participants With Either HER2-Negative BRCA-Mutated or Triple-Negative Breast Cancer With Molecular Disease Based on Presence of Circulating Tumor DNA After Definitive Therapy (ZEST)

Cancer type: Breast Cancer

Variant class: BRCA mutation

Other inclusion criteria: ERBB2 negative

Other identifiers: 213831, EudraCT Number: 2020-003973-23, GSK 213831 ZEST, SNCTP000004543, ZEST

Population segments: BRCA, Estrogen receptor positive, HER2 negative, Progesterone receptor positive, Second line, Stage I, Stage II, Stage III, Triple receptor negative

Phase: III

Therapy: niraparib

Locations: Canada, France, Ireland, Italy, Netherlands, Norway, Poland, Russian Federation, Spain, United Kingdom, United States

US State: IL

Contact: US GSK Clinical Trials Call Center [877-379-3718; GSKClinicalSupportHD@gsk.com]

NCT03462342
Combination ATR and PARP Inhibitor (CAPRI) Trial With AZD 6738 and Olaparib in Recurrent Ovarian Cancer

Cancer type: Ovarian Cancer

Variant class: BRCA mutation

Other identifiers: 20-320, CAPRI, ESR-16-12311, NCI-2018-02375, UPCC 15817

Population segments: BRCA, Fourth line or greater, Second line, Stage III, Stage IV, Third line

Phase: II

Therapies: ceralasertib, olaparib

Location: United States

US States: MA, MD, PA

Contact: Diego Rodriguez [215-614-0234; diegorod@pennmedicine.upenn.edu]

No NCT ID - see other identifier(s)
An Investigator-Initiated Phase II Trial Of Niraparib For Patients With BRCA-Mutated Unresectable / Recurrent Biliary Tract, Pancreatic And Other Gastrointestinal Cancer (NIR-B)

Cancer type: Cholangiocarcinoma, Colon Cancer, Gastrointestinal Stromal Tumor, Pancreatic Cancer, Rectal Cancer

Variant class: BRCA mutation

Other identifiers: jRCT2011200023, NIR-B

Population segments: BRCA, Second line, Stage III, Stage IV, Third line

Phase: II

Therapy: niraparib

Location: Japan

NCT04716686
Niraparib Monotherapy as Maintain and Recurrent Treatment of Endometrial Serous Carcinoma: A Multi-center, Open-label, Prospective Clinical Study

Cancer type: Endometrial Serous Adenocarcinoma

Variant class: BRCA mutation

Other identifier: ZL-2306-914

Population segments: BRCA, Fourth line or greater, Maintenance/Consolidation, Second line, Stage III, Stage IV, Third line

Phase: II

Therapy: niraparib

Location: China

Disclaimer: The data presented here is from a curated knowledgebase of publicly available information, but may not be exhaustive. The data version is 2021.11(003).

BRCA1 truncating mutation (continued)

NCT04348045
MAZEPPA: Phase II PRODIGE-GERCOR Study to Evaluate MAintenance Therapy With Olaparib or Selumetinib Plus Durvalumab According to BRCAness and KRAS Somatic Status Personalized in Metastatic Pancreatic Adenocarcinoma Patients

Cancer type: Pancreatic Cancer

Variant class: BRCA mutation

Other inclusion criteria: DPYD wild type

Other identifiers: D19-02, EudraCT Number: 2019-004366-18, MAZEPPA D19-02, MAZEPPA D19-02 PRODIGE 72, MAZEPPA_D19-02, PRODIGY 72 - MAZEPPA

Population segments: BRCA, KRAS, Maintenance/Consolidation, Stage IV

Phase: II

Therapy: olaparib

Location: France

NCT03025035
Open Label, Phase II Pilot Study of Immune Checkpoint Inhibition With Pembrolizumab in Combination With PARP Inhibition With Olaparib in Advanced BRCA-mutated or HDR-defect Breast Cancers

Cancer type: Breast Cancer

Variant class: BRCA mutation

Other identifier: IIT2015-18-Mita-MK3475

Population segments: BRCA, Estrogen receptor positive, HER2 negative, HER2 positive, Progesterone receptor positive, Second line, Stage III, Stage IV, Triple receptor negative

Phase: II

Therapies: pembrolizumab, olaparib

Location: United States

US State: CA

Contact: Parisa Mirzadehgan [310-967-4387; Parisa.Mirzadehgan@cshs.org]

NCT04666740
A Phase II Trial to Evaluate the Safety and Antitumor Activity of Pembrolizumab and OLApaRib (POLAR) Maintenance for Patients With Metastatic Pancreatic Ductal Adenocarcinoma and Homologous Recombination Deficiency and/or Exceptional Treatment Response to Platinum-Based Therapy

Cancer type: Pancreatic Cancer

Variant class: BRCA1 aberration

Other identifier: 20-481

Population segments: BRCA, Maintenance/Consolidation, Stage IV

Phase: II

Therapies: pembrolizumab, olaparib

Location: United States

US States: NJ, NY

Contact: Dr. Wungki Park [646-888-4543; yaqubiea@mskcc.org]

Disclaimer: The data presented here is from a curated knowledgebase of publicly available information, but may not be exhaustive. The data version is 2021.11(003).

BRCA1 truncating mutation (continued)

NCT02264678

A Modular Phase I, Open-Label, Multicentre Study to Assess the Safety, Tolerability, Pharmacokinetics and Preliminary Anti-tumour Activity of Ceralasertib in Combination With Cytotoxic Chemotherapy and/or DNA Damage Repair/Novel Anti-cancer Agents in Patients With Advanced Solid Malignancies

Cancer type: Breast Cancer, Ovarian Cancer

Variant class: BRCA mutation

Other inclusion criteria: ERBB2 negative

Other identifiers: 0C-14-9, 16-031, 18-342, D5330C00004, EudraCT Number: 2014-002233-66, IRAS ID 160200, NCI-2015-00687, Study 4

Population segments: Adenocarcinoma, BRCA, HER2 negative, Pulmonary, Second line, Stage III, Stage IV, Third line, Triple receptor negative

Exclusion criteria variant class: ERBB2 positive (Breast Cancer)

Phase: I

Therapies: ceralasertib, olaparib

Locations: France, Republic of Korea, United Kingdom, United States

US States: CA, MA, NY

Contact: AstraZeneca Clinical Study Information Center [877-240-9479; information.center@astrazeneca.com]

NCT04169841

Precision Medicine Phase II Study Evaluating the Efficacy of a Double Immunotherapy by Durvalumab and Tremelimumab Combined With Olaparib in Patients With Solid Cancers and Carriers of Homologous Recombination Repair Genes Mutation in Response or Stable After Olaparib Treatment

Cancer type: Breast Cancer, Endometrial Carcinoma, Head and Neck Cancer, Kidney Cancer, Non-Small Cell Lung Cancer, Ovarian Cancer, Pancreatic Cancer, Prostate Cancer

Variant class: HRR mutation

Other identifier: GUIDE2REPAIR

Population segments: Fourth line or greater, Hormone refractory, Maintenance/ Consolidation, Second line, Stage III, Stage IV, Third line

Exclusion criteria variant classes: ALK fusion (Lung Cancer), BRAF mutation (Lung Cancer), EGFR mutation (Lung Cancer), ROS1 mutation (Lung Cancer)

Phase: II

Therapies: durvalumab, tremelimumab, olaparib

Location: France

NCT04030559

Phase II Trial of PARP Inhibitor Niraparib for Men With High Risk Prostate Cancer and DNA Damage Response Defects

Cancer type: Prostate Cancer

Variant class: HRR mutation

Other identifiers: 1412676, NCI-2019-04365, UCDCC#279

Population segments: First line, Neoadjuvant, Stage I, Stage II, Stage IV

Phase: II

Therapy: niraparib

Location: United States

US State: CA

Contact: Marc Dall'Era [916-734-3771; mdallera@ucdavis.edu]

Disclaimer: The data presented here is from a curated knowledgebase of publicly available information, but may not be exhaustive. The data version is 2021.11(003).

BRCA1 truncating mutation (continued)

NCT04940637

A Phase II, Open-Label, Single Arm, Prospective, Multicenter Study of Niraparib Plus Dostarlimab in Patients With Advanced NSCLC and/or MPM, and Positive for PD-L1 Expression and Germline or Somatic Mutations in the HRR Genes

Cancer type: Mesothelioma, Non-Small Cell Lung Cancer

Variant class: HRR mutation

Other inclusion criteria: ALK fusion negative, CD274 expression, EGFRi sensitizing mutation negative, ROS1 fusion negative or CD274 expression

Other identifiers: EudraCT Number: 2020-000109-10, UNITO-001, UNITO-001/2020

Population segments: Second line, Stage III, Stage IV

Exclusion criteria variant classes: ALK fusion (Non-Small Cell Lung Cancer), EGFRi sensitizing mutation (Non-Small Cell Lung Cancer), ROS1 fusion (Non-Small Cell Lung Cancer)

Phase: II

Therapies: niraparib, dostarlimab

Location: Italy

NCT05002868

A Multi-center, Open-label, Phase I/Ib Study to Assess the Safety, Pharmacokinetics and Anti-tumor Activity of RP12146, a Poly (ADP-ribose) Polymerase (PARP) Inhibitor, in Patients With Locally Advanced or Metastatic Solid Tumors

Cancer type: Biliary Tract Carcinoma, Breast Cancer, Colorectal Cancer, Endometrial Carcinoma, Gastric Cancer, Ovarian Cancer, Pancreatic Cancer, Prostate Cancer, Small Cell Lung Cancer

Variant class: HRR mutation

Other identifier: RP12146-2101

Population segments: Extensive, Pulmonary, Second line, Stage III, Stage IV

Exclusion criteria variant class: ERBB2 positive (Breast Cancer)

Phase: I

Therapy: RP12146

Location: Poland

NCT03395197

A Phase III, Randomized, Double-blind, Placebo-controlled Study Of Talazoparib With Enzalutamide In Metastatic Castration-resistant Prostate Cancer

Cancer type: Castration-Resistant Prostate Cancer

Variant class: DNA repair mutation

Other identifiers: BASIC-ROYAL / HIGH-ROYAL, C3441021, CSET 2938, CTR20191443, CTR20191444, EudraCT Number: 2017-003295-31, GU 170 - C3441021, HCC 19-054, JapicCTI-194665, LH19.022, MOH_2019-05-16_007127, NCI-2018-01651, Pfizer C3441021, R19062M, RECF3951, RWF_C3441021 TALAPRO-2, TALAPRO-2, TX228665, UCI-18-47, VICCURO17115

Population segments: First line, Hormone refractory, Second line, Stage III, Stage IV

Phase: III

Therapies: talazoparib, hormone therapy

Locations: Argentina, Australia, Belgium, Brazil, Canada, Chile, China, Czech Republic, Finland, France, Germany, Hungary, Israel, Italy, Japan, New Zealand, Norway, Peru, Poland, Portugal, Republic of Korea, South Africa, Spain, Sweden, United Kingdom, United States

US States: AZ, CA, CO, FL, GA, IL, IN, KS, MO, NE, NJ, NY, OH, OK, OR, PA, SC, TN, TX, UT, VA, WI

Contact: Pfizer CT.gov Call Center [800-718-1021; ClinicalTrials.gov_Inquiries@pfizer.com]

Disclaimer: The data presented here is from a curated knowledgebase of publicly available information, but may not be exhaustive. The data version is 2021.11(003).

BRCA1 truncating mutation (continued)

NCT04821622

TALAPRO-3: A Phase III, Randomized, Double-blind, Study of Talazoparib With Enzalutamide Versus Placebo With Enzalutamide in Men With DDR Gene Mutated Metastatic Castration-sensitive Prostate Cancer

Cancer type: Prostate Adenocarcinoma

Variant class: DNA repair mutation

Other identifiers: C3441052, CTR20212191, EudraCT Number: 2021-000248-23, jRCT2031210098, TALAPRO-3

Population segments: Second line, Stage IV

Phase: III

Therapies: talazoparib, hormone therapy

Locations: Japan, Republic of Korea, United States

US States: CA, DC, FL, GA, IL, MI, NJ, NY, PA, SC, TX, UT

Contact: Pfizer CT.gov Call Center [800-718-1021; ClinicalTrials.gov_Inquiries@pfizer.com]

NCT04038502

An Open-label, Multicenter Phase II Study to Compare the Efficacy of Carboplatin as First-line Followed by Second-line Olaparib Versus Olaparib as First-line Followed by Second-line Carboplatin in the Treatment of Patients With Castration Resistant Prostate Cancer Containing Homologous Recombination Deficiency

Cancer type: Castration-Resistant Prostate Cancer

Variant class: DNA repair mutation

Other identifiers: 01781, 01783, 19-08, BRACeD, CX001963-01, ONCA-009-18F

Population segments: First line, Hormone refractory, Second line, Stage IV

Phase: II

Therapies: olaparib, chemotherapy

Location: United States

US States: CA, DC, FL, IL, MI, NC, NY, PA, WA

Contact: Dr. Robert B. Montgomery [206-277-6878; rbmontgo@u.washington.edu]

No NCT ID - see other identifier(s)

Single-arm Exploratory Study for Sintilimab Second-line or Later-line Treatment of Advanced Non-small Cell Lung Cancer with DDR Pathway Gene Mutation

Cancer type: Non-Small Cell Lung Cancer

Variant class: DNA repair mutation

Other identifier: ChiCTR1900023234

Population segments: Fourth line or greater, Second line, Stage III, Stage IV, Third line

Exclusion criteria variant classes: ALK fusion (Non-Small Cell Lung Cancer), EGFRi sensitizing mutation (Non-Small Cell Lung Cancer), ROS1 fusion (Non-Small Cell Lung Cancer)

Phase: I/II

Therapy: sintilimab

Location: China

Disclaimer: The data presented here is from a curated knowledgebase of publicly available information, but may not be exhaustive. The data version is 2021.11(003).

BRCA1 truncating mutation (continued)

NCT04095273

A Multicenter, Non-randomized, Open-label Phase Ib Study to Determine the Maximum Tolerated and Recommended Phase II Dose of the ATR Inhibitor BAY1895344 in Combination With Pembrolizumab and to Characterize Its Safety, Tolerability, Pharmacokinetics and Preliminary Anti-tumor Activity in Patients With Advanced Solid Tumors

Cancer type: Breast Cancer, Castration-Resistant Prostate Cancer, Colorectal Cancer, Gastric Cancer, Gastroesophageal Junction Adenocarcinoma, Non-Small Cell Lung Cancer, Pancreatic Cancer

Variant class: DNA repair mutation

Other inclusion criteria: ATM mutation negative or ATM mutation negative, ERBB2 negative, Hormone receptor positive or ATM underexpression

Other identifiers: 19741, EudraCT Number: 2018-003420-36, Keynote 919, NCI-2019-06734

Population segments: HER2 negative, Hormone refractory, Second line, Stage III, Stage IV

Phase: I

Therapies: BAY-1895344, pembrolizumab

Locations: Germany, Spain, Switzerland, United States

US States: CA, CT, MA, MD, NC, NY, OH, TX

Contact: Bayer Clinical Trials Contact [clinical-trials-contact@bayer.com]

NCT04849364

A Phase II Circulating Tumor DNA Enriched, Genomically Directed Post-neoadjuvant Trial for Patients With Residual Triple Negative Breast Cancer (PERSEVERE)

Cancer type: Triple Negative Breast Cancer

Variant class: DNA repair pathway

Other identifiers: HCRN BRE18-334, PERSEVERE

Population segments: Adjuvant, HER2 negative, Stage I, Stage II, Stage III, Triple receptor negative

Phase: II

Therapies: talazoparib, chemotherapy

Location: United States

US State: IN

Contact: Dr. Bryan P. Schneider [317-948-3885; bpschnei@iu.edu]

Disclaimer: The data presented here is from a curated knowledgebase of publicly available information, but may not be exhaustive. The data version is 2021.11(003).

BRCA1 deletion

NCT02286687

Phase II Study of the PARP Inhibitor Talazoparib in Advanced Cancer Patients With Somatic Alterations in BRCA1/2, Mutations/Deletions in Other BRCA Pathway Genes and Germline Mutation in BRCA1/2 (Not Breast or Ovarian Cancer)

Cancer type: Unspecified Cancer

Variant class: BRCA1 deletion

Other identifiers: 2013-0961, NCI-2014-02494

Population segments: Fourth line or greater, Stage III, Stage IV

Exclusion criteria variant classes: BRCA1 germline mutation (Breast Cancer, Ovarian Cancer), BRCA2 germline mutation (Breast Cancer, Ovarian Cancer)

Phase: II

Therapy: talazoparib

Location: United States

US State: TX

Contact: Dr. Sarina Piha-Paul [713-563-1930; spihapau@mdanderson.org]

NCT03297606

Canadian Profiling and Targeted Agent Utilization Trial (CAPTUR): A Phase II Basket Trial

Cancer type: Unspecified Solid Tumor

Variant class: BRCA1 aberration

Other identifiers: CA209-9DL, CAPTUR, ESR-17-12831, ML39800, PM1, WI233446

Population segments: Aggressive, Diffuse large B-cell lymphoma (DLBCL), Extranodal marginal zone B-cell lymphoma (MALT), First line, Follicular lymphoma (FL), Indolent, Lymphoblastic lymphoma (LBL), Mantle cell lymphoma (MCL), Other subtype, Second line, Stage III, Stage IV, Waldenstrom's macroglobulinemia (WM)

Phase: II

Therapy: olaparib

Location: Canada

NCT04497116

Phase I/IIa Study of the Safety, Pharmacokinetics, Pharmacodynamics and Preliminary Clinical Activity of RP-3500 Alone or in Combination With Talazoparib in Advanced Solid Tumors With ATR Inhibitor Sensitizing Mutations (TRESR Study)

Cancer type: Unspecified Solid Tumor

Variant class: BRCA1 aberration

Other identifiers: 20-521, EudraCT Number: 2020-000301-87, IRAS ID: 286609, NCI-2020-06869, RP-3500-01, TRESR, TRESR Study

Population segments: Second line, Stage III, Stage IV

Phase: I/II

Therapies: RP-3500, talazoparib

Locations: Canada, Denmark, United Kingdom, United States

US States: MA, NC, NY, RI, TN, TX

Contact: Dr. Peter Manley [857-322-5553; pmanley@reparerx.com]

Disclaimer: The data presented here is from a curated knowledgebase of publicly available information, but may not be exhaustive. The data version is 2021.11(003).

BRCA1 deletion (continued)

NCT04901702
A Randomized Phase I/II Study of Talazoparib or Temozolomide in Combination With Onivyde in Children With Recurrent Solid Malignancies and Ewing Sarcoma

Cancer type: Unspecified Solid Tumor

Variant class: HRR pathway

Other identifiers: EudraCT Number: 2021-003570-31, ONITT

Population segments: Second line, Stage IV

Exclusion criteria variant classes: HRR germline pathway (Central Nervous System Neoplasm, Ewing Sarcoma), HRR pathway (Central Nervous System Neoplasm, Ewing Sarcoma)

Phase: I/II

Therapies: talazoparib, chemotherapy

Locations: Canada, United States

US State: TN

Contact: Dr. Sara Federico [866-278-5833; referralinfo@stjude.org]

NCT03155620
NCI-COG Pediatric MATCH (Molecular Analysis for Therapy Choice) Screening Protocol

Cancer type: Unspecified Solid Tumor

Variant class: DNA repair pathway

Other identifiers: 17-729, 2017-0625, 20170841, APEC1621SC, COGAPEC1621, COGAPEC1621SC, N 55017 SC, NCI-2017-01251, NCI-COG Pediatric MATCH, Pediatric MATCH

Population segments: ALK, Aggressive, BRAF, FGFR, HRAS, Indolent, KRAS, NRAS, Pediatric or Adolescent, Second line, Stage III, Stage IV

Phase: II

Therapy: olaparib

Locations: Puerto Rico, United States

US States: AK, AL, AR, AZ, CA, CO, CT, DC, DE, FL, GA, HI, IA, ID, IL, IN, KY, LA, MA, MD, ME, MI, MN, MO, MS, NC, NE, NH, NJ, NV, NY, OH, OK, OR, PA, RI, SC, SD, TN, TX, UT, VA, VT, WA, WI

Contact: Multiple contacts: See www.clinicaltrials.gov for complete list of contacts.

NCT03233204
NCI-COG Pediatric MATCH (Molecular Analysis for Therapy Choice)- Phase 2 Subprotocol of Olaparib in Patients With Tumors Harboring Defects in DNA Damage Repair Genes

Cancer type: Unspecified Solid Tumor

Variant class: DNA repair pathway

Other identifiers: 20170841, APEC1621H, NCI-2017-00766

Population segments: Aggressive, Indolent, Pediatric or Adolescent, Second line, Stage III, Stage IV

Phase: II

Therapy: olaparib

Locations: Puerto Rico, United States

US States: AK, AL, AR, AZ, CA, CO, DC, DE, FL, GA, IA, ID, IL, IN, KY, LA, MD, ME, MI, MN, MO, MS, NC, ND, NE, NJ, NY, OH, OK, OR, PA, SC, SD, TN, TX, UT, VA, VT, WA, WI

Contact: Multiple contacts: See www.clinicaltrials.gov for complete list of contacts.

Disclaimer: The data presented here is from a curated knowledgebase of publicly available information, but may not be exhaustive. The data version is 2021.11(003).

BRCA1 deletion (continued)

NCT04267939
An Open-label Phase Ib Study to Determine the Maximum Tolerated and/or Recommended Phase II Dose of the ATR Inhibitor BAY 1895344 in Combination With PARP Inhibitor Niraparib, in Patients With Recurrent Advanced Solid Tumors and Ovarian Cancer

Cancer type: Ovarian Cancer, Unspecified Solid Tumor

Variant class: DNA repair pathway

Other identifiers: 18595, 19-810, 2019-0471, 20-222, EudraCT Number: 2018-003930-34, NCI-2020-01405

Population segments: Maintenance/Consolidation, Second line, Stage III, Stage IV

Exclusion criteria variant class: DNA repair pathway (Prostate Cancer)

Phase: I

Therapies: BAY-1895344, niraparib

Location: United States

US States: MA, NY, TX

Contact: Bayer Clinical Trials Contact [888-842-2937; clinical-trials-contact@bayer.com]

NCT04693468
Modular Phase IB Hypothesis-Testing, Biomarker-Driven, Talazoparib Combination Trial (TalaCom)

Cancer type: Unspecified Solid Tumor

Variant class: DNA repair pathway

Other identifiers: 2020-0436, NCI-2020-06041, TalaCom

Population segments: Hormone refractory, Second line, Stage III, Stage IV

Phase: I

Therapies: talazoparib, palbociclib, axitinib, crizotinib

Location: United States

US State: TX

Contact: Timothy A. Yap [713-563-1784; tyap@mdanderson.org]

NCT03810105
A Phase II Study of Olaparib and Durvalumab in Men With Castration Sensitive Biochemically Recurrent Non-Metastatic Prostate Cancer Harboring Mutations in DNA Damage Repair

Cancer type: Prostate Cancer

Variant class: BRCA1 deletion

Other identifiers: 18-480, NCI-2019-01627

Population segments: Adjuvant, Stage I, Stage II, Stage III

Phase: II

Therapies: durvalumab, olaparib

Location: United States

US States: CA, IL, MI, NJ, NY

Contact: Dr. Karen Autio [646-422-4632; autiok@mskcc.org]

Disclaimer: The data presented here is from a curated knowledgebase of publicly available information, but may not be exhaustive. The data version is 2021.11(003).

BRCA1 deletion (continued)

NCT03012321

BRCAAway: A Randomized Phase II Trial of Abiraterone, Olaparib, or Abiraterone + Olaparib in Patients With Metastatic Castration-Resistant Prostate Cancer With DNA Repair Defects

Cancer type: Castration-Resistant Prostate Cancer

Variant class: BRCA1 deletion

Other identifiers: 19137, BRCAAway, NCI-2016-01834, NU 16U05, NU_16U05, PCCTC #: c16-168, PCCTC c16-168, STU00203960

Population segments: First line, Hormone refractory, Stage IV

Phase: II

Therapies: hormone therapy, olaparib, steroid

Location: United States

US States: CA, FL, IL, IN, MD, MI, MN, MO, NC, NY, UT, VA

Contact: Study Coordinator [312-695-1301; cancertrials@northwestern.edu]

NCT03840967

A Phase II Study Evaluating Safety and Efficacy of Niraparib in Patients With Previously Treated Homologous Recombination (HR) Defective or Loss of Heterozygosity (LOH) High Metastatic Esophageal/Gastroesophageal Junction/ Proximal Gastric Adenocarcinoma

Cancer type: Esophageal Cancer, Gastric Cancer, Gastroesophageal Junction Adenocarcinoma

Variant class: BRCA1 deletion

Other identifiers: 20166, 71618, HCRN ESO17-325, NCI-2019-07395

Population segments: Second line, Stage III, Stage IV

Phase: II

Therapy: niraparib

Location: United States

US States: FL, IN, MI, NY

Contact: Dr. Shadia Jalal [317-274-3658; sjalal@iu.edu]

NCT04633902

Phase II Study of Olaparib in Combination With Pembrolizumab in Patients With Advanced Melanoma With Homologous Recombination (HR) Pathway Gene Mutation

Cancer type: Melanoma

Variant class: BRCA1 deletion

Other identifier: CPMC19-MEL01

Population segments: BRAF, Second line, Stage III, Stage IV

Phase: II

Therapies: olaparib, pembrolizumab

Location: United States

US State: CA

Contact: Dr. Kevin B. Kim [415-885-8600; ClinicalResearch@sutterhealth.org]

Disclaimer: The data presented here is from a curated knowledgebase of publicly available information, but may not be exhaustive. The data version is 2021.11(003).

BRCA1 deletion (continued)

NCT03248570
Phase II Open Label Study of Pembrolizumab in Patients With Metastatic Castration Resistant Prostate Cancer (mCRPC) With or Without DNA Damage Repair Defects

Cancer type: Castration-Resistant Prostate Cancer

Variant class: BRCA1 deletion

Other identifiers: 16557, 17-22165, CC 16557, NCI-2017-02408

Population segments: BRCA, Hormone refractory, Line of therapy N/A, Stage IV

Phase: II

Therapy: pembrolizumab

Location: United States

US State: CA

Contact: Patricia Li [415-476-5975; GUTrials@ucsf.edu]

NCT04740190
Combination Talazoparib - Carboplatin for Recurrent High-grade Glioma With DNA Damage Repair Deficiency (DDRd)

Cancer type: Anaplastic Astrocytoma, Anaplastic Oligoastrocytoma, Anaplastic Oligodendroglioma, Anaplastic Pleomorphic Xanthoastrocytoma, Diffuse Midline Glioma, Glioblastoma

Variant class: BRCA1 deletion

Other identifiers: TAC-GReD, UW-20396

Population segments: (N/A), BRCA, Second line

Phase: II

Therapies: talazoparib, chemotherapy

Location: Hong Kong

NCT04666740
A Phase II Trial to Evaluate the Safety and Antitumor Activity of Pembrolizumab and OLApaRib (POLAR) Maintenance for Patients With Metastatic Pancreatic Ductal Adenocarcinoma and Homologous Recombination Deficiency and/or Exceptional Treatment Response to Platinum-Based Therapy

Cancer type: Pancreatic Cancer

Variant class: BRCA1 aberration

Other identifier: 20-481

Population segments: BRCA, Maintenance/Consolidation, Stage IV

Phase: II

Therapies: pembrolizumab, olaparib

Location: United States

US States: NJ, NY

Contact: Dr. Wungki Park [646-888-4543; yaqubiea@mskcc.org]

NCT04030559
Phase II Trial of PARP Inhibitor Niraparib for Men With High Risk Prostate Cancer and DNA Damage Response Defects

Cancer type: Prostate Cancer

Variant class: HRR deletion

Other identifiers: 1412676, NCI-2019-04365, UCDCC#279

Population segments: First line, Neoadjuvant, Stage I, Stage II, Stage IV

Phase: II

Therapy: niraparib

Location: United States

US State: CA

Contact: Marc Dall'Era [916-734-3771; mdallera@ucdavis.edu]

BRCA1 deletion (continued)

NCT03344965
A Phase II Study of Olaparib Monotherapy in Metastatic Breast Cancer Patients With Germline or Somatic Mutations in DNA Repair Genes (Olaparib Expanded)

Cancer type: Breast Cancer

Variant class: DNA repair deletion

Other inclusion criteria: BRCA1 germline mutation negative, BRCA2 germline mutation negative

Other identifiers: 17-428, 18-414, NCI-2018-00778, TBCRC 048, TBCRC048

Population segments: BRCA, Estrogen receptor positive, HER2 negative, HER2 positive, Progesterone receptor positive, Second line, Stage IV

Exclusion criteria variant classes: BRCA1 germline mutation (Breast Cancer), BRCA2 germline mutation (Breast Cancer)

Phase: II

Therapy: olaparib

Location: United States

US States: AL, CA, IL, MA, MD, NC, NJ, NY, PA, WA

Contact: Dr. Nadine Tung [617-667-1962; ntung@bidmc.harvard.edu]

NCT03506997
PERSEUS1: Phase II Trial of the Immune Checkpoint Inhibitor Pembrolizumab for Patients Suffering From Metastatic Prostate Cancer

Cancer type: Castration-Resistant Prostate Cancer

Variant class: DNA repair pathway

Other inclusion criteria: MMR deficient

Other identifiers: CCR4559, EudraCT Number: 2017-000931-15, ICR-CTSU/2016/10060, IRAS ID 208952, PERSEUS1

Population segments: Hormone refractory, Second line, Stage IV

Phase: II

Therapy: pembrolizumab

Location: United Kingdom

NCT04849364
A Phase II Circulating Tumor DNA Enriched, Genomically Directed Post-neoadjuvant Trial for Patients With Residual Triple Negative Breast Cancer (PERSEVERE)

Cancer type: Triple Negative Breast Cancer

Variant class: DNA repair pathway

Other identifiers: HCRN BRE18-334, PERSEVERE

Population segments: Adjuvant, HER2 negative, Stage I, Stage II, Stage III, Triple receptor negative

Phase: II

Therapies: talazoparib, chemotherapy

Location: United States

US State: IN

Contact: Dr. Bryan P. Schneider [317-948-3885; bpschnei@iu.edu]

Disclaimer: The data presented here is from a curated knowledgebase of publicly available information, but may not be exhaustive. The data version is 2021.11(003).

BRCA1 deletion (continued)

NCT05002868

A Multi-center, Open-label, Phase I/Ib Study to Assess the Safety, Pharmacokinetics and Anti-tumor Activity of RP12146, a Poly (ADP-ribose) Polymerase (PARP) Inhibitor, in Patients With Locally Advanced or Metastatic Solid Tumors

Cancer type: Biliary Tract Carcinoma, Breast Cancer, Colorectal Cancer, Endometrial Carcinoma, Gastric Cancer, Ovarian Cancer, Pancreatic Cancer, Prostate Cancer, Small Cell Lung Cancer

Variant class: DNA repair pathway

Other identifier: RP12146-2101

Population segments: Extensive, Pulmonary, Second line, Stage III, Stage IV

Exclusion criteria variant class: ERBB2 positive (Breast Cancer)

Phase: I

Therapy: RP12146

Location: Poland

BRCA1 fusion

NCT03297606

Canadian Profiling and Targeted Agent Utilization Trial (CAPTUR): A Phase II Basket Trial

Cancer type: Unspecified Solid Tumor

Variant class: BRCA1 aberration

Other identifiers: CA209-9DL, CAPTUR, ESR-17-12831, ML39800, PM1, WI233446

Population segments: Aggressive, Diffuse large B-cell lymphoma (DLBCL), Extranodal marginal zone B-cell lymphoma (MALT), First line, Follicular lymphoma (FL), Indolent, Lymphoblastic lymphoma (LBL), Mantle cell lymphoma (MCL), Other subtype, Second line, Stage III, Stage IV, Waldenstrom's macroglobulinemia (WM)

Phase: II

Therapy: olaparib

Location: Canada

NCT04497116

Phase I/IIa Study of the Safety, Pharmacokinetics, Pharmacodynamics and Preliminary Clinical Activity of RP-3500 Alone or in Combination With Talazoparib in Advanced Solid Tumors With ATR Inhibitor Sensitizing Mutations (TRESR Study)

Cancer type: Unspecified Solid Tumor

Variant class: BRCA1 aberration

Other identifiers: 20-521, EudraCT Number: 2020-000301-87, IRAS ID: 286609, NCI-2020-06869, RP-3500-01, TRESR, TRESR Study

Population segments: Second line, Stage III, Stage IV

Phase: I/II

Therapies: RP-3500, talazoparib

Locations: Canada, Denmark, United Kingdom, United States

US States: MA, NC, NY, RI, TN, TX

Contact: Dr. Peter Manley [857-322-5553; pmanley@reparerx.com]

Disclaimer: The data presented here is from a curated knowledgebase of publicly available information, but may not be exhaustive. The data version is 2021.11(003).

BRCA1 fusion (continued)

NCT02286687
Phase II Study of the PARP Inhibitor Talazoparib in Advanced Cancer Patients With Somatic Alterations in BRCA1/2, Mutations/Deletions in Other BRCA Pathway Genes and Germline Mutation in BRCA1/2 (Not Breast or Ovarian Cancer)

Cancer type: Unspecified Cancer

Variant class: Fanconi anemia pathway

Other identifiers: 2013-0961, NCI-2014-02494

Population segments: Fourth line or greater, Stage III, Stage IV

Exclusion criteria variant classes: BRCA1 germline mutation (Breast Cancer, Ovarian Cancer), BRCA2 germline mutation (Breast Cancer, Ovarian Cancer)

Phase: II

Therapy: talazoparib

Location: United States

US State: TX

Contact: Dr. Sarina Piha-Paul [713-563-1930; spihapau@mdanderson.org]

NCT04901702
A Randomized Phase I/II Study of Talazoparib or Temozolomide in Combination With Onivyde in Children With Recurrent Solid Malignancies and Ewing Sarcoma

Cancer type: Unspecified Solid Tumor

Variant class: HRR pathway

Other identifiers: EudraCT Number: 2021-003570-31, ONITT

Population segments: Second line, Stage IV

Exclusion criteria variant classes: HRR germline pathway (Central Nervous System Neoplasm, Ewing Sarcoma), HRR pathway (Central Nervous System Neoplasm, Ewing Sarcoma)

Phase: I/II

Therapies: talazoparib, chemotherapy

Locations: Canada, United States

US State: TN

Contact: Dr. Sara Federico [866-278-5833; referralinfo@stjude.org]

NCT03155620
NCI-COG Pediatric MATCH (Molecular Analysis for Therapy Choice) Screening Protocol

Cancer type: Unspecified Solid Tumor

Variant class: DNA repair pathway

Other identifiers: 17-729, 2017-0625, 20170841, APEC1621SC, COGAPEC1621, COGAPEC1621SC, N 55017 SC, NCI-2017-01251, NCI-COG Pediatric MATCH, Pediatric MATCH

Population segments: ALK, Aggressive, BRAF, FGFR, HRAS, Indolent, KRAS, NRAS, Pediatric or Adolescent, Second line, Stage III, Stage IV

Phase: II

Therapy: olaparib

Locations: Puerto Rico, United States

US States: AK, AL, AR, AZ, CA, CO, CT, DC, DE, FL, GA, HI, IA, ID, IL, IN, KY, LA, MA, MD, ME, MI, MN, MO, MS, NC, NE, NH, NJ, NV, NY, OH, OK, OR, PA, RI, SC, SD, TN, TX, UT, VA, VT, WA, WI

Contact: Multiple contacts: See www.clinicaltrials.gov for complete list of contacts.

Disclaimer: The data presented here is from a curated knowledgebase of publicly available information, but may not be exhaustive. The data version is 2021.11(003).

BRCA1 fusion (continued)

NCT03233204
NCI-COG Pediatric MATCH (Molecular Analysis for Therapy Choice)- Phase 2 Subprotocol of Olaparib in Patients With Tumors Harboring Defects in DNA Damage Repair Genes

Cancer type: Unspecified Solid Tumor

Variant class: DNA repair pathway

Other identifiers: 20170841, APEC1621H, NCI-2017-00766

Population segments: Aggressive, Indolent, Pediatric or Adolescent, Second line, Stage III, Stage IV

Phase: II

Therapy: olaparib

Locations: Puerto Rico, United States

US States: AK, AL, AR, AZ, CA, CO, DC, DE, FL, GA, IA, ID, IL, IN, KY, LA, MD, ME, MI, MN, MO, MS, NC, ND, NE, NJ, NY, OH, OK, OR, PA, SC, SD, TN, TX, UT, VA, VT, WA, WI

Contact: Multiple contacts: See www.clinicaltrials.gov for complete list of contacts.

NCT04267939
An Open-label Phase Ib Study to Determine the Maximum Tolerated and/or Recommended Phase II Dose of the ATR Inhibitor BAY 1895344 in Combination With PARP Inhibitor Niraparib, in Patients With Recurrent Advanced Solid Tumors and Ovarian Cancer

Cancer type: Ovarian Cancer, Unspecified Solid Tumor

Variant class: DNA repair pathway

Other identifiers: 18595, 19-810, 2019-0471, 20-222, EudraCT Number: 2018-003930-34, NCI-2020-01405

Population segments: Maintenance/Consolidation, Second line, Stage III, Stage IV

Exclusion criteria variant class: DNA repair pathway (Prostate Cancer)

Phase: I

Therapies: BAY-1895344, niraparib

Location: United States

US States: MA, NY, TX

Contact: Bayer Clinical Trials Contact [888-842-2937; clinical-trials-contact@bayer.com]

NCT04693468
Modular Phase IB Hypothesis-Testing, Biomarker-Driven, Talazoparib Combination Trial (TalaCom)

Cancer type: Unspecified Solid Tumor

Variant class: DNA repair pathway

Other identifiers: 2020-0436, NCI-2020-06041, TalaCom

Population segments: Hormone refractory, Second line, Stage III, Stage IV

Phase: I

Therapies: talazoparib, palbociclib, axitinib, crizotinib

Location: United States

US State: TX

Contact: Timothy A. Yap [713-563-1784; tyap@mdanderson.org]

Disclaimer: The data presented here is from a curated knowledgebase of publicly available information, but may not be exhaustive. The data version is 2021.11(003).

BRCA1 fusion (continued)

NCT03840967
A Phase II Study Evaluating Safety and Efficacy of Niraparib in Patients With Previously Treated Homologous Recombination (HR) Defective or Loss of Heterozygosity (LOH) High Metastatic Esophageal/Gastroesophageal Junction/ Proximal Gastric Adenocarcinoma

Cancer type: Esophageal Cancer, Gastric Cancer, Gastroesophageal Junction Adenocarcinoma

Variant class: BRCA1 fusion

Other identifiers: 20166, 71618, HCRN ESO17-325, NCI-2019-07395

Population segments: Second line, Stage III, Stage IV

Phase: II

Therapy: niraparib

Location: United States

US States: FL, IN, MI, NY

Contact: Dr. Shadia Jalal [317-274-3658; sjalal@iu.edu]

NCT02502266
A Randomized Phase II/III Study of the Combination of Cediranib and Olaparib Compared to Cediranib or Olaparib Alone, or Standard of Care Chemotherapy in Women With Recurrent Platinum-Resistant or -Refractory Ovarian, Fallopian Tube, or Primary Peritoneal Cancer (COCOS)

Cancer type: Ovarian Cancer

Variant class: BRCA fusion

Other identifiers: 16-715, COCOS, GY005, GY005-, GYN 9814, jRCT2031190097, KCT0004526, N 33516, NCI-2015-00651, NRG OV1405, NRG-GY005, OVC2, OVM1405, S16-01681

Population segments: (N/A), BRCA, Fourth line or greater, Second line, Third line

Phase: II/III

Therapies: cediranib, olaparib

Locations: Japan, United States

US State: SD

Contact: Multiple contacts: See www.clinicaltrials.gov for complete list of contacts.

NCT04739800
A Randomized Phase II Trial of Triplet Therapy (a PD-L1 Inhibitor (Durvalumab) MEDI4736 in Combination With Olaparib and Cediranib) Compared to Olaparib and Cediranib or (Durvalumab) MEDI4736 and Cediranib or Standard of Care Chemotherapy in Women With Platinum-Resistant Recurrent Epithelial Ovarian Cancer, Primary Peritoneal or Fallopian Cancer Who Have Received Prior Bevacizumab

Cancer type: Ovarian Cancer

Variant class: BRCA fusion

Other identifiers: NCI-2021-00615, NRG-GY023

Population segments: BRCA, Fourth line or greater, Second line, Stage III, Stage IV, Third line

Phase: II

Therapies: durvalumab, olaparib, cediranib

Location: United States

US States: CA, GA, IA, ID, IL, MD, ME, MN, MO, MT, NC, NE, NV, NY, OH, OK, OR, PA, RI, TN, TX, WA, WI, WV

Contact: Multiple contacts: See www.clinicaltrials.gov for complete list of contacts.

Disclaimer: The data presented here is from a curated knowledgebase of publicly available information, but may not be exhaustive. The data version is 2021.11(003).

BRCA1 fusion (continued)

NCT04666740
A Phase II Trial to Evaluate the Safety and Antitumor Activity of Pembrolizumab and OLApaRib (POLAR) Maintenance for Patients With Metastatic Pancreatic Ductal Adenocarcinoma and Homologous Recombination Deficiency and/or Exceptional Treatment Response to Platinum-Based Therapy

Cancer type: Pancreatic Cancer

Variant class: BRCA1 aberration

Other identifier: 20-481

Population segments: BRCA, Maintenance/Consolidation, Stage IV

Phase: II

Therapies: pembrolizumab, olaparib

Location: United States

US States: NJ, NY

Contact: Dr. Wungki Park [646-888-4543; yaqubiea@mskcc.org]

NCT04633902
Phase II Study of Olaparib in Combination With Pembrolizumab in Patients With Advanced Melanoma With Homologous Recombination (HR) Pathway Gene Mutation

Cancer type: Melanoma

Variant class: HRR pathway

Other identifier: CPMC19-MEL01

Population segments: BRAF, Second line, Stage III, Stage IV

Phase: II

Therapies: olaparib, pembrolizumab

Location: United States

US State: CA

Contact: Dr. Kevin B. Kim [415-885-8600; ClinicalResearch@sutterhealth.org]

NCT03506997
PERSEUS1: Phase II Trial of the Immune Checkpoint Inhibitor Pembrolizumab for Patients Suffering From Metastatic Prostate Cancer

Cancer type: Castration-Resistant Prostate Cancer

Variant class: DNA repair pathway

Other inclusion criteria: MMR deficient

Other identifiers: CCR4559, EudraCT Number: 2017-000931-15, ICR-CTSU/2016/10060, IRAS ID 208952, PERSEUS1

Population segments: Hormone refractory, Second line, Stage IV

Phase: II

Therapy: pembrolizumab

Location: United Kingdom

NCT04849364
A Phase II Circulating Tumor DNA Enriched, Genomically Directed Post-neoadjuvant Trial for Patients With Residual Triple Negative Breast Cancer (PERSEVERE)

Cancer type: Triple Negative Breast Cancer

Variant class: DNA repair pathway

Other identifiers: HCRN BRE18-334, PERSEVERE

Population segments: Adjuvant, HER2 negative, Stage I, Stage II, Stage III, Triple receptor negative

Phase: II

Therapies: talazoparib, chemotherapy

Location: United States

US State: IN

Contact: Dr. Bryan P. Schneider [317-948-3885; bpschnei@iu.edu]

Disclaimer: The data presented here is from a curated knowledgebase of publicly available information, but may not be exhaustive. The data version is 2021.11(003).

BRCA1 fusion (continued)

NCT05002868
A Multi-center, Open-label, Phase I/Ib Study to Assess the Safety, Pharmacokinetics and Anti-tumor Activity of RP12146, a Poly (ADP-ribose) Polymerase (PARP) Inhibitor, in Patients With Locally Advanced or Metastatic Solid Tumors

Cancer type: Biliary Tract Carcinoma, Breast Cancer, Colorectal Cancer, Endometrial Carcinoma, Gastric Cancer, Ovarian Cancer, Pancreatic Cancer, Prostate Cancer, Small Cell Lung Cancer

Variant class: DNA repair pathway

Other identifier: RP12146-2101

Population segments: Extensive, Pulmonary, Second line, Stage III, Stage IV

Exclusion criteria variant class: ERBB2 positive (Breast Cancer)

Phase: I

Therapy: RP12146

Location: Poland

BRCA1 aberration

NCT03297606
Canadian Profiling and Targeted Agent Utilization Trial (CAPTUR): A Phase II Basket Trial

Cancer type: Unspecified Solid Tumor

Variant class: BRCA1 aberration

Other identifiers: CA209-9DL, CAPTUR, ESR-17-12831, ML39800, PM1, WI233446

Population segments: Aggressive, Diffuse large B-cell lymphoma (DLBCL), Extranodal marginal zone B-cell lymphoma (MALT), First line, Follicular lymphoma (FL), Indolent, Lymphoblastic lymphoma (LBL), Mantle cell lymphoma (MCL), Other subtype, Second line, Stage III, Stage IV, Waldenstrom's macroglobulinemia (WM)

Phase: II

Therapy: olaparib

Location: Canada

NCT04497116
Phase I/IIa Study of the Safety, Pharmacokinetics, Pharmacodynamics and Preliminary Clinical Activity of RP-3500 Alone or in Combination With Talazoparib in Advanced Solid Tumors With ATR Inhibitor Sensitizing Mutations (TRESR Study)

Cancer type: Unspecified Solid Tumor

Variant class: BRCA1 aberration

Other identifiers: 20-521, EudraCT Number: 2020-000301-87, IRAS ID: 286609, NCI-2020-06869, RP-3500-01, TRESR, TRESR Study

Population segments: Second line, Stage III, Stage IV

Phase: I/II

Therapies: RP-3500, talazoparib

Locations: Canada, Denmark, United Kingdom, United States

US States: MA, NC, NY, RI, TN, TX

Contact: Dr. Peter Manley [857-322-5553; pmanley@reparerx.com]

Disclaimer: The data presented here is from a curated knowledgebase of publicly available information, but may not be exhaustive. The data version is 2021.11(003).

BRCA1 aberration (continued)

NCT02286687
Phase II Study of the PARP Inhibitor Talazoparib in Advanced Cancer Patients With Somatic Alterations in BRCA1/2, Mutations/Deletions in Other BRCA Pathway Genes and Germline Mutation in BRCA1/2 (Not Breast or Ovarian Cancer)

Cancer type: Unspecified Cancer

Variant class: Fanconi anemia pathway

Other identifiers: 2013-0961, NCI-2014-02494

Population segments: Fourth line or greater, Stage III, Stage IV

Exclusion criteria variant classes: BRCA1 germline mutation (Breast Cancer, Ovarian Cancer), BRCA2 germline mutation (Breast Cancer, Ovarian Cancer)

Phase: II

Therapy: talazoparib

Location: United States

US State: TX

Contact: Dr. Sarina Piha-Paul [713-563-1930; spihapau@mdanderson.org]

NCT04901702
A Randomized Phase I/II Study of Talazoparib or Temozolomide in Combination With Onivyde in Children With Recurrent Solid Malignancies and Ewing Sarcoma

Cancer type: Unspecified Solid Tumor

Variant class: HRR pathway

Other identifiers: EudraCT Number: 2021-003570-31, ONITT

Population segments: Second line, Stage IV

Exclusion criteria variant classes: HRR germline pathway (Central Nervous System Neoplasm, Ewing Sarcoma), HRR pathway (Central Nervous System Neoplasm, Ewing Sarcoma)

Phase: I/II

Therapies: talazoparib, chemotherapy

Locations: Canada, United States

US State: TN

Contact: Dr. Sara Federico [866-278-5833; referralinfo@stjude.org]

NCT03155620
NCI-COG Pediatric MATCH (Molecular Analysis for Therapy Choice) Screening Protocol

Cancer type: Unspecified Solid Tumor

Variant class: DNA repair pathway

Other identifiers: 17-729, 2017-0625, 20170841, APEC1621SC, COGAPEC1621, COGAPEC1621SC, N 55017 SC, NCI-2017-01251, NCI-COG Pediatric MATCH, Pediatric MATCH

Population segments: ALK, Aggressive, BRAF, FGFR, HRAS, Indolent, KRAS, NRAS, Pediatric or Adolescent, Second line, Stage III, Stage IV

Phase: II

Therapy: olaparib

Locations: Puerto Rico, United States

US States: AK, AL, AR, AZ, CA, CO, CT, DC, DE, FL, GA, HI, IA, ID, IL, IN, KY, LA, MA, MD, ME, MI, MN, MO, MS, NC, NE, NH, NJ, NV, NY, OH, OK, OR, PA, RI, SC, SD, TN, TX, UT, VA, VT, WA, WI

Contact: Multiple contacts: See www.clinicaltrials.gov for complete list of contacts.

Disclaimer: The data presented here is from a curated knowledgebase of publicly available information, but may not be exhaustive. The data version is 2021.11(003).

BRCA1 aberration (continued)

NCT03233204
NCI-COG Pediatric MATCH (Molecular Analysis for Therapy Choice)- Phase 2 Subprotocol of Olaparib in Patients With Tumors Harboring Defects in DNA Damage Repair Genes

Cancer type: Unspecified Solid Tumor

Variant class: DNA repair pathway

Other identifiers: 20170841, APEC1621H, NCI-2017-00766

Population segments: Aggressive, Indolent, Pediatric or Adolescent, Second line, Stage III, Stage IV

Phase: II

Therapy: olaparib

Locations: Puerto Rico, United States

US States: AK, AL, AR, AZ, CA, CO, DC, DE, FL, GA, IA, ID, IL, IN, KY, LA, MD, ME, MI, MN, MO, MS, NC, ND, NE, NJ, NY, OH, OK, OR, PA, SC, SD, TN, TX, UT, VA, VT, WA, WI

Contact: Multiple contacts: See www.clinicaltrials.gov for complete list of contacts.

NCT04267939
An Open-label Phase Ib Study to Determine the Maximum Tolerated and/or Recommended Phase II Dose of the ATR Inhibitor BAY 1895344 in Combination With PARP Inhibitor Niraparib, in Patients With Recurrent Advanced Solid Tumors and Ovarian Cancer

Cancer type: Ovarian Cancer, Unspecified Solid Tumor

Variant class: DNA repair pathway

Other identifiers: 18595, 19-810, 2019-0471, 20-222, EudraCT Number: 2018-003930-34, NCI-2020-01405

Population segments: Maintenance/Consolidation, Second line, Stage III, Stage IV

Exclusion criteria variant class: DNA repair pathway (Prostate Cancer)

Phase: I

Therapies: BAY-1895344, niraparib

Location: United States

US States: MA, NY, TX

Contact: Bayer Clinical Trials Contact [888-842-2937; clinical-trials-contact@bayer.com]

NCT04693468
Modular Phase IB Hypothesis-Testing, Biomarker-Driven, Talazoparib Combination Trial (TalaCom)

Cancer type: Unspecified Solid Tumor

Variant class: DNA repair pathway

Other identifiers: 2020-0436, NCI-2020-06041, TalaCom

Population segments: Hormone refractory, Second line, Stage III, Stage IV

Phase: I

Therapies: talazoparib, palbociclib, axitinib, crizotinib

Location: United States

US State: TX

Contact: Timothy A. Yap [713-563-1784; tyap@mdanderson.org]

Disclaimer: The data presented here is from a curated knowledgebase of publicly available information, but may not be exhaustive. The data version is 2021.11(003).

BRCA1 aberration (continued)

NCT04666740

A Phase II Trial to Evaluate the Safety and Antitumor Activity of Pembrolizumab and OLApaRib (POLAR) Maintenance for Patients With Metastatic Pancreatic Ductal Adenocarcinoma and Homologous Recombination Deficiency and/or Exceptional Treatment Response to Platinum-Based Therapy

Cancer type: Pancreatic Cancer

Variant class: BRCA1 aberration

Other identifier: 20-481

Population segments: BRCA, Maintenance/Consolidation, Stage IV

Phase: II

Therapies: pembrolizumab, olaparib

Location: United States

US States: NJ, NY

Contact: Dr. Wungki Park [646-888-4543; yaqubiea@mskcc.org]

NCT04633902

Phase II Study of Olaparib in Combination With Pembrolizumab in Patients With Advanced Melanoma With Homologous Recombination (HR) Pathway Gene Mutation

Cancer type: Melanoma

Variant class: HRR pathway

Other identifier: CPMC19-MEL01

Population segments: BRAF, Second line, Stage III, Stage IV

Phase: II

Therapies: olaparib, pembrolizumab

Location: United States

US State: CA

Contact: Dr. Kevin B. Kim [415-885-8600; ClinicalResearch@sutterhealth.org]

NCT03506997

PERSEUS1: Phase II Trial of the Immune Checkpoint Inhibitor Pembrolizumab for Patients Suffering From Metastatic Prostate Cancer

Cancer type: Castration-Resistant Prostate Cancer

Variant class: DNA repair pathway

Other inclusion criteria: MMR deficient

Other identifiers: CCR4559, EudraCT Number: 2017-000931-15, ICR-CTSU/2016/10060, IRAS ID 208952, PERSEUS1

Population segments: Hormone refractory, Second line, Stage IV

Phase: II

Therapy: pembrolizumab

Location: United Kingdom

NCT04849364

A Phase II Circulating Tumor DNA Enriched, Genomically Directed Post-neoadjuvant Trial for Patients With Residual Triple Negative Breast Cancer (PERSEVERE)

Cancer type: Triple Negative Breast Cancer

Variant class: DNA repair pathway

Other identifiers: HCRN BRE18-334, PERSEVERE

Population segments: Adjuvant, HER2 negative, Stage I, Stage II, Stage III, Triple receptor negative

Phase: II

Therapies: talazoparib, chemotherapy

Location: United States

US State: IN

Contact: Dr. Bryan P. Schneider [317-948-3885; bpschnei@iu.edu]

BRCA1 aberration (continued)

NCT05002868
A Multi-center, Open-label, Phase I/Ib Study to Assess the Safety, Pharmacokinetics and Anti-tumor Activity of RP12146, a Poly (ADP-ribose) Polymerase (PARP) Inhibitor, in Patients With Locally Advanced or Metastatic Solid Tumors

Cancer type: Biliary Tract Carcinoma, Breast Cancer, Colorectal Cancer, Endometrial Carcinoma, Gastric Cancer, Ovarian Cancer, Pancreatic Cancer, Prostate Cancer, Small Cell Lung Cancer

Variant class: DNA repair pathway

Other identifier: RP12146-2101

Population segments: Extensive, Pulmonary, Second line, Stage III, Stage IV

Exclusion criteria variant class: ERBB2 positive (Breast Cancer)

Phase: I

Therapy: RP12146

Location: Poland

Relevant Biomarkers

Genomic Alteration	Relevant Therapies (In this cancer type)	Relevant Therapies (In other cancer type)	Clinical Trials
BRCA1 mutation<br>**Prognostic significance:** None<br>**Diagnostic significance:** None	None	**bevacizumab + olaparib** [1, 2]<br>**niraparib** [1]<br>**olaparib** [1, 2]<br>**rucaparib** [1, 2]	77
BRCA1 splice site mutation<br>**Prognostic significance:** None<br>**Diagnostic significance:** None	None	**bevacizumab + olaparib** [1, 2]<br>**niraparib** [1]<br>**olaparib** [1, 2]<br>**rucaparib** [1, 2]	77
BRCA1 truncating mutation<br>**Prognostic significance:** None<br>**Diagnostic significance:** None	None	**bevacizumab + olaparib** [1, 2]<br>**niraparib** [1]<br>**olaparib** [1, 2]<br>**rucaparib** [1, 2]	77
BRCA1 deletion<br>**Prognostic significance:** None<br>**Diagnostic significance:** None	None	None	21
BRCA1 fusion<br>**Prognostic significance:** None<br>**Diagnostic significance:** None	None	None	16
BRCA1 aberration<br>**Prognostic significance:** None<br>**Diagnostic significance:** None	None	None	13

Public data sources included in relevant therapies: FDA1, NCCN, **EMA**2, ESMO
Public data sources included in prognostic and diagnostic significance: NCCN, ESMO

Relevant Therapy Summary

● In this cancer type ○ In other cancer type ◐ In this cancer type and other cancer types ✖ No evidence

BRCA1 mutation

Relevant Therapy	FDA	NCCN	EMA	ESMO	Clinical Trials*
olaparib	○	○	○	○	◐ (II)
rucaparib	○	○	○	○	● (II)
bevacizumab + olaparib	○	○	○	○	✖
niraparib	○	○	✖	○	○ (III)
olaparib, pembrolizumab	✖	✖	✖	✖	◐ (II)
talazoparib	✖	✖	✖	✖	◐ (II)
talazoparib, chemotherapy	✖	✖	✖	✖	◐ (II)
atezolizumab	✖	✖	✖	✖	● (II)
olaparib, talazoparib, atezolizumab + talazoparib	✖	✖	✖	✖	● (II)
ATRN-119	✖	✖	✖	✖	● (I/II)
RP-3500, talazoparib	✖	✖	✖	✖	● (I/II)
BAY-1895344, niraparib	✖	✖	✖	✖	● (I)
copanlisib, olaparib, durvalumab	✖	✖	✖	✖	● (I)
HH-CYH33, olaparib	✖	✖	✖	✖	● (I)
HWH-340	✖	✖	✖	✖	● (I)
olaparib, adavosertib	✖	✖	✖	✖	● (I)
talazoparib, palbociclib, axitinib, crizotinib	✖	✖	✖	✖	● (I)
atezolizumab, chemotherapy, niraparib	✖	✖	✖	✖	○ (III)
cediranib, olaparib	✖	✖	✖	✖	○ (III)
rucaparib, hormone therapy	✖	✖	✖	✖	○ (III)
talazoparib, hormone therapy	✖	✖	✖	✖	○ (III)
ceralasertib, olaparib	✖	✖	✖	✖	○ (II)
durvalumab, olaparib	✖	✖	✖	✖	○ (II)
durvalumab, olaparib, hormone therapy	✖	✖	✖	✖	○ (II)
durvalumab, tremelimumab, olaparib	✖	✖	✖	✖	○ (II)
hormone therapy, olaparib, steroid	✖	✖	✖	✖	○ (II)
niraparib, dostarlimab	✖	✖	✖	✖	○ (II)
nivolumab, chemotherapy, hormone therapy	✖	✖	✖	✖	○ (II)
nivolumab, talazoparib	✖	✖	✖	✖	○ (II)

* Most advanced phase (IV, III, II/III, II, I/II, I) is shown and multiple clinical trials may be available.

Disclaimer: The data presented here is from a curated knowledgebase of publicly available information, but may not be exhaustive. The data version is 2021.11(003).

Relevant Therapy Summary (continued)

● In this cancer type ○ In other cancer type ◐ In this cancer type and other cancer types ✖ No evidence

BRCA1 mutation (continued)

Relevant Therapy	FDA	NCCN	EMA	ESMO	Clinical Trials*
olaparib, atezolizumab	✖	✖	✖	✖	○ (II)
olaparib, bevacizumab	✖	✖	✖	✖	○ (II)
olaparib, chemotherapy	✖	✖	✖	✖	○ (II)
pembrolizumab	✖	✖	✖	✖	○ (II)
pembrolizumab, olaparib	✖	✖	✖	✖	○ (II)
senaparib	✖	✖	✖	✖	○ (II)
copanlisib, rucaparib	✖	✖	✖	✖	○ (I/II)
olaparib, palbociclib, hormone therapy	✖	✖	✖	✖	○ (I/II)
rucaparib, nivolumab, ipilimumab	✖	✖	✖	✖	○ (I/II)
sintilimab	✖	✖	✖	✖	○ (I/II)
talazoparib, avelumab	✖	✖	✖	✖	○ (I/II)
BAY-1895344, pembrolizumab	✖	✖	✖	✖	○ (I)
pidnarulex	✖	✖	✖	✖	○ (I)
RP12146	✖	✖	✖	✖	○ (I)

BRCA1 splice site mutation

Relevant Therapy	FDA	NCCN	EMA	ESMO	Clinical Trials*
olaparib	○	○	○	○	◐ (II)
rucaparib	○	○	○	○	● (II)
bevacizumab + olaparib	○	○	○	○	✖
niraparib	○	○	✖	○	○ (III)
olaparib, pembrolizumab	✖	✖	✖	✖	◐ (II)
talazoparib	✖	✖	✖	✖	◐ (II)
talazoparib, chemotherapy	✖	✖	✖	✖	◐ (II)
atezolizumab	✖	✖	✖	✖	● (II)
olaparib, talazoparib, atezolizumab + talazoparib	✖	✖	✖	✖	● (II)
ATRN-119	✖	✖	✖	✖	● (I/II)
RP-3500, talazoparib	✖	✖	✖	✖	● (I/II)
BAY-1895344, niraparib	✖	✖	✖	✖	● (I)

* Most advanced phase (IV, III, II/III, II, I/II, I) is shown and multiple clinical trials may be available.

Disclaimer: The data presented here is from a curated knowledgebase of publicly available information, but may not be exhaustive. The data version is 2021.11(003).

Relevant Therapy Summary (continued)

● In this cancer type ○ In other cancer type ◐ In this cancer type and other cancer types ✖ No evidence

BRCA1 splice site mutation (continued)

Relevant Therapy	FDA	NCCN	EMA	ESMO	Clinical Trials*
copanlisib, olaparib, durvalumab	✖	✖	✖	✖	● (I)
HH-CYH33, olaparib	✖	✖	✖	✖	● (I)
HWH-340	✖	✖	✖	✖	● (I)
olaparib, adavosertib	✖	✖	✖	✖	● (I)
talazoparib, palbociclib, axitinib, crizotinib	✖	✖	✖	✖	● (I)
atezolizumab, chemotherapy, niraparib	✖	✖	✖	✖	○ (III)
cediranib, olaparib	✖	✖	✖	✖	○ (III)
rucaparib, hormone therapy	✖	✖	✖	✖	○ (III)
talazoparib, hormone therapy	✖	✖	✖	✖	○ (III)
ceralasertib, olaparib	✖	✖	✖	✖	○ (II)
durvalumab, olaparib	✖	✖	✖	✖	○ (II)
durvalumab, olaparib, hormone therapy	✖	✖	✖	✖	○ (II)
durvalumab, tremelimumab, olaparib	✖	✖	✖	✖	○ (II)
hormone therapy, olaparib, steroid	✖	✖	✖	✖	○ (II)
niraparib, dostarlimab	✖	✖	✖	✖	○ (II)
nivolumab, chemotherapy, hormone therapy	✖	✖	✖	✖	○ (II)
nivolumab, talazoparib	✖	✖	✖	✖	○ (II)
olaparib, atezolizumab	✖	✖	✖	✖	○ (II)
olaparib, bevacizumab	✖	✖	✖	✖	○ (II)
olaparib, chemotherapy	✖	✖	✖	✖	○ (II)
pembrolizumab	✖	✖	✖	✖	○ (II)
pembrolizumab, olaparib	✖	✖	✖	✖	○ (II)
senaparib	✖	✖	✖	✖	○ (II)
copanlisib, rucaparib	✖	✖	✖	✖	○ (I/II)
olaparib, palbociclib, hormone therapy	✖	✖	✖	✖	○ (I/II)
rucaparib, nivolumab, ipilimumab	✖	✖	✖	✖	○ (I/II)
sintilimab	✖	✖	✖	✖	○ (I/II)
talazoparib, avelumab	✖	✖	✖	✖	○ (I/II)
BAY-1895344, pembrolizumab	✖	✖	✖	✖	○ (I)

* Most advanced phase (IV, III, II/III, II, I/II, I) is shown and multiple clinical trials may be available.

Disclaimer: The data presented here is from a curated knowledgebase of publicly available information, but may not be exhaustive. The data version is 2021.11(003).

Relevant Therapy Summary (continued)

● In this cancer type ○ In other cancer type ◐ In this cancer type and other cancer types ✖ No evidence

BRCA1 splice site mutation (continued)

Relevant Therapy	FDA	NCCN	EMA	ESMO	Clinical Trials*
pidnarulex	✖	✖	✖	✖	○ (I)
RP12146	✖	✖	✖	✖	○ (I)

BRCA1 truncating mutation

Relevant Therapy	FDA	NCCN	EMA	ESMO	Clinical Trials*
olaparib	○	○	○	○	◐ (II)
rucaparib	○	○	○	○	● (II)
bevacizumab + olaparib	○	○	○	○	✖
niraparib	○	○	✖	○	○ (III)
olaparib, pembrolizumab	✖	✖	✖	✖	◐ (II)
talazoparib	✖	✖	✖	✖	◐ (II)
talazoparib, chemotherapy	✖	✖	✖	✖	◐ (II)
atezolizumab	✖	✖	✖	✖	● (II)
olaparib, talazoparib, atezolizumab + talazoparib	✖	✖	✖	✖	● (II)
ATRN-119	✖	✖	✖	✖	● (I/II)
RP-3500, talazoparib	✖	✖	✖	✖	● (I/II)
BAY-1895344, niraparib	✖	✖	✖	✖	● (I)
copanlisib, olaparib, durvalumab	✖	✖	✖	✖	● (I)
HH-CYH33, olaparib	✖	✖	✖	✖	● (I)
HWH-340	✖	✖	✖	✖	● (I)
olaparib, adavosertib	✖	✖	✖	✖	● (I)
talazoparib, palbociclib, axitinib, crizotinib	✖	✖	✖	✖	● (I)
atezolizumab, chemotherapy, niraparib	✖	✖	✖	✖	○ (III)
cediranib, olaparib	✖	✖	✖	✖	○ (III)
rucaparib, hormone therapy	✖	✖	✖	✖	○ (III)
talazoparib, hormone therapy	✖	✖	✖	✖	○ (III)
ceralasertib, olaparib	✖	✖	✖	✖	○ (II)
durvalumab, olaparib	✖	✖	✖	✖	○ (II)
durvalumab, olaparib, hormone therapy	✖	✖	✖	✖	○ (II)

* Most advanced phase (IV, III, II/III, II, I/II, I) is shown and multiple clinical trials may be available.

Disclaimer: The data presented here is from a curated knowledgebase of publicly available information, but may not be exhaustive. The data version is 2021.11(003).

Relevant Therapy Summary (continued)

● In this cancer type ○ In other cancer type ◐ In this cancer type and other cancer types ✖ No evidence

BRCA1 truncating mutation (continued)

Relevant Therapy	FDA	NCCN	EMA	ESMO	Clinical Trials*
durvalumab, tremelimumab, olaparib	✖	✖	✖	✖	○ (II)
hormone therapy, olaparib, steroid	✖	✖	✖	✖	○ (II)
niraparib, dostarlimab	✖	✖	✖	✖	○ (II)
nivolumab, chemotherapy, hormone therapy	✖	✖	✖	✖	○ (II)
nivolumab, talazoparib	✖	✖	✖	✖	○ (II)
olaparib, atezolizumab	✖	✖	✖	✖	○ (II)
olaparib, bevacizumab	✖	✖	✖	✖	○ (II)
olaparib, chemotherapy	✖	✖	✖	✖	○ (II)
pembrolizumab	✖	✖	✖	✖	○ (II)
pembrolizumab, olaparib	✖	✖	✖	✖	○ (II)
senaparib	✖	✖	✖	✖	○ (II)
copanlisib, rucaparib	✖	✖	✖	✖	○ (I/II)
olaparib, palbociclib, hormone therapy	✖	✖	✖	✖	○ (I/II)
rucaparib, nivolumab, ipilimumab	✖	✖	✖	✖	○ (I/II)
sintilimab	✖	✖	✖	✖	○ (I/II)
talazoparib, avelumab	✖	✖	✖	✖	○ (I/II)
BAY-1895344, pembrolizumab	✖	✖	✖	✖	○ (I)
pidnarulex	✖	✖	✖	✖	○ (I)
RP12146	✖	✖	✖	✖	○ (I)

BRCA1 deletion

Relevant Therapy	FDA	NCCN	EMA	ESMO	Clinical Trials*
olaparib	✖	✖	✖	✖	◐ (II)
talazoparib, chemotherapy	✖	✖	✖	✖	◐ (II)
talazoparib	✖	✖	✖	✖	● (II)
RP-3500, talazoparib	✖	✖	✖	✖	● (I/II)
BAY-1895344, niraparib	✖	✖	✖	✖	● (I)
talazoparib, palbociclib, axitinib, crizotinib	✖	✖	✖	✖	● (I)
durvalumab, olaparib	✖	✖	✖	✖	○ (II)

* Most advanced phase (IV, III, II/III, II, I/II, I) is shown and multiple clinical trials may be available.

Disclaimer: The data presented here is from a curated knowledgebase of publicly available information, but may not be exhaustive. The data version is 2021.11(003).

Relevant Therapy Summary (continued)

● In this cancer type ○ In other cancer type ◐ In this cancer type and other cancer types ✖ No evidence

BRCA1 deletion (continued)

Relevant Therapy	FDA	NCCN	EMA	ESMO	Clinical Trials*
hormone therapy, olaparib, steroid	✖	✖	✖	✖	○ (II)
niraparib	✖	✖	✖	✖	○ (II)
nivolumab, chemotherapy, hormone therapy	✖	✖	✖	✖	○ (II)
olaparib, pembrolizumab	✖	✖	✖	✖	○ (II)
pembrolizumab	✖	✖	✖	✖	○ (II)
pembrolizumab, olaparib	✖	✖	✖	✖	○ (II)
RP12146	✖	✖	✖	✖	○ (I)

BRCA1 fusion

Relevant Therapy	FDA	NCCN	EMA	ESMO	Clinical Trials*
talazoparib, chemotherapy	✖	✖	✖	✖	◐ (II)
olaparib	✖	✖	✖	✖	● (II)
talazoparib	✖	✖	✖	✖	● (II)
RP-3500, talazoparib	✖	✖	✖	✖	● (I/II)
BAY-1895344, niraparib	✖	✖	✖	✖	● (I)
talazoparib, palbociclib, axitinib, crizotinib	✖	✖	✖	✖	● (I)
cediranib, olaparib	✖	✖	✖	✖	○ (II/III)
durvalumab, olaparib, cediranib	✖	✖	✖	✖	○ (II)
niraparib	✖	✖	✖	✖	○ (II)
olaparib, pembrolizumab	✖	✖	✖	✖	○ (II)
pembrolizumab	✖	✖	✖	✖	○ (II)
pembrolizumab, olaparib	✖	✖	✖	✖	○ (II)
RP12146	✖	✖	✖	✖	○ (I)

BRCA1 aberration

Relevant Therapy	FDA	NCCN	EMA	ESMO	Clinical Trials*
talazoparib, chemotherapy	✖	✖	✖	✖	◐ (II)
olaparib	✖	✖	✖	✖	● (II)
talazoparib	✖	✖	✖	✖	● (II)

* Most advanced phase (IV, III, II/III, II, I/II, I) is shown and multiple clinical trials may be available.

Disclaimer: The data presented here is from a curated knowledgebase of publicly available information, but may not be exhaustive. The data version is 2021.11(003).

Relevant Therapy Summary (continued)

● In this cancer type ○ In other cancer type ◐ In this cancer type and other cancer types ✖ No evidence

BRCA1 aberration (continued)

Relevant Therapy	FDA	NCCN	EMA	ESMO	Clinical Trials*
RP-3500, talazoparib	✖	✖	✖	✖	● (I/II)
BAY-1895344, niraparib	✖	✖	✖	✖	● (I)
talazoparib, palbociclib, axitinib, crizotinib	✖	✖	✖	✖	● (I)
olaparib, pembrolizumab	✖	✖	✖	✖	○ (II)
pembrolizumab	✖	✖	✖	✖	○ (II)
pembrolizumab, olaparib	✖	✖	✖	✖	○ (II)
RP12146	✖	✖	✖	✖	○ (I)

* Most advanced phase (IV, III, II/III, II, I/II, I) is shown and multiple clinical trials may be available.

Relevant Biomarkers

Genomic Alteration	Relevant Therapies (In this cancer type)	Relevant Therapies (In other cancer type)	Clinical Trials
BRCA1 mutation<br>**Prognostic significance:** None<br>**Diagnostic significance:** None	None	**bevacizumab + olaparib** [1,2]<br>**niraparib** [1]<br>**olaparib** [1,2]<br>**rucaparib** [1,2]	77
BRCA1 splice site mutation<br>**Prognostic significance:** None<br>**Diagnostic significance:** None	None	**bevacizumab + olaparib** [1,2]<br>**niraparib** [1]<br>**olaparib** [1,2]<br>**rucaparib** [1,2]	77
BRCA1 truncating mutation<br>**Prognostic significance:** None<br>**Diagnostic significance:** None	None	**bevacizumab + olaparib** [1,2]<br>**niraparib** [1]<br>**olaparib** [1,2]<br>**rucaparib** [1,2]	77
BRCA1 deletion<br>**Prognostic significance:** None<br>**Diagnostic significance:** None	None	None	21
BRCA1 fusion<br>**Prognostic significance:** None<br>**Diagnostic significance:** None	None	None	16

Public data sources included in relevant therapies: FDA1, NCCN, **EMA**2, ESMO
Public data sources included in prognostic and diagnostic significance: NCCN, ESMO

Relevant Biomarkers (continued)

Genomic Alteration	Relevant Therapies (In this cancer type)	Relevant Therapies (In other cancer type)	Clinical Trials
BRCA1 aberration<br>**Prognostic significance:** None<br>**Diagnostic significance:** None	None	None	13

Public data sources included in relevant therapies: **FDA**1, NCCN, **EMA**2, ESMO
Public data sources included in prognostic and diagnostic significance: NCCN, ESMO

Clinical Trials Summary

BRCA1 mutation + BRCA1 splice site mutation + BRCA1 truncating mutation + BRCA1 deletion

NCT ID	Title	Phase
NCT04126070	A Phase II Multicohort Study of Nivolumab in Combination With Docetaxel and Androgen Deprivation Therapy in Metastatic Hormone Sensitive Prostate Cancer Patients With DNA Damage Repair Defects or Inflamed Tumors	II

BRCA1 mutation

NCT ID	Title	Phase
NCT02029001	A Two-period, Multicenter, Randomized, Open-label, Phase II Study Evaluating the Clinical Benefit of a Maintenance Treatment Targeting Tumor Molecular Alterations in Patients with Progressive Locally-advanced or Metastatic Solid Tumors.	II
NCT03967938	Efficacy of Olaparib in Advanced Cancers Occurring in Patients With Germline Mutations or Somatic Tumor Mutations in Homologous Recombination Genes	II
NCT02693535	Targeted Agent and Profiling Utilization Registry (TAPUR) Study.	II
NCT04171700	A Phase II Multicenter, Open-label Study of Rucaparib as Treatment for Solid Tumors Associated With Deleterious Mutations in Homologous Recombination Repair Genes	II
NCT02286687	Phase II Study of the PARP Inhibitor Talazoparib in Advanced Cancer Patients With Somatic Alterations in BRCA1/2, Mutations/Deletions in Other BRCA Pathway Genes and Germline Mutation in BRCA1/2 (Not Breast or Ovarian Cancer)	II
NCT04550494	A Pharmacodynamics-Driven Trial of Talazoparib, an Oral PARP Inhibitor, in Patients With Advanced Solid Tumors and Aberrations in Genes Involved in DNA Damage Response	II
NCT03842228	A Phase Ib Biomarker-Driven Combination Trial of Copanlisib, Olaparib, and Durvalumab (MEDI4736) in Patients With Advanced Solid Tumors	I
NCT04586335	Open Label, Phase Ib Study to Evaluate the Safety, Tolerability, Pharmacokinetics and Clinical Activity of CYH33, an Oral a-specific PI3K Inhibitor in Combination With Olaparib, an Oral PARP Inhibitor in Patients With Advanced Solid Tumors.	I
NCT03415659	A Phase I, Open-label, Single-center, Single/Multiple-dose, Dose-escalation/Dose-expansion Clinical Study on Tolerance and Pharmacokinetics of HWH340 Tablet in Patients With Advanced Solid Tumors	I
NCT04197713	Phase I Sequential Trial of Agents Against DNA Repair (STAR)	I
NCT03297606	Canadian Profiling and Targeted Agent Utilization Trial (CAPTUR): A Phase II Basket Trial	II
NCT04497116	Phase I/IIa Study of the Safety, Pharmacokinetics, Pharmacodynamics and Preliminary Clinical Activity of RP-3500 Alone or in Combination With Talazoparib in Advanced Solid Tumors With ATR Inhibitor Sensitizing Mutations (TRESR Study)	I/II

Disclaimer: The data presented here is from a curated knowledgebase of publicly available information, but may not be exhaustive. The data version is 2021.11(003).

Clinical Trials Summary (continued)

BRCA1 mutation (continued)

NCT ID	Title	Phase
NCT04123366	A Phase II Study of Olaparib in Combination With Pembrolizumab in Participants With Previously Treated, Homologous Recombination Repair Mutation (HRRm) and/or Homologous Recombination Deficiency (HRD)-Positive Advanced Cancer	II
NCT03767075	Basket of Baskets: A Modular, Open-label, Phase II, Multicentre Study To Evaluate Targeted Agents in Molecularly Selected Populations With Advanced Solid Tumours	II
NCT04905914	A Phase I/IIa, Open-Label, Safety, Pharmacokinetic, And Preliminary Efficacy Study Of Oral ATRN-119 In Patients With Advanced Solid Tumors	I/II
NCT04901702	A Randomized Phase I/II Study of Talazoparib or Temozolomide in Combination With Onivyde in Children With Recurrent Solid Malignancies and Ewing Sarcoma	I/II
NCT03155620	NCI-COG Pediatric MATCH (Molecular Analysis for Therapy Choice) Screening Protocol	II
NCT03233204	NCI-COG Pediatric MATCH (Molecular Analysis for Therapy Choice)- Phase 2 Subprotocol of Olaparib in Patients With Tumors Harboring Defects in DNA Damage Repair Genes	II
NCT04267939	An Open-label Phase Ib Study to Determine the Maximum Tolerated and/or Recommended Phase II Dose of the ATR Inhibitor BAY 1895344 in Combination With PARP Inhibitor Niraparib, in Patients With Recurrent Advanced Solid Tumors and Ovarian Cancer	I
NCT04693468	Modular Phase IB Hypothesis-Testing, Biomarker-Driven, Talazoparib Combination Trial (TalaCom)	I
NCT02975934	TRITON3: A Multicenter, Randomized, Open Label Phase III Study of Rucaparib Versus Physician's Choice of Therapy for Patients With Metastatic Castration Resistant Prostate Cancer Associated With Homologous Recombination Deficiency	III
NCT03810105	A Phase II Study of Olaparib and Durvalumab in Men With Castration Sensitive Biochemically Recurrent Non-Metastatic Prostate Cancer Harboring Mutations in DNA Damage Repair	II
NCT04336943	Durvalumab (MEDI4736) and Olaparib (AZD2281) for Treatment of Biochemically Recurrent Prostate Cancer in Men Predicted to Have a High Neoantigen Load: A Pilot Study	II
NCT04053322	An International Multicenter Phase II Trial of Durvalumab (MEDI4736) Plus OLAparib Plus Fulvestrant in Metastatic or Locally Advanced ER-positive, HER2-negative Breast Cancer Patients Selected Using Criteria That Predict Sensitivity to Olaparib	II
NCT03012321	BRCAAway: A Randomized Phase II Trial of Abiraterone, Olaparib, or Abiraterone + Olaparib in Patients With Metastatic Castration-Resistant Prostate Cancer With DNA Repair Defects	II
No NCT ID	Phase II Study To Evaluate The Efficacy And Safety Of Niraparib In Recurrent Or Persistent Rare Gynecologic Malignancies With Homologous Recombination Deficiency (JGOG2052)	II
NCT03553004	Niraparib in Metastatic Pancreatic Cancer After Previous Chemotherapy (NIRA-PANC): A Phase II Trial	II
NCT03601923	Phase II Proof-of-Concept Trial Testing the PARP Inhibitor Niraparib in Patients With Pancreatic Cancer Harboring Deficiencies in Homologous Recombination DNA Repair	II
NCT03840967	A Phase II Study Evaluating Safety and Efficacy of Niraparib in Patients With Previously Treated Homologous Recombination (HR) Defective or Loss of Heterozygosity (LOH) High Metastatic Esophageal/Gastroesophageal Junction/Proximal Gastric Adenocarcinoma	II
NCT03925350	A Phase II Study of Niraparib in Patients With Advanced Melanoma With Genetic Homologous Recombination (HR) Mutation / Alteration	II
NCT04493060	Phase II Study of Niraparib and TSR-042 in Patients With Germline or Somatic BRCA1/2 and PALB2-Related Pancreatic Cancer	II

Clinical Trials Summary (continued)

BRCA1 mutation (continued)

NCT ID	Title	Phase
NCT04187833	Phase II Trial of Nivolumab in Combination with Talazoparib in Patients with Unresectable or Metastatic Melanoma and Mutations in BRCA or BRCA-ness Genes.	II
NCT03344965	A Phase II Study of Olaparib Monotherapy in Metastatic Breast Cancer Patients With Germline or Somatic Mutations in DNA Repair Genes (Olaparib Expanded)	II
NCT03375307	A Phase II Study of Olaparib (AZD2281) in Patients With Metastatic/Advanced Urothelial Carcinoma With DNA-Repair Defects	II
NCT03742895	A Phase II Study of Olaparib Monotherapy in Participants With Previously Treated, Homologous Recombination Repair Mutation (HRRm) or Homologous Recombination Deficiency (HRD) Positive Advanced Cancer	II
NCT03786796	Phase II Study of Olaparib in Metastatic Renal Cell Carcinoma Patients Harboring a BAP-1 or Other DNA Repair Gene Mutations (ORCHID)	II
NCT04042831	A Phase II Study of Olaparib in Patients With Advanced Biliary Tract Cancer With Aberrant DNA Repair Gene Mutations	II
NCT04515836	Phase II Trial of Olaparib in Homologous Recombination Deficient (HRD) Malignant Mesothelioma	II
NCT04858334	APOLLO: A Randomized Phase II Double-Blind Study of Olaparib Versus Placebo Following Adjuvant Chemotherapy in Patients With Resected Pancreatic Cancer and a Pathogenic BRCA1, BRCA2 or PALB2 Mutation	II
NCT02849496	A Phase II Open-Label, Randomized Study of PARP Inhibition (Olaparib) Either Alone or in Combination With Anti-PD-L1 Therapy (Atezolizumab; MPDL3280A) in Homologous DNA Repair (HDR) Deficient, Locally Advanced or Metastatic Non-HER2-Positive Breast Cancer.	II
NCT04939662	Phase II, Single-arm Study of Olaparib and Bevacizumab Combination Therapy in Relapsed Small Cell Lung Cancer Subjects With DNA Damage Response and the Repair Pathway Alteration, ATM Deficiency, SLFN11 Positive, or POU2F3 Positive	II
NCT04261465	The NUVOLA TRIAL: Neoadjuvant Chemotherapy in Unresectable Ovarian Cancer With OLAparib and Weekly Carboplatin Plus Paclitaxel: A Phase II Open-label Multicentre Study	II
NCT04633902	Phase II Study of Olaparib in Combination With Pembrolizumab in Patients With Advanced Melanoma With Homologous Recombination (HR) Pathway Gene Mutation	II
NCT03248570	Phase II Open Label Study of Pembrolizumab in Patients With Metastatic Castration Resistant Prostate Cancer (mCRPC) With or Without DNA Damage Repair Defects	II
NCT03506997	PERSEUS1: Phase II Trial of the Immune Checkpoint Inhibitor Pembrolizumab for Patients Suffering From Metastatic Prostate Cancer	II
NCT04089189	A Phase II Study to Evaluate the Efficacy, Safety, and Tolerability of IMP4297 Capsules in Subjects With Germline and/or Systemic BRCA1/2 Mutation Positive Advanced Ovarian Cancer With at Least 2 Prior Lines of Standard Systemic Therapy	II
NCT03990896	Evaluation of Talazoparib, a PARP Inhibitor, in Patients With Somatic BRCA Mutant Metastatic Breast Cancer: Genotyping Based Clinical Trial	II
NCT04892693	Talazoparib in Advanced Breast Cancer Patients With Homologous Recombinant Deficiency: A Phase II Clinical and Exploratory Biomarker Study of Talazoparib	II
NCT04740190	Combination Talazoparib - Carboplatin for Recurrent High-grade Glioma With DNA Damage Repair Deficiency (DDRd)	II
NCT04253262	A Phase Ib/II Study of Copanlisib Combined With Rucaparib in Patients With Metastatic Castration-resistant Prostate Cancer	I/II

Disclaimer: The data presented here is from a curated knowledgebase of publicly available information, but may not be exhaustive. The data version is 2021.11(003).

Clinical Trials Summary (continued)

BRCA1 mutation (continued)

NCT ID	Title	Phase
NCT03685331	Harnessing Olaparib, Palbociclib and Endocrine Therapy: A Phase I/II Trial of Olaparib, Palbociclib and Fulvestrant in Patients With BRCA Mutation-associated, Hormone Receptor-positive, Human Epidermal Growth Factor Receptor 2 (HER2)-Negative Metastatic Breast Cancer (HOPE)	I/II
No NCT ID	An Open-Label, Randomised, Phase I/II Trial Of Rucaparib Combined With Nivolumab + or - Ipilimumab To Augment Response In Homologous Repair Deficient Patients With Relapsed Ovarian, Primary Peritoneal And Fallopian Tube Cancer	I/II
NCT03964532	TALAVE: A Pilot Trial of Induction Talazoparib Followed by Combination of Talazoparib and Avelumab in Advanced Breast Cancer	I/II
NCT04673448	Phase IB Trial of Niraparib and TSR-042 in Patients With BRCA-Mutated Breast, Pancreas or Ovary Cancer	I
NCT04890613	Phase Ib Expansion Study of CX-5461 in Patients With Solid Tumours and BRCA2 and/or PALB2 Mutation	I
NCT04598321	BrUOG 390: Neoadjuvant Treatment With Talazoparib for Women With Newly Diagnosed, Advanced Ovarian Cancer Associated With a Mutation in BRCA1 or BRCA2 (mBRCA)	I
NCT03598270	A Phase III Randomized, Double-blinded Trial of Platinum-based Chemotherapy With or Without Atezolizumab Followed by Niraparib Maintenance With or Without Atezolizumab in Patients With Recurrent Ovarian, Tubal or Peritoneal Cancer and Platinum Treatment-free Interval (TFIp) >6 Months	III
NCT03278717	International Phase III Randomised Study to Evaluate the Efficacy of Maintenance Therapy With Olaparib and Cediranib or Olaparib Alone in Patients With Relapsed Ovarian Cancer Following a Response to Platinum-based Chemotherapy	III
NCT04915755	A Randomized Phase III Double-Blinded Study Comparing the Efficacy and Safety of Niraparib to Placebo in Participants With Either HER2-Negative BRCA-Mutated or Triple-Negative Breast Cancer With Molecular Disease Based on Presence of Circulating Tumor DNA After Definitive Therapy (ZEST)	III
NCT03462342	Combination ATR and PARP Inhibitor (CAPRI) Trial With AZD 6738 and Olaparib in Recurrent Ovarian Cancer	II
No NCT ID	An Investigator-Initiated Phase II Trial Of Niraparib For Patients With BRCA-Mutated Unresectable / Recurrent Biliary Tract, Pancreatic And Other Gastrointestinal Cancer (NIR-B)	II
NCT04716686	Niraparib Monotherapy as Maintain and Recurrent Treatment of Endometrial Serous Carcinoma: A Multi-center, Open-label, Prospective Clinical Study	II
NCT04348045	MAZEPPA: Phase II PRODIGE-GERCOR Study to Evaluate MAintenance Therapy With Olaparib or Selumetinib Plus Durvalumab According to BRCAness and KRAS Somatic Status Personalized in Metastatic Pancreatic Adenocarcinoma Patients	II
NCT03025035	Open Label, Phase II Pilot Study of Immune Checkpoint Inhibition With Pembrolizumab in Combination With PARP Inhibition With Olaparib in Advanced BRCA-mutated or HDR-defect Breast Cancers	II
NCT04666740	A Phase II Trial to Evaluate the Safety and Antitumor Activity of Pembrolizumab and OLApaRib (POLAR) Maintenance for Patients With Metastatic Pancreatic Ductal Adenocarcinoma and Homologous Recombination Deficiency and/or Exceptional Treatment Response to Platinum-Based Therapy	II
NCT02264678	A Modular Phase I, Open-Label, Multicentre Study to Assess the Safety, Tolerability, Pharmacokinetics and Preliminary Anti-tumour Activity of Ceralasertib in Combination With Cytotoxic Chemotherapy and/or DNA Damage Repair/Novel Anti-cancer Agents in Patients With Advanced Solid Malignancies	I
NCT04169841	Precision Medicine Phase II Study Evaluating the Efficacy of a Double Immunotherapy by Durvalumab and Tremelimumab Combined With Olaparib in Patients With Solid Cancers and Carriers of Homologous Recombination Repair Genes Mutation in Response or Stable After Olaparib Treatment	II

Disclaimer: The data presented here is from a curated knowledgebase of publicly available information, but may not be exhaustive. The data version is 2021.11(003).

Clinical Trials Summary (continued)

BRCA1 mutation (continued)

NCT ID	Title	Phase
NCT04030559	Phase II Trial of PARP Inhibitor Niraparib for Men With High Risk Prostate Cancer and DNA Damage Response Defects	II
NCT04940637	A Phase II, Open-Label, Single Arm, Prospective, Multicenter Study of Niraparib Plus Dostarlimab in Patients With Advanced NSCLC and/or MPM, and Positive for PD-L1 Expression and Germline or Somatic Mutations in the HRR Genes	II
NCT05002868	A Multi-center, Open-label, Phase I/Ib Study to Assess the Safety, Pharmacokinetics and Anti-tumor Activity of RP12146, a Poly (ADP-ribose) Polymerase (PARP) Inhibitor, in Patients With Locally Advanced or Metastatic Solid Tumors	I
NCT03395197	A Phase III, Randomized, Double-blind, Placebo-controlled Study Of Talazoparib With Enzalutamide In Metastatic Castration-resistant Prostate Cancer	III
NCT04821622	TALAPRO-3: A Phase III, Randomized, Double-blind, Study of Talazoparib With Enzalutamide Versus Placebo With Enzalutamide in Men With DDR Gene Mutated Metastatic Castration-sensitive Prostate Cancer	III
NCT04038502	An Open-label, Multicenter Phase II Study to Compare the Efficacy of Carboplatin as First-line Followed by Second-line Olaparib Versus Olaparib as First-line Followed by Second-line Carboplatin in the Treatment of Patients With Castration Resistant Prostate Cancer Containing Homologous Recombination Deficiency	II
No NCT ID	Single-arm Exploratory Study for Sintilimab Second-line or Later-line Treatment of Advanced Non-small Cell Lung Cancer with DDR Pathway Gene Mutation	I/II
NCT04095273	A Multicenter, Non-randomized, Open-label Phase Ib Study to Determine the Maximum Tolerated and Recommended Phase II Dose of the ATR Inhibitor BAY1895344 in Combination With Pembrolizumab and to Characterize Its Safety, Tolerability, Pharmacokinetics and Preliminary Anti-tumor Activity in Patients With Advanced Solid Tumors	I
NCT04849364	A Phase II Circulating Tumor DNA Enriched, Genomically Directed Post-neoadjuvant Trial for Patients With Residual Triple Negative Breast Cancer (PERSEVERE)	II

BRCA1 splice site mutation

NCT ID	Title	Phase
NCT02029001	A Two-period, Multicenter, Randomized, Open-label, Phase II Study Evaluating the Clinical Benefit of a Maintenance Treatment Targeting Tumor Molecular Alterations in Patients with Progressive Locally-advanced or Metastatic Solid Tumors.	II
NCT03967938	Efficacy of Olaparib in Advanced Cancers Occurring in Patients With Germline Mutations or Somatic Tumor Mutations in Homologous Recombination Genes	II
NCT02693535	Targeted Agent and Profiling Utilization Registry (TAPUR) Study.	II
NCT04171700	A Phase II Multicenter, Open-label Study of Rucaparib as Treatment for Solid Tumors Associated With Deleterious Mutations in Homologous Recombination Repair Genes	II
NCT02286687	Phase II Study of the PARP Inhibitor Talazoparib in Advanced Cancer Patients With Somatic Alterations in BRCA1/2, Mutations/Deletions in Other BRCA Pathway Genes and Germline Mutation in BRCA1/2 (Not Breast or Ovarian Cancer)	II
NCT04550494	A Pharmacodynamics-Driven Trial of Talazoparib, an Oral PARP Inhibitor, in Patients With Advanced Solid Tumors and Aberrations in Genes Involved in DNA Damage Response	II

Clinical Trials Summary (continued)

BRCA1 splice site mutation (continued)

NCT ID	Title	Phase
NCT03842228	A Phase Ib Biomarker-Driven Combination Trial of Copanlisib, Olaparib, and Durvalumab (MEDI4736) in Patients With Advanced Solid Tumors	I
NCT04586335	Open Label, Phase Ib Study to Evaluate the Safety, Tolerability, Pharmacokinetics and Clinical Activity of CYH33, an Oral a-specific PI3K Inhibitor in Combination With Olaparib, an Oral PARP Inhibitor in Patients With Advanced Solid Tumors.	I
NCT03415659	A Phase I, Open-label, Single-center, Single/Multiple-dose, Dose-escalation/Dose-expansion Clinical Study on Tolerance and Pharmacokinetics of HWH340 Tablet in Patients With Advanced Solid Tumors	I
NCT04197713	Phase I Sequential Trial of Agents Against DNA Repair (STAR)	I
NCT03297606	Canadian Profiling and Targeted Agent Utilization Trial (CAPTUR): A Phase II Basket Trial	II
NCT04497116	Phase I/IIa Study of the Safety, Pharmacokinetics, Pharmacodynamics and Preliminary Clinical Activity of RP-3500 Alone or in Combination With Talazoparib in Advanced Solid Tumors With ATR Inhibitor Sensitizing Mutations (TRESR Study)	I/II
NCT04123366	A Phase II Study of Olaparib in Combination With Pembrolizumab in Participants With Previously Treated, Homologous Recombination Repair Mutation (HRRm) and/or Homologous Recombination Deficiency (HRD)-Positive Advanced Cancer	II
NCT03767075	Basket of Baskets: A Modular, Open-label, Phase II, Multicentre Study To Evaluate Targeted Agents in Molecularly Selected Populations With Advanced Solid Tumours	II
NCT04905914	A Phase I/IIa, Open-Label, Safety, Pharmacokinetic, And Preliminary Efficacy Study Of Oral ATRN-119 In Patients With Advanced Solid Tumors	I/II
NCT04901702	A Randomized Phase I/II Study of Talazoparib or Temozolomide in Combination With Onivyde in Children With Recurrent Solid Malignancies and Ewing Sarcoma	I/II
NCT03155620	NCI-COG Pediatric MATCH (Molecular Analysis for Therapy Choice) Screening Protocol	II
NCT03233204	NCI-COG Pediatric MATCH (Molecular Analysis for Therapy Choice)- Phase 2 Subprotocol of Olaparib in Patients With Tumors Harboring Defects in DNA Damage Repair Genes	II
NCT04267939	An Open-label Phase Ib Study to Determine the Maximum Tolerated and/or Recommended Phase II Dose of the ATR Inhibitor BAY 1895344 in Combination With PARP Inhibitor Niraparib, in Patients With Recurrent Advanced Solid Tumors and Ovarian Cancer	I
NCT04693468	Modular Phase IB Hypothesis-Testing, Biomarker-Driven, Talazoparib Combination Trial (TalaCom)	I
NCT03810105	A Phase II Study of Olaparib and Durvalumab in Men With Castration Sensitive Biochemically Recurrent Non-Metastatic Prostate Cancer Harboring Mutations in DNA Damage Repair	II
NCT02975934	TRITON3: A Multicenter, Randomized, Open Label Phase III Study of Rucaparib Versus Physician's Choice of Therapy for Patients With Metastatic Castration Resistant Prostate Cancer Associated With Homologous Recombination Deficiency	III
NCT04336943	Durvalumab (MEDI4736) and Olaparib (AZD2281) for Treatment of Biochemically Recurrent Prostate Cancer in Men Predicted to Have a High Neoantigen Load: A Pilot Study	II
NCT04053322	An International Multicenter Phase II Trial of Durvalumab (MEDI4736) Plus OLAparib Plus Fulvestrant in Metastatic or Locally Advanced ER-positive, HER2-negative Breast Cancer Patients Selected Using Criteria That Predict Sensitivity to Olaparib	II
NCT03012321	BRCAAway: A Randomized Phase II Trial of Abiraterone, Olaparib, or Abiraterone + Olaparib in Patients With Metastatic Castration-Resistant Prostate Cancer With DNA Repair Defects	II

Clinical Trials Summary (continued)

BRCA1 splice site mutation (continued)

NCT ID	Title	Phase
No NCT ID	Phase II Study To Evaluate The Efficacy And Safety Of Niraparib In Recurrent Or Persistent Rare Gynecologic Malignancies With Homologous Recombination Deficiency (JGOG2052)	II
NCT03553004	Niraparib in Metastatic Pancreatic Cancer After Previous Chemotherapy (NIRA-PANC): A Phase II Trial	II
NCT03601923	Phase II Proof-of-Concept Trial Testing the PARP Inhibitor Niraparib in Patients With Pancreatic Cancer Harboring Deficiencies in Homologous Recombination DNA Repair	II
NCT03840967	A Phase II Study Evaluating Safety and Efficacy of Niraparib in Patients With Previously Treated Homologous Recombination (HR) Defective or Loss of Heterozygosity (LOH) High Metastatic Esophageal/Gastroesophageal Junction/Proximal Gastric Adenocarcinoma	II
NCT03925350	A Phase II Study of Niraparib in Patients With Advanced Melanoma With Genetic Homologous Recombination (HR) Mutation / Alteration	II
NCT04493060	Phase II Study of Niraparib and TSR-042 in Patients With Germline or Somatic BRCA1/2 and PALB2-Related Pancreatic Cancer	II
NCT04187833	Phase II Trial of Nivolumab in Combination with Talazoparib in Patients with Unresectable or Metastatic Melanoma and Mutations in BRCA or BRCA-ness Genes.	II
NCT03344965	A Phase II Study of Olaparib Monotherapy in Metastatic Breast Cancer Patients With Germline or Somatic Mutations in DNA Repair Genes (Olaparib Expanded)	II
NCT03375307	A Phase II Study of Olaparib (AZD2281) in Patients With Metastatic/Advanced Urothelial Carcinoma With DNA-Repair Defects	II
NCT03742895	A Phase II Study of Olaparib Monotherapy in Participants With Previously Treated, Homologous Recombination Repair Mutation (HRRm) or Homologous Recombination Deficiency (HRD) Positive Advanced Cancer	II
NCT03786796	Phase II Study of Olaparib in Metastatic Renal Cell Carcinoma Patients Harboring a BAP-1 or Other DNA Repair Gene Mutations (ORCHID)	II
NCT04042831	A Phase II Study of Olaparib in Patients With Advanced Biliary Tract Cancer With Aberrant DNA Repair Gene Mutations	II
NCT04515836	Phase II Trial of Olaparib in Homologous Recombination Deficient (HRD) Malignant Mesothelioma	II
NCT04858334	APOLLO: A Randomized Phase II Double-Blind Study of Olaparib Versus Placebo Following Adjuvant Chemotherapy in Patients With Resected Pancreatic Cancer and a Pathogenic BRCA1, BRCA2 or PALB2 Mutation	II
NCT02849496	A Phase II Open-Label, Randomized Study of PARP Inhibition (Olaparib) Either Alone or in Combination With Anti-PD-L1 Therapy (Atezolizumab; MPDL3280A) in Homologous DNA Repair (HDR) Deficient, Locally Advanced or Metastatic Non-HER2-Positive Breast Cancer.	II
NCT04939662	Phase II, Single-arm Study of Olaparib and Bevacizumab Combination Therapy in Relapsed Small Cell Lung Cancer Subjects With DNA Damage Response and the Repair Pathway Alteration, ATM Deficiency, SLFN11 Positive, or POU2F3 Positive	II
NCT04261465	The NUVOLA TRIAL: Neoadjuvant Chemotherapy in Unresectable Ovarian Cancer With OLAparib and Weekly Carboplatin Plus Paclitaxel: A Phase II Open-label Multicentre Study	II
NCT04633902	Phase II Study of Olaparib in Combination With Pembrolizumab in Patients With Advanced Melanoma With Homologous Recombination (HR) Pathway Gene Mutation	II
NCT03248570	Phase II Open Label Study of Pembrolizumab in Patients With Metastatic Castration Resistant Prostate Cancer (mCRPC) With or Without DNA Damage Repair Defects	II

Disclaimer: The data presented here is from a curated knowledgebase of publicly available information, but may not be exhaustive. The data version is 2021.11(003).

Clinical Trials Summary (continued)

BRCA1 splice site mutation (continued)

NCT ID	Title	Phase
NCT03506997	PERSEUS1: Phase II Trial of the Immune Checkpoint Inhibitor Pembrolizumab for Patients Suffering From Metastatic Prostate Cancer	II
NCT04089189	A Phase II Study to Evaluate the Efficacy, Safety, and Tolerability of IMP4297 Capsules in Subjects With Germline and/or Systemic BRCA1/2 Mutation Positive Advanced Ovarian Cancer With at Least 2 Prior Lines of Standard Systemic Therapy	II
NCT03990896	Evaluation of Talazoparib, a PARP Inhibitor, in Patients With Somatic BRCA Mutant Metastatic Breast Cancer: Genotyping Based Clinical Trial	II
NCT04892693	Talazoparib in Advanced Breast Cancer Patients With Homologous Recombinant Deficiency: A Phase II Clinical and Exploratory Biomarker Study of Talazoparib	II
NCT04740190	Combination Talazoparib - Carboplatin for Recurrent High-grade Glioma With DNA Damage Repair Deficiency (DDRd)	II
NCT04253262	A Phase Ib/II Study of Copanlisib Combined With Rucaparib in Patients With Metastatic Castration-resistant Prostate Cancer	I/II
NCT03685331	Harnessing Olaparib, Palbociclib and Endocrine Therapy: A Phase I/II Trial of Olaparib, Palbociclib and Fulvestrant in Patients With BRCA Mutation-associated, Hormone Receptor-positive, Human Epidermal Growth Factor Receptor 2 (HER2)-Negative Metastatic Breast Cancer (HOPE)	I/II
No NCT ID	An Open-Label, Randomised, Phase I/II Trial Of Rucaparib Combined With Nivolumab + or - Ipilimumab To Augment Response In Homologous Repair Deficient Patients With Relapsed Ovarian, Primary Peritoneal And Fallopian Tube Cancer	I/II
NCT03964532	TALAVE: A Pilot Trial of Induction Talazoparib Followed by Combination of Talazoparib and Avelumab in Advanced Breast Cancer	I/II
NCT04673448	Phase IB Trial of Niraparib and TSR-042 in Patients With BRCA-Mutated Breast, Pancreas or Ovary Cancer	I
NCT04890613	Phase Ib Expansion Study of CX-5461 in Patients With Solid Tumours and BRCA2 and/or PALB2 Mutation	I
NCT04598321	BrUOG 390: Neoadjuvant Treatment With Talazoparib for Women With Newly Diagnosed, Advanced Ovarian Cancer Associated With a Mutation in BRCA1 or BRCA2 (mBRCA)	I
NCT03598270	A Phase III Randomized, Double-blinded Trial of Platinum-based Chemotherapy With or Without Atezolizumab Followed by Niraparib Maintenance With or Without Atezolizumab in Patients With Recurrent Ovarian, Tubal or Peritoneal Cancer and Platinum Treatment-free Interval (TFIp) >6 Months	III
NCT03278717	International Phase III Randomised Study to Evaluate the Efficacy of Maintenance Therapy With Olaparib and Cediranib or Olaparib Alone in Patients With Relapsed Ovarian Cancer Following a Response to Platinum-based Chemotherapy	III
NCT04915755	A Randomized Phase III Double-Blinded Study Comparing the Efficacy and Safety of Niraparib to Placebo in Participants With Either HER2-Negative BRCA-Mutated or Triple-Negative Breast Cancer With Molecular Disease Based on Presence of Circulating Tumor DNA After Definitive Therapy (ZEST)	III
NCT03462342	Combination ATR and PARP Inhibitor (CAPRI) Trial With AZD 6738 and Olaparib in Recurrent Ovarian Cancer	II
No NCT ID	An Investigator-Initiated Phase II Trial Of Niraparib For Patients With BRCA-Mutated Unresectable / Recurrent Biliary Tract, Pancreatic And Other Gastrointestinal Cancer (NIR-B)	II
NCT04716686	Niraparib Monotherapy as Maintain and Recurrent Treatment of Endometrial Serous Carcinoma: A Multi-center, Open-label, Prospective Clinical Study	II

Disclaimer: The data presented here is from a curated knowledgebase of publicly available information, but may not be exhaustive. The data version is 2021.11(003).

Clinical Trials Summary (continued)

BRCA1 splice site mutation (continued)

NCT ID	Title	Phase
NCT04348045	MAZEPPA: Phase II PRODIGE-GERCOR Study to Evaluate MAintenance Therapy With Olaparib or Selumetinib Plus Durvalumab According to BRCAness and KRAS Somatic Status Personalized in Metastatic Pancreatic Adenocarcinoma Patients	II
NCT03025035	Open Label, Phase II Pilot Study of Immune Checkpoint Inhibition With Pembrolizumab in Combination With PARP Inhibition With Olaparib in Advanced BRCA-mutated or HDR-defect Breast Cancers	II
NCT04666740	A Phase II Trial to Evaluate the Safety and Antitumor Activity of Pembrolizumab and OLApaRib (POLAR) Maintenance for Patients With Metastatic Pancreatic Ductal Adenocarcinoma and Homologous Recombination Deficiency and/or Exceptional Treatment Response to Platinum-Based Therapy	II
NCT02264678	A Modular Phase I, Open-Label, Multicentre Study to Assess the Safety, Tolerability, Pharmacokinetics and Preliminary Anti-tumour Activity of Ceralasertib in Combination With Cytotoxic Chemotherapy and/or DNA Damage Repair/Novel Anti-cancer Agents in Patients With Advanced Solid Malignancies	I
NCT04169841	Precision Medicine Phase II Study Evaluating the Efficacy of a Double Immunotherapy by Durvalumab and Tremelimumab Combined With Olaparib in Patients With Solid Cancers and Carriers of Homologous Recombination Repair Genes Mutation in Response or Stable After Olaparib Treatment	II
NCT04030559	Phase II Trial of PARP Inhibitor Niraparib for Men With High Risk Prostate Cancer and DNA Damage Response Defects	II
NCT04940637	A Phase II, Open-Label, Single Arm, Prospective, Multicenter Study of Niraparib Plus Dostarlimab in Patients With Advanced NSCLC and/or MPM, and Positive for PD-L1 Expression and Germline or Somatic Mutations in the HRR Genes	II
NCT05002868	A Multi-center, Open-label, Phase I/Ib Study to Assess the Safety, Pharmacokinetics and Anti-tumor Activity of RP12146, a Poly (ADP-ribose) Polymerase (PARP) Inhibitor, in Patients With Locally Advanced or Metastatic Solid Tumors	I
NCT03395197	A Phase III, Randomized, Double-blind, Placebo-controlled Study Of Talazoparib With Enzalutamide In Metastatic Castration-resistant Prostate Cancer	III
NCT04821622	TALAPRO-3: A Phase III, Randomized, Double-blind, Study of Talazoparib With Enzalutamide Versus Placebo With Enzalutamide in Men With DDR Gene Mutated Metastatic Castration-sensitive Prostate Cancer	III
NCT04038502	An Open-label, Multicenter Phase II Study to Compare the Efficacy of Carboplatin as First-line Followed by Second-line Olaparib Versus Olaparib as First-line Followed by Second-line Carboplatin in the Treatment of Patients With Castration Resistant Prostate Cancer Containing Homologous Recombination Deficiency	II
No NCT ID	Single-arm Exploratory Study for Sintilimab Second-line or Later-line Treatment of Advanced Non-small Cell Lung Cancer with DDR Pathway Gene Mutation	I/II
NCT04095273	A Multicenter, Non-randomized, Open-label Phase Ib Study to Determine the Maximum Tolerated and Recommended Phase II Dose of the ATR Inhibitor BAY1895344 in Combination With Pembrolizumab and to Characterize Its Safety, Tolerability, Pharmacokinetics and Preliminary Anti-tumor Activity in Patients With Advanced Solid Tumors	I
NCT04849364	A Phase II Circulating Tumor DNA Enriched, Genomically Directed Post-neoadjuvant Trial for Patients With Residual Triple Negative Breast Cancer (PERSEVERE)	II

Disclaimer: The data presented here is from a curated knowledgebase of publicly available information, but may not be exhaustive. The data version is 2021.11(003).

Clinical Trials Summary (continued)

BRCA1 truncating mutation

NCT ID	Title	Phase
NCT02029001	A Two-period, Multicenter, Randomized, Open-label, Phase II Study Evaluating the Clinical Benefit of a Maintenance Treatment Targeting Tumor Molecular Alterations in Patients with Progressive Locally-advanced or Metastatic Solid Tumors.	II
NCT03967938	Efficacy of Olaparib in Advanced Cancers Occurring in Patients With Germline Mutations or Somatic Tumor Mutations in Homologous Recombination Genes	II
NCT02693535	Targeted Agent and Profiling Utilization Registry (TAPUR) Study.	II
NCT04171700	A Phase II Multicenter, Open-label Study of Rucaparib as Treatment for Solid Tumors Associated With Deleterious Mutations in Homologous Recombination Repair Genes	II
NCT02286687	Phase II Study of the PARP Inhibitor Talazoparib in Advanced Cancer Patients With Somatic Alterations in BRCA1/2, Mutations/Deletions in Other BRCA Pathway Genes and Germline Mutation in BRCA1/2 (Not Breast or Ovarian Cancer)	II
NCT04550494	A Pharmacodynamics-Driven Trial of Talazoparib, an Oral PARP Inhibitor, in Patients With Advanced Solid Tumors and Aberrations in Genes Involved in DNA Damage Response	II
NCT03842228	A Phase Ib Biomarker-Driven Combination Trial of Copanlisib, Olaparib, and Durvalumab (MEDI4736) in Patients With Advanced Solid Tumors	I
NCT04586335	Open Label, Phase Ib Study to Evaluate the Safety, Tolerability, Pharmacokinetics and Clinical Activity of CYH33, an Oral a-specific PI3K Inhibitor in Combination With Olaparib, an Oral PARP Inhibitor in Patients With Advanced Solid Tumors.	I
NCT03415659	A Phase I, Open-label, Single-center, Single/Multiple-dose, Dose-escalation/Dose-expansion Clinical Study on Tolerance and Pharmacokinetics of HWH340 Tablet in Patients With Advanced Solid Tumors	I
NCT04197713	Phase I Sequential Trial of Agents Against DNA Repair (STAR)	I
NCT03297606	Canadian Profiling and Targeted Agent Utilization Trial (CAPTUR): A Phase II Basket Trial	II
NCT04497116	Phase I/IIa Study of the Safety, Pharmacokinetics, Pharmacodynamics and Preliminary Clinical Activity of RP-3500 Alone or in Combination With Talazoparib in Advanced Solid Tumors With ATR Inhibitor Sensitizing Mutations (TRESR Study)	I/II
NCT04123366	A Phase II Study of Olaparib in Combination With Pembrolizumab in Participants With Previously Treated, Homologous Recombination Repair Mutation (HRRm) and/or Homologous Recombination Deficiency (HRD)-Positive Advanced Cancer	II
NCT03767075	Basket of Baskets: A Modular, Open-label, Phase II, Multicentre Study To Evaluate Targeted Agents in Molecularly Selected Populations With Advanced Solid Tumours	II
NCT04905914	A Phase I/IIa, Open-Label, Safety, Pharmacokinetic, And Preliminary Efficacy Study Of Oral ATRN-119 In Patients With Advanced Solid Tumors	I/II
NCT04901702	A Randomized Phase I/II Study of Talazoparib or Temozolomide in Combination With Onivyde in Children With Recurrent Solid Malignancies and Ewing Sarcoma	I/II
NCT03155620	NCI-COG Pediatric MATCH (Molecular Analysis for Therapy Choice) Screening Protocol	II
NCT03233204	NCI-COG Pediatric MATCH (Molecular Analysis for Therapy Choice)- Phase 2 Subprotocol of Olaparib in Patients With Tumors Harboring Defects in DNA Damage Repair Genes	II
NCT04267939	An Open-label Phase Ib Study to Determine the Maximum Tolerated and/or Recommended Phase II Dose of the ATR Inhibitor BAY 1895344 in Combination With PARP Inhibitor Niraparib, in Patients With Recurrent Advanced Solid Tumors and Ovarian Cancer	I
NCT04693468	Modular Phase IB Hypothesis-Testing, Biomarker-Driven, Talazoparib Combination Trial (TalaCom)	I

Disclaimer: The data presented here is from a curated knowledgebase of publicly available information, but may not be exhaustive. The data version is 2021.11(003).

Clinical Trials Summary (continued)

BRCA1 truncating mutation (continued)

NCT ID	Title	Phase
NCT03810105	A Phase II Study of Olaparib and Durvalumab in Men With Castration Sensitive Biochemically Recurrent Non-Metastatic Prostate Cancer Harboring Mutations in DNA Damage Repair	II
NCT03840967	A Phase II Study Evaluating Safety and Efficacy of Niraparib in Patients With Previously Treated Homologous Recombination (HR) Defective or Loss of Heterozygosity (LOH) High Metastatic Esophageal/Gastroesophageal Junction/Proximal Gastric Adenocarcinoma	II
NCT02975934	TRITON3: A Multicenter, Randomized, Open Label Phase III Study of Rucaparib Versus Physician's Choice of Therapy for Patients With Metastatic Castration Resistant Prostate Cancer Associated With Homologous Recombination Deficiency	III
NCT04336943	Durvalumab (MEDI4736) and Olaparib (AZD2281) for Treatment of Biochemically Recurrent Prostate Cancer in Men Predicted to Have a High Neoantigen Load: A Pilot Study	II
NCT04053322	An International Multicenter Phase II Trial of Durvalumab (MEDI4736) Plus OLAparib Plus Fulvestrant in Metastatic or Locally Advanced ER-positive, HER2-negative Breast Cancer Patients Selected Using Criteria That Predict Sensitivity to Olaparib	II
NCT03012321	BRCAAway: A Randomized Phase II Trial of Abiraterone, Olaparib, or Abiraterone + Olaparib in Patients With Metastatic Castration-Resistant Prostate Cancer With DNA Repair Defects	II
No NCT ID	Phase II Study To Evaluate The Efficacy And Safety Of Niraparib In Recurrent Or Persistent Rare Gynecologic Malignancies With Homologous Recombination Deficiency (JGOG2052)	II
NCT03553004	Niraparib in Metastatic Pancreatic Cancer After Previous Chemotherapy (NIRA-PANC): A Phase II Trial	II
NCT03601923	Phase II Proof-of-Concept Trial Testing the PARP Inhibitor Niraparib in Patients With Pancreatic Cancer Harboring Deficiencies in Homologous Recombination DNA Repair	II
NCT03925350	A Phase II Study of Niraparib in Patients With Advanced Melanoma With Genetic Homologous Recombination (HR) Mutation / Alteration	II
NCT04493060	Phase II Study of Niraparib and TSR-042 in Patients With Germline or Somatic BRCA1/2 and PALB2-Related Pancreatic Cancer	II
NCT04187833	Phase II Trial of Nivolumab in Combination with Talazoparib in Patients with Unresectable or Metastatic Melanoma and Mutations in BRCA or BRCA-ness Genes.	II
NCT03344965	A Phase II Study of Olaparib Monotherapy in Metastatic Breast Cancer Patients With Germline or Somatic Mutations in DNA Repair Genes (Olaparib Expanded)	II
NCT03375307	A Phase II Study of Olaparib (AZD2281) in Patients With Metastatic/Advanced Urothelial Carcinoma With DNA-Repair Defects	II
NCT03742895	A Phase II Study of Olaparib Monotherapy in Participants With Previously Treated, Homologous Recombination Repair Mutation (HRRm) or Homologous Recombination Deficiency (HRD) Positive Advanced Cancer	II
NCT03786796	Phase II Study of Olaparib in Metastatic Renal Cell Carcinoma Patients Harboring a BAP-1 or Other DNA Repair Gene Mutations (ORCHID)	II
NCT04042831	A Phase II Study of Olaparib in Patients With Advanced Biliary Tract Cancer With Aberrant DNA Repair Gene Mutations	II
NCT04515836	Phase II Trial of Olaparib in Homologous Recombination Deficient (HRD) Malignant Mesothelioma	II
NCT04858334	APOLLO: A Randomized Phase II Double-Blind Study of Olaparib Versus Placebo Following Adjuvant Chemotherapy in Patients With Resected Pancreatic Cancer and a Pathogenic BRCA1, BRCA2 or PALB2 Mutation	II

Disclaimer: The data presented here is from a curated knowledgebase of publicly available information, but may not be exhaustive. The data version is 2021.11(003).

Clinical Trials Summary (continued)

BRCA1 truncating mutation (continued)

NCT ID	Title	Phase
NCT02849496	A Phase II Open-Label, Randomized Study of PARP Inhibition (Olaparib) Either Alone or in Combination With Anti-PD-L1 Therapy (Atezolizumab; MPDL3280A) in Homologous DNA Repair (HDR) Deficient, Locally Advanced or Metastatic Non-HER2-Positive Breast Cancer.	II
NCT04939662	Phase II, Single-arm Study of Olaparib and Bevacizumab Combination Therapy in Relapsed Small Cell Lung Cancer Subjects With DNA Damage Response and the Repair Pathway Alteration, ATM Deficiency, SLFN11 Positive, or POU2F3 Positive	II
NCT04261465	The NUVOLA TRIAL: Neoadjuvant Chemotherapy in Unresectable Ovarian Cancer With OLAparib and Weekly Carboplatin Plus Paclitaxel: A Phase II Open-label Multicentre Study	II
NCT04633902	Phase II Study of Olaparib in Combination With Pembrolizumab in Patients With Advanced Melanoma With Homologous Recombination (HR) Pathway Gene Mutation	II
NCT03248570	Phase II Open Label Study of Pembrolizumab in Patients With Metastatic Castration Resistant Prostate Cancer (mCRPC) With or Without DNA Damage Repair Defects	II
NCT03506997	PERSEUS1: Phase II Trial of the Immune Checkpoint Inhibitor Pembrolizumab for Patients Suffering From Metastatic Prostate Cancer	II
NCT04089189	A Phase II Study to Evaluate the Efficacy, Safety, and Tolerability of IMP4297 Capsules in Subjects With Germline and/or Systemic BRCA1/2 Mutation Positive Advanced Ovarian Cancer With at Least 2 Prior Lines of Standard Systemic Therapy	II
NCT03990896	Evaluation of Talazoparib, a PARP Inhibitor, in Patients With Somatic BRCA Mutant Metastatic Breast Cancer: Genotyping Based Clinical Trial	II
NCT04892693	Talazoparib in Advanced Breast Cancer Patients With Homologous Recombinant Deficiency: A Phase II Clinical and Exploratory Biomarker Study of Talazoparib	II
NCT04740190	Combination Talazoparib - Carboplatin for Recurrent High-grade Glioma With DNA Damage Repair Deficiency (DDRd)	II
NCT04253262	A Phase Ib/II Study of Copanlisib Combined With Rucaparib in Patients With Metastatic Castration-resistant Prostate Cancer	I/II
NCT03685331	Harnessing Olaparib, Palbociclib and Endocrine Therapy: A Phase I/II Trial of Olaparib, Palbociclib and Fulvestrant in Patients With BRCA Mutation-associated, Hormone Receptor-positive, Human Epidermal Growth Factor Receptor 2 (HER2)-Negative Metastatic Breast Cancer (HOPE)	I/II
No NCT ID	An Open-Label, Randomised, Phase I/II Trial Of Rucaparib Combined With Nivolumab + or - Ipilimumab To Augment Response In Homologous Repair Deficient Patients With Relapsed Ovarian, Primary Peritoneal And Fallopian Tube Cancer	I/II
NCT03964532	TALAVE: A Pilot Trial of Induction Talazoparib Followed by Combination of Talazoparib and Avelumab in Advanced Breast Cancer	I/II
NCT04673448	Phase IB Trial of Niraparib and TSR-042 in Patients With BRCA-Mutated Breast, Pancreas or Ovary Cancer	I
NCT04890613	Phase Ib Expansion Study of CX-5461 in Patients With Solid Tumours and BRCA2 and/or PALB2 Mutation	I
NCT04598321	BrUOG 390: Neoadjuvant Treatment With Talazoparib for Women With Newly Diagnosed, Advanced Ovarian Cancer Associated With a Mutation in BRCA1 or BRCA2 (mBRCA)	I
NCT03598270	A Phase III Randomized, Double-blinded Trial of Platinum-based Chemotherapy With or Without Atezolizumab Followed by Niraparib Maintenance With or Without Atezolizumab in Patients With Recurrent Ovarian, Tubal or Peritoneal Cancer and Platinum Treatment-free Interval (TFIp) >6 Months	III

Disclaimer: The data presented here is from a curated knowledgebase of publicly available information, but may not be exhaustive. The data version is 2021.11(003).

Clinical Trials Summary (continued)

BRCA1 truncating mutation (continued)

NCT ID	Title	Phase
NCT03278717	International Phase III Randomised Study to Evaluate the Efficacy of Maintenance Therapy With Olaparib and Cediranib or Olaparib Alone in Patients With Relapsed Ovarian Cancer Following a Response to Platinum-based Chemotherapy	III
NCT04915755	A Randomized Phase III Double-Blinded Study Comparing the Efficacy and Safety of Niraparib to Placebo in Participants With Either HER2-Negative BRCA-Mutated or Triple-Negative Breast Cancer With Molecular Disease Based on Presence of Circulating Tumor DNA After Definitive Therapy (ZEST)	III
NCT03462342	Combination ATR and PARP Inhibitor (CAPRI) Trial With AZD 6738 and Olaparib in Recurrent Ovarian Cancer	II
No NCT ID	An Investigator-Initiated Phase II Trial Of Niraparib For Patients With BRCA-Mutated Unresectable / Recurrent Biliary Tract, Pancreatic And Other Gastrointestinal Cancer (NIR-B)	II
NCT04716686	Niraparib Monotherapy as Maintain and Recurrent Treatment of Endometrial Serous Carcinoma: A Multi-center, Open-label, Prospective Clinical Study	II
NCT04348045	MAZEPPA: Phase II PRODIGE-GERCOR Study to Evaluate MAintenance Therapy With Olaparib or Selumetinib Plus Durvalumab According to BRCAness and KRAS Somatic Status Personalized in Metastatic Pancreatic Adenocarcinoma Patients	II
NCT03025035	Open Label, Phase II Pilot Study of Immune Checkpoint Inhibition With Pembrolizumab in Combination With PARP Inhibition With Olaparib in Advanced BRCA-mutated or HDR-defect Breast Cancers	II
NCT04666740	A Phase II Trial to Evaluate the Safety and Antitumor Activity of Pembrolizumab and OLApaRib (POLAR) Maintenance for Patients With Metastatic Pancreatic Ductal Adenocarcinoma and Homologous Recombination Deficiency and/or Exceptional Treatment Response to Platinum-Based Therapy	II
NCT02264678	A Modular Phase I, Open-Label, Multicentre Study to Assess the Safety, Tolerability, Pharmacokinetics and Preliminary Anti-tumour Activity of Ceralasertib in Combination With Cytotoxic Chemotherapy and/or DNA Damage Repair/Novel Anti-cancer Agents in Patients With Advanced Solid Malignancies	I
NCT04169841	Precision Medicine Phase II Study Evaluating the Efficacy of a Double Immunotherapy by Durvalumab and Tremelimumab Combined With Olaparib in Patients With Solid Cancers and Carriers of Homologous Recombination Repair Genes Mutation in Response or Stable After Olaparib Treatment	II
NCT04030559	Phase II Trial of PARP Inhibitor Niraparib for Men With High Risk Prostate Cancer and DNA Damage Response Defects	II
NCT04940637	A Phase II, Open-Label, Single Arm, Prospective, Multicenter Study of Niraparib Plus Dostarlimab in Patients With Advanced NSCLC and/or MPM, and Positive for PD-L1 Expression and Germline or Somatic Mutations in the HRR Genes	II
NCT05002868	A Multi-center, Open-label, Phase I/Ib Study to Assess the Safety, Pharmacokinetics and Anti-tumor Activity of RP12146, a Poly (ADP-ribose) Polymerase (PARP) Inhibitor, in Patients With Locally Advanced or Metastatic Solid Tumors	I
NCT03395197	A Phase III, Randomized, Double-blind, Placebo-controlled Study Of Talazoparib With Enzalutamide In Metastatic Castration-resistant Prostate Cancer	III
NCT04821622	TALAPRO-3: A Phase III, Randomized, Double-blind, Study of Talazoparib With Enzalutamide Versus Placebo With Enzalutamide in Men With DDR Gene Mutated Metastatic Castration-sensitive Prostate Cancer	III
NCT04038502	An Open-label, Multicenter Phase II Study to Compare the Efficacy of Carboplatin as First-line Followed by Second-line Olaparib Versus Olaparib as First-line Followed by Second-line Carboplatin in the Treatment of Patients With Castration Resistant Prostate Cancer Containing Homologous Recombination Deficiency	II

Disclaimer: The data presented here is from a curated knowledgebase of publicly available information, but may not be exhaustive. The data version is 2021.11(003).

Clinical Trials Summary (continued)

BRCA1 truncating mutation (continued)

NCT ID	Title	Phase
No NCT ID	Single-arm Exploratory Study for Sintilimab Second-line or Later-line Treatment of Advanced Non-small Cell Lung Cancer with DDR Pathway Gene Mutation	I/II
NCT04095273	A Multicenter, Non-randomized, Open-label Phase Ib Study to Determine the Maximum Tolerated and Recommended Phase II Dose of the ATR Inhibitor BAY1895344 in Combination With Pembrolizumab and to Characterize Its Safety, Tolerability, Pharmacokinetics and Preliminary Anti-tumor Activity in Patients With Advanced Solid Tumors	I
NCT04849364	A Phase II Circulating Tumor DNA Enriched, Genomically Directed Post-neoadjuvant Trial for Patients With Residual Triple Negative Breast Cancer (PERSEVERE)	II

BRCA1 deletion

NCT ID	Title	Phase
NCT02286687	Phase II Study of the PARP Inhibitor Talazoparib in Advanced Cancer Patients With Somatic Alterations in BRCA1/2, Mutations/Deletions in Other BRCA Pathway Genes and Germline Mutation in BRCA1/2 (Not Breast or Ovarian Cancer)	II
NCT03297606	Canadian Profiling and Targeted Agent Utilization Trial (CAPTUR): A Phase II Basket Trial	II
NCT04497116	Phase I/IIa Study of the Safety, Pharmacokinetics, Pharmacodynamics and Preliminary Clinical Activity of RP-3500 Alone or in Combination With Talazoparib in Advanced Solid Tumors With ATR Inhibitor Sensitizing Mutations (TRESR Study)	I/II
NCT04901702	A Randomized Phase I/II Study of Talazoparib or Temozolomide in Combination With Onivyde in Children With Recurrent Solid Malignancies and Ewing Sarcoma	I/II
NCT03155620	NCI-COG Pediatric MATCH (Molecular Analysis for Therapy Choice) Screening Protocol	II
NCT03233204	NCI-COG Pediatric MATCH (Molecular Analysis for Therapy Choice)- Phase 2 Subprotocol of Olaparib in Patients With Tumors Harboring Defects in DNA Damage Repair Genes	II
NCT04267939	An Open-label Phase Ib Study to Determine the Maximum Tolerated and/or Recommended Phase II Dose of the ATR Inhibitor BAY 1895344 in Combination With PARP Inhibitor Niraparib, in Patients With Recurrent Advanced Solid Tumors and Ovarian Cancer	I
NCT04693468	Modular Phase IB Hypothesis-Testing, Biomarker-Driven, Talazoparib Combination Trial (TalaCom)	I
NCT03810105	A Phase II Study of Olaparib and Durvalumab in Men With Castration Sensitive Biochemically Recurrent Non-Metastatic Prostate Cancer Harboring Mutations in DNA Damage Repair	II
NCT03012321	BRCAAway: A Randomized Phase II Trial of Abiraterone, Olaparib, or Abiraterone + Olaparib in Patients With Metastatic Castration-Resistant Prostate Cancer With DNA Repair Defects	II
NCT03840967	A Phase II Study Evaluating Safety and Efficacy of Niraparib in Patients With Previously Treated Homologous Recombination (HR) Defective or Loss of Heterozygosity (LOH) High Metastatic Esophageal/Gastroesophageal Junction/Proximal Gastric Adenocarcinoma	II
NCT04633902	Phase II Study of Olaparib in Combination With Pembrolizumab in Patients With Advanced Melanoma With Homologous Recombination (HR) Pathway Gene Mutation	II
NCT03248570	Phase II Open Label Study of Pembrolizumab in Patients With Metastatic Castration Resistant Prostate Cancer (mCRPC) With or Without DNA Damage Repair Defects	II
NCT04740190	Combination Talazoparib - Carboplatin for Recurrent High-grade Glioma With DNA Damage Repair Deficiency (DDRd)	II

Disclaimer: The data presented here is from a curated knowledgebase of publicly available information, but may not be exhaustive. The data version is 2021.11(003).

Clinical Trials Summary (continued)

BRCA1 deletion (continued)

NCT ID	Title	Phase
NCT04666740	A Phase II Trial to Evaluate the Safety and Antitumor Activity of Pembrolizumab and OLApaRib (POLAR) Maintenance for Patients With Metastatic Pancreatic Ductal Adenocarcinoma and Homologous Recombination Deficiency and/or Exceptional Treatment Response to Platinum-Based Therapy	II
NCT04030559	Phase II Trial of PARP Inhibitor Niraparib for Men With High Risk Prostate Cancer and DNA Damage Response Defects	II
NCT03344965	A Phase II Study of Olaparib Monotherapy in Metastatic Breast Cancer Patients With Germline or Somatic Mutations in DNA Repair Genes (Olaparib Expanded)	II
NCT03506997	PERSEUS1: Phase II Trial of the Immune Checkpoint Inhibitor Pembrolizumab for Patients Suffering From Metastatic Prostate Cancer	II
NCT04849364	A Phase II Circulating Tumor DNA Enriched, Genomically Directed Post-neoadjuvant Trial for Patients With Residual Triple Negative Breast Cancer (PERSEVERE)	II
NCT05002868	A Multi-center, Open-label, Phase I/Ib Study to Assess the Safety, Pharmacokinetics and Anti-tumor Activity of RP12146, a Poly (ADP-ribose) Polymerase (PARP) Inhibitor, in Patients With Locally Advanced or Metastatic Solid Tumors	I

BRCA1 fusion

NCT ID	Title	Phase
NCT03297606	Canadian Profiling and Targeted Agent Utilization Trial (CAPTUR): A Phase II Basket Trial	II
NCT04497116	Phase I/IIa Study of the Safety, Pharmacokinetics, Pharmacodynamics and Preliminary Clinical Activity of RP-3500 Alone or in Combination With Talazoparib in Advanced Solid Tumors With ATR Inhibitor Sensitizing Mutations (TRESR Study)	I/II
NCT02286687	Phase II Study of the PARP Inhibitor Talazoparib in Advanced Cancer Patients With Somatic Alterations in BRCA1/2, Mutations/Deletions in Other BRCA Pathway Genes and Germline Mutation in BRCA1/2 (Not Breast or Ovarian Cancer)	II
NCT04901702	A Randomized Phase I/II Study of Talazoparib or Temozolomide in Combination With Onivyde in Children With Recurrent Solid Malignancies and Ewing Sarcoma	I/II
NCT03155620	NCI-COG Pediatric MATCH (Molecular Analysis for Therapy Choice) Screening Protocol	II
NCT03233204	NCI-COG Pediatric MATCH (Molecular Analysis for Therapy Choice)- Phase 2 Subprotocol of Olaparib in Patients With Tumors Harboring Defects in DNA Damage Repair Genes	II
NCT04267939	An Open-label Phase Ib Study to Determine the Maximum Tolerated and/or Recommended Phase II Dose of the ATR Inhibitor BAY 1895344 in Combination With PARP Inhibitor Niraparib, in Patients With Recurrent Advanced Solid Tumors and Ovarian Cancer	I
NCT04693468	Modular Phase IB Hypothesis-Testing, Biomarker-Driven, Talazoparib Combination Trial (TalaCom)	I
NCT03840967	A Phase II Study Evaluating Safety and Efficacy of Niraparib in Patients With Previously Treated Homologous Recombination (HR) Defective or Loss of Heterozygosity (LOH) High Metastatic Esophageal/Gastroesophageal Junction/Proximal Gastric Adenocarcinoma	II
NCT02502266	A Randomized Phase II/III Study of the Combination of Cediranib and Olaparib Compared to Cediranib or Olaparib Alone, or Standard of Care Chemotherapy in Women With Recurrent Platinum-Resistant or -Refractory Ovarian, Fallopian Tube, or Primary Peritoneal Cancer (COCOS)	II/III

Clinical Trials Summary (continued)

BRCA1 fusion (continued)

NCT ID	Title	Phase
NCT04739800	A Randomized Phase II Trial of Triplet Therapy (a PD-L1 Inhibitor (Durvalumab) MEDI4736 in Combination With Olaparib and Cediranib) Compared to Olaparib and Cediranib or (Durvalumab) MEDI4736 and Cediranib or Standard of Care Chemotherapy in Women With Platinum-Resistant Recurrent Epithelial Ovarian Cancer, Primary Peritoneal or Fallopian Cancer Who Have Received Prior Bevacizumab	II
NCT04666740	A Phase II Trial to Evaluate the Safety and Antitumor Activity of Pembrolizumab and OLApaRib (POLAR) Maintenance for Patients With Metastatic Pancreatic Ductal Adenocarcinoma and Homologous Recombination Deficiency and/or Exceptional Treatment Response to Platinum-Based Therapy	II
NCT04633902	Phase II Study of Olaparib in Combination With Pembrolizumab in Patients With Advanced Melanoma With Homologous Recombination (HR) Pathway Gene Mutation	II
NCT03506997	PERSEUS1: Phase II Trial of the Immune Checkpoint Inhibitor Pembrolizumab for Patients Suffering From Metastatic Prostate Cancer	II
NCT04849364	A Phase II Circulating Tumor DNA Enriched, Genomically Directed Post-neoadjuvant Trial for Patients With Residual Triple Negative Breast Cancer (PERSEVERE)	II
NCT05002868	A Multi-center, Open-label, Phase I/Ib Study to Assess the Safety, Pharmacokinetics and Anti-tumor Activity of RP12146, a Poly (ADP-ribose) Polymerase (PARP) Inhibitor, in Patients With Locally Advanced or Metastatic Solid Tumors	I

BRCA1 aberration

NCT ID	Title	Phase
NCT03297606	Canadian Profiling and Targeted Agent Utilization Trial (CAPTUR): A Phase II Basket Trial	II
NCT04497116	Phase I/IIa Study of the Safety, Pharmacokinetics, Pharmacodynamics and Preliminary Clinical Activity of RP-3500 Alone or in Combination With Talazoparib in Advanced Solid Tumors With ATR Inhibitor Sensitizing Mutations (TRESR Study)	I/II
NCT02286687	Phase II Study of the PARP Inhibitor Talazoparib in Advanced Cancer Patients With Somatic Alterations in BRCA1/2, Mutations/Deletions in Other BRCA Pathway Genes and Germline Mutation in BRCA1/2 (Not Breast or Ovarian Cancer)	II
NCT04901702	A Randomized Phase I/II Study of Talazoparib or Temozolomide in Combination With Onivyde in Children With Recurrent Solid Malignancies and Ewing Sarcoma	I/II
NCT03155620	NCI-COG Pediatric MATCH (Molecular Analysis for Therapy Choice) Screening Protocol	II
NCT03233204	NCI-COG Pediatric MATCH (Molecular Analysis for Therapy Choice)- Phase 2 Subprotocol of Olaparib in Patients With Tumors Harboring Defects in DNA Damage Repair Genes	II
NCT04267939	An Open-label Phase Ib Study to Determine the Maximum Tolerated and/or Recommended Phase II Dose of the ATR Inhibitor BAY 1895344 in Combination With PARP Inhibitor Niraparib, in Patients With Recurrent Advanced Solid Tumors and Ovarian Cancer	I
NCT04693468	Modular Phase IB Hypothesis-Testing, Biomarker-Driven, Talazoparib Combination Trial (TalaCom)	I
NCT04666740	A Phase II Trial to Evaluate the Safety and Antitumor Activity of Pembrolizumab and OLApaRib (POLAR) Maintenance for Patients With Metastatic Pancreatic Ductal Adenocarcinoma and Homologous Recombination Deficiency and/or Exceptional Treatment Response to Platinum-Based Therapy	II
NCT04633902	Phase II Study of Olaparib in Combination With Pembrolizumab in Patients With Advanced Melanoma With Homologous Recombination (HR) Pathway Gene Mutation	II

Disclaimer: The data presented here is from a curated knowledgebase of publicly available information, but may not be exhaustive. The data version is 2021.11(003).

Clinical Trials Summary (continued)

BRCA1 aberration (continued)

NCT ID	Title	Phase
NCT03506997	PERSEUS1: Phase II Trial of the Immune Checkpoint Inhibitor Pembrolizumab for Patients Suffering From Metastatic Prostate Cancer	II
NCT04849364	A Phase II Circulating Tumor DNA Enriched, Genomically Directed Post-neoadjuvant Trial for Patients With Residual Triple Negative Breast Cancer (PERSEVERE)	II
NCT05002868	A Multi-center, Open-label, Phase I/Ib Study to Assess the Safety, Pharmacokinetics and Anti-tumor Activity of RP12146, a Poly (ADP-ribose) Polymerase (PARP) Inhibitor, in Patients With Locally Advanced or Metastatic Solid Tumors	I

Disclaimer: The data presented here is from a curated knowledgebase of publicly available information, but may not be exhaustive. The data version is 2021.11(003).

Sample Cancer Type: Unknown Primary Origin

Table of Contents

Table of Contents	Page
Relevant Therapy Details	2
Alert Details	26
Clinical Trials	27
Relevant Therapy Summary	113
Clinical Trials Summary	120

Report Highlights

6 Relevant Biomarkers
4 Therapies Available
80 Clinical Trials

Relevant Biomarkers

Genomic Alteration	Relevant Therapies (In this cancer type)	Relevant Therapies (In other cancer type)	Clinical Trials
BRCA2 mutation<br>**Prognostic significance:** None<br>**Diagnostic significance:** None	None	**bevacizumab + olaparib** [1, 2]<br>**niraparib** [1]<br>**olaparib** [1, 2]<br>**rucaparib** [1, 2]	78
BRCA2 splice site mutation<br>**Prognostic significance:** None<br>**Diagnostic significance:** None	None	**bevacizumab + olaparib** [1, 2]<br>**niraparib** [1]<br>**olaparib** [1, 2]<br>**rucaparib** [1, 2]	78
BRCA2 truncating mutation<br>**Prognostic significance:** None<br>**Diagnostic significance:** None	None	**bevacizumab + olaparib** [1, 2]<br>**niraparib** [1]<br>**olaparib** [1, 2]<br>**rucaparib** [1, 2]	78
BRCA2 deletion<br>**Prognostic significance:** None<br>**Diagnostic significance:** None	None	None	21
BRCA2 fusion<br>**Prognostic significance:** None<br>**Diagnostic significance:** None	None	None	16
BRCA2 aberration<br>**Prognostic significance:** None<br>**Diagnostic significance:** None	None	None	13

Public data sources included in relevant therapies: FDA1, NCCN, **EMA**2, ESMO
Public data sources included in prognostic and diagnostic significance: NCCN, ESMO

Disclaimer: The data presented here is from a curated knowledgebase of publicly available information, but may not be exhaustive. The data version is 2021.11(003).The content of this report has not been evaluated or approved by the FDA or other regulatory agencies.

Relevant Therapy Details

Current FDA Information

● In this cancer type ○ In other cancer type ◐ In this cancer type and other cancer types

FDA information is current as of 2021-10-13. For the most up-to-date information, search www.fda.gov.

BRCA2 mutation

○ niraparib

Cancer type: Ovarian Cancer **Label as of:** 2021-07-27 **Variant class:** BRCA2 mutation

Indications and usage:

ZEJULA® is a poly (ADP-ribose) polymerase (PARP) inhibitor indicated:

- for the maintenance treatment of adult patients with advanced epithelial ovarian, fallopian tube, or primary peritoneal cancer who are in a complete or partial response to first-line platinum-based chemotherapy.
- for the maintenance treatment of adult patients with recurrent epithelial ovarian, fallopian tube, or primary peritoneal cancer who are in a complete or partial response to platinum-based chemotherapy.
- for the treatment of adult patients with advanced ovarian, fallopian tube, or primary peritoneal cancer who have been treated with 3 or more prior chemotherapy regimens and whose cancer is associated with homologous recombination deficiency (HRD) positive status defined by either:
  - a deleterious or suspected deleterious BRCA mutation, or
  - genomic instability and who have progressed more than 6 months after response to the last platinum-based chemotherapy.

Select patients for therapy based on an FDA-approved companion diagnostic for ZEJULA®.

Reference:

https://www.accessdata.fda.gov/drugsatfda_docs/label/2021/208447s022s024lbl.pdf

Disclaimer: The data presented here is from a curated knowledgebase of publicly available information, but may not be exhaustive. The data version is 2021.11(003).

BRCA2 mutation (continued)

○ olaparib, bevacizumab + olaparib

Cancer type: Castration-Resistant Prostate Cancer, Ovarian Cancer **Label as of:** 2021-03-11 **Variant class:** BRCA2 mutation

Indications and usage:

LYNPARZA® is a poly (ADP-ribose) polymerase (PARP) inhibitor indicated:

Ovarian cancer

- for the maintenance treatment of adult patients with deleterious or suspected deleterious germline or somatic BRCA-mutated advanced epithelial ovarian, fallopian tube or primary peritoneal cancer who are in complete or partial response to first-line platinum-based chemotherapy. Select patients for therapy based on an FDA-approved companion diagnostic for LYNPARZA®.
- in combination with bevacizumab for the maintenance treatment of adult patients with advanced epithelial ovarian, fallopian tube or primary peritoneal cancer who are in complete or partial response to first-line platinum-based chemotherapy and whose cancer is associated with homologous recombination deficiency (HRD)-positive status defined by either:
  - a deleterious or suspected deleterious BRCA mutation, and/or
  - genomic instability.

Select patients for therapy based on an FDA-approved companion diagnostic for LYNPARZA®.

- for the maintenance treatment of adult patients with recurrent epithelial ovarian, fallopian tube or primary peritoneal cancer, who are in complete or partial response to platinum-based chemotherapy.
- for the treatment of adult patients with deleterious or suspected deleterious germline BRCA-mutated (gBRCAm) advanced ovarian cancer who have been treated with three or more prior lines of chemotherapy. Select patients for therapy based on an FDA-approved companion diagnostic for LYNPARZA®.

Breast cancer

- for the treatment of adult patients with deleterious or suspected deleterious gBRCAm, HER2-negative metastatic breast cancer who have been treated with chemotherapy in the neoadjuvant, adjuvant or metastatic setting. Patients with hormone receptor (HR)-positive breast cancer should have been treated with a prior endocrine therapy or be considered inappropriate for endocrine therapy. Select patients for therapy based on an FDA-approved companion diagnostic for LYNPARZA®.

Pancreatic cancer

- for the maintenance treatment of adult patients with deleterious or suspected deleterious gBRCAm metastatic pancreatic adenocarcinoma whose disease has not progressed on at least 16 weeks of a first-line platinum-based chemotherapy regimen. Select patients for therapy based on an FDA-approved companion diagnostic for LYNPARZA®.

Prostate cancer

- for the treatment of adult patients with deleterious or suspected deleterious germline or somatic homologous recombination repair (HRR) gene-mutated metastatic castration-resistant prostate cancer (mCRPC) who have progressed following prior treatment with enzalutamide or abiraterone. Select patients for therapy based on an FDA-approved companion diagnostic for LYNPARZA®.

Reference:

https://www.accessdata.fda.gov/drugsatfda_docs/label/2021/208558s019s020lbl.pdf

BRCA2 mutation (continued)

rucaparib

Cancer type: Castration-Resistant Prostate Cancer, Ovarian Cancer **Label as of:** 2021-09-30 **Variant class:** BRCA2 mutation

Indications and usage:

RUBRACA® is a poly (ADP-ribose) polymerase (PARP) inhibitor indicated:

Ovarian cancer

- for the maintenance treatment of adult patients with recurrent epithelial ovarian, fallopian tube, or primary peritoneal cancer who are in a complete or partial response to platinum-based chemotherapy.
- for the treatment of adult patients with a deleterious BRCA mutation (germline and/or somatic)-associated epithelial ovarian, fallopian tube, or primary peritoneal cancer who have been treated with two or more chemotherapies. Select patients for therapy based on an FDA-approved companion diagnostic for RUBRACA®.

Prostate cancer

- for the treatment of adult patients with a deleterious BRCA mutation (germline and/or somatic)-associated metastatic castration-resistant prostate cancer (mCRPC) who have been treated with androgen receptor-directed therapy and a taxane-based chemotherapy. Select patients for therapy based on an FDA-approved companion diagnostic for RUBRACA®.

This indication is approved under accelerated approval based on objective response rate and duration of response. Continued approval for this indication may be contingent upon verification and description of clinical benefit in confirmatory trials.

Reference:

https://www.accessdata.fda.gov/drugsatfda_docs/label/2021/209115s009lbl.pdf

BRCA2 splice site mutation

niraparib

Cancer type: Ovarian Cancer **Label as of:** 2021-07-27 **Variant class:** BRCA2 mutation

Indications and usage:

ZEJULA® is a poly (ADP-ribose) polymerase (PARP) inhibitor indicated:

- for the maintenance treatment of adult patients with advanced epithelial ovarian, fallopian tube, or primary peritoneal cancer who are in a complete or partial response to first-line platinum-based chemotherapy.
- for the maintenance treatment of adult patients with recurrent epithelial ovarian, fallopian tube, or primary peritoneal cancer who are in a complete or partial response to platinum-based chemotherapy.
- for the treatment of adult patients with advanced ovarian, fallopian tube, or primary peritoneal cancer who have been treated with 3 or more prior chemotherapy regimens and whose cancer is associated with homologous recombination deficiency (HRD) positive status defined by either:
  - a deleterious or suspected deleterious BRCA mutation, or
  - genomic instability and who have progressed more than 6 months after response to the last platinum-based chemotherapy.

Select patients for therapy based on an FDA-approved companion diagnostic for ZEJULA®.

Reference:

https://www.accessdata.fda.gov/drugsatfda_docs/label/2021/208447s022s024lbl.pdf

Disclaimer: The data presented here is from a curated knowledgebase of publicly available information, but may not be exhaustive. The data version is 2021.11(003).

BRCA2 splice site mutation (continued)

olaparib, bevacizumab + olaparib

Cancer type: Castration-Resistant Prostate Cancer, Ovarian Cancer **Label as of:** 2021-03-11 **Variant class:** BRCA2 mutation

Indications and usage:

LYNPARZA® is a poly (ADP-ribose) polymerase (PARP) inhibitor indicated:

Ovarian cancer

- for the maintenance treatment of adult patients with deleterious or suspected deleterious germline or somatic BRCA-mutated advanced epithelial ovarian, fallopian tube or primary peritoneal cancer who are in complete or partial response to first-line platinum-based chemotherapy. Select patients for therapy based on an FDA-approved companion diagnostic for LYNPARZA®.
- in combination with bevacizumab for the maintenance treatment of adult patients with advanced epithelial ovarian, fallopian tube or primary peritoneal cancer who are in complete or partial response to first-line platinum-based chemotherapy and whose cancer is associated with homologous recombination deficiency (HRD)-positive status defined by either:
  - a deleterious or suspected deleterious BRCA mutation, and/or
  - genomic instability.

Select patients for therapy based on an FDA-approved companion diagnostic for LYNPARZA®.

- for the maintenance treatment of adult patients with recurrent epithelial ovarian, fallopian tube or primary peritoneal cancer, who are in complete or partial response to platinum-based chemotherapy.
- for the treatment of adult patients with deleterious or suspected deleterious germline BRCA-mutated (gBRCAm) advanced ovarian cancer who have been treated with three or more prior lines of chemotherapy. Select patients for therapy based on an FDA-approved companion diagnostic for LYNPARZA®.

Breast cancer

- for the treatment of adult patients with deleterious or suspected deleterious gBRCAm, HER2-negative metastatic breast cancer who have been treated with chemotherapy in the neoadjuvant, adjuvant or metastatic setting. Patients with hormone receptor (HR)-positive breast cancer should have been treated with a prior endocrine therapy or be considered inappropriate for endocrine therapy. Select patients for therapy based on an FDA-approved companion diagnostic for LYNPARZA®.

Pancreatic cancer

- for the maintenance treatment of adult patients with deleterious or suspected deleterious gBRCAm metastatic pancreatic adenocarcinoma whose disease has not progressed on at least 16 weeks of a first-line platinum-based chemotherapy regimen. Select patients for therapy based on an FDA-approved companion diagnostic for LYNPARZA®.

Prostate cancer

- for the treatment of adult patients with deleterious or suspected deleterious germline or somatic homologous recombination repair (HRR) gene-mutated metastatic castration-resistant prostate cancer (mCRPC) who have progressed following prior treatment with enzalutamide or abiraterone. Select patients for therapy based on an FDA-approved companion diagnostic for LYNPARZA®.

Reference:

https://www.accessdata.fda.gov/drugsatfda_docs/label/2021/208558s019s020lbl.pdf

Disclaimer: The data presented here is from a curated knowledgebase of publicly available information, but may not be exhaustive. The data version is 2021.11(003).

BRCA2 splice site mutation (continued)

rucaparib

Cancer type: Castration-Resistant Prostate Cancer, Ovarian Cancer **Label as of:** 2021-09-30 **Variant class:** BRCA2 mutation

Indications and usage:

RUBRACA® is a poly (ADP-ribose) polymerase (PARP) inhibitor indicated:

Ovarian cancer

- for the maintenance treatment of adult patients with recurrent epithelial ovarian, fallopian tube, or primary peritoneal cancer who are in a complete or partial response to platinum-based chemotherapy.
- for the treatment of adult patients with a deleterious BRCA mutation (germline and/or somatic)-associated epithelial ovarian, fallopian tube, or primary peritoneal cancer who have been treated with two or more chemotherapies. Select patients for therapy based on an FDA-approved companion diagnostic for RUBRACA®.

Prostate cancer

- for the treatment of adult patients with a deleterious BRCA mutation (germline and/or somatic)-associated metastatic castration-resistant prostate cancer (mCRPC) who have been treated with androgen receptor-directed therapy and a taxane-based chemotherapy. Select patients for therapy based on an FDA-approved companion diagnostic for RUBRACA®.

This indication is approved under accelerated approval based on objective response rate and duration of response. Continued approval for this indication may be contingent upon verification and description of clinical benefit in confirmatory trials.

Reference:

https://www.accessdata.fda.gov/drugsatfda_docs/label/2021/209115s009lbl.pdf

BRCA2 truncating mutation

niraparib

Cancer type: Ovarian Cancer **Label as of:** 2021-07-27 **Variant class:** BRCA2 mutation

Indications and usage:

ZEJULA® is a poly (ADP-ribose) polymerase (PARP) inhibitor indicated:

- for the maintenance treatment of adult patients with advanced epithelial ovarian, fallopian tube, or primary peritoneal cancer who are in a complete or partial response to first-line platinum-based chemotherapy.
- for the maintenance treatment of adult patients with recurrent epithelial ovarian, fallopian tube, or primary peritoneal cancer who are in a complete or partial response to platinum-based chemotherapy.
- for the treatment of adult patients with advanced ovarian, fallopian tube, or primary peritoneal cancer who have been treated with 3 or more prior chemotherapy regimens and whose cancer is associated with homologous recombination deficiency (HRD) positive status defined by either:
  - a deleterious or suspected deleterious BRCA mutation, or
  - genomic instability and who have progressed more than 6 months after response to the last platinum-based chemotherapy.

Select patients for therapy based on an FDA-approved companion diagnostic for ZEJULA®.

Reference:

https://www.accessdata.fda.gov/drugsatfda_docs/label/2021/208447s022s024lbl.pdf

BRCA2 truncating mutation (continued)

○ olaparib, bevacizumab + olaparib

Cancer type: Castration-Resistant Prostate Cancer, Ovarian Cancer **Label as of:** 2021-03-11 **Variant class:** BRCA2 mutation

Indications and usage:

LYNPARZA® is a poly (ADP-ribose) polymerase (PARP) inhibitor indicated:

Ovarian cancer

- for the maintenance treatment of adult patients with deleterious or suspected deleterious germline or somatic BRCA-mutated advanced epithelial ovarian, fallopian tube or primary peritoneal cancer who are in complete or partial response to first-line platinum-based chemotherapy. Select patients for therapy based on an FDA-approved companion diagnostic for LYNPARZA®.
- in combination with bevacizumab for the maintenance treatment of adult patients with advanced epithelial ovarian, fallopian tube or primary peritoneal cancer who are in complete or partial response to first-line platinum-based chemotherapy and whose cancer is associated with homologous recombination deficiency (HRD)-positive status defined by either:
  - a deleterious or suspected deleterious BRCA mutation, and/or
  - genomic instability.

Select patients for therapy based on an FDA-approved companion diagnostic for LYNPARZA®.

- for the maintenance treatment of adult patients with recurrent epithelial ovarian, fallopian tube or primary peritoneal cancer, who are in complete or partial response to platinum-based chemotherapy.
- for the treatment of adult patients with deleterious or suspected deleterious germline BRCA-mutated (gBRCAm) advanced ovarian cancer who have been treated with three or more prior lines of chemotherapy. Select patients for therapy based on an FDA-approved companion diagnostic for LYNPARZA®.

Breast cancer

- for the treatment of adult patients with deleterious or suspected deleterious gBRCAm, HER2-negative metastatic breast cancer who have been treated with chemotherapy in the neoadjuvant, adjuvant or metastatic setting. Patients with hormone receptor (HR)-positive breast cancer should have been treated with a prior endocrine therapy or be considered inappropriate for endocrine therapy. Select patients for therapy based on an FDA-approved companion diagnostic for LYNPARZA®.

Pancreatic cancer

- for the maintenance treatment of adult patients with deleterious or suspected deleterious gBRCAm metastatic pancreatic adenocarcinoma whose disease has not progressed on at least 16 weeks of a first-line platinum-based chemotherapy regimen. Select patients for therapy based on an FDA-approved companion diagnostic for LYNPARZA®.

Prostate cancer

- for the treatment of adult patients with deleterious or suspected deleterious germline or somatic homologous recombination repair (HRR) gene-mutated metastatic castration-resistant prostate cancer (mCRPC) who have progressed following prior treatment with enzalutamide or abiraterone. Select patients for therapy based on an FDA-approved companion diagnostic for LYNPARZA®.

Reference:

https://www.accessdata.fda.gov/drugsatfda_docs/label/2021/208558s019s020lbl.pdf

Disclaimer: The data presented here is from a curated knowledgebase of publicly available information, but may not be exhaustive. The data version is 2021.11(003).

BRCA2 truncating mutation (continued)

rucaparib

Cancer type: Castration-Resistant Prostate Cancer, Ovarian Cancer **Label as of:** 2021-09-30 **Variant class:** BRCA2 mutation

Indications and usage:

RUBRACA® is a poly (ADP-ribose) polymerase (PARP) inhibitor indicated:

Ovarian cancer

- for the maintenance treatment of adult patients with recurrent epithelial ovarian, fallopian tube, or primary peritoneal cancer who are in a complete or partial response to platinum-based chemotherapy.
- for the treatment of adult patients with a deleterious BRCA mutation (germline and/or somatic)-associated epithelial ovarian, fallopian tube, or primary peritoneal cancer who have been treated with two or more chemotherapies. Select patients for therapy based on an FDA-approved companion diagnostic for RUBRACA®.

Prostate cancer

- for the treatment of adult patients with a deleterious BRCA mutation (germline and/or somatic)-associated metastatic castration-resistant prostate cancer (mCRPC) who have been treated with androgen receptor-directed therapy and a taxane-based chemotherapy. Select patients for therapy based on an FDA-approved companion diagnostic for RUBRACA®.

This indication is approved under accelerated approval based on objective response rate and duration of response. Continued approval for this indication may be contingent upon verification and description of clinical benefit in confirmatory trials.

Reference:

https://www.accessdata.fda.gov/drugsatfda_docs/label/2021/209115s009lbl.pdf

Current NCCN Information

● In this cancer type ○ In other cancer type ◐ In this cancer type and other cancer types

NCCN information is current as of 2021-10-01. For the most up-to-date information, search www.nccn.org.
For NCCN International Adaptations & Translations, search www.nccn.org/global/international_adaptations.aspx.

BRCA2 mutation

○ bevacizumab + olaparib

Cancer type: Ovarian Cancer **Variant class:** BRCA2 mutation

NCCN Recommendation category: 1

Population segment (Line of therapy):

- Epithelial, Fallopian Tube, Primary Peritoneal; Stage II, Stage III, Stage IV; Partial response, Complete response (Maintenance therapy)

Reference: NCCN Guidelines® - NCCN-Ovarian Cancer [Version 3.2021]

○ niraparib

Cancer type: Ovarian Cancer **Variant class:** BRCA2 mutation

NCCN Recommendation category: 1

Population segment (Line of therapy):

- Epithelial, Fallopian Tube, Primary Peritoneal; Persistent, Recurrent, Partial response, Complete response (Maintenance therapy); Useful in certain circumstances

Reference: NCCN Guidelines® - NCCN-Ovarian Cancer [Version 3.2021]

○ niraparib

Cancer type: Ovarian Cancer **Variant class:** BRCA2 mutation

NCCN Recommendation category: 1

Population segment (Line of therapy):

- Epithelial, Fallopian Tube, Primary Peritoneal; Stage II, Stage III, Stage IV; Partial response, Complete response (Maintenance therapy)

Reference: NCCN Guidelines® - NCCN-Ovarian Cancer [Version 3.2021]

○ olaparib

Cancer type: Ovarian Cancer **Variant class:** BRCA2 mutation

NCCN Recommendation category: 1

Population segment (Line of therapy):

- Epithelial, Fallopian Tube, Primary Peritoneal; Persistent, Recurrent, Partial response, Complete response (Maintenance therapy); Useful in certain circumstances

Reference: NCCN Guidelines® - NCCN-Ovarian Cancer [Version 3.2021]

Disclaimer: The data presented here is from a curated knowledgebase of publicly available information, but may not be exhaustive. The data version is 2021.11(003).

BRCA2 mutation (continued)

olaparib

Cancer type: Ovarian Cancer **Variant class:** BRCA2 mutation

NCCN Recommendation category: 1

Population segment (Line of therapy):
- Epithelial, Fallopian Tube, Primary Peritoneal; Stage II, Stage III, Stage IV; Partial response, Complete response (Maintenance therapy)

Reference: NCCN Guidelines® - NCCN-Ovarian Cancer [Version 3.2021]

olaparib

Cancer type: Castration-Resistant Prostate Cancer **Variant class:** BRCA2 mutation

NCCN Recommendation category: 1

Population segment (Line of therapy):
- Adenocarcinoma; Metastatic (Subsequent therapy); Useful in certain circumstances

Reference: NCCN Guidelines® - NCCN-Prostate Cancer [Version 1.2022]

rucaparib

Cancer type: Ovarian Cancer **Variant class:** BRCA2 mutation

NCCN Recommendation category: 1

Population segment (Line of therapy):
- Epithelial, Fallopian Tube, Primary Peritoneal; Persistent, Recurrent, Partial response, Complete response (Maintenance therapy); Useful in certain circumstances

Reference: NCCN Guidelines® - NCCN-Ovarian Cancer [Version 3.2021]

niraparib

Cancer type: Ovarian Cancer **Variant class:** BRCA2 mutation

NCCN Recommendation category: 2A

Population segment (Line of therapy):
- Epithelial, Fallopian Tube, Primary Peritoneal; Stage II, Stage III, Stage IV; Partial response, Complete response (Maintenance therapy)

Reference: NCCN Guidelines® - NCCN-Ovarian Cancer [Version 3.2021]

Disclaimer: The data presented here is from a curated knowledgebase of publicly available information, but may not be exhaustive. The data version is 2021.11(003).

BRCA2 mutation (continued)

niraparib

Cancer type: Ovarian Cancer **Variant class:** BRCA2 mutation

NCCN Recommendation category: 2A

Population segment (Line of therapy):
- Epithelial, Less Common Ovarian Cancers, Fallopian Tube, Primary Peritoneal; Recurrent (Recurrence therapy); Preferred intervention

Reference: NCCN Guidelines® - NCCN-Ovarian Cancer [Version 3.2021]

olaparib

Cancer type: Ovarian Cancer **Variant class:** BRCA2 mutation

NCCN Recommendation category: 2A

Population segment (Line of therapy):
- Epithelial, Fallopian Tube, Primary Peritoneal; Stage II, Stage III, Stage IV; Partial response, Complete response (Maintenance therapy)

Reference: NCCN Guidelines® - NCCN-Ovarian Cancer [Version 3.2021]

rucaparib

Cancer type: Ovarian Cancer **Variant class:** BRCA2 mutation

NCCN Recommendation category: 2A

Population segment (Line of therapy):
- Epithelial, Less Common Ovarian Cancers, Fallopian Tube, Primary Peritoneal; Recurrent (Recurrence therapy); Preferred intervention

Reference: NCCN Guidelines® - NCCN-Ovarian Cancer [Version 3.2021]

rucaparib

Cancer type: Castration-Resistant Prostate Cancer **Variant class:** BRCA2 mutation

NCCN Recommendation category: 2A

Population segment (Line of therapy):
- Adenocarcinoma; Metastatic (Subsequent therapy); Useful in certain circumstances

Reference: NCCN Guidelines® - NCCN-Prostate Cancer [Version 1.2022]

Disclaimer: The data presented here is from a curated knowledgebase of publicly available information, but may not be exhaustive. The data version is 2021.11(003).

BRCA2 mutation (continued)

olaparib

Cancer type: Castration-Resistant Prostate Cancer **Variant class:** BRCA2 mutation

NCCN Recommendation category: 2B

Population segment (Line of therapy):
- Adenocarcinoma; Visceral Metastases (Subsequent therapy); Useful in certain circumstances

Reference: NCCN Guidelines® - NCCN-Prostate Cancer [Version 1.2022]

rucaparib

Cancer type: Castration-Resistant Prostate Cancer **Variant class:** BRCA2 mutation

NCCN Recommendation category: 2B

Population segment (Line of therapy):
- Adenocarcinoma; Visceral Metastases (Subsequent therapy); Useful in certain circumstances

Reference: NCCN Guidelines® - NCCN-Prostate Cancer [Version 1.2022]

BRCA2 splice site mutation

bevacizumab + olaparib

Cancer type: Ovarian Cancer **Variant class:** BRCA2 mutation

NCCN Recommendation category: 1

Population segment (Line of therapy):
- Epithelial, Fallopian Tube, Primary Peritoneal; Stage II, Stage III, Stage IV; Partial response, Complete response (Maintenance therapy)

Reference: NCCN Guidelines® - NCCN-Ovarian Cancer [Version 3.2021]

niraparib

Cancer type: Ovarian Cancer **Variant class:** BRCA2 mutation

NCCN Recommendation category: 1

Population segment (Line of therapy):
- Epithelial, Fallopian Tube, Primary Peritoneal; Persistent, Recurrent, Partial response, Complete response (Maintenance therapy); Useful in certain circumstances

Reference: NCCN Guidelines® - NCCN-Ovarian Cancer [Version 3.2021]

BRCA2 splice site mutation (continued)

niraparib

Cancer type: Ovarian Cancer **Variant class:** BRCA2 mutation

NCCN Recommendation category: 1

Population segment (Line of therapy):
- Epithelial, Fallopian Tube, Primary Peritoneal; Stage II, Stage III, Stage IV; Partial response, Complete response (Maintenance therapy)

Reference: NCCN Guidelines® - NCCN-Ovarian Cancer [Version 3.2021]

---

olaparib

Cancer type: Ovarian Cancer **Variant class:** BRCA2 mutation

NCCN Recommendation category: 1

Population segment (Line of therapy):
- Epithelial, Fallopian Tube, Primary Peritoneal; Persistent, Recurrent, Partial response, Complete response (Maintenance therapy); Useful in certain circumstances

Reference: NCCN Guidelines® - NCCN-Ovarian Cancer [Version 3.2021]

---

olaparib

Cancer type: Ovarian Cancer **Variant class:** BRCA2 mutation

NCCN Recommendation category: 1

Population segment (Line of therapy):
- Epithelial, Fallopian Tube, Primary Peritoneal; Stage II, Stage III, Stage IV; Partial response, Complete response (Maintenance therapy)

Reference: NCCN Guidelines® - NCCN-Ovarian Cancer [Version 3.2021]

---

olaparib

Cancer type: Castration-Resistant Prostate Cancer **Variant class:** BRCA2 mutation

NCCN Recommendation category: 1

Population segment (Line of therapy):
- Adenocarcinoma; Metastatic (Subsequent therapy); Useful in certain circumstances

Reference: NCCN Guidelines® - NCCN-Prostate Cancer [Version 1.2022]

Disclaimer: The data presented here is from a curated knowledgebase of publicly available information, but may not be exhaustive. The data version is 2021.11(003).

BRCA2 splice site mutation (continued)

rucaparib

Cancer type: Ovarian Cancer **Variant class:** BRCA2 mutation

NCCN Recommendation category: 1

Population segment (Line of therapy):
- Epithelial, Fallopian Tube, Primary Peritoneal; Persistent, Recurrent, Partial response, Complete response (Maintenance therapy); Useful in certain circumstances

Reference: NCCN Guidelines® - NCCN-Ovarian Cancer [Version 3.2021]

niraparib

Cancer type: Ovarian Cancer **Variant class:** BRCA2 mutation

NCCN Recommendation category: 2A

Population segment (Line of therapy):
- Epithelial, Fallopian Tube, Primary Peritoneal; Stage II, Stage III, Stage IV; Partial response, Complete response (Maintenance therapy)

Reference: NCCN Guidelines® - NCCN-Ovarian Cancer [Version 3.2021]

niraparib

Cancer type: Ovarian Cancer **Variant class:** BRCA2 mutation

NCCN Recommendation category: 2A

Population segment (Line of therapy):
- Epithelial, Less Common Ovarian Cancers, Fallopian Tube, Primary Peritoneal; Recurrent (Recurrence therapy); Preferred intervention

Reference: NCCN Guidelines® - NCCN-Ovarian Cancer [Version 3.2021]

olaparib

Cancer type: Ovarian Cancer **Variant class:** BRCA2 mutation

NCCN Recommendation category: 2A

Population segment (Line of therapy):
- Epithelial, Fallopian Tube, Primary Peritoneal; Stage II, Stage III, Stage IV; Partial response, Complete response (Maintenance therapy)

Reference: NCCN Guidelines® - NCCN-Ovarian Cancer [Version 3.2021]

Disclaimer: The data presented here is from a curated knowledgebase of publicly available information, but may not be exhaustive. The data version is 2021.11(003).

BRCA2 splice site mutation (continued)

○ rucaparib

Cancer type: Ovarian Cancer **Variant class:** BRCA2 mutation

NCCN Recommendation category: 2A

Population segment (Line of therapy):
- Epithelial, Less Common Ovarian Cancers, Fallopian Tube, Primary Peritoneal; Recurrent (Recurrence therapy); Preferred intervention

Reference: NCCN Guidelines® - NCCN-Ovarian Cancer [Version 3.2021]

○ rucaparib

Cancer type: Castration-Resistant Prostate Cancer **Variant class:** BRCA2 mutation

NCCN Recommendation category: 2A

Population segment (Line of therapy):
- Adenocarcinoma; Metastatic (Subsequent therapy); Useful in certain circumstances

Reference: NCCN Guidelines® - NCCN-Prostate Cancer [Version 1.2022]

○ olaparib

Cancer type: Castration-Resistant Prostate Cancer **Variant class:** BRCA2 mutation

NCCN Recommendation category: 2B

Population segment (Line of therapy):
- Adenocarcinoma; Visceral Metastases (Subsequent therapy); Useful in certain circumstances

Reference: NCCN Guidelines® - NCCN-Prostate Cancer [Version 1.2022]

○ rucaparib

Cancer type: Castration-Resistant Prostate Cancer **Variant class:** BRCA2 mutation

NCCN Recommendation category: 2B

Population segment (Line of therapy):
- Adenocarcinoma; Visceral Metastases (Subsequent therapy); Useful in certain circumstances

Reference: NCCN Guidelines® - NCCN-Prostate Cancer [Version 1.2022]

Disclaimer: The data presented here is from a curated knowledgebase of publicly available information, but may not be exhaustive. The data version is 2021.11(003).

BRCA2 truncating mutation

○ bevacizumab + olaparib

Cancer type: Ovarian Cancer **Variant class:** BRCA2 mutation

NCCN Recommendation category: 1

Population segment (Line of therapy):

- Epithelial, Fallopian Tube, Primary Peritoneal; Stage II, Stage III, Stage IV; Partial response, Complete response (Maintenance therapy)

Reference: NCCN Guidelines® - NCCN-Ovarian Cancer [Version 3.2021]

○ niraparib

Cancer type: Ovarian Cancer **Variant class:** BRCA2 mutation

NCCN Recommendation category: 1

Population segment (Line of therapy):

- Epithelial, Fallopian Tube, Primary Peritoneal; Persistent, Recurrent, Partial response, Complete response (Maintenance therapy); Useful in certain circumstances

Reference: NCCN Guidelines® - NCCN-Ovarian Cancer [Version 3.2021]

○ niraparib

Cancer type: Ovarian Cancer **Variant class:** BRCA2 mutation

NCCN Recommendation category: 1

Population segment (Line of therapy):

- Epithelial, Fallopian Tube, Primary Peritoneal; Stage II, Stage III, Stage IV; Partial response, Complete response (Maintenance therapy)

Reference: NCCN Guidelines® - NCCN-Ovarian Cancer [Version 3.2021]

○ olaparib

Cancer type: Ovarian Cancer **Variant class:** BRCA2 mutation

NCCN Recommendation category: 1

Population segment (Line of therapy):

- Epithelial, Fallopian Tube, Primary Peritoneal; Persistent, Recurrent, Partial response, Complete response (Maintenance therapy); Useful in certain circumstances

Reference: NCCN Guidelines® - NCCN-Ovarian Cancer [Version 3.2021]

Disclaimer: The data presented here is from a curated knowledgebase of publicly available information, but may not be exhaustive. The data version is 2021.11(003).

BRCA2 truncating mutation (continued)

olaparib

Cancer type: Ovarian Cancer **Variant class:** BRCA2 mutation

NCCN Recommendation category: 1

Population segment (Line of therapy):
- Epithelial, Fallopian Tube, Primary Peritoneal; Stage II, Stage III, Stage IV; Partial response, Complete response (Maintenance therapy)

Reference: NCCN Guidelines® - NCCN-Ovarian Cancer [Version 3.2021]

olaparib

Cancer type: Castration-Resistant Prostate Cancer **Variant class:** BRCA2 mutation

NCCN Recommendation category: 1

Population segment (Line of therapy):
- Adenocarcinoma; Metastatic (Subsequent therapy); Useful in certain circumstances

Reference: NCCN Guidelines® - NCCN-Prostate Cancer [Version 1.2022]

rucaparib

Cancer type: Ovarian Cancer **Variant class:** BRCA2 mutation

NCCN Recommendation category: 1

Population segment (Line of therapy):
- Epithelial, Fallopian Tube, Primary Peritoneal; Persistent, Recurrent, Partial response, Complete response (Maintenance therapy); Useful in certain circumstances

Reference: NCCN Guidelines® - NCCN-Ovarian Cancer [Version 3.2021]

niraparib

Cancer type: Ovarian Cancer **Variant class:** BRCA2 mutation

NCCN Recommendation category: 2A

Population segment (Line of therapy):
- Epithelial, Fallopian Tube, Primary Peritoneal; Stage II, Stage III, Stage IV; Partial response, Complete response (Maintenance therapy)

Reference: NCCN Guidelines® - NCCN-Ovarian Cancer [Version 3.2021]

Disclaimer: The data presented here is from a curated knowledgebase of publicly available information, but may not be exhaustive. The data version is 2021.11(003).

BRCA2 truncating mutation (continued)

niraparib

Cancer type: Ovarian Cancer **Variant class:** BRCA2 mutation

NCCN Recommendation category: 2A

Population segment (Line of therapy):
- Epithelial, Less Common Ovarian Cancers, Fallopian Tube, Primary Peritoneal; Recurrent (Recurrence therapy); Preferred intervention

Reference: NCCN Guidelines® - NCCN-Ovarian Cancer [Version 3.2021]

olaparib

Cancer type: Ovarian Cancer **Variant class:** BRCA2 mutation

NCCN Recommendation category: 2A

Population segment (Line of therapy):
- Epithelial, Fallopian Tube, Primary Peritoneal; Stage II, Stage III, Stage IV; Partial response, Complete response (Maintenance therapy)

Reference: NCCN Guidelines® - NCCN-Ovarian Cancer [Version 3.2021]

rucaparib

Cancer type: Ovarian Cancer **Variant class:** BRCA2 mutation

NCCN Recommendation category: 2A

Population segment (Line of therapy):
- Epithelial, Less Common Ovarian Cancers, Fallopian Tube, Primary Peritoneal; Recurrent (Recurrence therapy); Preferred intervention

Reference: NCCN Guidelines® - NCCN-Ovarian Cancer [Version 3.2021]

rucaparib

Cancer type: Castration-Resistant Prostate Cancer **Variant class:** BRCA2 mutation

NCCN Recommendation category: 2A

Population segment (Line of therapy):
- Adenocarcinoma; Metastatic (Subsequent therapy); Useful in certain circumstances

Reference: NCCN Guidelines® - NCCN-Prostate Cancer [Version 1.2022]

Disclaimer: The data presented here is from a curated knowledgebase of publicly available information, but may not be exhaustive. The data version is 2021.11(003).

BRCA2 truncating mutation (continued)

olaparib

Cancer type: Castration-Resistant Prostate Cancer **Variant class:** BRCA2 mutation

NCCN Recommendation category: 2B

Population segment (Line of therapy):

- Adenocarcinoma; Visceral Metastases (Subsequent therapy); Useful in certain circumstances

Reference: NCCN Guidelines® - NCCN-Prostate Cancer [Version 1.2022]

---

rucaparib

Cancer type: Castration-Resistant Prostate Cancer **Variant class:** BRCA2 mutation

NCCN Recommendation category: 2B

Population segment (Line of therapy):

- Adenocarcinoma; Visceral Metastases (Subsequent therapy); Useful in certain circumstances

Reference: NCCN Guidelines® - NCCN-Prostate Cancer [Version 1.2022]

Disclaimer: The data presented here is from a curated knowledgebase of publicly available information, but may not be exhaustive. The data version is 2021.11(003).

Current EMA Information

● In this cancer type ○ In other cancer type ◐ In this cancer type and other cancer types

EMA information is current as of 2021-10-13. For the most up-to-date information, search www.ema.europa.eu/ema.

BRCA2 mutation

○ olaparib, bevacizumab + olaparib

Cancer type: Castration-Resistant Prostate Cancer, Ovarian Cancer **Label as of:** 2021-10-12 **Variant class:** BRCA2 mutation

Reference:

https://www.ema.europa.eu/en/documents/product-information/lynparza-epar-product-information_en.pdf

○ rucaparib

Cancer type: Ovarian Cancer **Label as of:** 2021-09-20 **Variant class:** BRCA2 mutation

Reference:

https://www.ema.europa.eu/en/documents/product-information/rubraca-epar-product-information_en.pdf

BRCA2 splice site mutation

○ olaparib, bevacizumab + olaparib

Cancer type: Castration-Resistant Prostate Cancer, Ovarian Cancer **Label as of:** 2021-10-12 **Variant class:** BRCA2 mutation

Reference:

https://www.ema.europa.eu/en/documents/product-information/lynparza-epar-product-information_en.pdf

○ rucaparib

Cancer type: Ovarian Cancer **Label as of:** 2021-09-20 **Variant class:** BRCA2 mutation

Reference:

https://www.ema.europa.eu/en/documents/product-information/rubraca-epar-product-information_en.pdf

BRCA2 truncating mutation

○ olaparib, bevacizumab + olaparib

Cancer type: Castration-Resistant Prostate Cancer, Ovarian Cancer **Label as of:** 2021-10-12 **Variant class:** BRCA2 mutation

Reference:

https://www.ema.europa.eu/en/documents/product-information/lynparza-epar-product-information_en.pdf

BRCA2 truncating mutation (continued)

○ rucaparib

Cancer type: Ovarian Cancer **Label as of:** 2021-09-20 **Variant class:** BRCA2 mutation

Reference:

https://www.ema.europa.eu/en/documents/product-information/rubraca-epar-product-information_en.pdf

Current ESMO Information

● In this cancer type ○ In other cancer type ◐ In this cancer type and other cancer types

ESMO information is current as of 2021-10-01. For the most up-to-date information, search www.esmo.org.

BRCA2 mutation

○ olaparib

Cancer type: Castration-Resistant Prostate Cancer **Variant class:** BRCA2 mutation

ESMO Level of Evidence/Grade of Recommendation: I / B

Population segment (Line of therapy):
- Metastatic, Progression (Line of therapy not specified)

Reference: ESMO Clinical Practice Guidelines - ESMO-Cancer of the Prostate [Ann Oncol (2020)]

○ bevacizumab + olaparib

Cancer type: Ovarian Cancer **Variant class:** BRCA mutation

ESMO Level of Evidence/Grade of Recommendation: I / A

Population segment (Line of therapy):
- Epithelial; Relapsed (Maintenance therapy); ESMO-MCBS v1.1 score: 3

Reference: ESMO Clinical Practice Guidelines - ESMO-Newly Diagnosed and Relapsed Epithelial Ovarian Carcinoma [Ann Oncol 2013; 24 (Suppl 6): vi24-vi32. (eUpdate: 19 July 2021, 01 April 2020, 21 September 2016; Corrigendum: 03 October 2018)]

○ niraparib

Cancer type: Ovarian Cancer **Variant class:** BRCA mutation

ESMO Level of Evidence/Grade of Recommendation: I / A

Population segment (Line of therapy):
- Epithelial; Relapsed (Maintenance therapy); ESMO-MCBS v1.1 score: 3

Reference: ESMO Clinical Practice Guidelines - ESMO-Newly Diagnosed and Relapsed Epithelial Ovarian Carcinoma [Ann Oncol 2013; 24 (Suppl 6): vi24-vi32. (eUpdate: 19 July 2021, 01 April 2020, 21 September 2016; Corrigendum: 03 October 2018)]

○ olaparib

Cancer type: Ovarian Cancer **Variant class:** BRCA mutation

ESMO Level of Evidence/Grade of Recommendation: I / A

Population segment (Line of therapy):
- Epithelial; Relapsed (Maintenance therapy); ESMO-MCBS v1.1 score: 4

Reference: ESMO Clinical Practice Guidelines - ESMO-Newly Diagnosed and Relapsed Epithelial Ovarian Carcinoma [Ann Oncol 2013; 24 (Suppl 6): vi24-vi32. (eUpdate: 19 July 2021, 01 April 2020, 21 September 2016; Corrigendum: 03 October 2018)]

Disclaimer: The data presented here is from a curated knowledgebase of publicly available information, but may not be exhaustive. The data version is 2021.11(003).

BRCA2 mutation (continued)

rucaparib

Cancer type: Ovarian Cancer **Variant class:** BRCA mutation

ESMO Level of Evidence/Grade of Recommendation: III / A

Population segment (Line of therapy):
- Epithelial; Recurrent (Line of therapy not specified)

Reference: ESMO Clinical Practice Guidelines - ESMO-Newly Diagnosed and Relapsed Epithelial Ovarian Carcinoma [Ann Oncol 2013; 24 (Suppl 6): vi24-vi32. (eUpdate: 19 July 2021, 01 April 2020, 21 September 2016; Corrigendum: 03 October 2018)]

BRCA2 splice site mutation

olaparib

Cancer type: Castration-Resistant Prostate Cancer **Variant class:** BRCA2 mutation

ESMO Level of Evidence/Grade of Recommendation: I / B

Population segment (Line of therapy):
- Metastatic, Progression (Line of therapy not specified)

Reference: ESMO Clinical Practice Guidelines - ESMO-Cancer of the Prostate [Ann Oncol (2020)]

bevacizumab + olaparib

Cancer type: Ovarian Cancer **Variant class:** BRCA mutation

ESMO Level of Evidence/Grade of Recommendation: I / A

Population segment (Line of therapy):
- Epithelial; Relapsed (Maintenance therapy); ESMO-MCBS v1.1 score: 3

Reference: ESMO Clinical Practice Guidelines - ESMO-Newly Diagnosed and Relapsed Epithelial Ovarian Carcinoma [Ann Oncol 2013; 24 (Suppl 6): vi24-vi32. (eUpdate: 19 July 2021, 01 April 2020, 21 September 2016; Corrigendum: 03 October 2018)]

niraparib

Cancer type: Ovarian Cancer **Variant class:** BRCA mutation

ESMO Level of Evidence/Grade of Recommendation: I / A

Population segment (Line of therapy):
- Epithelial; Relapsed (Maintenance therapy); ESMO-MCBS v1.1 score: 3

Reference: ESMO Clinical Practice Guidelines - ESMO-Newly Diagnosed and Relapsed Epithelial Ovarian Carcinoma [Ann Oncol 2013; 24 (Suppl 6): vi24-vi32. (eUpdate: 19 July 2021, 01 April 2020, 21 September 2016; Corrigendum: 03 October 2018)]

Disclaimer: The data presented here is from a curated knowledgebase of publicly available information, but may not be exhaustive. The data version is 2021.11(003).

BRCA2 splice site mutation (continued)

○ olaparib

Cancer type: Ovarian Cancer **Variant class:** BRCA mutation

ESMO Level of Evidence/Grade of Recommendation: I / A

Population segment (Line of therapy):
- Epithelial; Relapsed (Maintenance therapy); ESMO-MCBS v1.1 score: 4

Reference: ESMO Clinical Practice Guidelines - ESMO-Newly Diagnosed and Relapsed Epithelial Ovarian Carcinoma [Ann Oncol 2013; 24 (Suppl 6): vi24-vi32. (eUpdate: 19 July 2021, 01 April 2020, 21 September 2016; Corrigendum: 03 October 2018)]

○ rucaparib

Cancer type: Ovarian Cancer **Variant class:** BRCA mutation

ESMO Level of Evidence/Grade of Recommendation: III / A

Population segment (Line of therapy):
- Epithelial; Recurrent (Line of therapy not specified)

Reference: ESMO Clinical Practice Guidelines - ESMO-Newly Diagnosed and Relapsed Epithelial Ovarian Carcinoma [Ann Oncol 2013; 24 (Suppl 6): vi24-vi32. (eUpdate: 19 July 2021, 01 April 2020, 21 September 2016; Corrigendum: 03 October 2018)]

BRCA2 truncating mutation

○ olaparib

Cancer type: Castration-Resistant Prostate Cancer **Variant class:** BRCA2 mutation

ESMO Level of Evidence/Grade of Recommendation: I / B

Population segment (Line of therapy):
- Metastatic, Progression (Line of therapy not specified)

Reference: ESMO Clinical Practice Guidelines - ESMO-Cancer of the Prostate [Ann Oncol (2020)]

○ bevacizumab + olaparib

Cancer type: Ovarian Cancer **Variant class:** BRCA mutation

ESMO Level of Evidence/Grade of Recommendation: I / A

Population segment (Line of therapy):
- Epithelial; Relapsed (Maintenance therapy); ESMO-MCBS v1.1 score: 3

Reference: ESMO Clinical Practice Guidelines - ESMO-Newly Diagnosed and Relapsed Epithelial Ovarian Carcinoma [Ann Oncol 2013; 24 (Suppl 6): vi24-vi32. (eUpdate: 19 July 2021, 01 April 2020, 21 September 2016; Corrigendum: 03 October 2018)]

Disclaimer: The data presented here is from a curated knowledgebase of publicly available information, but may not be exhaustive. The data version is 2021.11(003).

BRCA2 truncating mutation (continued)

niraparib

Cancer type: Ovarian Cancer **Variant class:** BRCA mutation

ESMO Level of Evidence/Grade of Recommendation: I / A

Population segment (Line of therapy):
- Epithelial; Relapsed (Maintenance therapy); ESMO-MCBS v1.1 score: 3

Reference: ESMO Clinical Practice Guidelines - ESMO-Newly Diagnosed and Relapsed Epithelial Ovarian Carcinoma [Ann Oncol 2013; 24 (Suppl 6): vi24-vi32. (eUpdate: 19 July 2021, 01 April 2020, 21 September 2016; Corrigendum: 03 October 2018)]

olaparib

Cancer type: Ovarian Cancer **Variant class:** BRCA mutation

ESMO Level of Evidence/Grade of Recommendation: I / A

Population segment (Line of therapy):
- Epithelial; Relapsed (Maintenance therapy); ESMO-MCBS v1.1 score: 4

Reference: ESMO Clinical Practice Guidelines - ESMO-Newly Diagnosed and Relapsed Epithelial Ovarian Carcinoma [Ann Oncol 2013; 24 (Suppl 6): vi24-vi32. (eUpdate: 19 July 2021, 01 April 2020, 21 September 2016; Corrigendum: 03 October 2018)]

rucaparib

Cancer type: Ovarian Cancer **Variant class:** BRCA mutation

ESMO Level of Evidence/Grade of Recommendation: III / A

Population segment (Line of therapy):
- Epithelial; Recurrent (Line of therapy not specified)

Reference: ESMO Clinical Practice Guidelines - ESMO-Newly Diagnosed and Relapsed Epithelial Ovarian Carcinoma [Ann Oncol 2013; 24 (Suppl 6): vi24-vi32. (eUpdate: 19 July 2021, 01 April 2020, 21 September 2016; Corrigendum: 03 October 2018)]

Disclaimer: The data presented here is from a curated knowledgebase of publicly available information, but may not be exhaustive. The data version is 2021.11(003).

Alerts Informed By Public Data Sources

Current NCCN Information

Contraindicated Not recommended Resistance Breakthrough Fast Track

NCCN information is current as of 2021-10-01. For the most up-to-date information, search www.nccn.org.
For NCCN International Adaptations & Translations, search www.nccn.org/global/international_adaptations.aspx.

BRCA2 mutation

bevacizumab

Cancer type: Ovarian Cancer **Variant class:** BRCA2 mutation

Summary:

NCCN Guidelines® include the following supporting statement(s):

- "bevacizumab monotherapy is no longer recommended for patients with BRCA1/2 mutations"

Reference: NCCN Guidelines® - NCCN-Ovarian Cancer [Version 3.2021]

BRCA2 splice site mutation

bevacizumab

Cancer type: Ovarian Cancer **Variant class:** BRCA2 mutation

Summary:

NCCN Guidelines® include the following supporting statement(s):

- "bevacizumab monotherapy is no longer recommended for patients with BRCA1/2 mutations"

Reference: NCCN Guidelines® - NCCN-Ovarian Cancer [Version 3.2021]

BRCA2 truncating mutation

bevacizumab

Cancer type: Ovarian Cancer **Variant class:** BRCA2 mutation

Summary:

NCCN Guidelines® include the following supporting statement(s):

- "bevacizumab monotherapy is no longer recommended for patients with BRCA1/2 mutations"

Reference: NCCN Guidelines® - NCCN-Ovarian Cancer [Version 3.2021]

Disclaimer: The data presented here is from a curated knowledgebase of publicly available information, but may not be exhaustive. The data version is 2021.11(003).

Current Clinical Trials Information

Clinical Trials information is current as of 2021-10-01. For the most up-to-date information regarding a particular trial, search www.clinicaltrials.gov by NCT ID or search local clinical trials authority website by local identifier listed in 'Other identifiers'.

BRCA2 mutation + BRCA2 splice site mutation + BRCA2 truncating mutation + BRCA2 deletion

NCT04126070

A Phase II Multicohort Study of Nivolumab in Combination With Docetaxel and Androgen Deprivation Therapy in Metastatic Hormone Sensitive Prostate Cancer Patients With DNA Damage Repair Defects or Inflamed Tumors

Cancer type: Prostate Cancer

Variant classes: DNA repair deletion & DNA repair mutation

Other identifiers: 19-384, NCI-2020-02149

Population segments: First line, Stage IV

Phase: II

Therapies: nivolumab, chemotherapy, hormone therapy

Location: United States

US State: MA

Contact: Dr. Xiao X. Wei [617-632-4524; xiaox_wei@dfci.harvard.edu]

BRCA2 mutation

NCT02029001

A Two-period, Multicenter, Randomized, Open-label, Phase II Study Evaluating the Clinical Benefit of a Maintenance Treatment Targeting Tumor Molecular Alterations in Patients with Progressive Locally-advanced or Metastatic Solid Tumors.

Cancer type: Unspecified Solid Tumor

Variant class: BRCA2 mutation

Other identifiers: ET12-081, EudraCT Number: 2012-004510-34, MOST, ProfiLER

Population segments: Maintenance/Consolidation, Second line, Stage III, Stage IV, Third line

Exclusion criteria variant classes: ATM mutation (Gastric Cancer), BRCA1 germline mutation (Gastric Cancer), BRCA1 mutation (Gastric Cancer), BRCA2 germline mutation (Gastric Cancer), BRCA2 mutation (Gastric Cancer), HRR mutation (Gastric Cancer), PALB2 mutation (Gastric Cancer), RAD51 mutation (Gastric Cancer)

Phase: II

Therapy: olaparib

Location: France

NCT03967938

Efficacy of Olaparib in Advanced Cancers Occurring in Patients With Germline Mutations or Somatic Tumor Mutations in Homologous Recombination Genes

Cancer type: Unspecified Solid Tumor

Variant class: BRCA2 mutation

Other identifiers: 1-2018 BSMO, 1-2018BSMO, EudraCT Number: 2018-002966-37, Precision 2 - Olaparib

Population segments: Line of therapy N/A, Stage III, Stage IV

Exclusion criteria variant classes: BRCA1 germline mutation (Breast Cancer, Pancreatic Cancer, Prostate Cancer), BRCA2 germline mutation (Breast Cancer, Pancreatic Cancer, Prostate Cancer)

Phase: II

Therapy: olaparib

Location: Belgium

BRCA2 mutation (continued)

NCT02693535
Targeted Agent and Profiling Utilization Registry (TAPUR) Study.

Cancer type: Unspecified Solid Tumor

Variant class: BRCA2 mutation

Other identifiers: 20170529, NCI-2017-00510, Pro00014171, TAPUR

Population segments: Aggressive, BRAF, Diffuse large B-cell lymphoma (DLBCL), Extranodal marginal zone B-cell lymphoma (MALT), FGFR, First line, Follicular lymphoma (FL), Fourth line or greater, Indolent, Lymphoblastic lymphoma (LBL), Mantle cell lymphoma (MCL), Other subtype, Second line, Small lymphocytic lymphoma (SLL), Stage III, Stage IV, Third line, Waldenstrom's macroglobulinemia (WM)

Phase: II

Therapies: olaparib, talazoparib, atezolizumab + talazoparib

Location: United States

US States: AL, AZ, CA, FL, GA, HI, IL, IN, ME, MI, NC, ND, NE, NH, OH, OR, PA, SD, TX, UT, VA, WA

Contact: Pam Mangat [pam.mangat@asco.org]

NCT04171700
A Phase II Multicenter, Open-label Study of Rucaparib as Treatment for Solid Tumors Associated With Deleterious Mutations in Homologous Recombination Repair Genes

Cancer type: Unspecified Solid Tumor

Variant class: BRCA2 mutation

Other identifiers: 19-756, 20619, AAAS6974, CO-338-100, LODESTAR, NCI-2019-08226

Population segments: BRCA, Second line, Stage III, Stage IV

Exclusion criteria variant classes: BRCA1 germline mutation (Breast Cancer, Ovarian Cancer, Prostate Cancer), BRCA2 germline mutation (Breast Cancer, Ovarian Cancer, Prostate Cancer)

Phase: II

Therapy: rucaparib

Location: United States

US States: CA, FL, IA, IL, MA, MN, NY, OH, OK, PA, TN, WA

Contact: Clovis Oncology For North America [844-258-7662; medinfo@clovisoncology.com]

NCT02286687
Phase II Study of the PARP Inhibitor Talazoparib in Advanced Cancer Patients With Somatic Alterations in BRCA1/2, Mutations/Deletions in Other BRCA Pathway Genes and Germline Mutation in BRCA1/2 (Not Breast or Ovarian Cancer)

Cancer type: Unspecified Cancer

Variant class: BRCA2 mutation

Other identifiers: 2013-0961, NCI-2014-02494

Population segments: Fourth line or greater, Stage III, Stage IV

Exclusion criteria variant classes: BRCA1 germline mutation (Breast Cancer, Ovarian Cancer), BRCA2 germline mutation (Breast Cancer, Ovarian Cancer)

Phase: II

Therapy: talazoparib

Location: United States

US State: TX

Contact: Dr. Sarina Piha-Paul [713-563-1930; spihapau@mdanderson.org]

BRCA2 mutation (continued)

NCT04550494
A Pharmacodynamics-Driven Trial of Talazoparib, an Oral PARP Inhibitor, in Patients With Advanced Solid Tumors and Aberrations in Genes Involved in DNA Damage Response

Cancer type: Unspecified Solid Tumor

Variant class: BRCA2 mutation

Other identifiers: 000246, 000264-C, 10000264, 10371, NCI-2020-06906

Population segments: BRCA, Fourth line or greater, HER2 positive, Hormone refractory, Second line, Stage II, Stage III, Stage IV, Third line

Phase: II

Therapy: talazoparib

Location: United States

US State: MD

Contact: Multiple contacts: See www.clinicaltrials.gov for complete list of contacts.

NCT03842228
A Phase Ib Biomarker-Driven Combination Trial of Copanlisib, Olaparib, and Durvalumab (MEDI4736) in Patients With Advanced Solid Tumors

Cancer type: Unspecified Solid Tumor

Variant class: BRCA2 mutation

Other identifiers: 10217, 19-628, NCI10217, NCI-2019-00601

Population segments: Line of therapy N/A, Stage III, Stage IV

Phase: I

Therapies: copanlisib, olaparib, durvalumab

Location: United States

US States: MA, TX

Contact: Multiple contacts: See www.clinicaltrials.gov for complete list of contacts.

NCT04586335
Open Label, Phase Ib Study to Evaluate the Safety, Tolerability, Pharmacokinetics and Clinical Activity of CYH33, an Oral a-specific PI3K Inhibitor in Combination With Olaparib, an Oral PARP Inhibitor in Patients With Advanced Solid Tumors.

Cancer type: Breast Cancer, Endometrial Carcinoma, Ovarian Cancer, Prostate Cancer, Unspecified Solid Tumor

Variant class: BRCA2 mutation

Other identifiers: CTR20202339, CYH33-G102

Population segments: BRCA, Second line, Stage III, Stage IV

Phase: I

Therapies: HH-CYH33, olaparib

Locations: Australia, United States

US State: TX

Contact: Dr. Jason Sudia [908-380-1329; jason.sudia@haihepharma.com]

NCT03415659
A Phase I, Open-label, Single-center, Single/Multiple-dose, Dose-escalation/ Dose-expansion Clinical Study on Tolerance and Pharmacokinetics of HWH340 Tablet in Patients With Advanced Solid Tumors

Cancer type: Unspecified Solid Tumor

Variant class: BRCA2 mutation

Other identifiers: CTR20171649, HWH340-RFPA 20170821, RFPA 20170821

Population segments: BRCA, Fourth line or greater, HER2 negative, Second line, Stage III, Stage IV, Third line

Phase: I

Therapy: HWH-340

Location: China

Disclaimer: The data presented here is from a curated knowledgebase of publicly available information, but may not be exhaustive. The data version is 2021.11(003).

BRCA2 mutation (continued)

NCT04197713
Phase I Sequential Trial of Agents Against DNA Repair (STAR)

Cancer type: Unspecified Solid Tumor

Variant class: BRCA2 mutation

Other identifiers: (STAR), 10329, 2020-0347, NCI-2019-08262, STAR Study

Population segments: Second line, Stage III, Stage IV

Phase: I

Therapies: olaparib, adavosertib

Location: United States

US State: TX

Contact: Site Public Contact [877-632-6789; askmdanderson@mdanderson.org]

NCT03297606
Canadian Profiling and Targeted Agent Utilization Trial (CAPTUR): A Phase II Basket Trial

Cancer type: Unspecified Solid Tumor

Variant class: BRCA2 aberration

Other identifiers: CA209-9DL, CAPTUR, ESR-17-12831, ML39800, PM1, WI233446

Population segments: Aggressive, Diffuse large B-cell lymphoma (DLBCL), Extranodal marginal zone B-cell lymphoma (MALT), First line, Follicular lymphoma (FL), Indolent, Lymphoblastic lymphoma (LBL), Mantle cell lymphoma (MCL), Other subtype, Second line, Stage III, Stage IV, Waldenstrom's macroglobulinemia (WM)

Phase: II

Therapy: olaparib

Location: Canada

NCT04497116
Phase I/IIa Study of the Safety, Pharmacokinetics, Pharmacodynamics and Preliminary Clinical Activity of RP-3500 Alone or in Combination With Talazoparib in Advanced Solid Tumors With ATR Inhibitor Sensitizing Mutations (TRESR Study)

Cancer type: Unspecified Solid Tumor

Variant class: BRCA2 aberration

Other identifiers: 20-521, EudraCT Number: 2020-000301-87, IRAS ID: 286609, NCI-2020-06869, RP-3500-01, TRESR, TRESR Study

Population segments: Second line, Stage III, Stage IV

Phase: I/II

Therapies: RP-3500, talazoparib

Locations: Canada, Denmark, United Kingdom, United States

US States: MA, NC, NY, RI, TN, TX

Contact: Dr. Peter Manley [857-322-5553; pmanley@reparerx.com]

Disclaimer: The data presented here is from a curated knowledgebase of publicly available information, but may not be exhaustive. The data version is 2021.11(003).

BRCA2 mutation (continued)

NCT04123366
A Phase II Study of Olaparib in Combination With Pembrolizumab in Participants With Previously Treated, Homologous Recombination Repair Mutation (HRRm) and/or Homologous Recombination Deficiency (HRD)-Positive Advanced Cancer

Cancer type: Unspecified Solid Tumor

Variant class: HRR mutation

Other identifiers: 7339-007, EudraCT Number: 2019-001745-40, jRCT2011200025, KEYLYNK-007, MK-7339-007, NCI-2019-08758, PER-029-19

Population segments: BRCA, Second line, Stage III, Stage IV

Exclusion criteria variant classes: BRCA germline mutation (Breast Cancer, Ovarian Cancer), BRCA mutation (Breast Cancer, Ovarian Cancer)

Phase: II

Therapies: olaparib, pembrolizumab

Locations: Argentina, Australia, Canada, Colombia, France, Germany, Guatemala, Israel, Italy, Japan, Latvia, Mexico, Peru, Poland, Puerto Rico, Republic of Korea, Romania, South Africa, Spain, Sweden, Turkey, Ukraine, United States

US States: AL, AZ, CA, CO, FL, GA, KY, NJ, NY, OH, TX, UT, VA

Contact: Toll Free Number [888-577-8839; Trialsites@merck.com]

NCT03767075
Basket of Baskets: A Modular, Open-label, Phase II, Multicentre Study To Evaluate Targeted Agents in Molecularly Selected Populations With Advanced Solid Tumours

Cancer type: Unspecified Solid Tumor

Variant class: DNA repair mutation

Other identifiers: (Basket of Baskets) (BoB), EudraCT number: 2017-005108-89, MO39164, NL65937.031.18, VHIO17002

Population segments: Second line, Stage III, Stage IV

Phase: II

Therapy: atezolizumab

Locations: France, Germany, Netherlands, Spain, Sweden, United Kingdom

---

NCT04905914
A Phase I/IIa, Open-Label, Safety, Pharmacokinetic, And Preliminary Efficacy Study Of Oral ATRN-119 In Patients With Advanced Solid Tumors

Cancer type: Unspecified Solid Tumor

Variant class: DNA repair mutation

Other identifier: AR-276-01

Population segments: Line of therapy N/A, Stage III, Stage IV

Phase: I/II

Therapy: ATRN-119

Location: United States

US State: MA

Contact: Robert Hasson [619-540-6253; rhasson@pacificlinkconsulting.com]

Disclaimer: The data presented here is from a curated knowledgebase of publicly available information, but may not be exhaustive. The data version is 2021.11(003).

BRCA2 mutation (continued)

NCT04901702
A Randomized Phase I/II Study of Talazoparib or Temozolomide in Combination With Onivyde in Children With Recurrent Solid Malignancies and Ewing Sarcoma

Cancer type: Unspecified Solid Tumor

Variant class: HRR pathway

Other identifiers: EudraCT Number: 2021-003570-31, ONITT

Population segments: Second line, Stage IV

Exclusion criteria variant classes: HRR germline pathway (Central Nervous System Neoplasm, Ewing Sarcoma), HRR pathway (Central Nervous System Neoplasm, Ewing Sarcoma)

Phase: I/II

Therapies: talazoparib, chemotherapy

Locations: Canada, United States

US State: TN

Contact: Dr. Sara Federico [866-278-5833; referralinfo@stjude.org]

NCT03155620
NCI-COG Pediatric MATCH (Molecular Analysis for Therapy Choice) Screening Protocol

Cancer type: Unspecified Solid Tumor

Variant class: DNA repair pathway

Other identifiers: 17-729, 2017-0625, 20170841, APEC1621SC, COGAPEC1621, COGAPEC1621SC, N 55017 SC, NCI-2017-01251, NCI-COG Pediatric MATCH, Pediatric MATCH

Population segments: ALK, Aggressive, BRAF, FGFR, HRAS, Indolent, KRAS, NRAS, Pediatric or Adolescent, Second line, Stage III, Stage IV

Phase: II

Therapy: olaparib

Locations: Puerto Rico, United States

US States: AK, AL, AR, AZ, CA, CO, CT, DC, DE, FL, GA, HI, IA, ID, IL, IN, KY, LA, MA, MD, ME, MI, MN, MO, MS, NC, NE, NH, NJ, NV, NY, OH, OK, OR, PA, RI, SC, SD, TN, TX, UT, VA, VT, WA, WI

Contact: Multiple contacts: See www.clinicaltrials.gov for complete list of contacts.

NCT03233204
NCI-COG Pediatric MATCH (Molecular Analysis for Therapy Choice)- Phase 2 Subprotocol of Olaparib in Patients With Tumors Harboring Defects in DNA Damage Repair Genes

Cancer type: Unspecified Solid Tumor

Variant class: DNA repair pathway

Other identifiers: 20170841, APEC1621H, NCI-2017-00766

Population segments: Aggressive, Indolent, Pediatric or Adolescent, Second line, Stage III, Stage IV

Phase: II

Therapy: olaparib

Locations: Puerto Rico, United States

US States: AK, AL, AR, AZ, CA, CO, DC, DE, FL, GA, IA, ID, IL, IN, KY, LA, MD, ME, MI, MN, MO, MS, NC, ND, NE, NJ, NY, OH, OK, OR, PA, SC, SD, TN, TX, UT, VA, VT, WA, WI

Contact: Multiple contacts: See www.clinicaltrials.gov for complete list of contacts.

Disclaimer: The data presented here is from a curated knowledgebase of publicly available information, but may not be exhaustive. The data version is 2021.11(003).

BRCA2 mutation (continued)

NCT04267939
An Open-label Phase Ib Study to Determine the Maximum Tolerated and/or Recommended Phase II Dose of the ATR Inhibitor BAY 1895344 in Combination With PARP Inhibitor Niraparib, in Patients With Recurrent Advanced Solid Tumors and Ovarian Cancer

Cancer type: Ovarian Cancer, Unspecified Solid Tumor

Variant class: DNA repair pathway

Other identifiers: 18595, 19-810, 2019-0471, 20-222, EudraCT Number: 2018-003930-34, NCI-2020-01405

Population segments: Maintenance/Consolidation, Second line, Stage III, Stage IV

Exclusion criteria variant class: DNA repair pathway (Prostate Cancer)

Phase: I

Therapies: BAY-1895344, niraparib

Location: United States

US States: MA, NY, TX

Contact: Bayer Clinical Trials Contact [888-842-2937; clinical-trials-contact@bayer.com]

NCT04693468
Modular Phase IB Hypothesis-Testing, Biomarker-Driven, Talazoparib Combination Trial (TalaCom)

Cancer type: Unspecified Solid Tumor

Variant class: DNA repair pathway

Other identifiers: 2020-0436, NCI-2020-06041, TalaCom

Population segments: Hormone refractory, Second line, Stage III, Stage IV

Phase: I

Therapies: talazoparib, palbociclib, axitinib, crizotinib

Location: United States

US State: TX

Contact: Timothy A. Yap [713-563-1784; tyap@mdanderson.org]

NCT02975934
TRITON3: A Multicenter, Randomized, Open Label Phase III Study of Rucaparib Versus Physician's Choice of Therapy for Patients With Metastatic Castration Resistant Prostate Cancer Associated With Homologous Recombination Deficiency

Cancer type: Castration-Resistant Prostate Cancer

Variant class: BRCA2 mutation

Other identifiers: 16238, 1723GCCC, 18-146, 18-300, 20171185, 2018IS040, CO-338-063, CSET 2620, EudraCT Number: 2016-003163-20, GU 146, IGUP17020, IRAS ID: 222298, NCI-2017-01110, S16-02260, STAR Trial, TRITON 3_CO-338-063, TRITON3

Population segments: BRCA, First line, Hormone refractory, Stage IV

Phase: III

Therapies: rucaparib, hormone therapy

Locations: Australia, Belgium, Canada, Denmark, France, Germany, Ireland, Israel, Italy, Spain, United Kingdom, United States

US States: AZ, CA, CO, CT, DE, FL, HI, MD, MN, NC, NY, OH, OR, TN, TX, VA, WA

Contact: Clovis Oncology For North America [844-258-7662; medinfo@clovisoncology.com]

Disclaimer: The data presented here is from a curated knowledgebase of publicly available information, but may not be exhaustive. The data version is 2021.11(003).

BRCA2 mutation (continued)

NCT03810105
A Phase II Study of Olaparib and Durvalumab in Men With Castration Sensitive Biochemically Recurrent Non-Metastatic Prostate Cancer Harboring Mutations in DNA Damage Repair

Cancer type: Prostate Cancer

Variant class: BRCA2 mutation

Other identifiers: 18-480, NCI-2019-01627

Population segments: Adjuvant, Stage I, Stage II, Stage III

Phase: II

Therapies: durvalumab, olaparib

Location: United States

US States: CA, IL, MI, NJ, NY

Contact: Dr. Karen Autio [646-422-4632; autiok@mskcc.org]

NCT04336943
Durvalumab (MEDI4736) and Olaparib (AZD2281) for Treatment of Biochemically Recurrent Prostate Cancer in Men Predicted to Have a High Neoantigen Load: A Pilot Study

Cancer type: Prostate Adenocarcinoma

Variant class: BRCA2 mutation

Other identifiers: 10509, NCI-2020-01860, RG1007001

Population segments: Line of therapy N/A, Stage I, Stage II, Stage III

Phase: II

Therapies: durvalumab, olaparib

Location: United States

US State: WA

Contact: Michael Schweizer [206-606-6252; schweize@uw.edu]

NCT04053322
An International Multicenter Phase II Trial of Durvalumab (MEDI4736) Plus OLAparib Plus Fulvestrant in Metastatic or Locally Advanced ER-positive, HER2-negative Breast Cancer Patients Selected Using Criteria That Predict Sensitivity to Olaparib

Cancer type: Breast Cancer

Variant class: BRCA2 mutation

Other inclusion criteria: ERBB2 negative, ER positive

Other identifiers: CADUSEIME08, DOLAF, EudraCT Number: 2018-003832-57, UC-0140/1812

Population segments: BRCA, Estrogen receptor positive, FGFR, HER2 negative, Progesterone receptor positive, Second line, Stage III, Stage IV

Phase: II

Therapies: durvalumab, olaparib, hormone therapy

Location: France

Disclaimer: The data presented here is from a curated knowledgebase of publicly available information, but may not be exhaustive. The data version is 2021.11(003).

BRCA2 mutation (continued)

NCT03012321
BRCAAway: A Randomized Phase II Trial of Abiraterone, Olaparib, or Abiraterone + Olaparib in Patients With Metastatic Castration-Resistant Prostate Cancer With DNA Repair Defects

Cancer type: Castration-Resistant Prostate Cancer

Variant class: BRCA2 mutation

Other identifiers: 19137, BRCAAway, NCI-2016-01834, NU 16U05, NU_16U05, PCCTC #: c16-168, PCCTC c16-168, STU00203960

Population segments: First line, Hormone refractory, Stage IV

Phase: II

Therapies: hormone therapy, olaparib, steroid

Location: United States

US States: CA, FL, IL, IN, MD, MI, MN, MO, NC, NY, UT, VA

Contact: Study Coordinator [312-695-1301; cancertrials@northwestern.edu]

No NCT ID - see other identifier(s)
Phase II Study To Evaluate The Efficacy And Safety Of Niraparib In Recurrent Or Persistent Rare Gynecologic Malignancies With Homologous Recombination Deficiency (JGOG2052)

Cancer type: Cervical Cancer, Endometrial Carcinoma, Ovarian Cancer, Uterine Leiomyosarcoma

Variant class: BRCA2 mutation

Other identifiers: (JGOG2052), jRCT2031210264

Population segments: BRCA, Second line, Stage III, Stage IV

Phase: II

Therapy: niraparib

Location: Japan

NCT03553004
Niraparib in Metastatic Pancreatic Cancer After Previous Chemotherapy (NIRA-PANC): A Phase II Trial

Cancer type: Pancreatic Cancer

Variant class: BRCA2 mutation

Other identifiers: IIT-2017-NIRA-PANC, NIRA-PANC, STUDY00141862

Population segments: BRCA, Second line, Stage IV, Third line

Phase: II

Therapy: niraparib

Location: United States

US States: KS, MO

Contact: Clinical Trials Nurse Navigator [913-588-3671; navigation@kumc.edu]

NCT03601923
Phase II Proof-of-Concept Trial Testing the PARP Inhibitor Niraparib in Patients With Pancreatic Cancer Harboring Deficiencies in Homologous Recombination DNA Repair

Cancer type: Pancreatic Cancer

Variant class: BRCA2 mutation

Other identifiers: 18-207, NCI-2018-02395

Population segments: Second line, Stage IV

Phase: II

Therapy: niraparib

Location: United States

US State: MA

Contact: Dr. James Clreary [617-632-5960; JCLEARY@PARTNERS.ORG]

Disclaimer: The data presented here is from a curated knowledgebase of publicly available information, but may not be exhaustive. The data version is 2021.11(003).

BRCA2 mutation (continued)

NCT03840967
A Phase II Study Evaluating Safety and Efficacy of Niraparib in Patients With Previously Treated Homologous Recombination (HR) Defective or Loss of Heterozygosity (LOH) High Metastatic Esophageal/Gastroesophageal Junction/ Proximal Gastric Adenocarcinoma

Cancer type: Esophageal Cancer, Gastric Cancer, Gastroesophageal Junction Adenocarcinoma

Variant class: BRCA2 mutation

Other identifiers: 20166, 71618, HCRN ESO17-325, NCI-2019-07395

Population segments: Second line, Stage III, Stage IV

Phase: II

Therapy: niraparib

Location: United States

US States: FL, IN, MI, NY

Contact: Dr. Shadia Jalal [317-274-3658; sjalal@iu.edu]

NCT03925350
A Phase II Study of Niraparib in Patients With Advanced Melanoma With Genetic Homologous Recombination (HR) Mutation / Alteration

Cancer type: Melanoma

Variant class: BRCA2 mutation

Other identifier: CPMC17-MEL01

Population segments: Second line, Stage III, Stage IV

Phase: II

Therapy: niraparib

Location: United States

US State: CA

Contact: Laurel Brechtel [415-600-1654; BrechtLA@sutterhealth.org]

NCT04493060
Phase II Study of Niraparib and TSR-042 in Patients With Germline or Somatic BRCA1/2 and PALB2-Related Pancreatic Cancer

Cancer type: Pancreatic Cancer

Variant class: BRCA2 mutation

Other identifiers: MC1841, NCI-2020-05352

Population segments: BRCA, Second line, Stage IV, Third line

Phase: II

Therapies: niraparib, dostarlimab

Location: United States

US States: AZ, FL, MN

Contact: Multiple contacts: See www.clinicaltrials.gov for complete list of contacts.

NCT04717154
Phase II INSPIRE Trial: Ipilimumab With Nivolumab In Molecular-Selected Patients With Castration-Resistant Prostate Cancer.

Cancer type: Castration-Resistant Prostate Cancer

Variant class: BRCA2 mutation

Other identifiers: CA184-585, EudraCT Number: 2020-001240-25, INSPIRE, MOURO048 - INSPIRE, MOURO48

Population segments: BRCA, First line, Hormone refractory, Second line, Stage IV

Phase: II

Therapies: nivolumab, ipilimumab

Location: Netherlands

Disclaimer: The data presented here is from a curated knowledgebase of publicly available information, but may not be exhaustive. The data version is 2021.11(003).

BRCA2 mutation (continued)

NCT04187833
Phase II Trial of Nivolumab in Combination with Talazoparib in Patients with Unresectable or Metastatic Melanoma and Mutations in BRCA or BRCA-ness Genes.

Cancer type: Melanoma

Variant class: BRCA2 mutation

Other identifiers: CASE2619, NCI-2020-00416

Population segments: Second line, Stage III, Stage IV

Phase: II

Therapies: nivolumab, talazoparib

Location: United States

US State: OH

Contact: Dr. Pauline Funchain [866-223-8100; TaussigResearch@ccf.org]

NCT03344965
A Phase II Study of Olaparib Monotherapy in Metastatic Breast Cancer Patients With Germline or Somatic Mutations in DNA Repair Genes (Olaparib Expanded)

Cancer type: Breast Cancer

Variant class: BRCA2 mutation

Other identifiers: 17-428, 18-414, NCI-2018-00778, TBCRC 048, TBCRC048

Population segments: BRCA, Estrogen receptor positive, HER2 negative, HER2 positive, Progesterone receptor positive, Second line, Stage IV

Exclusion criteria variant classes: BRCA1 germline mutation (Breast Cancer), BRCA2 germline mutation (Breast Cancer)

Phase: II

Therapy: olaparib

Location: United States

US States: AL, CA, IL, MA, MD, NC, NJ, NY, PA, WA

Contact: Dr. Nadine Tung [617-667-1962; ntung@bidmc.harvard.edu]

NCT03375307
A Phase II Study of Olaparib (AZD2281) in Patients With Metastatic/Advanced Urothelial Carcinoma With DNA-Repair Defects

Cancer type: Bladder Urothelial Carcinoma

Variant class: BRCA2 mutation

Other identifiers: 10144, 19C0023, NCI-2017-02296, P162941

Population segments: BRCA, STK11, Second line, Stage III, Stage IV

Phase: II

Therapy: olaparib

Location: United States

US States: CA, CO, KY, MD, NY, OK

Contact: Multiple contacts: See www.clinicaltrials.gov for complete list of contacts.

Disclaimer: The data presented here is from a curated knowledgebase of publicly available information, but may not be exhaustive. The data version is 2021.11(003).

BRCA2 mutation (continued)

NCT03742895

A Phase II Study of Olaparib Monotherapy in Participants With Previously Treated, Homologous Recombination Repair Mutation (HRRm) or Homologous Recombination Deficiency (HRD) Positive Advanced Cancer

Cancer type: Breast Cancer

Variant class: BRCA2 mutation

Other inclusion criteria: BRCA1 germline mutation negative, BRCA2 germline mutation negative

Other identifiers: (MK-7339-002 / LYNK-002), 19-139, 194694, 2018-01891, 7339-002, 7339-002-00, EudraCT Number: 2018-003007-19, IRAS ID: 255158, JapicCTI-194694, LYNK002, LYNK-002, MK7339-002, MK-7339-002, MK7339-002-00, NCI-2018-03519, PER-046-18, SNCTP000003157

Population segments: BRCA, Fourth line or greater, Second line, Stage III, Stage IV, Third line

Exclusion criteria variant classes: BRCA germline mutation (Breast Cancer, Ovarian Cancer), BRCA mutation (Ovarian Cancer)

Phase: II

Therapy: olaparib

Locations: Argentina, Australia, Canada, Colombia, Denmark, France, Guatemala, Ireland, Israel, Italy, Mexico, Peru, Republic of Korea, Romania, Russian Federation, Spain, Switzerland, Turkey, United Kingdom, United States

US States: AZ, CA, CO, GA, KY, MA, MD, MI, NE, NJ, NY, SD, UT, WA

Contact: Toll Free Number [888-577-8839; Trialsites@merck.com]

NCT03786796

Phase II Study of Olaparib in Metastatic Renal Cell Carcinoma Patients Harboring a BAP-1 or Other DNA Repair Gene Mutations (ORCHID)

Cancer type: Kidney Cancer

Variant class: BRCA2 mutation

Other identifiers: CRMS-70750, J18166, NCI-2019-06309, ORCHID

Population segments: Fourth line or greater, Second line, Stage IV, Third line

Phase: II

Therapy: olaparib

Location: United States

US State: MD

Contact: Irina Rifkind [410-502-2043; irifkin1@jhmi.edu]

NCT04042831

A Phase II Study of Olaparib in Patients With Advanced Biliary Tract Cancer With Aberrant DNA Repair Gene Mutations

Cancer type: Biliary Tract Carcinoma

Variant class: BRCA2 mutation

Other identifiers: ACCRU-ICRN-1702, NCI-2019-04434

Population segments: Second line, Stage IV

Phase: II

Therapy: olaparib

Location: United States

US States: AZ, MN, NY, TX

Contact: Multiple contacts: See www.clinicaltrials.gov for complete list of contacts.

BRCA2 mutation (continued)

NCT04515836
Phase II Trial of Olaparib in Homologous Recombination Deficient (HRD) Malignant Mesothelioma

Cancer type: Mesothelioma

Variant class: BRCA2 mutation

Population segments: (N/A), Second line

Phase: II

Therapy: olaparib

Location: United States

US State: IL

Contact: Dr. Hedy L. Kindler [773-702-0360; hkindler@medicine.bsd.uchicago.edu]

NCT04858334
APOLLO: A Randomized Phase II Double-Blind Study of Olaparib Versus Placebo Following Adjuvant Chemotherapy in Patients With Resected Pancreatic Cancer and a Pathogenic BRCA1, BRCA2 or PALB2 Mutation

Cancer type: Pancreatic Cancer

Variant class: BRCA2 mutation

Other identifiers: APOLLO, CTSU/EA2192, EA2192, NCI-2020-05659

Population segments: Adjuvant, BRCA, Maintenance/Consolidation, Stage 0, Stage I, Stage II

Phase: II

Therapy: olaparib

Location: United States

US States: AZ, FL, IA, IL, IN, KS, MI, MO, NC, NJ, NM, NV, NY, OH, OR, PA, WA, WI

Contact: Multiple contacts: See www.clinicaltrials.gov for complete list of contacts.

NCT02849496
A Phase II Open-Label, Randomized Study of PARP Inhibition (Olaparib) Either Alone or in Combination With Anti-PD-L1 Therapy (Atezolizumab; MPDL3280A) in Homologous DNA Repair (HDR) Deficient, Locally Advanced or Metastatic Non-HER2-Positive Breast Cancer.

Cancer type: Breast Cancer

Variant class: BRCA2 mutation

Other inclusion criteria: ERBB2 negative

Other identifiers: 10020, 109587 (10020), 1608018258, 17-709, 18979, 5UM1CA186709-04, Clinical Trial 18979, NCI-2016-01130, OSU-17011, PHII-146

Population segments: BRCA, HER2 negative, Second line, Stage III, Stage IV

Phase: II

Therapies: olaparib, atezolizumab

Location: United States

US States: CA, CO, CT, FL, IL, MA, MD, MI, MO, NC, NY, OH, PA, TN, UT

Contact: Multiple contacts: See www.clinicaltrials.gov for complete list of contacts.

NCT04939662
Phase II, Single-arm Study of Olaparib and Bevacizumab Combination Therapy in Relapsed Small Cell Lung Cancer Subjects With DNA Damage Response and the Repair Pathway Alteration, ATM Deficiency, SLFN11 Positive, or POU2F3 Positive

Cancer type: Small Cell Lung Cancer

Variant class: BRCA2 mutation

Other identifier: SUKSES-B2

Population segments: (N/A), BRCA, Pulmonary, Second line, Third line

Phase: II

Therapies: olaparib, bevacizumab

Location: Republic of Korea

Disclaimer: The data presented here is from a curated knowledgebase of publicly available information, but may not be exhaustive. The data version is 2021.11(003).

BRCA2 mutation (continued)

NCT04261465
The NUVOLA TRIAL: Neoadjuvant Chemotherapy in Unresectable Ovarian Cancer With OLAparib and Weekly Carboplatin Plus Paclitaxel: A Phase II Open-label Multicentre Study

Cancer type: Ovarian Cancer

Variant class: BRCA2 mutation

Other identifiers: 2317, 2345, EudraCT Number: 2018-002687-65, NUVOLA, NUVOLA trial

Population segments: BRCA, Maintenance/Consolidation, Neoadjuvant, Stage III, Stage IV

Phase: II

Therapies: olaparib, chemotherapy

Location: Italy

NCT04633902
Phase II Study of Olaparib in Combination With Pembrolizumab in Patients With Advanced Melanoma With Homologous Recombination (HR) Pathway Gene Mutation

Cancer type: Melanoma

Variant class: BRCA2 mutation

Other identifier: CPMC19-MEL01

Population segments: BRAF, Second line, Stage III, Stage IV

Phase: II

Therapies: olaparib, pembrolizumab

Location: United States

US State: CA

Contact: Dr. Kevin B. Kim [415-885-8600; ClinicalResearch@sutterhealth.org]

NCT03248570
Phase II Open Label Study of Pembrolizumab in Patients With Metastatic Castration Resistant Prostate Cancer (mCRPC) With or Without DNA Damage Repair Defects

Cancer type: Castration-Resistant Prostate Cancer

Variant class: BRCA2 mutation

Other identifiers: 16557, 17-22165, CC 16557, NCI-2017-02408

Population segments: BRCA, Hormone refractory, Line of therapy N/A, Stage IV

Phase: II

Therapy: pembrolizumab

Location: United States

US State: CA

Contact: Patricia Li [415-476-5975; GUTrials@ucsf.edu]

NCT03506997
PERSEUS1: Phase II Trial of the Immune Checkpoint Inhibitor Pembrolizumab for Patients Suffering From Metastatic Prostate Cancer

Cancer type: Castration-Resistant Prostate Cancer

Variant class: BRCA2 mutation

Other identifiers: CCR4559, EudraCT Number: 2017-000931-15, ICR-CTSU/2016/10060, IRAS ID 208952, PERSEUS1

Population segments: Hormone refractory, Second line, Stage IV

Phase: II

Therapy: pembrolizumab

Location: United Kingdom

Disclaimer: The data presented here is from a curated knowledgebase of publicly available information, but may not be exhaustive. The data version is 2021.11(003).

BRCA2 mutation (continued)

NCT04089189
A Phase II Study to Evaluate the Efficacy, Safety, and Tolerability of IMP4297 Capsules in Subjects With Germline and/or Systemic BRCA1/2 Mutation Positive Advanced Ovarian Cancer With at Least 2 Prior Lines of Standard Systemic Therapy

Cancer type: Ovarian Cancer

Variant class: BRCA2 mutation

Other identifiers: CTR20190652, IMP4297-2018-CN01

Population segments: Stage III, Stage IV, Third line

Phase: II

Therapy: senaparib

Location: China

NCT03990896
Evaluation of Talazoparib, a PARP Inhibitor, in Patients With Somatic BRCA Mutant Metastatic Breast Cancer: Genotyping Based Clinical Trial

Cancer type: Breast Cancer, Triple Negative Breast Cancer

Variant class: BRCA2 mutation

Other inclusion criteria: ERBB2 negative, Hormone receptor positive

Other identifiers: 19-188, NCI-2019-07732

Population segments: BRCA, Estrogen receptor positive, Fourth line or greater, HER2 negative, Progesterone receptor positive, Second line, Stage IV, Third line, Triple receptor negative

Exclusion criteria variant classes: BRCA1 germline mutation (Breast Cancer, Triple Negative Breast Cancer), BRCA2 germline mutation (Breast Cancer, Triple Negative Breast Cancer)

Phase: II

Therapy: talazoparib

Location: United States

US States: MA, TX

Contact: Dr. Neelima Vidula [617-724-4000; nvidula@mgh.harvard.edu]

NCT04892693
Talazoparib in Advanced Breast Cancer Patients With Homologous Recombinant Deficiency: A Phase II Clinical and Exploratory Biomarker Study of Talazoparib

Cancer type: Breast Cancer

Variant class: BRCA2 mutation

Other identifier: H-2009-079-1157

Population segments: BRCA, Line of therapy N/A, Stage III, Stage IV

Phase: II

Therapy: talazoparib

Location: Republic of Korea

NCT04740190
Combination Talazoparib - Carboplatin for Recurrent High-grade Glioma With DNA Damage Repair Deficiency (DDRd)

Cancer type: Anaplastic Astrocytoma, Anaplastic Oligoastrocytoma, Anaplastic Oligodendroglioma, Anaplastic Pleomorphic Xanthoastrocytoma, Diffuse Midline Glioma, Glioblastoma

Variant class: BRCA2 mutation

Other identifiers: TAC-GReD, UW-20396

Population segments: (N/A), BRCA, Second line

Phase: II

Therapies: talazoparib, chemotherapy

Location: Hong Kong

Disclaimer: The data presented here is from a curated knowledgebase of publicly available information, but may not be exhaustive. The data version is 2021.11(003).

BRCA2 mutation (continued)

NCT04253262
A Phase Ib/II Study of Copanlisib Combined With Rucaparib in Patients With Metastatic Castration-resistant Prostate Cancer

Cancer type: Castration-Resistant Prostate Cancer

Variant class: BRCA2 mutation

Other identifiers: BrUOG 360, BrUOG360

Population segments: Hormone refractory, Second line, Stage IV

Phase: I/II

Therapies: copanlisib, rucaparib

Location: United States

US State: RI

Contact: BrUOG [401-863-3000; BrUOG@brown.edu]

NCT03685331
Harnessing Olaparib, Palbociclib and Endocrine Therapy: A Phase I/II Trial of Olaparib, Palbociclib and Fulvestrant in Patients With BRCA Mutation-associated, Hormone Receptor-positive, Human Epidermal Growth Factor Receptor 2 (HER2)-Negative Metastatic Breast Cancer (HOPE)

Cancer type: Breast Cancer

Variant class: BRCA2 mutation

Other inclusion criteria: ERBB2 negative, Hormone receptor positive

Other identifiers: (HOPE), NCI-2019-04210, UPCC 21118

Population segments: BRCA, Estrogen receptor positive, First line, HER2 negative, Progesterone receptor positive, Second line, Stage III, Stage IV, Third line

Phase: I/II

Therapies: olaparib, palbociclib, hormone therapy

Location: United States

US State: PA

Contact: Alexandra Torres [855-216-0098; PennCancerTrials@emergingmed.com]

No NCT ID - see other identifier(s)
An Open-Label, Randomised, Phase I/II Trial Of Rucaparib Combined With Nivolumab + or - Ipilimumab To Augment Response In Homologous Repair Deficient Patients With Relapsed Ovarian, Primary Peritoneal And Fallopian Tube Cancer

Cancer type: Ovarian Cancer

Variant class: BRCA2 mutation

Other identifiers: CeNturIOn, CeNturIOn-2016, EudraCT Number: 2017-004780-13, ISRCTN10490346

Population segments: (N/A), BRCA, Fourth line or greater, Second line, Third line

Phase: I/II

Therapies: rucaparib, nivolumab, ipilimumab

Location: United Kingdom

Disclaimer: The data presented here is from a curated knowledgebase of publicly available information, but may not be exhaustive. The data version is 2021.11(003).

BRCA2 mutation (continued)

NCT03964532
TALAVE: A Pilot Trial of Induction Talazoparib Followed by Combination of Talazoparib and Avelumab in Advanced Breast Cancer

Cancer type: Breast Cancer

Variant class: BRCA2 mutation

Other inclusion criteria: ERBB2 negative

Other identifiers: NCI-2019-06240, STUDY00000023, TALAVE

Population segments: Line of therapy N/A, Stage III, Stage IV

Phase: I/II

Therapies: talazoparib, avelumab

Location: United States

US States: DC, NC, UT

Contact: Dr. Julie Collins [202-444-2223; Julie.Collins@gunet.georgetown.edu]

NCT04673448
Phase IB Trial of Niraparib and TSR-042 in Patients With BRCA-Mutated Breast, Pancreas or Ovary Cancer

Cancer type: Breast Cancer, Ovarian Cancer, Pancreatic Cancer

Variant class: BRCA2 mutation

Other identifiers: 10020, NCI-2020-11636, RG1005140

Population segments: BRCA, Estrogen receptor positive, HER2 positive, Progesterone receptor positive, Second line, Stage III, Stage IV

Phase: I

Therapies: niraparib, dostarlimab

Location: United States

US State: WA

Contact: Elizabeth M. Swisher [206-543-3669; swishere@uw.edu]

NCT04598321
BrUOG 390: Neoadjuvant Treatment With Talazoparib for Women With Newly Diagnosed, Advanced Ovarian Cancer Associated With a Mutation in BRCA1 or BRCA2 (mBRCA)

Cancer type: Ovarian Cancer

Variant class: BRCA2 mutation

Other identifier: BrUOG 390

Population segments: BRCA, Maintenance/Consolidation, Neoadjuvant, Stage II, Stage III, Stage IV

Phase: I

Therapy: talazoparib

Location: United States

US State: RI

Contact: BrUOG [401-863-3000; BrUOG@Brown.edu]

Disclaimer: The data presented here is from a curated knowledgebase of publicly available information, but may not be exhaustive. The data version is 2021.11(003).

BRCA2 mutation (continued)

NCT03598270

A Phase III Randomized, Double-blinded Trial of Platinum-based Chemotherapy With or Without Atezolizumab Followed by Niraparib Maintenance With or Without Atezolizumab in Patients With Recurrent Ovarian, Tubal or Peritoneal Cancer and Platinum Treatment-free Interval (TFIp) >6 Months

Cancer type: Ovarian Cancer

Variant class: BRCA mutation

Other identifiers: AGO ovary 2.33, ANITA, BGOG-ov-41, ENGOT-ov41, ENGOT-OV41/GEICO 69-O/ANITA, ENGOT-ov41/GEICO/ANITA, ENGOT-Ov41/GEICO69-O/ANITA, EudraCT Number: 2018-000366-11, GEICO 69-O, GEICO-69-O

Population segments: (N/A), BRCA, Maintenance/Consolidation, Second line, Third line

Phase: III

Therapies: atezolizumab, chemotherapy, niraparib

Locations: Belgium, France, Germany, Italy, Spain

NCT03278717

International Phase III Randomised Study to Evaluate the Efficacy of Maintenance Therapy With Olaparib and Cediranib or Olaparib Alone in Patients With Relapsed Ovarian Cancer Following a Response to Platinum-based Chemotherapy

Cancer type: Ovarian Cancer

Variant class: BRCA mutation

Other identifiers: 36280, ACTRN12618000498291, ANZGOG 1415/2018, CRUK/15/074, CTC 0133, ENGOT-ov35, ENGOT-ov35/NCRI/ICON 9, EudraCT Number: 2017-000161-75, ICON9, NC174, OV26 (CRUK/UCL ICON9), UCL/14/0795

Population segments: BRCA, Maintenance/Consolidation, Second line, Stage III, Stage IV

Phase: III

Therapies: cediranib, olaparib

Locations: Australia, Canada, New Zealand, United Kingdom

NCT04915755

A Randomized Phase III Double-Blinded Study Comparing the Efficacy and Safety of Niraparib to Placebo in Participants With Either HER2-Negative BRCA-Mutated or Triple-Negative Breast Cancer With Molecular Disease Based on Presence of Circulating Tumor DNA After Definitive Therapy (ZEST)

Cancer type: Breast Cancer

Variant class: BRCA mutation

Other inclusion criteria: ERBB2 negative

Other identifiers: 213831, EudraCT Number: 2020-003973-23, GSK 213831 ZEST, SNCTP000004543, ZEST

Population segments: BRCA, Estrogen receptor positive, HER2 negative, Progesterone receptor positive, Second line, Stage I, Stage II, Stage III, Triple receptor negative

Phase: III

Therapy: niraparib

Locations: Canada, France, Ireland, Italy, Netherlands, Norway, Poland, Russian Federation, Spain, United Kingdom, United States

US State: IL

Contact: US GSK Clinical Trials Call Center [877-379-3718; GSKClinicalSupportHD@gsk.com]

Disclaimer: The data presented here is from a curated knowledgebase of publicly available information, but may not be exhaustive. The data version is 2021.11(003).

BRCA2 mutation (continued)

NCT03462342
Combination ATR and PARP Inhibitor (CAPRI) Trial With AZD 6738 and Olaparib in Recurrent Ovarian Cancer

Cancer type: Ovarian Cancer

Variant class: BRCA mutation

Other identifiers: 20-320, CAPRI, ESR-16-12311, NCI-2018-02375, UPCC 15817

Population segments: BRCA, Fourth line or greater, Second line, Stage III, Stage IV, Third line

Phase: II

Therapies: ceralasertib, olaparib

Location: United States

US States: MA, MD, PA

Contact: Diego Rodriguez [215-614-0234; diegorod@pennmedicine.upenn.edu]

No NCT ID - see other identifier(s)
An Investigator-Initiated Phase II Trial Of Niraparib For Patients With BRCA-Mutated Unresectable / Recurrent Biliary Tract, Pancreatic And Other Gastrointestinal Cancer (NIR-B)

Cancer type: Cholangiocarcinoma, Colon Cancer, Gastrointestinal Stromal Tumor, Pancreatic Cancer, Rectal Cancer

Variant class: BRCA mutation

Other identifiers: jRCT2011200023, NIR-B

Population segments: BRCA, Second line, Stage III, Stage IV, Third line

Phase: II

Therapy: niraparib

Location: Japan

NCT04716686
Niraparib Monotherapy as Maintain and Recurrent Treatment of Endometrial Serous Carcinoma: A Multi-center, Open-label, Prospective Clinical Study

Cancer type: Endometrial Serous Adenocarcinoma

Variant class: BRCA mutation

Other identifier: ZL-2306-914

Population segments: BRCA, Fourth line or greater, Maintenance/Consolidation, Second line, Stage III, Stage IV, Third line

Phase: II

Therapy: niraparib

Location: China

NCT04348045
MAZEPPA: Phase II PRODIGE-GERCOR Study to Evaluate MAintenance Therapy With Olaparib or Selumetinib Plus Durvalumab According to BRCAness and KRAS Somatic Status Personalized in Metastatic Pancreatic Adenocarcinoma Patients

Cancer type: Pancreatic Cancer

Variant class: BRCA mutation

Other inclusion criteria: DPYD wild type

Other identifiers: D19-02, EudraCT Number: 2019-004366-18, MAZEPPA D19-02, MAZEPPA D19-02 PRODIGE 72, MAZEPPA_D19-02, PRODIGY 72 - MAZEPPA

Population segments: BRCA, KRAS, Maintenance/Consolidation, Stage IV

Phase: II

Therapy: olaparib

Location: France

Disclaimer: The data presented here is from a curated knowledgebase of publicly available information, but may not be exhaustive. The data version is 2021.11(003).

BRCA2 mutation (continued)

NCT03025035

Open Label, Phase II Pilot Study of Immune Checkpoint Inhibition With Pembrolizumab in Combination With PARP Inhibition With Olaparib in Advanced BRCA-mutated or HDR-defect Breast Cancers

Cancer type: Breast Cancer

Variant class: BRCA mutation

Other identifier: IIT2015-18-Mita-MK3475

Population segments: BRCA, Estrogen receptor positive, HER2 negative, HER2 positive, Progesterone receptor positive, Second line, Stage III, Stage IV, Triple receptor negative

Phase: II

Therapies: pembrolizumab, olaparib

Location: United States

US State: CA

Contact: Parisa Mirzadehgan [310-967-4387; Parisa.Mirzadehgan@cshs.org]

NCT04666740

A Phase II Trial to Evaluate the Safety and Antitumor Activity of Pembrolizumab and OLApaRib (POLAR) Maintenance for Patients With Metastatic Pancreatic Ductal Adenocarcinoma and Homologous Recombination Deficiency and/or Exceptional Treatment Response to Platinum-Based Therapy

Cancer type: Pancreatic Cancer

Variant class: BRCA2 aberration

Other identifier: 20-481

Population segments: BRCA, Maintenance/Consolidation, Stage IV

Phase: II

Therapies: pembrolizumab, olaparib

Location: United States

US States: NJ, NY

Contact: Dr. Wungki Park [646-888-4543; yaqubiea@mskcc.org]

NCT02264678

A Modular Phase I, Open-Label, Multicentre Study to Assess the Safety, Tolerability, Pharmacokinetics and Preliminary Anti-tumour Activity of Ceralasertib in Combination With Cytotoxic Chemotherapy and/or DNA Damage Repair/Novel Anti-cancer Agents in Patients With Advanced Solid Malignancies

Cancer type: Breast Cancer, Ovarian Cancer

Variant class: BRCA mutation

Other inclusion criteria: ERBB2 negative

Other identifiers: 0C-14-9, 16-031, 18-342, D5330C00004, EudraCT Number: 2014-002233-66, IRAS ID 160200, NCI-2015-00687, Study 4

Population segments: Adenocarcinoma, BRCA, HER2 negative, Pulmonary, Second line, Stage III, Stage IV, Third line, Triple receptor negative

Exclusion criteria variant class: ERBB2 positive (Breast Cancer)

Phase: I

Therapies: ceralasertib, olaparib

Locations: France, Republic of Korea, United Kingdom, United States

US States: CA, MA, NY

Contact: AstraZeneca Clinical Study Information Center [877-240-9479; information.center@astrazeneca.com]

Disclaimer: The data presented here is from a curated knowledgebase of publicly available information, but may not be exhaustive. The data version is 2021.11(003).

BRCA2 mutation (continued)

NCT04169841

Precision Medicine Phase II Study Evaluating the Efficacy of a Double Immunotherapy by Durvalumab and Tremelimumab Combined With Olaparib in Patients With Solid Cancers and Carriers of Homologous Recombination Repair Genes Mutation in Response or Stable After Olaparib Treatment

Cancer type: Breast Cancer, Endometrial Carcinoma, Head and Neck Cancer, Kidney Cancer, Non-Small Cell Lung Cancer, Ovarian Cancer, Pancreatic Cancer, Prostate Cancer

Variant class: HRR mutation

Other identifier: GUIDE2REPAIR

Population segments: Fourth line or greater, Hormone refractory, Maintenance/ Consolidation, Second line, Stage III, Stage IV, Third line

Exclusion criteria variant classes: ALK fusion (Lung Cancer), BRAF mutation (Lung Cancer), EGFR mutation (Lung Cancer), ROS1 mutation (Lung Cancer)

Phase: II

Therapies: durvalumab, tremelimumab, olaparib

Location: France

NCT04030559

Phase II Trial of PARP Inhibitor Niraparib for Men With High Risk Prostate Cancer and DNA Damage Response Defects

Cancer type: Prostate Cancer

Variant class: HRR mutation

Other identifiers: 1412676, NCI-2019-04365, UCDCC#279

Population segments: First line, Neoadjuvant, Stage I, Stage II, Stage IV

Phase: II

Therapy: niraparib

Location: United States

US State: CA

Contact: Marc Dall'Era [916-734-3771; mdallera@ucdavis.edu]

NCT04940637

A Phase II, Open-Label, Single Arm, Prospective, Multicenter Study of Niraparib Plus Dostarlimab in Patients With Advanced NSCLC and/or MPM, and Positive for PD-L1 Expression and Germline or Somatic Mutations in the HRR Genes

Cancer type: Mesothelioma, Non-Small Cell Lung Cancer

Variant class: HRR mutation

Other inclusion criteria: ALK fusion negative, CD274 expression, EGFRi sensitizing mutation negative, ROS1 fusion negative or CD274 expression

Other identifiers: EudraCT Number: 2020-000109-10, UNITO-001, UNITO-001/2020

Population segments: Second line, Stage III, Stage IV

Exclusion criteria variant classes: ALK fusion (Non-Small Cell Lung Cancer), EGFRi sensitizing mutation (Non-Small Cell Lung Cancer), ROS1 fusion (Non-Small Cell Lung Cancer)

Phase: II

Therapies: niraparib, dostarlimab

Location: Italy

Disclaimer: The data presented here is from a curated knowledgebase of publicly available information, but may not be exhaustive. The data version is 2021.11(003).

BRCA2 mutation (continued)

NCT04890613
Phase Ib Expansion Study of CX-5461 in Patients With Solid Tumours and BRCA2 and/or PALB2 Mutation

Cancer type: Ovarian Cancer

Variant class: HRR mutation

Other identifier: CX-5461-04

Population segments: BRCA, Second line, Stage II, Stage III, Stage IV

Phase: I

Therapy: pidnarulex

Location: Canada

NCT05002868
A Multi-center, Open-label, Phase I/Ib Study to Assess the Safety, Pharmacokinetics and Anti-tumor Activity of RP12146, a Poly (ADP-ribose) Polymerase (PARP) Inhibitor, in Patients With Locally Advanced or Metastatic Solid Tumors

Cancer type: Biliary Tract Carcinoma, Breast Cancer, Colorectal Cancer, Endometrial Carcinoma, Gastric Cancer, Ovarian Cancer, Pancreatic Cancer, Prostate Cancer, Small Cell Lung Cancer

Variant class: HRR mutation

Other identifier: RP12146-2101

Population segments: Extensive, Pulmonary, Second line, Stage III, Stage IV

Exclusion criteria variant class: ERBB2 positive (Breast Cancer)

Phase: I

Therapy: RP12146

Location: Poland

NCT03395197
A Phase III, Randomized, Double-blind, Placebo-controlled Study Of Talazoparib With Enzalutamide In Metastatic Castration-resistant Prostate Cancer

Cancer type: Castration-Resistant Prostate Cancer

Variant class: DNA repair mutation

Other identifiers: BASIC-ROYAL / HIGH-ROYAL, C3441021, CSET 2938, CTR20191443, CTR20191444, EudraCT Number: 2017-003295-31, GU 170 - C3441021, HCC 19-054, JapicCTI-194665, LH19.022, MOH_2019-05-16_007127, NCI-2018-01651, Pfizer C3441021, R19062M, RECF3951, RWF_C3441021 TALAPRO-2, TALAPRO-2, TX228665, UCI-18-47, VICCURO17115

Population segments: First line, Hormone refractory, Second line, Stage III, Stage IV

Phase: III

Therapies: talazoparib, hormone therapy

Locations: Argentina, Australia, Belgium, Brazil, Canada, Chile, China, Czech Republic, Finland, France, Germany, Hungary, Israel, Italy, Japan, New Zealand, Norway, Peru, Poland, Portugal, Republic of Korea, South Africa, Spain, Sweden, United Kingdom, United States

US States: AZ, CA, CO, FL, GA, IL, IN, KS, MO, NE, NJ, NY, OH, OK, OR, PA, SC, TN, TX, UT, VA, WI

Contact: Pfizer CT.gov Call Center [800-718-1021; ClinicalTrials.gov_Inquiries@pfizer.com]

Disclaimer: The data presented here is from a curated knowledgebase of publicly available information, but may not be exhaustive. The data version is 2021.11(003).

BRCA2 mutation (continued)

NCT04821622

TALAPRO-3: A Phase III, Randomized, Double-blind, Study of Talazoparib With Enzalutamide Versus Placebo With Enzalutamide in Men With DDR Gene Mutated Metastatic Castration-sensitive Prostate Cancer

Cancer type: Prostate Adenocarcinoma

Variant class: DNA repair mutation

Other identifiers: C3441052, CTR20212191, EudraCT Number: 2021-000248-23, jRCT2031210098, TALAPRO-3

Population segments: Second line, Stage IV

Phase: III

Therapies: talazoparib, hormone therapy

Locations: Japan, Republic of Korea, United States

US States: CA, DC, FL, GA, IL, MI, NJ, NY, PA, SC, TX, UT

Contact: Pfizer CT.gov Call Center [800-718-1021; ClinicalTrials.gov_Inquiries@pfizer.com]

NCT04038502

An Open-label, Multicenter Phase II Study to Compare the Efficacy of Carboplatin as First-line Followed by Second-line Olaparib Versus Olaparib as First-line Followed by Second-line Carboplatin in the Treatment of Patients With Castration Resistant Prostate Cancer Containing Homologous Recombination Deficiency

Cancer type: Castration-Resistant Prostate Cancer

Variant class: DNA repair mutation

Other identifiers: 01781, 01783, 19-08, BRACeD, CX001963-01, ONCA-009-18F

Population segments: First line, Hormone refractory, Second line, Stage IV

Phase: II

Therapies: olaparib, chemotherapy

Location: United States

US States: CA, DC, FL, IL, MI, NC, NY, PA, WA

Contact: Dr. Robert B. Montgomery [206-277-6878; rbmontgo@u.washington.edu]

No NCT ID - see other identifier(s)

Single-arm Exploratory Study for Sintilimab Second-line or Later-line Treatment of Advanced Non-small Cell Lung Cancer with DDR Pathway Gene Mutation

Cancer type: Non-Small Cell Lung Cancer

Variant class: DNA repair mutation

Other identifier: ChiCTR1900023234

Population segments: Fourth line or greater, Second line, Stage III, Stage IV, Third line

Exclusion criteria variant classes: ALK fusion (Non-Small Cell Lung Cancer), EGFRi sensitizing mutation (Non-Small Cell Lung Cancer), ROS1 fusion (Non-Small Cell Lung Cancer)

Phase: I/II

Therapy: sintilimab

Location: China

BRCA2 mutation (continued)

NCT04095273

A Multicenter, Non-randomized, Open-label Phase Ib Study to Determine the Maximum Tolerated and Recommended Phase II Dose of the ATR Inhibitor BAY1895344 in Combination With Pembrolizumab and to Characterize Its Safety, Tolerability, Pharmacokinetics and Preliminary Anti-tumor Activity in Patients With Advanced Solid Tumors

Cancer type: Breast Cancer, Castration-Resistant Prostate Cancer, Colorectal Cancer, Gastric Cancer, Gastroesophageal Junction Adenocarcinoma, Non-Small Cell Lung Cancer, Pancreatic Cancer

Variant class: DNA repair mutation

Other inclusion criteria: ATM mutation negative or ATM mutation negative, ERBB2 negative, Hormone receptor positive or ATM underexpression

Other identifiers: 19741, EudraCT Number: 2018-003420-36, Keynote 919, NCI-2019-06734

Population segments: HER2 negative, Hormone refractory, Second line, Stage III, Stage IV

Phase: I

Therapies: BAY-1895344, pembrolizumab

Locations: Germany, Spain, Switzerland, United States

US States: CA, CT, MA, MD, NC, NY, OH, TX

Contact: Bayer Clinical Trials Contact [clinical-trials-contact@bayer.com]

NCT04849364

A Phase II Circulating Tumor DNA Enriched, Genomically Directed Post-neoadjuvant Trial for Patients With Residual Triple Negative Breast Cancer (PERSEVERE)

Cancer type: Triple Negative Breast Cancer

Variant class: DNA repair pathway

Other identifiers: HCRN BRE18-334, PERSEVERE

Population segments: Adjuvant, HER2 negative, Stage I, Stage II, Stage III, Triple receptor negative

Phase: II

Therapies: talazoparib, chemotherapy

Location: United States

US State: IN

Contact: Dr. Bryan P. Schneider [317-948-3885; bpschnei@iu.edu]

BRCA2 splice site mutation

NCT02029001
A Two-period, Multicenter, Randomized, Open-label, Phase II Study Evaluating the Clinical Benefit of a Maintenance Treatment Targeting Tumor Molecular Alterations in Patients with Progressive Locally-advanced or Metastatic Solid Tumors.

Cancer type: Unspecified Solid Tumor

Variant class: BRCA2 mutation

Other identifiers: ET12-081, EudraCT Number: 2012-004510-34, MOST, ProfiLER

Population segments: Maintenance/Consolidation, Second line, Stage III, Stage IV, Third line

Exclusion criteria variant classes: ATM mutation (Gastric Cancer), BRCA1 germline mutation (Gastric Cancer), BRCA1 mutation (Gastric Cancer), BRCA2 germline mutation (Gastric Cancer), BRCA2 mutation (Gastric Cancer), HRR mutation (Gastric Cancer), PALB2 mutation (Gastric Cancer), RAD51 mutation (Gastric Cancer)

Phase: II

Therapy: olaparib

Location: France

NCT03967938
Efficacy of Olaparib in Advanced Cancers Occurring in Patients With Germline Mutations or Somatic Tumor Mutations in Homologous Recombination Genes

Cancer type: Unspecified Solid Tumor

Variant class: BRCA2 mutation

Other identifiers: 1-2018 BSMO, 1-2018BSMO, EudraCT Number: 2018-002966-37, Precision 2 - Olaparib

Population segments: Line of therapy N/A, Stage III, Stage IV

Exclusion criteria variant classes: BRCA1 germline mutation (Breast Cancer, Pancreatic Cancer, Prostate Cancer), BRCA2 germline mutation (Breast Cancer, Pancreatic Cancer, Prostate Cancer)

Phase: II

Therapy: olaparib

Location: Belgium

NCT02693535
Targeted Agent and Profiling Utilization Registry (TAPUR) Study.

Cancer type: Unspecified Solid Tumor

Variant class: BRCA2 mutation

Other identifiers: 20170529, NCI-2017-00510, Pro00014171, TAPUR

Population segments: Aggressive, BRAF, Diffuse large B-cell lymphoma (DLBCL), Extranodal marginal zone B-cell lymphoma (MALT), FGFR, First line, Follicular lymphoma (FL), Fourth line or greater, Indolent, Lymphoblastic lymphoma (LBL), Mantle cell lymphoma (MCL), Other subtype, Second line, Small lymphocytic lymphoma (SLL), Stage III, Stage IV, Third line, Waldenstrom's macroglobulinemia (WM)

Phase: II

Therapies: olaparib, talazoparib, atezolizumab + talazoparib

Location: United States

US States: AL, AZ, CA, FL, GA, HI, IL, IN, ME, MI, NC, ND, NE, NH, OH, OR, PA, SD, TX, UT, VA, WA

Contact: Pam Mangat [pam.mangat@asco.org]

Disclaimer: The data presented here is from a curated knowledgebase of publicly available information, but may not be exhaustive. The data version is 2021.11(003).

BRCA2 splice site mutation (continued)

NCT04171700
A Phase II Multicenter, Open-label Study of Rucaparib as Treatment for Solid Tumors Associated With Deleterious Mutations in Homologous Recombination Repair Genes

Cancer type: Unspecified Solid Tumor

Variant class: BRCA2 mutation

Other identifiers: 19-756, 20619, AAAS6974, CO-338-100, LODESTAR, NCI-2019-08226

Population segments: BRCA, Second line, Stage III, Stage IV

Exclusion criteria variant classes: BRCA1 germline mutation (Breast Cancer, Ovarian Cancer, Prostate Cancer), BRCA2 germline mutation (Breast Cancer, Ovarian Cancer, Prostate Cancer)

Phase: II

Therapy: rucaparib

Location: United States

US States: CA, FL, IA, IL, MA, MN, NY, OH, OK, PA, TN, WA

Contact: Clovis Oncology For North America [844-258-7662; medinfo@clovisoncology.com]

NCT02286687
Phase II Study of the PARP Inhibitor Talazoparib in Advanced Cancer Patients With Somatic Alterations in BRCA1/2, Mutations/Deletions in Other BRCA Pathway Genes and Germline Mutation in BRCA1/2 (Not Breast or Ovarian Cancer)

Cancer type: Unspecified Cancer

Variant class: BRCA2 mutation

Other identifiers: 2013-0961, NCI-2014-02494

Population segments: Fourth line or greater, Stage III, Stage IV

Exclusion criteria variant classes: BRCA1 germline mutation (Breast Cancer, Ovarian Cancer), BRCA2 germline mutation (Breast Cancer, Ovarian Cancer)

Phase: II

Therapy: talazoparib

Location: United States

US State: TX

Contact: Dr. Sarina Piha-Paul [713-563-1930; spihapau@mdanderson.org]

NCT04550494
A Pharmacodynamics-Driven Trial of Talazoparib, an Oral PARP Inhibitor, in Patients With Advanced Solid Tumors and Aberrations in Genes Involved in DNA Damage Response

Cancer type: Unspecified Solid Tumor

Variant class: BRCA2 mutation

Other identifiers: 000246, 000264-C, 10000264, 10371, NCI-2020-06906

Population segments: BRCA, Fourth line or greater, HER2 positive, Hormone refractory, Second line, Stage II, Stage III, Stage IV, Third line

Phase: II

Therapy: talazoparib

Location: United States

US State: MD

Contact: Multiple contacts: See www.clinicaltrials.gov for complete list of contacts.

Disclaimer: The data presented here is from a curated knowledgebase of publicly available information, but may not be exhaustive. The data version is 2021.11(003).

BRCA2 splice site mutation (continued)

NCT03842228
A Phase Ib Biomarker-Driven Combination Trial of Copanlisib, Olaparib, and Durvalumab (MEDI4736) in Patients With Advanced Solid Tumors

Cancer type: Unspecified Solid Tumor

Variant class: BRCA2 mutation

Other identifiers: 10217, 19-628, NCI10217, NCI-2019-00601

Population segments: Line of therapy N/A, Stage III, Stage IV

Phase: I

Therapies: copanlisib, olaparib, durvalumab

Location: United States

US States: MA, TX

Contact: Multiple contacts: See www.clinicaltrials.gov for complete list of contacts.

NCT04586335
Open Label, Phase Ib Study to Evaluate the Safety, Tolerability, Pharmacokinetics and Clinical Activity of CYH33, an Oral a-specific PI3K Inhibitor in Combination With Olaparib, an Oral PARP Inhibitor in Patients With Advanced Solid Tumors.

Cancer type: Breast Cancer, Endometrial Carcinoma, Ovarian Cancer, Prostate Cancer, Unspecified Solid Tumor

Variant class: BRCA2 mutation

Other identifiers: CTR20202339, CYH33-G102

Population segments: BRCA, Second line, Stage III, Stage IV

Phase: I

Therapies: HH-CYH33, olaparib

Locations: Australia, United States

US State: TX

Contact: Dr. Jason Sudia [908-380-1329; jason.sudia@haihepharma.com]

NCT03415659
A Phase I, Open-label, Single-center, Single/Multiple-dose, Dose-escalation/ Dose-expansion Clinical Study on Tolerance and Pharmacokinetics of HWH340 Tablet in Patients With Advanced Solid Tumors

Cancer type: Unspecified Solid Tumor

Variant class: BRCA2 mutation

Other identifiers: CTR20171649, HWH340-RFPA 20170821, RFPA 20170821

Population segments: BRCA, Fourth line or greater, HER2 negative, Second line, Stage III, Stage IV, Third line

Phase: I

Therapy: HWH-340

Location: China

NCT04197713
Phase I Sequential Trial of Agents Against DNA Repair (STAR)

Cancer type: Unspecified Solid Tumor

Variant class: BRCA2 mutation

Other identifiers: (STAR), 10329, 2020-0347, NCI-2019-08262, STAR Study

Population segments: Second line, Stage III, Stage IV

Phase: I

Therapies: olaparib, adavosertib

Location: United States

US State: TX

Contact: Site Public Contact [877-632-6789; askmdanderson@mdanderson.org]

Disclaimer: The data presented here is from a curated knowledgebase of publicly available information, but may not be exhaustive. The data version is 2021.11(003).

BRCA2 splice site mutation (continued)

NCT03297606
Canadian Profiling and Targeted Agent Utilization Trial (CAPTUR): A Phase II Basket Trial

Cancer type: Unspecified Solid Tumor

Variant class: BRCA2 aberration

Other identifiers: CA209-9DL, CAPTUR, ESR-17-12831, ML39800, PM1, WI233446

Population segments: Aggressive, Diffuse large B-cell lymphoma (DLBCL), Extranodal marginal zone B-cell lymphoma (MALT), First line, Follicular lymphoma (FL), Indolent, Lymphoblastic lymphoma (LBL), Mantle cell lymphoma (MCL), Other subtype, Second line, Stage III, Stage IV, Waldenstrom's macroglobulinemia (WM)

Phase: II

Therapy: olaparib

Location: Canada

NCT04497116
Phase I/IIa Study of the Safety, Pharmacokinetics, Pharmacodynamics and Preliminary Clinical Activity of RP-3500 Alone or in Combination With Talazoparib in Advanced Solid Tumors With ATR Inhibitor Sensitizing Mutations (TRESR Study)

Cancer type: Unspecified Solid Tumor

Variant class: BRCA2 aberration

Other identifiers: 20-521, EudraCT Number: 2020-000301-87, IRAS ID: 286609, NCI-2020-06869, RP-3500-01, TRESR, TRESR Study

Population segments: Second line, Stage III, Stage IV

Phase: I/II

Therapies: RP-3500, talazoparib

Locations: Canada, Denmark, United Kingdom, United States

US States: MA, NC, NY, RI, TN, TX

Contact: Dr. Peter Manley [857-322-5553; pmanley@reparerx.com]

NCT04123366
A Phase II Study of Olaparib in Combination With Pembrolizumab in Participants With Previously Treated, Homologous Recombination Repair Mutation (HRRm) and/or Homologous Recombination Deficiency (HRD)-Positive Advanced Cancer

Cancer type: Unspecified Solid Tumor

Variant class: HRR mutation

Other identifiers: 7339-007, EudraCT Number: 2019-001745-40, jRCT2011200025, KEYLYNK-007, MK-7339-007, NCI-2019-08758, PER-029-19

Population segments: BRCA, Second line, Stage III, Stage IV

Exclusion criteria variant classes: BRCA germline mutation (Breast Cancer, Ovarian Cancer), BRCA mutation (Breast Cancer, Ovarian Cancer)

Phase: II

Therapies: olaparib, pembrolizumab

Locations: Argentina, Australia, Canada, Colombia, France, Germany, Guatemala, Israel, Italy, Japan, Latvia, Mexico, Peru, Poland, Puerto Rico, Republic of Korea, Romania, South Africa, Spain, Sweden, Turkey, Ukraine, United States

US States: AL, AZ, CA, CO, FL, GA, KY, NJ, NY, OH, TX, UT, VA

Contact: Toll Free Number [888-577-8839; Trialsites@merck.com]

Disclaimer: The data presented here is from a curated knowledgebase of publicly available information, but may not be exhaustive. The data version is 2021.11(003).

BRCA2 splice site mutation (continued)

NCT03767075
Basket of Baskets: A Modular, Open-label, Phase II, Multicentre Study To Evaluate Targeted Agents in Molecularly Selected Populations With Advanced Solid Tumours

Cancer type: Unspecified Solid Tumor

Variant class: DNA repair mutation

Other identifiers: (Basket of Baskets) (BoB), EudraCT number: 2017-005108-89, MO39164, NL65937.031.18, VHIO17002

Population segments: Second line, Stage III, Stage IV

Phase: II

Therapy: atezolizumab

Locations: France, Germany, Netherlands, Spain, Sweden, United Kingdom

---

NCT04905914
A Phase I/IIa, Open-Label, Safety, Pharmacokinetic, And Preliminary Efficacy Study Of Oral ATRN-119 In Patients With Advanced Solid Tumors

Cancer type: Unspecified Solid Tumor

Variant class: DNA repair mutation

Other identifier: AR-276-01

Population segments: Line of therapy N/A, Stage III, Stage IV

Phase: I/II

Therapy: ATRN-119

Location: United States

US State: MA

Contact: Robert Hasson [619-540-6253; rhasson@pacificlinkconsulting.com]

NCT04901702
A Randomized Phase I/II Study of Talazoparib or Temozolomide in Combination With Onivyde in Children With Recurrent Solid Malignancies and Ewing Sarcoma

Cancer type: Unspecified Solid Tumor

Variant class: HRR pathway

Other identifiers: EudraCT Number: 2021-003570-31, ONITT

Population segments: Second line, Stage IV

Exclusion criteria variant classes: HRR germline pathway (Central Nervous System Neoplasm, Ewing Sarcoma), HRR pathway (Central Nervous System Neoplasm, Ewing Sarcoma)

Phase: I/II

Therapies: talazoparib, chemotherapy

Locations: Canada, United States

US State: TN

Contact: Dr. Sara Federico [866-278-5833; referralinfo@stjude.org]

Disclaimer: The data presented here is from a curated knowledgebase of publicly available information, but may not be exhaustive. The data version is 2021.11(003).

BRCA2 splice site mutation (continued)

NCT03155620
NCI-COG Pediatric MATCH (Molecular Analysis for Therapy Choice) Screening Protocol

Cancer type: Unspecified Solid Tumor

Variant class: DNA repair pathway

Other identifiers: 17-729, 2017-0625, 20170841, APEC1621SC, COGAPEC1621, COGAPEC1621SC, N 55017 SC, NCI-2017-01251, NCI-COG Pediatric MATCH, Pediatric MATCH

Population segments: ALK, Aggressive, BRAF, FGFR, HRAS, Indolent, KRAS, NRAS, Pediatric or Adolescent, Second line, Stage III, Stage IV

Phase: II

Therapy: olaparib

Locations: Puerto Rico, United States

US States: AK, AL, AR, AZ, CA, CO, CT, DC, DE, FL, GA, HI, IA, ID, IL, IN, KY, LA, MA, MD, ME, MI, MN, MO, MS, NC, NE, NH, NJ, NV, NY, OH, OK, OR, PA, RI, SC, SD, TN, TX, UT, VA, VT, WA, WI

Contact: Multiple contacts: See www.clinicaltrials.gov for complete list of contacts.

NCT03233204
NCI-COG Pediatric MATCH (Molecular Analysis for Therapy Choice)- Phase 2 Subprotocol of Olaparib in Patients With Tumors Harboring Defects in DNA Damage Repair Genes

Cancer type: Unspecified Solid Tumor

Variant class: DNA repair pathway

Other identifiers: 20170841, APEC1621H, NCI-2017-00766

Population segments: Aggressive, Indolent, Pediatric or Adolescent, Second line, Stage III, Stage IV

Phase: II

Therapy: olaparib

Locations: Puerto Rico, United States

US States: AK, AL, AR, AZ, CA, CO, DC, DE, FL, GA, IA, ID, IL, IN, KY, LA, MD, ME, MI, MN, MO, MS, NC, ND, NE, NJ, NY, OH, OK, OR, PA, SC, SD, TN, TX, UT, VA, VT, WA, WI

Contact: Multiple contacts: See www.clinicaltrials.gov for complete list of contacts.

NCT04267939
An Open-label Phase Ib Study to Determine the Maximum Tolerated and/or Recommended Phase II Dose of the ATR Inhibitor BAY 1895344 in Combination With PARP Inhibitor Niraparib, in Patients With Recurrent Advanced Solid Tumors and Ovarian Cancer

Cancer type: Ovarian Cancer, Unspecified Solid Tumor

Variant class: DNA repair pathway

Other identifiers: 18595, 19-810, 2019-0471, 20-222, EudraCT Number: 2018-003930-34, NCI-2020-01405

Population segments: Maintenance/Consolidation, Second line, Stage III, Stage IV

Exclusion criteria variant class: DNA repair pathway (Prostate Cancer)

Phase: I

Therapies: BAY-1895344, niraparib

Location: United States

US States: MA, NY, TX

Contact: Bayer Clinical Trials Contact [888-842-2937; clinical-trials-contact@bayer.com]

BRCA2 splice site mutation (continued)

NCT04693468
Modular Phase IB Hypothesis-Testing, Biomarker-Driven, Talazoparib Combination Trial (TalaCom)

Cancer type: Unspecified Solid Tumor

Variant class: DNA repair pathway

Other identifiers: 2020-0436, NCI-2020-06041, TalaCom

Population segments: Hormone refractory, Second line, Stage III, Stage IV

Phase: I

Therapies: talazoparib, palbociclib, axitinib, crizotinib

Location: United States

US State: TX

Contact: Timothy A. Yap [713-563-1784; tyap@mdanderson.org]

NCT03810105
A Phase II Study of Olaparib and Durvalumab in Men With Castration Sensitive Biochemically Recurrent Non-Metastatic Prostate Cancer Harboring Mutations in DNA Damage Repair

Cancer type: Prostate Cancer

Variant class: BRCA2 splice site mutation or DNA repair splice site mutation

Other identifiers: 18-480, NCI-2019-01627

Population segments: Adjuvant, Stage I, Stage II, Stage III

Phase: II

Therapies: durvalumab, olaparib

Location: United States

US States: CA, IL, MI, NJ, NY

Contact: Dr. Karen Autio [646-422-4632; autiok@mskcc.org]

NCT02975934
TRITON3: A Multicenter, Randomized, Open Label Phase III Study of Rucaparib Versus Physician's Choice of Therapy for Patients With Metastatic Castration Resistant Prostate Cancer Associated With Homologous Recombination Deficiency

Cancer type: Castration-Resistant Prostate Cancer

Variant class: BRCA2 mutation

Other identifiers: 16238, 1723GCCC, 18-146, 18-300, 20171185, 2018IS040, CO-338-063, CSET 2620, EudraCT Number: 2016-003163-20, GU 146, IGUP17020, IRAS ID: 222298, NCI-2017-01110, S16-02260, STAR Trial, TRITON 3_CO-338-063, TRITON3

Population segments: BRCA, First line, Hormone refractory, Stage IV

Phase: III

Therapies: rucaparib, hormone therapy

Locations: Australia, Belgium, Canada, Denmark, France, Germany, Ireland, Israel, Italy, Spain, United Kingdom, United States

US States: AZ, CA, CO, CT, DE, FL, HI, MD, MN, NC, NY, OH, OR, TN, TX, VA, WA

Contact: Clovis Oncology For North America [844-258-7662; medinfo@clovisoncology.com]

Disclaimer: The data presented here is from a curated knowledgebase of publicly available information, but may not be exhaustive. The data version is 2021.11(003).

BRCA2 splice site mutation (continued)

NCT04336943
Durvalumab (MEDI4736) and Olaparib (AZD2281) for Treatment of Biochemically Recurrent Prostate Cancer in Men Predicted to Have a High Neoantigen Load: A Pilot Study

Cancer type: Prostate Adenocarcinoma

Variant class: BRCA2 mutation

Other identifiers: 10509, NCI-2020-01860, RG1007001

Population segments: Line of therapy N/A, Stage I, Stage II, Stage III

Phase: II

Therapies: durvalumab, olaparib

Location: United States

US State: WA

Contact: Michael Schweizer [206-606-6252; schweize@uw.edu]

NCT04053322
An International Multicenter Phase II Trial of Durvalumab (MEDI4736) Plus OLAparib Plus Fulvestrant in Metastatic or Locally Advanced ER-positive, HER2-negative Breast Cancer Patients Selected Using Criteria That Predict Sensitivity to Olaparib

Cancer type: Breast Cancer

Variant class: BRCA2 mutation

Other inclusion criteria: ERBB2 negative, ER positive

Other identifiers: CADUSEIME08, DOLAF, EudraCT Number: 2018-003832-57, UC-0140/1812

Population segments: BRCA, Estrogen receptor positive, FGFR, HER2 negative, Progesterone receptor positive, Second line, Stage III, Stage IV

Phase: II

Therapies: durvalumab, olaparib, hormone therapy

Location: France

NCT03012321
BRCAAway: A Randomized Phase II Trial of Abiraterone, Olaparib, or Abiraterone + Olaparib in Patients With Metastatic Castration-Resistant Prostate Cancer With DNA Repair Defects

Cancer type: Castration-Resistant Prostate Cancer

Variant class: BRCA2 mutation

Other identifiers: 19137, BRCAAway, NCI-2016-01834, NU 16U05, NU_16U05, PCCTC #: c16-168, PCCTC c16-168, STU00203960

Population segments: First line, Hormone refractory, Stage IV

Phase: II

Therapies: hormone therapy, olaparib, steroid

Location: United States

US States: CA, FL, IL, IN, MD, MI, MN, MO, NC, NY, UT, VA

Contact: Study Coordinator [312-695-1301; cancertrials@northwestern.edu]

Disclaimer: The data presented here is from a curated knowledgebase of publicly available information, but may not be exhaustive. The data version is 2021.11(003).

BRCA2 splice site mutation (continued)

No NCT ID - see other identifier(s)
Phase II Study To Evaluate The Efficacy And Safety Of Niraparib In Recurrent Or Persistent Rare Gynecologic Malignancies With Homologous Recombination Deficiency (JGOG2052)

Cancer type: Cervical Cancer, Endometrial Carcinoma, Ovarian Cancer, Uterine Leiomyosarcoma

Variant class: BRCA2 mutation

Other identifiers: (JGOG2052), jRCT2031210264

Population segments: BRCA, Second line, Stage III, Stage IV

Phase: II

Therapy: niraparib

Location: Japan

NCT03553004
Niraparib in Metastatic Pancreatic Cancer After Previous Chemotherapy (NIRA-PANC): A Phase II Trial

Cancer type: Pancreatic Cancer

Variant class: BRCA2 mutation

Other identifiers: IIT-2017-NIRA-PANC, NIRA-PANC, STUDY00141862

Population segments: BRCA, Second line, Stage IV, Third line

Phase: II

Therapy: niraparib

Location: United States

US States: KS, MO

Contact: Clinical Trials Nurse Navigator [913-588-3671; navigation@kumc.edu]

NCT03601923
Phase II Proof-of-Concept Trial Testing the PARP Inhibitor Niraparib in Patients With Pancreatic Cancer Harboring Deficiencies in Homologous Recombination DNA Repair

Cancer type: Pancreatic Cancer

Variant class: BRCA2 mutation

Other identifiers: 18-207, NCI-2018-02395

Population segments: Second line, Stage IV

Phase: II

Therapy: niraparib

Location: United States

US State: MA

Contact: Dr. James Clreary [617-632-5960; JCLEARY@PARTNERS.ORG]

NCT03840967
A Phase II Study Evaluating Safety and Efficacy of Niraparib in Patients With Previously Treated Homologous Recombination (HR) Defective or Loss of Heterozygosity (LOH) High Metastatic Esophageal/Gastroesophageal Junction/ Proximal Gastric Adenocarcinoma

Cancer type: Esophageal Cancer, Gastric Cancer, Gastroesophageal Junction Adenocarcinoma

Variant class: BRCA2 mutation

Other identifiers: 20166, 71618, HCRN ESO17-325, NCI-2019-07395

Population segments: Second line, Stage III, Stage IV

Phase: II

Therapy: niraparib

Location: United States

US States: FL, IN, MI, NY

Contact: Dr. Shadia Jalal [317-274-3658; sjalal@iu.edu]

Disclaimer: The data presented here is from a curated knowledgebase of publicly available information, but may not be exhaustive. The data version is 2021.11(003).

BRCA2 splice site mutation (continued)

NCT03925350
A Phase II Study of Niraparib in Patients With Advanced Melanoma With Genetic Homologous Recombination (HR) Mutation / Alteration

Cancer type: Melanoma

Variant class: BRCA2 mutation

Other identifier: CPMC17-MEL01

Population segments: Second line, Stage III, Stage IV

Phase: II

Therapy: niraparib

Location: United States

US State: CA

Contact: Laurel Brechtel [415-600-1654; BrechtLA@sutterhealth.org]

NCT04493060
Phase II Study of Niraparib and TSR-042 in Patients With Germline or Somatic BRCA1/2 and PALB2-Related Pancreatic Cancer

Cancer type: Pancreatic Cancer

Variant class: BRCA2 mutation

Other identifiers: MC1841, NCI-2020-05352

Population segments: BRCA, Second line, Stage IV, Third line

Phase: II

Therapies: niraparib, dostarlimab

Location: United States

US States: AZ, FL, MN

Contact: Multiple contacts: See www.clinicaltrials.gov for complete list of contacts.

NCT04717154
Phase II INSPIRE Trial: Ipilimumab With Nivolumab In Molecular-Selected Patients With Castration-Resistant Prostate Cancer.

Cancer type: Castration-Resistant Prostate Cancer

Variant class: BRCA2 mutation

Other identifiers: CA184-585, EudraCT Number: 2020-001240-25, INSPIRE, MOURO048 - INSPIRE, MOURO48

Population segments: BRCA, First line, Hormone refractory, Second line, Stage IV

Phase: II

Therapies: nivolumab, ipilimumab

Location: Netherlands

NCT04187833
Phase II Trial of Nivolumab in Combination with Talazoparib in Patients with Unresectable or Metastatic Melanoma and Mutations in BRCA or BRCA-ness Genes.

Cancer type: Melanoma

Variant class: BRCA2 mutation

Other identifiers: CASE2619, NCI-2020-00416

Population segments: Second line, Stage III, Stage IV

Phase: II

Therapies: nivolumab, talazoparib

Location: United States

US State: OH

Contact: Dr. Pauline Funchain [866-223-8100; TaussigResearch@ccf.org]

BRCA2 splice site mutation (continued)

NCT03344965
A Phase II Study of Olaparib Monotherapy in Metastatic Breast Cancer Patients With Germline or Somatic Mutations in DNA Repair Genes (Olaparib Expanded)

Cancer type: Breast Cancer

Variant class: BRCA2 mutation

Other identifiers: 17-428, 18-414, NCI-2018-00778, TBCRC 048, TBCRC048

Population segments: BRCA, Estrogen receptor positive, HER2 negative, HER2 positive, Progesterone receptor positive, Second line, Stage IV

Exclusion criteria variant classes: BRCA1 germline mutation (Breast Cancer), BRCA2 germline mutation (Breast Cancer)

Phase: II

Therapy: olaparib

Location: United States

US States: AL, CA, IL, MA, MD, NC, NJ, NY, PA, WA

Contact: Dr. Nadine Tung [617-667-1962; ntung@bidmc.harvard.edu]

NCT03375307
A Phase II Study of Olaparib (AZD2281) in Patients With Metastatic/Advanced Urothelial Carcinoma With DNA-Repair Defects

Cancer type: Bladder Urothelial Carcinoma

Variant class: BRCA2 mutation

Other identifiers: 10144, 19C0023, NCI-2017-02296, P162941

Population segments: BRCA, STK11, Second line, Stage III, Stage IV

Phase: II

Therapy: olaparib

Location: United States

US States: CA, CO, KY, MD, NY, OK

Contact: Multiple contacts: See www.clinicaltrials.gov for complete list of contacts.

NCT03742895
A Phase II Study of Olaparib Monotherapy in Participants With Previously Treated, Homologous Recombination Repair Mutation (HRRm) or Homologous Recombination Deficiency (HRD) Positive Advanced Cancer

Cancer type: Breast Cancer

Variant class: BRCA2 mutation

Other inclusion criteria: BRCA1 germline mutation negative, BRCA2 germline mutation negative

Other identifiers: (MK-7339-002 / LYNK-002), 19-139, 194694, 2018-01891, 7339-002, 7339-002-00, EudraCT Number: 2018-003007-19, IRAS ID: 255158, JapicCTI-194694, LYNK002, LYNK-002, MK7339-002, MK-7339-002, MK7339-002-00, NCI-2018-03519, PER-046-18, SNCTP000003157

Population segments: BRCA, Fourth line or greater, Second line, Stage III, Stage IV, Third line

Exclusion criteria variant classes: BRCA germline mutation (Breast Cancer, Ovarian Cancer), BRCA mutation (Ovarian Cancer)

Phase: II

Therapy: olaparib

Locations: Argentina, Australia, Canada, Colombia, Denmark, France, Guatemala, Ireland, Israel, Italy, Mexico, Peru, Republic of Korea, Romania, Russian Federation, Spain, Switzerland, Turkey, United Kingdom, United States

US States: AZ, CA, CO, GA, KY, MA, MD, MI, NE, NJ, NY, SD, UT, WA

Contact: Toll Free Number [888-577-8839; Trialsites@merck.com]

Disclaimer: The data presented here is from a curated knowledgebase of publicly available information, but may not be exhaustive. The data version is 2021.11(003).

BRCA2 splice site mutation (continued)

NCT03786796
Phase II Study of Olaparib in Metastatic Renal Cell Carcinoma Patients Harboring a BAP-1 or Other DNA Repair Gene Mutations (ORCHID)

Cancer type: Kidney Cancer

Variant class: BRCA2 mutation

Other identifiers: CRMS-70750, J18166, NCI-2019-06309, ORCHID

Population segments: Fourth line or greater, Second line, Stage IV, Third line

Phase: II

Therapy: olaparib

Location: United States

US State: MD

Contact: Irina Rifkind [410-502-2043; irifkin1@jhmi.edu]

NCT04042831
A Phase II Study of Olaparib in Patients With Advanced Biliary Tract Cancer With Aberrant DNA Repair Gene Mutations

Cancer type: Biliary Tract Carcinoma

Variant class: BRCA2 mutation

Other identifiers: ACCRU-ICRN-1702, NCI-2019-04434

Population segments: Second line, Stage IV

Phase: II

Therapy: olaparib

Location: United States

US States: AZ, MN, NY, TX

Contact: Multiple contacts: See www.clinicaltrials.gov for complete list of contacts.

NCT04515836
Phase II Trial of Olaparib in Homologous Recombination Deficient (HRD) Malignant Mesothelioma

Cancer type: Mesothelioma

Variant class: BRCA2 mutation

Population segments: (N/A), Second line

Phase: II

Therapy: olaparib

Location: United States

US State: IL

Contact: Dr. Hedy L. Kindler [773-702-0360; hkindler@medicine.bsd.uchicago.edu]

NCT04858334
APOLLO: A Randomized Phase II Double-Blind Study of Olaparib Versus Placebo Following Adjuvant Chemotherapy in Patients With Resected Pancreatic Cancer and a Pathogenic BRCA1, BRCA2 or PALB2 Mutation

Cancer type: Pancreatic Cancer

Variant class: BRCA2 mutation

Other identifiers: APOLLO, CTSU/EA2192, EA2192, NCI-2020-05659

Population segments: Adjuvant, BRCA, Maintenance/Consolidation, Stage 0, Stage I, Stage II

Phase: II

Therapy: olaparib

Location: United States

US States: AZ, FL, IA, IL, IN, KS, MI, MO, NC, NJ, NM, NV, NY, OH, OR, PA, WA, WI

Contact: Multiple contacts: See www.clinicaltrials.gov for complete list of contacts.

Disclaimer: The data presented here is from a curated knowledgebase of publicly available information, but may not be exhaustive. The data version is 2021.11(003).

BRCA2 splice site mutation (continued)

NCT02849496

A Phase II Open-Label, Randomized Study of PARP Inhibition (Olaparib) Either Alone or in Combination With Anti-PD-L1 Therapy (Atezolizumab; MPDL3280A) in Homologous DNA Repair (HDR) Deficient, Locally Advanced or Metastatic Non-HER2-Positive Breast Cancer.

Cancer type: Breast Cancer

Variant class: BRCA2 mutation

Other inclusion criteria: ERBB2 negative

Other identifiers: 10020, 109587 (10020), 1608018258, 17-709, 18979, 5UM1CA186709-04, Clinical Trial 18979, NCI-2016-01130, OSU-17011, PHII-146

Population segments: BRCA, HER2 negative, Second line, Stage III, Stage IV

Phase: II

Therapies: olaparib, atezolizumab

Location: United States

US States: CA, CO, CT, FL, IL, MA, MD, MI, MO, NC, NY, OH, PA, TN, UT

Contact: Multiple contacts: See www.clinicaltrials.gov for complete list of contacts.

NCT04939662

Phase II, Single-arm Study of Olaparib and Bevacizumab Combination Therapy in Relapsed Small Cell Lung Cancer Subjects With DNA Damage Response and the Repair Pathway Alteration, ATM Deficiency, SLFN11 Positive, or POU2F3 Positive

Cancer type: Small Cell Lung Cancer

Variant class: BRCA2 mutation

Other identifier: SUKSES-B2

Population segments: (N/A), BRCA, Pulmonary, Second line, Third line

Phase: II

Therapies: olaparib, bevacizumab

Location: Republic of Korea

NCT04261465

The NUVOLA TRIAL: Neoadjuvant Chemotherapy in Unresectable Ovarian Cancer With OLAparib and Weekly Carboplatin Plus Paclitaxel: A Phase II Open-label Multicentre Study

Cancer type: Ovarian Cancer

Variant class: BRCA2 mutation

Other identifiers: 2317, 2345, EudraCT Number: 2018-002687-65, NUVOLA, NUVOLA trial

Population segments: BRCA, Maintenance/Consolidation, Neoadjuvant, Stage III, Stage IV

Phase: II

Therapies: olaparib, chemotherapy

Location: Italy

NCT04633902

Phase II Study of Olaparib in Combination With Pembrolizumab in Patients With Advanced Melanoma With Homologous Recombination (HR) Pathway Gene Mutation

Cancer type: Melanoma

Variant class: BRCA2 mutation

Other identifier: CPMC19-MEL01

Population segments: BRAF, Second line, Stage III, Stage IV

Phase: II

Therapies: olaparib, pembrolizumab

Location: United States

US State: CA

Contact: Dr. Kevin B. Kim [415-885-8600; ClinicalResearch@sutterhealth.org]

Disclaimer: The data presented here is from a curated knowledgebase of publicly available information, but may not be exhaustive. The data version is 2021.11(003).

BRCA2 splice site mutation (continued)

NCT03248570
Phase II Open Label Study of Pembrolizumab in Patients With Metastatic Castration Resistant Prostate Cancer (mCRPC) With or Without DNA Damage Repair Defects

Cancer type: Castration-Resistant Prostate Cancer

Variant class: BRCA2 mutation

Other identifiers: 16557, 17-22165, CC 16557, NCI-2017-02408

Population segments: BRCA, Hormone refractory, Line of therapy N/A, Stage IV

Phase: II

Therapy: pembrolizumab

Location: United States

US State: CA

Contact: Patricia Li [415-476-5975; GUTrials@ucsf.edu]

NCT03506997
PERSEUS1: Phase II Trial of the Immune Checkpoint Inhibitor Pembrolizumab for Patients Suffering From Metastatic Prostate Cancer

Cancer type: Castration-Resistant Prostate Cancer

Variant class: BRCA2 mutation

Other identifiers: CCR4559, EudraCT Number: 2017-000931-15, ICR-CTSU/2016/10060, IRAS ID 208952, PERSEUS1

Population segments: Hormone refractory, Second line, Stage IV

Phase: II

Therapy: pembrolizumab

Location: United Kingdom

NCT04089189
A Phase II Study to Evaluate the Efficacy, Safety, and Tolerability of IMP4297 Capsules in Subjects With Germline and/or Systemic BRCA1/2 Mutation Positive Advanced Ovarian Cancer With at Least 2 Prior Lines of Standard Systemic Therapy

Cancer type: Ovarian Cancer

Variant class: BRCA2 mutation

Other identifiers: CTR20190652, IMP4297-2018-CN01

Population segments: Stage III, Stage IV, Third line

Phase: II

Therapy: senaparib

Location: China

NCT03990896
Evaluation of Talazoparib, a PARP Inhibitor, in Patients With Somatic BRCA Mutant Metastatic Breast Cancer: Genotyping Based Clinical Trial

Cancer type: Breast Cancer, Triple Negative Breast Cancer

Variant class: BRCA2 mutation

Other inclusion criteria: ERBB2 negative, Hormone receptor positive

Other identifiers: 19-188, NCI-2019-07732

Population segments: BRCA, Estrogen receptor positive, Fourth line or greater, HER2 negative, Progesterone receptor positive, Second line, Stage IV, Third line, Triple receptor negative

Exclusion criteria variant classes: BRCA1 germline mutation (Breast Cancer, Triple Negative Breast Cancer), BRCA2 germline mutation (Breast Cancer, Triple Negative Breast Cancer)

Phase: II

Therapy: talazoparib

Location: United States

US States: MA, TX

Contact: Dr. Neelima Vidula [617-724-4000; nvidula@mgh.harvard.edu]

Disclaimer: The data presented here is from a curated knowledgebase of publicly available information, but may not be exhaustive. The data version is 2021.11(003).

BRCA2 splice site mutation (continued)

NCT04892693
Talazoparib in Advanced Breast Cancer Patients With Homologous Recombinant Deficiency: A Phase II Clinical and Exploratory Biomarker Study of Talazoparib

Cancer type: Breast Cancer

Variant class: BRCA2 mutation

Other identifier: H-2009-079-1157

Population segments: BRCA, Line of therapy N/A, Stage III, Stage IV

Phase: II

Therapy: talazoparib

Location: Republic of Korea

NCT04740190
Combination Talazoparib - Carboplatin for Recurrent High-grade Glioma With DNA Damage Repair Deficiency (DDRd)

Cancer type: Anaplastic Astrocytoma, Anaplastic Oligoastrocytoma, Anaplastic Oligodendroglioma, Anaplastic Pleomorphic Xanthoastrocytoma, Diffuse Midline Glioma, Glioblastoma

Variant class: BRCA2 mutation

Other identifiers: TAC-GReD, UW-20396

Population segments: (N/A), BRCA, Second line

Phase: II

Therapies: talazoparib, chemotherapy

Location: Hong Kong

NCT04253262
A Phase Ib/II Study of Copanlisib Combined With Rucaparib in Patients With Metastatic Castration-resistant Prostate Cancer

Cancer type: Castration-Resistant Prostate Cancer

Variant class: BRCA2 mutation

Other identifiers: BrUOG 360, BrUOG360

Population segments: Hormone refractory, Second line, Stage IV

Phase: I/II

Therapies: copanlisib, rucaparib

Location: United States

US State: RI

Contact: BrUOG [401-863-3000; BrUOG@brown.edu]

NCT03685331
Harnessing Olaparib, Palbociclib and Endocrine Therapy: A Phase I/II Trial of Olaparib, Palbociclib and Fulvestrant in Patients With BRCA Mutation-associated, Hormone Receptor-positive, Human Epidermal Growth Factor Receptor 2 (HER2)-Negative Metastatic Breast Cancer (HOPE)

Cancer type: Breast Cancer

Variant class: BRCA2 mutation

Other inclusion criteria: ERBB2 negative, Hormone receptor positive

Other identifiers: (HOPE), NCI-2019-04210, UPCC 21118

Population segments: BRCA, Estrogen receptor positive, First line, HER2 negative, Progesterone receptor positive, Second line, Stage III, Stage IV, Third line

Phase: I/II

Therapies: olaparib, palbociclib, hormone therapy

Location: United States

US State: PA

Contact: Alexandra Torres [855-216-0098; PennCancerTrials@emergingmed.com]

Disclaimer: The data presented here is from a curated knowledgebase of publicly available information, but may not be exhaustive. The data version is 2021.11(003).

BRCA2 splice site mutation (continued)

No NCT ID - see other identifier(s)
An Open-Label, Randomised, Phase I/II Trial Of Rucaparib Combined With Nivolumab + or - Ipilimumab To Augment Response In Homologous Repair Deficient Patients With Relapsed Ovarian, Primary Peritoneal And Fallopian Tube Cancer

Cancer type: Ovarian Cancer

Variant class: BRCA2 mutation

Other identifiers: CeNturIOn, CeNturIOn-2016, EudraCT Number: 2017-004780-13, ISRCTN10490346

Population segments: (N/A), BRCA, Fourth line or greater, Second line, Third line

Phase: I/II

Therapies: rucaparib, nivolumab, ipilimumab

Location: United Kingdom

NCT03964532
TALAVE: A Pilot Trial of Induction Talazoparib Followed by Combination of Talazoparib and Avelumab in Advanced Breast Cancer

Cancer type: Breast Cancer

Variant class: BRCA2 mutation

Other inclusion criteria: ERBB2 negative

Other identifiers: NCI-2019-06240, STUDY00000023, TALAVE

Population segments: Line of therapy N/A, Stage III, Stage IV

Phase: I/II

Therapies: talazoparib, avelumab

Location: United States

US States: DC, NC, UT

Contact: Dr. Julie Collins [202-444-2223; Julie.Collins@gunet.georgetown.edu]

NCT04673448
Phase IB Trial of Niraparib and TSR-042 in Patients With BRCA-Mutated Breast, Pancreas or Ovary Cancer

Cancer type: Breast Cancer, Ovarian Cancer, Pancreatic Cancer

Variant class: BRCA2 mutation

Other identifiers: 10020, NCI-2020-11636, RG1005140

Population segments: BRCA, Estrogen receptor positive, HER2 positive, Progesterone receptor positive, Second line, Stage III, Stage IV

Phase: I

Therapies: niraparib, dostarlimab

Location: United States

US State: WA

Contact: Elizabeth M. Swisher [206-543-3669; swishere@uw.edu]

NCT04598321
BrUOG 390: Neoadjuvant Treatment With Talazoparib for Women With Newly Diagnosed, Advanced Ovarian Cancer Associated With a Mutation in BRCA1 or BRCA2 (mBRCA)

Cancer type: Ovarian Cancer

Variant class: BRCA2 mutation

Other identifier: BrUOG 390

Population segments: BRCA, Maintenance/Consolidation, Neoadjuvant, Stage II, Stage III, Stage IV

Phase: I

Therapy: talazoparib

Location: United States

US State: RI

Contact: BrUOG [401-863-3000; BrUOG@Brown.edu]

BRCA2 splice site mutation (continued)

NCT03598270
A Phase III Randomized, Double-blinded Trial of Platinum-based Chemotherapy With or Without Atezolizumab Followed by Niraparib Maintenance With or Without Atezolizumab in Patients With Recurrent Ovarian, Tubal or Peritoneal Cancer and Platinum Treatment-free Interval (TFIp) >6 Months

Cancer type: Ovarian Cancer

Variant class: BRCA mutation

Other identifiers: AGO ovary 2.33, ANITA, BGOG-ov-41, ENGOT-ov41, ENGOT-OV41/GEICO 69-O/ANITA, ENGOT-ov41/GEICO/ANITA, ENGOT-Ov41/GEICO69-O/ANITA, EudraCT Number: 2018-000366-11, GEICO 69-O, GEICO-69-O

Population segments: (N/A), BRCA, Maintenance/Consolidation, Second line, Third line

Phase: III

Therapies: atezolizumab, chemotherapy, niraparib

Locations: Belgium, France, Germany, Italy, Spain

NCT03278717
International Phase III Randomised Study to Evaluate the Efficacy of Maintenance Therapy With Olaparib and Cediranib or Olaparib Alone in Patients With Relapsed Ovarian Cancer Following a Response to Platinum-based Chemotherapy

Cancer type: Ovarian Cancer

Variant class: BRCA mutation

Other identifiers: 36280, ACTRN12618000498291, ANZGOG 1415/2018, CRUK/15/074, CTC 0133, ENGOT-ov35, ENGOT-ov35/NCRI/ICON 9, EudraCT Number: 2017-000161-75, ICON9, NC174, OV26 (CRUK/UCL ICON9), UCL/14/0795

Population segments: BRCA, Maintenance/Consolidation, Second line, Stage III, Stage IV

Phase: III

Therapies: cediranib, olaparib

Locations: Australia, Canada, New Zealand, United Kingdom

NCT04915755
A Randomized Phase III Double-Blinded Study Comparing the Efficacy and Safety of Niraparib to Placebo in Participants With Either HER2-Negative BRCA-Mutated or Triple-Negative Breast Cancer With Molecular Disease Based on Presence of Circulating Tumor DNA After Definitive Therapy (ZEST)

Cancer type: Breast Cancer

Variant class: BRCA mutation

Other inclusion criteria: ERBB2 negative

Other identifiers: 213831, EudraCT Number: 2020-003973-23, GSK 213831 ZEST, SNCTP000004543, ZEST

Population segments: BRCA, Estrogen receptor positive, HER2 negative, Progesterone receptor positive, Second line, Stage I, Stage II, Stage III, Triple receptor negative

Phase: III

Therapy: niraparib

Locations: Canada, France, Ireland, Italy, Netherlands, Norway, Poland, Russian Federation, Spain, United Kingdom, United States

US State: IL

Contact: US GSK Clinical Trials Call Center [877-379-3718; GSKClinicalSupportHD@gsk.com]

Disclaimer: The data presented here is from a curated knowledgebase of publicly available information, but may not be exhaustive. The data version is 2021.11(003).

BRCA2 splice site mutation (continued)

NCT03462342
Combination ATR and PARP Inhibitor (CAPRI) Trial With AZD 6738 and Olaparib in Recurrent Ovarian Cancer

Cancer type: Ovarian Cancer

Variant class: BRCA mutation

Other identifiers: 20-320, CAPRI, ESR-16-12311, NCI-2018-02375, UPCC 15817

Population segments: BRCA, Fourth line or greater, Second line, Stage III, Stage IV, Third line

Phase: II

Therapies: ceralasertib, olaparib

Location: United States

US States: MA, MD, PA

Contact: Diego Rodriguez [215-614-0234; diegorod@pennmedicine.upenn.edu]

No NCT ID - see other identifier(s)
An Investigator-Initiated Phase II Trial Of Niraparib For Patients With BRCA-Mutated Unresectable / Recurrent Biliary Tract, Pancreatic And Other Gastrointestinal Cancer (NIR-B)

Cancer type: Cholangiocarcinoma, Colon Cancer, Gastrointestinal Stromal Tumor, Pancreatic Cancer, Rectal Cancer

Variant class: BRCA mutation

Other identifiers: jRCT2011200023, NIR-B

Population segments: BRCA, Second line, Stage III, Stage IV, Third line

Phase: II

Therapy: niraparib

Location: Japan

NCT04716686
Niraparib Monotherapy as Maintain and Recurrent Treatment of Endometrial Serous Carcinoma: A Multi-center, Open-label, Prospective Clinical Study

Cancer type: Endometrial Serous Adenocarcinoma

Variant class: BRCA mutation

Other identifier: ZL-2306-914

Population segments: BRCA, Fourth line or greater, Maintenance/Consolidation, Second line, Stage III, Stage IV, Third line

Phase: II

Therapy: niraparib

Location: China

NCT04348045
MAZEPPA: Phase II PRODIGE-GERCOR Study to Evaluate MAintenance Therapy With Olaparib or Selumetinib Plus Durvalumab According to BRCAness and KRAS Somatic Status Personalized in Metastatic Pancreatic Adenocarcinoma Patients

Cancer type: Pancreatic Cancer

Variant class: BRCA mutation

Other inclusion criteria: DPYD wild type

Other identifiers: D19-02, EudraCT Number: 2019-004366-18, MAZEPPA D19-02, MAZEPPA D19-02 PRODIGE 72, MAZEPPA_D19-02, PRODIGY 72 - MAZEPPA

Population segments: BRCA, KRAS, Maintenance/Consolidation, Stage IV

Phase: II

Therapy: olaparib

Location: France

Disclaimer: The data presented here is from a curated knowledgebase of publicly available information, but may not be exhaustive. The data version is 2021.11(003).

BRCA2 splice site mutation (continued)

NCT03025035

Open Label, Phase II Pilot Study of Immune Checkpoint Inhibition With Pembrolizumab in Combination With PARP Inhibition With Olaparib in Advanced BRCA-mutated or HDR-defect Breast Cancers

Cancer type: Breast Cancer

Variant class: BRCA mutation

Other identifier: IIT2015-18-Mita-MK3475

Population segments: BRCA, Estrogen receptor positive, HER2 negative, HER2 positive, Progesterone receptor positive, Second line, Stage III, Stage IV, Triple receptor negative

Phase: II

Therapies: pembrolizumab, olaparib

Location: United States

US State: CA

Contact: Parisa Mirzadehgan [310-967-4387; Parisa.Mirzadehgan@cshs.org]

NCT04666740

A Phase II Trial to Evaluate the Safety and Antitumor Activity of Pembrolizumab and OLApaRib (POLAR) Maintenance for Patients With Metastatic Pancreatic Ductal Adenocarcinoma and Homologous Recombination Deficiency and/or Exceptional Treatment Response to Platinum-Based Therapy

Cancer type: Pancreatic Cancer

Variant class: BRCA2 aberration

Other identifier: 20-481

Population segments: BRCA, Maintenance/Consolidation, Stage IV

Phase: II

Therapies: pembrolizumab, olaparib

Location: United States

US States: NJ, NY

Contact: Dr. Wungki Park [646-888-4543; yaqubiea@mskcc.org]

NCT02264678

A Modular Phase I, Open-Label, Multicentre Study to Assess the Safety, Tolerability, Pharmacokinetics and Preliminary Anti-tumour Activity of Ceralasertib in Combination With Cytotoxic Chemotherapy and/or DNA Damage Repair/Novel Anti-cancer Agents in Patients With Advanced Solid Malignancies

Cancer type: Breast Cancer, Ovarian Cancer

Variant class: BRCA mutation

Other inclusion criteria: ERBB2 negative

Other identifiers: 0C-14-9, 16-031, 18-342, D5330C00004, EudraCT Number: 2014-002233-66, IRAS ID 160200, NCI-2015-00687, Study 4

Population segments: Adenocarcinoma, BRCA, HER2 negative, Pulmonary, Second line, Stage III, Stage IV, Third line, Triple receptor negative

Exclusion criteria variant class: ERBB2 positive (Breast Cancer)

Phase: I

Therapies: ceralasertib, olaparib

Locations: France, Republic of Korea, United Kingdom, United States

US States: CA, MA, NY

Contact: AstraZeneca Clinical Study Information Center [877-240-9479; information.center@astrazeneca.com]

Disclaimer: The data presented here is from a curated knowledgebase of publicly available information, but may not be exhaustive. The data version is 2021.11(003).

BRCA2 splice site mutation (continued)

NCT04169841

Precision Medicine Phase II Study Evaluating the Efficacy of a Double Immunotherapy by Durvalumab and Tremelimumab Combined With Olaparib in Patients With Solid Cancers and Carriers of Homologous Recombination Repair Genes Mutation in Response or Stable After Olaparib Treatment

Cancer type: Breast Cancer, Endometrial Carcinoma, Head and Neck Cancer, Kidney Cancer, Non-Small Cell Lung Cancer, Ovarian Cancer, Pancreatic Cancer, Prostate Cancer

Variant class: HRR mutation

Other identifier: GUIDE2REPAIR

Population segments: Fourth line or greater, Hormone refractory, Maintenance/ Consolidation, Second line, Stage III, Stage IV, Third line

Exclusion criteria variant classes: ALK fusion (Lung Cancer), BRAF mutation (Lung Cancer), EGFR mutation (Lung Cancer), ROS1 mutation (Lung Cancer)

Phase: II

Therapies: durvalumab, tremelimumab, olaparib

Location: France

NCT04030559

Phase II Trial of PARP Inhibitor Niraparib for Men With High Risk Prostate Cancer and DNA Damage Response Defects

Cancer type: Prostate Cancer

Variant class: HRR mutation

Other identifiers: 1412676, NCI-2019-04365, UCDCC#279

Population segments: First line, Neoadjuvant, Stage I, Stage II, Stage IV

Phase: II

Therapy: niraparib

Location: United States

US State: CA

Contact: Marc Dall'Era [916-734-3771; mdallera@ucdavis.edu]

NCT04940637

A Phase II, Open-Label, Single Arm, Prospective, Multicenter Study of Niraparib Plus Dostarlimab in Patients With Advanced NSCLC and/or MPM, and Positive for PD-L1 Expression and Germline or Somatic Mutations in the HRR Genes

Cancer type: Mesothelioma, Non-Small Cell Lung Cancer

Variant class: HRR mutation

Other inclusion criteria: ALK fusion negative, CD274 expression, EGFRi sensitizing mutation negative, ROS1 fusion negative or CD274 expression

Other identifiers: EudraCT Number: 2020-000109-10, UNITO-001, UNITO-001/2020

Population segments: Second line, Stage III, Stage IV

Exclusion criteria variant classes: ALK fusion (Non-Small Cell Lung Cancer), EGFRi sensitizing mutation (Non-Small Cell Lung Cancer), ROS1 fusion (Non-Small Cell Lung Cancer)

Phase: II

Therapies: niraparib, dostarlimab

Location: Italy

BRCA2 splice site mutation (continued)

NCT04890613
Phase Ib Expansion Study of CX-5461 in Patients With Solid Tumours and BRCA2 and/or PALB2 Mutation

Cancer type: Ovarian Cancer

Variant class: HRR mutation

Other identifier: CX-5461-04

Population segments: BRCA, Second line, Stage II, Stage III, Stage IV

Phase: I

Therapy: pidnarulex

Location: Canada

NCT05002868
A Multi-center, Open-label, Phase I/Ib Study to Assess the Safety, Pharmacokinetics and Anti-tumor Activity of RP12146, a Poly (ADP-ribose) Polymerase (PARP) Inhibitor, in Patients With Locally Advanced or Metastatic Solid Tumors

Cancer type: Biliary Tract Carcinoma, Breast Cancer, Colorectal Cancer, Endometrial Carcinoma, Gastric Cancer, Ovarian Cancer, Pancreatic Cancer, Prostate Cancer, Small Cell Lung Cancer

Variant class: HRR mutation

Other identifier: RP12146-2101

Population segments: Extensive, Pulmonary, Second line, Stage III, Stage IV

Exclusion criteria variant class: ERBB2 positive (Breast Cancer)

Phase: I

Therapy: RP12146

Location: Poland

NCT03395197
A Phase III, Randomized, Double-blind, Placebo-controlled Study Of Talazoparib With Enzalutamide In Metastatic Castration-resistant Prostate Cancer

Cancer type: Castration-Resistant Prostate Cancer

Variant class: DNA repair mutation

Other identifiers: BASIC-ROYAL / HIGH-ROYAL, C3441021, CSET 2938, CTR20191443, CTR20191444, EudraCT Number: 2017-003295-31, GU 170 - C3441021, HCC 19-054, JapicCTI-194665, LH19.022, MOH_2019-05-16_007127, NCI-2018-01651, Pfizer C3441021, R19062M, RECF3951, RWF_C3441021 TALAPRO-2, TALAPRO-2, TX228665, UCI-18-47, VICCURO17115

Population segments: First line, Hormone refractory, Second line, Stage III, Stage IV

Phase: III

Therapies: talazoparib, hormone therapy

Locations: Argentina, Australia, Belgium, Brazil, Canada, Chile, China, Czech Republic, Finland, France, Germany, Hungary, Israel, Italy, Japan, New Zealand, Norway, Peru, Poland, Portugal, Republic of Korea, South Africa, Spain, Sweden, United Kingdom, United States

US States: AZ, CA, CO, FL, GA, IL, IN, KS, MO, NE, NJ, NY, OH, OK, OR, PA, SC, TN, TX, UT, VA, WI

Contact: Pfizer CT.gov Call Center [800-718-1021; ClinicalTrials.gov_Inquiries@pfizer.com]

Disclaimer: The data presented here is from a curated knowledgebase of publicly available information, but may not be exhaustive. The data version is 2021.11(003).

BRCA2 splice site mutation (continued)

NCT04821622

TALAPRO-3: A Phase III, Randomized, Double-blind, Study of Talazoparib With Enzalutamide Versus Placebo With Enzalutamide in Men With DDR Gene Mutated Metastatic Castration-sensitive Prostate Cancer

Cancer type: Prostate Adenocarcinoma

Variant class: DNA repair mutation

Other identifiers: C3441052, CTR20212191, EudraCT Number: 2021-000248-23, jRCT2031210098, TALAPRO-3

Population segments: Second line, Stage IV

Phase: III

Therapies: talazoparib, hormone therapy

Locations: Japan, Republic of Korea, United States

US States: CA, DC, FL, GA, IL, MI, NJ, NY, PA, SC, TX, UT

Contact: Pfizer CT.gov Call Center [800-718-1021; ClinicalTrials.gov_Inquiries@pfizer.com]

NCT04038502

An Open-label, Multicenter Phase II Study to Compare the Efficacy of Carboplatin as First-line Followed by Second-line Olaparib Versus Olaparib as First-line Followed by Second-line Carboplatin in the Treatment of Patients With Castration Resistant Prostate Cancer Containing Homologous Recombination Deficiency

Cancer type: Castration-Resistant Prostate Cancer

Variant class: DNA repair mutation

Other identifiers: 01781, 01783, 19-08, BRACeD, CX001963-01, ONCA-009-18F

Population segments: First line, Hormone refractory, Second line, Stage IV

Phase: II

Therapies: olaparib, chemotherapy

Location: United States

US States: CA, DC, FL, IL, MI, NC, NY, PA, WA

Contact: Dr. Robert B. Montgomery [206-277-6878; rbmontgo@u.washington.edu]

No NCT ID - see other identifier(s)

Single-arm Exploratory Study for Sintilimab Second-line or Later-line Treatment of Advanced Non-small Cell Lung Cancer with DDR Pathway Gene Mutation

Cancer type: Non-Small Cell Lung Cancer

Variant class: DNA repair mutation

Other identifier: ChiCTR1900023234

Population segments: Fourth line or greater, Second line, Stage III, Stage IV, Third line

Exclusion criteria variant classes: ALK fusion (Non-Small Cell Lung Cancer), EGFRi sensitizing mutation (Non-Small Cell Lung Cancer), ROS1 fusion (Non-Small Cell Lung Cancer)

Phase: I/II

Therapy: sintilimab

Location: China

Disclaimer: The data presented here is from a curated knowledgebase of publicly available information, but may not be exhaustive. The data version is 2021.11(003).

BRCA2 splice site mutation (continued)

NCT04095273

A Multicenter, Non-randomized, Open-label Phase Ib Study to Determine the Maximum Tolerated and Recommended Phase II Dose of the ATR Inhibitor BAY1895344 in Combination With Pembrolizumab and to Characterize Its Safety, Tolerability, Pharmacokinetics and Preliminary Anti-tumor Activity in Patients With Advanced Solid Tumors

Cancer type: Breast Cancer, Castration-Resistant Prostate Cancer, Colorectal Cancer, Gastric Cancer, Gastroesophageal Junction Adenocarcinoma, Non-Small Cell Lung Cancer, Pancreatic Cancer

Variant class: DNA repair mutation

Other inclusion criteria: ATM mutation negative or ATM mutation negative, ERBB2 negative, Hormone receptor positive or ATM underexpression

Other identifiers: 19741, EudraCT Number: 2018-003420-36, Keynote 919, NCI-2019-06734

Population segments: HER2 negative, Hormone refractory, Second line, Stage III, Stage IV

Phase: I

Therapies: BAY-1895344, pembrolizumab

Locations: Germany, Spain, Switzerland, United States

US States: CA, CT, MA, MD, NC, NY, OH, TX

Contact: Bayer Clinical Trials Contact [clinical-trials-contact@bayer.com]

NCT04849364

A Phase II Circulating Tumor DNA Enriched, Genomically Directed Post-neoadjuvant Trial for Patients With Residual Triple Negative Breast Cancer (PERSEVERE)

Cancer type: Triple Negative Breast Cancer

Variant class: DNA repair pathway

Other identifiers: HCRN BRE18-334, PERSEVERE

Population segments: Adjuvant, HER2 negative, Stage I, Stage II, Stage III, Triple receptor negative

Phase: II

Therapies: talazoparib, chemotherapy

Location: United States

US State: IN

Contact: Dr. Bryan P. Schneider [317-948-3885; bpschnei@iu.edu]

Disclaimer: The data presented here is from a curated knowledgebase of publicly available information, but may not be exhaustive. The data version is 2021.11(003).

BRCA2 truncating mutation

NCT02029001

A Two-period, Multicenter, Randomized, Open-label, Phase II Study Evaluating the Clinical Benefit of a Maintenance Treatment Targeting Tumor Molecular Alterations in Patients with Progressive Locally-advanced or Metastatic Solid Tumors.

Cancer type: Unspecified Solid Tumor

Variant class: BRCA2 mutation

Other identifiers: ET12-081, EudraCT Number: 2012-004510-34, MOST, ProfiLER

Population segments: Maintenance/Consolidation, Second line, Stage III, Stage IV, Third line

Exclusion criteria variant classes: ATM mutation (Gastric Cancer), BRCA1 germline mutation (Gastric Cancer), BRCA1 mutation (Gastric Cancer), BRCA2 germline mutation (Gastric Cancer), BRCA2 mutation (Gastric Cancer), HRR mutation (Gastric Cancer), PALB2 mutation (Gastric Cancer), RAD51 mutation (Gastric Cancer)

Phase: II

Therapy: olaparib

Location: France

NCT03967938

Efficacy of Olaparib in Advanced Cancers Occurring in Patients With Germline Mutations or Somatic Tumor Mutations in Homologous Recombination Genes

Cancer type: Unspecified Solid Tumor

Variant class: BRCA2 mutation

Other identifiers: 1-2018 BSMO, 1-2018BSMO, EudraCT Number: 2018-002966-37, Precision 2 - Olaparib

Population segments: Line of therapy N/A, Stage III, Stage IV

Exclusion criteria variant classes: BRCA1 germline mutation (Breast Cancer, Pancreatic Cancer, Prostate Cancer), BRCA2 germline mutation (Breast Cancer, Pancreatic Cancer, Prostate Cancer)

Phase: II

Therapy: olaparib

Location: Belgium

NCT02693535

Targeted Agent and Profiling Utilization Registry (TAPUR) Study.

Cancer type: Unspecified Solid Tumor

Variant class: BRCA2 mutation

Other identifiers: 20170529, NCI-2017-00510, Pro00014171, TAPUR

Population segments: Aggressive, BRAF, Diffuse large B-cell lymphoma (DLBCL), Extranodal marginal zone B-cell lymphoma (MALT), FGFR, First line, Follicular lymphoma (FL), Fourth line or greater, Indolent, Lymphoblastic lymphoma (LBL), Mantle cell lymphoma (MCL), Other subtype, Second line, Small lymphocytic lymphoma (SLL), Stage III, Stage IV, Third line, Waldenstrom's macroglobulinemia (WM)

Phase: II

Therapies: olaparib, talazoparib, atezolizumab + talazoparib

Location: United States

US States: AL, AZ, CA, FL, GA, HI, IL, IN, ME, MI, NC, ND, NE, NH, OH, OR, PA, SD, TX, UT, VA, WA

Contact: Pam Mangat [pam.mangat@asco.org]

Disclaimer: The data presented here is from a curated knowledgebase of publicly available information, but may not be exhaustive. The data version is 2021.11(003).

BRCA2 truncating mutation (continued)

NCT04171700
A Phase II Multicenter, Open-label Study of Rucaparib as Treatment for Solid Tumors Associated With Deleterious Mutations in Homologous Recombination Repair Genes

Cancer type: Unspecified Solid Tumor

Variant class: BRCA2 mutation

Other identifiers: 19-756, 20619, AAAS6974, CO-338-100, LODESTAR, NCI-2019-08226

Population segments: BRCA, Second line, Stage III, Stage IV

Exclusion criteria variant classes: BRCA1 germline mutation (Breast Cancer, Ovarian Cancer, Prostate Cancer), BRCA2 germline mutation (Breast Cancer, Ovarian Cancer, Prostate Cancer)

Phase: II

Therapy: rucaparib

Location: United States

US States: CA, FL, IA, IL, MA, MN, NY, OH, OK, PA, TN, WA

Contact: Clovis Oncology For North America [844-258-7662; medinfo@clovisoncology.com]

NCT02286687
Phase II Study of the PARP Inhibitor Talazoparib in Advanced Cancer Patients With Somatic Alterations in BRCA1/2, Mutations/Deletions in Other BRCA Pathway Genes and Germline Mutation in BRCA1/2 (Not Breast or Ovarian Cancer)

Cancer type: Unspecified Cancer

Variant class: BRCA2 mutation

Other identifiers: 2013-0961, NCI-2014-02494

Population segments: Fourth line or greater, Stage III, Stage IV

Exclusion criteria variant classes: BRCA1 germline mutation (Breast Cancer, Ovarian Cancer), BRCA2 germline mutation (Breast Cancer, Ovarian Cancer)

Phase: II

Therapy: talazoparib

Location: United States

US State: TX

Contact: Dr. Sarina Piha-Paul [713-563-1930; spihapau@mdanderson.org]

NCT04550494
A Pharmacodynamics-Driven Trial of Talazoparib, an Oral PARP Inhibitor, in Patients With Advanced Solid Tumors and Aberrations in Genes Involved in DNA Damage Response

Cancer type: Unspecified Solid Tumor

Variant class: BRCA2 mutation

Other identifiers: 000246, 000264-C, 10000264, 10371, NCI-2020-06906

Population segments: BRCA, Fourth line or greater, HER2 positive, Hormone refractory, Second line, Stage II, Stage III, Stage IV, Third line

Phase: II

Therapy: talazoparib

Location: United States

US State: MD

Contact: Multiple contacts: See www.clinicaltrials.gov for complete list of contacts.

Disclaimer: The data presented here is from a curated knowledgebase of publicly available information, but may not be exhaustive. The data version is 2021.11(003).

BRCA2 truncating mutation (continued)

NCT03842228
A Phase Ib Biomarker-Driven Combination Trial of Copanlisib, Olaparib, and Durvalumab (MEDI4736) in Patients With Advanced Solid Tumors

Cancer type: Unspecified Solid Tumor

Variant class: BRCA2 mutation

Other identifiers: 10217, 19-628, NCI10217, NCI-2019-00601

Population segments: Line of therapy N/A, Stage III, Stage IV

Phase: I

Therapies: copanlisib, olaparib, durvalumab

Location: United States

US States: MA, TX

Contact: Multiple contacts: See www.clinicaltrials.gov for complete list of contacts.

NCT04586335
Open Label, Phase Ib Study to Evaluate the Safety, Tolerability, Pharmacokinetics and Clinical Activity of CYH33, an Oral a-specific PI3K Inhibitor in Combination With Olaparib, an Oral PARP Inhibitor in Patients With Advanced Solid Tumors.

Cancer type: Breast Cancer, Endometrial Carcinoma, Ovarian Cancer, Prostate Cancer, Unspecified Solid Tumor

Variant class: BRCA2 mutation

Other identifiers: CTR20202339, CYH33-G102

Population segments: BRCA, Second line, Stage III, Stage IV

Phase: I

Therapies: HH-CYH33, olaparib

Locations: Australia, United States

US State: TX

Contact: Dr. Jason Sudia [908-380-1329; jason.sudia@haihepharma.com]

NCT03415659
A Phase I, Open-label, Single-center, Single/Multiple-dose, Dose-escalation/ Dose-expansion Clinical Study on Tolerance and Pharmacokinetics of HWH340 Tablet in Patients With Advanced Solid Tumors

Cancer type: Unspecified Solid Tumor

Variant class: BRCA2 mutation

Other identifiers: CTR20171649, HWH340-RFPA 20170821, RFPA 20170821

Population segments: BRCA, Fourth line or greater, HER2 negative, Second line, Stage III, Stage IV, Third line

Phase: I

Therapy: HWH-340

Location: China

NCT04197713
Phase I Sequential Trial of Agents Against DNA Repair (STAR)

Cancer type: Unspecified Solid Tumor

Variant class: BRCA2 mutation

Other identifiers: (STAR), 10329, 2020-0347, NCI-2019-08262, STAR Study

Population segments: Second line, Stage III, Stage IV

Phase: I

Therapies: olaparib, adavosertib

Location: United States

US State: TX

Contact: Site Public Contact [877-632-6789; askmdanderson@mdanderson.org]

Disclaimer: The data presented here is from a curated knowledgebase of publicly available information, but may not be exhaustive. The data version is 2021.11(003).

BRCA2 truncating mutation (continued)

NCT03297606
Canadian Profiling and Targeted Agent Utilization Trial (CAPTUR): A Phase II Basket Trial

Cancer type: Unspecified Solid Tumor

Variant class: BRCA2 aberration

Other identifiers: CA209-9DL, CAPTUR, ESR-17-12831, ML39800, PM1, WI233446

Population segments: Aggressive, Diffuse large B-cell lymphoma (DLBCL), Extranodal marginal zone B-cell lymphoma (MALT), First line, Follicular lymphoma (FL), Indolent, Lymphoblastic lymphoma (LBL), Mantle cell lymphoma (MCL), Other subtype, Second line, Stage III, Stage IV, Waldenstrom's macroglobulinemia (WM)

Phase: II

Therapy: olaparib

Location: Canada

NCT04497116
Phase I/IIa Study of the Safety, Pharmacokinetics, Pharmacodynamics and Preliminary Clinical Activity of RP-3500 Alone or in Combination With Talazoparib in Advanced Solid Tumors With ATR Inhibitor Sensitizing Mutations (TRESR Study)

Cancer type: Unspecified Solid Tumor

Variant class: BRCA2 aberration

Other identifiers: 20-521, EudraCT Number: 2020-000301-87, IRAS ID: 286609, NCI-2020-06869, RP-3500-01, TRESR, TRESR Study

Population segments: Second line, Stage III, Stage IV

Phase: I/II

Therapies: RP-3500, talazoparib

Locations: Canada, Denmark, United Kingdom, United States

US States: MA, NC, NY, RI, TN, TX

Contact: Dr. Peter Manley [857-322-5553; pmanley@reparerx.com]

NCT04123366
A Phase II Study of Olaparib in Combination With Pembrolizumab in Participants With Previously Treated, Homologous Recombination Repair Mutation (HRRm) and/or Homologous Recombination Deficiency (HRD)-Positive Advanced Cancer

Cancer type: Unspecified Solid Tumor

Variant class: HRR mutation

Other identifiers: 7339-007, EudraCT Number: 2019-001745-40, jRCT2011200025, KEYLYNK-007, MK-7339-007, NCI-2019-08758, PER-029-19

Population segments: BRCA, Second line, Stage III, Stage IV

Exclusion criteria variant classes: BRCA germline mutation (Breast Cancer, Ovarian Cancer), BRCA mutation (Breast Cancer, Ovarian Cancer)

Phase: II

Therapies: olaparib, pembrolizumab

Locations: Argentina, Australia, Canada, Colombia, France, Germany, Guatemala, Israel, Italy, Japan, Latvia, Mexico, Peru, Poland, Puerto Rico, Republic of Korea, Romania, South Africa, Spain, Sweden, Turkey, Ukraine, United States

US States: AL, AZ, CA, CO, FL, GA, KY, NJ, NY, OH, TX, UT, VA

Contact: Toll Free Number [888-577-8839; Trialsites@merck.com]

Disclaimer: The data presented here is from a curated knowledgebase of publicly available information, but may not be exhaustive. The data version is 2021.11(003).

BRCA2 truncating mutation (continued)

NCT03767075
Basket of Baskets: A Modular, Open-label, Phase II, Multicentre Study To Evaluate Targeted Agents in Molecularly Selected Populations With Advanced Solid Tumours

Cancer type: Unspecified Solid Tumor

Variant class: DNA repair mutation

Other identifiers: (Basket of Baskets) (BoB), EudraCT number: 2017-005108-89, MO39164, NL65937.031.18, VHIO17002

Population segments: Second line, Stage III, Stage IV

Phase: II

Therapy: atezolizumab

Locations: France, Germany, Netherlands, Spain, Sweden, United Kingdom

---

NCT04905914
A Phase I/IIa, Open-Label, Safety, Pharmacokinetic, And Preliminary Efficacy Study Of Oral ATRN-119 In Patients With Advanced Solid Tumors

Cancer type: Unspecified Solid Tumor

Variant class: DNA repair mutation

Other identifier: AR-276-01

Population segments: Line of therapy N/A, Stage III, Stage IV

Phase: I/II

Therapy: ATRN-119

Location: United States

US State: MA

Contact: Robert Hasson [619-540-6253; rhasson@pacificlinkconsulting.com]

NCT04901702
A Randomized Phase I/II Study of Talazoparib or Temozolomide in Combination With Onivyde in Children With Recurrent Solid Malignancies and Ewing Sarcoma

Cancer type: Unspecified Solid Tumor

Variant class: HRR pathway

Other identifiers: EudraCT Number: 2021-003570-31, ONITT

Population segments: Second line, Stage IV

Exclusion criteria variant classes: HRR germline pathway (Central Nervous System Neoplasm, Ewing Sarcoma), HRR pathway (Central Nervous System Neoplasm, Ewing Sarcoma)

Phase: I/II

Therapies: talazoparib, chemotherapy

Locations: Canada, United States

US State: TN

Contact: Dr. Sara Federico [866-278-5833; referralinfo@stjude.org]

Disclaimer: The data presented here is from a curated knowledgebase of publicly available information, but may not be exhaustive. The data version is 2021.11(003).

BRCA2 truncating mutation (continued)

NCT03155620
NCI-COG Pediatric MATCH (Molecular Analysis for Therapy Choice) Screening Protocol

Cancer type: Unspecified Solid Tumor

Variant class: DNA repair pathway

Other identifiers: 17-729, 2017-0625, 20170841, APEC1621SC, COGAPEC1621, COGAPEC1621SC, N 55017 SC, NCI-2017-01251, NCI-COG Pediatric MATCH, Pediatric MATCH

Population segments: ALK, Aggressive, BRAF, FGFR, HRAS, Indolent, KRAS, NRAS, Pediatric or Adolescent, Second line, Stage III, Stage IV

Phase: II

Therapy: olaparib

Locations: Puerto Rico, United States

US States: AK, AL, AR, AZ, CA, CO, CT, DC, DE, FL, GA, HI, IA, ID, IL, IN, KY, LA, MA, MD, ME, MI, MN, MO, MS, NC, NE, NH, NJ, NV, NY, OH, OK, OR, PA, RI, SC, SD, TN, TX, UT, VA, VT, WA, WI

Contact: Multiple contacts: See www.clinicaltrials.gov for complete list of contacts.

NCT03233204
NCI-COG Pediatric MATCH (Molecular Analysis for Therapy Choice)- Phase 2 Subprotocol of Olaparib in Patients With Tumors Harboring Defects in DNA Damage Repair Genes

Cancer type: Unspecified Solid Tumor

Variant class: DNA repair pathway

Other identifiers: 20170841, APEC1621H, NCI-2017-00766

Population segments: Aggressive, Indolent, Pediatric or Adolescent, Second line, Stage III, Stage IV

Phase: II

Therapy: olaparib

Locations: Puerto Rico, United States

US States: AK, AL, AR, AZ, CA, CO, DC, DE, FL, GA, IA, ID, IL, IN, KY, LA, MD, ME, MI, MN, MO, MS, NC, ND, NE, NJ, NY, OH, OK, OR, PA, SC, SD, TN, TX, UT, VA, VT, WA, WI

Contact: Multiple contacts: See www.clinicaltrials.gov for complete list of contacts.

NCT04267939
An Open-label Phase Ib Study to Determine the Maximum Tolerated and/or Recommended Phase II Dose of the ATR Inhibitor BAY 1895344 in Combination With PARP Inhibitor Niraparib, in Patients With Recurrent Advanced Solid Tumors and Ovarian Cancer

Cancer type: Ovarian Cancer, Unspecified Solid Tumor

Variant class: DNA repair pathway

Other identifiers: 18595, 19-810, 2019-0471, 20-222, EudraCT Number: 2018-003930-34, NCI-2020-01405

Population segments: Maintenance/Consolidation, Second line, Stage III, Stage IV

Exclusion criteria variant class: DNA repair pathway (Prostate Cancer)

Phase: I

Therapies: BAY-1895344, niraparib

Location: United States

US States: MA, NY, TX

Contact: Bayer Clinical Trials Contact [888-842-2937; clinical-trials-contact@bayer.com]

Disclaimer: The data presented here is from a curated knowledgebase of publicly available information, but may not be exhaustive. The data version is 2021.11(003).

BRCA2 truncating mutation (continued)

NCT04693468
Modular Phase IB Hypothesis-Testing, Biomarker-Driven, Talazoparib Combination Trial (TalaCom)

Cancer type: Unspecified Solid Tumor

Variant class: DNA repair pathway

Other identifiers: 2020-0436, NCI-2020-06041, TalaCom

Population segments: Hormone refractory, Second line, Stage III, Stage IV

Phase: I

Therapies: talazoparib, palbociclib, axitinib, crizotinib

Location: United States

US State: TX

Contact: Timothy A. Yap [713-563-1784; tyap@mdanderson.org]

NCT03810105
A Phase II Study of Olaparib and Durvalumab in Men With Castration Sensitive Biochemically Recurrent Non-Metastatic Prostate Cancer Harboring Mutations in DNA Damage Repair

Cancer type: Prostate Cancer

Variant class: BRCA2 truncating mutation or BRCA2 mutation

Other identifiers: 18-480, NCI-2019-01627

Population segments: Adjuvant, Stage I, Stage II, Stage III

Phase: II

Therapies: durvalumab, olaparib

Location: United States

US States: CA, IL, MI, NJ, NY

Contact: Dr. Karen Autio [646-422-4632; autiok@mskcc.org]

NCT03840967
A Phase II Study Evaluating Safety and Efficacy of Niraparib in Patients With Previously Treated Homologous Recombination (HR) Defective or Loss of Heterozygosity (LOH) High Metastatic Esophageal/Gastroesophageal Junction/ Proximal Gastric Adenocarcinoma

Cancer type: Esophageal Cancer, Gastric Cancer, Gastroesophageal Junction Adenocarcinoma

Variant class: BRCA2 truncating mutation

Other identifiers: 20166, 71618, HCRN ESO17-325, NCI-2019-07395

Population segments: Second line, Stage III, Stage IV

Phase: II

Therapy: niraparib

Location: United States

US States: FL, IN, MI, NY

Contact: Dr. Shadia Jalal [317-274-3658; sjalal@iu.edu]

Disclaimer: The data presented here is from a curated knowledgebase of publicly available information, but may not be exhaustive. The data version is 2021.11(003).

BRCA2 truncating mutation (continued)

NCT02975934
TRITON3: A Multicenter, Randomized, Open Label Phase III Study of Rucaparib Versus Physician's Choice of Therapy for Patients With Metastatic Castration Resistant Prostate Cancer Associated With Homologous Recombination Deficiency

Cancer type: Castration-Resistant Prostate Cancer

Variant class: BRCA2 mutation

Other identifiers: 16238, 1723GCCC, 18-146, 18-300, 20171185, 2018IS040, CO-338-063, CSET 2620, EudraCT Number: 2016-003163-20, GU 146, IGUP17020, IRAS ID: 222298, NCI-2017-01110, S16-02260, STAR Trial, TRITON 3_CO-338-063, TRITON3

Population segments: BRCA, First line, Hormone refractory, Stage IV

Phase: III

Therapies: rucaparib, hormone therapy

Locations: Australia, Belgium, Canada, Denmark, France, Germany, Ireland, Israel, Italy, Spain, United Kingdom, United States

US States: AZ, CA, CO, CT, DE, FL, HI, MD, MN, NC, NY, OH, OR, TN, TX, VA, WA

Contact: Clovis Oncology For North America [844-258-7662; medinfo@clovisoncology.com]

NCT04336943
Durvalumab (MEDI4736) and Olaparib (AZD2281) for Treatment of Biochemically Recurrent Prostate Cancer in Men Predicted to Have a High Neoantigen Load: A Pilot Study

Cancer type: Prostate Adenocarcinoma

Variant class: BRCA2 mutation

Other identifiers: 10509, NCI-2020-01860, RG1007001

Population segments: Line of therapy N/A, Stage I, Stage II, Stage III

Phase: II

Therapies: durvalumab, olaparib

Location: United States

US State: WA

Contact: Michael Schweizer [206-606-6252; schweize@uw.edu]

NCT04053322
An International Multicenter Phase II Trial of Durvalumab (MEDI4736) Plus OLAparib Plus Fulvestrant in Metastatic or Locally Advanced ER-positive, HER2-negative Breast Cancer Patients Selected Using Criteria That Predict Sensitivity to Olaparib

Cancer type: Breast Cancer

Variant class: BRCA2 mutation

Other inclusion criteria: ERBB2 negative, ER positive

Other identifiers: CADUSEIME08, DOLAF, EudraCT Number: 2018-003832-57, UC-0140/1812

Population segments: BRCA, Estrogen receptor positive, FGFR, HER2 negative, Progesterone receptor positive, Second line, Stage III, Stage IV

Phase: II

Therapies: durvalumab, olaparib, hormone therapy

Location: France

Disclaimer: The data presented here is from a curated knowledgebase of publicly available information, but may not be exhaustive. The data version is 2021.11(003).

BRCA2 truncating mutation (continued)

NCT03012321
BRCAAway: A Randomized Phase II Trial of Abiraterone, Olaparib, or Abiraterone + Olaparib in Patients With Metastatic Castration-Resistant Prostate Cancer With DNA Repair Defects

Cancer type: Castration-Resistant Prostate Cancer

Variant class: BRCA2 mutation

Other identifiers: 19137, BRCAAway, NCI-2016-01834, NU 16U05, NU_16U05, PCCTC #: c16-168, PCCTC c16-168, STU00203960

Population segments: First line, Hormone refractory, Stage IV

Phase: II

Therapies: hormone therapy, olaparib, steroid

Location: United States

US States: CA, FL, IL, IN, MD, MI, MN, MO, NC, NY, UT, VA

Contact: Study Coordinator [312-695-1301; cancertrials@northwestern.edu]

No NCT ID - see other identifier(s)
Phase II Study To Evaluate The Efficacy And Safety Of Niraparib In Recurrent Or Persistent Rare Gynecologic Malignancies With Homologous Recombination Deficiency (JGOG2052)

Cancer type: Cervical Cancer, Endometrial Carcinoma, Ovarian Cancer, Uterine Leiomyosarcoma

Variant class: BRCA2 mutation

Other identifiers: (JGOG2052), jRCT2031210264

Population segments: BRCA, Second line, Stage III, Stage IV

Phase: II

Therapy: niraparib

Location: Japan

NCT03553004
Niraparib in Metastatic Pancreatic Cancer After Previous Chemotherapy (NIRA-PANC): A Phase II Trial

Cancer type: Pancreatic Cancer

Variant class: BRCA2 mutation

Other identifiers: IIT-2017-NIRA-PANC, NIRA-PANC, STUDY00141862

Population segments: BRCA, Second line, Stage IV, Third line

Phase: II

Therapy: niraparib

Location: United States

US States: KS, MO

Contact: Clinical Trials Nurse Navigator [913-588-3671; navigation@kumc.edu]

NCT03601923
Phase II Proof-of-Concept Trial Testing the PARP Inhibitor Niraparib in Patients With Pancreatic Cancer Harboring Deficiencies in Homologous Recombination DNA Repair

Cancer type: Pancreatic Cancer

Variant class: BRCA2 mutation

Other identifiers: 18-207, NCI-2018-02395

Population segments: Second line, Stage IV

Phase: II

Therapy: niraparib

Location: United States

US State: MA

Contact: Dr. James Clreary [617-632-5960; JCLEARY@PARTNERS.ORG]

Disclaimer: The data presented here is from a curated knowledgebase of publicly available information, but may not be exhaustive. The data version is 2021.11(003).

BRCA2 truncating mutation (continued)

NCT03925350
A Phase II Study of Niraparib in Patients With Advanced Melanoma With Genetic Homologous Recombination (HR) Mutation / Alteration

Cancer type: Melanoma

Variant class: BRCA2 mutation

Other identifier: CPMC17-MEL01

Population segments: Second line, Stage III, Stage IV

Phase: II

Therapy: niraparib

Location: United States

US State: CA

Contact: Laurel Brechtel [415-600-1654; BrechtLA@sutterhealth.org]

NCT04493060
Phase II Study of Niraparib and TSR-042 in Patients With Germline or Somatic BRCA1/2 and PALB2-Related Pancreatic Cancer

Cancer type: Pancreatic Cancer

Variant class: BRCA2 mutation

Other identifiers: MC1841, NCI-2020-05352

Population segments: BRCA, Second line, Stage IV, Third line

Phase: II

Therapies: niraparib, dostarlimab

Location: United States

US States: AZ, FL, MN

Contact: Multiple contacts: See www.clinicaltrials.gov for complete list of contacts.

NCT04717154
Phase II INSPIRE Trial: Ipilimumab With Nivolumab In Molecular-Selected Patients With Castration-Resistant Prostate Cancer.

Cancer type: Castration-Resistant Prostate Cancer

Variant class: BRCA2 mutation

Other identifiers: CA184-585, EudraCT Number: 2020-001240-25, INSPIRE, MOURO048 - INSPIRE, MOURO48

Population segments: BRCA, First line, Hormone refractory, Second line, Stage IV

Phase: II

Therapies: nivolumab, ipilimumab

Location: Netherlands

NCT04187833
Phase II Trial of Nivolumab in Combination with Talazoparib in Patients with Unresectable or Metastatic Melanoma and Mutations in BRCA or BRCA-ness Genes.

Cancer type: Melanoma

Variant class: BRCA2 mutation

Other identifiers: CASE2619, NCI-2020-00416

Population segments: Second line, Stage III, Stage IV

Phase: II

Therapies: nivolumab, talazoparib

Location: United States

US State: OH

Contact: Dr. Pauline Funchain [866-223-8100; TaussigResearch@ccf.org]

Disclaimer: The data presented here is from a curated knowledgebase of publicly available information, but may not be exhaustive. The data version is 2021.11(003).

BRCA2 truncating mutation (continued)

NCT03344965

A Phase II Study of Olaparib Monotherapy in Metastatic Breast Cancer Patients With Germline or Somatic Mutations in DNA Repair Genes (Olaparib Expanded)

Cancer type: Breast Cancer

Variant class: BRCA2 mutation

Other identifiers: 17-428, 18-414, NCI-2018-00778, TBCRC 048, TBCRC048

Population segments: BRCA, Estrogen receptor positive, HER2 negative, HER2 positive, Progesterone receptor positive, Second line, Stage IV

Exclusion criteria variant classes: BRCA1 germline mutation (Breast Cancer), BRCA2 germline mutation (Breast Cancer)

Phase: II

Therapy: olaparib

Location: United States

US States: AL, CA, IL, MA, MD, NC, NJ, NY, PA, WA

Contact: Dr. Nadine Tung [617-667-1962; ntung@bidmc.harvard.edu]

NCT03375307

A Phase II Study of Olaparib (AZD2281) in Patients With Metastatic/Advanced Urothelial Carcinoma With DNA-Repair Defects

Cancer type: Bladder Urothelial Carcinoma

Variant class: BRCA2 mutation

Other identifiers: 10144, 19C0023, NCI-2017-02296, P162941

Population segments: BRCA, STK11, Second line, Stage III, Stage IV

Phase: II

Therapy: olaparib

Location: United States

US States: CA, CO, KY, MD, NY, OK

Contact: Multiple contacts: See www.clinicaltrials.gov for complete list of contacts.

NCT03742895

A Phase II Study of Olaparib Monotherapy in Participants With Previously Treated, Homologous Recombination Repair Mutation (HRRm) or Homologous Recombination Deficiency (HRD) Positive Advanced Cancer

Cancer type: Breast Cancer

Variant class: BRCA2 mutation

Other inclusion criteria: BRCA1 germline mutation negative, BRCA2 germline mutation negative

Other identifiers: (MK-7339-002 / LYNK-002), 19-139, 194694, 2018-01891, 7339-002, 7339-002-00, EudraCT Number: 2018-003007-19, IRAS ID: 255158, JapicCTI-194694, LYNK002, LYNK-002, MK7339-002, MK-7339-002, MK7339-002-00, NCI-2018-03519, PER-046-18, SNCTP000003157

Population segments: BRCA, Fourth line or greater, Second line, Stage III, Stage IV, Third line

Exclusion criteria variant classes: BRCA germline mutation (Breast Cancer, Ovarian Cancer), BRCA mutation (Ovarian Cancer)

Phase: II

Therapy: olaparib

Locations: Argentina, Australia, Canada, Colombia, Denmark, France, Guatemala, Ireland, Israel, Italy, Mexico, Peru, Republic of Korea, Romania, Russian Federation, Spain, Switzerland, Turkey, United Kingdom, United States

US States: AZ, CA, CO, GA, KY, MA, MD, MI, NE, NJ, NY, SD, UT, WA

Contact: Toll Free Number [888-577-8839; Trialsites@merck.com]

Disclaimer: The data presented here is from a curated knowledgebase of publicly available information, but may not be exhaustive. The data version is 2021.11(003).

BRCA2 truncating mutation (continued)

NCT03786796
Phase II Study of Olaparib in Metastatic Renal Cell Carcinoma Patients Harboring a BAP-1 or Other DNA Repair Gene Mutations (ORCHID)

Cancer type: Kidney Cancer

Variant class: BRCA2 mutation

Other identifiers: CRMS-70750, J18166, NCI-2019-06309, ORCHID

Population segments: Fourth line or greater, Second line, Stage IV, Third line

Phase: II

Therapy: olaparib

Location: United States

US State: MD

Contact: Irina Rifkind [410-502-2043; irifkin1@jhmi.edu]

NCT04042831
A Phase II Study of Olaparib in Patients With Advanced Biliary Tract Cancer With Aberrant DNA Repair Gene Mutations

Cancer type: Biliary Tract Carcinoma

Variant class: BRCA2 mutation

Other identifiers: ACCRU-ICRN-1702, NCI-2019-04434

Population segments: Second line, Stage IV

Phase: II

Therapy: olaparib

Location: United States

US States: AZ, MN, NY, TX

Contact: Multiple contacts: See www.clinicaltrials.gov for complete list of contacts.

NCT04515836
Phase II Trial of Olaparib in Homologous Recombination Deficient (HRD) Malignant Mesothelioma

Cancer type: Mesothelioma

Variant class: BRCA2 mutation

Population segments: (N/A), Second line

Phase: II

Therapy: olaparib

Location: United States

US State: IL

Contact: Dr. Hedy L. Kindler [773-702-0360; hkindler@medicine.bsd.uchicago.edu]

NCT04858334
APOLLO: A Randomized Phase II Double-Blind Study of Olaparib Versus Placebo Following Adjuvant Chemotherapy in Patients With Resected Pancreatic Cancer and a Pathogenic BRCA1, BRCA2 or PALB2 Mutation

Cancer type: Pancreatic Cancer

Variant class: BRCA2 mutation

Other identifiers: APOLLO, CTSU/EA2192, EA2192, NCI-2020-05659

Population segments: Adjuvant, BRCA, Maintenance/Consolidation, Stage 0, Stage I, Stage II

Phase: II

Therapy: olaparib

Location: United States

US States: AZ, FL, IA, IL, IN, KS, MI, MO, NC, NJ, NM, NV, NY, OH, OR, PA, WA, WI

Contact: Multiple contacts: See www.clinicaltrials.gov for complete list of contacts.

Disclaimer: The data presented here is from a curated knowledgebase of publicly available information, but may not be exhaustive. The data version is 2021.11(003).

BRCA2 truncating mutation (continued)

NCT02849496

A Phase II Open-Label, Randomized Study of PARP Inhibition (Olaparib) Either Alone or in Combination With Anti-PD-L1 Therapy (Atezolizumab; MPDL3280A) in Homologous DNA Repair (HDR) Deficient, Locally Advanced or Metastatic Non-HER2-Positive Breast Cancer.

Cancer type: Breast Cancer

Variant class: BRCA2 mutation

Other inclusion criteria: ERBB2 negative

Other identifiers: 10020, 109587 (10020), 1608018258, 17-709, 18979, 5UM1CA186709-04, Clinical Trial 18979, NCI-2016-01130, OSU-17011, PHII-146

Population segments: BRCA, HER2 negative, Second line, Stage III, Stage IV

Phase: II

Therapies: olaparib, atezolizumab

Location: United States

US States: CA, CO, CT, FL, IL, MA, MD, MI, MO, NC, NY, OH, PA, TN, UT

Contact: Multiple contacts: See www.clinicaltrials.gov for complete list of contacts.

NCT04939662

Phase II, Single-arm Study of Olaparib and Bevacizumab Combination Therapy in Relapsed Small Cell Lung Cancer Subjects With DNA Damage Response and the Repair Pathway Alteration, ATM Deficiency, SLFN11 Positive, or POU2F3 Positive

Cancer type: Small Cell Lung Cancer

Variant class: BRCA2 mutation

Other identifier: SUKSES-B2

Population segments: (N/A), BRCA, Pulmonary, Second line, Third line

Phase: II

Therapies: olaparib, bevacizumab

Location: Republic of Korea

NCT04261465

The NUVOLA TRIAL: Neoadjuvant Chemotherapy in Unresectable Ovarian Cancer With OLAparib and Weekly Carboplatin Plus Paclitaxel: A Phase II Open-label Multicentre Study

Cancer type: Ovarian Cancer

Variant class: BRCA2 mutation

Other identifiers: 2317, 2345, EudraCT Number: 2018-002687-65, NUVOLA, NUVOLA trial

Population segments: BRCA, Maintenance/Consolidation, Neoadjuvant, Stage III, Stage IV

Phase: II

Therapies: olaparib, chemotherapy

Location: Italy

NCT04633902

Phase II Study of Olaparib in Combination With Pembrolizumab in Patients With Advanced Melanoma With Homologous Recombination (HR) Pathway Gene Mutation

Cancer type: Melanoma

Variant class: BRCA2 mutation

Other identifier: CPMC19-MEL01

Population segments: BRAF, Second line, Stage III, Stage IV

Phase: II

Therapies: olaparib, pembrolizumab

Location: United States

US State: CA

Contact: Dr. Kevin B. Kim [415-885-8600; ClinicalResearch@sutterhealth.org]

Disclaimer: The data presented here is from a curated knowledgebase of publicly available information, but may not be exhaustive. The data version is 2021.11(003).

BRCA2 truncating mutation (continued)

NCT03248570
Phase II Open Label Study of Pembrolizumab in Patients With Metastatic Castration Resistant Prostate Cancer (mCRPC) With or Without DNA Damage Repair Defects

Cancer type: Castration-Resistant Prostate Cancer

Variant class: BRCA2 mutation

Other identifiers: 16557, 17-22165, CC 16557, NCI-2017-02408

Population segments: BRCA, Hormone refractory, Line of therapy N/A, Stage IV

Phase: II

Therapy: pembrolizumab

Location: United States

US State: CA

Contact: Patricia Li [415-476-5975; GUTrials@ucsf.edu]

NCT03506997
PERSEUS1: Phase II Trial of the Immune Checkpoint Inhibitor Pembrolizumab for Patients Suffering From Metastatic Prostate Cancer

Cancer type: Castration-Resistant Prostate Cancer

Variant class: BRCA2 mutation

Other identifiers: CCR4559, EudraCT Number: 2017-000931-15, ICR-CTSU/2016/10060, IRAS ID 208952, PERSEUS1

Population segments: Hormone refractory, Second line, Stage IV

Phase: II

Therapy: pembrolizumab

Location: United Kingdom

NCT04089189
A Phase II Study to Evaluate the Efficacy, Safety, and Tolerability of IMP4297 Capsules in Subjects With Germline and/or Systemic BRCA1/2 Mutation Positive Advanced Ovarian Cancer With at Least 2 Prior Lines of Standard Systemic Therapy

Cancer type: Ovarian Cancer

Variant class: BRCA2 mutation

Other identifiers: CTR20190652, IMP4297-2018-CN01

Population segments: Stage III, Stage IV, Third line

Phase: II

Therapy: senaparib

Location: China

NCT03990896
Evaluation of Talazoparib, a PARP Inhibitor, in Patients With Somatic BRCA Mutant Metastatic Breast Cancer: Genotyping Based Clinical Trial

Cancer type: Breast Cancer, Triple Negative Breast Cancer

Variant class: BRCA2 mutation

Other inclusion criteria: ERBB2 negative, Hormone receptor positive

Other identifiers: 19-188, NCI-2019-07732

Population segments: BRCA, Estrogen receptor positive, Fourth line or greater, HER2 negative, Progesterone receptor positive, Second line, Stage IV, Third line, Triple receptor negative

Exclusion criteria variant classes: BRCA1 germline mutation (Breast Cancer, Triple Negative Breast Cancer), BRCA2 germline mutation (Breast Cancer, Triple Negative Breast Cancer)

Phase: II

Therapy: talazoparib

Location: United States

US States: MA, TX

Contact: Dr. Neelima Vidula [617-724-4000; nvidula@mgh.harvard.edu]

Disclaimer: The data presented here is from a curated knowledgebase of publicly available information, but may not be exhaustive. The data version is 2021.11(003).

BRCA2 truncating mutation (continued)

NCT04892693
Talazoparib in Advanced Breast Cancer Patients With Homologous Recombinant Deficiency: A Phase II Clinical and Exploratory Biomarker Study of Talazoparib

Cancer type: Breast Cancer

Variant class: BRCA2 mutation

Other identifier: H-2009-079-1157

Population segments: BRCA, Line of therapy N/A, Stage III, Stage IV

Phase: II

Therapy: talazoparib

Location: Republic of Korea

NCT04740190
Combination Talazoparib - Carboplatin for Recurrent High-grade Glioma With DNA Damage Repair Deficiency (DDRd)

Cancer type: Anaplastic Astrocytoma, Anaplastic Oligoastrocytoma, Anaplastic Oligodendroglioma, Anaplastic Pleomorphic Xanthoastrocytoma, Diffuse Midline Glioma, Glioblastoma

Variant class: BRCA2 mutation

Other identifiers: TAC-GReD, UW-20396

Population segments: (N/A), BRCA, Second line

Phase: II

Therapies: talazoparib, chemotherapy

Location: Hong Kong

NCT04253262
A Phase Ib/II Study of Copanlisib Combined With Rucaparib in Patients With Metastatic Castration-resistant Prostate Cancer

Cancer type: Castration-Resistant Prostate Cancer

Variant class: BRCA2 mutation

Other identifiers: BrUOG 360, BrUOG360

Population segments: Hormone refractory, Second line, Stage IV

Phase: I/II

Therapies: copanlisib, rucaparib

Location: United States

US State: RI

Contact: BrUOG [401-863-3000; BrUOG@brown.edu]

NCT03685331
Harnessing Olaparib, Palbociclib and Endocrine Therapy: A Phase I/II Trial of Olaparib, Palbociclib and Fulvestrant in Patients With BRCA Mutation-associated, Hormone Receptor-positive, Human Epidermal Growth Factor Receptor 2 (HER2)-Negative Metastatic Breast Cancer (HOPE)

Cancer type: Breast Cancer

Variant class: BRCA2 mutation

Other inclusion criteria: ERBB2 negative, Hormone receptor positive

Other identifiers: (HOPE), NCI-2019-04210, UPCC 21118

Population segments: BRCA, Estrogen receptor positive, First line, HER2 negative, Progesterone receptor positive, Second line, Stage III, Stage IV, Third line

Phase: I/II

Therapies: olaparib, palbociclib, hormone therapy

Location: United States

US State: PA

Contact: Alexandra Torres [855-216-0098; PennCancerTrials@emergingmed.com]

Disclaimer: The data presented here is from a curated knowledgebase of publicly available information, but may not be exhaustive. The data version is 2021.11(003).

BRCA2 truncating mutation (continued)

No NCT ID - see other identifier(s)
An Open-Label, Randomised, Phase I/II Trial Of Rucaparib Combined With Nivolumab + or - Ipilimumab To Augment Response In Homologous Repair Deficient Patients With Relapsed Ovarian, Primary Peritoneal And Fallopian Tube Cancer

Cancer type: Ovarian Cancer

Variant class: BRCA2 mutation

Other identifiers: CeNturIOn, CeNturIOn-2016, EudraCT Number: 2017-004780-13, ISRCTN10490346

Population segments: (N/A), BRCA, Fourth line or greater, Second line, Third line

Phase: I/II

Therapies: rucaparib, nivolumab, ipilimumab

Location: United Kingdom

NCT03964532
TALAVE: A Pilot Trial of Induction Talazoparib Followed by Combination of Talazoparib and Avelumab in Advanced Breast Cancer

Cancer type: Breast Cancer

Variant class: BRCA2 mutation

Other inclusion criteria: ERBB2 negative

Other identifiers: NCI-2019-06240, STUDY00000023, TALAVE

Population segments: Line of therapy N/A, Stage III, Stage IV

Phase: I/II

Therapies: talazoparib, avelumab

Location: United States

US States: DC, NC, UT

Contact: Dr. Julie Collins [202-444-2223; Julie.Collins@gunet.georgetown.edu]

NCT04673448
Phase IB Trial of Niraparib and TSR-042 in Patients With BRCA-Mutated Breast, Pancreas or Ovary Cancer

Cancer type: Breast Cancer, Ovarian Cancer, Pancreatic Cancer

Variant class: BRCA2 mutation

Other identifiers: 10020, NCI-2020-11636, RG1005140

Population segments: BRCA, Estrogen receptor positive, HER2 positive, Progesterone receptor positive, Second line, Stage III, Stage IV

Phase: I

Therapies: niraparib, dostarlimab

Location: United States

US State: WA

Contact: Elizabeth M. Swisher [206-543-3669; swishere@uw.edu]

NCT04598321
BrUOG 390: Neoadjuvant Treatment With Talazoparib for Women With Newly Diagnosed, Advanced Ovarian Cancer Associated With a Mutation in BRCA1 or BRCA2 (mBRCA)

Cancer type: Ovarian Cancer

Variant class: BRCA2 mutation

Other identifier: BrUOG 390

Population segments: BRCA, Maintenance/Consolidation, Neoadjuvant, Stage II, Stage III, Stage IV

Phase: I

Therapy: talazoparib

Location: United States

US State: RI

Contact: BrUOG [401-863-3000; BrUOG@Brown.edu]

BRCA2 truncating mutation (continued)

NCT03598270
A Phase III Randomized, Double-blinded Trial of Platinum-based Chemotherapy With or Without Atezolizumab Followed by Niraparib Maintenance With or Without Atezolizumab in Patients With Recurrent Ovarian, Tubal or Peritoneal Cancer and Platinum Treatment-free Interval (TFIp) >6 Months

Cancer type: Ovarian Cancer

Variant class: BRCA mutation

Other identifiers: AGO ovary 2.33, ANITA, BGOG-ov-41, ENGOT-ov41, ENGOT-OV41/GEICO 69-O/ANITA, ENGOT-ov41/GEICO/ANITA, ENGOT-Ov41/GEICO69-O/ANITA, EudraCT Number: 2018-000366-11, GEICO 69-O, GEICO-69-O

Population segments: (N/A), BRCA, Maintenance/Consolidation, Second line, Third line

Phase: III

Therapies: atezolizumab, chemotherapy, niraparib

Locations: Belgium, France, Germany, Italy, Spain

NCT03278717
International Phase III Randomised Study to Evaluate the Efficacy of Maintenance Therapy With Olaparib and Cediranib or Olaparib Alone in Patients With Relapsed Ovarian Cancer Following a Response to Platinum-based Chemotherapy

Cancer type: Ovarian Cancer

Variant class: BRCA mutation

Other identifiers: 36280, ACTRN12618000498291, ANZGOG 1415/2018, CRUK/15/074, CTC 0133, ENGOT-ov35, ENGOT-ov35/NCRI/ICON 9, EudraCT Number: 2017-000161-75, ICON9, NC174, OV26 (CRUK/UCL ICON9), UCL/14/0795

Population segments: BRCA, Maintenance/Consolidation, Second line, Stage III, Stage IV

Phase: III

Therapies: cediranib, olaparib

Locations: Australia, Canada, New Zealand, United Kingdom

NCT04915755
A Randomized Phase III Double-Blinded Study Comparing the Efficacy and Safety of Niraparib to Placebo in Participants With Either HER2-Negative BRCA-Mutated or Triple-Negative Breast Cancer With Molecular Disease Based on Presence of Circulating Tumor DNA After Definitive Therapy (ZEST)

Cancer type: Breast Cancer

Variant class: BRCA mutation

Other inclusion criteria: ERBB2 negative

Other identifiers: 213831, EudraCT Number: 2020-003973-23, GSK 213831 ZEST, SNCTP000004543, ZEST

Population segments: BRCA, Estrogen receptor positive, HER2 negative, Progesterone receptor positive, Second line, Stage I, Stage II, Stage III, Triple receptor negative

Phase: III

Therapy: niraparib

Locations: Canada, France, Ireland, Italy, Netherlands, Norway, Poland, Russian Federation, Spain, United Kingdom, United States

US State: IL

Contact: US GSK Clinical Trials Call Center [877-379-3718; GSKClinicalSupportHD@gsk.com]

Disclaimer: The data presented here is from a curated knowledgebase of publicly available information, but may not be exhaustive. The data version is 2021.11(003).

BRCA2 truncating mutation (continued)

NCT03462342
Combination ATR and PARP Inhibitor (CAPRI) Trial With AZD 6738 and Olaparib in Recurrent Ovarian Cancer

Cancer type: Ovarian Cancer

Variant class: BRCA mutation

Other identifiers: 20-320, CAPRI, ESR-16-12311, NCI-2018-02375, UPCC 15817

Population segments: BRCA, Fourth line or greater, Second line, Stage III, Stage IV, Third line

Phase: II

Therapies: ceralasertib, olaparib

Location: United States

US States: MA, MD, PA

Contact: Diego Rodriguez [215-614-0234; diegorod@pennmedicine.upenn.edu]

No NCT ID - see other identifier(s)
An Investigator-Initiated Phase II Trial Of Niraparib For Patients With BRCA-Mutated Unresectable / Recurrent Biliary Tract, Pancreatic And Other Gastrointestinal Cancer (NIR-B)

Cancer type: Cholangiocarcinoma, Colon Cancer, Gastrointestinal Stromal Tumor, Pancreatic Cancer, Rectal Cancer

Variant class: BRCA mutation

Other identifiers: jRCT2011200023, NIR-B

Population segments: BRCA, Second line, Stage III, Stage IV, Third line

Phase: II

Therapy: niraparib

Location: Japan

NCT04716686
Niraparib Monotherapy as Maintain and Recurrent Treatment of Endometrial Serous Carcinoma: A Multi-center, Open-label, Prospective Clinical Study

Cancer type: Endometrial Serous Adenocarcinoma

Variant class: BRCA mutation

Other identifier: ZL-2306-914

Population segments: BRCA, Fourth line or greater, Maintenance/Consolidation, Second line, Stage III, Stage IV, Third line

Phase: II

Therapy: niraparib

Location: China

NCT04348045
MAZEPPA: Phase II PRODIGE-GERCOR Study to Evaluate MAintenance Therapy With Olaparib or Selumetinib Plus Durvalumab According to BRCAness and KRAS Somatic Status Personalized in Metastatic Pancreatic Adenocarcinoma Patients

Cancer type: Pancreatic Cancer

Variant class: BRCA mutation

Other inclusion criteria: DPYD wild type

Other identifiers: D19-02, EudraCT Number: 2019-004366-18, MAZEPPA D19-02, MAZEPPA D19-02 PRODIGE 72, MAZEPPA_D19-02, PRODIGY 72 - MAZEPPA

Population segments: BRCA, KRAS, Maintenance/Consolidation, Stage IV

Phase: II

Therapy: olaparib

Location: France

Disclaimer: The data presented here is from a curated knowledgebase of publicly available information, but may not be exhaustive. The data version is 2021.11(003).

BRCA2 truncating mutation (continued)

NCT03025035

Open Label, Phase II Pilot Study of Immune Checkpoint Inhibition With Pembrolizumab in Combination With PARP Inhibition With Olaparib in Advanced BRCA-mutated or HDR-defect Breast Cancers

Cancer type: Breast Cancer

Variant class: BRCA mutation

Other identifier: IIT2015-18-Mita-MK3475

Population segments: BRCA, Estrogen receptor positive, HER2 negative, HER2 positive, Progesterone receptor positive, Second line, Stage III, Stage IV, Triple receptor negative

Phase: II

Therapies: pembrolizumab, olaparib

Location: United States

US State: CA

Contact: Parisa Mirzadehgan [310-967-4387; Parisa.Mirzadehgan@cshs.org]

NCT04666740

A Phase II Trial to Evaluate the Safety and Antitumor Activity of Pembrolizumab and OLApaRib (POLAR) Maintenance for Patients With Metastatic Pancreatic Ductal Adenocarcinoma and Homologous Recombination Deficiency and/or Exceptional Treatment Response to Platinum-Based Therapy

Cancer type: Pancreatic Cancer

Variant class: BRCA2 aberration

Other identifier: 20-481

Population segments: BRCA, Maintenance/Consolidation, Stage IV

Phase: II

Therapies: pembrolizumab, olaparib

Location: United States

US States: NJ, NY

Contact: Dr. Wungki Park [646-888-4543; yaqubiea@mskcc.org]

NCT02264678

A Modular Phase I, Open-Label, Multicentre Study to Assess the Safety, Tolerability, Pharmacokinetics and Preliminary Anti-tumour Activity of Ceralasertib in Combination With Cytotoxic Chemotherapy and/or DNA Damage Repair/Novel Anti-cancer Agents in Patients With Advanced Solid Malignancies

Cancer type: Breast Cancer, Ovarian Cancer

Variant class: BRCA mutation

Other inclusion criteria: ERBB2 negative

Other identifiers: 0C-14-9, 16-031, 18-342, D5330C00004, EudraCT Number: 2014-002233-66, IRAS ID 160200, NCI-2015-00687, Study 4

Population segments: Adenocarcinoma, BRCA, HER2 negative, Pulmonary, Second line, Stage III, Stage IV, Third line, Triple receptor negative

Exclusion criteria variant class: ERBB2 positive (Breast Cancer)

Phase: I

Therapies: ceralasertib, olaparib

Locations: France, Republic of Korea, United Kingdom, United States

US States: CA, MA, NY

Contact: AstraZeneca Clinical Study Information Center [877-240-9479; information.center@astrazeneca.com]

Disclaimer: The data presented here is from a curated knowledgebase of publicly available information, but may not be exhaustive. The data version is 2021.11(003).

BRCA2 truncating mutation (continued)

NCT04169841
Precision Medicine Phase II Study Evaluating the Efficacy of a Double Immunotherapy by Durvalumab and Tremelimumab Combined With Olaparib in Patients With Solid Cancers and Carriers of Homologous Recombination Repair Genes Mutation in Response or Stable After Olaparib Treatment

Cancer type: Breast Cancer, Endometrial Carcinoma, Head and Neck Cancer, Kidney Cancer, Non-Small Cell Lung Cancer, Ovarian Cancer, Pancreatic Cancer, Prostate Cancer

Variant class: HRR mutation

Other identifier: GUIDE2REPAIR

Population segments: Fourth line or greater, Hormone refractory, Maintenance/ Consolidation, Second line, Stage III, Stage IV, Third line

Exclusion criteria variant classes: ALK fusion (Lung Cancer), BRAF mutation (Lung Cancer), EGFR mutation (Lung Cancer), ROS1 mutation (Lung Cancer)

Phase: II

Therapies: durvalumab, tremelimumab, olaparib

Location: France

NCT04030559
Phase II Trial of PARP Inhibitor Niraparib for Men With High Risk Prostate Cancer and DNA Damage Response Defects

Cancer type: Prostate Cancer

Variant class: HRR mutation

Other identifiers: 1412676, NCI-2019-04365, UCDCC#279

Population segments: First line, Neoadjuvant, Stage I, Stage II, Stage IV

Phase: II

Therapy: niraparib

Location: United States

US State: CA

Contact: Marc Dall'Era [916-734-3771; mdallera@ucdavis.edu]

NCT04940637
A Phase II, Open-Label, Single Arm, Prospective, Multicenter Study of Niraparib Plus Dostarlimab in Patients With Advanced NSCLC and/or MPM, and Positive for PD-L1 Expression and Germline or Somatic Mutations in the HRR Genes

Cancer type: Mesothelioma, Non-Small Cell Lung Cancer

Variant class: HRR mutation

Other inclusion criteria: ALK fusion negative, CD274 expression, EGFRi sensitizing mutation negative, ROS1 fusion negative or CD274 expression

Other identifiers: EudraCT Number: 2020-000109-10, UNITO-001, UNITO-001/2020

Population segments: Second line, Stage III, Stage IV

Exclusion criteria variant classes: ALK fusion (Non-Small Cell Lung Cancer), EGFRi sensitizing mutation (Non-Small Cell Lung Cancer), ROS1 fusion (Non-Small Cell Lung Cancer)

Phase: II

Therapies: niraparib, dostarlimab

Location: Italy

BRCA2 truncating mutation (continued)

NCT04890613
Phase Ib Expansion Study of CX-5461 in Patients With Solid Tumours and BRCA2 and/or PALB2 Mutation

Cancer type: Ovarian Cancer

Variant class: HRR mutation

Other identifier: CX-5461-04

Population segments: BRCA, Second line, Stage II, Stage III, Stage IV

Phase: I

Therapy: pidnarulex

Location: Canada

NCT05002868
A Multi-center, Open-label, Phase I/Ib Study to Assess the Safety, Pharmacokinetics and Anti-tumor Activity of RP12146, a Poly (ADP-ribose) Polymerase (PARP) Inhibitor, in Patients With Locally Advanced or Metastatic Solid Tumors

Cancer type: Biliary Tract Carcinoma, Breast Cancer, Colorectal Cancer, Endometrial Carcinoma, Gastric Cancer, Ovarian Cancer, Pancreatic Cancer, Prostate Cancer, Small Cell Lung Cancer

Variant class: HRR mutation

Other identifier: RP12146-2101

Population segments: Extensive, Pulmonary, Second line, Stage III, Stage IV

Exclusion criteria variant class: ERBB2 positive (Breast Cancer)

Phase: I

Therapy: RP12146

Location: Poland

NCT03395197
A Phase III, Randomized, Double-blind, Placebo-controlled Study Of Talazoparib With Enzalutamide In Metastatic Castration-resistant Prostate Cancer

Cancer type: Castration-Resistant Prostate Cancer

Variant class: DNA repair mutation

Other identifiers: BASIC-ROYAL / HIGH-ROYAL, C3441021, CSET 2938, CTR20191443, CTR20191444, EudraCT Number: 2017-003295-31, GU 170 - C3441021, HCC 19-054, JapicCTI-194665, LH19.022, MOH_2019-05-16_007127, NCI-2018-01651, Pfizer C3441021, R19062M, RECF3951, RWF_C3441021 TALAPRO-2, TALAPRO-2, TX228665, UCI-18-47, VICCURO17115

Population segments: First line, Hormone refractory, Second line, Stage III, Stage IV

Phase: III

Therapies: talazoparib, hormone therapy

Locations: Argentina, Australia, Belgium, Brazil, Canada, Chile, China, Czech Republic, Finland, France, Germany, Hungary, Israel, Italy, Japan, New Zealand, Norway, Peru, Poland, Portugal, Republic of Korea, South Africa, Spain, Sweden, United Kingdom, United States

US States: AZ, CA, CO, FL, GA, IL, IN, KS, MO, NE, NJ, NY, OH, OK, OR, PA, SC, TN, TX, UT, VA, WI

Contact: Pfizer CT.gov Call Center [800-718-1021; ClinicalTrials.gov_Inquiries@pfizer.com]

Disclaimer: The data presented here is from a curated knowledgebase of publicly available information, but may not be exhaustive. The data version is 2021.11(003).

BRCA2 truncating mutation (continued)

NCT04821622

TALAPRO-3: A Phase III, Randomized, Double-blind, Study of Talazoparib With Enzalutamide Versus Placebo With Enzalutamide in Men With DDR Gene Mutated Metastatic Castration-sensitive Prostate Cancer

Cancer type: Prostate Adenocarcinoma

Variant class: DNA repair mutation

Other identifiers: C3441052, CTR20212191, EudraCT Number: 2021-000248-23, jRCT2031210098, TALAPRO-3

Population segments: Second line, Stage IV

Phase: III

Therapies: talazoparib, hormone therapy

Locations: Japan, Republic of Korea, United States

US States: CA, DC, FL, GA, IL, MI, NJ, NY, PA, SC, TX, UT

Contact: Pfizer CT.gov Call Center [800-718-1021; ClinicalTrials.gov_Inquiries@pfizer.com]

NCT04038502

An Open-label, Multicenter Phase II Study to Compare the Efficacy of Carboplatin as First-line Followed by Second-line Olaparib Versus Olaparib as First-line Followed by Second-line Carboplatin in the Treatment of Patients With Castration Resistant Prostate Cancer Containing Homologous Recombination Deficiency

Cancer type: Castration-Resistant Prostate Cancer

Variant class: DNA repair mutation

Other identifiers: 01781, 01783, 19-08, BRACeD, CX001963-01, ONCA-009-18F

Population segments: First line, Hormone refractory, Second line, Stage IV

Phase: II

Therapies: olaparib, chemotherapy

Location: United States

US States: CA, DC, FL, IL, MI, NC, NY, PA, WA

Contact: Dr. Robert B. Montgomery [206-277-6878; rbmontgo@u.washington.edu]

No NCT ID - see other identifier(s)

Single-arm Exploratory Study for Sintilimab Second-line or Later-line Treatment of Advanced Non-small Cell Lung Cancer with DDR Pathway Gene Mutation

Cancer type: Non-Small Cell Lung Cancer

Variant class: DNA repair mutation

Other identifier: ChiCTR1900023234

Population segments: Fourth line or greater, Second line, Stage III, Stage IV, Third line

Exclusion criteria variant classes: ALK fusion (Non-Small Cell Lung Cancer), EGFRi sensitizing mutation (Non-Small Cell Lung Cancer), ROS1 fusion (Non-Small Cell Lung Cancer)

Phase: I/II

Therapy: sintilimab

Location: China

Disclaimer: The data presented here is from a curated knowledgebase of publicly available information, but may not be exhaustive. The data version is 2021.11(003).

BRCA2 truncating mutation (continued)

NCT04095273

A Multicenter, Non-randomized, Open-label Phase Ib Study to Determine the Maximum Tolerated and Recommended Phase II Dose of the ATR Inhibitor BAY1895344 in Combination With Pembrolizumab and to Characterize Its Safety, Tolerability, Pharmacokinetics and Preliminary Anti-tumor Activity in Patients With Advanced Solid Tumors

Cancer type: Breast Cancer, Castration-Resistant Prostate Cancer, Colorectal Cancer, Gastric Cancer, Gastroesophageal Junction Adenocarcinoma, Non-Small Cell Lung Cancer, Pancreatic Cancer

Variant class: DNA repair mutation

Other inclusion criteria: ATM mutation negative or ATM mutation negative, ERBB2 negative, Hormone receptor positive or ATM underexpression

Other identifiers: 19741, EudraCT Number: 2018-003420-36, Keynote 919, NCI-2019-06734

Population segments: HER2 negative, Hormone refractory, Second line, Stage III, Stage IV

Phase: I

Therapies: BAY-1895344, pembrolizumab

Locations: Germany, Spain, Switzerland, United States

US States: CA, CT, MA, MD, NC, NY, OH, TX

Contact: Bayer Clinical Trials Contact [clinical-trials-contact@bayer.com]

NCT04849364

A Phase II Circulating Tumor DNA Enriched, Genomically Directed Post-neoadjuvant Trial for Patients With Residual Triple Negative Breast Cancer (PERSEVERE)

Cancer type: Triple Negative Breast Cancer

Variant class: DNA repair pathway

Other identifiers: HCRN BRE18-334, PERSEVERE

Population segments: Adjuvant, HER2 negative, Stage I, Stage II, Stage III, Triple receptor negative

Phase: II

Therapies: talazoparib, chemotherapy

Location: United States

US State: IN

Contact: Dr. Bryan P. Schneider [317-948-3885; bpschnei@iu.edu]

Disclaimer: The data presented here is from a curated knowledgebase of publicly available information, but may not be exhaustive. The data version is 2021.11(003).

BRCA2 deletion

NCT02286687

Phase II Study of the PARP Inhibitor Talazoparib in Advanced Cancer Patients With Somatic Alterations in BRCA1/2, Mutations/Deletions in Other BRCA Pathway Genes and Germline Mutation in BRCA1/2 (Not Breast or Ovarian Cancer)

Cancer type: Unspecified Cancer

Variant class: BRCA2 deletion

Other identifiers: 2013-0961, NCI-2014-02494

Population segments: Fourth line or greater, Stage III, Stage IV

Exclusion criteria variant classes: BRCA1 germline mutation (Breast Cancer, Ovarian Cancer), BRCA2 germline mutation (Breast Cancer, Ovarian Cancer)

Phase: II

Therapy: talazoparib

Location: United States

US State: TX

Contact: Dr. Sarina Piha-Paul [713-563-1930; spihapau@mdanderson.org]

NCT03297606

Canadian Profiling and Targeted Agent Utilization Trial (CAPTUR): A Phase II Basket Trial

Cancer type: Unspecified Solid Tumor

Variant class: BRCA2 aberration

Other identifiers: CA209-9DL, CAPTUR, ESR-17-12831, ML39800, PM1, WI233446

Population segments: Aggressive, Diffuse large B-cell lymphoma (DLBCL), Extranodal marginal zone B-cell lymphoma (MALT), First line, Follicular lymphoma (FL), Indolent, Lymphoblastic lymphoma (LBL), Mantle cell lymphoma (MCL), Other subtype, Second line, Stage III, Stage IV, Waldenstrom's macroglobulinemia (WM)

Phase: II

Therapy: olaparib

Location: Canada

NCT04497116

Phase I/IIa Study of the Safety, Pharmacokinetics, Pharmacodynamics and Preliminary Clinical Activity of RP-3500 Alone or in Combination With Talazoparib in Advanced Solid Tumors With ATR Inhibitor Sensitizing Mutations (TRESR Study)

Cancer type: Unspecified Solid Tumor

Variant class: BRCA2 aberration

Other identifiers: 20-521, EudraCT Number: 2020-000301-87, IRAS ID: 286609, NCI-2020-06869, RP-3500-01, TRESR, TRESR Study

Population segments: Second line, Stage III, Stage IV

Phase: I/II

Therapies: RP-3500, talazoparib

Locations: Canada, Denmark, United Kingdom, United States

US States: MA, NC, NY, RI, TN, TX

Contact: Dr. Peter Manley [857-322-5553; pmanley@reparerx.com]

Disclaimer: The data presented here is from a curated knowledgebase of publicly available information, but may not be exhaustive. The data version is 2021.11(003).

BRCA2 deletion (continued)

NCT04901702
A Randomized Phase I/II Study of Talazoparib or Temozolomide in Combination With Onivyde in Children With Recurrent Solid Malignancies and Ewing Sarcoma

Cancer type: Unspecified Solid Tumor

Variant class: HRR pathway

Other identifiers: EudraCT Number: 2021-003570-31, ONITT

Population segments: Second line, Stage IV

Exclusion criteria variant classes: HRR germline pathway (Central Nervous System Neoplasm, Ewing Sarcoma), HRR pathway (Central Nervous System Neoplasm, Ewing Sarcoma)

Phase: I/II

Therapies: talazoparib, chemotherapy

Locations: Canada, United States

US State: TN

Contact: Dr. Sara Federico [866-278-5833; referralinfo@stjude.org]

NCT03155620
NCI-COG Pediatric MATCH (Molecular Analysis for Therapy Choice) Screening Protocol

Cancer type: Unspecified Solid Tumor

Variant class: DNA repair pathway

Other identifiers: 17-729, 2017-0625, 20170841, APEC1621SC, COGAPEC1621, COGAPEC1621SC, N 55017 SC, NCI-2017-01251, NCI-COG Pediatric MATCH, Pediatric MATCH

Population segments: ALK, Aggressive, BRAF, FGFR, HRAS, Indolent, KRAS, NRAS, Pediatric or Adolescent, Second line, Stage III, Stage IV

Phase: II

Therapy: olaparib

Locations: Puerto Rico, United States

US States: AK, AL, AR, AZ, CA, CO, CT, DC, DE, FL, GA, HI, IA, ID, IL, IN, KY, LA, MA, MD, ME, MI, MN, MO, MS, NC, NE, NH, NJ, NV, NY, OH, OK, OR, PA, RI, SC, SD, TN, TX, UT, VA, VT, WA, WI

Contact: Multiple contacts: See www.clinicaltrials.gov for complete list of contacts.

NCT03233204
NCI-COG Pediatric MATCH (Molecular Analysis for Therapy Choice)- Phase 2 Subprotocol of Olaparib in Patients With Tumors Harboring Defects in DNA Damage Repair Genes

Cancer type: Unspecified Solid Tumor

Variant class: DNA repair pathway

Other identifiers: 20170841, APEC1621H, NCI-2017-00766

Population segments: Aggressive, Indolent, Pediatric or Adolescent, Second line, Stage III, Stage IV

Phase: II

Therapy: olaparib

Locations: Puerto Rico, United States

US States: AK, AL, AR, AZ, CA, CO, DC, DE, FL, GA, IA, ID, IL, IN, KY, LA, MD, ME, MI, MN, MO, MS, NC, ND, NE, NJ, NY, OH, OK, OR, PA, SC, SD, TN, TX, UT, VA, VT, WA, WI

Contact: Multiple contacts: See www.clinicaltrials.gov for complete list of contacts.

BRCA2 deletion (continued)

NCT04267939
An Open-label Phase Ib Study to Determine the Maximum Tolerated and/or Recommended Phase II Dose of the ATR Inhibitor BAY 1895344 in Combination With PARP Inhibitor Niraparib, in Patients With Recurrent Advanced Solid Tumors and Ovarian Cancer

Cancer type: Ovarian Cancer, Unspecified Solid Tumor

Variant class: DNA repair pathway

Other identifiers: 18595, 19-810, 2019-0471, 20-222, EudraCT Number: 2018-003930-34, NCI-2020-01405

Population segments: Maintenance/Consolidation, Second line, Stage III, Stage IV

Exclusion criteria variant class: DNA repair pathway (Prostate Cancer)

Phase: I

Therapies: BAY-1895344, niraparib

Location: United States

US States: MA, NY, TX

Contact: Bayer Clinical Trials Contact [888-842-2937; clinical-trials-contact@bayer.com]

NCT04693468
Modular Phase IB Hypothesis-Testing, Biomarker-Driven, Talazoparib Combination Trial (TalaCom)

Cancer type: Unspecified Solid Tumor

Variant class: DNA repair pathway

Other identifiers: 2020-0436, NCI-2020-06041, TalaCom

Population segments: Hormone refractory, Second line, Stage III, Stage IV

Phase: I

Therapies: talazoparib, palbociclib, axitinib, crizotinib

Location: United States

US State: TX

Contact: Timothy A. Yap [713-563-1784; tyap@mdanderson.org]

NCT03810105
A Phase II Study of Olaparib and Durvalumab in Men With Castration Sensitive Biochemically Recurrent Non-Metastatic Prostate Cancer Harboring Mutations in DNA Damage Repair

Cancer type: Prostate Cancer

Variant class: BRCA2 deletion

Other identifiers: 18-480, NCI-2019-01627

Population segments: Adjuvant, Stage I, Stage II, Stage III

Phase: II

Therapies: durvalumab, olaparib

Location: United States

US States: CA, IL, MI, NJ, NY

Contact: Dr. Karen Autio [646-422-4632; autiok@mskcc.org]

Disclaimer: The data presented here is from a curated knowledgebase of publicly available information, but may not be exhaustive. The data version is 2021.11(003).

BRCA2 deletion (continued)

NCT03012321

BRCAAway: A Randomized Phase II Trial of Abiraterone, Olaparib, or Abiraterone + Olaparib in Patients With Metastatic Castration-Resistant Prostate Cancer With DNA Repair Defects

Cancer type: Castration-Resistant Prostate Cancer

Variant class: BRCA2 deletion

Other identifiers: 19137, BRCAAway, NCI-2016-01834, NU 16U05, NU_16U05, PCCTC #: c16-168, PCCTC c16-168, STU00203960

Population segments: First line, Hormone refractory, Stage IV

Phase: II

Therapies: hormone therapy, olaparib, steroid

Location: United States

US States: CA, FL, IL, IN, MD, MI, MN, MO, NC, NY, UT, VA

Contact: Study Coordinator [312-695-1301; cancertrials@northwestern.edu]

NCT03840967

A Phase II Study Evaluating Safety and Efficacy of Niraparib in Patients With Previously Treated Homologous Recombination (HR) Defective or Loss of Heterozygosity (LOH) High Metastatic Esophageal/Gastroesophageal Junction/Proximal Gastric Adenocarcinoma

Cancer type: Esophageal Cancer, Gastric Cancer, Gastroesophageal Junction Adenocarcinoma

Variant class: BRCA2 deletion

Other identifiers: 20166, 71618, HCRN ESO17-325, NCI-2019-07395

Population segments: Second line, Stage III, Stage IV

Phase: II

Therapy: niraparib

Location: United States

US States: FL, IN, MI, NY

Contact: Dr. Shadia Jalal [317-274-3658; sjalal@iu.edu]

NCT04633902

Phase II Study of Olaparib in Combination With Pembrolizumab in Patients With Advanced Melanoma With Homologous Recombination (HR) Pathway Gene Mutation

Cancer type: Melanoma

Variant class: BRCA2 deletion

Other identifier: CPMC19-MEL01

Population segments: BRAF, Second line, Stage III, Stage IV

Phase: II

Therapies: olaparib, pembrolizumab

Location: United States

US State: CA

Contact: Dr. Kevin B. Kim [415-885-8600; ClinicalResearch@sutterhealth.org]

Disclaimer: The data presented here is from a curated knowledgebase of publicly available information, but may not be exhaustive. The data version is 2021.11(003).

BRCA2 deletion (continued)

NCT03248570
Phase II Open Label Study of Pembrolizumab in Patients With Metastatic Castration Resistant Prostate Cancer (mCRPC) With or Without DNA Damage Repair Defects

Cancer type: Castration-Resistant Prostate Cancer

Variant class: BRCA2 deletion

Other identifiers: 16557, 17-22165, CC 16557, NCI-2017-02408

Population segments: BRCA, Hormone refractory, Line of therapy N/A, Stage IV

Phase: II

Therapy: pembrolizumab

Location: United States

US State: CA

Contact: Patricia Li [415-476-5975; GUTrials@ucsf.edu]

NCT04740190
Combination Talazoparib - Carboplatin for Recurrent High-grade Glioma With DNA Damage Repair Deficiency (DDRd)

Cancer type: Anaplastic Astrocytoma, Anaplastic Oligoastrocytoma, Anaplastic Oligodendroglioma, Anaplastic Pleomorphic Xanthoastrocytoma, Diffuse Midline Glioma, Glioblastoma

Variant class: BRCA2 deletion

Other identifiers: TAC-GReD, UW-20396

Population segments: (N/A), BRCA, Second line

Phase: II

Therapies: talazoparib, chemotherapy

Location: Hong Kong

NCT04666740
A Phase II Trial to Evaluate the Safety and Antitumor Activity of Pembrolizumab and OLApaRib (POLAR) Maintenance for Patients With Metastatic Pancreatic Ductal Adenocarcinoma and Homologous Recombination Deficiency and/or Exceptional Treatment Response to Platinum-Based Therapy

Cancer type: Pancreatic Cancer

Variant class: BRCA2 aberration

Other identifier: 20-481

Population segments: BRCA, Maintenance/Consolidation, Stage IV

Phase: II

Therapies: pembrolizumab, olaparib

Location: United States

US States: NJ, NY

Contact: Dr. Wungki Park [646-888-4543; yaqubiea@mskcc.org]

NCT04030559
Phase II Trial of PARP Inhibitor Niraparib for Men With High Risk Prostate Cancer and DNA Damage Response Defects

Cancer type: Prostate Cancer

Variant class: HRR deletion

Other identifiers: 1412676, NCI-2019-04365, UCDCC#279

Population segments: First line, Neoadjuvant, Stage I, Stage II, Stage IV

Phase: II

Therapy: niraparib

Location: United States

US State: CA

Contact: Marc Dall'Era [916-734-3771; mdallera@ucdavis.edu]

Disclaimer: The data presented here is from a curated knowledgebase of publicly available information, but may not be exhaustive. The data version is 2021.11(003).

BRCA2 deletion (continued)

NCT03344965

A Phase II Study of Olaparib Monotherapy in Metastatic Breast Cancer Patients With Germline or Somatic Mutations in DNA Repair Genes (Olaparib Expanded)

Cancer type: Breast Cancer

Variant class: DNA repair deletion

Other inclusion criteria: BRCA1 germline mutation negative, BRCA2 germline mutation negative

Other identifiers: 17-428, 18-414, NCI-2018-00778, TBCRC 048, TBCRC048

Population segments: BRCA, Estrogen receptor positive, HER2 negative, HER2 positive, Progesterone receptor positive, Second line, Stage IV

Exclusion criteria variant classes: BRCA1 germline mutation (Breast Cancer), BRCA2 germline mutation (Breast Cancer)

Phase: II

Therapy: olaparib

Location: United States

US States: AL, CA, IL, MA, MD, NC, NJ, NY, PA, WA

Contact: Dr. Nadine Tung [617-667-1962; ntung@bidmc.harvard.edu]

NCT03506997

PERSEUS1: Phase II Trial of the Immune Checkpoint Inhibitor Pembrolizumab for Patients Suffering From Metastatic Prostate Cancer

Cancer type: Castration-Resistant Prostate Cancer

Variant class: DNA repair pathway

Other inclusion criteria: MMR deficient

Other identifiers: CCR4559, EudraCT Number: 2017-000931-15, ICR-CTSU/2016/10060, IRAS ID 208952, PERSEUS1

Population segments: Hormone refractory, Second line, Stage IV

Phase: II

Therapy: pembrolizumab

Location: United Kingdom

NCT04849364

A Phase II Circulating Tumor DNA Enriched, Genomically Directed Post-neoadjuvant Trial for Patients With Residual Triple Negative Breast Cancer (PERSEVERE)

Cancer type: Triple Negative Breast Cancer

Variant class: DNA repair pathway

Other identifiers: HCRN BRE18-334, PERSEVERE

Population segments: Adjuvant, HER2 negative, Stage I, Stage II, Stage III, Triple receptor negative

Phase: II

Therapies: talazoparib, chemotherapy

Location: United States

US State: IN

Contact: Dr. Bryan P. Schneider [317-948-3885; bpschnei@iu.edu]

Disclaimer: The data presented here is from a curated knowledgebase of publicly available information, but may not be exhaustive. The data version is 2021.11(003).

BRCA2 deletion (continued)

NCT05002868
A Multi-center, Open-label, Phase I/Ib Study to Assess the Safety, Pharmacokinetics and Anti-tumor Activity of RP12146, a Poly (ADP-ribose) Polymerase (PARP) Inhibitor, in Patients With Locally Advanced or Metastatic Solid Tumors

Cancer type: Biliary Tract Carcinoma, Breast Cancer, Colorectal Cancer, Endometrial Carcinoma, Gastric Cancer, Ovarian Cancer, Pancreatic Cancer, Prostate Cancer, Small Cell Lung Cancer

Variant class: DNA repair pathway

Other identifier: RP12146-2101

Population segments: Extensive, Pulmonary, Second line, Stage III, Stage IV

Exclusion criteria variant class: ERBB2 positive (Breast Cancer)

Phase: I

Therapy: RP12146

Location: Poland

BRCA2 fusion

NCT03297606
Canadian Profiling and Targeted Agent Utilization Trial (CAPTUR): A Phase II Basket Trial

Cancer type: Unspecified Solid Tumor

Variant class: BRCA2 aberration

Other identifiers: CA209-9DL, CAPTUR, ESR-17-12831, ML39800, PM1, WI233446

Population segments: Aggressive, Diffuse large B-cell lymphoma (DLBCL), Extranodal marginal zone B-cell lymphoma (MALT), First line, Follicular lymphoma (FL), Indolent, Lymphoblastic lymphoma (LBL), Mantle cell lymphoma (MCL), Other subtype, Second line, Stage III, Stage IV, Waldenstrom's macroglobulinemia (WM)

Phase: II

Therapy: olaparib

Location: Canada

NCT04497116
Phase I/IIa Study of the Safety, Pharmacokinetics, Pharmacodynamics and Preliminary Clinical Activity of RP-3500 Alone or in Combination With Talazoparib in Advanced Solid Tumors With ATR Inhibitor Sensitizing Mutations (TRESR Study)

Cancer type: Unspecified Solid Tumor

Variant class: BRCA2 aberration

Other identifiers: 20-521, EudraCT Number: 2020-000301-87, IRAS ID: 286609, NCI-2020-06869, RP-3500-01, TRESR, TRESR Study

Population segments: Second line, Stage III, Stage IV

Phase: I/II

Therapies: RP-3500, talazoparib

Locations: Canada, Denmark, United Kingdom, United States

US States: MA, NC, NY, RI, TN, TX

Contact: Dr. Peter Manley [857-322-5553; pmanley@reparerx.com]

BRCA2 fusion (continued)

NCT02286687
Phase II Study of the PARP Inhibitor Talazoparib in Advanced Cancer Patients With Somatic Alterations in BRCA1/2, Mutations/Deletions in Other BRCA Pathway Genes and Germline Mutation in BRCA1/2 (Not Breast or Ovarian Cancer)

Cancer type: Unspecified Cancer

Variant class: Fanconi anemia pathway

Other identifiers: 2013-0961, NCI-2014-02494

Population segments: Fourth line or greater, Stage III, Stage IV

Exclusion criteria variant classes: BRCA1 germline mutation (Breast Cancer, Ovarian Cancer), BRCA2 germline mutation (Breast Cancer, Ovarian Cancer)

Phase: II

Therapy: talazoparib

Location: United States

US State: TX

Contact: Dr. Sarina Piha-Paul [713-563-1930; spihapau@mdanderson.org]

NCT04901702
A Randomized Phase I/II Study of Talazoparib or Temozolomide in Combination With Onivyde in Children With Recurrent Solid Malignancies and Ewing Sarcoma

Cancer type: Unspecified Solid Tumor

Variant class: HRR pathway

Other identifiers: EudraCT Number: 2021-003570-31, ONITT

Population segments: Second line, Stage IV

Exclusion criteria variant classes: HRR germline pathway (Central Nervous System Neoplasm, Ewing Sarcoma), HRR pathway (Central Nervous System Neoplasm, Ewing Sarcoma)

Phase: I/II

Therapies: talazoparib, chemotherapy

Locations: Canada, United States

US State: TN

Contact: Dr. Sara Federico [866-278-5833; referralinfo@stjude.org]

NCT03155620
NCI-COG Pediatric MATCH (Molecular Analysis for Therapy Choice) Screening Protocol

Cancer type: Unspecified Solid Tumor

Variant class: DNA repair pathway

Other identifiers: 17-729, 2017-0625, 20170841, APEC1621SC, COGAPEC1621, COGAPEC1621SC, N 55017 SC, NCI-2017-01251, NCI-COG Pediatric MATCH, Pediatric MATCH

Population segments: ALK, Aggressive, BRAF, FGFR, HRAS, Indolent, KRAS, NRAS, Pediatric or Adolescent, Second line, Stage III, Stage IV

Phase: II

Therapy: olaparib

Locations: Puerto Rico, United States

US States: AK, AL, AR, AZ, CA, CO, CT, DC, DE, FL, GA, HI, IA, ID, IL, IN, KY, LA, MA, MD, ME, MI, MN, MO, MS, NC, NE, NH, NJ, NV, NY, OH, OK, OR, PA, RI, SC, SD, TN, TX, UT, VA, VT, WA, WI

Contact: Multiple contacts: See www.clinicaltrials.gov for complete list of contacts.

Disclaimer: The data presented here is from a curated knowledgebase of publicly available information, but may not be exhaustive. The data version is 2021.11(003).

BRCA2 fusion (continued)

NCT03233204
NCI-COG Pediatric MATCH (Molecular Analysis for Therapy Choice)- Phase 2 Subprotocol of Olaparib in Patients With Tumors Harboring Defects in DNA Damage Repair Genes

Cancer type: Unspecified Solid Tumor

Variant class: DNA repair pathway

Other identifiers: 20170841, APEC1621H, NCI-2017-00766

Population segments: Aggressive, Indolent, Pediatric or Adolescent, Second line, Stage III, Stage IV

Phase: II

Therapy: olaparib

Locations: Puerto Rico, United States

US States: AK, AL, AR, AZ, CA, CO, DC, DE, FL, GA, IA, ID, IL, IN, KY, LA, MD, ME, MI, MN, MO, MS, NC, ND, NE, NJ, NY, OH, OK, OR, PA, SC, SD, TN, TX, UT, VA, VT, WA, WI

Contact: Multiple contacts: See www.clinicaltrials.gov for complete list of contacts.

NCT04267939
An Open-label Phase Ib Study to Determine the Maximum Tolerated and/or Recommended Phase II Dose of the ATR Inhibitor BAY 1895344 in Combination With PARP Inhibitor Niraparib, in Patients With Recurrent Advanced Solid Tumors and Ovarian Cancer

Cancer type: Ovarian Cancer, Unspecified Solid Tumor

Variant class: DNA repair pathway

Other identifiers: 18595, 19-810, 2019-0471, 20-222, EudraCT Number: 2018-003930-34, NCI-2020-01405

Population segments: Maintenance/Consolidation, Second line, Stage III, Stage IV

Exclusion criteria variant class: DNA repair pathway (Prostate Cancer)

Phase: I

Therapies: BAY-1895344, niraparib

Location: United States

US States: MA, NY, TX

Contact: Bayer Clinical Trials Contact [888-842-2937; clinical-trials-contact@bayer.com]

NCT04693468
Modular Phase IB Hypothesis-Testing, Biomarker-Driven, Talazoparib Combination Trial (TalaCom)

Cancer type: Unspecified Solid Tumor

Variant class: DNA repair pathway

Other identifiers: 2020-0436, NCI-2020-06041, TalaCom

Population segments: Hormone refractory, Second line, Stage III, Stage IV

Phase: I

Therapies: talazoparib, palbociclib, axitinib, crizotinib

Location: United States

US State: TX

Contact: Timothy A. Yap [713-563-1784; tyap@mdanderson.org]

Disclaimer: The data presented here is from a curated knowledgebase of publicly available information, but may not be exhaustive. The data version is 2021.11(003).

BRCA2 fusion (continued)

NCT03840967
A Phase II Study Evaluating Safety and Efficacy of Niraparib in Patients With Previously Treated Homologous Recombination (HR) Defective or Loss of Heterozygosity (LOH) High Metastatic Esophageal/Gastroesophageal Junction/ Proximal Gastric Adenocarcinoma

Cancer type: Esophageal Cancer, Gastric Cancer, Gastroesophageal Junction Adenocarcinoma

Variant class: BRCA2 fusion

Other identifiers: 20166, 71618, HCRN ESO17-325, NCI-2019-07395

Population segments: Second line, Stage III, Stage IV

Phase: II

Therapy: niraparib

Location: United States

US States: FL, IN, MI, NY

Contact: Dr. Shadia Jalal [317-274-3658; sjalal@iu.edu]

NCT02502266
A Randomized Phase II/III Study of the Combination of Cediranib and Olaparib Compared to Cediranib or Olaparib Alone, or Standard of Care Chemotherapy in Women With Recurrent Platinum-Resistant or -Refractory Ovarian, Fallopian Tube, or Primary Peritoneal Cancer (COCOS)

Cancer type: Ovarian Cancer

Variant class: BRCA fusion

Other identifiers: 16-715, COCOS, GY005, GY005-, GYN 9814, jRCT2031190097, KCT0004526, N 33516, NCI-2015-00651, NRG OV1405, NRG-GY005, OVC2, OVM1405, S16-01681

Population segments: (N/A), BRCA, Fourth line or greater, Second line, Third line

Phase: II/III

Therapies: cediranib, olaparib

Locations: Japan, United States

US State: SD

Contact: Multiple contacts: See www.clinicaltrials.gov for complete list of contacts.

NCT04739800
A Randomized Phase II Trial of Triplet Therapy (a PD-L1 Inhibitor (Durvalumab) MEDI4736 in Combination With Olaparib and Cediranib) Compared to Olaparib and Cediranib or (Durvalumab) MEDI4736 and Cediranib or Standard of Care Chemotherapy in Women With Platinum-Resistant Recurrent Epithelial Ovarian Cancer, Primary Peritoneal or Fallopian Cancer Who Have Received Prior Bevacizumab

Cancer type: Ovarian Cancer

Variant class: BRCA fusion

Other identifiers: NCI-2021-00615, NRG-GY023

Population segments: BRCA, Fourth line or greater, Second line, Stage III, Stage IV, Third line

Phase: II

Therapies: durvalumab, olaparib, cediranib

Location: United States

US States: CA, GA, IA, ID, IL, MD, ME, MN, MO, MT, NC, NE, NV, NY, OH, OK, OR, PA, RI, TN, TX, WA, WI, WV

Contact: Multiple contacts: See www.clinicaltrials.gov for complete list of contacts.

Disclaimer: The data presented here is from a curated knowledgebase of publicly available information, but may not be exhaustive. The data version is 2021.11(003).

BRCA2 fusion (continued)

NCT04666740

A Phase II Trial to Evaluate the Safety and Antitumor Activity of Pembrolizumab and OLApaRib (POLAR) Maintenance for Patients With Metastatic Pancreatic Ductal Adenocarcinoma and Homologous Recombination Deficiency and/or Exceptional Treatment Response to Platinum-Based Therapy

Cancer type: Pancreatic Cancer

Variant class: BRCA2 aberration

Other identifier: 20-481

Population segments: BRCA, Maintenance/Consolidation, Stage IV

Phase: II

Therapies: pembrolizumab, olaparib

Location: United States

US States: NJ, NY

Contact: Dr. Wungki Park [646-888-4543; yaqubiea@mskcc.org]

NCT04633902

Phase II Study of Olaparib in Combination With Pembrolizumab in Patients With Advanced Melanoma With Homologous Recombination (HR) Pathway Gene Mutation

Cancer type: Melanoma

Variant class: HRR pathway

Other identifier: CPMC19-MEL01

Population segments: BRAF, Second line, Stage III, Stage IV

Phase: II

Therapies: olaparib, pembrolizumab

Location: United States

US State: CA

Contact: Dr. Kevin B. Kim [415-885-8600; ClinicalResearch@sutterhealth.org]

NCT03506997

PERSEUS1: Phase II Trial of the Immune Checkpoint Inhibitor Pembrolizumab for Patients Suffering From Metastatic Prostate Cancer

Cancer type: Castration-Resistant Prostate Cancer

Variant class: DNA repair pathway

Other inclusion criteria: MMR deficient

Other identifiers: CCR4559, EudraCT Number: 2017-000931-15, ICR-CTSU/2016/10060, IRAS ID 208952, PERSEUS1

Population segments: Hormone refractory, Second line, Stage IV

Phase: II

Therapy: pembrolizumab

Location: United Kingdom

NCT04849364

A Phase II Circulating Tumor DNA Enriched, Genomically Directed Post-neoadjuvant Trial for Patients With Residual Triple Negative Breast Cancer (PERSEVERE)

Cancer type: Triple Negative Breast Cancer

Variant class: DNA repair pathway

Other identifiers: HCRN BRE18-334, PERSEVERE

Population segments: Adjuvant, HER2 negative, Stage I, Stage II, Stage III, Triple receptor negative

Phase: II

Therapies: talazoparib, chemotherapy

Location: United States

US State: IN

Contact: Dr. Bryan P. Schneider [317-948-3885; bpschnei@iu.edu]

Disclaimer: The data presented here is from a curated knowledgebase of publicly available information, but may not be exhaustive. The data version is 2021.11(003).

BRCA2 fusion (continued)

NCT05002868

A Multi-center, Open-label, Phase I/Ib Study to Assess the Safety, Pharmacokinetics and Anti-tumor Activity of RP12146, a Poly (ADP-ribose) Polymerase (PARP) Inhibitor, in Patients With Locally Advanced or Metastatic Solid Tumors

Cancer type: Biliary Tract Carcinoma, Breast Cancer, Colorectal Cancer, Endometrial Carcinoma, Gastric Cancer, Ovarian Cancer, Pancreatic Cancer, Prostate Cancer, Small Cell Lung Cancer

Variant class: DNA repair pathway

Other identifier: RP12146-2101

Population segments: Extensive, Pulmonary, Second line, Stage III, Stage IV

Exclusion criteria variant class: ERBB2 positive (Breast Cancer)

Phase: I

Therapy: RP12146

Location: Poland

BRCA2 aberration

NCT03297606

Canadian Profiling and Targeted Agent Utilization Trial (CAPTUR): A Phase II Basket Trial

Cancer type: Unspecified Solid Tumor

Variant class: BRCA2 aberration

Other identifiers: CA209-9DL, CAPTUR, ESR-17-12831, ML39800, PM1, WI233446

Population segments: Aggressive, Diffuse large B-cell lymphoma (DLBCL), Extranodal marginal zone B-cell lymphoma (MALT), First line, Follicular lymphoma (FL), Indolent, Lymphoblastic lymphoma (LBL), Mantle cell lymphoma (MCL), Other subtype, Second line, Stage III, Stage IV, Waldenstrom's macroglobulinemia (WM)

Phase: II

Therapy: olaparib

Location: Canada

NCT04497116

Phase I/IIa Study of the Safety, Pharmacokinetics, Pharmacodynamics and Preliminary Clinical Activity of RP-3500 Alone or in Combination With Talazoparib in Advanced Solid Tumors With ATR Inhibitor Sensitizing Mutations (TRESR Study)

Cancer type: Unspecified Solid Tumor

Variant class: BRCA2 aberration

Other identifiers: 20-521, EudraCT Number: 2020-000301-87, IRAS ID: 286609, NCI-2020-06869, RP-3500-01, TRESR, TRESR Study

Population segments: Second line, Stage III, Stage IV

Phase: I/II

Therapies: RP-3500, talazoparib

Locations: Canada, Denmark, United Kingdom, United States

US States: MA, NC, NY, RI, TN, TX

Contact: Dr. Peter Manley [857-322-5553; pmanley@reparerx.com]

Disclaimer: The data presented here is from a curated knowledgebase of publicly available information, but may not be exhaustive. The data version is 2021.11(003).

BRCA2 aberration (continued)

NCT02286687
Phase II Study of the PARP Inhibitor Talazoparib in Advanced Cancer Patients With Somatic Alterations in BRCA1/2, Mutations/Deletions in Other BRCA Pathway Genes and Germline Mutation in BRCA1/2 (Not Breast or Ovarian Cancer)

Cancer type: Unspecified Cancer

Variant class: Fanconi anemia pathway

Other identifiers: 2013-0961, NCI-2014-02494

Population segments: Fourth line or greater, Stage III, Stage IV

Exclusion criteria variant classes: BRCA1 germline mutation (Breast Cancer, Ovarian Cancer), BRCA2 germline mutation (Breast Cancer, Ovarian Cancer)

Phase: II

Therapy: talazoparib

Location: United States

US State: TX

Contact: Dr. Sarina Piha-Paul [713-563-1930; spihapau@mdanderson.org]

NCT04901702
A Randomized Phase I/II Study of Talazoparib or Temozolomide in Combination With Onivyde in Children With Recurrent Solid Malignancies and Ewing Sarcoma

Cancer type: Unspecified Solid Tumor

Variant class: HRR pathway

Other identifiers: EudraCT Number: 2021-003570-31, ONITT

Population segments: Second line, Stage IV

Exclusion criteria variant classes: HRR germline pathway (Central Nervous System Neoplasm, Ewing Sarcoma), HRR pathway (Central Nervous System Neoplasm, Ewing Sarcoma)

Phase: I/II

Therapies: talazoparib, chemotherapy

Locations: Canada, United States

US State: TN

Contact: Dr. Sara Federico [866-278-5833; referralinfo@stjude.org]

NCT03155620
NCI-COG Pediatric MATCH (Molecular Analysis for Therapy Choice) Screening Protocol

Cancer type: Unspecified Solid Tumor

Variant class: DNA repair pathway

Other identifiers: 17-729, 2017-0625, 20170841, APEC1621SC, COGAPEC1621, COGAPEC1621SC, N 55017 SC, NCI-2017-01251, NCI-COG Pediatric MATCH, Pediatric MATCH

Population segments: ALK, Aggressive, BRAF, FGFR, HRAS, Indolent, KRAS, NRAS, Pediatric or Adolescent, Second line, Stage III, Stage IV

Phase: II

Therapy: olaparib

Locations: Puerto Rico, United States

US States: AK, AL, AR, AZ, CA, CO, CT, DC, DE, FL, GA, HI, IA, ID, IL, IN, KY, LA, MA, MD, ME, MI, MN, MO, MS, NC, NE, NH, NJ, NV, NY, OH, OK, OR, PA, RI, SC, SD, TN, TX, UT, VA, VT, WA, WI

Contact: Multiple contacts: See www.clinicaltrials.gov for complete list of contacts.

Disclaimer: The data presented here is from a curated knowledgebase of publicly available information, but may not be exhaustive. The data version is 2021.11(003).

BRCA2 aberration (continued)

NCT03233204
NCI-COG Pediatric MATCH (Molecular Analysis for Therapy Choice)- Phase 2 Subprotocol of Olaparib in Patients With Tumors Harboring Defects in DNA Damage Repair Genes

Cancer type: Unspecified Solid Tumor

Variant class: DNA repair pathway

Other identifiers: 20170841, APEC1621H, NCI-2017-00766

Population segments: Aggressive, Indolent, Pediatric or Adolescent, Second line, Stage III, Stage IV

Phase: II

Therapy: olaparib

Locations: Puerto Rico, United States

US States: AK, AL, AR, AZ, CA, CO, DC, DE, FL, GA, IA, ID, IL, IN, KY, LA, MD, ME, MI, MN, MO, MS, NC, ND, NE, NJ, NY, OH, OK, OR, PA, SC, SD, TN, TX, UT, VA, VT, WA, WI

Contact: Multiple contacts: See www.clinicaltrials.gov for complete list of contacts.

NCT04267939
An Open-label Phase Ib Study to Determine the Maximum Tolerated and/or Recommended Phase II Dose of the ATR Inhibitor BAY 1895344 in Combination With PARP Inhibitor Niraparib, in Patients With Recurrent Advanced Solid Tumors and Ovarian Cancer

Cancer type: Ovarian Cancer, Unspecified Solid Tumor

Variant class: DNA repair pathway

Other identifiers: 18595, 19-810, 2019-0471, 20-222, EudraCT Number: 2018-003930-34, NCI-2020-01405

Population segments: Maintenance/Consolidation, Second line, Stage III, Stage IV

Exclusion criteria variant class: DNA repair pathway (Prostate Cancer)

Phase: I

Therapies: BAY-1895344, niraparib

Location: United States

US States: MA, NY, TX

Contact: Bayer Clinical Trials Contact [888-842-2937; clinical-trials-contact@bayer.com]

NCT04693468
Modular Phase IB Hypothesis-Testing, Biomarker-Driven, Talazoparib Combination Trial (TalaCom)

Cancer type: Unspecified Solid Tumor

Variant class: DNA repair pathway

Other identifiers: 2020-0436, NCI-2020-06041, TalaCom

Population segments: Hormone refractory, Second line, Stage III, Stage IV

Phase: I

Therapies: talazoparib, palbociclib, axitinib, crizotinib

Location: United States

US State: TX

Contact: Timothy A. Yap [713-563-1784; tyap@mdanderson.org]

Disclaimer: The data presented here is from a curated knowledgebase of publicly available information, but may not be exhaustive. The data version is 2021.11(003).

BRCA2 aberration (continued)

NCT04666740
A Phase II Trial to Evaluate the Safety and Antitumor Activity of Pembrolizumab and OLApaRib (POLAR) Maintenance for Patients With Metastatic Pancreatic Ductal Adenocarcinoma and Homologous Recombination Deficiency and/or Exceptional Treatment Response to Platinum-Based Therapy

Cancer type: Pancreatic Cancer

Variant class: BRCA2 aberration

Other identifier: 20-481

Population segments: BRCA, Maintenance/Consolidation, Stage IV

Phase: II

Therapies: pembrolizumab, olaparib

Location: United States

US States: NJ, NY

Contact: Dr. Wungki Park [646-888-4543; yaqubiea@mskcc.org]

NCT04633902
Phase II Study of Olaparib in Combination With Pembrolizumab in Patients With Advanced Melanoma With Homologous Recombination (HR) Pathway Gene Mutation

Cancer type: Melanoma

Variant class: HRR pathway

Other identifier: CPMC19-MEL01

Population segments: BRAF, Second line, Stage III, Stage IV

Phase: II

Therapies: olaparib, pembrolizumab

Location: United States

US State: CA

Contact: Dr. Kevin B. Kim [415-885-8600; ClinicalResearch@sutterhealth.org]

NCT03506997
PERSEUS1: Phase II Trial of the Immune Checkpoint Inhibitor Pembrolizumab for Patients Suffering From Metastatic Prostate Cancer

Cancer type: Castration-Resistant Prostate Cancer

Variant class: DNA repair pathway

Other inclusion criteria: MMR deficient

Other identifiers: CCR4559, EudraCT Number: 2017-000931-15, ICR-CTSU/2016/10060, IRAS ID 208952, PERSEUS1

Population segments: Hormone refractory, Second line, Stage IV

Phase: II

Therapy: pembrolizumab

Location: United Kingdom

NCT04849364
A Phase II Circulating Tumor DNA Enriched, Genomically Directed Post-neoadjuvant Trial for Patients With Residual Triple Negative Breast Cancer (PERSEVERE)

Cancer type: Triple Negative Breast Cancer

Variant class: DNA repair pathway

Other identifiers: HCRN BRE18-334, PERSEVERE

Population segments: Adjuvant, HER2 negative, Stage I, Stage II, Stage III, Triple receptor negative

Phase: II

Therapies: talazoparib, chemotherapy

Location: United States

US State: IN

Contact: Dr. Bryan P. Schneider [317-948-3885; bpschnei@iu.edu]

Disclaimer: The data presented here is from a curated knowledgebase of publicly available information, but may not be exhaustive. The data version is 2021.11(003).

BRCA2 aberration (continued)

NCT05002868
A Multi-center, Open-label, Phase I/Ib Study to Assess the Safety, Pharmacokinetics and Anti-tumor Activity of RP12146, a Poly (ADP-ribose) Polymerase (PARP) Inhibitor, in Patients With Locally Advanced or Metastatic Solid Tumors

Cancer type: Biliary Tract Carcinoma, Breast Cancer, Colorectal Cancer, Endometrial Carcinoma, Gastric Cancer, Ovarian Cancer, Pancreatic Cancer, Prostate Cancer, Small Cell Lung Cancer

Variant class: DNA repair pathway

Other identifier: RP12146-2101

Population segments: Extensive, Pulmonary, Second line, Stage III, Stage IV

Exclusion criteria variant class: ERBB2 positive (Breast Cancer)

Phase: I

Therapy: RP12146

Location: Poland

Relevant Biomarkers

Genomic Alteration	Relevant Therapies (In this cancer type)	Relevant Therapies (In other cancer type)	Clinical Trials
BRCA2 mutation<br>**Prognostic significance:** None<br>**Diagnostic significance:** None	None	**bevacizumab + olaparib** [1, 2]<br>**niraparib** [1]<br>**olaparib** [1, 2]<br>**rucaparib** [1, 2]	78
BRCA2 splice site mutation<br>**Prognostic significance:** None<br>**Diagnostic significance:** None	None	**bevacizumab + olaparib** [1, 2]<br>**niraparib** [1]<br>**olaparib** [1, 2]<br>**rucaparib** [1, 2]	78
BRCA2 truncating mutation<br>**Prognostic significance:** None<br>**Diagnostic significance:** None	None	**bevacizumab + olaparib** [1, 2]<br>**niraparib** [1]<br>**olaparib** [1, 2]<br>**rucaparib** [1, 2]	78
BRCA2 deletion<br>**Prognostic significance:** None<br>**Diagnostic significance:** None	None	None	21
BRCA2 fusion<br>**Prognostic significance:** None<br>**Diagnostic significance:** None	None	None	16
BRCA2 aberration<br>**Prognostic significance:** None<br>**Diagnostic significance:** None	None	None	13

Public data sources included in relevant therapies: FDA1, NCCN, **EMA**2, ESMO
Public data sources included in prognostic and diagnostic significance: NCCN, ESMO

Relevant Therapy Summary

● In this cancer type ○ In other cancer type ◐ In this cancer type and other cancer types ✖ No evidence

BRCA2 mutation

Relevant Therapy	FDA	NCCN	EMA	ESMO	Clinical Trials*
olaparib	○	○	○	○	◐ (II)
rucaparib	○	○	○	○	● (II)
bevacizumab + olaparib	○	○	○	○	✖
niraparib	○	○	✖	○	○ (III)
olaparib, pembrolizumab	✖	✖	✖	✖	◐ (II)
talazoparib	✖	✖	✖	✖	◐ (II)
talazoparib, chemotherapy	✖	✖	✖	✖	◐ (II)
atezolizumab	✖	✖	✖	✖	● (II)
olaparib, talazoparib, atezolizumab + talazoparib	✖	✖	✖	✖	● (II)
ATRN-119	✖	✖	✖	✖	● (I/II)
RP-3500, talazoparib	✖	✖	✖	✖	● (I/II)
BAY-1895344, niraparib	✖	✖	✖	✖	● (I)
copanlisib, olaparib, durvalumab	✖	✖	✖	✖	● (I)
HH-CYH33, olaparib	✖	✖	✖	✖	● (I)
HWH-340	✖	✖	✖	✖	● (I)
olaparib, adavosertib	✖	✖	✖	✖	● (I)
talazoparib, palbociclib, axitinib, crizotinib	✖	✖	✖	✖	● (I)
atezolizumab, chemotherapy, niraparib	✖	✖	✖	✖	○ (III)
cediranib, olaparib	✖	✖	✖	✖	○ (III)
rucaparib, hormone therapy	✖	✖	✖	✖	○ (III)
talazoparib, hormone therapy	✖	✖	✖	✖	○ (III)
ceralasertib, olaparib	✖	✖	✖	✖	○ (II)
durvalumab, olaparib	✖	✖	✖	✖	○ (II)
durvalumab, olaparib, hormone therapy	✖	✖	✖	✖	○ (II)
durvalumab, tremelimumab, olaparib	✖	✖	✖	✖	○ (II)
hormone therapy, olaparib, steroid	✖	✖	✖	✖	○ (II)
niraparib, dostarlimab	✖	✖	✖	✖	○ (II)
nivolumab, chemotherapy, hormone therapy	✖	✖	✖	✖	○ (II)
nivolumab, ipilimumab	✖	✖	✖	✖	○ (II)

* Most advanced phase (IV, III, II/III, II, I/II, I) is shown and multiple clinical trials may be available.

Disclaimer: The data presented here is from a curated knowledgebase of publicly available information, but may not be exhaustive. The data version is 2021.11(003).

Relevant Therapy Summary (continued)

● In this cancer type ○ In other cancer type ◐ In this cancer type and other cancer types ✖ No evidence

BRCA2 mutation (continued)

Relevant Therapy	FDA	NCCN	EMA	ESMO	Clinical Trials*
nivolumab, talazoparib	✖	✖	✖	✖	○ (II)
olaparib, atezolizumab	✖	✖	✖	✖	○ (II)
olaparib, bevacizumab	✖	✖	✖	✖	○ (II)
olaparib, chemotherapy	✖	✖	✖	✖	○ (II)
pembrolizumab	✖	✖	✖	✖	○ (II)
pembrolizumab, olaparib	✖	✖	✖	✖	○ (II)
senaparib	✖	✖	✖	✖	○ (II)
copanlisib, rucaparib	✖	✖	✖	✖	○ (I/II)
olaparib, palbociclib, hormone therapy	✖	✖	✖	✖	○ (I/II)
rucaparib, nivolumab, ipilimumab	✖	✖	✖	✖	○ (I/II)
sintilimab	✖	✖	✖	✖	○ (I/II)
talazoparib, avelumab	✖	✖	✖	✖	○ (I/II)
BAY-1895344, pembrolizumab	✖	✖	✖	✖	○ (I)
pidnarulex	✖	✖	✖	✖	○ (I)
RP12146	✖	✖	✖	✖	○ (I)

BRCA2 splice site mutation

Relevant Therapy	FDA	NCCN	EMA	ESMO	Clinical Trials*
olaparib	○	○	○	○	◐ (II)
rucaparib	○	○	○	○	● (II)
bevacizumab + olaparib	○	○	○	○	✖
niraparib	○	○	✖	○	○ (III)
olaparib, pembrolizumab	✖	✖	✖	✖	◐ (II)
talazoparib	✖	✖	✖	✖	◐ (II)
talazoparib, chemotherapy	✖	✖	✖	✖	◐ (II)
atezolizumab	✖	✖	✖	✖	● (II)
olaparib, talazoparib, atezolizumab + talazoparib	✖	✖	✖	✖	● (II)
ATRN-119	✖	✖	✖	✖	● (I/II)
RP-3500, talazoparib	✖	✖	✖	✖	● (I/II)

* Most advanced phase (IV, III, II/III, II, I/II, I) is shown and multiple clinical trials may be available.

Disclaimer: The data presented here is from a curated knowledgebase of publicly available information, but may not be exhaustive. The data version is 2021.11(003).

Relevant Therapy Summary (continued)

● In this cancer type ○ In other cancer type ◐ In this cancer type and other cancer types ✖ No evidence

BRCA2 splice site mutation (continued)

Relevant Therapy	FDA	NCCN	EMA	ESMO	Clinical Trials*
BAY-1895344, niraparib	✖	✖	✖	✖	● (I)
copanlisib, olaparib, durvalumab	✖	✖	✖	✖	● (I)
HH-CYH33, olaparib	✖	✖	✖	✖	● (I)
HWH-340	✖	✖	✖	✖	● (I)
olaparib, adavosertib	✖	✖	✖	✖	● (I)
talazoparib, palbociclib, axitinib, crizotinib	✖	✖	✖	✖	● (I)
atezolizumab, chemotherapy, niraparib	✖	✖	✖	✖	○ (III)
cediranib, olaparib	✖	✖	✖	✖	○ (III)
rucaparib, hormone therapy	✖	✖	✖	✖	○ (III)
talazoparib, hormone therapy	✖	✖	✖	✖	○ (III)
ceralasertib, olaparib	✖	✖	✖	✖	○ (II)
durvalumab, olaparib	✖	✖	✖	✖	○ (II)
durvalumab, olaparib, hormone therapy	✖	✖	✖	✖	○ (II)
durvalumab, tremelimumab, olaparib	✖	✖	✖	✖	○ (II)
hormone therapy, olaparib, steroid	✖	✖	✖	✖	○ (II)
niraparib, dostarlimab	✖	✖	✖	✖	○ (II)
nivolumab, chemotherapy, hormone therapy	✖	✖	✖	✖	○ (II)
nivolumab, ipilimumab	✖	✖	✖	✖	○ (II)
nivolumab, talazoparib	✖	✖	✖	✖	○ (II)
olaparib, atezolizumab	✖	✖	✖	✖	○ (II)
olaparib, bevacizumab	✖	✖	✖	✖	○ (II)
olaparib, chemotherapy	✖	✖	✖	✖	○ (II)
pembrolizumab	✖	✖	✖	✖	○ (II)
pembrolizumab, olaparib	✖	✖	✖	✖	○ (II)
senaparib	✖	✖	✖	✖	○ (II)
copanlisib, rucaparib	✖	✖	✖	✖	○ (I/II)
olaparib, palbociclib, hormone therapy	✖	✖	✖	✖	○ (I/II)
rucaparib, nivolumab, ipilimumab	✖	✖	✖	✖	○ (I/II)
sintilimab	✖	✖	✖	✖	○ (I/II)

* Most advanced phase (IV, III, II/III, II, I/II, I) is shown and multiple clinical trials may be available.

Disclaimer: The data presented here is from a curated knowledgebase of publicly available information, but may not be exhaustive. The data version is 2021.11(003).

Relevant Therapy Summary (continued)

● In this cancer type ○ In other cancer type ◐ In this cancer type and other cancer types ✖ No evidence

BRCA2 splice site mutation (continued)

Relevant Therapy	FDA	NCCN	EMA	ESMO	Clinical Trials*
talazoparib, avelumab	✖	✖	✖	✖	○ (I/II)
BAY-1895344, pembrolizumab	✖	✖	✖	✖	○ (I)
pidnarulex	✖	✖	✖	✖	○ (I)
RP12146	✖	✖	✖	✖	○ (I)

BRCA2 truncating mutation

Relevant Therapy	FDA	NCCN	EMA	ESMO	Clinical Trials*
olaparib	○	○	○	○	◐ (II)
rucaparib	○	○	○	○	● (II)
bevacizumab + olaparib	○	○	○	○	✖
niraparib	○	○	✖	○	○ (III)
olaparib, pembrolizumab	✖	✖	✖	✖	◐ (II)
talazoparib	✖	✖	✖	✖	◐ (II)
talazoparib, chemotherapy	✖	✖	✖	✖	◐ (II)
atezolizumab	✖	✖	✖	✖	● (II)
olaparib, talazoparib, atezolizumab + talazoparib	✖	✖	✖	✖	● (II)
ATRN-119	✖	✖	✖	✖	● (I/II)
RP-3500, talazoparib	✖	✖	✖	✖	● (I/II)
BAY-1895344, niraparib	✖	✖	✖	✖	● (I)
copanlisib, olaparib, durvalumab	✖	✖	✖	✖	● (I)
HH-CYH33, olaparib	✖	✖	✖	✖	● (I)
HWH-340	✖	✖	✖	✖	● (I)
olaparib, adavosertib	✖	✖	✖	✖	● (I)
talazoparib, palbociclib, axitinib, crizotinib	✖	✖	✖	✖	● (I)
atezolizumab, chemotherapy, niraparib	✖	✖	✖	✖	○ (III)
cediranib, olaparib	✖	✖	✖	✖	○ (III)
rucaparib, hormone therapy	✖	✖	✖	✖	○ (III)
talazoparib, hormone therapy	✖	✖	✖	✖	○ (III)
ceralasertib, olaparib	✖	✖	✖	✖	○ (II)

* Most advanced phase (IV, III, II/III, II, I/II, I) is shown and multiple clinical trials may be available.

Relevant Therapy Summary (continued)

● In this cancer type ○ In other cancer type ◐ In this cancer type and other cancer types ✖ No evidence

BRCA2 truncating mutation (continued)

Relevant Therapy	FDA	NCCN	EMA	ESMO	Clinical Trials*
durvalumab, olaparib	✖	✖	✖	✖	○ (II)
durvalumab, olaparib, hormone therapy	✖	✖	✖	✖	○ (II)
durvalumab, tremelimumab, olaparib	✖	✖	✖	✖	○ (II)
hormone therapy, olaparib, steroid	✖	✖	✖	✖	○ (II)
niraparib, dostarlimab	✖	✖	✖	✖	○ (II)
nivolumab, chemotherapy, hormone therapy	✖	✖	✖	✖	○ (II)
nivolumab, ipilimumab	✖	✖	✖	✖	○ (II)
nivolumab, talazoparib	✖	✖	✖	✖	○ (II)
olaparib, atezolizumab	✖	✖	✖	✖	○ (II)
olaparib, bevacizumab	✖	✖	✖	✖	○ (II)
olaparib, chemotherapy	✖	✖	✖	✖	○ (II)
pembrolizumab	✖	✖	✖	✖	○ (II)
pembrolizumab, olaparib	✖	✖	✖	✖	○ (II)
senaparib	✖	✖	✖	✖	○ (II)
copanlisib, rucaparib	✖	✖	✖	✖	○ (I/II)
olaparib, palbociclib, hormone therapy	✖	✖	✖	✖	○ (I/II)
rucaparib, nivolumab, ipilimumab	✖	✖	✖	✖	○ (I/II)
sintilimab	✖	✖	✖	✖	○ (I/II)
talazoparib, avelumab	✖	✖	✖	✖	○ (I/II)
BAY-1895344, pembrolizumab	✖	✖	✖	✖	○ (I)
pidnarulex	✖	✖	✖	✖	○ (I)
RP12146	✖	✖	✖	✖	○ (I)

BRCA2 deletion

Relevant Therapy	FDA	NCCN	EMA	ESMO	Clinical Trials*
olaparib	✖	✖	✖	✖	◐ (II)
talazoparib, chemotherapy	✖	✖	✖	✖	◐ (II)
talazoparib	✖	✖	✖	✖	● (II)
RP-3500, talazoparib	✖	✖	✖	✖	● (I/II)

* Most advanced phase (IV, III, II/III, II, I/II, I) is shown and multiple clinical trials may be available.

Disclaimer: The data presented here is from a curated knowledgebase of publicly available information, but may not be exhaustive. The data version is 2021.11(003).

Relevant Therapy Summary (continued)

● In this cancer type ○ In other cancer type ◐ In this cancer type and other cancer types ✖ No evidence

BRCA2 deletion (continued)

Relevant Therapy	FDA	NCCN	EMA	ESMO	Clinical Trials*
BAY-1895344, niraparib	✖	✖	✖	✖	● (I)
talazoparib, palbociclib, axitinib, crizotinib	✖	✖	✖	✖	● (I)
durvalumab, olaparib	✖	✖	✖	✖	○ (II)
hormone therapy, olaparib, steroid	✖	✖	✖	✖	○ (II)
niraparib	✖	✖	✖	✖	○ (II)
nivolumab, chemotherapy, hormone therapy	✖	✖	✖	✖	○ (II)
olaparib, pembrolizumab	✖	✖	✖	✖	○ (II)
pembrolizumab	✖	✖	✖	✖	○ (II)
pembrolizumab, olaparib	✖	✖	✖	✖	○ (II)
RP12146	✖	✖	✖	✖	○ (I)

BRCA2 fusion

Relevant Therapy	FDA	NCCN	EMA	ESMO	Clinical Trials*
talazoparib, chemotherapy	✖	✖	✖	✖	◐ (II)
olaparib	✖	✖	✖	✖	● (II)
talazoparib	✖	✖	✖	✖	● (II)
RP-3500, talazoparib	✖	✖	✖	✖	● (I/II)
BAY-1895344, niraparib	✖	✖	✖	✖	● (I)
talazoparib, palbociclib, axitinib, crizotinib	✖	✖	✖	✖	● (I)
cediranib, olaparib	✖	✖	✖	✖	○ (II/III)
durvalumab, olaparib, cediranib	✖	✖	✖	✖	○ (II)
niraparib	✖	✖	✖	✖	○ (II)
olaparib, pembrolizumab	✖	✖	✖	✖	○ (II)
pembrolizumab	✖	✖	✖	✖	○ (II)
pembrolizumab, olaparib	✖	✖	✖	✖	○ (II)
RP12146	✖	✖	✖	✖	○ (I)

* Most advanced phase (IV, III, II/III, II, I/II, I) is shown and multiple clinical trials may be available.

Disclaimer: The data presented here is from a curated knowledgebase of publicly available information, but may not be exhaustive. The data version is 2021.11(003).

Relevant Therapy Summary (continued)

● In this cancer type ○ In other cancer type ◐ In this cancer type and other cancer types ✖ No evidence

BRCA2 aberration

Relevant Therapy	FDA	NCCN	EMA	ESMO	Clinical Trials*
talazoparib, chemotherapy	✖	✖	✖	✖	◐ (II)
olaparib	✖	✖	✖	✖	● (II)
talazoparib	✖	✖	✖	✖	● (II)
RP-3500, talazoparib	✖	✖	✖	✖	● (I/II)
BAY-1895344, niraparib	✖	✖	✖	✖	● (I)
talazoparib, palbociclib, axitinib, crizotinib	✖	✖	✖	✖	● (I)
olaparib, pembrolizumab	✖	✖	✖	✖	○ (II)
pembrolizumab	✖	✖	✖	✖	○ (II)
pembrolizumab, olaparib	✖	✖	✖	✖	○ (II)
RP12146	✖	✖	✖	✖	○ (I)

* Most advanced phase (IV, III, II/III, II, I/II, I) is shown and multiple clinical trials may be available.

Relevant Biomarkers

Genomic Alteration	Relevant Therapies (In this cancer type)	Relevant Therapies (In other cancer type)	Clinical Trials
BRCA2 mutation<br>**Prognostic significance:** None<br>**Diagnostic significance:** None	None	**bevacizumab + olaparib** [1,2]<br>**niraparib** [1]<br>**olaparib** [1,2]<br>**rucaparib** [1,2]	78
BRCA2 splice site mutation<br>**Prognostic significance:** None<br>**Diagnostic significance:** None	None	**bevacizumab + olaparib** [1,2]<br>**niraparib** [1]<br>**olaparib** [1,2]<br>**rucaparib** [1,2]	78
BRCA2 truncating mutation<br>**Prognostic significance:** None<br>**Diagnostic significance:** None	None	**bevacizumab + olaparib** [1,2]<br>**niraparib** [1]<br>**olaparib** [1,2]<br>**rucaparib** [1,2]	78
BRCA2 deletion<br>**Prognostic significance:** None<br>**Diagnostic significance:** None	None	None	21

Public data sources included in relevant therapies: FDA1, NCCN, **EMA**2, ESMO
Public data sources included in prognostic and diagnostic significance: NCCN, ESMO

Disclaimer: The data presented here is from a curated knowledgebase of publicly available information, but may not be exhaustive. The data version is 2021.11(003).

Relevant Biomarkers (continued)

Genomic Alteration	Relevant Therapies (In this cancer type)	Relevant Therapies (In other cancer type)	Clinical Trials
BRCA2 fusion<br>**Prognostic significance:** None<br>**Diagnostic significance:** None	None	None	16
BRCA2 aberration<br>**Prognostic significance:** None<br>**Diagnostic significance:** None	None	None	13

Public data sources included in relevant therapies: FDA1, NCCN, **EMA**2, ESMO
Public data sources included in prognostic and diagnostic significance: NCCN, ESMO

Clinical Trials Summary

BRCA2 mutation + BRCA2 splice site mutation + BRCA2 truncating mutation + BRCA2 deletion

NCT ID	Title	Phase
NCT04126070	A Phase II Multicohort Study of Nivolumab in Combination With Docetaxel and Androgen Deprivation Therapy in Metastatic Hormone Sensitive Prostate Cancer Patients With DNA Damage Repair Defects or Inflamed Tumors	II

BRCA2 mutation

NCT ID	Title	Phase
NCT02029001	A Two-period, Multicenter, Randomized, Open-label, Phase II Study Evaluating the Clinical Benefit of a Maintenance Treatment Targeting Tumor Molecular Alterations in Patients with Progressive Locally-advanced or Metastatic Solid Tumors.	II
NCT03967938	Efficacy of Olaparib in Advanced Cancers Occurring in Patients With Germline Mutations or Somatic Tumor Mutations in Homologous Recombination Genes	II
NCT02693535	Targeted Agent and Profiling Utilization Registry (TAPUR) Study.	II
NCT04171700	A Phase II Multicenter, Open-label Study of Rucaparib as Treatment for Solid Tumors Associated With Deleterious Mutations in Homologous Recombination Repair Genes	II
NCT02286687	Phase II Study of the PARP Inhibitor Talazoparib in Advanced Cancer Patients With Somatic Alterations in BRCA1/2, Mutations/Deletions in Other BRCA Pathway Genes and Germline Mutation in BRCA1/2 (Not Breast or Ovarian Cancer)	II
NCT04550494	A Pharmacodynamics-Driven Trial of Talazoparib, an Oral PARP Inhibitor, in Patients With Advanced Solid Tumors and Aberrations in Genes Involved in DNA Damage Response	II
NCT03842228	A Phase Ib Biomarker-Driven Combination Trial of Copanlisib, Olaparib, and Durvalumab (MEDI4736) in Patients With Advanced Solid Tumors	I
NCT04586335	Open Label, Phase Ib Study to Evaluate the Safety, Tolerability, Pharmacokinetics and Clinical Activity of CYH33, an Oral a-specific PI3K Inhibitor in Combination With Olaparib, an Oral PARP Inhibitor in Patients With Advanced Solid Tumors.	I
NCT03415659	A Phase I, Open-label, Single-center, Single/Multiple-dose, Dose-escalation/Dose-expansion Clinical Study on Tolerance and Pharmacokinetics of HWH340 Tablet in Patients With Advanced Solid Tumors	I
NCT04197713	Phase I Sequential Trial of Agents Against DNA Repair (STAR)	I
NCT03297606	Canadian Profiling and Targeted Agent Utilization Trial (CAPTUR): A Phase II Basket Trial	II

Disclaimer: The data presented here is from a curated knowledgebase of publicly available information, but may not be exhaustive. The data version is 2021.11(003).

Clinical Trials Summary (continued)

BRCA2 mutation (continued)

NCT ID	Title	Phase
NCT04497116	Phase I/IIa Study of the Safety, Pharmacokinetics, Pharmacodynamics and Preliminary Clinical Activity of RP-3500 Alone or in Combination With Talazoparib in Advanced Solid Tumors With ATR Inhibitor Sensitizing Mutations (TRESR Study)	I/II
NCT04123366	A Phase II Study of Olaparib in Combination With Pembrolizumab in Participants With Previously Treated, Homologous Recombination Repair Mutation (HRRm) and/or Homologous Recombination Deficiency (HRD)-Positive Advanced Cancer	II
NCT03767075	Basket of Baskets: A Modular, Open-label, Phase II, Multicentre Study To Evaluate Targeted Agents in Molecularly Selected Populations With Advanced Solid Tumours	II
NCT04905914	A Phase I/IIa, Open-Label, Safety, Pharmacokinetic, And Preliminary Efficacy Study Of Oral ATRN-119 In Patients With Advanced Solid Tumors	I/II
NCT04901702	A Randomized Phase I/II Study of Talazoparib or Temozolomide in Combination With Onivyde in Children With Recurrent Solid Malignancies and Ewing Sarcoma	I/II
NCT03155620	NCI-COG Pediatric MATCH (Molecular Analysis for Therapy Choice) Screening Protocol	II
NCT03233204	NCI-COG Pediatric MATCH (Molecular Analysis for Therapy Choice)- Phase 2 Subprotocol of Olaparib in Patients With Tumors Harboring Defects in DNA Damage Repair Genes	II
NCT04267939	An Open-label Phase Ib Study to Determine the Maximum Tolerated and/or Recommended Phase II Dose of the ATR Inhibitor BAY 1895344 in Combination With PARP Inhibitor Niraparib, in Patients With Recurrent Advanced Solid Tumors and Ovarian Cancer	I
NCT04693468	Modular Phase IB Hypothesis-Testing, Biomarker-Driven, Talazoparib Combination Trial (TalaCom)	I
NCT02975934	TRITON3: A Multicenter, Randomized, Open Label Phase III Study of Rucaparib Versus Physician's Choice of Therapy for Patients With Metastatic Castration Resistant Prostate Cancer Associated With Homologous Recombination Deficiency	III
NCT03810105	A Phase II Study of Olaparib and Durvalumab in Men With Castration Sensitive Biochemically Recurrent Non-Metastatic Prostate Cancer Harboring Mutations in DNA Damage Repair	II
NCT04336943	Durvalumab (MEDI4736) and Olaparib (AZD2281) for Treatment of Biochemically Recurrent Prostate Cancer in Men Predicted to Have a High Neoantigen Load: A Pilot Study	II
NCT04053322	An International Multicenter Phase II Trial of Durvalumab (MEDI4736) Plus OLAparib Plus Fulvestrant in Metastatic or Locally Advanced ER-positive, HER2-negative Breast Cancer Patients Selected Using Criteria That Predict Sensitivity to Olaparib	II
NCT03012321	BRCAAway: A Randomized Phase II Trial of Abiraterone, Olaparib, or Abiraterone + Olaparib in Patients With Metastatic Castration-Resistant Prostate Cancer With DNA Repair Defects	II
No NCT ID	Phase II Study To Evaluate The Efficacy And Safety Of Niraparib In Recurrent Or Persistent Rare Gynecologic Malignancies With Homologous Recombination Deficiency (JGOG2052)	II
NCT03553004	Niraparib in Metastatic Pancreatic Cancer After Previous Chemotherapy (NIRA-PANC): A Phase II Trial	II
NCT03601923	Phase II Proof-of-Concept Trial Testing the PARP Inhibitor Niraparib in Patients With Pancreatic Cancer Harboring Deficiencies in Homologous Recombination DNA Repair	II
NCT03840967	A Phase II Study Evaluating Safety and Efficacy of Niraparib in Patients With Previously Treated Homologous Recombination (HR) Defective or Loss of Heterozygosity (LOH) High Metastatic Esophageal/Gastroesophageal Junction/Proximal Gastric Adenocarcinoma	II
NCT03925350	A Phase II Study of Niraparib in Patients With Advanced Melanoma With Genetic Homologous Recombination (HR) Mutation / Alteration	II

Disclaimer: The data presented here is from a curated knowledgebase of publicly available information, but may not be exhaustive. The data version is 2021.11(003).

Clinical Trials Summary (continued)

BRCA2 mutation (continued)

NCT ID	Title	Phase
NCT04493060	Phase II Study of Niraparib and TSR-042 in Patients With Germline or Somatic BRCA1/2 and PALB2-Related Pancreatic Cancer	II
NCT04717154	Phase II INSPIRE Trial: Ipilimumab With Nivolumab In Molecular-Selected Patients With Castration-Resistant Prostate Cancer.	II
NCT04187833	Phase II Trial of Nivolumab in Combination with Talazoparib in Patients with Unresectable or Metastatic Melanoma and Mutations in BRCA or BRCA-ness Genes.	II
NCT03344965	A Phase II Study of Olaparib Monotherapy in Metastatic Breast Cancer Patients With Germline or Somatic Mutations in DNA Repair Genes (Olaparib Expanded)	II
NCT03375307	A Phase II Study of Olaparib (AZD2281) in Patients With Metastatic/Advanced Urothelial Carcinoma With DNA-Repair Defects	II
NCT03742895	A Phase II Study of Olaparib Monotherapy in Participants With Previously Treated, Homologous Recombination Repair Mutation (HRRm) or Homologous Recombination Deficiency (HRD) Positive Advanced Cancer	II
NCT03786796	Phase II Study of Olaparib in Metastatic Renal Cell Carcinoma Patients Harboring a BAP-1 or Other DNA Repair Gene Mutations (ORCHID)	II
NCT04042831	A Phase II Study of Olaparib in Patients With Advanced Biliary Tract Cancer With Aberrant DNA Repair Gene Mutations	II
NCT04515836	Phase II Trial of Olaparib in Homologous Recombination Deficient (HRD) Malignant Mesothelioma	II
NCT04858334	APOLLO: A Randomized Phase II Double-Blind Study of Olaparib Versus Placebo Following Adjuvant Chemotherapy in Patients With Resected Pancreatic Cancer and a Pathogenic BRCA1, BRCA2 or PALB2 Mutation	II
NCT02849496	A Phase II Open-Label, Randomized Study of PARP Inhibition (Olaparib) Either Alone or in Combination With Anti-PD-L1 Therapy (Atezolizumab; MPDL3280A) in Homologous DNA Repair (HDR) Deficient, Locally Advanced or Metastatic Non-HER2-Positive Breast Cancer.	II
NCT04939662	Phase II, Single-arm Study of Olaparib and Bevacizumab Combination Therapy in Relapsed Small Cell Lung Cancer Subjects With DNA Damage Response and the Repair Pathway Alteration, ATM Deficiency, SLFN11 Positive, or POU2F3 Positive	II
NCT04261465	The NUVOLA TRIAL: Neoadjuvant Chemotherapy in Unresectable Ovarian Cancer With OLAparib and Weekly Carboplatin Plus Paclitaxel: A Phase II Open-label Multicentre Study	II
NCT04633902	Phase II Study of Olaparib in Combination With Pembrolizumab in Patients With Advanced Melanoma With Homologous Recombination (HR) Pathway Gene Mutation	II
NCT03248570	Phase II Open Label Study of Pembrolizumab in Patients With Metastatic Castration Resistant Prostate Cancer (mCRPC) With or Without DNA Damage Repair Defects	II
NCT03506997	PERSEUS1: Phase II Trial of the Immune Checkpoint Inhibitor Pembrolizumab for Patients Suffering From Metastatic Prostate Cancer	II
NCT04089189	A Phase II Study to Evaluate the Efficacy, Safety, and Tolerability of IMP4297 Capsules in Subjects With Germline and/or Systemic BRCA1/2 Mutation Positive Advanced Ovarian Cancer With at Least 2 Prior Lines of Standard Systemic Therapy	II
NCT03990896	Evaluation of Talazoparib, a PARP Inhibitor, in Patients With Somatic BRCA Mutant Metastatic Breast Cancer: Genotyping Based Clinical Trial	II
NCT04892693	Talazoparib in Advanced Breast Cancer Patients With Homologous Recombinant Deficiency: A Phase II Clinical and Exploratory Biomarker Study of Talazoparib	II

Clinical Trials Summary (continued)

BRCA2 mutation (continued)

NCT ID	Title	Phase
NCT04740190	Combination Talazoparib - Carboplatin for Recurrent High-grade Glioma With DNA Damage Repair Deficiency (DDRd)	II
NCT04253262	A Phase Ib/II Study of Copanlisib Combined With Rucaparib in Patients With Metastatic Castration-resistant Prostate Cancer	I/II
NCT03685331	Harnessing Olaparib, Palbociclib and Endocrine Therapy: A Phase I/II Trial of Olaparib, Palbociclib and Fulvestrant in Patients With BRCA Mutation-associated, Hormone Receptor-positive, Human Epidermal Growth Factor Receptor 2 (HER2)-Negative Metastatic Breast Cancer (HOPE)	I/II
No NCT ID	An Open-Label, Randomised, Phase I/II Trial Of Rucaparib Combined With Nivolumab + or - Ipilimumab To Augment Response In Homologous Repair Deficient Patients With Relapsed Ovarian, Primary Peritoneal And Fallopian Tube Cancer	I/II
NCT03964532	TALAVE: A Pilot Trial of Induction Talazoparib Followed by Combination of Talazoparib and Avelumab in Advanced Breast Cancer	I/II
NCT04673448	Phase IB Trial of Niraparib and TSR-042 in Patients With BRCA-Mutated Breast, Pancreas or Ovary Cancer	I
NCT04598321	BrUOG 390: Neoadjuvant Treatment With Talazoparib for Women With Newly Diagnosed, Advanced Ovarian Cancer Associated With a Mutation in BRCA1 or BRCA2 (mBRCA)	I
NCT03598270	A Phase III Randomized, Double-blinded Trial of Platinum-based Chemotherapy With or Without Atezolizumab Followed by Niraparib Maintenance With or Without Atezolizumab in Patients With Recurrent Ovarian, Tubal or Peritoneal Cancer and Platinum Treatment-free Interval (TFIp) >6 Months	III
NCT03278717	International Phase III Randomised Study to Evaluate the Efficacy of Maintenance Therapy With Olaparib and Cediranib or Olaparib Alone in Patients With Relapsed Ovarian Cancer Following a Response to Platinum-based Chemotherapy	III
NCT04915755	A Randomized Phase III Double-Blinded Study Comparing the Efficacy and Safety of Niraparib to Placebo in Participants With Either HER2-Negative BRCA-Mutated or Triple-Negative Breast Cancer With Molecular Disease Based on Presence of Circulating Tumor DNA After Definitive Therapy (ZEST)	III
NCT03462342	Combination ATR and PARP Inhibitor (CAPRI) Trial With AZD 6738 and Olaparib in Recurrent Ovarian Cancer	II
No NCT ID	An Investigator-Initiated Phase II Trial Of Niraparib For Patients With BRCA-Mutated Unresectable / Recurrent Biliary Tract, Pancreatic And Other Gastrointestinal Cancer (NIR-B)	II
NCT04716686	Niraparib Monotherapy as Maintain and Recurrent Treatment of Endometrial Serous Carcinoma: A Multi-center, Open-label, Prospective Clinical Study	II
NCT04348045	MAZEPPA: Phase II PRODIGE-GERCOR Study to Evaluate MAintenance Therapy With Olaparib or Selumetinib Plus Durvalumab According to BRCAness and KRAS Somatic Status Personalized in Metastatic Pancreatic Adenocarcinoma Patients	II
NCT03025035	Open Label, Phase II Pilot Study of Immune Checkpoint Inhibition With Pembrolizumab in Combination With PARP Inhibition With Olaparib in Advanced BRCA-mutated or HDR-defect Breast Cancers	II
NCT04666740	A Phase II Trial to Evaluate the Safety and Antitumor Activity of Pembrolizumab and OLApaRib (POLAR) Maintenance for Patients With Metastatic Pancreatic Ductal Adenocarcinoma and Homologous Recombination Deficiency and/or Exceptional Treatment Response to Platinum-Based Therapy	II
NCT02264678	A Modular Phase I, Open-Label, Multicentre Study to Assess the Safety, Tolerability, Pharmacokinetics and Preliminary Anti-tumour Activity of Ceralasertib in Combination With Cytotoxic Chemotherapy and/or DNA Damage Repair/Novel Anti-cancer Agents in Patients With Advanced Solid Malignancies	I

Disclaimer: The data presented here is from a curated knowledgebase of publicly available information, but may not be exhaustive. The data version is 2021.11(003).

Clinical Trials Summary (continued)

BRCA2 mutation (continued)

NCT ID	Title	Phase
NCT04169841	Precision Medicine Phase II Study Evaluating the Efficacy of a Double Immunotherapy by Durvalumab and Tremelimumab Combined With Olaparib in Patients With Solid Cancers and Carriers of Homologous Recombination Repair Genes Mutation in Response or Stable After Olaparib Treatment	II
NCT04030559	Phase II Trial of PARP Inhibitor Niraparib for Men With High Risk Prostate Cancer and DNA Damage Response Defects	II
NCT04940637	A Phase II, Open-Label, Single Arm, Prospective, Multicenter Study of Niraparib Plus Dostarlimab in Patients With Advanced NSCLC and/or MPM, and Positive for PD-L1 Expression and Germline or Somatic Mutations in the HRR Genes	II
NCT04890613	Phase Ib Expansion Study of CX-5461 in Patients With Solid Tumours and BRCA2 and/or PALB2 Mutation	I
NCT05002868	A Multi-center, Open-label, Phase I/Ib Study to Assess the Safety, Pharmacokinetics and Anti-tumor Activity of RP12146, a Poly (ADP-ribose) Polymerase (PARP) Inhibitor, in Patients With Locally Advanced or Metastatic Solid Tumors	I
NCT03395197	A Phase III, Randomized, Double-blind, Placebo-controlled Study Of Talazoparib With Enzalutamide In Metastatic Castration-resistant Prostate Cancer	III
NCT04821622	TALAPRO-3: A Phase III, Randomized, Double-blind, Study of Talazoparib With Enzalutamide Versus Placebo With Enzalutamide in Men With DDR Gene Mutated Metastatic Castration-sensitive Prostate Cancer	III
NCT04038502	An Open-label, Multicenter Phase II Study to Compare the Efficacy of Carboplatin as First-line Followed by Second-line Olaparib Versus Olaparib as First-line Followed by Second-line Carboplatin in the Treatment of Patients With Castration Resistant Prostate Cancer Containing Homologous Recombination Deficiency	II
No NCT ID	Single-arm Exploratory Study for Sintilimab Second-line or Later-line Treatment of Advanced Non-small Cell Lung Cancer with DDR Pathway Gene Mutation	I/II
NCT04095273	A Multicenter, Non-randomized, Open-label Phase Ib Study to Determine the Maximum Tolerated and Recommended Phase II Dose of the ATR Inhibitor BAY1895344 in Combination With Pembrolizumab and to Characterize Its Safety, Tolerability, Pharmacokinetics and Preliminary Anti-tumor Activity in Patients With Advanced Solid Tumors	I
NCT04849364	A Phase II Circulating Tumor DNA Enriched, Genomically Directed Post-neoadjuvant Trial for Patients With Residual Triple Negative Breast Cancer (PERSEVERE)	II

BRCA2 splice site mutation

NCT ID	Title	Phase
NCT02029001	A Two-period, Multicenter, Randomized, Open-label, Phase II Study Evaluating the Clinical Benefit of a Maintenance Treatment Targeting Tumor Molecular Alterations in Patients with Progressive Locally-advanced or Metastatic Solid Tumors.	II
NCT03967938	Efficacy of Olaparib in Advanced Cancers Occurring in Patients With Germline Mutations or Somatic Tumor Mutations in Homologous Recombination Genes	II
NCT02693535	Targeted Agent and Profiling Utilization Registry (TAPUR) Study.	II
NCT04171700	A Phase II Multicenter, Open-label Study of Rucaparib as Treatment for Solid Tumors Associated With Deleterious Mutations in Homologous Recombination Repair Genes	II

Clinical Trials Summary (continued)

BRCA2 splice site mutation (continued)

NCT ID	Title	Phase
NCT02286687	Phase II Study of the PARP Inhibitor Talazoparib in Advanced Cancer Patients With Somatic Alterations in BRCA1/2, Mutations/Deletions in Other BRCA Pathway Genes and Germline Mutation in BRCA1/2 (Not Breast or Ovarian Cancer)	II
NCT04550494	A Pharmacodynamics-Driven Trial of Talazoparib, an Oral PARP Inhibitor, in Patients With Advanced Solid Tumors and Aberrations in Genes Involved in DNA Damage Response	II
NCT03842228	A Phase Ib Biomarker-Driven Combination Trial of Copanlisib, Olaparib, and Durvalumab (MEDI4736) in Patients With Advanced Solid Tumors	I
NCT04586335	Open Label, Phase Ib Study to Evaluate the Safety, Tolerability, Pharmacokinetics and Clinical Activity of CYH33, an Oral a-specific PI3K Inhibitor in Combination With Olaparib, an Oral PARP Inhibitor in Patients With Advanced Solid Tumors.	I
NCT03415659	A Phase I, Open-label, Single-center, Single/Multiple-dose, Dose-escalation/Dose-expansion Clinical Study on Tolerance and Pharmacokinetics of HWH340 Tablet in Patients With Advanced Solid Tumors	I
NCT04197713	Phase I Sequential Trial of Agents Against DNA Repair (STAR)	I
NCT03297606	Canadian Profiling and Targeted Agent Utilization Trial (CAPTUR): A Phase II Basket Trial	II
NCT04497116	Phase I/IIa Study of the Safety, Pharmacokinetics, Pharmacodynamics and Preliminary Clinical Activity of RP-3500 Alone or in Combination With Talazoparib in Advanced Solid Tumors With ATR Inhibitor Sensitizing Mutations (TRESR Study)	I/II
NCT04123366	A Phase II Study of Olaparib in Combination With Pembrolizumab in Participants With Previously Treated, Homologous Recombination Repair Mutation (HRRm) and/or Homologous Recombination Deficiency (HRD)-Positive Advanced Cancer	II
NCT03767075	Basket of Baskets: A Modular, Open-label, Phase II, Multicentre Study To Evaluate Targeted Agents in Molecularly Selected Populations With Advanced Solid Tumours	II
NCT04905914	A Phase I/IIa, Open-Label, Safety, Pharmacokinetic, And Preliminary Efficacy Study Of Oral ATRN-119 In Patients With Advanced Solid Tumors	I/II
NCT04901702	A Randomized Phase I/II Study of Talazoparib or Temozolomide in Combination With Onivyde in Children With Recurrent Solid Malignancies and Ewing Sarcoma	I/II
NCT03155620	NCI-COG Pediatric MATCH (Molecular Analysis for Therapy Choice) Screening Protocol	II
NCT03233204	NCI-COG Pediatric MATCH (Molecular Analysis for Therapy Choice)- Phase 2 Subprotocol of Olaparib in Patients With Tumors Harboring Defects in DNA Damage Repair Genes	II
NCT04267939	An Open-label Phase Ib Study to Determine the Maximum Tolerated and/or Recommended Phase II Dose of the ATR Inhibitor BAY 1895344 in Combination With PARP Inhibitor Niraparib, in Patients With Recurrent Advanced Solid Tumors and Ovarian Cancer	I
NCT04693468	Modular Phase IB Hypothesis-Testing, Biomarker-Driven, Talazoparib Combination Trial (TalaCom)	I
NCT03810105	A Phase II Study of Olaparib and Durvalumab in Men With Castration Sensitive Biochemically Recurrent Non-Metastatic Prostate Cancer Harboring Mutations in DNA Damage Repair	II
NCT02975934	TRITON3: A Multicenter, Randomized, Open Label Phase III Study of Rucaparib Versus Physician's Choice of Therapy for Patients With Metastatic Castration Resistant Prostate Cancer Associated With Homologous Recombination Deficiency	III
NCT04336943	Durvalumab (MEDI4736) and Olaparib (AZD2281) for Treatment of Biochemically Recurrent Prostate Cancer in Men Predicted to Have a High Neoantigen Load: A Pilot Study	II

Disclaimer: The data presented here is from a curated knowledgebase of publicly available information, but may not be exhaustive. The data version is 2021.11(003).

Clinical Trials Summary (continued)

BRCA2 splice site mutation (continued)

NCT ID	Title	Phase
NCT04053322	An International Multicenter Phase II Trial of Durvalumab (MEDI4736) Plus OLAparib Plus Fulvestrant in Metastatic or Locally Advanced ER-positive, HER2-negative Breast Cancer Patients Selected Using Criteria That Predict Sensitivity to Olaparib	II
NCT03012321	BRCAAway: A Randomized Phase II Trial of Abiraterone, Olaparib, or Abiraterone + Olaparib in Patients With Metastatic Castration-Resistant Prostate Cancer With DNA Repair Defects	II
No NCT ID	Phase II Study To Evaluate The Efficacy And Safety Of Niraparib In Recurrent Or Persistent Rare Gynecologic Malignancies With Homologous Recombination Deficiency (JGOG2052)	II
NCT03553004	Niraparib in Metastatic Pancreatic Cancer After Previous Chemotherapy (NIRA-PANC): A Phase II Trial	II
NCT03601923	Phase II Proof-of-Concept Trial Testing the PARP Inhibitor Niraparib in Patients With Pancreatic Cancer Harboring Deficiencies in Homologous Recombination DNA Repair	II
NCT03840967	A Phase II Study Evaluating Safety and Efficacy of Niraparib in Patients With Previously Treated Homologous Recombination (HR) Defective or Loss of Heterozygosity (LOH) High Metastatic Esophageal/Gastroesophageal Junction/Proximal Gastric Adenocarcinoma	II
NCT03925350	A Phase II Study of Niraparib in Patients With Advanced Melanoma With Genetic Homologous Recombination (HR) Mutation / Alteration	II
NCT04493060	Phase II Study of Niraparib and TSR-042 in Patients With Germline or Somatic BRCA1/2 and PALB2-Related Pancreatic Cancer	II
NCT04717154	Phase II INSPIRE Trial: Ipilimumab With Nivolumab In Molecular-Selected Patients With Castration-Resistant Prostate Cancer.	II
NCT04187833	Phase II Trial of Nivolumab in Combination with Talazoparib in Patients with Unresectable or Metastatic Melanoma and Mutations in BRCA or BRCA-ness Genes.	II
NCT03344965	A Phase II Study of Olaparib Monotherapy in Metastatic Breast Cancer Patients With Germline or Somatic Mutations in DNA Repair Genes (Olaparib Expanded)	II
NCT03375307	A Phase II Study of Olaparib (AZD2281) in Patients With Metastatic/Advanced Urothelial Carcinoma With DNA-Repair Defects	II
NCT03742895	A Phase II Study of Olaparib Monotherapy in Participants With Previously Treated, Homologous Recombination Repair Mutation (HRRm) or Homologous Recombination Deficiency (HRD) Positive Advanced Cancer	II
NCT03786796	Phase II Study of Olaparib in Metastatic Renal Cell Carcinoma Patients Harboring a BAP-1 or Other DNA Repair Gene Mutations (ORCHID)	II
NCT04042831	A Phase II Study of Olaparib in Patients With Advanced Biliary Tract Cancer With Aberrant DNA Repair Gene Mutations	II
NCT04515836	Phase II Trial of Olaparib in Homologous Recombination Deficient (HRD) Malignant Mesothelioma	II
NCT04858334	APOLLO: A Randomized Phase II Double-Blind Study of Olaparib Versus Placebo Following Adjuvant Chemotherapy in Patients With Resected Pancreatic Cancer and a Pathogenic BRCA1, BRCA2 or PALB2 Mutation	II
NCT02849496	A Phase II Open-Label, Randomized Study of PARP Inhibition (Olaparib) Either Alone or in Combination With Anti-PD-L1 Therapy (Atezolizumab; MPDL3280A) in Homologous DNA Repair (HDR) Deficient, Locally Advanced or Metastatic Non-HER2-Positive Breast Cancer.	II
NCT04939662	Phase II, Single-arm Study of Olaparib and Bevacizumab Combination Therapy in Relapsed Small Cell Lung Cancer Subjects With DNA Damage Response and the Repair Pathway Alteration, ATM Deficiency, SLFN11 Positive, or POU2F3 Positive	II

Clinical Trials Summary (continued)

BRCA2 splice site mutation (continued)

NCT ID	Title	Phase
NCT04261465	The NUVOLA TRIAL: Neoadjuvant Chemotherapy in Unresectable Ovarian Cancer With OLAparib and Weekly Carboplatin Plus Paclitaxel: A Phase II Open-label Multicentre Study	II
NCT04633902	Phase II Study of Olaparib in Combination With Pembrolizumab in Patients With Advanced Melanoma With Homologous Recombination (HR) Pathway Gene Mutation	II
NCT03248570	Phase II Open Label Study of Pembrolizumab in Patients With Metastatic Castration Resistant Prostate Cancer (mCRPC) With or Without DNA Damage Repair Defects	II
NCT03506997	PERSEUS1: Phase II Trial of the Immune Checkpoint Inhibitor Pembrolizumab for Patients Suffering From Metastatic Prostate Cancer	II
NCT04089189	A Phase II Study to Evaluate the Efficacy, Safety, and Tolerability of IMP4297 Capsules in Subjects With Germline and/or Systemic BRCA1/2 Mutation Positive Advanced Ovarian Cancer With at Least 2 Prior Lines of Standard Systemic Therapy	II
NCT03990896	Evaluation of Talazoparib, a PARP Inhibitor, in Patients With Somatic BRCA Mutant Metastatic Breast Cancer: Genotyping Based Clinical Trial	II
NCT04892693	Talazoparib in Advanced Breast Cancer Patients With Homologous Recombinant Deficiency: A Phase II Clinical and Exploratory Biomarker Study of Talazoparib	II
NCT04740190	Combination Talazoparib - Carboplatin for Recurrent High-grade Glioma With DNA Damage Repair Deficiency (DDRd)	II
NCT04253262	A Phase Ib/II Study of Copanlisib Combined With Rucaparib in Patients With Metastatic Castration-resistant Prostate Cancer	I/II
NCT03685331	Harnessing Olaparib, Palbociclib and Endocrine Therapy: A Phase I/II Trial of Olaparib, Palbociclib and Fulvestrant in Patients With BRCA Mutation-associated, Hormone Receptor-positive, Human Epidermal Growth Factor Receptor 2 (HER2)-Negative Metastatic Breast Cancer (HOPE)	I/II
No NCT ID	An Open-Label, Randomised, Phase I/II Trial Of Rucaparib Combined With Nivolumab + or - Ipilimumab To Augment Response In Homologous Repair Deficient Patients With Relapsed Ovarian, Primary Peritoneal And Fallopian Tube Cancer	I/II
NCT03964532	TALAVE: A Pilot Trial of Induction Talazoparib Followed by Combination of Talazoparib and Avelumab in Advanced Breast Cancer	I/II
NCT04673448	Phase IB Trial of Niraparib and TSR-042 in Patients With BRCA-Mutated Breast, Pancreas or Ovary Cancer	I
NCT04598321	BrUOG 390: Neoadjuvant Treatment With Talazoparib for Women With Newly Diagnosed, Advanced Ovarian Cancer Associated With a Mutation in BRCA1 or BRCA2 (mBRCA)	I
NCT03598270	A Phase III Randomized, Double-blinded Trial of Platinum-based Chemotherapy With or Without Atezolizumab Followed by Niraparib Maintenance With or Without Atezolizumab in Patients With Recurrent Ovarian, Tubal or Peritoneal Cancer and Platinum Treatment-free Interval (TFIp) >6 Months	III
NCT03278717	International Phase III Randomised Study to Evaluate the Efficacy of Maintenance Therapy With Olaparib and Cediranib or Olaparib Alone in Patients With Relapsed Ovarian Cancer Following a Response to Platinum-based Chemotherapy	III
NCT04915755	A Randomized Phase III Double-Blinded Study Comparing the Efficacy and Safety of Niraparib to Placebo in Participants With Either HER2-Negative BRCA-Mutated or Triple-Negative Breast Cancer With Molecular Disease Based on Presence of Circulating Tumor DNA After Definitive Therapy (ZEST)	III
NCT03462342	Combination ATR and PARP Inhibitor (CAPRI) Trial With AZD 6738 and Olaparib in Recurrent Ovarian Cancer	II

Disclaimer: The data presented here is from a curated knowledgebase of publicly available information, but may not be exhaustive. The data version is 2021.11(003).

Clinical Trials Summary (continued)

BRCA2 splice site mutation (continued)

NCT ID	Title	Phase
No NCT ID	An Investigator-Initiated Phase II Trial Of Niraparib For Patients With BRCA-Mutated Unresectable / Recurrent Biliary Tract, Pancreatic And Other Gastrointestinal Cancer (NIR-B)	II
NCT04716686	Niraparib Monotherapy as Maintain and Recurrent Treatment of Endometrial Serous Carcinoma: A Multi-center, Open-label, Prospective Clinical Study	II
NCT04348045	MAZEPPA: Phase II PRODIGE-GERCOR Study to Evaluate MAintenance Therapy With Olaparib or Selumetinib Plus Durvalumab According to BRCAness and KRAS Somatic Status Personalized in Metastatic Pancreatic Adenocarcinoma Patients	II
NCT03025035	Open Label, Phase II Pilot Study of Immune Checkpoint Inhibition With Pembrolizumab in Combination With PARP Inhibition With Olaparib in Advanced BRCA-mutated or HDR-defect Breast Cancers	II
NCT04666740	A Phase II Trial to Evaluate the Safety and Antitumor Activity of Pembrolizumab and OLApaRib (POLAR) Maintenance for Patients With Metastatic Pancreatic Ductal Adenocarcinoma and Homologous Recombination Deficiency and/or Exceptional Treatment Response to Platinum-Based Therapy	II
NCT02264678	A Modular Phase I, Open-Label, Multicentre Study to Assess the Safety, Tolerability, Pharmacokinetics and Preliminary Anti-tumour Activity of Ceralasertib in Combination With Cytotoxic Chemotherapy and/or DNA Damage Repair/Novel Anti-cancer Agents in Patients With Advanced Solid Malignancies	I
NCT04169841	Precision Medicine Phase II Study Evaluating the Efficacy of a Double Immunotherapy by Durvalumab and Tremelimumab Combined With Olaparib in Patients With Solid Cancers and Carriers of Homologous Recombination Repair Genes Mutation in Response or Stable After Olaparib Treatment	II
NCT04030559	Phase II Trial of PARP Inhibitor Niraparib for Men With High Risk Prostate Cancer and DNA Damage Response Defects	II
NCT04940637	A Phase II, Open-Label, Single Arm, Prospective, Multicenter Study of Niraparib Plus Dostarlimab in Patients With Advanced NSCLC and/or MPM, and Positive for PD-L1 Expression and Germline or Somatic Mutations in the HRR Genes	II
NCT04890613	Phase Ib Expansion Study of CX-5461 in Patients With Solid Tumours and BRCA2 and/or PALB2 Mutation	I
NCT05002868	A Multi-center, Open-label, Phase I/Ib Study to Assess the Safety, Pharmacokinetics and Anti-tumor Activity of RP12146, a Poly (ADP-ribose) Polymerase (PARP) Inhibitor, in Patients With Locally Advanced or Metastatic Solid Tumors	I
NCT03395197	A Phase III, Randomized, Double-blind, Placebo-controlled Study Of Talazoparib With Enzalutamide In Metastatic Castration-resistant Prostate Cancer	III
NCT04821622	TALAPRO-3: A Phase III, Randomized, Double-blind, Study of Talazoparib With Enzalutamide Versus Placebo With Enzalutamide in Men With DDR Gene Mutated Metastatic Castration-sensitive Prostate Cancer	III
NCT04038502	An Open-label, Multicenter Phase II Study to Compare the Efficacy of Carboplatin as First-line Followed by Second-line Olaparib Versus Olaparib as First-line Followed by Second-line Carboplatin in the Treatment of Patients With Castration Resistant Prostate Cancer Containing Homologous Recombination Deficiency	II
No NCT ID	Single-arm Exploratory Study for Sintilimab Second-line or Later-line Treatment of Advanced Non-small Cell Lung Cancer with DDR Pathway Gene Mutation	I/II
NCT04095273	A Multicenter, Non-randomized, Open-label Phase Ib Study to Determine the Maximum Tolerated and Recommended Phase II Dose of the ATR Inhibitor BAY1895344 in Combination With Pembrolizumab and to Characterize Its Safety, Tolerability, Pharmacokinetics and Preliminary Anti-tumor Activity in Patients With Advanced Solid Tumors	I

Disclaimer: The data presented here is from a curated knowledgebase of publicly available information, but may not be exhaustive. The data version is 2021.11(003).

Clinical Trials Summary (continued)

BRCA2 splice site mutation (continued)

NCT ID	Title	Phase
NCT04849364	A Phase II Circulating Tumor DNA Enriched, Genomically Directed Post-neoadjuvant Trial for Patients With Residual Triple Negative Breast Cancer (PERSEVERE)	II

BRCA2 truncating mutation

NCT ID	Title	Phase
NCT02029001	A Two-period, Multicenter, Randomized, Open-label, Phase II Study Evaluating the Clinical Benefit of a Maintenance Treatment Targeting Tumor Molecular Alterations in Patients with Progressive Locally-advanced or Metastatic Solid Tumors.	II
NCT03967938	Efficacy of Olaparib in Advanced Cancers Occurring in Patients With Germline Mutations or Somatic Tumor Mutations in Homologous Recombination Genes	II
NCT02693535	Targeted Agent and Profiling Utilization Registry (TAPUR) Study.	II
NCT04171700	A Phase II Multicenter, Open-label Study of Rucaparib as Treatment for Solid Tumors Associated With Deleterious Mutations in Homologous Recombination Repair Genes	II
NCT02286687	Phase II Study of the PARP Inhibitor Talazoparib in Advanced Cancer Patients With Somatic Alterations in BRCA1/2, Mutations/Deletions in Other BRCA Pathway Genes and Germline Mutation in BRCA1/2 (Not Breast or Ovarian Cancer)	II
NCT04550494	A Pharmacodynamics-Driven Trial of Talazoparib, an Oral PARP Inhibitor, in Patients With Advanced Solid Tumors and Aberrations in Genes Involved in DNA Damage Response	II
NCT03842228	A Phase Ib Biomarker-Driven Combination Trial of Copanlisib, Olaparib, and Durvalumab (MEDI4736) in Patients With Advanced Solid Tumors	I
NCT04586335	Open Label, Phase Ib Study to Evaluate the Safety, Tolerability, Pharmacokinetics and Clinical Activity of CYH33, an Oral a-specific PI3K Inhibitor in Combination With Olaparib, an Oral PARP Inhibitor in Patients With Advanced Solid Tumors.	I
NCT03415659	A Phase I, Open-label, Single-center, Single/Multiple-dose, Dose-escalation/Dose-expansion Clinical Study on Tolerance and Pharmacokinetics of HWH340 Tablet in Patients With Advanced Solid Tumors	I
NCT04197713	Phase I Sequential Trial of Agents Against DNA Repair (STAR)	I
NCT03297606	Canadian Profiling and Targeted Agent Utilization Trial (CAPTUR): A Phase II Basket Trial	II
NCT04497116	Phase I/IIa Study of the Safety, Pharmacokinetics, Pharmacodynamics and Preliminary Clinical Activity of RP-3500 Alone or in Combination With Talazoparib in Advanced Solid Tumors With ATR Inhibitor Sensitizing Mutations (TRESR Study)	I/II
NCT04123366	A Phase II Study of Olaparib in Combination With Pembrolizumab in Participants With Previously Treated, Homologous Recombination Repair Mutation (HRRm) and/or Homologous Recombination Deficiency (HRD)-Positive Advanced Cancer	II
NCT03767075	Basket of Baskets: A Modular, Open-label, Phase II, Multicentre Study To Evaluate Targeted Agents in Molecularly Selected Populations With Advanced Solid Tumours	II
NCT04905914	A Phase I/IIa, Open-Label, Safety, Pharmacokinetic, And Preliminary Efficacy Study Of Oral ATRN-119 In Patients With Advanced Solid Tumors	I/II
NCT04901702	A Randomized Phase I/II Study of Talazoparib or Temozolomide in Combination With Onivyde in Children With Recurrent Solid Malignancies and Ewing Sarcoma	I/II
NCT03155620	NCI-COG Pediatric MATCH (Molecular Analysis for Therapy Choice) Screening Protocol	II

Disclaimer: The data presented here is from a curated knowledgebase of publicly available information, but may not be exhaustive. The data version is 2021.11(003).

Clinical Trials Summary (continued)

BRCA2 truncating mutation (continued)

NCT ID	Title	Phase
NCT03233204	NCI-COG Pediatric MATCH (Molecular Analysis for Therapy Choice)- Phase 2 Subprotocol of Olaparib in Patients With Tumors Harboring Defects in DNA Damage Repair Genes	II
NCT04267939	An Open-label Phase Ib Study to Determine the Maximum Tolerated and/or Recommended Phase II Dose of the ATR Inhibitor BAY 1895344 in Combination With PARP Inhibitor Niraparib, in Patients With Recurrent Advanced Solid Tumors and Ovarian Cancer	I
NCT04693468	Modular Phase IB Hypothesis-Testing, Biomarker-Driven, Talazoparib Combination Trial (TalaCom)	I
NCT03810105	A Phase II Study of Olaparib and Durvalumab in Men With Castration Sensitive Biochemically Recurrent Non-Metastatic Prostate Cancer Harboring Mutations in DNA Damage Repair	II
NCT03840967	A Phase II Study Evaluating Safety and Efficacy of Niraparib in Patients With Previously Treated Homologous Recombination (HR) Defective or Loss of Heterozygosity (LOH) High Metastatic Esophageal/Gastroesophageal Junction/Proximal Gastric Adenocarcinoma	II
NCT02975934	TRITON3: A Multicenter, Randomized, Open Label Phase III Study of Rucaparib Versus Physician's Choice of Therapy for Patients With Metastatic Castration Resistant Prostate Cancer Associated With Homologous Recombination Deficiency	III
NCT04336943	Durvalumab (MEDI4736) and Olaparib (AZD2281) for Treatment of Biochemically Recurrent Prostate Cancer in Men Predicted to Have a High Neoantigen Load: A Pilot Study	II
NCT04053322	An International Multicenter Phase II Trial of Durvalumab (MEDI4736) Plus OLAparib Plus Fulvestrant in Metastatic or Locally Advanced ER-positive, HER2-negative Breast Cancer Patients Selected Using Criteria That Predict Sensitivity to Olaparib	II
NCT03012321	BRCAAway: A Randomized Phase II Trial of Abiraterone, Olaparib, or Abiraterone + Olaparib in Patients With Metastatic Castration-Resistant Prostate Cancer With DNA Repair Defects	II
No NCT ID	Phase II Study To Evaluate The Efficacy And Safety Of Niraparib In Recurrent Or Persistent Rare Gynecologic Malignancies With Homologous Recombination Deficiency (JGOG2052)	II
NCT03553004	Niraparib in Metastatic Pancreatic Cancer After Previous Chemotherapy (NIRA-PANC): A Phase II Trial	II
NCT03601923	Phase II Proof-of-Concept Trial Testing the PARP Inhibitor Niraparib in Patients With Pancreatic Cancer Harboring Deficiencies in Homologous Recombination DNA Repair	II
NCT03925350	A Phase II Study of Niraparib in Patients With Advanced Melanoma With Genetic Homologous Recombination (HR) Mutation / Alteration	II
NCT04493060	Phase II Study of Niraparib and TSR-042 in Patients With Germline or Somatic BRCA1/2 and PALB2-Related Pancreatic Cancer	II
NCT04717154	Phase II INSPIRE Trial: Ipilimumab With Nivolumab In Molecular-Selected Patients With Castration-Resistant Prostate Cancer.	II
NCT04187833	Phase II Trial of Nivolumab in Combination with Talazoparib in Patients with Unresectable or Metastatic Melanoma and Mutations in BRCA or BRCA-ness Genes.	II
NCT03344965	A Phase II Study of Olaparib Monotherapy in Metastatic Breast Cancer Patients With Germline or Somatic Mutations in DNA Repair Genes (Olaparib Expanded)	II
NCT03375307	A Phase II Study of Olaparib (AZD2281) in Patients With Metastatic/Advanced Urothelial Carcinoma With DNA-Repair Defects	II
NCT03742895	A Phase II Study of Olaparib Monotherapy in Participants With Previously Treated, Homologous Recombination Repair Mutation (HRRm) or Homologous Recombination Deficiency (HRD) Positive Advanced Cancer	II

Clinical Trials Summary (continued)

BRCA2 truncating mutation (continued)

NCT ID	Title	Phase
NCT03786796	Phase II Study of Olaparib in Metastatic Renal Cell Carcinoma Patients Harboring a BAP-1 or Other DNA Repair Gene Mutations (ORCHID)	II
NCT04042831	A Phase II Study of Olaparib in Patients With Advanced Biliary Tract Cancer With Aberrant DNA Repair Gene Mutations	II
NCT04515836	Phase II Trial of Olaparib in Homologous Recombination Deficient (HRD) Malignant Mesothelioma	II
NCT04858334	APOLLO: A Randomized Phase II Double-Blind Study of Olaparib Versus Placebo Following Adjuvant Chemotherapy in Patients With Resected Pancreatic Cancer and a Pathogenic BRCA1, BRCA2 or PALB2 Mutation	II
NCT02849496	A Phase II Open-Label, Randomized Study of PARP Inhibition (Olaparib) Either Alone or in Combination With Anti-PD-L1 Therapy (Atezolizumab; MPDL3280A) in Homologous DNA Repair (HDR) Deficient, Locally Advanced or Metastatic Non-HER2-Positive Breast Cancer.	II
NCT04939662	Phase II, Single-arm Study of Olaparib and Bevacizumab Combination Therapy in Relapsed Small Cell Lung Cancer Subjects With DNA Damage Response and the Repair Pathway Alteration, ATM Deficiency, SLFN11 Positive, or POU2F3 Positive	II
NCT04261465	The NUVOLA TRIAL: Neoadjuvant Chemotherapy in Unresectable Ovarian Cancer With OLAparib and Weekly Carboplatin Plus Paclitaxel: A Phase II Open-label Multicentre Study	II
NCT04633902	Phase II Study of Olaparib in Combination With Pembrolizumab in Patients With Advanced Melanoma With Homologous Recombination (HR) Pathway Gene Mutation	II
NCT03248570	Phase II Open Label Study of Pembrolizumab in Patients With Metastatic Castration Resistant Prostate Cancer (mCRPC) With or Without DNA Damage Repair Defects	II
NCT03506997	PERSEUS1: Phase II Trial of the Immune Checkpoint Inhibitor Pembrolizumab for Patients Suffering From Metastatic Prostate Cancer	II
NCT04089189	A Phase II Study to Evaluate the Efficacy, Safety, and Tolerability of IMP4297 Capsules in Subjects With Germline and/or Systemic BRCA1/2 Mutation Positive Advanced Ovarian Cancer With at Least 2 Prior Lines of Standard Systemic Therapy	II
NCT03990896	Evaluation of Talazoparib, a PARP Inhibitor, in Patients With Somatic BRCA Mutant Metastatic Breast Cancer: Genotyping Based Clinical Trial	II
NCT04892693	Talazoparib in Advanced Breast Cancer Patients With Homologous Recombinant Deficiency: A Phase II Clinical and Exploratory Biomarker Study of Talazoparib	II
NCT04740190	Combination Talazoparib - Carboplatin for Recurrent High-grade Glioma With DNA Damage Repair Deficiency (DDRd)	II
NCT04253262	A Phase Ib/II Study of Copanlisib Combined With Rucaparib in Patients With Metastatic Castration-resistant Prostate Cancer	I/II
NCT03685331	Harnessing Olaparib, Palbociclib and Endocrine Therapy: A Phase I/II Trial of Olaparib, Palbociclib and Fulvestrant in Patients With BRCA Mutation-associated, Hormone Receptor-positive, Human Epidermal Growth Factor Receptor 2 (HER2)-Negative Metastatic Breast Cancer (HOPE)	I/II
No NCT ID	An Open-Label, Randomised, Phase I/II Trial Of Rucaparib Combined With Nivolumab + or - Ipilimumab To Augment Response In Homologous Repair Deficient Patients With Relapsed Ovarian, Primary Peritoneal And Fallopian Tube Cancer	I/II
NCT03964532	TALAVE: A Pilot Trial of Induction Talazoparib Followed by Combination of Talazoparib and Avelumab in Advanced Breast Cancer	I/II

Disclaimer: The data presented here is from a curated knowledgebase of publicly available information, but may not be exhaustive. The data version is 2021.11(003).

Clinical Trials Summary (continued)

BRCA2 truncating mutation (continued)

NCT ID	Title	Phase
NCT04673448	Phase IB Trial of Niraparib and TSR-042 in Patients With BRCA-Mutated Breast, Pancreas or Ovary Cancer	I
NCT04598321	BrUOG 390: Neoadjuvant Treatment With Talazoparib for Women With Newly Diagnosed, Advanced Ovarian Cancer Associated With a Mutation in BRCA1 or BRCA2 (mBRCA)	I
NCT03598270	A Phase III Randomized, Double-blinded Trial of Platinum-based Chemotherapy With or Without Atezolizumab Followed by Niraparib Maintenance With or Without Atezolizumab in Patients With Recurrent Ovarian, Tubal or Peritoneal Cancer and Platinum Treatment-free Interval (TFIp) >6 Months	III
NCT03278717	International Phase III Randomised Study to Evaluate the Efficacy of Maintenance Therapy With Olaparib and Cediranib or Olaparib Alone in Patients With Relapsed Ovarian Cancer Following a Response to Platinum-based Chemotherapy	III
NCT04915755	A Randomized Phase III Double-Blinded Study Comparing the Efficacy and Safety of Niraparib to Placebo in Participants With Either HER2-Negative BRCA-Mutated or Triple-Negative Breast Cancer With Molecular Disease Based on Presence of Circulating Tumor DNA After Definitive Therapy (ZEST)	III
NCT03462342	Combination ATR and PARP Inhibitor (CAPRI) Trial With AZD 6738 and Olaparib in Recurrent Ovarian Cancer	II
No NCT ID	An Investigator-Initiated Phase II Trial Of Niraparib For Patients With BRCA-Mutated Unresectable / Recurrent Biliary Tract, Pancreatic And Other Gastrointestinal Cancer (NIR-B)	II
NCT04716686	Niraparib Monotherapy as Maintain and Recurrent Treatment of Endometrial Serous Carcinoma: A Multi-center, Open-label, Prospective Clinical Study	II
NCT04348045	MAZEPPA: Phase II PRODIGE-GERCOR Study to Evaluate MAintenance Therapy With Olaparib or Selumetinib Plus Durvalumab According to BRCAness and KRAS Somatic Status Personalized in Metastatic Pancreatic Adenocarcinoma Patients	II
NCT03025035	Open Label, Phase II Pilot Study of Immune Checkpoint Inhibition With Pembrolizumab in Combination With PARP Inhibition With Olaparib in Advanced BRCA-mutated or HDR-defect Breast Cancers	II
NCT04666740	A Phase II Trial to Evaluate the Safety and Antitumor Activity of Pembrolizumab and OLApaRib (POLAR) Maintenance for Patients With Metastatic Pancreatic Ductal Adenocarcinoma and Homologous Recombination Deficiency and/or Exceptional Treatment Response to Platinum-Based Therapy	II
NCT02264678	A Modular Phase I, Open-Label, Multicentre Study to Assess the Safety, Tolerability, Pharmacokinetics and Preliminary Anti-tumour Activity of Ceralasertib in Combination With Cytotoxic Chemotherapy and/or DNA Damage Repair/Novel Anti-cancer Agents in Patients With Advanced Solid Malignancies	I
NCT04169841	Precision Medicine Phase II Study Evaluating the Efficacy of a Double Immunotherapy by Durvalumab and Tremelimumab Combined With Olaparib in Patients With Solid Cancers and Carriers of Homologous Recombination Repair Genes Mutation in Response or Stable After Olaparib Treatment	II
NCT04030559	Phase II Trial of PARP Inhibitor Niraparib for Men With High Risk Prostate Cancer and DNA Damage Response Defects	II
NCT04940637	A Phase II, Open-Label, Single Arm, Prospective, Multicenter Study of Niraparib Plus Dostarlimab in Patients With Advanced NSCLC and/or MPM, and Positive for PD-L1 Expression and Germline or Somatic Mutations in the HRR Genes	II
NCT04890613	Phase Ib Expansion Study of CX-5461 in Patients With Solid Tumours and BRCA2 and/or PALB2 Mutation	I
NCT05002868	A Multi-center, Open-label, Phase I/Ib Study to Assess the Safety, Pharmacokinetics and Anti-tumor Activity of RP12146, a Poly (ADP-ribose) Polymerase (PARP) Inhibitor, in Patients With Locally Advanced or Metastatic Solid Tumors	I

Disclaimer: The data presented here is from a curated knowledgebase of publicly available information, but may not be exhaustive. The data version is 2021.11(003).

Clinical Trials Summary (continued)

BRCA2 truncating mutation (continued)

NCT ID	Title	Phase
NCT03395197	A Phase III, Randomized, Double-blind, Placebo-controlled Study Of Talazoparib With Enzalutamide In Metastatic Castration-resistant Prostate Cancer	III
NCT04821622	TALAPRO-3: A Phase III, Randomized, Double-blind, Study of Talazoparib With Enzalutamide Versus Placebo With Enzalutamide in Men With DDR Gene Mutated Metastatic Castration-sensitive Prostate Cancer	III
NCT04038502	An Open-label, Multicenter Phase II Study to Compare the Efficacy of Carboplatin as First-line Followed by Second-line Olaparib Versus Olaparib as First-line Followed by Second-line Carboplatin in the Treatment of Patients With Castration Resistant Prostate Cancer Containing Homologous Recombination Deficiency	II
No NCT ID	Single-arm Exploratory Study for Sintilimab Second-line or Later-line Treatment of Advanced Non-small Cell Lung Cancer with DDR Pathway Gene Mutation	I/II
NCT04095273	A Multicenter, Non-randomized, Open-label Phase Ib Study to Determine the Maximum Tolerated and Recommended Phase II Dose of the ATR Inhibitor BAY1895344 in Combination With Pembrolizumab and to Characterize Its Safety, Tolerability, Pharmacokinetics and Preliminary Anti-tumor Activity in Patients With Advanced Solid Tumors	I
NCT04849364	A Phase II Circulating Tumor DNA Enriched, Genomically Directed Post-neoadjuvant Trial for Patients With Residual Triple Negative Breast Cancer (PERSEVERE)	II

BRCA2 deletion

NCT ID	Title	Phase
NCT02286687	Phase II Study of the PARP Inhibitor Talazoparib in Advanced Cancer Patients With Somatic Alterations in BRCA1/2, Mutations/Deletions in Other BRCA Pathway Genes and Germline Mutation in BRCA1/2 (Not Breast or Ovarian Cancer)	II
NCT03297606	Canadian Profiling and Targeted Agent Utilization Trial (CAPTUR): A Phase II Basket Trial	II
NCT04497116	Phase I/IIa Study of the Safety, Pharmacokinetics, Pharmacodynamics and Preliminary Clinical Activity of RP-3500 Alone or in Combination With Talazoparib in Advanced Solid Tumors With ATR Inhibitor Sensitizing Mutations (TRESR Study)	I/II
NCT04901702	A Randomized Phase I/II Study of Talazoparib or Temozolomide in Combination With Onivyde in Children With Recurrent Solid Malignancies and Ewing Sarcoma	I/II
NCT03155620	NCI-COG Pediatric MATCH (Molecular Analysis for Therapy Choice) Screening Protocol	II
NCT03233204	NCI-COG Pediatric MATCH (Molecular Analysis for Therapy Choice)- Phase 2 Subprotocol of Olaparib in Patients With Tumors Harboring Defects in DNA Damage Repair Genes	II
NCT04267939	An Open-label Phase Ib Study to Determine the Maximum Tolerated and/or Recommended Phase II Dose of the ATR Inhibitor BAY 1895344 in Combination With PARP Inhibitor Niraparib, in Patients With Recurrent Advanced Solid Tumors and Ovarian Cancer	I
NCT04693468	Modular Phase IB Hypothesis-Testing, Biomarker-Driven, Talazoparib Combination Trial (TalaCom)	I
NCT03810105	A Phase II Study of Olaparib and Durvalumab in Men With Castration Sensitive Biochemically Recurrent Non-Metastatic Prostate Cancer Harboring Mutations in DNA Damage Repair	II
NCT03012321	BRCAAway: A Randomized Phase II Trial of Abiraterone, Olaparib, or Abiraterone + Olaparib in Patients With Metastatic Castration-Resistant Prostate Cancer With DNA Repair Defects	II

Disclaimer: The data presented here is from a curated knowledgebase of publicly available information, but may not be exhaustive. The data version is 2021.11(003).

Clinical Trials Summary (continued)

BRCA2 deletion (continued)

NCT ID	Title	Phase
NCT03840967	A Phase II Study Evaluating Safety and Efficacy of Niraparib in Patients With Previously Treated Homologous Recombination (HR) Defective or Loss of Heterozygosity (LOH) High Metastatic Esophageal/Gastroesophageal Junction/Proximal Gastric Adenocarcinoma	II
NCT04633902	Phase II Study of Olaparib in Combination With Pembrolizumab in Patients With Advanced Melanoma With Homologous Recombination (HR) Pathway Gene Mutation	II
NCT03248570	Phase II Open Label Study of Pembrolizumab in Patients With Metastatic Castration Resistant Prostate Cancer (mCRPC) With or Without DNA Damage Repair Defects	II
NCT04740190	Combination Talazoparib - Carboplatin for Recurrent High-grade Glioma With DNA Damage Repair Deficiency (DDRd)	II
NCT04666740	A Phase II Trial to Evaluate the Safety and Antitumor Activity of Pembrolizumab and OLApaRib (POLAR) Maintenance for Patients With Metastatic Pancreatic Ductal Adenocarcinoma and Homologous Recombination Deficiency and/or Exceptional Treatment Response to Platinum-Based Therapy	II
NCT04030559	Phase II Trial of PARP Inhibitor Niraparib for Men With High Risk Prostate Cancer and DNA Damage Response Defects	II
NCT03344965	A Phase II Study of Olaparib Monotherapy in Metastatic Breast Cancer Patients With Germline or Somatic Mutations in DNA Repair Genes (Olaparib Expanded)	II
NCT03506997	PERSEUS1: Phase II Trial of the Immune Checkpoint Inhibitor Pembrolizumab for Patients Suffering From Metastatic Prostate Cancer	II
NCT04849364	A Phase II Circulating Tumor DNA Enriched, Genomically Directed Post-neoadjuvant Trial for Patients With Residual Triple Negative Breast Cancer (PERSEVERE)	II
NCT05002868	A Multi-center, Open-label, Phase I/Ib Study to Assess the Safety, Pharmacokinetics and Anti-tumor Activity of RP12146, a Poly (ADP-ribose) Polymerase (PARP) Inhibitor, in Patients With Locally Advanced or Metastatic Solid Tumors	I

BRCA2 fusion

NCT ID	Title	Phase
NCT03297606	Canadian Profiling and Targeted Agent Utilization Trial (CAPTUR): A Phase II Basket Trial	II
NCT04497116	Phase I/IIa Study of the Safety, Pharmacokinetics, Pharmacodynamics and Preliminary Clinical Activity of RP-3500 Alone or in Combination With Talazoparib in Advanced Solid Tumors With ATR Inhibitor Sensitizing Mutations (TRESR Study)	I/II
NCT02286687	Phase II Study of the PARP Inhibitor Talazoparib in Advanced Cancer Patients With Somatic Alterations in BRCA1/2, Mutations/Deletions in Other BRCA Pathway Genes and Germline Mutation in BRCA1/2 (Not Breast or Ovarian Cancer)	II
NCT04901702	A Randomized Phase I/II Study of Talazoparib or Temozolomide in Combination With Onivyde in Children With Recurrent Solid Malignancies and Ewing Sarcoma	I/II
NCT03155620	NCI-COG Pediatric MATCH (Molecular Analysis for Therapy Choice) Screening Protocol	II
NCT03233204	NCI-COG Pediatric MATCH (Molecular Analysis for Therapy Choice)- Phase 2 Subprotocol of Olaparib in Patients With Tumors Harboring Defects in DNA Damage Repair Genes	II
NCT04267939	An Open-label Phase Ib Study to Determine the Maximum Tolerated and/or Recommended Phase II Dose of the ATR Inhibitor BAY 1895344 in Combination With PARP Inhibitor Niraparib, in Patients With Recurrent Advanced Solid Tumors and Ovarian Cancer	I

Disclaimer: The data presented here is from a curated knowledgebase of publicly available information, but may not be exhaustive. The data version is 2021.11(003).

Clinical Trials Summary (continued)

BRCA2 fusion (continued)

NCT ID	Title	Phase
NCT04693468	Modular Phase IB Hypothesis-Testing, Biomarker-Driven, Talazoparib Combination Trial (TalaCom)	I
NCT03840967	A Phase II Study Evaluating Safety and Efficacy of Niraparib in Patients With Previously Treated Homologous Recombination (HR) Defective or Loss of Heterozygosity (LOH) High Metastatic Esophageal/Gastroesophageal Junction/Proximal Gastric Adenocarcinoma	II
NCT02502266	A Randomized Phase II/III Study of the Combination of Cediranib and Olaparib Compared to Cediranib or Olaparib Alone, or Standard of Care Chemotherapy in Women With Recurrent Platinum-Resistant or -Refractory Ovarian, Fallopian Tube, or Primary Peritoneal Cancer (COCOS)	II/III
NCT04739800	A Randomized Phase II Trial of Triplet Therapy (a PD-L1 Inhibitor (Durvalumab) MEDI4736 in Combination With Olaparib and Cediranib) Compared to Olaparib and Cediranib or (Durvalumab) MEDI4736 and Cediranib or Standard of Care Chemotherapy in Women With Platinum-Resistant Recurrent Epithelial Ovarian Cancer, Primary Peritoneal or Fallopian Cancer Who Have Received Prior Bevacizumab	II
NCT04666740	A Phase II Trial to Evaluate the Safety and Antitumor Activity of Pembrolizumab and OLApaRib (POLAR) Maintenance for Patients With Metastatic Pancreatic Ductal Adenocarcinoma and Homologous Recombination Deficiency and/or Exceptional Treatment Response to Platinum-Based Therapy	II
NCT04633902	Phase II Study of Olaparib in Combination With Pembrolizumab in Patients With Advanced Melanoma With Homologous Recombination (HR) Pathway Gene Mutation	II
NCT03506997	PERSEUS1: Phase II Trial of the Immune Checkpoint Inhibitor Pembrolizumab for Patients Suffering From Metastatic Prostate Cancer	II
NCT04849364	A Phase II Circulating Tumor DNA Enriched, Genomically Directed Post-neoadjuvant Trial for Patients With Residual Triple Negative Breast Cancer (PERSEVERE)	II
NCT05002868	A Multi-center, Open-label, Phase I/Ib Study to Assess the Safety, Pharmacokinetics and Anti-tumor Activity of RP12146, a Poly (ADP-ribose) Polymerase (PARP) Inhibitor, in Patients With Locally Advanced or Metastatic Solid Tumors	I

BRCA2 aberration

NCT ID	Title	Phase
NCT03297606	Canadian Profiling and Targeted Agent Utilization Trial (CAPTUR): A Phase II Basket Trial	II
NCT04497116	Phase I/IIa Study of the Safety, Pharmacokinetics, Pharmacodynamics and Preliminary Clinical Activity of RP-3500 Alone or in Combination With Talazoparib in Advanced Solid Tumors With ATR Inhibitor Sensitizing Mutations (TRESR Study)	I/II
NCT02286687	Phase II Study of the PARP Inhibitor Talazoparib in Advanced Cancer Patients With Somatic Alterations in BRCA1/2, Mutations/Deletions in Other BRCA Pathway Genes and Germline Mutation in BRCA1/2 (Not Breast or Ovarian Cancer)	II
NCT04901702	A Randomized Phase I/II Study of Talazoparib or Temozolomide in Combination With Onivyde in Children With Recurrent Solid Malignancies and Ewing Sarcoma	I/II
NCT03155620	NCI-COG Pediatric MATCH (Molecular Analysis for Therapy Choice) Screening Protocol	II
NCT03233204	NCI-COG Pediatric MATCH (Molecular Analysis for Therapy Choice)- Phase 2 Subprotocol of Olaparib in Patients With Tumors Harboring Defects in DNA Damage Repair Genes	II
NCT04267939	An Open-label Phase Ib Study to Determine the Maximum Tolerated and/or Recommended Phase II Dose of the ATR Inhibitor BAY 1895344 in Combination With PARP Inhibitor Niraparib, in Patients With Recurrent Advanced Solid Tumors and Ovarian Cancer	I

Disclaimer: The data presented here is from a curated knowledgebase of publicly available information, but may not be exhaustive. The data version is 2021.11(003).

Clinical Trials Summary (continued)

BRCA2 aberration (continued)

NCT ID	Title	Phase
NCT04693468	Modular Phase IB Hypothesis-Testing, Biomarker-Driven, Talazoparib Combination Trial (TalaCom)	I
NCT04666740	A Phase II Trial to Evaluate the Safety and Antitumor Activity of Pembrolizumab and OLApaRib (POLAR) Maintenance for Patients With Metastatic Pancreatic Ductal Adenocarcinoma and Homologous Recombination Deficiency and/or Exceptional Treatment Response to Platinum-Based Therapy	II
NCT04633902	Phase II Study of Olaparib in Combination With Pembrolizumab in Patients With Advanced Melanoma With Homologous Recombination (HR) Pathway Gene Mutation	II
NCT03506997	PERSEUS1: Phase II Trial of the Immune Checkpoint Inhibitor Pembrolizumab for Patients Suffering From Metastatic Prostate Cancer	II
NCT04849364	A Phase II Circulating Tumor DNA Enriched, Genomically Directed Post-neoadjuvant Trial for Patients With Residual Triple Negative Breast Cancer (PERSEVERE)	II
NCT05002868	A Multi-center, Open-label, Phase I/Ib Study to Assess the Safety, Pharmacokinetics and Anti-tumor Activity of RP12146, a Poly (ADP-ribose) Polymerase (PARP) Inhibitor, in Patients With Locally Advanced or Metastatic Solid Tumors	I

Disclaimer: The data presented here is from a curated knowledgebase of publicly available information, but may not be exhaustive. The data version is 2021.11(003).

Sample Cancer Type: Unknown Primary Origin

Table of Contents

Table of Contents	Page
Relevant Therapy Details	**2**
Clinical Trials	**7**
Relevant Therapy Summary	**71**
Clinical Trials Summary	**76**

Report Highlights

6 Relevant Biomarkers
1 Therapies Available
57 Clinical Trials

Relevant Biomarkers

Genomic Alteration	Relevant Therapies (In this cancer type)	Relevant Therapies (In other cancer type)	Clinical Trials
CDK12 mutation<br>**Prognostic significance:** None<br>**Diagnostic significance:** None	None	**olaparib** [1]	57
CDK12 splice site mutation<br>**Prognostic significance:** None<br>**Diagnostic significance:** None	None	**olaparib** [1]	57
CDK12 truncating mutation<br>**Prognostic significance:** None<br>**Diagnostic significance:** None	None	**olaparib** [1]	57
CDK12 deletion<br>**Prognostic significance:** None<br>**Diagnostic significance:** None	None	None	19
CDK12 fusion<br>**Prognostic significance:** None<br>**Diagnostic significance:** None	None	None	11
CDK12 aberration<br>**Prognostic significance:** None<br>**Diagnostic significance:** None	None	None	10

Public data sources included in relevant therapies: FDA1, NCCN, **EMA**2, ESMO
Public data sources included in prognostic and diagnostic significance: NCCN, ESMO

Disclaimer: The data presented here is from a curated knowledgebase of publicly available information, but may not be exhaustive. The data version is 2021.11(003).The content of this report has not been evaluated or approved by the FDA or other regulatory agencies.

Relevant Therapy Details

Current FDA Information

● In this cancer type ○ In other cancer type ◑ In this cancer type and other cancer types

FDA information is current as of 2021-10-13. For the most up-to-date information, search www.fda.gov.

CDK12 mutation

○ olaparib

Cancer type: Castration-Resistant Prostate Cancer **Label as of:** 2021-03-11 **Variant class:** CDK12 mutation

Indications and usage:

LYNPARZA® is a poly (ADP-ribose) polymerase (PARP) inhibitor indicated:

Ovarian cancer

- for the maintenance treatment of adult patients with deleterious or suspected deleterious germline or somatic BRCA-mutated advanced epithelial ovarian, fallopian tube or primary peritoneal cancer who are in complete or partial response to first-line platinum-based chemotherapy. Select patients for therapy based on an FDA-approved companion diagnostic for LYNPARZA®.
- in combination with bevacizumab for the maintenance treatment of adult patients with advanced epithelial ovarian, fallopian tube or primary peritoneal cancer who are in complete or partial response to first-line platinum-based chemotherapy and whose cancer is associated with homologous recombination deficiency (HRD)-positive status defined by either:
  - a deleterious or suspected deleterious BRCA mutation, and/or
  - genomic instability.

Select patients for therapy based on an FDA-approved companion diagnostic for LYNPARZA®.

- for the maintenance treatment of adult patients with recurrent epithelial ovarian, fallopian tube or primary peritoneal cancer, who are in complete or partial response to platinum-based chemotherapy.
- for the treatment of adult patients with deleterious or suspected deleterious germline BRCA-mutated (gBRCAm) advanced ovarian cancer who have been treated with three or more prior lines of chemotherapy. Select patients for therapy based on an FDA-approved companion diagnostic for LYNPARZA®.

Breast cancer

- for the treatment of adult patients with deleterious or suspected deleterious gBRCAm, HER2-negative metastatic breast cancer who have been treated with chemotherapy in the neoadjuvant, adjuvant or metastatic setting. Patients with hormone receptor (HR)-positive breast cancer should have been treated with a prior endocrine therapy or be considered inappropriate for endocrine therapy. Select patients for therapy based on an FDA-approved companion diagnostic for LYNPARZA®.

Pancreatic cancer

- for the maintenance treatment of adult patients with deleterious or suspected deleterious gBRCAm metastatic pancreatic adenocarcinoma whose disease has not progressed on at least 16 weeks of a first-line platinum-based chemotherapy regimen. Select patients for therapy based on an FDA-approved companion diagnostic for LYNPARZA®.

Prostate cancer

- for the treatment of adult patients with deleterious or suspected deleterious germline or somatic homologous recombination repair (HRR) gene-mutated metastatic castration-resistant prostate cancer (mCRPC) who have progressed following prior treatment with enzalutamide or abiraterone. Select patients for therapy based on an FDA-approved companion diagnostic for LYNPARZA®.

Reference:

https://www.accessdata.fda.gov/drugsatfda_docs/label/2021/208558s019s020lbl.pdf

CDK12 splice site mutation

○ olaparib

Cancer type: Castration-Resistant Prostate Cancer **Label as of:** 2021-03-11 **Variant class:** CDK12 mutation

Indications and usage:

LYNPARZA® is a poly (ADP-ribose) polymerase (PARP) inhibitor indicated:

Ovarian cancer

- for the maintenance treatment of adult patients with deleterious or suspected deleterious germline or somatic BRCA-mutated advanced epithelial ovarian, fallopian tube or primary peritoneal cancer who are in complete or partial response to first-line platinum-based chemotherapy. Select patients for therapy based on an FDA-approved companion diagnostic for LYNPARZA®.
- in combination with bevacizumab for the maintenance treatment of adult patients with advanced epithelial ovarian, fallopian tube or primary peritoneal cancer who are in complete or partial response to first-line platinum-based chemotherapy and whose cancer is associated with homologous recombination deficiency (HRD)-positive status defined by either:
  - a deleterious or suspected deleterious BRCA mutation, and/or
  - genomic instability.

Select patients for therapy based on an FDA-approved companion diagnostic for LYNPARZA®.

- for the maintenance treatment of adult patients with recurrent epithelial ovarian, fallopian tube or primary peritoneal cancer, who are in complete or partial response to platinum-based chemotherapy.
- for the treatment of adult patients with deleterious or suspected deleterious germline BRCA-mutated (gBRCAm) advanced ovarian cancer who have been treated with three or more prior lines of chemotherapy. Select patients for therapy based on an FDA-approved companion diagnostic for LYNPARZA®.

Breast cancer

- for the treatment of adult patients with deleterious or suspected deleterious gBRCAm, HER2-negative metastatic breast cancer who have been treated with chemotherapy in the neoadjuvant, adjuvant or metastatic setting. Patients with hormone receptor (HR)-positive breast cancer should have been treated with a prior endocrine therapy or be considered inappropriate for endocrine therapy. Select patients for therapy based on an FDA-approved companion diagnostic for LYNPARZA®.

Pancreatic cancer

- for the maintenance treatment of adult patients with deleterious or suspected deleterious gBRCAm metastatic pancreatic adenocarcinoma whose disease has not progressed on at least 16 weeks of a first-line platinum-based chemotherapy regimen. Select patients for therapy based on an FDA-approved companion diagnostic for LYNPARZA®.

Prostate cancer

- for the treatment of adult patients with deleterious or suspected deleterious germline or somatic homologous recombination repair (HRR) gene-mutated metastatic castration-resistant prostate cancer (mCRPC) who have progressed following prior treatment with enzalutamide or abiraterone. Select patients for therapy based on an FDA-approved companion diagnostic for LYNPARZA®.

Reference:

https://www.accessdata.fda.gov/drugsatfda_docs/label/2021/208558s019s020lbl.pdf

Disclaimer: The data presented here is from a curated knowledgebase of publicly available information, but may not be exhaustive. The data version is 2021.11(003).

CDK12 truncating mutation

○ olaparib

Cancer type: Castration-Resistant Prostate Cancer **Label as of:** 2021-03-11 **Variant class:** CDK12 mutation

Indications and usage:

LYNPARZA® is a poly (ADP-ribose) polymerase (PARP) inhibitor indicated:

Ovarian cancer

- for the maintenance treatment of adult patients with deleterious or suspected deleterious germline or somatic BRCA-mutated advanced epithelial ovarian, fallopian tube or primary peritoneal cancer who are in complete or partial response to first-line platinum-based chemotherapy. Select patients for therapy based on an FDA-approved companion diagnostic for LYNPARZA®.
- in combination with bevacizumab for the maintenance treatment of adult patients with advanced epithelial ovarian, fallopian tube or primary peritoneal cancer who are in complete or partial response to first-line platinum-based chemotherapy and whose cancer is associated with homologous recombination deficiency (HRD)-positive status defined by either:
  - a deleterious or suspected deleterious BRCA mutation, and/or
  - genomic instability.

Select patients for therapy based on an FDA-approved companion diagnostic for LYNPARZA®.

- for the maintenance treatment of adult patients with recurrent epithelial ovarian, fallopian tube or primary peritoneal cancer, who are in complete or partial response to platinum-based chemotherapy.
- for the treatment of adult patients with deleterious or suspected deleterious germline BRCA-mutated (gBRCAm) advanced ovarian cancer who have been treated with three or more prior lines of chemotherapy. Select patients for therapy based on an FDA-approved companion diagnostic for LYNPARZA®.

Breast cancer

- for the treatment of adult patients with deleterious or suspected deleterious gBRCAm, HER2-negative metastatic breast cancer who have been treated with chemotherapy in the neoadjuvant, adjuvant or metastatic setting. Patients with hormone receptor (HR)-positive breast cancer should have been treated with a prior endocrine therapy or be considered inappropriate for endocrine therapy. Select patients for therapy based on an FDA-approved companion diagnostic for LYNPARZA®.

Pancreatic cancer

- for the maintenance treatment of adult patients with deleterious or suspected deleterious gBRCAm metastatic pancreatic adenocarcinoma whose disease has not progressed on at least 16 weeks of a first-line platinum-based chemotherapy regimen. Select patients for therapy based on an FDA-approved companion diagnostic for LYNPARZA®.

Prostate cancer

- for the treatment of adult patients with deleterious or suspected deleterious germline or somatic homologous recombination repair (HRR) gene-mutated metastatic castration-resistant prostate cancer (mCRPC) who have progressed following prior treatment with enzalutamide or abiraterone. Select patients for therapy based on an FDA-approved companion diagnostic for LYNPARZA®.

Reference:

https://www.accessdata.fda.gov/drugsatfda_docs/label/2021/208558s019s020lbl.pdf

Disclaimer: The data presented here is from a curated knowledgebase of publicly available information, but may not be exhaustive. The data version is 2021.11(003).

Current NCCN Information

● In this cancer type ○ In other cancer type ◐ In this cancer type and other cancer types

NCCN information is current as of 2021-10-01. For the most up-to-date information, search www.nccn.org.
For NCCN International Adaptations & Translations, search www.nccn.org/global/international_adaptations.aspx.

CDK12 mutation

○ olaparib

Cancer type: Castration-Resistant Prostate Cancer **Variant class:** CDK12 mutation

NCCN Recommendation category: 1

Population segment (Line of therapy):
- Adenocarcinoma; Metastatic (Subsequent therapy); Useful in certain circumstances

Reference: NCCN Guidelines® - NCCN-Prostate Cancer [Version 1.2022]

○ olaparib

Cancer type: Castration-Resistant Prostate Cancer **Variant class:** CDK12 mutation

NCCN Recommendation category: 2B

Population segment (Line of therapy):
- Adenocarcinoma; Visceral Metastases (Subsequent therapy); Useful in certain circumstances

Reference: NCCN Guidelines® - NCCN-Prostate Cancer [Version 1.2022]

CDK12 splice site mutation

○ olaparib

Cancer type: Castration-Resistant Prostate Cancer **Variant class:** CDK12 mutation

NCCN Recommendation category: 1

Population segment (Line of therapy):
- Adenocarcinoma; Metastatic (Subsequent therapy); Useful in certain circumstances

Reference: NCCN Guidelines® - NCCN-Prostate Cancer [Version 1.2022]

○ olaparib

Cancer type: Castration-Resistant Prostate Cancer **Variant class:** CDK12 mutation

NCCN Recommendation category: 2B

Population segment (Line of therapy):
- Adenocarcinoma; Visceral Metastases (Subsequent therapy); Useful in certain circumstances

Reference: NCCN Guidelines® - NCCN-Prostate Cancer [Version 1.2022]

Disclaimer: The data presented here is from a curated knowledgebase of publicly available information, but may not be exhaustive. The data version is 2021.11(003).

CDK12 truncating mutation

○ olaparib

Cancer type: Castration-Resistant Prostate Cancer **Variant class:** CDK12 mutation

NCCN Recommendation category: 1

Population segment (Line of therapy):

- Adenocarcinoma; Metastatic (Subsequent therapy); Useful in certain circumstances

Reference: NCCN Guidelines® - NCCN-Prostate Cancer [Version 1.2022]

○ olaparib

Cancer type: Castration-Resistant Prostate Cancer **Variant class:** CDK12 mutation

NCCN Recommendation category: 2B

Population segment (Line of therapy):

- Adenocarcinoma; Visceral Metastases (Subsequent therapy); Useful in certain circumstances

Reference: NCCN Guidelines® - NCCN-Prostate Cancer [Version 1.2022]

Disclaimer: The data presented here is from a curated knowledgebase of publicly available information, but may not be exhaustive. The data version is 2021.11(003).

Current Clinical Trials Information

Clinical Trials information is current as of 2021-10-01. For the most up-to-date information regarding a particular trial, search www.clinicaltrials.gov by NCT ID or search local clinical trials authority website by local identifier listed in 'Other identifiers'.

CDK12 mutation + CDK12 splice site mutation + CDK12 truncating mutation + CDK12 deletion

NCT04276376
A Multicenter, Open Label, Phase II Basket Trial Exploring the Efficacy And Safety of The Combination of Rucaparib (PARP Inhibitor) And Atezolizumab (Anti-PD-L1 Antibody) In Patients With DNA Repair-Deficient or Platinum-Sensitive Solid Tumors

Cancer type: Bladder Urothelial Carcinoma, Non-Small Cell Lung Cancer, Prostate Cancer, Unspecified Solid Tumor

Variant classes: CDK12 deletion & CDK12 mutation

Other identifiers: 2018/2727, ARIANES, EudraCT Number: 2018-001744-62

Population segments: Second line, Stage III, Stage IV

Exclusion criteria variant classes: ATM deletion (Breast Cancer, Ovarian Cancer), ATM mutation (Breast Cancer, Ovarian Cancer), BARD1 deletion (Breast Cancer, Ovarian Cancer), BARD1 mutation (Breast Cancer, Ovarian Cancer), BRCA1 germline deletion (Breast Cancer, Ovarian Cancer), BRCA1 germline mutation (Breast Cancer, Ovarian Cancer), BRCA2 germline deletion (Breast Cancer, Ovarian Cancer), BRCA2 germline mutation (Breast Cancer, Ovarian Cancer), BRIP1 deletion (Breast Cancer, Ovarian Cancer), BRIP1 mutation (Breast Cancer, Ovarian Cancer), CDK12 deletion (Breast Cancer, Ovarian Cancer), CDK12 mutation (Breast Cancer, Ovarian Cancer), CHEK2 deletion (Breast Cancer, Ovarian Cancer), CHEK2 mutation (Breast Cancer, Ovarian Cancer), FANCA deletion (Breast Cancer, Ovarian Cancer), FANCA mutation (Breast Cancer, Ovarian Cancer), NBN deletion (Breast Cancer, Ovarian Cancer), NBN mutation (Breast Cancer, Ovarian Cancer), PALB2 deletion (Breast Cancer, Ovarian Cancer), PALB2 mutation (Breast Cancer, Ovarian Cancer), RAD51 deletion (Breast Cancer, Ovarian Cancer), RAD51 mutation (Breast Cancer, Ovarian Cancer), RAD51C deletion (Breast Cancer, Ovarian Cancer), RAD51C mutation (Breast Cancer, Ovarian Cancer), RAD51D deletion (Breast Cancer, Ovarian Cancer), RAD51D mutation (Breast Cancer, Ovarian Cancer), RAD54L deletion (Breast Cancer, Ovarian Cancer), RAD54L mutation (Breast Cancer, Ovarian Cancer)

Phase: II

Therapies: rucaparib, atezolizumab

Location: France

NCT04126070
A Phase II Multicohort Study of Nivolumab in Combination With Docetaxel and Androgen Deprivation Therapy in Metastatic Hormone Sensitive Prostate Cancer Patients With DNA Damage Repair Defects or Inflamed Tumors

Cancer type: Prostate Cancer

Variant classes: DNA repair deletion & DNA repair mutation

Other identifiers: 19-384, NCI-2020-02149

Population segments: First line, Stage IV

Phase: II

Therapies: nivolumab, chemotherapy, hormone therapy

Location: United States

US State: MA

Contact: Dr. Xiao X. Wei [617-632-4524; xiaox_wei@dfci.harvard.edu]

Disclaimer: The data presented here is from a curated knowledgebase of publicly available information, but may not be exhaustive. The data version is 2021.11(003).

CDK12 mutation

NCT02693535
Targeted Agent and Profiling Utilization Registry (TAPUR) Study.

Cancer type: Unspecified Solid Tumor

Variant class: CDK12 mutation

Other identifiers: 20170529, NCI-2017-00510, Pro00014171, TAPUR

Population segments: Aggressive, BRAF, Diffuse large B-cell lymphoma (DLBCL), Extranodal marginal zone B-cell lymphoma (MALT), FGFR, First line, Follicular lymphoma (FL), Fourth line or greater, Indolent, Lymphoblastic lymphoma (LBL), Mantle cell lymphoma (MCL), Other subtype, Second line, Small lymphocytic lymphoma (SLL), Stage III, Stage IV, Third line, Waldenstrom's macroglobulinemia (WM)

Phase: II

Therapy: atezolizumab + talazoparib

Location: United States

US States: AL, AZ, CA, FL, GA, HI, IL, IN, ME, MI, NC, ND, NE, NH, OH, OR, PA, SD, TX, UT, VA, WA

Contact: Pam Mangat [pam.mangat@asco.org]

NCT03742895
A Phase II Study of Olaparib Monotherapy in Participants With Previously Treated, Homologous Recombination Repair Mutation (HRRm) or Homologous Recombination Deficiency (HRD) Positive Advanced Cancer

Cancer type: Unspecified Solid Tumor

Variant class: CDK12 mutation

Other identifiers: (MK-7339-002 / LYNK-002), 19-139, 194694, 2018-01891, 7339-002, 7339-002-00, EudraCT Number: 2018-003007-19, IRAS ID: 255158, JapicCTI-194694, LYNK002, LYNK-002, MK7339-002, MK-7339-002, MK7339-002-00, NCI-2018-03519, PER-046-18, SNCTP000003157

Population segments: BRCA, Fourth line or greater, Second line, Stage III, Stage IV, Third line

Exclusion criteria variant classes: BRCA germline mutation (Breast Cancer, Ovarian Cancer), BRCA mutation (Ovarian Cancer)

Phase: II

Therapy: olaparib

Locations: Argentina, Australia, Canada, Colombia, Denmark, France, Guatemala, Ireland, Israel, Italy, Mexico, Peru, Republic of Korea, Romania, Russian Federation, Spain, Switzerland, Turkey, United Kingdom, United States

US States: AZ, CA, CO, GA, KY, MA, MD, MI, NE, NJ, NY, SD, UT, WA

Contact: Toll Free Number [888-577-8839; Trialsites@merck.com]

NCT03967938
Efficacy of Olaparib in Advanced Cancers Occurring in Patients With Germline Mutations or Somatic Tumor Mutations in Homologous Recombination Genes

Cancer type: Unspecified Solid Tumor

Variant class: CDK12 mutation

Other identifiers: 1-2018 BSMO, 1-2018BSMO, EudraCT Number: 2018-002966-37, Precision 2 - Olaparib

Population segments: Line of therapy N/A, Stage III, Stage IV

Exclusion criteria variant classes: BRCA1 germline mutation (Breast Cancer, Pancreatic Cancer, Prostate Cancer), BRCA2 germline mutation (Breast Cancer, Pancreatic Cancer, Prostate Cancer)

Phase: II

Therapy: olaparib

Location: Belgium

Disclaimer: The data presented here is from a curated knowledgebase of publicly available information, but may not be exhaustive. The data version is 2021.11(003).

CDK12 mutation (continued)

NCT04550494

A Pharmacodynamics-Driven Trial of Talazoparib, an Oral PARP Inhibitor, in Patients With Advanced Solid Tumors and Aberrations in Genes Involved in DNA Damage Response

Cancer type: Unspecified Solid Tumor

Variant class: CDK12 mutation

Other identifiers: 000246, 000264-C, 10000264, 10371, NCI-2020-06906

Population segments: BRCA, Fourth line or greater, HER2 positive, Hormone refractory, Second line, Stage II, Stage III, Stage IV, Third line

Phase: II

Therapy: talazoparib

Location: United States

US State: MD

Contact: Multiple contacts: See www.clinicaltrials.gov for complete list of contacts.

NCT03842228

A Phase Ib Biomarker-Driven Combination Trial of Copanlisib, Olaparib, and Durvalumab (MEDI4736) in Patients With Advanced Solid Tumors

Cancer type: Unspecified Solid Tumor

Variant class: CDK12 mutation

Other identifiers: 10217, 19-628, NCI10217, NCI-2019-00601

Population segments: Line of therapy N/A, Stage III, Stage IV

Phase: I

Therapies: copanlisib, olaparib, durvalumab

Location: United States

US States: MA, TX

Contact: Multiple contacts: See www.clinicaltrials.gov for complete list of contacts.

NCT04586335

Open Label, Phase Ib Study to Evaluate the Safety, Tolerability, Pharmacokinetics and Clinical Activity of CYH33, an Oral a-specific PI3K Inhibitor in Combination With Olaparib, an Oral PARP Inhibitor in Patients With Advanced Solid Tumors.

Cancer type: Breast Cancer, Endometrial Carcinoma, Ovarian Cancer, Prostate Cancer, Unspecified Solid Tumor

Variant class: CDK12 mutation

Other identifiers: CTR20202339, CYH33-G102

Population segments: BRCA, Second line, Stage III, Stage IV

Phase: I

Therapies: HH-CYH33, olaparib

Locations: Australia, United States

US State: TX

Contact: Dr. Jason Sudia [908-380-1329; jason.sudia@haihepharma.com]

Disclaimer: The data presented here is from a curated knowledgebase of publicly available information, but may not be exhaustive. The data version is 2021.11(003).

CDK12 mutation (continued)

NCT02029001
A Two-period, Multicenter, Randomized, Open-label, Phase II Study Evaluating the Clinical Benefit of a Maintenance Treatment Targeting Tumor Molecular Alterations in Patients with Progressive Locally-advanced or Metastatic Solid Tumors.

Cancer type: Unspecified Solid Tumor

Variant class: HRR mutation

Other identifiers: ET12-081, EudraCT Number: 2012-004510-34, MOST, ProfiLER

Population segments: Maintenance/Consolidation, Second line, Stage III, Stage IV, Third line

Exclusion criteria variant classes: ATM mutation (Gastric Cancer), BRCA1 germline mutation (Gastric Cancer), BRCA1 mutation (Gastric Cancer), BRCA2 germline mutation (Gastric Cancer), BRCA2 mutation (Gastric Cancer), HRR mutation (Gastric Cancer), PALB2 mutation (Gastric Cancer), RAD51 mutation (Gastric Cancer)

Phase: II

Therapy: olaparib

Location: France

NCT04123366
A Phase II Study of Olaparib in Combination With Pembrolizumab in Participants With Previously Treated, Homologous Recombination Repair Mutation (HRRm) and/or Homologous Recombination Deficiency (HRD)-Positive Advanced Cancer

Cancer type: Unspecified Solid Tumor

Variant class: HRR mutation

Other identifiers: 7339-007, EudraCT Number: 2019-001745-40, jRCT2011200025, KEYLYNK-007, MK-7339-007, NCI-2019-08758, PER-029-19

Population segments: BRCA, Second line, Stage III, Stage IV

Exclusion criteria variant classes: BRCA germline mutation (Breast Cancer, Ovarian Cancer), BRCA mutation (Breast Cancer, Ovarian Cancer)

Phase: II

Therapies: olaparib, pembrolizumab

Locations: Argentina, Australia, Canada, Colombia, France, Germany, Guatemala, Israel, Italy, Japan, Latvia, Mexico, Peru, Poland, Puerto Rico, Republic of Korea, Romania, South Africa, Spain, Sweden, Turkey, Ukraine, United States

US States: AL, AZ, CA, CO, FL, GA, KY, NJ, NY, OH, TX, UT, VA

Contact: Toll Free Number [888-577-8839; Trialsites@merck.com]

NCT02401347
A Phase II Clinical Trial of the PARP Inhibitor Talazoparib in BRCA1 and BRCA2 Wild Type Patients With Advanced Triple Negative Breast Cancer and Homologous Recombination Deficiency or Advanced HER2 Negative Breast Cancer or Other Solid Tumors With a Mutation in Homologous Recombination Pathway Genes Talazoparib Beyond BRCA (TBB) Trial

Cancer type: Breast Cancer, Unspecified Solid Tumor

Variant class: HRR mutation

Other inclusion criteria: ERBB2 negative, Hormone receptor positive

Other identifiers: BRS0050, NCI-2015-00036, TBB

Population segments: Estrogen receptor positive, HER2 negative, Progesterone receptor positive, Second line, Stage III, Stage IV, Triple receptor negative

Exclusion criteria variant classes: BRCA1 germline mutation (Breast Cancer, Triple Negative Breast Cancer, Unspecified Solid Tumor), BRCA1 mutation (Breast Cancer, Triple Negative Breast Cancer, Unspecified Solid Tumor), BRCA2 germline mutation (Breast Cancer, Triple Negative Breast Cancer, Unspecified Solid Tumor), BRCA2 mutation (Breast Cancer, Triple Negative Breast Cancer, Unspecified Solid Tumor), ERBB2 positive (Breast Cancer)

Phase: II

Therapy: talazoparib

Location: United States

US State: CA

Contact: Amanda Selin [650-721-6977; aselin@stanford.edu]

Disclaimer: The data presented here is from a curated knowledgebase of publicly available information, but may not be exhaustive. The data version is 2021.11(003).

CDK12 mutation (continued)

NCT04497116

Phase I/IIa Study of the Safety, Pharmacokinetics, Pharmacodynamics and Preliminary Clinical Activity of RP-3500 Alone or in Combination With Talazoparib in Advanced Solid Tumors With ATR Inhibitor Sensitizing Mutations (TRESR Study)

Cancer type: Unspecified Solid Tumor

Variant class: CDK12 aberration

Other identifiers: 20-521, EudraCT Number: 2020-000301-87, IRAS ID: 286609, NCI-2020-06869, RP-3500-01, TRESR, TRESR Study

Population segments: Second line, Stage III, Stage IV

Phase: I/II

Therapies: RP-3500, talazoparib

Locations: Canada, Denmark, United Kingdom, United States

US States: MA, NC, NY, RI, TN, TX

Contact: Dr. Peter Manley [857-322-5553; pmanley@reparerx.com]

NCT03415659

A Phase I, Open-label, Single-center, Single/Multiple-dose, Dose-escalation/ Dose-expansion Clinical Study on Tolerance and Pharmacokinetics of HWH340 Tablet in Patients With Advanced Solid Tumors

Cancer type: Unspecified Solid Tumor

Variant class: HRR mutation

Other inclusion criteria: BRCA1 germline mutation negative, BRCA1 mutation negative, BRCA2 germline mutation negative, BRCA2 mutation negative

Other identifiers: CTR20171649, HWH340-RFPA 20170821, RFPA 20170821

Population segments: BRCA, Fourth line or greater, HER2 negative, Second line, Stage III, Stage IV, Third line

Phase: I

Therapy: HWH-340

Location: China

NCT03767075

Basket of Baskets: A Modular, Open-label, Phase II, Multicentre Study To Evaluate Targeted Agents in Molecularly Selected Populations With Advanced Solid Tumours

Cancer type: Unspecified Solid Tumor

Variant class: DNA repair mutation

Other identifiers: (Basket of Baskets) (BoB), EudraCT number: 2017-005108-89, MO39164, NL65937.031.18, VHIO17002

Population segments: Second line, Stage III, Stage IV

Phase: II

Therapy: atezolizumab

Locations: France, Germany, Netherlands, Spain, Sweden, United Kingdom

---

NCT04905914

A Phase I/IIa, Open-Label, Safety, Pharmacokinetic, And Preliminary Efficacy Study Of Oral ATRN-119 In Patients With Advanced Solid Tumors

Cancer type: Unspecified Solid Tumor

Variant class: DNA repair mutation

Other identifier: AR-276-01

Population segments: Line of therapy N/A, Stage III, Stage IV

Phase: I/II

Therapy: ATRN-119

Location: United States

US State: MA

Contact: Robert Hasson [619-540-6253; rhasson@pacificlinkconsulting.com]

Disclaimer: The data presented here is from a curated knowledgebase of publicly available information, but may not be exhaustive. The data version is 2021.11(003).

CDK12 mutation (continued)

NCT04901702
A Randomized Phase I/II Study of Talazoparib or Temozolomide in Combination With Onivyde in Children With Recurrent Solid Malignancies and Ewing Sarcoma

Cancer type: Unspecified Solid Tumor

Variant class: HRR pathway

Other identifiers: EudraCT Number: 2021-003570-31, ONITT

Population segments: Second line, Stage IV

Exclusion criteria variant classes: HRR germline pathway (Central Nervous System Neoplasm, Ewing Sarcoma), HRR pathway (Central Nervous System Neoplasm, Ewing Sarcoma)

Phase: I/II

Therapies: talazoparib, chemotherapy

Locations: Canada, United States

US State: TN

Contact: Dr. Sara Federico [866-278-5833; referralinfo@stjude.org]

NCT03155620
NCI-COG Pediatric MATCH (Molecular Analysis for Therapy Choice) Screening Protocol

Cancer type: Unspecified Solid Tumor

Variant class: DNA repair pathway

Other identifiers: 17-729, 2017-0625, 20170841, APEC1621SC, COGAPEC1621, COGAPEC1621SC, N 55017 SC, NCI-2017-01251, NCI-COG Pediatric MATCH, Pediatric MATCH

Population segments: ALK, Aggressive, BRAF, FGFR, HRAS, Indolent, KRAS, NRAS, Pediatric or Adolescent, Second line, Stage III, Stage IV

Phase: II

Therapy: olaparib

Locations: Puerto Rico, United States

US States: AK, AL, AR, AZ, CA, CO, CT, DC, DE, FL, GA, HI, IA, ID, IL, IN, KY, LA, MA, MD, ME, MI, MN, MO, MS, NC, NE, NH, NJ, NV, NY, OH, OK, OR, PA, RI, SC, SD, TN, TX, UT, VA, VT, WA, WI

Contact: Multiple contacts: See www.clinicaltrials.gov for complete list of contacts.

NCT03233204
NCI-COG Pediatric MATCH (Molecular Analysis for Therapy Choice)- Phase 2 Subprotocol of Olaparib in Patients With Tumors Harboring Defects in DNA Damage Repair Genes

Cancer type: Unspecified Solid Tumor

Variant class: DNA repair pathway

Other identifiers: 20170841, APEC1621H, NCI-2017-00766

Population segments: Aggressive, Indolent, Pediatric or Adolescent, Second line, Stage III, Stage IV

Phase: II

Therapy: olaparib

Locations: Puerto Rico, United States

US States: AK, AL, AR, AZ, CA, CO, DC, DE, FL, GA, IA, ID, IL, IN, KY, LA, MD, ME, MI, MN, MO, MS, NC, ND, NE, NJ, NY, OH, OK, OR, PA, SC, SD, TN, TX, UT, VA, VT, WA, WI

Contact: Multiple contacts: See www.clinicaltrials.gov for complete list of contacts.

Disclaimer: The data presented here is from a curated knowledgebase of publicly available information, but may not be exhaustive. The data version is 2021.11(003).

CDK12 mutation (continued)

NCT04267939
An Open-label Phase Ib Study to Determine the Maximum Tolerated and/or Recommended Phase II Dose of the ATR Inhibitor BAY 1895344 in Combination With PARP Inhibitor Niraparib, in Patients With Recurrent Advanced Solid Tumors and Ovarian Cancer

Cancer type: Ovarian Cancer, Unspecified Solid Tumor

Variant class: DNA repair pathway

Other identifiers: 18595, 19-810, 2019-0471, 20-222, EudraCT Number: 2018-003930-34, NCI-2020-01405

Population segments: Maintenance/Consolidation, Second line, Stage III, Stage IV

Exclusion criteria variant class: DNA repair pathway (Prostate Cancer)

Phase: I

Therapies: BAY-1895344, niraparib

Location: United States

US States: MA, NY, TX

Contact: Bayer Clinical Trials Contact [888-842-2937; clinical-trials-contact@bayer.com]

NCT04693468
Modular Phase IB Hypothesis-Testing, Biomarker-Driven, Talazoparib Combination Trial (TalaCom)

Cancer type: Unspecified Solid Tumor

Variant class: DNA repair pathway

Other identifiers: 2020-0436, NCI-2020-06041, TalaCom

Population segments: Hormone refractory, Second line, Stage III, Stage IV

Phase: I

Therapies: talazoparib, palbociclib, axitinib, crizotinib

Location: United States

US State: TX

Contact: Timothy A. Yap [713-563-1784; tyap@mdanderson.org]

NCT04751929
A Phase II Multi-Center Trial of Abemaciclib With or Without Atezolizumab in Metastatic Castration Resistant Prostate Cancer

Cancer type: Castration-Resistant Prostate Cancer

Variant class: CDK12 mutation

Other identifier: 20-701

Population segments: Hormone refractory, Second line, Stage IV

Phase: II

Therapies: abemaciclib, atezolizumab

Location: United States

US State: MA

Contact: Dr. Atish Choudhury [617-632-6328; achoudhury@partners.org]

Disclaimer: The data presented here is from a curated knowledgebase of publicly available information, but may not be exhaustive. The data version is 2021.11(003).

CDK12 mutation (continued)

NCT03810105

A Phase II Study of Olaparib and Durvalumab in Men With Castration Sensitive Biochemically Recurrent Non-Metastatic Prostate Cancer Harboring Mutations in DNA Damage Repair

Cancer type: Prostate Cancer

Variant class: CDK12 mutation

Other identifiers: 18-480, NCI-2019-01627

Population segments: Adjuvant, Stage I, Stage II, Stage III

Phase: II

Therapies: durvalumab, olaparib

Location: United States

US States: CA, IL, MI, NJ, NY

Contact: Dr. Karen Autio [646-422-4632; autiok@mskcc.org]

NCT04336943

Durvalumab (MEDI4736) and Olaparib (AZD2281) for Treatment of Biochemically Recurrent Prostate Cancer in Men Predicted to Have a High Neoantigen Load: A Pilot Study

Cancer type: Prostate Adenocarcinoma

Variant class: CDK12 mutation

Other identifiers: 10509, NCI-2020-01860, RG1007001

Population segments: Line of therapy N/A, Stage I, Stage II, Stage III

Phase: II

Therapies: durvalumab, olaparib

Location: United States

US State: WA

Contact: Michael Schweizer [206-606-6252; schweize@uw.edu]

NCT04053322

An International Multicenter Phase II Trial of Durvalumab (MEDI4736) Plus OLAparib Plus Fulvestrant in Metastatic or Locally Advanced ER-positive, HER2-negative Breast Cancer Patients Selected Using Criteria That Predict Sensitivity to Olaparib

Cancer type: Breast Cancer

Variant class: CDK12 mutation

Other inclusion criteria: ERBB2 negative, ER positive

Other identifiers: CADUSEIME08, DOLAF, EudraCT Number: 2018-003832-57, UC-0140/1812

Population segments: BRCA, Estrogen receptor positive, FGFR, HER2 negative, Progesterone receptor positive, Second line, Stage III, Stage IV

Phase: II

Therapies: durvalumab, olaparib, hormone therapy

Location: France

Disclaimer: The data presented here is from a curated knowledgebase of publicly available information, but may not be exhaustive. The data version is 2021.11(003).

CDK12 mutation (continued)

NCT05011383
High-dose Testosterone in Men With Metastatic Castration-resistant Prostate Cancer and ATM or CDK12 Deficiency

Cancer type: Castration-Resistant Prostate Cancer

Variant class: CDK12 mutation

Other identifiers: SPLP-003-20F, VA-BAT

Population segments: Hormone refractory, Second line, Stage IV

Phase: II

Therapy: hormone therapy

Location: United States

US State: WA

Contact: Dr. Robert B. Montgomery [206-277-6878; Robert.Montgomery@va.gov]

NCT03553004
Niraparib in Metastatic Pancreatic Cancer After Previous Chemotherapy (NIRA-PANC): A Phase II Trial

Cancer type: Pancreatic Cancer

Variant class: CDK12 mutation

Other identifiers: IIT-2017-NIRA-PANC, NIRA-PANC, STUDY00141862

Population segments: BRCA, Second line, Stage IV, Third line

Phase: II

Therapy: niraparib

Location: United States

US States: KS, MO

Contact: Clinical Trials Nurse Navigator [913-588-3671; navigation@kumc.edu]

NCT03840967
A Phase II Study Evaluating Safety and Efficacy of Niraparib in Patients With Previously Treated Homologous Recombination (HR) Defective or Loss of Heterozygosity (LOH) High Metastatic Esophageal/Gastroesophageal Junction/Proximal Gastric Adenocarcinoma

Cancer type: Esophageal Cancer, Gastric Cancer, Gastroesophageal Junction Adenocarcinoma

Variant class: CDK12 mutation

Other identifiers: 20166, 71618, HCRN ESO17-325, NCI-2019-07395

Population segments: Second line, Stage III, Stage IV

Phase: II

Therapy: niraparib

Location: United States

US States: FL, IN, MI, NY

Contact: Dr. Shadia Jalal [317-274-3658; sjalal@iu.edu]

NCT04030559
Phase II Trial of PARP Inhibitor Niraparib for Men With High Risk Prostate Cancer and DNA Damage Response Defects

Cancer type: Prostate Cancer

Variant class: CDK12 mutation

Other identifiers: 1412676, NCI-2019-04365, UCDCC#279

Population segments: First line, Neoadjuvant, Stage I, Stage II, Stage IV

Phase: II

Therapy: niraparib

Location: United States

US State: CA

Contact: Marc Dall'Era [916-734-3771; mdallera@ucdavis.edu]

Disclaimer: The data presented here is from a curated knowledgebase of publicly available information, but may not be exhaustive. The data version is 2021.11(003).

CDK12 mutation (continued)

NCT04019964
Nivolumab as a Non-Castrating Therapy for MMR-deficient and CDK12- Altered Prostate Cancer With PSA Recurrence After Local Therapy

Cancer type: Prostate Cancer

Variant class: CDK12 mutation

Other identifiers: CA209-76M, CRMS-71513, J1933, NCI-2020-03804

Population segments: (N/A), Hormone refractory, Second line

Phase: II

Therapy: nivolumab

Location: United States

US State: MD

Contact: Rana Sullivan [410-614-6337; tomalra@jhmi.edu]

NCT03012321
BRCAAway: A Randomized Phase II Trial of Abiraterone, Olaparib, or Abiraterone + Olaparib in Patients With Metastatic Castration-Resistant Prostate Cancer With DNA Repair Defects

Cancer type: Castration-Resistant Prostate Cancer

Variant class: CDK12 mutation

Other identifiers: 19137, BRCAAway, NCI-2016-01834, NU 16U05, NU_16U05, PCCTC #: c16-168, PCCTC c16-168, STU00203960

Population segments: First line, Hormone refractory, Stage IV

Phase: II

Therapy: olaparib

Location: United States

US States: CA, FL, IL, IN, MD, MI, MN, MO, NC, NY, UT, VA

Contact: Study Coordinator [312-695-1301; cancertrials@northwestern.edu]

NCT03786796
Phase II Study of Olaparib in Metastatic Renal Cell Carcinoma Patients Harboring a BAP-1 or Other DNA Repair Gene Mutations (ORCHID)

Cancer type: Kidney Cancer

Variant class: CDK12 mutation

Other identifiers: CRMS-70750, J18166, NCI-2019-06309, ORCHID

Population segments: Fourth line or greater, Second line, Stage IV, Third line

Phase: II

Therapy: olaparib

Location: United States

US State: MD

Contact: Irina Rifkind [410-502-2043; irifkin1@jhmi.edu]

NCT04515836
Phase II Trial of Olaparib in Homologous Recombination Deficient (HRD) Malignant Mesothelioma

Cancer type: Mesothelioma

Variant class: CDK12 mutation

Population segments: (N/A), Second line

Phase: II

Therapy: olaparib

Location: United States

US State: IL

Contact: Dr. Hedy L. Kindler [773-702-0360; hkindler@medicine.bsd.uchicago.edu]

Disclaimer: The data presented here is from a curated knowledgebase of publicly available information, but may not be exhaustive. The data version is 2021.11(003).

CDK12 mutation (continued)

NCT04104893
A Phase II Study Of A Checkpoint Inhibitor In Men With Progressive Metastatic Castrate Resistant Prostate Cancer Characterized By A Mismatch Repair Deficiency Or Biallelic CDK12 Inactivation

Cancer type: Castration-Resistant Prostate Cancer

Variant class: CDK12 mutation

Other identifiers: CHOMP, CSDR-003-18F, NCI-2020-02215

Population segments: First line, Hormone refractory, Stage IV

Phase: II

Therapy: pembrolizumab

Location: United States

US States: CA, FL, IL, MI, NC, NY, WA

Contact: Dr. Matthew B. Rettig [310-478-3711; matthew.rettig@va.gov]

NCT03533946
A Phase II Study of Rucaparib Monotherapy in Nonmetastatic, Hormone-Sensitive Prostate Cancer Demonstrating "BRCAness" Genotype (ROAR)

Cancer type: Prostate Cancer

Variant class: CDK12 mutation

Other identifiers: HCI111833, NCI-2018-01754, ROAR

Population segments: First line, Stage I, Stage II, Stage III

Phase: II

Therapy: rucaparib

Location: United States

US State: UT

Contact: Jill Broghammer [801-213-6232; jill.broghammer@hci.utah.edu]

NCT04892693
Talazoparib in Advanced Breast Cancer Patients With Homologous Recombinant Deficiency: A Phase II Clinical and Exploratory Biomarker Study of Talazoparib

Cancer type: Breast Cancer

Variant class: CDK12 mutation

Other identifier: H-2009-079-1157

Population segments: BRCA, Line of therapy N/A, Stage III, Stage IV

Phase: II

Therapy: talazoparib

Location: Republic of Korea

NCT04740190
Combination Talazoparib - Carboplatin for Recurrent High-grade Glioma With DNA Damage Repair Deficiency (DDRd)

Cancer type: Anaplastic Astrocytoma, Anaplastic Oligoastrocytoma, Anaplastic Oligodendroglioma, Anaplastic Pleomorphic Xanthoastrocytoma, Diffuse Midline Glioma, Glioblastoma

Variant class: CDK12 mutation

Other identifiers: TAC-GReD, UW-20396

Population segments: (N/A), BRCA, Second line

Phase: II

Therapies: talazoparib, chemotherapy

Location: Hong Kong

Disclaimer: The data presented here is from a curated knowledgebase of publicly available information, but may not be exhaustive. The data version is 2021.11(003).

CDK12 mutation (continued)

NCT04253262
A Phase Ib/II Study of Copanlisib Combined With Rucaparib in Patients With Metastatic Castration-resistant Prostate Cancer

Cancer type: Castration-Resistant Prostate Cancer

Variant class: CDK12 mutation

Other identifiers: BrUOG 360, BrUOG360

Population segments: Hormone refractory, Second line, Stage IV

Phase: I/II

Therapies: copanlisib, rucaparib

Location: United States

US State: RI

Contact: BrUOG [401-863-3000; BrUOG@brown.edu]

NCT04169841
Precision Medicine Phase II Study Evaluating the Efficacy of a Double Immunotherapy by Durvalumab and Tremelimumab Combined With Olaparib in Patients With Solid Cancers and Carriers of Homologous Recombination Repair Genes Mutation in Response or Stable After Olaparib Treatment

Cancer type: Breast Cancer, Endometrial Carcinoma, Head and Neck Cancer, Kidney Cancer, Non-Small Cell Lung Cancer, Ovarian Cancer, Pancreatic Cancer, Prostate Cancer

Variant class: HRR mutation

Other identifier: GUIDE2REPAIR

Population segments: Fourth line or greater, Hormone refractory, Maintenance/ Consolidation, Second line, Stage III, Stage IV, Third line

Exclusion criteria variant classes: ALK fusion (Lung Cancer), BRAF mutation (Lung Cancer), EGFR mutation (Lung Cancer), ROS1 mutation (Lung Cancer)

Phase: II

Therapies: durvalumab, tremelimumab, olaparib

Location: France

NCT03925350
A Phase II Study of Niraparib in Patients With Advanced Melanoma With Genetic Homologous Recombination (HR) Mutation / Alteration

Cancer type: Melanoma

Variant class: HRR mutation

Other identifier: CPMC17-MEL01

Population segments: Second line, Stage III, Stage IV

Phase: II

Therapy: niraparib

Location: United States

US State: CA

Contact: Laurel Brechtel [415-600-1654; BrechtLA@sutterhealth.org]

Disclaimer: The data presented here is from a curated knowledgebase of publicly available information, but may not be exhaustive. The data version is 2021.11(003).

CDK12 mutation (continued)

NCT04940637
A Phase II, Open-Label, Single Arm, Prospective, Multicenter Study of Niraparib Plus Dostarlimab in Patients With Advanced NSCLC and/or MPM, and Positive for PD-L1 Expression and Germline or Somatic Mutations in the HRR Genes

Cancer type: Mesothelioma, Non-Small Cell Lung Cancer

Variant class: HRR mutation

Other inclusion criteria: ALK fusion negative, CD274 expression, EGFRi sensitizing mutation negative, ROS1 fusion negative or CD274 expression

Other identifiers: EudraCT Number: 2020-000109-10, UNITO-001, UNITO-001/2020

Population segments: Second line, Stage III, Stage IV

Exclusion criteria variant classes: ALK fusion (Non-Small Cell Lung Cancer), EGFRi sensitizing mutation (Non-Small Cell Lung Cancer), ROS1 fusion (Non-Small Cell Lung Cancer)

Phase: II

Therapies: niraparib, dostarlimab

Location: Italy

NCT04042831
A Phase II Study of Olaparib in Patients With Advanced Biliary Tract Cancer With Aberrant DNA Repair Gene Mutations

Cancer type: Biliary Tract Carcinoma

Variant class: HRR mutation

Other identifiers: ACCRU-ICRN-1702, NCI-2019-04434

Population segments: Second line, Stage IV

Phase: II

Therapy: olaparib

Location: United States

US States: AZ, MN, NY, TX

Contact: Multiple contacts: See www.clinicaltrials.gov for complete list of contacts.

NCT04939662
Phase II, Single-arm Study of Olaparib and Bevacizumab Combination Therapy in Relapsed Small Cell Lung Cancer Subjects With DNA Damage Response and the Repair Pathway Alteration, ATM Deficiency, SLFN11 Positive, or POU2F3 Positive

Cancer type: Small Cell Lung Cancer

Variant class: HRR mutation

Other identifier: SUKSES-B2

Population segments: (N/A), BRCA, Pulmonary, Second line, Third line

Phase: II

Therapies: olaparib, bevacizumab

Location: Republic of Korea

NCT03506997
PERSEUS1: Phase II Trial of the Immune Checkpoint Inhibitor Pembrolizumab for Patients Suffering From Metastatic Prostate Cancer

Cancer type: Castration-Resistant Prostate Cancer

Variant class: HRR mutation

Other identifiers: CCR4559, EudraCT Number: 2017-000931-15, ICR-CTSU/2016/10060, IRAS ID 208952, PERSEUS1

Population segments: Hormone refractory, Second line, Stage IV

Phase: II

Therapy: pembrolizumab

Location: United Kingdom

CDK12 mutation (continued)

NCT02264678

A Modular Phase I, Open-Label, Multicentre Study to Assess the Safety, Tolerability, Pharmacokinetics and Preliminary Anti-tumour Activity of Ceralasertib in Combination With Cytotoxic Chemotherapy and/or DNA Damage Repair/Novel Anti-cancer Agents in Patients With Advanced Solid Malignancies

Cancer type: Triple Negative Breast Cancer

Variant class: HRR mutation

Other inclusion criteria: BRCA germline mutation negative, BRCA mutation negative

Other identifiers: 0C-14-9, 16-031, 18-342, D5330C00004, EudraCT Number: 2014-002233-66, IRAS ID 160200, NCI-2015-00687, Study 4

Population segments: Adenocarcinoma, BRCA, HER2 negative, Pulmonary, Second line, Stage III, Stage IV, Third line, Triple receptor negative

Exclusion criteria variant class: ERBB2 positive (Breast Cancer)

Phase: I

Therapies: ceralasertib, olaparib

Locations: France, Republic of Korea, United Kingdom, United States

US States: CA, MA, NY

Contact: AstraZeneca Clinical Study Information Center [877-240-9479; information.center@astrazeneca.com]

NCT04890613

Phase Ib Expansion Study of CX-5461 in Patients With Solid Tumours and BRCA2 and/or PALB2 Mutation

Cancer type: Ovarian Cancer

Variant class: HRR mutation

Other identifier: CX-5461-04

Population segments: BRCA, Second line, Stage II, Stage III, Stage IV

Phase: I

Therapy: pidnarulex

Location: Canada

NCT05002868

A Multi-center, Open-label, Phase I/Ib Study to Assess the Safety, Pharmacokinetics and Anti-tumor Activity of RP12146, a Poly (ADP-ribose) Polymerase (PARP) Inhibitor, in Patients With Locally Advanced or Metastatic Solid Tumors

Cancer type: Biliary Tract Carcinoma, Breast Cancer, Colorectal Cancer, Endometrial Carcinoma, Gastric Cancer, Ovarian Cancer, Pancreatic Cancer, Prostate Cancer, Small Cell Lung Cancer

Variant class: HRR mutation

Other identifier: RP12146-2101

Population segments: Extensive, Pulmonary, Second line, Stage III, Stage IV

Exclusion criteria variant class: ERBB2 positive (Breast Cancer)

Phase: I

Therapy: RP12146

Location: Poland

CDK12 mutation (continued)

NCT03395197

A Phase III, Randomized, Double-blind, Placebo-controlled Study Of Talazoparib With Enzalutamide In Metastatic Castration-resistant Prostate Cancer

Cancer type: Castration-Resistant Prostate Cancer

Variant class: DNA repair mutation

Other identifiers: BASIC-ROYAL / HIGH-ROYAL, C3441021, CSET 2938, CTR20191443, CTR20191444, EudraCT Number: 2017-003295-31, GU 170 - C3441021, HCC 19-054, JapicCTI-194665, LH19.022, MOH_2019-05-16_007127, NCI-2018-01651, Pfizer C3441021, R19062M, RECF3951, RWF_C3441021 TALAPRO-2, TALAPRO-2, TX228665, UCI-18-47, VICCURO17115

Population segments: First line, Hormone refractory, Second line, Stage III, Stage IV

Phase: III

Therapies: talazoparib, hormone therapy

Locations: Argentina, Australia, Belgium, Brazil, Canada, Chile, China, Czech Republic, Finland, France, Germany, Hungary, Israel, Italy, Japan, New Zealand, Norway, Peru, Poland, Portugal, Republic of Korea, South Africa, Spain, Sweden, United Kingdom, United States

US States: AZ, CA, CO, FL, GA, IL, IN, KS, MO, NE, NJ, NY, OH, OK, OR, PA, SC, TN, TX, UT, VA, WI

Contact: Pfizer CT.gov Call Center [800-718-1021; ClinicalTrials.gov_Inquiries@pfizer.com]

NCT04821622

TALAPRO-3: A Phase III, Randomized, Double-blind, Study of Talazoparib With Enzalutamide Versus Placebo With Enzalutamide in Men With DDR Gene Mutated Metastatic Castration-sensitive Prostate Cancer

Cancer type: Prostate Adenocarcinoma

Variant class: DNA repair mutation

Other identifiers: C3441052, CTR20212191, EudraCT Number: 2021-000248-23, jRCT2031210098, TALAPRO-3

Population segments: Second line, Stage IV

Phase: III

Therapies: talazoparib, hormone therapy

Locations: Japan, Republic of Korea, United States

US States: CA, DC, FL, GA, IL, MI, NJ, NY, PA, SC, TX, UT

Contact: Pfizer CT.gov Call Center [800-718-1021; ClinicalTrials.gov_Inquiries@pfizer.com]

NCT04187833

Phase II Trial of Nivolumab in Combination with Talazoparib in Patients with Unresectable or Metastatic Melanoma and Mutations in BRCA or BRCA-ness Genes.

Cancer type: Melanoma

Variant class: DNA repair mutation

Other identifiers: CASE2619, NCI-2020-00416

Population segments: Second line, Stage III, Stage IV

Phase: II

Therapies: nivolumab, talazoparib

Location: United States

US State: OH

Contact: Dr. Pauline Funchain [866-223-8100; TaussigResearch@ccf.org]

Disclaimer: The data presented here is from a curated knowledgebase of publicly available information, but may not be exhaustive. The data version is 2021.11(003).

CDK12 mutation (continued)

NCT03344965

A Phase II Study of Olaparib Monotherapy in Metastatic Breast Cancer Patients With Germline or Somatic Mutations in DNA Repair Genes (Olaparib Expanded)

Cancer type: Breast Cancer

Variant class: DNA repair mutation

Other inclusion criteria: BRCA1 germline mutation negative, BRCA2 germline mutation negative

Other identifiers: 17-428, 18-414, NCI-2018-00778, TBCRC 048, TBCRC048

Population segments: BRCA, Estrogen receptor positive, HER2 negative, HER2 positive, Progesterone receptor positive, Second line, Stage IV

Exclusion criteria variant classes: BRCA1 germline mutation (Breast Cancer), BRCA2 germline mutation (Breast Cancer)

Phase: II

Therapy: olaparib

Location: United States

US States: AL, CA, IL, MA, MD, NC, NJ, NY, PA, WA

Contact: Dr. Nadine Tung [617-667-1962; ntung@bidmc.harvard.edu]

NCT03375307

A Phase II Study of Olaparib (AZD2281) in Patients With Metastatic/Advanced Urothelial Carcinoma With DNA-Repair Defects

Cancer type: Bladder Urothelial Carcinoma

Variant class: DNA repair mutation

Other identifiers: 10144, 19C0023, NCI-2017-02296, P162941

Population segments: BRCA, STK11, Second line, Stage III, Stage IV

Phase: II

Therapy: olaparib

Location: United States

US States: CA, CO, KY, MD, NY, OK

Contact: Multiple contacts: See www.clinicaltrials.gov for complete list of contacts.

NCT04038502

An Open-label, Multicenter Phase II Study to Compare the Efficacy of Carboplatin as First-line Followed by Second-line Olaparib Versus Olaparib as First-line Followed by Second-line Carboplatin in the Treatment of Patients With Castration Resistant Prostate Cancer Containing Homologous Recombination Deficiency

Cancer type: Castration-Resistant Prostate Cancer

Variant class: DNA repair mutation

Other identifiers: 01781, 01783, 19-08, BRACeD, CX001963-01, ONCA-009-18F

Population segments: First line, Hormone refractory, Second line, Stage IV

Phase: II

Therapies: olaparib, chemotherapy

Location: United States

US States: CA, DC, FL, IL, MI, NC, NY, PA, WA

Contact: Dr. Robert B. Montgomery [206-277-6878; rbmontgo@u.washington.edu]

CDK12 mutation (continued)

NCT04633902
Phase II Study of Olaparib in Combination With Pembrolizumab in Patients With Advanced Melanoma With Homologous Recombination (HR) Pathway Gene Mutation

Cancer type: Melanoma

Variant class: HRR pathway

Other identifier: CPMC19-MEL01

Population segments: BRAF, Second line, Stage III, Stage IV

Phase: II

Therapies: olaparib, pembrolizumab

Location: United States

US State: CA

Contact: Dr. Kevin B. Kim [415-885-8600; ClinicalResearch@sutterhealth.org]

NCT03248570
Phase II Open Label Study of Pembrolizumab in Patients With Metastatic Castration Resistant Prostate Cancer (mCRPC) With or Without DNA Damage Repair Defects

Cancer type: Castration-Resistant Prostate Cancer

Variant class: DNA repair mutation

Other identifiers: 16557, 17-22165, CC 16557, NCI-2017-02408

Population segments: BRCA, Hormone refractory, Line of therapy N/A, Stage IV

Phase: II

Therapy: pembrolizumab

Location: United States

US State: CA

Contact: Patricia Li [415-476-5975; GUTrials@ucsf.edu]

No NCT ID - see other identifier(s)
Single-arm Exploratory Study for Sintilimab Second-line or Later-line Treatment of Advanced Non-small Cell Lung Cancer with DDR Pathway Gene Mutation

Cancer type: Non-Small Cell Lung Cancer

Variant class: DNA repair mutation

Other identifier: ChiCTR1900023234

Population segments: Fourth line or greater, Second line, Stage III, Stage IV, Third line

Exclusion criteria variant classes: ALK fusion (Non-Small Cell Lung Cancer), EGFRi sensitizing mutation (Non-Small Cell Lung Cancer), ROS1 fusion (Non-Small Cell Lung Cancer)

Phase: I/II

Therapy: sintilimab

Location: China

Disclaimer: The data presented here is from a curated knowledgebase of publicly available information, but may not be exhaustive. The data version is 2021.11(003).

CDK12 mutation (continued)

NCT04095273

A Multicenter, Non-randomized, Open-label Phase Ib Study to Determine the Maximum Tolerated and Recommended Phase II Dose of the ATR Inhibitor BAY1895344 in Combination With Pembrolizumab and to Characterize Its Safety, Tolerability, Pharmacokinetics and Preliminary Anti-tumor Activity in Patients With Advanced Solid Tumors

Cancer type: Breast Cancer, Castration-Resistant Prostate Cancer, Colorectal Cancer, Gastric Cancer, Gastroesophageal Junction Adenocarcinoma, Non-Small Cell Lung Cancer, Pancreatic Cancer

Variant class: DNA repair mutation

Other inclusion criteria: ATM mutation negative or ATM mutation negative, ERBB2 negative, Hormone receptor positive or ATM underexpression

Other identifiers: 19741, EudraCT Number: 2018-003420-36, Keynote 919, NCI-2019-06734

Population segments: HER2 negative, Hormone refractory, Second line, Stage III, Stage IV

Phase: I

Therapies: BAY-1895344, pembrolizumab

Locations: Germany, Spain, Switzerland, United States

US States: CA, CT, MA, MD, NC, NY, OH, TX

Contact: Bayer Clinical Trials Contact [clinical-trials-contact@bayer.com]

NCT04849364

A Phase II Circulating Tumor DNA Enriched, Genomically Directed Post-neoadjuvant Trial for Patients With Residual Triple Negative Breast Cancer (PERSEVERE)

Cancer type: Triple Negative Breast Cancer

Variant class: DNA repair pathway

Other identifiers: HCRN BRE18-334, PERSEVERE

Population segments: Adjuvant, HER2 negative, Stage I, Stage II, Stage III, Triple receptor negative

Phase: II

Therapies: talazoparib, chemotherapy

Location: United States

US State: IN

Contact: Dr. Bryan P. Schneider [317-948-3885; bpschnei@iu.edu]

CDK12 splice site mutation

NCT02693535
Targeted Agent and Profiling Utilization Registry (TAPUR) Study.

Cancer type: Unspecified Solid Tumor

Variant class: CDK12 mutation

Other identifiers: 20170529, NCI-2017-00510, Pro00014171, TAPUR

Population segments: Aggressive, BRAF, Diffuse large B-cell lymphoma (DLBCL), Extranodal marginal zone B-cell lymphoma (MALT), FGFR, First line, Follicular lymphoma (FL), Fourth line or greater, Indolent, Lymphoblastic lymphoma (LBL), Mantle cell lymphoma (MCL), Other subtype, Second line, Small lymphocytic lymphoma (SLL), Stage III, Stage IV, Third line, Waldenstrom's macroglobulinemia (WM)

Phase: II

Therapy: atezolizumab + talazoparib

Location: United States

US States: AL, AZ, CA, FL, GA, HI, IL, IN, ME, MI, NC, ND, NE, NH, OH, OR, PA, SD, TX, UT, VA, WA

Contact: Pam Mangat [pam.mangat@asco.org]

NCT03742895
A Phase II Study of Olaparib Monotherapy in Participants With Previously Treated, Homologous Recombination Repair Mutation (HRRm) or Homologous Recombination Deficiency (HRD) Positive Advanced Cancer

Cancer type: Unspecified Solid Tumor

Variant class: CDK12 mutation

Other identifiers: (MK-7339-002 / LYNK-002), 19-139, 194694, 2018-01891, 7339-002, 7339-002-00, EudraCT Number: 2018-003007-19, IRAS ID: 255158, JapicCTI-194694, LYNK002, LYNK-002, MK7339-002, MK-7339-002, MK7339-002-00, NCI-2018-03519, PER-046-18, SNCTP000003157

Population segments: BRCA, Fourth line or greater, Second line, Stage III, Stage IV, Third line

Exclusion criteria variant classes: BRCA germline mutation (Breast Cancer, Ovarian Cancer), BRCA mutation (Ovarian Cancer)

Phase: II

Therapy: olaparib

Locations: Argentina, Australia, Canada, Colombia, Denmark, France, Guatemala, Ireland, Israel, Italy, Mexico, Peru, Republic of Korea, Romania, Russian Federation, Spain, Switzerland, Turkey, United Kingdom, United States

US States: AZ, CA, CO, GA, KY, MA, MD, MI, NE, NJ, NY, SD, UT, WA

Contact: Toll Free Number [888-577-8839; Trialsites@merck.com]

NCT03967938
Efficacy of Olaparib in Advanced Cancers Occurring in Patients With Germline Mutations or Somatic Tumor Mutations in Homologous Recombination Genes

Cancer type: Unspecified Solid Tumor

Variant class: CDK12 mutation

Other identifiers: 1-2018 BSMO, 1-2018BSMO, EudraCT Number: 2018-002966-37, Precision 2 - Olaparib

Population segments: Line of therapy N/A, Stage III, Stage IV

Exclusion criteria variant classes: BRCA1 germline mutation (Breast Cancer, Pancreatic Cancer, Prostate Cancer), BRCA2 germline mutation (Breast Cancer, Pancreatic Cancer, Prostate Cancer)

Phase: II

Therapy: olaparib

Location: Belgium

Disclaimer: The data presented here is from a curated knowledgebase of publicly available information, but may not be exhaustive. The data version is 2021.11(003).

CDK12 splice site mutation (continued)

NCT04550494

A Pharmacodynamics-Driven Trial of Talazoparib, an Oral PARP Inhibitor, in Patients With Advanced Solid Tumors and Aberrations in Genes Involved in DNA Damage Response

Cancer type: Unspecified Solid Tumor

Variant class: CDK12 mutation

Other identifiers: 000246, 000264-C, 10000264, 10371, NCI-2020-06906

Population segments: BRCA, Fourth line or greater, HER2 positive, Hormone refractory, Second line, Stage II, Stage III, Stage IV, Third line

Phase: II

Therapy: talazoparib

Location: United States

US State: MD

Contact: Multiple contacts: See www.clinicaltrials.gov for complete list of contacts.

NCT03842228

A Phase Ib Biomarker-Driven Combination Trial of Copanlisib, Olaparib, and Durvalumab (MEDI4736) in Patients With Advanced Solid Tumors

Cancer type: Unspecified Solid Tumor

Variant class: CDK12 mutation

Other identifiers: 10217, 19-628, NCI10217, NCI-2019-00601

Population segments: Line of therapy N/A, Stage III, Stage IV

Phase: I

Therapies: copanlisib, olaparib, durvalumab

Location: United States

US States: MA, TX

Contact: Multiple contacts: See www.clinicaltrials.gov for complete list of contacts.

NCT04586335

Open Label, Phase Ib Study to Evaluate the Safety, Tolerability, Pharmacokinetics and Clinical Activity of CYH33, an Oral a-specific PI3K Inhibitor in Combination With Olaparib, an Oral PARP Inhibitor in Patients With Advanced Solid Tumors.

Cancer type: Breast Cancer, Endometrial Carcinoma, Ovarian Cancer, Prostate Cancer, Unspecified Solid Tumor

Variant class: CDK12 mutation

Other identifiers: CTR20202339, CYH33-G102

Population segments: BRCA, Second line, Stage III, Stage IV

Phase: I

Therapies: HH-CYH33, olaparib

Locations: Australia, United States

US State: TX

Contact: Dr. Jason Sudia [908-380-1329; jason.sudia@haihepharma.com]

Disclaimer: The data presented here is from a curated knowledgebase of publicly available information, but may not be exhaustive. The data version is 2021.11(003).

CDK12 splice site mutation (continued)

NCT02029001
A Two-period, Multicenter, Randomized, Open-label, Phase II Study Evaluating the Clinical Benefit of a Maintenance Treatment Targeting Tumor Molecular Alterations in Patients with Progressive Locally-advanced or Metastatic Solid Tumors.

Cancer type: Unspecified Solid Tumor

Variant class: HRR mutation

Other identifiers: ET12-081, EudraCT Number: 2012-004510-34, MOST, ProfiLER

Population segments: Maintenance/Consolidation, Second line, Stage III, Stage IV, Third line

Exclusion criteria variant classes: ATM mutation (Gastric Cancer), BRCA1 germline mutation (Gastric Cancer), BRCA1 mutation (Gastric Cancer), BRCA2 germline mutation (Gastric Cancer), BRCA2 mutation (Gastric Cancer), HRR mutation (Gastric Cancer), PALB2 mutation (Gastric Cancer), RAD51 mutation (Gastric Cancer)

Phase: II

Therapy: olaparib

Location: France

NCT04123366
A Phase II Study of Olaparib in Combination With Pembrolizumab in Participants With Previously Treated, Homologous Recombination Repair Mutation (HRRm) and/or Homologous Recombination Deficiency (HRD)-Positive Advanced Cancer

Cancer type: Unspecified Solid Tumor

Variant class: HRR mutation

Other identifiers: 7339-007, EudraCT Number: 2019-001745-40, jRCT2011200025, KEYLYNK-007, MK-7339-007, NCI-2019-08758, PER-029-19

Population segments: BRCA, Second line, Stage III, Stage IV

Exclusion criteria variant classes: BRCA germline mutation (Breast Cancer, Ovarian Cancer), BRCA mutation (Breast Cancer, Ovarian Cancer)

Phase: II

Therapies: olaparib, pembrolizumab

Locations: Argentina, Australia, Canada, Colombia, France, Germany, Guatemala, Israel, Italy, Japan, Latvia, Mexico, Peru, Poland, Puerto Rico, Republic of Korea, Romania, South Africa, Spain, Sweden, Turkey, Ukraine, United States

US States: AL, AZ, CA, CO, FL, GA, KY, NJ, NY, OH, TX, UT, VA

Contact: Toll Free Number [888-577-8839; Trialsites@merck.com]

NCT02401347
A Phase II Clinical Trial of the PARP Inhibitor Talazoparib in BRCA1 and BRCA2 Wild Type Patients With Advanced Triple Negative Breast Cancer and Homologous Recombination Deficiency or Advanced HER2 Negative Breast Cancer or Other Solid Tumors With a Mutation in Homologous Recombination Pathway Genes Talazoparib Beyond BRCA (TBB) Trial

Cancer type: Breast Cancer, Unspecified Solid Tumor

Variant class: HRR mutation

Other inclusion criteria: ERBB2 negative, Hormone receptor positive

Other identifiers: BRS0050, NCI-2015-00036, TBB

Population segments: Estrogen receptor positive, HER2 negative, Progesterone receptor positive, Second line, Stage III, Stage IV, Triple receptor negative

Exclusion criteria variant classes: BRCA1 germline mutation (Breast Cancer, Triple Negative Breast Cancer, Unspecified Solid Tumor), BRCA1 mutation (Breast Cancer, Triple Negative Breast Cancer, Unspecified Solid Tumor), BRCA2 germline mutation (Breast Cancer, Triple Negative Breast Cancer, Unspecified Solid Tumor), BRCA2 mutation (Breast Cancer, Triple Negative Breast Cancer, Unspecified Solid Tumor), ERBB2 positive (Breast Cancer)

Phase: II

Therapy: talazoparib

Location: United States

US State: CA

Contact: Amanda Selin [650-721-6977; aselin@stanford.edu]

CDK12 splice site mutation (continued)

NCT04497116
Phase I/IIa Study of the Safety, Pharmacokinetics, Pharmacodynamics and Preliminary Clinical Activity of RP-3500 Alone or in Combination With Talazoparib in Advanced Solid Tumors With ATR Inhibitor Sensitizing Mutations (TRESR Study)

Cancer type: Unspecified Solid Tumor

Variant class: CDK12 aberration

Other identifiers: 20-521, EudraCT Number: 2020-000301-87, IRAS ID: 286609, NCI-2020-06869, RP-3500-01, TRESR, TRESR Study

Population segments: Second line, Stage III, Stage IV

Phase: I/II

Therapies: RP-3500, talazoparib

Locations: Canada, Denmark, United Kingdom, United States

US States: MA, NC, NY, RI, TN, TX

Contact: Dr. Peter Manley [857-322-5553; pmanley@reparerx.com]

NCT03415659
A Phase I, Open-label, Single-center, Single/Multiple-dose, Dose-escalation/ Dose-expansion Clinical Study on Tolerance and Pharmacokinetics of HWH340 Tablet in Patients With Advanced Solid Tumors

Cancer type: Unspecified Solid Tumor

Variant class: HRR mutation

Other inclusion criteria: BRCA1 germline mutation negative, BRCA1 mutation negative, BRCA2 germline mutation negative, BRCA2 mutation negative

Other identifiers: CTR20171649, HWH340-RFPA 20170821, RFPA 20170821

Population segments: BRCA, Fourth line or greater, HER2 negative, Second line, Stage III, Stage IV, Third line

Phase: I

Therapy: HWH-340

Location: China

NCT03767075
Basket of Baskets: A Modular, Open-label, Phase II, Multicentre Study To Evaluate Targeted Agents in Molecularly Selected Populations With Advanced Solid Tumours

Cancer type: Unspecified Solid Tumor

Variant class: DNA repair mutation

Other identifiers: (Basket of Baskets) (BoB), EudraCT number: 2017-005108-89, MO39164, NL65937.031.18, VHIO17002

Population segments: Second line, Stage III, Stage IV

Phase: II

Therapy: atezolizumab

Locations: France, Germany, Netherlands, Spain, Sweden, United Kingdom

---

NCT04905914
A Phase I/IIa, Open-Label, Safety, Pharmacokinetic, And Preliminary Efficacy Study Of Oral ATRN-119 In Patients With Advanced Solid Tumors

Cancer type: Unspecified Solid Tumor

Variant class: DNA repair mutation

Other identifier: AR-276-01

Population segments: Line of therapy N/A, Stage III, Stage IV

Phase: I/II

Therapy: ATRN-119

Location: United States

US State: MA

Contact: Robert Hasson [619-540-6253; rhasson@pacificlinkconsulting.com]

Disclaimer: The data presented here is from a curated knowledgebase of publicly available information, but may not be exhaustive. The data version is 2021.11(003).

CDK12 splice site mutation (continued)

NCT04901702

A Randomized Phase I/II Study of Talazoparib or Temozolomide in Combination With Onivyde in Children With Recurrent Solid Malignancies and Ewing Sarcoma

Cancer type: Unspecified Solid Tumor

Variant class: HRR pathway

Other identifiers: EudraCT Number: 2021-003570-31, ONITT

Population segments: Second line, Stage IV

Exclusion criteria variant classes: HRR germline pathway (Central Nervous System Neoplasm, Ewing Sarcoma), HRR pathway (Central Nervous System Neoplasm, Ewing Sarcoma)

Phase: I/II

Therapies: talazoparib, chemotherapy

Locations: Canada, United States

US State: TN

Contact: Dr. Sara Federico [866-278-5833; referralinfo@stjude.org]

NCT03155620

NCI-COG Pediatric MATCH (Molecular Analysis for Therapy Choice) Screening Protocol

Cancer type: Unspecified Solid Tumor

Variant class: DNA repair pathway

Other identifiers: 17-729, 2017-0625, 20170841, APEC1621SC, COGAPEC1621, COGAPEC1621SC, N 55017 SC, NCI-2017-01251, NCI-COG Pediatric MATCH, Pediatric MATCH

Population segments: ALK, Aggressive, BRAF, FGFR, HRAS, Indolent, KRAS, NRAS, Pediatric or Adolescent, Second line, Stage III, Stage IV

Phase: II

Therapy: olaparib

Locations: Puerto Rico, United States

US States: AK, AL, AR, AZ, CA, CO, CT, DC, DE, FL, GA, HI, IA, ID, IL, IN, KY, LA, MA, MD, ME, MI, MN, MO, MS, NC, NE, NH, NJ, NV, NY, OH, OK, OR, PA, RI, SC, SD, TN, TX, UT, VA, VT, WA, WI

Contact: Multiple contacts: See www.clinicaltrials.gov for complete list of contacts.

NCT03233204

NCI-COG Pediatric MATCH (Molecular Analysis for Therapy Choice)- Phase 2 Subprotocol of Olaparib in Patients With Tumors Harboring Defects in DNA Damage Repair Genes

Cancer type: Unspecified Solid Tumor

Variant class: DNA repair pathway

Other identifiers: 20170841, APEC1621H, NCI-2017-00766

Population segments: Aggressive, Indolent, Pediatric or Adolescent, Second line, Stage III, Stage IV

Phase: II

Therapy: olaparib

Locations: Puerto Rico, United States

US States: AK, AL, AR, AZ, CA, CO, DC, DE, FL, GA, IA, ID, IL, IN, KY, LA, MD, ME, MI, MN, MO, MS, NC, ND, NE, NJ, NY, OH, OK, OR, PA, SC, SD, TN, TX, UT, VA, VT, WA, WI

Contact: Multiple contacts: See www.clinicaltrials.gov for complete list of contacts.

Disclaimer: The data presented here is from a curated knowledgebase of publicly available information, but may not be exhaustive. The data version is 2021.11(003).

CDK12 splice site mutation (continued)

NCT04267939
An Open-label Phase Ib Study to Determine the Maximum Tolerated and/or Recommended Phase II Dose of the ATR Inhibitor BAY 1895344 in Combination With PARP Inhibitor Niraparib, in Patients With Recurrent Advanced Solid Tumors and Ovarian Cancer

Cancer type: Ovarian Cancer, Unspecified Solid Tumor

Variant class: DNA repair pathway

Other identifiers: 18595, 19-810, 2019-0471, 20-222, EudraCT Number: 2018-003930-34, NCI-2020-01405

Population segments: Maintenance/Consolidation, Second line, Stage III, Stage IV

Exclusion criteria variant class: DNA repair pathway (Prostate Cancer)

Phase: I

Therapies: BAY-1895344, niraparib

Location: United States

US States: MA, NY, TX

Contact: Bayer Clinical Trials Contact [888-842-2937; clinical-trials-contact@bayer.com]

NCT04693468
Modular Phase IB Hypothesis-Testing, Biomarker-Driven, Talazoparib Combination Trial (TalaCom)

Cancer type: Unspecified Solid Tumor

Variant class: DNA repair pathway

Other identifiers: 2020-0436, NCI-2020-06041, TalaCom

Population segments: Hormone refractory, Second line, Stage III, Stage IV

Phase: I

Therapies: talazoparib, palbociclib, axitinib, crizotinib

Location: United States

US State: TX

Contact: Timothy A. Yap [713-563-1784; tyap@mdanderson.org]

NCT03810105
A Phase II Study of Olaparib and Durvalumab in Men With Castration Sensitive Biochemically Recurrent Non-Metastatic Prostate Cancer Harboring Mutations in DNA Damage Repair

Cancer type: Prostate Cancer

Variant class: CDK12 splice site mutation or DNA repair splice site mutation

Other identifiers: 18-480, NCI-2019-01627

Population segments: Adjuvant, Stage I, Stage II, Stage III

Phase: II

Therapies: durvalumab, olaparib

Location: United States

US States: CA, IL, MI, NJ, NY

Contact: Dr. Karen Autio [646-422-4632; autiok@mskcc.org]

Disclaimer: The data presented here is from a curated knowledgebase of publicly available information, but may not be exhaustive. The data version is 2021.11(003).

CDK12 splice site mutation (continued)

NCT04751929

A Phase II Multi-Center Trial of Abemaciclib With or Without Atezolizumab in Metastatic Castration Resistant Prostate Cancer

Cancer type: Castration-Resistant Prostate Cancer

Variant class: CDK12 mutation

Other identifier: 20-701

Population segments: Hormone refractory, Second line, Stage IV

Phase: II

Therapies: abemaciclib, atezolizumab

Location: United States

US State: MA

Contact: Dr. Atish Choudhury [617-632-6328; achoudhury@partners.org]

NCT04336943

Durvalumab (MEDI4736) and Olaparib (AZD2281) for Treatment of Biochemically Recurrent Prostate Cancer in Men Predicted to Have a High Neoantigen Load: A Pilot Study

Cancer type: Prostate Adenocarcinoma

Variant class: CDK12 mutation

Other identifiers: 10509, NCI-2020-01860, RG1007001

Population segments: Line of therapy N/A, Stage I, Stage II, Stage III

Phase: II

Therapies: durvalumab, olaparib

Location: United States

US State: WA

Contact: Michael Schweizer [206-606-6252; schweize@uw.edu]

NCT04053322

An International Multicenter Phase II Trial of Durvalumab (MEDI4736) Plus OLAparib Plus Fulvestrant in Metastatic or Locally Advanced ER-positive, HER2-negative Breast Cancer Patients Selected Using Criteria That Predict Sensitivity to Olaparib

Cancer type: Breast Cancer

Variant class: CDK12 mutation

Other inclusion criteria: ERBB2 negative, ER positive

Other identifiers: CADUSEIME08, DOLAF, EudraCT Number: 2018-003832-57, UC-0140/1812

Population segments: BRCA, Estrogen receptor positive, FGFR, HER2 negative, Progesterone receptor positive, Second line, Stage III, Stage IV

Phase: II

Therapies: durvalumab, olaparib, hormone therapy

Location: France

Disclaimer: The data presented here is from a curated knowledgebase of publicly available information, but may not be exhaustive. The data version is 2021.11(003).

CDK12 splice site mutation (continued)

NCT05011383
High-dose Testosterone in Men With Metastatic Castration-resistant Prostate Cancer and ATM or CDK12 Deficiency

Cancer type: Castration-Resistant Prostate Cancer

Variant class: CDK12 mutation

Other identifiers: SPLP-003-20F, VA-BAT

Population segments: Hormone refractory, Second line, Stage IV

Phase: II

Therapy: hormone therapy

Location: United States

US State: WA

Contact: Dr. Robert B. Montgomery [206-277-6878; Robert.Montgomery@va.gov]

NCT03553004
Niraparib in Metastatic Pancreatic Cancer After Previous Chemotherapy (NIRA-PANC): A Phase II Trial

Cancer type: Pancreatic Cancer

Variant class: CDK12 mutation

Other identifiers: IIT-2017-NIRA-PANC, NIRA-PANC, STUDY00141862

Population segments: BRCA, Second line, Stage IV, Third line

Phase: II

Therapy: niraparib

Location: United States

US States: KS, MO

Contact: Clinical Trials Nurse Navigator [913-588-3671; navigation@kumc.edu]

NCT03840967
A Phase II Study Evaluating Safety and Efficacy of Niraparib in Patients With Previously Treated Homologous Recombination (HR) Defective or Loss of Heterozygosity (LOH) High Metastatic Esophageal/Gastroesophageal Junction/Proximal Gastric Adenocarcinoma

Cancer type: Esophageal Cancer, Gastric Cancer, Gastroesophageal Junction Adenocarcinoma

Variant class: CDK12 mutation

Other identifiers: 20166, 71618, HCRN ESO17-325, NCI-2019-07395

Population segments: Second line, Stage III, Stage IV

Phase: II

Therapy: niraparib

Location: United States

US States: FL, IN, MI, NY

Contact: Dr. Shadia Jalal [317-274-3658; sjalal@iu.edu]

NCT04030559
Phase II Trial of PARP Inhibitor Niraparib for Men With High Risk Prostate Cancer and DNA Damage Response Defects

Cancer type: Prostate Cancer

Variant class: CDK12 mutation

Other identifiers: 1412676, NCI-2019-04365, UCDCC#279

Population segments: First line, Neoadjuvant, Stage I, Stage II, Stage IV

Phase: II

Therapy: niraparib

Location: United States

US State: CA

Contact: Marc Dall'Era [916-734-3771; mdallera@ucdavis.edu]

Disclaimer: The data presented here is from a curated knowledgebase of publicly available information, but may not be exhaustive. The data version is 2021.11(003).

CDK12 splice site mutation (continued)

NCT04019964
Nivolumab as a Non-Castrating Therapy for MMR-deficient and CDK12- Altered Prostate Cancer With PSA Recurrence After Local Therapy

Cancer type: Prostate Cancer

Variant class: CDK12 mutation

Other identifiers: CA209-76M, CRMS-71513, J1933, NCI-2020-03804

Population segments: (N/A), Hormone refractory, Second line

Phase: II

Therapy: nivolumab

Location: United States

US State: MD

Contact: Rana Sullivan [410-614-6337; tomalra@jhmi.edu]

NCT03012321
BRCAAway: A Randomized Phase II Trial of Abiraterone, Olaparib, or Abiraterone + Olaparib in Patients With Metastatic Castration-Resistant Prostate Cancer With DNA Repair Defects

Cancer type: Castration-Resistant Prostate Cancer

Variant class: CDK12 mutation

Other identifiers: 19137, BRCAAway, NCI-2016-01834, NU 16U05, NU_16U05, PCCTC #: c16-168, PCCTC c16-168, STU00203960

Population segments: First line, Hormone refractory, Stage IV

Phase: II

Therapy: olaparib

Location: United States

US States: CA, FL, IL, IN, MD, MI, MN, MO, NC, NY, UT, VA

Contact: Study Coordinator [312-695-1301; cancertrials@northwestern.edu]

NCT03786796
Phase II Study of Olaparib in Metastatic Renal Cell Carcinoma Patients Harboring a BAP-1 or Other DNA Repair Gene Mutations (ORCHID)

Cancer type: Kidney Cancer

Variant class: CDK12 mutation

Other identifiers: CRMS-70750, J18166, NCI-2019-06309, ORCHID

Population segments: Fourth line or greater, Second line, Stage IV, Third line

Phase: II

Therapy: olaparib

Location: United States

US State: MD

Contact: Irina Rifkind [410-502-2043; irifkin1@jhmi.edu]

NCT04515836
Phase II Trial of Olaparib in Homologous Recombination Deficient (HRD) Malignant Mesothelioma

Cancer type: Mesothelioma

Variant class: CDK12 mutation

Population segments: (N/A), Second line

Phase: II

Therapy: olaparib

Location: United States

US State: IL

Contact: Dr. Hedy L. Kindler [773-702-0360; hkindler@medicine.bsd.uchicago.edu]

CDK12 splice site mutation (continued)

NCT04104893

A Phase II Study Of A Checkpoint Inhibitor In Men With Progressive Metastatic Castrate Resistant Prostate Cancer Characterized By A Mismatch Repair Deficiency Or Biallelic CDK12 Inactivation

Cancer type: Castration-Resistant Prostate Cancer

Variant class: CDK12 mutation

Other identifiers: CHOMP, CSDR-003-18F, NCI-2020-02215

Population segments: First line, Hormone refractory, Stage IV

Phase: II

Therapy: pembrolizumab

Location: United States

US States: CA, FL, IL, MI, NC, NY, WA

Contact: Dr. Matthew B. Rettig [310-478-3711; matthew.rettig@va.gov]

NCT03533946

A Phase II Study of Rucaparib Monotherapy in Nonmetastatic, Hormone-Sensitive Prostate Cancer Demonstrating "BRCAness" Genotype (ROAR)

Cancer type: Prostate Cancer

Variant class: CDK12 mutation

Other identifiers: HCI111833, NCI-2018-01754, ROAR

Population segments: First line, Stage I, Stage II, Stage III

Phase: II

Therapy: rucaparib

Location: United States

US State: UT

Contact: Jill Broghammer [801-213-6232; jill.broghammer@hci.utah.edu]

NCT04892693

Talazoparib in Advanced Breast Cancer Patients With Homologous Recombinant Deficiency: A Phase II Clinical and Exploratory Biomarker Study of Talazoparib

Cancer type: Breast Cancer

Variant class: CDK12 mutation

Other identifier: H-2009-079-1157

Population segments: BRCA, Line of therapy N/A, Stage III, Stage IV

Phase: II

Therapy: talazoparib

Location: Republic of Korea

NCT04740190

Combination Talazoparib - Carboplatin for Recurrent High-grade Glioma With DNA Damage Repair Deficiency (DDRd)

Cancer type: Anaplastic Astrocytoma, Anaplastic Oligoastrocytoma, Anaplastic Oligodendroglioma, Anaplastic Pleomorphic Xanthoastrocytoma, Diffuse Midline Glioma, Glioblastoma

Variant class: CDK12 mutation

Other identifiers: TAC-GReD, UW-20396

Population segments: (N/A), BRCA, Second line

Phase: II

Therapies: talazoparib, chemotherapy

Location: Hong Kong

Disclaimer: The data presented here is from a curated knowledgebase of publicly available information, but may not be exhaustive. The data version is 2021.11(003).

CDK12 splice site mutation (continued)

NCT04253262

A Phase Ib/II Study of Copanlisib Combined With Rucaparib in Patients With Metastatic Castration-resistant Prostate Cancer

Cancer type: Castration-Resistant Prostate Cancer

Variant class: CDK12 mutation

Other identifiers: BrUOG 360, BrUOG360

Population segments: Hormone refractory, Second line, Stage IV

Phase: I/II

Therapies: copanlisib, rucaparib

Location: United States

US State: RI

Contact: BrUOG [401-863-3000; BrUOG@brown.edu]

NCT04169841

Precision Medicine Phase II Study Evaluating the Efficacy of a Double Immunotherapy by Durvalumab and Tremelimumab Combined With Olaparib in Patients With Solid Cancers and Carriers of Homologous Recombination Repair Genes Mutation in Response or Stable After Olaparib Treatment

Cancer type: Breast Cancer, Endometrial Carcinoma, Head and Neck Cancer, Kidney Cancer, Non-Small Cell Lung Cancer, Ovarian Cancer, Pancreatic Cancer, Prostate Cancer

Variant class: HRR mutation

Other identifier: GUIDE2REPAIR

Population segments: Fourth line or greater, Hormone refractory, Maintenance/ Consolidation, Second line, Stage III, Stage IV, Third line

Exclusion criteria variant classes: ALK fusion (Lung Cancer), BRAF mutation (Lung Cancer), EGFR mutation (Lung Cancer), ROS1 mutation (Lung Cancer)

Phase: II

Therapies: durvalumab, tremelimumab, olaparib

Location: France

NCT03925350

A Phase II Study of Niraparib in Patients With Advanced Melanoma With Genetic Homologous Recombination (HR) Mutation / Alteration

Cancer type: Melanoma

Variant class: HRR mutation

Other identifier: CPMC17-MEL01

Population segments: Second line, Stage III, Stage IV

Phase: II

Therapy: niraparib

Location: United States

US State: CA

Contact: Laurel Brechtel [415-600-1654; BrechtLA@sutterhealth.org]

Disclaimer: The data presented here is from a curated knowledgebase of publicly available information, but may not be exhaustive. The data version is 2021.11(003).

CDK12 splice site mutation (continued)

NCT04940637
A Phase II, Open-Label, Single Arm, Prospective, Multicenter Study of Niraparib Plus Dostarlimab in Patients With Advanced NSCLC and/or MPM, and Positive for PD-L1 Expression and Germline or Somatic Mutations in the HRR Genes

Cancer type: Mesothelioma, Non-Small Cell Lung Cancer

Variant class: HRR mutation

Other inclusion criteria: ALK fusion negative, CD274 expression, EGFRi sensitizing mutation negative, ROS1 fusion negative or CD274 expression

Other identifiers: EudraCT Number: 2020-000109-10, UNITO-001, UNITO-001/2020

Population segments: Second line, Stage III, Stage IV

Exclusion criteria variant classes: ALK fusion (Non-Small Cell Lung Cancer), EGFRi sensitizing mutation (Non-Small Cell Lung Cancer), ROS1 fusion (Non-Small Cell Lung Cancer)

Phase: II

Therapies: niraparib, dostarlimab

Location: Italy

NCT04042831
A Phase II Study of Olaparib in Patients With Advanced Biliary Tract Cancer With Aberrant DNA Repair Gene Mutations

Cancer type: Biliary Tract Carcinoma

Variant class: HRR mutation

Other identifiers: ACCRU-ICRN-1702, NCI-2019-04434

Population segments: Second line, Stage IV

Phase: II

Therapy: olaparib

Location: United States

US States: AZ, MN, NY, TX

Contact: Multiple contacts: See www.clinicaltrials.gov for complete list of contacts.

NCT04939662
Phase II, Single-arm Study of Olaparib and Bevacizumab Combination Therapy in Relapsed Small Cell Lung Cancer Subjects With DNA Damage Response and the Repair Pathway Alteration, ATM Deficiency, SLFN11 Positive, or POU2F3 Positive

Cancer type: Small Cell Lung Cancer

Variant class: HRR mutation

Other identifier: SUKSES-B2

Population segments: (N/A), BRCA, Pulmonary, Second line, Third line

Phase: II

Therapies: olaparib, bevacizumab

Location: Republic of Korea

NCT03506997
PERSEUS1: Phase II Trial of the Immune Checkpoint Inhibitor Pembrolizumab for Patients Suffering From Metastatic Prostate Cancer

Cancer type: Castration-Resistant Prostate Cancer

Variant class: HRR mutation

Other identifiers: CCR4559, EudraCT Number: 2017-000931-15, ICR-CTSU/2016/10060, IRAS ID 208952, PERSEUS1

Population segments: Hormone refractory, Second line, Stage IV

Phase: II

Therapy: pembrolizumab

Location: United Kingdom

CDK12 splice site mutation (continued)

NCT02264678
A Modular Phase I, Open-Label, Multicentre Study to Assess the Safety, Tolerability, Pharmacokinetics and Preliminary Anti-tumour Activity of Ceralasertib in Combination With Cytotoxic Chemotherapy and/or DNA Damage Repair/Novel Anti-cancer Agents in Patients With Advanced Solid Malignancies

Cancer type: Triple Negative Breast Cancer

Variant class: HRR mutation

Other inclusion criteria: BRCA germline mutation negative, BRCA mutation negative

Other identifiers: 0C-14-9, 16-031, 18-342, D5330C00004, EudraCT Number: 2014-002233-66, IRAS ID 160200, NCI-2015-00687, Study 4

Population segments: Adenocarcinoma, BRCA, HER2 negative, Pulmonary, Second line, Stage III, Stage IV, Third line, Triple receptor negative

Exclusion criteria variant class: ERBB2 positive (Breast Cancer)

Phase: I

Therapies: ceralasertib, olaparib

Locations: France, Republic of Korea, United Kingdom, United States

US States: CA, MA, NY

Contact: AstraZeneca Clinical Study Information Center [877-240-9479; information.center@astrazeneca.com]

NCT04890613
Phase Ib Expansion Study of CX-5461 in Patients With Solid Tumours and BRCA2 and/or PALB2 Mutation

Cancer type: Ovarian Cancer

Variant class: HRR mutation

Other identifier: CX-5461-04

Population segments: BRCA, Second line, Stage II, Stage III, Stage IV

Phase: I

Therapy: pidnarulex

Location: Canada

NCT05002868
A Multi-center, Open-label, Phase I/Ib Study to Assess the Safety, Pharmacokinetics and Anti-tumor Activity of RP12146, a Poly (ADP-ribose) Polymerase (PARP) Inhibitor, in Patients With Locally Advanced or Metastatic Solid Tumors

Cancer type: Biliary Tract Carcinoma, Breast Cancer, Colorectal Cancer, Endometrial Carcinoma, Gastric Cancer, Ovarian Cancer, Pancreatic Cancer, Prostate Cancer, Small Cell Lung Cancer

Variant class: HRR mutation

Other identifier: RP12146-2101

Population segments: Extensive, Pulmonary, Second line, Stage III, Stage IV

Exclusion criteria variant class: ERBB2 positive (Breast Cancer)

Phase: I

Therapy: RP12146

Location: Poland

CDK12 splice site mutation (continued)

NCT03395197
A Phase III, Randomized, Double-blind, Placebo-controlled Study Of Talazoparib With Enzalutamide In Metastatic Castration-resistant Prostate Cancer

Cancer type: Castration-Resistant Prostate Cancer

Variant class: DNA repair mutation

Other identifiers: BASIC-ROYAL / HIGH-ROYAL, C3441021, CSET 2938, CTR20191443, CTR20191444, EudraCT Number: 2017-003295-31, GU 170 - C3441021, HCC 19-054, JapicCTI-194665, LH19.022, MOH_2019-05-16_007127, NCI-2018-01651, Pfizer C3441021, R19062M, RECF3951, RWF_C3441021 TALAPRO-2, TALAPRO-2, TX228665, UCI-18-47, VICCURO17115

Population segments: First line, Hormone refractory, Second line, Stage III, Stage IV

Phase: III

Therapies: talazoparib, hormone therapy

Locations: Argentina, Australia, Belgium, Brazil, Canada, Chile, China, Czech Republic, Finland, France, Germany, Hungary, Israel, Italy, Japan, New Zealand, Norway, Peru, Poland, Portugal, Republic of Korea, South Africa, Spain, Sweden, United Kingdom, United States

US States: AZ, CA, CO, FL, GA, IL, IN, KS, MO, NE, NJ, NY, OH, OK, OR, PA, SC, TN, TX, UT, VA, WI

Contact: Pfizer CT.gov Call Center [800-718-1021; ClinicalTrials.gov_Inquiries@pfizer.com]

NCT04821622
TALAPRO-3: A Phase III, Randomized, Double-blind, Study of Talazoparib With Enzalutamide Versus Placebo With Enzalutamide in Men With DDR Gene Mutated Metastatic Castration-sensitive Prostate Cancer

Cancer type: Prostate Adenocarcinoma

Variant class: DNA repair mutation

Other identifiers: C3441052, CTR20212191, EudraCT Number: 2021-000248-23, jRCT2031210098, TALAPRO-3

Population segments: Second line, Stage IV

Phase: III

Therapies: talazoparib, hormone therapy

Locations: Japan, Republic of Korea, United States

US States: CA, DC, FL, GA, IL, MI, NJ, NY, PA, SC, TX, UT

Contact: Pfizer CT.gov Call Center [800-718-1021; ClinicalTrials.gov_Inquiries@pfizer.com]

NCT04187833
Phase II Trial of Nivolumab in Combination with Talazoparib in Patients with Unresectable or Metastatic Melanoma and Mutations in BRCA or BRCA-ness Genes.

Cancer type: Melanoma

Variant class: DNA repair mutation

Other identifiers: CASE2619, NCI-2020-00416

Population segments: Second line, Stage III, Stage IV

Phase: II

Therapies: nivolumab, talazoparib

Location: United States

US State: OH

Contact: Dr. Pauline Funchain [866-223-8100; TaussigResearch@ccf.org]

CDK12 splice site mutation (continued)

NCT03344965
A Phase II Study of Olaparib Monotherapy in Metastatic Breast Cancer Patients With Germline or Somatic Mutations in DNA Repair Genes (Olaparib Expanded)

Cancer type: Breast Cancer

Variant class: DNA repair mutation

Other inclusion criteria: BRCA1 germline mutation negative, BRCA2 germline mutation negative

Other identifiers: 17-428, 18-414, NCI-2018-00778, TBCRC 048, TBCRC048

Population segments: BRCA, Estrogen receptor positive, HER2 negative, HER2 positive, Progesterone receptor positive, Second line, Stage IV

Exclusion criteria variant classes: BRCA1 germline mutation (Breast Cancer), BRCA2 germline mutation (Breast Cancer)

Phase: II

Therapy: olaparib

Location: United States

US States: AL, CA, IL, MA, MD, NC, NJ, NY, PA, WA

Contact: Dr. Nadine Tung [617-667-1962; ntung@bidmc.harvard.edu]

NCT03375307
A Phase II Study of Olaparib (AZD2281) in Patients With Metastatic/Advanced Urothelial Carcinoma With DNA-Repair Defects

Cancer type: Bladder Urothelial Carcinoma

Variant class: DNA repair mutation

Other identifiers: 10144, 19C0023, NCI-2017-02296, P162941

Population segments: BRCA, STK11, Second line, Stage III, Stage IV

Phase: II

Therapy: olaparib

Location: United States

US States: CA, CO, KY, MD, NY, OK

Contact: Multiple contacts: See www.clinicaltrials.gov for complete list of contacts.

NCT04038502
An Open-label, Multicenter Phase II Study to Compare the Efficacy of Carboplatin as First-line Followed by Second-line Olaparib Versus Olaparib as First-line Followed by Second-line Carboplatin in the Treatment of Patients With Castration Resistant Prostate Cancer Containing Homologous Recombination Deficiency

Cancer type: Castration-Resistant Prostate Cancer

Variant class: DNA repair mutation

Other identifiers: 01781, 01783, 19-08, BRACeD, CX001963-01, ONCA-009-18F

Population segments: First line, Hormone refractory, Second line, Stage IV

Phase: II

Therapies: olaparib, chemotherapy

Location: United States

US States: CA, DC, FL, IL, MI, NC, NY, PA, WA

Contact: Dr. Robert B. Montgomery [206-277-6878; rbmontgo@u.washington.edu]

Disclaimer: The data presented here is from a curated knowledgebase of publicly available information, but may not be exhaustive. The data version is 2021.11(003).

CDK12 splice site mutation (continued)

NCT04633902
Phase II Study of Olaparib in Combination With Pembrolizumab in Patients With Advanced Melanoma With Homologous Recombination (HR) Pathway Gene Mutation

Cancer type: Melanoma

Variant class: HRR pathway

Other identifier: CPMC19-MEL01

Population segments: BRAF, Second line, Stage III, Stage IV

Phase: II

Therapies: olaparib, pembrolizumab

Location: United States

US State: CA

Contact: Dr. Kevin B. Kim [415-885-8600; ClinicalResearch@sutterhealth.org]

NCT03248570
Phase II Open Label Study of Pembrolizumab in Patients With Metastatic Castration Resistant Prostate Cancer (mCRPC) With or Without DNA Damage Repair Defects

Cancer type: Castration-Resistant Prostate Cancer

Variant class: DNA repair mutation

Other identifiers: 16557, 17-22165, CC 16557, NCI-2017-02408

Population segments: BRCA, Hormone refractory, Line of therapy N/A, Stage IV

Phase: II

Therapy: pembrolizumab

Location: United States

US State: CA

Contact: Patricia Li [415-476-5975; GUTrials@ucsf.edu]

No NCT ID - see other identifier(s)
Single-arm Exploratory Study for Sintilimab Second-line or Later-line Treatment of Advanced Non-small Cell Lung Cancer with DDR Pathway Gene Mutation

Cancer type: Non-Small Cell Lung Cancer

Variant class: DNA repair mutation

Other identifier: ChiCTR1900023234

Population segments: Fourth line or greater, Second line, Stage III, Stage IV, Third line

Exclusion criteria variant classes: ALK fusion (Non-Small Cell Lung Cancer), EGFRi sensitizing mutation (Non-Small Cell Lung Cancer), ROS1 fusion (Non-Small Cell Lung Cancer)

Phase: I/II

Therapy: sintilimab

Location: China

CDK12 splice site mutation (continued)

NCT04095273

A Multicenter, Non-randomized, Open-label Phase Ib Study to Determine the Maximum Tolerated and Recommended Phase II Dose of the ATR Inhibitor BAY1895344 in Combination With Pembrolizumab and to Characterize Its Safety, Tolerability, Pharmacokinetics and Preliminary Anti-tumor Activity in Patients With Advanced Solid Tumors

Cancer type: Breast Cancer, Castration-Resistant Prostate Cancer, Colorectal Cancer, Gastric Cancer, Gastroesophageal Junction Adenocarcinoma, Non-Small Cell Lung Cancer, Pancreatic Cancer

Variant class: DNA repair mutation

Other inclusion criteria: ATM mutation negative or ATM mutation negative, ERBB2 negative, Hormone receptor positive or ATM underexpression

Other identifiers: 19741, EudraCT Number: 2018-003420-36, Keynote 919, NCI-2019-06734

Population segments: HER2 negative, Hormone refractory, Second line, Stage III, Stage IV

Phase: I

Therapies: BAY-1895344, pembrolizumab

Locations: Germany, Spain, Switzerland, United States

US States: CA, CT, MA, MD, NC, NY, OH, TX

Contact: Bayer Clinical Trials Contact [clinical-trials-contact@bayer.com]

NCT04849364

A Phase II Circulating Tumor DNA Enriched, Genomically Directed Post-neoadjuvant Trial for Patients With Residual Triple Negative Breast Cancer (PERSEVERE)

Cancer type: Triple Negative Breast Cancer

Variant class: DNA repair pathway

Other identifiers: HCRN BRE18-334, PERSEVERE

Population segments: Adjuvant, HER2 negative, Stage I, Stage II, Stage III, Triple receptor negative

Phase: II

Therapies: talazoparib, chemotherapy

Location: United States

US State: IN

Contact: Dr. Bryan P. Schneider [317-948-3885; bpschnei@iu.edu]

CDK12 truncating mutation

NCT04550494

A Pharmacodynamics-Driven Trial of Talazoparib, an Oral PARP Inhibitor, in Patients With Advanced Solid Tumors and Aberrations in Genes Involved in DNA Damage Response

Cancer type: Unspecified Solid Tumor

Variant class: CDK12 truncating mutation

Other identifiers: 000246, 000264-C, 10000264, 10371, NCI-2020-06906

Population segments: BRCA, Fourth line or greater, HER2 positive, Hormone refractory, Second line, Stage II, Stage III, Stage IV, Third line

Phase: II

Therapy: talazoparib

Location: United States

US State: MD

Contact: Multiple contacts: See www.clinicaltrials.gov for complete list of contacts.

Disclaimer: The data presented here is from a curated knowledgebase of publicly available information, but may not be exhaustive. The data version is 2021.11(003).

CDK12 truncating mutation (continued)

NCT02693535
Targeted Agent and Profiling Utilization Registry (TAPUR) Study.

Cancer type: Unspecified Solid Tumor

Variant class: CDK12 mutation

Other identifiers: 20170529, NCI-2017-00510, Pro00014171, TAPUR

Population segments: Aggressive, BRAF, Diffuse large B-cell lymphoma (DLBCL), Extranodal marginal zone B-cell lymphoma (MALT), FGFR, First line, Follicular lymphoma (FL), Fourth line or greater, Indolent, Lymphoblastic lymphoma (LBL), Mantle cell lymphoma (MCL), Other subtype, Second line, Small lymphocytic lymphoma (SLL), Stage III, Stage IV, Third line, Waldenstrom's macroglobulinemia (WM)

Phase: II

Therapy: atezolizumab + talazoparib

Location: United States

US States: AL, AZ, CA, FL, GA, HI, IL, IN, ME, MI, NC, ND, NE, NH, OH, OR, PA, SD, TX, UT, VA, WA

Contact: Pam Mangat [pam.mangat@asco.org]

NCT03742895
A Phase II Study of Olaparib Monotherapy in Participants With Previously Treated, Homologous Recombination Repair Mutation (HRRm) or Homologous Recombination Deficiency (HRD) Positive Advanced Cancer

Cancer type: Unspecified Solid Tumor

Variant class: CDK12 mutation

Other identifiers: (MK-7339-002 / LYNK-002), 19-139, 194694, 2018-01891, 7339-002, 7339-002-00, EudraCT Number: 2018-003007-19, IRAS ID: 255158, JapicCTI-194694, LYNK002, LYNK-002, MK7339-002, MK-7339-002, MK7339-002-00, NCI-2018-03519, PER-046-18, SNCTP000003157

Population segments: BRCA, Fourth line or greater, Second line, Stage III, Stage IV, Third line

Exclusion criteria variant classes: BRCA germline mutation (Breast Cancer, Ovarian Cancer), BRCA mutation (Ovarian Cancer)

Phase: II

Therapy: olaparib

Locations: Argentina, Australia, Canada, Colombia, Denmark, France, Guatemala, Ireland, Israel, Italy, Mexico, Peru, Republic of Korea, Romania, Russian Federation, Spain, Switzerland, Turkey, United Kingdom, United States

US States: AZ, CA, CO, GA, KY, MA, MD, MI, NE, NJ, NY, SD, UT, WA

Contact: Toll Free Number [888-577-8839; Trialsites@merck.com]

NCT03967938
Efficacy of Olaparib in Advanced Cancers Occurring in Patients With Germline Mutations or Somatic Tumor Mutations in Homologous Recombination Genes

Cancer type: Unspecified Solid Tumor

Variant class: CDK12 mutation

Other identifiers: 1-2018 BSMO, 1-2018BSMO, EudraCT Number: 2018-002966-37, Precision 2 - Olaparib

Population segments: Line of therapy N/A, Stage III, Stage IV

Exclusion criteria variant classes: BRCA1 germline mutation (Breast Cancer, Pancreatic Cancer, Prostate Cancer), BRCA2 germline mutation (Breast Cancer, Pancreatic Cancer, Prostate Cancer)

Phase: II

Therapy: olaparib

Location: Belgium

Disclaimer: The data presented here is from a curated knowledgebase of publicly available information, but may not be exhaustive. The data version is 2021.11(003).

CDK12 truncating mutation (continued)

NCT03842228

A Phase Ib Biomarker-Driven Combination Trial of Copanlisib, Olaparib, and Durvalumab (MEDI4736) in Patients With Advanced Solid Tumors

Cancer type: Unspecified Solid Tumor

Variant class: CDK12 mutation

Other identifiers: 10217, 19-628, NCI10217, NCI-2019-00601

Population segments: Line of therapy N/A, Stage III, Stage IV

Phase: I

Therapies: copanlisib, olaparib, durvalumab

Location: United States

US States: MA, TX

Contact: Multiple contacts: See www.clinicaltrials.gov for complete list of contacts.

NCT04586335

Open Label, Phase Ib Study to Evaluate the Safety, Tolerability, Pharmacokinetics and Clinical Activity of CYH33, an Oral a-specific PI3K Inhibitor in Combination With Olaparib, an Oral PARP Inhibitor in Patients With Advanced Solid Tumors.

Cancer type: Breast Cancer, Endometrial Carcinoma, Ovarian Cancer, Prostate Cancer, Unspecified Solid Tumor

Variant class: CDK12 mutation

Other identifiers: CTR20202339, CYH33-G102

Population segments: BRCA, Second line, Stage III, Stage IV

Phase: I

Therapies: HH-CYH33, olaparib

Locations: Australia, United States

US State: TX

Contact: Dr. Jason Sudia [908-380-1329; jason.sudia@haihepharma.com]

NCT02029001

A Two-period, Multicenter, Randomized, Open-label, Phase II Study Evaluating the Clinical Benefit of a Maintenance Treatment Targeting Tumor Molecular Alterations in Patients with Progressive Locally-advanced or Metastatic Solid Tumors.

Cancer type: Unspecified Solid Tumor

Variant class: HRR mutation

Other identifiers: ET12-081, EudraCT Number: 2012-004510-34, MOST, ProfiLER

Population segments: Maintenance/Consolidation, Second line, Stage III, Stage IV, Third line

Exclusion criteria variant classes: ATM mutation (Gastric Cancer), BRCA1 germline mutation (Gastric Cancer), BRCA1 mutation (Gastric Cancer), BRCA2 germline mutation (Gastric Cancer), BRCA2 mutation (Gastric Cancer), HRR mutation (Gastric Cancer), PALB2 mutation (Gastric Cancer), RAD51 mutation (Gastric Cancer)

Phase: II

Therapy: olaparib

Location: France

Disclaimer: The data presented here is from a curated knowledgebase of publicly available information, but may not be exhaustive. The data version is 2021.11(003).

CDK12 truncating mutation (continued)

NCT04123366
A Phase II Study of Olaparib in Combination With Pembrolizumab in Participants With Previously Treated, Homologous Recombination Repair Mutation (HRRm) and/or Homologous Recombination Deficiency (HRD)-Positive Advanced Cancer

Cancer type: Unspecified Solid Tumor

Variant class: HRR mutation

Other identifiers: 7339-007, EudraCT Number: 2019-001745-40, jRCT2011200025, KEYLYNK-007, MK-7339-007, NCI-2019-08758, PER-029-19

Population segments: BRCA, Second line, Stage III, Stage IV

Exclusion criteria variant classes: BRCA germline mutation (Breast Cancer, Ovarian Cancer), BRCA mutation (Breast Cancer, Ovarian Cancer)

Phase: II

Therapies: olaparib, pembrolizumab

Locations: Argentina, Australia, Canada, Colombia, France, Germany, Guatemala, Israel, Italy, Japan, Latvia, Mexico, Peru, Poland, Puerto Rico, Republic of Korea, Romania, South Africa, Spain, Sweden, Turkey, Ukraine, United States

US States: AL, AZ, CA, CO, FL, GA, KY, NJ, NY, OH, TX, UT, VA

Contact: Toll Free Number [888-577-8839; Trialsites@merck.com]

NCT02401347
A Phase II Clinical Trial of the PARP Inhibitor Talazoparib in BRCA1 and BRCA2 Wild Type Patients With Advanced Triple Negative Breast Cancer and Homologous Recombination Deficiency or Advanced HER2 Negative Breast Cancer or Other Solid Tumors With a Mutation in Homologous Recombination Pathway Genes Talazoparib Beyond BRCA (TBB) Trial

Cancer type: Breast Cancer, Unspecified Solid Tumor

Variant class: HRR mutation

Other inclusion criteria: ERBB2 negative, Hormone receptor positive

Other identifiers: BRS0050, NCI-2015-00036, TBB

Population segments: Estrogen receptor positive, HER2 negative, Progesterone receptor positive, Second line, Stage III, Stage IV, Triple receptor negative

Exclusion criteria variant classes: BRCA1 germline mutation (Breast Cancer, Triple Negative Breast Cancer, Unspecified Solid Tumor), BRCA1 mutation (Breast Cancer, Triple Negative Breast Cancer, Unspecified Solid Tumor), BRCA2 germline mutation (Breast Cancer, Triple Negative Breast Cancer, Unspecified Solid Tumor), BRCA2 mutation (Breast Cancer, Triple Negative Breast Cancer, Unspecified Solid Tumor), ERBB2 positive (Breast Cancer)

Phase: II

Therapy: talazoparib

Location: United States

US State: CA

Contact: Amanda Selin [650-721-6977; aselin@stanford.edu]

NCT04497116
Phase I/IIa Study of the Safety, Pharmacokinetics, Pharmacodynamics and Preliminary Clinical Activity of RP-3500 Alone or in Combination With Talazoparib in Advanced Solid Tumors With ATR Inhibitor Sensitizing Mutations (TRESR Study)

Cancer type: Unspecified Solid Tumor

Variant class: CDK12 aberration

Other identifiers: 20-521, EudraCT Number: 2020-000301-87, IRAS ID: 286609, NCI-2020-06869, RP-3500-01, TRESR, TRESR Study

Population segments: Second line, Stage III, Stage IV

Phase: I/II

Therapies: RP-3500, talazoparib

Locations: Canada, Denmark, United Kingdom, United States

US States: MA, NC, NY, RI, TN, TX

Contact: Dr. Peter Manley [857-322-5553; pmanley@reparerx.com]

CDK12 truncating mutation (continued)

NCT03415659
A Phase I, Open-label, Single-center, Single/Multiple-dose, Dose-escalation/ Dose-expansion Clinical Study on Tolerance and Pharmacokinetics of HWH340 Tablet in Patients With Advanced Solid Tumors

Cancer type: Unspecified Solid Tumor

Variant class: HRR mutation

Other inclusion criteria: BRCA1 germline mutation negative, BRCA1 mutation negative, BRCA2 germline mutation negative, BRCA2 mutation negative

Other identifiers: CTR20171649, HWH340-RFPA 20170821, RFPA 20170821

Population segments: BRCA, Fourth line or greater, HER2 negative, Second line, Stage III, Stage IV, Third line

Phase: I

Therapy: HWH-340

Location: China

NCT03767075
Basket of Baskets: A Modular, Open-label, Phase II, Multicentre Study To Evaluate Targeted Agents in Molecularly Selected Populations With Advanced Solid Tumours

Cancer type: Unspecified Solid Tumor

Variant class: DNA repair mutation

Other identifiers: (Basket of Baskets) (BoB), EudraCT number: 2017-005108-89, MO39164, NL65937.031.18, VHIO17002

Population segments: Second line, Stage III, Stage IV

Phase: II

Therapy: atezolizumab

Locations: France, Germany, Netherlands, Spain, Sweden, United Kingdom

---

NCT04905914
A Phase I/IIa, Open-Label, Safety, Pharmacokinetic, And Preliminary Efficacy Study Of Oral ATRN-119 In Patients With Advanced Solid Tumors

Cancer type: Unspecified Solid Tumor

Variant class: DNA repair mutation

Other identifier: AR-276-01

Population segments: Line of therapy N/A, Stage III, Stage IV

Phase: I/II

Therapy: ATRN-119

Location: United States

US State: MA

Contact: Robert Hasson [619-540-6253; rhasson@pacificlinkconsulting.com]

Disclaimer: The data presented here is from a curated knowledgebase of publicly available information, but may not be exhaustive. The data version is 2021.11(003).

CDK12 truncating mutation (continued)

NCT04901702

A Randomized Phase I/II Study of Talazoparib or Temozolomide in Combination With Onivyde in Children With Recurrent Solid Malignancies and Ewing Sarcoma

Cancer type: Unspecified Solid Tumor

Variant class: HRR pathway

Other identifiers: EudraCT Number: 2021-003570-31, ONITT

Population segments: Second line, Stage IV

Exclusion criteria variant classes: HRR germline pathway (Central Nervous System Neoplasm, Ewing Sarcoma), HRR pathway (Central Nervous System Neoplasm, Ewing Sarcoma)

Phase: I/II

Therapies: talazoparib, chemotherapy

Locations: Canada, United States

US State: TN

Contact: Dr. Sara Federico [866-278-5833; referralinfo@stjude.org]

NCT03155620

NCI-COG Pediatric MATCH (Molecular Analysis for Therapy Choice) Screening Protocol

Cancer type: Unspecified Solid Tumor

Variant class: DNA repair pathway

Other identifiers: 17-729, 2017-0625, 20170841, APEC1621SC, COGAPEC1621, COGAPEC1621SC, N 55017 SC, NCI-2017-01251, NCI-COG Pediatric MATCH, Pediatric MATCH

Population segments: ALK, Aggressive, BRAF, FGFR, HRAS, Indolent, KRAS, NRAS, Pediatric or Adolescent, Second line, Stage III, Stage IV

Phase: II

Therapy: olaparib

Locations: Puerto Rico, United States

US States: AK, AL, AR, AZ, CA, CO, CT, DC, DE, FL, GA, HI, IA, ID, IL, IN, KY, LA, MA, MD, ME, MI, MN, MO, MS, NC, NE, NH, NJ, NV, NY, OH, OK, OR, PA, RI, SC, SD, TN, TX, UT, VA, VT, WA, WI

Contact: Multiple contacts: See www.clinicaltrials.gov for complete list of contacts.

NCT03233204

NCI-COG Pediatric MATCH (Molecular Analysis for Therapy Choice)- Phase 2 Subprotocol of Olaparib in Patients With Tumors Harboring Defects in DNA Damage Repair Genes

Cancer type: Unspecified Solid Tumor

Variant class: DNA repair pathway

Other identifiers: 20170841, APEC1621H, NCI-2017-00766

Population segments: Aggressive, Indolent, Pediatric or Adolescent, Second line, Stage III, Stage IV

Phase: II

Therapy: olaparib

Locations: Puerto Rico, United States

US States: AK, AL, AR, AZ, CA, CO, DC, DE, FL, GA, IA, ID, IL, IN, KY, LA, MD, ME, MI, MN, MO, MS, NC, ND, NE, NJ, NY, OH, OK, OR, PA, SC, SD, TN, TX, UT, VA, VT, WA, WI

Contact: Multiple contacts: See www.clinicaltrials.gov for complete list of contacts.

Disclaimer: The data presented here is from a curated knowledgebase of publicly available information, but may not be exhaustive. The data version is 2021.11(003).

CDK12 truncating mutation (continued)

NCT04267939
An Open-label Phase Ib Study to Determine the Maximum Tolerated and/or Recommended Phase II Dose of the ATR Inhibitor BAY 1895344 in Combination With PARP Inhibitor Niraparib, in Patients With Recurrent Advanced Solid Tumors and Ovarian Cancer

Cancer type: Ovarian Cancer, Unspecified Solid Tumor

Variant class: DNA repair pathway

Other identifiers: 18595, 19-810, 2019-0471, 20-222, EudraCT Number: 2018-003930-34, NCI-2020-01405

Population segments: Maintenance/Consolidation, Second line, Stage III, Stage IV

Exclusion criteria variant class: DNA repair pathway (Prostate Cancer)

Phase: I

Therapies: BAY-1895344, niraparib

Location: United States

US States: MA, NY, TX

Contact: Bayer Clinical Trials Contact [888-842-2937; clinical-trials-contact@bayer.com]

NCT04693468
Modular Phase IB Hypothesis-Testing, Biomarker-Driven, Talazoparib Combination Trial (TalaCom)

Cancer type: Unspecified Solid Tumor

Variant class: DNA repair pathway

Other identifiers: 2020-0436, NCI-2020-06041, TalaCom

Population segments: Hormone refractory, Second line, Stage III, Stage IV

Phase: I

Therapies: talazoparib, palbociclib, axitinib, crizotinib

Location: United States

US State: TX

Contact: Timothy A. Yap [713-563-1784; tyap@mdanderson.org]

NCT03810105
A Phase II Study of Olaparib and Durvalumab in Men With Castration Sensitive Biochemically Recurrent Non-Metastatic Prostate Cancer Harboring Mutations in DNA Damage Repair

Cancer type: Prostate Cancer

Variant class: CDK12 truncating mutation

Other identifiers: 18-480, NCI-2019-01627

Population segments: Adjuvant, Stage I, Stage II, Stage III

Phase: II

Therapies: durvalumab, olaparib

Location: United States

US States: CA, IL, MI, NJ, NY

Contact: Dr. Karen Autio [646-422-4632; autiok@mskcc.org]

Disclaimer: The data presented here is from a curated knowledgebase of publicly available information, but may not be exhaustive. The data version is 2021.11(003).

CDK12 truncating mutation (continued)

NCT03840967
A Phase II Study Evaluating Safety and Efficacy of Niraparib in Patients With Previously Treated Homologous Recombination (HR) Defective or Loss of Heterozygosity (LOH) High Metastatic Esophageal/Gastroesophageal Junction/ Proximal Gastric Adenocarcinoma

Cancer type: Esophageal Cancer, Gastric Cancer, Gastroesophageal Junction Adenocarcinoma

Variant class: CDK12 truncating mutation

Other identifiers: 20166, 71618, HCRN ESO17-325, NCI-2019-07395

Population segments: Second line, Stage III, Stage IV

Phase: II

Therapy: niraparib

Location: United States

US States: FL, IN, MI, NY

Contact: Dr. Shadia Jalal [317-274-3658; sjalal@iu.edu]

NCT04751929
A Phase II Multi-Center Trial of Abemaciclib With or Without Atezolizumab in Metastatic Castration Resistant Prostate Cancer

Cancer type: Castration-Resistant Prostate Cancer

Variant class: CDK12 mutation

Other identifier: 20-701

Population segments: Hormone refractory, Second line, Stage IV

Phase: II

Therapies: abemaciclib, atezolizumab

Location: United States

US State: MA

Contact: Dr. Atish Choudhury [617-632-6328; achoudhury@partners.org]

NCT04336943
Durvalumab (MEDI4736) and Olaparib (AZD2281) for Treatment of Biochemically Recurrent Prostate Cancer in Men Predicted to Have a High Neoantigen Load: A Pilot Study

Cancer type: Prostate Adenocarcinoma

Variant class: CDK12 mutation

Other identifiers: 10509, NCI-2020-01860, RG1007001

Population segments: Line of therapy N/A, Stage I, Stage II, Stage III

Phase: II

Therapies: durvalumab, olaparib

Location: United States

US State: WA

Contact: Michael Schweizer [206-606-6252; schweize@uw.edu]

Disclaimer: The data presented here is from a curated knowledgebase of publicly available information, but may not be exhaustive. The data version is 2021.11(003).

CDK12 truncating mutation (continued)

NCT04053322
An International Multicenter Phase II Trial of Durvalumab (MEDI4736) Plus OLAparib Plus Fulvestrant in Metastatic or Locally Advanced ER-positive, HER2-negative Breast Cancer Patients Selected Using Criteria That Predict Sensitivity to Olaparib

Cancer type: Breast Cancer

Variant class: CDK12 mutation

Other inclusion criteria: ERBB2 negative, ER positive

Other identifiers: CADUSEIME08, DOLAF, EudraCT Number: 2018-003832-57, UC-0140/1812

Population segments: BRCA, Estrogen receptor positive, FGFR, HER2 negative, Progesterone receptor positive, Second line, Stage III, Stage IV

Phase: II

Therapies: durvalumab, olaparib, hormone therapy

Location: France

NCT05011383
High-dose Testosterone in Men With Metastatic Castration-resistant Prostate Cancer and ATM or CDK12 Deficiency

Cancer type: Castration-Resistant Prostate Cancer

Variant class: CDK12 mutation

Other identifiers: SPLP-003-20F, VA-BAT

Population segments: Hormone refractory, Second line, Stage IV

Phase: II

Therapy: hormone therapy

Location: United States

US State: WA

Contact: Dr. Robert B. Montgomery [206-277-6878; Robert.Montgomery@va.gov]

NCT03553004
Niraparib in Metastatic Pancreatic Cancer After Previous Chemotherapy (NIRA-PANC): A Phase II Trial

Cancer type: Pancreatic Cancer

Variant class: CDK12 mutation

Other identifiers: IIT-2017-NIRA-PANC, NIRA-PANC, STUDY00141862

Population segments: BRCA, Second line, Stage IV, Third line

Phase: II

Therapy: niraparib

Location: United States

US States: KS, MO

Contact: Clinical Trials Nurse Navigator [913-588-3671; navigation@kumc.edu]

Disclaimer: The data presented here is from a curated knowledgebase of publicly available information, but may not be exhaustive. The data version is 2021.11(003).

CDK12 truncating mutation (continued)

NCT04030559
Phase II Trial of PARP Inhibitor Niraparib for Men With High Risk Prostate Cancer and DNA Damage Response Defects

Cancer type: Prostate Cancer

Variant class: CDK12 mutation

Other identifiers: 1412676, NCI-2019-04365, UCDCC#279

Population segments: First line, Neoadjuvant, Stage I, Stage II, Stage IV

Phase: II

Therapy: niraparib

Location: United States

US State: CA

Contact: Marc Dall'Era [916-734-3771; mdallera@ucdavis.edu]

NCT04019964
Nivolumab as a Non-Castrating Therapy for MMR-deficient and CDK12- Altered Prostate Cancer With PSA Recurrence After Local Therapy

Cancer type: Prostate Cancer

Variant class: CDK12 mutation

Other identifiers: CA209-76M, CRMS-71513, J1933, NCI-2020-03804

Population segments: (N/A), Hormone refractory, Second line

Phase: II

Therapy: nivolumab

Location: United States

US State: MD

Contact: Rana Sullivan [410-614-6337; tomalra@jhmi.edu]

NCT03012321
BRCAAway: A Randomized Phase II Trial of Abiraterone, Olaparib, or Abiraterone + Olaparib in Patients With Metastatic Castration-Resistant Prostate Cancer With DNA Repair Defects

Cancer type: Castration-Resistant Prostate Cancer

Variant class: CDK12 mutation

Other identifiers: 19137, BRCAAway, NCI-2016-01834, NU 16U05, NU_16U05, PCCTC #: c16-168, PCCTC c16-168, STU00203960

Population segments: First line, Hormone refractory, Stage IV

Phase: II

Therapy: olaparib

Location: United States

US States: CA, FL, IL, IN, MD, MI, MN, MO, NC, NY, UT, VA

Contact: Study Coordinator [312-695-1301; cancertrials@northwestern.edu]

NCT03786796
Phase II Study of Olaparib in Metastatic Renal Cell Carcinoma Patients Harboring a BAP-1 or Other DNA Repair Gene Mutations (ORCHID)

Cancer type: Kidney Cancer

Variant class: CDK12 mutation

Other identifiers: CRMS-70750, J18166, NCI-2019-06309, ORCHID

Population segments: Fourth line or greater, Second line, Stage IV, Third line

Phase: II

Therapy: olaparib

Location: United States

US State: MD

Contact: Irina Rifkind [410-502-2043; irifkin1@jhmi.edu]

Disclaimer: The data presented here is from a curated knowledgebase of publicly available information, but may not be exhaustive. The data version is 2021.11(003).

CDK12 truncating mutation (continued)

NCT04515836
Phase II Trial of Olaparib in Homologous Recombination Deficient (HRD) Malignant Mesothelioma

Cancer type: Mesothelioma

Variant class: CDK12 mutation

Population segments: (N/A), Second line

Phase: II

Therapy: olaparib

Location: United States

US State: IL

Contact: Dr. Hedy L. Kindler [773-702-0360; hkindler@medicine.bsd.uchicago.edu]

NCT04104893
A Phase II Study Of A Checkpoint Inhibitor In Men With Progressive Metastatic Castrate Resistant Prostate Cancer Characterized By A Mismatch Repair Deficiency Or Biallelic CDK12 Inactivation

Cancer type: Castration-Resistant Prostate Cancer

Variant class: CDK12 mutation

Other identifiers: CHOMP, CSDR-003-18F, NCI-2020-02215

Population segments: First line, Hormone refractory, Stage IV

Phase: II

Therapy: pembrolizumab

Location: United States

US States: CA, FL, IL, MI, NC, NY, WA

Contact: Dr. Matthew B. Rettig [310-478-3711; matthew.rettig@va.gov]

NCT03533946
A Phase II Study of Rucaparib Monotherapy in Nonmetastatic, Hormone-Sensitive Prostate Cancer Demonstrating "BRCAness" Genotype (ROAR)

Cancer type: Prostate Cancer

Variant class: CDK12 mutation

Other identifiers: HCI111833, NCI-2018-01754, ROAR

Population segments: First line, Stage I, Stage II, Stage III

Phase: II

Therapy: rucaparib

Location: United States

US State: UT

Contact: Jill Broghammer [801-213-6232; jill.broghammer@hci.utah.edu]

NCT04892693
Talazoparib in Advanced Breast Cancer Patients With Homologous Recombinant Deficiency: A Phase II Clinical and Exploratory Biomarker Study of Talazoparib

Cancer type: Breast Cancer

Variant class: CDK12 mutation

Other identifier: H-2009-079-1157

Population segments: BRCA, Line of therapy N/A, Stage III, Stage IV

Phase: II

Therapy: talazoparib

Location: Republic of Korea

Disclaimer: The data presented here is from a curated knowledgebase of publicly available information, but may not be exhaustive. The data version is 2021.11(003).

CDK12 truncating mutation (continued)

NCT04740190

Combination Talazoparib - Carboplatin for Recurrent High-grade Glioma With DNA Damage Repair Deficiency (DDRd)

Cancer type: Anaplastic Astrocytoma, Anaplastic Oligoastrocytoma, Anaplastic Oligodendroglioma, Anaplastic Pleomorphic Xanthoastrocytoma, Diffuse Midline Glioma, Glioblastoma

Variant class: CDK12 mutation

Other identifiers: TAC-GReD, UW-20396

Population segments: (N/A), BRCA, Second line

Phase: II

Therapies: talazoparib, chemotherapy

Location: Hong Kong

NCT04253262

A Phase Ib/II Study of Copanlisib Combined With Rucaparib in Patients With Metastatic Castration-resistant Prostate Cancer

Cancer type: Castration-Resistant Prostate Cancer

Variant class: CDK12 mutation

Other identifiers: BrUOG 360, BrUOG360

Population segments: Hormone refractory, Second line, Stage IV

Phase: I/II

Therapies: copanlisib, rucaparib

Location: United States

US State: RI

Contact: BrUOG [401-863-3000; BrUOG@brown.edu]

NCT04169841

Precision Medicine Phase II Study Evaluating the Efficacy of a Double Immunotherapy by Durvalumab and Tremelimumab Combined With Olaparib in Patients With Solid Cancers and Carriers of Homologous Recombination Repair Genes Mutation in Response or Stable After Olaparib Treatment

Cancer type: Breast Cancer, Endometrial Carcinoma, Head and Neck Cancer, Kidney Cancer, Non-Small Cell Lung Cancer, Ovarian Cancer, Pancreatic Cancer, Prostate Cancer

Variant class: HRR mutation

Other identifier: GUIDE2REPAIR

Population segments: Fourth line or greater, Hormone refractory, Maintenance/Consolidation, Second line, Stage III, Stage IV, Third line

Exclusion criteria variant classes: ALK fusion (Lung Cancer), BRAF mutation (Lung Cancer), EGFR mutation (Lung Cancer), ROS1 mutation (Lung Cancer)

Phase: II

Therapies: durvalumab, tremelimumab, olaparib

Location: France

Disclaimer: The data presented here is from a curated knowledgebase of publicly available information, but may not be exhaustive. The data version is 2021.11(003).

CDK12 truncating mutation (continued)

NCT03925350
A Phase II Study of Niraparib in Patients With Advanced Melanoma With Genetic Homologous Recombination (HR) Mutation / Alteration

Cancer type: Melanoma

Variant class: HRR mutation

Other identifier: CPMC17-MEL01

Population segments: Second line, Stage III, Stage IV

Phase: II

Therapy: niraparib

Location: United States

US State: CA

Contact: Laurel Brechtel [415-600-1654; BrechtLA@sutterhealth.org]

NCT04940637
A Phase II, Open-Label, Single Arm, Prospective, Multicenter Study of Niraparib Plus Dostarlimab in Patients With Advanced NSCLC and/or MPM, and Positive for PD-L1 Expression and Germline or Somatic Mutations in the HRR Genes

Cancer type: Mesothelioma, Non-Small Cell Lung Cancer

Variant class: HRR mutation

Other inclusion criteria: ALK fusion negative, CD274 expression, EGFRi sensitizing mutation negative, ROS1 fusion negative or CD274 expression

Other identifiers: EudraCT Number: 2020-000109-10, UNITO-001, UNITO-001/2020

Population segments: Second line, Stage III, Stage IV

Exclusion criteria variant classes: ALK fusion (Non-Small Cell Lung Cancer), EGFRi sensitizing mutation (Non-Small Cell Lung Cancer), ROS1 fusion (Non-Small Cell Lung Cancer)

Phase: II

Therapies: niraparib, dostarlimab

Location: Italy

NCT04042831
A Phase II Study of Olaparib in Patients With Advanced Biliary Tract Cancer With Aberrant DNA Repair Gene Mutations

Cancer type: Biliary Tract Carcinoma

Variant class: HRR mutation

Other identifiers: ACCRU-ICRN-1702, NCI-2019-04434

Population segments: Second line, Stage IV

Phase: II

Therapy: olaparib

Location: United States

US States: AZ, MN, NY, TX

Contact: Multiple contacts: See www.clinicaltrials.gov for complete list of contacts.

Disclaimer: The data presented here is from a curated knowledgebase of publicly available information, but may not be exhaustive. The data version is 2021.11(003).

CDK12 truncating mutation (continued)

NCT04939662

Phase II, Single-arm Study of Olaparib and Bevacizumab Combination Therapy in Relapsed Small Cell Lung Cancer Subjects With DNA Damage Response and the Repair Pathway Alteration, ATM Deficiency, SLFN11 Positive, or POU2F3 Positive

Cancer type: Small Cell Lung Cancer

Variant class: HRR mutation

Other identifier: SUKSES-B2

Population segments: (N/A), BRCA, Pulmonary, Second line, Third line

Phase: II

Therapies: olaparib, bevacizumab

Location: Republic of Korea

NCT03506997

PERSEUS1: Phase II Trial of the Immune Checkpoint Inhibitor Pembrolizumab for Patients Suffering From Metastatic Prostate Cancer

Cancer type: Castration-Resistant Prostate Cancer

Variant class: HRR mutation

Other identifiers: CCR4559, EudraCT Number: 2017-000931-15, ICR-CTSU/2016/10060, IRAS ID 208952, PERSEUS1

Population segments: Hormone refractory, Second line, Stage IV

Phase: II

Therapy: pembrolizumab

Location: United Kingdom

NCT02264678

A Modular Phase I, Open-Label, Multicentre Study to Assess the Safety, Tolerability, Pharmacokinetics and Preliminary Anti-tumour Activity of Ceralasertib in Combination With Cytotoxic Chemotherapy and/or DNA Damage Repair/Novel Anti-cancer Agents in Patients With Advanced Solid Malignancies

Cancer type: Triple Negative Breast Cancer

Variant class: HRR mutation

Other inclusion criteria: BRCA germline mutation negative, BRCA mutation negative

Other identifiers: 0C-14-9, 16-031, 18-342, D5330C00004, EudraCT Number: 2014-002233-66, IRAS ID 160200, NCI-2015-00687, Study 4

Population segments: Adenocarcinoma, BRCA, HER2 negative, Pulmonary, Second line, Stage III, Stage IV, Third line, Triple receptor negative

Exclusion criteria variant class: ERBB2 positive (Breast Cancer)

Phase: I

Therapies: ceralasertib, olaparib

Locations: France, Republic of Korea, United Kingdom, United States

US States: CA, MA, NY

Contact: AstraZeneca Clinical Study Information Center [877-240-9479; information.center@astrazeneca.com]

NCT04890613

Phase Ib Expansion Study of CX-5461 in Patients With Solid Tumours and BRCA2 and/or PALB2 Mutation

Cancer type: Ovarian Cancer

Variant class: HRR mutation

Other identifier: CX-5461-04

Population segments: BRCA, Second line, Stage II, Stage III, Stage IV

Phase: I

Therapy: pidnarulex

Location: Canada

Disclaimer: The data presented here is from a curated knowledgebase of publicly available information, but may not be exhaustive. The data version is 2021.11(003).

CDK12 truncating mutation (continued)

NCT05002868

A Multi-center, Open-label, Phase I/Ib Study to Assess the Safety, Pharmacokinetics and Anti-tumor Activity of RP12146, a Poly (ADP-ribose) Polymerase (PARP) Inhibitor, in Patients With Locally Advanced or Metastatic Solid Tumors

Cancer type: Biliary Tract Carcinoma, Breast Cancer, Colorectal Cancer, Endometrial Carcinoma, Gastric Cancer, Ovarian Cancer, Pancreatic Cancer, Prostate Cancer, Small Cell Lung Cancer

Variant class: HRR mutation

Other identifier: RP12146-2101

Population segments: Extensive, Pulmonary, Second line, Stage III, Stage IV

Exclusion criteria variant class: ERBB2 positive (Breast Cancer)

Phase: I

Therapy: RP12146

Location: Poland

NCT03395197

A Phase III, Randomized, Double-blind, Placebo-controlled Study Of Talazoparib With Enzalutamide In Metastatic Castration-resistant Prostate Cancer

Cancer type: Castration-Resistant Prostate Cancer

Variant class: DNA repair mutation

Other identifiers: BASIC-ROYAL / HIGH-ROYAL, C3441021, CSET 2938, CTR20191443, CTR20191444, EudraCT Number: 2017-003295-31, GU 170 - C3441021, HCC 19-054, JapicCTI-194665, LH19.022, MOH_2019-05-16_007127, NCI-2018-01651, Pfizer C3441021, R19062M, RECF3951, RWF_C3441021 TALAPRO-2, TALAPRO-2, TX228665, UCI-18-47, VICCURO17115

Population segments: First line, Hormone refractory, Second line, Stage III, Stage IV

Phase: III

Therapies: talazoparib, hormone therapy

Locations: Argentina, Australia, Belgium, Brazil, Canada, Chile, China, Czech Republic, Finland, France, Germany, Hungary, Israel, Italy, Japan, New Zealand, Norway, Peru, Poland, Portugal, Republic of Korea, South Africa, Spain, Sweden, United Kingdom, United States

US States: AZ, CA, CO, FL, GA, IL, IN, KS, MO, NE, NJ, NY, OH, OK, OR, PA, SC, TN, TX, UT, VA, WI

Contact: Pfizer CT.gov Call Center [800-718-1021; ClinicalTrials.gov_Inquiries@pfizer.com]

NCT04821622

TALAPRO-3: A Phase III, Randomized, Double-blind, Study of Talazoparib With Enzalutamide Versus Placebo With Enzalutamide in Men With DDR Gene Mutated Metastatic Castration-sensitive Prostate Cancer

Cancer type: Prostate Adenocarcinoma

Variant class: DNA repair mutation

Other identifiers: C3441052, CTR20212191, EudraCT Number: 2021-000248-23, jRCT2031210098, TALAPRO-3

Population segments: Second line, Stage IV

Phase: III

Therapies: talazoparib, hormone therapy

Locations: Japan, Republic of Korea, United States

US States: CA, DC, FL, GA, IL, MI, NJ, NY, PA, SC, TX, UT

Contact: Pfizer CT.gov Call Center [800-718-1021; ClinicalTrials.gov_Inquiries@pfizer.com]

Disclaimer: The data presented here is from a curated knowledgebase of publicly available information, but may not be exhaustive. The data version is 2021.11(003).

CDK12 truncating mutation (continued)

NCT04187833

Phase II Trial of Nivolumab in Combination with Talazoparib in Patients with Unresectable or Metastatic Melanoma and Mutations in BRCA or BRCA-ness Genes.

Cancer type: Melanoma

Variant class: DNA repair mutation

Other identifiers: CASE2619, NCI-2020-00416

Population segments: Second line, Stage III, Stage IV

Phase: II

Therapies: nivolumab, talazoparib

Location: United States

US State: OH

Contact: Dr. Pauline Funchain [866-223-8100; TaussigResearch@ccf.org]

NCT03344965

A Phase II Study of Olaparib Monotherapy in Metastatic Breast Cancer Patients With Germline or Somatic Mutations in DNA Repair Genes (Olaparib Expanded)

Cancer type: Breast Cancer

Variant class: DNA repair mutation

Other inclusion criteria: BRCA1 germline mutation negative, BRCA2 germline mutation negative

Other identifiers: 17-428, 18-414, NCI-2018-00778, TBCRC 048, TBCRC048

Population segments: BRCA, Estrogen receptor positive, HER2 negative, HER2 positive, Progesterone receptor positive, Second line, Stage IV

Exclusion criteria variant classes: BRCA1 germline mutation (Breast Cancer), BRCA2 germline mutation (Breast Cancer)

Phase: II

Therapy: olaparib

Location: United States

US States: AL, CA, IL, MA, MD, NC, NJ, NY, PA, WA

Contact: Dr. Nadine Tung [617-667-1962; ntung@bidmc.harvard.edu]

NCT03375307

A Phase II Study of Olaparib (AZD2281) in Patients With Metastatic/Advanced Urothelial Carcinoma With DNA-Repair Defects

Cancer type: Bladder Urothelial Carcinoma

Variant class: DNA repair mutation

Other identifiers: 10144, 19C0023, NCI-2017-02296, P162941

Population segments: BRCA, STK11, Second line, Stage III, Stage IV

Phase: II

Therapy: olaparib

Location: United States

US States: CA, CO, KY, MD, NY, OK

Contact: Multiple contacts: See www.clinicaltrials.gov for complete list of contacts.

Disclaimer: The data presented here is from a curated knowledgebase of publicly available information, but may not be exhaustive. The data version is 2021.11(003).

CDK12 truncating mutation (continued)

NCT04038502
An Open-label, Multicenter Phase II Study to Compare the Efficacy of Carboplatin as First-line Followed by Second-line Olaparib Versus Olaparib as First-line Followed by Second-line Carboplatin in the Treatment of Patients With Castration Resistant Prostate Cancer Containing Homologous Recombination Deficiency

Cancer type: Castration-Resistant Prostate Cancer

Variant class: DNA repair mutation

Other identifiers: 01781, 01783, 19-08, BRACeD, CX001963-01, ONCA-009-18F

Population segments: First line, Hormone refractory, Second line, Stage IV

Phase: II

Therapies: olaparib, chemotherapy

Location: United States

US States: CA, DC, FL, IL, MI, NC, NY, PA, WA

Contact: Dr. Robert B. Montgomery [206-277-6878; rbmontgo@u.washington.edu]

NCT04633902
Phase II Study of Olaparib in Combination With Pembrolizumab in Patients With Advanced Melanoma With Homologous Recombination (HR) Pathway Gene Mutation

Cancer type: Melanoma

Variant class: HRR pathway

Other identifier: CPMC19-MEL01

Population segments: BRAF, Second line, Stage III, Stage IV

Phase: II

Therapies: olaparib, pembrolizumab

Location: United States

US State: CA

Contact: Dr. Kevin B. Kim [415-885-8600; ClinicalResearch@sutterhealth.org]

NCT03248570
Phase II Open Label Study of Pembrolizumab in Patients With Metastatic Castration Resistant Prostate Cancer (mCRPC) With or Without DNA Damage Repair Defects

Cancer type: Castration-Resistant Prostate Cancer

Variant class: DNA repair mutation

Other identifiers: 16557, 17-22165, CC 16557, NCI-2017-02408

Population segments: BRCA, Hormone refractory, Line of therapy N/A, Stage IV

Phase: II

Therapy: pembrolizumab

Location: United States

US State: CA

Contact: Patricia Li [415-476-5975; GUTrials@ucsf.edu]

No NCT ID - see other identifier(s)
Single-arm Exploratory Study for Sintilimab Second-line or Later-line Treatment of Advanced Non-small Cell Lung Cancer with DDR Pathway Gene Mutation

Cancer type: Non-Small Cell Lung Cancer

Variant class: DNA repair mutation

Other identifier: ChiCTR1900023234

Population segments: Fourth line or greater, Second line, Stage III, Stage IV, Third line

Exclusion criteria variant classes: ALK fusion (Non-Small Cell Lung Cancer), EGFRi sensitizing mutation (Non-Small Cell Lung Cancer), ROS1 fusion (Non-Small Cell Lung Cancer)

Phase: I/II

Therapy: sintilimab

Location: China

Disclaimer: The data presented here is from a curated knowledgebase of publicly available information, but may not be exhaustive. The data version is 2021.11(003).

CDK12 truncating mutation (continued)

NCT04095273
A Multicenter, Non-randomized, Open-label Phase Ib Study to Determine the Maximum Tolerated and Recommended Phase II Dose of the ATR Inhibitor BAY1895344 in Combination With Pembrolizumab and to Characterize Its Safety, Tolerability, Pharmacokinetics and Preliminary Anti-tumor Activity in Patients With Advanced Solid Tumors

Cancer type: Breast Cancer, Castration-Resistant Prostate Cancer, Colorectal Cancer, Gastric Cancer, Gastroesophageal Junction Adenocarcinoma, Non-Small Cell Lung Cancer, Pancreatic Cancer

Variant class: DNA repair mutation

Other inclusion criteria: ATM mutation negative or ATM mutation negative, ERBB2 negative, Hormone receptor positive or ATM underexpression

Other identifiers: 19741, EudraCT Number: 2018-003420-36, Keynote 919, NCI-2019-06734

Population segments: HER2 negative, Hormone refractory, Second line, Stage III, Stage IV

Phase: I

Therapies: BAY-1895344, pembrolizumab

Locations: Germany, Spain, Switzerland, United States

US States: CA, CT, MA, MD, NC, NY, OH, TX

Contact: Bayer Clinical Trials Contact [clinical-trials-contact@bayer.com]

NCT04849364
A Phase II Circulating Tumor DNA Enriched, Genomically Directed Post-neoadjuvant Trial for Patients With Residual Triple Negative Breast Cancer (PERSEVERE)

Cancer type: Triple Negative Breast Cancer

Variant class: DNA repair pathway

Other identifiers: HCRN BRE18-334, PERSEVERE

Population segments: Adjuvant, HER2 negative, Stage I, Stage II, Stage III, Triple receptor negative

Phase: II

Therapies: talazoparib, chemotherapy

Location: United States

US State: IN

Contact: Dr. Bryan P. Schneider [317-948-3885; bpschnei@iu.edu]

CDK12 deletion

NCT04497116
Phase I/IIa Study of the Safety, Pharmacokinetics, Pharmacodynamics and Preliminary Clinical Activity of RP-3500 Alone or in Combination With Talazoparib in Advanced Solid Tumors With ATR Inhibitor Sensitizing Mutations (TRESR Study)

Cancer type: Unspecified Solid Tumor

Variant class: CDK12 aberration

Other identifiers: 20-521, EudraCT Number: 2020-000301-87, IRAS ID: 286609, NCI-2020-06869, RP-3500-01, TRESR, TRESR Study

Population segments: Second line, Stage III, Stage IV

Phase: I/II

Therapies: RP-3500, talazoparib

Locations: Canada, Denmark, United Kingdom, United States

US States: MA, NC, NY, RI, TN, TX

Contact: Dr. Peter Manley [857-322-5553; pmanley@reparerx.com]

Disclaimer: The data presented here is from a curated knowledgebase of publicly available information, but may not be exhaustive. The data version is 2021.11(003).

CDK12 deletion (continued)

NCT04901702
A Randomized Phase I/II Study of Talazoparib or Temozolomide in Combination With Onivyde in Children With Recurrent Solid Malignancies and Ewing Sarcoma

Cancer type: Unspecified Solid Tumor

Variant class: HRR pathway

Other identifiers: EudraCT Number: 2021-003570-31, ONITT

Population segments: Second line, Stage IV

Exclusion criteria variant classes: HRR germline pathway (Central Nervous System Neoplasm, Ewing Sarcoma), HRR pathway (Central Nervous System Neoplasm, Ewing Sarcoma)

Phase: I/II

Therapies: talazoparib, chemotherapy

Locations: Canada, United States

US State: TN

Contact: Dr. Sara Federico [866-278-5833; referralinfo@stjude.org]

NCT03155620
NCI-COG Pediatric MATCH (Molecular Analysis for Therapy Choice) Screening Protocol

Cancer type: Unspecified Solid Tumor

Variant class: DNA repair pathway

Other identifiers: 17-729, 2017-0625, 20170841, APEC1621SC, COGAPEC1621, COGAPEC1621SC, N 55017 SC, NCI-2017-01251, NCI-COG Pediatric MATCH, Pediatric MATCH

Population segments: ALK, Aggressive, BRAF, FGFR, HRAS, Indolent, KRAS, NRAS, Pediatric or Adolescent, Second line, Stage III, Stage IV

Phase: II

Therapy: olaparib

Locations: Puerto Rico, United States

US States: AK, AL, AR, AZ, CA, CO, CT, DC, DE, FL, GA, HI, IA, ID, IL, IN, KY, LA, MA, MD, ME, MI, MN, MO, MS, NC, NE, NH, NJ, NV, NY, OH, OK, OR, PA, RI, SC, SD, TN, TX, UT, VA, VT, WA, WI

Contact: Multiple contacts: See www.clinicaltrials.gov for complete list of contacts.

NCT03233204
NCI-COG Pediatric MATCH (Molecular Analysis for Therapy Choice)- Phase 2 Subprotocol of Olaparib in Patients With Tumors Harboring Defects in DNA Damage Repair Genes

Cancer type: Unspecified Solid Tumor

Variant class: DNA repair pathway

Other identifiers: 20170841, APEC1621H, NCI-2017-00766

Population segments: Aggressive, Indolent, Pediatric or Adolescent, Second line, Stage III, Stage IV

Phase: II

Therapy: olaparib

Locations: Puerto Rico, United States

US States: AK, AL, AR, AZ, CA, CO, DC, DE, FL, GA, IA, ID, IL, IN, KY, LA, MD, ME, MI, MN, MO, MS, NC, ND, NE, NJ, NY, OH, OK, OR, PA, SC, SD, TN, TX, UT, VA, VT, WA, WI

Contact: Multiple contacts: See www.clinicaltrials.gov for complete list of contacts.

Disclaimer: The data presented here is from a curated knowledgebase of publicly available information, but may not be exhaustive. The data version is 2021.11(003).

CDK12 deletion (continued)

NCT04267939
An Open-label Phase Ib Study to Determine the Maximum Tolerated and/or Recommended Phase II Dose of the ATR Inhibitor BAY 1895344 in Combination With PARP Inhibitor Niraparib, in Patients With Recurrent Advanced Solid Tumors and Ovarian Cancer

Cancer type: Ovarian Cancer, Unspecified Solid Tumor

Variant class: DNA repair pathway

Other identifiers: 18595, 19-810, 2019-0471, 20-222, EudraCT Number: 2018-003930-34, NCI-2020-01405

Population segments: Maintenance/Consolidation, Second line, Stage III, Stage IV

Exclusion criteria variant class: DNA repair pathway (Prostate Cancer)

Phase: I

Therapies: BAY-1895344, niraparib

Location: United States

US States: MA, NY, TX

Contact: Bayer Clinical Trials Contact [888-842-2937; clinical-trials-contact@bayer.com]

NCT04693468
Modular Phase IB Hypothesis-Testing, Biomarker-Driven, Talazoparib Combination Trial (TalaCom)

Cancer type: Unspecified Solid Tumor

Variant class: DNA repair pathway

Other identifiers: 2020-0436, NCI-2020-06041, TalaCom

Population segments: Hormone refractory, Second line, Stage III, Stage IV

Phase: I

Therapies: talazoparib, palbociclib, axitinib, crizotinib

Location: United States

US State: TX

Contact: Timothy A. Yap [713-563-1784; tyap@mdanderson.org]

NCT03810105
A Phase II Study of Olaparib and Durvalumab in Men With Castration Sensitive Biochemically Recurrent Non-Metastatic Prostate Cancer Harboring Mutations in DNA Damage Repair

Cancer type: Prostate Cancer

Variant class: CDK12 deletion

Other identifiers: 18-480, NCI-2019-01627

Population segments: Adjuvant, Stage I, Stage II, Stage III

Phase: II

Therapies: durvalumab, olaparib

Location: United States

US States: CA, IL, MI, NJ, NY

Contact: Dr. Karen Autio [646-422-4632; autiok@mskcc.org]

CDK12 deletion (continued)

NCT03840967

A Phase II Study Evaluating Safety and Efficacy of Niraparib in Patients With Previously Treated Homologous Recombination (HR) Defective or Loss of Heterozygosity (LOH) High Metastatic Esophageal/Gastroesophageal Junction/ Proximal Gastric Adenocarcinoma

Cancer type: Esophageal Cancer, Gastric Cancer, Gastroesophageal Junction Adenocarcinoma

Variant class: CDK12 deletion

Other identifiers: 20166, 71618, HCRN ESO17-325, NCI-2019-07395

Population segments: Second line, Stage III, Stage IV

Phase: II

Therapy: niraparib

Location: United States

US States: FL, IN, MI, NY

Contact: Dr. Shadia Jalal [317-274-3658; sjalal@iu.edu]

NCT04030559

Phase II Trial of PARP Inhibitor Niraparib for Men With High Risk Prostate Cancer and DNA Damage Response Defects

Cancer type: Prostate Cancer

Variant class: CDK12 deletion

Other identifiers: 1412676, NCI-2019-04365, UCDCC#279

Population segments: First line, Neoadjuvant, Stage I, Stage II, Stage IV

Phase: II

Therapy: niraparib

Location: United States

US State: CA

Contact: Marc Dall'Era [916-734-3771; mdallera@ucdavis.edu]

NCT04104893

A Phase II Study Of A Checkpoint Inhibitor In Men With Progressive Metastatic Castrate Resistant Prostate Cancer Characterized By A Mismatch Repair Deficiency Or Biallelic CDK12 Inactivation

Cancer type: Castration-Resistant Prostate Cancer

Variant class: CDK12 deletion

Other identifiers: CHOMP, CSDR-003-18F, NCI-2020-02215

Population segments: First line, Hormone refractory, Stage IV

Phase: II

Therapy: pembrolizumab

Location: United States

US States: CA, FL, IL, MI, NC, NY, WA

Contact: Dr. Matthew B. Rettig [310-478-3711; matthew.rettig@va.gov]

NCT04740190

Combination Talazoparib - Carboplatin for Recurrent High-grade Glioma With DNA Damage Repair Deficiency (DDRd)

Cancer type: Anaplastic Astrocytoma, Anaplastic Oligoastrocytoma, Anaplastic Oligodendroglioma, Anaplastic Pleomorphic Xanthoastrocytoma, Diffuse Midline Glioma, Glioblastoma

Variant class: CDK12 deletion

Other identifiers: TAC-GReD, UW-20396

Population segments: (N/A), BRCA, Second line

Phase: II

Therapies: talazoparib, chemotherapy

Location: Hong Kong

Disclaimer: The data presented here is from a curated knowledgebase of publicly available information, but may not be exhaustive. The data version is 2021.11(003).

CDK12 deletion (continued)

NCT03344965
A Phase II Study of Olaparib Monotherapy in Metastatic Breast Cancer Patients With Germline or Somatic Mutations in DNA Repair Genes (Olaparib Expanded)

Cancer type: Breast Cancer

Variant class: DNA repair deletion

Other inclusion criteria: BRCA1 germline mutation negative, BRCA2 germline mutation negative

Other identifiers: 17-428, 18-414, NCI-2018-00778, TBCRC 048, TBCRC048

Population segments: BRCA, Estrogen receptor positive, HER2 negative, HER2 positive, Progesterone receptor positive, Second line, Stage IV

Exclusion criteria variant classes: BRCA1 germline mutation (Breast Cancer), BRCA2 germline mutation (Breast Cancer)

Phase: II

Therapy: olaparib

Location: United States

US States: AL, CA, IL, MA, MD, NC, NJ, NY, PA, WA

Contact: Dr. Nadine Tung [617-667-1962; ntung@bidmc.harvard.edu]

NCT04633902
Phase II Study of Olaparib in Combination With Pembrolizumab in Patients With Advanced Melanoma With Homologous Recombination (HR) Pathway Gene Mutation

Cancer type: Melanoma

Variant class: HRR pathway

Other identifier: CPMC19-MEL01

Population segments: BRAF, Second line, Stage III, Stage IV

Phase: II

Therapies: olaparib, pembrolizumab

Location: United States

US State: CA

Contact: Dr. Kevin B. Kim [415-885-8600; ClinicalResearch@sutterhealth.org]

NCT03248570
Phase II Open Label Study of Pembrolizumab in Patients With Metastatic Castration Resistant Prostate Cancer (mCRPC) With or Without DNA Damage Repair Defects

Cancer type: Castration-Resistant Prostate Cancer

Variant class: DNA repair deletion

Other identifiers: 16557, 17-22165, CC 16557, NCI-2017-02408

Population segments: BRCA, Hormone refractory, Line of therapy N/A, Stage IV

Phase: II

Therapy: pembrolizumab

Location: United States

US State: CA

Contact: Patricia Li [415-476-5975; GUTrials@ucsf.edu]

Disclaimer: The data presented here is from a curated knowledgebase of publicly available information, but may not be exhaustive. The data version is 2021.11(003).

CDK12 deletion (continued)

NCT03506997
PERSEUS1: Phase II Trial of the Immune Checkpoint Inhibitor Pembrolizumab for Patients Suffering From Metastatic Prostate Cancer

Cancer type: Castration-Resistant Prostate Cancer

Variant class: DNA repair pathway

Other inclusion criteria: MMR deficient

Other identifiers: CCR4559, EudraCT Number: 2017-000931-15, ICR-CTSU/2016/10060, IRAS ID 208952, PERSEUS1

Population segments: Hormone refractory, Second line, Stage IV

Phase: II

Therapy: pembrolizumab

Location: United Kingdom

NCT04849364
A Phase II Circulating Tumor DNA Enriched, Genomically Directed Post-neoadjuvant Trial for Patients With Residual Triple Negative Breast Cancer (PERSEVERE)

Cancer type: Triple Negative Breast Cancer

Variant class: DNA repair pathway

Other identifiers: HCRN BRE18-334, PERSEVERE

Population segments: Adjuvant, HER2 negative, Stage I, Stage II, Stage III, Triple receptor negative

Phase: II

Therapies: talazoparib, chemotherapy

Location: United States

US State: IN

Contact: Dr. Bryan P. Schneider [317-948-3885; bpschnei@iu.edu]

NCT05002868
A Multi-center, Open-label, Phase I/Ib Study to Assess the Safety, Pharmacokinetics and Anti-tumor Activity of RP12146, a Poly (ADP-ribose) Polymerase (PARP) Inhibitor, in Patients With Locally Advanced or Metastatic Solid Tumors

Cancer type: Biliary Tract Carcinoma, Breast Cancer, Colorectal Cancer, Endometrial Carcinoma, Gastric Cancer, Ovarian Cancer, Pancreatic Cancer, Prostate Cancer, Small Cell Lung Cancer

Variant class: DNA repair pathway

Other identifier: RP12146-2101

Population segments: Extensive, Pulmonary, Second line, Stage III, Stage IV

Exclusion criteria variant class: ERBB2 positive (Breast Cancer)

Phase: I

Therapy: RP12146

Location: Poland

Disclaimer: The data presented here is from a curated knowledgebase of publicly available information, but may not be exhaustive. The data version is 2021.11(003).

CDK12 fusion

NCT04497116
Phase I/IIa Study of the Safety, Pharmacokinetics, Pharmacodynamics and Preliminary Clinical Activity of RP-3500 Alone or in Combination With Talazoparib in Advanced Solid Tumors With ATR Inhibitor Sensitizing Mutations (TRESR Study)

Cancer type: Unspecified Solid Tumor

Variant class: CDK12 aberration

Other identifiers: 20-521, EudraCT Number: 2020-000301-87, IRAS ID: 286609, NCI-2020-06869, RP-3500-01, TRESR, TRESR Study

Population segments: Second line, Stage III, Stage IV

Phase: I/II

Therapies: RP-3500, talazoparib

Locations: Canada, Denmark, United Kingdom, United States

US States: MA, NC, NY, RI, TN, TX

Contact: Dr. Peter Manley [857-322-5553; pmanley@reparerx.com]

NCT04901702
A Randomized Phase I/II Study of Talazoparib or Temozolomide in Combination With Onivyde in Children With Recurrent Solid Malignancies and Ewing Sarcoma

Cancer type: Unspecified Solid Tumor

Variant class: HRR pathway

Other identifiers: EudraCT Number: 2021-003570-31, ONITT

Population segments: Second line, Stage IV

Exclusion criteria variant classes: HRR germline pathway (Central Nervous System Neoplasm, Ewing Sarcoma), HRR pathway (Central Nervous System Neoplasm, Ewing Sarcoma)

Phase: I/II

Therapies: talazoparib, chemotherapy

Locations: Canada, United States

US State: TN

Contact: Dr. Sara Federico [866-278-5833; referralinfo@stjude.org]

NCT03155620
NCI-COG Pediatric MATCH (Molecular Analysis for Therapy Choice) Screening Protocol

Cancer type: Unspecified Solid Tumor

Variant class: DNA repair pathway

Other identifiers: 17-729, 2017-0625, 20170841, APEC1621SC, COGAPEC1621, COGAPEC1621SC, N 55017 SC, NCI-2017-01251, NCI-COG Pediatric MATCH, Pediatric MATCH

Population segments: ALK, Aggressive, BRAF, FGFR, HRAS, Indolent, KRAS, NRAS, Pediatric or Adolescent, Second line, Stage III, Stage IV

Phase: II

Therapy: olaparib

Locations: Puerto Rico, United States

US States: AK, AL, AR, AZ, CA, CO, CT, DC, DE, FL, GA, HI, IA, ID, IL, IN, KY, LA, MA, MD, ME, MI, MN, MO, MS, NC, NE, NH, NJ, NV, NY, OH, OK, OR, PA, RI, SC, SD, TN, TX, UT, VA, VT, WA, WI

Contact: Multiple contacts: See www.clinicaltrials.gov for complete list of contacts.

Disclaimer: The data presented here is from a curated knowledgebase of publicly available information, but may not be exhaustive. The data version is 2021.11(003).

CDK12 fusion (continued)

NCT03233204
NCI-COG Pediatric MATCH (Molecular Analysis for Therapy Choice)- Phase 2 Subprotocol of Olaparib in Patients With Tumors Harboring Defects in DNA Damage Repair Genes

Cancer type: Unspecified Solid Tumor

Variant class: DNA repair pathway

Other identifiers: 20170841, APEC1621H, NCI-2017-00766

Population segments: Aggressive, Indolent, Pediatric or Adolescent, Second line, Stage III, Stage IV

Phase: II

Therapy: olaparib

Locations: Puerto Rico, United States

US States: AK, AL, AR, AZ, CA, CO, DC, DE, FL, GA, IA, ID, IL, IN, KY, LA, MD, ME, MI, MN, MO, MS, NC, ND, NE, NJ, NY, OH, OK, OR, PA, SC, SD, TN, TX, UT, VA, VT, WA, WI

Contact: Multiple contacts: See www.clinicaltrials.gov for complete list of contacts.

NCT04267939
An Open-label Phase Ib Study to Determine the Maximum Tolerated and/or Recommended Phase II Dose of the ATR Inhibitor BAY 1895344 in Combination With PARP Inhibitor Niraparib, in Patients With Recurrent Advanced Solid Tumors and Ovarian Cancer

Cancer type: Ovarian Cancer, Unspecified Solid Tumor

Variant class: DNA repair pathway

Other identifiers: 18595, 19-810, 2019-0471, 20-222, EudraCT Number: 2018-003930-34, NCI-2020-01405

Population segments: Maintenance/Consolidation, Second line, Stage III, Stage IV

Exclusion criteria variant class: DNA repair pathway (Prostate Cancer)

Phase: I

Therapies: BAY-1895344, niraparib

Location: United States

US States: MA, NY, TX

Contact: Bayer Clinical Trials Contact [888-842-2937; clinical-trials-contact@bayer.com]

NCT04693468
Modular Phase IB Hypothesis-Testing, Biomarker-Driven, Talazoparib Combination Trial (TalaCom)

Cancer type: Unspecified Solid Tumor

Variant class: DNA repair pathway

Other identifiers: 2020-0436, NCI-2020-06041, TalaCom

Population segments: Hormone refractory, Second line, Stage III, Stage IV

Phase: I

Therapies: talazoparib, palbociclib, axitinib, crizotinib

Location: United States

US State: TX

Contact: Timothy A. Yap [713-563-1784; tyap@mdanderson.org]

Disclaimer: The data presented here is from a curated knowledgebase of publicly available information, but may not be exhaustive. The data version is 2021.11(003).

CDK12 fusion (continued)

NCT03840967
A Phase II Study Evaluating Safety and Efficacy of Niraparib in Patients With Previously Treated Homologous Recombination (HR) Defective or Loss of Heterozygosity (LOH) High Metastatic Esophageal/Gastroesophageal Junction/ Proximal Gastric Adenocarcinoma

Cancer type: Esophageal Cancer, Gastric Cancer, Gastroesophageal Junction Adenocarcinoma

Variant class: CDK12 fusion

Other identifiers: 20166, 71618, HCRN ESO17-325, NCI-2019-07395

Population segments: Second line, Stage III, Stage IV

Phase: II

Therapy: niraparib

Location: United States

US States: FL, IN, MI, NY

Contact: Dr. Shadia Jalal [317-274-3658; sjalal@iu.edu]

NCT04633902
Phase II Study of Olaparib in Combination With Pembrolizumab in Patients With Advanced Melanoma With Homologous Recombination (HR) Pathway Gene Mutation

Cancer type: Melanoma

Variant class: HRR pathway

Other identifier: CPMC19-MEL01

Population segments: BRAF, Second line, Stage III, Stage IV

Phase: II

Therapies: olaparib, pembrolizumab

Location: United States

US State: CA

Contact: Dr. Kevin B. Kim [415-885-8600; ClinicalResearch@sutterhealth.org]

NCT03506997
PERSEUS1: Phase II Trial of the Immune Checkpoint Inhibitor Pembrolizumab for Patients Suffering From Metastatic Prostate Cancer

Cancer type: Castration-Resistant Prostate Cancer

Variant class: DNA repair pathway

Other inclusion criteria: MMR deficient

Other identifiers: CCR4559, EudraCT Number: 2017-000931-15, ICR-CTSU/2016/10060, IRAS ID 208952, PERSEUS1

Population segments: Hormone refractory, Second line, Stage IV

Phase: II

Therapy: pembrolizumab

Location: United Kingdom

NCT04849364
A Phase II Circulating Tumor DNA Enriched, Genomically Directed Post-neoadjuvant Trial for Patients With Residual Triple Negative Breast Cancer (PERSEVERE)

Cancer type: Triple Negative Breast Cancer

Variant class: DNA repair pathway

Other identifiers: HCRN BRE18-334, PERSEVERE

Population segments: Adjuvant, HER2 negative, Stage I, Stage II, Stage III, Triple receptor negative

Phase: II

Therapies: talazoparib, chemotherapy

Location: United States

US State: IN

Contact: Dr. Bryan P. Schneider [317-948-3885; bpschnei@iu.edu]

Disclaimer: The data presented here is from a curated knowledgebase of publicly available information, but may not be exhaustive. The data version is 2021.11(003).

CDK12 fusion (continued)

NCT05002868
A Multi-center, Open-label, Phase I/Ib Study to Assess the Safety, Pharmacokinetics and Anti-tumor Activity of RP12146, a Poly (ADP-ribose) Polymerase (PARP) Inhibitor, in Patients With Locally Advanced or Metastatic Solid Tumors

Cancer type: Biliary Tract Carcinoma, Breast Cancer, Colorectal Cancer, Endometrial Carcinoma, Gastric Cancer, Ovarian Cancer, Pancreatic Cancer, Prostate Cancer, Small Cell Lung Cancer

Variant class: DNA repair pathway

Other identifier: RP12146-2101

Population segments: Extensive, Pulmonary, Second line, Stage III, Stage IV

Exclusion criteria variant class: ERBB2 positive (Breast Cancer)

Phase: I

Therapy: RP12146

Location: Poland

CDK12 aberration

NCT04497116
Phase I/IIa Study of the Safety, Pharmacokinetics, Pharmacodynamics and Preliminary Clinical Activity of RP-3500 Alone or in Combination With Talazoparib in Advanced Solid Tumors With ATR Inhibitor Sensitizing Mutations (TRESR Study)

Cancer type: Unspecified Solid Tumor

Variant class: CDK12 aberration

Other identifiers: 20-521, EudraCT Number: 2020-000301-87, IRAS ID: 286609, NCI-2020-06869, RP-3500-01, TRESR, TRESR Study

Population segments: Second line, Stage III, Stage IV

Phase: I/II

Therapies: RP-3500, talazoparib

Locations: Canada, Denmark, United Kingdom, United States

US States: MA, NC, NY, RI, TN, TX

Contact: Dr. Peter Manley [857-322-5553; pmanley@reparerx.com]

NCT04901702
A Randomized Phase I/II Study of Talazoparib or Temozolomide in Combination With Onivyde in Children With Recurrent Solid Malignancies and Ewing Sarcoma

Cancer type: Unspecified Solid Tumor

Variant class: HRR pathway

Other identifiers: EudraCT Number: 2021-003570-31, ONITT

Population segments: Second line, Stage IV

Exclusion criteria variant classes: HRR germline pathway (Central Nervous System Neoplasm, Ewing Sarcoma), HRR pathway (Central Nervous System Neoplasm, Ewing Sarcoma)

Phase: I/II

Therapies: talazoparib, chemotherapy

Locations: Canada, United States

US State: TN

Contact: Dr. Sara Federico [866-278-5833; referralinfo@stjude.org]

Disclaimer: The data presented here is from a curated knowledgebase of publicly available information, but may not be exhaustive. The data version is 2021.11(003).

CDK12 aberration (continued)

NCT03155620
NCI-COG Pediatric MATCH (Molecular Analysis for Therapy Choice) Screening Protocol

Cancer type: Unspecified Solid Tumor

Variant class: DNA repair pathway

Other identifiers: 17-729, 2017-0625, 20170841, APEC1621SC, COGAPEC1621, COGAPEC1621SC, N 55017 SC, NCI-2017-01251, NCI-COG Pediatric MATCH, Pediatric MATCH

Population segments: ALK, Aggressive, BRAF, FGFR, HRAS, Indolent, KRAS, NRAS, Pediatric or Adolescent, Second line, Stage III, Stage IV

Phase: II

Therapy: olaparib

Locations: Puerto Rico, United States

US States: AK, AL, AR, AZ, CA, CO, CT, DC, DE, FL, GA, HI, IA, ID, IL, IN, KY, LA, MA, MD, ME, MI, MN, MO, MS, NC, NE, NH, NJ, NV, NY, OH, OK, OR, PA, RI, SC, SD, TN, TX, UT, VA, VT, WA, WI

Contact: Multiple contacts: See www.clinicaltrials.gov for complete list of contacts.

NCT03233204
NCI-COG Pediatric MATCH (Molecular Analysis for Therapy Choice)- Phase 2 Subprotocol of Olaparib in Patients With Tumors Harboring Defects in DNA Damage Repair Genes

Cancer type: Unspecified Solid Tumor

Variant class: DNA repair pathway

Other identifiers: 20170841, APEC1621H, NCI-2017-00766

Population segments: Aggressive, Indolent, Pediatric or Adolescent, Second line, Stage III, Stage IV

Phase: II

Therapy: olaparib

Locations: Puerto Rico, United States

US States: AK, AL, AR, AZ, CA, CO, DC, DE, FL, GA, IA, ID, IL, IN, KY, LA, MD, ME, MI, MN, MO, MS, NC, ND, NE, NJ, NY, OH, OK, OR, PA, SC, SD, TN, TX, UT, VA, VT, WA, WI

Contact: Multiple contacts: See www.clinicaltrials.gov for complete list of contacts.

NCT04267939
An Open-label Phase Ib Study to Determine the Maximum Tolerated and/or Recommended Phase II Dose of the ATR Inhibitor BAY 1895344 in Combination With PARP Inhibitor Niraparib, in Patients With Recurrent Advanced Solid Tumors and Ovarian Cancer

Cancer type: Ovarian Cancer, Unspecified Solid Tumor

Variant class: DNA repair pathway

Other identifiers: 18595, 19-810, 2019-0471, 20-222, EudraCT Number: 2018-003930-34, NCI-2020-01405

Population segments: Maintenance/Consolidation, Second line, Stage III, Stage IV

Exclusion criteria variant class: DNA repair pathway (Prostate Cancer)

Phase: I

Therapies: BAY-1895344, niraparib

Location: United States

US States: MA, NY, TX

Contact: Bayer Clinical Trials Contact [888-842-2937; clinical-trials-contact@bayer.com]

Disclaimer: The data presented here is from a curated knowledgebase of publicly available information, but may not be exhaustive. The data version is 2021.11(003).

CDK12 aberration (continued)

NCT04693468
Modular Phase IB Hypothesis-Testing, Biomarker-Driven, Talazoparib Combination Trial (TalaCom)

Cancer type: Unspecified Solid Tumor

Variant class: DNA repair pathway

Other identifiers: 2020-0436, NCI-2020-06041, TalaCom

Population segments: Hormone refractory, Second line, Stage III, Stage IV

Phase: I

Therapies: talazoparib, palbociclib, axitinib, crizotinib

Location: United States

US State: TX

Contact: Timothy A. Yap [713-563-1784; tyap@mdanderson.org]

NCT04633902
Phase II Study of Olaparib in Combination With Pembrolizumab in Patients With Advanced Melanoma With Homologous Recombination (HR) Pathway Gene Mutation

Cancer type: Melanoma

Variant class: HRR pathway

Other identifier: CPMC19-MEL01

Population segments: BRAF, Second line, Stage III, Stage IV

Phase: II

Therapies: olaparib, pembrolizumab

Location: United States

US State: CA

Contact: Dr. Kevin B. Kim [415-885-8600; ClinicalResearch@sutterhealth.org]

NCT03506997
PERSEUS1: Phase II Trial of the Immune Checkpoint Inhibitor Pembrolizumab for Patients Suffering From Metastatic Prostate Cancer

Cancer type: Castration-Resistant Prostate Cancer

Variant class: DNA repair pathway

Other inclusion criteria: MMR deficient

Other identifiers: CCR4559, EudraCT Number: 2017-000931-15, ICR-CTSU/2016/10060, IRAS ID 208952, PERSEUS1

Population segments: Hormone refractory, Second line, Stage IV

Phase: II

Therapy: pembrolizumab

Location: United Kingdom

NCT04849364
A Phase II Circulating Tumor DNA Enriched, Genomically Directed Post-neoadjuvant Trial for Patients With Residual Triple Negative Breast Cancer (PERSEVERE)

Cancer type: Triple Negative Breast Cancer

Variant class: DNA repair pathway

Other identifiers: HCRN BRE18-334, PERSEVERE

Population segments: Adjuvant, HER2 negative, Stage I, Stage II, Stage III, Triple receptor negative

Phase: II

Therapies: talazoparib, chemotherapy

Location: United States

US State: IN

Contact: Dr. Bryan P. Schneider [317-948-3885; bpschnei@iu.edu]

Disclaimer: The data presented here is from a curated knowledgebase of publicly available information, but may not be exhaustive. The data version is 2021.11(003).

CDK12 aberration (continued)

NCT05002868
A Multi-center, Open-label, Phase I/Ib Study to Assess the Safety, Pharmacokinetics and Anti-tumor Activity of RP12146, a Poly (ADP-ribose) Polymerase (PARP) Inhibitor, in Patients With Locally Advanced or Metastatic Solid Tumors

Cancer type: Biliary Tract Carcinoma, Breast Cancer, Colorectal Cancer, Endometrial Carcinoma, Gastric Cancer, Ovarian Cancer, Pancreatic Cancer, Prostate Cancer, Small Cell Lung Cancer

Variant class: DNA repair pathway

Other identifier: RP12146-2101

Population segments: Extensive, Pulmonary, Second line, Stage III, Stage IV

Exclusion criteria variant class: ERBB2 positive (Breast Cancer)

Phase: I

Therapy: RP12146

Location: Poland

Relevant Biomarkers

Genomic Alteration	Relevant Therapies (In this cancer type)	Relevant Therapies (In other cancer type)	Clinical Trials
CDK12 mutation<br>**Prognostic significance:** None<br>**Diagnostic significance:** None	None	**olaparib** [1]	57
CDK12 splice site mutation<br>**Prognostic significance:** None<br>**Diagnostic significance:** None	None	**olaparib** [1]	57
CDK12 truncating mutation<br>**Prognostic significance:** None<br>**Diagnostic significance:** None	None	**olaparib** [1]	57
CDK12 deletion<br>**Prognostic significance:** None<br>**Diagnostic significance:** None	None	None	19
CDK12 fusion<br>**Prognostic significance:** None<br>**Diagnostic significance:** None	None	None	11
CDK12 aberration<br>**Prognostic significance:** None<br>**Diagnostic significance:** None	None	None	10

Public data sources included in relevant therapies: FDA1, NCCN, **EMA**2, ESMO
Public data sources included in prognostic and diagnostic significance: NCCN, ESMO

Relevant Therapy Summary

● In this cancer type ○ In other cancer type ◐ In this cancer type and other cancer types ✖ No evidence

CDK12 mutation

Relevant Therapy	FDA	NCCN	EMA	ESMO	Clinical Trials*
olaparib	○	○	✖	✖	◐ (II)
olaparib, pembrolizumab	✖	✖	✖	✖	◐ (II)
talazoparib	✖	✖	✖	✖	◐ (II)
talazoparib, chemotherapy	✖	✖	✖	✖	◐ (II)
atezolizumab	✖	✖	✖	✖	● (II)
atezolizumab + talazoparib	✖	✖	✖	✖	● (II)
rucaparib, atezolizumab	✖	✖	✖	✖	● (II)
ATRN-119	✖	✖	✖	✖	● (I/II)
RP-3500, talazoparib	✖	✖	✖	✖	● (I/II)
BAY-1895344, niraparib	✖	✖	✖	✖	● (I)
copanlisib, olaparib, durvalumab	✖	✖	✖	✖	● (I)
HH-CYH33, olaparib	✖	✖	✖	✖	● (I)
HWH-340	✖	✖	✖	✖	● (I)
talazoparib, palbociclib, axitinib, crizotinib	✖	✖	✖	✖	● (I)
talazoparib, hormone therapy	✖	✖	✖	✖	○ (III)
abemaciclib, atezolizumab	✖	✖	✖	✖	○ (II)
durvalumab, olaparib	✖	✖	✖	✖	○ (II)
durvalumab, olaparib, hormone therapy	✖	✖	✖	✖	○ (II)
durvalumab, tremelimumab, olaparib	✖	✖	✖	✖	○ (II)
hormone therapy	✖	✖	✖	✖	○ (II)
niraparib	✖	✖	✖	✖	○ (II)
niraparib, dostarlimab	✖	✖	✖	✖	○ (II)
nivolumab	✖	✖	✖	✖	○ (II)
nivolumab, chemotherapy, hormone therapy	✖	✖	✖	✖	○ (II)
nivolumab, talazoparib	✖	✖	✖	✖	○ (II)
olaparib, bevacizumab	✖	✖	✖	✖	○ (II)
olaparib, chemotherapy	✖	✖	✖	✖	○ (II)
pembrolizumab	✖	✖	✖	✖	○ (II)
rucaparib	✖	✖	✖	✖	○ (II)

* Most advanced phase (IV, III, II/III, II, I/II, I) is shown and multiple clinical trials may be available.

Disclaimer: The data presented here is from a curated knowledgebase of publicly available information, but may not be exhaustive. The data version is 2021.11(003).

Relevant Therapy Summary (continued)

● In this cancer type ○ In other cancer type ◐ In this cancer type and other cancer types ✖ No evidence

CDK12 mutation (continued)

Relevant Therapy	FDA	NCCN	EMA	ESMO	Clinical Trials*
copanlisib, rucaparib	✖	✖	✖	✖	○ (I/II)
sintilimab	✖	✖	✖	✖	○ (I/II)
BAY-1895344, pembrolizumab	✖	✖	✖	✖	○ (I)
ceralasertib, olaparib	✖	✖	✖	✖	○ (I)
pidnarulex	✖	✖	✖	✖	○ (I)
RP12146	✖	✖	✖	✖	○ (I)

CDK12 splice site mutation

Relevant Therapy	FDA	NCCN	EMA	ESMO	Clinical Trials*
olaparib	○	○	✖	✖	◐ (II)
olaparib, pembrolizumab	✖	✖	✖	✖	◐ (II)
talazoparib	✖	✖	✖	✖	◐ (II)
talazoparib, chemotherapy	✖	✖	✖	✖	◐ (II)
atezolizumab	✖	✖	✖	✖	● (II)
atezolizumab + talazoparib	✖	✖	✖	✖	● (II)
rucaparib, atezolizumab	✖	✖	✖	✖	● (II)
ATRN-119	✖	✖	✖	✖	● (I/II)
RP-3500, talazoparib	✖	✖	✖	✖	● (I/II)
BAY-1895344, niraparib	✖	✖	✖	✖	● (I)
copanlisib, olaparib, durvalumab	✖	✖	✖	✖	● (I)
HH-CYH33, olaparib	✖	✖	✖	✖	● (I)
HWH-340	✖	✖	✖	✖	● (I)
talazoparib, palbociclib, axitinib, crizotinib	✖	✖	✖	✖	● (I)
talazoparib, hormone therapy	✖	✖	✖	✖	○ (III)
abemaciclib, atezolizumab	✖	✖	✖	✖	○ (II)
durvalumab, olaparib	✖	✖	✖	✖	○ (II)
durvalumab, olaparib, hormone therapy	✖	✖	✖	✖	○ (II)
durvalumab, tremelimumab, olaparib	✖	✖	✖	✖	○ (II)
hormone therapy	✖	✖	✖	✖	○ (II)

* Most advanced phase (IV, III, II/III, II, I/II, I) is shown and multiple clinical trials may be available.

Disclaimer: The data presented here is from a curated knowledgebase of publicly available information, but may not be exhaustive. The data version is 2021.11(003).

Relevant Therapy Summary (continued)

● In this cancer type ○ In other cancer type ◐ In this cancer type and other cancer types ✖ No evidence

CDK12 splice site mutation (continued)

Relevant Therapy	FDA	NCCN	EMA	ESMO	Clinical Trials*
niraparib	✖	✖	✖	✖	○ (II)
niraparib, dostarlimab	✖	✖	✖	✖	○ (II)
nivolumab	✖	✖	✖	✖	○ (II)
nivolumab, chemotherapy, hormone therapy	✖	✖	✖	✖	○ (II)
nivolumab, talazoparib	✖	✖	✖	✖	○ (II)
olaparib, bevacizumab	✖	✖	✖	✖	○ (II)
olaparib, chemotherapy	✖	✖	✖	✖	○ (II)
pembrolizumab	✖	✖	✖	✖	○ (II)
rucaparib	✖	✖	✖	✖	○ (II)
copanlisib, rucaparib	✖	✖	✖	✖	○ (I/II)
sintilimab	✖	✖	✖	✖	○ (I/II)
BAY-1895344, pembrolizumab	✖	✖	✖	✖	○ (I)
ceralasertib, olaparib	✖	✖	✖	✖	○ (I)
pidnarulex	✖	✖	✖	✖	○ (I)
RP12146	✖	✖	✖	✖	○ (I)

CDK12 truncating mutation

Relevant Therapy	FDA	NCCN	EMA	ESMO	Clinical Trials*
olaparib	○	○	✖	✖	◐ (II)
olaparib, pembrolizumab	✖	✖	✖	✖	◐ (II)
talazoparib	✖	✖	✖	✖	◐ (II)
talazoparib, chemotherapy	✖	✖	✖	✖	◐ (II)
atezolizumab	✖	✖	✖	✖	● (II)
atezolizumab + talazoparib	✖	✖	✖	✖	● (II)
rucaparib, atezolizumab	✖	✖	✖	✖	● (II)
ATRN-119	✖	✖	✖	✖	● (I/II)
RP-3500, talazoparib	✖	✖	✖	✖	● (I/II)
BAY-1895344, niraparib	✖	✖	✖	✖	● (I)
copanlisib, olaparib, durvalumab	✖	✖	✖	✖	● (I)

* Most advanced phase (IV, III, II/III, II, I/II, I) is shown and multiple clinical trials may be available.

Disclaimer: The data presented here is from a curated knowledgebase of publicly available information, but may not be exhaustive. The data version is 2021.11(003).

Relevant Therapy Summary (continued)

● In this cancer type ○ In other cancer type ◐ In this cancer type and other cancer types ✖ No evidence

CDK12 truncating mutation (continued)

Relevant Therapy	FDA	NCCN	EMA	ESMO	Clinical Trials*
HH-CYH33, olaparib	✖	✖	✖	✖	● (I)
HWH-340	✖	✖	✖	✖	● (I)
talazoparib, palbociclib, axitinib, crizotinib	✖	✖	✖	✖	● (I)
talazoparib, hormone therapy	✖	✖	✖	✖	○ (III)
abemaciclib, atezolizumab	✖	✖	✖	✖	○ (II)
durvalumab, olaparib	✖	✖	✖	✖	○ (II)
durvalumab, olaparib, hormone therapy	✖	✖	✖	✖	○ (II)
durvalumab, tremelimumab, olaparib	✖	✖	✖	✖	○ (II)
hormone therapy	✖	✖	✖	✖	○ (II)
niraparib	✖	✖	✖	✖	○ (II)
niraparib, dostarlimab	✖	✖	✖	✖	○ (II)
nivolumab	✖	✖	✖	✖	○ (II)
nivolumab, chemotherapy, hormone therapy	✖	✖	✖	✖	○ (II)
nivolumab, talazoparib	✖	✖	✖	✖	○ (II)
olaparib, bevacizumab	✖	✖	✖	✖	○ (II)
olaparib, chemotherapy	✖	✖	✖	✖	○ (II)
pembrolizumab	✖	✖	✖	✖	○ (II)
rucaparib	✖	✖	✖	✖	○ (II)
copanlisib, rucaparib	✖	✖	✖	✖	○ (I/II)
sintilimab	✖	✖	✖	✖	○ (I/II)
BAY-1895344, pembrolizumab	✖	✖	✖	✖	○ (I)
ceralasertib, olaparib	✖	✖	✖	✖	○ (I)
pidnarulex	✖	✖	✖	✖	○ (I)
RP12146	✖	✖	✖	✖	○ (I)

CDK12 deletion

Relevant Therapy	FDA	NCCN	EMA	ESMO	Clinical Trials*
olaparib	✖	✖	✖	✖	◐ (II)
talazoparib, chemotherapy	✖	✖	✖	✖	◐ (II)

* Most advanced phase (IV, III, II/III, II, I/II, I) is shown and multiple clinical trials may be available.

Disclaimer: The data presented here is from a curated knowledgebase of publicly available information, but may not be exhaustive. The data version is 2021.11(003).

Relevant Therapy Summary (continued)

● In this cancer type ○ In other cancer type ◐ In this cancer type and other cancer types ✖ No evidence

CDK12 deletion (continued)

Relevant Therapy	FDA	NCCN	EMA	ESMO	Clinical Trials*
rucaparib, atezolizumab	✖	✖	✖	✖	● (II)
RP-3500, talazoparib	✖	✖	✖	✖	● (I/II)
BAY-1895344, niraparib	✖	✖	✖	✖	● (I)
talazoparib, palbociclib, axitinib, crizotinib	✖	✖	✖	✖	● (I)
durvalumab, olaparib	✖	✖	✖	✖	○ (II)
niraparib	✖	✖	✖	✖	○ (II)
nivolumab, chemotherapy, hormone therapy	✖	✖	✖	✖	○ (II)
olaparib, pembrolizumab	✖	✖	✖	✖	○ (II)
pembrolizumab	✖	✖	✖	✖	○ (II)
RP12146	✖	✖	✖	✖	○ (I)

CDK12 fusion

Relevant Therapy	FDA	NCCN	EMA	ESMO	Clinical Trials*
talazoparib, chemotherapy	✖	✖	✖	✖	◐ (II)
olaparib	✖	✖	✖	✖	● (II)
RP-3500, talazoparib	✖	✖	✖	✖	● (I/II)
BAY-1895344, niraparib	✖	✖	✖	✖	● (I)
talazoparib, palbociclib, axitinib, crizotinib	✖	✖	✖	✖	● (I)
niraparib	✖	✖	✖	✖	○ (II)
olaparib, pembrolizumab	✖	✖	✖	✖	○ (II)
pembrolizumab	✖	✖	✖	✖	○ (II)
RP12146	✖	✖	✖	✖	○ (I)

CDK12 aberration

Relevant Therapy	FDA	NCCN	EMA	ESMO	Clinical Trials*
talazoparib, chemotherapy	✖	✖	✖	✖	◐ (II)
olaparib	✖	✖	✖	✖	● (II)
RP-3500, talazoparib	✖	✖	✖	✖	● (I/II)
BAY-1895344, niraparib	✖	✖	✖	✖	● (I)

* Most advanced phase (IV, III, II/III, II, I/II, I) is shown and multiple clinical trials may be available.

Disclaimer: The data presented here is from a curated knowledgebase of publicly available information, but may not be exhaustive. The data version is 2021.11(003).

Relevant Therapy Summary (continued)

● In this cancer type ○ In other cancer type ◐ In this cancer type and other cancer types ✖ No evidence

CDK12 aberration (continued)

Relevant Therapy	FDA	NCCN	EMA	ESMO	Clinical Trials*
talazoparib, palbociclib, axitinib, crizotinib	✖	✖	✖	✖	● (I)
olaparib, pembrolizumab	✖	✖	✖	✖	○ (II)
pembrolizumab	✖	✖	✖	✖	○ (II)
RP12146	✖	✖	✖	✖	○ (I)

* Most advanced phase (IV, III, II/III, II, I/II, I) is shown and multiple clinical trials may be available.

Relevant Biomarkers

Genomic Alteration	Relevant Therapies (In this cancer type)	Relevant Therapies (In other cancer type)	Clinical Trials
CDK12 mutation<br>**Prognostic significance:** None<br>**Diagnostic significance:** None	None	**olaparib** [1]	57
CDK12 splice site mutation<br>**Prognostic significance:** None<br>**Diagnostic significance:** None	None	**olaparib** [1]	57
CDK12 truncating mutation<br>**Prognostic significance:** None<br>**Diagnostic significance:** None	None	**olaparib** [1]	57
CDK12 deletion<br>**Prognostic significance:** None<br>**Diagnostic significance:** None	None	None	19
CDK12 fusion<br>**Prognostic significance:** None<br>**Diagnostic significance:** None	None	None	11
CDK12 aberration<br>**Prognostic significance:** None<br>**Diagnostic significance:** None	None	None	10

Public data sources included in relevant therapies: FDA1, NCCN, **EMA**2, ESMO
Public data sources included in prognostic and diagnostic significance: NCCN, ESMO

Clinical Trials Summary

CDK12 mutation + CDK12 splice site mutation + CDK12 truncating mutation + CDK12 deletion

NCT ID	Title	Phase
NCT04276376	A Multicenter, Open Label, Phase II Basket Trial Exploring the Efficacy And Safety of The Combination of Rucaparib (PARP Inhibitor) And Atezolizumab (Anti-PD-L1 Antibody) In Patients With DNA Repair-Deficient or Platinum-Sensitive Solid Tumors	II

Clinical Trials Summary (continued)

CDK12 mutation + CDK12 splice site mutation + CDK12 truncating mutation + CDK12 deletion (continued)

NCT ID	Title	Phase
NCT04126070	A Phase II Multicohort Study of Nivolumab in Combination With Docetaxel and Androgen Deprivation Therapy in Metastatic Hormone Sensitive Prostate Cancer Patients With DNA Damage Repair Defects or Inflamed Tumors	II

CDK12 mutation

NCT ID	Title	Phase
NCT02693535	Targeted Agent and Profiling Utilization Registry (TAPUR) Study.	II
NCT03742895	A Phase II Study of Olaparib Monotherapy in Participants With Previously Treated, Homologous Recombination Repair Mutation (HRRm) or Homologous Recombination Deficiency (HRD) Positive Advanced Cancer	II
NCT03967938	Efficacy of Olaparib in Advanced Cancers Occurring in Patients With Germline Mutations or Somatic Tumor Mutations in Homologous Recombination Genes	II
NCT04550494	A Pharmacodynamics-Driven Trial of Talazoparib, an Oral PARP Inhibitor, in Patients With Advanced Solid Tumors and Aberrations in Genes Involved in DNA Damage Response	II
NCT03842228	A Phase Ib Biomarker-Driven Combination Trial of Copanlisib, Olaparib, and Durvalumab (MEDI4736) in Patients With Advanced Solid Tumors	I
NCT04586335	Open Label, Phase Ib Study to Evaluate the Safety, Tolerability, Pharmacokinetics and Clinical Activity of CYH33, an Oral a-specific PI3K Inhibitor in Combination With Olaparib, an Oral PARP Inhibitor in Patients With Advanced Solid Tumors.	I
NCT02029001	A Two-period, Multicenter, Randomized, Open-label, Phase II Study Evaluating the Clinical Benefit of a Maintenance Treatment Targeting Tumor Molecular Alterations in Patients with Progressive Locally-advanced or Metastatic Solid Tumors.	II
NCT04123366	A Phase II Study of Olaparib in Combination With Pembrolizumab in Participants With Previously Treated, Homologous Recombination Repair Mutation (HRRm) and/or Homologous Recombination Deficiency (HRD)-Positive Advanced Cancer	II
NCT02401347	A Phase II Clinical Trial of the PARP Inhibitor Talazoparib in BRCA1 and BRCA2 Wild Type Patients With Advanced Triple Negative Breast Cancer and Homologous Recombination Deficiency or Advanced HER2 Negative Breast Cancer or Other Solid Tumors With a Mutation in Homologous Recombination Pathway Genes Talazoparib Beyond BRCA (TBB) Trial	II
NCT04497116	Phase I/IIa Study of the Safety, Pharmacokinetics, Pharmacodynamics and Preliminary Clinical Activity of RP-3500 Alone or in Combination With Talazoparib in Advanced Solid Tumors With ATR Inhibitor Sensitizing Mutations (TRESR Study)	I/II
NCT03415659	A Phase I, Open-label, Single-center, Single/Multiple-dose, Dose-escalation/Dose-expansion Clinical Study on Tolerance and Pharmacokinetics of HWH340 Tablet in Patients With Advanced Solid Tumors	I
NCT03767075	Basket of Baskets: A Modular, Open-label, Phase II, Multicentre Study To Evaluate Targeted Agents in Molecularly Selected Populations With Advanced Solid Tumours	II
NCT04905914	A Phase I/IIa, Open-Label, Safety, Pharmacokinetic, And Preliminary Efficacy Study Of Oral ATRN-119 In Patients With Advanced Solid Tumors	I/II
NCT04901702	A Randomized Phase I/II Study of Talazoparib or Temozolomide in Combination With Onivyde in Children With Recurrent Solid Malignancies and Ewing Sarcoma	I/II
NCT03155620	NCI-COG Pediatric MATCH (Molecular Analysis for Therapy Choice) Screening Protocol	II

Clinical Trials Summary (continued)

CDK12 mutation (continued)

NCT ID	Title	Phase
NCT03233204	NCI-COG Pediatric MATCH (Molecular Analysis for Therapy Choice)- Phase 2 Subprotocol of Olaparib in Patients With Tumors Harboring Defects in DNA Damage Repair Genes	II
NCT04267939	An Open-label Phase Ib Study to Determine the Maximum Tolerated and/or Recommended Phase II Dose of the ATR Inhibitor BAY 1895344 in Combination With PARP Inhibitor Niraparib, in Patients With Recurrent Advanced Solid Tumors and Ovarian Cancer	I
NCT04693468	Modular Phase IB Hypothesis-Testing, Biomarker-Driven, Talazoparib Combination Trial (TalaCom)	I
NCT04751929	A Phase II Multi-Center Trial of Abemaciclib With or Without Atezolizumab in Metastatic Castration Resistant Prostate Cancer	II
NCT03810105	A Phase II Study of Olaparib and Durvalumab in Men With Castration Sensitive Biochemically Recurrent Non-Metastatic Prostate Cancer Harboring Mutations in DNA Damage Repair	II
NCT04336943	Durvalumab (MEDI4736) and Olaparib (AZD2281) for Treatment of Biochemically Recurrent Prostate Cancer in Men Predicted to Have a High Neoantigen Load: A Pilot Study	II
NCT04053322	An International Multicenter Phase II Trial of Durvalumab (MEDI4736) Plus OLAparib Plus Fulvestrant in Metastatic or Locally Advanced ER-positive, HER2-negative Breast Cancer Patients Selected Using Criteria That Predict Sensitivity to Olaparib	II
NCT05011383	High-dose Testosterone in Men With Metastatic Castration-resistant Prostate Cancer and ATM or CDK12 Deficiency	II
NCT03553004	Niraparib in Metastatic Pancreatic Cancer After Previous Chemotherapy (NIRA-PANC): A Phase II Trial	II
NCT03840967	A Phase II Study Evaluating Safety and Efficacy of Niraparib in Patients With Previously Treated Homologous Recombination (HR) Defective or Loss of Heterozygosity (LOH) High Metastatic Esophageal/Gastroesophageal Junction/Proximal Gastric Adenocarcinoma	II
NCT04030559	Phase II Trial of PARP Inhibitor Niraparib for Men With High Risk Prostate Cancer and DNA Damage Response Defects	II
NCT04019964	Nivolumab as a Non-Castrating Therapy for MMR-deficient and CDK12- Altered Prostate Cancer With PSA Recurrence After Local Therapy	II
NCT03012321	BRCAAway: A Randomized Phase II Trial of Abiraterone, Olaparib, or Abiraterone + Olaparib in Patients With Metastatic Castration-Resistant Prostate Cancer With DNA Repair Defects	II
NCT03786796	Phase II Study of Olaparib in Metastatic Renal Cell Carcinoma Patients Harboring a BAP-1 or Other DNA Repair Gene Mutations (ORCHID)	II
NCT04515836	Phase II Trial of Olaparib in Homologous Recombination Deficient (HRD) Malignant Mesothelioma	II
NCT04104893	A Phase II Study Of A Checkpoint Inhibitor In Men With Progressive Metastatic Castrate Resistant Prostate Cancer Characterized By A Mismatch Repair Deficiency Or Biallelic CDK12 Inactivation	II
NCT03533946	A Phase II Study of Rucaparib Monotherapy in Nonmetastatic, Hormone-Sensitive Prostate Cancer Demonstrating "BRCAness" Genotype (ROAR)	II
NCT04892693	Talazoparib in Advanced Breast Cancer Patients With Homologous Recombinant Deficiency: A Phase II Clinical and Exploratory Biomarker Study of Talazoparib	II
NCT04740190	Combination Talazoparib - Carboplatin for Recurrent High-grade Glioma With DNA Damage Repair Deficiency (DDRd)	II
NCT04253262	A Phase Ib/II Study of Copanlisib Combined With Rucaparib in Patients With Metastatic Castration-resistant Prostate Cancer	I/II

Disclaimer: The data presented here is from a curated knowledgebase of publicly available information, but may not be exhaustive. The data version is 2021.11(003).

Clinical Trials Summary (continued)

CDK12 mutation (continued)

NCT ID	Title	Phase
NCT04169841	Precision Medicine Phase II Study Evaluating the Efficacy of a Double Immunotherapy by Durvalumab and Tremelimumab Combined With Olaparib in Patients With Solid Cancers and Carriers of Homologous Recombination Repair Genes Mutation in Response or Stable After Olaparib Treatment	II
NCT03925350	A Phase II Study of Niraparib in Patients With Advanced Melanoma With Genetic Homologous Recombination (HR) Mutation / Alteration	II
NCT04940637	A Phase II, Open-Label, Single Arm, Prospective, Multicenter Study of Niraparib Plus Dostarlimab in Patients With Advanced NSCLC and/or MPM, and Positive for PD-L1 Expression and Germline or Somatic Mutations in the HRR Genes	II
NCT04042831	A Phase II Study of Olaparib in Patients With Advanced Biliary Tract Cancer With Aberrant DNA Repair Gene Mutations	II
NCT04939662	Phase II, Single-arm Study of Olaparib and Bevacizumab Combination Therapy in Relapsed Small Cell Lung Cancer Subjects With DNA Damage Response and the Repair Pathway Alteration, ATM Deficiency, SLFN11 Positive, or POU2F3 Positive	II
NCT03506997	PERSEUS1: Phase II Trial of the Immune Checkpoint Inhibitor Pembrolizumab for Patients Suffering From Metastatic Prostate Cancer	II
NCT02264678	A Modular Phase I, Open-Label, Multicentre Study to Assess the Safety, Tolerability, Pharmacokinetics and Preliminary Anti-tumour Activity of Ceralasertib in Combination With Cytotoxic Chemotherapy and/or DNA Damage Repair/Novel Anti-cancer Agents in Patients With Advanced Solid Malignancies	I
NCT04890613	Phase Ib Expansion Study of CX-5461 in Patients With Solid Tumours and BRCA2 and/or PALB2 Mutation	I
NCT05002868	A Multi-center, Open-label, Phase I/Ib Study to Assess the Safety, Pharmacokinetics and Anti-tumor Activity of RP12146, a Poly (ADP-ribose) Polymerase (PARP) Inhibitor, in Patients With Locally Advanced or Metastatic Solid Tumors	I
NCT03395197	A Phase III, Randomized, Double-blind, Placebo-controlled Study Of Talazoparib With Enzalutamide In Metastatic Castration-resistant Prostate Cancer	III
NCT04821622	TALAPRO-3: A Phase III, Randomized, Double-blind, Study of Talazoparib With Enzalutamide Versus Placebo With Enzalutamide in Men With DDR Gene Mutated Metastatic Castration-sensitive Prostate Cancer	III
NCT04187833	Phase II Trial of Nivolumab in Combination with Talazoparib in Patients with Unresectable or Metastatic Melanoma and Mutations in BRCA or BRCA-ness Genes.	II
NCT03344965	A Phase II Study of Olaparib Monotherapy in Metastatic Breast Cancer Patients With Germline or Somatic Mutations in DNA Repair Genes (Olaparib Expanded)	II
NCT03375307	A Phase II Study of Olaparib (AZD2281) in Patients With Metastatic/Advanced Urothelial Carcinoma With DNA-Repair Defects	II
NCT04038502	An Open-label, Multicenter Phase II Study to Compare the Efficacy of Carboplatin as First-line Followed by Second-line Olaparib Versus Olaparib as First-line Followed by Second-line Carboplatin in the Treatment of Patients With Castration Resistant Prostate Cancer Containing Homologous Recombination Deficiency	II
NCT04633902	Phase II Study of Olaparib in Combination With Pembrolizumab in Patients With Advanced Melanoma With Homologous Recombination (HR) Pathway Gene Mutation	II
NCT03248570	Phase II Open Label Study of Pembrolizumab in Patients With Metastatic Castration Resistant Prostate Cancer (mCRPC) With or Without DNA Damage Repair Defects	II

Clinical Trials Summary (continued)

CDK12 mutation (continued)

NCT ID	Title	Phase
No NCT ID	Single-arm Exploratory Study for Sintilimab Second-line or Later-line Treatment of Advanced Non-small Cell Lung Cancer with DDR Pathway Gene Mutation	I/II
NCT04095273	A Multicenter, Non-randomized, Open-label Phase Ib Study to Determine the Maximum Tolerated and Recommended Phase II Dose of the ATR Inhibitor BAY1895344 in Combination With Pembrolizumab and to Characterize Its Safety, Tolerability, Pharmacokinetics and Preliminary Anti-tumor Activity in Patients With Advanced Solid Tumors	I
NCT04849364	A Phase II Circulating Tumor DNA Enriched, Genomically Directed Post-neoadjuvant Trial for Patients With Residual Triple Negative Breast Cancer (PERSEVERE)	II

CDK12 splice site mutation

NCT ID	Title	Phase
NCT02693535	Targeted Agent and Profiling Utilization Registry (TAPUR) Study.	II
NCT03742895	A Phase II Study of Olaparib Monotherapy in Participants With Previously Treated, Homologous Recombination Repair Mutation (HRRm) or Homologous Recombination Deficiency (HRD) Positive Advanced Cancer	II
NCT03967938	Efficacy of Olaparib in Advanced Cancers Occurring in Patients With Germline Mutations or Somatic Tumor Mutations in Homologous Recombination Genes	II
NCT04550494	A Pharmacodynamics-Driven Trial of Talazoparib, an Oral PARP Inhibitor, in Patients With Advanced Solid Tumors and Aberrations in Genes Involved in DNA Damage Response	II
NCT03842228	A Phase Ib Biomarker-Driven Combination Trial of Copanlisib, Olaparib, and Durvalumab (MEDI4736) in Patients With Advanced Solid Tumors	I
NCT04586335	Open Label, Phase Ib Study to Evaluate the Safety, Tolerability, Pharmacokinetics and Clinical Activity of CYH33, an Oral a-specific PI3K Inhibitor in Combination With Olaparib, an Oral PARP Inhibitor in Patients With Advanced Solid Tumors.	I
NCT02029001	A Two-period, Multicenter, Randomized, Open-label, Phase II Study Evaluating the Clinical Benefit of a Maintenance Treatment Targeting Tumor Molecular Alterations in Patients with Progressive Locally-advanced or Metastatic Solid Tumors.	II
NCT04123366	A Phase II Study of Olaparib in Combination With Pembrolizumab in Participants With Previously Treated, Homologous Recombination Repair Mutation (HRRm) and/or Homologous Recombination Deficiency (HRD)-Positive Advanced Cancer	II
NCT02401347	A Phase II Clinical Trial of the PARP Inhibitor Talazoparib in BRCA1 and BRCA2 Wild Type Patients With Advanced Triple Negative Breast Cancer and Homologous Recombination Deficiency or Advanced HER2 Negative Breast Cancer or Other Solid Tumors With a Mutation in Homologous Recombination Pathway Genes Talazoparib Beyond BRCA (TBB) Trial	II
NCT04497116	Phase I/IIa Study of the Safety, Pharmacokinetics, Pharmacodynamics and Preliminary Clinical Activity of RP-3500 Alone or in Combination With Talazoparib in Advanced Solid Tumors With ATR Inhibitor Sensitizing Mutations (TRESR Study)	I/II
NCT03415659	A Phase I, Open-label, Single-center, Single/Multiple-dose, Dose-escalation/Dose-expansion Clinical Study on Tolerance and Pharmacokinetics of HWH340 Tablet in Patients With Advanced Solid Tumors	I
NCT03767075	Basket of Baskets: A Modular, Open-label, Phase II, Multicentre Study To Evaluate Targeted Agents in Molecularly Selected Populations With Advanced Solid Tumours	II

Disclaimer: The data presented here is from a curated knowledgebase of publicly available information, but may not be exhaustive. The data version is 2021.11(003).

Clinical Trials Summary (continued)

CDK12 splice site mutation (continued)

NCT ID	Title	Phase
NCT04905914	A Phase I/IIa, Open-Label, Safety, Pharmacokinetic, And Preliminary Efficacy Study Of Oral ATRN-119 In Patients With Advanced Solid Tumors	I/II
NCT04901702	A Randomized Phase I/II Study of Talazoparib or Temozolomide in Combination With Onivyde in Children With Recurrent Solid Malignancies and Ewing Sarcoma	I/II
NCT03155620	NCI-COG Pediatric MATCH (Molecular Analysis for Therapy Choice) Screening Protocol	II
NCT03233204	NCI-COG Pediatric MATCH (Molecular Analysis for Therapy Choice)- Phase 2 Subprotocol of Olaparib in Patients With Tumors Harboring Defects in DNA Damage Repair Genes	II
NCT04267939	An Open-label Phase Ib Study to Determine the Maximum Tolerated and/or Recommended Phase II Dose of the ATR Inhibitor BAY 1895344 in Combination With PARP Inhibitor Niraparib, in Patients With Recurrent Advanced Solid Tumors and Ovarian Cancer	I
NCT04693468	Modular Phase IB Hypothesis-Testing, Biomarker-Driven, Talazoparib Combination Trial (TalaCom)	I
NCT03810105	A Phase II Study of Olaparib and Durvalumab in Men With Castration Sensitive Biochemically Recurrent Non-Metastatic Prostate Cancer Harboring Mutations in DNA Damage Repair	II
NCT04751929	A Phase II Multi-Center Trial of Abemaciclib With or Without Atezolizumab in Metastatic Castration Resistant Prostate Cancer	II
NCT04336943	Durvalumab (MEDI4736) and Olaparib (AZD2281) for Treatment of Biochemically Recurrent Prostate Cancer in Men Predicted to Have a High Neoantigen Load: A Pilot Study	II
NCT04053322	An International Multicenter Phase II Trial of Durvalumab (MEDI4736) Plus OLAparib Plus Fulvestrant in Metastatic or Locally Advanced ER-positive, HER2-negative Breast Cancer Patients Selected Using Criteria That Predict Sensitivity to Olaparib	II
NCT05011383	High-dose Testosterone in Men With Metastatic Castration-resistant Prostate Cancer and ATM or CDK12 Deficiency	II
NCT03553004	Niraparib in Metastatic Pancreatic Cancer After Previous Chemotherapy (NIRA-PANC): A Phase II Trial	II
NCT03840967	A Phase II Study Evaluating Safety and Efficacy of Niraparib in Patients With Previously Treated Homologous Recombination (HR) Defective or Loss of Heterozygosity (LOH) High Metastatic Esophageal/Gastroesophageal Junction/Proximal Gastric Adenocarcinoma	II
NCT04030559	Phase II Trial of PARP Inhibitor Niraparib for Men With High Risk Prostate Cancer and DNA Damage Response Defects	II
NCT04019964	Nivolumab as a Non-Castrating Therapy for MMR-deficient and CDK12- Altered Prostate Cancer With PSA Recurrence After Local Therapy	II
NCT03012321	BRCAAway: A Randomized Phase II Trial of Abiraterone, Olaparib, or Abiraterone + Olaparib in Patients With Metastatic Castration-Resistant Prostate Cancer With DNA Repair Defects	II
NCT03786796	Phase II Study of Olaparib in Metastatic Renal Cell Carcinoma Patients Harboring a BAP-1 or Other DNA Repair Gene Mutations (ORCHID)	II
NCT04515836	Phase II Trial of Olaparib in Homologous Recombination Deficient (HRD) Malignant Mesothelioma	II
NCT04104893	A Phase II Study Of A Checkpoint Inhibitor In Men With Progressive Metastatic Castrate Resistant Prostate Cancer Characterized By A Mismatch Repair Deficiency Or Biallelic CDK12 Inactivation	II
NCT03533946	A Phase II Study of Rucaparib Monotherapy in Nonmetastatic, Hormone-Sensitive Prostate Cancer Demonstrating "BRCAness" Genotype (ROAR)	II
NCT04892693	Talazoparib in Advanced Breast Cancer Patients With Homologous Recombinant Deficiency: A Phase II Clinical and Exploratory Biomarker Study of Talazoparib	II

Clinical Trials Summary (continued)

CDK12 splice site mutation (continued)

NCT ID	Title	Phase
NCT04740190	Combination Talazoparib - Carboplatin for Recurrent High-grade Glioma With DNA Damage Repair Deficiency (DDRd)	II
NCT04253262	A Phase Ib/II Study of Copanlisib Combined With Rucaparib in Patients With Metastatic Castration-resistant Prostate Cancer	I/II
NCT04169841	Precision Medicine Phase II Study Evaluating the Efficacy of a Double Immunotherapy by Durvalumab and Tremelimumab Combined With Olaparib in Patients With Solid Cancers and Carriers of Homologous Recombination Repair Genes Mutation in Response or Stable After Olaparib Treatment	II
NCT03925350	A Phase II Study of Niraparib in Patients With Advanced Melanoma With Genetic Homologous Recombination (HR) Mutation / Alteration	II
NCT04940637	A Phase II, Open-Label, Single Arm, Prospective, Multicenter Study of Niraparib Plus Dostarlimab in Patients With Advanced NSCLC and/or MPM, and Positive for PD-L1 Expression and Germline or Somatic Mutations in the HRR Genes	II
NCT04042831	A Phase II Study of Olaparib in Patients With Advanced Biliary Tract Cancer With Aberrant DNA Repair Gene Mutations	II
NCT04939662	Phase II, Single-arm Study of Olaparib and Bevacizumab Combination Therapy in Relapsed Small Cell Lung Cancer Subjects With DNA Damage Response and the Repair Pathway Alteration, ATM Deficiency, SLFN11 Positive, or POU2F3 Positive	II
NCT03506997	PERSEUS1: Phase II Trial of the Immune Checkpoint Inhibitor Pembrolizumab for Patients Suffering From Metastatic Prostate Cancer	II
NCT02264678	A Modular Phase I, Open-Label, Multicentre Study to Assess the Safety, Tolerability, Pharmacokinetics and Preliminary Anti-tumour Activity of Ceralasertib in Combination With Cytotoxic Chemotherapy and/or DNA Damage Repair/Novel Anti-cancer Agents in Patients With Advanced Solid Malignancies	I
NCT04890613	Phase Ib Expansion Study of CX-5461 in Patients With Solid Tumours and BRCA2 and/or PALB2 Mutation	I
NCT05002868	A Multi-center, Open-label, Phase I/Ib Study to Assess the Safety, Pharmacokinetics and Anti-tumor Activity of RP12146, a Poly (ADP-ribose) Polymerase (PARP) Inhibitor, in Patients With Locally Advanced or Metastatic Solid Tumors	I
NCT03395197	A Phase III, Randomized, Double-blind, Placebo-controlled Study Of Talazoparib With Enzalutamide In Metastatic Castration-resistant Prostate Cancer	III
NCT04821622	TALAPRO-3: A Phase III, Randomized, Double-blind, Study of Talazoparib With Enzalutamide Versus Placebo With Enzalutamide in Men With DDR Gene Mutated Metastatic Castration-sensitive Prostate Cancer	III
NCT04187833	Phase II Trial of Nivolumab in Combination with Talazoparib in Patients with Unresectable or Metastatic Melanoma and Mutations in BRCA or BRCA-ness Genes.	II
NCT03344965	A Phase II Study of Olaparib Monotherapy in Metastatic Breast Cancer Patients With Germline or Somatic Mutations in DNA Repair Genes (Olaparib Expanded)	II
NCT03375307	A Phase II Study of Olaparib (AZD2281) in Patients With Metastatic/Advanced Urothelial Carcinoma With DNA-Repair Defects	II
NCT04038502	An Open-label, Multicenter Phase II Study to Compare the Efficacy of Carboplatin as First-line Followed by Second-line Olaparib Versus Olaparib as First-line Followed by Second-line Carboplatin in the Treatment of Patients With Castration Resistant Prostate Cancer Containing Homologous Recombination Deficiency	II

Disclaimer: The data presented here is from a curated knowledgebase of publicly available information, but may not be exhaustive. The data version is 2021.11(003).

Clinical Trials Summary (continued)

CDK12 splice site mutation (continued)

NCT ID	Title	Phase
NCT04633902	Phase II Study of Olaparib in Combination With Pembrolizumab in Patients With Advanced Melanoma With Homologous Recombination (HR) Pathway Gene Mutation	II
NCT03248570	Phase II Open Label Study of Pembrolizumab in Patients With Metastatic Castration Resistant Prostate Cancer (mCRPC) With or Without DNA Damage Repair Defects	II
No NCT ID	Single-arm Exploratory Study for Sintilimab Second-line or Later-line Treatment of Advanced Non-small Cell Lung Cancer with DDR Pathway Gene Mutation	I/II
NCT04095273	A Multicenter, Non-randomized, Open-label Phase Ib Study to Determine the Maximum Tolerated and Recommended Phase II Dose of the ATR Inhibitor BAY1895344 in Combination With Pembrolizumab and to Characterize Its Safety, Tolerability, Pharmacokinetics and Preliminary Anti-tumor Activity in Patients With Advanced Solid Tumors	I
NCT04849364	A Phase II Circulating Tumor DNA Enriched, Genomically Directed Post-neoadjuvant Trial for Patients With Residual Triple Negative Breast Cancer (PERSEVERE)	II

CDK12 truncating mutation

NCT ID	Title	Phase
NCT04550494	A Pharmacodynamics-Driven Trial of Talazoparib, an Oral PARP Inhibitor, in Patients With Advanced Solid Tumors and Aberrations in Genes Involved in DNA Damage Response	II
NCT02693535	Targeted Agent and Profiling Utilization Registry (TAPUR) Study.	II
NCT03742895	A Phase II Study of Olaparib Monotherapy in Participants With Previously Treated, Homologous Recombination Repair Mutation (HRRm) or Homologous Recombination Deficiency (HRD) Positive Advanced Cancer	II
NCT03967938	Efficacy of Olaparib in Advanced Cancers Occurring in Patients With Germline Mutations or Somatic Tumor Mutations in Homologous Recombination Genes	II
NCT03842228	A Phase Ib Biomarker-Driven Combination Trial of Copanlisib, Olaparib, and Durvalumab (MEDI4736) in Patients With Advanced Solid Tumors	I
NCT04586335	Open Label, Phase Ib Study to Evaluate the Safety, Tolerability, Pharmacokinetics and Clinical Activity of CYH33, an Oral a-specific PI3K Inhibitor in Combination With Olaparib, an Oral PARP Inhibitor in Patients With Advanced Solid Tumors.	I
NCT02029001	A Two-period, Multicenter, Randomized, Open-label, Phase II Study Evaluating the Clinical Benefit of a Maintenance Treatment Targeting Tumor Molecular Alterations in Patients with Progressive Locally-advanced or Metastatic Solid Tumors.	II
NCT04123366	A Phase II Study of Olaparib in Combination With Pembrolizumab in Participants With Previously Treated, Homologous Recombination Repair Mutation (HRRm) and/or Homologous Recombination Deficiency (HRD)-Positive Advanced Cancer	II
NCT02401347	A Phase II Clinical Trial of the PARP Inhibitor Talazoparib in BRCA1 and BRCA2 Wild Type Patients With Advanced Triple Negative Breast Cancer and Homologous Recombination Deficiency or Advanced HER2 Negative Breast Cancer or Other Solid Tumors With a Mutation in Homologous Recombination Pathway Genes Talazoparib Beyond BRCA (TBB) Trial	II
NCT04497116	Phase I/IIa Study of the Safety, Pharmacokinetics, Pharmacodynamics and Preliminary Clinical Activity of RP-3500 Alone or in Combination With Talazoparib in Advanced Solid Tumors With ATR Inhibitor Sensitizing Mutations (TRESR Study)	I/II

Clinical Trials Summary (continued)

CDK12 truncating mutation (continued)

NCT ID	Title	Phase
NCT03415659	A Phase I, Open-label, Single-center, Single/Multiple-dose, Dose-escalation/Dose-expansion Clinical Study on Tolerance and Pharmacokinetics of HWH340 Tablet in Patients With Advanced Solid Tumors	I
NCT03767075	Basket of Baskets: A Modular, Open-label, Phase II, Multicentre Study To Evaluate Targeted Agents in Molecularly Selected Populations With Advanced Solid Tumours	II
NCT04905914	A Phase I/IIa, Open-Label, Safety, Pharmacokinetic, And Preliminary Efficacy Study Of Oral ATRN-119 In Patients With Advanced Solid Tumors	I/II
NCT04901702	A Randomized Phase I/II Study of Talazoparib or Temozolomide in Combination With Onivyde in Children With Recurrent Solid Malignancies and Ewing Sarcoma	I/II
NCT03155620	NCI-COG Pediatric MATCH (Molecular Analysis for Therapy Choice) Screening Protocol	II
NCT03233204	NCI-COG Pediatric MATCH (Molecular Analysis for Therapy Choice)- Phase 2 Subprotocol of Olaparib in Patients With Tumors Harboring Defects in DNA Damage Repair Genes	II
NCT04267939	An Open-label Phase Ib Study to Determine the Maximum Tolerated and/or Recommended Phase II Dose of the ATR Inhibitor BAY 1895344 in Combination With PARP Inhibitor Niraparib, in Patients With Recurrent Advanced Solid Tumors and Ovarian Cancer	I
NCT04693468	Modular Phase IB Hypothesis-Testing, Biomarker-Driven, Talazoparib Combination Trial (TalaCom)	I
NCT03810105	A Phase II Study of Olaparib and Durvalumab in Men With Castration Sensitive Biochemically Recurrent Non-Metastatic Prostate Cancer Harboring Mutations in DNA Damage Repair	II
NCT03840967	A Phase II Study Evaluating Safety and Efficacy of Niraparib in Patients With Previously Treated Homologous Recombination (HR) Defective or Loss of Heterozygosity (LOH) High Metastatic Esophageal/Gastroesophageal Junction/Proximal Gastric Adenocarcinoma	II
NCT04751929	A Phase II Multi-Center Trial of Abemaciclib With or Without Atezolizumab in Metastatic Castration Resistant Prostate Cancer	II
NCT04336943	Durvalumab (MEDI4736) and Olaparib (AZD2281) for Treatment of Biochemically Recurrent Prostate Cancer in Men Predicted to Have a High Neoantigen Load: A Pilot Study	II
NCT04053322	An International Multicenter Phase II Trial of Durvalumab (MEDI4736) Plus OLAparib Plus Fulvestrant in Metastatic or Locally Advanced ER-positive, HER2-negative Breast Cancer Patients Selected Using Criteria That Predict Sensitivity to Olaparib	II
NCT05011383	High-dose Testosterone in Men With Metastatic Castration-resistant Prostate Cancer and ATM or CDK12 Deficiency	II
NCT03553004	Niraparib in Metastatic Pancreatic Cancer After Previous Chemotherapy (NIRA-PANC): A Phase II Trial	II
NCT04030559	Phase II Trial of PARP Inhibitor Niraparib for Men With High Risk Prostate Cancer and DNA Damage Response Defects	II
NCT04019964	Nivolumab as a Non-Castrating Therapy for MMR-deficient and CDK12- Altered Prostate Cancer With PSA Recurrence After Local Therapy	II
NCT03012321	BRCAAway: A Randomized Phase II Trial of Abiraterone, Olaparib, or Abiraterone + Olaparib in Patients With Metastatic Castration-Resistant Prostate Cancer With DNA Repair Defects	II
NCT03786796	Phase II Study of Olaparib in Metastatic Renal Cell Carcinoma Patients Harboring a BAP-1 or Other DNA Repair Gene Mutations (ORCHID)	II
NCT04515836	Phase II Trial of Olaparib in Homologous Recombination Deficient (HRD) Malignant Mesothelioma	II
NCT04104893	A Phase II Study Of A Checkpoint Inhibitor In Men With Progressive Metastatic Castrate Resistant Prostate Cancer Characterized By A Mismatch Repair Deficiency Or Biallelic CDK12 Inactivation	II

Disclaimer: The data presented here is from a curated knowledgebase of publicly available information, but may not be exhaustive. The data version is 2021.11(003).

Clinical Trials Summary (continued)

CDK12 truncating mutation (continued)

NCT ID	Title	Phase
NCT03533946	A Phase II Study of Rucaparib Monotherapy in Nonmetastatic, Hormone-Sensitive Prostate Cancer Demonstrating "BRCAness" Genotype (ROAR)	II
NCT04892693	Talazoparib in Advanced Breast Cancer Patients With Homologous Recombinant Deficiency: A Phase II Clinical and Exploratory Biomarker Study of Talazoparib	II
NCT04740190	Combination Talazoparib - Carboplatin for Recurrent High-grade Glioma With DNA Damage Repair Deficiency (DDRd)	II
NCT04253262	A Phase Ib/II Study of Copanlisib Combined With Rucaparib in Patients With Metastatic Castration-resistant Prostate Cancer	I/II
NCT04169841	Precision Medicine Phase II Study Evaluating the Efficacy of a Double Immunotherapy by Durvalumab and Tremelimumab Combined With Olaparib in Patients With Solid Cancers and Carriers of Homologous Recombination Repair Genes Mutation in Response or Stable After Olaparib Treatment	II
NCT03925350	A Phase II Study of Niraparib in Patients With Advanced Melanoma With Genetic Homologous Recombination (HR) Mutation / Alteration	II
NCT04940637	A Phase II, Open-Label, Single Arm, Prospective, Multicenter Study of Niraparib Plus Dostarlimab in Patients With Advanced NSCLC and/or MPM, and Positive for PD-L1 Expression and Germline or Somatic Mutations in the HRR Genes	II
NCT04042831	A Phase II Study of Olaparib in Patients With Advanced Biliary Tract Cancer With Aberrant DNA Repair Gene Mutations	II
NCT04939662	Phase II, Single-arm Study of Olaparib and Bevacizumab Combination Therapy in Relapsed Small Cell Lung Cancer Subjects With DNA Damage Response and the Repair Pathway Alteration, ATM Deficiency, SLFN11 Positive, or POU2F3 Positive	II
NCT03506997	PERSEUS1: Phase II Trial of the Immune Checkpoint Inhibitor Pembrolizumab for Patients Suffering From Metastatic Prostate Cancer	II
NCT02264678	A Modular Phase I, Open-Label, Multicentre Study to Assess the Safety, Tolerability, Pharmacokinetics and Preliminary Anti-tumour Activity of Ceralasertib in Combination With Cytotoxic Chemotherapy and/or DNA Damage Repair/Novel Anti-cancer Agents in Patients With Advanced Solid Malignancies	I
NCT04890613	Phase Ib Expansion Study of CX-5461 in Patients With Solid Tumours and BRCA2 and/or PALB2 Mutation	I
NCT05002868	A Multi-center, Open-label, Phase I/Ib Study to Assess the Safety, Pharmacokinetics and Anti-tumor Activity of RP12146, a Poly (ADP-ribose) Polymerase (PARP) Inhibitor, in Patients With Locally Advanced or Metastatic Solid Tumors	I
NCT03395197	A Phase III, Randomized, Double-blind, Placebo-controlled Study Of Talazoparib With Enzalutamide In Metastatic Castration-resistant Prostate Cancer	III
NCT04821622	TALAPRO-3: A Phase III, Randomized, Double-blind, Study of Talazoparib With Enzalutamide Versus Placebo With Enzalutamide in Men With DDR Gene Mutated Metastatic Castration-sensitive Prostate Cancer	III
NCT04187833	Phase II Trial of Nivolumab in Combination with Talazoparib in Patients with Unresectable or Metastatic Melanoma and Mutations in BRCA or BRCA-ness Genes.	II
NCT03344965	A Phase II Study of Olaparib Monotherapy in Metastatic Breast Cancer Patients With Germline or Somatic Mutations in DNA Repair Genes (Olaparib Expanded)	II
NCT03375307	A Phase II Study of Olaparib (AZD2281) in Patients With Metastatic/Advanced Urothelial Carcinoma With DNA-Repair Defects	II

Disclaimer: The data presented here is from a curated knowledgebase of publicly available information, but may not be exhaustive. The data version is 2021.11(003).

Clinical Trials Summary (continued)

CDK12 truncating mutation (continued)

NCT ID	Title	Phase
NCT04038502	An Open-label, Multicenter Phase II Study to Compare the Efficacy of Carboplatin as First-line Followed by Second-line Olaparib Versus Olaparib as First-line Followed by Second-line Carboplatin in the Treatment of Patients With Castration Resistant Prostate Cancer Containing Homologous Recombination Deficiency	II
NCT04633902	Phase II Study of Olaparib in Combination With Pembrolizumab in Patients With Advanced Melanoma With Homologous Recombination (HR) Pathway Gene Mutation	II
NCT03248570	Phase II Open Label Study of Pembrolizumab in Patients With Metastatic Castration Resistant Prostate Cancer (mCRPC) With or Without DNA Damage Repair Defects	II
No NCT ID	Single-arm Exploratory Study for Sintilimab Second-line or Later-line Treatment of Advanced Non-small Cell Lung Cancer with DDR Pathway Gene Mutation	I/II
NCT04095273	A Multicenter, Non-randomized, Open-label Phase Ib Study to Determine the Maximum Tolerated and Recommended Phase II Dose of the ATR Inhibitor BAY1895344 in Combination With Pembrolizumab and to Characterize Its Safety, Tolerability, Pharmacokinetics and Preliminary Anti-tumor Activity in Patients With Advanced Solid Tumors	I
NCT04849364	A Phase II Circulating Tumor DNA Enriched, Genomically Directed Post-neoadjuvant Trial for Patients With Residual Triple Negative Breast Cancer (PERSEVERE)	II

CDK12 deletion

NCT ID	Title	Phase
NCT04497116	Phase I/IIa Study of the Safety, Pharmacokinetics, Pharmacodynamics and Preliminary Clinical Activity of RP-3500 Alone or in Combination With Talazoparib in Advanced Solid Tumors With ATR Inhibitor Sensitizing Mutations (TRESR Study)	I/II
NCT04901702	A Randomized Phase I/II Study of Talazoparib or Temozolomide in Combination With Onivyde in Children With Recurrent Solid Malignancies and Ewing Sarcoma	I/II
NCT03155620	NCI-COG Pediatric MATCH (Molecular Analysis for Therapy Choice) Screening Protocol	II
NCT03233204	NCI-COG Pediatric MATCH (Molecular Analysis for Therapy Choice)- Phase 2 Subprotocol of Olaparib in Patients With Tumors Harboring Defects in DNA Damage Repair Genes	II
NCT04267939	An Open-label Phase Ib Study to Determine the Maximum Tolerated and/or Recommended Phase II Dose of the ATR Inhibitor BAY 1895344 in Combination With PARP Inhibitor Niraparib, in Patients With Recurrent Advanced Solid Tumors and Ovarian Cancer	I
NCT04693468	Modular Phase IB Hypothesis-Testing, Biomarker-Driven, Talazoparib Combination Trial (TalaCom)	I
NCT03810105	A Phase II Study of Olaparib and Durvalumab in Men With Castration Sensitive Biochemically Recurrent Non-Metastatic Prostate Cancer Harboring Mutations in DNA Damage Repair	II
NCT03840967	A Phase II Study Evaluating Safety and Efficacy of Niraparib in Patients With Previously Treated Homologous Recombination (HR) Defective or Loss of Heterozygosity (LOH) High Metastatic Esophageal/Gastroesophageal Junction/Proximal Gastric Adenocarcinoma	II
NCT04030559	Phase II Trial of PARP Inhibitor Niraparib for Men With High Risk Prostate Cancer and DNA Damage Response Defects	II
NCT04104893	A Phase II Study Of A Checkpoint Inhibitor In Men With Progressive Metastatic Castrate Resistant Prostate Cancer Characterized By A Mismatch Repair Deficiency Or Biallelic CDK12 Inactivation	II

Clinical Trials Summary (continued)

CDK12 deletion (continued)

NCT ID	Title	Phase
NCT04740190	Combination Talazoparib - Carboplatin for Recurrent High-grade Glioma With DNA Damage Repair Deficiency (DDRd)	II
NCT03344965	A Phase II Study of Olaparib Monotherapy in Metastatic Breast Cancer Patients With Germline or Somatic Mutations in DNA Repair Genes (Olaparib Expanded)	II
NCT04633902	Phase II Study of Olaparib in Combination With Pembrolizumab in Patients With Advanced Melanoma With Homologous Recombination (HR) Pathway Gene Mutation	II
NCT03248570	Phase II Open Label Study of Pembrolizumab in Patients With Metastatic Castration Resistant Prostate Cancer (mCRPC) With or Without DNA Damage Repair Defects	II
NCT03506997	PERSEUS1: Phase II Trial of the Immune Checkpoint Inhibitor Pembrolizumab for Patients Suffering From Metastatic Prostate Cancer	II
NCT04849364	A Phase II Circulating Tumor DNA Enriched, Genomically Directed Post-neoadjuvant Trial for Patients With Residual Triple Negative Breast Cancer (PERSEVERE)	II
NCT05002868	A Multi-center, Open-label, Phase I/Ib Study to Assess the Safety, Pharmacokinetics and Anti-tumor Activity of RP12146, a Poly (ADP-ribose) Polymerase (PARP) Inhibitor, in Patients With Locally Advanced or Metastatic Solid Tumors	I

CDK12 fusion

NCT ID	Title	Phase
NCT04497116	Phase I/IIa Study of the Safety, Pharmacokinetics, Pharmacodynamics and Preliminary Clinical Activity of RP-3500 Alone or in Combination With Talazoparib in Advanced Solid Tumors With ATR Inhibitor Sensitizing Mutations (TRESR Study)	I/II
NCT04901702	A Randomized Phase I/II Study of Talazoparib or Temozolomide in Combination With Onivyde in Children With Recurrent Solid Malignancies and Ewing Sarcoma	I/II
NCT03155620	NCI-COG Pediatric MATCH (Molecular Analysis for Therapy Choice) Screening Protocol	II
NCT03233204	NCI-COG Pediatric MATCH (Molecular Analysis for Therapy Choice)- Phase 2 Subprotocol of Olaparib in Patients With Tumors Harboring Defects in DNA Damage Repair Genes	II
NCT04267939	An Open-label Phase Ib Study to Determine the Maximum Tolerated and/or Recommended Phase II Dose of the ATR Inhibitor BAY 1895344 in Combination With PARP Inhibitor Niraparib, in Patients With Recurrent Advanced Solid Tumors and Ovarian Cancer	I
NCT04693468	Modular Phase IB Hypothesis-Testing, Biomarker-Driven, Talazoparib Combination Trial (TalaCom)	I
NCT03840967	A Phase II Study Evaluating Safety and Efficacy of Niraparib in Patients With Previously Treated Homologous Recombination (HR) Defective or Loss of Heterozygosity (LOH) High Metastatic Esophageal/Gastroesophageal Junction/Proximal Gastric Adenocarcinoma	II
NCT04633902	Phase II Study of Olaparib in Combination With Pembrolizumab in Patients With Advanced Melanoma With Homologous Recombination (HR) Pathway Gene Mutation	II
NCT03506997	PERSEUS1: Phase II Trial of the Immune Checkpoint Inhibitor Pembrolizumab for Patients Suffering From Metastatic Prostate Cancer	II
NCT04849364	A Phase II Circulating Tumor DNA Enriched, Genomically Directed Post-neoadjuvant Trial for Patients With Residual Triple Negative Breast Cancer (PERSEVERE)	II

Clinical Trials Summary (continued)

CDK12 fusion (continued)

NCT ID	Title	Phase
NCT05002868	A Multi-center, Open-label, Phase I/Ib Study to Assess the Safety, Pharmacokinetics and Anti-tumor Activity of RP12146, a Poly (ADP-ribose) Polymerase (PARP) Inhibitor, in Patients With Locally Advanced or Metastatic Solid Tumors	I

CDK12 aberration

NCT ID	Title	Phase
NCT04497116	Phase I/IIa Study of the Safety, Pharmacokinetics, Pharmacodynamics and Preliminary Clinical Activity of RP-3500 Alone or in Combination With Talazoparib in Advanced Solid Tumors With ATR Inhibitor Sensitizing Mutations (TRESR Study)	I/II
NCT04901702	A Randomized Phase I/II Study of Talazoparib or Temozolomide in Combination With Onivyde in Children With Recurrent Solid Malignancies and Ewing Sarcoma	I/II
NCT03155620	NCI-COG Pediatric MATCH (Molecular Analysis for Therapy Choice) Screening Protocol	II
NCT03233204	NCI-COG Pediatric MATCH (Molecular Analysis for Therapy Choice)- Phase 2 Subprotocol of Olaparib in Patients With Tumors Harboring Defects in DNA Damage Repair Genes	II
NCT04267939	An Open-label Phase Ib Study to Determine the Maximum Tolerated and/or Recommended Phase II Dose of the ATR Inhibitor BAY 1895344 in Combination With PARP Inhibitor Niraparib, in Patients With Recurrent Advanced Solid Tumors and Ovarian Cancer	I
NCT04693468	Modular Phase IB Hypothesis-Testing, Biomarker-Driven, Talazoparib Combination Trial (TalaCom)	I
NCT04633902	Phase II Study of Olaparib in Combination With Pembrolizumab in Patients With Advanced Melanoma With Homologous Recombination (HR) Pathway Gene Mutation	II
NCT03506997	PERSEUS1: Phase II Trial of the Immune Checkpoint Inhibitor Pembrolizumab for Patients Suffering From Metastatic Prostate Cancer	II
NCT04849364	A Phase II Circulating Tumor DNA Enriched, Genomically Directed Post-neoadjuvant Trial for Patients With Residual Triple Negative Breast Cancer (PERSEVERE)	II
NCT05002868	A Multi-center, Open-label, Phase I/Ib Study to Assess the Safety, Pharmacokinetics and Anti-tumor Activity of RP12146, a Poly (ADP-ribose) Polymerase (PARP) Inhibitor, in Patients With Locally Advanced or Metastatic Solid Tumors	I

Disclaimer: The data presented here is from a curated knowledgebase of publicly available information, but may not be exhaustive. The data version is 2021.11(003).

Sample Cancer Type: Unknown Primary Origin

Table of Contents

Table of Contents	Page
Relevant Therapy Details	2
Clinical Trials	5
Relevant Therapy Summary	40
Clinical Trials Summary	43

Report Highlights

3 Relevant Biomarkers
1 Therapies Available
49 Clinical Trials

Relevant Biomarkers

Genomic Alteration	Relevant Therapies (In this cancer type)	Relevant Therapies (In other cancer type)	Clinical Trials
CHEK1 mutation<br>**Prognostic significance:** None<br>**Diagnostic significance:** None	None	**olaparib** [1]	49
CHEK1 truncating mutation<br>**Prognostic significance:** None<br>**Diagnostic significance:** None	None	**olaparib** [1]	49
CHEK1 deletion<br>**Prognostic significance:** None<br>**Diagnostic significance:** None	None	None	15

Public data sources included in relevant therapies: FDA1, NCCN, **EMA**2, ESMO
Public data sources included in prognostic and diagnostic significance: NCCN, ESMO

Disclaimer: The data presented here is from a curated knowledgebase of publicly available information, but may not be exhaustive. The data version is 2021.11(003).The content of this report has not been evaluated or approved by the FDA or other regulatory agencies.

Relevant Therapy Details

Current FDA Information

● In this cancer type ○ In other cancer type ◐ In this cancer type and other cancer types

FDA information is current as of 2021-10-13. For the most up-to-date information, search www.fda.gov.

CHEK1 mutation

○ olaparib

Cancer type: Castration-Resistant Prostate Cancer **Label as of:** 2021-03-11 **Variant class:** CHEK1 mutation

Indications and usage:

LYNPARZA® is a poly (ADP-ribose) polymerase (PARP) inhibitor indicated:

Ovarian cancer

- for the maintenance treatment of adult patients with deleterious or suspected deleterious germline or somatic BRCA-mutated advanced epithelial ovarian, fallopian tube or primary peritoneal cancer who are in complete or partial response to first-line platinum-based chemotherapy. Select patients for therapy based on an FDA-approved companion diagnostic for LYNPARZA®.
- in combination with bevacizumab for the maintenance treatment of adult patients with advanced epithelial ovarian, fallopian tube or primary peritoneal cancer who are in complete or partial response to first-line platinum-based chemotherapy and whose cancer is associated with homologous recombination deficiency (HRD)-positive status defined by either:
  - a deleterious or suspected deleterious BRCA mutation, and/or
  - genomic instability.

Select patients for therapy based on an FDA-approved companion diagnostic for LYNPARZA®.

- for the maintenance treatment of adult patients with recurrent epithelial ovarian, fallopian tube or primary peritoneal cancer, who are in complete or partial response to platinum-based chemotherapy.
- for the treatment of adult patients with deleterious or suspected deleterious germline BRCA-mutated (gBRCAm) advanced ovarian cancer who have been treated with three or more prior lines of chemotherapy. Select patients for therapy based on an FDA-approved companion diagnostic for LYNPARZA®.

Breast cancer

- for the treatment of adult patients with deleterious or suspected deleterious gBRCAm, HER2-negative metastatic breast cancer who have been treated with chemotherapy in the neoadjuvant, adjuvant or metastatic setting. Patients with hormone receptor (HR)-positive breast cancer should have been treated with a prior endocrine therapy or be considered inappropriate for endocrine therapy. Select patients for therapy based on an FDA-approved companion diagnostic for LYNPARZA®.

Pancreatic cancer

- for the maintenance treatment of adult patients with deleterious or suspected deleterious gBRCAm metastatic pancreatic adenocarcinoma whose disease has not progressed on at least 16 weeks of a first-line platinum-based chemotherapy regimen. Select patients for therapy based on an FDA-approved companion diagnostic for LYNPARZA®.

Prostate cancer

- for the treatment of adult patients with deleterious or suspected deleterious germline or somatic homologous recombination repair (HRR) gene-mutated metastatic castration-resistant prostate cancer (mCRPC) who have progressed following prior treatment with enzalutamide or abiraterone. Select patients for therapy based on an FDA-approved companion diagnostic for LYNPARZA®.

Reference:

https://www.accessdata.fda.gov/drugsatfda_docs/label/2021/208558s019s020lbl.pdf

CHEK1 truncating mutation

olaparib

Cancer type: Castration-Resistant Prostate Cancer **Label as of:** 2021-03-11 **Variant class:** CHEK1 mutation

Indications and usage:

LYNPARZA® is a poly (ADP-ribose) polymerase (PARP) inhibitor indicated:

Ovarian cancer

- for the maintenance treatment of adult patients with deleterious or suspected deleterious germline or somatic BRCA-mutated advanced epithelial ovarian, fallopian tube or primary peritoneal cancer who are in complete or partial response to first-line platinum-based chemotherapy. Select patients for therapy based on an FDA-approved companion diagnostic for LYNPARZA®.
- in combination with bevacizumab for the maintenance treatment of adult patients with advanced epithelial ovarian, fallopian tube or primary peritoneal cancer who are in complete or partial response to first-line platinum-based chemotherapy and whose cancer is associated with homologous recombination deficiency (HRD)-positive status defined by either:
  - a deleterious or suspected deleterious BRCA mutation, and/or
  - genomic instability.

Select patients for therapy based on an FDA-approved companion diagnostic for LYNPARZA®.

- for the maintenance treatment of adult patients with recurrent epithelial ovarian, fallopian tube or primary peritoneal cancer, who are in complete or partial response to platinum-based chemotherapy.
- for the treatment of adult patients with deleterious or suspected deleterious germline BRCA-mutated (gBRCAm) advanced ovarian cancer who have been treated with three or more prior lines of chemotherapy. Select patients for therapy based on an FDA-approved companion diagnostic for LYNPARZA®.

Breast cancer

- for the treatment of adult patients with deleterious or suspected deleterious gBRCAm, HER2-negative metastatic breast cancer who have been treated with chemotherapy in the neoadjuvant, adjuvant or metastatic setting. Patients with hormone receptor (HR)-positive breast cancer should have been treated with a prior endocrine therapy or be considered inappropriate for endocrine therapy. Select patients for therapy based on an FDA-approved companion diagnostic for LYNPARZA®.

Pancreatic cancer

- for the maintenance treatment of adult patients with deleterious or suspected deleterious gBRCAm metastatic pancreatic adenocarcinoma whose disease has not progressed on at least 16 weeks of a first-line platinum-based chemotherapy regimen. Select patients for therapy based on an FDA-approved companion diagnostic for LYNPARZA®.

Prostate cancer

- for the treatment of adult patients with deleterious or suspected deleterious germline or somatic homologous recombination repair (HRR) gene-mutated metastatic castration-resistant prostate cancer (mCRPC) who have progressed following prior treatment with enzalutamide or abiraterone. Select patients for therapy based on an FDA-approved companion diagnostic for LYNPARZA®.

Reference:

https://www.accessdata.fda.gov/drugsatfda_docs/label/2021/208558s019s020lbl.pdf

Disclaimer: The data presented here is from a curated knowledgebase of publicly available information, but may not be exhaustive. The data version is 2021.11(003).

Current NCCN Information

● In this cancer type ○ In other cancer type ◐ In this cancer type and other cancer types

NCCN information is current as of 2021-10-01. For the most up-to-date information, search www.nccn.org.
For NCCN International Adaptations & Translations, search www.nccn.org/global/international_adaptations.aspx.

CHEK1 mutation

○ olaparib

Cancer type: Castration-Resistant Prostate Cancer **Variant class:** CHEK1 mutation

NCCN Recommendation category: 1

Population segment (Line of therapy):
- Adenocarcinoma; Metastatic (Subsequent therapy); Useful in certain circumstances

Reference: NCCN Guidelines® - NCCN-Prostate Cancer [Version 1.2022]

○ olaparib

Cancer type: Castration-Resistant Prostate Cancer **Variant class:** CHEK1 mutation

NCCN Recommendation category: 2B

Population segment (Line of therapy):
- Adenocarcinoma; Visceral Metastases (Subsequent therapy); Useful in certain circumstances

Reference: NCCN Guidelines® - NCCN-Prostate Cancer [Version 1.2022]

CHEK1 truncating mutation

○ olaparib

Cancer type: Castration-Resistant Prostate Cancer **Variant class:** CHEK1 mutation

NCCN Recommendation category: 1

Population segment (Line of therapy):
- Adenocarcinoma; Metastatic (Subsequent therapy); Useful in certain circumstances

Reference: NCCN Guidelines® - NCCN-Prostate Cancer [Version 1.2022]

○ olaparib

Cancer type: Castration-Resistant Prostate Cancer **Variant class:** CHEK1 mutation

NCCN Recommendation category: 2B

Population segment (Line of therapy):
- Adenocarcinoma; Visceral Metastases (Subsequent therapy); Useful in certain circumstances

Reference: NCCN Guidelines® - NCCN-Prostate Cancer [Version 1.2022]

Disclaimer: The data presented here is from a curated knowledgebase of publicly available information, but may not be exhaustive. The data version is 2021.11(003).

Current Clinical Trials Information

Clinical Trials information is current as of 2021-10-01. For the most up-to-date information regarding a particular trial, search www.clinicaltrials.gov by NCT ID or search local clinical trials authority website by local identifier listed in 'Other identifiers'.

CHEK1 mutation + CHEK1 truncating mutation + CHEK1 deletion

NCT04126070

A Phase II Multicohort Study of Nivolumab in Combination With Docetaxel and Androgen Deprivation Therapy in Metastatic Hormone Sensitive Prostate Cancer Patients With DNA Damage Repair Defects or Inflamed Tumors

Cancer type: Prostate Cancer

Variant classes: DNA repair deletion & DNA repair mutation

Other identifiers: 19-384, NCI-2020-02149

Population segments: First line, Stage IV

Phase: II

Therapies: nivolumab, chemotherapy, hormone therapy

Location: United States

US State: MA

Contact: Dr. Xiao X. Wei [617-632-4524; xiaox_wei@dfci.harvard.edu]

CHEK1 mutation

NCT03742895

A Phase II Study of Olaparib Monotherapy in Participants With Previously Treated, Homologous Recombination Repair Mutation (HRRm) or Homologous Recombination Deficiency (HRD) Positive Advanced Cancer

Cancer type: Unspecified Solid Tumor

Variant class: CHEK1 mutation

Other identifiers: (MK-7339-002 / LYNK-002), 19-139, 194694, 2018-01891, 7339-002, 7339-002-00, EudraCT Number: 2018-003007-19, IRAS ID: 255158, JapicCTI-194694, LYNK002, LYNK-002, MK7339-002, MK-7339-002, MK7339-002-00, NCI-2018-03519, PER-046-18, SNCTP000003157

Population segments: BRCA, Fourth line or greater, Second line, Stage III, Stage IV, Third line

Exclusion criteria variant classes: BRCA germline mutation (Breast Cancer, Ovarian Cancer), BRCA mutation (Ovarian Cancer)

Phase: II

Therapy: olaparib

Locations: Argentina, Australia, Canada, Colombia, Denmark, France, Guatemala, Ireland, Israel, Italy, Mexico, Peru, Republic of Korea, Romania, Russian Federation, Spain, Switzerland, Turkey, United Kingdom, United States

US States: AZ, CA, CO, GA, KY, MA, MD, MI, NE, NJ, NY, SD, UT, WA

Contact: Toll Free Number [888-577-8839; Trialsites@merck.com]

Disclaimer: The data presented here is from a curated knowledgebase of publicly available information, but may not be exhaustive. The data version is 2021.11(003).

CHEK1 mutation (continued)

NCT03967938
Efficacy of Olaparib in Advanced Cancers Occurring in Patients With Germline Mutations or Somatic Tumor Mutations in Homologous Recombination Genes

Cancer type: Unspecified Solid Tumor

Variant class: CHEK1 mutation

Other identifiers: 1-2018 BSMO, 1-2018BSMO, EudraCT Number: 2018-002966-37, Precision 2 - Olaparib

Population segments: Line of therapy N/A, Stage III, Stage IV

Exclusion criteria variant classes: BRCA1 germline mutation (Breast Cancer, Pancreatic Cancer, Prostate Cancer), BRCA2 germline mutation (Breast Cancer, Pancreatic Cancer, Prostate Cancer)

Phase: II

Therapy: olaparib

Location: Belgium

NCT04550494
A Pharmacodynamics-Driven Trial of Talazoparib, an Oral PARP Inhibitor, in Patients With Advanced Solid Tumors and Aberrations in Genes Involved in DNA Damage Response

Cancer type: Unspecified Solid Tumor

Variant class: CHEK1 mutation

Other identifiers: 000246, 000264-C, 10000264, 10371, NCI-2020-06906

Population segments: BRCA, Fourth line or greater, HER2 positive, Hormone refractory, Second line, Stage II, Stage III, Stage IV, Third line

Phase: II

Therapy: talazoparib

Location: United States

US State: MD

Contact: Multiple contacts: See www.clinicaltrials.gov for complete list of contacts.

NCT03842228
A Phase Ib Biomarker-Driven Combination Trial of Copanlisib, Olaparib, and Durvalumab (MEDI4736) in Patients With Advanced Solid Tumors

Cancer type: Unspecified Solid Tumor

Variant class: CHEK1 mutation

Other identifiers: 10217, 19-628, NCI10217, NCI-2019-00601

Population segments: Line of therapy N/A, Stage III, Stage IV

Phase: I

Therapies: copanlisib, olaparib, durvalumab

Location: United States

US States: MA, TX

Contact: Multiple contacts: See www.clinicaltrials.gov for complete list of contacts.

Disclaimer: The data presented here is from a curated knowledgebase of publicly available information, but may not be exhaustive. The data version is 2021.11(003).

CHEK1 mutation (continued)

NCT04586335
Open Label, Phase Ib Study to Evaluate the Safety, Tolerability, Pharmacokinetics and Clinical Activity of CYH33, an Oral a-specific PI3K Inhibitor in Combination With Olaparib, an Oral PARP Inhibitor in Patients With Advanced Solid Tumors.

Cancer type: Breast Cancer, Endometrial Carcinoma, Ovarian Cancer, Prostate Cancer, Unspecified Solid Tumor

Variant class: CHEK1 mutation

Other identifiers: CTR20202339, CYH33-G102

Population segments: BRCA, Second line, Stage III, Stage IV

Phase: I

Therapies: HH-CYH33, olaparib

Locations: Australia, United States

US State: TX

Contact: Dr. Jason Sudia [908-380-1329; jason.sudia@haihepharma.com]

NCT02029001
A Two-period, Multicenter, Randomized, Open-label, Phase II Study Evaluating the Clinical Benefit of a Maintenance Treatment Targeting Tumor Molecular Alterations in Patients with Progressive Locally-advanced or Metastatic Solid Tumors.

Cancer type: Unspecified Solid Tumor

Variant class: HRR mutation

Other identifiers: ET12-081, EudraCT Number: 2012-004510-34, MOST, ProfiLER

Population segments: Maintenance/Consolidation, Second line, Stage III, Stage IV, Third line

Exclusion criteria variant classes: ATM mutation (Gastric Cancer), BRCA1 germline mutation (Gastric Cancer), BRCA1 mutation (Gastric Cancer), BRCA2 germline mutation (Gastric Cancer), BRCA2 mutation (Gastric Cancer), HRR mutation (Gastric Cancer), PALB2 mutation (Gastric Cancer), RAD51 mutation (Gastric Cancer)

Phase: II

Therapy: olaparib

Location: France

NCT04123366
A Phase II Study of Olaparib in Combination With Pembrolizumab in Participants With Previously Treated, Homologous Recombination Repair Mutation (HRRm) and/or Homologous Recombination Deficiency (HRD)-Positive Advanced Cancer

Cancer type: Unspecified Solid Tumor

Variant class: HRR mutation

Other identifiers: 7339-007, EudraCT Number: 2019-001745-40, jRCT2011200025, KEYLYNK-007, MK-7339-007, NCI-2019-08758, PER-029-19

Population segments: BRCA, Second line, Stage III, Stage IV

Exclusion criteria variant classes: BRCA germline mutation (Breast Cancer, Ovarian Cancer), BRCA mutation (Breast Cancer, Ovarian Cancer)

Phase: II

Therapies: olaparib, pembrolizumab

Locations: Argentina, Australia, Canada, Colombia, France, Germany, Guatemala, Israel, Italy, Japan, Latvia, Mexico, Peru, Poland, Puerto Rico, Republic of Korea, Romania, South Africa, Spain, Sweden, Turkey, Ukraine, United States

US States: AL, AZ, CA, CO, FL, GA, KY, NJ, NY, OH, TX, UT, VA

Contact: Toll Free Number [888-577-8839; Trialsites@merck.com]

Disclaimer: The data presented here is from a curated knowledgebase of publicly available information, but may not be exhaustive. The data version is 2021.11(003).

CHEK1 mutation (continued)

NCT02401347

A Phase II Clinical Trial of the PARP Inhibitor Talazoparib in BRCA1 and BRCA2 Wild Type Patients With Advanced Triple Negative Breast Cancer and Homologous Recombination Deficiency or Advanced HER2 Negative Breast Cancer or Other Solid Tumors With a Mutation in Homologous Recombination Pathway Genes Talazoparib Beyond BRCA (TBB) Trial

Cancer type: Breast Cancer, Unspecified Solid Tumor

Variant class: HRR mutation

Other inclusion criteria: ERBB2 negative, Hormone receptor positive

Other identifiers: BRS0050, NCI-2015-00036, TBB

Population segments: Estrogen receptor positive, HER2 negative, Progesterone receptor positive, Second line, Stage III, Stage IV, Triple receptor negative

Exclusion criteria variant classes: BRCA1 germline mutation (Breast Cancer, Triple Negative Breast Cancer, Unspecified Solid Tumor), BRCA1 mutation (Breast Cancer, Triple Negative Breast Cancer, Unspecified Solid Tumor), BRCA2 germline mutation (Breast Cancer, Triple Negative Breast Cancer, Unspecified Solid Tumor), BRCA2 mutation (Breast Cancer, Triple Negative Breast Cancer, Unspecified Solid Tumor), ERBB2 positive (Breast Cancer)

Phase: II

Therapy: talazoparib

Location: United States

US State: CA

Contact: Amanda Selin [650-721-6977; aselin@stanford.edu]

NCT03415659

A Phase I, Open-label, Single-center, Single/Multiple-dose, Dose-escalation/ Dose-expansion Clinical Study on Tolerance and Pharmacokinetics of HWH340 Tablet in Patients With Advanced Solid Tumors

Cancer type: Unspecified Solid Tumor

Variant class: HRR mutation

Other inclusion criteria: BRCA1 germline mutation negative, BRCA1 mutation negative, BRCA2 germline mutation negative, BRCA2 mutation negative

Other identifiers: CTR20171649, HWH340-RFPA 20170821, RFPA 20170821

Population segments: BRCA, Fourth line or greater, HER2 negative, Second line, Stage III, Stage IV, Third line

Phase: I

Therapy: HWH-340

Location: China

NCT03767075

Basket of Baskets: A Modular, Open-label, Phase II, Multicentre Study To Evaluate Targeted Agents in Molecularly Selected Populations With Advanced Solid Tumours

Cancer type: Unspecified Solid Tumor

Variant class: DNA repair mutation

Other identifiers: (Basket of Baskets) (BoB), EudraCT number: 2017-005108-89, MO39164, NL65937.031.18, VHIO17002

Population segments: Second line, Stage III, Stage IV

Phase: II

Therapy: atezolizumab

Locations: France, Germany, Netherlands, Spain, Sweden, United Kingdom

---

Disclaimer: The data presented here is from a curated knowledgebase of publicly available information, but may not be exhaustive. The data version is 2021.11(003).

CHEK1 mutation (continued)

NCT04905914
A Phase I/IIa, Open-Label, Safety, Pharmacokinetic, And Preliminary Efficacy Study Of Oral ATRN-119 In Patients With Advanced Solid Tumors

Cancer type: Unspecified Solid Tumor

Variant class: DNA repair mutation

Other identifier: AR-276-01

Population segments: Line of therapy N/A, Stage III, Stage IV

Phase: I/II

Therapy: ATRN-119

Location: United States

US State: MA

Contact: Robert Hasson [619-540-6253; rhasson@pacificlinkconsulting.com]

NCT04901702
A Randomized Phase I/II Study of Talazoparib or Temozolomide in Combination With Onivyde in Children With Recurrent Solid Malignancies and Ewing Sarcoma

Cancer type: Unspecified Solid Tumor

Variant class: HRR pathway

Other identifiers: EudraCT Number: 2021-003570-31, ONITT

Population segments: Second line, Stage IV

Exclusion criteria variant classes: HRR germline pathway (Central Nervous System Neoplasm, Ewing Sarcoma), HRR pathway (Central Nervous System Neoplasm, Ewing Sarcoma)

Phase: I/II

Therapies: talazoparib, chemotherapy

Locations: Canada, United States

US State: TN

Contact: Dr. Sara Federico [866-278-5833; referralinfo@stjude.org]

NCT03155620
NCI-COG Pediatric MATCH (Molecular Analysis for Therapy Choice) Screening Protocol

Cancer type: Unspecified Solid Tumor

Variant class: DNA repair pathway

Other identifiers: 17-729, 2017-0625, 20170841, APEC1621SC, COGAPEC1621, COGAPEC1621SC, N 55017 SC, NCI-2017-01251, NCI-COG Pediatric MATCH, Pediatric MATCH

Population segments: ALK, Aggressive, BRAF, FGFR, HRAS, Indolent, KRAS, NRAS, Pediatric or Adolescent, Second line, Stage III, Stage IV

Phase: II

Therapy: olaparib

Locations: Puerto Rico, United States

US States: AK, AL, AR, AZ, CA, CO, CT, DC, DE, FL, GA, HI, IA, ID, IL, IN, KY, LA, MA, MD, ME, MI, MN, MO, MS, NC, NE, NH, NJ, NV, NY, OH, OK, OR, PA, RI, SC, SD, TN, TX, UT, VA, VT, WA, WI

Contact: Multiple contacts: See www.clinicaltrials.gov for complete list of contacts.

Disclaimer: The data presented here is from a curated knowledgebase of publicly available information, but may not be exhaustive. The data version is 2021.11(003).

CHEK1 mutation (continued)

NCT03233204
NCI-COG Pediatric MATCH (Molecular Analysis for Therapy Choice)- Phase 2 Subprotocol of Olaparib in Patients With Tumors Harboring Defects in DNA Damage Repair Genes

Cancer type: Unspecified Solid Tumor

Variant class: DNA repair pathway

Other identifiers: 20170841, APEC1621H, NCI-2017-00766

Population segments: Aggressive, Indolent, Pediatric or Adolescent, Second line, Stage III, Stage IV

Phase: II

Therapy: olaparib

Locations: Puerto Rico, United States

US States: AK, AL, AR, AZ, CA, CO, DC, DE, FL, GA, IA, ID, IL, IN, KY, LA, MD, ME, MI, MN, MO, MS, NC, ND, NE, NJ, NY, OH, OK, OR, PA, SC, SD, TN, TX, UT, VA, VT, WA, WI

Contact: Multiple contacts: See www.clinicaltrials.gov for complete list of contacts.

NCT04267939
An Open-label Phase Ib Study to Determine the Maximum Tolerated and/or Recommended Phase II Dose of the ATR Inhibitor BAY 1895344 in Combination With PARP Inhibitor Niraparib, in Patients With Recurrent Advanced Solid Tumors and Ovarian Cancer

Cancer type: Ovarian Cancer, Unspecified Solid Tumor

Variant class: DNA repair pathway

Other identifiers: 18595, 19-810, 2019-0471, 20-222, EudraCT Number: 2018-003930-34, NCI-2020-01405

Population segments: Maintenance/Consolidation, Second line, Stage III, Stage IV

Exclusion criteria variant class: DNA repair pathway (Prostate Cancer)

Phase: I

Therapies: BAY-1895344, niraparib

Location: United States

US States: MA, NY, TX

Contact: Bayer Clinical Trials Contact [888-842-2937; clinical-trials-contact@bayer.com]

NCT04693468
Modular Phase IB Hypothesis-Testing, Biomarker-Driven, Talazoparib Combination Trial (TalaCom)

Cancer type: Unspecified Solid Tumor

Variant class: DNA repair pathway

Other identifiers: 2020-0436, NCI-2020-06041, TalaCom

Population segments: Hormone refractory, Second line, Stage III, Stage IV

Phase: I

Therapies: talazoparib, palbociclib, axitinib, crizotinib

Location: United States

US State: TX

Contact: Timothy A. Yap [713-563-1784; tyap@mdanderson.org]

Disclaimer: The data presented here is from a curated knowledgebase of publicly available information, but may not be exhaustive. The data version is 2021.11(003).

CHEK1 mutation (continued)

NCT04053322
An International Multicenter Phase II Trial of Durvalumab (MEDI4736) Plus OLAparib Plus Fulvestrant in Metastatic or Locally Advanced ER-positive, HER2-negative Breast Cancer Patients Selected Using Criteria That Predict Sensitivity to Olaparib

Cancer type: Breast Cancer

Variant class: CHEK1 mutation

Other inclusion criteria: ERBB2 negative, ER positive

Other identifiers: CADUSEIME08, DOLAF, EudraCT Number: 2018-003832-57, UC-0140/1812

Population segments: BRCA, Estrogen receptor positive, FGFR, HER2 negative, Progesterone receptor positive, Second line, Stage III, Stage IV

Phase: II

Therapies: durvalumab, olaparib, hormone therapy

Location: France

NCT03553004
Niraparib in Metastatic Pancreatic Cancer After Previous Chemotherapy (NIRA-PANC): A Phase II Trial

Cancer type: Pancreatic Cancer

Variant class: CHEK1 mutation

Other identifiers: IIT-2017-NIRA-PANC, NIRA-PANC, STUDY00141862

Population segments: BRCA, Second line, Stage IV, Third line

Phase: II

Therapy: niraparib

Location: United States

US States: KS, MO

Contact: Clinical Trials Nurse Navigator [913-588-3671; navigation@kumc.edu]

NCT04030559
Phase II Trial of PARP Inhibitor Niraparib for Men With High Risk Prostate Cancer and DNA Damage Response Defects

Cancer type: Prostate Cancer

Variant class: CHEK1 mutation

Other identifiers: 1412676, NCI-2019-04365, UCDCC#279

Population segments: First line, Neoadjuvant, Stage I, Stage II, Stage IV

Phase: II

Therapy: niraparib

Location: United States

US State: CA

Contact: Marc Dall'Era [916-734-3771; mdallera@ucdavis.edu]

Disclaimer: The data presented here is from a curated knowledgebase of publicly available information, but may not be exhaustive. The data version is 2021.11(003).

CHEK1 mutation (continued)

NCT04187833
Phase II Trial of Nivolumab in Combination with Talazoparib in Patients with Unresectable or Metastatic Melanoma and Mutations in BRCA or BRCA-ness Genes.

Cancer type: Melanoma

Variant class: CHEK1 mutation

Other identifiers: CASE2619, NCI-2020-00416

Population segments: Second line, Stage III, Stage IV

Phase: II

Therapies: nivolumab, talazoparib

Location: United States

US State: OH

Contact: Dr. Pauline Funchain [866-223-8100; TaussigResearch@ccf.org]

NCT03375307
A Phase II Study of Olaparib (AZD2281) in Patients With Metastatic/Advanced Urothelial Carcinoma With DNA-Repair Defects

Cancer type: Bladder Urothelial Carcinoma

Variant class: CHEK1 mutation

Other identifiers: 10144, 19C0023, NCI-2017-02296, P162941

Population segments: BRCA, STK11, Second line, Stage III, Stage IV

Phase: II

Therapy: olaparib

Location: United States

US States: CA, CO, KY, MD, NY, OK

Contact: Multiple contacts: See www.clinicaltrials.gov for complete list of contacts.

NCT03786796
Phase II Study of Olaparib in Metastatic Renal Cell Carcinoma Patients Harboring a BAP-1 or Other DNA Repair Gene Mutations (ORCHID)

Cancer type: Kidney Cancer

Variant class: CHEK1 mutation

Other identifiers: CRMS-70750, J18166, NCI-2019-06309, ORCHID

Population segments: Fourth line or greater, Second line, Stage IV, Third line

Phase: II

Therapy: olaparib

Location: United States

US State: MD

Contact: Irina Rifkind [410-502-2043; irifkin1@jhmi.edu]

NCT04515836
Phase II Trial of Olaparib in Homologous Recombination Deficient (HRD) Malignant Mesothelioma

Cancer type: Mesothelioma

Variant class: CHEK1 mutation

Population segments: (N/A), Second line

Phase: II

Therapy: olaparib

Location: United States

US State: IL

Contact: Dr. Hedy L. Kindler [773-702-0360; hkindler@medicine.bsd.uchicago.edu]

Disclaimer: The data presented here is from a curated knowledgebase of publicly available information, but may not be exhaustive. The data version is 2021.11(003).

CHEK1 mutation (continued)

NCT03533946
A Phase II Study of Rucaparib Monotherapy in Nonmetastatic, Hormone-Sensitive Prostate Cancer Demonstrating "BRCAness" Genotype (ROAR)

Cancer type: Prostate Cancer

Variant class: CHEK1 mutation

Other identifiers: HCI111833, NCI-2018-01754, ROAR

Population segments: First line, Stage I, Stage II, Stage III

Phase: II

Therapy: rucaparib

Location: United States

US State: UT

Contact: Jill Broghammer [801-213-6232; jill.broghammer@hci.utah.edu]

NCT04892693
Talazoparib in Advanced Breast Cancer Patients With Homologous Recombinant Deficiency: A Phase II Clinical and Exploratory Biomarker Study of Talazoparib

Cancer type: Breast Cancer

Variant class: CHEK1 mutation

Other identifier: H-2009-079-1157

Population segments: BRCA, Line of therapy N/A, Stage III, Stage IV

Phase: II

Therapy: talazoparib

Location: Republic of Korea

NCT04740190
Combination Talazoparib - Carboplatin for Recurrent High-grade Glioma With DNA Damage Repair Deficiency (DDRd)

Cancer type: Anaplastic Astrocytoma, Anaplastic Oligoastrocytoma, Anaplastic Oligodendroglioma, Anaplastic Pleomorphic Xanthoastrocytoma, Diffuse Midline Glioma, Glioblastoma

Variant class: CHEK1 mutation

Other identifiers: TAC-GReD, UW-20396

Population segments: (N/A), BRCA, Second line

Phase: II

Therapies: talazoparib, chemotherapy

Location: Hong Kong

NCT04253262
A Phase Ib/II Study of Copanlisib Combined With Rucaparib in Patients With Metastatic Castration-resistant Prostate Cancer

Cancer type: Castration-Resistant Prostate Cancer

Variant class: CHEK1 mutation

Other identifiers: BrUOG 360, BrUOG360

Population segments: Hormone refractory, Second line, Stage IV

Phase: I/II

Therapies: copanlisib, rucaparib

Location: United States

US State: RI

Contact: BrUOG [401-863-3000; BrUOG@brown.edu]

Disclaimer: The data presented here is from a curated knowledgebase of publicly available information, but may not be exhaustive. The data version is 2021.11(003).

CHEK1 mutation (continued)

NCT04336943
Durvalumab (MEDI4736) and Olaparib (AZD2281) for Treatment of Biochemically Recurrent Prostate Cancer in Men Predicted to Have a High Neoantigen Load: A Pilot Study

Cancer type: Prostate Adenocarcinoma

Variant class: HRR mutation

Other identifiers: 10509, NCI-2020-01860, RG1007001

Population segments: Line of therapy N/A, Stage I, Stage II, Stage III

Phase: II

Therapies: durvalumab, olaparib

Location: United States

US State: WA

Contact: Michael Schweizer [206-606-6252; schweize@uw.edu]

NCT04169841
Precision Medicine Phase II Study Evaluating the Efficacy of a Double Immunotherapy by Durvalumab and Tremelimumab Combined With Olaparib in Patients With Solid Cancers and Carriers of Homologous Recombination Repair Genes Mutation in Response or Stable After Olaparib Treatment

Cancer type: Breast Cancer, Endometrial Carcinoma, Head and Neck Cancer, Kidney Cancer, Non-Small Cell Lung Cancer, Ovarian Cancer, Pancreatic Cancer, Prostate Cancer

Variant class: HRR mutation

Other identifier: GUIDE2REPAIR

Population segments: Fourth line or greater, Hormone refractory, Maintenance/ Consolidation, Second line, Stage III, Stage IV, Third line

Exclusion criteria variant classes: ALK fusion (Lung Cancer), BRAF mutation (Lung Cancer), EGFR mutation (Lung Cancer), ROS1 mutation (Lung Cancer)

Phase: II

Therapies: durvalumab, tremelimumab, olaparib

Location: France

NCT03925350
A Phase II Study of Niraparib in Patients With Advanced Melanoma With Genetic Homologous Recombination (HR) Mutation / Alteration

Cancer type: Melanoma

Variant class: HRR mutation

Other identifier: CPMC17-MEL01

Population segments: Second line, Stage III, Stage IV

Phase: II

Therapy: niraparib

Location: United States

US State: CA

Contact: Laurel Brechtel [415-600-1654; BrechtLA@sutterhealth.org]

Disclaimer: The data presented here is from a curated knowledgebase of publicly available information, but may not be exhaustive. The data version is 2021.11(003).

CHEK1 mutation (continued)

NCT04940637

A Phase II, Open-Label, Single Arm, Prospective, Multicenter Study of Niraparib Plus Dostarlimab in Patients With Advanced NSCLC and/or MPM, and Positive for PD-L1 Expression and Germline or Somatic Mutations in the HRR Genes

Cancer type: Mesothelioma, Non-Small Cell Lung Cancer

Variant class: HRR mutation

Other inclusion criteria: ALK fusion negative, CD274 expression, EGFRi sensitizing mutation negative, ROS1 fusion negative or CD274 expression

Other identifiers: EudraCT Number: 2020-000109-10, UNITO-001, UNITO-001/2020

Population segments: Second line, Stage III, Stage IV

Exclusion criteria variant classes: ALK fusion (Non-Small Cell Lung Cancer), EGFRi sensitizing mutation (Non-Small Cell Lung Cancer), ROS1 fusion (Non-Small Cell Lung Cancer)

Phase: II

Therapies: niraparib, dostarlimab

Location: Italy

NCT04042831

A Phase II Study of Olaparib in Patients With Advanced Biliary Tract Cancer With Aberrant DNA Repair Gene Mutations

Cancer type: Biliary Tract Carcinoma

Variant class: HRR mutation

Other identifiers: ACCRU-ICRN-1702, NCI-2019-04434

Population segments: Second line, Stage IV

Phase: II

Therapy: olaparib

Location: United States

US States: AZ, MN, NY, TX

Contact: Multiple contacts: See www.clinicaltrials.gov for complete list of contacts.

NCT04939662

Phase II, Single-arm Study of Olaparib and Bevacizumab Combination Therapy in Relapsed Small Cell Lung Cancer Subjects With DNA Damage Response and the Repair Pathway Alteration, ATM Deficiency, SLFN11 Positive, or POU2F3 Positive

Cancer type: Small Cell Lung Cancer

Variant class: HRR mutation

Other identifier: SUKSES-B2

Population segments: (N/A), BRCA, Pulmonary, Second line, Third line

Phase: II

Therapies: olaparib, bevacizumab

Location: Republic of Korea

NCT03506997

PERSEUS1: Phase II Trial of the Immune Checkpoint Inhibitor Pembrolizumab for Patients Suffering From Metastatic Prostate Cancer

Cancer type: Castration-Resistant Prostate Cancer

Variant class: HRR mutation

Other identifiers: CCR4559, EudraCT Number: 2017-000931-15, ICR-CTSU/2016/10060, IRAS ID 208952, PERSEUS1

Population segments: Hormone refractory, Second line, Stage IV

Phase: II

Therapy: pembrolizumab

Location: United Kingdom

CHEK1 mutation (continued)

NCT02264678
A Modular Phase I, Open-Label, Multicentre Study to Assess the Safety, Tolerability, Pharmacokinetics and Preliminary Anti-tumour Activity of Ceralasertib in Combination With Cytotoxic Chemotherapy and/or DNA Damage Repair/Novel Anti-cancer Agents in Patients With Advanced Solid Malignancies

Cancer type: Triple Negative Breast Cancer

Variant class: HRR mutation

Other inclusion criteria: BRCA germline mutation negative, BRCA mutation negative

Other identifiers: 0C-14-9, 16-031, 18-342, D5330C00004, EudraCT Number: 2014-002233-66, IRAS ID 160200, NCI-2015-00687, Study 4

Population segments: Adenocarcinoma, BRCA, HER2 negative, Pulmonary, Second line, Stage III, Stage IV, Third line, Triple receptor negative

Exclusion criteria variant class: ERBB2 positive (Breast Cancer)

Phase: I

Therapies: ceralasertib, olaparib

Locations: France, Republic of Korea, United Kingdom, United States

US States: CA, MA, NY

Contact: AstraZeneca Clinical Study Information Center [877-240-9479; information.center@astrazeneca.com]

NCT04890613
Phase Ib Expansion Study of CX-5461 in Patients With Solid Tumours and BRCA2 and/or PALB2 Mutation

Cancer type: Ovarian Cancer

Variant class: HRR mutation

Other identifier: CX-5461-04

Population segments: BRCA, Second line, Stage II, Stage III, Stage IV

Phase: I

Therapy: pidnarulex

Location: Canada

NCT05002868
A Multi-center, Open-label, Phase I/Ib Study to Assess the Safety, Pharmacokinetics and Anti-tumor Activity of RP12146, a Poly (ADP-ribose) Polymerase (PARP) Inhibitor, in Patients With Locally Advanced or Metastatic Solid Tumors

Cancer type: Biliary Tract Carcinoma, Breast Cancer, Colorectal Cancer, Endometrial Carcinoma, Gastric Cancer, Ovarian Cancer, Pancreatic Cancer, Prostate Cancer, Small Cell Lung Cancer

Variant class: HRR mutation

Other identifier: RP12146-2101

Population segments: Extensive, Pulmonary, Second line, Stage III, Stage IV

Exclusion criteria variant class: ERBB2 positive (Breast Cancer)

Phase: I

Therapy: RP12146

Location: Poland

Disclaimer: The data presented here is from a curated knowledgebase of publicly available information, but may not be exhaustive. The data version is 2021.11(003).

CHEK1 mutation (continued)

NCT03395197
A Phase III, Randomized, Double-blind, Placebo-controlled Study Of Talazoparib With Enzalutamide In Metastatic Castration-resistant Prostate Cancer

Cancer type: Castration-Resistant Prostate Cancer

Variant class: DNA repair mutation

Other identifiers: BASIC-ROYAL / HIGH-ROYAL, C3441021, CSET 2938, CTR20191443, CTR20191444, EudraCT Number: 2017-003295-31, GU 170 - C3441021, HCC 19-054, JapicCTI-194665, LH19.022, MOH_2019-05-16_007127, NCI-2018-01651, Pfizer C3441021, R19062M, RECF3951, RWF_C3441021 TALAPRO-2, TALAPRO-2, TX228665, UCI-18-47, VICCURO17115

Population segments: First line, Hormone refractory, Second line, Stage III, Stage IV

Phase: III

Therapies: talazoparib, hormone therapy

Locations: Argentina, Australia, Belgium, Brazil, Canada, Chile, China, Czech Republic, Finland, France, Germany, Hungary, Israel, Italy, Japan, New Zealand, Norway, Peru, Poland, Portugal, Republic of Korea, South Africa, Spain, Sweden, United Kingdom, United States

US States: AZ, CA, CO, FL, GA, IL, IN, KS, MO, NE, NJ, NY, OH, OK, OR, PA, SC, TN, TX, UT, VA, WI

Contact: Pfizer CT.gov Call Center [800-718-1021; ClinicalTrials.gov_Inquiries@pfizer.com]

NCT04821622
TALAPRO-3: A Phase III, Randomized, Double-blind, Study of Talazoparib With Enzalutamide Versus Placebo With Enzalutamide in Men With DDR Gene Mutated Metastatic Castration-sensitive Prostate Cancer

Cancer type: Prostate Adenocarcinoma

Variant class: DNA repair mutation

Other identifiers: C3441052, CTR20212191, EudraCT Number: 2021-000248-23, jRCT2031210098, TALAPRO-3

Population segments: Second line, Stage IV

Phase: III

Therapies: talazoparib, hormone therapy

Locations: Japan, Republic of Korea, United States

US States: CA, DC, FL, GA, IL, MI, NJ, NY, PA, SC, TX, UT

Contact: Pfizer CT.gov Call Center [800-718-1021; ClinicalTrials.gov_Inquiries@pfizer.com]

NCT03810105
A Phase II Study of Olaparib and Durvalumab in Men With Castration Sensitive Biochemically Recurrent Non-Metastatic Prostate Cancer Harboring Mutations in DNA Damage Repair

Cancer type: Prostate Cancer

Variant class: DNA repair mutation

Other identifiers: 18-480, NCI-2019-01627

Population segments: Adjuvant, Stage I, Stage II, Stage III

Phase: II

Therapies: durvalumab, olaparib

Location: United States

US States: CA, IL, MI, NJ, NY

Contact: Dr. Karen Autio [646-422-4632; autiok@mskcc.org]

Disclaimer: The data presented here is from a curated knowledgebase of publicly available information, but may not be exhaustive. The data version is 2021.11(003).

CHEK1 mutation (continued)

NCT03012321

BRCAAway: A Randomized Phase II Trial of Abiraterone, Olaparib, or Abiraterone + Olaparib in Patients With Metastatic Castration-Resistant Prostate Cancer With DNA Repair Defects

Cancer type: Castration-Resistant Prostate Cancer

Variant class: DNA repair mutation

Other identifiers: 19137, BRCAAway, NCI-2016-01834, NU 16U05, NU_16U05, PCCTC #: c16-168, PCCTC c16-168, STU00203960

Population segments: First line, Hormone refractory, Stage IV

Phase: II

Therapy: olaparib

Location: United States

US States: CA, FL, IL, IN, MD, MI, MN, MO, NC, NY, UT, VA

Contact: Study Coordinator [312-695-1301; cancertrials@northwestern.edu]

NCT03344965

A Phase II Study of Olaparib Monotherapy in Metastatic Breast Cancer Patients With Germline or Somatic Mutations in DNA Repair Genes (Olaparib Expanded)

Cancer type: Breast Cancer

Variant class: DNA repair mutation

Other inclusion criteria: BRCA1 germline mutation negative, BRCA2 germline mutation negative

Other identifiers: 17-428, 18-414, NCI-2018-00778, TBCRC 048, TBCRC048

Population segments: BRCA, Estrogen receptor positive, HER2 negative, HER2 positive, Progesterone receptor positive, Second line, Stage IV

Exclusion criteria variant classes: BRCA1 germline mutation (Breast Cancer), BRCA2 germline mutation (Breast Cancer)

Phase: II

Therapy: olaparib

Location: United States

US States: AL, CA, IL, MA, MD, NC, NJ, NY, PA, WA

Contact: Dr. Nadine Tung [617-667-1962; ntung@bidmc.harvard.edu]

NCT04038502

An Open-label, Multicenter Phase II Study to Compare the Efficacy of Carboplatin as First-line Followed by Second-line Olaparib Versus Olaparib as First-line Followed by Second-line Carboplatin in the Treatment of Patients With Castration Resistant Prostate Cancer Containing Homologous Recombination Deficiency

Cancer type: Castration-Resistant Prostate Cancer

Variant class: DNA repair mutation

Other identifiers: 01781, 01783, 19-08, BRACeD, CX001963-01, ONCA-009-18F

Population segments: First line, Hormone refractory, Second line, Stage IV

Phase: II

Therapies: olaparib, chemotherapy

Location: United States

US States: CA, DC, FL, IL, MI, NC, NY, PA, WA

Contact: Dr. Robert B. Montgomery [206-277-6878; rbmontgo@u.washington.edu]

Disclaimer: The data presented here is from a curated knowledgebase of publicly available information, but may not be exhaustive. The data version is 2021.11(003).

CHEK1 mutation (continued)

NCT04633902
Phase II Study of Olaparib in Combination With Pembrolizumab in Patients With Advanced Melanoma With Homologous Recombination (HR) Pathway Gene Mutation

Cancer type: Melanoma

Variant class: HRR pathway

Other identifier: CPMC19-MEL01

Population segments: BRAF, Second line, Stage III, Stage IV

Phase: II

Therapies: olaparib, pembrolizumab

Location: United States

US State: CA

Contact: Dr. Kevin B. Kim [415-885-8600; ClinicalResearch@sutterhealth.org]

NCT03248570
Phase II Open Label Study of Pembrolizumab in Patients With Metastatic Castration Resistant Prostate Cancer (mCRPC) With or Without DNA Damage Repair Defects

Cancer type: Castration-Resistant Prostate Cancer

Variant class: DNA repair mutation

Other identifiers: 16557, 17-22165, CC 16557, NCI-2017-02408

Population segments: BRCA, Hormone refractory, Line of therapy N/A, Stage IV

Phase: II

Therapy: pembrolizumab

Location: United States

US State: CA

Contact: Patricia Li [415-476-5975; GUTrials@ucsf.edu]

No NCT ID - see other identifier(s)
Single-arm Exploratory Study for Sintilimab Second-line or Later-line Treatment of Advanced Non-small Cell Lung Cancer with DDR Pathway Gene Mutation

Cancer type: Non-Small Cell Lung Cancer

Variant class: DNA repair mutation

Other identifier: ChiCTR1900023234

Population segments: Fourth line or greater, Second line, Stage III, Stage IV, Third line

Exclusion criteria variant classes: ALK fusion (Non-Small Cell Lung Cancer), EGFRi sensitizing mutation (Non-Small Cell Lung Cancer), ROS1 fusion (Non-Small Cell Lung Cancer)

Phase: I/II

Therapy: sintilimab

Location: China

Disclaimer: The data presented here is from a curated knowledgebase of publicly available information, but may not be exhaustive. The data version is 2021.11(003).

CHEK1 mutation (continued)

NCT04095273

A Multicenter, Non-randomized, Open-label Phase Ib Study to Determine the Maximum Tolerated and Recommended Phase II Dose of the ATR Inhibitor BAY1895344 in Combination With Pembrolizumab and to Characterize Its Safety, Tolerability, Pharmacokinetics and Preliminary Anti-tumor Activity in Patients With Advanced Solid Tumors

Cancer type: Breast Cancer, Castration-Resistant Prostate Cancer, Colorectal Cancer, Gastric Cancer, Gastroesophageal Junction Adenocarcinoma, Non-Small Cell Lung Cancer, Pancreatic Cancer

Variant class: DNA repair mutation

Other inclusion criteria: ATM mutation negative or ATM mutation negative, ERBB2 negative, Hormone receptor positive or ATM underexpression

Other identifiers: 19741, EudraCT Number: 2018-003420-36, Keynote 919, NCI-2019-06734

Population segments: HER2 negative, Hormone refractory, Second line, Stage III, Stage IV

Phase: I

Therapies: BAY-1895344, pembrolizumab

Locations: Germany, Spain, Switzerland, United States

US States: CA, CT, MA, MD, NC, NY, OH, TX

Contact: Bayer Clinical Trials Contact [clinical-trials-contact@bayer.com]

NCT04849364

A Phase II Circulating Tumor DNA Enriched, Genomically Directed Post-neoadjuvant Trial for Patients With Residual Triple Negative Breast Cancer (PERSEVERE)

Cancer type: Triple Negative Breast Cancer

Variant class: DNA repair pathway

Other identifiers: HCRN BRE18-334, PERSEVERE

Population segments: Adjuvant, HER2 negative, Stage I, Stage II, Stage III, Triple receptor negative

Phase: II

Therapies: talazoparib, chemotherapy

Location: United States

US State: IN

Contact: Dr. Bryan P. Schneider [317-948-3885; bpschnei@iu.edu]

CHEK1 truncating mutation

NCT04550494

A Pharmacodynamics-Driven Trial of Talazoparib, an Oral PARP Inhibitor, in Patients With Advanced Solid Tumors and Aberrations in Genes Involved in DNA Damage Response

Cancer type: Unspecified Solid Tumor

Variant class: CHEK1 truncating mutation

Other identifiers: 000246, 000264-C, 10000264, 10371, NCI-2020-06906

Population segments: BRCA, Fourth line or greater, HER2 positive, Hormone refractory, Second line, Stage II, Stage III, Stage IV, Third line

Phase: II

Therapy: talazoparib

Location: United States

US State: MD

Contact: Multiple contacts: See www.clinicaltrials.gov for complete list of contacts.

Disclaimer: The data presented here is from a curated knowledgebase of publicly available information, but may not be exhaustive. The data version is 2021.11(003).

CHEK1 truncating mutation (continued)

NCT03742895
A Phase II Study of Olaparib Monotherapy in Participants With Previously Treated, Homologous Recombination Repair Mutation (HRRm) or Homologous Recombination Deficiency (HRD) Positive Advanced Cancer

Cancer type: Unspecified Solid Tumor

Variant class: CHEK1 mutation

Other identifiers: (MK-7339-002 / LYNK-002), 19-139, 194694, 2018-01891, 7339-002, 7339-002-00, EudraCT Number: 2018-003007-19, IRAS ID: 255158, JapicCTI-194694, LYNK002, LYNK-002, MK7339-002, MK-7339-002, MK7339-002-00, NCI-2018-03519, PER-046-18, SNCTP000003157

Population segments: BRCA, Fourth line or greater, Second line, Stage III, Stage IV, Third line

Exclusion criteria variant classes: BRCA germline mutation (Breast Cancer, Ovarian Cancer), BRCA mutation (Ovarian Cancer)

Phase: II

Therapy: olaparib

Locations: Argentina, Australia, Canada, Colombia, Denmark, France, Guatemala, Ireland, Israel, Italy, Mexico, Peru, Republic of Korea, Romania, Russian Federation, Spain, Switzerland, Turkey, United Kingdom, United States

US States: AZ, CA, CO, GA, KY, MA, MD, MI, NE, NJ, NY, SD, UT, WA

Contact: Toll Free Number [888-577-8839; Trialsites@merck.com]

NCT03967938
Efficacy of Olaparib in Advanced Cancers Occurring in Patients With Germline Mutations or Somatic Tumor Mutations in Homologous Recombination Genes

Cancer type: Unspecified Solid Tumor

Variant class: CHEK1 mutation

Other identifiers: 1-2018 BSMO, 1-2018BSMO, EudraCT Number: 2018-002966-37, Precision 2 - Olaparib

Population segments: Line of therapy N/A, Stage III, Stage IV

Exclusion criteria variant classes: BRCA1 germline mutation (Breast Cancer, Pancreatic Cancer, Prostate Cancer), BRCA2 germline mutation (Breast Cancer, Pancreatic Cancer, Prostate Cancer)

Phase: II

Therapy: olaparib

Location: Belgium

NCT03842228
A Phase Ib Biomarker-Driven Combination Trial of Copanlisib, Olaparib, and Durvalumab (MEDI4736) in Patients With Advanced Solid Tumors

Cancer type: Unspecified Solid Tumor

Variant class: CHEK1 mutation

Other identifiers: 10217, 19-628, NCI10217, NCI-2019-00601

Population segments: Line of therapy N/A, Stage III, Stage IV

Phase: I

Therapies: copanlisib, olaparib, durvalumab

Location: United States

US States: MA, TX

Contact: Multiple contacts: See www.clinicaltrials.gov for complete list of contacts.

Disclaimer: The data presented here is from a curated knowledgebase of publicly available information, but may not be exhaustive. The data version is 2021.11(003).

CHEK1 truncating mutation (continued)

NCT04586335
Open Label, Phase Ib Study to Evaluate the Safety, Tolerability, Pharmacokinetics and Clinical Activity of CYH33, an Oral a-specific PI3K Inhibitor in Combination With Olaparib, an Oral PARP Inhibitor in Patients With Advanced Solid Tumors.

Cancer type: Breast Cancer, Endometrial Carcinoma, Ovarian Cancer, Prostate Cancer, Unspecified Solid Tumor

Variant class: CHEK1 mutation

Other identifiers: CTR20202339, CYH33-G102

Population segments: BRCA, Second line, Stage III, Stage IV

Phase: I

Therapies: HH-CYH33, olaparib

Locations: Australia, United States

US State: TX

Contact: Dr. Jason Sudia [908-380-1329; jason.sudia@haihepharma.com]

NCT02029001
A Two-period, Multicenter, Randomized, Open-label, Phase II Study Evaluating the Clinical Benefit of a Maintenance Treatment Targeting Tumor Molecular Alterations in Patients with Progressive Locally-advanced or Metastatic Solid Tumors.

Cancer type: Unspecified Solid Tumor

Variant class: HRR mutation

Other identifiers: ET12-081, EudraCT Number: 2012-004510-34, MOST, ProfiLER

Population segments: Maintenance/Consolidation, Second line, Stage III, Stage IV, Third line

Exclusion criteria variant classes: ATM mutation (Gastric Cancer), BRCA1 germline mutation (Gastric Cancer), BRCA1 mutation (Gastric Cancer), BRCA2 germline mutation (Gastric Cancer), BRCA2 mutation (Gastric Cancer), HRR mutation (Gastric Cancer), PALB2 mutation (Gastric Cancer), RAD51 mutation (Gastric Cancer)

Phase: II

Therapy: olaparib

Location: France

NCT04123366
A Phase II Study of Olaparib in Combination With Pembrolizumab in Participants With Previously Treated, Homologous Recombination Repair Mutation (HRRm) and/or Homologous Recombination Deficiency (HRD)-Positive Advanced Cancer

Cancer type: Unspecified Solid Tumor

Variant class: HRR mutation

Other identifiers: 7339-007, EudraCT Number: 2019-001745-40, jRCT2011200025, KEYLYNK-007, MK-7339-007, NCI-2019-08758, PER-029-19

Population segments: BRCA, Second line, Stage III, Stage IV

Exclusion criteria variant classes: BRCA germline mutation (Breast Cancer, Ovarian Cancer), BRCA mutation (Breast Cancer, Ovarian Cancer)

Phase: II

Therapies: olaparib, pembrolizumab

Locations: Argentina, Australia, Canada, Colombia, France, Germany, Guatemala, Israel, Italy, Japan, Latvia, Mexico, Peru, Poland, Puerto Rico, Republic of Korea, Romania, South Africa, Spain, Sweden, Turkey, Ukraine, United States

US States: AL, AZ, CA, CO, FL, GA, KY, NJ, NY, OH, TX, UT, VA

Contact: Toll Free Number [888-577-8839; Trialsites@merck.com]

Disclaimer: The data presented here is from a curated knowledgebase of publicly available information, but may not be exhaustive. The data version is 2021.11(003).

CHEK1 truncating mutation (continued)

NCT02401347

A Phase II Clinical Trial of the PARP Inhibitor Talazoparib in BRCA1 and BRCA2 Wild Type Patients With Advanced Triple Negative Breast Cancer and Homologous Recombination Deficiency or Advanced HER2 Negative Breast Cancer or Other Solid Tumors With a Mutation in Homologous Recombination Pathway Genes Talazoparib Beyond BRCA (TBB) Trial

Cancer type: Breast Cancer, Unspecified Solid Tumor

Variant class: HRR mutation

Other inclusion criteria: ERBB2 negative, Hormone receptor positive

Other identifiers: BRS0050, NCI-2015-00036, TBB

Population segments: Estrogen receptor positive, HER2 negative, Progesterone receptor positive, Second line, Stage III, Stage IV, Triple receptor negative

Exclusion criteria variant classes: BRCA1 germline mutation (Breast Cancer, Triple Negative Breast Cancer, Unspecified Solid Tumor), BRCA1 mutation (Breast Cancer, Triple Negative Breast Cancer, Unspecified Solid Tumor), BRCA2 germline mutation (Breast Cancer, Triple Negative Breast Cancer, Unspecified Solid Tumor), BRCA2 mutation (Breast Cancer, Triple Negative Breast Cancer, Unspecified Solid Tumor), ERBB2 positive (Breast Cancer)

Phase: II

Therapy: talazoparib

Location: United States

US State: CA

Contact: Amanda Selin [650-721-6977; aselin@stanford.edu]

NCT03415659

A Phase I, Open-label, Single-center, Single/Multiple-dose, Dose-escalation/ Dose-expansion Clinical Study on Tolerance and Pharmacokinetics of HWH340 Tablet in Patients With Advanced Solid Tumors

Cancer type: Unspecified Solid Tumor

Variant class: HRR mutation

Other inclusion criteria: BRCA1 germline mutation negative, BRCA1 mutation negative, BRCA2 germline mutation negative, BRCA2 mutation negative

Other identifiers: CTR20171649, HWH340-RFPA 20170821, RFPA 20170821

Population segments: BRCA, Fourth line or greater, HER2 negative, Second line, Stage III, Stage IV, Third line

Phase: I

Therapy: HWH-340

Location: China

NCT03767075

Basket of Baskets: A Modular, Open-label, Phase II, Multicentre Study To Evaluate Targeted Agents in Molecularly Selected Populations With Advanced Solid Tumours

Cancer type: Unspecified Solid Tumor

Variant class: DNA repair mutation

Other identifiers: (Basket of Baskets) (BoB), EudraCT number: 2017-005108-89, MO39164, NL65937.031.18, VHIO17002

Population segments: Second line, Stage III, Stage IV

Phase: II

Therapy: atezolizumab

Locations: France, Germany, Netherlands, Spain, Sweden, United Kingdom

---

Disclaimer: The data presented here is from a curated knowledgebase of publicly available information, but may not be exhaustive. The data version is 2021.11(003).

CHEK1 truncating mutation (continued)

NCT04905914
A Phase I/IIa, Open-Label, Safety, Pharmacokinetic, And Preliminary Efficacy Study Of Oral ATRN-119 In Patients With Advanced Solid Tumors

Cancer type: Unspecified Solid Tumor

Variant class: DNA repair mutation

Other identifier: AR-276-01

Population segments: Line of therapy N/A, Stage III, Stage IV

Phase: I/II

Therapy: ATRN-119

Location: United States

US State: MA

Contact: Robert Hasson [619-540-6253; rhasson@pacificlinkconsulting.com]

NCT04901702
A Randomized Phase I/II Study of Talazoparib or Temozolomide in Combination With Onivyde in Children With Recurrent Solid Malignancies and Ewing Sarcoma

Cancer type: Unspecified Solid Tumor

Variant class: HRR pathway

Other identifiers: EudraCT Number: 2021-003570-31, ONITT

Population segments: Second line, Stage IV

Exclusion criteria variant classes: HRR germline pathway (Central Nervous System Neoplasm, Ewing Sarcoma), HRR pathway (Central Nervous System Neoplasm, Ewing Sarcoma)

Phase: I/II

Therapies: talazoparib, chemotherapy

Locations: Canada, United States

US State: TN

Contact: Dr. Sara Federico [866-278-5833; referralinfo@stjude.org]

NCT03155620
NCI-COG Pediatric MATCH (Molecular Analysis for Therapy Choice) Screening Protocol

Cancer type: Unspecified Solid Tumor

Variant class: DNA repair pathway

Other identifiers: 17-729, 2017-0625, 20170841, APEC1621SC, COGAPEC1621, COGAPEC1621SC, N 55017 SC, NCI-2017-01251, NCI-COG Pediatric MATCH, Pediatric MATCH

Population segments: ALK, Aggressive, BRAF, FGFR, HRAS, Indolent, KRAS, NRAS, Pediatric or Adolescent, Second line, Stage III, Stage IV

Phase: II

Therapy: olaparib

Locations: Puerto Rico, United States

US States: AK, AL, AR, AZ, CA, CO, CT, DC, DE, FL, GA, HI, IA, ID, IL, IN, KY, LA, MA, MD, ME, MI, MN, MO, MS, NC, NE, NH, NJ, NV, NY, OH, OK, OR, PA, RI, SC, SD, TN, TX, UT, VA, VT, WA, WI

Contact: Multiple contacts: See www.clinicaltrials.gov for complete list of contacts.

Disclaimer: The data presented here is from a curated knowledgebase of publicly available information, but may not be exhaustive. The data version is 2021.11(003).

CHEK1 truncating mutation (continued)

NCT03233204
NCI-COG Pediatric MATCH (Molecular Analysis for Therapy Choice)- Phase 2 Subprotocol of Olaparib in Patients With Tumors Harboring Defects in DNA Damage Repair Genes

Cancer type: Unspecified Solid Tumor

Variant class: DNA repair pathway

Other identifiers: 20170841, APEC1621H, NCI-2017-00766

Population segments: Aggressive, Indolent, Pediatric or Adolescent, Second line, Stage III, Stage IV

Phase: II

Therapy: olaparib

Locations: Puerto Rico, United States

US States: AK, AL, AR, AZ, CA, CO, DC, DE, FL, GA, IA, ID, IL, IN, KY, LA, MD, ME, MI, MN, MO, MS, NC, ND, NE, NJ, NY, OH, OK, OR, PA, SC, SD, TN, TX, UT, VA, VT, WA, WI

Contact: Multiple contacts: See www.clinicaltrials.gov for complete list of contacts.

NCT04267939
An Open-label Phase Ib Study to Determine the Maximum Tolerated and/or Recommended Phase II Dose of the ATR Inhibitor BAY 1895344 in Combination With PARP Inhibitor Niraparib, in Patients With Recurrent Advanced Solid Tumors and Ovarian Cancer

Cancer type: Ovarian Cancer, Unspecified Solid Tumor

Variant class: DNA repair pathway

Other identifiers: 18595, 19-810, 2019-0471, 20-222, EudraCT Number: 2018-003930-34, NCI-2020-01405

Population segments: Maintenance/Consolidation, Second line, Stage III, Stage IV

Exclusion criteria variant class: DNA repair pathway (Prostate Cancer)

Phase: I

Therapies: BAY-1895344, niraparib

Location: United States

US States: MA, NY, TX

Contact: Bayer Clinical Trials Contact [888-842-2937; clinical-trials-contact@bayer.com]

NCT04693468
Modular Phase IB Hypothesis-Testing, Biomarker-Driven, Talazoparib Combination Trial (TalaCom)

Cancer type: Unspecified Solid Tumor

Variant class: DNA repair pathway

Other identifiers: 2020-0436, NCI-2020-06041, TalaCom

Population segments: Hormone refractory, Second line, Stage III, Stage IV

Phase: I

Therapies: talazoparib, palbociclib, axitinib, crizotinib

Location: United States

US State: TX

Contact: Timothy A. Yap [713-563-1784; tyap@mdanderson.org]

Disclaimer: The data presented here is from a curated knowledgebase of publicly available information, but may not be exhaustive. The data version is 2021.11(003).

CHEK1 truncating mutation (continued)

NCT04053322
An International Multicenter Phase II Trial of Durvalumab (MEDI4736) Plus OLAparib Plus Fulvestrant in Metastatic or Locally Advanced ER-positive, HER2-negative Breast Cancer Patients Selected Using Criteria That Predict Sensitivity to Olaparib

Cancer type: Breast Cancer

Variant class: CHEK1 mutation

Other inclusion criteria: ERBB2 negative, ER positive

Other identifiers: CADUSEIME08, DOLAF, EudraCT Number: 2018-003832-57, UC-0140/1812

Population segments: BRCA, Estrogen receptor positive, FGFR, HER2 negative, Progesterone receptor positive, Second line, Stage III, Stage IV

Phase: II

Therapies: durvalumab, olaparib, hormone therapy

Location: France

NCT03553004
Niraparib in Metastatic Pancreatic Cancer After Previous Chemotherapy (NIRA-PANC): A Phase II Trial

Cancer type: Pancreatic Cancer

Variant class: CHEK1 mutation

Other identifiers: IIT-2017-NIRA-PANC, NIRA-PANC, STUDY00141862

Population segments: BRCA, Second line, Stage IV, Third line

Phase: II

Therapy: niraparib

Location: United States

US States: KS, MO

Contact: Clinical Trials Nurse Navigator [913-588-3671; navigation@kumc.edu]

NCT04030559
Phase II Trial of PARP Inhibitor Niraparib for Men With High Risk Prostate Cancer and DNA Damage Response Defects

Cancer type: Prostate Cancer

Variant class: CHEK1 mutation

Other identifiers: 1412676, NCI-2019-04365, UCDCC#279

Population segments: First line, Neoadjuvant, Stage I, Stage II, Stage IV

Phase: II

Therapy: niraparib

Location: United States

US State: CA

Contact: Marc Dall'Era [916-734-3771; mdallera@ucdavis.edu]

Disclaimer: The data presented here is from a curated knowledgebase of publicly available information, but may not be exhaustive. The data version is 2021.11(003).

CHEK1 truncating mutation (continued)

NCT04187833
Phase II Trial of Nivolumab in Combination with Talazoparib in Patients with Unresectable or Metastatic Melanoma and Mutations in BRCA or BRCA-ness Genes.

Cancer type: Melanoma

Variant class: CHEK1 mutation

Other identifiers: CASE2619, NCI-2020-00416

Population segments: Second line, Stage III, Stage IV

Phase: II

Therapies: nivolumab, talazoparib

Location: United States

US State: OH

Contact: Dr. Pauline Funchain [866-223-8100; TaussigResearch@ccf.org]

NCT03375307
A Phase II Study of Olaparib (AZD2281) in Patients With Metastatic/Advanced Urothelial Carcinoma With DNA-Repair Defects

Cancer type: Bladder Urothelial Carcinoma

Variant class: CHEK1 mutation

Other identifiers: 10144, 19C0023, NCI-2017-02296, P162941

Population segments: BRCA, STK11, Second line, Stage III, Stage IV

Phase: II

Therapy: olaparib

Location: United States

US States: CA, CO, KY, MD, NY, OK

Contact: Multiple contacts: See www.clinicaltrials.gov for complete list of contacts.

NCT03786796
Phase II Study of Olaparib in Metastatic Renal Cell Carcinoma Patients Harboring a BAP-1 or Other DNA Repair Gene Mutations (ORCHID)

Cancer type: Kidney Cancer

Variant class: CHEK1 mutation

Other identifiers: CRMS-70750, J18166, NCI-2019-06309, ORCHID

Population segments: Fourth line or greater, Second line, Stage IV, Third line

Phase: II

Therapy: olaparib

Location: United States

US State: MD

Contact: Irina Rifkind [410-502-2043; irifkin1@jhmi.edu]

NCT04515836
Phase II Trial of Olaparib in Homologous Recombination Deficient (HRD) Malignant Mesothelioma

Cancer type: Mesothelioma

Variant class: CHEK1 mutation

Population segments: (N/A), Second line

Phase: II

Therapy: olaparib

Location: United States

US State: IL

Contact: Dr. Hedy L. Kindler [773-702-0360; hkindler@medicine.bsd.uchicago.edu]

Disclaimer: The data presented here is from a curated knowledgebase of publicly available information, but may not be exhaustive. The data version is 2021.11(003).

CHEK1 truncating mutation (continued)

NCT03533946
A Phase II Study of Rucaparib Monotherapy in Nonmetastatic, Hormone-Sensitive Prostate Cancer Demonstrating "BRCAness" Genotype (ROAR)

Cancer type: Prostate Cancer

Variant class: CHEK1 mutation

Other identifiers: HCI111833, NCI-2018-01754, ROAR

Population segments: First line, Stage I, Stage II, Stage III

Phase: II

Therapy: rucaparib

Location: United States

US State: UT

Contact: Jill Broghammer [801-213-6232; jill.broghammer@hci.utah.edu]

NCT04892693
Talazoparib in Advanced Breast Cancer Patients With Homologous Recombinant Deficiency: A Phase II Clinical and Exploratory Biomarker Study of Talazoparib

Cancer type: Breast Cancer

Variant class: CHEK1 mutation

Other identifier: H-2009-079-1157

Population segments: BRCA, Line of therapy N/A, Stage III, Stage IV

Phase: II

Therapy: talazoparib

Location: Republic of Korea

NCT04740190
Combination Talazoparib - Carboplatin for Recurrent High-grade Glioma With DNA Damage Repair Deficiency (DDRd)

Cancer type: Anaplastic Astrocytoma, Anaplastic Oligoastrocytoma, Anaplastic Oligodendroglioma, Anaplastic Pleomorphic Xanthoastrocytoma, Diffuse Midline Glioma, Glioblastoma

Variant class: CHEK1 mutation

Other identifiers: TAC-GReD, UW-20396

Population segments: (N/A), BRCA, Second line

Phase: II

Therapies: talazoparib, chemotherapy

Location: Hong Kong

NCT04253262
A Phase Ib/II Study of Copanlisib Combined With Rucaparib in Patients With Metastatic Castration-resistant Prostate Cancer

Cancer type: Castration-Resistant Prostate Cancer

Variant class: CHEK1 mutation

Other identifiers: BrUOG 360, BrUOG360

Population segments: Hormone refractory, Second line, Stage IV

Phase: I/II

Therapies: copanlisib, rucaparib

Location: United States

US State: RI

Contact: BrUOG [401-863-3000; BrUOG@brown.edu]

Disclaimer: The data presented here is from a curated knowledgebase of publicly available information, but may not be exhaustive. The data version is 2021.11(003).

CHEK1 truncating mutation (continued)

NCT03810105
A Phase II Study of Olaparib and Durvalumab in Men With Castration Sensitive Biochemically Recurrent Non-Metastatic Prostate Cancer Harboring Mutations in DNA Damage Repair

Cancer type: Prostate Cancer

Variant class: DNA repair truncating mutation

Other identifiers: 18-480, NCI-2019-01627

Population segments: Adjuvant, Stage I, Stage II, Stage III

Phase: II

Therapies: durvalumab, olaparib

Location: United States

US States: CA, IL, MI, NJ, NY

Contact: Dr. Karen Autio [646-422-4632; autiok@mskcc.org]

NCT04336943
Durvalumab (MEDI4736) and Olaparib (AZD2281) for Treatment of Biochemically Recurrent Prostate Cancer in Men Predicted to Have a High Neoantigen Load: A Pilot Study

Cancer type: Prostate Adenocarcinoma

Variant class: HRR mutation

Other identifiers: 10509, NCI-2020-01860, RG1007001

Population segments: Line of therapy N/A, Stage I, Stage II, Stage III

Phase: II

Therapies: durvalumab, olaparib

Location: United States

US State: WA

Contact: Michael Schweizer [206-606-6252; schweize@uw.edu]

NCT04169841
Precision Medicine Phase II Study Evaluating the Efficacy of a Double Immunotherapy by Durvalumab and Tremelimumab Combined With Olaparib in Patients With Solid Cancers and Carriers of Homologous Recombination Repair Genes Mutation in Response or Stable After Olaparib Treatment

Cancer type: Breast Cancer, Endometrial Carcinoma, Head and Neck Cancer, Kidney Cancer, Non-Small Cell Lung Cancer, Ovarian Cancer, Pancreatic Cancer, Prostate Cancer

Variant class: HRR mutation

Other identifier: GUIDE2REPAIR

Population segments: Fourth line or greater, Hormone refractory, Maintenance/Consolidation, Second line, Stage III, Stage IV, Third line

Exclusion criteria variant classes: ALK fusion (Lung Cancer), BRAF mutation (Lung Cancer), EGFR mutation (Lung Cancer), ROS1 mutation (Lung Cancer)

Phase: II

Therapies: durvalumab, tremelimumab, olaparib

Location: France

CHEK1 truncating mutation (continued)

NCT03925350
A Phase II Study of Niraparib in Patients With Advanced Melanoma With Genetic Homologous Recombination (HR) Mutation / Alteration

Cancer type: Melanoma

Variant class: HRR mutation

Other identifier: CPMC17-MEL01

Population segments: Second line, Stage III, Stage IV

Phase: II

Therapy: niraparib

Location: United States

US State: CA

Contact: Laurel Brechtel [415-600-1654; BrechtLA@sutterhealth.org]

NCT04940637
A Phase II, Open-Label, Single Arm, Prospective, Multicenter Study of Niraparib Plus Dostarlimab in Patients With Advanced NSCLC and/or MPM, and Positive for PD-L1 Expression and Germline or Somatic Mutations in the HRR Genes

Cancer type: Mesothelioma, Non-Small Cell Lung Cancer

Variant class: HRR mutation

Other inclusion criteria: ALK fusion negative, CD274 expression, EGFRi sensitizing mutation negative, ROS1 fusion negative or CD274 expression

Other identifiers: EudraCT Number: 2020-000109-10, UNITO-001, UNITO-001/2020

Population segments: Second line, Stage III, Stage IV

Exclusion criteria variant classes: ALK fusion (Non-Small Cell Lung Cancer), EGFRi sensitizing mutation (Non-Small Cell Lung Cancer), ROS1 fusion (Non-Small Cell Lung Cancer)

Phase: II

Therapies: niraparib, dostarlimab

Location: Italy

NCT04042831
A Phase II Study of Olaparib in Patients With Advanced Biliary Tract Cancer With Aberrant DNA Repair Gene Mutations

Cancer type: Biliary Tract Carcinoma

Variant class: HRR mutation

Other identifiers: ACCRU-ICRN-1702, NCI-2019-04434

Population segments: Second line, Stage IV

Phase: II

Therapy: olaparib

Location: United States

US States: AZ, MN, NY, TX

Contact: Multiple contacts: See www.clinicaltrials.gov for complete list of contacts.

Disclaimer: The data presented here is from a curated knowledgebase of publicly available information, but may not be exhaustive. The data version is 2021.11(003).

CHEK1 truncating mutation (continued)

NCT04939662
Phase II, Single-arm Study of Olaparib and Bevacizumab Combination Therapy in Relapsed Small Cell Lung Cancer Subjects With DNA Damage Response and the Repair Pathway Alteration, ATM Deficiency, SLFN11 Positive, or POU2F3 Positive

Cancer type: Small Cell Lung Cancer

Variant class: HRR mutation

Other identifier: SUKSES-B2

Population segments: (N/A), BRCA, Pulmonary, Second line, Third line

Phase: II

Therapies: olaparib, bevacizumab

Location: Republic of Korea

NCT03506997
PERSEUS1: Phase II Trial of the Immune Checkpoint Inhibitor Pembrolizumab for Patients Suffering From Metastatic Prostate Cancer

Cancer type: Castration-Resistant Prostate Cancer

Variant class: HRR mutation

Other identifiers: CCR4559, EudraCT Number: 2017-000931-15, ICR-CTSU/2016/10060, IRAS ID 208952, PERSEUS1

Population segments: Hormone refractory, Second line, Stage IV

Phase: II

Therapy: pembrolizumab

Location: United Kingdom

NCT02264678
A Modular Phase I, Open-Label, Multicentre Study to Assess the Safety, Tolerability, Pharmacokinetics and Preliminary Anti-tumour Activity of Ceralasertib in Combination With Cytotoxic Chemotherapy and/or DNA Damage Repair/Novel Anti-cancer Agents in Patients With Advanced Solid Malignancies

Cancer type: Triple Negative Breast Cancer

Variant class: HRR mutation

Other inclusion criteria: BRCA germline mutation negative, BRCA mutation negative

Other identifiers: 0C-14-9, 16-031, 18-342, D5330C00004, EudraCT Number: 2014-002233-66, IRAS ID 160200, NCI-2015-00687, Study 4

Population segments: Adenocarcinoma, BRCA, HER2 negative, Pulmonary, Second line, Stage III, Stage IV, Third line, Triple receptor negative

Exclusion criteria variant class: ERBB2 positive (Breast Cancer)

Phase: I

Therapies: ceralasertib, olaparib

Locations: France, Republic of Korea, United Kingdom, United States

US States: CA, MA, NY

Contact: AstraZeneca Clinical Study Information Center [877-240-9479; information.center@astrazeneca.com]

NCT04890613
Phase Ib Expansion Study of CX-5461 in Patients With Solid Tumours and BRCA2 and/or PALB2 Mutation

Cancer type: Ovarian Cancer

Variant class: HRR mutation

Other identifier: CX-5461-04

Population segments: BRCA, Second line, Stage II, Stage III, Stage IV

Phase: I

Therapy: pidnarulex

Location: Canada

Disclaimer: The data presented here is from a curated knowledgebase of publicly available information, but may not be exhaustive. The data version is 2021.11(003).

CHEK1 truncating mutation (continued)

NCT05002868

A Multi-center, Open-label, Phase I/Ib Study to Assess the Safety, Pharmacokinetics and Anti-tumor Activity of RP12146, a Poly (ADP-ribose) Polymerase (PARP) Inhibitor, in Patients With Locally Advanced or Metastatic Solid Tumors

Cancer type: Biliary Tract Carcinoma, Breast Cancer, Colorectal Cancer, Endometrial Carcinoma, Gastric Cancer, Ovarian Cancer, Pancreatic Cancer, Prostate Cancer, Small Cell Lung Cancer

Variant class: HRR mutation

Other identifier: RP12146-2101

Population segments: Extensive, Pulmonary, Second line, Stage III, Stage IV

Exclusion criteria variant class: ERBB2 positive (Breast Cancer)

Phase: I

Therapy: RP12146

Location: Poland

NCT03395197

A Phase III, Randomized, Double-blind, Placebo-controlled Study Of Talazoparib With Enzalutamide In Metastatic Castration-resistant Prostate Cancer

Cancer type: Castration-Resistant Prostate Cancer

Variant class: DNA repair mutation

Other identifiers: BASIC-ROYAL / HIGH-ROYAL, C3441021, CSET 2938, CTR20191443, CTR20191444, EudraCT Number: 2017-003295-31, GU 170 - C3441021, HCC 19-054, JapicCTI-194665, LH19.022, MOH_2019-05-16_007127, NCI-2018-01651, Pfizer C3441021, R19062M, RECF3951, RWF_C3441021 TALAPRO-2, TALAPRO-2, TX228665, UCI-18-47, VICCURO17115

Population segments: First line, Hormone refractory, Second line, Stage III, Stage IV

Phase: III

Therapies: talazoparib, hormone therapy

Locations: Argentina, Australia, Belgium, Brazil, Canada, Chile, China, Czech Republic, Finland, France, Germany, Hungary, Israel, Italy, Japan, New Zealand, Norway, Peru, Poland, Portugal, Republic of Korea, South Africa, Spain, Sweden, United Kingdom, United States

US States: AZ, CA, CO, FL, GA, IL, IN, KS, MO, NE, NJ, NY, OH, OK, OR, PA, SC, TN, TX, UT, VA, WI

Contact: Pfizer CT.gov Call Center [800-718-1021; ClinicalTrials.gov_Inquiries@pfizer.com]

NCT04821622

TALAPRO-3: A Phase III, Randomized, Double-blind, Study of Talazoparib With Enzalutamide Versus Placebo With Enzalutamide in Men With DDR Gene Mutated Metastatic Castration-sensitive Prostate Cancer

Cancer type: Prostate Adenocarcinoma

Variant class: DNA repair mutation

Other identifiers: C3441052, CTR20212191, EudraCT Number: 2021-000248-23, jRCT2031210098, TALAPRO-3

Population segments: Second line, Stage IV

Phase: III

Therapies: talazoparib, hormone therapy

Locations: Japan, Republic of Korea, United States

US States: CA, DC, FL, GA, IL, MI, NJ, NY, PA, SC, TX, UT

Contact: Pfizer CT.gov Call Center [800-718-1021; ClinicalTrials.gov_Inquiries@pfizer.com]

Disclaimer: The data presented here is from a curated knowledgebase of publicly available information, but may not be exhaustive. The data version is 2021.11(003).

CHEK1 truncating mutation (continued)

NCT03012321

BRCAAway: A Randomized Phase II Trial of Abiraterone, Olaparib, or Abiraterone + Olaparib in Patients With Metastatic Castration-Resistant Prostate Cancer With DNA Repair Defects

Cancer type: Castration-Resistant Prostate Cancer

Variant class: DNA repair mutation

Other identifiers: 19137, BRCAAway, NCI-2016-01834, NU 16U05, NU_16U05, PCCTC #: c16-168, PCCTC c16-168, STU00203960

Population segments: First line, Hormone refractory, Stage IV

Phase: II

Therapy: olaparib

Location: United States

US States: CA, FL, IL, IN, MD, MI, MN, MO, NC, NY, UT, VA

Contact: Study Coordinator [312-695-1301; cancertrials@northwestern.edu]

NCT03344965

A Phase II Study of Olaparib Monotherapy in Metastatic Breast Cancer Patients With Germline or Somatic Mutations in DNA Repair Genes (Olaparib Expanded)

Cancer type: Breast Cancer

Variant class: DNA repair mutation

Other inclusion criteria: BRCA1 germline mutation negative, BRCA2 germline mutation negative

Other identifiers: 17-428, 18-414, NCI-2018-00778, TBCRC 048, TBCRC048

Population segments: BRCA, Estrogen receptor positive, HER2 negative, HER2 positive, Progesterone receptor positive, Second line, Stage IV

Exclusion criteria variant classes: BRCA1 germline mutation (Breast Cancer), BRCA2 germline mutation (Breast Cancer)

Phase: II

Therapy: olaparib

Location: United States

US States: AL, CA, IL, MA, MD, NC, NJ, NY, PA, WA

Contact: Dr. Nadine Tung [617-667-1962; ntung@bidmc.harvard.edu]

NCT04038502

An Open-label, Multicenter Phase II Study to Compare the Efficacy of Carboplatin as First-line Followed by Second-line Olaparib Versus Olaparib as First-line Followed by Second-line Carboplatin in the Treatment of Patients With Castration Resistant Prostate Cancer Containing Homologous Recombination Deficiency

Cancer type: Castration-Resistant Prostate Cancer

Variant class: DNA repair mutation

Other identifiers: 01781, 01783, 19-08, BRACeD, CX001963-01, ONCA-009-18F

Population segments: First line, Hormone refractory, Second line, Stage IV

Phase: II

Therapies: olaparib, chemotherapy

Location: United States

US States: CA, DC, FL, IL, MI, NC, NY, PA, WA

Contact: Dr. Robert B. Montgomery [206-277-6878; rbmontgo@u.washington.edu]

Disclaimer: The data presented here is from a curated knowledgebase of publicly available information, but may not be exhaustive. The data version is 2021.11(003).

CHEK1 truncating mutation (continued)

NCT04633902
Phase II Study of Olaparib in Combination With Pembrolizumab in Patients With Advanced Melanoma With Homologous Recombination (HR) Pathway Gene Mutation

Cancer type: Melanoma

Variant class: HRR pathway

Other identifier: CPMC19-MEL01

Population segments: BRAF, Second line, Stage III, Stage IV

Phase: II

Therapies: olaparib, pembrolizumab

Location: United States

US State: CA

Contact: Dr. Kevin B. Kim [415-885-8600; ClinicalResearch@sutterhealth.org]

NCT03248570
Phase II Open Label Study of Pembrolizumab in Patients With Metastatic Castration Resistant Prostate Cancer (mCRPC) With or Without DNA Damage Repair Defects

Cancer type: Castration-Resistant Prostate Cancer

Variant class: DNA repair mutation

Other identifiers: 16557, 17-22165, CC 16557, NCI-2017-02408

Population segments: BRCA, Hormone refractory, Line of therapy N/A, Stage IV

Phase: II

Therapy: pembrolizumab

Location: United States

US State: CA

Contact: Patricia Li [415-476-5975; GUTrials@ucsf.edu]

No NCT ID - see other identifier(s)
Single-arm Exploratory Study for Sintilimab Second-line or Later-line Treatment of Advanced Non-small Cell Lung Cancer with DDR Pathway Gene Mutation

Cancer type: Non-Small Cell Lung Cancer

Variant class: DNA repair mutation

Other identifier: ChiCTR1900023234

Population segments: Fourth line or greater, Second line, Stage III, Stage IV, Third line

Exclusion criteria variant classes: ALK fusion (Non-Small Cell Lung Cancer), EGFRi sensitizing mutation (Non-Small Cell Lung Cancer), ROS1 fusion (Non-Small Cell Lung Cancer)

Phase: I/II

Therapy: sintilimab

Location: China

Disclaimer: The data presented here is from a curated knowledgebase of publicly available information, but may not be exhaustive. The data version is 2021.11(003).

CHEK1 truncating mutation (continued)

NCT04095273

A Multicenter, Non-randomized, Open-label Phase Ib Study to Determine the Maximum Tolerated and Recommended Phase II Dose of the ATR Inhibitor BAY1895344 in Combination With Pembrolizumab and to Characterize Its Safety, Tolerability, Pharmacokinetics and Preliminary Anti-tumor Activity in Patients With Advanced Solid Tumors

Cancer type: Breast Cancer, Castration-Resistant Prostate Cancer, Colorectal Cancer, Gastric Cancer, Gastroesophageal Junction Adenocarcinoma, Non-Small Cell Lung Cancer, Pancreatic Cancer

Variant class: DNA repair mutation

Other inclusion criteria: ATM mutation negative or ATM mutation negative, ERBB2 negative, Hormone receptor positive or ATM underexpression

Other identifiers: 19741, EudraCT Number: 2018-003420-36, Keynote 919, NCI-2019-06734

Population segments: HER2 negative, Hormone refractory, Second line, Stage III, Stage IV

Phase: I

Therapies: BAY-1895344, pembrolizumab

Locations: Germany, Spain, Switzerland, United States

US States: CA, CT, MA, MD, NC, NY, OH, TX

Contact: Bayer Clinical Trials Contact [clinical-trials-contact@bayer.com]

NCT04849364

A Phase II Circulating Tumor DNA Enriched, Genomically Directed Post-neoadjuvant Trial for Patients With Residual Triple Negative Breast Cancer (PERSEVERE)

Cancer type: Triple Negative Breast Cancer

Variant class: DNA repair pathway

Other identifiers: HCRN BRE18-334, PERSEVERE

Population segments: Adjuvant, HER2 negative, Stage I, Stage II, Stage III, Triple receptor negative

Phase: II

Therapies: talazoparib, chemotherapy

Location: United States

US State: IN

Contact: Dr. Bryan P. Schneider [317-948-3885; bpschnei@iu.edu]

CHEK1 deletion

NCT04901702
A Randomized Phase I/II Study of Talazoparib or Temozolomide in Combination With Onivyde in Children With Recurrent Solid Malignancies and Ewing Sarcoma

Cancer type: Unspecified Solid Tumor

Variant class: HRR pathway

Other identifiers: EudraCT Number: 2021-003570-31, ONITT

Population segments: Second line, Stage IV

Exclusion criteria variant classes: HRR germline pathway (Central Nervous System Neoplasm, Ewing Sarcoma), HRR pathway (Central Nervous System Neoplasm, Ewing Sarcoma)

Phase: I/II

Therapies: talazoparib, chemotherapy

Locations: Canada, United States

US State: TN

Contact: Dr. Sara Federico [866-278-5833; referralinfo@stjude.org]

NCT03155620
NCI-COG Pediatric MATCH (Molecular Analysis for Therapy Choice) Screening Protocol

Cancer type: Unspecified Solid Tumor

Variant class: DNA repair pathway

Other identifiers: 17-729, 2017-0625, 20170841, APEC1621SC, COGAPEC1621, COGAPEC1621SC, N 55017 SC, NCI-2017-01251, NCI-COG Pediatric MATCH, Pediatric MATCH

Population segments: ALK, Aggressive, BRAF, FGFR, HRAS, Indolent, KRAS, NRAS, Pediatric or Adolescent, Second line, Stage III, Stage IV

Phase: II

Therapy: olaparib

Locations: Puerto Rico, United States

US States: AK, AL, AR, AZ, CA, CO, CT, DC, DE, FL, GA, HI, IA, ID, IL, IN, KY, LA, MA, MD, ME, MI, MN, MO, MS, NC, NE, NH, NJ, NV, NY, OH, OK, OR, PA, RI, SC, SD, TN, TX, UT, VA, VT, WA, WI

Contact: Multiple contacts: See www.clinicaltrials.gov for complete list of contacts.

NCT03233204
NCI-COG Pediatric MATCH (Molecular Analysis for Therapy Choice)- Phase 2 Subprotocol of Olaparib in Patients With Tumors Harboring Defects in DNA Damage Repair Genes

Cancer type: Unspecified Solid Tumor

Variant class: DNA repair pathway

Other identifiers: 20170841, APEC1621H, NCI-2017-00766

Population segments: Aggressive, Indolent, Pediatric or Adolescent, Second line, Stage III, Stage IV

Phase: II

Therapy: olaparib

Locations: Puerto Rico, United States

US States: AK, AL, AR, AZ, CA, CO, DC, DE, FL, GA, IA, ID, IL, IN, KY, LA, MD, ME, MI, MN, MO, MS, NC, ND, NE, NJ, NY, OH, OK, OR, PA, SC, SD, TN, TX, UT, VA, VT, WA, WI

Contact: Multiple contacts: See www.clinicaltrials.gov for complete list of contacts.

CHEK1 deletion (continued)

NCT04267939
An Open-label Phase Ib Study to Determine the Maximum Tolerated and/or Recommended Phase II Dose of the ATR Inhibitor BAY 1895344 in Combination With PARP Inhibitor Niraparib, in Patients With Recurrent Advanced Solid Tumors and Ovarian Cancer

Cancer type: Ovarian Cancer, Unspecified Solid Tumor

Variant class: DNA repair pathway

Other identifiers: 18595, 19-810, 2019-0471, 20-222, EudraCT Number: 2018-003930-34, NCI-2020-01405

Population segments: Maintenance/Consolidation, Second line, Stage III, Stage IV

Exclusion criteria variant class: DNA repair pathway (Prostate Cancer)

Phase: I

Therapies: BAY-1895344, niraparib

Location: United States

US States: MA, NY, TX

Contact: Bayer Clinical Trials Contact [888-842-2937; clinical-trials-contact@bayer.com]

NCT04693468
Modular Phase IB Hypothesis-Testing, Biomarker-Driven, Talazoparib Combination Trial (TalaCom)

Cancer type: Unspecified Solid Tumor

Variant class: DNA repair pathway

Other identifiers: 2020-0436, NCI-2020-06041, TalaCom

Population segments: Hormone refractory, Second line, Stage III, Stage IV

Phase: I

Therapies: talazoparib, palbociclib, axitinib, crizotinib

Location: United States

US State: TX

Contact: Timothy A. Yap [713-563-1784; tyap@mdanderson.org]

NCT04030559
Phase II Trial of PARP Inhibitor Niraparib for Men With High Risk Prostate Cancer and DNA Damage Response Defects

Cancer type: Prostate Cancer

Variant class: CHEK1 deletion

Other identifiers: 1412676, NCI-2019-04365, UCDCC#279

Population segments: First line, Neoadjuvant, Stage I, Stage II, Stage IV

Phase: II

Therapy: niraparib

Location: United States

US State: CA

Contact: Marc Dall'Era [916-734-3771; mdallera@ucdavis.edu]

Disclaimer: The data presented here is from a curated knowledgebase of publicly available information, but may not be exhaustive. The data version is 2021.11(003).

CHEK1 deletion (continued)

NCT04740190

Combination Talazoparib - Carboplatin for Recurrent High-grade Glioma With DNA Damage Repair Deficiency (DDRd)

Cancer type: Anaplastic Astrocytoma, Anaplastic Oligoastrocytoma, Anaplastic Oligodendroglioma, Anaplastic Pleomorphic Xanthoastrocytoma, Diffuse Midline Glioma, Glioblastoma

Variant class: CHEK1 deletion

Other identifiers: TAC-GReD, UW-20396

Population segments: (N/A), BRCA, Second line

Phase: II

Therapies: talazoparib, chemotherapy

Location: Hong Kong

NCT03810105

A Phase II Study of Olaparib and Durvalumab in Men With Castration Sensitive Biochemically Recurrent Non-Metastatic Prostate Cancer Harboring Mutations in DNA Damage Repair

Cancer type: Prostate Cancer

Variant class: DNA repair deletion

Other identifiers: 18-480, NCI-2019-01627

Population segments: Adjuvant, Stage I, Stage II, Stage III

Phase: II

Therapies: durvalumab, olaparib

Location: United States

US States: CA, IL, MI, NJ, NY

Contact: Dr. Karen Autio [646-422-4632; autiok@mskcc.org]

NCT03344965

A Phase II Study of Olaparib Monotherapy in Metastatic Breast Cancer Patients With Germline or Somatic Mutations in DNA Repair Genes (Olaparib Expanded)

Cancer type: Breast Cancer

Variant class: DNA repair deletion

Other inclusion criteria: BRCA1 germline mutation negative, BRCA2 germline mutation negative

Other identifiers: 17-428, 18-414, NCI-2018-00778, TBCRC 048, TBCRC048

Population segments: BRCA, Estrogen receptor positive, HER2 negative, HER2 positive, Progesterone receptor positive, Second line, Stage IV

Exclusion criteria variant classes: BRCA1 germline mutation (Breast Cancer), BRCA2 germline mutation (Breast Cancer)

Phase: II

Therapy: olaparib

Location: United States

US States: AL, CA, IL, MA, MD, NC, NJ, NY, PA, WA

Contact: Dr. Nadine Tung [617-667-1962; ntung@bidmc.harvard.edu]

CHEK1 deletion (continued)

NCT04633902
Phase II Study of Olaparib in Combination With Pembrolizumab in Patients With Advanced Melanoma With Homologous Recombination (HR) Pathway Gene Mutation

Cancer type: Melanoma

Variant class: HRR pathway

Other identifier: CPMC19-MEL01

Population segments: BRAF, Second line, Stage III, Stage IV

Phase: II

Therapies: olaparib, pembrolizumab

Location: United States

US State: CA

Contact: Dr. Kevin B. Kim [415-885-8600; ClinicalResearch@sutterhealth.org]

NCT03248570
Phase II Open Label Study of Pembrolizumab in Patients With Metastatic Castration Resistant Prostate Cancer (mCRPC) With or Without DNA Damage Repair Defects

Cancer type: Castration-Resistant Prostate Cancer

Variant class: DNA repair deletion

Other identifiers: 16557, 17-22165, CC 16557, NCI-2017-02408

Population segments: BRCA, Hormone refractory, Line of therapy N/A, Stage IV

Phase: II

Therapy: pembrolizumab

Location: United States

US State: CA

Contact: Patricia Li [415-476-5975; GUTrials@ucsf.edu]

NCT03506997
PERSEUS1: Phase II Trial of the Immune Checkpoint Inhibitor Pembrolizumab for Patients Suffering From Metastatic Prostate Cancer

Cancer type: Castration-Resistant Prostate Cancer

Variant class: DNA repair pathway

Other inclusion criteria: MMR deficient

Other identifiers: CCR4559, EudraCT Number: 2017-000931-15, ICR-CTSU/2016/10060, IRAS ID 208952, PERSEUS1

Population segments: Hormone refractory, Second line, Stage IV

Phase: II

Therapy: pembrolizumab

Location: United Kingdom

NCT04849364
A Phase II Circulating Tumor DNA Enriched, Genomically Directed Post-neoadjuvant Trial for Patients With Residual Triple Negative Breast Cancer (PERSEVERE)

Cancer type: Triple Negative Breast Cancer

Variant class: DNA repair pathway

Other identifiers: HCRN BRE18-334, PERSEVERE

Population segments: Adjuvant, HER2 negative, Stage I, Stage II, Stage III, Triple receptor negative

Phase: II

Therapies: talazoparib, chemotherapy

Location: United States

US State: IN

Contact: Dr. Bryan P. Schneider [317-948-3885; bpschnei@iu.edu]

Disclaimer: The data presented here is from a curated knowledgebase of publicly available information, but may not be exhaustive. The data version is 2021.11(003).

CHEK1 deletion (continued)

NCT05002868
A Multi-center, Open-label, Phase I/Ib Study to Assess the Safety, Pharmacokinetics and Anti-tumor Activity of RP12146, a Poly (ADP-ribose) Polymerase (PARP) Inhibitor, in Patients With Locally Advanced or Metastatic Solid Tumors

Cancer type: Biliary Tract Carcinoma, Breast Cancer, Colorectal Cancer, Endometrial Carcinoma, Gastric Cancer, Ovarian Cancer, Pancreatic Cancer, Prostate Cancer, Small Cell Lung Cancer

Variant class: DNA repair pathway

Other identifier: RP12146-2101

Population segments: Extensive, Pulmonary, Second line, Stage III, Stage IV

Exclusion criteria variant class: ERBB2 positive (Breast Cancer)

Phase: I

Therapy: RP12146

Location: Poland

Relevant Biomarkers

Genomic Alteration	Relevant Therapies (In this cancer type)	Relevant Therapies (In other cancer type)	Clinical Trials
CHEK1 mutation<br>**Prognostic significance:** None<br>**Diagnostic significance:** None	None	**olaparib** [1]	49
CHEK1 truncating mutation<br>**Prognostic significance:** None<br>**Diagnostic significance:** None	None	**olaparib** [1]	49
CHEK1 deletion<br>**Prognostic significance:** None<br>**Diagnostic significance:** None	None	None	15

Public data sources included in relevant therapies: FDA1, NCCN, **EMA**2, ESMO
Public data sources included in prognostic and diagnostic significance: NCCN, ESMO

Relevant Therapy Summary

● In this cancer type ○ In other cancer type ◐ In this cancer type and other cancer types ✖ No evidence

CHEK1 mutation

Relevant Therapy	FDA	NCCN	EMA	ESMO	Clinical Trials*
olaparib	○	○	✖	✖	◐ (II)
olaparib, pembrolizumab	✖	✖	✖	✖	◐ (II)
talazoparib	✖	✖	✖	✖	◐ (II)
talazoparib, chemotherapy	✖	✖	✖	✖	◐ (II)
atezolizumab	✖	✖	✖	✖	● (II)
ATRN-119	✖	✖	✖	✖	● (I/II)

* Most advanced phase (IV, III, II/III, II, I/II, I) is shown and multiple clinical trials may be available.

Disclaimer: The data presented here is from a curated knowledgebase of publicly available information, but may not be exhaustive. The data version is 2021.11(003).

Relevant Therapy Summary (continued)

● In this cancer type ○ In other cancer type ◐ In this cancer type and other cancer types ✖ No evidence

CHEK1 mutation (continued)

Relevant Therapy	FDA	NCCN	EMA	ESMO	Clinical Trials*
BAY-1895344, niraparib	✖	✖	✖	✖	● (I)
copanlisib, olaparib, durvalumab	✖	✖	✖	✖	● (I)
HH-CYH33, olaparib	✖	✖	✖	✖	● (I)
HWH-340	✖	✖	✖	✖	● (I)
talazoparib, palbociclib, axitinib, crizotinib	✖	✖	✖	✖	● (I)
talazoparib, hormone therapy	✖	✖	✖	✖	○ (III)
durvalumab, olaparib	✖	✖	✖	✖	○ (II)
durvalumab, olaparib, hormone therapy	✖	✖	✖	✖	○ (II)
durvalumab, tremelimumab, olaparib	✖	✖	✖	✖	○ (II)
niraparib	✖	✖	✖	✖	○ (II)
niraparib, dostarlimab	✖	✖	✖	✖	○ (II)
nivolumab, chemotherapy, hormone therapy	✖	✖	✖	✖	○ (II)
nivolumab, talazoparib	✖	✖	✖	✖	○ (II)
olaparib, bevacizumab	✖	✖	✖	✖	○ (II)
olaparib, chemotherapy	✖	✖	✖	✖	○ (II)
pembrolizumab	✖	✖	✖	✖	○ (II)
rucaparib	✖	✖	✖	✖	○ (II)
copanlisib, rucaparib	✖	✖	✖	✖	○ (I/II)
sintilimab	✖	✖	✖	✖	○ (I/II)
BAY-1895344, pembrolizumab	✖	✖	✖	✖	○ (I)
ceralasertib, olaparib	✖	✖	✖	✖	○ (I)
pidnarulex	✖	✖	✖	✖	○ (I)
RP12146	✖	✖	✖	✖	○ (I)

CHEK1 truncating mutation

Relevant Therapy	FDA	NCCN	EMA	ESMO	Clinical Trials*
olaparib	○	○	✖	✖	◐ (II)
olaparib, pembrolizumab	✖	✖	✖	✖	◐ (II)
talazoparib	✖	✖	✖	✖	◐ (II)

* Most advanced phase (IV, III, II/III, II, I/II, I) is shown and multiple clinical trials may be available.

Disclaimer: The data presented here is from a curated knowledgebase of publicly available information, but may not be exhaustive. The data version is 2021.11(003).

Relevant Therapy Summary (continued)

● In this cancer type ○ In other cancer type ◐ In this cancer type and other cancer types ✖ No evidence

CHEK1 truncating mutation (continued)

Relevant Therapy	FDA	NCCN	EMA	ESMO	Clinical Trials*
talazoparib, chemotherapy	✖	✖	✖	✖	◐ (II)
atezolizumab	✖	✖	✖	✖	● (II)
ATRN-119	✖	✖	✖	✖	● (I/II)
BAY-1895344, niraparib	✖	✖	✖	✖	● (I)
copanlisib, olaparib, durvalumab	✖	✖	✖	✖	● (I)
HH-CYH33, olaparib	✖	✖	✖	✖	● (I)
HWH-340	✖	✖	✖	✖	● (I)
talazoparib, palbociclib, axitinib, crizotinib	✖	✖	✖	✖	● (I)
talazoparib, hormone therapy	✖	✖	✖	✖	○ (III)
durvalumab, olaparib	✖	✖	✖	✖	○ (II)
durvalumab, olaparib, hormone therapy	✖	✖	✖	✖	○ (II)
durvalumab, tremelimumab, olaparib	✖	✖	✖	✖	○ (II)
niraparib	✖	✖	✖	✖	○ (II)
niraparib, dostarlimab	✖	✖	✖	✖	○ (II)
nivolumab, chemotherapy, hormone therapy	✖	✖	✖	✖	○ (II)
nivolumab, talazoparib	✖	✖	✖	✖	○ (II)
olaparib, bevacizumab	✖	✖	✖	✖	○ (II)
olaparib, chemotherapy	✖	✖	✖	✖	○ (II)
pembrolizumab	✖	✖	✖	✖	○ (II)
rucaparib	✖	✖	✖	✖	○ (II)
copanlisib, rucaparib	✖	✖	✖	✖	○ (I/II)
sintilimab	✖	✖	✖	✖	○ (I/II)
BAY-1895344, pembrolizumab	✖	✖	✖	✖	○ (I)
ceralasertib, olaparib	✖	✖	✖	✖	○ (I)
pidnarulex	✖	✖	✖	✖	○ (I)
RP12146	✖	✖	✖	✖	○ (I)

* Most advanced phase (IV, III, II/III, II, I/II, I) is shown and multiple clinical trials may be available.

Disclaimer: The data presented here is from a curated knowledgebase of publicly available information, but may not be exhaustive. The data version is 2021.11(003).

Relevant Therapy Summary (continued)

● In this cancer type ○ In other cancer type ◐ In this cancer type and other cancer types ✖ No evidence

CHEK1 deletion

Relevant Therapy	FDA	NCCN	EMA	ESMO	Clinical Trials*
olaparib	✖	✖	✖	✖	◐ (II)
talazoparib, chemotherapy	✖	✖	✖	✖	◐ (II)
BAY-1895344, niraparib	✖	✖	✖	✖	● (I)
talazoparib, palbociclib, axitinib, crizotinib	✖	✖	✖	✖	● (I)
durvalumab, olaparib	✖	✖	✖	✖	○ (II)
niraparib	✖	✖	✖	✖	○ (II)
nivolumab, chemotherapy, hormone therapy	✖	✖	✖	✖	○ (II)
olaparib, pembrolizumab	✖	✖	✖	✖	○ (II)
pembrolizumab	✖	✖	✖	✖	○ (II)
RP12146	✖	✖	✖	✖	○ (I)

* Most advanced phase (IV, III, II/III, II, I/II, I) is shown and multiple clinical trials may be available.

Relevant Biomarkers

Genomic Alteration	Relevant Therapies (In this cancer type)	Relevant Therapies (In other cancer type)	Clinical Trials
CHEK1 mutation<br>**Prognostic significance:** None<br>**Diagnostic significance:** None	None	**olaparib** [1]	49
CHEK1 truncating mutation<br>**Prognostic significance:** None<br>**Diagnostic significance:** None	None	**olaparib** [1]	49
CHEK1 deletion<br>**Prognostic significance:** None<br>**Diagnostic significance:** None	None	None	15

Public data sources included in relevant therapies: FDA[1], NCCN, **EMA**[2], ESMO
Public data sources included in prognostic and diagnostic significance: NCCN, ESMO

Clinical Trials Summary

CHEK1 mutation + CHEK1 truncating mutation + CHEK1 deletion

NCT ID	Title	Phase
NCT04126070	A Phase II Multicohort Study of Nivolumab in Combination With Docetaxel and Androgen Deprivation Therapy in Metastatic Hormone Sensitive Prostate Cancer Patients With DNA Damage Repair Defects or Inflamed Tumors	II

Disclaimer: The data presented here is from a curated knowledgebase of publicly available information, but may not be exhaustive. The data version is 2021.11(003).

Clinical Trials Summary (continued)

CHEK1 mutation

NCT ID	Title	Phase
NCT03742895	A Phase II Study of Olaparib Monotherapy in Participants With Previously Treated, Homologous Recombination Repair Mutation (HRRm) or Homologous Recombination Deficiency (HRD) Positive Advanced Cancer	II
NCT03967938	Efficacy of Olaparib in Advanced Cancers Occurring in Patients With Germline Mutations or Somatic Tumor Mutations in Homologous Recombination Genes	II
NCT04550494	A Pharmacodynamics-Driven Trial of Talazoparib, an Oral PARP Inhibitor, in Patients With Advanced Solid Tumors and Aberrations in Genes Involved in DNA Damage Response	II
NCT03842228	A Phase Ib Biomarker-Driven Combination Trial of Copanlisib, Olaparib, and Durvalumab (MEDI4736) in Patients With Advanced Solid Tumors	I
NCT04586335	Open Label, Phase Ib Study to Evaluate the Safety, Tolerability, Pharmacokinetics and Clinical Activity of CYH33, an Oral a-specific PI3K Inhibitor in Combination With Olaparib, an Oral PARP Inhibitor in Patients With Advanced Solid Tumors.	I
NCT02029001	A Two-period, Multicenter, Randomized, Open-label, Phase II Study Evaluating the Clinical Benefit of a Maintenance Treatment Targeting Tumor Molecular Alterations in Patients with Progressive Locally-advanced or Metastatic Solid Tumors.	II
NCT04123366	A Phase II Study of Olaparib in Combination With Pembrolizumab in Participants With Previously Treated, Homologous Recombination Repair Mutation (HRRm) and/or Homologous Recombination Deficiency (HRD)-Positive Advanced Cancer	II
NCT02401347	A Phase II Clinical Trial of the PARP Inhibitor Talazoparib in BRCA1 and BRCA2 Wild Type Patients With Advanced Triple Negative Breast Cancer and Homologous Recombination Deficiency or Advanced HER2 Negative Breast Cancer or Other Solid Tumors With a Mutation in Homologous Recombination Pathway Genes Talazoparib Beyond BRCA (TBB) Trial	II
NCT03415659	A Phase I, Open-label, Single-center, Single/Multiple-dose, Dose-escalation/Dose-expansion Clinical Study on Tolerance and Pharmacokinetics of HWH340 Tablet in Patients With Advanced Solid Tumors	I
NCT03767075	Basket of Baskets: A Modular, Open-label, Phase II, Multicentre Study To Evaluate Targeted Agents in Molecularly Selected Populations With Advanced Solid Tumours	II
NCT04905914	A Phase I/IIa, Open-Label, Safety, Pharmacokinetic, And Preliminary Efficacy Study Of Oral ATRN-119 In Patients With Advanced Solid Tumors	I/II
NCT04901702	A Randomized Phase I/II Study of Talazoparib or Temozolomide in Combination With Onivyde in Children With Recurrent Solid Malignancies and Ewing Sarcoma	I/II
NCT03155620	NCI-COG Pediatric MATCH (Molecular Analysis for Therapy Choice) Screening Protocol	II
NCT03233204	NCI-COG Pediatric MATCH (Molecular Analysis for Therapy Choice)- Phase 2 Subprotocol of Olaparib in Patients With Tumors Harboring Defects in DNA Damage Repair Genes	II
NCT04267939	An Open-label Phase Ib Study to Determine the Maximum Tolerated and/or Recommended Phase II Dose of the ATR Inhibitor BAY 1895344 in Combination With PARP Inhibitor Niraparib, in Patients With Recurrent Advanced Solid Tumors and Ovarian Cancer	I
NCT04693468	Modular Phase IB Hypothesis-Testing, Biomarker-Driven, Talazoparib Combination Trial (TalaCom)	I
NCT04053322	An International Multicenter Phase II Trial of Durvalumab (MEDI4736) Plus OLAparib Plus Fulvestrant in Metastatic or Locally Advanced ER-positive, HER2-negative Breast Cancer Patients Selected Using Criteria That Predict Sensitivity to Olaparib	II
NCT03553004	Niraparib in Metastatic Pancreatic Cancer After Previous Chemotherapy (NIRA-PANC): A Phase II Trial	II

Clinical Trials Summary (continued)

CHEK1 mutation (continued)

NCT ID	Title	Phase
NCT04030559	Phase II Trial of PARP Inhibitor Niraparib for Men With High Risk Prostate Cancer and DNA Damage Response Defects	II
NCT04187833	Phase II Trial of Nivolumab in Combination with Talazoparib in Patients with Unresectable or Metastatic Melanoma and Mutations in BRCA or BRCA-ness Genes.	II
NCT03375307	A Phase II Study of Olaparib (AZD2281) in Patients With Metastatic/Advanced Urothelial Carcinoma With DNA-Repair Defects	II
NCT03786796	Phase II Study of Olaparib in Metastatic Renal Cell Carcinoma Patients Harboring a BAP-1 or Other DNA Repair Gene Mutations (ORCHID)	II
NCT04515836	Phase II Trial of Olaparib in Homologous Recombination Deficient (HRD) Malignant Mesothelioma	II
NCT03533946	A Phase II Study of Rucaparib Monotherapy in Nonmetastatic, Hormone-Sensitive Prostate Cancer Demonstrating "BRCAness" Genotype (ROAR)	II
NCT04892693	Talazoparib in Advanced Breast Cancer Patients With Homologous Recombinant Deficiency: A Phase II Clinical and Exploratory Biomarker Study of Talazoparib	II
NCT04740190	Combination Talazoparib - Carboplatin for Recurrent High-grade Glioma With DNA Damage Repair Deficiency (DDRd)	II
NCT04253262	A Phase Ib/II Study of Copanlisib Combined With Rucaparib in Patients With Metastatic Castration-resistant Prostate Cancer	I/II
NCT04336943	Durvalumab (MEDI4736) and Olaparib (AZD2281) for Treatment of Biochemically Recurrent Prostate Cancer in Men Predicted to Have a High Neoantigen Load: A Pilot Study	II
NCT04169841	Precision Medicine Phase II Study Evaluating the Efficacy of a Double Immunotherapy by Durvalumab and Tremelimumab Combined With Olaparib in Patients With Solid Cancers and Carriers of Homologous Recombination Repair Genes Mutation in Response or Stable After Olaparib Treatment	II
NCT03925350	A Phase II Study of Niraparib in Patients With Advanced Melanoma With Genetic Homologous Recombination (HR) Mutation / Alteration	II
NCT04940637	A Phase II, Open-Label, Single Arm, Prospective, Multicenter Study of Niraparib Plus Dostarlimab in Patients With Advanced NSCLC and/or MPM, and Positive for PD-L1 Expression and Germline or Somatic Mutations in the HRR Genes	II
NCT04042831	A Phase II Study of Olaparib in Patients With Advanced Biliary Tract Cancer With Aberrant DNA Repair Gene Mutations	II
NCT04939662	Phase II, Single-arm Study of Olaparib and Bevacizumab Combination Therapy in Relapsed Small Cell Lung Cancer Subjects With DNA Damage Response and the Repair Pathway Alteration, ATM Deficiency, SLFN11 Positive, or POU2F3 Positive	II
NCT03506997	PERSEUS1: Phase II Trial of the Immune Checkpoint Inhibitor Pembrolizumab for Patients Suffering From Metastatic Prostate Cancer	II
NCT02264678	A Modular Phase I, Open-Label, Multicentre Study to Assess the Safety, Tolerability, Pharmacokinetics and Preliminary Anti-tumour Activity of Ceralasertib in Combination With Cytotoxic Chemotherapy and/or DNA Damage Repair/Novel Anti-cancer Agents in Patients With Advanced Solid Malignancies	I
NCT04890613	Phase Ib Expansion Study of CX-5461 in Patients With Solid Tumours and BRCA2 and/or PALB2 Mutation	I
NCT05002868	A Multi-center, Open-label, Phase I/Ib Study to Assess the Safety, Pharmacokinetics and Anti-tumor Activity of RP12146, a Poly (ADP-ribose) Polymerase (PARP) Inhibitor, in Patients With Locally Advanced or Metastatic Solid Tumors	I

Clinical Trials Summary (continued)

CHEK1 mutation (continued)

NCT ID	Title	Phase
NCT03395197	A Phase III, Randomized, Double-blind, Placebo-controlled Study Of Talazoparib With Enzalutamide In Metastatic Castration-resistant Prostate Cancer	III
NCT04821622	TALAPRO-3: A Phase III, Randomized, Double-blind, Study of Talazoparib With Enzalutamide Versus Placebo With Enzalutamide in Men With DDR Gene Mutated Metastatic Castration-sensitive Prostate Cancer	III
NCT03810105	A Phase II Study of Olaparib and Durvalumab in Men With Castration Sensitive Biochemically Recurrent Non-Metastatic Prostate Cancer Harboring Mutations in DNA Damage Repair	II
NCT03012321	BRCAAway: A Randomized Phase II Trial of Abiraterone, Olaparib, or Abiraterone + Olaparib in Patients With Metastatic Castration-Resistant Prostate Cancer With DNA Repair Defects	II
NCT03344965	A Phase II Study of Olaparib Monotherapy in Metastatic Breast Cancer Patients With Germline or Somatic Mutations in DNA Repair Genes (Olaparib Expanded)	II
NCT04038502	An Open-label, Multicenter Phase II Study to Compare the Efficacy of Carboplatin as First-line Followed by Second-line Olaparib Versus Olaparib as First-line Followed by Second-line Carboplatin in the Treatment of Patients With Castration Resistant Prostate Cancer Containing Homologous Recombination Deficiency	II
NCT04633902	Phase II Study of Olaparib in Combination With Pembrolizumab in Patients With Advanced Melanoma With Homologous Recombination (HR) Pathway Gene Mutation	II
NCT03248570	Phase II Open Label Study of Pembrolizumab in Patients With Metastatic Castration Resistant Prostate Cancer (mCRPC) With or Without DNA Damage Repair Defects	II
No NCT ID	Single-arm Exploratory Study for Sintilimab Second-line or Later-line Treatment of Advanced Non-small Cell Lung Cancer with DDR Pathway Gene Mutation	I/II
NCT04095273	A Multicenter, Non-randomized, Open-label Phase Ib Study to Determine the Maximum Tolerated and Recommended Phase II Dose of the ATR Inhibitor BAY1895344 in Combination With Pembrolizumab and to Characterize Its Safety, Tolerability, Pharmacokinetics and Preliminary Anti-tumor Activity in Patients With Advanced Solid Tumors	I
NCT04849364	A Phase II Circulating Tumor DNA Enriched, Genomically Directed Post-neoadjuvant Trial for Patients With Residual Triple Negative Breast Cancer (PERSEVERE)	II

CHEK1 truncating mutation

NCT ID	Title	Phase
NCT04550494	A Pharmacodynamics-Driven Trial of Talazoparib, an Oral PARP Inhibitor, in Patients With Advanced Solid Tumors and Aberrations in Genes Involved in DNA Damage Response	II
NCT03742895	A Phase II Study of Olaparib Monotherapy in Participants With Previously Treated, Homologous Recombination Repair Mutation (HRRm) or Homologous Recombination Deficiency (HRD) Positive Advanced Cancer	II
NCT03967938	Efficacy of Olaparib in Advanced Cancers Occurring in Patients With Germline Mutations or Somatic Tumor Mutations in Homologous Recombination Genes	II
NCT03842228	A Phase Ib Biomarker-Driven Combination Trial of Copanlisib, Olaparib, and Durvalumab (MEDI4736) in Patients With Advanced Solid Tumors	I
NCT04586335	Open Label, Phase Ib Study to Evaluate the Safety, Tolerability, Pharmacokinetics and Clinical Activity of CYH33, an Oral a-specific PI3K Inhibitor in Combination With Olaparib, an Oral PARP Inhibitor in Patients With Advanced Solid Tumors.	I

Disclaimer: The data presented here is from a curated knowledgebase of publicly available information, but may not be exhaustive. The data version is 2021.11(003).

Clinical Trials Summary (continued)

CHEK1 truncating mutation (continued)

NCT ID	Title	Phase
NCT02029001	A Two-period, Multicenter, Randomized, Open-label, Phase II Study Evaluating the Clinical Benefit of a Maintenance Treatment Targeting Tumor Molecular Alterations in Patients with Progressive Locally-advanced or Metastatic Solid Tumors.	II
NCT04123366	A Phase II Study of Olaparib in Combination With Pembrolizumab in Participants With Previously Treated, Homologous Recombination Repair Mutation (HRRm) and/or Homologous Recombination Deficiency (HRD)-Positive Advanced Cancer	II
NCT02401347	A Phase II Clinical Trial of the PARP Inhibitor Talazoparib in BRCA1 and BRCA2 Wild Type Patients With Advanced Triple Negative Breast Cancer and Homologous Recombination Deficiency or Advanced HER2 Negative Breast Cancer or Other Solid Tumors With a Mutation in Homologous Recombination Pathway Genes Talazoparib Beyond BRCA (TBB) Trial	II
NCT03415659	A Phase I, Open-label, Single-center, Single/Multiple-dose, Dose-escalation/Dose-expansion Clinical Study on Tolerance and Pharmacokinetics of HWH340 Tablet in Patients With Advanced Solid Tumors	I
NCT03767075	Basket of Baskets: A Modular, Open-label, Phase II, Multicentre Study To Evaluate Targeted Agents in Molecularly Selected Populations With Advanced Solid Tumours	II
NCT04905914	A Phase I/IIa, Open-Label, Safety, Pharmacokinetic, And Preliminary Efficacy Study Of Oral ATRN-119 In Patients With Advanced Solid Tumors	I/II
NCT04901702	A Randomized Phase I/II Study of Talazoparib or Temozolomide in Combination With Onivyde in Children With Recurrent Solid Malignancies and Ewing Sarcoma	I/II
NCT03155620	NCI-COG Pediatric MATCH (Molecular Analysis for Therapy Choice) Screening Protocol	II
NCT03233204	NCI-COG Pediatric MATCH (Molecular Analysis for Therapy Choice)- Phase 2 Subprotocol of Olaparib in Patients With Tumors Harboring Defects in DNA Damage Repair Genes	II
NCT04267939	An Open-label Phase Ib Study to Determine the Maximum Tolerated and/or Recommended Phase II Dose of the ATR Inhibitor BAY 1895344 in Combination With PARP Inhibitor Niraparib, in Patients With Recurrent Advanced Solid Tumors and Ovarian Cancer	I
NCT04693468	Modular Phase IB Hypothesis-Testing, Biomarker-Driven, Talazoparib Combination Trial (TalaCom)	I
NCT04053322	An International Multicenter Phase II Trial of Durvalumab (MEDI4736) Plus OLAparib Plus Fulvestrant in Metastatic or Locally Advanced ER-positive, HER2-negative Breast Cancer Patients Selected Using Criteria That Predict Sensitivity to Olaparib	II
NCT03553004	Niraparib in Metastatic Pancreatic Cancer After Previous Chemotherapy (NIRA-PANC): A Phase II Trial	II
NCT04030559	Phase II Trial of PARP Inhibitor Niraparib for Men With High Risk Prostate Cancer and DNA Damage Response Defects	II
NCT04187833	Phase II Trial of Nivolumab in Combination with Talazoparib in Patients with Unresectable or Metastatic Melanoma and Mutations in BRCA or BRCA-ness Genes.	II
NCT03375307	A Phase II Study of Olaparib (AZD2281) in Patients With Metastatic/Advanced Urothelial Carcinoma With DNA-Repair Defects	II
NCT03786796	Phase II Study of Olaparib in Metastatic Renal Cell Carcinoma Patients Harboring a BAP-1 or Other DNA Repair Gene Mutations (ORCHID)	II
NCT04515836	Phase II Trial of Olaparib in Homologous Recombination Deficient (HRD) Malignant Mesothelioma	II
NCT03533946	A Phase II Study of Rucaparib Monotherapy in Nonmetastatic, Hormone-Sensitive Prostate Cancer Demonstrating "BRCAness" Genotype (ROAR)	II

Clinical Trials Summary (continued)

CHEK1 truncating mutation (continued)

NCT ID	Title	Phase
NCT04892693	Talazoparib in Advanced Breast Cancer Patients With Homologous Recombinant Deficiency: A Phase II Clinical and Exploratory Biomarker Study of Talazoparib	II
NCT04740190	Combination Talazoparib - Carboplatin for Recurrent High-grade Glioma With DNA Damage Repair Deficiency (DDRd)	II
NCT04253262	A Phase Ib/II Study of Copanlisib Combined With Rucaparib in Patients With Metastatic Castration-resistant Prostate Cancer	I/II
NCT03810105	A Phase II Study of Olaparib and Durvalumab in Men With Castration Sensitive Biochemically Recurrent Non-Metastatic Prostate Cancer Harboring Mutations in DNA Damage Repair	II
NCT04336943	Durvalumab (MEDI4736) and Olaparib (AZD2281) for Treatment of Biochemically Recurrent Prostate Cancer in Men Predicted to Have a High Neoantigen Load: A Pilot Study	II
NCT04169841	Precision Medicine Phase II Study Evaluating the Efficacy of a Double Immunotherapy by Durvalumab and Tremelimumab Combined With Olaparib in Patients With Solid Cancers and Carriers of Homologous Recombination Repair Genes Mutation in Response or Stable After Olaparib Treatment	II
NCT03925350	A Phase II Study of Niraparib in Patients With Advanced Melanoma With Genetic Homologous Recombination (HR) Mutation / Alteration	II
NCT04940637	A Phase II, Open-Label, Single Arm, Prospective, Multicenter Study of Niraparib Plus Dostarlimab in Patients With Advanced NSCLC and/or MPM, and Positive for PD-L1 Expression and Germline or Somatic Mutations in the HRR Genes	II
NCT04042831	A Phase II Study of Olaparib in Patients With Advanced Biliary Tract Cancer With Aberrant DNA Repair Gene Mutations	II
NCT04939662	Phase II, Single-arm Study of Olaparib and Bevacizumab Combination Therapy in Relapsed Small Cell Lung Cancer Subjects With DNA Damage Response and the Repair Pathway Alteration, ATM Deficiency, SLFN11 Positive, or POU2F3 Positive	II
NCT03506997	PERSEUS1: Phase II Trial of the Immune Checkpoint Inhibitor Pembrolizumab for Patients Suffering From Metastatic Prostate Cancer	II
NCT02264678	A Modular Phase I, Open-Label, Multicentre Study to Assess the Safety, Tolerability, Pharmacokinetics and Preliminary Anti-tumour Activity of Ceralasertib in Combination With Cytotoxic Chemotherapy and/or DNA Damage Repair/Novel Anti-cancer Agents in Patients With Advanced Solid Malignancies	I
NCT04890613	Phase Ib Expansion Study of CX-5461 in Patients With Solid Tumours and BRCA2 and/or PALB2 Mutation	I
NCT05002868	A Multi-center, Open-label, Phase I/Ib Study to Assess the Safety, Pharmacokinetics and Anti-tumor Activity of RP12146, a Poly (ADP-ribose) Polymerase (PARP) Inhibitor, in Patients With Locally Advanced or Metastatic Solid Tumors	I
NCT03395197	A Phase III, Randomized, Double-blind, Placebo-controlled Study Of Talazoparib With Enzalutamide In Metastatic Castration-resistant Prostate Cancer	III
NCT04821622	TALAPRO-3: A Phase III, Randomized, Double-blind, Study of Talazoparib With Enzalutamide Versus Placebo With Enzalutamide in Men With DDR Gene Mutated Metastatic Castration-sensitive Prostate Cancer	III
NCT03012321	BRCAAway: A Randomized Phase II Trial of Abiraterone, Olaparib, or Abiraterone + Olaparib in Patients With Metastatic Castration-Resistant Prostate Cancer With DNA Repair Defects	II
NCT03344965	A Phase II Study of Olaparib Monotherapy in Metastatic Breast Cancer Patients With Germline or Somatic Mutations in DNA Repair Genes (Olaparib Expanded)	II

Disclaimer: The data presented here is from a curated knowledgebase of publicly available information, but may not be exhaustive. The data version is 2021.11(003).

Clinical Trials Summary (continued)

CHEK1 truncating mutation (continued)

NCT ID	Title	Phase
NCT04038502	An Open-label, Multicenter Phase II Study to Compare the Efficacy of Carboplatin as First-line Followed by Second-line Olaparib Versus Olaparib as First-line Followed by Second-line Carboplatin in the Treatment of Patients With Castration Resistant Prostate Cancer Containing Homologous Recombination Deficiency	II
NCT04633902	Phase II Study of Olaparib in Combination With Pembrolizumab in Patients With Advanced Melanoma With Homologous Recombination (HR) Pathway Gene Mutation	II
NCT03248570	Phase II Open Label Study of Pembrolizumab in Patients With Metastatic Castration Resistant Prostate Cancer (mCRPC) With or Without DNA Damage Repair Defects	II
No NCT ID	Single-arm Exploratory Study for Sintilimab Second-line or Later-line Treatment of Advanced Non-small Cell Lung Cancer with DDR Pathway Gene Mutation	I/II
NCT04095273	A Multicenter, Non-randomized, Open-label Phase Ib Study to Determine the Maximum Tolerated and Recommended Phase II Dose of the ATR Inhibitor BAY1895344 in Combination With Pembrolizumab and to Characterize Its Safety, Tolerability, Pharmacokinetics and Preliminary Anti-tumor Activity in Patients With Advanced Solid Tumors	I
NCT04849364	A Phase II Circulating Tumor DNA Enriched, Genomically Directed Post-neoadjuvant Trial for Patients With Residual Triple Negative Breast Cancer (PERSEVERE)	II

CHEK1 deletion

NCT ID	Title	Phase
NCT04901702	A Randomized Phase I/II Study of Talazoparib or Temozolomide in Combination With Onivyde in Children With Recurrent Solid Malignancies and Ewing Sarcoma	I/II
NCT03155620	NCI-COG Pediatric MATCH (Molecular Analysis for Therapy Choice) Screening Protocol	II
NCT03233204	NCI-COG Pediatric MATCH (Molecular Analysis for Therapy Choice)- Phase 2 Subprotocol of Olaparib in Patients With Tumors Harboring Defects in DNA Damage Repair Genes	II
NCT04267939	An Open-label Phase Ib Study to Determine the Maximum Tolerated and/or Recommended Phase II Dose of the ATR Inhibitor BAY 1895344 in Combination With PARP Inhibitor Niraparib, in Patients With Recurrent Advanced Solid Tumors and Ovarian Cancer	I
NCT04693468	Modular Phase IB Hypothesis-Testing, Biomarker-Driven, Talazoparib Combination Trial (TalaCom)	I
NCT04030559	Phase II Trial of PARP Inhibitor Niraparib for Men With High Risk Prostate Cancer and DNA Damage Response Defects	II
NCT04740190	Combination Talazoparib - Carboplatin for Recurrent High-grade Glioma With DNA Damage Repair Deficiency (DDRd)	II
NCT03810105	A Phase II Study of Olaparib and Durvalumab in Men With Castration Sensitive Biochemically Recurrent Non-Metastatic Prostate Cancer Harboring Mutations in DNA Damage Repair	II
NCT03344965	A Phase II Study of Olaparib Monotherapy in Metastatic Breast Cancer Patients With Germline or Somatic Mutations in DNA Repair Genes (Olaparib Expanded)	II
NCT04633902	Phase II Study of Olaparib in Combination With Pembrolizumab in Patients With Advanced Melanoma With Homologous Recombination (HR) Pathway Gene Mutation	II
NCT03248570	Phase II Open Label Study of Pembrolizumab in Patients With Metastatic Castration Resistant Prostate Cancer (mCRPC) With or Without DNA Damage Repair Defects	II

Disclaimer: The data presented here is from a curated knowledgebase of publicly available information, but may not be exhaustive. The data version is 2021.11(003).

Clinical Trials Summary (continued)

CHEK1 deletion (continued)

NCT ID	Title	Phase
NCT03506997	PERSEUS1: Phase II Trial of the Immune Checkpoint Inhibitor Pembrolizumab for Patients Suffering From Metastatic Prostate Cancer	II
NCT04849364	A Phase II Circulating Tumor DNA Enriched, Genomically Directed Post-neoadjuvant Trial for Patients With Residual Triple Negative Breast Cancer (PERSEVERE)	II
NCT05002868	A Multi-center, Open-label, Phase I/Ib Study to Assess the Safety, Pharmacokinetics and Anti-tumor Activity of RP12146, a Poly (ADP-ribose) Polymerase (PARP) Inhibitor, in Patients With Locally Advanced or Metastatic Solid Tumors	I

Disclaimer: The data presented here is from a curated knowledgebase of publicly available information, but may not be exhaustive. The data version is 2021.11(003).

Sample Cancer Type: Unknown Primary Origin

Table of Contents

Table of Contents	Page
Relevant Therapy Details	1
Alert Details	7
Clinical Trials	9
Relevant Therapy Summary	35
Clinical Trials Summary	38

Report Highlights

1 Relevant Biomarkers
8 Therapies Available
90 Clinical Trials

Relevant Biomarkers

Genomic Alteration	Relevant Therapies (In this cancer type)	Relevant Therapies (In other cancer type)	Clinical Trials
EGFR A289T mutation	None	afatinib<br>bevacizumab + erlotinib<br>bevacizumab + gefitinib<br>dacomitinib<br>erlotinib<br>erlotinib + ramucirumab<br>gefitinib<br>gefitinib + chemotherapy	90

Prognostic significance: None
Diagnostic significance: None

Public data sources included in relevant therapies: FDA1, NCCN, **EMA**2, ESMO
Public data sources included in prognostic and diagnostic significance: NCCN, ESMO

Relevant Therapy Details

Current ESMO Information

● In this cancer type ○ In other cancer type ◐ In this cancer type and other cancer types

ESMO information is current as of 2021-10-01. For the most up-to-date information, search www.esmo.org.

EGFR A289T mutation

○ afatinib

Cancer type: Non-Small Cell Lung Cancer **Variant class:** EGFR activating mutation

ESMO Level of Evidence/Grade of Recommendation: I / A

Population segment (Line of therapy):
- Stage IV (First-line therapy)

Reference: ESMO Clinical Practice Guidelines - ESMO-Metastatic Non-Small-Cell Lung Cancer [Online Guideline (15SEP2020 - https://www.esmo.org/guidelines/lung-and-chest-tumours/clinical-practice-living-guidelines-metastatic-non-small-cell-lung-cancer); Ann Oncol (2018) 29 (suppl 4): iv192–iv237.]

Disclaimer: The data presented here is from a curated knowledgebase of publicly available information, but may not be exhaustive. The data version is 2021.11(003).The content of this report has not been evaluated or approved by the FDA or other regulatory agencies.

EGFR A289T mutation (continued)

bevacizumab + erlotinib

Cancer type: Non-Small Cell Lung Cancer **Variant class:** EGFR activating mutation

ESMO Level of Evidence/Grade of Recommendation: I / A

Population segment (Line of therapy):
- Stage IV (First-line therapy)

Reference: ESMO Clinical Practice Guidelines - ESMO-Metastatic Non-Small-Cell Lung Cancer [Online Guideline (15SEP2020 - https://www.esmo.org/guidelines/lung-and-chest-tumours/clinical-practice-living-guidelines-metastatic-non-small-cell-lung-cancer); Ann Oncol (2018) 29 (suppl 4): iv192–iv237.]

bevacizumab + gefitinib

Cancer type: Non-Small Cell Lung Cancer **Variant class:** EGFR activating mutation

ESMO Level of Evidence/Grade of Recommendation: I / A

Population segment (Line of therapy):
- Stage IV (First-line therapy)

Reference: ESMO Clinical Practice Guidelines - ESMO-Metastatic Non-Small-Cell Lung Cancer [Online Guideline (15SEP2020 - https://www.esmo.org/guidelines/lung-and-chest-tumours/clinical-practice-living-guidelines-metastatic-non-small-cell-lung-cancer); Ann Oncol (2018) 29 (suppl 4): iv192–iv237.]

dacomitinib

Cancer type: Non-Small Cell Lung Cancer **Variant class:** EGFR activating mutation

ESMO Level of Evidence/Grade of Recommendation: I / A

Population segment (Line of therapy):
- Stage IV (First-line therapy)

Reference: ESMO Clinical Practice Guidelines - ESMO-Metastatic Non-Small-Cell Lung Cancer [Online Guideline (15SEP2020 - https://www.esmo.org/guidelines/lung-and-chest-tumours/clinical-practice-living-guidelines-metastatic-non-small-cell-lung-cancer); Ann Oncol (2018) 29 (suppl 4): iv192–iv237.]

erlotinib

Cancer type: Non-Small Cell Lung Cancer **Variant class:** EGFR activating mutation

ESMO Level of Evidence/Grade of Recommendation: I / A

Population segment (Line of therapy):
- Stage IV (First-line therapy)

Reference: ESMO Clinical Practice Guidelines - ESMO-Metastatic Non-Small-Cell Lung Cancer [Online Guideline (15SEP2020 - https://www.esmo.org/guidelines/lung-and-chest-tumours/clinical-practice-living-guidelines-metastatic-non-small-cell-lung-cancer); Ann Oncol (2018) 29 (suppl 4): iv192–iv237.]

Disclaimer: The data presented here is from a curated knowledgebase of publicly available information, but may not be exhaustive. The data version is 2021.11(003).

EGFR A289T mutation (continued)

erlotinib + ramucirumab

Cancer type: Non-Small Cell Lung Cancer **Variant class:** EGFR activating mutation

ESMO Level of Evidence/Grade of Recommendation: I / A

Population segment (Line of therapy):
- Stage IV (First-line therapy)

Reference: ESMO Clinical Practice Guidelines - ESMO-Metastatic Non-Small-Cell Lung Cancer [Online Guideline (15SEP2020 - https://www.esmo.org/guidelines/lung-and-chest-tumours/clinical-practice-living-guidelines-metastatic-non-small-cell-lung-cancer); Ann Oncol (2018) 29 (suppl 4): iv192–iv237.]

gefitinib

Cancer type: Non-Small Cell Lung Cancer **Variant class:** EGFR activating mutation

ESMO Level of Evidence/Grade of Recommendation: I / A

Population segment (Line of therapy):
- Stage IV (First-line therapy)

Reference: ESMO Clinical Practice Guidelines - ESMO-Metastatic Non-Small-Cell Lung Cancer [Online Guideline (15SEP2020 - https://www.esmo.org/guidelines/lung-and-chest-tumours/clinical-practice-living-guidelines-metastatic-non-small-cell-lung-cancer); Ann Oncol (2018) 29 (suppl 4): iv192–iv237.]

gefitinib + carboplatin + pemetrexed

Cancer type: Non-Small Cell Lung Cancer **Variant class:** EGFR activating mutation

ESMO Level of Evidence/Grade of Recommendation: I / A

Population segment (Line of therapy):
- Stage IV (First-line therapy)

Reference: ESMO Clinical Practice Guidelines - ESMO-Metastatic Non-Small-Cell Lung Cancer [Online Guideline (15SEP2020 - https://www.esmo.org/guidelines/lung-and-chest-tumours/clinical-practice-living-guidelines-metastatic-non-small-cell-lung-cancer); Ann Oncol (2018) 29 (suppl 4): iv192–iv237.]

bevacizumab + erlotinib

Cancer type: Non-Small Cell Lung Cancer **Variant class:** EGFR activating mutation

ESMO Level of Evidence/Grade of Recommendation: I / B

Population segment (Line of therapy):
- Stage IV (First-line therapy); ESMO-MCBS v1.1 score: 3

Reference: ESMO Clinical Practice Guidelines - ESMO-Metastatic Non-Small-Cell Lung Cancer [Online Guideline (15SEP2020 - https://www.esmo.org/guidelines/lung-and-chest-tumours/clinical-practice-living-guidelines-metastatic-non-small-cell-lung-cancer); Ann Oncol (2018) 29 (suppl 4): iv192–iv237.]

Disclaimer: The data presented here is from a curated knowledgebase of publicly available information, but may not be exhaustive. The data version is 2021.11(003).

EGFR A289T mutation (continued)

○ bevacizumab + gefitinib

Cancer type: Non-Small Cell Lung Cancer **Variant class:** EGFR activating mutation

ESMO Level of Evidence/Grade of Recommendation: I / B

Population segment (Line of therapy):
- Stage IV (First-line therapy); ESMO-MCBS v1.1 score: 3

Reference: ESMO Clinical Practice Guidelines - ESMO-Metastatic Non-Small-Cell Lung Cancer [Online Guideline (15SEP2020 - https://www.esmo.org/guidelines/lung-and-chest-tumours/clinical-practice-living-guidelines-metastatic-non-small-cell-lung-cancer); Ann Oncol (2018) 29 (suppl 4): iv192–iv237.]

○ erlotinib + ramucirumab

Cancer type: Non-Small Cell Lung Cancer **Variant class:** EGFR activating mutation

ESMO Level of Evidence/Grade of Recommendation: I / B

Population segment (Line of therapy):
- Stage IV (First-line therapy); ESMO-MCBS v1.1 score: 3

Reference: ESMO Clinical Practice Guidelines - ESMO-Metastatic Non-Small-Cell Lung Cancer [Online Guideline (15SEP2020 - https://www.esmo.org/guidelines/lung-and-chest-tumours/clinical-practice-living-guidelines-metastatic-non-small-cell-lung-cancer); Ann Oncol (2018) 29 (suppl 4): iv192–iv237.]

○ gefitinib + carboplatin + pemetrexed

Cancer type: Non-Small Cell Lung Cancer **Variant class:** EGFR activating mutation

ESMO Level of Evidence/Grade of Recommendation: I / B

Population segment (Line of therapy):
- Advanced (First-line therapy)

Reference: ESMO Clinical Practice Guidelines - ESMO-Metastatic Non-Small-Cell Lung Cancer [Online Guideline (15SEP2020 - https://www.esmo.org/guidelines/lung-and-chest-tumours/clinical-practice-living-guidelines-metastatic-non-small-cell-lung-cancer); Ann Oncol (2018) 29 (suppl 4): iv192–iv237.]

○ afatinib

Cancer type: Non-Small Cell Lung Cancer **Variant class:** EGFR activating mutation

ESMO Level of Evidence/Grade of Recommendation: III / A

Population segment (Line of therapy):
- Stage IV (First-line therapy)

Reference: ESMO Clinical Practice Guidelines - ESMO-Metastatic Non-Small-Cell Lung Cancer [Online Guideline (15SEP2020 - https://www.esmo.org/guidelines/lung-and-chest-tumours/clinical-practice-living-guidelines-metastatic-non-small-cell-lung-cancer); Ann Oncol (2018) 29 (suppl 4): iv192–iv237.]

Disclaimer: The data presented here is from a curated knowledgebase of publicly available information, but may not be exhaustive. The data version is 2021.11(003).

EGFR A289T mutation (continued)

○ bevacizumab + erlotinib

Cancer type: Non-Small Cell Lung Cancer **Variant class:** EGFR activating mutation

ESMO Level of Evidence/Grade of Recommendation: III / A

Population segment (Line of therapy):
- Stage IV (First-line therapy)

Reference: ESMO Clinical Practice Guidelines - ESMO-Metastatic Non-Small-Cell Lung Cancer [Online Guideline (15SEP2020 - https://www.esmo.org/guidelines/lung-and-chest-tumours/clinical-practice-living-guidelines-metastatic-non-small-cell-lung-cancer); Ann Oncol (2018) 29 (suppl 4): iv192–iv237.]

○ bevacizumab + gefitinib

Cancer type: Non-Small Cell Lung Cancer **Variant class:** EGFR activating mutation

ESMO Level of Evidence/Grade of Recommendation: III / A

Population segment (Line of therapy):
- Stage IV (First-line therapy)

Reference: ESMO Clinical Practice Guidelines - ESMO-Metastatic Non-Small-Cell Lung Cancer [Online Guideline (15SEP2020 - https://www.esmo.org/guidelines/lung-and-chest-tumours/clinical-practice-living-guidelines-metastatic-non-small-cell-lung-cancer); Ann Oncol (2018) 29 (suppl 4): iv192–iv237.]

○ dacomitinib

Cancer type: Non-Small Cell Lung Cancer **Variant class:** EGFR activating mutation

ESMO Level of Evidence/Grade of Recommendation: III / A

Population segment (Line of therapy):
- Stage IV (First-line therapy)

Reference: ESMO Clinical Practice Guidelines - ESMO-Metastatic Non-Small-Cell Lung Cancer [Online Guideline (15SEP2020 - https://www.esmo.org/guidelines/lung-and-chest-tumours/clinical-practice-living-guidelines-metastatic-non-small-cell-lung-cancer); Ann Oncol (2018) 29 (suppl 4): iv192–iv237.]

○ erlotinib

Cancer type: Non-Small Cell Lung Cancer **Variant class:** EGFR activating mutation

ESMO Level of Evidence/Grade of Recommendation: III / A

Population segment (Line of therapy):
- Stage IV (First-line therapy)

Reference: ESMO Clinical Practice Guidelines - ESMO-Metastatic Non-Small-Cell Lung Cancer [Online Guideline (15SEP2020 - https://www.esmo.org/guidelines/lung-and-chest-tumours/clinical-practice-living-guidelines-metastatic-non-small-cell-lung-cancer); Ann Oncol (2018) 29 (suppl 4): iv192–iv237.]

Disclaimer: The data presented here is from a curated knowledgebase of publicly available information, but may not be exhaustive. The data version is 2021.11(003).

EGFR A289T mutation (continued)

○ erlotinib + ramucirumab

Cancer type: Non-Small Cell Lung Cancer **Variant class:** EGFR activating mutation

ESMO Level of Evidence/Grade of Recommendation: III / A

Population segment (Line of therapy):

- Stage IV (First-line therapy)

Reference: ESMO Clinical Practice Guidelines - ESMO-Metastatic Non-Small-Cell Lung Cancer [Online Guideline (15SEP2020 - https://www.esmo.org/guidelines/lung-and-chest-tumours/clinical-practice-living-guidelines-metastatic-non-small-cell-lung-cancer); Ann Oncol (2018) 29 (suppl 4): iv192–iv237.]

○ gefitinib

Cancer type: Non-Small Cell Lung Cancer **Variant class:** EGFR activating mutation

ESMO Level of Evidence/Grade of Recommendation: III / A

Population segment (Line of therapy):

- Stage IV (First-line therapy)

Reference: ESMO Clinical Practice Guidelines - ESMO-Metastatic Non-Small-Cell Lung Cancer [Online Guideline (15SEP2020 - https://www.esmo.org/guidelines/lung-and-chest-tumours/clinical-practice-living-guidelines-metastatic-non-small-cell-lung-cancer); Ann Oncol (2018) 29 (suppl 4): iv192–iv237.]

○ gefitinib + carboplatin + pemetrexed

Cancer type: Non-Small Cell Lung Cancer **Variant class:** EGFR activating mutation

ESMO Level of Evidence/Grade of Recommendation: III / A

Population segment (Line of therapy):

- Stage IV (First-line therapy)

Reference: ESMO Clinical Practice Guidelines - ESMO-Metastatic Non-Small-Cell Lung Cancer [Online Guideline (15SEP2020 - https://www.esmo.org/guidelines/lung-and-chest-tumours/clinical-practice-living-guidelines-metastatic-non-small-cell-lung-cancer); Ann Oncol (2018) 29 (suppl 4): iv192–iv237.]

Disclaimer: The data presented here is from a curated knowledgebase of publicly available information, but may not be exhaustive. The data version is 2021.11(003).

Alerts Informed By Public Data Sources

Current FDA Information

Contraindicated | Not recommended | Resistance | Breakthrough | Fast Track

FDA information is current as of 2021-10-13. For the most up-to-date information, search www.fda.gov.

EGFR A289T mutation

osimertinib + quaratusugene ozeplasmid

Cancer type: Non-Small Cell Lung Cancer **Variant class:** EGFR mutation

Supporting Statement:
The FDA has granted Fast Track Designation to the immunogene therapy, quaratusugene ozeplasmid, in combination with EGFR inhibitor osimertinib for the treatment of non-small cell lung cancer (NSCLC) with EFGR mutations that progressed after treatment with osimertinib alone.

Reference:

https://www.genprex.com/news/genprex-receives-u-s-fda-fast-track-designation-for-gene-therapy-that-targets-lung-cancer/

Current NCCN Information

Contraindicated | Not recommended | Resistance | Breakthrough | Fast Track

NCCN information is current as of 2021-10-01. For the most up-to-date information, search www.nccn.org.
For NCCN International Adaptations & Translations, search www.nccn.org/global/international_adaptations.aspx.

EGFR A289T mutation

atezolizumab

Cancer type: Non-Small Cell Lung Cancer **Variant class:** EGFR mutation

Summary:
NCCN Guidelines® include the following supporting statement(s):
- "subsequent therapy with pembrolizumab, nivolumab, or atezolizumab is not recommended in patients with EGFR mutations or ALK fusions."

Reference: NCCN Guidelines® - NCCN-Non-Small Cell Lung Cancer [Version 6.2021]

nivolumab

Cancer type: Non-Small Cell Lung Cancer **Variant class:** EGFR mutation

Summary:
NCCN Guidelines® include the following supporting statement(s):
- "subsequent therapy with pembrolizumab, nivolumab, or atezolizumab is not recommended in patients with EGFR mutations or ALK fusions."

Reference: NCCN Guidelines® - NCCN-Non-Small Cell Lung Cancer [Version 6.2021]

Disclaimer: The data presented here is from a curated knowledgebase of publicly available information, but may not be exhaustive. The data version is 2021.11(003).

EGFR A289T mutation (continued)

pembrolizumab

Cancer type: Non-Small Cell Lung Cancer **Variant class:** EGFR mutation

Summary:
NCCN Guidelines® include the following supporting statement(s):

- "subsequent therapy with pembrolizumab, nivolumab, or atezolizumab is not recommended in patients with EGFR mutations or ALK fusions."

Reference: NCCN Guidelines® - NCCN-Non-Small Cell Lung Cancer [Version 6.2021]

Disclaimer: The data presented here is from a curated knowledgebase of publicly available information, but may not be exhaustive. The data version is 2021.11(003).

Current Clinical Trials Information

Clinical Trials information is current as of 2021-10-01. For the most up-to-date information regarding a particular trial, search www.clinicaltrials.gov by NCT ID or search local clinical trials authority website by local identifier listed in 'Other identifiers'.

EGFR A289T mutation

NCT04172597
A Phase II Study of Poziotinib in Patients With EGFR or HER2 Activating Mutations in Advanced Malignancies

Cancer type: Anaplastic Astrocytoma, Anaplastic Oligoastrocytoma, Anaplastic Oligodendroglioma, Anaplastic Pleomorphic Xanthoastrocytoma, Diffuse Midline Glioma, Glioblastoma, Unspecified Solid Tumor

Variant class: EGFR A289T mutation

Other identifiers: NCI-2020-13871, SPI-POZ-203

Population segments: EGFR, Estrogen receptor positive, HER2 negative, HER2 positive, Second line, Stage IV

Exclusion criteria variant classes: EGFR A289T mutation (Non-Small Cell Lung Cancer), EGFR A864V mutation (Non-Small Cell Lung Cancer), EGFR E709K mutation (Non-Small Cell Lung Cancer), EGFR E746_A750del mutation (Non-Small Cell Lung Cancer), EGFR G598V mutation (Non-Small Cell Lung Cancer), EGFR G719 mutation (Non-Small Cell Lung Cancer), EGFR L858R mutation (Non-Small Cell Lung Cancer), EGFR L861Q mutation (Non-Small Cell Lung Cancer), EGFR P596L mutation (Non-Small Cell Lung Cancer), EGFR R108K mutation (Non-Small Cell Lung Cancer), EGFR R222C mutation (Non-Small Cell Lung Cancer), EGFR R831C mutation (Non-Small Cell Lung Cancer), EGFR R831H mutation (Non-Small Cell Lung Cancer), EGFR S768I mutation (Non-Small Cell Lung Cancer), EGFR T790M mutation (Unspecified Solid Tumor), EGFR V742I mutation (Non-Small Cell Lung Cancer), EGFR V769M mutation (Non-Small Cell Lung Cancer), EGFR V774M mutation (Non-Small Cell Lung Cancer), EGFR exon 20 insertion (Non-Small Cell Lung Cancer), EGFR vIII (Non-Small Cell Lung Cancer), ERBB2 D769H mutation (Non-Small Cell Lung Cancer), ERBB2 D769N mutation (Non-Small Cell Lung Cancer), ERBB2 D769Y mutation (Non-Small Cell Lung Cancer), ERBB2 I655V mutation (Non-Small Cell Lung Cancer), ERBB2 I767M mutation (Non-Small Cell Lung Cancer), ERBB2 L755 mutation (Non-Small Cell Lung Cancer), ERBB2 L786V mutation (Non-Small Cell Lung Cancer), ERBB2 L869R mutation (Non-Small Cell Lung Cancer), ERBB2 R678Q mutation (Non-Small Cell Lung Cancer), ERBB2 S310F mutation (Non-Small Cell Lung Cancer), ERBB2 S310Y mutation (Non-Small Cell Lung Cancer), ERBB2 T733I mutation (Non-Small Cell Lung Cancer), ERBB2 T798I mutation (Unspecified Solid Tumor), ERBB2 T798M mutation (Unspecified Solid Tumor), ERBB2 T862I mutation (Non-Small Cell Lung Cancer), ERBB2 V659E mutation (Non-Small Cell Lung Cancer), ERBB2 V697L mutation (Non-Small Cell Lung Cancer), ERBB2 V773M mutation (Non-Small Cell Lung Cancer), ERBB2 V777L mutation (Non-Small Cell Lung Cancer), ERBB2 V777M mutation (Non-Small Cell Lung Cancer), ERBB2 V842I mutation (Non-Small Cell Lung Cancer), ERBB2 amplification (Breast Cancer, Gastric Cancer), ERBB2 exon 20 insertion (Non-Small Cell Lung Cancer), ERBB2 overexpression (Breast Cancer, Gastric Cancer)

Phase: II

Therapy: poziotinib

Location: United States

US State: CA

Contact: Dr. Lyndah Dreiling [949-788-6700; lyndah.dreiling@sppirx.com]

EGFR A289T mutation (continued)

NCT02465060
Molecular Analysis for Therapy Choice (MATCH).

Cancer type: Unspecified Solid Tumor

Variant class: EGFR activating mutation

Other identifiers: 15-188, 15-7002, 16-750, 20150671, 275215-Z1E, AAAP9159, CTMS# 15-2082, CTSU/EAY131, EAY131, ECOG-ACRIN EAY131, MATCH, NCI-2015-00054, NCI-MATCH

Population segments: Adenocarcinoma, Aggressive, BRAF, BRCA, Classical, EGFR, FGFR, Fourth line or greater, HER2 positive, HRAS, Indolent, Large Cell, NRAS, Nodular lymphocyte-predominant, Pulmonary, Second line, Squamous Cell, Stage III, Stage IV, Third line, Unspecified

Exclusion criteria variant class: EGFR activating mutation (Glioblastoma, Non-Small Cell Lung Cancer, Small Cell Lung Cancer)

Phase: II

Therapy: afatinib

Locations: Guam, Puerto Rico, United States

US States: AK, AL, AR, AZ, CA, CO, CT, DC, DE, FL, GA, HI, IA, ID, IL, IN, KS, KY, LA, MA, MD, ME, MI, MN, MO, MS, MT, NC, ND, NE, NH, NJ, NV, NY, OH, OK, OR, PA, RI, SC, SD, TN, TX, UT, VA, VT, WA, WI, WV, WY

Contact: Multiple contacts: See www.clinicaltrials.gov for complete list of contacts.

NCT03810872
An Open Explorative Phase II, Open Label Study of Afatinib in the Treatment of Advanced Cancer Carrying an EGFR, a HER2 or a HER3 Mutation

Cancer type: Unspecified Cancer

Variant class: EGFR activating mutation

Other identifiers: 1200.264, 1200264, EudraCT Number: 2016-003411-34, Precision 2, Precision 2 - 1200.264, Precision 2 - Afatinib

Population segments: EGFR, HER2 positive, Second line, Squamous Cell, Stage III, Stage IV

Phase: II

Therapy: afatinib

Location: Belgium

NCT04591431
The Rome Trial From Histology to Target: the Road to Personalize Target Therapy and Immunotherapy

Cancer type: Biliary Tract Carcinoma, Breast Cancer, Colorectal Cancer, Esophageal Cancer, Gastric Cancer, Gastroesophageal Junction Adenocarcinoma, Hepatocellular Carcinoma, Liver Cancer, Non-Small Cell Lung Cancer, Pancreatic Cancer, Unspecified Solid Tumor

Variant class: EGFR mutation

Other identifiers: EudraCT Number: 2018-002190-21, MAR-BAS-18-005, ROME

Population segments: Second line, Stage IV

Phase: II

Therapy: erlotinib

Location: Italy

Disclaimer: The data presented here is from a curated knowledgebase of publicly available information, but may not be exhaustive. The data version is 2021.11(003).

EGFR A289T mutation (continued)

NCT03239015
Efficacy and Safety of Targeted Precision Therapy in Refractory Tumor With Druggable Molecular Event

Cancer type: Unspecified Solid Tumor

Variant class: EGFR mutation

Other identifier: HETIAN64

Population segments: (N/A), Second line

Phase: II

Therapies: gefitinib, erlotinib, afatinib

Location: China

NCT03065387
Phase I Study of the Pan-ERBB Inhibitor Neratinib Given in Combination With Everolimus, Palbociclib, or Trametinib in Advanced Cancer Subjects With EGFR Mutation/Amplification, HER2 Mutation/Amplification, or HER3/4 Mutation or KRAS Mutation

Cancer type: Unspecified Solid Tumor

Variant class: EGFR mutation

Other identifiers: 2016-0430, NCI-2018-01218

Population segments: EGFR, HER2 negative, HER2 positive, Second line, Stage III, Stage IV

Phase: I

Therapies: neratinib, palbociclib, everolimus, trametinib

Location: United States

US State: TX

Contact: Dr. Sarina Piha-Paul [713-563-1930; spihapau@mdanderson.org]

No NCT ID - see other identifier(s)
Phase I Clinical Study With Advanced Solid Tumors KBP-5209 Treatment

Cancer type: Unspecified Solid Tumor

Variant class: EGFR mutation

Other identifiers: 5209-CPK-1002, CTR20150792

Population segments: EGFR, HER2 positive, Second line, Stage III, Stage IV

Phase: I

Therapy: pirotinib

Location: China

NCT04128085
A Phase I, Open-label, Multicenter, Dose Escalation and Expansion Study to Evaluate the Tolerance and Pharmacokinetics of TQB3804 in Subjects With Advanced Malignant Tumors

Cancer type: Unspecified Solid Tumor

Variant class: EGFR mutation

Other identifiers: CTR20192284, TQB3804-I-01

Population segments: EGFR, First line, Second line, Stage III, Stage IV

Phase: I

Therapy: TQB 3804

Location: China

Disclaimer: The data presented here is from a curated knowledgebase of publicly available information, but may not be exhaustive. The data version is 2021.11(003).

EGFR A289T mutation (continued)

NCT03841110
FT500 as Monotherapy and in Combination With Immune Checkpoint Inhibitors in Subjects With Advanced Solid Tumors (Phase I)

Cancer type: Unspecified Solid Tumor

Variant class: EGFR positive

Other identifiers: 2018-0635, FT500-101, NCI-2019-01692

Population segments: Aggressive, Classical, EGFR, Extensive, Fourth line or greater, HER2 positive, Indolent, Merkel, Nodular lymphocyte-predominant, Pulmonary, Second line, Stage III, Stage IV, Third line

Phase: I

Therapies: FT500, nivolumab, pembrolizumab, atezolizumab

Location: United States

US States: CA, MN, NJ, TX

Contact: Kimberly Musni [858-875-1800; clinical@fatetherapeutics.com]

NCT04412616
A Phase I, Multicenter, Open-label Study to Evaluate the Safety, Tolerability, Pharmacokinetics, Immunogenicity, and Preliminary Evidence of Antitumor Activity of ZZ06 in Adult Patients With Advanced EGFR Positive Solid Tumor Malignancies

Cancer type: Unspecified Solid Tumor

Variant class: EGFR positive

Other identifiers: 8379643, CTR20210099, NCI-2021-00829, ZZ06-2020A

Population segments: EGFR, Second line, Stage III, Stage IV

Phase: I

Therapy: ZZ06

Location: United States

US States: CA, KS, NY

Contact: Shiqi Bai [894-364-2700; baishiqi@intelli-crown.com]

NCT03297606
Canadian Profiling and Targeted Agent Utilization Trial (CAPTUR): A Phase II Basket Trial

Cancer type: Unspecified Solid Tumor

Variant class: EGFR aberration

Other identifiers: CA209-9DL, CAPTUR, ESR-17-12831, ML39800, PM1, WI233446

Population segments: Aggressive, Diffuse large B-cell lymphoma (DLBCL), Extranodal marginal zone B-cell lymphoma (MALT), First line, Follicular lymphoma (FL), Indolent, Lymphoblastic lymphoma (LBL), Mantle cell lymphoma (MCL), Other subtype, Second line, Stage III, Stage IV, Waldenstrom's macroglobulinemia (WM)

Phase: II

Therapy: erlotinib

Location: Canada

Disclaimer: The data presented here is from a curated knowledgebase of publicly available information, but may not be exhaustive. The data version is 2021.11(003).

EGFR A289T mutation (continued)

NCT04429542
First-in-Human, Phase I/Ib, Open-label, Multicenter Study of Bifunctional EGFR/TGFß Fusion Protein BCA101 Alone and in Combination With Pembrolizumab in Patients With EGFR-Driven Advanced Solid Tumors

Cancer type: Gastric Cancer, Glioblastoma, Head and Neck Cancer, Hepatocellular Carcinoma, Non-Small Cell Lung Cancer, Ovarian Cancer, Pancreatic Cancer, Rectal Cancer, Thyroid Gland Anaplastic Carcinoma, Triple Negative Breast Cancer, Unspecified Solid Tumor, Uveal Melanoma

Variant class: EGFR aberration

Other identifiers: 20-147, 2020-0153, BCA101X1101, NCI-2020-04796

Population segments: Anaplastic, EGFR, Fourth line or greater, HER2 negative, KRAS, Second line, Stage III, Stage IV, Third line, Triple receptor negative

Phase: I

Therapy: BCA101

Locations: Canada, United States

US States: MA, NY, PA, TX

Contact: David Bohr [617-800-0335; info@bicara.com]

NCT04511533
Single Arm Study to Evaluate the Safety of Dacomitinib for the First-Line Treatment of Participants in India With Metastatic Non-Small Cell Lung Cancer With Epidermal Growth Factor Receptor (EGFR)-Activating Mutations

Cancer type: Non-Small Cell Lung Cancer

Variant class: EGFR activating mutation

Other identifiers: A7471064, CTRI/2020/07/026825

Population segments: ALK, CNS mets, First line, Stage IV

Phase: IV

Therapy: dacomitinib

Location: India

No NCT ID - see other identifier(s)
The Continuous Evaluation of EGFR Mutation in EGFR-mutation Positive Lung Cancer Patients During EGFR TKI Treatment.

Cancer type: Non-Small Cell Lung Cancer

Variant class: EGFR activating mutation

Other identifier: UMIN000037232

Population segments: EGFR, Line of therapy N/A, N/A

Phase: IV

Therapy: EGFR tyrosine kinase inhibitor

Location: Japan

NCT03991403
Study of Atezolizumab in Combination With Carboplatin + Paclitaxel +Bevacizumab vs With Pemetrexed + Cisplatin or Carboplatin With Stage IV Non-Squamous Non-Small Cell Lung Cancer with EGFR (+) or ALK (+)

Cancer type: Non-Small Cell Lung Cancer

Variant class: EGFR activating mutation

Other identifiers: 2019-03-027, ATLAS Trial, KCT0004043

Population segments: ALK, Adenocarcinoma, EGFR, Large Cell, Maintenance/Consolidation, Second line, Stage III, Stage IV

Phase: III

Therapies: atezolizumab, bevacizumab, chemotherapy

Location: Republic of Korea

Disclaimer: The data presented here is from a curated knowledgebase of publicly available information, but may not be exhaustive. The data version is 2021.11(003).

EGFR A289T mutation (continued)

NCT03088059
A Pilot Study of Personalized Biomarker-based Treatment Strategy or Immunotherapy in Patients With Recurrent/Metastatic Squamous Cell Carcinoma of the Head and Neck

Cancer type: Head and Neck Cancer

Variant class: EGFR activating mutation

Other inclusion criteria: CDKN2A underexpression, HRAS wild type, KRAS wild type, NRAS wild type

Other identifiers: 1559-HNCG, EORTC HN1559, EORTC-1559-HNCG, EORTC-HNCG-1559, EudraCT Number: 2017-000086-74, IRAS ID 236404, RECF3462, UPSTREAM

Population segments: EGFR, HER2 positive, Second line, Stage IV

Phase: II

Therapy: afatinib

Locations: Belgium, France, Italy, United Kingdom

NCT04147351
A Phase II Study of Atezolizumab in Combination With Bevacizumab, Carboplatin or Cisplatin, and Pemetrexed for EGFR-mutant Metastatic Non-small Cell Lung Cancer Patients After Failure of EGFR Tyrosine Kinase Inhibitors.

Cancer type: Non-Small Cell Lung Cancer

Variant class: EGFR activating mutation

Other identifier: 201908090MIFA

Population segments: EGFR, Maintenance/Consolidation, Second line, Stage III, Stage IV

Exclusion criteria variant classes: EGFR T790M mutation (Non-Small Cell Lung Cancer), EGFR exon 20 insertion (Non-Small Cell Lung Cancer)

Phase: II

Therapies: atezolizumab, bevacizumab, chemotherapy

Location: Taiwan

NCT04811001
A Randomised Non-comparative, Phase II Study Investigating the Best Epidermal Growth Factor Receptor Tyrosine Kinase Inhibitor (EGFR-TKI) Sequence in Advanced or Metastatic Non Small-Cell Lung Cancer (NSCLC) Harboring EGFR Mutations

Cancer type: Non-Small Cell Lung Cancer

Variant class: EGFR activating mutation

Other inclusion criteria: ALK fusion negative, BRAF mutation negative, ERBB2 mutation negative, KRAS mutation negative, MET mutation negative, ROS1 fusion negative

Other identifier: CAPLAND

Population segments: EGFR, First line, Second line, Stage III, Stage IV

Phase: II

Therapies: dacomitinib, osimertinib

Location: Italy

Disclaimer: The data presented here is from a curated knowledgebase of publicly available information, but may not be exhaustive. The data version is 2021.11(003).

EGFR A289T mutation (continued)

NCT04484142
Phase II, Single-arm, Open-label Study of DS-1062a in Advanced or Metastatic Non-small Cell Lung Cancer With Actionable Genomic Alterations and Progressed On or After Applicable Targeted Therapy and Platinum Based Chemotherapy (TROPION-Lung05)

Cancer type: Non-Small Cell Lung Cancer

Variant class: EGFR activating mutation

Other identifiers: 20-602, DS1062-A-U202, EudraCT Number: 2020-002774-27, jRCT2041200097, TROPION-Lung05

Population segments: ALK, BRAF, EGFR, Fourth line or greater, Second line, Stage III, Stage IV, Third line

Exclusion criteria variant class: KRAS mutation (Non-Small Cell Lung Cancer)

Phase: II

Therapy: datopotamab deruxtecan

Locations: France, Hungary, Italy, Japan, Netherlands, Republic of Korea, Spain, Taiwan, United States

US States: CA, FL, MA, MD, MI, MO, NJ, NY, OH, TN, TX, VA, WA

Contact: (US sites only) Daiichi Sankyo Contact for Clinical Trial Information [908-992-6400; CTRinfo@dsi.com]

No NCT ID - see other identifier(s)
ITAC 2 TRIAL: Intermittent TKI and Chemotherapy for Patients with Advanced Non-Small Cell Lung Cancer

Cancer type: Non-Small Cell Lung Cancer

Variant class: EGFR activating mutation

Other identifiers: EudraCT Number: 2010-023362-44, ITAC, ITAC 2

Population segments: Adenocarcinoma, EGFR, First line, Large Cell, Maintenance/Consolidation, Stage III, Stage IV

Phase: II

Therapies: erlotinib, chemotherapy

Location: Slovenia

NCT03151161
A Prospective,Multi-center, Open-labeled Phase II Randomized and Comparative Clinical Study of First Line Intermittent and Maintenance of Icotinib in Combination With Pemetrexed/Carboplatin Compared With Icotinib Single Drug in IIIB/IV Non Small Cell Lung Cancer With Epidermal Growth Factor Receptor (EGFR) Mutation

Cancer type: Non-Small Cell Lung Cancer

Variant class: EGFR activating mutation

Other identifier: 201512001

Population segments: Adenocarcinoma, EGFR, First line, Large Cell, Maintenance/Consolidation, Stage III, Stage IV

Phase: II

Therapies: icotinib hydrochloride, chemotherapy

Location: China

Disclaimer: The data presented here is from a curated knowledgebase of publicly available information, but may not be exhaustive. The data version is 2021.11(003).

EGFR A289T mutation (continued)

NCT03292133
A Phase II Study of EGF816 and Gefitinib in TKI-naive EGFR-mutant Non-Small Cell Lung Cancer

Cancer type: Non-Small Cell Lung Cancer

Variant class: EGFR activating mutation

Other identifiers: 17-291, NCI-2017-02352

Population segments: EGFR, Second line, Stage I, Stage II, Stage III, Stage IV

Phase: II

Therapies: nazartinib, gefitinib

Location: United States

US State: MA

Contact: Beth Kennedy [617-724-1223; EAKENNEDY@mgh.harvard.edu]

NCT04335292
Osimertinib Then Chemotherapy in EGFR-mutated Lung Cancer With Osimertinib Third-line Rechallenge

Cancer type: Non-Small Cell Lung Cancer

Variant class: EGFR activating mutation

Other inclusion criteria: EGFR exon 20 insertion negative

Other identifier: OCELOT

Population segments: EGFR, Stage III, Stage IV, Third line

Phase: II

Therapy: osimertinib

Location: Canada

NCT04410796
A Phase II Randomized Study of Osimertinib Versus Osimertinib Plus Chemotherapy for Patients With Metastatic EGFR-Mutant Lung Cancers That Have Detectable EGFR-Mutant cfDNA in Plasma After Initiation of Osimertinib

Cancer type: Non-Small Cell Lung Cancer

Variant class: EGFR activating mutation

Other identifiers: 20-011, NCI-2020-04069

Population segments: EGFR, First line, Second line, Stage IV

Phase: II

Therapies: osimertinib, chemotherapy

Location: United States

US States: MD, NJ, NY, TN

Contact: Dr. Helena Yu [646-608-2252; yuh@mskcc.org]

Disclaimer: The data presented here is from a curated knowledgebase of publicly available information, but may not be exhaustive. The data version is 2021.11(003).

EGFR A289T mutation (continued)

NCT03318939
A Phase II Study of Poziotinib in Patients With Non-Small Cell Lung Cancer (NSCLC), Locally Advanced or Metastatic, With EGFR or HER2 Exon 20 Insertion Mutation (ZENITH20).

Cancer type: Non-Small Cell Lung Cancer

Variant class: EGFR activating mutation

Other identifiers: 18-059, 19387, 2017-0857, 20172584, EudraCT Number: 2018-001868-36, NCI-2018-00078, NL66741.078.18, POZITIVE20-1, SPECTRUM20, SPI-POZ-202, UCI-17-101, ZENITH20, ZENITH20-1

Population segments: EGFR, First line, Fourth line or greater, HER2 positive, Second line, Stage III, Stage IV, Third line

Exclusion criteria variant classes: EGFR C797S mutation (Non-Small Cell Lung Cancer), EGFR G779F mutation (Non-Small Cell Lung Cancer), EGFR G796A mutation (Non-Small Cell Lung Cancer), EGFR T790M mutation (Non-Small Cell Lung Cancer), EGFR V769L mutation (Non-Small Cell Lung Cancer), EGFR V774M mutation (Non-Small Cell Lung Cancer)

Phase: II

Therapy: poziotinib

Locations: Belgium, Canada, Israel, Italy, Netherlands, United States

US States: AZ, CA, CT, DC, FL, GA, IL, MA, MD, MI, MN, NC, NY, OH, TN, TX, VA, WA

Contact: Dr. Lyndah Dreiling [949-788-6700; spi-poz-202@sppirx.com]

NCT04862780
A Phase I/II Study Targeting Acquired Resistance Mechanisms in Patients With EGFR Mutant Non-Small Cell Lung Cancer

Cancer type: Non-Small Cell Lung Cancer

Variant class: EGFR activating mutation

Other identifiers: 2021-0267, BLU-945-1101

Population segments: Adenocarcinoma, EGFR, Second line, Stage IV

Exclusion criteria variant classes: ALK aberration (Non-Small Cell Lung Cancer), BRAF V600E mutation (Non-Small Cell Lung Cancer), EGFR exon 20 mutation (Non-Small Cell Lung Cancer), ERBB2 aberration (Non-Small Cell Lung Cancer), KRAS aberration (Non-Small Cell Lung Cancer), MET aberration (Non-Small Cell Lung Cancer), NTRK1 aberration (Non-Small Cell Lung Cancer), NTRK2 aberration (Non-Small Cell Lung Cancer), NTRK3 aberration (Non-Small Cell Lung Cancer), RET aberration (Non-Small Cell Lung Cancer), ROS1 aberration (Non-Small Cell Lung Cancer)

Phase: I/II

Therapy: BLU-945

Location: United States

US States: CA, MA, NY, TN, TX

Contact: Blueprint Medicines [617-714-6707; medinfo@blueprintmedicines.com]

No NCT ID - see other identifier(s)
Phase I/II Study of Brigatinib plus Panitumumab in Patients with Advanced EGFR-mutated Non-small Cell Lung Cancer Harboring C797S Resistant Mutation to Osimertinib

Cancer type: Non-Small Cell Lung Cancer

Variant class: EGFR activating mutation

Other identifiers: BEBOP, jRCT2031200231

Population segments: EGFR, Second line, Stage III, Stage IV

Phase: I/II

Therapies: brigatinib, panitumumab

Location: Japan

Disclaimer: The data presented here is from a curated knowledgebase of publicly available information, but may not be exhaustive. The data version is 2021.11(003).

EGFR A289T mutation (continued)

NCT02716116
A Phase I/II Study of the Safety, Pharmacokinetics, and Anti-Tumor Activity of the Oral EGFR/HER2 Inhibitor TAK-788 (AP32788) in Non-Small Cell Lung Cancer.

Cancer type: Biliary Tract Carcinoma, Bladder Urothelial Carcinoma, Breast Cancer, Esophageal Cancer, Gastric Cancer, Head and Neck Cancer, Non-Small Cell Lung Cancer

Variant class: EGFR activating mutation

Other identifiers: 16-0208, 16-143, 16-447, 2016-1030, AP32788-15-101, EudraCT Number: 2016-001271-68, IRAS ID 257847, JapicCTI-195000, LUN0082, NCI-2016-00587, RECF3968, SHS_492405 PARM CARI 17177, TAK15101-A15101, Takeda EXCLAIM, U1111-1217-7205, UCI-17-83, VICCTHO1614

Population segments: CNS mets, EGFR, First line, Fourth line or greater, HER2 positive, Second line, Stage III, Stage IV, Third line

Phase: I/II

Therapies: mobocertinib, chemotherapy

Location: United States

US States: AZ, CA, FL, GA, NC, VA

Contact: Takeda Contact [877-825-3327; medinfoUS@takeda.com]

NCT04486833
A Phase I/II Open-Label, Dose-escalation and Clinical Response Study of GPX-001 in Combination With Osimertinib in Patients With Advanced, Metastatic EGFR-mutant Non-small Cell Lung Cancer

Cancer type: Non-Small Cell Lung Cancer

Variant class: EGFR activating mutation

Other identifiers: Acclaim-1, GEN-104

Population segments: EGFR, Second line, Stage III, Stage IV

Phase: I/II

Therapies: quaratusugene ozeplasmid, osimertinib

Location: United States

US State: TX

Contact: VP of Clinical Operations [877-774-4679; sinman@genprex.com]

No NCT ID - see other identifier(s)
A Phase I Study Afatinib In Combination Of Osimertinib In Patients With Relapsed Non-Small Cell Lung Cancer After Failure of Prior Osimertinib

Cancer type: Non-Small Cell Lung Cancer

Variant class: EGFR activating mutation

Other identifiers: AfaOsi trial, jRCTs051180008, UMIN000031501

Population segments: EGFR, Second line, Stage III, Stage IV

Phase: I

Therapies: afatinib, osimertinib

Location: Japan

Disclaimer: The data presented here is from a curated knowledgebase of publicly available information, but may not be exhaustive. The data version is 2021.11(003).

EGFR A289T mutation (continued)

NCT03755102
A Pilot Study of Dacomitinib With or Without Osimertinib for Patients With Metastatic EGFR Mutant Lung Cancers With Disease Progressionon Osimertinib

Cancer type: Non-Small Cell Lung Cancer

Variant class: EGFR activating mutation

Other identifiers: 18-341, NCI-2018-03226

Population segments: EGFR, Second line, Stage IV

Exclusion criteria variant classes: ALK fusion (Non-Small Cell Lung Cancer), MET amplification (Non-Small Cell Lung Cancer), RET fusion (Non-Small Cell Lung Cancer)

Phase: I

Therapy: dacomitinib

Location: United States

US States: NJ, NY

Contact: Dr. Helen Yu [646-888-4274; YuH@mskcc.org]

NCT04762199
A Phase Ib Safety and Pharmacodynamic Study of MER Tyrosine Kinase Inhibitor, MRX-2843, in Combination With Osimertinib in Advanced EGFR Mutant Non-Small Cell Lung Cancer

Cancer type: Non-Small Cell Lung Cancer

Variant class: EGFR activating mutation

Other inclusion criteria: EGFR T790M negative

Other identifiers: NCI-2020-08392, STUDY00001681, WINSHIP5153-20

Population segments: EGFR, Second line, Stage IV

Phase: I

Therapies: MRX-2843, osimertinib

Location: United States

US State: GA

Contact: Dr. Taofeek Owonikoko [404-778-4576; towonik@emory.edu]

NCT03891615
Phase I Study of Niraparib in Combination With Osimertinib in EGFR-Mutated Advanced Lung Cancer

Cancer type: Non-Small Cell Lung Cancer

Variant class: EGFR activating mutation

Other identifiers: 18-613, NCI-2019-03870

Population segments: EGFR, Line of therapy N/A, Stage IV

Phase: I

Therapies: niraparib, osimertinib

Location: United States

US State: MA

Contact: Dr. Zofia Piotrowska [617-643-9707; zofia.piotrowska@mgh.harvard.edu]

Disclaimer: The data presented here is from a curated knowledgebase of publicly available information, but may not be exhaustive. The data version is 2021.11(003).

EGFR A289T mutation (continued)

NCT04250545

A Phase I Trial of MLN0128 (Sapanisertib) and CB-839 HCl (Telaglenastat) in Advanced NSCLC Patients

Cancer type: Non-Small Cell Lung Cancer

Variant class: EGFR activating mutation

Other identifiers: 10327, NCI 10327, NCI-2020-00478, PHI-113

Population segments: ALK, Adenocarcinoma, BRAF, EGFR, KRAS, Large Cell, Second line, Stage III, Stage IV

Phase: I

Therapies: telaglenastat, sapanisertib

Location: United States

US States: CA, NY

Contact: Multiple contacts: See www.clinicaltrials.gov for complete list of contacts.

NCT03114319

An Open-label, Multi-center, Phase I, Dose Finding Study of Oral TNO155 in Adult Patients With Advanced Solid Tumors.

Cancer type: Non-Small Cell Lung Cancer

Variant class: EGFR activating mutation

Other inclusion criteria: BRAF wild type, RAS wild type

Other identifiers: 17-251, 17-266, CTNO155X2101, EudraCT Number: 2016-001861-10, JapicCTI-184236, NCI-2017-01726, NL60195.056.16, TNO155X2101

Population segments: EGFR, Second line, Stage III, Stage IV

Exclusion criteria variant classes: BRAF activating mutation (Unspecified Solid Tumor), HRAS activating mutation (Unspecified Solid Tumor), KRAS activating mutation (Unspecified Solid Tumor), NRAS activating mutation (Unspecified Solid Tumor), PTPN11 activating mutation (Unspecified Solid Tumor)

Phase: I

Therapies: TNO-155, nazartinib

Locations: Canada, Japan, Netherlands, Republic of Korea, Singapore, Spain, Taiwan, United States

US States: MA, NY, TN

Contact: Novartis Pharmaceuticals [888-669-6682; trialandresults.registries@novartis.com]

Disclaimer: The data presented here is from a curated knowledgebase of publicly available information, but may not be exhaustive. The data version is 2021.11(003).

EGFR A289T mutation (continued)

NCT04197934
Phase I Study to Evaluate Safety, Tolerability, Pharmacokinetics and Anti-Tumor Activity of WSD0922-FUFU

Cancer type: Non-Small Cell Lung Cancer

Variant class: EGFR activating mutation

Other identifiers: MC1914, NCI-2019-07825

Population segments: EGFR, First line, Second line, Stage IV

Exclusion criteria variant classes: EGFR C797S mutation & EGFR L858R mutation & EGFR T790M mutation (Anaplastic Astrocytoma IDH-wild type, Glioblastoma IDH-wild type, Non-Small Cell Lung Cancer), EGFR C797S mutation & EGFR T790M mutation & EGFR exon 19 deletion (Anaplastic Astrocytoma IDH-wild type, Glioblastoma IDH-wild type, Non-Small Cell Lung Cancer), EGFR L858R mutation & EGFR T790M mutation (Anaplastic Astrocytoma IDH-wild type, Glioblastoma IDH-wild type, Non-Small Cell Lung Cancer), EGFR T790M mutation & EGFR exon 19 deletion (Anaplastic Astrocytoma IDH-wild type, Glioblastoma IDH-wild type, Non-Small Cell Lung Cancer)

Phase: I

Therapy: WSD-0922

Location: United States

US States: AZ, FL, MN

Contact: Multiple contacts: See www.clinicaltrials.gov for complete list of contacts.

NCT04413201
AFAMOSI: Prospective, Randomized, Multicenter Phase IV Study to Evaluate the Efficacy and Safety of Afatinib Followed by Osimertinib Compared to Osimertinib in Patients With EGFRmutated/T790M Mutation Negative Non-squamous NSCLC in the First-line Setting

Cancer type: Non-Small Cell Lung Cancer

Variant class: EGFR mutation

Other inclusion criteria: EGFR T790M negative

Other identifiers: AFAMOSI, EudraCT Number: 2019-002197-31

Population segments: Adenocarcinoma, EGFR, First line, Large Cell, Stage III, Stage IV

Exclusion criteria variant class: EGFR T790M mutation (Non-Small Cell Lung Cancer)

Phase: IV

Therapies: afatinib, osimertinib

Location: Germany

No NCT ID - see other identifier(s)
Apatinib Combined With EGFR-TKI For Patients With EGFR Mutation Who Failed EGFR-TKI: A Prospective Study

Cancer type: Non-Small Cell Lung Cancer

Variant class: EGFR mutation

Other identifier: ChiCTR1800017863

Population segments: EGFR, Second line, Stage IV

Phase: IV

Therapy: apatinib + EGFR tyrosine kinase inhibitor

Location: China

Disclaimer: The data presented here is from a curated knowledgebase of publicly available information, but may not be exhaustive. The data version is 2021.11(003).

EGFR A289T mutation (continued)

No NCT ID - see other identifier(s)
A Real World Study Of Apatinib Combined With Gefitinib In The Treatment Of EGFRm+ Advanced Non-Squamous Non-Small Cell Lung Cancer

Cancer type: Non-Small Cell Lung Cancer

Variant class: EGFR mutation

Other identifier: ChiCTR2000040093

Population segments: Adenocarcinoma, EGFR, First line, Large Cell, Stage III, Stage IV

Phase: IV

Therapies: apatinib, gefitinib

Location: China

No NCT ID - see other identifier(s)
Clinical Study Of Combined Action Of Gefitinib And Brain Radiotherapy On EGFR-Mutated Non-Small-Cell Lung Cancer Patients With Brain Metastasis

Cancer type: Non-Small Cell Lung Cancer

Variant class: EGFR mutation

Other identifier: ChiCTR-OOC-17012107

Population segments: CNS mets, EGFR, Line of therapy N/A, N/A

Phase: IV

Therapies: gefitinib, radiation therapy

Location: China

No NCT ID - see other identifier(s)
A Multicenter, Open-Label, Single Arm Pilot Study: Osimertinib As Neoadjuvant Treatment For Resectable Stage II-IIIA EGFR Mutant Lung Adenocarcinoma.

Cancer type: Non-Small Cell Lung Cancer

Variant class: EGFR mutation

Other identifiers: ChiCTR1800016948, NEOS

Population segments: Adenocarcinoma, EGFR, Neoadjuvant, Stage II, Stage III

Exclusion criteria variant class: EGFR exon 20 insertion (Non-Small Cell Lung Cancer)

Phase: IV

Therapy: osimertinib

Location: China

NCT03992885
Combination Therapy With Icotinib, Pemetrexed and Platinum in Patients With Metastatic Non-squamous Non-small Cell Lung Cancer With EGFR Mutations Who Did Not Progress After Pemetrexed in Combination With Platinum-based Chemotherapy:a Single-arm, Open, Multicenter Clinical Study.

Cancer type: Non-Small Cell Lung Cancer

Variant class: EGFR mutation

Other identifier: E2019161

Population segments: Adenocarcinoma, EGFR, Large Cell, Maintenance/Consolidation, Second line, Stage IV

Phase: III

Therapies: icotinib hydrochloride, chemotherapy

Location: China

Disclaimer: The data presented here is from a curated knowledgebase of publicly available information, but may not be exhaustive. The data version is 2021.11(003).

EGFR A289T mutation (continued)

No NCT ID - see other identifier(s)
A Phase II Study Of Afatinib For Advanced Non-Small Cell Lung Cancer With Uncommon Epidermal Growth Factor Receptor (EGFR) Mutation Including Compound Mutation Detected By Next Generation Sequencing

Cancer type: Non-Small Cell Lung Cancer

Variant class: EGFR mutation

Other identifier: jRCTs041190027

Population segments: EGFR, First line, Stage III, Stage IV

Exclusion criteria variant classes: EGFR T790M mutation (Non-Small Cell Lung Cancer), EGFR exon 20 insertion (Non-Small Cell Lung Cancer)

Phase: II

Therapy: afatinib

Location: Japan

NCT04785742
An Open, Multicenter Phase II Clinical Study to Evaluate the Safety and Efficacy of Almonertinib in Patients With Advanced NSCLC With Rare Mutations in EGFR

Cancer type: Non-Small Cell Lung Cancer

Variant class: EGFR mutation

Other inclusion criteria: EGFR exon 19 deletion negative, EGFR exon 20 insertion negative, EGFR L858R mutation negative

Other identifier: YX-L-202008

Population segments: EGFR, First line, Second line, Stage III, Stage IV

Exclusion criteria variant classes: EGFR L858R mutation (Non-Small Cell Lung Cancer), EGFR exon 19 deletion (Non-Small Cell Lung Cancer)

Phase: II

Therapy: almonertinib

Location: China

No NCT ID - see other identifier(s)
Safety and efficacy analysis of Ametinib combined with Pemetrexed intraoperatively for treatment of EGFR mutant lung cancer with meningeal metastases after first-line TKI failure

Cancer type: Lung Cancer

Variant class: EGFR mutation

Other identifier: ChiCTR2100047017

Population segments: Adenocarcinoma, CNS mets, EGFR, Second line, Stage IV

Phase: II

Therapies: almonertinib, chemotherapy

Location: China

No NCT ID - see other identifier(s)
A Single-center, Open Label, Phase II Study of Anlotinib as Second/Third-line Treatment for Advanced Non-small Cell Lung Cancer

Cancer type: Non-Small Cell Lung Cancer

Variant class: EGFR mutation

Other inclusion criteria: EGFR T790M negative

Other identifier: ChiCTR1800017585

Population segments: EGFR, Second line, Stage III, Stage IV, Third line

Phase: II

Therapy: anlotinib hydrochloride

Location: China

Disclaimer: The data presented here is from a curated knowledgebase of publicly available information, but may not be exhaustive. The data version is 2021.11(003).

EGFR A289T mutation (continued)

NCT04619563
A Single-arm Exploratory Clinical Study of Anlotinib Hydrochloride Combined With Docetaxel in EGFR Mutations Advanced Non Small Cell Lung Cancer Patients Who Have Progressed After Targeted Therapy and Chemotherapy

Cancer type: Non-Small Cell Lung Cancer

Variant class: EGFR mutation

Other inclusion criteria: ALK mutation negative

Other identifier: SDZLEC2020-052-02

Population segments: Adenocarcinoma, EGFR, Large Cell, Maintenance/Consolidation, Second line, Stage III, Stage IV

Phase: II

Therapies: anlotinib hydrochloride, chemotherapy

Location: China

No NCT ID - see other identifier(s)
Clinical Study And Safety Analysis On The Treatment Of Advanced Non-Small Cell Lung Cancer With Anlotinib And Gefitinib

Cancer type: Non-Small Cell Lung Cancer

Variant class: EGFR mutation

Other identifier: ChiCTR2000030525

Population segments: EGFR, Second line, Stage II, Stage III, Stage IV

Phase: II

Therapies: anlotinib hydrochloride, gefitinib

Location: China

NCT04245085
A Randomised Non-comparative Open Label Phase II Trial of Atezolizumab Plus Bevacizumab, With Carboplatin-paclitaxel or Pemetrexed, in EGFR-mutant Non-small Cell Lung Carcinoma With Acquired Resistance

Cancer type: Non-Small Cell Lung Cancer

Variant class: EGFR mutation

Other identifiers: ABC-lung, EOTP-ABC, ETOP 15-19, ETOP 15-19 ABC-lung, ETOP_15-19, EudraCT Number: 2019-001687-30, KCT0005588, MO40586, NCC2020-0267

Population segments: Adenocarcinoma, EGFR, First line, Large Cell, Second line, Stage III, Stage IV, Third line

Phase: II

Therapies: atezolizumab, bevacizumab, chemotherapy

Locations: Germany, Singapore, Spain, Switzerland

No NCT ID - see other identifier(s)
Osimertinib Combined Bevacizumab in Untreated Epidermal Growth Factor Receptor Mutated Non-small-cell Lung Cancer Patients with Malignant Pleural And/Or Pericardial Effusion -phase II Trial

Cancer type: Non-Small Cell Lung Cancer

Variant class: EGFR mutation

Other identifiers: SPIRAL II, UMIN000028071

Population segments: Adenocarcinoma, EGFR, First line, Large Cell, Stage IV

Phase: II

Therapies: bevacizumab, osimertinib

Location: Japan

Disclaimer: The data presented here is from a curated knowledgebase of publicly available information, but may not be exhaustive. The data version is 2021.11(003).

EGFR A289T mutation (continued)

No NCT ID - see other identifier(s)
Randomized Controlled Trial for EGFR-TKIs Plus S-1 or EGFR-TKIs as the First-Line Therapy for Patients with Advanced Non-small Cell Lung Cancer Harboring EGFR Mutations

Cancer type: Non-Small Cell Lung Cancer

Variant class: EGFR mutation

Other identifier: ChiCTR1900020520

Population segments: EGFR, First line, Stage III, Stage IV

Phase: II

Therapies: EGFR tyrosine kinase inhibitor + chemotherapy, EGFR tyrosine kinase inhibitor

Location: China

No NCT ID - see other identifier(s)
EGFR-TKI Combined With Stereotactic Body Radiation Therapy Versus TKI alone for Stage IV Oncogene-Driven Non-Small Cell Lung Cancer.

Cancer type: Non-Small Cell Lung Cancer

Variant class: EGFR mutation

Other identifier: ChiCTR1900027030

Population segments: EGFR, Line of therapy N/A, Stage IV

Phase: II

Therapies: EGFR tyrosine kinase inhibitor, radiation therapy

Location: China

NCT03904823
An Open, Single-arm, Multi-center, Phase II Clinical Trial of Famitinib Combined With Epidermal Growth Factor Receptor (EGFR) Inhibitor HS-10296 in Patients With Advanced EGFR-mutant Non-Small Cell Lung Cancer (NSCLC)

Cancer type: Non-Small Cell Lung Cancer

Variant class: EGFR mutation

Other identifiers: CTR20190458, CTR20191893, FMTN-II-203-NSCLC

Population segments: EGFR, First line, Stage III, Stage IV

Phase: II

Therapies: famitinib, almonertinib

Location: China

NCT01784549
A Multi-center Phase II Randomized Study of Customized Neoadjuvant Therapy Versus Standard Chemotherapy in Non-small Cell Lung Cancer (NSLC) Patients With Resectable Stage IIIA (N2) Disease

Cancer type: Non-Small Cell Lung Cancer

Variant class: EGFR mutation

Other identifiers: CONTEST, CONTEST TRIAL, CONTEST TRIAL RF-2009-1530324, CONTEST-TRIAL, EudraCT Number: 2011-005267-24, RF-2009-1530324

Population segments: Adenocarcinoma, EGFR, Large Cell, Neoadjuvant, Other subtype, Squamous Cell, Stage III

Phase: II

Therapy: gefitinib

Location: Italy

Disclaimer: The data presented here is from a curated knowledgebase of publicly available information, but may not be exhaustive. The data version is 2021.11(003).

EGFR A289T mutation (continued)

No NCT ID - see other identifier(s)
Gefitinib Plus Pemetrexed Combined With Bevacizumab Or Carboplatin In First-LineTreatment Of Stage IV EGFR Mutant Non-Squamous Non-Small Cell Lung Cancer

Cancer type: Non-Small Cell Lung Cancer

Variant class: EGFR mutation

Other identifier: ChiCTR2100043395

Population segments: Adenocarcinoma, EGFR, First line, Large Cell, Stage IV

Phase: II

Therapies: gefitinib, bevacizumab, chemotherapy

Location: China

No NCT ID - see other identifier(s)
Open-Label, Single arm, Phase II trial to Evaluate Efficacy and Safety of Nivolumab as Maintenance Therapy Following Platinum-based Chemotherapy in Non-Small Cell Lung Cancer Patients after Tyrosine Kinase Inhibitor Therapy

Cancer type: Non-Small Cell Lung Cancer

Variant class: EGFR mutation

Other identifiers: EGFR_IO, KCT0004541

Population segments: ALK, EGFR, Maintenance/Consolidation, Second line, Stage III, Stage IV

Phase: II

Therapy: nivolumab

Location: Republic of Korea

NCT04538378
Phase II Trial of Olaparib (LYNPARZA) Plus Durvalumab (IMFINZI) in EGFR-Mutated Adenocarcinomas That Transform to Small Cell Lung Cancer (SCLC) and Other Neuroendocrine Tumors.

Cancer type: Non-Small Cell Lung Cancer

Variant class: EGFR mutation

Other identifiers: 200149, 20-C-0149, NCI-20-C-0149

Population segments: (N/A), Adenocarcinoma, EGFR, Pulmonary, Second line, Unspecified

Phase: II

Therapies: olaparib, durvalumab

Location: United States

US State: MD

Contact: Linda C. Sciuto [240-760-6117; lsciuto@mail.nih.gov]

No NCT ID - see other identifier(s)
Phase II trial of osimertinib for rare EGFR mutation-positive non-small cell lung cancer

Cancer type: Non-Small Cell Lung Cancer

Variant class: EGFR mutation

Other inclusion criteria: EGFR exon 19 deletion negative, EGFR exon 20 insertion negative, EGFR L858R mutation negative, EGFR T790M negative

Other identifiers: jRCTs071200002, TCOG-LC1901, UNICORN

Population segments: Adenocarcinoma, EGFR, First line, Stage III, Stage IV

Exclusion criteria variant classes: ALK fusion (Non-Small Cell Lung Cancer), BRAF mutation (Non-Small Cell Lung Cancer), EGFR L858R mutation (Non-Small Cell Lung Cancer), EGFR T790M mutation (Non-Small Cell Lung Cancer), EGFR exon 19 deletion (Non-Small Cell Lung Cancer), EGFR exon 20 insertion (Non-Small Cell Lung Cancer), ROS1 fusion (Non-Small Cell Lung Cancer)

Phase: II

Therapy: osimertinib

Location: Japan

Disclaimer: The data presented here is from a curated knowledgebase of publicly available information, but may not be exhaustive. The data version is 2021.11(003).

EGFR A289T mutation (continued)

NCT03460275
Osimertinib as First-line Therapy for Patients With EGFR Mutation-positive Locally Advanced or Metastatic Non-squamous Non-Small Cell Lung Cancer(NSCLC), a Single-Arm, Open-Label, Prospective, Multicenter, Phase II Clinical Trial

Cancer type: Non-Small Cell Lung Cancer

Variant class: EGFR mutation

Other identifier: A2018-001

Population segments: Adenocarcinoma, EGFR, First line, Large Cell, Stage III, Stage IV

Exclusion criteria variant class: EGFR exon 20 insertion (Non-Small Cell Lung Cancer)

Phase: II

Therapy: osimertinib

Location: China

No NCT ID - see other identifier(s)
Efficacy Of Osimertinib With Platinum And Pemetrexed In EGFR Mutant Non-Small Cell Lung Cancer Patients Bearing CNS Metastasis, And Have Systemic Progression But Stable Intracranial Disease On Osimertinib Resistance. (EPONA)

Cancer type: Non-Small Cell Lung Cancer

Variant class: EGFR mutation

Other identifiers: (EPONA), jRCTs071200029, TORG1938 (EPONA Study)

Population segments: Adenocarcinoma, CNS mets, EGFR, Large Cell, Second line, Stage IV

Phase: II

Therapies: osimertinib, chemotherapy

Location: Japan

NCT03087071
A Phase II Enrichment Study of Panitumumab as a Single Agent or in Combination With Trametinib in Anti-EGFR-Refractory Stage IV Colorectal Cancer Patients

Cancer type: Colorectal Cancer

Variant class: EGFR mutation

Other identifiers: 2016-0338, NCI-2017-00868

Population segments: Second line, Stage IV

Phase: II

Therapy: panitumumab

Location: United States

US State: TX

Contact: Christine Parseghian [713-795-9280; cparseghian@mdanderson.org]

No NCT ID - see other identifier(s)
An Exploratory Clinical Study Of PD-1 Inhibitor Combined With Chemotherapy In The Treatment Of Advanced Non-small Cell Lung Cancer With EGFR Mutation Positive And T790M Negative After Failure Of TKI Combined With Antiangiogenic Drugs

Cancer type: Non-Small Cell Lung Cancer

Variant class: EGFR mutation

Other inclusion criteria: EGFR T790M negative

Other identifier: ChiCTR2000039514

Population segments: Adenocarcinoma, EGFR, Large Cell, Second line, Squamous Cell, Stage III, Stage IV

Phase: II

Therapies: PD-1 Inhibitor, chemotherapy

Location: China

EGFR A289T mutation (continued)

NCT03574402
An Open-label, Multi-center, Phase II Umbrella Study to Assess Efficacy of Targeted Therapy or Immunotherapy Directed by Next Generation Sequencing (NGS) in Chinese Patients With Advanced NSCLC (TRUMP)

Cancer type: Non-Small Cell Lung Cancer

Variant class: EGFR mutation

Other identifiers: CTONG1702, TRUMP

Population segments: ALK, EGFR, Second line, Stage III, Stage IV

Phase: II

Therapy: pirotinib

Location: China

No NCT ID - see other identifier(s)
A Multicenter, Open-label Phase IIb Clinical Study to Evaluate the Efficacy and Safety of SZMD in Patients with Locally Advanced or Metastatic Lung Cancer (Non-resistant Rare EGFR Mutations Only, Including L861Q, G719X and/or S768I)

Cancer type: Non-Small Cell Lung Cancer

Variant class: EGFR mutation

Other identifier: ChiCTR2100051136

Population segments: EGFR, Second line, Stage III, Stage IV

Phase: II

Therapy: sutetinib

Location: China

No NCT ID - see other identifier(s)
A Single-Center, Open-Label , Non-Randomized Control Clinical Trial On Clinical Features And Medical Treatment Of Advanced NSCLC With Rare Gene Mutations

Cancer type: Non-Small Cell Lung Cancer

Variant class: EGFR mutation

Other identifier: ChiCTR1800017709

Population segments: BRAF, EGFR, KRAS, Second line, Stage III, Stage IV

Exclusion criteria variant classes: ALK fusion (Non-Small Cell Lung Cancer), EGFR L858R mutation (Non-Small Cell Lung Cancer), EGFR exon 19 deletion (Non-Small Cell Lung Cancer)

Phase: II

Therapies: targeted therapy, chemotherapy

Location: China

No NCT ID - see other identifier(s)
Clinical Study of Combined Action of the First Generation of TKIs and Brain Radiotherapy on EGFR-Mutated Non-Small-Cell Lung Cancer Patients with Brain Metastasis

Cancer type: Non-Small Cell Lung Cancer

Variant class: EGFR mutation

Other identifier: ChiCTR-IOR-17012098

Population segments: EGFR, Line of therapy N/A, Stage IV

Phase: II

Therapies: tyrosine kinase inhibitors, radiation therapy

Location: China

Disclaimer: The data presented here is from a curated knowledgebase of publicly available information, but may not be exhaustive. The data version is 2021.11(003).

EGFR A289T mutation (continued)

NCT03706287

Efficacy and Safety of Anlotinib Combined With Platinum Plus Pemetrexed in T790M Mutation Negative Metastastic Non-squamous Non-small-cell Lung Cancer After Progression on First-line EGFR TKI: a Phase II, Muti-center, Single Arm Study

Cancer type: Non-Small Cell Lung Cancer

Variant class: EGFR mutation

Other inclusion criteria: EGFR T790M negative

Other identifier: ALTER-L022

Population segments: Adenocarcinoma, EGFR, Large Cell, Second line, Stage III, Stage IV

Phase: I/II

Therapies: anlotinib hydrochloride, chemotherapy

Location: China

NCT04993391

A Dose-escalation, Dose-extension and Efficacy-extension, Phase I/II Clinical Study to Evaluate the Safety, Tolerability, Pharmacokinetic Profile and Preliminary Efficacy of AP-L1898 Capsule for the Treatment of the Patients With Locally Advanced or Metastatic Non-small Cell Lung Cancer

Cancer type: Non-Small Cell Lung Cancer

Variant class: EGFR mutation

Other identifiers: CTR20211347, JS111-001-I

Population segments: EGFR, Second line, Stage III, Stage IV

Phase: I/II

Therapy: AP-L1898

Location: China

No NCT ID - see other identifier(s)

A Multicenter, Single-Arm, Clinical Study of TQB2450 Plus Anlotinib in EGFR-Positive Non-Small Cell Lung Cancer Patients After Failure of EGFR TKI Therapy

Cancer type: Non-Small Cell Lung Cancer

Variant class: EGFR mutation

Other identifier: ChiCTR1900026273

Population segments: EGFR, Second line, Stage III, Stage IV

Phase: I/II

Therapies: CBT-502, anlotinib hydrochloride

Location: China

NCT03974022

A Phase I/II, Open-Label, Multicenter Study to Assess the Safety, Tolerability, Pharmacokinetics and Anti-tumor Efficacy of DZD9008 in Patients With Advanced Non-Small Cell Lung Cancer (NSCLC) With EGFR or HER2 Mutation

Cancer type: Non-Small Cell Lung Cancer

Variant class: EGFR mutation

Other identifiers: 19-2390, 19-280, DZ2019E0001, NCI-2019-08532, UCI-19-65, WU-KONG1

Population segments: EGFR, HER2 positive, Line of therapy N/A, Stage III, Stage IV

Phase: I/II

Therapy: DZD-9008

Locations: Australia, Taiwan, United States

US States: CA, CO, MA

Contact: Dr. Li Zheng [li.zheng@dizalpharma.com]

Disclaimer: The data presented here is from a curated knowledgebase of publicly available information, but may not be exhaustive. The data version is 2021.11(003).

EGFR A289T mutation (continued)

NCT03797391
First-in-human, Phase I/II, Multicenter, Open-Label Study of EMB-01 in Patients With Advanced/Metastatic Solid Tumors

Cancer type: Non-Small Cell Lung Cancer

Variant class: EGFR mutation

Other identifiers: CTR20190241, EMB01X101, NCI-2019-01391

Population segments: EGFR, Second line, Stage III, Stage IV

Phase: I/II

Therapy: EMB01

Locations: China, United States

US States: MA, MI, OH

Contact: Dr. YINGXI ZHANG [CT.info@epimab.com]

No NCT ID - see other identifier(s)
A phase I/II study of erlotinib/carboplatin/pemetrexed/bevacizumab in chemotherapy-naive patients with EGFR mutation positive advanced non-squamous non-small-cell lung cancer

Cancer type: Non-Small Cell Lung Cancer

Variant class: EGFR mutation

Other identifier: UMIN000013202

Population segments: Adenocarcinoma, EGFR, First line, Large Cell, Stage III, Stage IV

Exclusion criteria variant class: EGFR T790M mutation (Non-Small Cell Lung Cancer)

Phase: I/II

Therapies: erlotinib, chemotherapy, bevacizumab

Location: Japan

NCT03758287
A Phase Ib, Multi-center, Open Label Study of Ningetinib (CT053PTSA) in Combination With Gefitinib in Stage IIIB or IV NSCLC Patients With EGFR Mutation and T790M Negative Who Have Progressed After EGFR TKI Therapy

Cancer type: Non-Small Cell Lung Cancer

Variant class: EGFR mutation

Other inclusion criteria: EGFR T790M negative

Other identifiers: CTR20160875, PCD-DCT053-16-001

Population segments: EGFR, Fourth line or greater, Second line, Stage III, Stage IV, Third line

Phase: I/II

Therapies: ningetinib, gefitinib

Location: China

No NCT ID - see other identifier(s)
A Phase Ib/II Umbrella Study to Evaluate the Safety and Efficacy of Varlitinib in Combination with Weekly Paclitaxel in HER1/HER2 Co-Expressing Advanced or Metastatic Gastric Cancer

Cancer type: Gastric Cancer

Variant class: EGFR mutation

Other inclusion criteria: ERBB2 expression

Other identifiers: 4-2018-1011, KCT0003583, KM-13

Population segments: EGFR, HER2 negative, HER2 positive, Second line, Stage III, Stage IV

Phase: I/II

Therapy: varlitinib + chemotherapy

Location: Republic of Korea

Disclaimer: The data presented here is from a curated knowledgebase of publicly available information, but may not be exhaustive. The data version is 2021.11(003).

EGFR A289T mutation (continued)

NCT03711422

A Dose Finding Study of Continuous and Intermittent High-dose (HDI) Afatinib (EGFR TKI) on CNS Metastases and Leptomeningeal Disease (LMD) in Patients With Advanced Refractory EGFR Mutation Positive Non-small Cell Lung Cancer

Cancer type: Non-Small Cell Lung Cancer

Variant class: EGFR mutation

Other identifier: 1200.275

Population segments: CNS mets, EGFR, Second line, Stage III, Stage IV

Phase: I

Therapy: afatinib

Location: Singapore

NCT02609776

A Phase I, First-in-Human, Open-Label, Dose Escalation Study of JNJ-61186372, a Human Bispecific EGFR and cMet Antibody, in Subjects With Advanced Non-Small Cell Lung Cancer.

Cancer type: Non-Small Cell Lung Cancer

Variant class: EGFR mutation

Other identifiers: 18137, 18-456, 19389, 61186372EDI1001, CHRYSALIS, CR108064, CTR20190589, EudraCT Number: 2018-003908-38, JapicCTI-184169, NCI-2018-00140, RWF_61186372EDI1001, S17-01799

Population segments: EGFR, First line, Fourth line or greater, Second line, Stage III, Stage IV, Third line

Phase: I

Therapies: amivantamab, chemotherapy

Locations: Australia, Canada, China, France, Japan, Republic of Korea, Spain, Taiwan, United Kingdom, United States

US States: CA, FL, IL, MA, MD, MI, MN, MO, NY, OR, PA, VA

NCT04528836

A Phase I/IB First-in-Human Study of the SHP2 Inhibitor BBP-398 (Formerly Known as IACS-15509) in Patients With Advanced Solid Tumors

Cancer type: Non-Small Cell Lung Cancer

Variant class: EGFR mutation

Other identifier: NAV-1001

Population segments: Second line, Stage II, Stage III

Exclusion criteria variant classes: BRAF V600 mutation (Non-Small Cell Lung Cancer, Unspecified Solid Tumor), HRAS Q61 mutation (Non-Small Cell Lung Cancer, Unspecified Solid Tumor), KRAS Q61 mutation (Non-Small Cell Lung Cancer, Unspecified Solid Tumor), NRAS Q61 mutation (Non-Small Cell Lung Cancer, Unspecified Solid Tumor), PTPN11 activating mutation (Non-Small Cell Lung Cancer, Unspecified Solid Tumor)

Phase: I

Therapy: BBP-398

Location: United States

US States: CO, TX, UT

Contact: Vaeling Clinical Operations Lead [408-203-1726; vaeling.miller@bridgebio.com]

Disclaimer: The data presented here is from a curated knowledgebase of publicly available information, but may not be exhaustive. The data version is 2021.11(003).

EGFR A289T mutation (continued)

No NCT ID - see other identifier(s)
Feasibility Study of Pemetrexed / Bevacizumab / Erlotinib in Chemotherapy Naive Patients With Non- Small Cell Lung Cancer Harboring EGFR Mutation

Cancer type: Non-Small Cell Lung Cancer

Variant class: EGFR mutation

Other identifier: UMIN000014477

Population segments: Adenocarcinoma, EGFR, First line, Large Cell, Stage III, Stage IV

Exclusion criteria variant class: EGFR T790M mutation (Non-Small Cell Lung Cancer)

Phase: I

Therapy: bevacizumab + erlotinib + chemotherapy

Location: Japan

No NCT ID - see other identifier(s)
Phase I Study of DZD9008 in EGFR or HER2 Mutant NSCLC Chinese Patients

Cancer type: Non-Small Cell Lung Cancer

Variant class: EGFR mutation

Other identifiers: CTR20192097, DZ2019E0002

Population segments: CNS mets, EGFR, HER2 positive, Second line, Stage III, Stage IV

Phase: I

Therapy: DZD-9008

Location: China

NCT04077463
An Open-label Phase I/Ib Study to Evaluate the Safety and Pharmacokinetics of JNJ-73841937 (Lazertinib), a Third Generation EGFR-TKI, as Monotherapy or in Combinations With JNJ-61186372, a Human Bispecific EGFR and cMet Antibody in Participants With Advanced Non-Small Cell Lung Cancer

Cancer type: Non-Small Cell Lung Cancer

Variant class: EGFR mutation

Other identifiers: 20-492, 73841937NSC1001, AAAT1655, CHRYSALIS-2, CR108656, CTR20202097, EudraCT Number: 2020-000747-31, Janssen ChrysalisNSC 1001, JapicCTI-194934, NSC 1001, S20-00901

Population segments: EGFR, First line, Fourth line or greater, Second line, Stage III, Stage IV, Third line

Phase: I

Therapies: lazertinib, amivantamab, chemotherapy

Locations: China, France, Germany, Italy, Japan, Puerto Rico, Republic of Korea, Spain, Taiwan, United States

US States: CA, FL, MA, MI, MO, NY, OR, PA, UT, VA, WA

Contact: Study Contact [844-434-4210; JNJ.CT@sylogent.com]

NCT04013542
A Pilot Trial of Ipilimumab-Nivolumab in Local-Regionally Advanced Non Small Cell Lung Cancer (NSCLC)

Cancer type: Non-Small Cell Lung Cancer

Variant class: EGFR mutation

Other identifiers: 2018-0836, NCI-2019-03205

Population segments: ALK, EGFR, First line, Maintenance/Consolidation, Stage II, Stage III

Phase: I

Therapies: nivolumab, ipilimumab, radiation therapy

Location: United States

US State: TX

Contact: Anne S. Tsao [713-792-6363; astsao@mdanderson.org]

Disclaimer: The data presented here is from a curated knowledgebase of publicly available information, but may not be exhaustive. The data version is 2021.11(003).

EGFR A289T mutation (continued)

NCT04140526

Safety, Pharmacokinetics (PK), and Efficacy of ONC-392 as a Single Agent and in Combination With Pembrolizumab in Advanced Solid Tumors and NSCLC: An Open Label Phase IA/IB Study. Preserve CTLA4 Checkpoint Function (PRESERVE-001)

Cancer type: Non-Small Cell Lung Cancer

Variant class: EGFR mutation

Other identifiers: 20193108, NCI-2020-06149, ONC-392-001, PRESERVE-001

Population segments: ALK, EGFR, First line, Merkel, Pulmonary, Second line, Stage III, Stage IV, Third line

Phase: I

Therapies: ONC-392, osimertinib

Location: United States

US States: CA, MD, OH, UT

Contact: Dr. Pan Zheng [202-751-6823; pzheng@oncoc4.com]

No NCT ID - see other identifier(s)

Study Of Immunologic Factor In Re-Biopsy Specimen, Peritumoral BALF, And The Peripheral Blood For Predicting Response To Osimertinib In NSCLC Patients

Cancer type: Non-Small Cell Lung Cancer

Variant class: EGFR mutation

Other identifier: UMIN000023853

Population segments: EGFR, N/A, Second line or greater/Refractory/Relapsed

Phase: I

Therapy: osimertinib

Location: Japan

No NCT ID - see other identifier(s)

Pharmacokinetic and dose finding study of osimertinib in patients with impaired renal function and low body weight

Cancer type: Non-Small Cell Lung Cancer

Variant class: EGFR mutation

Other identifiers: jRCTs031180232, UMIN000033301

Population segments: EGFR, Line of therapy N/A, Stage III, Stage IV

Phase: I

Therapy: osimertinib

Location: Japan

NCT03800134

A Phase III, Double-blind, Placebo-controlled, Multi-center International Study of Neoadjuvant/Adjuvant Durvalumab for the Treatment of Patients With Resectable Stages II and III Non-small Cell Lung Cancer (AEGEAN)

Cancer type: Non-Small Cell Lung Cancer

Variant class: EGFR mutation status

Other inclusion criteria: CD274 expression status

Other identifiers: AEGEAN, CTR20200932, CTRI/2019/06/019634, D9106C00001, EudraCT Number: 2018-002997-29, JapicCTI-194656, NCI-2019-01822, NL72968.056.20, PHRR190321-002089, Pro00090077

Population segments: ALK, Adenocarcinoma, Adjuvant, EGFR, Large Cell, Neoadjuvant, Squamous Cell, Stage II, Stage III

Phase: III

Therapies: durvalumab, chemotherapy

Locations: Argentina, Austria, Belgium, Brazil, Bulgaria, Canada, Chile, China, Costa Rica, France, Germany, Hungary, India, Italy, Japan, Mexico, Netherlands, Peru, Philippines, Poland, Puerto Rico, Republic of Korea, Russian Federation, Spain, Taiwan, Thailand, United States, Viet Nam

US States: AZ, CA, FL, IL, KS, KY, MD, MN, NC, NJ, NY, OR, PA, SC, TX, VA, WA

Contact: AstraZeneca Clinical Study Information Center [877-240-9479; information.center@astrazeneca.com]

Disclaimer: The data presented here is from a curated knowledgebase of publicly available information, but may not be exhaustive. The data version is 2021.11(003).

EGFR A289T mutation (continued)

No NCT ID - see other identifier(s)
A Pilot Study for Apatinib Mesylate Combined with Gefitinib in First-line Treatment of Lung Adenocarcinoma with Malignant Pleural Effusion or Pericardial Effusion

Cancer type: Non-Small Cell Lung Cancer

Variant class: EGFR positive

Other identifier: ChiCTR1800017332

Population segments: Adenocarcinoma, EGFR, First line, N/A

Phase: IV

Therapies: apatinib, gefitinib

Location: China

NCT04824079
Study of Kenaitinib in Patients With Advanced Non-small Cell Lung Cancer With Brain Metastasis or Progression of Brain Metastasis After Treatment With EGFR Inhibitor(s)

Cancer type: Non-Small Cell Lung Cancer

Variant class: EGFR positive

Other identifiers: CTR20200640, MEDO-007-20002

Population segments: CNS mets, EGFR, Second line, Stage III, Stage IV

Phase: II

Therapy: keynatinib

Location: China

NCT03542799
Phase I/II Study of EGFR-IL12-CART Cells for Patients With Metastatic Colorectal Cancer

Cancer type: Colorectal Cancer

Variant class: EGFR positive

Other identifiers: CN-0093, EGFRCART, Protocol. PGCAR/EGFR012

Population segments: Line of therapy N/A, Stage IV

Phase: I/II

Therapy: CART-EGFR/IL12 (Pregene)

Location: China

No NCT ID - see other identifier(s)
A Phase Ib Study to Evaluate the Safety and Efficacy of Combination Therapy With Nivolumab and Photoimmunotherapy (PIT) Using ASP-1929 for the Patients With Unresectable Advanced / Recurrent Gastric Cancer or Esophageal Cancer

Cancer type: Esophageal Cancer, Gastric Cancer

Variant class: EGFR positive

Other identifiers: EPOC1901, GE-PIT, JapicCTI-194969

Population segments: EGFR, Second line, Stage III, Stage IV

Phase: I

Therapies: nivolumab, cetuximab sarotalocan

Location: Japan

Disclaimer: The data presented here is from a curated knowledgebase of publicly available information, but may not be exhaustive. The data version is 2021.11(003).

EGFR A289T mutation (continued)

NCT03919292
Phase 1/2 Study of Neratinib and Divalproex Sodium (Valproate) in Advanced Solid Tumors, With an Expansion Cohort in Ras-Mutated Cancers

Cancer type: Glioblastoma

Variant class: EGFR aberration

Other identifiers: MCC-17-13821, NCI-2019-02675

Population segments: KRAS, NRAS, Second line, Stage III, Stage IV

Phase: I/II

Therapies: neratinib, valproic acid

Location: United States

US State: VA

Contact: Caryn Weir [804-628-2310; cweir@vcu.edu]

Relevant Biomarkers

Genomic Alteration	Relevant Therapies (In this cancer type)	Relevant Therapies (In other cancer type)	Clinical Trials
EGFR A289T mutation	None	afatinib<br>bevacizumab + erlotinib<br>bevacizumab + gefitinib<br>dacomitinib<br>erlotinib<br>erlotinib + ramucirumab<br>gefitinib<br>gefitinib + chemotherapy	90

Prognostic significance: None
Diagnostic significance: None

Public data sources included in relevant therapies: FDA[1], NCCN, **EMA**[2], ESMO
Public data sources included in prognostic and diagnostic significance: NCCN, ESMO

Relevant Therapy Summary

● In this cancer type ○ In other cancer type ◐ In this cancer type and other cancer types ✖ No evidence

EGFR A289T mutation

Relevant Therapy	FDA	NCCN	EMA	ESMO	Clinical Trials*
afatinib	✖	✖	✖	○	◐ (II)
erlotinib	✖	✖	✖	○	● (II)
dacomitinib	✖	✖	✖	○	○ (IV)
gefitinib	✖	✖	✖	○	○ (II)
bevacizumab + erlotinib	✖	✖	✖	○	✖
bevacizumab + gefitinib	✖	✖	✖	○	✖
erlotinib + ramucirumab	✖	✖	✖	○	✖
gefitinib + carboplatin + pemetrexed	✖	✖	✖	○	✖

* Most advanced phase (IV, III, II/III, II, I/II, I) is shown and multiple clinical trials may be available.

Disclaimer: The data presented here is from a curated knowledgebase of publicly available information, but may not be exhaustive. The data version is 2021.11(003).

Relevant Therapy Summary (continued)

● In this cancer type ○ In other cancer type ◐ In this cancer type and other cancer types ✖ No evidence

EGFR A289T mutation (continued)

Relevant Therapy	FDA	NCCN	EMA	ESMO	Clinical Trials*
pirotinib	✖	✖	✖	✖	◐ (II)
poziotinib	✖	✖	✖	✖	◐ (II)
gefitinib, erlotinib, afatinib	✖	✖	✖	✖	● (II)
BCA101	✖	✖	✖	✖	● (I)
FT500, nivolumab, pembrolizumab, atezolizumab	✖	✖	✖	✖	● (I)
neratinib, palbociclib, everolimus, trametinib	✖	✖	✖	✖	● (I)
TQB 3804	✖	✖	✖	✖	● (I)
ZZ06	✖	✖	✖	✖	● (I)
afatinib, osimertinib	✖	✖	✖	✖	○ (IV)
apatinib + EGFR tyrosine kinase inhibitor	✖	✖	✖	✖	○ (IV)
apatinib, gefitinib	✖	✖	✖	✖	○ (IV)
EGFR tyrosine kinase inhibitor	✖	✖	✖	✖	○ (IV)
gefitinib, radiation therapy	✖	✖	✖	✖	○ (IV)
osimertinib	✖	✖	✖	✖	○ (IV)
atezolizumab, bevacizumab, chemotherapy	✖	✖	✖	✖	○ (III)
durvalumab, chemotherapy	✖	✖	✖	✖	○ (III)
icotinib hydrochloride, chemotherapy	✖	✖	✖	✖	○ (III)
almonertinib	✖	✖	✖	✖	○ (II)
almonertinib, chemotherapy	✖	✖	✖	✖	○ (II)
anlotinib hydrochloride	✖	✖	✖	✖	○ (II)
anlotinib hydrochloride, chemotherapy	✖	✖	✖	✖	○ (II)
anlotinib hydrochloride, gefitinib	✖	✖	✖	✖	○ (II)
bevacizumab, osimertinib	✖	✖	✖	✖	○ (II)
dacomitinib, osimertinib	✖	✖	✖	✖	○ (II)
datopotamab deruxtecan	✖	✖	✖	✖	○ (II)
EGFR tyrosine kinase inhibitor + chemotherapy, EGFR tyrosine kinase inhibitor	✖	✖	✖	✖	○ (II)
EGFR tyrosine kinase inhibitor, radiation therapy	✖	✖	✖	✖	○ (II)
erlotinib, chemotherapy	✖	✖	✖	✖	○ (II)

* Most advanced phase (IV, III, II/III, II, I/II, I) is shown and multiple clinical trials may be available.

Disclaimer: The data presented here is from a curated knowledgebase of publicly available information, but may not be exhaustive. The data version is 2021.11(003).

Relevant Therapy Summary (continued)

● In this cancer type ○ In other cancer type ◐ In this cancer type and other cancer types ✖ No evidence

EGFR A289T mutation (continued)

Relevant Therapy	FDA	NCCN	EMA	ESMO	Clinical Trials*
famitinib, almonertinib	✖	✖	✖	✖	○ (II)
gefitinib, bevacizumab, chemotherapy	✖	✖	✖	✖	○ (II)
keynatinib	✖	✖	✖	✖	○ (II)
nazartinib, gefitinib	✖	✖	✖	✖	○ (II)
nivolumab	✖	✖	✖	✖	○ (II)
olaparib, durvalumab	✖	✖	✖	✖	○ (II)
osimertinib, chemotherapy	✖	✖	✖	✖	○ (II)
panitumumab	✖	✖	✖	✖	○ (II)
PD-1 Inhibitor, chemotherapy	✖	✖	✖	✖	○ (II)
sutetinib	✖	✖	✖	✖	○ (II)
targeted therapy, chemotherapy	✖	✖	✖	✖	○ (II)
tyrosine kinase inhibitors, radiation therapy	✖	✖	✖	✖	○ (II)
AP-L1898	✖	✖	✖	✖	○ (I/II)
BLU-945	✖	✖	✖	✖	○ (I/II)
brigatinib, panitumumab	✖	✖	✖	✖	○ (I/II)
CART-EGFR/IL12 (Pregene)	✖	✖	✖	✖	○ (I/II)
CBT-502, anlotinib hydrochloride	✖	✖	✖	✖	○ (I/II)
DZD-9008	✖	✖	✖	✖	○ (I/II)
EMB01	✖	✖	✖	✖	○ (I/II)
erlotinib, chemotherapy, bevacizumab	✖	✖	✖	✖	○ (I/II)
mobocertinib, chemotherapy	✖	✖	✖	✖	○ (I/II)
neratinib, valproic acid	✖	✖	✖	✖	○ (I/II)
ningetinib, gefitinib	✖	✖	✖	✖	○ (I/II)
quaratusugene ozeplasmid, osimertinib	✖	✖	✖	✖	○ (I/II)
varlitinib + chemotherapy	✖	✖	✖	✖	○ (I/II)
amivantamab, chemotherapy	✖	✖	✖	✖	○ (I)
BBP-398	✖	✖	✖	✖	○ (I)
bevacizumab + erlotinib + chemotherapy	✖	✖	✖	✖	○ (I)
lazertinib, amivantamab, chemotherapy	✖	✖	✖	✖	○ (I)

* Most advanced phase (IV, III, II/III, II, I/II, I) is shown and multiple clinical trials may be available.

Disclaimer: The data presented here is from a curated knowledgebase of publicly available information, but may not be exhaustive. The data version is 2021.11(003).

Relevant Therapy Summary (continued)

● In this cancer type ○ In other cancer type ◐ In this cancer type and other cancer types ✖ No evidence

EGFR A289T mutation (continued)

Relevant Therapy	FDA	NCCN	EMA	ESMO	Clinical Trials*
MRX-2843, osimertinib	✖	✖	✖	✖	○ (I)
niraparib, osimertinib	✖	✖	✖	✖	○ (I)
nivolumab, cetuximab sarotalocan	✖	✖	✖	✖	○ (I)
nivolumab, ipilimumab, radiation therapy	✖	✖	✖	✖	○ (I)
ONC-392, osimertinib	✖	✖	✖	✖	○ (I)
telaglenastat, sapanisertib	✖	✖	✖	✖	○ (I)
TNO-155, nazartinib	✖	✖	✖	✖	○ (I)
WSD-0922	✖	✖	✖	✖	○ (I)

* Most advanced phase (IV, III, II/III, II, I/II, I) is shown and multiple clinical trials may be available.

Relevant Biomarkers

Genomic Alteration	Relevant Therapies (In this cancer type)	Relevant Therapies (In other cancer type)	Clinical Trials
EGFR A289T mutation	None	afatinib<br>bevacizumab + erlotinib<br>bevacizumab + gefitinib<br>dacomitinib<br>erlotinib<br>erlotinib + ramucirumab<br>gefitinib<br>gefitinib + chemotherapy	90

Prognostic significance: None
Diagnostic significance: None

Public data sources included in relevant therapies: FDA1, NCCN, **EMA**2, ESMO
Public data sources included in prognostic and diagnostic significance: NCCN, ESMO

Clinical Trials Summary

EGFR A289T mutation

NCT ID	Title	Phase
NCT04172597	A Phase II Study of Poziotinib in Patients With EGFR or HER2 Activating Mutations in Advanced Malignancies	II
NCT02465060	Molecular Analysis for Therapy Choice (MATCH).	II
NCT03810872	An Open Explorative Phase II, Open Label Study of Afatinib in the Treatment of Advanced Cancer Carrying an EGFR, a HER2 or a HER3 Mutation	II
NCT04591431	The Rome Trial From Histology to Target: the Road to Personalize Target Therapy and Immunotherapy	II

Disclaimer: The data presented here is from a curated knowledgebase of publicly available information, but may not be exhaustive. The data version is 2021.11(003).

Clinical Trials Summary (continued)

EGFR A289T mutation (continued)

NCT ID	Title	Phase
NCT03239015	Efficacy and Safety of Targeted Precision Therapy in Refractory Tumor With Druggable Molecular Event	II
NCT03065387	Phase I Study of the Pan-ERBB Inhibitor Neratinib Given in Combination With Everolimus, Palbociclib, or Trametinib in Advanced Cancer Subjects With EGFR Mutation/Amplification, HER2 Mutation/ Amplification, or HER3/4 Mutation or KRAS Mutation	I
No NCT ID	Phase I Clinical Study With Advanced Solid Tumors KBP-5209 Treatment	I
NCT04128085	A Phase I, Open-label, Multicenter, Dose Escalation and Expansion Study to Evaluate the Tolerance and Pharmacokinetics of TQB3804 in Subjects With Advanced Malignant Tumors	I
NCT03841110	FT500 as Monotherapy and in Combination With Immune Checkpoint Inhibitors in Subjects With Advanced Solid Tumors (Phase I)	I
NCT04412616	A Phase I, Multicenter, Open-label Study to Evaluate the Safety, Tolerability, Pharmacokinetics, Immunogenicity, and Preliminary Evidence of Antitumor Activity of ZZ06 in Adult Patients With Advanced EGFR Positive Solid Tumor Malignancies	I
NCT03297606	Canadian Profiling and Targeted Agent Utilization Trial (CAPTUR): A Phase II Basket Trial	II
NCT04429542	First-in-Human, Phase I/Ib, Open-label, Multicenter Study of Bifunctional EGFR/TGFß Fusion Protein BCA101 Alone and in Combination With Pembrolizumab in Patients With EGFR-Driven Advanced Solid Tumors	I
NCT04511533	Single Arm Study to Evaluate the Safety of Dacomitinib for the First-Line Treatment of Participants in India With Metastatic Non-Small Cell Lung Cancer With Epidermal Growth Factor Receptor (EGFR)-Activating Mutations	IV
No NCT ID	The Continuous Evaluation of EGFR Mutation in EGFR-mutation Positive Lung Cancer Patients During EGFR TKI Treatment.	IV
NCT03991403	Study of Atezolizumab in Combination With Carboplatin + Paclitaxel +Bevacizumab vs With Pemetrexed + Cisplatin or Carboplatin With Stage IV Non-Squamous Non-Small Cell Lung Cancer with EGFR (+) or ALK (+)	III
NCT03088059	A Pilot Study of Personalized Biomarker-based Treatment Strategy or Immunotherapy in Patients With Recurrent/Metastatic Squamous Cell Carcinoma of the Head and Neck	II
NCT04147351	A Phase II Study of Atezolizumab in Combination With Bevacizumab, Carboplatin or Cisplatin, and Pemetrexed for EGFR-mutant Metastatic Non-small Cell Lung Cancer Patients After Failure of EGFR Tyrosine Kinase Inhibitors.	II
NCT04811001	A Randomised Non-comparative, Phase II Study Investigating the Best Epidermal Growth Factor Receptor Tyrosine Kinase Inhibitor (EGFR-TKI) Sequence in Advanced or Metastatic Non Small-Cell Lung Cancer (NSCLC) Harboring EGFR Mutations	II
NCT04484142	Phase II, Single-arm, Open-label Study of DS-1062a in Advanced or Metastatic Non-small Cell Lung Cancer With Actionable Genomic Alterations and Progressed On or After Applicable Targeted Therapy and Platinum Based Chemotherapy (TROPION-Lung05)	II
No NCT ID	ITAC 2 TRIAL: Intermittent TKI and Chemotherapy for Patients with Advanced Non-Small Cell Lung Cancer	II
NCT03151161	A Prospective,Multi-center, Open-labeled Phase II Randomized and Comparative Clinical Study of First Line Intermittent and Maintenance of Icotinib in Combination With Pemetrexed/Carboplatin Compared With Icotinib Single Drug in IIIB/IV Non Small Cell Lung Cancer With Epidermal Growth Factor Receptor (EGFR) Mutation	II
NCT03292133	A Phase II Study of EGF816 and Gefitinib in TKI-naive EGFR-mutant Non-Small Cell Lung Cancer	II

Disclaimer: The data presented here is from a curated knowledgebase of publicly available information, but may not be exhaustive. The data version is 2021.11(003).

Clinical Trials Summary (continued)

EGFR A289T mutation (continued)

NCT ID	Title	Phase
NCT04335292	Osimertinib Then Chemotherapy in EGFR-mutated Lung Cancer With Osimertinib Third-line Rechallenge	II
NCT04410796	A Phase II Randomized Study of Osimertinib Versus Osimertinib Plus Chemotherapy for Patients With Metastatic EGFR-Mutant Lung Cancers That Have Detectable EGFR-Mutant cfDNA in Plasma After Initiation of Osimertinib	II
NCT03318939	A Phase II Study of Poziotinib in Patients With Non-Small Cell Lung Cancer (NSCLC), Locally Advanced or Metastatic, With EGFR or HER2 Exon 20 Insertion Mutation (ZENITH20).	II
NCT04862780	A Phase I/II Study Targeting Acquired Resistance Mechanisms in Patients With EGFR Mutant Non-Small Cell Lung Cancer	I/II
No NCT ID	Phase I/II Study of Brigatinib plus Panitumumab in Patients with Advanced EGFR-mutated Non-small Cell Lung Cancer Harboring C797S Resistant Mutation to Osimertinib	I/II
NCT02716116	A Phase I/II Study of the Safety, Pharmacokinetics, and Anti-Tumor Activity of the Oral EGFR/HER2 Inhibitor TAK-788 (AP32788) in Non-Small Cell Lung Cancer.	I/II
NCT04486833	A Phase I/II Open-Label, Dose-escalation and Clinical Response Study of GPX-001 in Combination With Osimertinib in Patients With Advanced, Metastatic EGFR-mutant Non-small Cell Lung Cancer	I/II
No NCT ID	A Phase I Study Afatinib In Combination Of Osimertinib In Patients With Relapsed Non-Small Cell Lung Cancer After Failure of Prior Osimertinib	I
NCT03755102	A Pilot Study of Dacomitinib With or Without Osimertinib for Patients With Metastatic EGFR Mutant Lung Cancers With Disease Progressionon Osimertinib	I
NCT04762199	A Phase Ib Safety and Pharmacodynamic Study of MER Tyrosine Kinase Inhibitor, MRX-2843, in Combination With Osimertinib in Advanced EGFR Mutant Non-Small Cell Lung Cancer	I
NCT03891615	Phase I Study of Niraparib in Combination With Osimertinib in EGFR-Mutated Advanced Lung Cancer	I
NCT04250545	A Phase I Trial of MLN0128 (Sapanisertib) and CB-839 HCl (Telaglenastat) in Advanced NSCLC Patients	I
NCT03114319	An Open-label, Multi-center, Phase I, Dose Finding Study of Oral TNO155 in Adult Patients With Advanced Solid Tumors.	I
NCT04197934	Phase I Study to Evaluate Safety, Tolerability, Pharmacokinetics and Anti-Tumor Activity of WSD0922-FUFU	I
NCT04413201	AFAMOSI: Prospective, Randomized, Multicenter Phase IV Study to Evaluate the Efficacy and Safety of Afatinib Followed by Osimertinib Compared to Osimertinib in Patients With EGFRmutated/T790M Mutation Negative Non-squamous NSCLC in the First-line Setting	IV
No NCT ID	Apatinib Combined With EGFR-TKI For Patients With EGFR Mutation Who Failed EGFR-TKI: A Prospective Study	IV
No NCT ID	A Real World Study Of Apatinib Combined With Gefitinib In The Treatment Of EGFRm+ Advanced Non-Squamous Non-Small Cell Lung Cancer	IV
No NCT ID	Clinical Study Of Combined Action Of Gefitinib And Brain Radiotherapy On EGFR-Mutated Non-Small-Cell Lung Cancer Patients With Brain Metastasis	IV
No NCT ID	A Multicenter, Open-Label, Single Arm Pilot Study: Osimertinib As Neoadjuvant Treatment For Resectable Stage II-IIIA EGFR Mutant Lung Adenocarcinoma.	IV
NCT03992885	Combination Therapy With Icotinib, Pemetrexed and Platinum in Patients With Metastatic Non-squamous Non-small Cell Lung Cancer With EGFR Mutations Who Did Not Progress After Pemetrexed in Combination With Platinum-based Chemotherapy:a Single-arm, Open, Multicenter Clinical Study.	III

Disclaimer: The data presented here is from a curated knowledgebase of publicly available information, but may not be exhaustive. The data version is 2021.11(003).

Clinical Trials Summary (continued)

EGFR A289T mutation (continued)

NCT ID	Title	Phase
No NCT ID	A Phase II Study Of Afatinib For Advanced Non-Small Cell Lung Cancer With Uncommon Epidermal Growth Factor Receptor (EGFR) Mutation Including Compound Mutation Detected By Next Generation Sequencing	II
NCT04785742	An Open, Multicenter Phase II Clinical Study to Evaluate the Safety and Efficacy of Almonertinib in Patients With Advanced NSCLC With Rare Mutations in EGFR	II
No NCT ID	Safety and efficacy analysis of Ametinib combined with Pemetrexed intraoperatively for treatment of EGFR mutant lung cancer with meningeal metastases after first-line TKI failure	II
No NCT ID	A Single-center, Open Label, Phase II Study of Anlotinib as Second/Third-line Treatment for Advanced Non-small Cell Lung Cancer	II
NCT04619563	A Single-arm Exploratory Clinical Study of Anlotinib Hydrochloride Combined With Docetaxel in EGFR Mutations Advanced Non Small Cell Lung Cancer Patients Who Have Progressed After Targeted Therapy and Chemotherapy	II
No NCT ID	Clinical Study And Safety Analysis On The Treatment Of Advanced Non-Small Cell Lung Cancer With Anlotinib And Gefitinib	II
NCT04245085	A Randomised Non-comparative Open Label Phase II Trial of Atezolizumab Plus Bevacizumab, With Carboplatin-paclitaxel or Pemetrexed, in EGFR-mutant Non-small Cell Lung Carcinoma With Acquired Resistance	II
No NCT ID	Osimertinib Combined Bevacizumab in Untreated Epidermal Growth Factor Receptor Mutated Non-small-cell Lung Cancer Patients with Malignant Pleural And/Or Pericardial Effusion -phase II Trial	II
No NCT ID	Randomized Controlled Trial for EGFR-TKIs Plus S-1 or EGFR-TKIs as the First-Line Therapy for Patients with Advanced Non-small Cell Lung Cancer Harboring EGFR Mutations	II
No NCT ID	EGFR-TKI Combined With Stereotactic Body Radiation Therapy Versus TKI alone for Stage IV Oncogene-Driven Non-Small Cell Lung Cancer.	II
NCT03904823	An Open, Single-arm, Multi-center, Phase II Clinical Trial of Famitinib Combined With Epidermal Growth Factor Receptor (EGFR) Inhibitor HS-10296 in Patients With Advanced EGFR-mutant Non-Small Cell Lung Cancer (NSCLC)	II
NCT01784549	A Multi-center Phase II Randomized Study of Customized Neoadjuvant Therapy Versus Standard Chemotherapy in Non-small Cell Lung Cancer (NSLC) Patients With Resectable Stage IIIA (N2) Disease	II
No NCT ID	Gefitinib Plus Pemetrexed Combined With Bevacizumab Or Carboplatin In First-LineTreatment Of Stage IV EGFR Mutant Non-Squamous Non-Small Cell Lung Cancer	II
No NCT ID	Open-Label, Single arm, Phase II trial to Evaluate Efficacy and Safety of Nivolumab as Maintenance Therapy Following Platinum-based Chemotherapy in Non-Small Cell Lung Cancer Patients after Tyrosine Kinase Inhibitor Therapy	II
NCT04538378	Phase II Trial of Olaparib (LYNPARZA) Plus Durvalumab (IMFINZI) in EGFR-Mutated Adenocarcinomas That Transform to Small Cell Lung Cancer (SCLC) and Other Neuroendocrine Tumors.	II
No NCT ID	Phase II trial of osimertinib for rare EGFR mutation-positive non-small cell lung cancer	II
NCT03460275	Osimertinib as First-line Therapy for Patients With EGFR Mutation-positive Locally Advanced or Metastatic Non-squamous Non-Small Cell Lung Cancer(NSCLC), a Single-Arm, Open-Label, Prospective, Multicenter, Phase II Clinical Trial	II
No NCT ID	Efficacy Of Osimertinib With Platinum And Pemetrexed In EGFR Mutant Non-Small Cell Lung Cancer Patients Bearing CNS Metastasis, And Have Systemic Progression But Stable Intracranial Disease On Osimertinib Resistance. (EPONA)	II

Disclaimer: The data presented here is from a curated knowledgebase of publicly available information, but may not be exhaustive. The data version is 2021.11(003).

Clinical Trials Summary (continued)

EGFR A289T mutation (continued)

NCT ID	Title	Phase
NCT03087071	A Phase II Enrichment Study of Panitumumab as a Single Agent or in Combination With Trametinib in Anti-EGFR-Refractory Stage IV Colorectal Cancer Patients	II
No NCT ID	An Exploratory Clinical Study Of PD-1 Inhibitor Combined With Chemotherapy In The Treatment Of Advanced Non-small Cell Lung Cancer With EGFR Mutation Positive And T790M Negative After Failure Of TKI Combined With Antiangiogenic Drugs	II
NCT03574402	An Open-label, Multi-center, Phase II Umbrella Study to Assess Efficacy of Targeted Therapy or Immunotherapy Directed by Next Generation Sequencing (NGS) in Chinese Patients With Advanced NSCLC (TRUMP)	II
No NCT ID	A Multicenter, Open-label Phase IIb Clinical Study to Evaluate the Efficacy and Safety of SZMD in Patients with Locally Advanced or Metastatic Lung Cancer (Non-resistant Rare EGFR Mutations Only, Including L861Q, G719X and/or S768I)	II
No NCT ID	A Single-Center, Open-Label , Non-Randomized Control Clinical Trial On Clinical Features And Medical Treatment Of Advanced NSCLC With Rare Gene Mutations	II
No NCT ID	Clinical Study of Combined Action of the First Generation of TKIs and Brain Radiotherapy on EGFR-Mutated Non-Small-Cell Lung Cancer Patients with Brain Metastasis	II
NCT03706287	Efficacy and Safety of Anlotinib Combined With Platinum Plus Pemetrexed in T790M Mutation Negative Metastastic Non-squamous Non-small-cell Lung Cancer After Progression on First-line EGFR TKI: a Phase II, Muti-center, Single Arm Study	I/II
NCT04993391	A Dose-escalation, Dose-extension and Efficacy-extension, Phase I/II Clinical Study to Evaluate the Safety, Tolerability, Pharmacokinetic Profile and Preliminary Efficacy of AP-L1898 Capsule for the Treatment of the Patients With Locally Advanced or Metastatic Non-small Cell Lung Cancer	I/II
No NCT ID	A Multicenter, Single-Arm, Clinical Study of TQB2450 Plus Anlotinib in EGFR-Positive Non-Small Cell Lung Cancer Patients After Failure of EGFR TKI Therapy	I/II
NCT03974022	A Phase I/II, Open-Label, Multicenter Study to Assess the Safety, Tolerability, Pharmacokinetics and Anti-tumor Efficacy of DZD9008 in Patients With Advanced Non-Small Cell Lung Cancer (NSCLC) With EGFR or HER2 Mutation	I/II
NCT03797391	First-in-human, Phase I/II, Multicenter, Open-Label Study of EMB-01 in Patients With Advanced/ Metastatic Solid Tumors	I/II
No NCT ID	A phase I/II study of erlotinib/carboplatin/pemetrexed/bevacizumab in chemotherapy-naive patients with EGFR mutation positive advanced non-squamous non-small-cell lung cancer	I/II
NCT03758287	A Phase Ib, Multi-center, Open Label Study of Ningetinib (CT053PTSA) in Combination With Gefitinib in Stage IIIB or IV NSCLC Patients With EGFR Mutation and T790M Negative Who Have Progressed After EGFR TKI Therapy	I/II
No NCT ID	A Phase Ib/II Umbrella Study to Evaluate the Safety and Efficacy of Varlitinib in Combination with Weekly Paclitaxel in HER1/HER2 Co-Expressing Advanced or Metastatic Gastric Cancer	I/II
NCT03711422	A Dose Finding Study of Continuous and Intermittent High-dose (HDI) Afatinib (EGFR TKI) on CNS Metastases and Leptomeningeal Disease (LMD) in Patients With Advanced Refractory EGFR Mutation Positive Non-small Cell Lung Cancer	I
NCT02609776	A Phase I, First-in-Human, Open-Label, Dose Escalation Study of JNJ-61186372, a Human Bispecific EGFR and cMet Antibody, in Subjects With Advanced Non-Small Cell Lung Cancer.	I
NCT04528836	A Phase I/IB First-in-Human Study of the SHP2 Inhibitor BBP-398 (Formerly Known as IACS-15509) in Patients With Advanced Solid Tumors	I

Clinical Trials Summary (continued)

EGFR A289T mutation (continued)

NCT ID	Title	Phase
No NCT ID	Feasibility Study of Pemetrexed / Bevacizumab / Erlotinib in Chemotherapy Naive Patients With Non-Small Cell Lung Cancer Harboring EGFR Mutation	I
No NCT ID	Phase I Study of DZD9008 in EGFR or HER2 Mutant NSCLC Chinese Patients	I
NCT04077463	An Open-label Phase I/Ib Study to Evaluate the Safety and Pharmacokinetics of JNJ-73841937 (Lazertinib), a Third Generation EGFR-TKI, as Monotherapy or in Combinations With JNJ-61186372, a Human Bispecific EGFR and cMet Antibody in Participants With Advanced Non-Small Cell Lung Cancer	I
NCT04013542	A Pilot Trial of Ipilimumab-Nivolumab in Local-Regionally Advanced Non Small Cell Lung Cancer (NSCLC)	I
NCT04140526	Safety, Pharmacokinetics (PK), and Efficacy of ONC-392 as a Single Agent and in Combination With Pembrolizumab in Advanced Solid Tumors and NSCLC: An Open Label Phase IA/IB Study. Preserve CTLA4 Checkpoint Function (PRESERVE-001)	I
No NCT ID	Study Of Immunologic Factor In Re-Biopsy Specimen, Peritumoral BALF, And The Peripheral Blood For Predicting Response To Osimertinib In NSCLC Patients	I
No NCT ID	Pharmacokinetic and dose finding study of osimertinib in patients with impaired renal function and low body weight	I
NCT03800134	A Phase III, Double-blind, Placebo-controlled, Multi-center International Study of Neoadjuvant/Adjuvant Durvalumab for the Treatment of Patients With Resectable Stages II and III Non-small Cell Lung Cancer (AEGEAN)	III
No NCT ID	A Pilot Study for Apatinib Mesylate Combined with Gefitinib in First-line Treatment of Lung Adenocarcinoma with Malignant Pleural Effusion or Pericardial Effusion	IV
NCT04824079	Study of Kenaitinib in Patients With Advanced Non-small Cell Lung Cancer With Brain Metastasis or Progression of Brain Metastasis After Treatment With EGFR Inhibitor(s)	II
NCT03542799	Phase I/II Study of EGFR-IL12-CART Cells for Patients With Metastatic Colorectal Cancer	I/II
No NCT ID	A Phase Ib Study to Evaluate the Safety and Efficacy of Combination Therapy With Nivolumab and Photoimmunotherapy (PIT) Using ASP-1929 for the Patients With Unresectable Advanced / Recurrent Gastric Cancer or Esophageal Cancer	I
NCT03919292	Phase 1/2 Study of Neratinib and Divalproex Sodium (Valproate) in Advanced Solid Tumors, With an Expansion Cohort in Ras-Mutated Cancers	I/II

Disclaimer: The data presented here is from a curated knowledgebase of publicly available information, but may not be exhaustive. The data version is 2021.11(003).

Sample Cancer Type: Unknown Primary Origin

Table of Contents

Table of Contents	Page
Relevant Therapy Details	**3**
Alert Details	**13**
Clinical Trials	**23**
Relevant Therapy Summary	**193**
Clinical Trials Summary	**210**

Report Highlights

20 Relevant Biomarkers
1 Therapies Available
38 Clinical Trials

Relevant Biomarkers

Genomic Alteration	Relevant Therapies (In this cancer type)	Relevant Therapies (In other cancer type)	Clinical Trials
FGFR3 G370C mutation<br>**Prognostic significance:** None<br>**Diagnostic significance:** None	None	**erdafitinib** [1]	35
FGFR3 R248C mutation<br>**Prognostic significance:** None<br>**Diagnostic significance:** None	None	**erdafitinib** [1]	35
FGFR3 S249C mutation<br>**Prognostic significance:** None<br>**Diagnostic significance:** None	None	**erdafitinib** [1]	35
FGFR3-TACC3 fusion<br>**Prognostic significance:** None<br>**Diagnostic significance:** None	None	**erdafitinib** [1]	31
FGFR3-BAIAP2L1 fusion<br>**Prognostic significance:** None<br>**Diagnostic significance:** None	None	**erdafitinib** [1]	30
FGFR3 Y373C mutation<br>**Prognostic significance:** None<br>**Diagnostic significance:** None	None	**erdafitinib** [1]	29
FGFR3 K650E mutation<br>**Prognostic significance:** None<br>**Diagnostic significance:** None	None	erdafitinib	33
FGFR3 K650M mutation<br>**Prognostic significance:** None<br>**Diagnostic significance:** None	None	erdafitinib	32
FGFR3 K650Q mutation<br>**Prognostic significance:** None<br>**Diagnostic significance:** None	None	erdafitinib	32
FGFR3 K650T mutation<br>**Prognostic significance:** None<br>**Diagnostic significance:** None	None	erdafitinib	32

Public data sources included in relevant therapies: FDA[1], NCCN, **EMA**[2], ESMO
Public data sources included in prognostic and diagnostic significance: NCCN, ESMO

Disclaimer: The data presented here is from a curated knowledgebase of publicly available information, but may not be exhaustive. The data version is 2021.11(003).The content of this report has not been evaluated or approved by the FDA or other regulatory agencies.

Relevant Biomarkers (continued)

Genomic Alteration	Relevant Therapies (In this cancer type)	Relevant Therapies (In other cancer type)	Clinical Trials
FGFR3 activating mutation<br>**Prognostic significance:** None<br>**Diagnostic significance:** None	None	erdafitinib	31
FGFR3 K652E mutation<br>**Prognostic significance:** None<br>**Diagnostic significance:** None	None	erdafitinib	31
FGFR3 fusion<br>**Prognostic significance:** None<br>**Diagnostic significance:** None	None	erdafitinib	27
FGFR3 A391E mutation<br>**Prognostic significance:** None<br>**Diagnostic significance:** None	None	erdafitinib	26
FGFR3 KD mutation<br>**Prognostic significance:** None<br>**Diagnostic significance:** None	None	erdafitinib	25
FGFR3 mutation<br>**Prognostic significance:** None<br>**Diagnostic significance:** None	None	erdafitinib	25
FGFR3 V555 mutation<br>**Prognostic significance:** None<br>**Diagnostic significance:** None	None	erdafitinib	25
FGFR3 V557 mutation<br>**Prognostic significance:** None<br>**Diagnostic significance:** None	None	erdafitinib	25
FGFR3 amplification<br>**Prognostic significance:** None<br>**Diagnostic significance:** None	None	None	21
FGFR3 aberration<br>**Prognostic significance:** None<br>**Diagnostic significance:** None	None	None	13

Public data sources included in relevant therapies: FDA[1], NCCN, **EMA**[2], ESMO
Public data sources included in prognostic and diagnostic significance: NCCN, ESMO

Disclaimer: The data presented here is from a curated knowledgebase of publicly available information, but may not be exhaustive. The data version is 2021.11(003).

Relevant Therapy Details

Current FDA Information

● In this cancer type ○ In other cancer type ◐ In this cancer type and other cancer types

FDA information is current as of 2021-10-13. For the most up-to-date information, search www.fda.gov.

FGFR3 G370C mutation

○ erdafitinib

Cancer type: Bladder Urothelial Carcinoma **Label as of:** 2020-04-02 **Variant class:** FGFR3 G370C mutation

Indications and usage:

BALVERSA® is a kinase inhibitor indicated for the treatment of adult patients with locally advanced or metastatic urothelial carcinoma that has

- susceptible FGFR3 or FGFR2 genetic alterations and
- progressed during or following at least one line of prior platinum-containing chemotherapy including within 12 months of neoadjuvant or adjuvant platinum-containing chemotherapy.

Select patients for therapy based on an FDA-approved companion diagnostic for BALVERSA®.

This indication is approved under accelerated approval based on tumor response rate. Continued approval for this indication may be contingent upon verification and description of clinical benefit in confirmatory trials.

Reference:

https://www.accessdata.fda.gov/drugsatfda_docs/label/2020/212018s001lbl.pdf

FGFR3 R248C mutation

○ erdafitinib

Cancer type: Bladder Urothelial Carcinoma **Label as of:** 2020-04-02 **Variant class:** FGFR3 R248C mutation

Indications and usage:

BALVERSA® is a kinase inhibitor indicated for the treatment of adult patients with locally advanced or metastatic urothelial carcinoma that has

- susceptible FGFR3 or FGFR2 genetic alterations and
- progressed during or following at least one line of prior platinum-containing chemotherapy including within 12 months of neoadjuvant or adjuvant platinum-containing chemotherapy.

Select patients for therapy based on an FDA-approved companion diagnostic for BALVERSA®.

This indication is approved under accelerated approval based on tumor response rate. Continued approval for this indication may be contingent upon verification and description of clinical benefit in confirmatory trials.

Reference:

https://www.accessdata.fda.gov/drugsatfda_docs/label/2020/212018s001lbl.pdf

FGFR3 S249C mutation

○ erdafitinib

Cancer type: Bladder Urothelial Carcinoma **Label as of:** 2020-04-02 **Variant class:** FGFR3 S249C mutation

Indications and usage:

BALVERSA® is a kinase inhibitor indicated for the treatment of adult patients with locally advanced or metastatic urothelial carcinoma that has

- susceptible FGFR3 or FGFR2 genetic alterations and
- progressed during or following at least one line of prior platinum-containing chemotherapy including within 12 months of neoadjuvant or adjuvant platinum-containing chemotherapy.

Select patients for therapy based on an FDA-approved companion diagnostic for BALVERSA®.

This indication is approved under accelerated approval based on tumor response rate. Continued approval for this indication may be contingent upon verification and description of clinical benefit in confirmatory trials.

Reference:

https://www.accessdata.fda.gov/drugsatfda_docs/label/2020/212018s001lbl.pdf

FGFR3-TACC3 fusion

○ erdafitinib

Cancer type: Bladder Urothelial Carcinoma **Label as of:** 2020-04-02 **Variant class:** FGFR3-TACC3 fusion

Indications and usage:

BALVERSA® is a kinase inhibitor indicated for the treatment of adult patients with locally advanced or metastatic urothelial carcinoma that has

- susceptible FGFR3 or FGFR2 genetic alterations and
- progressed during or following at least one line of prior platinum-containing chemotherapy including within 12 months of neoadjuvant or adjuvant platinum-containing chemotherapy.

Select patients for therapy based on an FDA-approved companion diagnostic for BALVERSA®.

This indication is approved under accelerated approval based on tumor response rate. Continued approval for this indication may be contingent upon verification and description of clinical benefit in confirmatory trials.

Reference:

https://www.accessdata.fda.gov/drugsatfda_docs/label/2020/212018s001lbl.pdf

Disclaimer: The data presented here is from a curated knowledgebase of publicly available information, but may not be exhaustive. The data version is 2021.11(003).

FGFR3-BAIAP2L1 fusion

erdafitinib

Cancer type: Bladder Urothelial Carcinoma **Label as of:** 2020-04-02 **Variant class:** FGFR3-BAIAP2L1 fusion

Indications and usage:

BALVERSA® is a kinase inhibitor indicated for the treatment of adult patients with locally advanced or metastatic urothelial carcinoma that has

- susceptible FGFR3 or FGFR2 genetic alterations and
- progressed during or following at least one line of prior platinum-containing chemotherapy including within 12 months of neoadjuvant or adjuvant platinum-containing chemotherapy.

Select patients for therapy based on an FDA-approved companion diagnostic for BALVERSA®.

This indication is approved under accelerated approval based on tumor response rate. Continued approval for this indication may be contingent upon verification and description of clinical benefit in confirmatory trials.

Reference:

https://www.accessdata.fda.gov/drugsatfda_docs/label/2020/212018s001lbl.pdf

FGFR3 Y373C mutation

erdafitinib

Cancer type: Bladder Urothelial Carcinoma **Label as of:** 2020-04-02 **Variant class:** FGFR3 Y373C mutation

Indications and usage:

BALVERSA® is a kinase inhibitor indicated for the treatment of adult patients with locally advanced or metastatic urothelial carcinoma that has

- susceptible FGFR3 or FGFR2 genetic alterations and
- progressed during or following at least one line of prior platinum-containing chemotherapy including within 12 months of neoadjuvant or adjuvant platinum-containing chemotherapy.

Select patients for therapy based on an FDA-approved companion diagnostic for BALVERSA®.

This indication is approved under accelerated approval based on tumor response rate. Continued approval for this indication may be contingent upon verification and description of clinical benefit in confirmatory trials.

Reference:

https://www.accessdata.fda.gov/drugsatfda_docs/label/2020/212018s001lbl.pdf

Disclaimer: The data presented here is from a curated knowledgebase of publicly available information, but may not be exhaustive. The data version is 2021.11(003).

Current NCCN Information

● In this cancer type ○ In other cancer type ◐ In this cancer type and other cancer types

NCCN information is current as of 2021-10-01. For the most up-to-date information, search www.nccn.org.
For NCCN International Adaptations & Translations, search www.nccn.org/global/international_adaptations.aspx.

FGFR3 G370C mutation

○ erdafitinib

Cancer type: Bladder Cancer **Variant class:** FGFR3 G370C mutation

NCCN Recommendation category: 2A

Population segment (Line of therapy):
- Stage IV; Locally Advanced, Metastatic (Second-line therapy); Preferred intervention, Other recommended intervention
- Stage IV; Locally Advanced, Metastatic (Subsequent therapy); Preferred intervention

Reference: NCCN Guidelines® - NCCN-Bladder Cancer [Version 4.2021]

FGFR3 R248C mutation

○ erdafitinib

Cancer type: Bladder Cancer **Variant class:** FGFR3 R248C mutation

NCCN Recommendation category: 2A

Population segment (Line of therapy):
- Stage IV; Locally Advanced, Metastatic (Second-line therapy); Preferred intervention, Other recommended intervention
- Stage IV; Locally Advanced, Metastatic (Subsequent therapy); Preferred intervention

Reference: NCCN Guidelines® - NCCN-Bladder Cancer [Version 4.2021]

FGFR3 S249C mutation

○ erdafitinib

Cancer type: Bladder Cancer **Variant class:** FGFR3 S249C mutation

NCCN Recommendation category: 2A

Population segment (Line of therapy):
- Stage IV; Locally Advanced, Metastatic (Second-line therapy); Preferred intervention, Other recommended intervention
- Stage IV; Locally Advanced, Metastatic (Subsequent therapy); Preferred intervention

Reference: NCCN Guidelines® - NCCN-Bladder Cancer [Version 4.2021]

Disclaimer: The data presented here is from a curated knowledgebase of publicly available information, but may not be exhaustive. The data version is 2021.11(003).

FGFR3-TACC3 fusion

erdafitinib

Cancer type: Bladder Cancer **Variant class:** FGFR3-TACC3 fusion

NCCN Recommendation category: 2A

Population segment (Line of therapy):
- Stage IV; Locally Advanced, Metastatic (Second-line therapy); Preferred intervention, Other recommended intervention
- Stage IV; Locally Advanced, Metastatic (Subsequent therapy); Preferred intervention

Reference: NCCN Guidelines® - NCCN-Bladder Cancer [Version 4.2021]

FGFR3-BAIAP2L1 fusion

erdafitinib

Cancer type: Bladder Cancer **Variant class:** FGFR3-BAIAP2L1 fusion

NCCN Recommendation category: 2A

Population segment (Line of therapy):
- Stage IV; Locally Advanced, Metastatic (Second-line therapy); Preferred intervention, Other recommended intervention
- Stage IV; Locally Advanced, Metastatic (Subsequent therapy); Preferred intervention

Reference: NCCN Guidelines® - NCCN-Bladder Cancer [Version 4.2021]

FGFR3 Y373C mutation

erdafitinib

Cancer type: Bladder Cancer **Variant class:** FGFR3 Y373C mutation

NCCN Recommendation category: 2A

Population segment (Line of therapy):
- Stage IV; Locally Advanced, Metastatic (Second-line therapy); Preferred intervention, Other recommended intervention
- Stage IV; Locally Advanced, Metastatic (Subsequent therapy); Preferred intervention

Reference: NCCN Guidelines® - NCCN-Bladder Cancer [Version 4.2021]

Disclaimer: The data presented here is from a curated knowledgebase of publicly available information, but may not be exhaustive. The data version is 2021.11(003).

Current ESMO Information

● In this cancer type ○ In other cancer type ◐ In this cancer type and other cancer types

ESMO information is current as of 2021-10-01. For the most up-to-date information, search www.esmo.org.

FGFR3 G370C mutation

○ erdafitinib

Cancer type: Bladder Urothelial Carcinoma **Variant class:** FGFR3 mutation

ESMO Level of Evidence/Grade of Recommendation: III / B

Population segment (Line of therapy):
- Advanced, Metastatic, Refractory (Subsequent therapy)

Reference: ESMO Clinical Practice Guidelines - ESMO-Bladder Cancer [Ann Oncol (2014) 25 (suppl 3): iii40-iii48. (eUpdate: 16 July 2020, 16 December 2019, 16 December 2019)]

FGFR3 R248C mutation

○ erdafitinib

Cancer type: Bladder Urothelial Carcinoma **Variant class:** FGFR3 mutation

ESMO Level of Evidence/Grade of Recommendation: III / B

Population segment (Line of therapy):
- Advanced, Metastatic, Refractory (Subsequent therapy)

Reference: ESMO Clinical Practice Guidelines - ESMO-Bladder Cancer [Ann Oncol (2014) 25 (suppl 3): iii40-iii48. (eUpdate: 16 July 2020, 16 December 2019, 16 December 2019)]

FGFR3 S249C mutation

○ erdafitinib

Cancer type: Bladder Urothelial Carcinoma **Variant class:** FGFR3 mutation

ESMO Level of Evidence/Grade of Recommendation: III / B

Population segment (Line of therapy):
- Advanced, Metastatic, Refractory (Subsequent therapy)

Reference: ESMO Clinical Practice Guidelines - ESMO-Bladder Cancer [Ann Oncol (2014) 25 (suppl 3): iii40-iii48. (eUpdate: 16 July 2020, 16 December 2019, 16 December 2019)]

Disclaimer: The data presented here is from a curated knowledgebase of publicly available information, but may not be exhaustive. The data version is 2021.11(003).

FGFR3-TACC3 fusion

○ erdafitinib

Cancer type: Bladder Urothelial Carcinoma **Variant class:** FGFR3 fusion

ESMO Level of Evidence/Grade of Recommendation: III / B

Population segment (Line of therapy):
- Advanced, Metastatic, Refractory (Subsequent therapy)

Reference: ESMO Clinical Practice Guidelines - ESMO-Bladder Cancer [Ann Oncol (2014) 25 (suppl 3): iii40-iii48. (eUpdate: 16 July 2020, 16 December 2019, 16 December 2019)]

FGFR3-BAIAP2L1 fusion

○ erdafitinib

Cancer type: Bladder Urothelial Carcinoma **Variant class:** FGFR3 fusion

ESMO Level of Evidence/Grade of Recommendation: III / B

Population segment (Line of therapy):
- Advanced, Metastatic, Refractory (Subsequent therapy)

Reference: ESMO Clinical Practice Guidelines - ESMO-Bladder Cancer [Ann Oncol (2014) 25 (suppl 3): iii40-iii48. (eUpdate: 16 July 2020, 16 December 2019, 16 December 2019)]

FGFR3 Y373C mutation

○ erdafitinib

Cancer type: Bladder Urothelial Carcinoma **Variant class:** FGFR3 mutation

ESMO Level of Evidence/Grade of Recommendation: III / B

Population segment (Line of therapy):
- Advanced, Metastatic, Refractory (Subsequent therapy)

Reference: ESMO Clinical Practice Guidelines - ESMO-Bladder Cancer [Ann Oncol (2014) 25 (suppl 3): iii40-iii48. (eUpdate: 16 July 2020, 16 December 2019, 16 December 2019)]

FGFR3 K650E mutation

○ erdafitinib

Cancer type: Bladder Urothelial Carcinoma **Variant class:** FGFR3 mutation

ESMO Level of Evidence/Grade of Recommendation: III / B

Population segment (Line of therapy):
- Advanced, Metastatic, Refractory (Subsequent therapy)

Reference: ESMO Clinical Practice Guidelines - ESMO-Bladder Cancer [Ann Oncol (2014) 25 (suppl 3): iii40-iii48. (eUpdate: 16 July 2020, 16 December 2019, 16 December 2019)]

Disclaimer: The data presented here is from a curated knowledgebase of publicly available information, but may not be exhaustive. The data version is 2021.11(003).

FGFR3 K650M mutation

○ erdafitinib

Cancer type: Bladder Urothelial Carcinoma **Variant class:** FGFR3 mutation

ESMO Level of Evidence/Grade of Recommendation: III / B

Population segment (Line of therapy):

- Advanced, Metastatic, Refractory (Subsequent therapy)

Reference: ESMO Clinical Practice Guidelines - ESMO-Bladder Cancer [Ann Oncol (2014) 25 (suppl 3): iii40-iii48. (eUpdate: 16 July 2020, 16 December 2019, 16 December 2019)]

FGFR3 K650Q mutation

○ erdafitinib

Cancer type: Bladder Urothelial Carcinoma **Variant class:** FGFR3 mutation

ESMO Level of Evidence/Grade of Recommendation: III / B

Population segment (Line of therapy):

- Advanced, Metastatic, Refractory (Subsequent therapy)

Reference: ESMO Clinical Practice Guidelines - ESMO-Bladder Cancer [Ann Oncol (2014) 25 (suppl 3): iii40-iii48. (eUpdate: 16 July 2020, 16 December 2019, 16 December 2019)]

FGFR3 K650T mutation

○ erdafitinib

Cancer type: Bladder Urothelial Carcinoma **Variant class:** FGFR3 mutation

ESMO Level of Evidence/Grade of Recommendation: III / B

Population segment (Line of therapy):

- Advanced, Metastatic, Refractory (Subsequent therapy)

Reference: ESMO Clinical Practice Guidelines - ESMO-Bladder Cancer [Ann Oncol (2014) 25 (suppl 3): iii40-iii48. (eUpdate: 16 July 2020, 16 December 2019, 16 December 2019)]

FGFR3 activating mutation

○ erdafitinib

Cancer type: Bladder Urothelial Carcinoma **Variant class:** FGFR3 mutation

ESMO Level of Evidence/Grade of Recommendation: III / B

Population segment (Line of therapy):

- Advanced, Metastatic, Refractory (Subsequent therapy)

Reference: ESMO Clinical Practice Guidelines - ESMO-Bladder Cancer [Ann Oncol (2014) 25 (suppl 3): iii40-iii48. (eUpdate: 16 July 2020, 16 December 2019, 16 December 2019)]

Disclaimer: The data presented here is from a curated knowledgebase of publicly available information, but may not be exhaustive. The data version is 2021.11(003).

FGFR3 K652E mutation

erdafitinib

Cancer type: Bladder Urothelial Carcinoma **Variant class:** FGFR3 mutation

ESMO Level of Evidence/Grade of Recommendation: III / B

Population segment (Line of therapy):
- Advanced, Metastatic, Refractory (Subsequent therapy)

Reference: ESMO Clinical Practice Guidelines - ESMO-Bladder Cancer [Ann Oncol (2014) 25 (suppl 3): iii40-iii48. (eUpdate: 16 July 2020, 16 December 2019, 16 December 2019)]

FGFR3 fusion

erdafitinib

Cancer type: Bladder Urothelial Carcinoma **Variant class:** FGFR3 fusion

ESMO Level of Evidence/Grade of Recommendation: III / B

Population segment (Line of therapy):
- Advanced, Metastatic, Refractory (Subsequent therapy)

Reference: ESMO Clinical Practice Guidelines - ESMO-Bladder Cancer [Ann Oncol (2014) 25 (suppl 3): iii40-iii48. (eUpdate: 16 July 2020, 16 December 2019, 16 December 2019)]

FGFR3 A391E mutation

erdafitinib

Cancer type: Bladder Urothelial Carcinoma **Variant class:** FGFR3 mutation

ESMO Level of Evidence/Grade of Recommendation: III / B

Population segment (Line of therapy):
- Advanced, Metastatic, Refractory (Subsequent therapy)

Reference: ESMO Clinical Practice Guidelines - ESMO-Bladder Cancer [Ann Oncol (2014) 25 (suppl 3): iii40-iii48. (eUpdate: 16 July 2020, 16 December 2019, 16 December 2019)]

FGFR3 KD mutation

erdafitinib

Cancer type: Bladder Urothelial Carcinoma **Variant class:** FGFR3 mutation

ESMO Level of Evidence/Grade of Recommendation: III / B

Population segment (Line of therapy):
- Advanced, Metastatic, Refractory (Subsequent therapy)

Reference: ESMO Clinical Practice Guidelines - ESMO-Bladder Cancer [Ann Oncol (2014) 25 (suppl 3): iii40-iii48. (eUpdate: 16 July 2020, 16 December 2019, 16 December 2019)]

Disclaimer: The data presented here is from a curated knowledgebase of publicly available information, but may not be exhaustive. The data version is 2021.11(003).

FGFR3 mutation

erdafitinib

Cancer type: Bladder Urothelial Carcinoma **Variant class:** FGFR3 mutation

ESMO Level of Evidence/Grade of Recommendation: III / B

Population segment (Line of therapy):
- Advanced, Metastatic, Refractory (Subsequent therapy)

Reference: ESMO Clinical Practice Guidelines - ESMO-Bladder Cancer [Ann Oncol (2014) 25 (suppl 3): iii40-iii48. (eUpdate: 16 July 2020, 16 December 2019, 16 December 2019)]

FGFR3 V555 mutation

erdafitinib

Cancer type: Bladder Urothelial Carcinoma **Variant class:** FGFR3 mutation

ESMO Level of Evidence/Grade of Recommendation: III / B

Population segment (Line of therapy):
- Advanced, Metastatic, Refractory (Subsequent therapy)

Reference: ESMO Clinical Practice Guidelines - ESMO-Bladder Cancer [Ann Oncol (2014) 25 (suppl 3): iii40-iii48. (eUpdate: 16 July 2020, 16 December 2019, 16 December 2019)]

FGFR3 V557 mutation

erdafitinib

Cancer type: Bladder Urothelial Carcinoma **Variant class:** FGFR3 mutation

ESMO Level of Evidence/Grade of Recommendation: III / B

Population segment (Line of therapy):
- Advanced, Metastatic, Refractory (Subsequent therapy)

Reference: ESMO Clinical Practice Guidelines - ESMO-Bladder Cancer [Ann Oncol (2014) 25 (suppl 3): iii40-iii48. (eUpdate: 16 July 2020, 16 December 2019, 16 December 2019)]

Disclaimer: The data presented here is from a curated knowledgebase of publicly available information, but may not be exhaustive. The data version is 2021.11(003).

Alerts Informed By Public Data Sources

Current FDA Information

Contraindicated | Not recommended | Resistance | Breakthrough | Fast Track

FDA information is current as of 2021-10-13. For the most up-to-date information, search www.fda.gov.

FGFR3 G370C mutation

vofatamab

Cancer type: Bladder Urothelial Carcinoma **Variant class:** FGFR3 mutation

Supporting Statement:
The FDA has granted Fast Track Designation to the FGFR3-targeted monoclonal antibody, vofatamab, for FGFR3 mutations or fusions in advanced or metastatic urothelial carcinoma.

Reference:

https://www.healio.com/news/hematology-oncology/20190107/fda-grants-fast-track-designation-to-vofatamab-for-bladder-cancer-subset

Debio 1347

Cancer type: Solid Tumor **Variant class:** FGFR3 aberration

Supporting Statement:
The FDA has granted Fast Track Designation to the FGFR 1-3 inhibitor, debio 1347, for FGFR1/2/3 alterations in unresectable or metastatic solid tumors.

Reference:

https://www.debiopharm.com/drug-development/press-releases/fda-grants-fast-track-designation-to-debiopharm-internationals-debio-1347-for-the-treatment-of-patients-with-unresectable-or-metastatic-tumors-with-a-specific-fgfr-gene-alteration/

FGFR3 R248C mutation

vofatamab

Cancer type: Bladder Urothelial Carcinoma **Variant class:** FGFR3 mutation

Supporting Statement:
The FDA has granted Fast Track Designation to the FGFR3-targeted monoclonal antibody, vofatamab, for FGFR3 mutations or fusions in advanced or metastatic urothelial carcinoma.

Reference:

https://www.healio.com/news/hematology-oncology/20190107/fda-grants-fast-track-designation-to-vofatamab-for-bladder-cancer-subset

Disclaimer: The data presented here is from a curated knowledgebase of publicly available information, but may not be exhaustive. The data version is 2021.11(003).

FGFR3 R248C mutation (continued)

Debio 1347

Cancer type: Solid Tumor **Variant class:** FGFR3 aberration

Supporting Statement:
The FDA has granted Fast Track Designation to the FGFR 1-3 inhibitor, debio 1347, for FGFR1/2/3 alterations in unresectable or metastatic solid tumors.

Reference:

https://www.debiopharm.com/drug-development/press-releases/fda-grants-fast-track-designation-to-debiopharm-internationals-debio-1347-for-the-treatment-of-patients-with-unresectable-or-metastatic-tumors-with-a-specific-fgfr-gene-alteration/

FGFR3 S249C mutation

vofatamab

Cancer type: Bladder Urothelial Carcinoma **Variant class:** FGFR3 mutation

Supporting Statement:
The FDA has granted Fast Track Designation to the FGFR3-targeted monoclonal antibody, vofatamab, for FGFR3 mutations or fusions in advanced or metastatic urothelial carcinoma.

Reference:

https://www.healio.com/news/hematology-oncology/20190107/fda-grants-fast-track-designation-to-vofatamab-for-bladder-cancer-subset

Debio 1347

Cancer type: Solid Tumor **Variant class:** FGFR3 aberration

Supporting Statement:
The FDA has granted Fast Track Designation to the FGFR 1-3 inhibitor, debio 1347, for FGFR1/2/3 alterations in unresectable or metastatic solid tumors.

Reference:

https://www.debiopharm.com/drug-development/press-releases/fda-grants-fast-track-designation-to-debiopharm-internationals-debio-1347-for-the-treatment-of-patients-with-unresectable-or-metastatic-tumors-with-a-specific-fgfr-gene-alteration/

FGFR3-TACC3 fusion

vofatamab

Cancer type: Bladder Urothelial Carcinoma **Variant class:** FGFR3 fusion

Supporting Statement:
The FDA has granted Fast Track Designation to the FGFR3-targeted monoclonal antibody, vofatamab, for FGFR3 mutations or fusions in advanced or metastatic urothelial carcinoma.

Reference:

https://www.healio.com/news/hematology-oncology/20190107/fda-grants-fast-track-designation-to-vofatamab-for-bladder-cancer-subset

Disclaimer: The data presented here is from a curated knowledgebase of publicly available information, but may not be exhaustive. The data version is 2021.11(003).

FGFR3-TACC3 fusion (continued)

Debio 1347

Cancer type: Solid Tumor **Variant class:** FGFR3 aberration

Supporting Statement:
The FDA has granted Fast Track Designation to the FGFR 1-3 inhibitor, debio 1347, for FGFR1/2/3 alterations in unresectable or metastatic solid tumors.

Reference:

https://www.debiopharm.com/drug-development/press-releases/fda-grants-fast-track-designation-to-debiopharm-internationals-debio-1347-for-the-treatment-of-patients-with-unresectable-or-metastatic-tumors-with-a-specific-fgfr-gene-alteration/

FGFR3-BAIAP2L1 fusion

vofatamab

Cancer type: Bladder Urothelial Carcinoma **Variant class:** FGFR3 fusion

Supporting Statement:
The FDA has granted Fast Track Designation to the FGFR3-targeted monoclonal antibody, vofatamab, for FGFR3 mutations or fusions in advanced or metastatic urothelial carcinoma.

Reference:

https://www.healio.com/news/hematology-oncology/20190107/fda-grants-fast-track-designation-to-vofatamab-for-bladder-cancer-subset

Debio 1347

Cancer type: Solid Tumor **Variant class:** FGFR3 aberration

Supporting Statement:
The FDA has granted Fast Track Designation to the FGFR 1-3 inhibitor, debio 1347, for FGFR1/2/3 alterations in unresectable or metastatic solid tumors.

Reference:

https://www.debiopharm.com/drug-development/press-releases/fda-grants-fast-track-designation-to-debiopharm-internationals-debio-1347-for-the-treatment-of-patients-with-unresectable-or-metastatic-tumors-with-a-specific-fgfr-gene-alteration/

FGFR3 Y373C mutation

vofatamab

Cancer type: Bladder Urothelial Carcinoma **Variant class:** FGFR3 mutation

Supporting Statement:
The FDA has granted Fast Track Designation to the FGFR3-targeted monoclonal antibody, vofatamab, for FGFR3 mutations or fusions in advanced or metastatic urothelial carcinoma.

Reference:

https://www.healio.com/news/hematology-oncology/20190107/fda-grants-fast-track-designation-to-vofatamab-for-bladder-cancer-subset

Disclaimer: The data presented here is from a curated knowledgebase of publicly available information, but may not be exhaustive. The data version is 2021.11(003).

FGFR3 Y373C mutation (continued)

Debio 1347

Cancer type: Solid Tumor **Variant class:** FGFR3 aberration

Supporting Statement:
The FDA has granted Fast Track Designation to the FGFR 1-3 inhibitor, debio 1347, for FGFR1/2/3 alterations in unresectable or metastatic solid tumors.

Reference:

https://www.debiopharm.com/drug-development/press-releases/fda-grants-fast-track-designation-to-debiopharm-internationals-debio-1347-for-the-treatment-of-patients-with-unresectable-or-metastatic-tumors-with-a-specific-fgfr-gene-alteration/

FGFR3 K650E mutation

vofatamab

Cancer type: Bladder Urothelial Carcinoma **Variant class:** FGFR3 mutation

Supporting Statement:
The FDA has granted Fast Track Designation to the FGFR3-targeted monoclonal antibody, vofatamab, for FGFR3 mutations or fusions in advanced or metastatic urothelial carcinoma.

Reference:

https://www.healio.com/news/hematology-oncology/20190107/fda-grants-fast-track-designation-to-vofatamab-for-bladder-cancer-subset

Debio 1347

Cancer type: Solid Tumor **Variant class:** FGFR3 aberration

Supporting Statement:
The FDA has granted Fast Track Designation to the FGFR 1-3 inhibitor, debio 1347, for FGFR1/2/3 alterations in unresectable or metastatic solid tumors.

Reference:

https://www.debiopharm.com/drug-development/press-releases/fda-grants-fast-track-designation-to-debiopharm-internationals-debio-1347-for-the-treatment-of-patients-with-unresectable-or-metastatic-tumors-with-a-specific-fgfr-gene-alteration/

FGFR3 K650M mutation

vofatamab

Cancer type: Bladder Urothelial Carcinoma **Variant class:** FGFR3 mutation

Supporting Statement:
The FDA has granted Fast Track Designation to the FGFR3-targeted monoclonal antibody, vofatamab, for FGFR3 mutations or fusions in advanced or metastatic urothelial carcinoma.

Reference:

https://www.healio.com/news/hematology-oncology/20190107/fda-grants-fast-track-designation-to-vofatamab-for-bladder-cancer-subset

Disclaimer: The data presented here is from a curated knowledgebase of publicly available information, but may not be exhaustive. The data version is 2021.11(003).

FGFR3 K650M mutation (continued)

Debio 1347

Cancer type: Solid Tumor **Variant class:** FGFR3 aberration

Supporting Statement:
The FDA has granted Fast Track Designation to the FGFR 1-3 inhibitor, debio 1347, for FGFR1/2/3 alterations in unresectable or metastatic solid tumors.

Reference:

https://www.debiopharm.com/drug-development/press-releases/fda-grants-fast-track-designation-to-debiopharm-internationals-debio-1347-for-the-treatment-of-patients-with-unresectable-or-metastatic-tumors-with-a-specific-fgfr-gene-alteration/

FGFR3 K650Q mutation

vofatamab

Cancer type: Bladder Urothelial Carcinoma **Variant class:** FGFR3 mutation

Supporting Statement:
The FDA has granted Fast Track Designation to the FGFR3-targeted monoclonal antibody, vofatamab, for FGFR3 mutations or fusions in advanced or metastatic urothelial carcinoma.

Reference:

https://www.healio.com/news/hematology-oncology/20190107/fda-grants-fast-track-designation-to-vofatamab-for-bladder-cancer-subset

Debio 1347

Cancer type: Solid Tumor **Variant class:** FGFR3 aberration

Supporting Statement:
The FDA has granted Fast Track Designation to the FGFR 1-3 inhibitor, debio 1347, for FGFR1/2/3 alterations in unresectable or metastatic solid tumors.

Reference:

https://www.debiopharm.com/drug-development/press-releases/fda-grants-fast-track-designation-to-debiopharm-internationals-debio-1347-for-the-treatment-of-patients-with-unresectable-or-metastatic-tumors-with-a-specific-fgfr-gene-alteration/

FGFR3 K650T mutation

vofatamab

Cancer type: Bladder Urothelial Carcinoma **Variant class:** FGFR3 mutation

Supporting Statement:
The FDA has granted Fast Track Designation to the FGFR3-targeted monoclonal antibody, vofatamab, for FGFR3 mutations or fusions in advanced or metastatic urothelial carcinoma.

Reference:

https://www.healio.com/news/hematology-oncology/20190107/fda-grants-fast-track-designation-to-vofatamab-for-bladder-cancer-subset

FGFR3 K650T mutation (continued)

Debio 1347

Cancer type: Solid Tumor **Variant class:** FGFR3 aberration

Supporting Statement:
The FDA has granted Fast Track Designation to the FGFR 1-3 inhibitor, debio 1347, for FGFR1/2/3 alterations in unresectable or metastatic solid tumors.

Reference:

https://www.debiopharm.com/drug-development/press-releases/fda-grants-fast-track-designation-to-debiopharm-internationals-debio-1347-for-the-treatment-of-patients-with-unresectable-or-metastatic-tumors-with-a-specific-fgfr-gene-alteration/

FGFR3 activating mutation

vofatamab

Cancer type: Bladder Urothelial Carcinoma **Variant class:** FGFR3 mutation

Supporting Statement:
The FDA has granted Fast Track Designation to the FGFR3-targeted monoclonal antibody, vofatamab, for FGFR3 mutations or fusions in advanced or metastatic urothelial carcinoma.

Reference:

https://www.healio.com/news/hematology-oncology/20190107/fda-grants-fast-track-designation-to-vofatamab-for-bladder-cancer-subset

Debio 1347

Cancer type: Solid Tumor **Variant class:** FGFR3 aberration

Supporting Statement:
The FDA has granted Fast Track Designation to the FGFR 1-3 inhibitor, debio 1347, for FGFR1/2/3 alterations in unresectable or metastatic solid tumors.

Reference:

https://www.debiopharm.com/drug-development/press-releases/fda-grants-fast-track-designation-to-debiopharm-internationals-debio-1347-for-the-treatment-of-patients-with-unresectable-or-metastatic-tumors-with-a-specific-fgfr-gene-alteration/

FGFR3 K652E mutation

vofatamab

Cancer type: Bladder Urothelial Carcinoma **Variant class:** FGFR3 mutation

Supporting Statement:
The FDA has granted Fast Track Designation to the FGFR3-targeted monoclonal antibody, vofatamab, for FGFR3 mutations or fusions in advanced or metastatic urothelial carcinoma.

Reference:

https://www.healio.com/news/hematology-oncology/20190107/fda-grants-fast-track-designation-to-vofatamab-for-bladder-cancer-subset

Disclaimer: The data presented here is from a curated knowledgebase of publicly available information, but may not be exhaustive. The data version is 2021.11(003).

FGFR3 K652E mutation (continued)

Debio 1347

Cancer type: Solid Tumor **Variant class:** FGFR3 aberration

Supporting Statement:
The FDA has granted Fast Track Designation to the FGFR 1-3 inhibitor, debio 1347, for FGFR1/2/3 alterations in unresectable or metastatic solid tumors.

Reference:

https://www.debiopharm.com/drug-development/press-releases/fda-grants-fast-track-designation-to-debiopharm-internationals-debio-1347-for-the-treatment-of-patients-with-unresectable-or-metastatic-tumors-with-a-specific-fgfr-gene-alteration/

FGFR3 fusion

vofatamab

Cancer type: Bladder Urothelial Carcinoma **Variant class:** FGFR3 fusion

Supporting Statement:
The FDA has granted Fast Track Designation to the FGFR3-targeted monoclonal antibody, vofatamab, for FGFR3 mutations or fusions in advanced or metastatic urothelial carcinoma.

Reference:

https://www.healio.com/news/hematology-oncology/20190107/fda-grants-fast-track-designation-to-vofatamab-for-bladder-cancer-subset

Debio 1347

Cancer type: Solid Tumor **Variant class:** FGFR3 aberration

Supporting Statement:
The FDA has granted Fast Track Designation to the FGFR 1-3 inhibitor, debio 1347, for FGFR1/2/3 alterations in unresectable or metastatic solid tumors.

Reference:

https://www.debiopharm.com/drug-development/press-releases/fda-grants-fast-track-designation-to-debiopharm-internationals-debio-1347-for-the-treatment-of-patients-with-unresectable-or-metastatic-tumors-with-a-specific-fgfr-gene-alteration/

FGFR3 A391E mutation

vofatamab

Cancer type: Bladder Urothelial Carcinoma **Variant class:** FGFR3 mutation

Supporting Statement:
The FDA has granted Fast Track Designation to the FGFR3-targeted monoclonal antibody, vofatamab, for FGFR3 mutations or fusions in advanced or metastatic urothelial carcinoma.

Reference:

https://www.healio.com/news/hematology-oncology/20190107/fda-grants-fast-track-designation-to-vofatamab-for-bladder-cancer-subset

Disclaimer: The data presented here is from a curated knowledgebase of publicly available information, but may not be exhaustive. The data version is 2021.11(003).

FGFR3 A391E mutation (continued)

Debio 1347

Cancer type: Solid Tumor **Variant class:** FGFR3 aberration

Supporting Statement:
The FDA has granted Fast Track Designation to the FGFR 1-3 inhibitor, debio 1347, for FGFR1/2/3 alterations in unresectable or metastatic solid tumors.

Reference:

https://www.debiopharm.com/drug-development/press-releases/fda-grants-fast-track-designation-to-debiopharm-internationals-debio-1347-for-the-treatment-of-patients-with-unresectable-or-metastatic-tumors-with-a-specific-fgfr-gene-alteration/

FGFR3 KD mutation

vofatamab

Cancer type: Bladder Urothelial Carcinoma **Variant class:** FGFR3 mutation

Supporting Statement:
The FDA has granted Fast Track Designation to the FGFR3-targeted monoclonal antibody, vofatamab, for FGFR3 mutations or fusions in advanced or metastatic urothelial carcinoma.

Reference:

https://www.healio.com/news/hematology-oncology/20190107/fda-grants-fast-track-designation-to-vofatamab-for-bladder-cancer-subset

Debio 1347

Cancer type: Solid Tumor **Variant class:** FGFR3 aberration

Supporting Statement:
The FDA has granted Fast Track Designation to the FGFR 1-3 inhibitor, debio 1347, for FGFR1/2/3 alterations in unresectable or metastatic solid tumors.

Reference:

https://www.debiopharm.com/drug-development/press-releases/fda-grants-fast-track-designation-to-debiopharm-internationals-debio-1347-for-the-treatment-of-patients-with-unresectable-or-metastatic-tumors-with-a-specific-fgfr-gene-alteration/

FGFR3 mutation

vofatamab

Cancer type: Bladder Urothelial Carcinoma **Variant class:** FGFR3 mutation

Supporting Statement:
The FDA has granted Fast Track Designation to the FGFR3-targeted monoclonal antibody, vofatamab, for FGFR3 mutations or fusions in advanced or metastatic urothelial carcinoma.

Reference:

https://www.healio.com/news/hematology-oncology/20190107/fda-grants-fast-track-designation-to-vofatamab-for-bladder-cancer-subset

FGFR3 mutation (continued)

Debio 1347

Cancer type: Solid Tumor **Variant class:** FGFR3 aberration

Supporting Statement:
The FDA has granted Fast Track Designation to the FGFR 1-3 inhibitor, debio 1347, for FGFR1/2/3 alterations in unresectable or metastatic solid tumors.

Reference:

https://www.debiopharm.com/drug-development/press-releases/fda-grants-fast-track-designation-to-debiopharm-internationals-debio-1347-for-the-treatment-of-patients-with-unresectable-or-metastatic-tumors-with-a-specific-fgfr-gene-alteration/

FGFR3 V555 mutation

vofatamab

Cancer type: Bladder Urothelial Carcinoma **Variant class:** FGFR3 mutation

Supporting Statement:
The FDA has granted Fast Track Designation to the FGFR3-targeted monoclonal antibody, vofatamab, for FGFR3 mutations or fusions in advanced or metastatic urothelial carcinoma.

Reference:

https://www.healio.com/news/hematology-oncology/20190107/fda-grants-fast-track-designation-to-vofatamab-for-bladder-cancer-subset

Debio 1347

Cancer type: Solid Tumor **Variant class:** FGFR3 aberration

Supporting Statement:
The FDA has granted Fast Track Designation to the FGFR 1-3 inhibitor, debio 1347, for FGFR1/2/3 alterations in unresectable or metastatic solid tumors.

Reference:

https://www.debiopharm.com/drug-development/press-releases/fda-grants-fast-track-designation-to-debiopharm-internationals-debio-1347-for-the-treatment-of-patients-with-unresectable-or-metastatic-tumors-with-a-specific-fgfr-gene-alteration/

FGFR3 V557 mutation

vofatamab

Cancer type: Bladder Urothelial Carcinoma **Variant class:** FGFR3 mutation

Supporting Statement:
The FDA has granted Fast Track Designation to the FGFR3-targeted monoclonal antibody, vofatamab, for FGFR3 mutations or fusions in advanced or metastatic urothelial carcinoma.

Reference:

https://www.healio.com/news/hematology-oncology/20190107/fda-grants-fast-track-designation-to-vofatamab-for-bladder-cancer-subset

Disclaimer: The data presented here is from a curated knowledgebase of publicly available information, but may not be exhaustive. The data version is 2021.11(003).

FGFR3 V557 mutation (continued)

Debio 1347

Cancer type: Solid Tumor **Variant class:** FGFR3 aberration

Supporting Statement:
The FDA has granted Fast Track Designation to the FGFR 1-3 inhibitor, debio 1347, for FGFR1/2/3 alterations in unresectable or metastatic solid tumors.

Reference:

https://www.debiopharm.com/drug-development/press-releases/fda-grants-fast-track-designation-to-debiopharm-internationals-debio-1347-for-the-treatment-of-patients-with-unresectable-or-metastatic-tumors-with-a-specific-fgfr-gene-alteration/

FGFR3 amplification

Debio 1347

Cancer type: Solid Tumor **Variant class:** FGFR3 aberration

Supporting Statement:
The FDA has granted Fast Track Designation to the FGFR 1-3 inhibitor, debio 1347, for FGFR1/2/3 alterations in unresectable or metastatic solid tumors.

Reference:

https://www.debiopharm.com/drug-development/press-releases/fda-grants-fast-track-designation-to-debiopharm-internationals-debio-1347-for-the-treatment-of-patients-with-unresectable-or-metastatic-tumors-with-a-specific-fgfr-gene-alteration/

FGFR3 aberration

Debio 1347

Cancer type: Solid Tumor **Variant class:** FGFR3 aberration

Supporting Statement:
The FDA has granted Fast Track Designation to the FGFR 1-3 inhibitor, debio 1347, for FGFR1/2/3 alterations in unresectable or metastatic solid tumors.

Reference:

https://www.debiopharm.com/drug-development/press-releases/fda-grants-fast-track-designation-to-debiopharm-internationals-debio-1347-for-the-treatment-of-patients-with-unresectable-or-metastatic-tumors-with-a-specific-fgfr-gene-alteration/

Disclaimer: The data presented here is from a curated knowledgebase of publicly available information, but may not be exhaustive. The data version is 2021.11(003).

Current Clinical Trials Information

Clinical Trials information is current as of 2021-10-01. For the most up-to-date information regarding a particular trial, search www.clinicaltrials.gov by NCT ID or search local clinical trials authority website by local identifier listed in 'Other identifiers'.

FGFR3 G370C mutation

NCT05019794
A Phase IIa of Infigratinib in Subjects With Locally Advanced or Metastatic Gastric Cancer or Gastroesophageal Junction Adenocarcinoma With FGFR2 Amplification or Other Advanced Solid Tumors With Other FGFR Alterations

Cancer type: Gastric Cancer, Gastroesophageal Junction Adenocarcinoma, Unspecified Solid Tumor

Variant class: FGFR3 activating mutation

Other identifiers: CTR20210275, LB1001-201

Population segments: FGFR, Fourth line or greater, Second line, Stage III, Stage IV, Third line

Phase: II

Therapy: infigratinib

Location: China

NCT03155620
NCI-COG Pediatric MATCH (Molecular Analysis for Therapy Choice) Screening Protocol

Cancer type: Unspecified Solid Tumor

Variant class: FGFR3 mutation

Other identifiers: 17-729, 2017-0625, 20170841, APEC1621SC, COGAPEC1621, COGAPEC1621SC, N 55017 SC, NCI-2017-01251, NCI-COG Pediatric MATCH, Pediatric MATCH

Population segments: ALK, Aggressive, BRAF, FGFR, HRAS, Indolent, KRAS, NRAS, Pediatric or Adolescent, Second line, Stage III, Stage IV

Phase: II

Therapy: erdafitinib

Locations: Puerto Rico, United States

US States: AK, AL, AR, AZ, CA, CO, CT, DC, DE, FL, GA, HI, IA, ID, IL, IN, KY, LA, MA, MD, ME, MI, MN, MO, MS, NC, NE, NH, NJ, NV, NY, OH, OK, OR, PA, RI, SC, SD, TN, TX, UT, VA, VT, WA, WI

Contact: Multiple contacts: See www.clinicaltrials.gov for complete list of contacts.

NCT03210714
NCI-COG Pediatric MATCH (Molecular Analysis for Therapy Choice) - Phase II Subprotocol of JNJ-42756493 (Erdafitinib) in Patients With Tumors Harboring FGFR 1/2/3/4 Alterations

Cancer type: Unspecified Solid Tumor

Variant class: FGFR3 mutation

Other identifiers: APEC1621B, NCI-2017-01159, NCI-COG Pediatric MATCH, Pediatric MATCH

Population segments: Aggressive, FGFR, Indolent, Pediatric or Adolescent, Second line, Stage III, Stage IV

Phase: II

Therapy: erdafitinib

Locations: Puerto Rico, United States

US States: AK, AL, AR, AZ, CA, CO, CT, DC, DE, FL, GA, IA, ID, IL, IN, KY, LA, MD, ME, MI, MN, MO, MS, NC, ND, NE, NJ, NY, OH, OK, OR, PA, SC, SD, TN, TX, UT, VA, VT, WA, WI

Contact: Multiple contacts: See www.clinicaltrials.gov for complete list of contacts.

FGFR3 G370C mutation (continued)

No NCT ID - see other identifier(s)
A Multicenter Phase II Basket-type Clinical Trial to Evaluate Efficacy and Safety of TAS-120 in Patients with Advanced Solid Malignancies with FGFR Alterations in Circulating Tumor DNA

Cancer type: Unspecified Solid Tumor

Variant class: FGFR3 mutation

Other identifiers: EPOC 1805, JapicCTI-194624, JapicCTI-194796, TiFFANY

Population segments: Second line, Stage III, Stage IV

Phase: II

Therapy: futibatinib

Location: Japan

NCT04233567
A Phase II Study of Oral Infigratinib in Adult Patients With Advanced or Metastatic Solid Tumors With FGFR1-3 Gene Fusions or Other FGFR Genetic Alterations

Cancer type: Unspecified Solid Tumor

Variant class: FGFR activating mutation

Other identifiers: NCI-2019-06869, OSU-19041

Population segments: Second line, Stage III, Stage IV

Phase: II

Therapy: infigratinib

Location: United States

US State: OH

Contact: The Ohio State University Comprehensive Cancer Center [800-293-5066; OSUCCCClinicaltrials@osumc.edu]

NCT02693535
Targeted Agent and Profiling Utilization Registry (TAPUR) Study.

Cancer type: Unspecified Solid Tumor

Variant class: FGFR3 mutation

Other identifiers: 20170529, NCI-2017-00510, Pro00014171, TAPUR

Population segments: Aggressive, BRAF, Diffuse large B-cell lymphoma (DLBCL), Extranodal marginal zone B-cell lymphoma (MALT), FGFR, First line, Follicular lymphoma (FL), Fourth line or greater, Indolent, Lymphoblastic lymphoma (LBL), Mantle cell lymphoma (MCL), Other subtype, Second line, Small lymphocytic lymphoma (SLL), Stage III, Stage IV, Third line, Waldenstrom's macroglobulinemia (WM)

Phase: II

Therapy: sunitinib

Location: United States

US States: AL, AZ, CA, FL, GA, HI, IL, IN, ME, MI, NC, ND, NE, NH, OH, OR, PA, SD, TX, UT, VA, WA

Contact: Pam Mangat [pam.mangat@asco.org]

Disclaimer: The data presented here is from a curated knowledgebase of publicly available information, but may not be exhaustive. The data version is 2021.11(003).

FGFR3 G370C mutation (continued)

NCT04591431
The Rome Trial From Histology to Target: the Road to Personalize Target Therapy and Immunotherapy

Cancer type: Biliary Tract Carcinoma, Breast Cancer, Colorectal Cancer, Esophageal Cancer, Gastric Cancer, Gastroesophageal Junction Adenocarcinoma, Hepatocellular Carcinoma, Liver Cancer, Non-Small Cell Lung Cancer, Pancreatic Cancer, Unspecified Solid Tumor

Variant class: FGFR3 aberration

Other identifiers: EudraCT Number: 2018-002190-21, MAR-BAS-18-005, ROME

Population segments: Second line, Stage IV

Phase: II

Therapy: pemigatinib

Location: Italy

NCT02691793
Study to Evaluate the Safety and Efficacy of Sunitinib, in Subject With Refractory Solid Tumors

Cancer type: Unspecified Solid Tumor

Variant class: FGFR mutation

Other identifier: 2016-02-098

Population segments: (N/A), FGFR, Second line

Phase: II

Therapy: sunitinib

Location: Republic of Korea

NCT03297606
Canadian Profiling and Targeted Agent Utilization Trial (CAPTUR): A Phase II Basket Trial

Cancer type: Unspecified Solid Tumor

Variant class: FGFR3 aberration

Other identifiers: CA209-9DL, CAPTUR, ESR-17-12831, ML39800, PM1, WI233446

Population segments: Aggressive, Diffuse large B-cell lymphoma (DLBCL), Extranodal marginal zone B-cell lymphoma (MALT), First line, Follicular lymphoma (FL), Indolent, Lymphoblastic lymphoma (LBL), Mantle cell lymphoma (MCL), Other subtype, Second line, Stage III, Stage IV, Waldenstrom's macroglobulinemia (WM)

Phase: II

Therapy: sunitinib

Location: Canada

NCT03929965
Anlotinib in Advanced Solid Tumors With FGFR Alteration

Cancer type: Unspecified Solid Tumor

Variant class: FGFR mutation

Other identifier: ASTFA

Population segments: Second line, Stage III, Stage IV, Third line

Phase: I

Therapy: anlotinib hydrochloride

Location: China

Disclaimer: The data presented here is from a curated knowledgebase of publicly available information, but may not be exhaustive. The data version is 2021.11(003).

FGFR3 G370C mutation (continued)

NCT04565275
A Multi-center Open-label, Phase I/II Clinical Trial to Evaluate the Safety, Tolerability, Pharmacokinetics, and Pharmacodynamics of ICP-192 in Patients With Advanced Solid Tumors and FGFR Gene Alterations.

Cancer type: Bladder Urothelial Carcinoma, Cholangiocarcinoma, Unspecified Solid Tumor

Variant class: FGFR aberration

Other identifier: ICP-CL-00303

Population segments: FGFR, Second line, Stage III, Stage IV

Phase: I/II

Therapy: ICP-192

Location: United States

US States: AZ, CO, FL, MN

Contact: Olivia Yang [609-524-0684; olivia.yang@INNOCAREPHARMA.COM]

No NCT ID - see other identifier(s)
Phase I/II Study of TAS-117 In Combination With TAS-120 In Patients With Advanced Solid Tumors

Cancer type: Unspecified Solid Tumor

Variant class: FGFR aberration

Other identifier: JapicCTI-194864

Population segments: FGFR, First line, Squamous Cell, Stage III, Stage IV

Phase: I/II

Therapies: TAS-117, futibatinib

Location: Japan

No NCT ID - see other identifier(s)
A Phase Ib Study to Assess the Safety, Tolerability, and Efficacy of TAS-120 (Futibatinib) in Combination with MK-3475 (Pembrolizumab) in Patients with Solid Tumors.

Cancer type: Unspecified Solid Tumor

Variant class: FGFR aberration

Other identifier: JapicCTI-195063

Population segments: FGFR, Line of therapy N/A, Stage III, Stage IV

Phase: I

Therapies: futibatinib, pembrolizumab

Location: Japan

NCT03390504
A Phase III Study of Erdafitinib Compared With Vinflunine or Docetaxel or Pembrolizumab in Subjects With Advanced Urothelial Cancer and Selected FGFR Gene Aberrations

Cancer type: Bladder Urothelial Carcinoma

Variant class: FGFR3 G370C mutation

Other identifiers: 2018-0027, 42756493BLC3001, 42756493-BLC3001, CR108401, CTR20182097, CTRI/2018/07/014718, EudraCT Number: 2017-002932-18, INT2, IRAS ID 238051, JapicCTI-183949, JNJ-42756493, NCI-2018-01443, NL64531.091.18, THOR

Population segments: FGFR, Second line, Stage III, Stage IV

Phase: III

Therapies: erdafitinib, pembrolizumab

Locations: Argentina, Australia, Austria, Belgium, Brazil, Canada, China, France, Germany, Greece, Hungary, Israel, Italy, Japan, Mexico, Netherlands, Poland, Portugal, Republic of Korea, Russian Federation, Spain, Taiwan, Turkey, Ukraine, United Kingdom, United States

US States: CA, DC, FL, IL, KY, MD, NC, NH, NV, NY, OH, TX, VA

Contact: Study Contact [844-434-4210; JNJ.CT@sylogent.com]

Disclaimer: The data presented here is from a curated knowledgebase of publicly available information, but may not be exhaustive. The data version is 2021.11(003).

FGFR3 G370C mutation (continued)

NCT04197986

Phase III, Multicenter, Double-Blind, Randomized, Placebo-Controlled Trial of Infigratinib for the Adjuvant Treatment of Subjects With Invasive Urothelial Carcinoma With Susceptible FGFR3 Genetic Alterations (PROOF 302).

Cancer type: Bladder Urothelial Carcinoma

Variant class: FGFR3 G370C mutation

Other identifiers: EudraCT Number: 2019-003248-63, NCI-2020-00610, PROOF 302, QBGJ398-302

Population segments: Adjuvant, FGFR, Stage II, Stage III

Exclusion criteria variant classes: FGFR3 A391E mutation (Bladder Small Cell Neuroendocrine Carcinoma), FGFR3 G370C mutation (Bladder Small Cell Neuroendocrine Carcinoma), FGFR3 K650E mutation (Bladder Small Cell Neuroendocrine Carcinoma), FGFR3 K650M mutation (Bladder Small Cell Neuroendocrine Carcinoma), FGFR3 K650Q mutation (Bladder Small Cell Neuroendocrine Carcinoma), FGFR3 K650T mutation (Bladder Small Cell Neuroendocrine Carcinoma), FGFR3 R248C mutation (Bladder Small Cell Neuroendocrine Carcinoma), FGFR3 S249C mutation (Bladder Small Cell Neuroendocrine Carcinoma), FGFR3 Y373C mutation (Bladder Small Cell Neuroendocrine Carcinoma), FGFR3 fusion (Bladder Small Cell Neuroendocrine Carcinoma)

Phase: III

Therapy: infigratinib

Locations: Belgium, Canada, France, Germany, Italy, Netherlands, Puerto Rico, Spain, United Kingdom, United States

US States: AL, AZ, CA, CO, DC, FL, GA, IL, IN, LA, MA, MD, MO, NC, NE, NJ, NY, OH, OK, SC, TN, TX, UT, VA, WA, WV

Contact: QED Therapeutics [877-280-5655; Proof302.ct@qedtx.com]

NCT02365597

A Phase II, Two-arm Multicenter, Open-Label Study to Determine the Efficacy and the Safety of Two Different Dose Regimens of a Pan-FGFR Tyrosine Kinase Inhibitor JNJ-42756493 in Subjects With Metastatic or Surgically Unresectable Urothelial Cancer With FGFR Genomic Alterations.

Cancer type: Bladder Urothelial Carcinoma

Variant class: FGFR3 G370C mutation

Other identifiers: 42756493BLC2001, BLC2001, CR105065, DRKS00009448, EudracT Number: 2014-002408-26, IRAS ID 177346, NCI-2015-00818, REec-2015-1451

Population segments: FGFR, First line, Fourth line or greater, Second line, Stage III, Stage IV, Third line

Phase: II

Therapy: erdafitinib

Locations: France, Spain, United States

US State: TX

NCT04492293

A Phase II, Multicenter, Single Arm, Open-lable Study of ICP-192 in Subjects with Surgically Unresectable or Metastatic Bladder Urothelial Cancer with FGFR Genetic Aberrations.

Cancer type: Bladder Urothelial Carcinoma

Variant class: FGFR3 G370C mutation

Other identifiers: CTR20200688, ICP-CL-00302

Population segments: FGFR, Second line, Stage III, Stage IV

Phase: II

Therapy: ICP-192

Location: China

Disclaimer: The data presented here is from a curated knowledgebase of publicly available information, but may not be exhaustive. The data version is 2021.11(003).

FGFR3 G370C mutation (continued)

NCT04936295
A Phase II Study of the Efficacy and Tolerability of Fulvestrant Plus Anlotinib in HR(+)/HER2(-) Metastatic Breast Cancer Patients With FGFR Mutation

Cancer type: Breast Cancer

Variant class: FGFR3 activating mutation

Other inclusion criteria: ERBB2 negative, Hormone receptor positive

Other identifier: SYSU005

Population segments: Estrogen receptor positive, FGFR, First line, HER2 negative, HER2 positive, Progesterone receptor positive, Stage IV

Phase: II

Therapies: hormone therapy, anlotinib hydrochloride

Location: China

NCT04096417
A Phase II, Multicenter, Single-Arm Study of Pemigatinib in Patients With Metastatic or Unresectable Colorectal Cancer Harboring FGFR Alterations

Cancer type: Colorectal Cancer

Variant class: FGFR3 activating mutation

Other identifiers: ACCRU-GI-1701, NCI-2019-05877, VICCGI2028

Population segments: FGFR, Second line, Stage III, Stage IV

Phase: II

Therapy: pemigatinib

Location: United States

US States: AZ, GA, MN, NC, TN

Contact: Multiple contacts: See www.clinicaltrials.gov for complete list of contacts.

NCT04045613
An Open-label Multi-cohort Phase Ib/II Study of Derazantinib and Atezolizumab in Patients With Urothelial Cancer Expressing Activating Molecular FGFR Aberrations.

Cancer type: Bladder Urothelial Carcinoma

Variant class: FGFR3 activating mutation

Other inclusion criteria: CD274 expression

Other identifiers: CSET 3012, DZB-CS-201, EudraCT Number: 2019-000359-15, FIDES-02, NCI-2019-08162, RECF4112, SNCTP000004480

Population segments: FGFR, Fourth line or greater, Second line, Stage IV, Third line

Phase: I/II

Therapies: derazantinib, atezolizumab

Locations: Australia, Austria, Belgium, Canada, Czech Republic, France, Germany, Hungary, Italy, Poland, Republic of Korea, Spain, Switzerland, United Kingdom, United States

US States: GA, IL, KS, KY, NY, SC, TX, VA, WA

Contact: Dr. Manuel HÃ¤ckl [manuel.haeckl@basilea.com]

Disclaimer: The data presented here is from a curated knowledgebase of publicly available information, but may not be exhaustive. The data version is 2021.11(003).

FGFR3 G370C mutation (continued)

NCT04053322
An International Multicenter Phase II Trial of Durvalumab (MEDI4736) Plus OLAparib Plus Fulvestrant in Metastatic or Locally Advanced ER-positive, HER2-negative Breast Cancer Patients Selected Using Criteria That Predict Sensitivity to Olaparib

Cancer type: Breast Cancer

Variant class: FGFR3 mutation

Other inclusion criteria: ERBB2 negative, ER positive

Other identifiers: CADUSEIME08, DOLAF, EudraCT Number: 2018-003832-57, UC-0140/1812

Population segments: BRCA, Estrogen receptor positive, FGFR, HER2 negative, Progesterone receptor positive, Second line, Stage III, Stage IV

Phase: II

Therapies: durvalumab, olaparib, hormone therapy

Location: France

NCT03827850
A Phase II Trial to Evaluate Efficacy and Safety of Erdafitinib in Patients With Advanced Squamous Non Small Cell Lung Carcinoma (sqNSCLC) Harboring Fibroblast Growth Factor Receptor (FGFR) Genetic Alterations After Relapse of Standard Therapy.

Cancer type: Non-Small Cell Lung Cancer

Variant class: FGFR activating mutation

Other identifiers: EudraCT Number: 2018-000399-13, FIND, FIND Trial, Uni-Koeln-3254

Population segments: FGFR, Second line, Squamous Cell, Stage III, Stage IV

Exclusion criteria variant classes: ALK aberration (Non-Small Cell Lung Cancer), BRAF aberration (Non-Small Cell Lung Cancer), EGFR aberration (Non-Small Cell Lung Cancer), NTRK aberration (Non-Small Cell Lung Cancer), ROS1 aberration (Non-Small Cell Lung Cancer)

Phase: II

Therapy: erdafitinib

Location: Germany

NCT04601857
A Phase II Study Evaluating Futibatinib (TAS 120) Plus Pembrolizumab in the Treatment of Advanced or Metastatic Urothelial Carcinoma

Cancer type: Bladder Urothelial Carcinoma

Variant class: FGFR3 mutation

Other identifiers: EudraCT Number: 2020-000945-15, TAS-120-203

Population segments: FGFR, First line, Stage III, Stage IV

Phase: II

Therapies: futibatinib, pembrolizumab

Location: United States

US State: MI

Contact: Dr. Maciej Gil [609-250-7336; clinicaltrialinfo@taihooncology.com]

Disclaimer: The data presented here is from a curated knowledgebase of publicly available information, but may not be exhaustive. The data version is 2021.11(003).

FGFR3 G370C mutation (continued)

NCT04294277
Open-label, Single-arm, Phase II Study, Evaluating Safety and Efficacy of INCB054828 (Pemigatinib) as Adjuvant Therapy for Molecularly-selected, High-risk Patients With Urothelial Carcinoma Who Have Received Radical Surgery

Cancer type: Bladder Urothelial Carcinoma

Variant class: FGFR3 mutation

Other identifiers: EAU RF 2018-02, EAURF2018-02, EudraCT Number: 2017-004426-15, EudraCT Number: 2019-001833-14, PEGASUS

Population segments: Adjuvant, FGFR, Stage III, Stage IV

Phase: II

Therapy: pemigatinib

Location: Italy

NCT04077255
Anti-EGFR Antibody (GC-1118) in Combination With Weekly Paclitaxel as a Second-line Therapy for Gastric and Gastroesophageal Junction Adenocarcinomas With Amplifications

Cancer type: Gastric Cancer, Gastroesophageal Junction Adenocarcinoma

Variant class: FGFR3 mutation

Other identifiers: 31973, KCT0004314, NCC2019-0196

Population segments: EGFR, FGFR, Second line, Stage IV

Phase: II

Therapies: rogaratinib, chemotherapy

Location: Republic of Korea

No NCT ID - see other identifier(s)
Phase I Clinical Study of BPI-17509 In Patients With Advanced Solid Tumors

Cancer type: Bladder Cancer, Solid Tumor

Variant class: FGFR3 mutation

Other identifiers: BTP-26811, CTR20192140

Population segments: Second line, Stage III, Stage IV

Phase: I

Therapy: BPI-17509

Location: China

NCT04172675
A Randomized Phase II Study of Erdafitinib Versus Investigator Choice of Intravesical Chemotherapy in Subjects Who Received Bacillus Calmette-Guerin (BCG) and Recurred With High Risk Non-Muscle-Invasive Bladder Cancer (NMIBC) and FGFR Mutations or Fusions

Cancer type: Bladder Urothelial Carcinoma

Variant class: FGFR mutation

Other identifiers: 42756493BLC2003, 42756493BLC2003, CR108699, CTR20201841, CTRI/2021/08/036009, EudraCT Number: 2019-002449-39, JapicCTI-205145, NCI-2020-00674, RG1121102

Population segments: FGFR, Maintenance/Consolidation, Second line, Stage 0

Phase: II

Therapy: erdafitinib

Locations: Argentina, Australia, Belgium, Brazil, China, Czech Republic, France, Germany, Italy, Japan, Poland, Republic of Korea, Spain, Taiwan, United Kingdom, United States

US States: AZ, CA, CO, FL, GA, IL, KS, NC, NY, OH, PA, TN, TX, WA, WI

Contact: Study Contact [844-434-4210; JNJ.CT@sylogent.com]

Disclaimer: The data presented here is from a curated knowledgebase of publicly available information, but may not be exhaustive. The data version is 2021.11(003).

FGFR3 G370C mutation (continued)

NCT02150967
A Phase II Multicenter, Single Arm Study of Oral BGJ398 in Adult Patients with Advanced or Metastatic Cholangiocarcinoma with FGFR2 Gene Fusions or Other FGFR Genetic Alterations who Failed or are Intolerant to Platinum-based Chemotherapy.

Cancer type: Cholangiocarcinoma

Variant class: FGFR activating aberration

Other identifiers: 14-183, 14-2200, 14-328, 15452, 2014-0372, 2014-081, CBGJ398X2204, EudraCT Number: 2013-005085-19, NCI-2014-01310, REec-2014-1022, Trial 3GB-14-1

Population segments: FGFR, Fourth line or greater, Second line, Stage III, Stage IV, Third line

Phase: II

Therapy: infigratinib

Locations: Belgium, Germany, Singapore, Spain, Thailand, United Kingdom, United States

US States: CA, MA, MI, NY, OH, TX

Contact: QED Therapeutics [877-280-5655; clinicaltrials@QEDTx.com]

NCT04595747
Phase II Study of Rogaratinib (BAY 1163877) in the Treatment of Patients With Sarcoma Harboring Alterations in Fibroblast Growth Factor Receptor (FGFR) 1-4 and SDH-Deficient Gastrointestinal Stromal Tumor (GIST)

Cancer type: Soft Tissue Sarcoma

Variant class: FGFR3 aberration

Other identifiers: 10411, NCI-2020-08011

Population segments: FGFR, Second line, Stage III, Stage IV

Phase: II

Therapy: rogaratinib

Location: United States

US States: CA, MA, MD, NY

Contact: Multiple contacts: See www.clinicaltrials.gov for complete list of contacts.

NCT04004975
Phase II Clinical Trials on Anlotinib for the Treatment of Recurrent Glioblastoma

Cancer type: Glioblastoma

Variant class: FGFR mutation

Other identifier: ShandongCHI006

Population segments: (N/A), FGFR, Second line

Phase: I/II

Therapy: anlotinib hydrochloride

Location: China

NCT04149691
A Phase I, Open-label, Multicentre, Dose Escalation Study to Assess Safety, Tolerability and Pharmacokinetics of Oral CPL304110, in Adult Subjects With Advanced Solid Malignancies

Cancer type: Bladder Cancer, Gastric Cancer, Non-Small Cell Lung Cancer

Variant class: FGFR3 aberration

Other identifier: 01FGFR2018

Population segments: FGFR, Line of therapy N/A, Squamous Cell, Stage III, Stage IV

Phase: I

Therapy: CPL-304-110

Location: Poland

Disclaimer: The data presented here is from a curated knowledgebase of publicly available information, but may not be exhaustive. The data version is 2021.11(003).

FGFR3 G370C mutation (continued)

NCT03473743
A Phase Ib-II Study to Evaluate Safety, Efficacy, Pharmacokinetics, and Pharmacodynamics of Various Regimens of Erdafitinib in Subjects With Metastatic or Locally Advanced Urothelial Cancer

Cancer type: Bladder Urothelial Carcinoma

Variant class: FGFR aberration

Other identifiers: 17165, 2018-0142, 42756493BLC2002, CR108445, EudraCT Number: 2017-001980-19, NCI-2018-00997, NORSE, USO 17165, USOR 17165

Population segments: FGFR, First line, Fourth line or greater, Second line, Stage IV, Third line

Phase: I/II

Therapies: erdafitinib, cetrelimab

Locations: Belarus, Belgium, Brazil, France, Italy, Poland, Republic of Korea, Russian Federation, Spain, Taiwan, Turkey, United Kingdom, United States

US States: CO, MD, NC, NY, PA, TX, VA

Contact: Study Contact [844-434-4210; JNJ.CT@sylogent.com]

NCT02393248
A Phase I/II, Open-Label, Dose-Escalation, Safety and Tolerability Study of INCB054828 in Subjects With Advanced Malignancies - (FIGHT-101)

Cancer type: Bladder Urothelial Carcinoma, Breast Cancer, Cholangiocarcinoma, Endometrial Carcinoma, Gastric Cancer, Non-Small Cell Lung Cancer, Solid Tumor

Variant class: FGFR aberration

Other identifiers: 00061567, 2014-1099, 201503012, EudraCT Number: 2016-002831-14, FIGHT-101, INCB 54828-101, INCB54828-101, MC# 15-03, NCI-2015-00611, OSU-15241

Population segments: FGFR, First line, Fourth line or greater, Second line, Squamous Cell, Stage III, Stage IV, Third line

Phase: I/II

Therapies: pemigatinib, pembrolizumab, trastuzumab, chemotherapy, retifanlimab

Location: United States

US States: FL, NJ, TX

Contact: Incyte Call Center [855-463-3463]

NCT04572295
An Open-label Phase Ib Study of E7090 Monotherapy and in Combination With Other Anticancer Agents in Subjects With ER+, HER2- Recurrent/Metastatic Breast Cancer

Cancer type: Breast Cancer

Variant class: FGFR aberration

Other inclusion criteria: ERBB2 negative, ER positive

Other identifiers: E7090-J081-102, JapicCTI-205429

Population segments: Estrogen receptor positive, HER2 negative, Second line, Stage IV

Phase: I

Therapies: E-7090, hormone therapy

Location: Japan

Disclaimer: The data presented here is from a curated knowledgebase of publicly available information, but may not be exhaustive. The data version is 2021.11(003).

FGFR3 G370C mutation (continued)

NCT04504331
A Phase IB Study of Infigratinib in Combination With Tamoxifen in Hormone Receptor Positive, HER2 Negative, FGFR Altered Advanced Breast Cancer

Cancer type: Breast Cancer

Variant class: FGFR aberration

Other inclusion criteria: ERBB2 negative, Hormone receptor positive

Other identifiers: BRS0113, K08CA252457

Population segments: Estrogen receptor positive, HER2 negative, Progesterone receptor positive, Second line, Stage III, Stage IV, Triple receptor negative

Phase: I

Therapies: infigratinib, hormone therapy

Location: United States

US State: CA

Contact: Allison K. Zhang [650-736-5790; axkong@stanford.edu]

FGFR3 R248C mutation

NCT05019794
A Phase IIa of Infigratinib in Subjects With Locally Advanced or Metastatic Gastric Cancer or Gastroesophageal Junction Adenocarcinoma With FGFR2 Amplification or Other Advanced Solid Tumors With Other FGFR Alterations

Cancer type: Gastric Cancer, Gastroesophageal Junction Adenocarcinoma, Unspecified Solid Tumor

Variant class: FGFR3 activating mutation

Other identifiers: CTR20210275, LB1001-201

Population segments: FGFR, Fourth line or greater, Second line, Stage III, Stage IV, Third line

Phase: II

Therapy: infigratinib

Location: China

NCT03155620
NCI-COG Pediatric MATCH (Molecular Analysis for Therapy Choice) Screening Protocol

Cancer type: Unspecified Solid Tumor

Variant class: FGFR3 mutation

Other identifiers: 17-729, 2017-0625, 20170841, APEC1621SC, COGAPEC1621, COGAPEC1621SC, N 55017 SC, NCI-2017-01251, NCI-COG Pediatric MATCH, Pediatric MATCH

Population segments: ALK, Aggressive, BRAF, FGFR, HRAS, Indolent, KRAS, NRAS, Pediatric or Adolescent, Second line, Stage III, Stage IV

Phase: II

Therapy: erdafitinib

Locations: Puerto Rico, United States

US States: AK, AL, AR, AZ, CA, CO, CT, DC, DE, FL, GA, HI, IA, ID, IL, IN, KY, LA, MA, MD, ME, MI, MN, MO, MS, NC, NE, NH, NJ, NV, NY, OH, OK, OR, PA, RI, SC, SD, TN, TX, UT, VA, VT, WA, WI

Contact: Multiple contacts: See www.clinicaltrials.gov for complete list of contacts.

Disclaimer: The data presented here is from a curated knowledgebase of publicly available information, but may not be exhaustive. The data version is 2021.11(003).

FGFR3 R248C mutation (continued)

NCT03210714

NCI-COG Pediatric MATCH (Molecular Analysis for Therapy Choice) - Phase II Subprotocol of JNJ-42756493 (Erdafitinib) in Patients With Tumors Harboring FGFR 1/2/3/4 Alterations

Cancer type: Unspecified Solid Tumor

Variant class: FGFR3 mutation

Other identifiers: APEC1621B, NCI-2017-01159, NCI-COG Pediatric MATCH, Pediatric MATCH

Population segments: Aggressive, FGFR, Indolent, Pediatric or Adolescent, Second line, Stage III, Stage IV

Phase: II

Therapy: erdafitinib

Locations: Puerto Rico, United States

US States: AK, AL, AR, AZ, CA, CO, CT, DC, DE, FL, GA, IA, ID, IL, IN, KY, LA, MD, ME, MI, MN, MO, MS, NC, ND, NE, NJ, NY, OH, OK, OR, PA, SC, SD, TN, TX, UT, VA, VT, WA, WI

Contact: Multiple contacts: See www.clinicaltrials.gov for complete list of contacts.

No NCT ID - see other identifier(s)

A Multicenter Phase II Basket-type Clinical Trial to Evaluate Efficacy and Safety of TAS-120 in Patients with Advanced Solid Malignancies with FGFR Alterations in Circulating Tumor DNA

Cancer type: Unspecified Solid Tumor

Variant class: FGFR3 mutation

Other identifiers: EPOC 1805, JapicCTI-194624, JapicCTI-194796, TiFFANY

Population segments: Second line, Stage III, Stage IV

Phase: II

Therapy: futibatinib

Location: Japan

NCT04233567

A Phase II Study of Oral Infigratinib in Adult Patients With Advanced or Metastatic Solid Tumors With FGFR1-3 Gene Fusions or Other FGFR Genetic Alterations

Cancer type: Unspecified Solid Tumor

Variant class: FGFR activating mutation

Other identifiers: NCI-2019-06869, OSU-19041

Population segments: Second line, Stage III, Stage IV

Phase: II

Therapy: infigratinib

Location: United States

US State: OH

Contact: The Ohio State University Comprehensive Cancer Center [800-293-5066; OSUCCCClinicaltrials@osumc.edu]

Disclaimer: The data presented here is from a curated knowledgebase of publicly available information, but may not be exhaustive. The data version is 2021.11(003).

FGFR3 R248C mutation (continued)

NCT02693535
Targeted Agent and Profiling Utilization Registry (TAPUR) Study.

Cancer type: Unspecified Solid Tumor

Variant class: FGFR3 mutation

Other identifiers: 20170529, NCI-2017-00510, Pro00014171, TAPUR

Population segments: Aggressive, BRAF, Diffuse large B-cell lymphoma (DLBCL), Extranodal marginal zone B-cell lymphoma (MALT), FGFR, First line, Follicular lymphoma (FL), Fourth line or greater, Indolent, Lymphoblastic lymphoma (LBL), Mantle cell lymphoma (MCL), Other subtype, Second line, Small lymphocytic lymphoma (SLL), Stage III, Stage IV, Third line, Waldenstrom's macroglobulinemia (WM)

Phase: II

Therapy: sunitinib

Location: United States

US States: AL, AZ, CA, FL, GA, HI, IL, IN, ME, MI, NC, ND, NE, NH, OH, OR, PA, SD, TX, UT, VA, WA

Contact: Pam Mangat [pam.mangat@asco.org]

NCT04591431
The Rome Trial From Histology to Target: the Road to Personalize Target Therapy and Immunotherapy

Cancer type: Biliary Tract Carcinoma, Breast Cancer, Colorectal Cancer, Esophageal Cancer, Gastric Cancer, Gastroesophageal Junction Adenocarcinoma, Hepatocellular Carcinoma, Liver Cancer, Non-Small Cell Lung Cancer, Pancreatic Cancer, Unspecified Solid Tumor

Variant class: FGFR3 aberration

Other identifiers: EudraCT Number: 2018-002190-21, MAR-BAS-18-005, ROME

Population segments: Second line, Stage IV

Phase: II

Therapy: pemigatinib

Location: Italy

NCT02691793
Study to Evaluate the Safety and Efficacy of Sunitinib, in Subject With Refractory Solid Tumors

Cancer type: Unspecified Solid Tumor

Variant class: FGFR mutation

Other identifier: 2016-02-098

Population segments: (N/A), FGFR, Second line

Phase: II

Therapy: sunitinib

Location: Republic of Korea

Disclaimer: The data presented here is from a curated knowledgebase of publicly available information, but may not be exhaustive. The data version is 2021.11(003).

FGFR3 R248C mutation (continued)

NCT03297606
Canadian Profiling and Targeted Agent Utilization Trial (CAPTUR): A Phase II Basket Trial

Cancer type: Unspecified Solid Tumor

Variant class: FGFR3 aberration

Other identifiers: CA209-9DL, CAPTUR, ESR-17-12831, ML39800, PM1, WI233446

Population segments: Aggressive, Diffuse large B-cell lymphoma (DLBCL), Extranodal marginal zone B-cell lymphoma (MALT), First line, Follicular lymphoma (FL), Indolent, Lymphoblastic lymphoma (LBL), Mantle cell lymphoma (MCL), Other subtype, Second line, Stage III, Stage IV, Waldenstrom's macroglobulinemia (WM)

Phase: II

Therapy: sunitinib

Location: Canada

NCT03929965
Anlotinib in Advanced Solid Tumors With FGFR Alteration

Cancer type: Unspecified Solid Tumor

Variant class: FGFR mutation

Other identifier: ASTFA

Population segments: Second line, Stage III, Stage IV, Third line

Phase: I

Therapy: anlotinib hydrochloride

Location: China

NCT04565275
A Multi-center Open-label, Phase I/II Clinical Trial to Evaluate the Safety, Tolerability, Pharmacokinetics, and Pharmacodynamics of ICP-192 in Patients With Advanced Solid Tumors and FGFR Gene Alterations.

Cancer type: Bladder Urothelial Carcinoma, Cholangiocarcinoma, Unspecified Solid Tumor

Variant class: FGFR aberration

Other identifier: ICP-CL-00303

Population segments: FGFR, Second line, Stage III, Stage IV

Phase: I/II

Therapy: ICP-192

Location: United States

US States: AZ, CO, FL, MN

Contact: Olivia Yang [609-524-0684; olivia.yang@INNOCAREPHARMA.COM]

No NCT ID - see other identifier(s)
Phase I/II Study of TAS-117 In Combination With TAS-120 In Patients With Advanced Solid Tumors

Cancer type: Unspecified Solid Tumor

Variant class: FGFR aberration

Other identifier: JapicCTI-194864

Population segments: FGFR, First line, Squamous Cell, Stage III, Stage IV

Phase: I/II

Therapies: TAS-117, futibatinib

Location: Japan

FGFR3 R248C mutation (continued)

No NCT ID - see other identifier(s)
A Phase Ib Study to Assess the Safety, Tolerability, and Efficacy of TAS-120 (Futibatinib) in Combination with MK-3475 (Pembrolizumab) in Patients with Solid Tumors.

Cancer type: Unspecified Solid Tumor

Variant class: FGFR aberration

Other identifier: JapicCTI-195063

Population segments: FGFR, Line of therapy N/A, Stage III, Stage IV

Phase: I

Therapies: futibatinib, pembrolizumab

Location: Japan

NCT03390504

A Phase III Study of Erdafitinib Compared With Vinflunine or Docetaxel or Pembrolizumab in Subjects With Advanced Urothelial Cancer and Selected FGFR Gene Aberrations

Cancer type: Bladder Urothelial Carcinoma

Variant class: FGFR3 R248C mutation

Other identifiers: 2018-0027, 42756493BLC3001, 42756493-BLC3001, CR108401, CTR20182097, CTRI/2018/07/014718, EudraCT Number: 2017-002932-18, INT2, IRAS ID 238051, JapicCTI-183949, JNJ-42756493, NCI-2018-01443, NL64531.091.18, THOR

Population segments: FGFR, Second line, Stage III, Stage IV

Phase: III

Therapies: erdafitinib, pembrolizumab

Locations: Argentina, Australia, Austria, Belgium, Brazil, Canada, China, France, Germany, Greece, Hungary, Israel, Italy, Japan, Mexico, Netherlands, Poland, Portugal, Republic of Korea, Russian Federation, Spain, Taiwan, Turkey, Ukraine, United Kingdom, United States

US States: CA, DC, FL, IL, KY, MD, NC, NH, NV, NY, OH, TX, VA

Contact: Study Contact [844-434-4210; JNJ.CT@sylogent.com]

Disclaimer: The data presented here is from a curated knowledgebase of publicly available information, but may not be exhaustive. The data version is 2021.11(003).

FGFR3 R248C mutation (continued)

NCT04197986

Phase III, Multicenter, Double-Blind, Randomized, Placebo-Controlled Trial of Infigratinib for the Adjuvant Treatment of Subjects With Invasive Urothelial Carcinoma With Susceptible FGFR3 Genetic Alterations (PROOF 302).

Cancer type: Bladder Urothelial Carcinoma

Variant class: FGFR3 R248C mutation

Other identifiers: EudraCT Number: 2019-003248-63, NCI-2020-00610, PROOF 302, QBGJ398-302

Population segments: Adjuvant, FGFR, Stage II, Stage III

Exclusion criteria variant classes: FGFR3 A391E mutation (Bladder Small Cell Neuroendocrine Carcinoma), FGFR3 G370C mutation (Bladder Small Cell Neuroendocrine Carcinoma), FGFR3 K650E mutation (Bladder Small Cell Neuroendocrine Carcinoma), FGFR3 K650M mutation (Bladder Small Cell Neuroendocrine Carcinoma), FGFR3 K650Q mutation (Bladder Small Cell Neuroendocrine Carcinoma), FGFR3 K650T mutation (Bladder Small Cell Neuroendocrine Carcinoma), FGFR3 R248C mutation (Bladder Small Cell Neuroendocrine Carcinoma), FGFR3 S249C mutation (Bladder Small Cell Neuroendocrine Carcinoma), FGFR3 Y373C mutation (Bladder Small Cell Neuroendocrine Carcinoma), FGFR3 fusion (Bladder Small Cell Neuroendocrine Carcinoma)

Phase: III

Therapy: infigratinib

Locations: Belgium, Canada, France, Germany, Italy, Netherlands, Puerto Rico, Spain, United Kingdom, United States

US States: AL, AZ, CA, CO, DC, FL, GA, IL, IN, LA, MA, MD, MO, NC, NE, NJ, NY, OH, OK, SC, TN, TX, UT, VA, WA, WV

Contact: QED Therapeutics [877-280-5655; Proof302.ct@qedtx.com]

NCT02365597

A Phase II, Two-arm Multicenter, Open-Label Study to Determine the Efficacy and the Safety of Two Different Dose Regimens of a Pan-FGFR Tyrosine Kinase Inhibitor JNJ-42756493 in Subjects With Metastatic or Surgically Unresectable Urothelial Cancer With FGFR Genomic Alterations.

Cancer type: Bladder Urothelial Carcinoma

Variant class: FGFR3 R248C mutation

Other identifiers: 42756493BLC2001, BLC2001, CR105065, DRKS00009448, EudracT Number: 2014-002408-26, IRAS ID 177346, NCI-2015-00818, REec-2015-1451

Population segments: FGFR, First line, Fourth line or greater, Second line, Stage III, Stage IV, Third line

Phase: II

Therapy: erdafitinib

Locations: France, Spain, United States

US State: TX

NCT04492293

A Phase II, Multicenter, Single Arm, Open-lable Study of ICP-192 in Subjects with Surgically Unresectable or Metastatic Bladder Urothelial Cancer with FGFR Genetic Aberrations.

Cancer type: Bladder Urothelial Carcinoma

Variant class: FGFR3 R248C mutation

Other identifiers: CTR20200688, ICP-CL-00302

Population segments: FGFR, Second line, Stage III, Stage IV

Phase: II

Therapy: ICP-192

Location: China

Disclaimer: The data presented here is from a curated knowledgebase of publicly available information, but may not be exhaustive. The data version is 2021.11(003).

FGFR3 R248C mutation (continued)

NCT04936295

A Phase II Study of the Efficacy and Tolerability of Fulvestrant Plus Anlotinib in HR(+)/HER2(-) Metastatic Breast Cancer Patients With FGFR Mutation

Cancer type: Breast Cancer

Variant class: FGFR3 activating mutation

Other inclusion criteria: ERBB2 negative, Hormone receptor positive

Other identifier: SYSU005

Population segments: Estrogen receptor positive, FGFR, First line, HER2 negative, HER2 positive, Progesterone receptor positive, Stage IV

Phase: II

Therapies: hormone therapy, anlotinib hydrochloride

Location: China

NCT04096417

A Phase II, Multicenter, Single-Arm Study of Pemigatinib in Patients With Metastatic or Unresectable Colorectal Cancer Harboring FGFR Alterations

Cancer type: Colorectal Cancer

Variant class: FGFR3 activating mutation

Other identifiers: ACCRU-GI-1701, NCI-2019-05877, VICCGI2028

Population segments: FGFR, Second line, Stage III, Stage IV

Phase: II

Therapy: pemigatinib

Location: United States

US States: AZ, GA, MN, NC, TN

Contact: Multiple contacts: See www.clinicaltrials.gov for complete list of contacts.

NCT04045613

An Open-label Multi-cohort Phase Ib/II Study of Derazantinib and Atezolizumab in Patients With Urothelial Cancer Expressing Activating Molecular FGFR Aberrations.

Cancer type: Bladder Urothelial Carcinoma

Variant class: FGFR3 activating mutation

Other inclusion criteria: CD274 expression

Other identifiers: CSET 3012, DZB-CS-201, EudraCT Number: 2019-000359-15, FIDES-02, NCI-2019-08162, RECF4112, SNCTP000004480

Population segments: FGFR, Fourth line or greater, Second line, Stage IV, Third line

Phase: I/II

Therapies: derazantinib, atezolizumab

Locations: Australia, Austria, Belgium, Canada, Czech Republic, France, Germany, Hungary, Italy, Poland, Republic of Korea, Spain, Switzerland, United Kingdom, United States

US States: GA, IL, KS, KY, NY, SC, TX, VA, WA

Contact: Dr. Manuel HÃ¤ckl [manuel.haeckl@basilea.com]

Disclaimer: The data presented here is from a curated knowledgebase of publicly available information, but may not be exhaustive. The data version is 2021.11(003).

FGFR3 R248C mutation (continued)

NCT04053322
An International Multicenter Phase II Trial of Durvalumab (MEDI4736) Plus OLAparib Plus Fulvestrant in Metastatic or Locally Advanced ER-positive, HER2-negative Breast Cancer Patients Selected Using Criteria That Predict Sensitivity to Olaparib

Cancer type: Breast Cancer

Variant class: FGFR3 mutation

Other inclusion criteria: ERBB2 negative, ER positive

Other identifiers: CADUSEIME08, DOLAF, EudraCT Number: 2018-003832-57, UC-0140/1812

Population segments: BRCA, Estrogen receptor positive, FGFR, HER2 negative, Progesterone receptor positive, Second line, Stage III, Stage IV

Phase: II

Therapies: durvalumab, olaparib, hormone therapy

Location: France

NCT03827850
A Phase II Trial to Evaluate Efficacy and Safety of Erdafitinib in Patients With Advanced Squamous Non Small Cell Lung Carcinoma (sqNSCLC) Harboring Fibroblast Growth Factor Receptor (FGFR) Genetic Alterations After Relapse of Standard Therapy.

Cancer type: Non-Small Cell Lung Cancer

Variant class: FGFR activating mutation

Other identifiers: EudraCT Number: 2018-000399-13, FIND, FIND Trial, Uni-Koeln-3254

Population segments: FGFR, Second line, Squamous Cell, Stage III, Stage IV

Exclusion criteria variant classes: ALK aberration (Non-Small Cell Lung Cancer), BRAF aberration (Non-Small Cell Lung Cancer), EGFR aberration (Non-Small Cell Lung Cancer), NTRK aberration (Non-Small Cell Lung Cancer), ROS1 aberration (Non-Small Cell Lung Cancer)

Phase: II

Therapy: erdafitinib

Location: Germany

NCT04601857
A Phase II Study Evaluating Futibatinib (TAS 120) Plus Pembrolizumab in the Treatment of Advanced or Metastatic Urothelial Carcinoma

Cancer type: Bladder Urothelial Carcinoma

Variant class: FGFR3 mutation

Other identifiers: EudraCT Number: 2020-000945-15, TAS-120-203

Population segments: FGFR, First line, Stage III, Stage IV

Phase: II

Therapies: futibatinib, pembrolizumab

Location: United States

US State: MI

Contact: Dr. Maciej Gil [609-250-7336; clinicaltrialinfo@taihooncology.com]

FGFR3 R248C mutation (continued)

NCT04294277
Open-label, Single-arm, Phase II Study, Evaluating Safety and Efficacy of INCB054828 (Pemigatinib) as Adjuvant Therapy for Molecularly-selected, High-risk Patients With Urothelial Carcinoma Who Have Received Radical Surgery

Cancer type: Bladder Urothelial Carcinoma

Variant class: FGFR3 mutation

Other identifiers: EAU RF 2018-02, EAURF2018-02, EudraCT Number: 2017-004426-15, EudraCT Number: 2019-001833-14, PEGASUS

Population segments: Adjuvant, FGFR, Stage III, Stage IV

Phase: II

Therapy: pemigatinib

Location: Italy

NCT04077255
Anti-EGFR Antibody (GC-1118) in Combination With Weekly Paclitaxel as a Second-line Therapy for Gastric and Gastroesophageal Junction Adenocarcinomas With Amplifications

Cancer type: Gastric Cancer, Gastroesophageal Junction Adenocarcinoma

Variant class: FGFR3 mutation

Other identifiers: 31973, KCT0004314, NCC2019-0196

Population segments: EGFR, FGFR, Second line, Stage IV

Phase: II

Therapies: rogaratinib, chemotherapy

Location: Republic of Korea

No NCT ID - see other identifier(s)
Phase I Clinical Study of BPI-17509 In Patients With Advanced Solid Tumors

Cancer type: Bladder Cancer, Solid Tumor

Variant class: FGFR3 mutation

Other identifiers: BTP-26811, CTR20192140

Population segments: Second line, Stage III, Stage IV

Phase: I

Therapy: BPI-17509

Location: China

NCT04172675
A Randomized Phase II Study of Erdafitinib Versus Investigator Choice of Intravesical Chemotherapy in Subjects Who Received Bacillus Calmette-Guerin (BCG) and Recurred With High Risk Non-Muscle-Invasive Bladder Cancer (NMIBC) and FGFR Mutations or Fusions

Cancer type: Bladder Urothelial Carcinoma

Variant class: FGFR mutation

Other identifiers: 42756493BLC2003, 42756493BLC2003, CR108699, CTR20201841, CTRI/2021/08/036009, EudraCT Number: 2019-002449-39, JapicCTI-205145, NCI-2020-00674, RG1121102

Population segments: FGFR, Maintenance/Consolidation, Second line, Stage 0

Phase: II

Therapy: erdafitinib

Locations: Argentina, Australia, Belgium, Brazil, China, Czech Republic, France, Germany, Italy, Japan, Poland, Republic of Korea, Spain, Taiwan, United Kingdom, United States

US States: AZ, CA, CO, FL, GA, IL, KS, NC, NY, OH, PA, TN, TX, WA, WI

Contact: Study Contact [844-434-4210; JNJ.CT@sylogent.com]

Disclaimer: The data presented here is from a curated knowledgebase of publicly available information, but may not be exhaustive. The data version is 2021.11(003).

FGFR3 R248C mutation (continued)

NCT02150967
A Phase II Multicenter, Single Arm Study of Oral BGJ398 in Adult Patients with Advanced or Metastatic Cholangiocarcinoma with FGFR2 Gene Fusions or Other FGFR Genetic Alterations who Failed or are Intolerant to Platinum-based Chemotherapy.

Cancer type: Cholangiocarcinoma

Variant class: FGFR activating aberration

Other identifiers: 14-183, 14-2200, 14-328, 15452, 2014-0372, 2014-081, CBGJ398X2204, EudraCT Number: 2013-005085-19, NCI-2014-01310, REec-2014-1022, Trial 3GB-14-1

Population segments: FGFR, Fourth line or greater, Second line, Stage III, Stage IV, Third line

Phase: II

Therapy: infigratinib

Locations: Belgium, Germany, Singapore, Spain, Thailand, United Kingdom, United States

US States: CA, MA, MI, NY, OH, TX

Contact: QED Therapeutics [877-280-5655; clinicaltrials@QEDTx.com]

NCT04595747
Phase II Study of Rogaratinib (BAY 1163877) in the Treatment of Patients With Sarcoma Harboring Alterations in Fibroblast Growth Factor Receptor (FGFR) 1-4 and SDH-Deficient Gastrointestinal Stromal Tumor (GIST)

Cancer type: Soft Tissue Sarcoma

Variant class: FGFR3 aberration

Other identifiers: 10411, NCI-2020-08011

Population segments: FGFR, Second line, Stage III, Stage IV

Phase: II

Therapy: rogaratinib

Location: United States

US States: CA, MA, MD, NY

Contact: Multiple contacts: See www.clinicaltrials.gov for complete list of contacts.

NCT04004975
Phase II Clinical Trials on Anlotinib for the Treatment of Recurrent Glioblastoma

Cancer type: Glioblastoma

Variant class: FGFR mutation

Other identifier: ShandongCHI006

Population segments: (N/A), FGFR, Second line

Phase: I/II

Therapy: anlotinib hydrochloride

Location: China

NCT04149691
A Phase I, Open-label, Multicentre, Dose Escalation Study to Assess Safety, Tolerability and Pharmacokinetics of Oral CPL304110, in Adult Subjects With Advanced Solid Malignancies

Cancer type: Bladder Cancer, Gastric Cancer, Non-Small Cell Lung Cancer

Variant class: FGFR3 aberration

Other identifier: 01FGFR2018

Population segments: FGFR, Line of therapy N/A, Squamous Cell, Stage III, Stage IV

Phase: I

Therapy: CPL-304-110

Location: Poland

FGFR3 R248C mutation (continued)

NCT03473743
A Phase Ib-II Study to Evaluate Safety, Efficacy, Pharmacokinetics, and Pharmacodynamics of Various Regimens of Erdafitinib in Subjects With Metastatic or Locally Advanced Urothelial Cancer

Cancer type: Bladder Urothelial Carcinoma

Variant class: FGFR aberration

Other identifiers: 17165, 2018-0142, 42756493BLC2002, CR108445, EudraCT Number: 2017-001980-19, NCI-2018-00997, NORSE, USO 17165, USOR 17165

Population segments: FGFR, First line, Fourth line or greater, Second line, Stage IV, Third line

Phase: I/II

Therapies: erdafitinib, cetrelimab

Locations: Belarus, Belgium, Brazil, France, Italy, Poland, Republic of Korea, Russian Federation, Spain, Taiwan, Turkey, United Kingdom, United States

US States: CO, MD, NC, NY, PA, TX, VA

Contact: Study Contact [844-434-4210; JNJ.CT@sylogent.com]

NCT02393248
A Phase I/II, Open-Label, Dose-Escalation, Safety and Tolerability Study of INCB054828 in Subjects With Advanced Malignancies - (FIGHT-101)

Cancer type: Bladder Urothelial Carcinoma, Breast Cancer, Cholangiocarcinoma, Endometrial Carcinoma, Gastric Cancer, Non-Small Cell Lung Cancer, Solid Tumor

Variant class: FGFR aberration

Other identifiers: 00061567, 2014-1099, 201503012, EudraCT Number: 2016-002831-14, FIGHT-101, INCB 54828-101, INCB54828-101, MC# 15-03, NCI-2015-00611, OSU-15241

Population segments: FGFR, First line, Fourth line or greater, Second line, Squamous Cell, Stage III, Stage IV, Third line

Phase: I/II

Therapies: pemigatinib, pembrolizumab, trastuzumab, chemotherapy, retifanlimab

Location: United States

US States: FL, NJ, TX

Contact: Incyte Call Center [855-463-3463]

NCT04572295
An Open-label Phase Ib Study of E7090 Monotherapy and in Combination With Other Anticancer Agents in Subjects With ER+, HER2- Recurrent/Metastatic Breast Cancer

Cancer type: Breast Cancer

Variant class: FGFR aberration

Other inclusion criteria: ERBB2 negative, ER positive

Other identifiers: E7090-J081-102, JapicCTI-205429

Population segments: Estrogen receptor positive, HER2 negative, Second line, Stage IV

Phase: I

Therapies: E-7090, hormone therapy

Location: Japan

Disclaimer: The data presented here is from a curated knowledgebase of publicly available information, but may not be exhaustive. The data version is 2021.11(003).

FGFR3 R248C mutation (continued)

NCT04504331
A Phase IB Study of Infigratinib in Combination With Tamoxifen in Hormone Receptor Positive, HER2 Negative, FGFR Altered Advanced Breast Cancer

Cancer type: Breast Cancer

Variant class: FGFR aberration

Other inclusion criteria: ERBB2 negative, Hormone receptor positive

Other identifiers: BRS0113, K08CA252457

Population segments: Estrogen receptor positive, HER2 negative, Progesterone receptor positive, Second line, Stage III, Stage IV, Triple receptor negative

Phase: I

Therapies: infigratinib, hormone therapy

Location: United States

US State: CA

Contact: Allison K. Zhang [650-736-5790; axkong@stanford.edu]

FGFR3 S249C mutation

NCT05019794
A Phase IIa of Infigratinib in Subjects With Locally Advanced or Metastatic Gastric Cancer or Gastroesophageal Junction Adenocarcinoma With FGFR2 Amplification or Other Advanced Solid Tumors With Other FGFR Alterations

Cancer type: Gastric Cancer, Gastroesophageal Junction Adenocarcinoma, Unspecified Solid Tumor

Variant class: FGFR3 activating mutation

Other identifiers: CTR20210275, LB1001-201

Population segments: FGFR, Fourth line or greater, Second line, Stage III, Stage IV, Third line

Phase: II

Therapy: infigratinib

Location: China

NCT03155620
NCI-COG Pediatric MATCH (Molecular Analysis for Therapy Choice) Screening Protocol

Cancer type: Unspecified Solid Tumor

Variant class: FGFR3 mutation

Other identifiers: 17-729, 2017-0625, 20170841, APEC1621SC, COGAPEC1621, COGAPEC1621SC, N 55017 SC, NCI-2017-01251, NCI-COG Pediatric MATCH, Pediatric MATCH

Population segments: ALK, Aggressive, BRAF, FGFR, HRAS, Indolent, KRAS, NRAS, Pediatric or Adolescent, Second line, Stage III, Stage IV

Phase: II

Therapy: erdafitinib

Locations: Puerto Rico, United States

US States: AK, AL, AR, AZ, CA, CO, CT, DC, DE, FL, GA, HI, IA, ID, IL, IN, KY, LA, MA, MD, ME, MI, MN, MO, MS, NC, NE, NH, NJ, NV, NY, OH, OK, OR, PA, RI, SC, SD, TN, TX, UT, VA, VT, WA, WI

Contact: Multiple contacts: See www.clinicaltrials.gov for complete list of contacts.

Disclaimer: The data presented here is from a curated knowledgebase of publicly available information, but may not be exhaustive. The data version is 2021.11(003).

FGFR3 S249C mutation (continued)

NCT03210714
NCI-COG Pediatric MATCH (Molecular Analysis for Therapy Choice) - Phase II Subprotocol of JNJ-42756493 (Erdafitinib) in Patients With Tumors Harboring FGFR 1/2/3/4 Alterations

Cancer type: Unspecified Solid Tumor

Variant class: FGFR3 mutation

Other identifiers: APEC1621B, NCI-2017-01159, NCI-COG Pediatric MATCH, Pediatric MATCH

Population segments: Aggressive, FGFR, Indolent, Pediatric or Adolescent, Second line, Stage III, Stage IV

Phase: II

Therapy: erdafitinib

Locations: Puerto Rico, United States

US States: AK, AL, AR, AZ, CA, CO, CT, DC, DE, FL, GA, IA, ID, IL, IN, KY, LA, MD, ME, MI, MN, MO, MS, NC, ND, NE, NJ, NY, OH, OK, OR, PA, SC, SD, TN, TX, UT, VA, VT, WA, WI

Contact: Multiple contacts: See www.clinicaltrials.gov for complete list of contacts.

No NCT ID - see other identifier(s)
A Multicenter Phase II Basket-type Clinical Trial to Evaluate Efficacy and Safety of TAS-120 in Patients with Advanced Solid Malignancies with FGFR Alterations in Circulating Tumor DNA

Cancer type: Unspecified Solid Tumor

Variant class: FGFR3 mutation

Other identifiers: EPOC 1805, JapicCTI-194624, JapicCTI-194796, TiFFANY

Population segments: Second line, Stage III, Stage IV

Phase: II

Therapy: futibatinib

Location: Japan

NCT04233567
A Phase II Study of Oral Infigratinib in Adult Patients With Advanced or Metastatic Solid Tumors With FGFR1-3 Gene Fusions or Other FGFR Genetic Alterations

Cancer type: Unspecified Solid Tumor

Variant class: FGFR activating mutation

Other identifiers: NCI-2019-06869, OSU-19041

Population segments: Second line, Stage III, Stage IV

Phase: II

Therapy: infigratinib

Location: United States

US State: OH

Contact: The Ohio State University Comprehensive Cancer Center [800-293-5066; OSUCCCClinicaltrials@osumc.edu]

Disclaimer: The data presented here is from a curated knowledgebase of publicly available information, but may not be exhaustive. The data version is 2021.11(003).

FGFR3 S249C mutation (continued)

NCT02693535
Targeted Agent and Profiling Utilization Registry (TAPUR) Study.

Cancer type: Unspecified Solid Tumor

Variant class: FGFR3 mutation

Other identifiers: 20170529, NCI-2017-00510, Pro00014171, TAPUR

Population segments: Aggressive, BRAF, Diffuse large B-cell lymphoma (DLBCL), Extranodal marginal zone B-cell lymphoma (MALT), FGFR, First line, Follicular lymphoma (FL), Fourth line or greater, Indolent, Lymphoblastic lymphoma (LBL), Mantle cell lymphoma (MCL), Other subtype, Second line, Small lymphocytic lymphoma (SLL), Stage III, Stage IV, Third line, Waldenstrom's macroglobulinemia (WM)

Phase: II

Therapy: sunitinib

Location: United States

US States: AL, AZ, CA, FL, GA, HI, IL, IN, ME, MI, NC, ND, NE, NH, OH, OR, PA, SD, TX, UT, VA, WA

Contact: Pam Mangat [pam.mangat@asco.org]

NCT04591431
The Rome Trial From Histology to Target: the Road to Personalize Target Therapy and Immunotherapy

Cancer type: Biliary Tract Carcinoma, Breast Cancer, Colorectal Cancer, Esophageal Cancer, Gastric Cancer, Gastroesophageal Junction Adenocarcinoma, Hepatocellular Carcinoma, Liver Cancer, Non-Small Cell Lung Cancer, Pancreatic Cancer, Unspecified Solid Tumor

Variant class: FGFR3 aberration

Other identifiers: EudraCT Number: 2018-002190-21, MAR-BAS-18-005, ROME

Population segments: Second line, Stage IV

Phase: II

Therapy: pemigatinib

Location: Italy

NCT02691793
Study to Evaluate the Safety and Efficacy of Sunitinib, in Subject With Refractory Solid Tumors

Cancer type: Unspecified Solid Tumor

Variant class: FGFR mutation

Other identifier: 2016-02-098

Population segments: (N/A), FGFR, Second line

Phase: II

Therapy: sunitinib

Location: Republic of Korea

FGFR3 S249C mutation (continued)

NCT03297606
Canadian Profiling and Targeted Agent Utilization Trial (CAPTUR): A Phase II Basket Trial

Cancer type: Unspecified Solid Tumor

Variant class: FGFR3 aberration

Other identifiers: CA209-9DL, CAPTUR, ESR-17-12831, ML39800, PM1, WI233446

Population segments: Aggressive, Diffuse large B-cell lymphoma (DLBCL), Extranodal marginal zone B-cell lymphoma (MALT), First line, Follicular lymphoma (FL), Indolent, Lymphoblastic lymphoma (LBL), Mantle cell lymphoma (MCL), Other subtype, Second line, Stage III, Stage IV, Waldenstrom's macroglobulinemia (WM)

Phase: II

Therapy: sunitinib

Location: Canada

NCT03929965
Anlotinib in Advanced Solid Tumors With FGFR Alteration

Cancer type: Unspecified Solid Tumor

Variant class: FGFR mutation

Other identifier: ASTFA

Population segments: Second line, Stage III, Stage IV, Third line

Phase: I

Therapy: anlotinib hydrochloride

Location: China

NCT04565275
A Multi-center Open-label, Phase I/II Clinical Trial to Evaluate the Safety, Tolerability, Pharmacokinetics, and Pharmacodynamics of ICP-192 in Patients With Advanced Solid Tumors and FGFR Gene Alterations.

Cancer type: Bladder Urothelial Carcinoma, Cholangiocarcinoma, Unspecified Solid Tumor

Variant class: FGFR aberration

Other identifier: ICP-CL-00303

Population segments: FGFR, Second line, Stage III, Stage IV

Phase: I/II

Therapy: ICP-192

Location: United States

US States: AZ, CO, FL, MN

Contact: Olivia Yang [609-524-0684; olivia.yang@INNOCAREPHARMA.COM]

No NCT ID - see other identifier(s)
Phase I/II Study of TAS-117 In Combination With TAS-120 In Patients With Advanced Solid Tumors

Cancer type: Unspecified Solid Tumor

Variant class: FGFR aberration

Other identifier: JapicCTI-194864

Population segments: FGFR, First line, Squamous Cell, Stage III, Stage IV

Phase: I/II

Therapies: TAS-117, futibatinib

Location: Japan

Disclaimer: The data presented here is from a curated knowledgebase of publicly available information, but may not be exhaustive. The data version is 2021.11(003).

FGFR3 S249C mutation (continued)

No NCT ID - see other identifier(s)
A Phase Ib Study to Assess the Safety, Tolerability, and Efficacy of TAS-120 (Futibatinib) in Combination with MK-3475 (Pembrolizumab) in Patients with Solid Tumors.

Cancer type: Unspecified Solid Tumor

Variant class: FGFR aberration

Other identifier: JapicCTI-195063

Population segments: FGFR, Line of therapy N/A, Stage III, Stage IV

Phase: I

Therapies: futibatinib, pembrolizumab

Location: Japan

NCT03390504
A Phase III Study of Erdafitinib Compared With Vinflunine or Docetaxel or Pembrolizumab in Subjects With Advanced Urothelial Cancer and Selected FGFR Gene Aberrations

Cancer type: Bladder Urothelial Carcinoma

Variant class: FGFR3 S249C mutation

Other identifiers: 2018-0027, 42756493BLC3001, 42756493-BLC3001, CR108401, CTR20182097, CTRI/2018/07/014718, EudraCT Number: 2017-002932-18, INT2, IRAS ID 238051, JapicCTI-183949, JNJ-42756493, NCI-2018-01443, NL64531.091.18, THOR

Population segments: FGFR, Second line, Stage III, Stage IV

Phase: III

Therapies: erdafitinib, pembrolizumab

Locations: Argentina, Australia, Austria, Belgium, Brazil, Canada, China, France, Germany, Greece, Hungary, Israel, Italy, Japan, Mexico, Netherlands, Poland, Portugal, Republic of Korea, Russian Federation, Spain, Taiwan, Turkey, Ukraine, United Kingdom, United States

US States: CA, DC, FL, IL, KY, MD, NC, NH, NV, NY, OH, TX, VA

Contact: Study Contact [844-434-4210; JNJ.CT@sylogent.com]

FGFR3 S249C mutation (continued)

NCT04197986

Phase III, Multicenter, Double-Blind, Randomized, Placebo-Controlled Trial of Infigratinib for the Adjuvant Treatment of Subjects With Invasive Urothelial Carcinoma With Susceptible FGFR3 Genetic Alterations (PROOF 302).

Cancer type: Bladder Urothelial Carcinoma

Variant class: FGFR3 S249C mutation

Other identifiers: EudraCT Number: 2019-003248-63, NCI-2020-00610, PROOF 302, QBGJ398-302

Population segments: Adjuvant, FGFR, Stage II, Stage III

Exclusion criteria variant classes: FGFR3 A391E mutation (Bladder Small Cell Neuroendocrine Carcinoma), FGFR3 G370C mutation (Bladder Small Cell Neuroendocrine Carcinoma), FGFR3 K650E mutation (Bladder Small Cell Neuroendocrine Carcinoma), FGFR3 K650M mutation (Bladder Small Cell Neuroendocrine Carcinoma), FGFR3 K650Q mutation (Bladder Small Cell Neuroendocrine Carcinoma), FGFR3 K650T mutation (Bladder Small Cell Neuroendocrine Carcinoma), FGFR3 R248C mutation (Bladder Small Cell Neuroendocrine Carcinoma), FGFR3 S249C mutation (Bladder Small Cell Neuroendocrine Carcinoma), FGFR3 Y373C mutation (Bladder Small Cell Neuroendocrine Carcinoma), FGFR3 fusion (Bladder Small Cell Neuroendocrine Carcinoma)

Phase: III

Therapy: infigratinib

Locations: Belgium, Canada, France, Germany, Italy, Netherlands, Puerto Rico, Spain, United Kingdom, United States

US States: AL, AZ, CA, CO, DC, FL, GA, IL, IN, LA, MA, MD, MO, NC, NE, NJ, NY, OH, OK, SC, TN, TX, UT, VA, WA, WV

Contact: QED Therapeutics [877-280-5655; Proof302.ct@qedtx.com]

NCT02365597

A Phase II, Two-arm Multicenter, Open-Label Study to Determine the Efficacy and the Safety of Two Different Dose Regimens of a Pan-FGFR Tyrosine Kinase Inhibitor JNJ-42756493 in Subjects With Metastatic or Surgically Unresectable Urothelial Cancer With FGFR Genomic Alterations.

Cancer type: Bladder Urothelial Carcinoma

Variant class: FGFR3 S249C mutation

Other identifiers: 42756493BLC2001, BLC2001, CR105065, DRKS00009448, EudracT Number: 2014-002408-26, IRAS ID 177346, NCI-2015-00818, REec-2015-1451

Population segments: FGFR, First line, Fourth line or greater, Second line, Stage III, Stage IV, Third line

Phase: II

Therapy: erdafitinib

Locations: France, Spain, United States

US State: TX

NCT04492293

A Phase II, Multicenter, Single Arm, Open-lable Study of ICP-192 in Subjects with Surgically Unresectable or Metastatic Bladder Urothelial Cancer with FGFR Genetic Aberrations.

Cancer type: Bladder Urothelial Carcinoma

Variant class: FGFR3 S249C mutation

Other identifiers: CTR20200688, ICP-CL-00302

Population segments: FGFR, Second line, Stage III, Stage IV

Phase: II

Therapy: ICP-192

Location: China

FGFR3 S249C mutation (continued)

NCT04936295
A Phase II Study of the Efficacy and Tolerability of Fulvestrant Plus Anlotinib in HR(+)/HER2(-) Metastatic Breast Cancer Patients With FGFR Mutation

Cancer type: Breast Cancer

Variant class: FGFR3 activating mutation

Other inclusion criteria: ERBB2 negative, Hormone receptor positive

Other identifier: SYSU005

Population segments: Estrogen receptor positive, FGFR, First line, HER2 negative, HER2 positive, Progesterone receptor positive, Stage IV

Phase: II

Therapies: hormone therapy, anlotinib hydrochloride

Location: China

NCT04096417
A Phase II, Multicenter, Single-Arm Study of Pemigatinib in Patients With Metastatic or Unresectable Colorectal Cancer Harboring FGFR Alterations

Cancer type: Colorectal Cancer

Variant class: FGFR3 activating mutation

Other identifiers: ACCRU-GI-1701, NCI-2019-05877, VICCGI2028

Population segments: FGFR, Second line, Stage III, Stage IV

Phase: II

Therapy: pemigatinib

Location: United States

US States: AZ, GA, MN, NC, TN

Contact: Multiple contacts: See www.clinicaltrials.gov for complete list of contacts.

NCT04045613
An Open-label Multi-cohort Phase Ib/II Study of Derazantinib and Atezolizumab in Patients With Urothelial Cancer Expressing Activating Molecular FGFR Aberrations.

Cancer type: Bladder Urothelial Carcinoma

Variant class: FGFR3 activating mutation

Other inclusion criteria: CD274 expression

Other identifiers: CSET 3012, DZB-CS-201, EudraCT Number: 2019-000359-15, FIDES-02, NCI-2019-08162, RECF4112, SNCTP000004480

Population segments: FGFR, Fourth line or greater, Second line, Stage IV, Third line

Phase: I/II

Therapies: derazantinib, atezolizumab

Locations: Australia, Austria, Belgium, Canada, Czech Republic, France, Germany, Hungary, Italy, Poland, Republic of Korea, Spain, Switzerland, United Kingdom, United States

US States: GA, IL, KS, KY, NY, SC, TX, VA, WA

Contact: Dr. Manuel HÃ¤ckl [manuel.haeckl@basilea.com]

Disclaimer: The data presented here is from a curated knowledgebase of publicly available information, but may not be exhaustive. The data version is 2021.11(003).

FGFR3 S249C mutation (continued)

NCT04053322

An International Multicenter Phase II Trial of Durvalumab (MEDI4736) Plus OLAparib Plus Fulvestrant in Metastatic or Locally Advanced ER-positive, HER2-negative Breast Cancer Patients Selected Using Criteria That Predict Sensitivity to Olaparib

Cancer type: Breast Cancer

Variant class: FGFR3 mutation

Other inclusion criteria: ERBB2 negative, ER positive

Other identifiers: CADUSEIME08, DOLAF, EudraCT Number: 2018-003832-57, UC-0140/1812

Population segments: BRCA, Estrogen receptor positive, FGFR, HER2 negative, Progesterone receptor positive, Second line, Stage III, Stage IV

Phase: II

Therapies: durvalumab, olaparib, hormone therapy

Location: France

NCT03827850

A Phase II Trial to Evaluate Efficacy and Safety of Erdafitinib in Patients With Advanced Squamous Non Small Cell Lung Carcinoma (sqNSCLC) Harboring Fibroblast Growth Factor Receptor (FGFR) Genetic Alterations After Relapse of Standard Therapy.

Cancer type: Non-Small Cell Lung Cancer

Variant class: FGFR activating mutation

Other identifiers: EudraCT Number: 2018-000399-13, FIND, FIND Trial, Uni-Koeln-3254

Population segments: FGFR, Second line, Squamous Cell, Stage III, Stage IV

Exclusion criteria variant classes: ALK aberration (Non-Small Cell Lung Cancer), BRAF aberration (Non-Small Cell Lung Cancer), EGFR aberration (Non-Small Cell Lung Cancer), NTRK aberration (Non-Small Cell Lung Cancer), ROS1 aberration (Non-Small Cell Lung Cancer)

Phase: II

Therapy: erdafitinib

Location: Germany

NCT04601857

A Phase II Study Evaluating Futibatinib (TAS 120) Plus Pembrolizumab in the Treatment of Advanced or Metastatic Urothelial Carcinoma

Cancer type: Bladder Urothelial Carcinoma

Variant class: FGFR3 mutation

Other identifiers: EudraCT Number: 2020-000945-15, TAS-120-203

Population segments: FGFR, First line, Stage III, Stage IV

Phase: II

Therapies: futibatinib, pembrolizumab

Location: United States

US State: MI

Contact: Dr. Maciej Gil [609-250-7336; clinicaltrialinfo@taihooncology.com]

Disclaimer: The data presented here is from a curated knowledgebase of publicly available information, but may not be exhaustive. The data version is 2021.11(003).

FGFR3 S249C mutation (continued)

NCT04294277
Open-label, Single-arm, Phase II Study, Evaluating Safety and Efficacy of INCB054828 (Pemigatinib) as Adjuvant Therapy for Molecularly-selected, High-risk Patients With Urothelial Carcinoma Who Have Received Radical Surgery

Cancer type: Bladder Urothelial Carcinoma

Variant class: FGFR3 mutation

Other identifiers: EAU RF 2018-02, EAURF2018-02, EudraCT Number: 2017-004426-15, EudraCT Number: 2019-001833-14, PEGASUS

Population segments: Adjuvant, FGFR, Stage III, Stage IV

Phase: II

Therapy: pemigatinib

Location: Italy

NCT04077255
Anti-EGFR Antibody (GC-1118) in Combination With Weekly Paclitaxel as a Second-line Therapy for Gastric and Gastroesophageal Junction Adenocarcinomas With Amplifications

Cancer type: Gastric Cancer, Gastroesophageal Junction Adenocarcinoma

Variant class: FGFR3 mutation

Other identifiers: 31973, KCT0004314, NCC2019-0196

Population segments: EGFR, FGFR, Second line, Stage IV

Phase: II

Therapies: rogaratinib, chemotherapy

Location: Republic of Korea

No NCT ID - see other identifier(s)
Phase I Clinical Study of BPI-17509 In Patients With Advanced Solid Tumors

Cancer type: Bladder Cancer, Solid Tumor

Variant class: FGFR3 mutation

Other identifiers: BTP-26811, CTR20192140

Population segments: Second line, Stage III, Stage IV

Phase: I

Therapy: BPI-17509

Location: China

NCT04172675
A Randomized Phase II Study of Erdafitinib Versus Investigator Choice of Intravesical Chemotherapy in Subjects Who Received Bacillus Calmette-Guerin (BCG) and Recurred With High Risk Non-Muscle-Invasive Bladder Cancer (NMIBC) and FGFR Mutations or Fusions

Cancer type: Bladder Urothelial Carcinoma

Variant class: FGFR mutation

Other identifiers: 42756493BLC2003, 42756493BLC2003, CR108699, CTR20201841, CTRI/2021/08/036009, EudraCT Number: 2019-002449-39, JapicCTI-205145, NCI-2020-00674, RG1121102

Population segments: FGFR, Maintenance/Consolidation, Second line, Stage 0

Phase: II

Therapy: erdafitinib

Locations: Argentina, Australia, Belgium, Brazil, China, Czech Republic, France, Germany, Italy, Japan, Poland, Republic of Korea, Spain, Taiwan, United Kingdom, United States

US States: AZ, CA, CO, FL, GA, IL, KS, NC, NY, OH, PA, TN, TX, WA, WI

Contact: Study Contact [844-434-4210; JNJ.CT@sylogent.com]

Disclaimer: The data presented here is from a curated knowledgebase of publicly available information, but may not be exhaustive. The data version is 2021.11(003).

FGFR3 S249C mutation (continued)

NCT02150967
A Phase II Multicenter, Single Arm Study of Oral BGJ398 in Adult Patients with Advanced or Metastatic Cholangiocarcinoma with FGFR2 Gene Fusions or Other FGFR Genetic Alterations who Failed or are Intolerant to Platinum-based Chemotherapy.

Cancer type: Cholangiocarcinoma

Variant class: FGFR activating aberration

Other identifiers: 14-183, 14-2200, 14-328, 15452, 2014-0372, 2014-081, CBGJ398X2204, EudraCT Number: 2013-005085-19, NCI-2014-01310, REec-2014-1022, Trial 3GB-14-1

Population segments: FGFR, Fourth line or greater, Second line, Stage III, Stage IV, Third line

Phase: II

Therapy: infigratinib

Locations: Belgium, Germany, Singapore, Spain, Thailand, United Kingdom, United States

US States: CA, MA, MI, NY, OH, TX

Contact: QED Therapeutics [877-280-5655; clinicaltrials@QEDTx.com]

NCT04595747
Phase II Study of Rogaratinib (BAY 1163877) in the Treatment of Patients With Sarcoma Harboring Alterations in Fibroblast Growth Factor Receptor (FGFR) 1-4 and SDH-Deficient Gastrointestinal Stromal Tumor (GIST)

Cancer type: Soft Tissue Sarcoma

Variant class: FGFR3 aberration

Other identifiers: 10411, NCI-2020-08011

Population segments: FGFR, Second line, Stage III, Stage IV

Phase: II

Therapy: rogaratinib

Location: United States

US States: CA, MA, MD, NY

Contact: Multiple contacts: See www.clinicaltrials.gov for complete list of contacts.

NCT04004975
Phase II Clinical Trials on Anlotinib for the Treatment of Recurrent Glioblastoma

Cancer type: Glioblastoma

Variant class: FGFR mutation

Other identifier: ShandongCHI006

Population segments: (N/A), FGFR, Second line

Phase: I/II

Therapy: anlotinib hydrochloride

Location: China

NCT04149691
A Phase I, Open-label, Multicentre, Dose Escalation Study to Assess Safety, Tolerability and Pharmacokinetics of Oral CPL304110, in Adult Subjects With Advanced Solid Malignancies

Cancer type: Bladder Cancer, Gastric Cancer, Non-Small Cell Lung Cancer

Variant class: FGFR3 aberration

Other identifier: 01FGFR2018

Population segments: FGFR, Line of therapy N/A, Squamous Cell, Stage III, Stage IV

Phase: I

Therapy: CPL-304-110

Location: Poland

FGFR3 S249C mutation (continued)

NCT03473743

A Phase Ib-II Study to Evaluate Safety, Efficacy, Pharmacokinetics, and Pharmacodynamics of Various Regimens of Erdafitinib in Subjects With Metastatic or Locally Advanced Urothelial Cancer

Cancer type: Bladder Urothelial Carcinoma

Variant class: FGFR aberration

Other identifiers: 17165, 2018-0142, 42756493BLC2002, CR108445, EudraCT Number: 2017-001980-19, NCI-2018-00997, NORSE, USO 17165, USOR 17165

Population segments: FGFR, First line, Fourth line or greater, Second line, Stage IV, Third line

Phase: I/II

Therapies: erdafitinib, cetrelimab

Locations: Belarus, Belgium, Brazil, France, Italy, Poland, Republic of Korea, Russian Federation, Spain, Taiwan, Turkey, United Kingdom, United States

US States: CO, MD, NC, NY, PA, TX, VA

Contact: Study Contact [844-434-4210; JNJ.CT@sylogent.com]

NCT02393248

A Phase I/II, Open-Label, Dose-Escalation, Safety and Tolerability Study of INCB054828 in Subjects With Advanced Malignancies - (FIGHT-101)

Cancer type: Bladder Urothelial Carcinoma, Breast Cancer, Cholangiocarcinoma, Endometrial Carcinoma, Gastric Cancer, Non-Small Cell Lung Cancer, Solid Tumor

Variant class: FGFR aberration

Other identifiers: 00061567, 2014-1099, 201503012, EudraCT Number: 2016-002831-14, FIGHT-101, INCB 54828-101, INCB54828-101, MC# 15-03, NCI-2015-00611, OSU-15241

Population segments: FGFR, First line, Fourth line or greater, Second line, Squamous Cell, Stage III, Stage IV, Third line

Phase: I/II

Therapies: pemigatinib, pembrolizumab, trastuzumab, chemotherapy, retifanlimab

Location: United States

US States: FL, NJ, TX

Contact: Incyte Call Center [855-463-3463]

NCT04572295

An Open-label Phase Ib Study of E7090 Monotherapy and in Combination With Other Anticancer Agents in Subjects With ER+, HER2- Recurrent/Metastatic Breast Cancer

Cancer type: Breast Cancer

Variant class: FGFR aberration

Other inclusion criteria: ERBB2 negative, ER positive

Other identifiers: E7090-J081-102, JapicCTI-205429

Population segments: Estrogen receptor positive, HER2 negative, Second line, Stage IV

Phase: I

Therapies: E-7090, hormone therapy

Location: Japan

FGFR3 S249C mutation (continued)

NCT04504331
A Phase IB Study of Infigratinib in Combination With Tamoxifen in Hormone Receptor Positive, HER2 Negative, FGFR Altered Advanced Breast Cancer

Cancer type: Breast Cancer

Variant class: FGFR aberration

Other inclusion criteria: ERBB2 negative, Hormone receptor positive

Other identifiers: BRS0113, K08CA252457

Population segments: Estrogen receptor positive, HER2 negative, Progesterone receptor positive, Second line, Stage III, Stage IV, Triple receptor negative

Phase: I

Therapies: infigratinib, hormone therapy

Location: United States

US State: CA

Contact: Allison K. Zhang [650-736-5790; axkong@stanford.edu]

FGFR3-TACC3 fusion

No NCT ID - see other identifier(s)
A Multicenter Phase II Basket-type Clinical Trial to Evaluate Efficacy and Safety of TAS-120 in Patients with Advanced Solid Malignancies with FGFR Alterations in Circulating Tumor DNA

Cancer type: Unspecified Solid Tumor

Variant class: FGFR3 fusion

Other identifiers: EPOC 1805, JapicCTI-194624, JapicCTI-194796, TiFFANY

Population segments: Second line, Stage III, Stage IV

Phase: II

Therapy: futibatinib

Location: Japan

NCT04189445
A Phase II Study of Futibatinib in Patients With Specific FGFR Aberrations

Cancer type: Unspecified Solid Tumor

Variant class: FGFR3 fusion

Other identifiers: EudraCT Number: 2019-004084-49, JapicCTI-205312, NCI-2020-03407, NL73545.056.20, TAS-120-202, UW20046

Population segments: (N/A), Aggressive, Classical, FGFR, Indolent, Nodular lymphocyte-predominant, Second line, Stage III, Stage IV, Third line

Exclusion criteria variant classes: FGFR1 fusion (Central Nervous System Neoplasm, Intrahepatic Cholangiocarcinoma), FGFR2 fusion (Central Nervous System Neoplasm, Intrahepatic Cholangiocarcinoma), FGFR3 fusion (Central Nervous System Neoplasm, Intrahepatic Cholangiocarcinoma), FGFR4 fusion (Central Nervous System Neoplasm, Intrahepatic Cholangiocarcinoma)

Phase: II

Therapy: futibatinib

Locations: Japan, Republic of Korea, United Kingdom, United States

US States: AZ, CA, TX, WI

Contact: Dr. Osamu Takahashi [609-250-7336; clinicaltrialinfo@taihooncology.com]

Disclaimer: The data presented here is from a curated knowledgebase of publicly available information, but may not be exhaustive. The data version is 2021.11(003).

FGFR3-TACC3 fusion (continued)

NCT04233567
A Phase II Study of Oral Infigratinib in Adult Patients With Advanced or Metastatic Solid Tumors With FGFR1-3 Gene Fusions or Other FGFR Genetic Alterations

Cancer type: Unspecified Solid Tumor

Variant class: FGFR3 fusion

Other identifiers: NCI-2019-06869, OSU-19041

Population segments: Second line, Stage III, Stage IV

Phase: II

Therapy: infigratinib

Location: United States

US State: OH

Contact: The Ohio State University Comprehensive Cancer Center [800-293-5066; OSUCCCClinicaltrials@osumc.edu]

NCT05019794
A Phase IIa of Infigratinib in Subjects With Locally Advanced or Metastatic Gastric Cancer or Gastroesophageal Junction Adenocarcinoma With FGFR2 Amplification or Other Advanced Solid Tumors With Other FGFR Alterations

Cancer type: Gastric Cancer, Gastroesophageal Junction Adenocarcinoma, Unspecified Solid Tumor

Variant class: FGFR3 fusion

Other identifiers: CTR20210275, LB1001-201

Population segments: FGFR, Fourth line or greater, Second line, Stage III, Stage IV, Third line

Phase: II

Therapy: infigratinib

Location: China

NCT03929965
Anlotinib in Advanced Solid Tumors With FGFR Alteration

Cancer type: Unspecified Solid Tumor

Variant class: FGFR fusion

Other identifier: ASTFA

Population segments: Second line, Stage III, Stage IV, Third line

Phase: I

Therapy: anlotinib hydrochloride

Location: China

NCT04591431
The Rome Trial From Histology to Target: the Road to Personalize Target Therapy and Immunotherapy

Cancer type: Biliary Tract Carcinoma, Breast Cancer, Colorectal Cancer, Esophageal Cancer, Gastric Cancer, Gastroesophageal Junction Adenocarcinoma, Hepatocellular Carcinoma, Liver Cancer, Non-Small Cell Lung Cancer, Pancreatic Cancer, Unspecified Solid Tumor

Variant class: FGFR3 aberration

Other identifiers: EudraCT Number: 2018-002190-21, MAR-BAS-18-005, ROME

Population segments: Second line, Stage IV

Phase: II

Therapy: pemigatinib

Location: Italy

Disclaimer: The data presented here is from a curated knowledgebase of publicly available information, but may not be exhaustive. The data version is 2021.11(003).

FGFR3-TACC3 fusion (continued)

NCT03297606
Canadian Profiling and Targeted Agent Utilization Trial (CAPTUR): A Phase II Basket Trial

Cancer type: Unspecified Solid Tumor

Variant class: FGFR3 aberration

Other identifiers: CA209-9DL, CAPTUR, ESR-17-12831, ML39800, PM1, WI233446

Population segments: Aggressive, Diffuse large B-cell lymphoma (DLBCL), Extranodal marginal zone B-cell lymphoma (MALT), First line, Follicular lymphoma (FL), Indolent, Lymphoblastic lymphoma (LBL), Mantle cell lymphoma (MCL), Other subtype, Second line, Stage III, Stage IV, Waldenstrom's macroglobulinemia (WM)

Phase: II

Therapy: sunitinib

Location: Canada

NCT04565275
A Multi-center Open-label, Phase I/II Clinical Trial to Evaluate the Safety, Tolerability, Pharmacokinetics, and Pharmacodynamics of ICP-192 in Patients With Advanced Solid Tumors and FGFR Gene Alterations.

Cancer type: Bladder Urothelial Carcinoma, Cholangiocarcinoma, Unspecified Solid Tumor

Variant class: FGFR aberration

Other identifier: ICP-CL-00303

Population segments: FGFR, Second line, Stage III, Stage IV

Phase: I/II

Therapy: ICP-192

Location: United States

US States: AZ, CO, FL, MN

Contact: Olivia Yang [609-524-0684; olivia.yang@INNOCAREPHARMA.COM]

No NCT ID - see other identifier(s)
Phase I/II Study of TAS-117 In Combination With TAS-120 In Patients With Advanced Solid Tumors

Cancer type: Unspecified Solid Tumor

Variant class: FGFR aberration

Other identifier: JapicCTI-194864

Population segments: FGFR, First line, Squamous Cell, Stage III, Stage IV

Phase: I/II

Therapies: TAS-117, futibatinib

Location: Japan

No NCT ID - see other identifier(s)
A Phase Ib Study to Assess the Safety, Tolerability, and Efficacy of TAS-120 (Futibatinib) in Combination with MK-3475 (Pembrolizumab) in Patients with Solid Tumors.

Cancer type: Unspecified Solid Tumor

Variant class: FGFR aberration

Other identifier: JapicCTI-195063

Population segments: FGFR, Line of therapy N/A, Stage III, Stage IV

Phase: I

Therapies: futibatinib, pembrolizumab

Location: Japan

Disclaimer: The data presented here is from a curated knowledgebase of publicly available information, but may not be exhaustive. The data version is 2021.11(003).

FGFR3-TACC3 fusion (continued)

NCT03390504

A Phase III Study of Erdafitinib Compared With Vinflunine or Docetaxel or Pembrolizumab in Subjects With Advanced Urothelial Cancer and Selected FGFR Gene Aberrations

Cancer type: Bladder Urothelial Carcinoma

Variant class: FGFR3-TACC3 fusion

Other identifiers: 2018-0027, 42756493BLC3001, 42756493-BLC3001, CR108401, CTR20182097, CTRI/2018/07/014718, EudraCT Number: 2017-002932-18, INT2, IRAS ID 238051, JapicCTI-183949, JNJ-42756493, NCI-2018-01443, NL64531.091.18, THOR

Population segments: FGFR, Second line, Stage III, Stage IV

Phase: III

Therapies: erdafitinib, pembrolizumab

Locations: Argentina, Australia, Austria, Belgium, Brazil, Canada, China, France, Germany, Greece, Hungary, Israel, Italy, Japan, Mexico, Netherlands, Poland, Portugal, Republic of Korea, Russian Federation, Spain, Taiwan, Turkey, Ukraine, United Kingdom, United States

US States: CA, DC, FL, IL, KY, MD, NC, NH, NV, NY, OH, TX, VA

Contact: Study Contact [844-434-4210; JNJ.CT@sylogent.com]

NCT02365597

A Phase II, Two-arm Multicenter, Open-Label Study to Determine the Efficacy and the Safety of Two Different Dose Regimens of a Pan-FGFR Tyrosine Kinase Inhibitor JNJ-42756493 in Subjects With Metastatic or Surgically Unresectable Urothelial Cancer With FGFR Genomic Alterations.

Cancer type: Bladder Urothelial Carcinoma

Variant class: FGFR3-TACC3 fusion

Other identifiers: 42756493BLC2001, BLC2001, CR105065, DRKS00009448, EudracT Number: 2014-002408-26, IRAS ID 177346, NCI-2015-00818, REec-2015-1451

Population segments: FGFR, First line, Fourth line or greater, Second line, Stage III, Stage IV, Third line

Phase: II

Therapy: erdafitinib

Locations: France, Spain, United States

US State: TX

NCT04492293

A Phase II, Multicenter, Single Arm, Open-lable Study of ICP-192 in Subjects with Surgically Unresectable or Metastatic Bladder Urothelial Cancer with FGFR Genetic Aberrations.

Cancer type: Bladder Urothelial Carcinoma

Variant class: FGFR3-TACC3 fusion

Other identifiers: CTR20200688, ICP-CL-00302

Population segments: FGFR, Second line, Stage III, Stage IV

Phase: II

Therapy: ICP-192

Location: China

Disclaimer: The data presented here is from a curated knowledgebase of publicly available information, but may not be exhaustive. The data version is 2021.11(003).

FGFR3-TACC3 fusion (continued)

NCT04424966
A Phase 0 Study of Infigratinib in Recurrent High-Grade Glioma Participants Scheduled for Resection to Evaluate Central Nervous System (CNS) Penetration With PK Triggered Expansion Cohort

Cancer type: Anaplastic Astrocytoma, Anaplastic Oligoastrocytoma, Anaplastic Oligodendroglioma, Anaplastic Pleomorphic Xanthoastrocytoma, Diffuse Midline Glioma, Glioblastoma

Variant class: FGFR3-TACC3 fusion

Other identifiers: 2020-08, 20-500-092-34-38

Population segments: (N/A), Neoadjuvant

Phase: I

Therapy: infigratinib

Location: United States

US State: AZ

Contact: Phase 0 Navigator [602-406-8605; research@ivybraintumorcenter.org]

NCT04197986
Phase III, Multicenter, Double-Blind, Randomized, Placebo-Controlled Trial of Infigratinib for the Adjuvant Treatment of Subjects With Invasive Urothelial Carcinoma With Susceptible FGFR3 Genetic Alterations (PROOF 302).

Cancer type: Bladder Urothelial Carcinoma

Variant class: FGFR3 fusion

Other identifiers: EudraCT Number: 2019-003248-63, NCI-2020-00610, PROOF 302, QBGJ398-302

Population segments: Adjuvant, FGFR, Stage II, Stage III

Exclusion criteria variant classes: FGFR3 A391E mutation (Bladder Small Cell Neuroendocrine Carcinoma), FGFR3 G370C mutation (Bladder Small Cell Neuroendocrine Carcinoma), FGFR3 K650E mutation (Bladder Small Cell Neuroendocrine Carcinoma), FGFR3 K650M mutation (Bladder Small Cell Neuroendocrine Carcinoma), FGFR3 K650Q mutation (Bladder Small Cell Neuroendocrine Carcinoma), FGFR3 K650T mutation (Bladder Small Cell Neuroendocrine Carcinoma), FGFR3 R248C mutation (Bladder Small Cell Neuroendocrine Carcinoma), FGFR3 S249C mutation (Bladder Small Cell Neuroendocrine Carcinoma), FGFR3 Y373C mutation (Bladder Small Cell Neuroendocrine Carcinoma), FGFR3 fusion (Bladder Small Cell Neuroendocrine Carcinoma)

Phase: III

Therapy: infigratinib

Locations: Belgium, Canada, France, Germany, Italy, Netherlands, Puerto Rico, Spain, United Kingdom, United States

US States: AL, AZ, CA, CO, DC, FL, GA, IL, IN, LA, MA, MD, MO, NC, NE, NJ, NY, OH, OK, SC, TN, TX, UT, VA, WA, WV

Contact: QED Therapeutics [877-280-5655; Proof302.ct@qedtx.com]

Disclaimer: The data presented here is from a curated knowledgebase of publicly available information, but may not be exhaustive. The data version is 2021.11(003).

FGFR3-TACC3 fusion (continued)

NCT04601857
A Phase II Study Evaluating Futibatinib (TAS 120) Plus Pembrolizumab in the Treatment of Advanced or Metastatic Urothelial Carcinoma

Cancer type: Bladder Urothelial Carcinoma

Variant class: FGFR3 fusion

Other identifiers: EudraCT Number: 2020-000945-15, TAS-120-203

Population segments: FGFR, First line, Stage III, Stage IV

Phase: II

Therapies: futibatinib, pembrolizumab

Location: United States

US State: MI

Contact: Dr. Maciej Gil [609-250-7336; clinicaltrialinfo@taihooncology.com]

NCT04936295
A Phase II Study of the Efficacy and Tolerability of Fulvestrant Plus Anlotinib in HR(+)/HER2(-) Metastatic Breast Cancer Patients With FGFR Mutation

Cancer type: Breast Cancer

Variant class: FGFR3 fusion

Other inclusion criteria: ERBB2 negative, Hormone receptor positive

Other identifier: SYSU005

Population segments: Estrogen receptor positive, FGFR, First line, HER2 negative, HER2 positive, Progesterone receptor positive, Stage IV

Phase: II

Therapies: hormone therapy, anlotinib hydrochloride

Location: China

NCT04096417
A Phase II, Multicenter, Single-Arm Study of Pemigatinib in Patients With Metastatic or Unresectable Colorectal Cancer Harboring FGFR Alterations

Cancer type: Colorectal Cancer

Variant class: FGFR3 fusion

Other identifiers: ACCRU-GI-1701, NCI-2019-05877, VICCGI2028

Population segments: FGFR, Second line, Stage III, Stage IV

Phase: II

Therapy: pemigatinib

Location: United States

US States: AZ, GA, MN, NC, TN

Contact: Multiple contacts: See www.clinicaltrials.gov for complete list of contacts.

NCT04294277
Open-label, Single-arm, Phase II Study, Evaluating Safety and Efficacy of INCB054828 (Pemigatinib) as Adjuvant Therapy for Molecularly-selected, High-risk Patients With Urothelial Carcinoma Who Have Received Radical Surgery

Cancer type: Bladder Urothelial Carcinoma

Variant class: FGFR3 fusion

Other identifiers: EAU RF 2018-02, EAURF2018-02, EudraCT Number: 2017-004426-15, EudraCT Number: 2019-001833-14, PEGASUS

Population segments: Adjuvant, FGFR, Stage III, Stage IV

Phase: II

Therapy: pemigatinib

Location: Italy

FGFR3-TACC3 fusion (continued)

NCT04077255
Anti-EGFR Antibody (GC-1118) in Combination With Weekly Paclitaxel as a Second-line Therapy for Gastric and Gastroesophageal Junction Adenocarcinomas With Amplifications

Cancer type: Gastric Cancer, Gastroesophageal Junction Adenocarcinoma

Variant class: FGFR3 fusion

Other identifiers: 31973, KCT0004314, NCC2019-0196

Population segments: EGFR, FGFR, Second line, Stage IV

Phase: II

Therapies: rogaratinib, chemotherapy

Location: Republic of Korea

NCT04045613
An Open-label Multi-cohort Phase Ib/II Study of Derazantinib and Atezolizumab in Patients With Urothelial Cancer Expressing Activating Molecular FGFR Aberrations.

Cancer type: Bladder Urothelial Carcinoma

Variant class: FGFR3 fusion

Other inclusion criteria: CD274 expression

Other identifiers: CSET 3012, DZB-CS-201, EudraCT Number: 2019-000359-15, FIDES-02, NCI-2019-08162, RECF4112, SNCTP000004480

Population segments: FGFR, Fourth line or greater, Second line, Stage IV, Third line

Phase: I/II

Therapies: derazantinib, atezolizumab

Locations: Australia, Austria, Belgium, Canada, Czech Republic, France, Germany, Hungary, Italy, Poland, Republic of Korea, Spain, Switzerland, United Kingdom, United States

US States: GA, IL, KS, KY, NY, SC, TX, VA, WA

Contact: Dr. Manuel HÃ¤ckl [manuel.haeckl@basilea.com]

No NCT ID - see other identifier(s)
Phase I Clinical Study of BPI-17509 In Patients With Advanced Solid Tumors

Cancer type: Bladder Cancer, Solid Tumor

Variant class: FGFR3 fusion

Other identifiers: BTP-26811, CTR20192140

Population segments: Second line, Stage III, Stage IV

Phase: I

Therapy: BPI-17509

Location: China

NCT03827850
A Phase II Trial to Evaluate Efficacy and Safety of Erdafitinib in Patients With Advanced Squamous Non Small Cell Lung Carcinoma (sqNSCLC) Harboring Fibroblast Growth Factor Receptor (FGFR) Genetic Alterations After Relapse of Standard Therapy.

Cancer type: Non-Small Cell Lung Cancer

Variant class: FGFR fusion

Other identifiers: EudraCT Number: 2018-000399-13, FIND, FIND Trial, Uni-Koeln-3254

Population segments: FGFR, Second line, Squamous Cell, Stage III, Stage IV

Exclusion criteria variant classes: ALK aberration (Non-Small Cell Lung Cancer), BRAF aberration (Non-Small Cell Lung Cancer), EGFR aberration (Non-Small Cell Lung Cancer), NTRK aberration (Non-Small Cell Lung Cancer), ROS1 aberration (Non-Small Cell Lung Cancer)

Phase: II

Therapy: erdafitinib

Location: Germany

Disclaimer: The data presented here is from a curated knowledgebase of publicly available information, but may not be exhaustive. The data version is 2021.11(003).

FGFR3-TACC3 fusion (continued)

NCT04172675

A Randomized Phase II Study of Erdafitinib Versus Investigator Choice of Intravesical Chemotherapy in Subjects Who Received Bacillus Calmette-Guerin (BCG) and Recurred With High Risk Non-Muscle-Invasive Bladder Cancer (NMIBC) and FGFR Mutations or Fusions

Cancer type: Bladder Urothelial Carcinoma

Variant class: FGFR fusion

Other identifiers: 42756493BLC2003, 42756493BLC2003, CR108699, CTR20201841, CTRI/2021/08/036009, EudraCT Number: 2019-002449-39, JapicCTI-205145, NCI-2020-00674, RG1121102

Population segments: FGFR, Maintenance/Consolidation, Second line, Stage 0

Phase: II

Therapy: erdafitinib

Locations: Argentina, Australia, Belgium, Brazil, China, Czech Republic, France, Germany, Italy, Japan, Poland, Republic of Korea, Spain, Taiwan, United Kingdom, United States

US States: AZ, CA, CO, FL, GA, IL, KS, NC, NY, OH, PA, TN, TX, WA, WI

Contact: Study Contact [844-434-4210; JNJ.CT@sylogent.com]

NCT02150967

A Phase II Multicenter, Single Arm Study of Oral BGJ398 in Adult Patients with Advanced or Metastatic Cholangiocarcinoma with FGFR2 Gene Fusions or Other FGFR Genetic Alterations who Failed or are Intolerant to Platinum-based Chemotherapy.

Cancer type: Cholangiocarcinoma

Variant class: FGFR activating aberration

Other identifiers: 14-183, 14-2200, 14-328, 15452, 2014-0372, 2014-081, CBGJ398X2204, EudraCT Number: 2013-005085-19, NCI-2014-01310, REec-2014-1022, Trial 3GB-14-1

Population segments: FGFR, Fourth line or greater, Second line, Stage III, Stage IV, Third line

Phase: II

Therapy: infigratinib

Locations: Belgium, Germany, Singapore, Spain, Thailand, United Kingdom, United States

US States: CA, MA, MI, NY, OH, TX

Contact: QED Therapeutics [877-280-5655; clinicaltrials@QEDTx.com]

NCT04595747

Phase II Study of Rogaratinib (BAY 1163877) in the Treatment of Patients With Sarcoma Harboring Alterations in Fibroblast Growth Factor Receptor (FGFR) 1-4 and SDH-Deficient Gastrointestinal Stromal Tumor (GIST)

Cancer type: Soft Tissue Sarcoma

Variant class: FGFR3 aberration

Other identifiers: 10411, NCI-2020-08011

Population segments: FGFR, Second line, Stage III, Stage IV

Phase: II

Therapy: rogaratinib

Location: United States

US States: CA, MA, MD, NY

Contact: Multiple contacts: See www.clinicaltrials.gov for complete list of contacts.

Disclaimer: The data presented here is from a curated knowledgebase of publicly available information, but may not be exhaustive. The data version is 2021.11(003).

FGFR3-TACC3 fusion (continued)

NCT04149691
A Phase I, Open-label, Multicentre, Dose Escalation Study to Assess Safety, Tolerability and Pharmacokinetics of Oral CPL304110, in Adult Subjects With Advanced Solid Malignancies

Cancer type: Bladder Cancer, Gastric Cancer, Non-Small Cell Lung Cancer

Variant class: FGFR3 aberration

Other identifier: 01FGFR2018

Population segments: FGFR, Line of therapy N/A, Squamous Cell, Stage III, Stage IV

Phase: I

Therapy: CPL-304-110

Location: Poland

NCT03473743
A Phase Ib-II Study to Evaluate Safety, Efficacy, Pharmacokinetics, and Pharmacodynamics of Various Regimens of Erdafitinib in Subjects With Metastatic or Locally Advanced Urothelial Cancer

Cancer type: Bladder Urothelial Carcinoma

Variant class: FGFR aberration

Other identifiers: 17165, 2018-0142, 42756493BLC2002, CR108445, EudraCT Number: 2017-001980-19, NCI-2018-00997, NORSE, USO 17165, USOR 17165

Population segments: FGFR, First line, Fourth line or greater, Second line, Stage IV, Third line

Phase: I/II

Therapies: erdafitinib, cetrelimab

Locations: Belarus, Belgium, Brazil, France, Italy, Poland, Republic of Korea, Russian Federation, Spain, Taiwan, Turkey, United Kingdom, United States

US States: CO, MD, NC, NY, PA, TX, VA

Contact: Study Contact [844-434-4210; JNJ.CT@sylogent.com]

NCT02393248
A Phase I/II, Open-Label, Dose-Escalation, Safety and Tolerability Study of INCB054828 in Subjects With Advanced Malignancies - (FIGHT-101)

Cancer type: Bladder Urothelial Carcinoma, Breast Cancer, Cholangiocarcinoma, Endometrial Carcinoma, Gastric Cancer, Non-Small Cell Lung Cancer, Solid Tumor

Variant class: FGFR aberration

Other identifiers: 00061567, 2014-1099, 201503012, EudraCT Number: 2016-002831-14, FIGHT-101, INCB 54828-101, INCB54828-101, MC# 15-03, NCI-2015-00611, OSU-15241

Population segments: FGFR, First line, Fourth line or greater, Second line, Squamous Cell, Stage III, Stage IV, Third line

Phase: I/II

Therapies: pemigatinib, pembrolizumab, trastuzumab, chemotherapy, retifanlimab

Location: United States

US States: FL, NJ, TX

Contact: Incyte Call Center [855-463-3463]

Disclaimer: The data presented here is from a curated knowledgebase of publicly available information, but may not be exhaustive. The data version is 2021.11(003).

FGFR3-TACC3 fusion (continued)

NCT04572295

An Open-label Phase Ib Study of E7090 Monotherapy and in Combination With Other Anticancer Agents in Subjects With ER+, HER2- Recurrent/Metastatic Breast Cancer

Cancer type: Breast Cancer

Variant class: FGFR aberration

Other inclusion criteria: ERBB2 negative, ER positive

Other identifiers: E7090-J081-102, JapicCTI-205429

Population segments: Estrogen receptor positive, HER2 negative, Second line, Stage IV

Phase: I

Therapies: E-7090, hormone therapy

Location: Japan

NCT04504331

A Phase IB Study of Infigratinib in Combination With Tamoxifen in Hormone Receptor Positive, HER2 Negative, FGFR Altered Advanced Breast Cancer

Cancer type: Breast Cancer

Variant class: FGFR aberration

Other inclusion criteria: ERBB2 negative, Hormone receptor positive

Other identifiers: BRS0113, K08CA252457

Population segments: Estrogen receptor positive, HER2 negative, Progesterone receptor positive, Second line, Stage III, Stage IV, Triple receptor negative

Phase: I

Therapies: infigratinib, hormone therapy

Location: United States

US State: CA

Contact: Allison K. Zhang [650-736-5790; axkong@stanford.edu]

FGFR3-BAIAP2L1 fusion

No NCT ID - see other identifier(s)

A Multicenter Phase II Basket-type Clinical Trial to Evaluate Efficacy and Safety of TAS-120 in Patients with Advanced Solid Malignancies with FGFR Alterations in Circulating Tumor DNA

Cancer type: Unspecified Solid Tumor

Variant class: FGFR3 fusion

Other identifiers: EPOC 1805, JapicCTI-194624, JapicCTI-194796, TiFFANY

Population segments: Second line, Stage III, Stage IV

Phase: II

Therapy: futibatinib

Location: Japan

FGFR3-BAIAP2L1 fusion (continued)

NCT04189445
A Phase II Study of Futibatinib in Patients With Specific FGFR Aberrations

Cancer type: Unspecified Solid Tumor

Variant class: FGFR3 fusion

Other identifiers: EudraCT Number: 2019-004084-49, JapicCTI-205312, NCI-2020-03407, NL73545.056.20, TAS-120-202, UW20046

Population segments: (N/A), Aggressive, Classical, FGFR, Indolent, Nodular lymphocyte-predominant, Second line, Stage III, Stage IV, Third line

Exclusion criteria variant classes: FGFR1 fusion (Central Nervous System Neoplasm, Intrahepatic Cholangiocarcinoma), FGFR2 fusion (Central Nervous System Neoplasm, Intrahepatic Cholangiocarcinoma), FGFR3 fusion (Central Nervous System Neoplasm, Intrahepatic Cholangiocarcinoma), FGFR4 fusion (Central Nervous System Neoplasm, Intrahepatic Cholangiocarcinoma)

Phase: II

Therapy: futibatinib

Locations: Japan, Republic of Korea, United Kingdom, United States

US States: AZ, CA, TX, WI

Contact: Dr. Osamu Takahashi [609-250-7336; clinicaltrialinfo@taihooncology.com]

NCT04233567
A Phase II Study of Oral Infigratinib in Adult Patients With Advanced or Metastatic Solid Tumors With FGFR1-3 Gene Fusions or Other FGFR Genetic Alterations

Cancer type: Unspecified Solid Tumor

Variant class: FGFR3 fusion

Other identifiers: NCI-2019-06869, OSU-19041

Population segments: Second line, Stage III, Stage IV

Phase: II

Therapy: infigratinib

Location: United States

US State: OH

Contact: The Ohio State University Comprehensive Cancer Center [800-293-5066; OSUCCCClinicaltrials@osumc.edu]

NCT05019794
A Phase IIa of Infigratinib in Subjects With Locally Advanced or Metastatic Gastric Cancer or Gastroesophageal Junction Adenocarcinoma With FGFR2 Amplification or Other Advanced Solid Tumors With Other FGFR Alterations

Cancer type: Gastric Cancer, Gastroesophageal Junction Adenocarcinoma, Unspecified Solid Tumor

Variant class: FGFR3 fusion

Other identifiers: CTR20210275, LB1001-201

Population segments: FGFR, Fourth line or greater, Second line, Stage III, Stage IV, Third line

Phase: II

Therapy: infigratinib

Location: China

Disclaimer: The data presented here is from a curated knowledgebase of publicly available information, but may not be exhaustive. The data version is 2021.11(003).

FGFR3-BAIAP2L1 fusion (continued)

NCT03929965
Anlotinib in Advanced Solid Tumors With FGFR Alteration

Cancer type: Unspecified Solid Tumor

Variant class: FGFR fusion

Other identifier: ASTFA

Population segments: Second line, Stage III, Stage IV, Third line

Phase: I

Therapy: anlotinib hydrochloride

Location: China

NCT04591431
The Rome Trial From Histology to Target: the Road to Personalize Target Therapy and Immunotherapy

Cancer type: Biliary Tract Carcinoma, Breast Cancer, Colorectal Cancer, Esophageal Cancer, Gastric Cancer, Gastroesophageal Junction Adenocarcinoma, Hepatocellular Carcinoma, Liver Cancer, Non-Small Cell Lung Cancer, Pancreatic Cancer, Unspecified Solid Tumor

Variant class: FGFR3 aberration

Other identifiers: EudraCT Number: 2018-002190-21, MAR-BAS-18-005, ROME

Population segments: Second line, Stage IV

Phase: II

Therapy: pemigatinib

Location: Italy

NCT03297606
Canadian Profiling and Targeted Agent Utilization Trial (CAPTUR): A Phase II Basket Trial

Cancer type: Unspecified Solid Tumor

Variant class: FGFR3 aberration

Other identifiers: CA209-9DL, CAPTUR, ESR-17-12831, ML39800, PM1, WI233446

Population segments: Aggressive, Diffuse large B-cell lymphoma (DLBCL), Extranodal marginal zone B-cell lymphoma (MALT), First line, Follicular lymphoma (FL), Indolent, Lymphoblastic lymphoma (LBL), Mantle cell lymphoma (MCL), Other subtype, Second line, Stage III, Stage IV, Waldenstrom's macroglobulinemia (WM)

Phase: II

Therapy: sunitinib

Location: Canada

NCT04565275
A Multi-center Open-label, Phase I/II Clinical Trial to Evaluate the Safety, Tolerability, Pharmacokinetics, and Pharmacodynamics of ICP-192 in Patients With Advanced Solid Tumors and FGFR Gene Alterations.

Cancer type: Bladder Urothelial Carcinoma, Cholangiocarcinoma, Unspecified Solid Tumor

Variant class: FGFR aberration

Other identifier: ICP-CL-00303

Population segments: FGFR, Second line, Stage III, Stage IV

Phase: I/II

Therapy: ICP-192

Location: United States

US States: AZ, CO, FL, MN

Contact: Olivia Yang [609-524-0684; olivia.yang@INNOCAREPHARMA.COM]

Disclaimer: The data presented here is from a curated knowledgebase of publicly available information, but may not be exhaustive. The data version is 2021.11(003).

FGFR3-BAIAP2L1 fusion (continued)

No NCT ID - see other identifier(s)
Phase I/II Study of TAS-117 In Combination With TAS-120 In Patients With Advanced Solid Tumors

Cancer type: Unspecified Solid Tumor

Variant class: FGFR aberration

Other identifier: JapicCTI-194864

Population segments: FGFR, First line, Squamous Cell, Stage III, Stage IV

Phase: I/II

Therapies: TAS-117, futibatinib

Location: Japan

No NCT ID - see other identifier(s)
A Phase Ib Study to Assess the Safety, Tolerability, and Efficacy of TAS-120 (Futibatinib) in Combination with MK-3475 (Pembrolizumab) in Patients with Solid Tumors.

Cancer type: Unspecified Solid Tumor

Variant class: FGFR aberration

Other identifier: JapicCTI-195063

Population segments: FGFR, Line of therapy N/A, Stage III, Stage IV

Phase: I

Therapies: futibatinib, pembrolizumab

Location: Japan

NCT03390504
A Phase III Study of Erdafitinib Compared With Vinflunine or Docetaxel or Pembrolizumab in Subjects With Advanced Urothelial Cancer and Selected FGFR Gene Aberrations

Cancer type: Bladder Urothelial Carcinoma

Variant class: FGFR3-BAIAP2L1 fusion

Other identifiers: 2018-0027, 42756493BLC3001, 42756493-BLC3001, CR108401, CTR20182097, CTRI/2018/07/014718, EudraCT Number: 2017-002932-18, INT2, IRAS ID 238051, JapicCTI-183949, JNJ-42756493, NCI-2018-01443, NL64531.091.18, THOR

Population segments: FGFR, Second line, Stage III, Stage IV

Phase: III

Therapies: erdafitinib, pembrolizumab

Locations: Argentina, Australia, Austria, Belgium, Brazil, Canada, China, France, Germany, Greece, Hungary, Israel, Italy, Japan, Mexico, Netherlands, Poland, Portugal, Republic of Korea, Russian Federation, Spain, Taiwan, Turkey, Ukraine, United Kingdom, United States

US States: CA, DC, FL, IL, KY, MD, NC, NH, NV, NY, OH, TX, VA

Contact: Study Contact [844-434-4210; JNJ.CT@sylogent.com]

NCT02365597
A Phase II, Two-arm Multicenter, Open-Label Study to Determine the Efficacy and the Safety of Two Different Dose Regimens of a Pan-FGFR Tyrosine Kinase Inhibitor JNJ-42756493 in Subjects With Metastatic or Surgically Unresectable Urothelial Cancer With FGFR Genomic Alterations.

Cancer type: Bladder Urothelial Carcinoma

Variant class: FGFR3-BAIAP2L1 fusion

Other identifiers: 42756493BLC2001, BLC2001, CR105065, DRKS00009448, EudracT Number: 2014-002408-26, IRAS ID 177346, NCI-2015-00818, REec-2015-1451

Population segments: FGFR, First line, Fourth line or greater, Second line, Stage III, Stage IV, Third line

Phase: II

Therapy: erdafitinib

Locations: France, Spain, United States

US State: TX

Disclaimer: The data presented here is from a curated knowledgebase of publicly available information, but may not be exhaustive. The data version is 2021.11(003).

FGFR3-BAIAP2L1 fusion (continued)

NCT04492293
A Phase II, Multicenter, Single Arm, Open-lable Study of ICP-192 in Subjects with Surgically Unresectable or Metastatic Bladder Urothelial Cancer with FGFR Genetic Aberrations.

Cancer type: Bladder Urothelial Carcinoma

Variant class: FGFR3-BAIAP2L1 fusion

Other identifiers: CTR20200688, ICP-CL-00302

Population segments: FGFR, Second line, Stage III, Stage IV

Phase: II

Therapy: ICP-192

Location: China

NCT04197986
Phase III, Multicenter, Double-Blind, Randomized, Placebo-Controlled Trial of Infigratinib for the Adjuvant Treatment of Subjects With Invasive Urothelial Carcinoma With Susceptible FGFR3 Genetic Alterations (PROOF 302).

Cancer type: Bladder Urothelial Carcinoma

Variant class: FGFR3 fusion

Other identifiers: EudraCT Number: 2019-003248-63, NCI-2020-00610, PROOF 302, QBGJ398-302

Population segments: Adjuvant, FGFR, Stage II, Stage III

Exclusion criteria variant classes: FGFR3 A391E mutation (Bladder Small Cell Neuroendocrine Carcinoma), FGFR3 G370C mutation (Bladder Small Cell Neuroendocrine Carcinoma), FGFR3 K650E mutation (Bladder Small Cell Neuroendocrine Carcinoma), FGFR3 K650M mutation (Bladder Small Cell Neuroendocrine Carcinoma), FGFR3 K650Q mutation (Bladder Small Cell Neuroendocrine Carcinoma), FGFR3 K650T mutation (Bladder Small Cell Neuroendocrine Carcinoma), FGFR3 R248C mutation (Bladder Small Cell Neuroendocrine Carcinoma), FGFR3 S249C mutation (Bladder Small Cell Neuroendocrine Carcinoma), FGFR3 Y373C mutation (Bladder Small Cell Neuroendocrine Carcinoma), FGFR3 fusion (Bladder Small Cell Neuroendocrine Carcinoma)

Phase: III

Therapy: infigratinib

Locations: Belgium, Canada, France, Germany, Italy, Netherlands, Puerto Rico, Spain, United Kingdom, United States

US States: AL, AZ, CA, CO, DC, FL, GA, IL, IN, LA, MA, MD, MO, NC, NE, NJ, NY, OH, OK, SC, TN, TX, UT, VA, WA, WV

Contact: QED Therapeutics [877-280-5655; Proof302.ct@qedtx.com]

NCT04601857
A Phase II Study Evaluating Futibatinib (TAS 120) Plus Pembrolizumab in the Treatment of Advanced or Metastatic Urothelial Carcinoma

Cancer type: Bladder Urothelial Carcinoma

Variant class: FGFR3 fusion

Other identifiers: EudraCT Number: 2020-000945-15, TAS-120-203

Population segments: FGFR, First line, Stage III, Stage IV

Phase: II

Therapies: futibatinib, pembrolizumab

Location: United States

US State: MI

Contact: Dr. Maciej Gil [609-250-7336; clinicaltrialinfo@taihooncology.com]

Disclaimer: The data presented here is from a curated knowledgebase of publicly available information, but may not be exhaustive. The data version is 2021.11(003).

FGFR3-BAIAP2L1 fusion (continued)

NCT04936295
A Phase II Study of the Efficacy and Tolerability of Fulvestrant Plus Anlotinib in HR(+)/HER2(-) Metastatic Breast Cancer Patients With FGFR Mutation

Cancer type: Breast Cancer

Variant class: FGFR3 fusion

Other inclusion criteria: ERBB2 negative, Hormone receptor positive

Other identifier: SYSU005

Population segments: Estrogen receptor positive, FGFR, First line, HER2 negative, HER2 positive, Progesterone receptor positive, Stage IV

Phase: II

Therapies: hormone therapy, anlotinib hydrochloride

Location: China

NCT04096417
A Phase II, Multicenter, Single-Arm Study of Pemigatinib in Patients With Metastatic or Unresectable Colorectal Cancer Harboring FGFR Alterations

Cancer type: Colorectal Cancer

Variant class: FGFR3 fusion

Other identifiers: ACCRU-GI-1701, NCI-2019-05877, VICCGI2028

Population segments: FGFR, Second line, Stage III, Stage IV

Phase: II

Therapy: pemigatinib

Location: United States

US States: AZ, GA, MN, NC, TN

Contact: Multiple contacts: See www.clinicaltrials.gov for complete list of contacts.

NCT04294277
Open-label, Single-arm, Phase II Study, Evaluating Safety and Efficacy of INCB054828 (Pemigatinib) as Adjuvant Therapy for Molecularly-selected, High-risk Patients With Urothelial Carcinoma Who Have Received Radical Surgery

Cancer type: Bladder Urothelial Carcinoma

Variant class: FGFR3 fusion

Other identifiers: EAU RF 2018-02, EAURF2018-02, EudraCT Number: 2017-004426-15, EudraCT Number: 2019-001833-14, PEGASUS

Population segments: Adjuvant, FGFR, Stage III, Stage IV

Phase: II

Therapy: pemigatinib

Location: Italy

NCT04077255
Anti-EGFR Antibody (GC-1118) in Combination With Weekly Paclitaxel as a Second-line Therapy for Gastric and Gastroesophageal Junction Adenocarcinomas With Amplifications

Cancer type: Gastric Cancer, Gastroesophageal Junction Adenocarcinoma

Variant class: FGFR3 fusion

Other identifiers: 31973, KCT0004314, NCC2019-0196

Population segments: EGFR, FGFR, Second line, Stage IV

Phase: II

Therapies: rogaratinib, chemotherapy

Location: Republic of Korea

Disclaimer: The data presented here is from a curated knowledgebase of publicly available information, but may not be exhaustive. The data version is 2021.11(003).

FGFR3-BAIAP2L1 fusion (continued)

NCT04045613

An Open-label Multi-cohort Phase Ib/II Study of Derazantinib and Atezolizumab in Patients With Urothelial Cancer Expressing Activating Molecular FGFR Aberrations.

Cancer type: Bladder Urothelial Carcinoma

Variant class: FGFR3 fusion

Other inclusion criteria: CD274 expression

Other identifiers: CSET 3012, DZB-CS-201, EudraCT Number: 2019-000359-15, FIDES-02, NCI-2019-08162, RECF4112, SNCTP000004480

Population segments: FGFR, Fourth line or greater, Second line, Stage IV, Third line

Phase: I/II

Therapies: derazantinib, atezolizumab

Locations: Australia, Austria, Belgium, Canada, Czech Republic, France, Germany, Hungary, Italy, Poland, Republic of Korea, Spain, Switzerland, United Kingdom, United States

US States: GA, IL, KS, KY, NY, SC, TX, VA, WA

Contact: Dr. Manuel HÃ¤ckl [manuel.haeckl@basilea.com]

No NCT ID - see other identifier(s)

Phase I Clinical Study of BPI-17509 In Patients With Advanced Solid Tumors

Cancer type: Bladder Cancer, Solid Tumor

Variant class: FGFR3 fusion

Other identifiers: BTP-26811, CTR20192140

Population segments: Second line, Stage III, Stage IV

Phase: I

Therapy: BPI-17509

Location: China

NCT03827850

A Phase II Trial to Evaluate Efficacy and Safety of Erdafitinib in Patients With Advanced Squamous Non Small Cell Lung Carcinoma (sqNSCLC) Harboring Fibroblast Growth Factor Receptor (FGFR) Genetic Alterations After Relapse of Standard Therapy.

Cancer type: Non-Small Cell Lung Cancer

Variant class: FGFR fusion

Other identifiers: EudraCT Number: 2018-000399-13, FIND, FIND Trial, Uni-Koeln-3254

Population segments: FGFR, Second line, Squamous Cell, Stage III, Stage IV

Exclusion criteria variant classes: ALK aberration (Non-Small Cell Lung Cancer), BRAF aberration (Non-Small Cell Lung Cancer), EGFR aberration (Non-Small Cell Lung Cancer), NTRK aberration (Non-Small Cell Lung Cancer), ROS1 aberration (Non-Small Cell Lung Cancer)

Phase: II

Therapy: erdafitinib

Location: Germany

Disclaimer: The data presented here is from a curated knowledgebase of publicly available information, but may not be exhaustive. The data version is 2021.11(003).

FGFR3-BAIAP2L1 fusion (continued)

NCT04172675
A Randomized Phase II Study of Erdafitinib Versus Investigator Choice of Intravesical Chemotherapy in Subjects Who Received Bacillus Calmette-Guerin (BCG) and Recurred With High Risk Non-Muscle-Invasive Bladder Cancer (NMIBC) and FGFR Mutations or Fusions

Cancer type: Bladder Urothelial Carcinoma

Variant class: FGFR fusion

Other identifiers: 42756493BLC2003, 42756493BLC2003, CR108699, CTR20201841, CTRI/2021/08/036009, EudraCT Number: 2019-002449-39, JapicCTI-205145, NCI-2020-00674, RG1121102

Population segments: FGFR, Maintenance/Consolidation, Second line, Stage 0

Phase: II

Therapy: erdafitinib

Locations: Argentina, Australia, Belgium, Brazil, China, Czech Republic, France, Germany, Italy, Japan, Poland, Republic of Korea, Spain, Taiwan, United Kingdom, United States

US States: AZ, CA, CO, FL, GA, IL, KS, NC, NY, OH, PA, TN, TX, WA, WI

Contact: Study Contact [844-434-4210; JNJ.CT@sylogent.com]

NCT02150967
A Phase II Multicenter, Single Arm Study of Oral BGJ398 in Adult Patients with Advanced or Metastatic Cholangiocarcinoma with FGFR2 Gene Fusions or Other FGFR Genetic Alterations who Failed or are Intolerant to Platinum-based Chemotherapy.

Cancer type: Cholangiocarcinoma

Variant class: FGFR activating aberration

Other identifiers: 14-183, 14-2200, 14-328, 15452, 2014-0372, 2014-081, CBGJ398X2204, EudraCT Number: 2013-005085-19, NCI-2014-01310, REec-2014-1022, Trial 3GB-14-1

Population segments: FGFR, Fourth line or greater, Second line, Stage III, Stage IV, Third line

Phase: II

Therapy: infigratinib

Locations: Belgium, Germany, Singapore, Spain, Thailand, United Kingdom, United States

US States: CA, MA, MI, NY, OH, TX

Contact: QED Therapeutics [877-280-5655; clinicaltrials@QEDTx.com]

NCT04595747
Phase II Study of Rogaratinib (BAY 1163877) in the Treatment of Patients With Sarcoma Harboring Alterations in Fibroblast Growth Factor Receptor (FGFR) 1-4 and SDH-Deficient Gastrointestinal Stromal Tumor (GIST)

Cancer type: Soft Tissue Sarcoma

Variant class: FGFR3 aberration

Other identifiers: 10411, NCI-2020-08011

Population segments: FGFR, Second line, Stage III, Stage IV

Phase: II

Therapy: rogaratinib

Location: United States

US States: CA, MA, MD, NY

Contact: Multiple contacts: See www.clinicaltrials.gov for complete list of contacts.

Disclaimer: The data presented here is from a curated knowledgebase of publicly available information, but may not be exhaustive. The data version is 2021.11(003).

FGFR3-BAIAP2L1 fusion (continued)

NCT04149691

A Phase I, Open-label, Multicentre, Dose Escalation Study to Assess Safety, Tolerability and Pharmacokinetics of Oral CPL304110, in Adult Subjects With Advanced Solid Malignancies

Cancer type: Bladder Cancer, Gastric Cancer, Non-Small Cell Lung Cancer

Variant class: FGFR3 aberration

Other identifier: 01FGFR2018

Population segments: FGFR, Line of therapy N/A, Squamous Cell, Stage III, Stage IV

Phase: I

Therapy: CPL-304-110

Location: Poland

NCT03473743

A Phase Ib-II Study to Evaluate Safety, Efficacy, Pharmacokinetics, and Pharmacodynamics of Various Regimens of Erdafitinib in Subjects With Metastatic or Locally Advanced Urothelial Cancer

Cancer type: Bladder Urothelial Carcinoma

Variant class: FGFR aberration

Other identifiers: 17165, 2018-0142, 42756493BLC2002, CR108445, EudraCT Number: 2017-001980-19, NCI-2018-00997, NORSE, USO 17165, USOR 17165

Population segments: FGFR, First line, Fourth line or greater, Second line, Stage IV, Third line

Phase: I/II

Therapies: erdafitinib, cetrelimab

Locations: Belarus, Belgium, Brazil, France, Italy, Poland, Republic of Korea, Russian Federation, Spain, Taiwan, Turkey, United Kingdom, United States

US States: CO, MD, NC, NY, PA, TX, VA

Contact: Study Contact [844-434-4210; JNJ.CT@sylogent.com]

NCT02393248

A Phase I/II, Open-Label, Dose-Escalation, Safety and Tolerability Study of INCB054828 in Subjects With Advanced Malignancies - (FIGHT-101)

Cancer type: Bladder Urothelial Carcinoma, Breast Cancer, Cholangiocarcinoma, Endometrial Carcinoma, Gastric Cancer, Non-Small Cell Lung Cancer, Solid Tumor

Variant class: FGFR aberration

Other identifiers: 00061567, 2014-1099, 201503012, EudraCT Number: 2016-002831-14, FIGHT-101, INCB 54828-101, INCB54828-101, MC# 15-03, NCI-2015-00611, OSU-15241

Population segments: FGFR, First line, Fourth line or greater, Second line, Squamous Cell, Stage III, Stage IV, Third line

Phase: I/II

Therapies: pemigatinib, pembrolizumab, trastuzumab, chemotherapy, retifanlimab

Location: United States

US States: FL, NJ, TX

Contact: Incyte Call Center [855-463-3463]

Disclaimer: The data presented here is from a curated knowledgebase of publicly available information, but may not be exhaustive. The data version is 2021.11(003).

FGFR3-BAIAP2L1 fusion (continued)

NCT04572295
An Open-label Phase Ib Study of E7090 Monotherapy and in Combination With Other Anticancer Agents in Subjects With ER+, HER2- Recurrent/Metastatic Breast Cancer

Cancer type: Breast Cancer

Variant class: FGFR aberration

Other inclusion criteria: ERBB2 negative, ER positive

Other identifiers: E7090-J081-102, JapicCTI-205429

Population segments: Estrogen receptor positive, HER2 negative, Second line, Stage IV

Phase: I

Therapies: E-7090, hormone therapy

Location: Japan

NCT04504331
A Phase IB Study of Infigratinib in Combination With Tamoxifen in Hormone Receptor Positive, HER2 Negative, FGFR Altered Advanced Breast Cancer

Cancer type: Breast Cancer

Variant class: FGFR aberration

Other inclusion criteria: ERBB2 negative, Hormone receptor positive

Other identifiers: BRS0113, K08CA252457

Population segments: Estrogen receptor positive, HER2 negative, Progesterone receptor positive, Second line, Stage III, Stage IV, Triple receptor negative

Phase: I

Therapies: infigratinib, hormone therapy

Location: United States

US State: CA

Contact: Allison K. Zhang [650-736-5790; axkong@stanford.edu]

FGFR3 Y373C mutation

NCT03155620
NCI-COG Pediatric MATCH (Molecular Analysis for Therapy Choice) Screening Protocol

Cancer type: Unspecified Solid Tumor

Variant class: FGFR3 mutation

Other identifiers: 17-729, 2017-0625, 20170841, APEC1621SC, COGAPEC1621, COGAPEC1621SC, N 55017 SC, NCI-2017-01251, NCI-COG Pediatric MATCH, Pediatric MATCH

Population segments: ALK, Aggressive, BRAF, FGFR, HRAS, Indolent, KRAS, NRAS, Pediatric or Adolescent, Second line, Stage III, Stage IV

Phase: II

Therapy: erdafitinib

Locations: Puerto Rico, United States

US States: AK, AL, AR, AZ, CA, CO, CT, DC, DE, FL, GA, HI, IA, ID, IL, IN, KY, LA, MA, MD, ME, MI, MN, MO, MS, NC, NE, NH, NJ, NV, NY, OH, OK, OR, PA, RI, SC, SD, TN, TX, UT, VA, VT, WA, WI

Contact: Multiple contacts: See www.clinicaltrials.gov for complete list of contacts.

Disclaimer: The data presented here is from a curated knowledgebase of publicly available information, but may not be exhaustive. The data version is 2021.11(003).

FGFR3 Y373C mutation (continued)

NCT03210714
NCI-COG Pediatric MATCH (Molecular Analysis for Therapy Choice) - Phase II Subprotocol of JNJ-42756493 (Erdafitinib) in Patients With Tumors Harboring FGFR 1/2/3/4 Alterations

Cancer type: Unspecified Solid Tumor

Variant class: FGFR3 mutation

Other identifiers: APEC1621B, NCI-2017-01159, NCI-COG Pediatric MATCH, Pediatric MATCH

Population segments: Aggressive, FGFR, Indolent, Pediatric or Adolescent, Second line, Stage III, Stage IV

Phase: II

Therapy: erdafitinib

Locations: Puerto Rico, United States

US States: AK, AL, AR, AZ, CA, CO, CT, DC, DE, FL, GA, IA, ID, IL, IN, KY, LA, MD, ME, MI, MN, MO, MS, NC, ND, NE, NJ, NY, OH, OK, OR, PA, SC, SD, TN, TX, UT, VA, VT, WA, WI

Contact: Multiple contacts: See www.clinicaltrials.gov for complete list of contacts.

No NCT ID - see other identifier(s)
A Multicenter Phase II Basket-type Clinical Trial to Evaluate Efficacy and Safety of TAS-120 in Patients with Advanced Solid Malignancies with FGFR Alterations in Circulating Tumor DNA

Cancer type: Unspecified Solid Tumor

Variant class: FGFR3 mutation

Other identifiers: EPOC 1805, JapicCTI-194624, JapicCTI-194796, TiFFANY

Population segments: Second line, Stage III, Stage IV

Phase: II

Therapy: futibatinib

Location: Japan

NCT02693535
Targeted Agent and Profiling Utilization Registry (TAPUR) Study.

Cancer type: Unspecified Solid Tumor

Variant class: FGFR3 mutation

Other identifiers: 20170529, NCI-2017-00510, Pro00014171, TAPUR

Population segments: Aggressive, BRAF, Diffuse large B-cell lymphoma (DLBCL), Extranodal marginal zone B-cell lymphoma (MALT), FGFR, First line, Follicular lymphoma (FL), Fourth line or greater, Indolent, Lymphoblastic lymphoma (LBL), Mantle cell lymphoma (MCL), Other subtype, Second line, Small lymphocytic lymphoma (SLL), Stage III, Stage IV, Third line, Waldenstrom's macroglobulinemia (WM)

Phase: II

Therapy: sunitinib

Location: United States

US States: AL, AZ, CA, FL, GA, HI, IL, IN, ME, MI, NC, ND, NE, NH, OH, OR, PA, SD, TX, UT, VA, WA

Contact: Pam Mangat [pam.mangat@asco.org]

Disclaimer: The data presented here is from a curated knowledgebase of publicly available information, but may not be exhaustive. The data version is 2021.11(003).

FGFR3 Y373C mutation (continued)

NCT04591431
The Rome Trial From Histology to Target: the Road to Personalize Target Therapy and Immunotherapy

Cancer type: Biliary Tract Carcinoma, Breast Cancer, Colorectal Cancer, Esophageal Cancer, Gastric Cancer, Gastroesophageal Junction Adenocarcinoma, Hepatocellular Carcinoma, Liver Cancer, Non-Small Cell Lung Cancer, Pancreatic Cancer, Unspecified Solid Tumor

Variant class: FGFR3 aberration

Other identifiers: EudraCT Number: 2018-002190-21, MAR-BAS-18-005, ROME

Population segments: Second line, Stage IV

Phase: II

Therapy: pemigatinib

Location: Italy

NCT02691793
Study to Evaluate the Safety and Efficacy of Sunitinib, in Subject With Refractory Solid Tumors

Cancer type: Unspecified Solid Tumor

Variant class: FGFR mutation

Other identifier: 2016-02-098

Population segments: (N/A), FGFR, Second line

Phase: II

Therapy: sunitinib

Location: Republic of Korea

NCT03297606
Canadian Profiling and Targeted Agent Utilization Trial (CAPTUR): A Phase II Basket Trial

Cancer type: Unspecified Solid Tumor

Variant class: FGFR3 aberration

Other identifiers: CA209-9DL, CAPTUR, ESR-17-12831, ML39800, PM1, WI233446

Population segments: Aggressive, Diffuse large B-cell lymphoma (DLBCL), Extranodal marginal zone B-cell lymphoma (MALT), First line, Follicular lymphoma (FL), Indolent, Lymphoblastic lymphoma (LBL), Mantle cell lymphoma (MCL), Other subtype, Second line, Stage III, Stage IV, Waldenstrom's macroglobulinemia (WM)

Phase: II

Therapy: sunitinib

Location: Canada

NCT03929965
Anlotinib in Advanced Solid Tumors With FGFR Alteration

Cancer type: Unspecified Solid Tumor

Variant class: FGFR mutation

Other identifier: ASTFA

Population segments: Second line, Stage III, Stage IV, Third line

Phase: I

Therapy: anlotinib hydrochloride

Location: China

Disclaimer: The data presented here is from a curated knowledgebase of publicly available information, but may not be exhaustive. The data version is 2021.11(003).

FGFR3 Y373C mutation (continued)

NCT04565275
A Multi-center Open-label, Phase I/II Clinical Trial to Evaluate the Safety, Tolerability, Pharmacokinetics, and Pharmacodynamics of ICP-192 in Patients With Advanced Solid Tumors and FGFR Gene Alterations.

Cancer type: Bladder Urothelial Carcinoma, Cholangiocarcinoma, Unspecified Solid Tumor

Variant class: FGFR aberration

Other identifier: ICP-CL-00303

Population segments: FGFR, Second line, Stage III, Stage IV

Phase: I/II

Therapy: ICP-192

Location: United States

US States: AZ, CO, FL, MN

Contact: Olivia Yang [609-524-0684; olivia.yang@INNOCAREPHARMA.COM]

No NCT ID - see other identifier(s)
Phase I/II Study of TAS-117 In Combination With TAS-120 In Patients With Advanced Solid Tumors

Cancer type: Unspecified Solid Tumor

Variant class: FGFR aberration

Other identifier: JapicCTI-194864

Population segments: FGFR, First line, Squamous Cell, Stage III, Stage IV

Phase: I/II

Therapies: TAS-117, futibatinib

Location: Japan

No NCT ID - see other identifier(s)
A Phase Ib Study to Assess the Safety, Tolerability, and Efficacy of TAS-120 (Futibatinib) in Combination with MK-3475 (Pembrolizumab) in Patients with Solid Tumors.

Cancer type: Unspecified Solid Tumor

Variant class: FGFR aberration

Other identifier: JapicCTI-195063

Population segments: FGFR, Line of therapy N/A, Stage III, Stage IV

Phase: I

Therapies: futibatinib, pembrolizumab

Location: Japan

NCT03390504
A Phase III Study of Erdafitinib Compared With Vinflunine or Docetaxel or Pembrolizumab in Subjects With Advanced Urothelial Cancer and Selected FGFR Gene Aberrations

Cancer type: Bladder Urothelial Carcinoma

Variant class: FGFR3 Y373C mutation

Other identifiers: 2018-0027, 42756493BLC3001, 42756493-BLC3001, CR108401, CTR20182097, CTRI/2018/07/014718, EudraCT Number: 2017-002932-18, INT2, IRAS ID 238051, JapicCTI-183949, JNJ-42756493, NCI-2018-01443, NL64531.091.18, THOR

Population segments: FGFR, Second line, Stage III, Stage IV

Phase: III

Therapies: erdafitinib, pembrolizumab

Locations: Argentina, Australia, Austria, Belgium, Brazil, Canada, China, France, Germany, Greece, Hungary, Israel, Italy, Japan, Mexico, Netherlands, Poland, Portugal, Republic of Korea, Russian Federation, Spain, Taiwan, Turkey, Ukraine, United Kingdom, United States

US States: CA, DC, FL, IL, KY, MD, NC, NH, NV, NY, OH, TX, VA

Contact: Study Contact [844-434-4210; JNJ.CT@sylogent.com]

Disclaimer: The data presented here is from a curated knowledgebase of publicly available information, but may not be exhaustive. The data version is 2021.11(003).

FGFR3 Y373C mutation (continued)

NCT04197986

Phase III, Multicenter, Double-Blind, Randomized, Placebo-Controlled Trial of Infigratinib for the Adjuvant Treatment of Subjects With Invasive Urothelial Carcinoma With Susceptible FGFR3 Genetic Alterations (PROOF 302).

Cancer type: Bladder Urothelial Carcinoma

Variant class: FGFR3 Y373C mutation

Other identifiers: EudraCT Number: 2019-003248-63, NCI-2020-00610, PROOF 302, QBGJ398-302

Population segments: Adjuvant, FGFR, Stage II, Stage III

Exclusion criteria variant classes: FGFR3 A391E mutation (Bladder Small Cell Neuroendocrine Carcinoma), FGFR3 G370C mutation (Bladder Small Cell Neuroendocrine Carcinoma), FGFR3 K650E mutation (Bladder Small Cell Neuroendocrine Carcinoma), FGFR3 K650M mutation (Bladder Small Cell Neuroendocrine Carcinoma), FGFR3 K650Q mutation (Bladder Small Cell Neuroendocrine Carcinoma), FGFR3 K650T mutation (Bladder Small Cell Neuroendocrine Carcinoma), FGFR3 R248C mutation (Bladder Small Cell Neuroendocrine Carcinoma), FGFR3 S249C mutation (Bladder Small Cell Neuroendocrine Carcinoma), FGFR3 Y373C mutation (Bladder Small Cell Neuroendocrine Carcinoma), FGFR3 fusion (Bladder Small Cell Neuroendocrine Carcinoma)

Phase: III

Therapy: infigratinib

Locations: Belgium, Canada, France, Germany, Italy, Netherlands, Puerto Rico, Spain, United Kingdom, United States

US States: AL, AZ, CA, CO, DC, FL, GA, IL, IN, LA, MA, MD, MO, NC, NE, NJ, NY, OH, OK, SC, TN, TX, UT, VA, WA, WV

Contact: QED Therapeutics [877-280-5655; Proof302.ct@qedtx.com]

NCT02365597

A Phase II, Two-arm Multicenter, Open-Label Study to Determine the Efficacy and the Safety of Two Different Dose Regimens of a Pan-FGFR Tyrosine Kinase Inhibitor JNJ-42756493 in Subjects With Metastatic or Surgically Unresectable Urothelial Cancer With FGFR Genomic Alterations.

Cancer type: Bladder Urothelial Carcinoma

Variant class: FGFR3 Y373C mutation

Other identifiers: 42756493BLC2001, BLC2001, CR105065, DRKS00009448, EudracT Number: 2014-002408-26, IRAS ID 177346, NCI-2015-00818, REec-2015-1451

Population segments: FGFR, First line, Fourth line or greater, Second line, Stage III, Stage IV, Third line

Phase: II

Therapy: erdafitinib

Locations: France, Spain, United States

US State: TX

NCT04492293

A Phase II, Multicenter, Single Arm, Open-lable Study of ICP-192 in Subjects with Surgically Unresectable or Metastatic Bladder Urothelial Cancer with FGFR Genetic Aberrations.

Cancer type: Bladder Urothelial Carcinoma

Variant class: FGFR3 Y373C mutation

Other identifiers: CTR20200688, ICP-CL-00302

Population segments: FGFR, Second line, Stage III, Stage IV

Phase: II

Therapy: ICP-192

Location: China

Disclaimer: The data presented here is from a curated knowledgebase of publicly available information, but may not be exhaustive. The data version is 2021.11(003).

FGFR3 Y373C mutation (continued)

NCT04053322

An International Multicenter Phase II Trial of Durvalumab (MEDI4736) Plus OLAparib Plus Fulvestrant in Metastatic or Locally Advanced ER-positive, HER2-negative Breast Cancer Patients Selected Using Criteria That Predict Sensitivity to Olaparib

Cancer type: Breast Cancer

Variant class: FGFR3 mutation

Other inclusion criteria: ERBB2 negative, ER positive

Other identifiers: CADUSEIME08, DOLAF, EudraCT Number: 2018-003832-57, UC-0140/1812

Population segments: BRCA, Estrogen receptor positive, FGFR, HER2 negative, Progesterone receptor positive, Second line, Stage III, Stage IV

Phase: II

Therapies: durvalumab, olaparib, hormone therapy

Location: France

NCT04601857

A Phase II Study Evaluating Futibatinib (TAS 120) Plus Pembrolizumab in the Treatment of Advanced or Metastatic Urothelial Carcinoma

Cancer type: Bladder Urothelial Carcinoma

Variant class: FGFR3 mutation

Other identifiers: EudraCT Number: 2020-000945-15, TAS-120-203

Population segments: FGFR, First line, Stage III, Stage IV

Phase: II

Therapies: futibatinib, pembrolizumab

Location: United States

US State: MI

Contact: Dr. Maciej Gil [609-250-7336; clinicaltrialinfo@taihooncology.com]

NCT04096417

A Phase II, Multicenter, Single-Arm Study of Pemigatinib in Patients With Metastatic or Unresectable Colorectal Cancer Harboring FGFR Alterations

Cancer type: Colorectal Cancer

Variant class: FGFR3 mutation

Other identifiers: ACCRU-GI-1701, NCI-2019-05877, VICCGI2028

Population segments: FGFR, Second line, Stage III, Stage IV

Phase: II

Therapy: pemigatinib

Location: United States

US States: AZ, GA, MN, NC, TN

Contact: Multiple contacts: See www.clinicaltrials.gov for complete list of contacts.

NCT04294277

Open-label, Single-arm, Phase II Study, Evaluating Safety and Efficacy of INCB054828 (Pemigatinib) as Adjuvant Therapy for Molecularly-selected, High-risk Patients With Urothelial Carcinoma Who Have Received Radical Surgery

Cancer type: Bladder Urothelial Carcinoma

Variant class: FGFR3 mutation

Other identifiers: EAU RF 2018-02, EAURF2018-02, EudraCT Number: 2017-004426-15, EudraCT Number: 2019-001833-14, PEGASUS

Population segments: Adjuvant, FGFR, Stage III, Stage IV

Phase: II

Therapy: pemigatinib

Location: Italy

Disclaimer: The data presented here is from a curated knowledgebase of publicly available information, but may not be exhaustive. The data version is 2021.11(003).

FGFR3 Y373C mutation (continued)

NCT04077255
Anti-EGFR Antibody (GC-1118) in Combination With Weekly Paclitaxel as a Second-line Therapy for Gastric and Gastroesophageal Junction Adenocarcinomas With Amplifications

Cancer type: Gastric Cancer, Gastroesophageal Junction Adenocarcinoma

Variant class: FGFR3 mutation

Other identifiers: 31973, KCT0004314, NCC2019-0196

Population segments: EGFR, FGFR, Second line, Stage IV

Phase: II

Therapies: rogaratinib, chemotherapy

Location: Republic of Korea

No NCT ID - see other identifier(s)
Phase I Clinical Study of BPI-17509 In Patients With Advanced Solid Tumors

Cancer type: Bladder Cancer, Solid Tumor

Variant class: FGFR3 mutation

Other identifiers: BTP-26811, CTR20192140

Population segments: Second line, Stage III, Stage IV

Phase: I

Therapy: BPI-17509

Location: China

NCT04172675
A Randomized Phase II Study of Erdafitinib Versus Investigator Choice of Intravesical Chemotherapy in Subjects Who Received Bacillus Calmette-Guerin (BCG) and Recurred With High Risk Non-Muscle-Invasive Bladder Cancer (NMIBC) and FGFR Mutations or Fusions

Cancer type: Bladder Urothelial Carcinoma

Variant class: FGFR mutation

Other identifiers: 42756493BLC2003, 42756493BLC2003, CR108699, CTR20201841, CTRI/2021/08/036009, EudraCT Number: 2019-002449-39, JapicCTI-205145, NCI-2020-00674, RG1121102

Population segments: FGFR, Maintenance/Consolidation, Second line, Stage 0

Phase: II

Therapy: erdafitinib

Locations: Argentina, Australia, Belgium, Brazil, China, Czech Republic, France, Germany, Italy, Japan, Poland, Republic of Korea, Spain, Taiwan, United Kingdom, United States

US States: AZ, CA, CO, FL, GA, IL, KS, NC, NY, OH, PA, TN, TX, WA, WI

Contact: Study Contact [844-434-4210; JNJ.CT@sylogent.com]

NCT04595747
Phase II Study of Rogaratinib (BAY 1163877) in the Treatment of Patients With Sarcoma Harboring Alterations in Fibroblast Growth Factor Receptor (FGFR) 1-4 and SDH-Deficient Gastrointestinal Stromal Tumor (GIST)

Cancer type: Soft Tissue Sarcoma

Variant class: FGFR3 aberration

Other identifiers: 10411, NCI-2020-08011

Population segments: FGFR, Second line, Stage III, Stage IV

Phase: II

Therapy: rogaratinib

Location: United States

US States: CA, MA, MD, NY

Contact: Multiple contacts: See www.clinicaltrials.gov for complete list of contacts.

FGFR3 Y373C mutation (continued)

NCT04004975
Phase II Clinical Trials on Anlotinib for the Treatment of Recurrent Glioblastoma

Cancer type: Glioblastoma

Variant class: FGFR mutation

Other identifier: ShandongCHI006

Population segments: (N/A), FGFR, Second line

Phase: I/II

Therapy: anlotinib hydrochloride

Location: China

NCT04149691
A Phase I, Open-label, Multicentre, Dose Escalation Study to Assess Safety, Tolerability and Pharmacokinetics of Oral CPL304110, in Adult Subjects With Advanced Solid Malignancies

Cancer type: Bladder Cancer, Gastric Cancer, Non-Small Cell Lung Cancer

Variant class: FGFR3 aberration

Other identifier: 01FGFR2018

Population segments: FGFR, Line of therapy N/A, Squamous Cell, Stage III, Stage IV

Phase: I

Therapy: CPL-304-110

Location: Poland

NCT03473743
A Phase Ib-II Study to Evaluate Safety, Efficacy, Pharmacokinetics, and Pharmacodynamics of Various Regimens of Erdafitinib in Subjects With Metastatic or Locally Advanced Urothelial Cancer

Cancer type: Bladder Urothelial Carcinoma

Variant class: FGFR aberration

Other identifiers: 17165, 2018-0142, 42756493BLC2002, CR108445, EudraCT Number: 2017-001980-19, NCI-2018-00997, NORSE, USO 17165, USOR 17165

Population segments: FGFR, First line, Fourth line or greater, Second line, Stage IV, Third line

Phase: I/II

Therapies: erdafitinib, cetrelimab

Locations: Belarus, Belgium, Brazil, France, Italy, Poland, Republic of Korea, Russian Federation, Spain, Taiwan, Turkey, United Kingdom, United States

US States: CO, MD, NC, NY, PA, TX, VA

Contact: Study Contact [844-434-4210; JNJ.CT@sylogent.com]

NCT02393248
A Phase I/II, Open-Label, Dose-Escalation, Safety and Tolerability Study of INCB054828 in Subjects With Advanced Malignancies - (FIGHT-101)

Cancer type: Bladder Urothelial Carcinoma, Breast Cancer, Cholangiocarcinoma, Endometrial Carcinoma, Gastric Cancer, Non-Small Cell Lung Cancer, Solid Tumor

Variant class: FGFR aberration

Other identifiers: 00061567, 2014-1099, 201503012, EudraCT Number: 2016-002831-14, FIGHT-101, INCB 54828-101, INCB54828-101, MC# 15-03, NCI-2015-00611, OSU-15241

Population segments: FGFR, First line, Fourth line or greater, Second line, Squamous Cell, Stage III, Stage IV, Third line

Phase: I/II

Therapies: pemigatinib, pembrolizumab, trastuzumab, chemotherapy, retifanlimab

Location: United States

US States: FL, NJ, TX

Contact: Incyte Call Center [855-463-3463]

Disclaimer: The data presented here is from a curated knowledgebase of publicly available information, but may not be exhaustive. The data version is 2021.11(003).

FGFR3 Y373C mutation (continued)

NCT04572295
An Open-label Phase Ib Study of E7090 Monotherapy and in Combination With Other Anticancer Agents in Subjects With ER+, HER2- Recurrent/Metastatic Breast Cancer

Cancer type: Breast Cancer

Variant class: FGFR aberration

Other inclusion criteria: ERBB2 negative, ER positive

Other identifiers: E7090-J081-102, JapicCTI-205429

Population segments: Estrogen receptor positive, HER2 negative, Second line, Stage IV

Phase: I

Therapies: E-7090, hormone therapy

Location: Japan

NCT04504331
A Phase IB Study of Infigratinib in Combination With Tamoxifen in Hormone Receptor Positive, HER2 Negative, FGFR Altered Advanced Breast Cancer

Cancer type: Breast Cancer

Variant class: FGFR aberration

Other inclusion criteria: ERBB2 negative, Hormone receptor positive

Other identifiers: BRS0113, K08CA252457

Population segments: Estrogen receptor positive, HER2 negative, Progesterone receptor positive, Second line, Stage III, Stage IV, Triple receptor negative

Phase: I

Therapies: infigratinib, hormone therapy

Location: United States

US State: CA

Contact: Allison K. Zhang [650-736-5790; axkong@stanford.edu]

FGFR3 K650E mutation

NCT05019794
A Phase IIa of Infigratinib in Subjects With Locally Advanced or Metastatic Gastric Cancer or Gastroesophageal Junction Adenocarcinoma With FGFR2 Amplification or Other Advanced Solid Tumors With Other FGFR Alterations

Cancer type: Gastric Cancer, Gastroesophageal Junction Adenocarcinoma, Unspecified Solid Tumor

Variant class: FGFR3 activating mutation

Other identifiers: CTR20210275, LB1001-201

Population segments: FGFR, Fourth line or greater, Second line, Stage III, Stage IV, Third line

Phase: II

Therapy: infigratinib

Location: China

Disclaimer: The data presented here is from a curated knowledgebase of publicly available information, but may not be exhaustive. The data version is 2021.11(003).

FGFR3 K650E mutation (continued)

NCT03155620
NCI-COG Pediatric MATCH (Molecular Analysis for Therapy Choice) Screening Protocol

Cancer type: Unspecified Solid Tumor

Variant class: FGFR3 mutation

Other identifiers: 17-729, 2017-0625, 20170841, APEC1621SC, COGAPEC1621, COGAPEC1621SC, N 55017 SC, NCI-2017-01251, NCI-COG Pediatric MATCH, Pediatric MATCH

Population segments: ALK, Aggressive, BRAF, FGFR, HRAS, Indolent, KRAS, NRAS, Pediatric or Adolescent, Second line, Stage III, Stage IV

Phase: II

Therapy: erdafitinib

Locations: Puerto Rico, United States

US States: AK, AL, AR, AZ, CA, CO, CT, DC, DE, FL, GA, HI, IA, ID, IL, IN, KY, LA, MA, MD, ME, MI, MN, MO, MS, NC, NE, NH, NJ, NV, NY, OH, OK, OR, PA, RI, SC, SD, TN, TX, UT, VA, VT, WA, WI

Contact: Multiple contacts: See www.clinicaltrials.gov for complete list of contacts.

NCT03210714
NCI-COG Pediatric MATCH (Molecular Analysis for Therapy Choice) - Phase II Subprotocol of JNJ-42756493 (Erdafitinib) in Patients With Tumors Harboring FGFR 1/2/3/4 Alterations

Cancer type: Unspecified Solid Tumor

Variant class: FGFR3 mutation

Other identifiers: APEC1621B, NCI-2017-01159, NCI-COG Pediatric MATCH, Pediatric MATCH

Population segments: Aggressive, FGFR, Indolent, Pediatric or Adolescent, Second line, Stage III, Stage IV

Phase: II

Therapy: erdafitinib

Locations: Puerto Rico, United States

US States: AK, AL, AR, AZ, CA, CO, CT, DC, DE, FL, GA, IA, ID, IL, IN, KY, LA, MD, ME, MI, MN, MO, MS, NC, ND, NE, NJ, NY, OH, OK, OR, PA, SC, SD, TN, TX, UT, VA, VT, WA, WI

Contact: Multiple contacts: See www.clinicaltrials.gov for complete list of contacts.

No NCT ID - see other identifier(s)
A Multicenter Phase II Basket-type Clinical Trial to Evaluate Efficacy and Safety of TAS-120 in Patients with Advanced Solid Malignancies with FGFR Alterations in Circulating Tumor DNA

Cancer type: Unspecified Solid Tumor

Variant class: FGFR3 mutation

Other identifiers: EPOC 1805, JapicCTI-194624, JapicCTI-194796, TiFFANY

Population segments: Second line, Stage III, Stage IV

Phase: II

Therapy: futibatinib

Location: Japan

Disclaimer: The data presented here is from a curated knowledgebase of publicly available information, but may not be exhaustive. The data version is 2021.11(003).

FGFR3 K650E mutation (continued)

NCT04233567

A Phase II Study of Oral Infigratinib in Adult Patients With Advanced or Metastatic Solid Tumors With FGFR1-3 Gene Fusions or Other FGFR Genetic Alterations

Cancer type: Unspecified Solid Tumor

Variant class: FGFR activating mutation

Other identifiers: NCI-2019-06869, OSU-19041

Population segments: Second line, Stage III, Stage IV

Phase: II

Therapy: infigratinib

Location: United States

US State: OH

Contact: The Ohio State University Comprehensive Cancer Center [800-293-5066; OSUCCCClinicaltrials@osumc.edu]

NCT02693535

Targeted Agent and Profiling Utilization Registry (TAPUR) Study.

Cancer type: Unspecified Solid Tumor

Variant class: FGFR3 mutation

Other identifiers: 20170529, NCI-2017-00510, Pro00014171, TAPUR

Population segments: Aggressive, BRAF, Diffuse large B-cell lymphoma (DLBCL), Extranodal marginal zone B-cell lymphoma (MALT), FGFR, First line, Follicular lymphoma (FL), Fourth line or greater, Indolent, Lymphoblastic lymphoma (LBL), Mantle cell lymphoma (MCL), Other subtype, Second line, Small lymphocytic lymphoma (SLL), Stage III, Stage IV, Third line, Waldenstrom's macroglobulinemia (WM)

Phase: II

Therapy: sunitinib

Location: United States

US States: AL, AZ, CA, FL, GA, HI, IL, IN, ME, MI, NC, ND, NE, NH, OH, OR, PA, SD, TX, UT, VA, WA

Contact: Pam Mangat [pam.mangat@asco.org]

NCT04591431

The Rome Trial From Histology to Target: the Road to Personalize Target Therapy and Immunotherapy

Cancer type: Biliary Tract Carcinoma, Breast Cancer, Colorectal Cancer, Esophageal Cancer, Gastric Cancer, Gastroesophageal Junction Adenocarcinoma, Hepatocellular Carcinoma, Liver Cancer, Non-Small Cell Lung Cancer, Pancreatic Cancer, Unspecified Solid Tumor

Variant class: FGFR3 aberration

Other identifiers: EudraCT Number: 2018-002190-21, MAR-BAS-18-005, ROME

Population segments: Second line, Stage IV

Phase: II

Therapy: pemigatinib

Location: Italy

Disclaimer: The data presented here is from a curated knowledgebase of publicly available information, but may not be exhaustive. The data version is 2021.11(003).

FGFR3 K650E mutation (continued)

NCT02691793
Study to Evaluate the Safety and Efficacy of Sunitinib, in Subject With Refractory Solid Tumors

Cancer type: Unspecified Solid Tumor

Variant class: FGFR mutation

Other identifier: 2016-02-098

Population segments: (N/A), FGFR, Second line

Phase: II

Therapy: sunitinib

Location: Republic of Korea

NCT03297606
Canadian Profiling and Targeted Agent Utilization Trial (CAPTUR): A Phase II Basket Trial

Cancer type: Unspecified Solid Tumor

Variant class: FGFR3 aberration

Other identifiers: CA209-9DL, CAPTUR, ESR-17-12831, ML39800, PM1, WI233446

Population segments: Aggressive, Diffuse large B-cell lymphoma (DLBCL), Extranodal marginal zone B-cell lymphoma (MALT), First line, Follicular lymphoma (FL), Indolent, Lymphoblastic lymphoma (LBL), Mantle cell lymphoma (MCL), Other subtype, Second line, Stage III, Stage IV, Waldenstrom's macroglobulinemia (WM)

Phase: II

Therapy: sunitinib

Location: Canada

NCT03929965
Anlotinib in Advanced Solid Tumors With FGFR Alteration

Cancer type: Unspecified Solid Tumor

Variant class: FGFR mutation

Other identifier: ASTFA

Population segments: Second line, Stage III, Stage IV, Third line

Phase: I

Therapy: anlotinib hydrochloride

Location: China

NCT04565275
A Multi-center Open-label, Phase I/II Clinical Trial to Evaluate the Safety, Tolerability, Pharmacokinetics, and Pharmacodynamics of ICP-192 in Patients With Advanced Solid Tumors and FGFR Gene Alterations.

Cancer type: Bladder Urothelial Carcinoma, Cholangiocarcinoma, Unspecified Solid Tumor

Variant class: FGFR aberration

Other identifier: ICP-CL-00303

Population segments: FGFR, Second line, Stage III, Stage IV

Phase: I/II

Therapy: ICP-192

Location: United States

US States: AZ, CO, FL, MN

Contact: Olivia Yang [609-524-0684; olivia.yang@INNOCAREPHARMA.COM]

Disclaimer: The data presented here is from a curated knowledgebase of publicly available information, but may not be exhaustive. The data version is 2021.11(003).

FGFR3 K650E mutation (continued)

No NCT ID - see other identifier(s)
Phase I/II Study of TAS-117 In Combination With TAS-120 In Patients With Advanced Solid Tumors

Cancer type: Unspecified Solid Tumor

Variant class: FGFR aberration

Other identifier: JapicCTI-194864

Population segments: FGFR, First line, Squamous Cell, Stage III, Stage IV

Phase: I/II

Therapies: TAS-117, futibatinib

Location: Japan

No NCT ID - see other identifier(s)
A Phase Ib Study to Assess the Safety, Tolerability, and Efficacy of TAS-120 (Futibatinib) in Combination with MK-3475 (Pembrolizumab) in Patients with Solid Tumors.

Cancer type: Unspecified Solid Tumor

Variant class: FGFR aberration

Other identifier: JapicCTI-195063

Population segments: FGFR, Line of therapy N/A, Stage III, Stage IV

Phase: I

Therapies: futibatinib, pembrolizumab

Location: Japan

NCT04197986
Phase III, Multicenter, Double-Blind, Randomized, Placebo-Controlled Trial of Infigratinib for the Adjuvant Treatment of Subjects With Invasive Urothelial Carcinoma With Susceptible FGFR3 Genetic Alterations (PROOF 302).

Cancer type: Bladder Urothelial Carcinoma

Variant class: FGFR3 K650E mutation

Other identifiers: EudraCT Number: 2019-003248-63, NCI-2020-00610, PROOF 302, QBGJ398-302

Population segments: Adjuvant, FGFR, Stage II, Stage III

Exclusion criteria variant classes: FGFR3 A391E mutation (Bladder Small Cell Neuroendocrine Carcinoma), FGFR3 G370C mutation (Bladder Small Cell Neuroendocrine Carcinoma), FGFR3 K650E mutation (Bladder Small Cell Neuroendocrine Carcinoma), FGFR3 K650M mutation (Bladder Small Cell Neuroendocrine Carcinoma), FGFR3 K650Q mutation (Bladder Small Cell Neuroendocrine Carcinoma), FGFR3 K650T mutation (Bladder Small Cell Neuroendocrine Carcinoma), FGFR3 R248C mutation (Bladder Small Cell Neuroendocrine Carcinoma), FGFR3 S249C mutation (Bladder Small Cell Neuroendocrine Carcinoma), FGFR3 Y373C mutation (Bladder Small Cell Neuroendocrine Carcinoma), FGFR3 fusion (Bladder Small Cell Neuroendocrine Carcinoma)

Phase: III

Therapy: infigratinib

Locations: Belgium, Canada, France, Germany, Italy, Netherlands, Puerto Rico, Spain, United Kingdom, United States

US States: AL, AZ, CA, CO, DC, FL, GA, IL, IN, LA, MA, MD, MO, NC, NE, NJ, NY, OH, OK, SC, TN, TX, UT, VA, WA, WV

Contact: QED Therapeutics [877-280-5655; Proof302.ct@qedtx.com]

Disclaimer: The data presented here is from a curated knowledgebase of publicly available information, but may not be exhaustive. The data version is 2021.11(003).

FGFR3 K650E mutation (continued)

NCT04424966
A Phase 0 Study of Infigratinib in Recurrent High-Grade Glioma Participants Scheduled for Resection to Evaluate Central Nervous System (CNS) Penetration With PK Triggered Expansion Cohort

Cancer type: Anaplastic Astrocytoma, Anaplastic Oligoastrocytoma, Anaplastic Oligodendroglioma, Anaplastic Pleomorphic Xanthoastrocytoma, Diffuse Midline Glioma, Glioblastoma

Variant class: FGFR3 K650E mutation

Other identifiers: 2020-08, 20-500-092-34-38

Population segments: (N/A), Neoadjuvant

Phase: I

Therapy: infigratinib

Location: United States

US State: AZ

Contact: Phase 0 Navigator [602-406-8605; research@ivybraintumorcenter.org]

NCT04936295
A Phase II Study of the Efficacy and Tolerability of Fulvestrant Plus Anlotinib in HR(+)/HER2(-) Metastatic Breast Cancer Patients With FGFR Mutation

Cancer type: Breast Cancer

Variant class: FGFR3 activating mutation

Other inclusion criteria: ERBB2 negative, Hormone receptor positive

Other identifier: SYSU005

Population segments: Estrogen receptor positive, FGFR, First line, HER2 negative, HER2 positive, Progesterone receptor positive, Stage IV

Phase: II

Therapies: hormone therapy, anlotinib hydrochloride

Location: China

NCT04096417
A Phase II, Multicenter, Single-Arm Study of Pemigatinib in Patients With Metastatic or Unresectable Colorectal Cancer Harboring FGFR Alterations

Cancer type: Colorectal Cancer

Variant class: FGFR3 activating mutation

Other identifiers: ACCRU-GI-1701, NCI-2019-05877, VICCGI2028

Population segments: FGFR, Second line, Stage III, Stage IV

Phase: II

Therapy: pemigatinib

Location: United States

US States: AZ, GA, MN, NC, TN

Contact: Multiple contacts: See www.clinicaltrials.gov for complete list of contacts.

Disclaimer: The data presented here is from a curated knowledgebase of publicly available information, but may not be exhaustive. The data version is 2021.11(003).

FGFR3 K650E mutation (continued)

NCT04045613

An Open-label Multi-cohort Phase Ib/II Study of Derazantinib and Atezolizumab in Patients With Urothelial Cancer Expressing Activating Molecular FGFR Aberrations.

Cancer type: Bladder Urothelial Carcinoma

Variant class: FGFR3 activating mutation

Other inclusion criteria: CD274 expression

Other identifiers: CSET 3012, DZB-CS-201, EudraCT Number: 2019-000359-15, FIDES-02, NCI-2019-08162, RECF4112, SNCTP000004480

Population segments: FGFR, Fourth line or greater, Second line, Stage IV, Third line

Phase: I/II

Therapies: derazantinib, atezolizumab

Locations: Australia, Austria, Belgium, Canada, Czech Republic, France, Germany, Hungary, Italy, Poland, Republic of Korea, Spain, Switzerland, United Kingdom, United States

US States: GA, IL, KS, KY, NY, SC, TX, VA, WA

Contact: Dr. Manuel HÃ¤ckl [manuel.haeckl@basilea.com]

NCT04053322

An International Multicenter Phase II Trial of Durvalumab (MEDI4736) Plus OLAparib Plus Fulvestrant in Metastatic or Locally Advanced ER-positive, HER2-negative Breast Cancer Patients Selected Using Criteria That Predict Sensitivity to Olaparib

Cancer type: Breast Cancer

Variant class: FGFR3 mutation

Other inclusion criteria: ERBB2 negative, ER positive

Other identifiers: CADUSEIME08, DOLAF, EudraCT Number: 2018-003832-57, UC-0140/1812

Population segments: BRCA, Estrogen receptor positive, FGFR, HER2 negative, Progesterone receptor positive, Second line, Stage III, Stage IV

Phase: II

Therapies: durvalumab, olaparib, hormone therapy

Location: France

NCT03827850

A Phase II Trial to Evaluate Efficacy and Safety of Erdafitinib in Patients With Advanced Squamous Non Small Cell Lung Carcinoma (sqNSCLC) Harboring Fibroblast Growth Factor Receptor (FGFR) Genetic Alterations After Relapse of Standard Therapy.

Cancer type: Non-Small Cell Lung Cancer

Variant class: FGFR activating mutation

Other identifiers: EudraCT Number: 2018-000399-13, FIND, FIND Trial, Uni-Koeln-3254

Population segments: FGFR, Second line, Squamous Cell, Stage III, Stage IV

Exclusion criteria variant classes: ALK aberration (Non-Small Cell Lung Cancer), BRAF aberration (Non-Small Cell Lung Cancer), EGFR aberration (Non-Small Cell Lung Cancer), NTRK aberration (Non-Small Cell Lung Cancer), ROS1 aberration (Non-Small Cell Lung Cancer)

Phase: II

Therapy: erdafitinib

Location: Germany

Disclaimer: The data presented here is from a curated knowledgebase of publicly available information, but may not be exhaustive. The data version is 2021.11(003).

FGFR3 K650E mutation (continued)

NCT04601857
A Phase II Study Evaluating Futibatinib (TAS 120) Plus Pembrolizumab in the Treatment of Advanced or Metastatic Urothelial Carcinoma

Cancer type: Bladder Urothelial Carcinoma

Variant class: FGFR3 mutation

Other identifiers: EudraCT Number: 2020-000945-15, TAS-120-203

Population segments: FGFR, First line, Stage III, Stage IV

Phase: II

Therapies: futibatinib, pembrolizumab

Location: United States

US State: MI

Contact: Dr. Maciej Gil [609-250-7336; clinicaltrialinfo@taihooncology.com]

NCT04294277
Open-label, Single-arm, Phase II Study, Evaluating Safety and Efficacy of INCB054828 (Pemigatinib) as Adjuvant Therapy for Molecularly-selected, High-risk Patients With Urothelial Carcinoma Who Have Received Radical Surgery

Cancer type: Bladder Urothelial Carcinoma

Variant class: FGFR3 mutation

Other identifiers: EAU RF 2018-02, EAURF2018-02, EudraCT Number: 2017-004426-15, EudraCT Number: 2019-001833-14, PEGASUS

Population segments: Adjuvant, FGFR, Stage III, Stage IV

Phase: II

Therapy: pemigatinib

Location: Italy

NCT04077255
Anti-EGFR Antibody (GC-1118) in Combination With Weekly Paclitaxel as a Second-line Therapy for Gastric and Gastroesophageal Junction Adenocarcinomas With Amplifications

Cancer type: Gastric Cancer, Gastroesophageal Junction Adenocarcinoma

Variant class: FGFR3 mutation

Other identifiers: 31973, KCT0004314, NCC2019-0196

Population segments: EGFR, FGFR, Second line, Stage IV

Phase: II

Therapies: rogaratinib, chemotherapy

Location: Republic of Korea

No NCT ID - see other identifier(s)
Phase I Clinical Study of BPI-17509 In Patients With Advanced Solid Tumors

Cancer type: Bladder Cancer, Solid Tumor

Variant class: FGFR3 mutation

Other identifiers: BTP-26811, CTR20192140

Population segments: Second line, Stage III, Stage IV

Phase: I

Therapy: BPI-17509

Location: China

FGFR3 K650E mutation (continued)

NCT04172675

A Randomized Phase II Study of Erdafitinib Versus Investigator Choice of Intravesical Chemotherapy in Subjects Who Received Bacillus Calmette-Guerin (BCG) and Recurred With High Risk Non-Muscle-Invasive Bladder Cancer (NMIBC) and FGFR Mutations or Fusions

Cancer type: Bladder Urothelial Carcinoma

Variant class: FGFR mutation

Other identifiers: 42756493BLC2003, 42756493BLC2003, CR108699, CTR20201841, CTRI/2021/08/036009, EudraCT Number: 2019-002449-39, JapicCTI-205145, NCI-2020-00674, RG1121102

Population segments: FGFR, Maintenance/Consolidation, Second line, Stage 0

Phase: II

Therapy: erdafitinib

Locations: Argentina, Australia, Belgium, Brazil, China, Czech Republic, France, Germany, Italy, Japan, Poland, Republic of Korea, Spain, Taiwan, United Kingdom, United States

US States: AZ, CA, CO, FL, GA, IL, KS, NC, NY, OH, PA, TN, TX, WA, WI

Contact: Study Contact [844-434-4210; JNJ.CT@sylogent.com]

NCT02150967

A Phase II Multicenter, Single Arm Study of Oral BGJ398 in Adult Patients with Advanced or Metastatic Cholangiocarcinoma with FGFR2 Gene Fusions or Other FGFR Genetic Alterations who Failed or are Intolerant to Platinum-based Chemotherapy.

Cancer type: Cholangiocarcinoma

Variant class: FGFR activating aberration

Other identifiers: 14-183, 14-2200, 14-328, 15452, 2014-0372, 2014-081, CBGJ398X2204, EudraCT Number: 2013-005085-19, NCI-2014-01310, REec-2014-1022, Trial 3GB-14-1

Population segments: FGFR, Fourth line or greater, Second line, Stage III, Stage IV, Third line

Phase: II

Therapy: infigratinib

Locations: Belgium, Germany, Singapore, Spain, Thailand, United Kingdom, United States

US States: CA, MA, MI, NY, OH, TX

Contact: QED Therapeutics [877-280-5655; clinicaltrials@QEDTx.com]

NCT04595747

Phase II Study of Rogaratinib (BAY 1163877) in the Treatment of Patients With Sarcoma Harboring Alterations in Fibroblast Growth Factor Receptor (FGFR) 1-4 and SDH-Deficient Gastrointestinal Stromal Tumor (GIST)

Cancer type: Soft Tissue Sarcoma

Variant class: FGFR3 aberration

Other identifiers: 10411, NCI-2020-08011

Population segments: FGFR, Second line, Stage III, Stage IV

Phase: II

Therapy: rogaratinib

Location: United States

US States: CA, MA, MD, NY

Contact: Multiple contacts: See www.clinicaltrials.gov for complete list of contacts.

Disclaimer: The data presented here is from a curated knowledgebase of publicly available information, but may not be exhaustive. The data version is 2021.11(003).

FGFR3 K650E mutation (continued)

NCT04004975
Phase II Clinical Trials on Anlotinib for the Treatment of Recurrent Glioblastoma

Cancer type: Glioblastoma

Variant class: FGFR mutation

Other identifier: ShandongCHI006

Population segments: (N/A), FGFR, Second line

Phase: I/II

Therapy: anlotinib hydrochloride

Location: China

NCT04149691
A Phase I, Open-label, Multicentre, Dose Escalation Study to Assess Safety, Tolerability and Pharmacokinetics of Oral CPL304110, in Adult Subjects With Advanced Solid Malignancies

Cancer type: Bladder Cancer, Gastric Cancer, Non-Small Cell Lung Cancer

Variant class: FGFR3 aberration

Other identifier: 01FGFR2018

Population segments: FGFR, Line of therapy N/A, Squamous Cell, Stage III, Stage IV

Phase: I

Therapy: CPL-304-110

Location: Poland

NCT03473743
A Phase Ib-II Study to Evaluate Safety, Efficacy, Pharmacokinetics, and Pharmacodynamics of Various Regimens of Erdafitinib in Subjects With Metastatic or Locally Advanced Urothelial Cancer

Cancer type: Bladder Urothelial Carcinoma

Variant class: FGFR aberration

Other identifiers: 17165, 2018-0142, 42756493BLC2002, CR108445, EudraCT Number: 2017-001980-19, NCI-2018-00997, NORSE, USO 17165, USOR 17165

Population segments: FGFR, First line, Fourth line or greater, Second line, Stage IV, Third line

Phase: I/II

Therapies: erdafitinib, cetrelimab

Locations: Belarus, Belgium, Brazil, France, Italy, Poland, Republic of Korea, Russian Federation, Spain, Taiwan, Turkey, United Kingdom, United States

US States: CO, MD, NC, NY, PA, TX, VA

Contact: Study Contact [844-434-4210; JNJ.CT@sylogent.com]

NCT02393248
A Phase I/II, Open-Label, Dose-Escalation, Safety and Tolerability Study of INCB054828 in Subjects With Advanced Malignancies - (FIGHT-101)

Cancer type: Bladder Urothelial Carcinoma, Breast Cancer, Cholangiocarcinoma, Endometrial Carcinoma, Gastric Cancer, Non-Small Cell Lung Cancer, Solid Tumor

Variant class: FGFR aberration

Other identifiers: 00061567, 2014-1099, 201503012, EudraCT Number: 2016-002831-14, FIGHT-101, INCB 54828-101, INCB54828-101, MC# 15-03, NCI-2015-00611, OSU-15241

Population segments: FGFR, First line, Fourth line or greater, Second line, Squamous Cell, Stage III, Stage IV, Third line

Phase: I/II

Therapies: pemigatinib, pembrolizumab, trastuzumab, chemotherapy, retifanlimab

Location: United States

US States: FL, NJ, TX

Contact: Incyte Call Center [855-463-3463]

Disclaimer: The data presented here is from a curated knowledgebase of publicly available information, but may not be exhaustive. The data version is 2021.11(003).

FGFR3 K650E mutation (continued)

NCT04572295
An Open-label Phase Ib Study of E7090 Monotherapy and in Combination With Other Anticancer Agents in Subjects With ER+, HER2- Recurrent/Metastatic Breast Cancer

Cancer type: Breast Cancer

Variant class: FGFR aberration

Other inclusion criteria: ERBB2 negative, ER positive

Other identifiers: E7090-J081-102, JapicCTI-205429

Population segments: Estrogen receptor positive, HER2 negative, Second line, Stage IV

Phase: I

Therapies: E-7090, hormone therapy

Location: Japan

NCT04504331
A Phase IB Study of Infigratinib in Combination With Tamoxifen in Hormone Receptor Positive, HER2 Negative, FGFR Altered Advanced Breast Cancer

Cancer type: Breast Cancer

Variant class: FGFR aberration

Other inclusion criteria: ERBB2 negative, Hormone receptor positive

Other identifiers: BRS0113, K08CA252457

Population segments: Estrogen receptor positive, HER2 negative, Progesterone receptor positive, Second line, Stage III, Stage IV, Triple receptor negative

Phase: I

Therapies: infigratinib, hormone therapy

Location: United States

US State: CA

Contact: Allison K. Zhang [650-736-5790; axkong@stanford.edu]

FGFR3 K650M mutation

NCT05019794
A Phase IIa of Infigratinib in Subjects With Locally Advanced or Metastatic Gastric Cancer or Gastroesophageal Junction Adenocarcinoma With FGFR2 Amplification or Other Advanced Solid Tumors With Other FGFR Alterations

Cancer type: Gastric Cancer, Gastroesophageal Junction Adenocarcinoma, Unspecified Solid Tumor

Variant class: FGFR3 activating mutation

Other identifiers: CTR20210275, LB1001-201

Population segments: FGFR, Fourth line or greater, Second line, Stage III, Stage IV, Third line

Phase: II

Therapy: infigratinib

Location: China

Disclaimer: The data presented here is from a curated knowledgebase of publicly available information, but may not be exhaustive. The data version is 2021.11(003).

FGFR3 K650M mutation (continued)

NCT03155620
NCI-COG Pediatric MATCH (Molecular Analysis for Therapy Choice) Screening Protocol

Cancer type: Unspecified Solid Tumor

Variant class: FGFR3 mutation

Other identifiers: 17-729, 2017-0625, 20170841, APEC1621SC, COGAPEC1621, COGAPEC1621SC, N 55017 SC, NCI-2017-01251, NCI-COG Pediatric MATCH, Pediatric MATCH

Population segments: ALK, Aggressive, BRAF, FGFR, HRAS, Indolent, KRAS, NRAS, Pediatric or Adolescent, Second line, Stage III, Stage IV

Phase: II

Therapy: erdafitinib

Locations: Puerto Rico, United States

US States: AK, AL, AR, AZ, CA, CO, CT, DC, DE, FL, GA, HI, IA, ID, IL, IN, KY, LA, MA, MD, ME, MI, MN, MO, MS, NC, NE, NH, NJ, NV, NY, OH, OK, OR, PA, RI, SC, SD, TN, TX, UT, VA, VT, WA, WI

Contact: Multiple contacts: See www.clinicaltrials.gov for complete list of contacts.

NCT03210714
NCI-COG Pediatric MATCH (Molecular Analysis for Therapy Choice) - Phase II Subprotocol of JNJ-42756493 (Erdafitinib) in Patients With Tumors Harboring FGFR 1/2/3/4 Alterations

Cancer type: Unspecified Solid Tumor

Variant class: FGFR3 mutation

Other identifiers: APEC1621B, NCI-2017-01159, NCI-COG Pediatric MATCH, Pediatric MATCH

Population segments: Aggressive, FGFR, Indolent, Pediatric or Adolescent, Second line, Stage III, Stage IV

Phase: II

Therapy: erdafitinib

Locations: Puerto Rico, United States

US States: AK, AL, AR, AZ, CA, CO, CT, DC, DE, FL, GA, IA, ID, IL, IN, KY, LA, MD, ME, MI, MN, MO, MS, NC, ND, NE, NJ, NY, OH, OK, OR, PA, SC, SD, TN, TX, UT, VA, VT, WA, WI

Contact: Multiple contacts: See www.clinicaltrials.gov for complete list of contacts.

No NCT ID - see other identifier(s)
A Multicenter Phase II Basket-type Clinical Trial to Evaluate Efficacy and Safety of TAS-120 in Patients with Advanced Solid Malignancies with FGFR Alterations in Circulating Tumor DNA

Cancer type: Unspecified Solid Tumor

Variant class: FGFR3 mutation

Other identifiers: EPOC 1805, JapicCTI-194624, JapicCTI-194796, TiFFANY

Population segments: Second line, Stage III, Stage IV

Phase: II

Therapy: futibatinib

Location: Japan

FGFR3 K650M mutation (continued)

NCT04233567

A Phase II Study of Oral Infigratinib in Adult Patients With Advanced or Metastatic Solid Tumors With FGFR1-3 Gene Fusions or Other FGFR Genetic Alterations

Cancer type: Unspecified Solid Tumor

Variant class: FGFR activating mutation

Other identifiers: NCI-2019-06869, OSU-19041

Population segments: Second line, Stage III, Stage IV

Phase: II

Therapy: infigratinib

Location: United States

US State: OH

Contact: The Ohio State University Comprehensive Cancer Center [800-293-5066; OSUCCCClinicaltrials@osumc.edu]

NCT02693535

Targeted Agent and Profiling Utilization Registry (TAPUR) Study.

Cancer type: Unspecified Solid Tumor

Variant class: FGFR3 mutation

Other identifiers: 20170529, NCI-2017-00510, Pro00014171, TAPUR

Population segments: Aggressive, BRAF, Diffuse large B-cell lymphoma (DLBCL), Extranodal marginal zone B-cell lymphoma (MALT), FGFR, First line, Follicular lymphoma (FL), Fourth line or greater, Indolent, Lymphoblastic lymphoma (LBL), Mantle cell lymphoma (MCL), Other subtype, Second line, Small lymphocytic lymphoma (SLL), Stage III, Stage IV, Third line, Waldenstrom's macroglobulinemia (WM)

Phase: II

Therapy: sunitinib

Location: United States

US States: AL, AZ, CA, FL, GA, HI, IL, IN, ME, MI, NC, ND, NE, NH, OH, OR, PA, SD, TX, UT, VA, WA

Contact: Pam Mangat [pam.mangat@asco.org]

NCT04591431

The Rome Trial From Histology to Target: the Road to Personalize Target Therapy and Immunotherapy

Cancer type: Biliary Tract Carcinoma, Breast Cancer, Colorectal Cancer, Esophageal Cancer, Gastric Cancer, Gastroesophageal Junction Adenocarcinoma, Hepatocellular Carcinoma, Liver Cancer, Non-Small Cell Lung Cancer, Pancreatic Cancer, Unspecified Solid Tumor

Variant class: FGFR3 aberration

Other identifiers: EudraCT Number: 2018-002190-21, MAR-BAS-18-005, ROME

Population segments: Second line, Stage IV

Phase: II

Therapy: pemigatinib

Location: Italy

Disclaimer: The data presented here is from a curated knowledgebase of publicly available information, but may not be exhaustive. The data version is 2021.11(003).

FGFR3 K650M mutation (continued)

NCT02691793
Study to Evaluate the Safety and Efficacy of Sunitinib, in Subject With Refractory Solid Tumors

Cancer type: Unspecified Solid Tumor

Variant class: FGFR mutation

Other identifier: 2016-02-098

Population segments: (N/A), FGFR, Second line

Phase: II

Therapy: sunitinib

Location: Republic of Korea

NCT03297606
Canadian Profiling and Targeted Agent Utilization Trial (CAPTUR): A Phase II Basket Trial

Cancer type: Unspecified Solid Tumor

Variant class: FGFR3 aberration

Other identifiers: CA209-9DL, CAPTUR, ESR-17-12831, ML39800, PM1, WI233446

Population segments: Aggressive, Diffuse large B-cell lymphoma (DLBCL), Extranodal marginal zone B-cell lymphoma (MALT), First line, Follicular lymphoma (FL), Indolent, Lymphoblastic lymphoma (LBL), Mantle cell lymphoma (MCL), Other subtype, Second line, Stage III, Stage IV, Waldenstrom's macroglobulinemia (WM)

Phase: II

Therapy: sunitinib

Location: Canada

NCT03929965
Anlotinib in Advanced Solid Tumors With FGFR Alteration

Cancer type: Unspecified Solid Tumor

Variant class: FGFR mutation

Other identifier: ASTFA

Population segments: Second line, Stage III, Stage IV, Third line

Phase: I

Therapy: anlotinib hydrochloride

Location: China

NCT04565275
A Multi-center Open-label, Phase I/II Clinical Trial to Evaluate the Safety, Tolerability, Pharmacokinetics, and Pharmacodynamics of ICP-192 in Patients With Advanced Solid Tumors and FGFR Gene Alterations.

Cancer type: Bladder Urothelial Carcinoma, Cholangiocarcinoma, Unspecified Solid Tumor

Variant class: FGFR aberration

Other identifier: ICP-CL-00303

Population segments: FGFR, Second line, Stage III, Stage IV

Phase: I/II

Therapy: ICP-192

Location: United States

US States: AZ, CO, FL, MN

Contact: Olivia Yang [609-524-0684; olivia.yang@INNOCAREPHARMA.COM]

FGFR3 K650M mutation (continued)

No NCT ID - see other identifier(s)
Phase I/II Study of TAS-117 In Combination With TAS-120 In Patients With Advanced Solid Tumors

Cancer type: Unspecified Solid Tumor

Variant class: FGFR aberration

Other identifier: JapicCTI-194864

Population segments: FGFR, First line, Squamous Cell, Stage III, Stage IV

Phase: I/II

Therapies: TAS-117, futibatinib

Location: Japan

No NCT ID - see other identifier(s)
A Phase Ib Study to Assess the Safety, Tolerability, and Efficacy of TAS-120 (Futibatinib) in Combination with MK-3475 (Pembrolizumab) in Patients with Solid Tumors.

Cancer type: Unspecified Solid Tumor

Variant class: FGFR aberration

Other identifier: JapicCTI-195063

Population segments: FGFR, Line of therapy N/A, Stage III, Stage IV

Phase: I

Therapies: futibatinib, pembrolizumab

Location: Japan

NCT04197986
Phase III, Multicenter, Double-Blind, Randomized, Placebo-Controlled Trial of Infigratinib for the Adjuvant Treatment of Subjects With Invasive Urothelial Carcinoma With Susceptible FGFR3 Genetic Alterations (PROOF 302).

Cancer type: Bladder Urothelial Carcinoma

Variant class: FGFR3 K650M mutation

Other identifiers: EudraCT Number: 2019-003248-63, NCI-2020-00610, PROOF 302, QBGJ398-302

Population segments: Adjuvant, FGFR, Stage II, Stage III

Exclusion criteria variant classes: FGFR3 A391E mutation (Bladder Small Cell Neuroendocrine Carcinoma), FGFR3 G370C mutation (Bladder Small Cell Neuroendocrine Carcinoma), FGFR3 K650E mutation (Bladder Small Cell Neuroendocrine Carcinoma), FGFR3 K650M mutation (Bladder Small Cell Neuroendocrine Carcinoma), FGFR3 K650Q mutation (Bladder Small Cell Neuroendocrine Carcinoma), FGFR3 K650T mutation (Bladder Small Cell Neuroendocrine Carcinoma), FGFR3 R248C mutation (Bladder Small Cell Neuroendocrine Carcinoma), FGFR3 S249C mutation (Bladder Small Cell Neuroendocrine Carcinoma), FGFR3 Y373C mutation (Bladder Small Cell Neuroendocrine Carcinoma), FGFR3 fusion (Bladder Small Cell Neuroendocrine Carcinoma)

Phase: III

Therapy: infigratinib

Locations: Belgium, Canada, France, Germany, Italy, Netherlands, Puerto Rico, Spain, United Kingdom, United States

US States: AL, AZ, CA, CO, DC, FL, GA, IL, IN, LA, MA, MD, MO, NC, NE, NJ, NY, OH, OK, SC, TN, TX, UT, VA, WA, WV

Contact: QED Therapeutics [877-280-5655; Proof302.ct@qedtx.com]

Disclaimer: The data presented here is from a curated knowledgebase of publicly available information, but may not be exhaustive. The data version is 2021.11(003).

FGFR3 K650M mutation (continued)

NCT04936295

A Phase II Study of the Efficacy and Tolerability of Fulvestrant Plus Anlotinib in HR(+)/HER2(-) Metastatic Breast Cancer Patients With FGFR Mutation

Cancer type: Breast Cancer

Variant class: FGFR3 activating mutation

Other inclusion criteria: ERBB2 negative, Hormone receptor positive

Other identifier: SYSU005

Population segments: Estrogen receptor positive, FGFR, First line, HER2 negative, HER2 positive, Progesterone receptor positive, Stage IV

Phase: II

Therapies: hormone therapy, anlotinib hydrochloride

Location: China

NCT04096417

A Phase II, Multicenter, Single-Arm Study of Pemigatinib in Patients With Metastatic or Unresectable Colorectal Cancer Harboring FGFR Alterations

Cancer type: Colorectal Cancer

Variant class: FGFR3 activating mutation

Other identifiers: ACCRU-GI-1701, NCI-2019-05877, VICCGI2028

Population segments: FGFR, Second line, Stage III, Stage IV

Phase: II

Therapy: pemigatinib

Location: United States

US States: AZ, GA, MN, NC, TN

Contact: Multiple contacts: See www.clinicaltrials.gov for complete list of contacts.

NCT04045613

An Open-label Multi-cohort Phase Ib/II Study of Derazantinib and Atezolizumab in Patients With Urothelial Cancer Expressing Activating Molecular FGFR Aberrations.

Cancer type: Bladder Urothelial Carcinoma

Variant class: FGFR3 activating mutation

Other inclusion criteria: CD274 expression

Other identifiers: CSET 3012, DZB-CS-201, EudraCT Number: 2019-000359-15, FIDES-02, NCI-2019-08162, RECF4112, SNCTP000004480

Population segments: FGFR, Fourth line or greater, Second line, Stage IV, Third line

Phase: I/II

Therapies: derazantinib, atezolizumab

Locations: Australia, Austria, Belgium, Canada, Czech Republic, France, Germany, Hungary, Italy, Poland, Republic of Korea, Spain, Switzerland, United Kingdom, United States

US States: GA, IL, KS, KY, NY, SC, TX, VA, WA

Contact: Dr. Manuel HÃ¤ckl [manuel.haeckl@basilea.com]

Disclaimer: The data presented here is from a curated knowledgebase of publicly available information, but may not be exhaustive. The data version is 2021.11(003).

FGFR3 K650M mutation (continued)

NCT04053322

An International Multicenter Phase II Trial of Durvalumab (MEDI4736) Plus OLAparib Plus Fulvestrant in Metastatic or Locally Advanced ER-positive, HER2-negative Breast Cancer Patients Selected Using Criteria That Predict Sensitivity to Olaparib

Cancer type: Breast Cancer

Variant class: FGFR3 mutation

Other inclusion criteria: ERBB2 negative, ER positive

Other identifiers: CADUSEIME08, DOLAF, EudraCT Number: 2018-003832-57, UC-0140/1812

Population segments: BRCA, Estrogen receptor positive, FGFR, HER2 negative, Progesterone receptor positive, Second line, Stage III, Stage IV

Phase: II

Therapies: durvalumab, olaparib, hormone therapy

Location: France

NCT03827850

A Phase II Trial to Evaluate Efficacy and Safety of Erdafitinib in Patients With Advanced Squamous Non Small Cell Lung Carcinoma (sqNSCLC) Harboring Fibroblast Growth Factor Receptor (FGFR) Genetic Alterations After Relapse of Standard Therapy.

Cancer type: Non-Small Cell Lung Cancer

Variant class: FGFR activating mutation

Other identifiers: EudraCT Number: 2018-000399-13, FIND, FIND Trial, Uni-Koeln-3254

Population segments: FGFR, Second line, Squamous Cell, Stage III, Stage IV

Exclusion criteria variant classes: ALK aberration (Non-Small Cell Lung Cancer), BRAF aberration (Non-Small Cell Lung Cancer), EGFR aberration (Non-Small Cell Lung Cancer), NTRK aberration (Non-Small Cell Lung Cancer), ROS1 aberration (Non-Small Cell Lung Cancer)

Phase: II

Therapy: erdafitinib

Location: Germany

NCT04601857

A Phase II Study Evaluating Futibatinib (TAS 120) Plus Pembrolizumab in the Treatment of Advanced or Metastatic Urothelial Carcinoma

Cancer type: Bladder Urothelial Carcinoma

Variant class: FGFR3 mutation

Other identifiers: EudraCT Number: 2020-000945-15, TAS-120-203

Population segments: FGFR, First line, Stage III, Stage IV

Phase: II

Therapies: futibatinib, pembrolizumab

Location: United States

US State: MI

Contact: Dr. Maciej Gil [609-250-7336; clinicaltrialinfo@taihooncology.com]

Disclaimer: The data presented here is from a curated knowledgebase of publicly available information, but may not be exhaustive. The data version is 2021.11(003).

FGFR3 K650M mutation (continued)

NCT04294277
Open-label, Single-arm, Phase II Study, Evaluating Safety and Efficacy of INCB054828 (Pemigatinib) as Adjuvant Therapy for Molecularly-selected, High-risk Patients With Urothelial Carcinoma Who Have Received Radical Surgery

Cancer type: Bladder Urothelial Carcinoma

Variant class: FGFR3 mutation

Other identifiers: EAU RF 2018-02, EAURF2018-02, EudraCT Number: 2017-004426-15, EudraCT Number: 2019-001833-14, PEGASUS

Population segments: Adjuvant, FGFR, Stage III, Stage IV

Phase: II

Therapy: pemigatinib

Location: Italy

NCT04077255
Anti-EGFR Antibody (GC-1118) in Combination With Weekly Paclitaxel as a Second-line Therapy for Gastric and Gastroesophageal Junction Adenocarcinomas With Amplifications

Cancer type: Gastric Cancer, Gastroesophageal Junction Adenocarcinoma

Variant class: FGFR3 mutation

Other identifiers: 31973, KCT0004314, NCC2019-0196

Population segments: EGFR, FGFR, Second line, Stage IV

Phase: II

Therapies: rogaratinib, chemotherapy

Location: Republic of Korea

No NCT ID - see other identifier(s)
Phase I Clinical Study of BPI-17509 In Patients With Advanced Solid Tumors

Cancer type: Bladder Cancer, Solid Tumor

Variant class: FGFR3 mutation

Other identifiers: BTP-26811, CTR20192140

Population segments: Second line, Stage III, Stage IV

Phase: I

Therapy: BPI-17509

Location: China

NCT04172675
A Randomized Phase II Study of Erdafitinib Versus Investigator Choice of Intravesical Chemotherapy in Subjects Who Received Bacillus Calmette-Guerin (BCG) and Recurred With High Risk Non-Muscle-Invasive Bladder Cancer (NMIBC) and FGFR Mutations or Fusions

Cancer type: Bladder Urothelial Carcinoma

Variant class: FGFR mutation

Other identifiers: 42756493BLC2003, 42756493BLC2003, CR108699, CTR20201841, CTRI/2021/08/036009, EudraCT Number: 2019-002449-39, JapicCTI-205145, NCI-2020-00674, RG1121102

Population segments: FGFR, Maintenance/Consolidation, Second line, Stage 0

Phase: II

Therapy: erdafitinib

Locations: Argentina, Australia, Belgium, Brazil, China, Czech Republic, France, Germany, Italy, Japan, Poland, Republic of Korea, Spain, Taiwan, United Kingdom, United States

US States: AZ, CA, CO, FL, GA, IL, KS, NC, NY, OH, PA, TN, TX, WA, WI

Contact: Study Contact [844-434-4210; JNJ.CT@sylogent.com]

FGFR3 K650M mutation (continued)

NCT02150967
A Phase II Multicenter, Single Arm Study of Oral BGJ398 in Adult Patients with Advanced or Metastatic Cholangiocarcinoma with FGFR2 Gene Fusions or Other FGFR Genetic Alterations who Failed or are Intolerant to Platinum-based Chemotherapy.

Cancer type: Cholangiocarcinoma

Variant class: FGFR activating aberration

Other identifiers: 14-183, 14-2200, 14-328, 15452, 2014-0372, 2014-081, CBGJ398X2204, EudraCT Number: 2013-005085-19, NCI-2014-01310, REec-2014-1022, Trial 3GB-14-1

Population segments: FGFR, Fourth line or greater, Second line, Stage III, Stage IV, Third line

Phase: II

Therapy: infigratinib

Locations: Belgium, Germany, Singapore, Spain, Thailand, United Kingdom, United States

US States: CA, MA, MI, NY, OH, TX

Contact: QED Therapeutics [877-280-5655; clinicaltrials@QEDTx.com]

NCT04595747
Phase II Study of Rogaratinib (BAY 1163877) in the Treatment of Patients With Sarcoma Harboring Alterations in Fibroblast Growth Factor Receptor (FGFR) 1-4 and SDH-Deficient Gastrointestinal Stromal Tumor (GIST)

Cancer type: Soft Tissue Sarcoma

Variant class: FGFR3 aberration

Other identifiers: 10411, NCI-2020-08011

Population segments: FGFR, Second line, Stage III, Stage IV

Phase: II

Therapy: rogaratinib

Location: United States

US States: CA, MA, MD, NY

Contact: Multiple contacts: See www.clinicaltrials.gov for complete list of contacts.

NCT04004975
Phase II Clinical Trials on Anlotinib for the Treatment of Recurrent Glioblastoma

Cancer type: Glioblastoma

Variant class: FGFR mutation

Other identifier: ShandongCHI006

Population segments: (N/A), FGFR, Second line

Phase: I/II

Therapy: anlotinib hydrochloride

Location: China

NCT04149691
A Phase I, Open-label, Multicentre, Dose Escalation Study to Assess Safety, Tolerability and Pharmacokinetics of Oral CPL304110, in Adult Subjects With Advanced Solid Malignancies

Cancer type: Bladder Cancer, Gastric Cancer, Non-Small Cell Lung Cancer

Variant class: FGFR3 aberration

Other identifier: 01FGFR2018

Population segments: FGFR, Line of therapy N/A, Squamous Cell, Stage III, Stage IV

Phase: I

Therapy: CPL-304-110

Location: Poland

FGFR3 K650M mutation (continued)

NCT03473743
A Phase Ib-II Study to Evaluate Safety, Efficacy, Pharmacokinetics, and Pharmacodynamics of Various Regimens of Erdafitinib in Subjects With Metastatic or Locally Advanced Urothelial Cancer

Cancer type: Bladder Urothelial Carcinoma

Variant class: FGFR aberration

Other identifiers: 17165, 2018-0142, 42756493BLC2002, CR108445, EudraCT Number: 2017-001980-19, NCI-2018-00997, NORSE, USO 17165, USOR 17165

Population segments: FGFR, First line, Fourth line or greater, Second line, Stage IV, Third line

Phase: I/II

Therapies: erdafitinib, cetrelimab

Locations: Belarus, Belgium, Brazil, France, Italy, Poland, Republic of Korea, Russian Federation, Spain, Taiwan, Turkey, United Kingdom, United States

US States: CO, MD, NC, NY, PA, TX, VA

Contact: Study Contact [844-434-4210; JNJ.CT@sylogent.com]

NCT02393248
A Phase I/II, Open-Label, Dose-Escalation, Safety and Tolerability Study of INCB054828 in Subjects With Advanced Malignancies - (FIGHT-101)

Cancer type: Bladder Urothelial Carcinoma, Breast Cancer, Cholangiocarcinoma, Endometrial Carcinoma, Gastric Cancer, Non-Small Cell Lung Cancer, Solid Tumor

Variant class: FGFR aberration

Other identifiers: 00061567, 2014-1099, 201503012, EudraCT Number: 2016-002831-14, FIGHT-101, INCB 54828-101, INCB54828-101, MC# 15-03, NCI-2015-00611, OSU-15241

Population segments: FGFR, First line, Fourth line or greater, Second line, Squamous Cell, Stage III, Stage IV, Third line

Phase: I/II

Therapies: pemigatinib, pembrolizumab, trastuzumab, chemotherapy, retifanlimab

Location: United States

US States: FL, NJ, TX

Contact: Incyte Call Center [855-463-3463]

NCT04572295
An Open-label Phase Ib Study of E7090 Monotherapy and in Combination With Other Anticancer Agents in Subjects With ER+, HER2- Recurrent/Metastatic Breast Cancer

Cancer type: Breast Cancer

Variant class: FGFR aberration

Other inclusion criteria: ERBB2 negative, ER positive

Other identifiers: E7090-J081-102, JapicCTI-205429

Population segments: Estrogen receptor positive, HER2 negative, Second line, Stage IV

Phase: I

Therapies: E-7090, hormone therapy

Location: Japan

FGFR3 K650M mutation (continued)

NCT04504331
A Phase IB Study of Infigratinib in Combination With Tamoxifen in Hormone Receptor Positive, HER2 Negative, FGFR Altered Advanced Breast Cancer

Cancer type: Breast Cancer

Variant class: FGFR aberration

Other inclusion criteria: ERBB2 negative, Hormone receptor positive

Other identifiers: BRS0113, K08CA252457

Population segments: Estrogen receptor positive, HER2 negative, Progesterone receptor positive, Second line, Stage III, Stage IV, Triple receptor negative

Phase: I

Therapies: infigratinib, hormone therapy

Location: United States

US State: CA

Contact: Allison K. Zhang [650-736-5790; axkong@stanford.edu]

FGFR3 K650Q mutation

NCT05019794
A Phase IIa of Infigratinib in Subjects With Locally Advanced or Metastatic Gastric Cancer or Gastroesophageal Junction Adenocarcinoma With FGFR2 Amplification or Other Advanced Solid Tumors With Other FGFR Alterations

Cancer type: Gastric Cancer, Gastroesophageal Junction Adenocarcinoma, Unspecified Solid Tumor

Variant class: FGFR3 activating mutation

Other identifiers: CTR20210275, LB1001-201

Population segments: FGFR, Fourth line or greater, Second line, Stage III, Stage IV, Third line

Phase: II

Therapy: infigratinib

Location: China

NCT03155620
NCI-COG Pediatric MATCH (Molecular Analysis for Therapy Choice) Screening Protocol

Cancer type: Unspecified Solid Tumor

Variant class: FGFR3 mutation

Other identifiers: 17-729, 2017-0625, 20170841, APEC1621SC, COGAPEC1621, COGAPEC1621SC, N 55017 SC, NCI-2017-01251, NCI-COG Pediatric MATCH, Pediatric MATCH

Population segments: ALK, Aggressive, BRAF, FGFR, HRAS, Indolent, KRAS, NRAS, Pediatric or Adolescent, Second line, Stage III, Stage IV

Phase: II

Therapy: erdafitinib

Locations: Puerto Rico, United States

US States: AK, AL, AR, AZ, CA, CO, CT, DC, DE, FL, GA, HI, IA, ID, IL, IN, KY, LA, MA, MD, ME, MI, MN, MO, MS, NC, NE, NH, NJ, NV, NY, OH, OK, OR, PA, RI, SC, SD, TN, TX, UT, VA, VT, WA, WI

Contact: Multiple contacts: See www.clinicaltrials.gov for complete list of contacts.

Disclaimer: The data presented here is from a curated knowledgebase of publicly available information, but may not be exhaustive. The data version is 2021.11(003).

FGFR3 K650Q mutation (continued)

NCT03210714
NCI-COG Pediatric MATCH (Molecular Analysis for Therapy Choice) - Phase II Subprotocol of JNJ-42756493 (Erdafitinib) in Patients With Tumors Harboring FGFR 1/2/3/4 Alterations

Cancer type: Unspecified Solid Tumor

Variant class: FGFR3 mutation

Other identifiers: APEC1621B, NCI-2017-01159, NCI-COG Pediatric MATCH, Pediatric MATCH

Population segments: Aggressive, FGFR, Indolent, Pediatric or Adolescent, Second line, Stage III, Stage IV

Phase: II

Therapy: erdafitinib

Locations: Puerto Rico, United States

US States: AK, AL, AR, AZ, CA, CO, CT, DC, DE, FL, GA, IA, ID, IL, IN, KY, LA, MD, ME, MI, MN, MO, MS, NC, ND, NE, NJ, NY, OH, OK, OR, PA, SC, SD, TN, TX, UT, VA, VT, WA, WI

Contact: Multiple contacts: See www.clinicaltrials.gov for complete list of contacts.

No NCT ID - see other identifier(s)
A Multicenter Phase II Basket-type Clinical Trial to Evaluate Efficacy and Safety of TAS-120 in Patients with Advanced Solid Malignancies with FGFR Alterations in Circulating Tumor DNA

Cancer type: Unspecified Solid Tumor

Variant class: FGFR3 mutation

Other identifiers: EPOC 1805, JapicCTI-194624, JapicCTI-194796, TiFFANY

Population segments: Second line, Stage III, Stage IV

Phase: II

Therapy: futibatinib

Location: Japan

NCT04233567
A Phase II Study of Oral Infigratinib in Adult Patients With Advanced or Metastatic Solid Tumors With FGFR1-3 Gene Fusions or Other FGFR Genetic Alterations

Cancer type: Unspecified Solid Tumor

Variant class: FGFR activating mutation

Other identifiers: NCI-2019-06869, OSU-19041

Population segments: Second line, Stage III, Stage IV

Phase: II

Therapy: infigratinib

Location: United States

US State: OH

Contact: The Ohio State University Comprehensive Cancer Center [800-293-5066; OSUCCCClinicaltrials@osumc.edu]

Disclaimer: The data presented here is from a curated knowledgebase of publicly available information, but may not be exhaustive. The data version is 2021.11(003).

FGFR3 K650Q mutation (continued)

NCT02693535
Targeted Agent and Profiling Utilization Registry (TAPUR) Study.

Cancer type: Unspecified Solid Tumor

Variant class: FGFR3 mutation

Other identifiers: 20170529, NCI-2017-00510, Pro00014171, TAPUR

Population segments: Aggressive, BRAF, Diffuse large B-cell lymphoma (DLBCL), Extranodal marginal zone B-cell lymphoma (MALT), FGFR, First line, Follicular lymphoma (FL), Fourth line or greater, Indolent, Lymphoblastic lymphoma (LBL), Mantle cell lymphoma (MCL), Other subtype, Second line, Small lymphocytic lymphoma (SLL), Stage III, Stage IV, Third line, Waldenstrom's macroglobulinemia (WM)

Phase: II

Therapy: sunitinib

Location: United States

US States: AL, AZ, CA, FL, GA, HI, IL, IN, ME, MI, NC, ND, NE, NH, OH, OR, PA, SD, TX, UT, VA, WA

Contact: Pam Mangat [pam.mangat@asco.org]

NCT04591431
The Rome Trial From Histology to Target: the Road to Personalize Target Therapy and Immunotherapy

Cancer type: Biliary Tract Carcinoma, Breast Cancer, Colorectal Cancer, Esophageal Cancer, Gastric Cancer, Gastroesophageal Junction Adenocarcinoma, Hepatocellular Carcinoma, Liver Cancer, Non-Small Cell Lung Cancer, Pancreatic Cancer, Unspecified Solid Tumor

Variant class: FGFR3 aberration

Other identifiers: EudraCT Number: 2018-002190-21, MAR-BAS-18-005, ROME

Population segments: Second line, Stage IV

Phase: II

Therapy: pemigatinib

Location: Italy

NCT02691793
Study to Evaluate the Safety and Efficacy of Sunitinib, in Subject With Refractory Solid Tumors

Cancer type: Unspecified Solid Tumor

Variant class: FGFR mutation

Other identifier: 2016-02-098

Population segments: (N/A), FGFR, Second line

Phase: II

Therapy: sunitinib

Location: Republic of Korea

Disclaimer: The data presented here is from a curated knowledgebase of publicly available information, but may not be exhaustive. The data version is 2021.11(003).

FGFR3 K650Q mutation (continued)

NCT03297606
Canadian Profiling and Targeted Agent Utilization Trial (CAPTUR): A Phase II Basket Trial

Cancer type: Unspecified Solid Tumor

Variant class: FGFR3 aberration

Other identifiers: CA209-9DL, CAPTUR, ESR-17-12831, ML39800, PM1, WI233446

Population segments: Aggressive, Diffuse large B-cell lymphoma (DLBCL), Extranodal marginal zone B-cell lymphoma (MALT), First line, Follicular lymphoma (FL), Indolent, Lymphoblastic lymphoma (LBL), Mantle cell lymphoma (MCL), Other subtype, Second line, Stage III, Stage IV, Waldenstrom's macroglobulinemia (WM)

Phase: II

Therapy: sunitinib

Location: Canada

NCT03929965
Anlotinib in Advanced Solid Tumors With FGFR Alteration

Cancer type: Unspecified Solid Tumor

Variant class: FGFR mutation

Other identifier: ASTFA

Population segments: Second line, Stage III, Stage IV, Third line

Phase: I

Therapy: anlotinib hydrochloride

Location: China

NCT04565275
A Multi-center Open-label, Phase I/II Clinical Trial to Evaluate the Safety, Tolerability, Pharmacokinetics, and Pharmacodynamics of ICP-192 in Patients With Advanced Solid Tumors and FGFR Gene Alterations.

Cancer type: Bladder Urothelial Carcinoma, Cholangiocarcinoma, Unspecified Solid Tumor

Variant class: FGFR aberration

Other identifier: ICP-CL-00303

Population segments: FGFR, Second line, Stage III, Stage IV

Phase: I/II

Therapy: ICP-192

Location: United States

US States: AZ, CO, FL, MN

Contact: Olivia Yang [609-524-0684; olivia.yang@INNOCAREPHARMA.COM]

No NCT ID - see other identifier(s)
Phase I/II Study of TAS-117 In Combination With TAS-120 In Patients With Advanced Solid Tumors

Cancer type: Unspecified Solid Tumor

Variant class: FGFR aberration

Other identifier: JapicCTI-194864

Population segments: FGFR, First line, Squamous Cell, Stage III, Stage IV

Phase: I/II

Therapies: TAS-117, futibatinib

Location: Japan

Disclaimer: The data presented here is from a curated knowledgebase of publicly available information, but may not be exhaustive. The data version is 2021.11(003).

FGFR3 K650Q mutation (continued)

No NCT ID - see other identifier(s)
A Phase Ib Study to Assess the Safety, Tolerability, and Efficacy of TAS-120 (Futibatinib) in Combination with MK-3475 (Pembrolizumab) in Patients with Solid Tumors.

Cancer type: Unspecified Solid Tumor

Variant class: FGFR aberration

Other identifier: JapicCTI-195063

Population segments: FGFR, Line of therapy N/A, Stage III, Stage IV

Phase: I

Therapies: futibatinib, pembrolizumab

Location: Japan

NCT04197986
Phase III, Multicenter, Double-Blind, Randomized, Placebo-Controlled Trial of Infigratinib for the Adjuvant Treatment of Subjects With Invasive Urothelial Carcinoma With Susceptible FGFR3 Genetic Alterations (PROOF 302).

Cancer type: Bladder Urothelial Carcinoma

Variant class: FGFR3 K650Q mutation

Other identifiers: EudraCT Number: 2019-003248-63, NCI-2020-00610, PROOF 302, QBGJ398-302

Population segments: Adjuvant, FGFR, Stage II, Stage III

Exclusion criteria variant classes: FGFR3 A391E mutation (Bladder Small Cell Neuroendocrine Carcinoma), FGFR3 G370C mutation (Bladder Small Cell Neuroendocrine Carcinoma), FGFR3 K650E mutation (Bladder Small Cell Neuroendocrine Carcinoma), FGFR3 K650M mutation (Bladder Small Cell Neuroendocrine Carcinoma), FGFR3 K650Q mutation (Bladder Small Cell Neuroendocrine Carcinoma), FGFR3 K650T mutation (Bladder Small Cell Neuroendocrine Carcinoma), FGFR3 R248C mutation (Bladder Small Cell Neuroendocrine Carcinoma), FGFR3 S249C mutation (Bladder Small Cell Neuroendocrine Carcinoma), FGFR3 Y373C mutation (Bladder Small Cell Neuroendocrine Carcinoma), FGFR3 fusion (Bladder Small Cell Neuroendocrine Carcinoma)

Phase: III

Therapy: infigratinib

Locations: Belgium, Canada, France, Germany, Italy, Netherlands, Puerto Rico, Spain, United Kingdom, United States

US States: AL, AZ, CA, CO, DC, FL, GA, IL, IN, LA, MA, MD, MO, NC, NE, NJ, NY, OH, OK, SC, TN, TX, UT, VA, WA, WV

Contact: QED Therapeutics [877-280-5655; Proof302.ct@qedtx.com]

NCT04936295
A Phase II Study of the Efficacy and Tolerability of Fulvestrant Plus Anlotinib in HR(+)/HER2(-) Metastatic Breast Cancer Patients With FGFR Mutation

Cancer type: Breast Cancer

Variant class: FGFR3 activating mutation

Other inclusion criteria: ERBB2 negative, Hormone receptor positive

Other identifier: SYSU005

Population segments: Estrogen receptor positive, FGFR, First line, HER2 negative, HER2 positive, Progesterone receptor positive, Stage IV

Phase: II

Therapies: hormone therapy, anlotinib hydrochloride

Location: China

Disclaimer: The data presented here is from a curated knowledgebase of publicly available information, but may not be exhaustive. The data version is 2021.11(003).

FGFR3 K650Q mutation (continued)

NCT04096417

A Phase II, Multicenter, Single-Arm Study of Pemigatinib in Patients With Metastatic or Unresectable Colorectal Cancer Harboring FGFR Alterations

Cancer type: Colorectal Cancer

Variant class: FGFR3 activating mutation

Other identifiers: ACCRU-GI-1701, NCI-2019-05877, VICCGI2028

Population segments: FGFR, Second line, Stage III, Stage IV

Phase: II

Therapy: pemigatinib

Location: United States

US States: AZ, GA, MN, NC, TN

Contact: Multiple contacts: See www.clinicaltrials.gov for complete list of contacts.

NCT04045613

An Open-label Multi-cohort Phase Ib/II Study of Derazantinib and Atezolizumab in Patients With Urothelial Cancer Expressing Activating Molecular FGFR Aberrations.

Cancer type: Bladder Urothelial Carcinoma

Variant class: FGFR3 activating mutation

Other inclusion criteria: CD274 expression

Other identifiers: CSET 3012, DZB-CS-201, EudraCT Number: 2019-000359-15, FIDES-02, NCI-2019-08162, RECF4112, SNCTP000004480

Population segments: FGFR, Fourth line or greater, Second line, Stage IV, Third line

Phase: I/II

Therapies: derazantinib, atezolizumab

Locations: Australia, Austria, Belgium, Canada, Czech Republic, France, Germany, Hungary, Italy, Poland, Republic of Korea, Spain, Switzerland, United Kingdom, United States

US States: GA, IL, KS, KY, NY, SC, TX, VA, WA

Contact: Dr. Manuel HÃ¤ckl [manuel.haeckl@basilea.com]

NCT04053322

An International Multicenter Phase II Trial of Durvalumab (MEDI4736) Plus OLAparib Plus Fulvestrant in Metastatic or Locally Advanced ER-positive, HER2-negative Breast Cancer Patients Selected Using Criteria That Predict Sensitivity to Olaparib

Cancer type: Breast Cancer

Variant class: FGFR3 mutation

Other inclusion criteria: ERBB2 negative, ER positive

Other identifiers: CADUSEIME08, DOLAF, EudraCT Number: 2018-003832-57, UC-0140/1812

Population segments: BRCA, Estrogen receptor positive, FGFR, HER2 negative, Progesterone receptor positive, Second line, Stage III, Stage IV

Phase: II

Therapies: durvalumab, olaparib, hormone therapy

Location: France

FGFR3 K650Q mutation (continued)

NCT03827850

A Phase II Trial to Evaluate Efficacy and Safety of Erdafitinib in Patients With Advanced Squamous Non Small Cell Lung Carcinoma (sqNSCLC) Harboring Fibroblast Growth Factor Receptor (FGFR) Genetic Alterations After Relapse of Standard Therapy.

Cancer type: Non-Small Cell Lung Cancer

Variant class: FGFR activating mutation

Other identifiers: EudraCT Number: 2018-000399-13, FIND, FIND Trial, Uni-Koeln-3254

Population segments: FGFR, Second line, Squamous Cell, Stage III, Stage IV

Exclusion criteria variant classes: ALK aberration (Non-Small Cell Lung Cancer), BRAF aberration (Non-Small Cell Lung Cancer), EGFR aberration (Non-Small Cell Lung Cancer), NTRK aberration (Non-Small Cell Lung Cancer), ROS1 aberration (Non-Small Cell Lung Cancer)

Phase: II

Therapy: erdafitinib

Location: Germany

NCT04601857

A Phase II Study Evaluating Futibatinib (TAS 120) Plus Pembrolizumab in the Treatment of Advanced or Metastatic Urothelial Carcinoma

Cancer type: Bladder Urothelial Carcinoma

Variant class: FGFR3 mutation

Other identifiers: EudraCT Number: 2020-000945-15, TAS-120-203

Population segments: FGFR, First line, Stage III, Stage IV

Phase: II

Therapies: futibatinib, pembrolizumab

Location: United States

US State: MI

Contact: Dr. Maciej Gil [609-250-7336; clinicaltrialinfo@taihooncology.com]

NCT04294277

Open-label, Single-arm, Phase II Study, Evaluating Safety and Efficacy of INCB054828 (Pemigatinib) as Adjuvant Therapy for Molecularly-selected, High-risk Patients With Urothelial Carcinoma Who Have Received Radical Surgery

Cancer type: Bladder Urothelial Carcinoma

Variant class: FGFR3 mutation

Other identifiers: EAU RF 2018-02, EAURF2018-02, EudraCT Number: 2017-004426-15, EudraCT Number: 2019-001833-14, PEGASUS

Population segments: Adjuvant, FGFR, Stage III, Stage IV

Phase: II

Therapy: pemigatinib

Location: Italy

NCT04077255

Anti-EGFR Antibody (GC-1118) in Combination With Weekly Paclitaxel as a Second-line Therapy for Gastric and Gastroesophageal Junction Adenocarcinomas With Amplifications

Cancer type: Gastric Cancer, Gastroesophageal Junction Adenocarcinoma

Variant class: FGFR3 mutation

Other identifiers: 31973, KCT0004314, NCC2019-0196

Population segments: EGFR, FGFR, Second line, Stage IV

Phase: II

Therapies: rogaratinib, chemotherapy

Location: Republic of Korea

Disclaimer: The data presented here is from a curated knowledgebase of publicly available information, but may not be exhaustive. The data version is 2021.11(003).

FGFR3 K650Q mutation (continued)

No NCT ID - see other identifier(s)
Phase I Clinical Study of BPI-17509 In Patients With Advanced Solid Tumors

Cancer type: Bladder Cancer, Solid Tumor

Variant class: FGFR3 mutation

Other identifiers: BTP-26811, CTR20192140

Population segments: Second line, Stage III, Stage IV

Phase: I

Therapy: BPI-17509

Location: China

NCT04172675
A Randomized Phase II Study of Erdafitinib Versus Investigator Choice of Intravesical Chemotherapy in Subjects Who Received Bacillus Calmette-Guerin (BCG) and Recurred With High Risk Non-Muscle-Invasive Bladder Cancer (NMIBC) and FGFR Mutations or Fusions

Cancer type: Bladder Urothelial Carcinoma

Variant class: FGFR mutation

Other identifiers: 42756493BLC2003, 42756493BLC2003, CR108699, CTR20201841, CTRI/2021/08/036009, EudraCT Number: 2019-002449-39, JapicCTI-205145, NCI-2020-00674, RG1121102

Population segments: FGFR, Maintenance/Consolidation, Second line, Stage 0

Phase: II

Therapy: erdafitinib

Locations: Argentina, Australia, Belgium, Brazil, China, Czech Republic, France, Germany, Italy, Japan, Poland, Republic of Korea, Spain, Taiwan, United Kingdom, United States

US States: AZ, CA, CO, FL, GA, IL, KS, NC, NY, OH, PA, TN, TX, WA, WI

Contact: Study Contact [844-434-4210; JNJ.CT@sylogent.com]

NCT02150967
A Phase II Multicenter, Single Arm Study of Oral BGJ398 in Adult Patients with Advanced or Metastatic Cholangiocarcinoma with FGFR2 Gene Fusions or Other FGFR Genetic Alterations who Failed or are Intolerant to Platinum-based Chemotherapy.

Cancer type: Cholangiocarcinoma

Variant class: FGFR activating aberration

Other identifiers: 14-183, 14-2200, 14-328, 15452, 2014-0372, 2014-081, CBGJ398X2204, EudraCT Number: 2013-005085-19, NCI-2014-01310, REec-2014-1022, Trial 3GB-14-1

Population segments: FGFR, Fourth line or greater, Second line, Stage III, Stage IV, Third line

Phase: II

Therapy: infigratinib

Locations: Belgium, Germany, Singapore, Spain, Thailand, United Kingdom, United States

US States: CA, MA, MI, NY, OH, TX

Contact: QED Therapeutics [877-280-5655; clinicaltrials@QEDTx.com]

Disclaimer: The data presented here is from a curated knowledgebase of publicly available information, but may not be exhaustive. The data version is 2021.11(003).

FGFR3 K650Q mutation (continued)

NCT04595747
Phase II Study of Rogaratinib (BAY 1163877) in the Treatment of Patients With Sarcoma Harboring Alterations in Fibroblast Growth Factor Receptor (FGFR) 1-4 and SDH-Deficient Gastrointestinal Stromal Tumor (GIST)

Cancer type: Soft Tissue Sarcoma

Variant class: FGFR3 aberration

Other identifiers: 10411, NCI-2020-08011

Population segments: FGFR, Second line, Stage III, Stage IV

Phase: II

Therapy: rogaratinib

Location: United States

US States: CA, MA, MD, NY

Contact: Multiple contacts: See www.clinicaltrials.gov for complete list of contacts.

NCT04004975
Phase II Clinical Trials on Anlotinib for the Treatment of Recurrent Glioblastoma

Cancer type: Glioblastoma

Variant class: FGFR mutation

Other identifier: ShandongCHI006

Population segments: (N/A), FGFR, Second line

Phase: I/II

Therapy: anlotinib hydrochloride

Location: China

NCT04149691
A Phase I, Open-label, Multicentre, Dose Escalation Study to Assess Safety, Tolerability and Pharmacokinetics of Oral CPL304110, in Adult Subjects With Advanced Solid Malignancies

Cancer type: Bladder Cancer, Gastric Cancer, Non-Small Cell Lung Cancer

Variant class: FGFR3 aberration

Other identifier: 01FGFR2018

Population segments: FGFR, Line of therapy N/A, Squamous Cell, Stage III, Stage IV

Phase: I

Therapy: CPL-304-110

Location: Poland

NCT03473743
A Phase Ib-II Study to Evaluate Safety, Efficacy, Pharmacokinetics, and Pharmacodynamics of Various Regimens of Erdafitinib in Subjects With Metastatic or Locally Advanced Urothelial Cancer

Cancer type: Bladder Urothelial Carcinoma

Variant class: FGFR aberration

Other identifiers: 17165, 2018-0142, 42756493BLC2002, CR108445, EudraCT Number: 2017-001980-19, NCI-2018-00997, NORSE, USO 17165, USOR 17165

Population segments: FGFR, First line, Fourth line or greater, Second line, Stage IV, Third line

Phase: I/II

Therapies: erdafitinib, cetrelimab

Locations: Belarus, Belgium, Brazil, France, Italy, Poland, Republic of Korea, Russian Federation, Spain, Taiwan, Turkey, United Kingdom, United States

US States: CO, MD, NC, NY, PA, TX, VA

Contact: Study Contact [844-434-4210; JNJ.CT@sylogent.com]

FGFR3 K650Q mutation (continued)

NCT02393248
A Phase I/II, Open-Label, Dose-Escalation, Safety and Tolerability Study of INCB054828 in Subjects With Advanced Malignancies - (FIGHT-101)

Cancer type: Bladder Urothelial Carcinoma, Breast Cancer, Cholangiocarcinoma, Endometrial Carcinoma, Gastric Cancer, Non-Small Cell Lung Cancer, Solid Tumor

Variant class: FGFR aberration

Other identifiers: 00061567, 2014-1099, 201503012, EudraCT Number: 2016-002831-14, FIGHT-101, INCB 54828-101, INCB54828-101, MC# 15-03, NCI-2015-00611, OSU-15241

Population segments: FGFR, First line, Fourth line or greater, Second line, Squamous Cell, Stage III, Stage IV, Third line

Phase: I/II

Therapies: pemigatinib, pembrolizumab, trastuzumab, chemotherapy, retifanlimab

Location: United States

US States: FL, NJ, TX

Contact: Incyte Call Center [855-463-3463]

NCT04572295
An Open-label Phase Ib Study of E7090 Monotherapy and in Combination With Other Anticancer Agents in Subjects With ER+, HER2- Recurrent/Metastatic Breast Cancer

Cancer type: Breast Cancer

Variant class: FGFR aberration

Other inclusion criteria: ERBB2 negative, ER positive

Other identifiers: E7090-J081-102, JapicCTI-205429

Population segments: Estrogen receptor positive, HER2 negative, Second line, Stage IV

Phase: I

Therapies: E-7090, hormone therapy

Location: Japan

NCT04504331
A Phase IB Study of Infigratinib in Combination With Tamoxifen in Hormone Receptor Positive, HER2 Negative, FGFR Altered Advanced Breast Cancer

Cancer type: Breast Cancer

Variant class: FGFR aberration

Other inclusion criteria: ERBB2 negative, Hormone receptor positive

Other identifiers: BRS0113, K08CA252457

Population segments: Estrogen receptor positive, HER2 negative, Progesterone receptor positive, Second line, Stage III, Stage IV, Triple receptor negative

Phase: I

Therapies: infigratinib, hormone therapy

Location: United States

US State: CA

Contact: Allison K. Zhang [650-736-5790; axkong@stanford.edu]

Disclaimer: The data presented here is from a curated knowledgebase of publicly available information, but may not be exhaustive. The data version is 2021.11(003).

FGFR3 K650T mutation

NCT05019794
A Phase IIa of Infigratinib in Subjects With Locally Advanced or Metastatic Gastric Cancer or Gastroesophageal Junction Adenocarcinoma With FGFR2 Amplification or Other Advanced Solid Tumors With Other FGFR Alterations

Cancer type: Gastric Cancer, Gastroesophageal Junction Adenocarcinoma, Unspecified Solid Tumor

Variant class: FGFR3 activating mutation

Other identifiers: CTR20210275, LB1001-201

Population segments: FGFR, Fourth line or greater, Second line, Stage III, Stage IV, Third line

Phase: II

Therapy: infigratinib

Location: China

NCT03155620
NCI-COG Pediatric MATCH (Molecular Analysis for Therapy Choice) Screening Protocol

Cancer type: Unspecified Solid Tumor

Variant class: FGFR3 mutation

Other identifiers: 17-729, 2017-0625, 20170841, APEC1621SC, COGAPEC1621, COGAPEC1621SC, N 55017 SC, NCI-2017-01251, NCI-COG Pediatric MATCH, Pediatric MATCH

Population segments: ALK, Aggressive, BRAF, FGFR, HRAS, Indolent, KRAS, NRAS, Pediatric or Adolescent, Second line, Stage III, Stage IV

Phase: II

Therapy: erdafitinib

Locations: Puerto Rico, United States

US States: AK, AL, AR, AZ, CA, CO, CT, DC, DE, FL, GA, HI, IA, ID, IL, IN, KY, LA, MA, MD, ME, MI, MN, MO, MS, NC, NE, NH, NJ, NV, NY, OH, OK, OR, PA, RI, SC, SD, TN, TX, UT, VA, VT, WA, WI

Contact: Multiple contacts: See www.clinicaltrials.gov for complete list of contacts.

NCT03210714
NCI-COG Pediatric MATCH (Molecular Analysis for Therapy Choice) - Phase II Subprotocol of JNJ-42756493 (Erdafitinib) in Patients With Tumors Harboring FGFR 1/2/3/4 Alterations

Cancer type: Unspecified Solid Tumor

Variant class: FGFR3 mutation

Other identifiers: APEC1621B, NCI-2017-01159, NCI-COG Pediatric MATCH, Pediatric MATCH

Population segments: Aggressive, FGFR, Indolent, Pediatric or Adolescent, Second line, Stage III, Stage IV

Phase: II

Therapy: erdafitinib

Locations: Puerto Rico, United States

US States: AK, AL, AR, AZ, CA, CO, CT, DC, DE, FL, GA, IA, ID, IL, IN, KY, LA, MD, ME, MI, MN, MO, MS, NC, ND, NE, NJ, NY, OH, OK, OR, PA, SC, SD, TN, TX, UT, VA, VT, WA, WI

Contact: Multiple contacts: See www.clinicaltrials.gov for complete list of contacts.

Disclaimer: The data presented here is from a curated knowledgebase of publicly available information, but may not be exhaustive. The data version is 2021.11(003).

FGFR3 K650T mutation (continued)

No NCT ID - see other identifier(s)
A Multicenter Phase II Basket-type Clinical Trial to Evaluate Efficacy and Safety of TAS-120 in Patients with Advanced Solid Malignancies with FGFR Alterations in Circulating Tumor DNA

Cancer type: Unspecified Solid Tumor

Variant class: FGFR3 mutation

Other identifiers: EPOC 1805, JapicCTI-194624, JapicCTI-194796, TiFFANY

Population segments: Second line, Stage III, Stage IV

Phase: II

Therapy: futibatinib

Location: Japan

NCT04233567
A Phase II Study of Oral Infigratinib in Adult Patients With Advanced or Metastatic Solid Tumors With FGFR1-3 Gene Fusions or Other FGFR Genetic Alterations

Cancer type: Unspecified Solid Tumor

Variant class: FGFR activating mutation

Other identifiers: NCI-2019-06869, OSU-19041

Population segments: Second line, Stage III, Stage IV

Phase: II

Therapy: infigratinib

Location: United States

US State: OH

Contact: The Ohio State University Comprehensive Cancer Center [800-293-5066; OSUCCCClinicaltrials@osumc.edu]

NCT02693535
Targeted Agent and Profiling Utilization Registry (TAPUR) Study.

Cancer type: Unspecified Solid Tumor

Variant class: FGFR3 mutation

Other identifiers: 20170529, NCI-2017-00510, Pro00014171, TAPUR

Population segments: Aggressive, BRAF, Diffuse large B-cell lymphoma (DLBCL), Extranodal marginal zone B-cell lymphoma (MALT), FGFR, First line, Follicular lymphoma (FL), Fourth line or greater, Indolent, Lymphoblastic lymphoma (LBL), Mantle cell lymphoma (MCL), Other subtype, Second line, Small lymphocytic lymphoma (SLL), Stage III, Stage IV, Third line, Waldenstrom's macroglobulinemia (WM)

Phase: II

Therapy: sunitinib

Location: United States

US States: AL, AZ, CA, FL, GA, HI, IL, IN, ME, MI, NC, ND, NE, NH, OH, OR, PA, SD, TX, UT, VA, WA

Contact: Pam Mangat [pam.mangat@asco.org]

Disclaimer: The data presented here is from a curated knowledgebase of publicly available information, but may not be exhaustive. The data version is 2021.11(003).

FGFR3 K650T mutation (continued)

NCT04591431
The Rome Trial From Histology to Target: the Road to Personalize Target Therapy and Immunotherapy

Cancer type: Biliary Tract Carcinoma, Breast Cancer, Colorectal Cancer, Esophageal Cancer, Gastric Cancer, Gastroesophageal Junction Adenocarcinoma, Hepatocellular Carcinoma, Liver Cancer, Non-Small Cell Lung Cancer, Pancreatic Cancer, Unspecified Solid Tumor

Variant class: FGFR3 aberration

Other identifiers: EudraCT Number: 2018-002190-21, MAR-BAS-18-005, ROME

Population segments: Second line, Stage IV

Phase: II

Therapy: pemigatinib

Location: Italy

NCT02691793
Study to Evaluate the Safety and Efficacy of Sunitinib, in Subject With Refractory Solid Tumors

Cancer type: Unspecified Solid Tumor

Variant class: FGFR mutation

Other identifier: 2016-02-098

Population segments: (N/A), FGFR, Second line

Phase: II

Therapy: sunitinib

Location: Republic of Korea

NCT03297606
Canadian Profiling and Targeted Agent Utilization Trial (CAPTUR): A Phase II Basket Trial

Cancer type: Unspecified Solid Tumor

Variant class: FGFR3 aberration

Other identifiers: CA209-9DL, CAPTUR, ESR-17-12831, ML39800, PM1, WI233446

Population segments: Aggressive, Diffuse large B-cell lymphoma (DLBCL), Extranodal marginal zone B-cell lymphoma (MALT), First line, Follicular lymphoma (FL), Indolent, Lymphoblastic lymphoma (LBL), Mantle cell lymphoma (MCL), Other subtype, Second line, Stage III, Stage IV, Waldenstrom's macroglobulinemia (WM)

Phase: II

Therapy: sunitinib

Location: Canada

NCT03929965
Anlotinib in Advanced Solid Tumors With FGFR Alteration

Cancer type: Unspecified Solid Tumor

Variant class: FGFR mutation

Other identifier: ASTFA

Population segments: Second line, Stage III, Stage IV, Third line

Phase: I

Therapy: anlotinib hydrochloride

Location: China

Disclaimer: The data presented here is from a curated knowledgebase of publicly available information, but may not be exhaustive. The data version is 2021.11(003).

FGFR3 K650T mutation (continued)

NCT04565275
A Multi-center Open-label, Phase I/II Clinical Trial to Evaluate the Safety, Tolerability, Pharmacokinetics, and Pharmacodynamics of ICP-192 in Patients With Advanced Solid Tumors and FGFR Gene Alterations.

Cancer type: Bladder Urothelial Carcinoma, Cholangiocarcinoma, Unspecified Solid Tumor

Variant class: FGFR aberration

Other identifier: ICP-CL-00303

Population segments: FGFR, Second line, Stage III, Stage IV

Phase: I/II

Therapy: ICP-192

Location: United States

US States: AZ, CO, FL, MN

Contact: Olivia Yang [609-524-0684; olivia.yang@INNOCAREPHARMA.COM]

No NCT ID - see other identifier(s)
Phase I/II Study of TAS-117 In Combination With TAS-120 In Patients With Advanced Solid Tumors

Cancer type: Unspecified Solid Tumor

Variant class: FGFR aberration

Other identifier: JapicCTI-194864

Population segments: FGFR, First line, Squamous Cell, Stage III, Stage IV

Phase: I/II

Therapies: TAS-117, futibatinib

Location: Japan

No NCT ID - see other identifier(s)
A Phase Ib Study to Assess the Safety, Tolerability, and Efficacy of TAS-120 (Futibatinib) in Combination with MK-3475 (Pembrolizumab) in Patients with Solid Tumors.

Cancer type: Unspecified Solid Tumor

Variant class: FGFR aberration

Other identifier: JapicCTI-195063

Population segments: FGFR, Line of therapy N/A, Stage III, Stage IV

Phase: I

Therapies: futibatinib, pembrolizumab

Location: Japan

Disclaimer: The data presented here is from a curated knowledgebase of publicly available information, but may not be exhaustive. The data version is 2021.11(003).

FGFR3 K650T mutation (continued)

NCT04197986
Phase III, Multicenter, Double-Blind, Randomized, Placebo-Controlled Trial of Infigratinib for the Adjuvant Treatment of Subjects With Invasive Urothelial Carcinoma With Susceptible FGFR3 Genetic Alterations (PROOF 302).

Cancer type: Bladder Urothelial Carcinoma

Variant class: FGFR3 K650T mutation

Other identifiers: EudraCT Number: 2019-003248-63, NCI-2020-00610, PROOF 302, QBGJ398-302

Population segments: Adjuvant, FGFR, Stage II, Stage III

Exclusion criteria variant classes: FGFR3 A391E mutation (Bladder Small Cell Neuroendocrine Carcinoma), FGFR3 G370C mutation (Bladder Small Cell Neuroendocrine Carcinoma), FGFR3 K650E mutation (Bladder Small Cell Neuroendocrine Carcinoma), FGFR3 K650M mutation (Bladder Small Cell Neuroendocrine Carcinoma), FGFR3 K650Q mutation (Bladder Small Cell Neuroendocrine Carcinoma), FGFR3 K650T mutation (Bladder Small Cell Neuroendocrine Carcinoma), FGFR3 R248C mutation (Bladder Small Cell Neuroendocrine Carcinoma), FGFR3 S249C mutation (Bladder Small Cell Neuroendocrine Carcinoma), FGFR3 Y373C mutation (Bladder Small Cell Neuroendocrine Carcinoma), FGFR3 fusion (Bladder Small Cell Neuroendocrine Carcinoma)

Phase: III

Therapy: infigratinib

Locations: Belgium, Canada, France, Germany, Italy, Netherlands, Puerto Rico, Spain, United Kingdom, United States

US States: AL, AZ, CA, CO, DC, FL, GA, IL, IN, LA, MA, MD, MO, NC, NE, NJ, NY, OH, OK, SC, TN, TX, UT, VA, WA, WV

Contact: QED Therapeutics [877-280-5655; Proof302.ct@qedtx.com]

NCT04936295
A Phase II Study of the Efficacy and Tolerability of Fulvestrant Plus Anlotinib in HR(+)/HER2(-) Metastatic Breast Cancer Patients With FGFR Mutation

Cancer type: Breast Cancer

Variant class: FGFR3 activating mutation

Other inclusion criteria: ERBB2 negative, Hormone receptor positive

Other identifier: SYSU005

Population segments: Estrogen receptor positive, FGFR, First line, HER2 negative, HER2 positive, Progesterone receptor positive, Stage IV

Phase: II

Therapies: hormone therapy, anlotinib hydrochloride

Location: China

NCT04096417
A Phase II, Multicenter, Single-Arm Study of Pemigatinib in Patients With Metastatic or Unresectable Colorectal Cancer Harboring FGFR Alterations

Cancer type: Colorectal Cancer

Variant class: FGFR3 activating mutation

Other identifiers: ACCRU-GI-1701, NCI-2019-05877, VICCGI2028

Population segments: FGFR, Second line, Stage III, Stage IV

Phase: II

Therapy: pemigatinib

Location: United States

US States: AZ, GA, MN, NC, TN

Contact: Multiple contacts: See www.clinicaltrials.gov for complete list of contacts.

Disclaimer: The data presented here is from a curated knowledgebase of publicly available information, but may not be exhaustive. The data version is 2021.11(003).

FGFR3 K650T mutation (continued)

NCT04045613

An Open-label Multi-cohort Phase Ib/II Study of Derazantinib and Atezolizumab in Patients With Urothelial Cancer Expressing Activating Molecular FGFR Aberrations.

Cancer type: Bladder Urothelial Carcinoma

Variant class: FGFR3 activating mutation

Other inclusion criteria: CD274 expression

Other identifiers: CSET 3012, DZB-CS-201, EudraCT Number: 2019-000359-15, FIDES-02, NCI-2019-08162, RECF4112, SNCTP000004480

Population segments: FGFR, Fourth line or greater, Second line, Stage IV, Third line

Phase: I/II

Therapies: derazantinib, atezolizumab

Locations: Australia, Austria, Belgium, Canada, Czech Republic, France, Germany, Hungary, Italy, Poland, Republic of Korea, Spain, Switzerland, United Kingdom, United States

US States: GA, IL, KS, KY, NY, SC, TX, VA, WA

Contact: Dr. Manuel HÃ¤ckl [manuel.haeckl@basilea.com]

NCT04053322

An International Multicenter Phase II Trial of Durvalumab (MEDI4736) Plus OLAparib Plus Fulvestrant in Metastatic or Locally Advanced ER-positive, HER2-negative Breast Cancer Patients Selected Using Criteria That Predict Sensitivity to Olaparib

Cancer type: Breast Cancer

Variant class: FGFR3 mutation

Other inclusion criteria: ERBB2 negative, ER positive

Other identifiers: CADUSEIME08, DOLAF, EudraCT Number: 2018-003832-57, UC-0140/1812

Population segments: BRCA, Estrogen receptor positive, FGFR, HER2 negative, Progesterone receptor positive, Second line, Stage III, Stage IV

Phase: II

Therapies: durvalumab, olaparib, hormone therapy

Location: France

NCT03827850

A Phase II Trial to Evaluate Efficacy and Safety of Erdafitinib in Patients With Advanced Squamous Non Small Cell Lung Carcinoma (sqNSCLC) Harboring Fibroblast Growth Factor Receptor (FGFR) Genetic Alterations After Relapse of Standard Therapy.

Cancer type: Non-Small Cell Lung Cancer

Variant class: FGFR activating mutation

Other identifiers: EudraCT Number: 2018-000399-13, FIND, FIND Trial, Uni-Koeln-3254

Population segments: FGFR, Second line, Squamous Cell, Stage III, Stage IV

Exclusion criteria variant classes: ALK aberration (Non-Small Cell Lung Cancer), BRAF aberration (Non-Small Cell Lung Cancer), EGFR aberration (Non-Small Cell Lung Cancer), NTRK aberration (Non-Small Cell Lung Cancer), ROS1 aberration (Non-Small Cell Lung Cancer)

Phase: II

Therapy: erdafitinib

Location: Germany

FGFR3 K650T mutation (continued)

NCT04601857
A Phase II Study Evaluating Futibatinib (TAS 120) Plus Pembrolizumab in the Treatment of Advanced or Metastatic Urothelial Carcinoma

Cancer type: Bladder Urothelial Carcinoma

Variant class: FGFR3 mutation

Other identifiers: EudraCT Number: 2020-000945-15, TAS-120-203

Population segments: FGFR, First line, Stage III, Stage IV

Phase: II

Therapies: futibatinib, pembrolizumab

Location: United States

US State: MI

Contact: Dr. Maciej Gil [609-250-7336; clinicaltrialinfo@taihooncology.com]

NCT04294277
Open-label, Single-arm, Phase II Study, Evaluating Safety and Efficacy of INCB054828 (Pemigatinib) as Adjuvant Therapy for Molecularly-selected, High-risk Patients With Urothelial Carcinoma Who Have Received Radical Surgery

Cancer type: Bladder Urothelial Carcinoma

Variant class: FGFR3 mutation

Other identifiers: EAU RF 2018-02, EAURF2018-02, EudraCT Number: 2017-004426-15, EudraCT Number: 2019-001833-14, PEGASUS

Population segments: Adjuvant, FGFR, Stage III, Stage IV

Phase: II

Therapy: pemigatinib

Location: Italy

NCT04077255
Anti-EGFR Antibody (GC-1118) in Combination With Weekly Paclitaxel as a Second-line Therapy for Gastric and Gastroesophageal Junction Adenocarcinomas With Amplifications

Cancer type: Gastric Cancer, Gastroesophageal Junction Adenocarcinoma

Variant class: FGFR3 mutation

Other identifiers: 31973, KCT0004314, NCC2019-0196

Population segments: EGFR, FGFR, Second line, Stage IV

Phase: II

Therapies: rogaratinib, chemotherapy

Location: Republic of Korea

No NCT ID - see other identifier(s)
Phase I Clinical Study of BPI-17509 In Patients With Advanced Solid Tumors

Cancer type: Bladder Cancer, Solid Tumor

Variant class: FGFR3 mutation

Other identifiers: BTP-26811, CTR20192140

Population segments: Second line, Stage III, Stage IV

Phase: I

Therapy: BPI-17509

Location: China

FGFR3 K650T mutation (continued)

NCT04172675

A Randomized Phase II Study of Erdafitinib Versus Investigator Choice of Intravesical Chemotherapy in Subjects Who Received Bacillus Calmette-Guerin (BCG) and Recurred With High Risk Non-Muscle-Invasive Bladder Cancer (NMIBC) and FGFR Mutations or Fusions

Cancer type: Bladder Urothelial Carcinoma

Variant class: FGFR mutation

Other identifiers: 42756493BLC2003, 42756493BLC2003, CR108699, CTR20201841, CTRI/2021/08/036009, EudraCT Number: 2019-002449-39, JapicCTI-205145, NCI-2020-00674, RG1121102

Population segments: FGFR, Maintenance/Consolidation, Second line, Stage 0

Phase: II

Therapy: erdafitinib

Locations: Argentina, Australia, Belgium, Brazil, China, Czech Republic, France, Germany, Italy, Japan, Poland, Republic of Korea, Spain, Taiwan, United Kingdom, United States

US States: AZ, CA, CO, FL, GA, IL, KS, NC, NY, OH, PA, TN, TX, WA, WI

Contact: Study Contact [844-434-4210; JNJ.CT@sylogent.com]

NCT02150967

A Phase II Multicenter, Single Arm Study of Oral BGJ398 in Adult Patients with Advanced or Metastatic Cholangiocarcinoma with FGFR2 Gene Fusions or Other FGFR Genetic Alterations who Failed or are Intolerant to Platinum-based Chemotherapy.

Cancer type: Cholangiocarcinoma

Variant class: FGFR activating aberration

Other identifiers: 14-183, 14-2200, 14-328, 15452, 2014-0372, 2014-081, CBGJ398X2204, EudraCT Number: 2013-005085-19, NCI-2014-01310, REec-2014-1022, Trial 3GB-14-1

Population segments: FGFR, Fourth line or greater, Second line, Stage III, Stage IV, Third line

Phase: II

Therapy: infigratinib

Locations: Belgium, Germany, Singapore, Spain, Thailand, United Kingdom, United States

US States: CA, MA, MI, NY, OH, TX

Contact: QED Therapeutics [877-280-5655; clinicaltrials@QEDTx.com]

NCT04595747

Phase II Study of Rogaratinib (BAY 1163877) in the Treatment of Patients With Sarcoma Harboring Alterations in Fibroblast Growth Factor Receptor (FGFR) 1-4 and SDH-Deficient Gastrointestinal Stromal Tumor (GIST)

Cancer type: Soft Tissue Sarcoma

Variant class: FGFR3 aberration

Other identifiers: 10411, NCI-2020-08011

Population segments: FGFR, Second line, Stage III, Stage IV

Phase: II

Therapy: rogaratinib

Location: United States

US States: CA, MA, MD, NY

Contact: Multiple contacts: See www.clinicaltrials.gov for complete list of contacts.

Disclaimer: The data presented here is from a curated knowledgebase of publicly available information, but may not be exhaustive. The data version is 2021.11(003).

FGFR3 K650T mutation (continued)

NCT04004975
Phase II Clinical Trials on Anlotinib for the Treatment of Recurrent Glioblastoma

Cancer type: Glioblastoma

Variant class: FGFR mutation

Other identifier: ShandongCHI006

Population segments: (N/A), FGFR, Second line

Phase: I/II

Therapy: anlotinib hydrochloride

Location: China

NCT04149691
A Phase I, Open-label, Multicentre, Dose Escalation Study to Assess Safety, Tolerability and Pharmacokinetics of Oral CPL304110, in Adult Subjects With Advanced Solid Malignancies

Cancer type: Bladder Cancer, Gastric Cancer, Non-Small Cell Lung Cancer

Variant class: FGFR3 aberration

Other identifier: 01FGFR2018

Population segments: FGFR, Line of therapy N/A, Squamous Cell, Stage III, Stage IV

Phase: I

Therapy: CPL-304-110

Location: Poland

NCT03473743
A Phase Ib-II Study to Evaluate Safety, Efficacy, Pharmacokinetics, and Pharmacodynamics of Various Regimens of Erdafitinib in Subjects With Metastatic or Locally Advanced Urothelial Cancer

Cancer type: Bladder Urothelial Carcinoma

Variant class: FGFR aberration

Other identifiers: 17165, 2018-0142, 42756493BLC2002, CR108445, EudraCT Number: 2017-001980-19, NCI-2018-00997, NORSE, USO 17165, USOR 17165

Population segments: FGFR, First line, Fourth line or greater, Second line, Stage IV, Third line

Phase: I/II

Therapies: erdafitinib, cetrelimab

Locations: Belarus, Belgium, Brazil, France, Italy, Poland, Republic of Korea, Russian Federation, Spain, Taiwan, Turkey, United Kingdom, United States

US States: CO, MD, NC, NY, PA, TX, VA

Contact: Study Contact [844-434-4210; JNJ.CT@sylogent.com]

NCT02393248
A Phase I/II, Open-Label, Dose-Escalation, Safety and Tolerability Study of INCB054828 in Subjects With Advanced Malignancies - (FIGHT-101)

Cancer type: Bladder Urothelial Carcinoma, Breast Cancer, Cholangiocarcinoma, Endometrial Carcinoma, Gastric Cancer, Non-Small Cell Lung Cancer, Solid Tumor

Variant class: FGFR aberration

Other identifiers: 00061567, 2014-1099, 201503012, EudraCT Number: 2016-002831-14, FIGHT-101, INCB 54828-101, INCB54828-101, MC# 15-03, NCI-2015-00611, OSU-15241

Population segments: FGFR, First line, Fourth line or greater, Second line, Squamous Cell, Stage III, Stage IV, Third line

Phase: I/II

Therapies: pemigatinib, pembrolizumab, trastuzumab, chemotherapy, retifanlimab

Location: United States

US States: FL, NJ, TX

Contact: Incyte Call Center [855-463-3463]

Disclaimer: The data presented here is from a curated knowledgebase of publicly available information, but may not be exhaustive. The data version is 2021.11(003).

FGFR3 K650T mutation (continued)

NCT04572295
An Open-label Phase Ib Study of E7090 Monotherapy and in Combination With Other Anticancer Agents in Subjects With ER+, HER2- Recurrent/Metastatic Breast Cancer

Cancer type: Breast Cancer

Variant class: FGFR aberration

Other inclusion criteria: ERBB2 negative, ER positive

Other identifiers: E7090-J081-102, JapicCTI-205429

Population segments: Estrogen receptor positive, HER2 negative, Second line, Stage IV

Phase: I

Therapies: E-7090, hormone therapy

Location: Japan

NCT04504331
A Phase IB Study of Infigratinib in Combination With Tamoxifen in Hormone Receptor Positive, HER2 Negative, FGFR Altered Advanced Breast Cancer

Cancer type: Breast Cancer

Variant class: FGFR aberration

Other inclusion criteria: ERBB2 negative, Hormone receptor positive

Other identifiers: BRS0113, K08CA252457

Population segments: Estrogen receptor positive, HER2 negative, Progesterone receptor positive, Second line, Stage III, Stage IV, Triple receptor negative

Phase: I

Therapies: infigratinib, hormone therapy

Location: United States

US State: CA

Contact: Allison K. Zhang [650-736-5790; axkong@stanford.edu]

FGFR3 activating mutation

NCT05019794
A Phase IIa of Infigratinib in Subjects With Locally Advanced or Metastatic Gastric Cancer or Gastroesophageal Junction Adenocarcinoma With FGFR2 Amplification or Other Advanced Solid Tumors With Other FGFR Alterations

Cancer type: Gastric Cancer, Gastroesophageal Junction Adenocarcinoma, Unspecified Solid Tumor

Variant class: FGFR3 activating mutation

Other identifiers: CTR20210275, LB1001-201

Population segments: FGFR, Fourth line or greater, Second line, Stage III, Stage IV, Third line

Phase: II

Therapy: infigratinib

Location: China

Disclaimer: The data presented here is from a curated knowledgebase of publicly available information, but may not be exhaustive. The data version is 2021.11(003).

FGFR3 activating mutation (continued)

NCT03155620
NCI-COG Pediatric MATCH (Molecular Analysis for Therapy Choice) Screening Protocol

Cancer type: Unspecified Solid Tumor

Variant class: FGFR3 mutation

Other identifiers: 17-729, 2017-0625, 20170841, APEC1621SC, COGAPEC1621, COGAPEC1621SC, N 55017 SC, NCI-2017-01251, NCI-COG Pediatric MATCH, Pediatric MATCH

Population segments: ALK, Aggressive, BRAF, FGFR, HRAS, Indolent, KRAS, NRAS, Pediatric or Adolescent, Second line, Stage III, Stage IV

Phase: II

Therapy: erdafitinib

Locations: Puerto Rico, United States

US States: AK, AL, AR, AZ, CA, CO, CT, DC, DE, FL, GA, HI, IA, ID, IL, IN, KY, LA, MA, MD, ME, MI, MN, MO, MS, NC, NE, NH, NJ, NV, NY, OH, OK, OR, PA, RI, SC, SD, TN, TX, UT, VA, VT, WA, WI

Contact: Multiple contacts: See www.clinicaltrials.gov for complete list of contacts.

NCT03210714
NCI-COG Pediatric MATCH (Molecular Analysis for Therapy Choice) - Phase II Subprotocol of JNJ-42756493 (Erdafitinib) in Patients With Tumors Harboring FGFR 1/2/3/4 Alterations

Cancer type: Unspecified Solid Tumor

Variant class: FGFR3 mutation

Other identifiers: APEC1621B, NCI-2017-01159, NCI-COG Pediatric MATCH, Pediatric MATCH

Population segments: Aggressive, FGFR, Indolent, Pediatric or Adolescent, Second line, Stage III, Stage IV

Phase: II

Therapy: erdafitinib

Locations: Puerto Rico, United States

US States: AK, AL, AR, AZ, CA, CO, CT, DC, DE, FL, GA, IA, ID, IL, IN, KY, LA, MD, ME, MI, MN, MO, MS, NC, ND, NE, NJ, NY, OH, OK, OR, PA, SC, SD, TN, TX, UT, VA, VT, WA, WI

Contact: Multiple contacts: See www.clinicaltrials.gov for complete list of contacts.

No NCT ID - see other identifier(s)
A Multicenter Phase II Basket-type Clinical Trial to Evaluate Efficacy and Safety of TAS-120 in Patients with Advanced Solid Malignancies with FGFR Alterations in Circulating Tumor DNA

Cancer type: Unspecified Solid Tumor

Variant class: FGFR3 mutation

Other identifiers: EPOC 1805, JapicCTI-194624, JapicCTI-194796, TiFFANY

Population segments: Second line, Stage III, Stage IV

Phase: II

Therapy: futibatinib

Location: Japan

FGFR3 activating mutation (continued)

NCT04233567
A Phase II Study of Oral Infigratinib in Adult Patients With Advanced or Metastatic Solid Tumors With FGFR1-3 Gene Fusions or Other FGFR Genetic Alterations

Cancer type: Unspecified Solid Tumor

Variant class: FGFR activating mutation

Other identifiers: NCI-2019-06869, OSU-19041

Population segments: Second line, Stage III, Stage IV

Phase: II

Therapy: infigratinib

Location: United States

US State: OH

Contact: The Ohio State University Comprehensive Cancer Center [800-293-5066; OSUCCCClinicaltrials@osumc.edu]

NCT02693535
Targeted Agent and Profiling Utilization Registry (TAPUR) Study.

Cancer type: Unspecified Solid Tumor

Variant class: FGFR3 mutation

Other identifiers: 20170529, NCI-2017-00510, Pro00014171, TAPUR

Population segments: Aggressive, BRAF, Diffuse large B-cell lymphoma (DLBCL), Extranodal marginal zone B-cell lymphoma (MALT), FGFR, First line, Follicular lymphoma (FL), Fourth line or greater, Indolent, Lymphoblastic lymphoma (LBL), Mantle cell lymphoma (MCL), Other subtype, Second line, Small lymphocytic lymphoma (SLL), Stage III, Stage IV, Third line, Waldenstrom's macroglobulinemia (WM)

Phase: II

Therapy: sunitinib

Location: United States

US States: AL, AZ, CA, FL, GA, HI, IL, IN, ME, MI, NC, ND, NE, NH, OH, OR, PA, SD, TX, UT, VA, WA

Contact: Pam Mangat [pam.mangat@asco.org]

NCT04591431
The Rome Trial From Histology to Target: the Road to Personalize Target Therapy and Immunotherapy

Cancer type: Biliary Tract Carcinoma, Breast Cancer, Colorectal Cancer, Esophageal Cancer, Gastric Cancer, Gastroesophageal Junction Adenocarcinoma, Hepatocellular Carcinoma, Liver Cancer, Non-Small Cell Lung Cancer, Pancreatic Cancer, Unspecified Solid Tumor

Variant class: FGFR3 aberration

Other identifiers: EudraCT Number: 2018-002190-21, MAR-BAS-18-005, ROME

Population segments: Second line, Stage IV

Phase: II

Therapy: pemigatinib

Location: Italy

Disclaimer: The data presented here is from a curated knowledgebase of publicly available information, but may not be exhaustive. The data version is 2021.11(003).

FGFR3 activating mutation (continued)

NCT02691793
Study to Evaluate the Safety and Efficacy of Sunitinib, in Subject With Refractory Solid Tumors

Cancer type: Unspecified Solid Tumor

Variant class: FGFR mutation

Other identifier: 2016-02-098

Population segments: (N/A), FGFR, Second line

Phase: II

Therapy: sunitinib

Location: Republic of Korea

NCT03297606
Canadian Profiling and Targeted Agent Utilization Trial (CAPTUR): A Phase II Basket Trial

Cancer type: Unspecified Solid Tumor

Variant class: FGFR3 aberration

Other identifiers: CA209-9DL, CAPTUR, ESR-17-12831, ML39800, PM1, WI233446

Population segments: Aggressive, Diffuse large B-cell lymphoma (DLBCL), Extranodal marginal zone B-cell lymphoma (MALT), First line, Follicular lymphoma (FL), Indolent, Lymphoblastic lymphoma (LBL), Mantle cell lymphoma (MCL), Other subtype, Second line, Stage III, Stage IV, Waldenstrom's macroglobulinemia (WM)

Phase: II

Therapy: sunitinib

Location: Canada

NCT03929965
Anlotinib in Advanced Solid Tumors With FGFR Alteration

Cancer type: Unspecified Solid Tumor

Variant class: FGFR mutation

Other identifier: ASTFA

Population segments: Second line, Stage III, Stage IV, Third line

Phase: I

Therapy: anlotinib hydrochloride

Location: China

NCT04565275
A Multi-center Open-label, Phase I/II Clinical Trial to Evaluate the Safety, Tolerability, Pharmacokinetics, and Pharmacodynamics of ICP-192 in Patients With Advanced Solid Tumors and FGFR Gene Alterations.

Cancer type: Bladder Urothelial Carcinoma, Cholangiocarcinoma, Unspecified Solid Tumor

Variant class: FGFR aberration

Other identifier: ICP-CL-00303

Population segments: FGFR, Second line, Stage III, Stage IV

Phase: I/II

Therapy: ICP-192

Location: United States

US States: AZ, CO, FL, MN

Contact: Olivia Yang [609-524-0684; olivia.yang@INNOCAREPHARMA.COM]

Disclaimer: The data presented here is from a curated knowledgebase of publicly available information, but may not be exhaustive. The data version is 2021.11(003).

FGFR3 activating mutation (continued)

No NCT ID - see other identifier(s)
Phase I/II Study of TAS-117 In Combination With TAS-120 In Patients With Advanced Solid Tumors

Cancer type: Unspecified Solid Tumor

Variant class: FGFR aberration

Other identifier: JapicCTI-194864

Population segments: FGFR, First line, Squamous Cell, Stage III, Stage IV

Phase: I/II

Therapies: TAS-117, futibatinib

Location: Japan

No NCT ID - see other identifier(s)
A Phase Ib Study to Assess the Safety, Tolerability, and Efficacy of TAS-120 (Futibatinib) in Combination with MK-3475 (Pembrolizumab) in Patients with Solid Tumors.

Cancer type: Unspecified Solid Tumor

Variant class: FGFR aberration

Other identifier: JapicCTI-195063

Population segments: FGFR, Line of therapy N/A, Stage III, Stage IV

Phase: I

Therapies: futibatinib, pembrolizumab

Location: Japan

NCT04936295
A Phase II Study of the Efficacy and Tolerability of Fulvestrant Plus Anlotinib in HR(+)/HER2(-) Metastatic Breast Cancer Patients With FGFR Mutation

Cancer type: Breast Cancer

Variant class: FGFR3 activating mutation

Other inclusion criteria: ERBB2 negative, Hormone receptor positive

Other identifier: SYSU005

Population segments: Estrogen receptor positive, FGFR, First line, HER2 negative, HER2 positive, Progesterone receptor positive, Stage IV

Phase: II

Therapies: hormone therapy, anlotinib hydrochloride

Location: China

NCT04096417
A Phase II, Multicenter, Single-Arm Study of Pemigatinib in Patients With Metastatic or Unresectable Colorectal Cancer Harboring FGFR Alterations

Cancer type: Colorectal Cancer

Variant class: FGFR3 activating mutation

Other identifiers: ACCRU-GI-1701, NCI-2019-05877, VICCGI2028

Population segments: FGFR, Second line, Stage III, Stage IV

Phase: II

Therapy: pemigatinib

Location: United States

US States: AZ, GA, MN, NC, TN

Contact: Multiple contacts: See www.clinicaltrials.gov for complete list of contacts.

Disclaimer: The data presented here is from a curated knowledgebase of publicly available information, but may not be exhaustive. The data version is 2021.11(003).

FGFR3 activating mutation (continued)

NCT04045613

An Open-label Multi-cohort Phase Ib/II Study of Derazantinib and Atezolizumab in Patients With Urothelial Cancer Expressing Activating Molecular FGFR Aberrations.

Cancer type: Bladder Urothelial Carcinoma

Variant class: FGFR3 activating mutation

Other inclusion criteria: CD274 expression

Other identifiers: CSET 3012, DZB-CS-201, EudraCT Number: 2019-000359-15, FIDES-02, NCI-2019-08162, RECF4112, SNCTP000004480

Population segments: FGFR, Fourth line or greater, Second line, Stage IV, Third line

Phase: I/II

Therapies: derazantinib, atezolizumab

Locations: Australia, Austria, Belgium, Canada, Czech Republic, France, Germany, Hungary, Italy, Poland, Republic of Korea, Spain, Switzerland, United Kingdom, United States

US States: GA, IL, KS, KY, NY, SC, TX, VA, WA

Contact: Dr. Manuel HÃ¤ckl [manuel.haeckl@basilea.com]

NCT04053322

An International Multicenter Phase II Trial of Durvalumab (MEDI4736) Plus OLAparib Plus Fulvestrant in Metastatic or Locally Advanced ER-positive, HER2-negative Breast Cancer Patients Selected Using Criteria That Predict Sensitivity to Olaparib

Cancer type: Breast Cancer

Variant class: FGFR3 mutation

Other inclusion criteria: ERBB2 negative, ER positive

Other identifiers: CADUSEIME08, DOLAF, EudraCT Number: 2018-003832-57, UC-0140/1812

Population segments: BRCA, Estrogen receptor positive, FGFR, HER2 negative, Progesterone receptor positive, Second line, Stage III, Stage IV

Phase: II

Therapies: durvalumab, olaparib, hormone therapy

Location: France

NCT03827850

A Phase II Trial to Evaluate Efficacy and Safety of Erdafitinib in Patients With Advanced Squamous Non Small Cell Lung Carcinoma (sqNSCLC) Harboring Fibroblast Growth Factor Receptor (FGFR) Genetic Alterations After Relapse of Standard Therapy.

Cancer type: Non-Small Cell Lung Cancer

Variant class: FGFR activating mutation

Other identifiers: EudraCT Number: 2018-000399-13, FIND, FIND Trial, Uni-Koeln-3254

Population segments: FGFR, Second line, Squamous Cell, Stage III, Stage IV

Exclusion criteria variant classes: ALK aberration (Non-Small Cell Lung Cancer), BRAF aberration (Non-Small Cell Lung Cancer), EGFR aberration (Non-Small Cell Lung Cancer), NTRK aberration (Non-Small Cell Lung Cancer), ROS1 aberration (Non-Small Cell Lung Cancer)

Phase: II

Therapy: erdafitinib

Location: Germany

FGFR3 activating mutation (continued)

NCT04601857
A Phase II Study Evaluating Futibatinib (TAS 120) Plus Pembrolizumab in the Treatment of Advanced or Metastatic Urothelial Carcinoma

Cancer type: Bladder Urothelial Carcinoma

Variant class: FGFR3 mutation

Other identifiers: EudraCT Number: 2020-000945-15, TAS-120-203

Population segments: FGFR, First line, Stage III, Stage IV

Phase: II

Therapies: futibatinib, pembrolizumab

Location: United States

US State: MI

Contact: Dr. Maciej Gil [609-250-7336; clinicaltrialinfo@taihooncology.com]

NCT04294277
Open-label, Single-arm, Phase II Study, Evaluating Safety and Efficacy of INCB054828 (Pemigatinib) as Adjuvant Therapy for Molecularly-selected, High-risk Patients With Urothelial Carcinoma Who Have Received Radical Surgery

Cancer type: Bladder Urothelial Carcinoma

Variant class: FGFR3 mutation

Other identifiers: EAU RF 2018-02, EAURF2018-02, EudraCT Number: 2017-004426-15, EudraCT Number: 2019-001833-14, PEGASUS

Population segments: Adjuvant, FGFR, Stage III, Stage IV

Phase: II

Therapy: pemigatinib

Location: Italy

NCT04077255
Anti-EGFR Antibody (GC-1118) in Combination With Weekly Paclitaxel as a Second-line Therapy for Gastric and Gastroesophageal Junction Adenocarcinomas With Amplifications

Cancer type: Gastric Cancer, Gastroesophageal Junction Adenocarcinoma

Variant class: FGFR3 mutation

Other identifiers: 31973, KCT0004314, NCC2019-0196

Population segments: EGFR, FGFR, Second line, Stage IV

Phase: II

Therapies: rogaratinib, chemotherapy

Location: Republic of Korea

No NCT ID - see other identifier(s)
Phase I Clinical Study of BPI-17509 In Patients With Advanced Solid Tumors

Cancer type: Bladder Cancer, Solid Tumor

Variant class: FGFR3 mutation

Other identifiers: BTP-26811, CTR20192140

Population segments: Second line, Stage III, Stage IV

Phase: I

Therapy: BPI-17509

Location: China

Disclaimer: The data presented here is from a curated knowledgebase of publicly available information, but may not be exhaustive. The data version is 2021.11(003).

FGFR3 activating mutation (continued)

NCT04172675
A Randomized Phase II Study of Erdafitinib Versus Investigator Choice of Intravesical Chemotherapy in Subjects Who Received Bacillus Calmette-Guerin (BCG) and Recurred With High Risk Non-Muscle-Invasive Bladder Cancer (NMIBC) and FGFR Mutations or Fusions

Cancer type: Bladder Urothelial Carcinoma

Variant class: FGFR mutation

Other identifiers: 42756493BLC2003, 42756493BLC2003, CR108699, CTR20201841, CTRI/2021/08/036009, EudraCT Number: 2019-002449-39, JapicCTI-205145, NCI-2020-00674, RG1121102

Population segments: FGFR, Maintenance/Consolidation, Second line, Stage 0

Phase: II

Therapy: erdafitinib

Locations: Argentina, Australia, Belgium, Brazil, China, Czech Republic, France, Germany, Italy, Japan, Poland, Republic of Korea, Spain, Taiwan, United Kingdom, United States

US States: AZ, CA, CO, FL, GA, IL, KS, NC, NY, OH, PA, TN, TX, WA, WI

Contact: Study Contact [844-434-4210; JNJ.CT@sylogent.com]

NCT02150967
A Phase II Multicenter, Single Arm Study of Oral BGJ398 in Adult Patients with Advanced or Metastatic Cholangiocarcinoma with FGFR2 Gene Fusions or Other FGFR Genetic Alterations who Failed or are Intolerant to Platinum-based Chemotherapy.

Cancer type: Cholangiocarcinoma

Variant class: FGFR activating aberration

Other identifiers: 14-183, 14-2200, 14-328, 15452, 2014-0372, 2014-081, CBGJ398X2204, EudraCT Number: 2013-005085-19, NCI-2014-01310, REec-2014-1022, Trial 3GB-14-1

Population segments: FGFR, Fourth line or greater, Second line, Stage III, Stage IV, Third line

Phase: II

Therapy: infigratinib

Locations: Belgium, Germany, Singapore, Spain, Thailand, United Kingdom, United States

US States: CA, MA, MI, NY, OH, TX

Contact: QED Therapeutics [877-280-5655; clinicaltrials@QEDTx.com]

NCT04595747
Phase II Study of Rogaratinib (BAY 1163877) in the Treatment of Patients With Sarcoma Harboring Alterations in Fibroblast Growth Factor Receptor (FGFR) 1-4 and SDH-Deficient Gastrointestinal Stromal Tumor (GIST)

Cancer type: Soft Tissue Sarcoma

Variant class: FGFR3 aberration

Other identifiers: 10411, NCI-2020-08011

Population segments: FGFR, Second line, Stage III, Stage IV

Phase: II

Therapy: rogaratinib

Location: United States

US States: CA, MA, MD, NY

Contact: Multiple contacts: See www.clinicaltrials.gov for complete list of contacts.

Disclaimer: The data presented here is from a curated knowledgebase of publicly available information, but may not be exhaustive. The data version is 2021.11(003).

FGFR3 activating mutation (continued)

NCT04004975
Phase II Clinical Trials on Anlotinib for the Treatment of Recurrent Glioblastoma

Cancer type: Glioblastoma

Variant class: FGFR mutation

Other identifier: ShandongCHI006

Population segments: (N/A), FGFR, Second line

Phase: I/II

Therapy: anlotinib hydrochloride

Location: China

NCT04149691
A Phase I, Open-label, Multicentre, Dose Escalation Study to Assess Safety, Tolerability and Pharmacokinetics of Oral CPL304110, in Adult Subjects With Advanced Solid Malignancies

Cancer type: Bladder Cancer, Gastric Cancer, Non-Small Cell Lung Cancer

Variant class: FGFR3 aberration

Other identifier: 01FGFR2018

Population segments: FGFR, Line of therapy N/A, Squamous Cell, Stage III, Stage IV

Phase: I

Therapy: CPL-304-110

Location: Poland

NCT03473743
A Phase Ib-II Study to Evaluate Safety, Efficacy, Pharmacokinetics, and Pharmacodynamics of Various Regimens of Erdafitinib in Subjects With Metastatic or Locally Advanced Urothelial Cancer

Cancer type: Bladder Urothelial Carcinoma

Variant class: FGFR aberration

Other identifiers: 17165, 2018-0142, 42756493BLC2002, CR108445, EudraCT Number: 2017-001980-19, NCI-2018-00997, NORSE, USO 17165, USOR 17165

Population segments: FGFR, First line, Fourth line or greater, Second line, Stage IV, Third line

Phase: I/II

Therapies: erdafitinib, cetrelimab

Locations: Belarus, Belgium, Brazil, France, Italy, Poland, Republic of Korea, Russian Federation, Spain, Taiwan, Turkey, United Kingdom, United States

US States: CO, MD, NC, NY, PA, TX, VA

Contact: Study Contact [844-434-4210; JNJ.CT@sylogent.com]

NCT02393248
A Phase I/II, Open-Label, Dose-Escalation, Safety and Tolerability Study of INCB054828 in Subjects With Advanced Malignancies - (FIGHT-101)

Cancer type: Bladder Urothelial Carcinoma, Breast Cancer, Cholangiocarcinoma, Endometrial Carcinoma, Gastric Cancer, Non-Small Cell Lung Cancer, Solid Tumor

Variant class: FGFR aberration

Other identifiers: 00061567, 2014-1099, 201503012, EudraCT Number: 2016-002831-14, FIGHT-101, INCB 54828-101, INCB54828-101, MC# 15-03, NCI-2015-00611, OSU-15241

Population segments: FGFR, First line, Fourth line or greater, Second line, Squamous Cell, Stage III, Stage IV, Third line

Phase: I/II

Therapies: pemigatinib, pembrolizumab, trastuzumab, chemotherapy, retifanlimab

Location: United States

US States: FL, NJ, TX

Contact: Incyte Call Center [855-463-3463]

Disclaimer: The data presented here is from a curated knowledgebase of publicly available information, but may not be exhaustive. The data version is 2021.11(003).

FGFR3 activating mutation (continued)

NCT04572295
An Open-label Phase Ib Study of E7090 Monotherapy and in Combination With Other Anticancer Agents in Subjects With ER+, HER2- Recurrent/Metastatic Breast Cancer

Cancer type: Breast Cancer

Variant class: FGFR aberration

Other inclusion criteria: ERBB2 negative, ER positive

Other identifiers: E7090-J081-102, JapicCTI-205429

Population segments: Estrogen receptor positive, HER2 negative, Second line, Stage IV

Phase: I

Therapies: E-7090, hormone therapy

Location: Japan

NCT04504331
A Phase IB Study of Infigratinib in Combination With Tamoxifen in Hormone Receptor Positive, HER2 Negative, FGFR Altered Advanced Breast Cancer

Cancer type: Breast Cancer

Variant class: FGFR aberration

Other inclusion criteria: ERBB2 negative, Hormone receptor positive

Other identifiers: BRS0113, K08CA252457

Population segments: Estrogen receptor positive, HER2 negative, Progesterone receptor positive, Second line, Stage III, Stage IV, Triple receptor negative

Phase: I

Therapies: infigratinib, hormone therapy

Location: United States

US State: CA

Contact: Allison K. Zhang [650-736-5790; axkong@stanford.edu]

FGFR3 K652E mutation

NCT05019794
A Phase IIa of Infigratinib in Subjects With Locally Advanced or Metastatic Gastric Cancer or Gastroesophageal Junction Adenocarcinoma With FGFR2 Amplification or Other Advanced Solid Tumors With Other FGFR Alterations

Cancer type: Gastric Cancer, Gastroesophageal Junction Adenocarcinoma, Unspecified Solid Tumor

Variant class: FGFR3 activating mutation

Other identifiers: CTR20210275, LB1001-201

Population segments: FGFR, Fourth line or greater, Second line, Stage III, Stage IV, Third line

Phase: II

Therapy: infigratinib

Location: China

FGFR3 K652E mutation (continued)

NCT03155620
NCI-COG Pediatric MATCH (Molecular Analysis for Therapy Choice) Screening Protocol

Cancer type: Unspecified Solid Tumor

Variant class: FGFR3 mutation

Other identifiers: 17-729, 2017-0625, 20170841, APEC1621SC, COGAPEC1621, COGAPEC1621SC, N 55017 SC, NCI-2017-01251, NCI-COG Pediatric MATCH, Pediatric MATCH

Population segments: ALK, Aggressive, BRAF, FGFR, HRAS, Indolent, KRAS, NRAS, Pediatric or Adolescent, Second line, Stage III, Stage IV

Phase: II

Therapy: erdafitinib

Locations: Puerto Rico, United States

US States: AK, AL, AR, AZ, CA, CO, CT, DC, DE, FL, GA, HI, IA, ID, IL, IN, KY, LA, MA, MD, ME, MI, MN, MO, MS, NC, NE, NH, NJ, NV, NY, OH, OK, OR, PA, RI, SC, SD, TN, TX, UT, VA, VT, WA, WI

Contact: Multiple contacts: See www.clinicaltrials.gov for complete list of contacts.

NCT03210714
NCI-COG Pediatric MATCH (Molecular Analysis for Therapy Choice) - Phase II Subprotocol of JNJ-42756493 (Erdafitinib) in Patients With Tumors Harboring FGFR 1/2/3/4 Alterations

Cancer type: Unspecified Solid Tumor

Variant class: FGFR3 mutation

Other identifiers: APEC1621B, NCI-2017-01159, NCI-COG Pediatric MATCH, Pediatric MATCH

Population segments: Aggressive, FGFR, Indolent, Pediatric or Adolescent, Second line, Stage III, Stage IV

Phase: II

Therapy: erdafitinib

Locations: Puerto Rico, United States

US States: AK, AL, AR, AZ, CA, CO, CT, DC, DE, FL, GA, IA, ID, IL, IN, KY, LA, MD, ME, MI, MN, MO, MS, NC, ND, NE, NJ, NY, OH, OK, OR, PA, SC, SD, TN, TX, UT, VA, VT, WA, WI

Contact: Multiple contacts: See www.clinicaltrials.gov for complete list of contacts.

No NCT ID - see other identifier(s)
A Multicenter Phase II Basket-type Clinical Trial to Evaluate Efficacy and Safety of TAS-120 in Patients with Advanced Solid Malignancies with FGFR Alterations in Circulating Tumor DNA

Cancer type: Unspecified Solid Tumor

Variant class: FGFR3 mutation

Other identifiers: EPOC 1805, JapicCTI-194624, JapicCTI-194796, TiFFANY

Population segments: Second line, Stage III, Stage IV

Phase: II

Therapy: futibatinib

Location: Japan

FGFR3 K652E mutation (continued)

NCT04233567
A Phase II Study of Oral Infigratinib in Adult Patients With Advanced or Metastatic Solid Tumors With FGFR1-3 Gene Fusions or Other FGFR Genetic Alterations

Cancer type: Unspecified Solid Tumor

Variant class: FGFR activating mutation

Other identifiers: NCI-2019-06869, OSU-19041

Population segments: Second line, Stage III, Stage IV

Phase: II

Therapy: infigratinib

Location: United States

US State: OH

Contact: The Ohio State University Comprehensive Cancer Center [800-293-5066; OSUCCCClinicaltrials@osumc.edu]

NCT02693535
Targeted Agent and Profiling Utilization Registry (TAPUR) Study.

Cancer type: Unspecified Solid Tumor

Variant class: FGFR3 mutation

Other identifiers: 20170529, NCI-2017-00510, Pro00014171, TAPUR

Population segments: Aggressive, BRAF, Diffuse large B-cell lymphoma (DLBCL), Extranodal marginal zone B-cell lymphoma (MALT), FGFR, First line, Follicular lymphoma (FL), Fourth line or greater, Indolent, Lymphoblastic lymphoma (LBL), Mantle cell lymphoma (MCL), Other subtype, Second line, Small lymphocytic lymphoma (SLL), Stage III, Stage IV, Third line, Waldenstrom's macroglobulinemia (WM)

Phase: II

Therapy: sunitinib

Location: United States

US States: AL, AZ, CA, FL, GA, HI, IL, IN, ME, MI, NC, ND, NE, NH, OH, OR, PA, SD, TX, UT, VA, WA

Contact: Pam Mangat [pam.mangat@asco.org]

NCT04591431
The Rome Trial From Histology to Target: the Road to Personalize Target Therapy and Immunotherapy

Cancer type: Biliary Tract Carcinoma, Breast Cancer, Colorectal Cancer, Esophageal Cancer, Gastric Cancer, Gastroesophageal Junction Adenocarcinoma, Hepatocellular Carcinoma, Liver Cancer, Non-Small Cell Lung Cancer, Pancreatic Cancer, Unspecified Solid Tumor

Variant class: FGFR3 aberration

Other identifiers: EudraCT Number: 2018-002190-21, MAR-BAS-18-005, ROME

Population segments: Second line, Stage IV

Phase: II

Therapy: pemigatinib

Location: Italy

Disclaimer: The data presented here is from a curated knowledgebase of publicly available information, but may not be exhaustive. The data version is 2021.11(003).

FGFR3 K652E mutation (continued)

NCT02691793
Study to Evaluate the Safety and Efficacy of Sunitinib, in Subject With Refractory Solid Tumors

Cancer type: Unspecified Solid Tumor

Variant class: FGFR mutation

Other identifier: 2016-02-098

Population segments: (N/A), FGFR, Second line

Phase: II

Therapy: sunitinib

Location: Republic of Korea

NCT03297606
Canadian Profiling and Targeted Agent Utilization Trial (CAPTUR): A Phase II Basket Trial

Cancer type: Unspecified Solid Tumor

Variant class: FGFR3 aberration

Other identifiers: CA209-9DL, CAPTUR, ESR-17-12831, ML39800, PM1, WI233446

Population segments: Aggressive, Diffuse large B-cell lymphoma (DLBCL), Extranodal marginal zone B-cell lymphoma (MALT), First line, Follicular lymphoma (FL), Indolent, Lymphoblastic lymphoma (LBL), Mantle cell lymphoma (MCL), Other subtype, Second line, Stage III, Stage IV, Waldenstrom's macroglobulinemia (WM)

Phase: II

Therapy: sunitinib

Location: Canada

NCT03929965
Anlotinib in Advanced Solid Tumors With FGFR Alteration

Cancer type: Unspecified Solid Tumor

Variant class: FGFR mutation

Other identifier: ASTFA

Population segments: Second line, Stage III, Stage IV, Third line

Phase: I

Therapy: anlotinib hydrochloride

Location: China

NCT04565275
A Multi-center Open-label, Phase I/II Clinical Trial to Evaluate the Safety, Tolerability, Pharmacokinetics, and Pharmacodynamics of ICP-192 in Patients With Advanced Solid Tumors and FGFR Gene Alterations.

Cancer type: Bladder Urothelial Carcinoma, Cholangiocarcinoma, Unspecified Solid Tumor

Variant class: FGFR aberration

Other identifier: ICP-CL-00303

Population segments: FGFR, Second line, Stage III, Stage IV

Phase: I/II

Therapy: ICP-192

Location: United States

US States: AZ, CO, FL, MN

Contact: Olivia Yang [609-524-0684; olivia.yang@INNOCAREPHARMA.COM]

Disclaimer: The data presented here is from a curated knowledgebase of publicly available information, but may not be exhaustive. The data version is 2021.11(003).

FGFR3 K652E mutation (continued)

No NCT ID - see other identifier(s)
Phase I/II Study of TAS-117 In Combination With TAS-120 In Patients With Advanced Solid Tumors

Cancer type: Unspecified Solid Tumor

Variant class: FGFR aberration

Other identifier: JapicCTI-194864

Population segments: FGFR, First line, Squamous Cell, Stage III, Stage IV

Phase: I/II

Therapies: TAS-117, futibatinib

Location: Japan

No NCT ID - see other identifier(s)
A Phase Ib Study to Assess the Safety, Tolerability, and Efficacy of TAS-120 (Futibatinib) in Combination with MK-3475 (Pembrolizumab) in Patients with Solid Tumors.

Cancer type: Unspecified Solid Tumor

Variant class: FGFR aberration

Other identifier: JapicCTI-195063

Population segments: FGFR, Line of therapy N/A, Stage III, Stage IV

Phase: I

Therapies: futibatinib, pembrolizumab

Location: Japan

NCT04936295
A Phase II Study of the Efficacy and Tolerability of Fulvestrant Plus Anlotinib in HR(+)/HER2(-) Metastatic Breast Cancer Patients With FGFR Mutation

Cancer type: Breast Cancer

Variant class: FGFR3 activating mutation

Other inclusion criteria: ERBB2 negative, Hormone receptor positive

Other identifier: SYSU005

Population segments: Estrogen receptor positive, FGFR, First line, HER2 negative, HER2 positive, Progesterone receptor positive, Stage IV

Phase: II

Therapies: hormone therapy, anlotinib hydrochloride

Location: China

NCT04096417
A Phase II, Multicenter, Single-Arm Study of Pemigatinib in Patients With Metastatic or Unresectable Colorectal Cancer Harboring FGFR Alterations

Cancer type: Colorectal Cancer

Variant class: FGFR3 activating mutation

Other identifiers: ACCRU-GI-1701, NCI-2019-05877, VICCGI2028

Population segments: FGFR, Second line, Stage III, Stage IV

Phase: II

Therapy: pemigatinib

Location: United States

US States: AZ, GA, MN, NC, TN

Contact: Multiple contacts: See www.clinicaltrials.gov for complete list of contacts.

Disclaimer: The data presented here is from a curated knowledgebase of publicly available information, but may not be exhaustive. The data version is 2021.11(003).

FGFR3 K652E mutation (continued)

NCT04045613
An Open-label Multi-cohort Phase Ib/II Study of Derazantinib and Atezolizumab in Patients With Urothelial Cancer Expressing Activating Molecular FGFR Aberrations.

Cancer type: Bladder Urothelial Carcinoma

Variant class: FGFR3 activating mutation

Other inclusion criteria: CD274 expression

Other identifiers: CSET 3012, DZB-CS-201, EudraCT Number: 2019-000359-15, FIDES-02, NCI-2019-08162, RECF4112, SNCTP000004480

Population segments: FGFR, Fourth line or greater, Second line, Stage IV, Third line

Phase: I/II

Therapies: derazantinib, atezolizumab

Locations: Australia, Austria, Belgium, Canada, Czech Republic, France, Germany, Hungary, Italy, Poland, Republic of Korea, Spain, Switzerland, United Kingdom, United States

US States: GA, IL, KS, KY, NY, SC, TX, VA, WA

Contact: Dr. Manuel HÃ¤ckl [manuel.haeckl@basilea.com]

NCT04053322
An International Multicenter Phase II Trial of Durvalumab (MEDI4736) Plus OLAparib Plus Fulvestrant in Metastatic or Locally Advanced ER-positive, HER2-negative Breast Cancer Patients Selected Using Criteria That Predict Sensitivity to Olaparib

Cancer type: Breast Cancer

Variant class: FGFR3 mutation

Other inclusion criteria: ERBB2 negative, ER positive

Other identifiers: CADUSEIME08, DOLAF, EudraCT Number: 2018-003832-57, UC-0140/1812

Population segments: BRCA, Estrogen receptor positive, FGFR, HER2 negative, Progesterone receptor positive, Second line, Stage III, Stage IV

Phase: II

Therapies: durvalumab, olaparib, hormone therapy

Location: France

NCT03827850
A Phase II Trial to Evaluate Efficacy and Safety of Erdafitinib in Patients With Advanced Squamous Non Small Cell Lung Carcinoma (sqNSCLC) Harboring Fibroblast Growth Factor Receptor (FGFR) Genetic Alterations After Relapse of Standard Therapy.

Cancer type: Non-Small Cell Lung Cancer

Variant class: FGFR activating mutation

Other identifiers: EudraCT Number: 2018-000399-13, FIND, FIND Trial, Uni-Koeln-3254

Population segments: FGFR, Second line, Squamous Cell, Stage III, Stage IV

Exclusion criteria variant classes: ALK aberration (Non-Small Cell Lung Cancer), BRAF aberration (Non-Small Cell Lung Cancer), EGFR aberration (Non-Small Cell Lung Cancer), NTRK aberration (Non-Small Cell Lung Cancer), ROS1 aberration (Non-Small Cell Lung Cancer)

Phase: II

Therapy: erdafitinib

Location: Germany

Disclaimer: The data presented here is from a curated knowledgebase of publicly available information, but may not be exhaustive. The data version is 2021.11(003).

FGFR3 K652E mutation (continued)

NCT04601857
A Phase II Study Evaluating Futibatinib (TAS 120) Plus Pembrolizumab in the Treatment of Advanced or Metastatic Urothelial Carcinoma

Cancer type: Bladder Urothelial Carcinoma

Variant class: FGFR3 mutation

Other identifiers: EudraCT Number: 2020-000945-15, TAS-120-203

Population segments: FGFR, First line, Stage III, Stage IV

Phase: II

Therapies: futibatinib, pembrolizumab

Location: United States

US State: MI

Contact: Dr. Maciej Gil [609-250-7336; clinicaltrialinfo@taihooncology.com]

NCT04294277
Open-label, Single-arm, Phase II Study, Evaluating Safety and Efficacy of INCB054828 (Pemigatinib) as Adjuvant Therapy for Molecularly-selected, High-risk Patients With Urothelial Carcinoma Who Have Received Radical Surgery

Cancer type: Bladder Urothelial Carcinoma

Variant class: FGFR3 mutation

Other identifiers: EAU RF 2018-02, EAURF2018-02, EudraCT Number: 2017-004426-15, EudraCT Number: 2019-001833-14, PEGASUS

Population segments: Adjuvant, FGFR, Stage III, Stage IV

Phase: II

Therapy: pemigatinib

Location: Italy

NCT04077255
Anti-EGFR Antibody (GC-1118) in Combination With Weekly Paclitaxel as a Second-line Therapy for Gastric and Gastroesophageal Junction Adenocarcinomas With Amplifications

Cancer type: Gastric Cancer, Gastroesophageal Junction Adenocarcinoma

Variant class: FGFR3 mutation

Other identifiers: 31973, KCT0004314, NCC2019-0196

Population segments: EGFR, FGFR, Second line, Stage IV

Phase: II

Therapies: rogaratinib, chemotherapy

Location: Republic of Korea

No NCT ID - see other identifier(s)
Phase I Clinical Study of BPI-17509 In Patients With Advanced Solid Tumors

Cancer type: Bladder Cancer, Solid Tumor

Variant class: FGFR3 mutation

Other identifiers: BTP-26811, CTR20192140

Population segments: Second line, Stage III, Stage IV

Phase: I

Therapy: BPI-17509

Location: China

FGFR3 K652E mutation (continued)

NCT04172675

A Randomized Phase II Study of Erdafitinib Versus Investigator Choice of Intravesical Chemotherapy in Subjects Who Received Bacillus Calmette-Guerin (BCG) and Recurred With High Risk Non-Muscle-Invasive Bladder Cancer (NMIBC) and FGFR Mutations or Fusions

Cancer type: Bladder Urothelial Carcinoma

Variant class: FGFR mutation

Other identifiers: 42756493BLC2003, 42756493BLC2003, CR108699, CTR20201841, CTRI/2021/08/036009, EudraCT Number: 2019-002449-39, JapicCTI-205145, NCI-2020-00674, RG1121102

Population segments: FGFR, Maintenance/Consolidation, Second line, Stage 0

Phase: II

Therapy: erdafitinib

Locations: Argentina, Australia, Belgium, Brazil, China, Czech Republic, France, Germany, Italy, Japan, Poland, Republic of Korea, Spain, Taiwan, United Kingdom, United States

US States: AZ, CA, CO, FL, GA, IL, KS, NC, NY, OH, PA, TN, TX, WA, WI

Contact: Study Contact [844-434-4210; JNJ.CT@sylogent.com]

NCT02150967

A Phase II Multicenter, Single Arm Study of Oral BGJ398 in Adult Patients with Advanced or Metastatic Cholangiocarcinoma with FGFR2 Gene Fusions or Other FGFR Genetic Alterations who Failed or are Intolerant to Platinum-based Chemotherapy.

Cancer type: Cholangiocarcinoma

Variant class: FGFR activating aberration

Other identifiers: 14-183, 14-2200, 14-328, 15452, 2014-0372, 2014-081, CBGJ398X2204, EudraCT Number: 2013-005085-19, NCI-2014-01310, REec-2014-1022, Trial 3GB-14-1

Population segments: FGFR, Fourth line or greater, Second line, Stage III, Stage IV, Third line

Phase: II

Therapy: infigratinib

Locations: Belgium, Germany, Singapore, Spain, Thailand, United Kingdom, United States

US States: CA, MA, MI, NY, OH, TX

Contact: QED Therapeutics [877-280-5655; clinicaltrials@QEDTx.com]

NCT04595747

Phase II Study of Rogaratinib (BAY 1163877) in the Treatment of Patients With Sarcoma Harboring Alterations in Fibroblast Growth Factor Receptor (FGFR) 1-4 and SDH-Deficient Gastrointestinal Stromal Tumor (GIST)

Cancer type: Soft Tissue Sarcoma

Variant class: FGFR3 aberration

Other identifiers: 10411, NCI-2020-08011

Population segments: FGFR, Second line, Stage III, Stage IV

Phase: II

Therapy: rogaratinib

Location: United States

US States: CA, MA, MD, NY

Contact: Multiple contacts: See www.clinicaltrials.gov for complete list of contacts.

Disclaimer: The data presented here is from a curated knowledgebase of publicly available information, but may not be exhaustive. The data version is 2021.11(003).

FGFR3 K652E mutation (continued)

NCT04004975
Phase II Clinical Trials on Anlotinib for the Treatment of Recurrent Glioblastoma

Cancer type: Glioblastoma

Variant class: FGFR mutation

Other identifier: ShandongCHI006

Population segments: (N/A), FGFR, Second line

Phase: I/II

Therapy: anlotinib hydrochloride

Location: China

NCT04149691
A Phase I, Open-label, Multicentre, Dose Escalation Study to Assess Safety, Tolerability and Pharmacokinetics of Oral CPL304110, in Adult Subjects With Advanced Solid Malignancies

Cancer type: Bladder Cancer, Gastric Cancer, Non-Small Cell Lung Cancer

Variant class: FGFR3 aberration

Other identifier: 01FGFR2018

Population segments: FGFR, Line of therapy N/A, Squamous Cell, Stage III, Stage IV

Phase: I

Therapy: CPL-304-110

Location: Poland

NCT03473743
A Phase Ib-II Study to Evaluate Safety, Efficacy, Pharmacokinetics, and Pharmacodynamics of Various Regimens of Erdafitinib in Subjects With Metastatic or Locally Advanced Urothelial Cancer

Cancer type: Bladder Urothelial Carcinoma

Variant class: FGFR aberration

Other identifiers: 17165, 2018-0142, 42756493BLC2002, CR108445, EudraCT Number: 2017-001980-19, NCI-2018-00997, NORSE, USO 17165, USOR 17165

Population segments: FGFR, First line, Fourth line or greater, Second line, Stage IV, Third line

Phase: I/II

Therapies: erdafitinib, cetrelimab

Locations: Belarus, Belgium, Brazil, France, Italy, Poland, Republic of Korea, Russian Federation, Spain, Taiwan, Turkey, United Kingdom, United States

US States: CO, MD, NC, NY, PA, TX, VA

Contact: Study Contact [844-434-4210; JNJ.CT@sylogent.com]

NCT02393248
A Phase I/II, Open-Label, Dose-Escalation, Safety and Tolerability Study of INCB054828 in Subjects With Advanced Malignancies - (FIGHT-101)

Cancer type: Bladder Urothelial Carcinoma, Breast Cancer, Cholangiocarcinoma, Endometrial Carcinoma, Gastric Cancer, Non-Small Cell Lung Cancer, Solid Tumor

Variant class: FGFR aberration

Other identifiers: 00061567, 2014-1099, 201503012, EudraCT Number: 2016-002831-14, FIGHT-101, INCB 54828-101, INCB54828-101, MC# 15-03, NCI-2015-00611, OSU-15241

Population segments: FGFR, First line, Fourth line or greater, Second line, Squamous Cell, Stage III, Stage IV, Third line

Phase: I/II

Therapies: pemigatinib, pembrolizumab, trastuzumab, chemotherapy, retifanlimab

Location: United States

US States: FL, NJ, TX

Contact: Incyte Call Center [855-463-3463]

Disclaimer: The data presented here is from a curated knowledgebase of publicly available information, but may not be exhaustive. The data version is 2021.11(003).

FGFR3 K652E mutation (continued)

NCT04572295

An Open-label Phase Ib Study of E7090 Monotherapy and in Combination With Other Anticancer Agents in Subjects With ER+, HER2- Recurrent/Metastatic Breast Cancer

Cancer type: Breast Cancer

Variant class: FGFR aberration

Other inclusion criteria: ERBB2 negative, ER positive

Other identifiers: E7090-J081-102, JapicCTI-205429

Population segments: Estrogen receptor positive, HER2 negative, Second line, Stage IV

Phase: I

Therapies: E-7090, hormone therapy

Location: Japan

NCT04504331

A Phase IB Study of Infigratinib in Combination With Tamoxifen in Hormone Receptor Positive, HER2 Negative, FGFR Altered Advanced Breast Cancer

Cancer type: Breast Cancer

Variant class: FGFR aberration

Other inclusion criteria: ERBB2 negative, Hormone receptor positive

Other identifiers: BRS0113, K08CA252457

Population segments: Estrogen receptor positive, HER2 negative, Progesterone receptor positive, Second line, Stage III, Stage IV, Triple receptor negative

Phase: I

Therapies: infigratinib, hormone therapy

Location: United States

US State: CA

Contact: Allison K. Zhang [650-736-5790; axkong@stanford.edu]

FGFR3 fusion

No NCT ID - see other identifier(s)

A Multicenter Phase II Basket-type Clinical Trial to Evaluate Efficacy and Safety of TAS-120 in Patients with Advanced Solid Malignancies with FGFR Alterations in Circulating Tumor DNA

Cancer type: Unspecified Solid Tumor

Variant class: FGFR3 fusion

Other identifiers: EPOC 1805, JapicCTI-194624, JapicCTI-194796, TiFFANY

Population segments: Second line, Stage III, Stage IV

Phase: II

Therapy: futibatinib

Location: Japan

Disclaimer: The data presented here is from a curated knowledgebase of publicly available information, but may not be exhaustive. The data version is 2021.11(003).

FGFR3 fusion (continued)

NCT04189445
A Phase II Study of Futibatinib in Patients With Specific FGFR Aberrations

Cancer type: Unspecified Solid Tumor

Variant class: FGFR3 fusion

Other identifiers: EudraCT Number: 2019-004084-49, JapicCTI-205312, NCI-2020-03407, NL73545.056.20, TAS-120-202, UW20046

Population segments: (N/A), Aggressive, Classical, FGFR, Indolent, Nodular lymphocyte-predominant, Second line, Stage III, Stage IV, Third line

Exclusion criteria variant classes: FGFR1 fusion (Central Nervous System Neoplasm, Intrahepatic Cholangiocarcinoma), FGFR2 fusion (Central Nervous System Neoplasm, Intrahepatic Cholangiocarcinoma), FGFR3 fusion (Central Nervous System Neoplasm, Intrahepatic Cholangiocarcinoma), FGFR4 fusion (Central Nervous System Neoplasm, Intrahepatic Cholangiocarcinoma)

Phase: II

Therapy: futibatinib

Locations: Japan, Republic of Korea, United Kingdom, United States

US States: AZ, CA, TX, WI

Contact: Dr. Osamu Takahashi [609-250-7336; clinicaltrialinfo@taihooncology.com]

NCT04233567
A Phase II Study of Oral Infigratinib in Adult Patients With Advanced or Metastatic Solid Tumors With FGFR1-3 Gene Fusions or Other FGFR Genetic Alterations

Cancer type: Unspecified Solid Tumor

Variant class: FGFR3 fusion

Other identifiers: NCI-2019-06869, OSU-19041

Population segments: Second line, Stage III, Stage IV

Phase: II

Therapy: infigratinib

Location: United States

US State: OH

Contact: The Ohio State University Comprehensive Cancer Center [800-293-5066; OSUCCCClinicaltrials@osumc.edu]

NCT05019794
A Phase IIa of Infigratinib in Subjects With Locally Advanced or Metastatic Gastric Cancer or Gastroesophageal Junction Adenocarcinoma With FGFR2 Amplification or Other Advanced Solid Tumors With Other FGFR Alterations

Cancer type: Gastric Cancer, Gastroesophageal Junction Adenocarcinoma, Unspecified Solid Tumor

Variant class: FGFR3 fusion

Other identifiers: CTR20210275, LB1001-201

Population segments: FGFR, Fourth line or greater, Second line, Stage III, Stage IV, Third line

Phase: II

Therapy: infigratinib

Location: China

FGFR3 fusion (continued)

NCT03929965
Anlotinib in Advanced Solid Tumors With FGFR Alteration

Cancer type: Unspecified Solid Tumor

Variant class: FGFR fusion

Other identifier: ASTFA

Population segments: Second line, Stage III, Stage IV, Third line

Phase: I

Therapy: anlotinib hydrochloride

Location: China

NCT04591431
The Rome Trial From Histology to Target: the Road to Personalize Target Therapy and Immunotherapy

Cancer type: Biliary Tract Carcinoma, Breast Cancer, Colorectal Cancer, Esophageal Cancer, Gastric Cancer, Gastroesophageal Junction Adenocarcinoma, Hepatocellular Carcinoma, Liver Cancer, Non-Small Cell Lung Cancer, Pancreatic Cancer, Unspecified Solid Tumor

Variant class: FGFR3 aberration

Other identifiers: EudraCT Number: 2018-002190-21, MAR-BAS-18-005, ROME

Population segments: Second line, Stage IV

Phase: II

Therapy: pemigatinib

Location: Italy

NCT03297606
Canadian Profiling and Targeted Agent Utilization Trial (CAPTUR): A Phase II Basket Trial

Cancer type: Unspecified Solid Tumor

Variant class: FGFR3 aberration

Other identifiers: CA209-9DL, CAPTUR, ESR-17-12831, ML39800, PM1, WI233446

Population segments: Aggressive, Diffuse large B-cell lymphoma (DLBCL), Extranodal marginal zone B-cell lymphoma (MALT), First line, Follicular lymphoma (FL), Indolent, Lymphoblastic lymphoma (LBL), Mantle cell lymphoma (MCL), Other subtype, Second line, Stage III, Stage IV, Waldenstrom's macroglobulinemia (WM)

Phase: II

Therapy: sunitinib

Location: Canada

NCT04565275
A Multi-center Open-label, Phase I/II Clinical Trial to Evaluate the Safety, Tolerability, Pharmacokinetics, and Pharmacodynamics of ICP-192 in Patients With Advanced Solid Tumors and FGFR Gene Alterations.

Cancer type: Bladder Urothelial Carcinoma, Cholangiocarcinoma, Unspecified Solid Tumor

Variant class: FGFR aberration

Other identifier: ICP-CL-00303

Population segments: FGFR, Second line, Stage III, Stage IV

Phase: I/II

Therapy: ICP-192

Location: United States

US States: AZ, CO, FL, MN

Contact: Olivia Yang [609-524-0684; olivia.yang@INNOCAREPHARMA.COM]

Disclaimer: The data presented here is from a curated knowledgebase of publicly available information, but may not be exhaustive. The data version is 2021.11(003).

FGFR3 fusion (continued)

No NCT ID - see other identifier(s)
Phase I/II Study of TAS-117 In Combination With TAS-120 In Patients With Advanced Solid Tumors

Cancer type: Unspecified Solid Tumor

Variant class: FGFR aberration

Other identifier: JapicCTI-194864

Population segments: FGFR, First line, Squamous Cell, Stage III, Stage IV

Phase: I/II

Therapies: TAS-117, futibatinib

Location: Japan

No NCT ID - see other identifier(s)
A Phase Ib Study to Assess the Safety, Tolerability, and Efficacy of TAS-120 (Futibatinib) in Combination with MK-3475 (Pembrolizumab) in Patients with Solid Tumors.

Cancer type: Unspecified Solid Tumor

Variant class: FGFR aberration

Other identifier: JapicCTI-195063

Population segments: FGFR, Line of therapy N/A, Stage III, Stage IV

Phase: I

Therapies: futibatinib, pembrolizumab

Location: Japan

NCT04197986
Phase III, Multicenter, Double-Blind, Randomized, Placebo-Controlled Trial of Infigratinib for the Adjuvant Treatment of Subjects With Invasive Urothelial Carcinoma With Susceptible FGFR3 Genetic Alterations (PROOF 302).

Cancer type: Bladder Urothelial Carcinoma

Variant class: FGFR3 fusion

Other identifiers: EudraCT Number: 2019-003248-63, NCI-2020-00610, PROOF 302, QBGJ398-302

Population segments: Adjuvant, FGFR, Stage II, Stage III

Exclusion criteria variant classes: FGFR3 A391E mutation (Bladder Small Cell Neuroendocrine Carcinoma), FGFR3 G370C mutation (Bladder Small Cell Neuroendocrine Carcinoma), FGFR3 K650E mutation (Bladder Small Cell Neuroendocrine Carcinoma), FGFR3 K650M mutation (Bladder Small Cell Neuroendocrine Carcinoma), FGFR3 K650Q mutation (Bladder Small Cell Neuroendocrine Carcinoma), FGFR3 K650T mutation (Bladder Small Cell Neuroendocrine Carcinoma), FGFR3 R248C mutation (Bladder Small Cell Neuroendocrine Carcinoma), FGFR3 S249C mutation (Bladder Small Cell Neuroendocrine Carcinoma), FGFR3 Y373C mutation (Bladder Small Cell Neuroendocrine Carcinoma), FGFR3 fusion (Bladder Small Cell Neuroendocrine Carcinoma)

Phase: III

Therapy: infigratinib

Locations: Belgium, Canada, France, Germany, Italy, Netherlands, Puerto Rico, Spain, United Kingdom, United States

US States: AL, AZ, CA, CO, DC, FL, GA, IL, IN, LA, MA, MD, MO, NC, NE, NJ, NY, OH, OK, SC, TN, TX, UT, VA, WA, WV

Contact: QED Therapeutics [877-280-5655; Proof302.ct@qedtx.com]

Disclaimer: The data presented here is from a curated knowledgebase of publicly available information, but may not be exhaustive. The data version is 2021.11(003).

FGFR3 fusion (continued)

NCT04601857
A Phase II Study Evaluating Futibatinib (TAS 120) Plus Pembrolizumab in the Treatment of Advanced or Metastatic Urothelial Carcinoma

Cancer type: Bladder Urothelial Carcinoma

Variant class: FGFR3 fusion

Other identifiers: EudraCT Number: 2020-000945-15, TAS-120-203

Population segments: FGFR, First line, Stage III, Stage IV

Phase: II

Therapies: futibatinib, pembrolizumab

Location: United States

US State: MI

Contact: Dr. Maciej Gil [609-250-7336; clinicaltrialinfo@taihooncology.com]

NCT04936295
A Phase II Study of the Efficacy and Tolerability of Fulvestrant Plus Anlotinib in HR(+)/HER2(-) Metastatic Breast Cancer Patients With FGFR Mutation

Cancer type: Breast Cancer

Variant class: FGFR3 fusion

Other inclusion criteria: ERBB2 negative, Hormone receptor positive

Other identifier: SYSU005

Population segments: Estrogen receptor positive, FGFR, First line, HER2 negative, HER2 positive, Progesterone receptor positive, Stage IV

Phase: II

Therapies: hormone therapy, anlotinib hydrochloride

Location: China

NCT04096417
A Phase II, Multicenter, Single-Arm Study of Pemigatinib in Patients With Metastatic or Unresectable Colorectal Cancer Harboring FGFR Alterations

Cancer type: Colorectal Cancer

Variant class: FGFR3 fusion

Other identifiers: ACCRU-GI-1701, NCI-2019-05877, VICCGI2028

Population segments: FGFR, Second line, Stage III, Stage IV

Phase: II

Therapy: pemigatinib

Location: United States

US States: AZ, GA, MN, NC, TN

Contact: Multiple contacts: See www.clinicaltrials.gov for complete list of contacts.

NCT04294277
Open-label, Single-arm, Phase II Study, Evaluating Safety and Efficacy of INCB054828 (Pemigatinib) as Adjuvant Therapy for Molecularly-selected, High-risk Patients With Urothelial Carcinoma Who Have Received Radical Surgery

Cancer type: Bladder Urothelial Carcinoma

Variant class: FGFR3 fusion

Other identifiers: EAU RF 2018-02, EAURF2018-02, EudraCT Number: 2017-004426-15, EudraCT Number: 2019-001833-14, PEGASUS

Population segments: Adjuvant, FGFR, Stage III, Stage IV

Phase: II

Therapy: pemigatinib

Location: Italy

Disclaimer: The data presented here is from a curated knowledgebase of publicly available information, but may not be exhaustive. The data version is 2021.11(003).

FGFR3 fusion (continued)

NCT04077255
Anti-EGFR Antibody (GC-1118) in Combination With Weekly Paclitaxel as a Second-line Therapy for Gastric and Gastroesophageal Junction Adenocarcinomas With Amplifications

Cancer type: Gastric Cancer, Gastroesophageal Junction Adenocarcinoma

Variant class: FGFR3 fusion

Other identifiers: 31973, KCT0004314, NCC2019-0196

Population segments: EGFR, FGFR, Second line, Stage IV

Phase: II

Therapies: rogaratinib, chemotherapy

Location: Republic of Korea

NCT04045613
An Open-label Multi-cohort Phase Ib/II Study of Derazantinib and Atezolizumab in Patients With Urothelial Cancer Expressing Activating Molecular FGFR Aberrations.

Cancer type: Bladder Urothelial Carcinoma

Variant class: FGFR3 fusion

Other inclusion criteria: CD274 expression

Other identifiers: CSET 3012, DZB-CS-201, EudraCT Number: 2019-000359-15, FIDES-02, NCI-2019-08162, RECF4112, SNCTP000004480

Population segments: FGFR, Fourth line or greater, Second line, Stage IV, Third line

Phase: I/II

Therapies: derazantinib, atezolizumab

Locations: Australia, Austria, Belgium, Canada, Czech Republic, France, Germany, Hungary, Italy, Poland, Republic of Korea, Spain, Switzerland, United Kingdom, United States

US States: GA, IL, KS, KY, NY, SC, TX, VA, WA

Contact: Dr. Manuel HÃ¤ckl [manuel.haeckl@basilea.com]

No NCT ID - see other identifier(s)
Phase I Clinical Study of BPI-17509 In Patients With Advanced Solid Tumors

Cancer type: Bladder Cancer, Solid Tumor

Variant class: FGFR3 fusion

Other identifiers: BTP-26811, CTR20192140

Population segments: Second line, Stage III, Stage IV

Phase: I

Therapy: BPI-17509

Location: China

NCT03827850
A Phase II Trial to Evaluate Efficacy and Safety of Erdafitinib in Patients With Advanced Squamous Non Small Cell Lung Carcinoma (sqNSCLC) Harboring Fibroblast Growth Factor Receptor (FGFR) Genetic Alterations After Relapse of Standard Therapy.

Cancer type: Non-Small Cell Lung Cancer

Variant class: FGFR fusion

Other identifiers: EudraCT Number: 2018-000399-13, FIND, FIND Trial, Uni-Koeln-3254

Population segments: FGFR, Second line, Squamous Cell, Stage III, Stage IV

Exclusion criteria variant classes: ALK aberration (Non-Small Cell Lung Cancer), BRAF aberration (Non-Small Cell Lung Cancer), EGFR aberration (Non-Small Cell Lung Cancer), NTRK aberration (Non-Small Cell Lung Cancer), ROS1 aberration (Non-Small Cell Lung Cancer)

Phase: II

Therapy: erdafitinib

Location: Germany

Disclaimer: The data presented here is from a curated knowledgebase of publicly available information, but may not be exhaustive. The data version is 2021.11(003).

FGFR3 fusion (continued)

NCT04172675
A Randomized Phase II Study of Erdafitinib Versus Investigator Choice of Intravesical Chemotherapy in Subjects Who Received Bacillus Calmette-Guerin (BCG) and Recurred With High Risk Non-Muscle-Invasive Bladder Cancer (NMIBC) and FGFR Mutations or Fusions

Cancer type: Bladder Urothelial Carcinoma

Variant class: FGFR fusion

Other identifiers: 42756493BLC2003, 42756493BLC2003, CR108699, CTR20201841, CTRI/2021/08/036009, EudraCT Number: 2019-002449-39, JapicCTI-205145, NCI-2020-00674, RG1121102

Population segments: FGFR, Maintenance/Consolidation, Second line, Stage 0

Phase: II

Therapy: erdafitinib

Locations: Argentina, Australia, Belgium, Brazil, China, Czech Republic, France, Germany, Italy, Japan, Poland, Republic of Korea, Spain, Taiwan, United Kingdom, United States

US States: AZ, CA, CO, FL, GA, IL, KS, NC, NY, OH, PA, TN, TX, WA, WI

Contact: Study Contact [844-434-4210; JNJ.CT@sylogent.com]

NCT02150967
A Phase II Multicenter, Single Arm Study of Oral BGJ398 in Adult Patients with Advanced or Metastatic Cholangiocarcinoma with FGFR2 Gene Fusions or Other FGFR Genetic Alterations who Failed or are Intolerant to Platinum-based Chemotherapy.

Cancer type: Cholangiocarcinoma

Variant class: FGFR activating aberration

Other identifiers: 14-183, 14-2200, 14-328, 15452, 2014-0372, 2014-081, CBGJ398X2204, EudraCT Number: 2013-005085-19, NCI-2014-01310, REec-2014-1022, Trial 3GB-14-1

Population segments: FGFR, Fourth line or greater, Second line, Stage III, Stage IV, Third line

Phase: II

Therapy: infigratinib

Locations: Belgium, Germany, Singapore, Spain, Thailand, United Kingdom, United States

US States: CA, MA, MI, NY, OH, TX

Contact: QED Therapeutics [877-280-5655; clinicaltrials@QEDTx.com]

NCT04595747
Phase II Study of Rogaratinib (BAY 1163877) in the Treatment of Patients With Sarcoma Harboring Alterations in Fibroblast Growth Factor Receptor (FGFR) 1-4 and SDH-Deficient Gastrointestinal Stromal Tumor (GIST)

Cancer type: Soft Tissue Sarcoma

Variant class: FGFR3 aberration

Other identifiers: 10411, NCI-2020-08011

Population segments: FGFR, Second line, Stage III, Stage IV

Phase: II

Therapy: rogaratinib

Location: United States

US States: CA, MA, MD, NY

Contact: Multiple contacts: See www.clinicaltrials.gov for complete list of contacts.

Disclaimer: The data presented here is from a curated knowledgebase of publicly available information, but may not be exhaustive. The data version is 2021.11(003).

FGFR3 fusion (continued)

NCT04149691

A Phase I, Open-label, Multicentre, Dose Escalation Study to Assess Safety, Tolerability and Pharmacokinetics of Oral CPL304110, in Adult Subjects With Advanced Solid Malignancies

Cancer type: Bladder Cancer, Gastric Cancer, Non-Small Cell Lung Cancer

Variant class: FGFR3 aberration

Other identifier: 01FGFR2018

Population segments: FGFR, Line of therapy N/A, Squamous Cell, Stage III, Stage IV

Phase: I

Therapy: CPL-304-110

Location: Poland

NCT03473743

A Phase Ib-II Study to Evaluate Safety, Efficacy, Pharmacokinetics, and Pharmacodynamics of Various Regimens of Erdafitinib in Subjects With Metastatic or Locally Advanced Urothelial Cancer

Cancer type: Bladder Urothelial Carcinoma

Variant class: FGFR aberration

Other identifiers: 17165, 2018-0142, 42756493BLC2002, CR108445, EudraCT Number: 2017-001980-19, NCI-2018-00997, NORSE, USO 17165, USOR 17165

Population segments: FGFR, First line, Fourth line or greater, Second line, Stage IV, Third line

Phase: I/II

Therapies: erdafitinib, cetrelimab

Locations: Belarus, Belgium, Brazil, France, Italy, Poland, Republic of Korea, Russian Federation, Spain, Taiwan, Turkey, United Kingdom, United States

US States: CO, MD, NC, NY, PA, TX, VA

Contact: Study Contact [844-434-4210; JNJ.CT@sylogent.com]

NCT02393248

A Phase I/II, Open-Label, Dose-Escalation, Safety and Tolerability Study of INCB054828 in Subjects With Advanced Malignancies - (FIGHT-101)

Cancer type: Bladder Urothelial Carcinoma, Breast Cancer, Cholangiocarcinoma, Endometrial Carcinoma, Gastric Cancer, Non-Small Cell Lung Cancer, Solid Tumor

Variant class: FGFR aberration

Other identifiers: 00061567, 2014-1099, 201503012, EudraCT Number: 2016-002831-14, FIGHT-101, INCB 54828-101, INCB54828-101, MC# 15-03, NCI-2015-00611, OSU-15241

Population segments: FGFR, First line, Fourth line or greater, Second line, Squamous Cell, Stage III, Stage IV, Third line

Phase: I/II

Therapies: pemigatinib, pembrolizumab, trastuzumab, chemotherapy, retifanlimab

Location: United States

US States: FL, NJ, TX

Contact: Incyte Call Center [855-463-3463]

FGFR3 fusion (continued)

NCT04572295

An Open-label Phase Ib Study of E7090 Monotherapy and in Combination With Other Anticancer Agents in Subjects With ER+, HER2- Recurrent/Metastatic Breast Cancer

Cancer type: Breast Cancer

Variant class: FGFR aberration

Other inclusion criteria: ERBB2 negative, ER positive

Other identifiers: E7090-J081-102, JapicCTI-205429

Population segments: Estrogen receptor positive, HER2 negative, Second line, Stage IV

Phase: I

Therapies: E-7090, hormone therapy

Location: Japan

NCT04504331

A Phase IB Study of Infigratinib in Combination With Tamoxifen in Hormone Receptor Positive, HER2 Negative, FGFR Altered Advanced Breast Cancer

Cancer type: Breast Cancer

Variant class: FGFR aberration

Other inclusion criteria: ERBB2 negative, Hormone receptor positive

Other identifiers: BRS0113, K08CA252457

Population segments: Estrogen receptor positive, HER2 negative, Progesterone receptor positive, Second line, Stage III, Stage IV, Triple receptor negative

Phase: I

Therapies: infigratinib, hormone therapy

Location: United States

US State: CA

Contact: Allison K. Zhang [650-736-5790; axkong@stanford.edu]

FGFR3 A391E mutation

NCT03155620

NCI-COG Pediatric MATCH (Molecular Analysis for Therapy Choice) Screening Protocol

Cancer type: Unspecified Solid Tumor

Variant class: FGFR3 mutation

Other identifiers: 17-729, 2017-0625, 20170841, APEC1621SC, COGAPEC1621, COGAPEC1621SC, N 55017 SC, NCI-2017-01251, NCI-COG Pediatric MATCH, Pediatric MATCH

Population segments: ALK, Aggressive, BRAF, FGFR, HRAS, Indolent, KRAS, NRAS, Pediatric or Adolescent, Second line, Stage III, Stage IV

Phase: II

Therapy: erdafitinib

Locations: Puerto Rico, United States

US States: AK, AL, AR, AZ, CA, CO, CT, DC, DE, FL, GA, HI, IA, ID, IL, IN, KY, LA, MA, MD, ME, MI, MN, MO, MS, NC, NE, NH, NJ, NV, NY, OH, OK, OR, PA, RI, SC, SD, TN, TX, UT, VA, VT, WA, WI

Contact: Multiple contacts: See www.clinicaltrials.gov for complete list of contacts.

Disclaimer: The data presented here is from a curated knowledgebase of publicly available information, but may not be exhaustive. The data version is 2021.11(003).

FGFR3 A391E mutation (continued)

NCT03210714
NCI-COG Pediatric MATCH (Molecular Analysis for Therapy Choice) - Phase II Subprotocol of JNJ-42756493 (Erdafitinib) in Patients With Tumors Harboring FGFR 1/2/3/4 Alterations

Cancer type: Unspecified Solid Tumor

Variant class: FGFR3 mutation

Other identifiers: APEC1621B, NCI-2017-01159, NCI-COG Pediatric MATCH, Pediatric MATCH

Population segments: Aggressive, FGFR, Indolent, Pediatric or Adolescent, Second line, Stage III, Stage IV

Phase: II

Therapy: erdafitinib

Locations: Puerto Rico, United States

US States: AK, AL, AR, AZ, CA, CO, CT, DC, DE, FL, GA, IA, ID, IL, IN, KY, LA, MD, ME, MI, MN, MO, MS, NC, ND, NE, NJ, NY, OH, OK, OR, PA, SC, SD, TN, TX, UT, VA, VT, WA, WI

Contact: Multiple contacts: See www.clinicaltrials.gov for complete list of contacts.

No NCT ID - see other identifier(s)
A Multicenter Phase II Basket-type Clinical Trial to Evaluate Efficacy and Safety of TAS-120 in Patients with Advanced Solid Malignancies with FGFR Alterations in Circulating Tumor DNA

Cancer type: Unspecified Solid Tumor

Variant class: FGFR3 mutation

Other identifiers: EPOC 1805, JapicCTI-194624, JapicCTI-194796, TiFFANY

Population segments: Second line, Stage III, Stage IV

Phase: II

Therapy: futibatinib

Location: Japan

NCT02693535
Targeted Agent and Profiling Utilization Registry (TAPUR) Study.

Cancer type: Unspecified Solid Tumor

Variant class: FGFR3 mutation

Other identifiers: 20170529, NCI-2017-00510, Pro00014171, TAPUR

Population segments: Aggressive, BRAF, Diffuse large B-cell lymphoma (DLBCL), Extranodal marginal zone B-cell lymphoma (MALT), FGFR, First line, Follicular lymphoma (FL), Fourth line or greater, Indolent, Lymphoblastic lymphoma (LBL), Mantle cell lymphoma (MCL), Other subtype, Second line, Small lymphocytic lymphoma (SLL), Stage III, Stage IV, Third line, Waldenstrom's macroglobulinemia (WM)

Phase: II

Therapy: sunitinib

Location: United States

US States: AL, AZ, CA, FL, GA, HI, IL, IN, ME, MI, NC, ND, NE, NH, OH, OR, PA, SD, TX, UT, VA, WA

Contact: Pam Mangat [pam.mangat@asco.org]

Disclaimer: The data presented here is from a curated knowledgebase of publicly available information, but may not be exhaustive. The data version is 2021.11(003).

FGFR3 A391E mutation (continued)

NCT04591431
The Rome Trial From Histology to Target: the Road to Personalize Target Therapy and Immunotherapy

Cancer type: Biliary Tract Carcinoma, Breast Cancer, Colorectal Cancer, Esophageal Cancer, Gastric Cancer, Gastroesophageal Junction Adenocarcinoma, Hepatocellular Carcinoma, Liver Cancer, Non-Small Cell Lung Cancer, Pancreatic Cancer, Unspecified Solid Tumor

Variant class: FGFR3 aberration

Other identifiers: EudraCT Number: 2018-002190-21, MAR-BAS-18-005, ROME

Population segments: Second line, Stage IV

Phase: II

Therapy: pemigatinib

Location: Italy

NCT02691793
Study to Evaluate the Safety and Efficacy of Sunitinib, in Subject With Refractory Solid Tumors

Cancer type: Unspecified Solid Tumor

Variant class: FGFR mutation

Other identifier: 2016-02-098

Population segments: (N/A), FGFR, Second line

Phase: II

Therapy: sunitinib

Location: Republic of Korea

NCT03297606
Canadian Profiling and Targeted Agent Utilization Trial (CAPTUR): A Phase II Basket Trial

Cancer type: Unspecified Solid Tumor

Variant class: FGFR3 aberration

Other identifiers: CA209-9DL, CAPTUR, ESR-17-12831, ML39800, PM1, WI233446

Population segments: Aggressive, Diffuse large B-cell lymphoma (DLBCL), Extranodal marginal zone B-cell lymphoma (MALT), First line, Follicular lymphoma (FL), Indolent, Lymphoblastic lymphoma (LBL), Mantle cell lymphoma (MCL), Other subtype, Second line, Stage III, Stage IV, Waldenstrom's macroglobulinemia (WM)

Phase: II

Therapy: sunitinib

Location: Canada

NCT03929965
Anlotinib in Advanced Solid Tumors With FGFR Alteration

Cancer type: Unspecified Solid Tumor

Variant class: FGFR mutation

Other identifier: ASTFA

Population segments: Second line, Stage III, Stage IV, Third line

Phase: I

Therapy: anlotinib hydrochloride

Location: China

FGFR3 A391E mutation (continued)

NCT04565275
A Multi-center Open-label, Phase I/II Clinical Trial to Evaluate the Safety, Tolerability, Pharmacokinetics, and Pharmacodynamics of ICP-192 in Patients With Advanced Solid Tumors and FGFR Gene Alterations.

Cancer type: Bladder Urothelial Carcinoma, Cholangiocarcinoma, Unspecified Solid Tumor

Variant class: FGFR aberration

Other identifier: ICP-CL-00303

Population segments: FGFR, Second line, Stage III, Stage IV

Phase: I/II

Therapy: ICP-192

Location: United States

US States: AZ, CO, FL, MN

Contact: Olivia Yang [609-524-0684; olivia.yang@INNOCAREPHARMA.COM]

No NCT ID - see other identifier(s)
Phase I/II Study of TAS-117 In Combination With TAS-120 In Patients With Advanced Solid Tumors

Cancer type: Unspecified Solid Tumor

Variant class: FGFR aberration

Other identifier: JapicCTI-194864

Population segments: FGFR, First line, Squamous Cell, Stage III, Stage IV

Phase: I/II

Therapies: TAS-117, futibatinib

Location: Japan

No NCT ID - see other identifier(s)
A Phase Ib Study to Assess the Safety, Tolerability, and Efficacy of TAS-120 (Futibatinib) in Combination with MK-3475 (Pembrolizumab) in Patients with Solid Tumors.

Cancer type: Unspecified Solid Tumor

Variant class: FGFR aberration

Other identifier: JapicCTI-195063

Population segments: FGFR, Line of therapy N/A, Stage III, Stage IV

Phase: I

Therapies: futibatinib, pembrolizumab

Location: Japan

Disclaimer: The data presented here is from a curated knowledgebase of publicly available information, but may not be exhaustive. The data version is 2021.11(003).

FGFR3 A391E mutation (continued)

NCT04197986

Phase III, Multicenter, Double-Blind, Randomized, Placebo-Controlled Trial of Infigratinib for the Adjuvant Treatment of Subjects With Invasive Urothelial Carcinoma With Susceptible FGFR3 Genetic Alterations (PROOF 302).

Cancer type: Bladder Urothelial Carcinoma

Variant class: FGFR3 A391E mutation

Other identifiers: EudraCT Number: 2019-003248-63, NCI-2020-00610, PROOF 302, QBGJ398-302

Population segments: Adjuvant, FGFR, Stage II, Stage III

Exclusion criteria variant classes: FGFR3 A391E mutation (Bladder Small Cell Neuroendocrine Carcinoma), FGFR3 G370C mutation (Bladder Small Cell Neuroendocrine Carcinoma), FGFR3 K650E mutation (Bladder Small Cell Neuroendocrine Carcinoma), FGFR3 K650M mutation (Bladder Small Cell Neuroendocrine Carcinoma), FGFR3 K650Q mutation (Bladder Small Cell Neuroendocrine Carcinoma), FGFR3 K650T mutation (Bladder Small Cell Neuroendocrine Carcinoma), FGFR3 R248C mutation (Bladder Small Cell Neuroendocrine Carcinoma), FGFR3 S249C mutation (Bladder Small Cell Neuroendocrine Carcinoma), FGFR3 Y373C mutation (Bladder Small Cell Neuroendocrine Carcinoma), FGFR3 fusion (Bladder Small Cell Neuroendocrine Carcinoma)

Phase: III

Therapy: infigratinib

Locations: Belgium, Canada, France, Germany, Italy, Netherlands, Puerto Rico, Spain, United Kingdom, United States

US States: AL, AZ, CA, CO, DC, FL, GA, IL, IN, LA, MA, MD, MO, NC, NE, NJ, NY, OH, OK, SC, TN, TX, UT, VA, WA, WV

Contact: QED Therapeutics [877-280-5655; Proof302.ct@qedtx.com]

NCT04053322

An International Multicenter Phase II Trial of Durvalumab (MEDI4736) Plus OLAparib Plus Fulvestrant in Metastatic or Locally Advanced ER-positive, HER2-negative Breast Cancer Patients Selected Using Criteria That Predict Sensitivity to Olaparib

Cancer type: Breast Cancer

Variant class: FGFR3 mutation

Other inclusion criteria: ERBB2 negative, ER positive

Other identifiers: CADUSEIME08, DOLAF, EudraCT Number: 2018-003832-57, UC-0140/1812

Population segments: BRCA, Estrogen receptor positive, FGFR, HER2 negative, Progesterone receptor positive, Second line, Stage III, Stage IV

Phase: II

Therapies: durvalumab, olaparib, hormone therapy

Location: France

NCT04601857

A Phase II Study Evaluating Futibatinib (TAS 120) Plus Pembrolizumab in the Treatment of Advanced or Metastatic Urothelial Carcinoma

Cancer type: Bladder Urothelial Carcinoma

Variant class: FGFR3 mutation

Other identifiers: EudraCT Number: 2020-000945-15, TAS-120-203

Population segments: FGFR, First line, Stage III, Stage IV

Phase: II

Therapies: futibatinib, pembrolizumab

Location: United States

US State: MI

Contact: Dr. Maciej Gil [609-250-7336; clinicaltrialinfo@taihooncology.com]

Disclaimer: The data presented here is from a curated knowledgebase of publicly available information, but may not be exhaustive. The data version is 2021.11(003).

FGFR3 A391E mutation (continued)

NCT04096417
A Phase II, Multicenter, Single-Arm Study of Pemigatinib in Patients With Metastatic or Unresectable Colorectal Cancer Harboring FGFR Alterations

Cancer type: Colorectal Cancer

Variant class: FGFR3 mutation

Other identifiers: ACCRU-GI-1701, NCI-2019-05877, VICCGI2028

Population segments: FGFR, Second line, Stage III, Stage IV

Phase: II

Therapy: pemigatinib

Location: United States

US States: AZ, GA, MN, NC, TN

Contact: Multiple contacts: See www.clinicaltrials.gov for complete list of contacts.

NCT04294277
Open-label, Single-arm, Phase II Study, Evaluating Safety and Efficacy of INCB054828 (Pemigatinib) as Adjuvant Therapy for Molecularly-selected, High-risk Patients With Urothelial Carcinoma Who Have Received Radical Surgery

Cancer type: Bladder Urothelial Carcinoma

Variant class: FGFR3 mutation

Other identifiers: EAU RF 2018-02, EAURF2018-02, EudraCT Number: 2017-004426-15, EudraCT Number: 2019-001833-14, PEGASUS

Population segments: Adjuvant, FGFR, Stage III, Stage IV

Phase: II

Therapy: pemigatinib

Location: Italy

NCT04077255
Anti-EGFR Antibody (GC-1118) in Combination With Weekly Paclitaxel as a Second-line Therapy for Gastric and Gastroesophageal Junction Adenocarcinomas With Amplifications

Cancer type: Gastric Cancer, Gastroesophageal Junction Adenocarcinoma

Variant class: FGFR3 mutation

Other identifiers: 31973, KCT0004314, NCC2019-0196

Population segments: EGFR, FGFR, Second line, Stage IV

Phase: II

Therapies: rogaratinib, chemotherapy

Location: Republic of Korea

No NCT ID - see other identifier(s)
Phase I Clinical Study of BPI-17509 In Patients With Advanced Solid Tumors

Cancer type: Bladder Cancer, Solid Tumor

Variant class: FGFR3 mutation

Other identifiers: BTP-26811, CTR20192140

Population segments: Second line, Stage III, Stage IV

Phase: I

Therapy: BPI-17509

Location: China

Disclaimer: The data presented here is from a curated knowledgebase of publicly available information, but may not be exhaustive. The data version is 2021.11(003).

FGFR3 A391E mutation (continued)

NCT04172675
A Randomized Phase II Study of Erdafitinib Versus Investigator Choice of Intravesical Chemotherapy in Subjects Who Received Bacillus Calmette-Guerin (BCG) and Recurred With High Risk Non-Muscle-Invasive Bladder Cancer (NMIBC) and FGFR Mutations or Fusions

Cancer type: Bladder Urothelial Carcinoma

Variant class: FGFR mutation

Other identifiers: 42756493BLC2003, 42756493BLC2003, CR108699, CTR20201841, CTRI/2021/08/036009, EudraCT Number: 2019-002449-39, JapicCTI-205145, NCI-2020-00674, RG1121102

Population segments: FGFR, Maintenance/Consolidation, Second line, Stage 0

Phase: II

Therapy: erdafitinib

Locations: Argentina, Australia, Belgium, Brazil, China, Czech Republic, France, Germany, Italy, Japan, Poland, Republic of Korea, Spain, Taiwan, United Kingdom, United States

US States: AZ, CA, CO, FL, GA, IL, KS, NC, NY, OH, PA, TN, TX, WA, WI

Contact: Study Contact [844-434-4210; JNJ.CT@sylogent.com]

NCT04595747
Phase II Study of Rogaratinib (BAY 1163877) in the Treatment of Patients With Sarcoma Harboring Alterations in Fibroblast Growth Factor Receptor (FGFR) 1-4 and SDH-Deficient Gastrointestinal Stromal Tumor (GIST)

Cancer type: Soft Tissue Sarcoma

Variant class: FGFR3 aberration

Other identifiers: 10411, NCI-2020-08011

Population segments: FGFR, Second line, Stage III, Stage IV

Phase: II

Therapy: rogaratinib

Location: United States

US States: CA, MA, MD, NY

Contact: Multiple contacts: See www.clinicaltrials.gov for complete list of contacts.

NCT04004975
Phase II Clinical Trials on Anlotinib for the Treatment of Recurrent Glioblastoma

Cancer type: Glioblastoma

Variant class: FGFR mutation

Other identifier: ShandongCHI006

Population segments: (N/A), FGFR, Second line

Phase: I/II

Therapy: anlotinib hydrochloride

Location: China

NCT04149691
A Phase I, Open-label, Multicentre, Dose Escalation Study to Assess Safety, Tolerability and Pharmacokinetics of Oral CPL304110, in Adult Subjects With Advanced Solid Malignancies

Cancer type: Bladder Cancer, Gastric Cancer, Non-Small Cell Lung Cancer

Variant class: FGFR3 aberration

Other identifier: 01FGFR2018

Population segments: FGFR, Line of therapy N/A, Squamous Cell, Stage III, Stage IV

Phase: I

Therapy: CPL-304-110

Location: Poland

FGFR3 A391E mutation (continued)

NCT03473743
A Phase Ib-II Study to Evaluate Safety, Efficacy, Pharmacokinetics, and Pharmacodynamics of Various Regimens of Erdafitinib in Subjects With Metastatic or Locally Advanced Urothelial Cancer

Cancer type: Bladder Urothelial Carcinoma

Variant class: FGFR aberration

Other identifiers: 17165, 2018-0142, 42756493BLC2002, CR108445, EudraCT Number: 2017-001980-19, NCI-2018-00997, NORSE, USO 17165, USOR 17165

Population segments: FGFR, First line, Fourth line or greater, Second line, Stage IV, Third line

Phase: I/II

Therapies: erdafitinib, cetrelimab

Locations: Belarus, Belgium, Brazil, France, Italy, Poland, Republic of Korea, Russian Federation, Spain, Taiwan, Turkey, United Kingdom, United States

US States: CO, MD, NC, NY, PA, TX, VA

Contact: Study Contact [844-434-4210; JNJ.CT@sylogent.com]

NCT02393248
A Phase I/II, Open-Label, Dose-Escalation, Safety and Tolerability Study of INCB054828 in Subjects With Advanced Malignancies - (FIGHT-101)

Cancer type: Bladder Urothelial Carcinoma, Breast Cancer, Cholangiocarcinoma, Endometrial Carcinoma, Gastric Cancer, Non-Small Cell Lung Cancer, Solid Tumor

Variant class: FGFR aberration

Other identifiers: 00061567, 2014-1099, 201503012, EudraCT Number: 2016-002831-14, FIGHT-101, INCB 54828-101, INCB54828-101, MC# 15-03, NCI-2015-00611, OSU-15241

Population segments: FGFR, First line, Fourth line or greater, Second line, Squamous Cell, Stage III, Stage IV, Third line

Phase: I/II

Therapies: pemigatinib, pembrolizumab, trastuzumab, chemotherapy, retifanlimab

Location: United States

US States: FL, NJ, TX

Contact: Incyte Call Center [855-463-3463]

NCT04572295
An Open-label Phase Ib Study of E7090 Monotherapy and in Combination With Other Anticancer Agents in Subjects With ER+, HER2- Recurrent/Metastatic Breast Cancer

Cancer type: Breast Cancer

Variant class: FGFR aberration

Other inclusion criteria: ERBB2 negative, ER positive

Other identifiers: E7090-J081-102, JapicCTI-205429

Population segments: Estrogen receptor positive, HER2 negative, Second line, Stage IV

Phase: I

Therapies: E-7090, hormone therapy

Location: Japan

Disclaimer: The data presented here is from a curated knowledgebase of publicly available information, but may not be exhaustive. The data version is 2021.11(003).

FGFR3 A391E mutation (continued)

NCT04504331
A Phase IB Study of Infigratinib in Combination With Tamoxifen in Hormone Receptor Positive, HER2 Negative, FGFR Altered Advanced Breast Cancer

Cancer type: Breast Cancer

Variant class: FGFR aberration

Other inclusion criteria: ERBB2 negative, Hormone receptor positive

Other identifiers: BRS0113, K08CA252457

Population segments: Estrogen receptor positive, HER2 negative, Progesterone receptor positive, Second line, Stage III, Stage IV, Triple receptor negative

Phase: I

Therapies: infigratinib, hormone therapy

Location: United States

US State: CA

Contact: Allison K. Zhang [650-736-5790; axkong@stanford.edu]

FGFR3 KD mutation

NCT03155620
NCI-COG Pediatric MATCH (Molecular Analysis for Therapy Choice) Screening Protocol

Cancer type: Unspecified Solid Tumor

Variant class: FGFR3 mutation

Other identifiers: 17-729, 2017-0625, 20170841, APEC1621SC, COGAPEC1621, COGAPEC1621SC, N 55017 SC, NCI-2017-01251, NCI-COG Pediatric MATCH, Pediatric MATCH

Population segments: ALK, Aggressive, BRAF, FGFR, HRAS, Indolent, KRAS, NRAS, Pediatric or Adolescent, Second line, Stage III, Stage IV

Phase: II

Therapy: erdafitinib

Locations: Puerto Rico, United States

US States: AK, AL, AR, AZ, CA, CO, CT, DC, DE, FL, GA, HI, IA, ID, IL, IN, KY, LA, MA, MD, ME, MI, MN, MO, MS, NC, NE, NH, NJ, NV, NY, OH, OK, OR, PA, RI, SC, SD, TN, TX, UT, VA, VT, WA, WI

Contact: Multiple contacts: See www.clinicaltrials.gov for complete list of contacts.

NCT03210714
NCI-COG Pediatric MATCH (Molecular Analysis for Therapy Choice) - Phase II Subprotocol of JNJ-42756493 (Erdafitinib) in Patients With Tumors Harboring FGFR 1/2/3/4 Alterations

Cancer type: Unspecified Solid Tumor

Variant class: FGFR3 mutation

Other identifiers: APEC1621B, NCI-2017-01159, NCI-COG Pediatric MATCH, Pediatric MATCH

Population segments: Aggressive, FGFR, Indolent, Pediatric or Adolescent, Second line, Stage III, Stage IV

Phase: II

Therapy: erdafitinib

Locations: Puerto Rico, United States

US States: AK, AL, AR, AZ, CA, CO, CT, DC, DE, FL, GA, IA, ID, IL, IN, KY, LA, MD, ME, MI, MN, MO, MS, NC, ND, NE, NJ, NY, OH, OK, OR, PA, SC, SD, TN, TX, UT, VA, VT, WA, WI

Contact: Multiple contacts: See www.clinicaltrials.gov for complete list of contacts.

Disclaimer: The data presented here is from a curated knowledgebase of publicly available information, but may not be exhaustive. The data version is 2021.11(003).

FGFR3 KD mutation (continued)

No NCT ID - see other identifier(s)
A Multicenter Phase II Basket-type Clinical Trial to Evaluate Efficacy and Safety of TAS-120 in Patients with Advanced Solid Malignancies with FGFR Alterations in Circulating Tumor DNA

Cancer type: Unspecified Solid Tumor

Variant class: FGFR3 mutation

Other identifiers: EPOC 1805, JapicCTI-194624, JapicCTI-194796, TiFFANY

Population segments: Second line, Stage III, Stage IV

Phase: II

Therapy: futibatinib

Location: Japan

NCT02693535
Targeted Agent and Profiling Utilization Registry (TAPUR) Study.

Cancer type: Unspecified Solid Tumor

Variant class: FGFR3 mutation

Other identifiers: 20170529, NCI-2017-00510, Pro00014171, TAPUR

Population segments: Aggressive, BRAF, Diffuse large B-cell lymphoma (DLBCL), Extranodal marginal zone B-cell lymphoma (MALT), FGFR, First line, Follicular lymphoma (FL), Fourth line or greater, Indolent, Lymphoblastic lymphoma (LBL), Mantle cell lymphoma (MCL), Other subtype, Second line, Small lymphocytic lymphoma (SLL), Stage III, Stage IV, Third line, Waldenstrom's macroglobulinemia (WM)

Phase: II

Therapy: sunitinib

Location: United States

US States: AL, AZ, CA, FL, GA, HI, IL, IN, ME, MI, NC, ND, NE, NH, OH, OR, PA, SD, TX, UT, VA, WA

Contact: Pam Mangat [pam.mangat@asco.org]

NCT04591431
The Rome Trial From Histology to Target: the Road to Personalize Target Therapy and Immunotherapy

Cancer type: Biliary Tract Carcinoma, Breast Cancer, Colorectal Cancer, Esophageal Cancer, Gastric Cancer, Gastroesophageal Junction Adenocarcinoma, Hepatocellular Carcinoma, Liver Cancer, Non-Small Cell Lung Cancer, Pancreatic Cancer, Unspecified Solid Tumor

Variant class: FGFR3 aberration

Other identifiers: EudraCT Number: 2018-002190-21, MAR-BAS-18-005, ROME

Population segments: Second line, Stage IV

Phase: II

Therapy: pemigatinib

Location: Italy

NCT02691793
Study to Evaluate the Safety and Efficacy of Sunitinib, in Subject With Refractory Solid Tumors

Cancer type: Unspecified Solid Tumor

Variant class: FGFR mutation

Other identifier: 2016-02-098

Population segments: (N/A), FGFR, Second line

Phase: II

Therapy: sunitinib

Location: Republic of Korea

Disclaimer: The data presented here is from a curated knowledgebase of publicly available information, but may not be exhaustive. The data version is 2021.11(003).

FGFR3 KD mutation (continued)

NCT03297606
Canadian Profiling and Targeted Agent Utilization Trial (CAPTUR): A Phase II Basket Trial

Cancer type: Unspecified Solid Tumor

Variant class: FGFR3 aberration

Other identifiers: CA209-9DL, CAPTUR, ESR-17-12831, ML39800, PM1, WI233446

Population segments: Aggressive, Diffuse large B-cell lymphoma (DLBCL), Extranodal marginal zone B-cell lymphoma (MALT), First line, Follicular lymphoma (FL), Indolent, Lymphoblastic lymphoma (LBL), Mantle cell lymphoma (MCL), Other subtype, Second line, Stage III, Stage IV, Waldenstrom's macroglobulinemia (WM)

Phase: II

Therapy: sunitinib

Location: Canada

NCT03929965
Anlotinib in Advanced Solid Tumors With FGFR Alteration

Cancer type: Unspecified Solid Tumor

Variant class: FGFR mutation

Other identifier: ASTFA

Population segments: Second line, Stage III, Stage IV, Third line

Phase: I

Therapy: anlotinib hydrochloride

Location: China

NCT04565275
A Multi-center Open-label, Phase I/II Clinical Trial to Evaluate the Safety, Tolerability, Pharmacokinetics, and Pharmacodynamics of ICP-192 in Patients With Advanced Solid Tumors and FGFR Gene Alterations.

Cancer type: Bladder Urothelial Carcinoma, Cholangiocarcinoma, Unspecified Solid Tumor

Variant class: FGFR aberration

Other identifier: ICP-CL-00303

Population segments: FGFR, Second line, Stage III, Stage IV

Phase: I/II

Therapy: ICP-192

Location: United States

US States: AZ, CO, FL, MN

Contact: Olivia Yang [609-524-0684; olivia.yang@INNOCAREPHARMA.COM]

No NCT ID - see other identifier(s)
Phase I/II Study of TAS-117 In Combination With TAS-120 In Patients With Advanced Solid Tumors

Cancer type: Unspecified Solid Tumor

Variant class: FGFR aberration

Other identifier: JapicCTI-194864

Population segments: FGFR, First line, Squamous Cell, Stage III, Stage IV

Phase: I/II

Therapies: TAS-117, futibatinib

Location: Japan

Disclaimer: The data presented here is from a curated knowledgebase of publicly available information, but may not be exhaustive. The data version is 2021.11(003).

FGFR3 KD mutation (continued)

No NCT ID - see other identifier(s)
A Phase Ib Study to Assess the Safety, Tolerability, and Efficacy of TAS-120 (Futibatinib) in Combination with MK-3475 (Pembrolizumab) in Patients with Solid Tumors.

Cancer type: Unspecified Solid Tumor

Variant class: FGFR aberration

Other identifier: JapicCTI-195063

Population segments: FGFR, Line of therapy N/A, Stage III, Stage IV

Phase: I

Therapies: futibatinib, pembrolizumab

Location: Japan

NCT04053322
An International Multicenter Phase II Trial of Durvalumab (MEDI4736) Plus OLAparib Plus Fulvestrant in Metastatic or Locally Advanced ER-positive, HER2-negative Breast Cancer Patients Selected Using Criteria That Predict Sensitivity to Olaparib

Cancer type: Breast Cancer

Variant class: FGFR3 mutation

Other inclusion criteria: ERBB2 negative, ER positive

Other identifiers: CADUSEIME08, DOLAF, EudraCT Number: 2018-003832-57, UC-0140/1812

Population segments: BRCA, Estrogen receptor positive, FGFR, HER2 negative, Progesterone receptor positive, Second line, Stage III, Stage IV

Phase: II

Therapies: durvalumab, olaparib, hormone therapy

Location: France

NCT04601857
A Phase II Study Evaluating Futibatinib (TAS 120) Plus Pembrolizumab in the Treatment of Advanced or Metastatic Urothelial Carcinoma

Cancer type: Bladder Urothelial Carcinoma

Variant class: FGFR3 mutation

Other identifiers: EudraCT Number: 2020-000945-15, TAS-120-203

Population segments: FGFR, First line, Stage III, Stage IV

Phase: II

Therapies: futibatinib, pembrolizumab

Location: United States

US State: MI

Contact: Dr. Maciej Gil [609-250-7336; clinicaltrialinfo@taihooncology.com]

NCT04096417
A Phase II, Multicenter, Single-Arm Study of Pemigatinib in Patients With Metastatic or Unresectable Colorectal Cancer Harboring FGFR Alterations

Cancer type: Colorectal Cancer

Variant class: FGFR3 mutation

Other identifiers: ACCRU-GI-1701, NCI-2019-05877, VICCGI2028

Population segments: FGFR, Second line, Stage III, Stage IV

Phase: II

Therapy: pemigatinib

Location: United States

US States: AZ, GA, MN, NC, TN

Contact: Multiple contacts: See www.clinicaltrials.gov for complete list of contacts.

Disclaimer: The data presented here is from a curated knowledgebase of publicly available information, but may not be exhaustive. The data version is 2021.11(003).

FGFR3 KD mutation (continued)

NCT04294277
Open-label, Single-arm, Phase II Study, Evaluating Safety and Efficacy of INCB054828 (Pemigatinib) as Adjuvant Therapy for Molecularly-selected, High-risk Patients With Urothelial Carcinoma Who Have Received Radical Surgery

Cancer type: Bladder Urothelial Carcinoma

Variant class: FGFR3 mutation

Other identifiers: EAU RF 2018-02, EAURF2018-02, EudraCT Number: 2017-004426-15, EudraCT Number: 2019-001833-14, PEGASUS

Population segments: Adjuvant, FGFR, Stage III, Stage IV

Phase: II

Therapy: pemigatinib

Location: Italy

NCT04077255
Anti-EGFR Antibody (GC-1118) in Combination With Weekly Paclitaxel as a Second-line Therapy for Gastric and Gastroesophageal Junction Adenocarcinomas With Amplifications

Cancer type: Gastric Cancer, Gastroesophageal Junction Adenocarcinoma

Variant class: FGFR3 mutation

Other identifiers: 31973, KCT0004314, NCC2019-0196

Population segments: EGFR, FGFR, Second line, Stage IV

Phase: II

Therapies: rogaratinib, chemotherapy

Location: Republic of Korea

No NCT ID - see other identifier(s)
Phase I Clinical Study of BPI-17509 In Patients With Advanced Solid Tumors

Cancer type: Bladder Cancer, Solid Tumor

Variant class: FGFR3 mutation

Other identifiers: BTP-26811, CTR20192140

Population segments: Second line, Stage III, Stage IV

Phase: I

Therapy: BPI-17509

Location: China

NCT04172675
A Randomized Phase II Study of Erdafitinib Versus Investigator Choice of Intravesical Chemotherapy in Subjects Who Received Bacillus Calmette-Guerin (BCG) and Recurred With High Risk Non-Muscle-Invasive Bladder Cancer (NMIBC) and FGFR Mutations or Fusions

Cancer type: Bladder Urothelial Carcinoma

Variant class: FGFR mutation

Other identifiers: 42756493BLC2003, 42756493BLC2003, CR108699, CTR20201841, CTRI/2021/08/036009, EudraCT Number: 2019-002449-39, JapicCTI-205145, NCI-2020-00674, RG1121102

Population segments: FGFR, Maintenance/Consolidation, Second line, Stage 0

Phase: II

Therapy: erdafitinib

Locations: Argentina, Australia, Belgium, Brazil, China, Czech Republic, France, Germany, Italy, Japan, Poland, Republic of Korea, Spain, Taiwan, United Kingdom, United States

US States: AZ, CA, CO, FL, GA, IL, KS, NC, NY, OH, PA, TN, TX, WA, WI

Contact: Study Contact [844-434-4210; JNJ.CT@sylogent.com]

FGFR3 KD mutation (continued)

NCT04595747
Phase II Study of Rogaratinib (BAY 1163877) in the Treatment of Patients With Sarcoma Harboring Alterations in Fibroblast Growth Factor Receptor (FGFR) 1-4 and SDH-Deficient Gastrointestinal Stromal Tumor (GIST)

Cancer type: Soft Tissue Sarcoma

Variant class: FGFR3 aberration

Other identifiers: 10411, NCI-2020-08011

Population segments: FGFR, Second line, Stage III, Stage IV

Phase: II

Therapy: rogaratinib

Location: United States

US States: CA, MA, MD, NY

Contact: Multiple contacts: See www.clinicaltrials.gov for complete list of contacts.

NCT04004975
Phase II Clinical Trials on Anlotinib for the Treatment of Recurrent Glioblastoma

Cancer type: Glioblastoma

Variant class: FGFR mutation

Other identifier: ShandongCHI006

Population segments: (N/A), FGFR, Second line

Phase: I/II

Therapy: anlotinib hydrochloride

Location: China

NCT04149691
A Phase I, Open-label, Multicentre, Dose Escalation Study to Assess Safety, Tolerability and Pharmacokinetics of Oral CPL304110, in Adult Subjects With Advanced Solid Malignancies

Cancer type: Bladder Cancer, Gastric Cancer, Non-Small Cell Lung Cancer

Variant class: FGFR3 aberration

Other identifier: 01FGFR2018

Population segments: FGFR, Line of therapy N/A, Squamous Cell, Stage III, Stage IV

Phase: I

Therapy: CPL-304-110

Location: Poland

NCT03473743
A Phase Ib-II Study to Evaluate Safety, Efficacy, Pharmacokinetics, and Pharmacodynamics of Various Regimens of Erdafitinib in Subjects With Metastatic or Locally Advanced Urothelial Cancer

Cancer type: Bladder Urothelial Carcinoma

Variant class: FGFR aberration

Other identifiers: 17165, 2018-0142, 42756493BLC2002, CR108445, EudraCT Number: 2017-001980-19, NCI-2018-00997, NORSE, USO 17165, USOR 17165

Population segments: FGFR, First line, Fourth line or greater, Second line, Stage IV, Third line

Phase: I/II

Therapies: erdafitinib, cetrelimab

Locations: Belarus, Belgium, Brazil, France, Italy, Poland, Republic of Korea, Russian Federation, Spain, Taiwan, Turkey, United Kingdom, United States

US States: CO, MD, NC, NY, PA, TX, VA

Contact: Study Contact [844-434-4210; JNJ.CT@sylogent.com]

Disclaimer: The data presented here is from a curated knowledgebase of publicly available information, but may not be exhaustive. The data version is 2021.11(003).

FGFR3 KD mutation (continued)

NCT02393248
A Phase I/II, Open-Label, Dose-Escalation, Safety and Tolerability Study of INCB054828 in Subjects With Advanced Malignancies - (FIGHT-101)

Cancer type: Bladder Urothelial Carcinoma, Breast Cancer, Cholangiocarcinoma, Endometrial Carcinoma, Gastric Cancer, Non-Small Cell Lung Cancer, Solid Tumor

Variant class: FGFR aberration

Other identifiers: 00061567, 2014-1099, 201503012, EudraCT Number: 2016-002831-14, FIGHT-101, INCB 54828-101, INCB54828-101, MC# 15-03, NCI-2015-00611, OSU-15241

Population segments: FGFR, First line, Fourth line or greater, Second line, Squamous Cell, Stage III, Stage IV, Third line

Phase: I/II

Therapies: pemigatinib, pembrolizumab, trastuzumab, chemotherapy, retifanlimab

Location: United States

US States: FL, NJ, TX

Contact: Incyte Call Center [855-463-3463]

NCT04572295
An Open-label Phase Ib Study of E7090 Monotherapy and in Combination With Other Anticancer Agents in Subjects With ER+, HER2- Recurrent/Metastatic Breast Cancer

Cancer type: Breast Cancer

Variant class: FGFR aberration

Other inclusion criteria: ERBB2 negative, ER positive

Other identifiers: E7090-J081-102, JapicCTI-205429

Population segments: Estrogen receptor positive, HER2 negative, Second line, Stage IV

Phase: I

Therapies: E-7090, hormone therapy

Location: Japan

NCT04504331
A Phase IB Study of Infigratinib in Combination With Tamoxifen in Hormone Receptor Positive, HER2 Negative, FGFR Altered Advanced Breast Cancer

Cancer type: Breast Cancer

Variant class: FGFR aberration

Other inclusion criteria: ERBB2 negative, Hormone receptor positive

Other identifiers: BRS0113, K08CA252457

Population segments: Estrogen receptor positive, HER2 negative, Progesterone receptor positive, Second line, Stage III, Stage IV, Triple receptor negative

Phase: I

Therapies: infigratinib, hormone therapy

Location: United States

US State: CA

Contact: Allison K. Zhang [650-736-5790; axkong@stanford.edu]

Disclaimer: The data presented here is from a curated knowledgebase of publicly available information, but may not be exhaustive. The data version is 2021.11(003).

FGFR3 mutation

NCT03155620
NCI-COG Pediatric MATCH (Molecular Analysis for Therapy Choice) Screening Protocol

Cancer type: Unspecified Solid Tumor

Variant class: FGFR3 mutation

Other identifiers: 17-729, 2017-0625, 20170841, APEC1621SC, COGAPEC1621, COGAPEC1621SC, N 55017 SC, NCI-2017-01251, NCI-COG Pediatric MATCH, Pediatric MATCH

Population segments: ALK, Aggressive, BRAF, FGFR, HRAS, Indolent, KRAS, NRAS, Pediatric or Adolescent, Second line, Stage III, Stage IV

Phase: II

Therapy: erdafitinib

Locations: Puerto Rico, United States

US States: AK, AL, AR, AZ, CA, CO, CT, DC, DE, FL, GA, HI, IA, ID, IL, IN, KY, LA, MA, MD, ME, MI, MN, MO, MS, NC, NE, NH, NJ, NV, NY, OH, OK, OR, PA, RI, SC, SD, TN, TX, UT, VA, VT, WA, WI

Contact: Multiple contacts: See www.clinicaltrials.gov for complete list of contacts.

NCT03210714
NCI-COG Pediatric MATCH (Molecular Analysis for Therapy Choice) - Phase II Subprotocol of JNJ-42756493 (Erdafitinib) in Patients With Tumors Harboring FGFR 1/2/3/4 Alterations

Cancer type: Unspecified Solid Tumor

Variant class: FGFR3 mutation

Other identifiers: APEC1621B, NCI-2017-01159, NCI-COG Pediatric MATCH, Pediatric MATCH

Population segments: Aggressive, FGFR, Indolent, Pediatric or Adolescent, Second line, Stage III, Stage IV

Phase: II

Therapy: erdafitinib

Locations: Puerto Rico, United States

US States: AK, AL, AR, AZ, CA, CO, CT, DC, DE, FL, GA, IA, ID, IL, IN, KY, LA, MD, ME, MI, MN, MO, MS, NC, ND, NE, NJ, NY, OH, OK, OR, PA, SC, SD, TN, TX, UT, VA, VT, WA, WI

Contact: Multiple contacts: See www.clinicaltrials.gov for complete list of contacts.

No NCT ID - see other identifier(s)
A Multicenter Phase II Basket-type Clinical Trial to Evaluate Efficacy and Safety of TAS-120 in Patients with Advanced Solid Malignancies with FGFR Alterations in Circulating Tumor DNA

Cancer type: Unspecified Solid Tumor

Variant class: FGFR3 mutation

Other identifiers: EPOC 1805, JapicCTI-194624, JapicCTI-194796, TiFFANY

Population segments: Second line, Stage III, Stage IV

Phase: II

Therapy: futibatinib

Location: Japan

FGFR3 mutation (continued)

NCT02693535
Targeted Agent and Profiling Utilization Registry (TAPUR) Study.

Cancer type: Unspecified Solid Tumor

Variant class: FGFR3 mutation

Other identifiers: 20170529, NCI-2017-00510, Pro00014171, TAPUR

Population segments: Aggressive, BRAF, Diffuse large B-cell lymphoma (DLBCL), Extranodal marginal zone B-cell lymphoma (MALT), FGFR, First line, Follicular lymphoma (FL), Fourth line or greater, Indolent, Lymphoblastic lymphoma (LBL), Mantle cell lymphoma (MCL), Other subtype, Second line, Small lymphocytic lymphoma (SLL), Stage III, Stage IV, Third line, Waldenstrom's macroglobulinemia (WM)

Phase: II

Therapy: sunitinib

Location: United States

US States: AL, AZ, CA, FL, GA, HI, IL, IN, ME, MI, NC, ND, NE, NH, OH, OR, PA, SD, TX, UT, VA, WA

Contact: Pam Mangat [pam.mangat@asco.org]

NCT04591431
The Rome Trial From Histology to Target: the Road to Personalize Target Therapy and Immunotherapy

Cancer type: Biliary Tract Carcinoma, Breast Cancer, Colorectal Cancer, Esophageal Cancer, Gastric Cancer, Gastroesophageal Junction Adenocarcinoma, Hepatocellular Carcinoma, Liver Cancer, Non-Small Cell Lung Cancer, Pancreatic Cancer, Unspecified Solid Tumor

Variant class: FGFR3 aberration

Other identifiers: EudraCT Number: 2018-002190-21, MAR-BAS-18-005, ROME

Population segments: Second line, Stage IV

Phase: II

Therapy: pemigatinib

Location: Italy

NCT02691793
Study to Evaluate the Safety and Efficacy of Sunitinib, in Subject With Refractory Solid Tumors

Cancer type: Unspecified Solid Tumor

Variant class: FGFR mutation

Other identifier: 2016-02-098

Population segments: (N/A), FGFR, Second line

Phase: II

Therapy: sunitinib

Location: Republic of Korea

Disclaimer: The data presented here is from a curated knowledgebase of publicly available information, but may not be exhaustive. The data version is 2021.11(003).

FGFR3 mutation (continued)

**NCT03297606**<br>Canadian Profiling and Targeted Agent Utilization Trial (CAPTUR): A Phase II Basket Trial<br>**Cancer type:** Unspecified Solid Tumor<br>**Variant class:** FGFR3 aberration	**Other identifiers:** CA209-9DL, CAPTUR, ESR-17-12831, ML39800, PM1, WI233446<br>**Population segments:** Aggressive, Diffuse large B-cell lymphoma (DLBCL), Extranodal marginal zone B-cell lymphoma (MALT), First line, Follicular lymphoma (FL), Indolent, Lymphoblastic lymphoma (LBL), Mantle cell lymphoma (MCL), Other subtype, Second line, Stage III, Stage IV, Waldenstrom's macroglobulinemia (WM)<br>**Phase:** II<br>**Therapy:** sunitinib<br>**Location:** Canada
**NCT03929965**<br>Anlotinib in Advanced Solid Tumors With FGFR Alteration<br>**Cancer type:** Unspecified Solid Tumor<br>**Variant class:** FGFR mutation	**Other identifier:** ASTFA<br>**Population segments:** Second line, Stage III, Stage IV, Third line<br>**Phase:** I<br>**Therapy:** anlotinib hydrochloride<br>**Location:** China
**NCT04565275**<br>A Multi-center Open-label, Phase I/II Clinical Trial to Evaluate the Safety, Tolerability, Pharmacokinetics, and Pharmacodynamics of ICP-192 in Patients With Advanced Solid Tumors and FGFR Gene Alterations.<br>**Cancer type:** Bladder Urothelial Carcinoma, Cholangiocarcinoma, Unspecified Solid Tumor<br>**Variant class:** FGFR aberration	**Other identifier:** ICP-CL-00303<br>**Population segments:** FGFR, Second line, Stage III, Stage IV<br>**Phase:** I/II<br>**Therapy:** ICP-192<br>**Location:** United States<br>**US States:** AZ, CO, FL, MN<br>**Contact:** Olivia Yang [609-524-0684; olivia.yang@INNOCAREPHARMA.COM]
**No NCT ID - see other identifier(s)**<br>Phase I/II Study of TAS-117 In Combination With TAS-120 In Patients With Advanced Solid Tumors<br>**Cancer type:** Unspecified Solid Tumor<br>**Variant class:** FGFR aberration	**Other identifier:** JapicCTI-194864<br>**Population segments:** FGFR, First line, Squamous Cell, Stage III, Stage IV<br>**Phase:** I/II<br>**Therapies:** TAS-117, futibatinib<br>**Location:** Japan

FGFR3 mutation (continued)

No NCT ID - see other identifier(s)
A Phase Ib Study to Assess the Safety, Tolerability, and Efficacy of TAS-120 (Futibatinib) in Combination with MK-3475 (Pembrolizumab) in Patients with Solid Tumors.

Cancer type: Unspecified Solid Tumor

Variant class: FGFR aberration

Other identifier: JapicCTI-195063

Population segments: FGFR, Line of therapy N/A, Stage III, Stage IV

Phase: I

Therapies: futibatinib, pembrolizumab

Location: Japan

NCT04053322
An International Multicenter Phase II Trial of Durvalumab (MEDI4736) Plus OLAparib Plus Fulvestrant in Metastatic or Locally Advanced ER-positive, HER2-negative Breast Cancer Patients Selected Using Criteria That Predict Sensitivity to Olaparib

Cancer type: Breast Cancer

Variant class: FGFR3 mutation

Other inclusion criteria: ERBB2 negative, ER positive

Other identifiers: CADUSEIME08, DOLAF, EudraCT Number: 2018-003832-57, UC-0140/1812

Population segments: BRCA, Estrogen receptor positive, FGFR, HER2 negative, Progesterone receptor positive, Second line, Stage III, Stage IV

Phase: II

Therapies: durvalumab, olaparib, hormone therapy

Location: France

NCT04601857
A Phase II Study Evaluating Futibatinib (TAS 120) Plus Pembrolizumab in the Treatment of Advanced or Metastatic Urothelial Carcinoma

Cancer type: Bladder Urothelial Carcinoma

Variant class: FGFR3 mutation

Other identifiers: EudraCT Number: 2020-000945-15, TAS-120-203

Population segments: FGFR, First line, Stage III, Stage IV

Phase: II

Therapies: futibatinib, pembrolizumab

Location: United States

US State: MI

Contact: Dr. Maciej Gil [609-250-7336; clinicaltrialinfo@taihooncology.com]

NCT04096417
A Phase II, Multicenter, Single-Arm Study of Pemigatinib in Patients With Metastatic or Unresectable Colorectal Cancer Harboring FGFR Alterations

Cancer type: Colorectal Cancer

Variant class: FGFR3 mutation

Other identifiers: ACCRU-GI-1701, NCI-2019-05877, VICCGI2028

Population segments: FGFR, Second line, Stage III, Stage IV

Phase: II

Therapy: pemigatinib

Location: United States

US States: AZ, GA, MN, NC, TN

Contact: Multiple contacts: See www.clinicaltrials.gov for complete list of contacts.

Disclaimer: The data presented here is from a curated knowledgebase of publicly available information, but may not be exhaustive. The data version is 2021.11(003).

FGFR3 mutation (continued)

NCT04294277
Open-label, Single-arm, Phase II Study, Evaluating Safety and Efficacy of INCB054828 (Pemigatinib) as Adjuvant Therapy for Molecularly-selected, High-risk Patients With Urothelial Carcinoma Who Have Received Radical Surgery

Cancer type: Bladder Urothelial Carcinoma

Variant class: FGFR3 mutation

Other identifiers: EAU RF 2018-02, EAURF2018-02, EudraCT Number: 2017-004426-15, EudraCT Number: 2019-001833-14, PEGASUS

Population segments: Adjuvant, FGFR, Stage III, Stage IV

Phase: II

Therapy: pemigatinib

Location: Italy

NCT04077255
Anti-EGFR Antibody (GC-1118) in Combination With Weekly Paclitaxel as a Second-line Therapy for Gastric and Gastroesophageal Junction Adenocarcinomas With Amplifications

Cancer type: Gastric Cancer, Gastroesophageal Junction Adenocarcinoma

Variant class: FGFR3 mutation

Other identifiers: 31973, KCT0004314, NCC2019-0196

Population segments: EGFR, FGFR, Second line, Stage IV

Phase: II

Therapies: rogaratinib, chemotherapy

Location: Republic of Korea

No NCT ID - see other identifier(s)
Phase I Clinical Study of BPI-17509 In Patients With Advanced Solid Tumors

Cancer type: Bladder Cancer, Solid Tumor

Variant class: FGFR3 mutation

Other identifiers: BTP-26811, CTR20192140

Population segments: Second line, Stage III, Stage IV

Phase: I

Therapy: BPI-17509

Location: China

NCT04172675
A Randomized Phase II Study of Erdafitinib Versus Investigator Choice of Intravesical Chemotherapy in Subjects Who Received Bacillus Calmette-Guerin (BCG) and Recurred With High Risk Non-Muscle-Invasive Bladder Cancer (NMIBC) and FGFR Mutations or Fusions

Cancer type: Bladder Urothelial Carcinoma

Variant class: FGFR mutation

Other identifiers: 42756493BLC2003, 42756493BLC2003, CR108699, CTR20201841, CTRI/2021/08/036009, EudraCT Number: 2019-002449-39, JapicCTI-205145, NCI-2020-00674, RG1121102

Population segments: FGFR, Maintenance/Consolidation, Second line, Stage 0

Phase: II

Therapy: erdafitinib

Locations: Argentina, Australia, Belgium, Brazil, China, Czech Republic, France, Germany, Italy, Japan, Poland, Republic of Korea, Spain, Taiwan, United Kingdom, United States

US States: AZ, CA, CO, FL, GA, IL, KS, NC, NY, OH, PA, TN, TX, WA, WI

Contact: Study Contact [844-434-4210; JNJ.CT@sylogent.com]

Disclaimer: The data presented here is from a curated knowledgebase of publicly available information, but may not be exhaustive. The data version is 2021.11(003).

FGFR3 mutation (continued)

NCT04595747

Phase II Study of Rogaratinib (BAY 1163877) in the Treatment of Patients With Sarcoma Harboring Alterations in Fibroblast Growth Factor Receptor (FGFR) 1-4 and SDH-Deficient Gastrointestinal Stromal Tumor (GIST)

Cancer type: Soft Tissue Sarcoma

Variant class: FGFR3 aberration

Other identifiers: 10411, NCI-2020-08011

Population segments: FGFR, Second line, Stage III, Stage IV

Phase: II

Therapy: rogaratinib

Location: United States

US States: CA, MA, MD, NY

Contact: Multiple contacts: See www.clinicaltrials.gov for complete list of contacts.

NCT04004975

Phase II Clinical Trials on Anlotinib for the Treatment of Recurrent Glioblastoma

Cancer type: Glioblastoma

Variant class: FGFR mutation

Other identifier: ShandongCHI006

Population segments: (N/A), FGFR, Second line

Phase: I/II

Therapy: anlotinib hydrochloride

Location: China

NCT04149691

A Phase I, Open-label, Multicentre, Dose Escalation Study to Assess Safety, Tolerability and Pharmacokinetics of Oral CPL304110, in Adult Subjects With Advanced Solid Malignancies

Cancer type: Bladder Cancer, Gastric Cancer, Non-Small Cell Lung Cancer

Variant class: FGFR3 aberration

Other identifier: 01FGFR2018

Population segments: FGFR, Line of therapy N/A, Squamous Cell, Stage III, Stage IV

Phase: I

Therapy: CPL-304-110

Location: Poland

NCT03473743

A Phase Ib-II Study to Evaluate Safety, Efficacy, Pharmacokinetics, and Pharmacodynamics of Various Regimens of Erdafitinib in Subjects With Metastatic or Locally Advanced Urothelial Cancer

Cancer type: Bladder Urothelial Carcinoma

Variant class: FGFR aberration

Other identifiers: 17165, 2018-0142, 42756493BLC2002, CR108445, EudraCT Number: 2017-001980-19, NCI-2018-00997, NORSE, USO 17165, USOR 17165

Population segments: FGFR, First line, Fourth line or greater, Second line, Stage IV, Third line

Phase: I/II

Therapies: erdafitinib, cetrelimab

Locations: Belarus, Belgium, Brazil, France, Italy, Poland, Republic of Korea, Russian Federation, Spain, Taiwan, Turkey, United Kingdom, United States

US States: CO, MD, NC, NY, PA, TX, VA

Contact: Study Contact [844-434-4210; JNJ.CT@sylogent.com]

Disclaimer: The data presented here is from a curated knowledgebase of publicly available information, but may not be exhaustive. The data version is 2021.11(003).

FGFR3 mutation (continued)

NCT02393248
A Phase I/II, Open-Label, Dose-Escalation, Safety and Tolerability Study of INCB054828 in Subjects With Advanced Malignancies - (FIGHT-101)

Cancer type: Bladder Urothelial Carcinoma, Breast Cancer, Cholangiocarcinoma, Endometrial Carcinoma, Gastric Cancer, Non-Small Cell Lung Cancer, Solid Tumor

Variant class: FGFR aberration

Other identifiers: 00061567, 2014-1099, 201503012, EudraCT Number: 2016-002831-14, FIGHT-101, INCB 54828-101, INCB54828-101, MC# 15-03, NCI-2015-00611, OSU-15241

Population segments: FGFR, First line, Fourth line or greater, Second line, Squamous Cell, Stage III, Stage IV, Third line

Phase: I/II

Therapies: pemigatinib, pembrolizumab, trastuzumab, chemotherapy, retifanlimab

Location: United States

US States: FL, NJ, TX

Contact: Incyte Call Center [855-463-3463]

NCT04572295
An Open-label Phase Ib Study of E7090 Monotherapy and in Combination With Other Anticancer Agents in Subjects With ER+, HER2- Recurrent/Metastatic Breast Cancer

Cancer type: Breast Cancer

Variant class: FGFR aberration

Other inclusion criteria: ERBB2 negative, ER positive

Other identifiers: E7090-J081-102, JapicCTI-205429

Population segments: Estrogen receptor positive, HER2 negative, Second line, Stage IV

Phase: I

Therapies: E-7090, hormone therapy

Location: Japan

NCT04504331
A Phase IB Study of Infigratinib in Combination With Tamoxifen in Hormone Receptor Positive, HER2 Negative, FGFR Altered Advanced Breast Cancer

Cancer type: Breast Cancer

Variant class: FGFR aberration

Other inclusion criteria: ERBB2 negative, Hormone receptor positive

Other identifiers: BRS0113, K08CA252457

Population segments: Estrogen receptor positive, HER2 negative, Progesterone receptor positive, Second line, Stage III, Stage IV, Triple receptor negative

Phase: I

Therapies: infigratinib, hormone therapy

Location: United States

US State: CA

Contact: Allison K. Zhang [650-736-5790; axkong@stanford.edu]

Disclaimer: The data presented here is from a curated knowledgebase of publicly available information, but may not be exhaustive. The data version is 2021.11(003).

FGFR3 V555 mutation

NCT03155620
NCI-COG Pediatric MATCH (Molecular Analysis for Therapy Choice) Screening Protocol

Cancer type: Unspecified Solid Tumor

Variant class: FGFR3 mutation

Other identifiers: 17-729, 2017-0625, 20170841, APEC1621SC, COGAPEC1621, COGAPEC1621SC, N 55017 SC, NCI-2017-01251, NCI-COG Pediatric MATCH, Pediatric MATCH

Population segments: ALK, Aggressive, BRAF, FGFR, HRAS, Indolent, KRAS, NRAS, Pediatric or Adolescent, Second line, Stage III, Stage IV

Phase: II

Therapy: erdafitinib

Locations: Puerto Rico, United States

US States: AK, AL, AR, AZ, CA, CO, CT, DC, DE, FL, GA, HI, IA, ID, IL, IN, KY, LA, MA, MD, ME, MI, MN, MO, MS, NC, NE, NH, NJ, NV, NY, OH, OK, OR, PA, RI, SC, SD, TN, TX, UT, VA, VT, WA, WI

Contact: Multiple contacts: See www.clinicaltrials.gov for complete list of contacts.

NCT03210714
NCI-COG Pediatric MATCH (Molecular Analysis for Therapy Choice) - Phase II Subprotocol of JNJ-42756493 (Erdafitinib) in Patients With Tumors Harboring FGFR 1/2/3/4 Alterations

Cancer type: Unspecified Solid Tumor

Variant class: FGFR3 mutation

Other identifiers: APEC1621B, NCI-2017-01159, NCI-COG Pediatric MATCH, Pediatric MATCH

Population segments: Aggressive, FGFR, Indolent, Pediatric or Adolescent, Second line, Stage III, Stage IV

Phase: II

Therapy: erdafitinib

Locations: Puerto Rico, United States

US States: AK, AL, AR, AZ, CA, CO, CT, DC, DE, FL, GA, IA, ID, IL, IN, KY, LA, MD, ME, MI, MN, MO, MS, NC, ND, NE, NJ, NY, OH, OK, OR, PA, SC, SD, TN, TX, UT, VA, VT, WA, WI

Contact: Multiple contacts: See www.clinicaltrials.gov for complete list of contacts.

No NCT ID - see other identifier(s)
A Multicenter Phase II Basket-type Clinical Trial to Evaluate Efficacy and Safety of TAS-120 in Patients with Advanced Solid Malignancies with FGFR Alterations in Circulating Tumor DNA

Cancer type: Unspecified Solid Tumor

Variant class: FGFR3 mutation

Other identifiers: EPOC 1805, JapicCTI-194624, JapicCTI-194796, TiFFANY

Population segments: Second line, Stage III, Stage IV

Phase: II

Therapy: futibatinib

Location: Japan

Disclaimer: The data presented here is from a curated knowledgebase of publicly available information, but may not be exhaustive. The data version is 2021.11(003).

FGFR3 V555 mutation (continued)

NCT02693535
Targeted Agent and Profiling Utilization Registry (TAPUR) Study.

Cancer type: Unspecified Solid Tumor

Variant class: FGFR3 mutation

Other identifiers: 20170529, NCI-2017-00510, Pro00014171, TAPUR

Population segments: Aggressive, BRAF, Diffuse large B-cell lymphoma (DLBCL), Extranodal marginal zone B-cell lymphoma (MALT), FGFR, First line, Follicular lymphoma (FL), Fourth line or greater, Indolent, Lymphoblastic lymphoma (LBL), Mantle cell lymphoma (MCL), Other subtype, Second line, Small lymphocytic lymphoma (SLL), Stage III, Stage IV, Third line, Waldenstrom's macroglobulinemia (WM)

Phase: II

Therapy: sunitinib

Location: United States

US States: AL, AZ, CA, FL, GA, HI, IL, IN, ME, MI, NC, ND, NE, NH, OH, OR, PA, SD, TX, UT, VA, WA

Contact: Pam Mangat [pam.mangat@asco.org]

NCT04591431
The Rome Trial From Histology to Target: the Road to Personalize Target Therapy and Immunotherapy

Cancer type: Biliary Tract Carcinoma, Breast Cancer, Colorectal Cancer, Esophageal Cancer, Gastric Cancer, Gastroesophageal Junction Adenocarcinoma, Hepatocellular Carcinoma, Liver Cancer, Non-Small Cell Lung Cancer, Pancreatic Cancer, Unspecified Solid Tumor

Variant class: FGFR3 aberration

Other identifiers: EudraCT Number: 2018-002190-21, MAR-BAS-18-005, ROME

Population segments: Second line, Stage IV

Phase: II

Therapy: pemigatinib

Location: Italy

NCT02691793
Study to Evaluate the Safety and Efficacy of Sunitinib, in Subject With Refractory Solid Tumors

Cancer type: Unspecified Solid Tumor

Variant class: FGFR mutation

Other identifier: 2016-02-098

Population segments: (N/A), FGFR, Second line

Phase: II

Therapy: sunitinib

Location: Republic of Korea

Disclaimer: The data presented here is from a curated knowledgebase of publicly available information, but may not be exhaustive. The data version is 2021.11(003).

FGFR3 V555 mutation (continued)

NCT03297606
Canadian Profiling and Targeted Agent Utilization Trial (CAPTUR): A Phase II Basket Trial

Cancer type: Unspecified Solid Tumor

Variant class: FGFR3 aberration

Other identifiers: CA209-9DL, CAPTUR, ESR-17-12831, ML39800, PM1, WI233446

Population segments: Aggressive, Diffuse large B-cell lymphoma (DLBCL), Extranodal marginal zone B-cell lymphoma (MALT), First line, Follicular lymphoma (FL), Indolent, Lymphoblastic lymphoma (LBL), Mantle cell lymphoma (MCL), Other subtype, Second line, Stage III, Stage IV, Waldenstrom's macroglobulinemia (WM)

Phase: II

Therapy: sunitinib

Location: Canada

NCT03929965
Anlotinib in Advanced Solid Tumors With FGFR Alteration

Cancer type: Unspecified Solid Tumor

Variant class: FGFR mutation

Other identifier: ASTFA

Population segments: Second line, Stage III, Stage IV, Third line

Phase: I

Therapy: anlotinib hydrochloride

Location: China

NCT04565275
A Multi-center Open-label, Phase I/II Clinical Trial to Evaluate the Safety, Tolerability, Pharmacokinetics, and Pharmacodynamics of ICP-192 in Patients With Advanced Solid Tumors and FGFR Gene Alterations.

Cancer type: Bladder Urothelial Carcinoma, Cholangiocarcinoma, Unspecified Solid Tumor

Variant class: FGFR aberration

Other identifier: ICP-CL-00303

Population segments: FGFR, Second line, Stage III, Stage IV

Phase: I/II

Therapy: ICP-192

Location: United States

US States: AZ, CO, FL, MN

Contact: Olivia Yang [609-524-0684; olivia.yang@INNOCAREPHARMA.COM]

No NCT ID - see other identifier(s)
Phase I/II Study of TAS-117 In Combination With TAS-120 In Patients With Advanced Solid Tumors

Cancer type: Unspecified Solid Tumor

Variant class: FGFR aberration

Other identifier: JapicCTI-194864

Population segments: FGFR, First line, Squamous Cell, Stage III, Stage IV

Phase: I/II

Therapies: TAS-117, futibatinib

Location: Japan

Disclaimer: The data presented here is from a curated knowledgebase of publicly available information, but may not be exhaustive. The data version is 2021.11(003).

FGFR3 V555 mutation (continued)

No NCT ID - see other identifier(s)
A Phase Ib Study to Assess the Safety, Tolerability, and Efficacy of TAS-120 (Futibatinib) in Combination with MK-3475 (Pembrolizumab) in Patients with Solid Tumors.

Cancer type: Unspecified Solid Tumor

Variant class: FGFR aberration

Other identifier: JapicCTI-195063

Population segments: FGFR, Line of therapy N/A, Stage III, Stage IV

Phase: I

Therapies: futibatinib, pembrolizumab

Location: Japan

NCT04053322
An International Multicenter Phase II Trial of Durvalumab (MEDI4736) Plus OLAparib Plus Fulvestrant in Metastatic or Locally Advanced ER-positive, HER2-negative Breast Cancer Patients Selected Using Criteria That Predict Sensitivity to Olaparib

Cancer type: Breast Cancer

Variant class: FGFR3 mutation

Other inclusion criteria: ERBB2 negative, ER positive

Other identifiers: CADUSEIME08, DOLAF, EudraCT Number: 2018-003832-57, UC-0140/1812

Population segments: BRCA, Estrogen receptor positive, FGFR, HER2 negative, Progesterone receptor positive, Second line, Stage III, Stage IV

Phase: II

Therapies: durvalumab, olaparib, hormone therapy

Location: France

NCT04601857
A Phase II Study Evaluating Futibatinib (TAS 120) Plus Pembrolizumab in the Treatment of Advanced or Metastatic Urothelial Carcinoma

Cancer type: Bladder Urothelial Carcinoma

Variant class: FGFR3 mutation

Other identifiers: EudraCT Number: 2020-000945-15, TAS-120-203

Population segments: FGFR, First line, Stage III, Stage IV

Phase: II

Therapies: futibatinib, pembrolizumab

Location: United States

US State: MI

Contact: Dr. Maciej Gil [609-250-7336; clinicaltrialinfo@taihooncology.com]

NCT04096417
A Phase II, Multicenter, Single-Arm Study of Pemigatinib in Patients With Metastatic or Unresectable Colorectal Cancer Harboring FGFR Alterations

Cancer type: Colorectal Cancer

Variant class: FGFR3 mutation

Other identifiers: ACCRU-GI-1701, NCI-2019-05877, VICCGI2028

Population segments: FGFR, Second line, Stage III, Stage IV

Phase: II

Therapy: pemigatinib

Location: United States

US States: AZ, GA, MN, NC, TN

Contact: Multiple contacts: See www.clinicaltrials.gov for complete list of contacts.

Disclaimer: The data presented here is from a curated knowledgebase of publicly available information, but may not be exhaustive. The data version is 2021.11(003).

FGFR3 V555 mutation (continued)

NCT04294277
Open-label, Single-arm, Phase II Study, Evaluating Safety and Efficacy of INCB054828 (Pemigatinib) as Adjuvant Therapy for Molecularly-selected, High-risk Patients With Urothelial Carcinoma Who Have Received Radical Surgery

Cancer type: Bladder Urothelial Carcinoma

Variant class: FGFR3 mutation

Other identifiers: EAU RF 2018-02, EAURF2018-02, EudraCT Number: 2017-004426-15, EudraCT Number: 2019-001833-14, PEGASUS

Population segments: Adjuvant, FGFR, Stage III, Stage IV

Phase: II

Therapy: pemigatinib

Location: Italy

NCT04077255
Anti-EGFR Antibody (GC-1118) in Combination With Weekly Paclitaxel as a Second-line Therapy for Gastric and Gastroesophageal Junction Adenocarcinomas With Amplifications

Cancer type: Gastric Cancer, Gastroesophageal Junction Adenocarcinoma

Variant class: FGFR3 mutation

Other identifiers: 31973, KCT0004314, NCC2019-0196

Population segments: EGFR, FGFR, Second line, Stage IV

Phase: II

Therapies: rogaratinib, chemotherapy

Location: Republic of Korea

No NCT ID - see other identifier(s)
Phase I Clinical Study of BPI-17509 In Patients With Advanced Solid Tumors

Cancer type: Bladder Cancer, Solid Tumor

Variant class: FGFR3 mutation

Other identifiers: BTP-26811, CTR20192140

Population segments: Second line, Stage III, Stage IV

Phase: I

Therapy: BPI-17509

Location: China

NCT04172675
A Randomized Phase II Study of Erdafitinib Versus Investigator Choice of Intravesical Chemotherapy in Subjects Who Received Bacillus Calmette-Guerin (BCG) and Recurred With High Risk Non-Muscle-Invasive Bladder Cancer (NMIBC) and FGFR Mutations or Fusions

Cancer type: Bladder Urothelial Carcinoma

Variant class: FGFR mutation

Other identifiers: 42756493BLC2003, 42756493BLC2003, CR108699, CTR20201841, CTRI/2021/08/036009, EudraCT Number: 2019-002449-39, JapicCTI-205145, NCI-2020-00674, RG1121102

Population segments: FGFR, Maintenance/Consolidation, Second line, Stage 0

Phase: II

Therapy: erdafitinib

Locations: Argentina, Australia, Belgium, Brazil, China, Czech Republic, France, Germany, Italy, Japan, Poland, Republic of Korea, Spain, Taiwan, United Kingdom, United States

US States: AZ, CA, CO, FL, GA, IL, KS, NC, NY, OH, PA, TN, TX, WA, WI

Contact: Study Contact [844-434-4210; JNJ.CT@sylogent.com]

Disclaimer: The data presented here is from a curated knowledgebase of publicly available information, but may not be exhaustive. The data version is 2021.11(003).

FGFR3 V555 mutation (continued)

NCT04595747
Phase II Study of Rogaratinib (BAY 1163877) in the Treatment of Patients With Sarcoma Harboring Alterations in Fibroblast Growth Factor Receptor (FGFR) 1-4 and SDH-Deficient Gastrointestinal Stromal Tumor (GIST)

Cancer type: Soft Tissue Sarcoma

Variant class: FGFR3 aberration

Other identifiers: 10411, NCI-2020-08011

Population segments: FGFR, Second line, Stage III, Stage IV

Phase: II

Therapy: rogaratinib

Location: United States

US States: CA, MA, MD, NY

Contact: Multiple contacts: See www.clinicaltrials.gov for complete list of contacts.

NCT04004975
Phase II Clinical Trials on Anlotinib for the Treatment of Recurrent Glioblastoma

Cancer type: Glioblastoma

Variant class: FGFR mutation

Other identifier: ShandongCHI006

Population segments: (N/A), FGFR, Second line

Phase: I/II

Therapy: anlotinib hydrochloride

Location: China

NCT04149691
A Phase I, Open-label, Multicentre, Dose Escalation Study to Assess Safety, Tolerability and Pharmacokinetics of Oral CPL304110, in Adult Subjects With Advanced Solid Malignancies

Cancer type: Bladder Cancer, Gastric Cancer, Non-Small Cell Lung Cancer

Variant class: FGFR3 aberration

Other identifier: 01FGFR2018

Population segments: FGFR, Line of therapy N/A, Squamous Cell, Stage III, Stage IV

Phase: I

Therapy: CPL-304-110

Location: Poland

NCT03473743
A Phase Ib-II Study to Evaluate Safety, Efficacy, Pharmacokinetics, and Pharmacodynamics of Various Regimens of Erdafitinib in Subjects With Metastatic or Locally Advanced Urothelial Cancer

Cancer type: Bladder Urothelial Carcinoma

Variant class: FGFR aberration

Other identifiers: 17165, 2018-0142, 42756493BLC2002, CR108445, EudraCT Number: 2017-001980-19, NCI-2018-00997, NORSE, USO 17165, USOR 17165

Population segments: FGFR, First line, Fourth line or greater, Second line, Stage IV, Third line

Phase: I/II

Therapies: erdafitinib, cetrelimab

Locations: Belarus, Belgium, Brazil, France, Italy, Poland, Republic of Korea, Russian Federation, Spain, Taiwan, Turkey, United Kingdom, United States

US States: CO, MD, NC, NY, PA, TX, VA

Contact: Study Contact [844-434-4210; JNJ.CT@sylogent.com]

Disclaimer: The data presented here is from a curated knowledgebase of publicly available information, but may not be exhaustive. The data version is 2021.11(003).

FGFR3 V555 mutation (continued)

NCT02393248

A Phase I/II, Open-Label, Dose-Escalation, Safety and Tolerability Study of INCB054828 in Subjects With Advanced Malignancies - (FIGHT-101)

Cancer type: Bladder Urothelial Carcinoma, Breast Cancer, Cholangiocarcinoma, Endometrial Carcinoma, Gastric Cancer, Non-Small Cell Lung Cancer, Solid Tumor

Variant class: FGFR aberration

Other identifiers: 00061567, 2014-1099, 201503012, EudraCT Number: 2016-002831-14, FIGHT-101, INCB 54828-101, INCB54828-101, MC# 15-03, NCI-2015-00611, OSU-15241

Population segments: FGFR, First line, Fourth line or greater, Second line, Squamous Cell, Stage III, Stage IV, Third line

Phase: I/II

Therapies: pemigatinib, pembrolizumab, trastuzumab, chemotherapy, retifanlimab

Location: United States

US States: FL, NJ, TX

Contact: Incyte Call Center [855-463-3463]

NCT04572295

An Open-label Phase Ib Study of E7090 Monotherapy and in Combination With Other Anticancer Agents in Subjects With ER+, HER2- Recurrent/Metastatic Breast Cancer

Cancer type: Breast Cancer

Variant class: FGFR aberration

Other inclusion criteria: ERBB2 negative, ER positive

Other identifiers: E7090-J081-102, JapicCTI-205429

Population segments: Estrogen receptor positive, HER2 negative, Second line, Stage IV

Phase: I

Therapies: E-7090, hormone therapy

Location: Japan

NCT04504331

A Phase IB Study of Infigratinib in Combination With Tamoxifen in Hormone Receptor Positive, HER2 Negative, FGFR Altered Advanced Breast Cancer

Cancer type: Breast Cancer

Variant class: FGFR aberration

Other inclusion criteria: ERBB2 negative, Hormone receptor positive

Other identifiers: BRS0113, K08CA252457

Population segments: Estrogen receptor positive, HER2 negative, Progesterone receptor positive, Second line, Stage III, Stage IV, Triple receptor negative

Phase: I

Therapies: infigratinib, hormone therapy

Location: United States

US State: CA

Contact: Allison K. Zhang [650-736-5790; axkong@stanford.edu]

FGFR3 V557 mutation

NCT03155620
NCI-COG Pediatric MATCH (Molecular Analysis for Therapy Choice) Screening Protocol

Cancer type: Unspecified Solid Tumor

Variant class: FGFR3 mutation

Other identifiers: 17-729, 2017-0625, 20170841, APEC1621SC, COGAPEC1621, COGAPEC1621SC, N 55017 SC, NCI-2017-01251, NCI-COG Pediatric MATCH, Pediatric MATCH

Population segments: ALK, Aggressive, BRAF, FGFR, HRAS, Indolent, KRAS, NRAS, Pediatric or Adolescent, Second line, Stage III, Stage IV

Phase: II

Therapy: erdafitinib

Locations: Puerto Rico, United States

US States: AK, AL, AR, AZ, CA, CO, CT, DC, DE, FL, GA, HI, IA, ID, IL, IN, KY, LA, MA, MD, ME, MI, MN, MO, MS, NC, NE, NH, NJ, NV, NY, OH, OK, OR, PA, RI, SC, SD, TN, TX, UT, VA, VT, WA, WI

Contact: Multiple contacts: See www.clinicaltrials.gov for complete list of contacts.

NCT03210714
NCI-COG Pediatric MATCH (Molecular Analysis for Therapy Choice) - Phase II Subprotocol of JNJ-42756493 (Erdafitinib) in Patients With Tumors Harboring FGFR 1/2/3/4 Alterations

Cancer type: Unspecified Solid Tumor

Variant class: FGFR3 mutation

Other identifiers: APEC1621B, NCI-2017-01159, NCI-COG Pediatric MATCH, Pediatric MATCH

Population segments: Aggressive, FGFR, Indolent, Pediatric or Adolescent, Second line, Stage III, Stage IV

Phase: II

Therapy: erdafitinib

Locations: Puerto Rico, United States

US States: AK, AL, AR, AZ, CA, CO, CT, DC, DE, FL, GA, IA, ID, IL, IN, KY, LA, MD, ME, MI, MN, MO, MS, NC, ND, NE, NJ, NY, OH, OK, OR, PA, SC, SD, TN, TX, UT, VA, VT, WA, WI

Contact: Multiple contacts: See www.clinicaltrials.gov for complete list of contacts.

No NCT ID - see other identifier(s)
A Multicenter Phase II Basket-type Clinical Trial to Evaluate Efficacy and Safety of TAS-120 in Patients with Advanced Solid Malignancies with FGFR Alterations in Circulating Tumor DNA

Cancer type: Unspecified Solid Tumor

Variant class: FGFR3 mutation

Other identifiers: EPOC 1805, JapicCTI-194624, JapicCTI-194796, TiFFANY

Population segments: Second line, Stage III, Stage IV

Phase: II

Therapy: futibatinib

Location: Japan

Disclaimer: The data presented here is from a curated knowledgebase of publicly available information, but may not be exhaustive. The data version is 2021.11(003).

FGFR3 V557 mutation (continued)

NCT02693535
Targeted Agent and Profiling Utilization Registry (TAPUR) Study.

Cancer type: Unspecified Solid Tumor

Variant class: FGFR3 mutation

Other identifiers: 20170529, NCI-2017-00510, Pro00014171, TAPUR

Population segments: Aggressive, BRAF, Diffuse large B-cell lymphoma (DLBCL), Extranodal marginal zone B-cell lymphoma (MALT), FGFR, First line, Follicular lymphoma (FL), Fourth line or greater, Indolent, Lymphoblastic lymphoma (LBL), Mantle cell lymphoma (MCL), Other subtype, Second line, Small lymphocytic lymphoma (SLL), Stage III, Stage IV, Third line, Waldenstrom's macroglobulinemia (WM)

Phase: II

Therapy: sunitinib

Location: United States

US States: AL, AZ, CA, FL, GA, HI, IL, IN, ME, MI, NC, ND, NE, NH, OH, OR, PA, SD, TX, UT, VA, WA

Contact: Pam Mangat [pam.mangat@asco.org]

NCT04591431
The Rome Trial From Histology to Target: the Road to Personalize Target Therapy and Immunotherapy

Cancer type: Biliary Tract Carcinoma, Breast Cancer, Colorectal Cancer, Esophageal Cancer, Gastric Cancer, Gastroesophageal Junction Adenocarcinoma, Hepatocellular Carcinoma, Liver Cancer, Non-Small Cell Lung Cancer, Pancreatic Cancer, Unspecified Solid Tumor

Variant class: FGFR3 aberration

Other identifiers: EudraCT Number: 2018-002190-21, MAR-BAS-18-005, ROME

Population segments: Second line, Stage IV

Phase: II

Therapy: pemigatinib

Location: Italy

NCT02691793
Study to Evaluate the Safety and Efficacy of Sunitinib, in Subject With Refractory Solid Tumors

Cancer type: Unspecified Solid Tumor

Variant class: FGFR mutation

Other identifier: 2016-02-098

Population segments: (N/A), FGFR, Second line

Phase: II

Therapy: sunitinib

Location: Republic of Korea

Disclaimer: The data presented here is from a curated knowledgebase of publicly available information, but may not be exhaustive. The data version is 2021.11(003).

FGFR3 V557 mutation (continued)

NCT03297606
Canadian Profiling and Targeted Agent Utilization Trial (CAPTUR): A Phase II Basket Trial

Cancer type: Unspecified Solid Tumor

Variant class: FGFR3 aberration

Other identifiers: CA209-9DL, CAPTUR, ESR-17-12831, ML39800, PM1, WI233446

Population segments: Aggressive, Diffuse large B-cell lymphoma (DLBCL), Extranodal marginal zone B-cell lymphoma (MALT), First line, Follicular lymphoma (FL), Indolent, Lymphoblastic lymphoma (LBL), Mantle cell lymphoma (MCL), Other subtype, Second line, Stage III, Stage IV, Waldenstrom's macroglobulinemia (WM)

Phase: II

Therapy: sunitinib

Location: Canada

NCT03929965
Anlotinib in Advanced Solid Tumors With FGFR Alteration

Cancer type: Unspecified Solid Tumor

Variant class: FGFR mutation

Other identifier: ASTFA

Population segments: Second line, Stage III, Stage IV, Third line

Phase: I

Therapy: anlotinib hydrochloride

Location: China

NCT04565275
A Multi-center Open-label, Phase I/II Clinical Trial to Evaluate the Safety, Tolerability, Pharmacokinetics, and Pharmacodynamics of ICP-192 in Patients With Advanced Solid Tumors and FGFR Gene Alterations.

Cancer type: Bladder Urothelial Carcinoma, Cholangiocarcinoma, Unspecified Solid Tumor

Variant class: FGFR aberration

Other identifier: ICP-CL-00303

Population segments: FGFR, Second line, Stage III, Stage IV

Phase: I/II

Therapy: ICP-192

Location: United States

US States: AZ, CO, FL, MN

Contact: Olivia Yang [609-524-0684; olivia.yang@INNOCAREPHARMA.COM]

No NCT ID - see other identifier(s)
Phase I/II Study of TAS-117 In Combination With TAS-120 In Patients With Advanced Solid Tumors

Cancer type: Unspecified Solid Tumor

Variant class: FGFR aberration

Other identifier: JapicCTI-194864

Population segments: FGFR, First line, Squamous Cell, Stage III, Stage IV

Phase: I/II

Therapies: TAS-117, futibatinib

Location: Japan

FGFR3 V557 mutation (continued)

No NCT ID - see other identifier(s)
A Phase Ib Study to Assess the Safety, Tolerability, and Efficacy of TAS-120 (Futibatinib) in Combination with MK-3475 (Pembrolizumab) in Patients with Solid Tumors.

Cancer type: Unspecified Solid Tumor

Variant class: FGFR aberration

Other identifier: JapicCTI-195063

Population segments: FGFR, Line of therapy N/A, Stage III, Stage IV

Phase: I

Therapies: futibatinib, pembrolizumab

Location: Japan

NCT04053322
An International Multicenter Phase II Trial of Durvalumab (MEDI4736) Plus OLAparib Plus Fulvestrant in Metastatic or Locally Advanced ER-positive, HER2-negative Breast Cancer Patients Selected Using Criteria That Predict Sensitivity to Olaparib

Cancer type: Breast Cancer

Variant class: FGFR3 mutation

Other inclusion criteria: ERBB2 negative, ER positive

Other identifiers: CADUSEIME08, DOLAF, EudraCT Number: 2018-003832-57, UC-0140/1812

Population segments: BRCA, Estrogen receptor positive, FGFR, HER2 negative, Progesterone receptor positive, Second line, Stage III, Stage IV

Phase: II

Therapies: durvalumab, olaparib, hormone therapy

Location: France

NCT04601857
A Phase II Study Evaluating Futibatinib (TAS 120) Plus Pembrolizumab in the Treatment of Advanced or Metastatic Urothelial Carcinoma

Cancer type: Bladder Urothelial Carcinoma

Variant class: FGFR3 mutation

Other identifiers: EudraCT Number: 2020-000945-15, TAS-120-203

Population segments: FGFR, First line, Stage III, Stage IV

Phase: II

Therapies: futibatinib, pembrolizumab

Location: United States

US State: MI

Contact: Dr. Maciej Gil [609-250-7336; clinicaltrialinfo@taihooncology.com]

NCT04096417
A Phase II, Multicenter, Single-Arm Study of Pemigatinib in Patients With Metastatic or Unresectable Colorectal Cancer Harboring FGFR Alterations

Cancer type: Colorectal Cancer

Variant class: FGFR3 mutation

Other identifiers: ACCRU-GI-1701, NCI-2019-05877, VICCGI2028

Population segments: FGFR, Second line, Stage III, Stage IV

Phase: II

Therapy: pemigatinib

Location: United States

US States: AZ, GA, MN, NC, TN

Contact: Multiple contacts: See www.clinicaltrials.gov for complete list of contacts.

Disclaimer: The data presented here is from a curated knowledgebase of publicly available information, but may not be exhaustive. The data version is 2021.11(003).

FGFR3 V557 mutation (continued)

NCT04294277
Open-label, Single-arm, Phase II Study, Evaluating Safety and Efficacy of INCB054828 (Pemigatinib) as Adjuvant Therapy for Molecularly-selected, High-risk Patients With Urothelial Carcinoma Who Have Received Radical Surgery

Cancer type: Bladder Urothelial Carcinoma

Variant class: FGFR3 mutation

Other identifiers: EAU RF 2018-02, EAURF2018-02, EudraCT Number: 2017-004426-15, EudraCT Number: 2019-001833-14, PEGASUS

Population segments: Adjuvant, FGFR, Stage III, Stage IV

Phase: II

Therapy: pemigatinib

Location: Italy

NCT04077255
Anti-EGFR Antibody (GC-1118) in Combination With Weekly Paclitaxel as a Second-line Therapy for Gastric and Gastroesophageal Junction Adenocarcinomas With Amplifications

Cancer type: Gastric Cancer, Gastroesophageal Junction Adenocarcinoma

Variant class: FGFR3 mutation

Other identifiers: 31973, KCT0004314, NCC2019-0196

Population segments: EGFR, FGFR, Second line, Stage IV

Phase: II

Therapies: rogaratinib, chemotherapy

Location: Republic of Korea

No NCT ID - see other identifier(s)
Phase I Clinical Study of BPI-17509 In Patients With Advanced Solid Tumors

Cancer type: Bladder Cancer, Solid Tumor

Variant class: FGFR3 mutation

Other identifiers: BTP-26811, CTR20192140

Population segments: Second line, Stage III, Stage IV

Phase: I

Therapy: BPI-17509

Location: China

NCT04172675
A Randomized Phase II Study of Erdafitinib Versus Investigator Choice of Intravesical Chemotherapy in Subjects Who Received Bacillus Calmette-Guerin (BCG) and Recurred With High Risk Non-Muscle-Invasive Bladder Cancer (NMIBC) and FGFR Mutations or Fusions

Cancer type: Bladder Urothelial Carcinoma

Variant class: FGFR mutation

Other identifiers: 42756493BLC2003, 42756493BLC2003, CR108699, CTR20201841, CTRI/2021/08/036009, EudraCT Number: 2019-002449-39, JapicCTI-205145, NCI-2020-00674, RG1121102

Population segments: FGFR, Maintenance/Consolidation, Second line, Stage 0

Phase: II

Therapy: erdafitinib

Locations: Argentina, Australia, Belgium, Brazil, China, Czech Republic, France, Germany, Italy, Japan, Poland, Republic of Korea, Spain, Taiwan, United Kingdom, United States

US States: AZ, CA, CO, FL, GA, IL, KS, NC, NY, OH, PA, TN, TX, WA, WI

Contact: Study Contact [844-434-4210; JNJ.CT@sylogent.com]

Disclaimer: The data presented here is from a curated knowledgebase of publicly available information, but may not be exhaustive. The data version is 2021.11(003).

FGFR3 V557 mutation (continued)

NCT04595747
Phase II Study of Rogaratinib (BAY 1163877) in the Treatment of Patients With Sarcoma Harboring Alterations in Fibroblast Growth Factor Receptor (FGFR) 1-4 and SDH-Deficient Gastrointestinal Stromal Tumor (GIST)

Cancer type: Soft Tissue Sarcoma

Variant class: FGFR3 aberration

Other identifiers: 10411, NCI-2020-08011

Population segments: FGFR, Second line, Stage III, Stage IV

Phase: II

Therapy: rogaratinib

Location: United States

US States: CA, MA, MD, NY

Contact: Multiple contacts: See www.clinicaltrials.gov for complete list of contacts.

NCT04004975
Phase II Clinical Trials on Anlotinib for the Treatment of Recurrent Glioblastoma

Cancer type: Glioblastoma

Variant class: FGFR mutation

Other identifier: ShandongCHI006

Population segments: (N/A), FGFR, Second line

Phase: I/II

Therapy: anlotinib hydrochloride

Location: China

NCT04149691
A Phase I, Open-label, Multicentre, Dose Escalation Study to Assess Safety, Tolerability and Pharmacokinetics of Oral CPL304110, in Adult Subjects With Advanced Solid Malignancies

Cancer type: Bladder Cancer, Gastric Cancer, Non-Small Cell Lung Cancer

Variant class: FGFR3 aberration

Other identifier: 01FGFR2018

Population segments: FGFR, Line of therapy N/A, Squamous Cell, Stage III, Stage IV

Phase: I

Therapy: CPL-304-110

Location: Poland

NCT03473743
A Phase Ib-II Study to Evaluate Safety, Efficacy, Pharmacokinetics, and Pharmacodynamics of Various Regimens of Erdafitinib in Subjects With Metastatic or Locally Advanced Urothelial Cancer

Cancer type: Bladder Urothelial Carcinoma

Variant class: FGFR aberration

Other identifiers: 17165, 2018-0142, 42756493BLC2002, CR108445, EudraCT Number: 2017-001980-19, NCI-2018-00997, NORSE, USO 17165, USOR 17165

Population segments: FGFR, First line, Fourth line or greater, Second line, Stage IV, Third line

Phase: I/II

Therapies: erdafitinib, cetrelimab

Locations: Belarus, Belgium, Brazil, France, Italy, Poland, Republic of Korea, Russian Federation, Spain, Taiwan, Turkey, United Kingdom, United States

US States: CO, MD, NC, NY, PA, TX, VA

Contact: Study Contact [844-434-4210; JNJ.CT@sylogent.com]

FGFR3 V557 mutation (continued)

NCT02393248
A Phase I/II, Open-Label, Dose-Escalation, Safety and Tolerability Study of INCB054828 in Subjects With Advanced Malignancies - (FIGHT-101)

Cancer type: Bladder Urothelial Carcinoma, Breast Cancer, Cholangiocarcinoma, Endometrial Carcinoma, Gastric Cancer, Non-Small Cell Lung Cancer, Solid Tumor

Variant class: FGFR aberration

Other identifiers: 00061567, 2014-1099, 201503012, EudraCT Number: 2016-002831-14, FIGHT-101, INCB 54828-101, INCB54828-101, MC# 15-03, NCI-2015-00611, OSU-15241

Population segments: FGFR, First line, Fourth line or greater, Second line, Squamous Cell, Stage III, Stage IV, Third line

Phase: I/II

Therapies: pemigatinib, pembrolizumab, trastuzumab, chemotherapy, retifanlimab

Location: United States

US States: FL, NJ, TX

Contact: Incyte Call Center [855-463-3463]

NCT04572295
An Open-label Phase Ib Study of E7090 Monotherapy and in Combination With Other Anticancer Agents in Subjects With ER+, HER2- Recurrent/Metastatic Breast Cancer

Cancer type: Breast Cancer

Variant class: FGFR aberration

Other inclusion criteria: ERBB2 negative, ER positive

Other identifiers: E7090-J081-102, JapicCTI-205429

Population segments: Estrogen receptor positive, HER2 negative, Second line, Stage IV

Phase: I

Therapies: E-7090, hormone therapy

Location: Japan

NCT04504331
A Phase IB Study of Infigratinib in Combination With Tamoxifen in Hormone Receptor Positive, HER2 Negative, FGFR Altered Advanced Breast Cancer

Cancer type: Breast Cancer

Variant class: FGFR aberration

Other inclusion criteria: ERBB2 negative, Hormone receptor positive

Other identifiers: BRS0113, K08CA252457

Population segments: Estrogen receptor positive, HER2 negative, Progesterone receptor positive, Second line, Stage III, Stage IV, Triple receptor negative

Phase: I

Therapies: infigratinib, hormone therapy

Location: United States

US State: CA

Contact: Allison K. Zhang [650-736-5790; axkong@stanford.edu]

FGFR3 amplification

NCT02693535
Targeted Agent and Profiling Utilization Registry (TAPUR) Study.

Cancer type: Unspecified Solid Tumor

Variant class: FGFR3 amplification

Other identifiers: 20170529, NCI-2017-00510, Pro00014171, TAPUR

Population segments: Aggressive, BRAF, Diffuse large B-cell lymphoma (DLBCL), Extranodal marginal zone B-cell lymphoma (MALT), FGFR, First line, Follicular lymphoma (FL), Fourth line or greater, Indolent, Lymphoblastic lymphoma (LBL), Mantle cell lymphoma (MCL), Other subtype, Second line, Small lymphocytic lymphoma (SLL), Stage III, Stage IV, Third line, Waldenstrom's macroglobulinemia (WM)

Phase: II

Therapy: sunitinib

Location: United States

US States: AL, AZ, CA, FL, GA, HI, IL, IN, ME, MI, NC, ND, NE, NH, OH, OR, PA, SD, TX, UT, VA, WA

Contact: Pam Mangat [pam.mangat@asco.org]

NCT04233567
A Phase II Study of Oral Infigratinib in Adult Patients With Advanced or Metastatic Solid Tumors With FGFR1-3 Gene Fusions or Other FGFR Genetic Alterations

Cancer type: Unspecified Solid Tumor

Variant class: FGFR amplification

Other identifiers: NCI-2019-06869, OSU-19041

Population segments: Second line, Stage III, Stage IV

Phase: II

Therapy: infigratinib

Location: United States

US State: OH

Contact: The Ohio State University Comprehensive Cancer Center [800-293-5066; OSUCCCClinicaltrials@osumc.edu]

NCT04591431
The Rome Trial From Histology to Target: the Road to Personalize Target Therapy and Immunotherapy

Cancer type: Biliary Tract Carcinoma, Breast Cancer, Colorectal Cancer, Esophageal Cancer, Gastric Cancer, Gastroesophageal Junction Adenocarcinoma, Hepatocellular Carcinoma, Liver Cancer, Non-Small Cell Lung Cancer, Pancreatic Cancer, Unspecified Solid Tumor

Variant class: FGFR3 aberration

Other identifiers: EudraCT Number: 2018-002190-21, MAR-BAS-18-005, ROME

Population segments: Second line, Stage IV

Phase: II

Therapy: pemigatinib

Location: Italy

Disclaimer: The data presented here is from a curated knowledgebase of publicly available information, but may not be exhaustive. The data version is 2021.11(003).

FGFR3 amplification (continued)

NCT03297606
Canadian Profiling and Targeted Agent Utilization Trial (CAPTUR): A Phase II Basket Trial

Cancer type: Unspecified Solid Tumor

Variant class: FGFR3 aberration

Other identifiers: CA209-9DL, CAPTUR, ESR-17-12831, ML39800, PM1, WI233446

Population segments: Aggressive, Diffuse large B-cell lymphoma (DLBCL), Extranodal marginal zone B-cell lymphoma (MALT), First line, Follicular lymphoma (FL), Indolent, Lymphoblastic lymphoma (LBL), Mantle cell lymphoma (MCL), Other subtype, Second line, Stage III, Stage IV, Waldenstrom's macroglobulinemia (WM)

Phase: II

Therapy: sunitinib

Location: Canada

NCT04565275
A Multi-center Open-label, Phase I/II Clinical Trial to Evaluate the Safety, Tolerability, Pharmacokinetics, and Pharmacodynamics of ICP-192 in Patients With Advanced Solid Tumors and FGFR Gene Alterations.

Cancer type: Bladder Urothelial Carcinoma, Cholangiocarcinoma, Unspecified Solid Tumor

Variant class: FGFR aberration

Other identifier: ICP-CL-00303

Population segments: FGFR, Second line, Stage III, Stage IV

Phase: I/II

Therapy: ICP-192

Location: United States

US States: AZ, CO, FL, MN

Contact: Olivia Yang [609-524-0684; olivia.yang@INNOCAREPHARMA.COM]

No NCT ID - see other identifier(s)
Phase I/II Study of TAS-117 In Combination With TAS-120 In Patients With Advanced Solid Tumors

Cancer type: Unspecified Solid Tumor

Variant class: FGFR aberration

Other identifier: JapicCTI-194864

Population segments: FGFR, First line, Squamous Cell, Stage III, Stage IV

Phase: I/II

Therapies: TAS-117, futibatinib

Location: Japan

No NCT ID - see other identifier(s)
A Phase Ib Study to Assess the Safety, Tolerability, and Efficacy of TAS-120 (Futibatinib) in Combination with MK-3475 (Pembrolizumab) in Patients with Solid Tumors.

Cancer type: Unspecified Solid Tumor

Variant class: FGFR aberration

Other identifier: JapicCTI-195063

Population segments: FGFR, Line of therapy N/A, Stage III, Stage IV

Phase: I

Therapies: futibatinib, pembrolizumab

Location: Japan

Disclaimer: The data presented here is from a curated knowledgebase of publicly available information, but may not be exhaustive. The data version is 2021.11(003).

FGFR3 amplification (continued)

NCT04936295
A Phase II Study of the Efficacy and Tolerability of Fulvestrant Plus Anlotinib in HR(+)/HER2(-) Metastatic Breast Cancer Patients With FGFR Mutation

Cancer type: Breast Cancer

Variant class: FGFR3 amplification

Other inclusion criteria: ERBB2 negative, Hormone receptor positive

Other identifier: SYSU005

Population segments: Estrogen receptor positive, FGFR, First line, HER2 negative, HER2 positive, Progesterone receptor positive, Stage IV

Phase: II

Therapies: hormone therapy, anlotinib hydrochloride

Location: China

NCT04096417
A Phase II, Multicenter, Single-Arm Study of Pemigatinib in Patients With Metastatic or Unresectable Colorectal Cancer Harboring FGFR Alterations

Cancer type: Colorectal Cancer

Variant class: FGFR3 amplification

Other identifiers: ACCRU-GI-1701, NCI-2019-05877, VICCGI2028

Population segments: FGFR, Second line, Stage III, Stage IV

Phase: II

Therapy: pemigatinib

Location: United States

US States: AZ, GA, MN, NC, TN

Contact: Multiple contacts: See www.clinicaltrials.gov for complete list of contacts.

NCT04077255
Anti-EGFR Antibody (GC-1118) in Combination With Weekly Paclitaxel as a Second-line Therapy for Gastric and Gastroesophageal Junction Adenocarcinomas With Amplifications

Cancer type: Gastric Cancer, Gastroesophageal Junction Adenocarcinoma

Variant class: FGFR3 amplification

Other identifiers: 31973, KCT0004314, NCC2019-0196

Population segments: EGFR, FGFR, Second line, Stage IV

Phase: II

Therapies: rogaratinib, chemotherapy

Location: Republic of Korea

NCT04483505
Rogaratinib, Palbociclib and Fulvestrant in Advanced Hormone Receptor Positive, FGFR1/2/3-positive Breast Cancer: Phase I Clinical Trial Plus an Expansion Cohort

Cancer type: Breast Cancer

Variant class: FGFR3 amplification

Other inclusion criteria: ERBB2 negative, Hormone receptor positive

Other identifiers: EudraCT Number: 2020-000055-12, ROGABREAST

Population segments: Estrogen receptor positive, FGFR, HER2 negative, Progesterone receptor positive, Second line, Stage III, Stage IV

Phase: I

Therapies: rogaratinib, palbociclib, hormone therapy

Location: Spain

Disclaimer: The data presented here is from a curated knowledgebase of publicly available information, but may not be exhaustive. The data version is 2021.11(003).

FGFR3 amplification (continued)

NCT03827850

A Phase II Trial to Evaluate Efficacy and Safety of Erdafitinib in Patients With Advanced Squamous Non Small Cell Lung Carcinoma (sqNSCLC) Harboring Fibroblast Growth Factor Receptor (FGFR) Genetic Alterations After Relapse of Standard Therapy.

Cancer type: Non-Small Cell Lung Cancer

Variant class: FGFR activating aberration

Other identifiers: EudraCT Number: 2018-000399-13, FIND, FIND Trial, Uni-Koeln-3254

Population segments: FGFR, Second line, Squamous Cell, Stage III, Stage IV

Exclusion criteria variant classes: ALK aberration (Non-Small Cell Lung Cancer), BRAF aberration (Non-Small Cell Lung Cancer), EGFR aberration (Non-Small Cell Lung Cancer), NTRK aberration (Non-Small Cell Lung Cancer), ROS1 aberration (Non-Small Cell Lung Cancer)

Phase: II

Therapy: erdafitinib

Location: Germany

NCT02150967

A Phase II Multicenter, Single Arm Study of Oral BGJ398 in Adult Patients with Advanced or Metastatic Cholangiocarcinoma with FGFR2 Gene Fusions or Other FGFR Genetic Alterations who Failed or are Intolerant to Platinum-based Chemotherapy.

Cancer type: Cholangiocarcinoma

Variant class: FGFR activating aberration

Other identifiers: 14-183, 14-2200, 14-328, 15452, 2014-0372, 2014-081, CBGJ398X2204, EudraCT Number: 2013-005085-19, NCI-2014-01310, REec-2014-1022, Trial 3GB-14-1

Population segments: FGFR, Fourth line or greater, Second line, Stage III, Stage IV, Third line

Phase: II

Therapy: infigratinib

Locations: Belgium, Germany, Singapore, Spain, Thailand, United Kingdom, United States

US States: CA, MA, MI, NY, OH, TX

Contact: QED Therapeutics [877-280-5655; clinicaltrials@QEDTx.com]

NCT04595747

Phase II Study of Rogaratinib (BAY 1163877) in the Treatment of Patients With Sarcoma Harboring Alterations in Fibroblast Growth Factor Receptor (FGFR) 1-4 and SDH-Deficient Gastrointestinal Stromal Tumor (GIST)

Cancer type: Soft Tissue Sarcoma

Variant class: FGFR3 aberration

Other identifiers: 10411, NCI-2020-08011

Population segments: FGFR, Second line, Stage III, Stage IV

Phase: II

Therapy: rogaratinib

Location: United States

US States: CA, MA, MD, NY

Contact: Multiple contacts: See www.clinicaltrials.gov for complete list of contacts.

Disclaimer: The data presented here is from a curated knowledgebase of publicly available information, but may not be exhaustive. The data version is 2021.11(003).

FGFR3 amplification (continued)

NCT04149691
A Phase I, Open-label, Multicentre, Dose Escalation Study to Assess Safety, Tolerability and Pharmacokinetics of Oral CPL304110, in Adult Subjects With Advanced Solid Malignancies

Cancer type: Bladder Cancer, Gastric Cancer, Non-Small Cell Lung Cancer

Variant class: FGFR3 aberration

Other identifier: 01FGFR2018

Population segments: FGFR, Line of therapy N/A, Squamous Cell, Stage III, Stage IV

Phase: I

Therapy: CPL-304-110

Location: Poland

NCT04601857
A Phase II Study Evaluating Futibatinib (TAS 120) Plus Pembrolizumab in the Treatment of Advanced or Metastatic Urothelial Carcinoma

Cancer type: Bladder Urothelial Carcinoma

Variant class: FGFR aberration

Other identifiers: EudraCT Number: 2020-000945-15, TAS-120-203

Population segments: FGFR, First line, Stage III, Stage IV

Phase: II

Therapies: futibatinib, pembrolizumab

Location: United States

US State: MI

Contact: Dr. Maciej Gil [609-250-7336; clinicaltrialinfo@taihooncology.com]

NCT03473743
A Phase Ib-II Study to Evaluate Safety, Efficacy, Pharmacokinetics, and Pharmacodynamics of Various Regimens of Erdafitinib in Subjects With Metastatic or Locally Advanced Urothelial Cancer

Cancer type: Bladder Urothelial Carcinoma

Variant class: FGFR aberration

Other identifiers: 17165, 2018-0142, 42756493BLC2002, CR108445, EudraCT Number: 2017-001980-19, NCI-2018-00997, NORSE, USO 17165, USOR 17165

Population segments: FGFR, First line, Fourth line or greater, Second line, Stage IV, Third line

Phase: I/II

Therapies: erdafitinib, cetrelimab

Locations: Belarus, Belgium, Brazil, France, Italy, Poland, Republic of Korea, Russian Federation, Spain, Taiwan, Turkey, United Kingdom, United States

US States: CO, MD, NC, NY, PA, TX, VA

Contact: Study Contact [844-434-4210; JNJ.CT@sylogent.com]

Disclaimer: The data presented here is from a curated knowledgebase of publicly available information, but may not be exhaustive. The data version is 2021.11(003).

FGFR3 amplification (continued)

NCT02393248
A Phase I/II, Open-Label, Dose-Escalation, Safety and Tolerability Study of INCB054828 in Subjects With Advanced Malignancies - (FIGHT-101)

Cancer type: Bladder Urothelial Carcinoma, Breast Cancer, Cholangiocarcinoma, Endometrial Carcinoma, Gastric Cancer, Non-Small Cell Lung Cancer, Solid Tumor

Variant class: FGFR aberration

Other identifiers: 00061567, 2014-1099, 201503012, EudraCT Number: 2016-002831-14, FIGHT-101, INCB 54828-101, INCB54828-101, MC# 15-03, NCI-2015-00611, OSU-15241

Population segments: FGFR, First line, Fourth line or greater, Second line, Squamous Cell, Stage III, Stage IV, Third line

Phase: I/II

Therapies: pemigatinib, pembrolizumab, trastuzumab, chemotherapy, retifanlimab

Location: United States

US States: FL, NJ, TX

Contact: Incyte Call Center [855-463-3463]

No NCT ID - see other identifier(s)
Phase I Clinical Study of BPI-17509 In Patients With Advanced Solid Tumors

Cancer type: Solid Tumor

Variant class: FGFR aberration

Other identifiers: BTP-26811, CTR20192140

Population segments: Second line, Stage III, Stage IV

Phase: I

Therapy: BPI-17509

Location: China

NCT04572295
An Open-label Phase Ib Study of E7090 Monotherapy and in Combination With Other Anticancer Agents in Subjects With ER+, HER2- Recurrent/Metastatic Breast Cancer

Cancer type: Breast Cancer

Variant class: FGFR aberration

Other inclusion criteria: ERBB2 negative, ER positive

Other identifiers: E7090-J081-102, JapicCTI-205429

Population segments: Estrogen receptor positive, HER2 negative, Second line, Stage IV

Phase: I

Therapies: E-7090, hormone therapy

Location: Japan

NCT04504331
A Phase IB Study of Infigratinib in Combination With Tamoxifen in Hormone Receptor Positive, HER2 Negative, FGFR Altered Advanced Breast Cancer

Cancer type: Breast Cancer

Variant class: FGFR aberration

Other inclusion criteria: ERBB2 negative, Hormone receptor positive

Other identifiers: BRS0113, K08CA252457

Population segments: Estrogen receptor positive, HER2 negative, Progesterone receptor positive, Second line, Stage III, Stage IV, Triple receptor negative

Phase: I

Therapies: infigratinib, hormone therapy

Location: United States

US State: CA

Contact: Allison K. Zhang [650-736-5790; axkong@stanford.edu]

Disclaimer: The data presented here is from a curated knowledgebase of publicly available information, but may not be exhaustive. The data version is 2021.11(003).

FGFR3 aberration

NCT04591431
The Rome Trial From Histology to Target: the Road to Personalize Target Therapy and Immunotherapy

Cancer type: Biliary Tract Carcinoma, Breast Cancer, Colorectal Cancer, Esophageal Cancer, Gastric Cancer, Gastroesophageal Junction Adenocarcinoma, Hepatocellular Carcinoma, Liver Cancer, Non-Small Cell Lung Cancer, Pancreatic Cancer, Unspecified Solid Tumor

Variant class: FGFR3 aberration

Other identifiers: EudraCT Number: 2018-002190-21, MAR-BAS-18-005, ROME

Population segments: Second line, Stage IV

Phase: II

Therapy: pemigatinib

Location: Italy

NCT03297606
Canadian Profiling and Targeted Agent Utilization Trial (CAPTUR): A Phase II Basket Trial

Cancer type: Unspecified Solid Tumor

Variant class: FGFR3 aberration

Other identifiers: CA209-9DL, CAPTUR, ESR-17-12831, ML39800, PM1, WI233446

Population segments: Aggressive, Diffuse large B-cell lymphoma (DLBCL), Extranodal marginal zone B-cell lymphoma (MALT), First line, Follicular lymphoma (FL), Indolent, Lymphoblastic lymphoma (LBL), Mantle cell lymphoma (MCL), Other subtype, Second line, Stage III, Stage IV, Waldenstrom's macroglobulinemia (WM)

Phase: II

Therapy: sunitinib

Location: Canada

NCT04565275
A Multi-center Open-label, Phase I/II Clinical Trial to Evaluate the Safety, Tolerability, Pharmacokinetics, and Pharmacodynamics of ICP-192 in Patients With Advanced Solid Tumors and FGFR Gene Alterations.

Cancer type: Bladder Urothelial Carcinoma, Cholangiocarcinoma, Unspecified Solid Tumor

Variant class: FGFR aberration

Other identifier: ICP-CL-00303

Population segments: FGFR, Second line, Stage III, Stage IV

Phase: I/II

Therapy: ICP-192

Location: United States

US States: AZ, CO, FL, MN

Contact: Olivia Yang [609-524-0684; olivia.yang@INNOCAREPHARMA.COM]

No NCT ID - see other identifier(s)
Phase I/II Study of TAS-117 In Combination With TAS-120 In Patients With Advanced Solid Tumors

Cancer type: Unspecified Solid Tumor

Variant class: FGFR aberration

Other identifier: JapicCTI-194864

Population segments: FGFR, First line, Squamous Cell, Stage III, Stage IV

Phase: I/II

Therapies: TAS-117, futibatinib

Location: Japan

FGFR3 aberration (continued)

No NCT ID - see other identifier(s)
A Phase Ib Study to Assess the Safety, Tolerability, and Efficacy of TAS-120 (Futibatinib) in Combination with MK-3475 (Pembrolizumab) in Patients with Solid Tumors.

Cancer type: Unspecified Solid Tumor

Variant class: FGFR aberration

Other identifier: JapicCTI-195063

Population segments: FGFR, Line of therapy N/A, Stage III, Stage IV

Phase: I

Therapies: futibatinib, pembrolizumab

Location: Japan

NCT04595747
Phase II Study of Rogaratinib (BAY 1163877) in the Treatment of Patients With Sarcoma Harboring Alterations in Fibroblast Growth Factor Receptor (FGFR) 1-4 and SDH-Deficient Gastrointestinal Stromal Tumor (GIST)

Cancer type: Soft Tissue Sarcoma

Variant class: FGFR3 aberration

Other identifiers: 10411, NCI-2020-08011

Population segments: FGFR, Second line, Stage III, Stage IV

Phase: II

Therapy: rogaratinib

Location: United States

US States: CA, MA, MD, NY

Contact: Multiple contacts: See www.clinicaltrials.gov for complete list of contacts.

NCT04149691
A Phase I, Open-label, Multicentre, Dose Escalation Study to Assess Safety, Tolerability and Pharmacokinetics of Oral CPL304110, in Adult Subjects With Advanced Solid Malignancies

Cancer type: Bladder Cancer, Gastric Cancer, Non-Small Cell Lung Cancer

Variant class: FGFR3 aberration

Other identifier: 01FGFR2018

Population segments: FGFR, Line of therapy N/A, Squamous Cell, Stage III, Stage IV

Phase: I

Therapy: CPL-304-110

Location: Poland

NCT04601857
A Phase II Study Evaluating Futibatinib (TAS 120) Plus Pembrolizumab in the Treatment of Advanced or Metastatic Urothelial Carcinoma

Cancer type: Bladder Urothelial Carcinoma

Variant class: FGFR aberration

Other identifiers: EudraCT Number: 2020-000945-15, TAS-120-203

Population segments: FGFR, First line, Stage III, Stage IV

Phase: II

Therapies: futibatinib, pembrolizumab

Location: United States

US State: MI

Contact: Dr. Maciej Gil [609-250-7336; clinicaltrialinfo@taihooncology.com]

Disclaimer: The data presented here is from a curated knowledgebase of publicly available information, but may not be exhaustive. The data version is 2021.11(003).

FGFR3 aberration (continued)

NCT03473743
A Phase Ib-II Study to Evaluate Safety, Efficacy, Pharmacokinetics, and Pharmacodynamics of Various Regimens of Erdafitinib in Subjects With Metastatic or Locally Advanced Urothelial Cancer

Cancer type: Bladder Urothelial Carcinoma

Variant class: FGFR aberration

Other identifiers: 17165, 2018-0142, 42756493BLC2002, CR108445, EudraCT Number: 2017-001980-19, NCI-2018-00997, NORSE, USO 17165, USOR 17165

Population segments: FGFR, First line, Fourth line or greater, Second line, Stage IV, Third line

Phase: I/II

Therapies: erdafitinib, cetrelimab

Locations: Belarus, Belgium, Brazil, France, Italy, Poland, Republic of Korea, Russian Federation, Spain, Taiwan, Turkey, United Kingdom, United States

US States: CO, MD, NC, NY, PA, TX, VA

Contact: Study Contact [844-434-4210; JNJ.CT@sylogent.com]

NCT02393248
A Phase I/II, Open-Label, Dose-Escalation, Safety and Tolerability Study of INCB054828 in Subjects With Advanced Malignancies - (FIGHT-101)

Cancer type: Bladder Urothelial Carcinoma, Breast Cancer, Cholangiocarcinoma, Endometrial Carcinoma, Gastric Cancer, Non-Small Cell Lung Cancer, Solid Tumor

Variant class: FGFR aberration

Other identifiers: 00061567, 2014-1099, 201503012, EudraCT Number: 2016-002831-14, FIGHT-101, INCB 54828-101, INCB54828-101, MC# 15-03, NCI-2015-00611, OSU-15241

Population segments: FGFR, First line, Fourth line or greater, Second line, Squamous Cell, Stage III, Stage IV, Third line

Phase: I/II

Therapies: pemigatinib, pembrolizumab, trastuzumab, chemotherapy, retifanlimab

Location: United States

US States: FL, NJ, TX

Contact: Incyte Call Center [855-463-3463]

No NCT ID - see other identifier(s)
Phase I Clinical Study of BPI-17509 In Patients With Advanced Solid Tumors

Cancer type: Solid Tumor

Variant class: FGFR aberration

Other identifiers: BTP-26811, CTR20192140

Population segments: Second line, Stage III, Stage IV

Phase: I

Therapy: BPI-17509

Location: China

Disclaimer: The data presented here is from a curated knowledgebase of publicly available information, but may not be exhaustive. The data version is 2021.11(003).

FGFR3 aberration (continued)

NCT04572295
An Open-label Phase Ib Study of E7090 Monotherapy and in Combination With Other Anticancer Agents in Subjects With ER+, HER2- Recurrent/Metastatic Breast Cancer

Cancer type: Breast Cancer

Variant class: FGFR aberration

Other inclusion criteria: ERBB2 negative, ER positive

Other identifiers: E7090-J081-102, JapicCTI-205429

Population segments: Estrogen receptor positive, HER2 negative, Second line, Stage IV

Phase: I

Therapies: E-7090, hormone therapy

Location: Japan

NCT04504331
A Phase IB Study of Infigratinib in Combination With Tamoxifen in Hormone Receptor Positive, HER2 Negative, FGFR Altered Advanced Breast Cancer

Cancer type: Breast Cancer

Variant class: FGFR aberration

Other inclusion criteria: ERBB2 negative, Hormone receptor positive

Other identifiers: BRS0113, K08CA252457

Population segments: Estrogen receptor positive, HER2 negative, Progesterone receptor positive, Second line, Stage III, Stage IV, Triple receptor negative

Phase: I

Therapies: infigratinib, hormone therapy

Location: United States

US State: CA

Contact: Allison K. Zhang [650-736-5790; axkong@stanford.edu]

Relevant Biomarkers

Genomic Alteration	Relevant Therapies (In this cancer type)	Relevant Therapies (In other cancer type)	Clinical Trials
FGFR3 G370C mutation<br>**Prognostic significance:** None<br>**Diagnostic significance:** None	None	**erdafitinib** [1]	35
FGFR3 R248C mutation<br>**Prognostic significance:** None<br>**Diagnostic significance:** None	None	**erdafitinib** [1]	35
FGFR3 S249C mutation<br>**Prognostic significance:** None<br>**Diagnostic significance:** None	None	**erdafitinib** [1]	35
FGFR3-TACC3 fusion<br>**Prognostic significance:** None<br>**Diagnostic significance:** None	None	**erdafitinib** [1]	31
FGFR3-BAIAP2L1 fusion<br>**Prognostic significance:** None<br>**Diagnostic significance:** None	None	**erdafitinib** [1]	30

Public data sources included in relevant therapies: FDA1, NCCN, **EMA**2, ESMO
Public data sources included in prognostic and diagnostic significance: NCCN, ESMO

Disclaimer: The data presented here is from a curated knowledgebase of publicly available information, but may not be exhaustive. The data version is 2021.11(003).

Relevant Biomarkers (continued)

Genomic Alteration	Relevant Therapies (In this cancer type)	Relevant Therapies (In other cancer type)	Clinical Trials
FGFR3 Y373C mutation<br>**Prognostic significance:** None<br>**Diagnostic significance:** None	None	**erdafitinib** [1]	29
FGFR3 K650E mutation<br>**Prognostic significance:** None<br>**Diagnostic significance:** None	None	erdafitinib	33
FGFR3 K650M mutation<br>**Prognostic significance:** None<br>**Diagnostic significance:** None	None	erdafitinib	32
FGFR3 K650Q mutation<br>**Prognostic significance:** None<br>**Diagnostic significance:** None	None	erdafitinib	32
FGFR3 K650T mutation<br>**Prognostic significance:** None<br>**Diagnostic significance:** None	None	erdafitinib	32
FGFR3 activating mutation<br>**Prognostic significance:** None<br>**Diagnostic significance:** None	None	erdafitinib	31
FGFR3 K652E mutation<br>**Prognostic significance:** None<br>**Diagnostic significance:** None	None	erdafitinib	31
FGFR3 fusion<br>**Prognostic significance:** None<br>**Diagnostic significance:** None	None	erdafitinib	27
FGFR3 A391E mutation<br>**Prognostic significance:** None<br>**Diagnostic significance:** None	None	erdafitinib	26
FGFR3 KD mutation<br>**Prognostic significance:** None<br>**Diagnostic significance:** None	None	erdafitinib	25
FGFR3 mutation<br>**Prognostic significance:** None<br>**Diagnostic significance:** None	None	erdafitinib	25
FGFR3 V555 mutation<br>**Prognostic significance:** None<br>**Diagnostic significance:** None	None	erdafitinib	25
FGFR3 V557 mutation<br>**Prognostic significance:** None<br>**Diagnostic significance:** None	None	erdafitinib	25

Public data sources included in relevant therapies: FDA[1], NCCN, **EMA**[2], ESMO
Public data sources included in prognostic and diagnostic significance: NCCN, ESMO

Disclaimer: The data presented here is from a curated knowledgebase of publicly available information, but may not be exhaustive. The data version is 2021.11(003).

Relevant Biomarkers (continued)

Genomic Alteration	Relevant Therapies (In this cancer type)	Relevant Therapies (In other cancer type)	Clinical Trials
FGFR3 amplification<br>**Prognostic significance:** None<br>**Diagnostic significance:** None	None	None	21
FGFR3 aberration<br>**Prognostic significance:** None<br>**Diagnostic significance:** None	None	None	13

Public data sources included in relevant therapies: FDA[1], NCCN, **EMA**[2], ESMO
Public data sources included in prognostic and diagnostic significance: NCCN, ESMO

Relevant Therapy Summary

● In this cancer type ○ In other cancer type ◐ In this cancer type and other cancer types ✖ No evidence

FGFR3 G370C mutation

Relevant Therapy	FDA	NCCN	EMA	ESMO	Clinical Trials*
erdafitinib	○	○	✖	○	◐ (II)
infigratinib	✖	✖	✖	✖	◐ (III)
futibatinib, pembrolizumab	✖	✖	✖	✖	◐ (II)
ICP-192	✖	✖	✖	✖	◐ (II)
pemigatinib	✖	✖	✖	✖	◐ (II)
anlotinib hydrochloride	✖	✖	✖	✖	◐ (I/II)
futibatinib	✖	✖	✖	✖	● (II)
sunitinib	✖	✖	✖	✖	● (II)
TAS-117, futibatinib	✖	✖	✖	✖	● (I/II)
erdafitinib, pembrolizumab	✖	✖	✖	✖	○ (III)
durvalumab, olaparib, hormone therapy	✖	✖	✖	✖	○ (II)
hormone therapy, anlotinib hydrochloride	✖	✖	✖	✖	○ (II)
rogaratinib	✖	✖	✖	✖	○ (II)
rogaratinib, chemotherapy	✖	✖	✖	✖	○ (II)
derazantinib, atezolizumab	✖	✖	✖	✖	○ (I/II)
erdafitinib, cetrelimab	✖	✖	✖	✖	○ (I/II)
pemigatinib, pembrolizumab, trastuzumab, chemotherapy, retifanlimab	✖	✖	✖	✖	○ (I/II)
BPI-17509	✖	✖	✖	✖	○ (I)
CPL-304-110	✖	✖	✖	✖	○ (I)

* Most advanced phase (IV, III, II/III, II, I/II, I) is shown and multiple clinical trials may be available.

Disclaimer: The data presented here is from a curated knowledgebase of publicly available information, but may not be exhaustive. The data version is 2021.11(003).

Relevant Therapy Summary (continued)

● In this cancer type ○ In other cancer type ◐ In this cancer type and other cancer types ✖ No evidence

FGFR3 G370C mutation (continued)

Relevant Therapy	FDA	NCCN	EMA	ESMO	Clinical Trials*
E-7090, hormone therapy	✖	✖	✖	✖	○ (I)
infigratinib, hormone therapy	✖	✖	✖	✖	○ (I)

FGFR3 R248C mutation

Relevant Therapy	FDA	NCCN	EMA	ESMO	Clinical Trials*
erdafitinib	○	○	✖	○	◐ (II)
infigratinib	✖	✖	✖	✖	◐ (III)
futibatinib, pembrolizumab	✖	✖	✖	✖	◐ (II)
ICP-192	✖	✖	✖	✖	◐ (II)
pemigatinib	✖	✖	✖	✖	◐ (II)
anlotinib hydrochloride	✖	✖	✖	✖	◐ (I/II)
futibatinib	✖	✖	✖	✖	● (II)
sunitinib	✖	✖	✖	✖	● (II)
TAS-117, futibatinib	✖	✖	✖	✖	● (I/II)
erdafitinib, pembrolizumab	✖	✖	✖	✖	○ (III)
durvalumab, olaparib, hormone therapy	✖	✖	✖	✖	○ (II)
hormone therapy, anlotinib hydrochloride	✖	✖	✖	✖	○ (II)
rogaratinib	✖	✖	✖	✖	○ (II)
rogaratinib, chemotherapy	✖	✖	✖	✖	○ (II)
derazantinib, atezolizumab	✖	✖	✖	✖	○ (I/II)
erdafitinib, cetrelimab	✖	✖	✖	✖	○ (I/II)
pemigatinib, pembrolizumab, trastuzumab, chemotherapy, retifanlimab	✖	✖	✖	✖	○ (I/II)
BPI-17509	✖	✖	✖	✖	○ (I)
CPL-304-110	✖	✖	✖	✖	○ (I)
E-7090, hormone therapy	✖	✖	✖	✖	○ (I)
infigratinib, hormone therapy	✖	✖	✖	✖	○ (I)

* Most advanced phase (IV, III, II/III, II, I/II, I) is shown and multiple clinical trials may be available.

Disclaimer: The data presented here is from a curated knowledgebase of publicly available information, but may not be exhaustive. The data version is 2021.11(003).

Relevant Therapy Summary (continued)

● In this cancer type ○ In other cancer type ◐ In this cancer type and other cancer types ✖ No evidence

FGFR3 S249C mutation

Relevant Therapy	FDA	NCCN	EMA	ESMO	Clinical Trials*
erdafitinib	○	○	✖	○	◐ (II)
infigratinib	✖	✖	✖	✖	◐ (III)
futibatinib, pembrolizumab	✖	✖	✖	✖	◐ (II)
ICP-192	✖	✖	✖	✖	◐ (II)
pemigatinib	✖	✖	✖	✖	◐ (II)
anlotinib hydrochloride	✖	✖	✖	✖	◐ (I/II)
futibatinib	✖	✖	✖	✖	● (II)
sunitinib	✖	✖	✖	✖	● (II)
TAS-117, futibatinib	✖	✖	✖	✖	● (I/II)
erdafitinib, pembrolizumab	✖	✖	✖	✖	○ (III)
durvalumab, olaparib, hormone therapy	✖	✖	✖	✖	○ (II)
hormone therapy, anlotinib hydrochloride	✖	✖	✖	✖	○ (II)
rogaratinib	✖	✖	✖	✖	○ (II)
rogaratinib, chemotherapy	✖	✖	✖	✖	○ (II)
derazantinib, atezolizumab	✖	✖	✖	✖	○ (I/II)
erdafitinib, cetrelimab	✖	✖	✖	✖	○ (I/II)
pemigatinib, pembrolizumab, trastuzumab, chemotherapy, retifanlimab	✖	✖	✖	✖	○ (I/II)
BPI-17509	✖	✖	✖	✖	○ (I)
CPL-304-110	✖	✖	✖	✖	○ (I)
E-7090, hormone therapy	✖	✖	✖	✖	○ (I)
infigratinib, hormone therapy	✖	✖	✖	✖	○ (I)

FGFR3-TACC3 fusion

Relevant Therapy	FDA	NCCN	EMA	ESMO	Clinical Trials*
erdafitinib	○	○	✖	○	○ (II)
infigratinib	✖	✖	✖	✖	◐ (III)
futibatinib, pembrolizumab	✖	✖	✖	✖	◐ (II)
ICP-192	✖	✖	✖	✖	◐ (II)

* Most advanced phase (IV, III, II/III, II, I/II, I) is shown and multiple clinical trials may be available.

Disclaimer: The data presented here is from a curated knowledgebase of publicly available information, but may not be exhaustive. The data version is 2021.11(003).

Relevant Therapy Summary (continued)

● In this cancer type ○ In other cancer type ◐ In this cancer type and other cancer types ✕ No evidence

FGFR3-TACC3 fusion (continued)

Relevant Therapy	FDA	NCCN	EMA	ESMO	Clinical Trials*
pemigatinib	✕	✕	✕	✕	◐ (II)
futibatinib	✕	✕	✕	✕	● (II)
sunitinib	✕	✕	✕	✕	● (II)
TAS-117, futibatinib	✕	✕	✕	✕	● (I/II)
anlotinib hydrochloride	✕	✕	✕	✕	● (I)
erdafitinib, pembrolizumab	✕	✕	✕	✕	○ (III)
hormone therapy, anlotinib hydrochloride	✕	✕	✕	✕	○ (II)
rogaratinib	✕	✕	✕	✕	○ (II)
rogaratinib, chemotherapy	✕	✕	✕	✕	○ (II)
derazantinib, atezolizumab	✕	✕	✕	✕	○ (I/II)
erdafitinib, cetrelimab	✕	✕	✕	✕	○ (I/II)
pemigatinib, pembrolizumab, trastuzumab, chemotherapy, retifanlimab	✕	✕	✕	✕	○ (I/II)
BPI-17509	✕	✕	✕	✕	○ (I)
CPL-304-110	✕	✕	✕	✕	○ (I)
E-7090, hormone therapy	✕	✕	✕	✕	○ (I)
infigratinib, hormone therapy	✕	✕	✕	✕	○ (I)

FGFR3-BAIAP2L1 fusion

Relevant Therapy	FDA	NCCN	EMA	ESMO	Clinical Trials*
erdafitinib	○	○	✕	○	○ (II)
infigratinib	✕	✕	✕	✕	◐ (III)
futibatinib, pembrolizumab	✕	✕	✕	✕	◐ (II)
ICP-192	✕	✕	✕	✕	◐ (II)
pemigatinib	✕	✕	✕	✕	◐ (II)
futibatinib	✕	✕	✕	✕	● (II)
sunitinib	✕	✕	✕	✕	● (II)
TAS-117, futibatinib	✕	✕	✕	✕	● (I/II)
anlotinib hydrochloride	✕	✕	✕	✕	● (I)

* Most advanced phase (IV, III, II/III, II, I/II, I) is shown and multiple clinical trials may be available.

Disclaimer: The data presented here is from a curated knowledgebase of publicly available information, but may not be exhaustive. The data version is 2021.11(003).

Relevant Therapy Summary (continued)

● In this cancer type ○ In other cancer type ◐ In this cancer type and other cancer types ✖ No evidence

FGFR3-BAIAP2L1 fusion (continued)

Relevant Therapy	FDA	NCCN	EMA	ESMO	Clinical Trials*
erdafitinib, pembrolizumab	✖	✖	✖	✖	○ (III)
hormone therapy, anlotinib hydrochloride	✖	✖	✖	✖	○ (II)
rogaratinib	✖	✖	✖	✖	○ (II)
rogaratinib, chemotherapy	✖	✖	✖	✖	○ (II)
derazantinib, atezolizumab	✖	✖	✖	✖	○ (I/II)
erdafitinib, cetrelimab	✖	✖	✖	✖	○ (I/II)
pemigatinib, pembrolizumab, trastuzumab, chemotherapy, retifanlimab	✖	✖	✖	✖	○ (I/II)
BPI-17509	✖	✖	✖	✖	○ (I)
CPL-304-110	✖	✖	✖	✖	○ (I)
E-7090, hormone therapy	✖	✖	✖	✖	○ (I)
infigratinib, hormone therapy	✖	✖	✖	✖	○ (I)

FGFR3 Y373C mutation

Relevant Therapy	FDA	NCCN	EMA	ESMO	Clinical Trials*
erdafitinib	○	○	✖	○	◐ (II)
futibatinib, pembrolizumab	✖	✖	✖	✖	◐ (II)
ICP-192	✖	✖	✖	✖	◐ (II)
pemigatinib	✖	✖	✖	✖	◐ (II)
anlotinib hydrochloride	✖	✖	✖	✖	◐ (I/II)
futibatinib	✖	✖	✖	✖	● (II)
sunitinib	✖	✖	✖	✖	● (II)
TAS-117, futibatinib	✖	✖	✖	✖	● (I/II)
erdafitinib, pembrolizumab	✖	✖	✖	✖	○ (III)
infigratinib	✖	✖	✖	✖	○ (III)
durvalumab, olaparib, hormone therapy	✖	✖	✖	✖	○ (II)
rogaratinib	✖	✖	✖	✖	○ (II)
rogaratinib, chemotherapy	✖	✖	✖	✖	○ (II)
erdafitinib, cetrelimab	✖	✖	✖	✖	○ (I/II)

* Most advanced phase (IV, III, II/III, II, I/II, I) is shown and multiple clinical trials may be available.

Disclaimer: The data presented here is from a curated knowledgebase of publicly available information, but may not be exhaustive. The data version is 2021.11(003).

Relevant Therapy Summary (continued)

● In this cancer type ○ In other cancer type ◐ In this cancer type and other cancer types ✖ No evidence

FGFR3 Y373C mutation (continued)

Relevant Therapy	FDA	NCCN	EMA	ESMO	Clinical Trials*
pemigatinib, pembrolizumab, trastuzumab, chemotherapy, retifanlimab	✖	✖	✖	✖	○ (I/II)
BPI-17509	✖	✖	✖	✖	○ (I)
CPL-304-110	✖	✖	✖	✖	○ (I)
E-7090, hormone therapy	✖	✖	✖	✖	○ (I)
infigratinib, hormone therapy	✖	✖	✖	✖	○ (I)

FGFR3 K650E mutation

Relevant Therapy	FDA	NCCN	EMA	ESMO	Clinical Trials*
erdafitinib	✖	✖	✖	○	◐ (II)
infigratinib	✖	✖	✖	✖	◐ (III)
futibatinib, pembrolizumab	✖	✖	✖	✖	◐ (II)
pemigatinib	✖	✖	✖	✖	◐ (II)
anlotinib hydrochloride	✖	✖	✖	✖	◐ (I/II)
futibatinib	✖	✖	✖	✖	● (II)
sunitinib	✖	✖	✖	✖	● (II)
ICP-192	✖	✖	✖	✖	● (I/II)
TAS-117, futibatinib	✖	✖	✖	✖	● (I/II)
durvalumab, olaparib, hormone therapy	✖	✖	✖	✖	○ (II)
hormone therapy, anlotinib hydrochloride	✖	✖	✖	✖	○ (II)
rogaratinib	✖	✖	✖	✖	○ (II)
rogaratinib, chemotherapy	✖	✖	✖	✖	○ (II)
derazantinib, atezolizumab	✖	✖	✖	✖	○ (I/II)
erdafitinib, cetrelimab	✖	✖	✖	✖	○ (I/II)
pemigatinib, pembrolizumab, trastuzumab, chemotherapy, retifanlimab	✖	✖	✖	✖	○ (I/II)
BPI-17509	✖	✖	✖	✖	○ (I)
CPL-304-110	✖	✖	✖	✖	○ (I)
E-7090, hormone therapy	✖	✖	✖	✖	○ (I)
infigratinib, hormone therapy	✖	✖	✖	✖	○ (I)

* Most advanced phase (IV, III, II/III, II, I/II, I) is shown and multiple clinical trials may be available.

Disclaimer: The data presented here is from a curated knowledgebase of publicly available information, but may not be exhaustive. The data version is 2021.11(003).

Relevant Therapy Summary (continued)

● In this cancer type ○ In other cancer type ◐ In this cancer type and other cancer types ✖ No evidence

FGFR3 K650M mutation

Relevant Therapy	FDA	NCCN	EMA	ESMO	Clinical Trials*
erdafitinib	✖	✖	✖	○	◐ (II)
infigratinib	✖	✖	✖	✖	◐ (III)
futibatinib, pembrolizumab	✖	✖	✖	✖	◐ (II)
pemigatinib	✖	✖	✖	✖	◐ (II)
anlotinib hydrochloride	✖	✖	✖	✖	◐ (I/II)
futibatinib	✖	✖	✖	✖	● (II)
sunitinib	✖	✖	✖	✖	● (II)
ICP-192	✖	✖	✖	✖	● (I/II)
TAS-117, futibatinib	✖	✖	✖	✖	● (I/II)
durvalumab, olaparib, hormone therapy	✖	✖	✖	✖	○ (II)
hormone therapy, anlotinib hydrochloride	✖	✖	✖	✖	○ (II)
rogaratinib	✖	✖	✖	✖	○ (II)
rogaratinib, chemotherapy	✖	✖	✖	✖	○ (II)
derazantinib, atezolizumab	✖	✖	✖	✖	○ (I/II)
erdafitinib, cetrelimab	✖	✖	✖	✖	○ (I/II)
pemigatinib, pembrolizumab, trastuzumab, chemotherapy, retifanlimab	✖	✖	✖	✖	○ (I/II)
BPI-17509	✖	✖	✖	✖	○ (I)
CPL-304-110	✖	✖	✖	✖	○ (I)
E-7090, hormone therapy	✖	✖	✖	✖	○ (I)
infigratinib, hormone therapy	✖	✖	✖	✖	○ (I)

FGFR3 K650Q mutation

Relevant Therapy	FDA	NCCN	EMA	ESMO	Clinical Trials*
erdafitinib	✖	✖	✖	○	◐ (II)
infigratinib	✖	✖	✖	✖	◐ (III)
futibatinib, pembrolizumab	✖	✖	✖	✖	◐ (II)
pemigatinib	✖	✖	✖	✖	◐ (II)
anlotinib hydrochloride	✖	✖	✖	✖	◐ (I/II)

* Most advanced phase (IV, III, II/III, II, I/II, I) is shown and multiple clinical trials may be available.

Disclaimer: The data presented here is from a curated knowledgebase of publicly available information, but may not be exhaustive. The data version is 2021.11(003).

Relevant Therapy Summary (continued)

● In this cancer type ○ In other cancer type ◐ In this cancer type and other cancer types ✖ No evidence

FGFR3 K650Q mutation (continued)

Relevant Therapy	FDA	NCCN	EMA	ESMO	Clinical Trials*
futibatinib	✖	✖	✖	✖	● (II)
sunitinib	✖	✖	✖	✖	● (II)
ICP-192	✖	✖	✖	✖	● (I/II)
TAS-117, futibatinib	✖	✖	✖	✖	● (I/II)
durvalumab, olaparib, hormone therapy	✖	✖	✖	✖	○ (II)
hormone therapy, anlotinib hydrochloride	✖	✖	✖	✖	○ (II)
rogaratinib	✖	✖	✖	✖	○ (II)
rogaratinib, chemotherapy	✖	✖	✖	✖	○ (II)
derazantinib, atezolizumab	✖	✖	✖	✖	○ (I/II)
erdafitinib, cetrelimab	✖	✖	✖	✖	○ (I/II)
pemigatinib, pembrolizumab, trastuzumab, chemotherapy, retifanlimab	✖	✖	✖	✖	○ (I/II)
BPI-17509	✖	✖	✖	✖	○ (I)
CPL-304-110	✖	✖	✖	✖	○ (I)
E-7090, hormone therapy	✖	✖	✖	✖	○ (I)
infigratinib, hormone therapy	✖	✖	✖	✖	○ (I)

FGFR3 K650T mutation

Relevant Therapy	FDA	NCCN	EMA	ESMO	Clinical Trials*
erdafitinib	✖	✖	✖	○	◐ (II)
infigratinib	✖	✖	✖	✖	◐ (III)
futibatinib, pembrolizumab	✖	✖	✖	✖	◐ (II)
pemigatinib	✖	✖	✖	✖	◐ (II)
anlotinib hydrochloride	✖	✖	✖	✖	◐ (I/II)
futibatinib	✖	✖	✖	✖	● (II)
sunitinib	✖	✖	✖	✖	● (II)
ICP-192	✖	✖	✖	✖	● (I/II)
TAS-117, futibatinib	✖	✖	✖	✖	● (I/II)
durvalumab, olaparib, hormone therapy	✖	✖	✖	✖	○ (II)

* Most advanced phase (IV, III, II/III, II, I/II, I) is shown and multiple clinical trials may be available.

Disclaimer: The data presented here is from a curated knowledgebase of publicly available information, but may not be exhaustive. The data version is 2021.11(003).

Relevant Therapy Summary (continued)

● In this cancer type ○ In other cancer type ◐ In this cancer type and other cancer types ✖ No evidence

FGFR3 K650T mutation (continued)

Relevant Therapy	FDA	NCCN	EMA	ESMO	Clinical Trials*
hormone therapy, anlotinib hydrochloride	✖	✖	✖	✖	○ (II)
rogaratinib	✖	✖	✖	✖	○ (II)
rogaratinib, chemotherapy	✖	✖	✖	✖	○ (II)
derazantinib, atezolizumab	✖	✖	✖	✖	○ (I/II)
erdafitinib, cetrelimab	✖	✖	✖	✖	○ (I/II)
pemigatinib, pembrolizumab, trastuzumab, chemotherapy, retifanlimab	✖	✖	✖	✖	○ (I/II)
BPI-17509	✖	✖	✖	✖	○ (I)
CPL-304-110	✖	✖	✖	✖	○ (I)
E-7090, hormone therapy	✖	✖	✖	✖	○ (I)
infigratinib, hormone therapy	✖	✖	✖	✖	○ (I)

FGFR3 activating mutation

Relevant Therapy	FDA	NCCN	EMA	ESMO	Clinical Trials*
erdafitinib	✖	✖	✖	○	◐ (II)
futibatinib, pembrolizumab	✖	✖	✖	✖	◐ (II)
infigratinib	✖	✖	✖	✖	◐ (II)
pemigatinib	✖	✖	✖	✖	◐ (II)
anlotinib hydrochloride	✖	✖	✖	✖	◐ (I/II)
futibatinib	✖	✖	✖	✖	● (II)
sunitinib	✖	✖	✖	✖	● (II)
ICP-192	✖	✖	✖	✖	● (I/II)
TAS-117, futibatinib	✖	✖	✖	✖	● (I/II)
durvalumab, olaparib, hormone therapy	✖	✖	✖	✖	○ (II)
hormone therapy, anlotinib hydrochloride	✖	✖	✖	✖	○ (II)
rogaratinib	✖	✖	✖	✖	○ (II)
rogaratinib, chemotherapy	✖	✖	✖	✖	○ (II)
derazantinib, atezolizumab	✖	✖	✖	✖	○ (I/II)
erdafitinib, cetrelimab	✖	✖	✖	✖	○ (I/II)

* Most advanced phase (IV, III, II/III, II, I/II, I) is shown and multiple clinical trials may be available.

Disclaimer: The data presented here is from a curated knowledgebase of publicly available information, but may not be exhaustive. The data version is 2021.11(003).

Relevant Therapy Summary (continued)

● In this cancer type ○ In other cancer type ◐ In this cancer type and other cancer types ✖ No evidence

FGFR3 activating mutation (continued)

Relevant Therapy	FDA	NCCN	EMA	ESMO	Clinical Trials*
pemigatinib, pembrolizumab, trastuzumab, chemotherapy, retifanlimab	✖	✖	✖	✖	○ (I/II)
BPI-17509	✖	✖	✖	✖	○ (I)
CPL-304-110	✖	✖	✖	✖	○ (I)
E-7090, hormone therapy	✖	✖	✖	✖	○ (I)
infigratinib, hormone therapy	✖	✖	✖	✖	○ (I)

FGFR3 K652E mutation

Relevant Therapy	FDA	NCCN	EMA	ESMO	Clinical Trials*
erdafitinib	✖	✖	✖	○	◐ (II)
futibatinib, pembrolizumab	✖	✖	✖	✖	◐ (II)
infigratinib	✖	✖	✖	✖	◐ (II)
pemigatinib	✖	✖	✖	✖	◐ (II)
anlotinib hydrochloride	✖	✖	✖	✖	◐ (I/II)
futibatinib	✖	✖	✖	✖	● (II)
sunitinib	✖	✖	✖	✖	● (II)
ICP-192	✖	✖	✖	✖	● (I/II)
TAS-117, futibatinib	✖	✖	✖	✖	● (I/II)
durvalumab, olaparib, hormone therapy	✖	✖	✖	✖	○ (II)
hormone therapy, anlotinib hydrochloride	✖	✖	✖	✖	○ (II)
rogaratinib	✖	✖	✖	✖	○ (II)
rogaratinib, chemotherapy	✖	✖	✖	✖	○ (II)
derazantinib, atezolizumab	✖	✖	✖	✖	○ (I/II)
erdafitinib, cetrelimab	✖	✖	✖	✖	○ (I/II)
pemigatinib, pembrolizumab, trastuzumab, chemotherapy, retifanlimab	✖	✖	✖	✖	○ (I/II)
BPI-17509	✖	✖	✖	✖	○ (I)
CPL-304-110	✖	✖	✖	✖	○ (I)
E-7090, hormone therapy	✖	✖	✖	✖	○ (I)
infigratinib, hormone therapy	✖	✖	✖	✖	○ (I)

* Most advanced phase (IV, III, II/III, II, I/II, I) is shown and multiple clinical trials may be available.

Disclaimer: The data presented here is from a curated knowledgebase of publicly available information, but may not be exhaustive. The data version is 2021.11(003).

Relevant Therapy Summary (continued)

● In this cancer type ○ In other cancer type ◐ In this cancer type and other cancer types ✖ No evidence

FGFR3 fusion

Relevant Therapy	FDA	NCCN	EMA	ESMO	Clinical Trials*
erdafitinib	✖	✖	✖	○	○ (II)
infigratinib	✖	✖	✖	✖	◐ (III)
futibatinib, pembrolizumab	✖	✖	✖	✖	◐ (II)
pemigatinib	✖	✖	✖	✖	◐ (II)
futibatinib	✖	✖	✖	✖	● (II)
sunitinib	✖	✖	✖	✖	● (II)
ICP-192	✖	✖	✖	✖	● (I/II)
TAS-117, futibatinib	✖	✖	✖	✖	● (I/II)
anlotinib hydrochloride	✖	✖	✖	✖	● (I)
hormone therapy, anlotinib hydrochloride	✖	✖	✖	✖	○ (II)
rogaratinib	✖	✖	✖	✖	○ (II)
rogaratinib, chemotherapy	✖	✖	✖	✖	○ (II)
derazantinib, atezolizumab	✖	✖	✖	✖	○ (I/II)
erdafitinib, cetrelimab	✖	✖	✖	✖	○ (I/II)
pemigatinib, pembrolizumab, trastuzumab, chemotherapy, retifanlimab	✖	✖	✖	✖	○ (I/II)
BPI-17509	✖	✖	✖	✖	○ (I)
CPL-304-110	✖	✖	✖	✖	○ (I)
E-7090, hormone therapy	✖	✖	✖	✖	○ (I)
infigratinib, hormone therapy	✖	✖	✖	✖	○ (I)

FGFR3 A391E mutation

Relevant Therapy	FDA	NCCN	EMA	ESMO	Clinical Trials*
erdafitinib	✖	✖	✖	○	◐ (II)
futibatinib, pembrolizumab	✖	✖	✖	✖	◐ (II)
pemigatinib	✖	✖	✖	✖	◐ (II)
anlotinib hydrochloride	✖	✖	✖	✖	◐ (I/II)
futibatinib	✖	✖	✖	✖	● (II)
sunitinib	✖	✖	✖	✖	● (II)

* Most advanced phase (IV, III, II/III, II, I/II, I) is shown and multiple clinical trials may be available.

Disclaimer: The data presented here is from a curated knowledgebase of publicly available information, but may not be exhaustive. The data version is 2021.11(003).

Relevant Therapy Summary (continued)

● In this cancer type ○ In other cancer type ◐ In this cancer type and other cancer types ✖ No evidence

FGFR3 A391E mutation (continued)

Relevant Therapy	FDA	NCCN	EMA	ESMO	Clinical Trials*
ICP-192	✖	✖	✖	✖	● (I/II)
TAS-117, futibatinib	✖	✖	✖	✖	● (I/II)
infigratinib	✖	✖	✖	✖	○ (III)
durvalumab, olaparib, hormone therapy	✖	✖	✖	✖	○ (II)
rogaratinib	✖	✖	✖	✖	○ (II)
rogaratinib, chemotherapy	✖	✖	✖	✖	○ (II)
erdafitinib, cetrelimab	✖	✖	✖	✖	○ (I/II)
pemigatinib, pembrolizumab, trastuzumab, chemotherapy, retifanlimab	✖	✖	✖	✖	○ (I/II)
BPI-17509	✖	✖	✖	✖	○ (I)
CPL-304-110	✖	✖	✖	✖	○ (I)
E-7090, hormone therapy	✖	✖	✖	✖	○ (I)
infigratinib, hormone therapy	✖	✖	✖	✖	○ (I)

FGFR3 KD mutation

Relevant Therapy	FDA	NCCN	EMA	ESMO	Clinical Trials*
erdafitinib	✖	✖	✖	○	◐ (II)
futibatinib, pembrolizumab	✖	✖	✖	✖	◐ (II)
pemigatinib	✖	✖	✖	✖	◐ (II)
anlotinib hydrochloride	✖	✖	✖	✖	◐ (I/II)
futibatinib	✖	✖	✖	✖	● (II)
sunitinib	✖	✖	✖	✖	● (II)
ICP-192	✖	✖	✖	✖	● (I/II)
TAS-117, futibatinib	✖	✖	✖	✖	● (I/II)
durvalumab, olaparib, hormone therapy	✖	✖	✖	✖	○ (II)
rogaratinib	✖	✖	✖	✖	○ (II)
rogaratinib, chemotherapy	✖	✖	✖	✖	○ (II)
erdafitinib, cetrelimab	✖	✖	✖	✖	○ (I/II)
pemigatinib, pembrolizumab, trastuzumab, chemotherapy, retifanlimab	✖	✖	✖	✖	○ (I/II)

* Most advanced phase (IV, III, II/III, II, I/II, I) is shown and multiple clinical trials may be available.

Disclaimer: The data presented here is from a curated knowledgebase of publicly available information, but may not be exhaustive. The data version is 2021.11(003).

Relevant Therapy Summary (continued)

● In this cancer type ○ In other cancer type ◐ In this cancer type and other cancer types ✖ No evidence

FGFR3 KD mutation (continued)

Relevant Therapy	FDA	NCCN	EMA	ESMO	Clinical Trials*
BPI-17509	✖	✖	✖	✖	○ (I)
CPL-304-110	✖	✖	✖	✖	○ (I)
E-7090, hormone therapy	✖	✖	✖	✖	○ (I)
infigratinib, hormone therapy	✖	✖	✖	✖	○ (I)

FGFR3 mutation

Relevant Therapy	FDA	NCCN	EMA	ESMO	Clinical Trials*
erdafitinib	✖	✖	✖	○	◐ (II)
futibatinib, pembrolizumab	✖	✖	✖	✖	◐ (II)
pemigatinib	✖	✖	✖	✖	◐ (II)
anlotinib hydrochloride	✖	✖	✖	✖	◐ (I/II)
futibatinib	✖	✖	✖	✖	● (II)
sunitinib	✖	✖	✖	✖	● (II)
ICP-192	✖	✖	✖	✖	● (I/II)
TAS-117, futibatinib	✖	✖	✖	✖	● (I/II)
durvalumab, olaparib, hormone therapy	✖	✖	✖	✖	○ (II)
rogaratinib	✖	✖	✖	✖	○ (II)
rogaratinib, chemotherapy	✖	✖	✖	✖	○ (II)
erdafitinib, cetrelimab	✖	✖	✖	✖	○ (I/II)
pemigatinib, pembrolizumab, trastuzumab, chemotherapy, retifanlimab	✖	✖	✖	✖	○ (I/II)
BPI-17509	✖	✖	✖	✖	○ (I)
CPL-304-110	✖	✖	✖	✖	○ (I)
E-7090, hormone therapy	✖	✖	✖	✖	○ (I)
infigratinib, hormone therapy	✖	✖	✖	✖	○ (I)

FGFR3 V555 mutation

Relevant Therapy	FDA	NCCN	EMA	ESMO	Clinical Trials*
erdafitinib	✖	✖	✖	○	◐ (II)

* Most advanced phase (IV, III, II/III, II, I/II, I) is shown and multiple clinical trials may be available.

Disclaimer: The data presented here is from a curated knowledgebase of publicly available information, but may not be exhaustive. The data version is 2021.11(003).

Relevant Therapy Summary (continued)

● In this cancer type ○ In other cancer type ◐ In this cancer type and other cancer types ✖ No evidence

FGFR3 V555 mutation (continued)

Relevant Therapy	FDA	NCCN	EMA	ESMO	Clinical Trials*
futibatinib, pembrolizumab	✖	✖	✖	✖	◐ (II)
pemigatinib	✖	✖	✖	✖	◐ (II)
anlotinib hydrochloride	✖	✖	✖	✖	◐ (I/II)
futibatinib	✖	✖	✖	✖	● (II)
sunitinib	✖	✖	✖	✖	● (II)
ICP-192	✖	✖	✖	✖	● (I/II)
TAS-117, futibatinib	✖	✖	✖	✖	● (I/II)
durvalumab, olaparib, hormone therapy	✖	✖	✖	✖	○ (II)
rogaratinib	✖	✖	✖	✖	○ (II)
rogaratinib, chemotherapy	✖	✖	✖	✖	○ (II)
erdafitinib, cetrelimab	✖	✖	✖	✖	○ (I/II)
pemigatinib, pembrolizumab, trastuzumab, chemotherapy, retifanlimab	✖	✖	✖	✖	○ (I/II)
BPI-17509	✖	✖	✖	✖	○ (I)
CPL-304-110	✖	✖	✖	✖	○ (I)
E-7090, hormone therapy	✖	✖	✖	✖	○ (I)
infigratinib, hormone therapy	✖	✖	✖	✖	○ (I)

FGFR3 V557 mutation

Relevant Therapy	FDA	NCCN	EMA	ESMO	Clinical Trials*
erdafitinib	✖	✖	✖	○	◐ (II)
futibatinib, pembrolizumab	✖	✖	✖	✖	◐ (II)
pemigatinib	✖	✖	✖	✖	◐ (II)
anlotinib hydrochloride	✖	✖	✖	✖	◐ (I/II)
futibatinib	✖	✖	✖	✖	● (II)
sunitinib	✖	✖	✖	✖	● (II)
ICP-192	✖	✖	✖	✖	● (I/II)
TAS-117, futibatinib	✖	✖	✖	✖	● (I/II)
durvalumab, olaparib, hormone therapy	✖	✖	✖	✖	○ (II)

* Most advanced phase (IV, III, II/III, II, I/II, I) is shown and multiple clinical trials may be available.

Disclaimer: The data presented here is from a curated knowledgebase of publicly available information, but may not be exhaustive. The data version is 2021.11(003).

Relevant Therapy Summary (continued)

● In this cancer type ○ In other cancer type ◐ In this cancer type and other cancer types ✖ No evidence

FGFR3 V557 mutation (continued)

Relevant Therapy	FDA	NCCN	EMA	ESMO	Clinical Trials*
rogaratinib	✖	✖	✖	✖	○ (II)
rogaratinib, chemotherapy	✖	✖	✖	✖	○ (II)
erdafitinib, cetrelimab	✖	✖	✖	✖	○ (I/II)
pemigatinib, pembrolizumab, trastuzumab, chemotherapy, retifanlimab	✖	✖	✖	✖	○ (I/II)
BPI-17509	✖	✖	✖	✖	○ (I)
CPL-304-110	✖	✖	✖	✖	○ (I)
E-7090, hormone therapy	✖	✖	✖	✖	○ (I)
infigratinib, hormone therapy	✖	✖	✖	✖	○ (I)

FGFR3 amplification

Relevant Therapy	FDA	NCCN	EMA	ESMO	Clinical Trials*
futibatinib, pembrolizumab	✖	✖	✖	✖	◐ (II)
infigratinib	✖	✖	✖	✖	◐ (II)
pemigatinib	✖	✖	✖	✖	◐ (II)
sunitinib	✖	✖	✖	✖	● (II)
ICP-192	✖	✖	✖	✖	● (I/II)
TAS-117, futibatinib	✖	✖	✖	✖	● (I/II)
erdafitinib	✖	✖	✖	✖	○ (II)
hormone therapy, anlotinib hydrochloride	✖	✖	✖	✖	○ (II)
rogaratinib	✖	✖	✖	✖	○ (II)
rogaratinib, chemotherapy	✖	✖	✖	✖	○ (II)
erdafitinib, cetrelimab	✖	✖	✖	✖	○ (I/II)
pemigatinib, pembrolizumab, trastuzumab, chemotherapy, retifanlimab	✖	✖	✖	✖	○ (I/II)
BPI-17509	✖	✖	✖	✖	○ (I)
CPL-304-110	✖	✖	✖	✖	○ (I)
E-7090, hormone therapy	✖	✖	✖	✖	○ (I)
infigratinib, hormone therapy	✖	✖	✖	✖	○ (I)
rogaratinib, palbociclib, hormone therapy	✖	✖	✖	✖	○ (I)

* Most advanced phase (IV, III, II/III, II, I/II, I) is shown and multiple clinical trials may be available.

Disclaimer: The data presented here is from a curated knowledgebase of publicly available information, but may not be exhaustive. The data version is 2021.11(003).

Relevant Therapy Summary (continued)

● In this cancer type ○ In other cancer type ◐ In this cancer type and other cancer types ✖ No evidence

FGFR3 aberration

Relevant Therapy	FDA	NCCN	EMA	ESMO	Clinical Trials*
futibatinib, pembrolizumab	✖	✖	✖	✖	◐ (II)
pemigatinib	✖	✖	✖	✖	● (II)
sunitinib	✖	✖	✖	✖	● (II)
ICP-192	✖	✖	✖	✖	● (I/II)
TAS-117, futibatinib	✖	✖	✖	✖	● (I/II)
rogaratinib	✖	✖	✖	✖	○ (II)
erdafitinib, cetrelimab	✖	✖	✖	✖	○ (I/II)
pemigatinib, pembrolizumab, trastuzumab, chemotherapy, retifanlimab	✖	✖	✖	✖	○ (I/II)
BPI-17509	✖	✖	✖	✖	○ (I)
CPL-304-110	✖	✖	✖	✖	○ (I)
E-7090, hormone therapy	✖	✖	✖	✖	○ (I)
infigratinib, hormone therapy	✖	✖	✖	✖	○ (I)

* Most advanced phase (IV, III, II/III, II, I/II, I) is shown and multiple clinical trials may be available.

Relevant Biomarkers

Genomic Alteration	Relevant Therapies (In this cancer type)	Relevant Therapies (In other cancer type)	Clinical Trials
FGFR3 G370C mutation<br>**Prognostic significance:** None<br>**Diagnostic significance:** None	None	**erdafitinib** [1]	35
FGFR3 R248C mutation<br>**Prognostic significance:** None<br>**Diagnostic significance:** None	None	**erdafitinib** [1]	35
FGFR3 S249C mutation<br>**Prognostic significance:** None<br>**Diagnostic significance:** None	None	**erdafitinib** [1]	35
FGFR3-TACC3 fusion<br>**Prognostic significance:** None<br>**Diagnostic significance:** None	None	**erdafitinib** [1]	31
FGFR3-BAIAP2L1 fusion<br>**Prognostic significance:** None<br>**Diagnostic significance:** None	None	**erdafitinib** [1]	30

Public data sources included in relevant therapies: FDA1, NCCN, **EMA**2, ESMO
Public data sources included in prognostic and diagnostic significance: NCCN, ESMO

Relevant Biomarkers (continued)

Genomic Alteration	Relevant Therapies (In this cancer type)	Relevant Therapies (In other cancer type)	Clinical Trials
FGFR3 Y373C mutation<br>**Prognostic significance:** None<br>**Diagnostic significance:** None	None	**erdafitinib** [1]	29
FGFR3 K650E mutation<br>**Prognostic significance:** None<br>**Diagnostic significance:** None	None	erdafitinib	33
FGFR3 K650M mutation<br>**Prognostic significance:** None<br>**Diagnostic significance:** None	None	erdafitinib	32
FGFR3 K650Q mutation<br>**Prognostic significance:** None<br>**Diagnostic significance:** None	None	erdafitinib	32
FGFR3 K650T mutation<br>**Prognostic significance:** None<br>**Diagnostic significance:** None	None	erdafitinib	32
FGFR3 activating mutation<br>**Prognostic significance:** None<br>**Diagnostic significance:** None	None	erdafitinib	31
FGFR3 K652E mutation<br>**Prognostic significance:** None<br>**Diagnostic significance:** None	None	erdafitinib	31
FGFR3 fusion<br>**Prognostic significance:** None<br>**Diagnostic significance:** None	None	erdafitinib	27
FGFR3 A391E mutation<br>**Prognostic significance:** None<br>**Diagnostic significance:** None	None	erdafitinib	26
FGFR3 KD mutation<br>**Prognostic significance:** None<br>**Diagnostic significance:** None	None	erdafitinib	25
FGFR3 mutation<br>**Prognostic significance:** None<br>**Diagnostic significance:** None	None	erdafitinib	25
FGFR3 V555 mutation<br>**Prognostic significance:** None<br>**Diagnostic significance:** None	None	erdafitinib	25
FGFR3 V557 mutation<br>**Prognostic significance:** None<br>**Diagnostic significance:** None	None	erdafitinib	25

Public data sources included in relevant therapies: FDA[1], NCCN, **EMA**[2], ESMO
Public data sources included in prognostic and diagnostic significance: NCCN, ESMO

Disclaimer: The data presented here is from a curated knowledgebase of publicly available information, but may not be exhaustive. The data version is 2021.11(003).

Relevant Biomarkers (continued)

Genomic Alteration	Relevant Therapies (In this cancer type)	Relevant Therapies (In other cancer type)	Clinical Trials
FGFR3 amplification<br>**Prognostic significance:** None<br>**Diagnostic significance:** None	None	None	21
FGFR3 aberration<br>**Prognostic significance:** None<br>**Diagnostic significance:** None	None	None	13

Public data sources included in relevant therapies: FDA1, NCCN, **EMA**2, ESMO
Public data sources included in prognostic and diagnostic significance: NCCN, ESMO

Clinical Trials Summary

FGFR3 G370C mutation

NCT ID	Title	Phase
NCT05019794	A Phase IIa of Infigratinib in Subjects With Locally Advanced or Metastatic Gastric Cancer or Gastroesophageal Junction Adenocarcinoma With FGFR2 Amplification or Other Advanced Solid Tumors With Other FGFR Alterations	II
NCT03155620	NCI-COG Pediatric MATCH (Molecular Analysis for Therapy Choice) Screening Protocol	II
NCT03210714	NCI-COG Pediatric MATCH (Molecular Analysis for Therapy Choice) - Phase II Subprotocol of JNJ-42756493 (Erdafitinib) in Patients With Tumors Harboring FGFR 1/2/3/4 Alterations	II
No NCT ID	A Multicenter Phase II Basket-type Clinical Trial to Evaluate Efficacy and Safety of TAS-120 in Patients with Advanced Solid Malignancies with FGFR Alterations in Circulating Tumor DNA	II
NCT04233567	A Phase II Study of Oral Infigratinib in Adult Patients With Advanced or Metastatic Solid Tumors With FGFR1-3 Gene Fusions or Other FGFR Genetic Alterations	II
NCT02693535	Targeted Agent and Profiling Utilization Registry (TAPUR) Study.	II
NCT04591431	The Rome Trial From Histology to Target: the Road to Personalize Target Therapy and Immunotherapy	II
NCT02691793	Study to Evaluate the Safety and Efficacy of Sunitinib, in Subject With Refractory Solid Tumors	II
NCT03297606	Canadian Profiling and Targeted Agent Utilization Trial (CAPTUR): A Phase II Basket Trial	II
NCT03929965	Anlotinib in Advanced Solid Tumors With FGFR Alteration	I
NCT04565275	A Multi-center Open-label, Phase I/II Clinical Trial to Evaluate the Safety, Tolerability, Pharmacokinetics, and Pharmacodynamics of ICP-192 in Patients With Advanced Solid Tumors and FGFR Gene Alterations.	I/II
No NCT ID	Phase I/II Study of TAS-117 In Combination With TAS-120 In Patients With Advanced Solid Tumors	I/II
No NCT ID	A Phase Ib Study to Assess the Safety, Tolerability, and Efficacy of TAS-120 (Futibatinib) in Combination with MK-3475 (Pembrolizumab) in Patients with Solid Tumors.	I
NCT03390504	A Phase III Study of Erdafitinib Compared With Vinflunine or Docetaxel or Pembrolizumab in Subjects With Advanced Urothelial Cancer and Selected FGFR Gene Aberrations	III
NCT04197986	Phase III, Multicenter, Double-Blind, Randomized, Placebo-Controlled Trial of Infigratinib for the Adjuvant Treatment of Subjects With Invasive Urothelial Carcinoma With Susceptible FGFR3 Genetic Alterations (PROOF 302).	III

Disclaimer: The data presented here is from a curated knowledgebase of publicly available information, but may not be exhaustive. The data version is 2021.11(003).

Clinical Trials Summary (continued)

FGFR3 G370C mutation (continued)

NCT ID	Title	Phase
NCT02365597	A Phase II, Two-arm Multicenter, Open-Label Study to Determine the Efficacy and the Safety of Two Different Dose Regimens of a Pan-FGFR Tyrosine Kinase Inhibitor JNJ-42756493 in Subjects With Metastatic or Surgically Unresectable Urothelial Cancer With FGFR Genomic Alterations.	II
NCT04492293	A Phase II, Multicenter, Single Arm, Open-lable Study of ICP-192 in Subjects with Surgically Unresectable or Metastatic Bladder Urothelial Cancer with FGFR Genetic Aberrations.	II
NCT04936295	A Phase II Study of the Efficacy and Tolerability of Fulvestrant Plus Anlotinib in HR(+)/HER2(-) Metastatic Breast Cancer Patients With FGFR Mutation	II
NCT04096417	A Phase II, Multicenter, Single-Arm Study of Pemigatinib in Patients With Metastatic or Unresectable Colorectal Cancer Harboring FGFR Alterations	II
NCT04045613	An Open-label Multi-cohort Phase Ib/II Study of Derazantinib and Atezolizumab in Patients With Urothelial Cancer Expressing Activating Molecular FGFR Aberrations.	I/II
NCT04053322	An International Multicenter Phase II Trial of Durvalumab (MEDI4736) Plus OLAparib Plus Fulvestrant in Metastatic or Locally Advanced ER-positive, HER2-negative Breast Cancer Patients Selected Using Criteria That Predict Sensitivity to Olaparib	II
NCT03827850	A Phase II Trial to Evaluate Efficacy and Safety of Erdafitinib in Patients With Advanced Squamous Non Small Cell Lung Carcinoma (sqNSCLC) Harboring Fibroblast Growth Factor Receptor (FGFR) Genetic Alterations After Relapse of Standard Therapy.	II
NCT04601857	A Phase II Study Evaluating Futibatinib (TAS 120) Plus Pembrolizumab in the Treatment of Advanced or Metastatic Urothelial Carcinoma	II
NCT04294277	Open-label, Single-arm, Phase II Study, Evaluating Safety and Efficacy of INCB054828 (Pemigatinib) as Adjuvant Therapy for Molecularly-selected, High-risk Patients With Urothelial Carcinoma Who Have Received Radical Surgery	II
NCT04077255	Anti-EGFR Antibody (GC-1118) in Combination With Weekly Paclitaxel as a Second-line Therapy for Gastric and Gastroesophageal Junction Adenocarcinomas With Amplifications	II
No NCT ID	Phase I Clinical Study of BPI-17509 In Patients With Advanced Solid Tumors	I
NCT04172675	A Randomized Phase II Study of Erdafitinib Versus Investigator Choice of Intravesical Chemotherapy in Subjects Who Received Bacillus Calmette-Guerin (BCG) and Recurred With High Risk Non-Muscle-Invasive Bladder Cancer (NMIBC) and FGFR Mutations or Fusions	II
NCT02150967	A Phase II Multicenter, Single Arm Study of Oral BGJ398 in Adult Patients with Advanced or Metastatic Cholangiocarcinoma with FGFR2 Gene Fusions or Other FGFR Genetic Alterations who Failed or are Intolerant to Platinum-based Chemotherapy.	II
NCT04595747	Phase II Study of Rogaratinib (BAY 1163877) in the Treatment of Patients With Sarcoma Harboring Alterations in Fibroblast Growth Factor Receptor (FGFR) 1-4 and SDH-Deficient Gastrointestinal Stromal Tumor (GIST)	II
NCT04004975	Phase II Clinical Trials on Anlotinib for the Treatment of Recurrent Glioblastoma	I/II
NCT04149691	A Phase I, Open-label, Multicentre, Dose Escalation Study to Assess Safety, Tolerability and Pharmacokinetics of Oral CPL304110, in Adult Subjects With Advanced Solid Malignancies	I
NCT03473743	A Phase Ib-II Study to Evaluate Safety, Efficacy, Pharmacokinetics, and Pharmacodynamics of Various Regimens of Erdafitinib in Subjects With Metastatic or Locally Advanced Urothelial Cancer	I/II
NCT02393248	A Phase I/II, Open-Label, Dose-Escalation, Safety and Tolerability Study of INCB054828 in Subjects With Advanced Malignancies - (FIGHT-101)	I/II

Disclaimer: The data presented here is from a curated knowledgebase of publicly available information, but may not be exhaustive. The data version is 2021.11(003).

Clinical Trials Summary (continued)

FGFR3 G370C mutation (continued)

NCT ID	Title	Phase
NCT04572295	An Open-label Phase Ib Study of E7090 Monotherapy and in Combination With Other Anticancer Agents in Subjects With ER+, HER2- Recurrent/Metastatic Breast Cancer	I
NCT04504331	A Phase IB Study of Infigratinib in Combination With Tamoxifen in Hormone Receptor Positive, HER2 Negative, FGFR Altered Advanced Breast Cancer	I

FGFR3 R248C mutation

NCT ID	Title	Phase
NCT05019794	A Phase IIa of Infigratinib in Subjects With Locally Advanced or Metastatic Gastric Cancer or Gastroesophageal Junction Adenocarcinoma With FGFR2 Amplification or Other Advanced Solid Tumors With Other FGFR Alterations	II
NCT03155620	NCI-COG Pediatric MATCH (Molecular Analysis for Therapy Choice) Screening Protocol	II
NCT03210714	NCI-COG Pediatric MATCH (Molecular Analysis for Therapy Choice) - Phase II Subprotocol of JNJ-42756493 (Erdafitinib) in Patients With Tumors Harboring FGFR 1/2/3/4 Alterations	II
No NCT ID	A Multicenter Phase II Basket-type Clinical Trial to Evaluate Efficacy and Safety of TAS-120 in Patients with Advanced Solid Malignancies with FGFR Alterations in Circulating Tumor DNA	II
NCT04233567	A Phase II Study of Oral Infigratinib in Adult Patients With Advanced or Metastatic Solid Tumors With FGFR1-3 Gene Fusions or Other FGFR Genetic Alterations	II
NCT02693535	Targeted Agent and Profiling Utilization Registry (TAPUR) Study.	II
NCT04591431	The Rome Trial From Histology to Target: the Road to Personalize Target Therapy and Immunotherapy	II
NCT02691793	Study to Evaluate the Safety and Efficacy of Sunitinib, in Subject With Refractory Solid Tumors	II
NCT03297606	Canadian Profiling and Targeted Agent Utilization Trial (CAPTUR): A Phase II Basket Trial	II
NCT03929965	Anlotinib in Advanced Solid Tumors With FGFR Alteration	I
NCT04565275	A Multi-center Open-label, Phase I/II Clinical Trial to Evaluate the Safety, Tolerability, Pharmacokinetics, and Pharmacodynamics of ICP-192 in Patients With Advanced Solid Tumors and FGFR Gene Alterations.	I/II
No NCT ID	Phase I/II Study of TAS-117 In Combination With TAS-120 In Patients With Advanced Solid Tumors	I/II
No NCT ID	A Phase Ib Study to Assess the Safety, Tolerability, and Efficacy of TAS-120 (Futibatinib) in Combination with MK-3475 (Pembrolizumab) in Patients with Solid Tumors.	I
NCT03390504	A Phase III Study of Erdafitinib Compared With Vinflunine or Docetaxel or Pembrolizumab in Subjects With Advanced Urothelial Cancer and Selected FGFR Gene Aberrations	III
NCT04197986	Phase III, Multicenter, Double-Blind, Randomized, Placebo-Controlled Trial of Infigratinib for the Adjuvant Treatment of Subjects With Invasive Urothelial Carcinoma With Susceptible FGFR3 Genetic Alterations (PROOF 302).	III
NCT02365597	A Phase II, Two-arm Multicenter, Open-Label Study to Determine the Efficacy and the Safety of Two Different Dose Regimens of a Pan-FGFR Tyrosine Kinase Inhibitor JNJ-42756493 in Subjects With Metastatic or Surgically Unresectable Urothelial Cancer With FGFR Genomic Alterations.	II
NCT04492293	A Phase II, Multicenter, Single Arm, Open-lable Study of ICP-192 in Subjects with Surgically Unresectable or Metastatic Bladder Urothelial Cancer with FGFR Genetic Aberrations.	II

Disclaimer: The data presented here is from a curated knowledgebase of publicly available information, but may not be exhaustive. The data version is 2021.11(003).

Clinical Trials Summary (continued)

FGFR3 R248C mutation (continued)

NCT ID	Title	Phase
NCT04936295	A Phase II Study of the Efficacy and Tolerability of Fulvestrant Plus Anlotinib in HR(+)/HER2(-) Metastatic Breast Cancer Patients With FGFR Mutation	II
NCT04096417	A Phase II, Multicenter, Single-Arm Study of Pemigatinib in Patients With Metastatic or Unresectable Colorectal Cancer Harboring FGFR Alterations	II
NCT04045613	An Open-label Multi-cohort Phase Ib/II Study of Derazantinib and Atezolizumab in Patients With Urothelial Cancer Expressing Activating Molecular FGFR Aberrations.	I/II
NCT04053322	An International Multicenter Phase II Trial of Durvalumab (MEDI4736) Plus OLAparib Plus Fulvestrant in Metastatic or Locally Advanced ER-positive, HER2-negative Breast Cancer Patients Selected Using Criteria That Predict Sensitivity to Olaparib	II
NCT03827850	A Phase II Trial to Evaluate Efficacy and Safety of Erdafitinib in Patients With Advanced Squamous Non Small Cell Lung Carcinoma (sqNSCLC) Harboring Fibroblast Growth Factor Receptor (FGFR) Genetic Alterations After Relapse of Standard Therapy.	II
NCT04601857	A Phase II Study Evaluating Futibatinib (TAS 120) Plus Pembrolizumab in the Treatment of Advanced or Metastatic Urothelial Carcinoma	II
NCT04294277	Open-label, Single-arm, Phase II Study, Evaluating Safety and Efficacy of INCB054828 (Pemigatinib) as Adjuvant Therapy for Molecularly-selected, High-risk Patients With Urothelial Carcinoma Who Have Received Radical Surgery	II
NCT04077255	Anti-EGFR Antibody (GC-1118) in Combination With Weekly Paclitaxel as a Second-line Therapy for Gastric and Gastroesophageal Junction Adenocarcinomas With Amplifications	II
No NCT ID	Phase I Clinical Study of BPI-17509 In Patients With Advanced Solid Tumors	I
NCT04172675	A Randomized Phase II Study of Erdafitinib Versus Investigator Choice of Intravesical Chemotherapy in Subjects Who Received Bacillus Calmette-Guerin (BCG) and Recurred With High Risk Non-Muscle-Invasive Bladder Cancer (NMIBC) and FGFR Mutations or Fusions	II
NCT02150967	A Phase II Multicenter, Single Arm Study of Oral BGJ398 in Adult Patients with Advanced or Metastatic Cholangiocarcinoma with FGFR2 Gene Fusions or Other FGFR Genetic Alterations who Failed or are Intolerant to Platinum-based Chemotherapy.	II
NCT04595747	Phase II Study of Rogaratinib (BAY 1163877) in the Treatment of Patients With Sarcoma Harboring Alterations in Fibroblast Growth Factor Receptor (FGFR) 1-4 and SDH-Deficient Gastrointestinal Stromal Tumor (GIST)	II
NCT04004975	Phase II Clinical Trials on Anlotinib for the Treatment of Recurrent Glioblastoma	I/II
NCT04149691	A Phase I, Open-label, Multicentre, Dose Escalation Study to Assess Safety, Tolerability and Pharmacokinetics of Oral CPL304110, in Adult Subjects With Advanced Solid Malignancies	I
NCT03473743	A Phase Ib-II Study to Evaluate Safety, Efficacy, Pharmacokinetics, and Pharmacodynamics of Various Regimens of Erdafitinib in Subjects With Metastatic or Locally Advanced Urothelial Cancer	I/II
NCT02393248	A Phase I/II, Open-Label, Dose-Escalation, Safety and Tolerability Study of INCB054828 in Subjects With Advanced Malignancies - (FIGHT-101)	I/II
NCT04572295	An Open-label Phase Ib Study of E7090 Monotherapy and in Combination With Other Anticancer Agents in Subjects With ER+, HER2- Recurrent/Metastatic Breast Cancer	I
NCT04504331	A Phase IB Study of Infigratinib in Combination With Tamoxifen in Hormone Receptor Positive, HER2 Negative, FGFR Altered Advanced Breast Cancer	I

Disclaimer: The data presented here is from a curated knowledgebase of publicly available information, but may not be exhaustive. The data version is 2021.11(003).

Clinical Trials Summary (continued)

FGFR3 S249C mutation

NCT ID	Title	Phase
NCT05019794	A Phase IIa of Infigratinib in Subjects With Locally Advanced or Metastatic Gastric Cancer or Gastroesophageal Junction Adenocarcinoma With FGFR2 Amplification or Other Advanced Solid Tumors With Other FGFR Alterations	II
NCT03155620	NCI-COG Pediatric MATCH (Molecular Analysis for Therapy Choice) Screening Protocol	II
NCT03210714	NCI-COG Pediatric MATCH (Molecular Analysis for Therapy Choice) - Phase II Subprotocol of JNJ-42756493 (Erdafitinib) in Patients With Tumors Harboring FGFR 1/2/3/4 Alterations	II
No NCT ID	A Multicenter Phase II Basket-type Clinical Trial to Evaluate Efficacy and Safety of TAS-120 in Patients with Advanced Solid Malignancies with FGFR Alterations in Circulating Tumor DNA	II
NCT04233567	A Phase II Study of Oral Infigratinib in Adult Patients With Advanced or Metastatic Solid Tumors With FGFR1-3 Gene Fusions or Other FGFR Genetic Alterations	II
NCT02693535	Targeted Agent and Profiling Utilization Registry (TAPUR) Study.	II
NCT04591431	The Rome Trial From Histology to Target: the Road to Personalize Target Therapy and Immunotherapy	II
NCT02691793	Study to Evaluate the Safety and Efficacy of Sunitinib, in Subject With Refractory Solid Tumors	II
NCT03297606	Canadian Profiling and Targeted Agent Utilization Trial (CAPTUR): A Phase II Basket Trial	II
NCT03929965	Anlotinib in Advanced Solid Tumors With FGFR Alteration	I
NCT04565275	A Multi-center Open-label, Phase I/II Clinical Trial to Evaluate the Safety, Tolerability, Pharmacokinetics, and Pharmacodynamics of ICP-192 in Patients With Advanced Solid Tumors and FGFR Gene Alterations.	I/II
No NCT ID	Phase I/II Study of TAS-117 In Combination With TAS-120 In Patients With Advanced Solid Tumors	I/II
No NCT ID	A Phase Ib Study to Assess the Safety, Tolerability, and Efficacy of TAS-120 (Futibatinib) in Combination with MK-3475 (Pembrolizumab) in Patients with Solid Tumors.	I
NCT03390504	A Phase III Study of Erdafitinib Compared With Vinflunine or Docetaxel or Pembrolizumab in Subjects With Advanced Urothelial Cancer and Selected FGFR Gene Aberrations	III
NCT04197986	Phase III, Multicenter, Double-Blind, Randomized, Placebo-Controlled Trial of Infigratinib for the Adjuvant Treatment of Subjects With Invasive Urothelial Carcinoma With Susceptible FGFR3 Genetic Alterations (PROOF 302).	III
NCT02365597	A Phase II, Two-arm Multicenter, Open-Label Study to Determine the Efficacy and the Safety of Two Different Dose Regimens of a Pan-FGFR Tyrosine Kinase Inhibitor JNJ-42756493 in Subjects With Metastatic or Surgically Unresectable Urothelial Cancer With FGFR Genomic Alterations.	II
NCT04492293	A Phase II, Multicenter, Single Arm, Open-lable Study of ICP-192 in Subjects with Surgically Unresectable or Metastatic Bladder Urothelial Cancer with FGFR Genetic Aberrations.	II
NCT04936295	A Phase II Study of the Efficacy and Tolerability of Fulvestrant Plus Anlotinib in HR(+)/HER2(-) Metastatic Breast Cancer Patients With FGFR Mutation	II
NCT04096417	A Phase II, Multicenter, Single-Arm Study of Pemigatinib in Patients With Metastatic or Unresectable Colorectal Cancer Harboring FGFR Alterations	II
NCT04045613	An Open-label Multi-cohort Phase Ib/II Study of Derazantinib and Atezolizumab in Patients With Urothelial Cancer Expressing Activating Molecular FGFR Aberrations.	I/II
NCT04053322	An International Multicenter Phase II Trial of Durvalumab (MEDI4736) Plus OLAparib Plus Fulvestrant in Metastatic or Locally Advanced ER-positive, HER2-negative Breast Cancer Patients Selected Using Criteria That Predict Sensitivity to Olaparib	II

Clinical Trials Summary (continued)

FGFR3 S249C mutation (continued)

NCT ID	Title	Phase
NCT03827850	A Phase II Trial to Evaluate Efficacy and Safety of Erdafitinib in Patients With Advanced Squamous Non Small Cell Lung Carcinoma (sqNSCLC) Harboring Fibroblast Growth Factor Receptor (FGFR) Genetic Alterations After Relapse of Standard Therapy.	II
NCT04601857	A Phase II Study Evaluating Futibatinib (TAS 120) Plus Pembrolizumab in the Treatment of Advanced or Metastatic Urothelial Carcinoma	II
NCT04294277	Open-label, Single-arm, Phase II Study, Evaluating Safety and Efficacy of INCB054828 (Pemigatinib) as Adjuvant Therapy for Molecularly-selected, High-risk Patients With Urothelial Carcinoma Who Have Received Radical Surgery	II
NCT04077255	Anti-EGFR Antibody (GC-1118) in Combination With Weekly Paclitaxel as a Second-line Therapy for Gastric and Gastroesophageal Junction Adenocarcinomas With Amplifications	II
No NCT ID	Phase I Clinical Study of BPI-17509 In Patients With Advanced Solid Tumors	I
NCT04172675	A Randomized Phase II Study of Erdafitinib Versus Investigator Choice of Intravesical Chemotherapy in Subjects Who Received Bacillus Calmette-Guerin (BCG) and Recurred With High Risk Non-Muscle-Invasive Bladder Cancer (NMIBC) and FGFR Mutations or Fusions	II
NCT02150967	A Phase II Multicenter, Single Arm Study of Oral BGJ398 in Adult Patients with Advanced or Metastatic Cholangiocarcinoma with FGFR2 Gene Fusions or Other FGFR Genetic Alterations who Failed or are Intolerant to Platinum-based Chemotherapy.	II
NCT04595747	Phase II Study of Rogaratinib (BAY 1163877) in the Treatment of Patients With Sarcoma Harboring Alterations in Fibroblast Growth Factor Receptor (FGFR) 1-4 and SDH-Deficient Gastrointestinal Stromal Tumor (GIST)	II
NCT04004975	Phase II Clinical Trials on Anlotinib for the Treatment of Recurrent Glioblastoma	I/II
NCT04149691	A Phase I, Open-label, Multicentre, Dose Escalation Study to Assess Safety, Tolerability and Pharmacokinetics of Oral CPL304110, in Adult Subjects With Advanced Solid Malignancies	I
NCT03473743	A Phase Ib-II Study to Evaluate Safety, Efficacy, Pharmacokinetics, and Pharmacodynamics of Various Regimens of Erdafitinib in Subjects With Metastatic or Locally Advanced Urothelial Cancer	I/II
NCT02393248	A Phase I/II, Open-Label, Dose-Escalation, Safety and Tolerability Study of INCB054828 in Subjects With Advanced Malignancies - (FIGHT-101)	I/II
NCT04572295	An Open-label Phase Ib Study of E7090 Monotherapy and in Combination With Other Anticancer Agents in Subjects With ER+, HER2- Recurrent/Metastatic Breast Cancer	I
NCT04504331	A Phase IB Study of Infigratinib in Combination With Tamoxifen in Hormone Receptor Positive, HER2 Negative, FGFR Altered Advanced Breast Cancer	I

FGFR3-TACC3 fusion

NCT ID	Title	Phase
No NCT ID	A Multicenter Phase II Basket-type Clinical Trial to Evaluate Efficacy and Safety of TAS-120 in Patients with Advanced Solid Malignancies with FGFR Alterations in Circulating Tumor DNA	II
NCT04189445	A Phase II Study of Futibatinib in Patients With Specific FGFR Aberrations	II
NCT04233567	A Phase II Study of Oral Infigratinib in Adult Patients With Advanced or Metastatic Solid Tumors With FGFR1-3 Gene Fusions or Other FGFR Genetic Alterations	II

Disclaimer: The data presented here is from a curated knowledgebase of publicly available information, but may not be exhaustive. The data version is 2021.11(003).

Clinical Trials Summary (continued)

FGFR3-TACC3 fusion (continued)

NCT ID	Title	Phase
NCT05019794	A Phase IIa of Infigratinib in Subjects With Locally Advanced or Metastatic Gastric Cancer or Gastroesophageal Junction Adenocarcinoma With FGFR2 Amplification or Other Advanced Solid Tumors With Other FGFR Alterations	II
NCT03929965	Anlotinib in Advanced Solid Tumors With FGFR Alteration	I
NCT04591431	The Rome Trial From Histology to Target: the Road to Personalize Target Therapy and Immunotherapy	II
NCT03297606	Canadian Profiling and Targeted Agent Utilization Trial (CAPTUR): A Phase II Basket Trial	II
NCT04565275	A Multi-center Open-label, Phase I/II Clinical Trial to Evaluate the Safety, Tolerability, Pharmacokinetics, and Pharmacodynamics of ICP-192 in Patients With Advanced Solid Tumors and FGFR Gene Alterations.	I/II
No NCT ID	Phase I/II Study of TAS-117 In Combination With TAS-120 In Patients With Advanced Solid Tumors	I/II
No NCT ID	A Phase Ib Study to Assess the Safety, Tolerability, and Efficacy of TAS-120 (Futibatinib) in Combination with MK-3475 (Pembrolizumab) in Patients with Solid Tumors.	I
NCT03390504	A Phase III Study of Erdafitinib Compared With Vinflunine or Docetaxel or Pembrolizumab in Subjects With Advanced Urothelial Cancer and Selected FGFR Gene Aberrations	III
NCT02365597	A Phase II, Two-arm Multicenter, Open-Label Study to Determine the Efficacy and the Safety of Two Different Dose Regimens of a Pan-FGFR Tyrosine Kinase Inhibitor JNJ-42756493 in Subjects With Metastatic or Surgically Unresectable Urothelial Cancer With FGFR Genomic Alterations.	II
NCT04492293	A Phase II, Multicenter, Single Arm, Open-lable Study of ICP-192 in Subjects with Surgically Unresectable or Metastatic Bladder Urothelial Cancer with FGFR Genetic Aberrations.	II
NCT04424966	A Phase 0 Study of Infigratinib in Recurrent High-Grade Glioma Participants Scheduled for Resection to Evaluate Central Nervous System (CNS) Penetration With PK Triggered Expansion Cohort	I
NCT04197986	Phase III, Multicenter, Double-Blind, Randomized, Placebo-Controlled Trial of Infigratinib for the Adjuvant Treatment of Subjects With Invasive Urothelial Carcinoma With Susceptible FGFR3 Genetic Alterations (PROOF 302).	III
NCT04601857	A Phase II Study Evaluating Futibatinib (TAS 120) Plus Pembrolizumab in the Treatment of Advanced or Metastatic Urothelial Carcinoma	II
NCT04936295	A Phase II Study of the Efficacy and Tolerability of Fulvestrant Plus Anlotinib in HR(+)/HER2(-) Metastatic Breast Cancer Patients With FGFR Mutation	II
NCT04096417	A Phase II, Multicenter, Single-Arm Study of Pemigatinib in Patients With Metastatic or Unresectable Colorectal Cancer Harboring FGFR Alterations	II
NCT04294277	Open-label, Single-arm, Phase II Study, Evaluating Safety and Efficacy of INCB054828 (Pemigatinib) as Adjuvant Therapy for Molecularly-selected, High-risk Patients With Urothelial Carcinoma Who Have Received Radical Surgery	II
NCT04077255	Anti-EGFR Antibody (GC-1118) in Combination With Weekly Paclitaxel as a Second-line Therapy for Gastric and Gastroesophageal Junction Adenocarcinomas With Amplifications	II
NCT04045613	An Open-label Multi-cohort Phase Ib/II Study of Derazantinib and Atezolizumab in Patients With Urothelial Cancer Expressing Activating Molecular FGFR Aberrations.	I/II
No NCT ID	Phase I Clinical Study of BPI-17509 In Patients With Advanced Solid Tumors	I
NCT03827850	A Phase II Trial to Evaluate Efficacy and Safety of Erdafitinib in Patients With Advanced Squamous Non Small Cell Lung Carcinoma (sqNSCLC) Harboring Fibroblast Growth Factor Receptor (FGFR) Genetic Alterations After Relapse of Standard Therapy.	II

Disclaimer: The data presented here is from a curated knowledgebase of publicly available information, but may not be exhaustive. The data version is 2021.11(003).

Clinical Trials Summary (continued)

FGFR3-TACC3 fusion (continued)

NCT ID	Title	Phase
NCT04172675	A Randomized Phase II Study of Erdafitinib Versus Investigator Choice of Intravesical Chemotherapy in Subjects Who Received Bacillus Calmette-Guerin (BCG) and Recurred With High Risk Non-Muscle-Invasive Bladder Cancer (NMIBC) and FGFR Mutations or Fusions	II
NCT02150967	A Phase II Multicenter, Single Arm Study of Oral BGJ398 in Adult Patients with Advanced or Metastatic Cholangiocarcinoma with FGFR2 Gene Fusions or Other FGFR Genetic Alterations who Failed or are Intolerant to Platinum-based Chemotherapy.	II
NCT04595747	Phase II Study of Rogaratinib (BAY 1163877) in the Treatment of Patients With Sarcoma Harboring Alterations in Fibroblast Growth Factor Receptor (FGFR) 1-4 and SDH-Deficient Gastrointestinal Stromal Tumor (GIST)	II
NCT04149691	A Phase I, Open-label, Multicentre, Dose Escalation Study to Assess Safety, Tolerability and Pharmacokinetics of Oral CPL304110, in Adult Subjects With Advanced Solid Malignancies	I
NCT03473743	A Phase Ib-II Study to Evaluate Safety, Efficacy, Pharmacokinetics, and Pharmacodynamics of Various Regimens of Erdafitinib in Subjects With Metastatic or Locally Advanced Urothelial Cancer	I/II
NCT02393248	A Phase I/II, Open-Label, Dose-Escalation, Safety and Tolerability Study of INCB054828 in Subjects With Advanced Malignancies - (FIGHT-101)	I/II
NCT04572295	An Open-label Phase Ib Study of E7090 Monotherapy and in Combination With Other Anticancer Agents in Subjects With ER+, HER2- Recurrent/Metastatic Breast Cancer	I
NCT04504331	A Phase IB Study of Infigratinib in Combination With Tamoxifen in Hormone Receptor Positive, HER2 Negative, FGFR Altered Advanced Breast Cancer	I

FGFR3-BAIAP2L1 fusion

NCT ID	Title	Phase
No NCT ID	A Multicenter Phase II Basket-type Clinical Trial to Evaluate Efficacy and Safety of TAS-120 in Patients with Advanced Solid Malignancies with FGFR Alterations in Circulating Tumor DNA	II
NCT04189445	A Phase II Study of Futibatinib in Patients With Specific FGFR Aberrations	II
NCT04233567	A Phase II Study of Oral Infigratinib in Adult Patients With Advanced or Metastatic Solid Tumors With FGFR1-3 Gene Fusions or Other FGFR Genetic Alterations	II
NCT05019794	A Phase IIa of Infigratinib in Subjects With Locally Advanced or Metastatic Gastric Cancer or Gastroesophageal Junction Adenocarcinoma With FGFR2 Amplification or Other Advanced Solid Tumors With Other FGFR Alterations	II
NCT03929965	Anlotinib in Advanced Solid Tumors With FGFR Alteration	I
NCT04591431	The Rome Trial From Histology to Target: the Road to Personalize Target Therapy and Immunotherapy	II
NCT03297606	Canadian Profiling and Targeted Agent Utilization Trial (CAPTUR): A Phase II Basket Trial	II
NCT04565275	A Multi-center Open-label, Phase I/II Clinical Trial to Evaluate the Safety, Tolerability, Pharmacokinetics, and Pharmacodynamics of ICP-192 in Patients With Advanced Solid Tumors and FGFR Gene Alterations.	I/II
No NCT ID	Phase I/II Study of TAS-117 In Combination With TAS-120 In Patients With Advanced Solid Tumors	I/II
No NCT ID	A Phase Ib Study to Assess the Safety, Tolerability, and Efficacy of TAS-120 (Futibatinib) in Combination with MK-3475 (Pembrolizumab) in Patients with Solid Tumors.	I

Disclaimer: The data presented here is from a curated knowledgebase of publicly available information, but may not be exhaustive. The data version is 2021.11(003).

Clinical Trials Summary (continued)

FGFR3-BAIAP2L1 fusion (continued)

NCT ID	Title	Phase
NCT03390504	A Phase III Study of Erdafitinib Compared With Vinflunine or Docetaxel or Pembrolizumab in Subjects With Advanced Urothelial Cancer and Selected FGFR Gene Aberrations	III
NCT02365597	A Phase II, Two-arm Multicenter, Open-Label Study to Determine the Efficacy and the Safety of Two Different Dose Regimens of a Pan-FGFR Tyrosine Kinase Inhibitor JNJ-42756493 in Subjects With Metastatic or Surgically Unresectable Urothelial Cancer With FGFR Genomic Alterations.	II
NCT04492293	A Phase II, Multicenter, Single Arm, Open-lable Study of ICP-192 in Subjects with Surgically Unresectable or Metastatic Bladder Urothelial Cancer with FGFR Genetic Aberrations.	II
NCT04197986	Phase III, Multicenter, Double-Blind, Randomized, Placebo-Controlled Trial of Infigratinib for the Adjuvant Treatment of Subjects With Invasive Urothelial Carcinoma With Susceptible FGFR3 Genetic Alterations (PROOF 302).	III
NCT04601857	A Phase II Study Evaluating Futibatinib (TAS 120) Plus Pembrolizumab in the Treatment of Advanced or Metastatic Urothelial Carcinoma	II
NCT04936295	A Phase II Study of the Efficacy and Tolerability of Fulvestrant Plus Anlotinib in HR(+)/HER2(-) Metastatic Breast Cancer Patients With FGFR Mutation	II
NCT04096417	A Phase II, Multicenter, Single-Arm Study of Pemigatinib in Patients With Metastatic or Unresectable Colorectal Cancer Harboring FGFR Alterations	II
NCT04294277	Open-label, Single-arm, Phase II Study, Evaluating Safety and Efficacy of INCB054828 (Pemigatinib) as Adjuvant Therapy for Molecularly-selected, High-risk Patients With Urothelial Carcinoma Who Have Received Radical Surgery	II
NCT04077255	Anti-EGFR Antibody (GC-1118) in Combination With Weekly Paclitaxel as a Second-line Therapy for Gastric and Gastroesophageal Junction Adenocarcinomas With Amplifications	II
NCT04045613	An Open-label Multi-cohort Phase Ib/II Study of Derazantinib and Atezolizumab in Patients With Urothelial Cancer Expressing Activating Molecular FGFR Aberrations.	I/II
No NCT ID	Phase I Clinical Study of BPI-17509 In Patients With Advanced Solid Tumors	I
NCT03827850	A Phase II Trial to Evaluate Efficacy and Safety of Erdafitinib in Patients With Advanced Squamous Non Small Cell Lung Carcinoma (sqNSCLC) Harboring Fibroblast Growth Factor Receptor (FGFR) Genetic Alterations After Relapse of Standard Therapy.	II
NCT04172675	A Randomized Phase II Study of Erdafitinib Versus Investigator Choice of Intravesical Chemotherapy in Subjects Who Received Bacillus Calmette-Guerin (BCG) and Recurred With High Risk Non-Muscle-Invasive Bladder Cancer (NMIBC) and FGFR Mutations or Fusions	II
NCT02150967	A Phase II Multicenter, Single Arm Study of Oral BGJ398 in Adult Patients with Advanced or Metastatic Cholangiocarcinoma with FGFR2 Gene Fusions or Other FGFR Genetic Alterations who Failed or are Intolerant to Platinum-based Chemotherapy.	II
NCT04595747	Phase II Study of Rogaratinib (BAY 1163877) in the Treatment of Patients With Sarcoma Harboring Alterations in Fibroblast Growth Factor Receptor (FGFR) 1-4 and SDH-Deficient Gastrointestinal Stromal Tumor (GIST)	II
NCT04149691	A Phase I, Open-label, Multicentre, Dose Escalation Study to Assess Safety, Tolerability and Pharmacokinetics of Oral CPL304110, in Adult Subjects With Advanced Solid Malignancies	I
NCT03473743	A Phase Ib-II Study to Evaluate Safety, Efficacy, Pharmacokinetics, and Pharmacodynamics of Various Regimens of Erdafitinib in Subjects With Metastatic or Locally Advanced Urothelial Cancer	I/II
NCT02393248	A Phase I/II, Open-Label, Dose-Escalation, Safety and Tolerability Study of INCB054828 in Subjects With Advanced Malignancies - (FIGHT-101)	I/II

Disclaimer: The data presented here is from a curated knowledgebase of publicly available information, but may not be exhaustive. The data version is 2021.11(003).

Clinical Trials Summary (continued)

FGFR3-BAIAP2L1 fusion (continued)

NCT ID	Title	Phase
NCT04572295	An Open-label Phase Ib Study of E7090 Monotherapy and in Combination With Other Anticancer Agents in Subjects With ER+, HER2- Recurrent/Metastatic Breast Cancer	I
NCT04504331	A Phase IB Study of Infigratinib in Combination With Tamoxifen in Hormone Receptor Positive, HER2 Negative, FGFR Altered Advanced Breast Cancer	I

FGFR3 Y373C mutation

NCT ID	Title	Phase
NCT03155620	NCI-COG Pediatric MATCH (Molecular Analysis for Therapy Choice) Screening Protocol	II
NCT03210714	NCI-COG Pediatric MATCH (Molecular Analysis for Therapy Choice) - Phase II Subprotocol of JNJ-42756493 (Erdafitinib) in Patients With Tumors Harboring FGFR 1/2/3/4 Alterations	II
No NCT ID	A Multicenter Phase II Basket-type Clinical Trial to Evaluate Efficacy and Safety of TAS-120 in Patients with Advanced Solid Malignancies with FGFR Alterations in Circulating Tumor DNA	II
NCT02693535	Targeted Agent and Profiling Utilization Registry (TAPUR) Study.	II
NCT04591431	The Rome Trial From Histology to Target: the Road to Personalize Target Therapy and Immunotherapy	II
NCT02691793	Study to Evaluate the Safety and Efficacy of Sunitinib, in Subject With Refractory Solid Tumors	II
NCT03297606	Canadian Profiling and Targeted Agent Utilization Trial (CAPTUR): A Phase II Basket Trial	II
NCT03929965	Anlotinib in Advanced Solid Tumors With FGFR Alteration	I
NCT04565275	A Multi-center Open-label, Phase I/II Clinical Trial to Evaluate the Safety, Tolerability, Pharmacokinetics, and Pharmacodynamics of ICP-192 in Patients With Advanced Solid Tumors and FGFR Gene Alterations.	I/II
No NCT ID	Phase I/II Study of TAS-117 In Combination With TAS-120 In Patients With Advanced Solid Tumors	I/II
No NCT ID	A Phase Ib Study to Assess the Safety, Tolerability, and Efficacy of TAS-120 (Futibatinib) in Combination with MK-3475 (Pembrolizumab) in Patients with Solid Tumors.	I
NCT03390504	A Phase III Study of Erdafitinib Compared With Vinflunine or Docetaxel or Pembrolizumab in Subjects With Advanced Urothelial Cancer and Selected FGFR Gene Aberrations	III
NCT04197986	Phase III, Multicenter, Double-Blind, Randomized, Placebo-Controlled Trial of Infigratinib for the Adjuvant Treatment of Subjects With Invasive Urothelial Carcinoma With Susceptible FGFR3 Genetic Alterations (PROOF 302).	III
NCT02365597	A Phase II, Two-arm Multicenter, Open-Label Study to Determine the Efficacy and the Safety of Two Different Dose Regimens of a Pan-FGFR Tyrosine Kinase Inhibitor JNJ-42756493 in Subjects With Metastatic or Surgically Unresectable Urothelial Cancer With FGFR Genomic Alterations.	II
NCT04492293	A Phase II, Multicenter, Single Arm, Open-lable Study of ICP-192 in Subjects with Surgically Unresectable or Metastatic Bladder Urothelial Cancer with FGFR Genetic Aberrations.	II
NCT04053322	An International Multicenter Phase II Trial of Durvalumab (MEDI4736) Plus OLAparib Plus Fulvestrant in Metastatic or Locally Advanced ER-positive, HER2-negative Breast Cancer Patients Selected Using Criteria That Predict Sensitivity to Olaparib	II
NCT04601857	A Phase II Study Evaluating Futibatinib (TAS 120) Plus Pembrolizumab in the Treatment of Advanced or Metastatic Urothelial Carcinoma	II

Clinical Trials Summary (continued)

FGFR3 Y373C mutation (continued)

NCT ID	Title	Phase
NCT04096417	A Phase II, Multicenter, Single-Arm Study of Pemigatinib in Patients With Metastatic or Unresectable Colorectal Cancer Harboring FGFR Alterations	II
NCT04294277	Open-label, Single-arm, Phase II Study, Evaluating Safety and Efficacy of INCB054828 (Pemigatinib) as Adjuvant Therapy for Molecularly-selected, High-risk Patients With Urothelial Carcinoma Who Have Received Radical Surgery	II
NCT04077255	Anti-EGFR Antibody (GC-1118) in Combination With Weekly Paclitaxel as a Second-line Therapy for Gastric and Gastroesophageal Junction Adenocarcinomas With Amplifications	II
No NCT ID	Phase I Clinical Study of BPI-17509 In Patients With Advanced Solid Tumors	I
NCT04172675	A Randomized Phase II Study of Erdafitinib Versus Investigator Choice of Intravesical Chemotherapy in Subjects Who Received Bacillus Calmette-Guerin (BCG) and Recurred With High Risk Non-Muscle-Invasive Bladder Cancer (NMIBC) and FGFR Mutations or Fusions	II
NCT04595747	Phase II Study of Rogaratinib (BAY 1163877) in the Treatment of Patients With Sarcoma Harboring Alterations in Fibroblast Growth Factor Receptor (FGFR) 1-4 and SDH-Deficient Gastrointestinal Stromal Tumor (GIST)	II
NCT04004975	Phase II Clinical Trials on Anlotinib for the Treatment of Recurrent Glioblastoma	I/II
NCT04149691	A Phase I, Open-label, Multicentre, Dose Escalation Study to Assess Safety, Tolerability and Pharmacokinetics of Oral CPL304110, in Adult Subjects With Advanced Solid Malignancies	I
NCT03473743	A Phase Ib-II Study to Evaluate Safety, Efficacy, Pharmacokinetics, and Pharmacodynamics of Various Regimens of Erdafitinib in Subjects With Metastatic or Locally Advanced Urothelial Cancer	I/II
NCT02393248	A Phase I/II, Open-Label, Dose-Escalation, Safety and Tolerability Study of INCB054828 in Subjects With Advanced Malignancies - (FIGHT-101)	I/II
NCT04572295	An Open-label Phase Ib Study of E7090 Monotherapy and in Combination With Other Anticancer Agents in Subjects With ER+, HER2- Recurrent/Metastatic Breast Cancer	I
NCT04504331	A Phase IB Study of Infigratinib in Combination With Tamoxifen in Hormone Receptor Positive, HER2 Negative, FGFR Altered Advanced Breast Cancer	I

FGFR3 K650E mutation

NCT ID	Title	Phase
NCT05019794	A Phase IIa of Infigratinib in Subjects With Locally Advanced or Metastatic Gastric Cancer or Gastroesophageal Junction Adenocarcinoma With FGFR2 Amplification or Other Advanced Solid Tumors With Other FGFR Alterations	II
NCT03155620	NCI-COG Pediatric MATCH (Molecular Analysis for Therapy Choice) Screening Protocol	II
NCT03210714	NCI-COG Pediatric MATCH (Molecular Analysis for Therapy Choice) - Phase II Subprotocol of JNJ-42756493 (Erdafitinib) in Patients With Tumors Harboring FGFR 1/2/3/4 Alterations	II
No NCT ID	A Multicenter Phase II Basket-type Clinical Trial to Evaluate Efficacy and Safety of TAS-120 in Patients with Advanced Solid Malignancies with FGFR Alterations in Circulating Tumor DNA	II
NCT04233567	A Phase II Study of Oral Infigratinib in Adult Patients With Advanced or Metastatic Solid Tumors With FGFR1-3 Gene Fusions or Other FGFR Genetic Alterations	II
NCT02693535	Targeted Agent and Profiling Utilization Registry (TAPUR) Study.	II
NCT04591431	The Rome Trial From Histology to Target: the Road to Personalize Target Therapy and Immunotherapy	II

Disclaimer: The data presented here is from a curated knowledgebase of publicly available information, but may not be exhaustive. The data version is 2021.11(003).

Clinical Trials Summary (continued)

FGFR3 K650E mutation (continued)

NCT ID	Title	Phase
NCT02691793	Study to Evaluate the Safety and Efficacy of Sunitinib, in Subject With Refractory Solid Tumors	II
NCT03297606	Canadian Profiling and Targeted Agent Utilization Trial (CAPTUR): A Phase II Basket Trial	II
NCT03929965	Anlotinib in Advanced Solid Tumors With FGFR Alteration	I
NCT04565275	A Multi-center Open-label, Phase I/II Clinical Trial to Evaluate the Safety, Tolerability, Pharmacokinetics, and Pharmacodynamics of ICP-192 in Patients With Advanced Solid Tumors and FGFR Gene Alterations.	I/II
No NCT ID	Phase I/II Study of TAS-117 In Combination With TAS-120 In Patients With Advanced Solid Tumors	I/II
No NCT ID	A Phase Ib Study to Assess the Safety, Tolerability, and Efficacy of TAS-120 (Futibatinib) in Combination with MK-3475 (Pembrolizumab) in Patients with Solid Tumors.	I
NCT04197986	Phase III, Multicenter, Double-Blind, Randomized, Placebo-Controlled Trial of Infigratinib for the Adjuvant Treatment of Subjects With Invasive Urothelial Carcinoma With Susceptible FGFR3 Genetic Alterations (PROOF 302).	III
NCT04424966	A Phase 0 Study of Infigratinib in Recurrent High-Grade Glioma Participants Scheduled for Resection to Evaluate Central Nervous System (CNS) Penetration With PK Triggered Expansion Cohort	I
NCT04936295	A Phase II Study of the Efficacy and Tolerability of Fulvestrant Plus Anlotinib in HR(+)/HER2(-) Metastatic Breast Cancer Patients With FGFR Mutation	II
NCT04096417	A Phase II, Multicenter, Single-Arm Study of Pemigatinib in Patients With Metastatic or Unresectable Colorectal Cancer Harboring FGFR Alterations	II
NCT04045613	An Open-label Multi-cohort Phase Ib/II Study of Derazantinib and Atezolizumab in Patients With Urothelial Cancer Expressing Activating Molecular FGFR Aberrations.	I/II
NCT04053322	An International Multicenter Phase II Trial of Durvalumab (MEDI4736) Plus OLAparib Plus Fulvestrant in Metastatic or Locally Advanced ER-positive, HER2-negative Breast Cancer Patients Selected Using Criteria That Predict Sensitivity to Olaparib	II
NCT03827850	A Phase II Trial to Evaluate Efficacy and Safety of Erdafitinib in Patients With Advanced Squamous Non Small Cell Lung Carcinoma (sqNSCLC) Harboring Fibroblast Growth Factor Receptor (FGFR) Genetic Alterations After Relapse of Standard Therapy.	II
NCT04601857	A Phase II Study Evaluating Futibatinib (TAS 120) Plus Pembrolizumab in the Treatment of Advanced or Metastatic Urothelial Carcinoma	II
NCT04294277	Open-label, Single-arm, Phase II Study, Evaluating Safety and Efficacy of INCB054828 (Pemigatinib) as Adjuvant Therapy for Molecularly-selected, High-risk Patients With Urothelial Carcinoma Who Have Received Radical Surgery	II
NCT04077255	Anti-EGFR Antibody (GC-1118) in Combination With Weekly Paclitaxel as a Second-line Therapy for Gastric and Gastroesophageal Junction Adenocarcinomas With Amplifications	II
No NCT ID	Phase I Clinical Study of BPI-17509 In Patients With Advanced Solid Tumors	I
NCT04172675	A Randomized Phase II Study of Erdafitinib Versus Investigator Choice of Intravesical Chemotherapy in Subjects Who Received Bacillus Calmette-Guerin (BCG) and Recurred With High Risk Non-Muscle-Invasive Bladder Cancer (NMIBC) and FGFR Mutations or Fusions	II
NCT02150967	A Phase II Multicenter, Single Arm Study of Oral BGJ398 in Adult Patients with Advanced or Metastatic Cholangiocarcinoma with FGFR2 Gene Fusions or Other FGFR Genetic Alterations who Failed or are Intolerant to Platinum-based Chemotherapy.	II

Disclaimer: The data presented here is from a curated knowledgebase of publicly available information, but may not be exhaustive. The data version is 2021.11(003).

Clinical Trials Summary (continued)

FGFR3 K650E mutation (continued)

NCT ID	Title	Phase
NCT04595747	Phase II Study of Rogaratinib (BAY 1163877) in the Treatment of Patients With Sarcoma Harboring Alterations in Fibroblast Growth Factor Receptor (FGFR) 1-4 and SDH-Deficient Gastrointestinal Stromal Tumor (GIST)	II
NCT04004975	Phase II Clinical Trials on Anlotinib for the Treatment of Recurrent Glioblastoma	I/II
NCT04149691	A Phase I, Open-label, Multicentre, Dose Escalation Study to Assess Safety, Tolerability and Pharmacokinetics of Oral CPL304110, in Adult Subjects With Advanced Solid Malignancies	I
NCT03473743	A Phase Ib-II Study to Evaluate Safety, Efficacy, Pharmacokinetics, and Pharmacodynamics of Various Regimens of Erdafitinib in Subjects With Metastatic or Locally Advanced Urothelial Cancer	I/II
NCT02393248	A Phase I/II, Open-Label, Dose-Escalation, Safety and Tolerability Study of INCB054828 in Subjects With Advanced Malignancies - (FIGHT-101)	I/II
NCT04572295	An Open-label Phase Ib Study of E7090 Monotherapy and in Combination With Other Anticancer Agents in Subjects With ER+, HER2- Recurrent/Metastatic Breast Cancer	I
NCT04504331	A Phase IB Study of Infigratinib in Combination With Tamoxifen in Hormone Receptor Positive, HER2 Negative, FGFR Altered Advanced Breast Cancer	I

FGFR3 K650M mutation

NCT ID	Title	Phase
NCT05019794	A Phase IIa of Infigratinib in Subjects With Locally Advanced or Metastatic Gastric Cancer or Gastroesophageal Junction Adenocarcinoma With FGFR2 Amplification or Other Advanced Solid Tumors With Other FGFR Alterations	II
NCT03155620	NCI-COG Pediatric MATCH (Molecular Analysis for Therapy Choice) Screening Protocol	II
NCT03210714	NCI-COG Pediatric MATCH (Molecular Analysis for Therapy Choice) - Phase II Subprotocol of JNJ-42756493 (Erdafitinib) in Patients With Tumors Harboring FGFR 1/2/3/4 Alterations	II
No NCT ID	A Multicenter Phase II Basket-type Clinical Trial to Evaluate Efficacy and Safety of TAS-120 in Patients with Advanced Solid Malignancies with FGFR Alterations in Circulating Tumor DNA	II
NCT04233567	A Phase II Study of Oral Infigratinib in Adult Patients With Advanced or Metastatic Solid Tumors With FGFR1-3 Gene Fusions or Other FGFR Genetic Alterations	II
NCT02693535	Targeted Agent and Profiling Utilization Registry (TAPUR) Study.	II
NCT04591431	The Rome Trial From Histology to Target: the Road to Personalize Target Therapy and Immunotherapy	II
NCT02691793	Study to Evaluate the Safety and Efficacy of Sunitinib, in Subject With Refractory Solid Tumors	II
NCT03297606	Canadian Profiling and Targeted Agent Utilization Trial (CAPTUR): A Phase II Basket Trial	II
NCT03929965	Anlotinib in Advanced Solid Tumors With FGFR Alteration	I
NCT04565275	A Multi-center Open-label, Phase I/II Clinical Trial to Evaluate the Safety, Tolerability, Pharmacokinetics, and Pharmacodynamics of ICP-192 in Patients With Advanced Solid Tumors and FGFR Gene Alterations.	I/II
No NCT ID	Phase I/II Study of TAS-117 In Combination With TAS-120 In Patients With Advanced Solid Tumors	I/II
No NCT ID	A Phase Ib Study to Assess the Safety, Tolerability, and Efficacy of TAS-120 (Futibatinib) in Combination with MK-3475 (Pembrolizumab) in Patients with Solid Tumors.	I

Clinical Trials Summary (continued)

FGFR3 K650M mutation (continued)

NCT ID	Title	Phase
NCT04197986	Phase III, Multicenter, Double-Blind, Randomized, Placebo-Controlled Trial of Infigratinib for the Adjuvant Treatment of Subjects With Invasive Urothelial Carcinoma With Susceptible FGFR3 Genetic Alterations (PROOF 302).	III
NCT04936295	A Phase II Study of the Efficacy and Tolerability of Fulvestrant Plus Anlotinib in HR(+)/HER2(-) Metastatic Breast Cancer Patients With FGFR Mutation	II
NCT04096417	A Phase II, Multicenter, Single-Arm Study of Pemigatinib in Patients With Metastatic or Unresectable Colorectal Cancer Harboring FGFR Alterations	II
NCT04045613	An Open-label Multi-cohort Phase Ib/II Study of Derazantinib and Atezolizumab in Patients With Urothelial Cancer Expressing Activating Molecular FGFR Aberrations.	I/II
NCT04053322	An International Multicenter Phase II Trial of Durvalumab (MEDI4736) Plus OLAparib Plus Fulvestrant in Metastatic or Locally Advanced ER-positive, HER2-negative Breast Cancer Patients Selected Using Criteria That Predict Sensitivity to Olaparib	II
NCT03827850	A Phase II Trial to Evaluate Efficacy and Safety of Erdafitinib in Patients With Advanced Squamous Non Small Cell Lung Carcinoma (sqNSCLC) Harboring Fibroblast Growth Factor Receptor (FGFR) Genetic Alterations After Relapse of Standard Therapy.	II
NCT04601857	A Phase II Study Evaluating Futibatinib (TAS 120) Plus Pembrolizumab in the Treatment of Advanced or Metastatic Urothelial Carcinoma	II
NCT04294277	Open-label, Single-arm, Phase II Study, Evaluating Safety and Efficacy of INCB054828 (Pemigatinib) as Adjuvant Therapy for Molecularly-selected, High-risk Patients With Urothelial Carcinoma Who Have Received Radical Surgery	II
NCT04077255	Anti-EGFR Antibody (GC-1118) in Combination With Weekly Paclitaxel as a Second-line Therapy for Gastric and Gastroesophageal Junction Adenocarcinomas With Amplifications	II
No NCT ID	Phase I Clinical Study of BPI-17509 In Patients With Advanced Solid Tumors	I
NCT04172675	A Randomized Phase II Study of Erdafitinib Versus Investigator Choice of Intravesical Chemotherapy in Subjects Who Received Bacillus Calmette-Guerin (BCG) and Recurred With High Risk Non-Muscle-Invasive Bladder Cancer (NMIBC) and FGFR Mutations or Fusions	II
NCT02150967	A Phase II Multicenter, Single Arm Study of Oral BGJ398 in Adult Patients with Advanced or Metastatic Cholangiocarcinoma with FGFR2 Gene Fusions or Other FGFR Genetic Alterations who Failed or are Intolerant to Platinum-based Chemotherapy.	II
NCT04595747	Phase II Study of Rogaratinib (BAY 1163877) in the Treatment of Patients With Sarcoma Harboring Alterations in Fibroblast Growth Factor Receptor (FGFR) 1-4 and SDH-Deficient Gastrointestinal Stromal Tumor (GIST)	II
NCT04004975	Phase II Clinical Trials on Anlotinib for the Treatment of Recurrent Glioblastoma	I/II
NCT04149691	A Phase I, Open-label, Multicentre, Dose Escalation Study to Assess Safety, Tolerability and Pharmacokinetics of Oral CPL304110, in Adult Subjects With Advanced Solid Malignancies	I
NCT03473743	A Phase Ib-II Study to Evaluate Safety, Efficacy, Pharmacokinetics, and Pharmacodynamics of Various Regimens of Erdafitinib in Subjects With Metastatic or Locally Advanced Urothelial Cancer	I/II
NCT02393248	A Phase I/II, Open-Label, Dose-Escalation, Safety and Tolerability Study of INCB054828 in Subjects With Advanced Malignancies - (FIGHT-101)	I/II
NCT04572295	An Open-label Phase Ib Study of E7090 Monotherapy and in Combination With Other Anticancer Agents in Subjects With ER+, HER2- Recurrent/Metastatic Breast Cancer	I

Disclaimer: The data presented here is from a curated knowledgebase of publicly available information, but may not be exhaustive. The data version is 2021.11(003).

Clinical Trials Summary (continued)

FGFR3 K650M mutation (continued)

NCT ID	Title	Phase
NCT04504331	A Phase IB Study of Infigratinib in Combination With Tamoxifen in Hormone Receptor Positive, HER2 Negative, FGFR Altered Advanced Breast Cancer	I

FGFR3 K650Q mutation

NCT ID	Title	Phase
NCT05019794	A Phase IIa of Infigratinib in Subjects With Locally Advanced or Metastatic Gastric Cancer or Gastroesophageal Junction Adenocarcinoma With FGFR2 Amplification or Other Advanced Solid Tumors With Other FGFR Alterations	II
NCT03155620	NCI-COG Pediatric MATCH (Molecular Analysis for Therapy Choice) Screening Protocol	II
NCT03210714	NCI-COG Pediatric MATCH (Molecular Analysis for Therapy Choice) - Phase II Subprotocol of JNJ-42756493 (Erdafitinib) in Patients With Tumors Harboring FGFR 1/2/3/4 Alterations	II
No NCT ID	A Multicenter Phase II Basket-type Clinical Trial to Evaluate Efficacy and Safety of TAS-120 in Patients with Advanced Solid Malignancies with FGFR Alterations in Circulating Tumor DNA	II
NCT04233567	A Phase II Study of Oral Infigratinib in Adult Patients With Advanced or Metastatic Solid Tumors With FGFR1-3 Gene Fusions or Other FGFR Genetic Alterations	II
NCT02693535	Targeted Agent and Profiling Utilization Registry (TAPUR) Study.	II
NCT04591431	The Rome Trial From Histology to Target: the Road to Personalize Target Therapy and Immunotherapy	II
NCT02691793	Study to Evaluate the Safety and Efficacy of Sunitinib, in Subject With Refractory Solid Tumors	II
NCT03297606	Canadian Profiling and Targeted Agent Utilization Trial (CAPTUR): A Phase II Basket Trial	II
NCT03929965	Anlotinib in Advanced Solid Tumors With FGFR Alteration	I
NCT04565275	A Multi-center Open-label, Phase I/II Clinical Trial to Evaluate the Safety, Tolerability, Pharmacokinetics, and Pharmacodynamics of ICP-192 in Patients With Advanced Solid Tumors and FGFR Gene Alterations.	I/II
No NCT ID	Phase I/II Study of TAS-117 In Combination With TAS-120 In Patients With Advanced Solid Tumors	I/II
No NCT ID	A Phase Ib Study to Assess the Safety, Tolerability, and Efficacy of TAS-120 (Futibatinib) in Combination with MK-3475 (Pembrolizumab) in Patients with Solid Tumors.	I
NCT04197986	Phase III, Multicenter, Double-Blind, Randomized, Placebo-Controlled Trial of Infigratinib for the Adjuvant Treatment of Subjects With Invasive Urothelial Carcinoma With Susceptible FGFR3 Genetic Alterations (PROOF 302).	III
NCT04936295	A Phase II Study of the Efficacy and Tolerability of Fulvestrant Plus Anlotinib in HR(+)/HER2(-) Metastatic Breast Cancer Patients With FGFR Mutation	II
NCT04096417	A Phase II, Multicenter, Single-Arm Study of Pemigatinib in Patients With Metastatic or Unresectable Colorectal Cancer Harboring FGFR Alterations	II
NCT04045613	An Open-label Multi-cohort Phase Ib/II Study of Derazantinib and Atezolizumab in Patients With Urothelial Cancer Expressing Activating Molecular FGFR Aberrations.	I/II
NCT04053322	An International Multicenter Phase II Trial of Durvalumab (MEDI4736) Plus OLAparib Plus Fulvestrant in Metastatic or Locally Advanced ER-positive, HER2-negative Breast Cancer Patients Selected Using Criteria That Predict Sensitivity to Olaparib	II

Clinical Trials Summary (continued)

FGFR3 K650Q mutation (continued)

NCT ID	Title	Phase
NCT03827850	A Phase II Trial to Evaluate Efficacy and Safety of Erdafitinib in Patients With Advanced Squamous Non Small Cell Lung Carcinoma (sqNSCLC) Harboring Fibroblast Growth Factor Receptor (FGFR) Genetic Alterations After Relapse of Standard Therapy.	II
NCT04601857	A Phase II Study Evaluating Futibatinib (TAS 120) Plus Pembrolizumab in the Treatment of Advanced or Metastatic Urothelial Carcinoma	II
NCT04294277	Open-label, Single-arm, Phase II Study, Evaluating Safety and Efficacy of INCB054828 (Pemigatinib) as Adjuvant Therapy for Molecularly-selected, High-risk Patients With Urothelial Carcinoma Who Have Received Radical Surgery	II
NCT04077255	Anti-EGFR Antibody (GC-1118) in Combination With Weekly Paclitaxel as a Second-line Therapy for Gastric and Gastroesophageal Junction Adenocarcinomas With Amplifications	II
No NCT ID	Phase I Clinical Study of BPI-17509 In Patients With Advanced Solid Tumors	I
NCT04172675	A Randomized Phase II Study of Erdafitinib Versus Investigator Choice of Intravesical Chemotherapy in Subjects Who Received Bacillus Calmette-Guerin (BCG) and Recurred With High Risk Non-Muscle-Invasive Bladder Cancer (NMIBC) and FGFR Mutations or Fusions	II
NCT02150967	A Phase II Multicenter, Single Arm Study of Oral BGJ398 in Adult Patients with Advanced or Metastatic Cholangiocarcinoma with FGFR2 Gene Fusions or Other FGFR Genetic Alterations who Failed or are Intolerant to Platinum-based Chemotherapy.	II
NCT04595747	Phase II Study of Rogaratinib (BAY 1163877) in the Treatment of Patients With Sarcoma Harboring Alterations in Fibroblast Growth Factor Receptor (FGFR) 1-4 and SDH-Deficient Gastrointestinal Stromal Tumor (GIST)	II
NCT04004975	Phase II Clinical Trials on Anlotinib for the Treatment of Recurrent Glioblastoma	I/II
NCT04149691	A Phase I, Open-label, Multicentre, Dose Escalation Study to Assess Safety, Tolerability and Pharmacokinetics of Oral CPL304110, in Adult Subjects With Advanced Solid Malignancies	I
NCT03473743	A Phase Ib-II Study to Evaluate Safety, Efficacy, Pharmacokinetics, and Pharmacodynamics of Various Regimens of Erdafitinib in Subjects With Metastatic or Locally Advanced Urothelial Cancer	I/II
NCT02393248	A Phase I/II, Open-Label, Dose-Escalation, Safety and Tolerability Study of INCB054828 in Subjects With Advanced Malignancies - (FIGHT-101)	I/II
NCT04572295	An Open-label Phase Ib Study of E7090 Monotherapy and in Combination With Other Anticancer Agents in Subjects With ER+, HER2- Recurrent/Metastatic Breast Cancer	I
NCT04504331	A Phase IB Study of Infigratinib in Combination With Tamoxifen in Hormone Receptor Positive, HER2 Negative, FGFR Altered Advanced Breast Cancer	I

FGFR3 K650T mutation

NCT ID	Title	Phase
NCT05019794	A Phase IIa of Infigratinib in Subjects With Locally Advanced or Metastatic Gastric Cancer or Gastroesophageal Junction Adenocarcinoma With FGFR2 Amplification or Other Advanced Solid Tumors With Other FGFR Alterations	II
NCT03155620	NCI-COG Pediatric MATCH (Molecular Analysis for Therapy Choice) Screening Protocol	II
NCT03210714	NCI-COG Pediatric MATCH (Molecular Analysis for Therapy Choice) - Phase II Subprotocol of JNJ-42756493 (Erdafitinib) in Patients With Tumors Harboring FGFR 1/2/3/4 Alterations	II

Clinical Trials Summary (continued)

FGFR3 K650T mutation (continued)

NCT ID	Title	Phase
No NCT ID	A Multicenter Phase II Basket-type Clinical Trial to Evaluate Efficacy and Safety of TAS-120 in Patients with Advanced Solid Malignancies with FGFR Alterations in Circulating Tumor DNA	II
NCT04233567	A Phase II Study of Oral Infigratinib in Adult Patients With Advanced or Metastatic Solid Tumors With FGFR1-3 Gene Fusions or Other FGFR Genetic Alterations	II
NCT02693535	Targeted Agent and Profiling Utilization Registry (TAPUR) Study.	II
NCT04591431	The Rome Trial From Histology to Target: the Road to Personalize Target Therapy and Immunotherapy	II
NCT02691793	Study to Evaluate the Safety and Efficacy of Sunitinib, in Subject With Refractory Solid Tumors	II
NCT03297606	Canadian Profiling and Targeted Agent Utilization Trial (CAPTUR): A Phase II Basket Trial	II
NCT03929965	Anlotinib in Advanced Solid Tumors With FGFR Alteration	I
NCT04565275	A Multi-center Open-label, Phase I/II Clinical Trial to Evaluate the Safety, Tolerability, Pharmacokinetics, and Pharmacodynamics of ICP-192 in Patients With Advanced Solid Tumors and FGFR Gene Alterations.	I/II
No NCT ID	Phase I/II Study of TAS-117 In Combination With TAS-120 In Patients With Advanced Solid Tumors	I/II
No NCT ID	A Phase Ib Study to Assess the Safety, Tolerability, and Efficacy of TAS-120 (Futibatinib) in Combination with MK-3475 (Pembrolizumab) in Patients with Solid Tumors.	I
NCT04197986	Phase III, Multicenter, Double-Blind, Randomized, Placebo-Controlled Trial of Infigratinib for the Adjuvant Treatment of Subjects With Invasive Urothelial Carcinoma With Susceptible FGFR3 Genetic Alterations (PROOF 302).	III
NCT04936295	A Phase II Study of the Efficacy and Tolerability of Fulvestrant Plus Anlotinib in HR(+)/HER2(-) Metastatic Breast Cancer Patients With FGFR Mutation	II
NCT04096417	A Phase II, Multicenter, Single-Arm Study of Pemigatinib in Patients With Metastatic or Unresectable Colorectal Cancer Harboring FGFR Alterations	II
NCT04045613	An Open-label Multi-cohort Phase Ib/II Study of Derazantinib and Atezolizumab in Patients With Urothelial Cancer Expressing Activating Molecular FGFR Aberrations.	I/II
NCT04053322	An International Multicenter Phase II Trial of Durvalumab (MEDI4736) Plus OLAparib Plus Fulvestrant in Metastatic or Locally Advanced ER-positive, HER2-negative Breast Cancer Patients Selected Using Criteria That Predict Sensitivity to Olaparib	II
NCT03827850	A Phase II Trial to Evaluate Efficacy and Safety of Erdafitinib in Patients With Advanced Squamous Non Small Cell Lung Carcinoma (sqNSCLC) Harboring Fibroblast Growth Factor Receptor (FGFR) Genetic Alterations After Relapse of Standard Therapy.	II
NCT04601857	A Phase II Study Evaluating Futibatinib (TAS 120) Plus Pembrolizumab in the Treatment of Advanced or Metastatic Urothelial Carcinoma	II
NCT04294277	Open-label, Single-arm, Phase II Study, Evaluating Safety and Efficacy of INCB054828 (Pemigatinib) as Adjuvant Therapy for Molecularly-selected, High-risk Patients With Urothelial Carcinoma Who Have Received Radical Surgery	II
NCT04077255	Anti-EGFR Antibody (GC-1118) in Combination With Weekly Paclitaxel as a Second-line Therapy for Gastric and Gastroesophageal Junction Adenocarcinomas With Amplifications	II
No NCT ID	Phase I Clinical Study of BPI-17509 In Patients With Advanced Solid Tumors	I
NCT04172675	A Randomized Phase II Study of Erdafitinib Versus Investigator Choice of Intravesical Chemotherapy in Subjects Who Received Bacillus Calmette-Guerin (BCG) and Recurred With High Risk Non-Muscle-Invasive Bladder Cancer (NMIBC) and FGFR Mutations or Fusions	II

Disclaimer: The data presented here is from a curated knowledgebase of publicly available information, but may not be exhaustive. The data version is 2021.11(003).

Clinical Trials Summary (continued)

FGFR3 K650T mutation (continued)

NCT ID	Title	Phase
NCT02150967	A Phase II Multicenter, Single Arm Study of Oral BGJ398 in Adult Patients with Advanced or Metastatic Cholangiocarcinoma with FGFR2 Gene Fusions or Other FGFR Genetic Alterations who Failed or are Intolerant to Platinum-based Chemotherapy.	II
NCT04595747	Phase II Study of Rogaratinib (BAY 1163877) in the Treatment of Patients With Sarcoma Harboring Alterations in Fibroblast Growth Factor Receptor (FGFR) 1-4 and SDH-Deficient Gastrointestinal Stromal Tumor (GIST)	II
NCT04004975	Phase II Clinical Trials on Anlotinib for the Treatment of Recurrent Glioblastoma	I/II
NCT04149691	A Phase I, Open-label, Multicentre, Dose Escalation Study to Assess Safety, Tolerability and Pharmacokinetics of Oral CPL304110, in Adult Subjects With Advanced Solid Malignancies	I
NCT03473743	A Phase Ib-II Study to Evaluate Safety, Efficacy, Pharmacokinetics, and Pharmacodynamics of Various Regimens of Erdafitinib in Subjects With Metastatic or Locally Advanced Urothelial Cancer	I/II
NCT02393248	A Phase I/II, Open-Label, Dose-Escalation, Safety and Tolerability Study of INCB054828 in Subjects With Advanced Malignancies - (FIGHT-101)	I/II
NCT04572295	An Open-label Phase Ib Study of E7090 Monotherapy and in Combination With Other Anticancer Agents in Subjects With ER+, HER2- Recurrent/Metastatic Breast Cancer	I
NCT04504331	A Phase IB Study of Infigratinib in Combination With Tamoxifen in Hormone Receptor Positive, HER2 Negative, FGFR Altered Advanced Breast Cancer	I

FGFR3 activating mutation

NCT ID	Title	Phase
NCT05019794	A Phase IIa of Infigratinib in Subjects With Locally Advanced or Metastatic Gastric Cancer or Gastroesophageal Junction Adenocarcinoma With FGFR2 Amplification or Other Advanced Solid Tumors With Other FGFR Alterations	II
NCT03155620	NCI-COG Pediatric MATCH (Molecular Analysis for Therapy Choice) Screening Protocol	II
NCT03210714	NCI-COG Pediatric MATCH (Molecular Analysis for Therapy Choice) - Phase II Subprotocol of JNJ-42756493 (Erdafitinib) in Patients With Tumors Harboring FGFR 1/2/3/4 Alterations	II
No NCT ID	A Multicenter Phase II Basket-type Clinical Trial to Evaluate Efficacy and Safety of TAS-120 in Patients with Advanced Solid Malignancies with FGFR Alterations in Circulating Tumor DNA	II
NCT04233567	A Phase II Study of Oral Infigratinib in Adult Patients With Advanced or Metastatic Solid Tumors With FGFR1-3 Gene Fusions or Other FGFR Genetic Alterations	II
NCT02693535	Targeted Agent and Profiling Utilization Registry (TAPUR) Study.	II
NCT04591431	The Rome Trial From Histology to Target: the Road to Personalize Target Therapy and Immunotherapy	II
NCT02691793	Study to Evaluate the Safety and Efficacy of Sunitinib, in Subject With Refractory Solid Tumors	II
NCT03297606	Canadian Profiling and Targeted Agent Utilization Trial (CAPTUR): A Phase II Basket Trial	II
NCT03929965	Anlotinib in Advanced Solid Tumors With FGFR Alteration	I
NCT04565275	A Multi-center Open-label, Phase I/II Clinical Trial to Evaluate the Safety, Tolerability, Pharmacokinetics, and Pharmacodynamics of ICP-192 in Patients With Advanced Solid Tumors and FGFR Gene Alterations.	I/II
No NCT ID	Phase I/II Study of TAS-117 In Combination With TAS-120 In Patients With Advanced Solid Tumors	I/II

Disclaimer: The data presented here is from a curated knowledgebase of publicly available information, but may not be exhaustive. The data version is 2021.11(003).

Clinical Trials Summary (continued)

FGFR3 activating mutation (continued)

NCT ID	Title	Phase
No NCT ID	A Phase Ib Study to Assess the Safety, Tolerability, and Efficacy of TAS-120 (Futibatinib) in Combination with MK-3475 (Pembrolizumab) in Patients with Solid Tumors.	I
NCT04936295	A Phase II Study of the Efficacy and Tolerability of Fulvestrant Plus Anlotinib in HR(+)/HER2(-) Metastatic Breast Cancer Patients With FGFR Mutation	II
NCT04096417	A Phase II, Multicenter, Single-Arm Study of Pemigatinib in Patients With Metastatic or Unresectable Colorectal Cancer Harboring FGFR Alterations	II
NCT04045613	An Open-label Multi-cohort Phase Ib/II Study of Derazantinib and Atezolizumab in Patients With Urothelial Cancer Expressing Activating Molecular FGFR Aberrations.	I/II
NCT04053322	An International Multicenter Phase II Trial of Durvalumab (MEDI4736) Plus OLAparib Plus Fulvestrant in Metastatic or Locally Advanced ER-positive, HER2-negative Breast Cancer Patients Selected Using Criteria That Predict Sensitivity to Olaparib	II
NCT03827850	A Phase II Trial to Evaluate Efficacy and Safety of Erdafitinib in Patients With Advanced Squamous Non Small Cell Lung Carcinoma (sqNSCLC) Harboring Fibroblast Growth Factor Receptor (FGFR) Genetic Alterations After Relapse of Standard Therapy.	II
NCT04601857	A Phase II Study Evaluating Futibatinib (TAS 120) Plus Pembrolizumab in the Treatment of Advanced or Metastatic Urothelial Carcinoma	II
NCT04294277	Open-label, Single-arm, Phase II Study, Evaluating Safety and Efficacy of INCB054828 (Pemigatinib) as Adjuvant Therapy for Molecularly-selected, High-risk Patients With Urothelial Carcinoma Who Have Received Radical Surgery	II
NCT04077255	Anti-EGFR Antibody (GC-1118) in Combination With Weekly Paclitaxel as a Second-line Therapy for Gastric and Gastroesophageal Junction Adenocarcinomas With Amplifications	II
No NCT ID	Phase I Clinical Study of BPI-17509 In Patients With Advanced Solid Tumors	I
NCT04172675	A Randomized Phase II Study of Erdafitinib Versus Investigator Choice of Intravesical Chemotherapy in Subjects Who Received Bacillus Calmette-Guerin (BCG) and Recurred With High Risk Non-Muscle-Invasive Bladder Cancer (NMIBC) and FGFR Mutations or Fusions	II
NCT02150967	A Phase II Multicenter, Single Arm Study of Oral BGJ398 in Adult Patients with Advanced or Metastatic Cholangiocarcinoma with FGFR2 Gene Fusions or Other FGFR Genetic Alterations who Failed or are Intolerant to Platinum-based Chemotherapy.	II
NCT04595747	Phase II Study of Rogaratinib (BAY 1163877) in the Treatment of Patients With Sarcoma Harboring Alterations in Fibroblast Growth Factor Receptor (FGFR) 1-4 and SDH-Deficient Gastrointestinal Stromal Tumor (GIST)	II
NCT04004975	Phase II Clinical Trials on Anlotinib for the Treatment of Recurrent Glioblastoma	I/II
NCT04149691	A Phase I, Open-label, Multicentre, Dose Escalation Study to Assess Safety, Tolerability and Pharmacokinetics of Oral CPL304110, in Adult Subjects With Advanced Solid Malignancies	I
NCT03473743	A Phase Ib-II Study to Evaluate Safety, Efficacy, Pharmacokinetics, and Pharmacodynamics of Various Regimens of Erdafitinib in Subjects With Metastatic or Locally Advanced Urothelial Cancer	I/II
NCT02393248	A Phase I/II, Open-Label, Dose-Escalation, Safety and Tolerability Study of INCB054828 in Subjects With Advanced Malignancies - (FIGHT-101)	I/II
NCT04572295	An Open-label Phase Ib Study of E7090 Monotherapy and in Combination With Other Anticancer Agents in Subjects With ER+, HER2- Recurrent/Metastatic Breast Cancer	I
NCT04504331	A Phase IB Study of Infigratinib in Combination With Tamoxifen in Hormone Receptor Positive, HER2 Negative, FGFR Altered Advanced Breast Cancer	I

Disclaimer: The data presented here is from a curated knowledgebase of publicly available information, but may not be exhaustive. The data version is 2021.11(003).

Clinical Trials Summary (continued)

FGFR3 K652E mutation

NCT ID	Title	Phase
NCT05019794	A Phase IIa of Infigratinib in Subjects With Locally Advanced or Metastatic Gastric Cancer or Gastroesophageal Junction Adenocarcinoma With FGFR2 Amplification or Other Advanced Solid Tumors With Other FGFR Alterations	II
NCT03155620	NCI-COG Pediatric MATCH (Molecular Analysis for Therapy Choice) Screening Protocol	II
NCT03210714	NCI-COG Pediatric MATCH (Molecular Analysis for Therapy Choice) - Phase II Subprotocol of JNJ-42756493 (Erdafitinib) in Patients With Tumors Harboring FGFR 1/2/3/4 Alterations	II
No NCT ID	A Multicenter Phase II Basket-type Clinical Trial to Evaluate Efficacy and Safety of TAS-120 in Patients with Advanced Solid Malignancies with FGFR Alterations in Circulating Tumor DNA	II
NCT04233567	A Phase II Study of Oral Infigratinib in Adult Patients With Advanced or Metastatic Solid Tumors With FGFR1-3 Gene Fusions or Other FGFR Genetic Alterations	II
NCT02693535	Targeted Agent and Profiling Utilization Registry (TAPUR) Study.	II
NCT04591431	The Rome Trial From Histology to Target: the Road to Personalize Target Therapy and Immunotherapy	II
NCT02691793	Study to Evaluate the Safety and Efficacy of Sunitinib, in Subject With Refractory Solid Tumors	II
NCT03297606	Canadian Profiling and Targeted Agent Utilization Trial (CAPTUR): A Phase II Basket Trial	II
NCT03929965	Anlotinib in Advanced Solid Tumors With FGFR Alteration	I
NCT04565275	A Multi-center Open-label, Phase I/II Clinical Trial to Evaluate the Safety, Tolerability, Pharmacokinetics, and Pharmacodynamics of ICP-192 in Patients With Advanced Solid Tumors and FGFR Gene Alterations.	I/II
No NCT ID	Phase I/II Study of TAS-117 In Combination With TAS-120 In Patients With Advanced Solid Tumors	I/II
No NCT ID	A Phase Ib Study to Assess the Safety, Tolerability, and Efficacy of TAS-120 (Futibatinib) in Combination with MK-3475 (Pembrolizumab) in Patients with Solid Tumors.	I
NCT04936295	A Phase II Study of the Efficacy and Tolerability of Fulvestrant Plus Anlotinib in HR(+)/HER2(-) Metastatic Breast Cancer Patients With FGFR Mutation	II
NCT04096417	A Phase II, Multicenter, Single-Arm Study of Pemigatinib in Patients With Metastatic or Unresectable Colorectal Cancer Harboring FGFR Alterations	II
NCT04045613	An Open-label Multi-cohort Phase Ib/II Study of Derazantinib and Atezolizumab in Patients With Urothelial Cancer Expressing Activating Molecular FGFR Aberrations.	I/II
NCT04053322	An International Multicenter Phase II Trial of Durvalumab (MEDI4736) Plus OLAparib Plus Fulvestrant in Metastatic or Locally Advanced ER-positive, HER2-negative Breast Cancer Patients Selected Using Criteria That Predict Sensitivity to Olaparib	II
NCT03827850	A Phase II Trial to Evaluate Efficacy and Safety of Erdafitinib in Patients With Advanced Squamous Non Small Cell Lung Carcinoma (sqNSCLC) Harboring Fibroblast Growth Factor Receptor (FGFR) Genetic Alterations After Relapse of Standard Therapy.	II
NCT04601857	A Phase II Study Evaluating Futibatinib (TAS 120) Plus Pembrolizumab in the Treatment of Advanced or Metastatic Urothelial Carcinoma	II
NCT04294277	Open-label, Single-arm, Phase II Study, Evaluating Safety and Efficacy of INCB054828 (Pemigatinib) as Adjuvant Therapy for Molecularly-selected, High-risk Patients With Urothelial Carcinoma Who Have Received Radical Surgery	II
NCT04077255	Anti-EGFR Antibody (GC-1118) in Combination With Weekly Paclitaxel as a Second-line Therapy for Gastric and Gastroesophageal Junction Adenocarcinomas With Amplifications	II

Disclaimer: The data presented here is from a curated knowledgebase of publicly available information, but may not be exhaustive. The data version is 2021.11(003).

Clinical Trials Summary (continued)

FGFR3 K652E mutation (continued)

NCT ID	Title	Phase
No NCT ID	Phase I Clinical Study of BPI-17509 In Patients With Advanced Solid Tumors	I
NCT04172675	A Randomized Phase II Study of Erdafitinib Versus Investigator Choice of Intravesical Chemotherapy in Subjects Who Received Bacillus Calmette-Guerin (BCG) and Recurred With High Risk Non-Muscle-Invasive Bladder Cancer (NMIBC) and FGFR Mutations or Fusions	II
NCT02150967	A Phase II Multicenter, Single Arm Study of Oral BGJ398 in Adult Patients with Advanced or Metastatic Cholangiocarcinoma with FGFR2 Gene Fusions or Other FGFR Genetic Alterations who Failed or are Intolerant to Platinum-based Chemotherapy.	II
NCT04595747	Phase II Study of Rogaratinib (BAY 1163877) in the Treatment of Patients With Sarcoma Harboring Alterations in Fibroblast Growth Factor Receptor (FGFR) 1-4 and SDH-Deficient Gastrointestinal Stromal Tumor (GIST)	II
NCT04004975	Phase II Clinical Trials on Anlotinib for the Treatment of Recurrent Glioblastoma	I/II
NCT04149691	A Phase I, Open-label, Multicentre, Dose Escalation Study to Assess Safety, Tolerability and Pharmacokinetics of Oral CPL304110, in Adult Subjects With Advanced Solid Malignancies	I
NCT03473743	A Phase Ib-II Study to Evaluate Safety, Efficacy, Pharmacokinetics, and Pharmacodynamics of Various Regimens of Erdafitinib in Subjects With Metastatic or Locally Advanced Urothelial Cancer	I/II
NCT02393248	A Phase I/II, Open-Label, Dose-Escalation, Safety and Tolerability Study of INCB054828 in Subjects With Advanced Malignancies - (FIGHT-101)	I/II
NCT04572295	An Open-label Phase Ib Study of E7090 Monotherapy and in Combination With Other Anticancer Agents in Subjects With ER+, HER2- Recurrent/Metastatic Breast Cancer	I
NCT04504331	A Phase IB Study of Infigratinib in Combination With Tamoxifen in Hormone Receptor Positive, HER2 Negative, FGFR Altered Advanced Breast Cancer	I

FGFR3 fusion

NCT ID	Title	Phase
No NCT ID	A Multicenter Phase II Basket-type Clinical Trial to Evaluate Efficacy and Safety of TAS-120 in Patients with Advanced Solid Malignancies with FGFR Alterations in Circulating Tumor DNA	II
NCT04189445	A Phase II Study of Futibatinib in Patients With Specific FGFR Aberrations	II
NCT04233567	A Phase II Study of Oral Infigratinib in Adult Patients With Advanced or Metastatic Solid Tumors With FGFR1-3 Gene Fusions or Other FGFR Genetic Alterations	II
NCT05019794	A Phase IIa of Infigratinib in Subjects With Locally Advanced or Metastatic Gastric Cancer or Gastroesophageal Junction Adenocarcinoma With FGFR2 Amplification or Other Advanced Solid Tumors With Other FGFR Alterations	II
NCT03929965	Anlotinib in Advanced Solid Tumors With FGFR Alteration	I
NCT04591431	The Rome Trial From Histology to Target: the Road to Personalize Target Therapy and Immunotherapy	II
NCT03297606	Canadian Profiling and Targeted Agent Utilization Trial (CAPTUR): A Phase II Basket Trial	II
NCT04565275	A Multi-center Open-label, Phase I/II Clinical Trial to Evaluate the Safety, Tolerability, Pharmacokinetics, and Pharmacodynamics of ICP-192 in Patients With Advanced Solid Tumors and FGFR Gene Alterations.	I/II
No NCT ID	Phase I/II Study of TAS-117 In Combination With TAS-120 In Patients With Advanced Solid Tumors	I/II

Clinical Trials Summary (continued)

FGFR3 fusion (continued)

NCT ID	Title	Phase
No NCT ID	A Phase Ib Study to Assess the Safety, Tolerability, and Efficacy of TAS-120 (Futibatinib) in Combination with MK-3475 (Pembrolizumab) in Patients with Solid Tumors.	I
NCT04197986	Phase III, Multicenter, Double-Blind, Randomized, Placebo-Controlled Trial of Infigratinib for the Adjuvant Treatment of Subjects With Invasive Urothelial Carcinoma With Susceptible FGFR3 Genetic Alterations (PROOF 302).	III
NCT04601857	A Phase II Study Evaluating Futibatinib (TAS 120) Plus Pembrolizumab in the Treatment of Advanced or Metastatic Urothelial Carcinoma	II
NCT04936295	A Phase II Study of the Efficacy and Tolerability of Fulvestrant Plus Anlotinib in HR(+)/HER2(-) Metastatic Breast Cancer Patients With FGFR Mutation	II
NCT04096417	A Phase II, Multicenter, Single-Arm Study of Pemigatinib in Patients With Metastatic or Unresectable Colorectal Cancer Harboring FGFR Alterations	II
NCT04294277	Open-label, Single-arm, Phase II Study, Evaluating Safety and Efficacy of INCB054828 (Pemigatinib) as Adjuvant Therapy for Molecularly-selected, High-risk Patients With Urothelial Carcinoma Who Have Received Radical Surgery	II
NCT04077255	Anti-EGFR Antibody (GC-1118) in Combination With Weekly Paclitaxel as a Second-line Therapy for Gastric and Gastroesophageal Junction Adenocarcinomas With Amplifications	II
NCT04045613	An Open-label Multi-cohort Phase Ib/II Study of Derazantinib and Atezolizumab in Patients With Urothelial Cancer Expressing Activating Molecular FGFR Aberrations.	I/II
No NCT ID	Phase I Clinical Study of BPI-17509 In Patients With Advanced Solid Tumors	I
NCT03827850	A Phase II Trial to Evaluate Efficacy and Safety of Erdafitinib in Patients With Advanced Squamous Non Small Cell Lung Carcinoma (sqNSCLC) Harboring Fibroblast Growth Factor Receptor (FGFR) Genetic Alterations After Relapse of Standard Therapy.	II
NCT04172675	A Randomized Phase II Study of Erdafitinib Versus Investigator Choice of Intravesical Chemotherapy in Subjects Who Received Bacillus Calmette-Guerin (BCG) and Recurred With High Risk Non-Muscle-Invasive Bladder Cancer (NMIBC) and FGFR Mutations or Fusions	II
NCT02150967	A Phase II Multicenter, Single Arm Study of Oral BGJ398 in Adult Patients with Advanced or Metastatic Cholangiocarcinoma with FGFR2 Gene Fusions or Other FGFR Genetic Alterations who Failed or are Intolerant to Platinum-based Chemotherapy.	II
NCT04595747	Phase II Study of Rogaratinib (BAY 1163877) in the Treatment of Patients With Sarcoma Harboring Alterations in Fibroblast Growth Factor Receptor (FGFR) 1-4 and SDH-Deficient Gastrointestinal Stromal Tumor (GIST)	II
NCT04149691	A Phase I, Open-label, Multicentre, Dose Escalation Study to Assess Safety, Tolerability and Pharmacokinetics of Oral CPL304110, in Adult Subjects With Advanced Solid Malignancies	I
NCT03473743	A Phase Ib-II Study to Evaluate Safety, Efficacy, Pharmacokinetics, and Pharmacodynamics of Various Regimens of Erdafitinib in Subjects With Metastatic or Locally Advanced Urothelial Cancer	I/II
NCT02393248	A Phase I/II, Open-Label, Dose-Escalation, Safety and Tolerability Study of INCB054828 in Subjects With Advanced Malignancies - (FIGHT-101)	I/II
NCT04572295	An Open-label Phase Ib Study of E7090 Monotherapy and in Combination With Other Anticancer Agents in Subjects With ER+, HER2- Recurrent/Metastatic Breast Cancer	I
NCT04504331	A Phase IB Study of Infigratinib in Combination With Tamoxifen in Hormone Receptor Positive, HER2 Negative, FGFR Altered Advanced Breast Cancer	I

Disclaimer: The data presented here is from a curated knowledgebase of publicly available information, but may not be exhaustive. The data version is 2021.11(003).

Clinical Trials Summary (continued)

FGFR3 A391E mutation

NCT ID	Title	Phase
NCT03155620	NCI-COG Pediatric MATCH (Molecular Analysis for Therapy Choice) Screening Protocol	II
NCT03210714	NCI-COG Pediatric MATCH (Molecular Analysis for Therapy Choice) - Phase II Subprotocol of JNJ-42756493 (Erdafitinib) in Patients With Tumors Harboring FGFR 1/2/3/4 Alterations	II
No NCT ID	A Multicenter Phase II Basket-type Clinical Trial to Evaluate Efficacy and Safety of TAS-120 in Patients with Advanced Solid Malignancies with FGFR Alterations in Circulating Tumor DNA	II
NCT02693535	Targeted Agent and Profiling Utilization Registry (TAPUR) Study.	II
NCT04591431	The Rome Trial From Histology to Target: the Road to Personalize Target Therapy and Immunotherapy	II
NCT02691793	Study to Evaluate the Safety and Efficacy of Sunitinib, in Subject With Refractory Solid Tumors	II
NCT03297606	Canadian Profiling and Targeted Agent Utilization Trial (CAPTUR): A Phase II Basket Trial	II
NCT03929965	Anlotinib in Advanced Solid Tumors With FGFR Alteration	I
NCT04565275	A Multi-center Open-label, Phase I/II Clinical Trial to Evaluate the Safety, Tolerability, Pharmacokinetics, and Pharmacodynamics of ICP-192 in Patients With Advanced Solid Tumors and FGFR Gene Alterations.	I/II
No NCT ID	Phase I/II Study of TAS-117 In Combination With TAS-120 In Patients With Advanced Solid Tumors	I/II
No NCT ID	A Phase Ib Study to Assess the Safety, Tolerability, and Efficacy of TAS-120 (Futibatinib) in Combination with MK-3475 (Pembrolizumab) in Patients with Solid Tumors.	I
NCT04197986	Phase III, Multicenter, Double-Blind, Randomized, Placebo-Controlled Trial of Infigratinib for the Adjuvant Treatment of Subjects With Invasive Urothelial Carcinoma With Susceptible FGFR3 Genetic Alterations (PROOF 302).	III
NCT04053322	An International Multicenter Phase II Trial of Durvalumab (MEDI4736) Plus OLAparib Plus Fulvestrant in Metastatic or Locally Advanced ER-positive, HER2-negative Breast Cancer Patients Selected Using Criteria That Predict Sensitivity to Olaparib	II
NCT04601857	A Phase II Study Evaluating Futibatinib (TAS 120) Plus Pembrolizumab in the Treatment of Advanced or Metastatic Urothelial Carcinoma	II
NCT04096417	A Phase II, Multicenter, Single-Arm Study of Pemigatinib in Patients With Metastatic or Unresectable Colorectal Cancer Harboring FGFR Alterations	II
NCT04294277	Open-label, Single-arm, Phase II Study, Evaluating Safety and Efficacy of INCB054828 (Pemigatinib) as Adjuvant Therapy for Molecularly-selected, High-risk Patients With Urothelial Carcinoma Who Have Received Radical Surgery	II
NCT04077255	Anti-EGFR Antibody (GC-1118) in Combination With Weekly Paclitaxel as a Second-line Therapy for Gastric and Gastroesophageal Junction Adenocarcinomas With Amplifications	II
No NCT ID	Phase I Clinical Study of BPI-17509 In Patients With Advanced Solid Tumors	I
NCT04172675	A Randomized Phase II Study of Erdafitinib Versus Investigator Choice of Intravesical Chemotherapy in Subjects Who Received Bacillus Calmette-Guerin (BCG) and Recurred With High Risk Non-Muscle-Invasive Bladder Cancer (NMIBC) and FGFR Mutations or Fusions	II
NCT04595747	Phase II Study of Rogaratinib (BAY 1163877) in the Treatment of Patients With Sarcoma Harboring Alterations in Fibroblast Growth Factor Receptor (FGFR) 1-4 and SDH-Deficient Gastrointestinal Stromal Tumor (GIST)	II
NCT04004975	Phase II Clinical Trials on Anlotinib for the Treatment of Recurrent Glioblastoma	I/II

Disclaimer: The data presented here is from a curated knowledgebase of publicly available information, but may not be exhaustive. The data version is 2021.11(003).

Clinical Trials Summary (continued)

FGFR3 A391E mutation (continued)

NCT ID	Title	Phase
NCT04149691	A Phase I, Open-label, Multicentre, Dose Escalation Study to Assess Safety, Tolerability and Pharmacokinetics of Oral CPL304110, in Adult Subjects With Advanced Solid Malignancies	I
NCT03473743	A Phase Ib-II Study to Evaluate Safety, Efficacy, Pharmacokinetics, and Pharmacodynamics of Various Regimens of Erdafitinib in Subjects With Metastatic or Locally Advanced Urothelial Cancer	I/II
NCT02393248	A Phase I/II, Open-Label, Dose-Escalation, Safety and Tolerability Study of INCB054828 in Subjects With Advanced Malignancies - (FIGHT-101)	I/II
NCT04572295	An Open-label Phase Ib Study of E7090 Monotherapy and in Combination With Other Anticancer Agents in Subjects With ER+, HER2- Recurrent/Metastatic Breast Cancer	I
NCT04504331	A Phase IB Study of Infigratinib in Combination With Tamoxifen in Hormone Receptor Positive, HER2 Negative, FGFR Altered Advanced Breast Cancer	I

FGFR3 KD mutation

NCT ID	Title	Phase
NCT03155620	NCI-COG Pediatric MATCH (Molecular Analysis for Therapy Choice) Screening Protocol	II
NCT03210714	NCI-COG Pediatric MATCH (Molecular Analysis for Therapy Choice) - Phase II Subprotocol of JNJ-42756493 (Erdafitinib) in Patients With Tumors Harboring FGFR 1/2/3/4 Alterations	II
No NCT ID	A Multicenter Phase II Basket-type Clinical Trial to Evaluate Efficacy and Safety of TAS-120 in Patients with Advanced Solid Malignancies with FGFR Alterations in Circulating Tumor DNA	II
NCT02693535	Targeted Agent and Profiling Utilization Registry (TAPUR) Study.	II
NCT04591431	The Rome Trial From Histology to Target: the Road to Personalize Target Therapy and Immunotherapy	II
NCT02691793	Study to Evaluate the Safety and Efficacy of Sunitinib, in Subject With Refractory Solid Tumors	II
NCT03297606	Canadian Profiling and Targeted Agent Utilization Trial (CAPTUR): A Phase II Basket Trial	II
NCT03929965	Anlotinib in Advanced Solid Tumors With FGFR Alteration	I
NCT04565275	A Multi-center Open-label, Phase I/II Clinical Trial to Evaluate the Safety, Tolerability, Pharmacokinetics, and Pharmacodynamics of ICP-192 in Patients With Advanced Solid Tumors and FGFR Gene Alterations.	I/II
No NCT ID	Phase I/II Study of TAS-117 In Combination With TAS-120 In Patients With Advanced Solid Tumors	I/II
No NCT ID	A Phase Ib Study to Assess the Safety, Tolerability, and Efficacy of TAS-120 (Futibatinib) in Combination with MK-3475 (Pembrolizumab) in Patients with Solid Tumors.	I
NCT04053322	An International Multicenter Phase II Trial of Durvalumab (MEDI4736) Plus OLAparib Plus Fulvestrant in Metastatic or Locally Advanced ER-positive, HER2-negative Breast Cancer Patients Selected Using Criteria That Predict Sensitivity to Olaparib	II
NCT04601857	A Phase II Study Evaluating Futibatinib (TAS 120) Plus Pembrolizumab in the Treatment of Advanced or Metastatic Urothelial Carcinoma	II
NCT04096417	A Phase II, Multicenter, Single-Arm Study of Pemigatinib in Patients With Metastatic or Unresectable Colorectal Cancer Harboring FGFR Alterations	II
NCT04294277	Open-label, Single-arm, Phase II Study, Evaluating Safety and Efficacy of INCB054828 (Pemigatinib) as Adjuvant Therapy for Molecularly-selected, High-risk Patients With Urothelial Carcinoma Who Have Received Radical Surgery	II

Disclaimer: The data presented here is from a curated knowledgebase of publicly available information, but may not be exhaustive. The data version is 2021.11(003).

Clinical Trials Summary (continued)

FGFR3 KD mutation (continued)

NCT ID	Title	Phase
NCT04077255	Anti-EGFR Antibody (GC-1118) in Combination With Weekly Paclitaxel as a Second-line Therapy for Gastric and Gastroesophageal Junction Adenocarcinomas With Amplifications	II
No NCT ID	Phase I Clinical Study of BPI-17509 In Patients With Advanced Solid Tumors	I
NCT04172675	A Randomized Phase II Study of Erdafitinib Versus Investigator Choice of Intravesical Chemotherapy in Subjects Who Received Bacillus Calmette-Guerin (BCG) and Recurred With High Risk Non-Muscle-Invasive Bladder Cancer (NMIBC) and FGFR Mutations or Fusions	II
NCT04595747	Phase II Study of Rogaratinib (BAY 1163877) in the Treatment of Patients With Sarcoma Harboring Alterations in Fibroblast Growth Factor Receptor (FGFR) 1-4 and SDH-Deficient Gastrointestinal Stromal Tumor (GIST)	II
NCT04004975	Phase II Clinical Trials on Anlotinib for the Treatment of Recurrent Glioblastoma	I/II
NCT04149691	A Phase I, Open-label, Multicentre, Dose Escalation Study to Assess Safety, Tolerability and Pharmacokinetics of Oral CPL304110, in Adult Subjects With Advanced Solid Malignancies	I
NCT03473743	A Phase Ib-II Study to Evaluate Safety, Efficacy, Pharmacokinetics, and Pharmacodynamics of Various Regimens of Erdafitinib in Subjects With Metastatic or Locally Advanced Urothelial Cancer	I/II
NCT02393248	A Phase I/II, Open-Label, Dose-Escalation, Safety and Tolerability Study of INCB054828 in Subjects With Advanced Malignancies - (FIGHT-101)	I/II
NCT04572295	An Open-label Phase Ib Study of E7090 Monotherapy and in Combination With Other Anticancer Agents in Subjects With ER+, HER2- Recurrent/Metastatic Breast Cancer	I
NCT04504331	A Phase IB Study of Infigratinib in Combination With Tamoxifen in Hormone Receptor Positive, HER2 Negative, FGFR Altered Advanced Breast Cancer	I

FGFR3 mutation

NCT ID	Title	Phase
NCT03155620	NCI-COG Pediatric MATCH (Molecular Analysis for Therapy Choice) Screening Protocol	II
NCT03210714	NCI-COG Pediatric MATCH (Molecular Analysis for Therapy Choice) - Phase II Subprotocol of JNJ-42756493 (Erdafitinib) in Patients With Tumors Harboring FGFR 1/2/3/4 Alterations	II
No NCT ID	A Multicenter Phase II Basket-type Clinical Trial to Evaluate Efficacy and Safety of TAS-120 in Patients with Advanced Solid Malignancies with FGFR Alterations in Circulating Tumor DNA	II
NCT02693535	Targeted Agent and Profiling Utilization Registry (TAPUR) Study.	II
NCT04591431	The Rome Trial From Histology to Target: the Road to Personalize Target Therapy and Immunotherapy	II
NCT02691793	Study to Evaluate the Safety and Efficacy of Sunitinib, in Subject With Refractory Solid Tumors	II
NCT03297606	Canadian Profiling and Targeted Agent Utilization Trial (CAPTUR): A Phase II Basket Trial	II
NCT03929965	Anlotinib in Advanced Solid Tumors With FGFR Alteration	I
NCT04565275	A Multi-center Open-label, Phase I/II Clinical Trial to Evaluate the Safety, Tolerability, Pharmacokinetics, and Pharmacodynamics of ICP-192 in Patients With Advanced Solid Tumors and FGFR Gene Alterations.	I/II
No NCT ID	Phase I/II Study of TAS-117 In Combination With TAS-120 In Patients With Advanced Solid Tumors	I/II

Disclaimer: The data presented here is from a curated knowledgebase of publicly available information, but may not be exhaustive. The data version is 2021.11(003).

Clinical Trials Summary (continued)

FGFR3 mutation (continued)

NCT ID	Title	Phase
No NCT ID	A Phase Ib Study to Assess the Safety, Tolerability, and Efficacy of TAS-120 (Futibatinib) in Combination with MK-3475 (Pembrolizumab) in Patients with Solid Tumors.	I
NCT04053322	An International Multicenter Phase II Trial of Durvalumab (MEDI4736) Plus OLAparib Plus Fulvestrant in Metastatic or Locally Advanced ER-positive, HER2-negative Breast Cancer Patients Selected Using Criteria That Predict Sensitivity to Olaparib	II
NCT04601857	A Phase II Study Evaluating Futibatinib (TAS 120) Plus Pembrolizumab in the Treatment of Advanced or Metastatic Urothelial Carcinoma	II
NCT04096417	A Phase II, Multicenter, Single-Arm Study of Pemigatinib in Patients With Metastatic or Unresectable Colorectal Cancer Harboring FGFR Alterations	II
NCT04294277	Open-label, Single-arm, Phase II Study, Evaluating Safety and Efficacy of INCB054828 (Pemigatinib) as Adjuvant Therapy for Molecularly-selected, High-risk Patients With Urothelial Carcinoma Who Have Received Radical Surgery	II
NCT04077255	Anti-EGFR Antibody (GC-1118) in Combination With Weekly Paclitaxel as a Second-line Therapy for Gastric and Gastroesophageal Junction Adenocarcinomas With Amplifications	II
No NCT ID	Phase I Clinical Study of BPI-17509 In Patients With Advanced Solid Tumors	I
NCT04172675	A Randomized Phase II Study of Erdafitinib Versus Investigator Choice of Intravesical Chemotherapy in Subjects Who Received Bacillus Calmette-Guerin (BCG) and Recurred With High Risk Non-Muscle-Invasive Bladder Cancer (NMIBC) and FGFR Mutations or Fusions	II
NCT04595747	Phase II Study of Rogaratinib (BAY 1163877) in the Treatment of Patients With Sarcoma Harboring Alterations in Fibroblast Growth Factor Receptor (FGFR) 1-4 and SDH-Deficient Gastrointestinal Stromal Tumor (GIST)	II
NCT04004975	Phase II Clinical Trials on Anlotinib for the Treatment of Recurrent Glioblastoma	I/II
NCT04149691	A Phase I, Open-label, Multicentre, Dose Escalation Study to Assess Safety, Tolerability and Pharmacokinetics of Oral CPL304110, in Adult Subjects With Advanced Solid Malignancies	I
NCT03473743	A Phase Ib-II Study to Evaluate Safety, Efficacy, Pharmacokinetics, and Pharmacodynamics of Various Regimens of Erdafitinib in Subjects With Metastatic or Locally Advanced Urothelial Cancer	I/II
NCT02393248	A Phase I/II, Open-Label, Dose-Escalation, Safety and Tolerability Study of INCB054828 in Subjects With Advanced Malignancies - (FIGHT-101)	I/II
NCT04572295	An Open-label Phase Ib Study of E7090 Monotherapy and in Combination With Other Anticancer Agents in Subjects With ER+, HER2- Recurrent/Metastatic Breast Cancer	I
NCT04504331	A Phase IB Study of Infigratinib in Combination With Tamoxifen in Hormone Receptor Positive, HER2 Negative, FGFR Altered Advanced Breast Cancer	I

FGFR3 V555 mutation

NCT ID	Title	Phase
NCT03155620	NCI-COG Pediatric MATCH (Molecular Analysis for Therapy Choice) Screening Protocol	II
NCT03210714	NCI-COG Pediatric MATCH (Molecular Analysis for Therapy Choice) - Phase II Subprotocol of JNJ-42756493 (Erdafitinib) in Patients With Tumors Harboring FGFR 1/2/3/4 Alterations	II
No NCT ID	A Multicenter Phase II Basket-type Clinical Trial to Evaluate Efficacy and Safety of TAS-120 in Patients with Advanced Solid Malignancies with FGFR Alterations in Circulating Tumor DNA	II

Disclaimer: The data presented here is from a curated knowledgebase of publicly available information, but may not be exhaustive. The data version is 2021.11(003).

Clinical Trials Summary (continued)

FGFR3 V555 mutation (continued)

NCT ID	Title	Phase
NCT02693535	Targeted Agent and Profiling Utilization Registry (TAPUR) Study.	II
NCT04591431	The Rome Trial From Histology to Target: the Road to Personalize Target Therapy and Immunotherapy	II
NCT02691793	Study to Evaluate the Safety and Efficacy of Sunitinib, in Subject With Refractory Solid Tumors	II
NCT03297606	Canadian Profiling and Targeted Agent Utilization Trial (CAPTUR): A Phase II Basket Trial	II
NCT03929965	Anlotinib in Advanced Solid Tumors With FGFR Alteration	I
NCT04565275	A Multi-center Open-label, Phase I/II Clinical Trial to Evaluate the Safety, Tolerability, Pharmacokinetics, and Pharmacodynamics of ICP-192 in Patients With Advanced Solid Tumors and FGFR Gene Alterations.	I/II
No NCT ID	Phase I/II Study of TAS-117 In Combination With TAS-120 In Patients With Advanced Solid Tumors	I/II
No NCT ID	A Phase Ib Study to Assess the Safety, Tolerability, and Efficacy of TAS-120 (Futibatinib) in Combination with MK-3475 (Pembrolizumab) in Patients with Solid Tumors.	I
NCT04053322	An International Multicenter Phase II Trial of Durvalumab (MEDI4736) Plus OLAparib Plus Fulvestrant in Metastatic or Locally Advanced ER-positive, HER2-negative Breast Cancer Patients Selected Using Criteria That Predict Sensitivity to Olaparib	II
NCT04601857	A Phase II Study Evaluating Futibatinib (TAS 120) Plus Pembrolizumab in the Treatment of Advanced or Metastatic Urothelial Carcinoma	II
NCT04096417	A Phase II, Multicenter, Single-Arm Study of Pemigatinib in Patients With Metastatic or Unresectable Colorectal Cancer Harboring FGFR Alterations	II
NCT04294277	Open-label, Single-arm, Phase II Study, Evaluating Safety and Efficacy of INCB054828 (Pemigatinib) as Adjuvant Therapy for Molecularly-selected, High-risk Patients With Urothelial Carcinoma Who Have Received Radical Surgery	II
NCT04077255	Anti-EGFR Antibody (GC-1118) in Combination With Weekly Paclitaxel as a Second-line Therapy for Gastric and Gastroesophageal Junction Adenocarcinomas With Amplifications	II
No NCT ID	Phase I Clinical Study of BPI-17509 In Patients With Advanced Solid Tumors	I
NCT04172675	A Randomized Phase II Study of Erdafitinib Versus Investigator Choice of Intravesical Chemotherapy in Subjects Who Received Bacillus Calmette-Guerin (BCG) and Recurred With High Risk Non-Muscle-Invasive Bladder Cancer (NMIBC) and FGFR Mutations or Fusions	II
NCT04595747	Phase II Study of Rogaratinib (BAY 1163877) in the Treatment of Patients With Sarcoma Harboring Alterations in Fibroblast Growth Factor Receptor (FGFR) 1-4 and SDH-Deficient Gastrointestinal Stromal Tumor (GIST)	II
NCT04004975	Phase II Clinical Trials on Anlotinib for the Treatment of Recurrent Glioblastoma	I/II
NCT04149691	A Phase I, Open-label, Multicentre, Dose Escalation Study to Assess Safety, Tolerability and Pharmacokinetics of Oral CPL304110, in Adult Subjects With Advanced Solid Malignancies	I
NCT03473743	A Phase Ib-II Study to Evaluate Safety, Efficacy, Pharmacokinetics, and Pharmacodynamics of Various Regimens of Erdafitinib in Subjects With Metastatic or Locally Advanced Urothelial Cancer	I/II
NCT02393248	A Phase I/II, Open-Label, Dose-Escalation, Safety and Tolerability Study of INCB054828 in Subjects With Advanced Malignancies - (FIGHT-101)	I/II
NCT04572295	An Open-label Phase Ib Study of E7090 Monotherapy and in Combination With Other Anticancer Agents in Subjects With ER+, HER2- Recurrent/Metastatic Breast Cancer	I

Disclaimer: The data presented here is from a curated knowledgebase of publicly available information, but may not be exhaustive. The data version is 2021.11(003).

Clinical Trials Summary (continued)

FGFR3 V555 mutation (continued)

NCT ID	Title	Phase
NCT04504331	A Phase IB Study of Infigratinib in Combination With Tamoxifen in Hormone Receptor Positive, HER2 Negative, FGFR Altered Advanced Breast Cancer	I

FGFR3 V557 mutation

NCT ID	Title	Phase
NCT03155620	NCI-COG Pediatric MATCH (Molecular Analysis for Therapy Choice) Screening Protocol	II
NCT03210714	NCI-COG Pediatric MATCH (Molecular Analysis for Therapy Choice) - Phase II Subprotocol of JNJ-42756493 (Erdafitinib) in Patients With Tumors Harboring FGFR 1/2/3/4 Alterations	II
No NCT ID	A Multicenter Phase II Basket-type Clinical Trial to Evaluate Efficacy and Safety of TAS-120 in Patients with Advanced Solid Malignancies with FGFR Alterations in Circulating Tumor DNA	II
NCT02693535	Targeted Agent and Profiling Utilization Registry (TAPUR) Study.	II
NCT04591431	The Rome Trial From Histology to Target: the Road to Personalize Target Therapy and Immunotherapy	II
NCT02691793	Study to Evaluate the Safety and Efficacy of Sunitinib, in Subject With Refractory Solid Tumors	II
NCT03297606	Canadian Profiling and Targeted Agent Utilization Trial (CAPTUR): A Phase II Basket Trial	II
NCT03929965	Anlotinib in Advanced Solid Tumors With FGFR Alteration	I
NCT04565275	A Multi-center Open-label, Phase I/II Clinical Trial to Evaluate the Safety, Tolerability, Pharmacokinetics, and Pharmacodynamics of ICP-192 in Patients With Advanced Solid Tumors and FGFR Gene Alterations.	I/II
No NCT ID	Phase I/II Study of TAS-117 In Combination With TAS-120 In Patients With Advanced Solid Tumors	I/II
No NCT ID	A Phase Ib Study to Assess the Safety, Tolerability, and Efficacy of TAS-120 (Futibatinib) in Combination with MK-3475 (Pembrolizumab) in Patients with Solid Tumors.	I
NCT04053322	An International Multicenter Phase II Trial of Durvalumab (MEDI4736) Plus OLAparib Plus Fulvestrant in Metastatic or Locally Advanced ER-positive, HER2-negative Breast Cancer Patients Selected Using Criteria That Predict Sensitivity to Olaparib	II
NCT04601857	A Phase II Study Evaluating Futibatinib (TAS 120) Plus Pembrolizumab in the Treatment of Advanced or Metastatic Urothelial Carcinoma	II
NCT04096417	A Phase II, Multicenter, Single-Arm Study of Pemigatinib in Patients With Metastatic or Unresectable Colorectal Cancer Harboring FGFR Alterations	II
NCT04294277	Open-label, Single-arm, Phase II Study, Evaluating Safety and Efficacy of INCB054828 (Pemigatinib) as Adjuvant Therapy for Molecularly-selected, High-risk Patients With Urothelial Carcinoma Who Have Received Radical Surgery	II
NCT04077255	Anti-EGFR Antibody (GC-1118) in Combination With Weekly Paclitaxel as a Second-line Therapy for Gastric and Gastroesophageal Junction Adenocarcinomas With Amplifications	II
No NCT ID	Phase I Clinical Study of BPI-17509 In Patients With Advanced Solid Tumors	I
NCT04172675	A Randomized Phase II Study of Erdafitinib Versus Investigator Choice of Intravesical Chemotherapy in Subjects Who Received Bacillus Calmette-Guerin (BCG) and Recurred With High Risk Non-Muscle-Invasive Bladder Cancer (NMIBC) and FGFR Mutations or Fusions	II

Clinical Trials Summary (continued)

FGFR3 V557 mutation (continued)

NCT ID	Title	Phase
NCT04595747	Phase II Study of Rogaratinib (BAY 1163877) in the Treatment of Patients With Sarcoma Harboring Alterations in Fibroblast Growth Factor Receptor (FGFR) 1-4 and SDH-Deficient Gastrointestinal Stromal Tumor (GIST)	II
NCT04004975	Phase II Clinical Trials on Anlotinib for the Treatment of Recurrent Glioblastoma	I/II
NCT04149691	A Phase I, Open-label, Multicentre, Dose Escalation Study to Assess Safety, Tolerability and Pharmacokinetics of Oral CPL304110, in Adult Subjects With Advanced Solid Malignancies	I
NCT03473743	A Phase Ib-II Study to Evaluate Safety, Efficacy, Pharmacokinetics, and Pharmacodynamics of Various Regimens of Erdafitinib in Subjects With Metastatic or Locally Advanced Urothelial Cancer	I/II
NCT02393248	A Phase I/II, Open-Label, Dose-Escalation, Safety and Tolerability Study of INCB054828 in Subjects With Advanced Malignancies - (FIGHT-101)	I/II
NCT04572295	An Open-label Phase Ib Study of E7090 Monotherapy and in Combination With Other Anticancer Agents in Subjects With ER+, HER2- Recurrent/Metastatic Breast Cancer	I
NCT04504331	A Phase IB Study of Infigratinib in Combination With Tamoxifen in Hormone Receptor Positive, HER2 Negative, FGFR Altered Advanced Breast Cancer	I

FGFR3 amplification

NCT ID	Title	Phase
NCT02693535	Targeted Agent and Profiling Utilization Registry (TAPUR) Study.	II
NCT04233567	A Phase II Study of Oral Infigratinib in Adult Patients With Advanced or Metastatic Solid Tumors With FGFR1-3 Gene Fusions or Other FGFR Genetic Alterations	II
NCT04591431	The Rome Trial From Histology to Target: the Road to Personalize Target Therapy and Immunotherapy	II
NCT03297606	Canadian Profiling and Targeted Agent Utilization Trial (CAPTUR): A Phase II Basket Trial	II
NCT04565275	A Multi-center Open-label, Phase I/II Clinical Trial to Evaluate the Safety, Tolerability, Pharmacokinetics, and Pharmacodynamics of ICP-192 in Patients With Advanced Solid Tumors and FGFR Gene Alterations.	I/II
No NCT ID	Phase I/II Study of TAS-117 In Combination With TAS-120 In Patients With Advanced Solid Tumors	I/II
No NCT ID	A Phase Ib Study to Assess the Safety, Tolerability, and Efficacy of TAS-120 (Futibatinib) in Combination with MK-3475 (Pembrolizumab) in Patients with Solid Tumors.	I
NCT04936295	A Phase II Study of the Efficacy and Tolerability of Fulvestrant Plus Anlotinib in HR(+)/HER2(-) Metastatic Breast Cancer Patients With FGFR Mutation	II
NCT04096417	A Phase II, Multicenter, Single-Arm Study of Pemigatinib in Patients With Metastatic or Unresectable Colorectal Cancer Harboring FGFR Alterations	II
NCT04077255	Anti-EGFR Antibody (GC-1118) in Combination With Weekly Paclitaxel as a Second-line Therapy for Gastric and Gastroesophageal Junction Adenocarcinomas With Amplifications	II
NCT04483505	Rogaratinib, Palbociclib and Fulvestrant in Advanced Hormone Receptor Positive, FGFR1/2/3-positive Breast Cancer: Phase I Clinical Trial Plus an Expansion Cohort	I
NCT03827850	A Phase II Trial to Evaluate Efficacy and Safety of Erdafitinib in Patients With Advanced Squamous Non Small Cell Lung Carcinoma (sqNSCLC) Harboring Fibroblast Growth Factor Receptor (FGFR) Genetic Alterations After Relapse of Standard Therapy.	II

Disclaimer: The data presented here is from a curated knowledgebase of publicly available information, but may not be exhaustive. The data version is 2021.11(003).

Clinical Trials Summary (continued)

FGFR3 amplification (continued)

NCT ID	Title	Phase
NCT02150967	A Phase II Multicenter, Single Arm Study of Oral BGJ398 in Adult Patients with Advanced or Metastatic Cholangiocarcinoma with FGFR2 Gene Fusions or Other FGFR Genetic Alterations who Failed or are Intolerant to Platinum-based Chemotherapy.	II
NCT04595747	Phase II Study of Rogaratinib (BAY 1163877) in the Treatment of Patients With Sarcoma Harboring Alterations in Fibroblast Growth Factor Receptor (FGFR) 1-4 and SDH-Deficient Gastrointestinal Stromal Tumor (GIST)	II
NCT04149691	A Phase I, Open-label, Multicentre, Dose Escalation Study to Assess Safety, Tolerability and Pharmacokinetics of Oral CPL304110, in Adult Subjects With Advanced Solid Malignancies	I
NCT04601857	A Phase II Study Evaluating Futibatinib (TAS 120) Plus Pembrolizumab in the Treatment of Advanced or Metastatic Urothelial Carcinoma	II
NCT03473743	A Phase Ib-II Study to Evaluate Safety, Efficacy, Pharmacokinetics, and Pharmacodynamics of Various Regimens of Erdafitinib in Subjects With Metastatic or Locally Advanced Urothelial Cancer	I/II
NCT02393248	A Phase I/II, Open-Label, Dose-Escalation, Safety and Tolerability Study of INCB054828 in Subjects With Advanced Malignancies - (FIGHT-101)	I/II
No NCT ID	Phase I Clinical Study of BPI-17509 In Patients With Advanced Solid Tumors	I
NCT04572295	An Open-label Phase Ib Study of E7090 Monotherapy and in Combination With Other Anticancer Agents in Subjects With ER+, HER2- Recurrent/Metastatic Breast Cancer	I
NCT04504331	A Phase IB Study of Infigratinib in Combination With Tamoxifen in Hormone Receptor Positive, HER2 Negative, FGFR Altered Advanced Breast Cancer	I

FGFR3 aberration

NCT ID	Title	Phase
NCT04591431	The Rome Trial From Histology to Target: the Road to Personalize Target Therapy and Immunotherapy	II
NCT03297606	Canadian Profiling and Targeted Agent Utilization Trial (CAPTUR): A Phase II Basket Trial	II
NCT04565275	A Multi-center Open-label, Phase I/II Clinical Trial to Evaluate the Safety, Tolerability, Pharmacokinetics, and Pharmacodynamics of ICP-192 in Patients With Advanced Solid Tumors and FGFR Gene Alterations.	I/II
No NCT ID	Phase I/II Study of TAS-117 In Combination With TAS-120 In Patients With Advanced Solid Tumors	I/II
No NCT ID	A Phase Ib Study to Assess the Safety, Tolerability, and Efficacy of TAS-120 (Futibatinib) in Combination with MK-3475 (Pembrolizumab) in Patients with Solid Tumors.	I
NCT04595747	Phase II Study of Rogaratinib (BAY 1163877) in the Treatment of Patients With Sarcoma Harboring Alterations in Fibroblast Growth Factor Receptor (FGFR) 1-4 and SDH-Deficient Gastrointestinal Stromal Tumor (GIST)	II
NCT04149691	A Phase I, Open-label, Multicentre, Dose Escalation Study to Assess Safety, Tolerability and Pharmacokinetics of Oral CPL304110, in Adult Subjects With Advanced Solid Malignancies	I
NCT04601857	A Phase II Study Evaluating Futibatinib (TAS 120) Plus Pembrolizumab in the Treatment of Advanced or Metastatic Urothelial Carcinoma	II
NCT03473743	A Phase Ib-II Study to Evaluate Safety, Efficacy, Pharmacokinetics, and Pharmacodynamics of Various Regimens of Erdafitinib in Subjects With Metastatic or Locally Advanced Urothelial Cancer	I/II

Clinical Trials Summary (continued)

FGFR3 aberration (continued)

NCT ID	Title	Phase
NCT02393248	A Phase I/II, Open-Label, Dose-Escalation, Safety and Tolerability Study of INCB054828 in Subjects With Advanced Malignancies - (FIGHT-101)	I/II
No NCT ID	Phase I Clinical Study of BPI-17509 In Patients With Advanced Solid Tumors	I
NCT04572295	An Open-label Phase Ib Study of E7090 Monotherapy and in Combination With Other Anticancer Agents in Subjects With ER+, HER2- Recurrent/Metastatic Breast Cancer	I
NCT04504331	A Phase IB Study of Infigratinib in Combination With Tamoxifen in Hormone Receptor Positive, HER2 Negative, FGFR Altered Advanced Breast Cancer	I

Sample Cancer Type: Unknown Primary Origin

Table of Contents | Page

Table of Contents	Page
Relevant Therapy Details	**3**
Clinical Trials	**18**
Relevant Therapy Summary	**222**
Clinical Trials Summary	**245**

Report Highlights

21 Relevant Biomarkers
1 Therapies Available
37 Clinical Trials

Relevant Biomarkers

Genomic Alteration	Relevant Therapies (In this cancer type)	Relevant Therapies (In other cancer type)	Clinical Trials
PIK3CA E542K mutation<br>**Prognostic significance:** None<br>**Diagnostic significance:** None	None	**alpelisib + hormone therapy** [1, 2]	37
PIK3CA E545K mutation<br>**Prognostic significance:** None<br>**Diagnostic significance:** None	None	**alpelisib + hormone therapy** [1, 2]	37
PIK3CA H1047L mutation<br>**Prognostic significance:** None<br>**Diagnostic significance:** None	None	**alpelisib + hormone therapy** [1, 2]	37
PIK3CA H1047R mutation<br>**Prognostic significance:** None<br>**Diagnostic significance:** None	None	**alpelisib + hormone therapy** [1, 2]	37
PIK3CA H1047Y mutation<br>**Prognostic significance:** None<br>**Diagnostic significance:** None	None	**alpelisib + hormone therapy** [1, 2]	36
PIK3CA E545A mutation<br>**Prognostic significance:** None<br>**Diagnostic significance:** None	None	**alpelisib + hormone therapy** [1, 2]	33
PIK3CA E545D [c.1635G>T]<br>**Prognostic significance:** None<br>**Diagnostic significance:** None	None	**alpelisib + hormone therapy** [1, 2]	33
PIK3CA E545G mutation<br>**Prognostic significance:** None<br>**Diagnostic significance:** None	None	**alpelisib + hormone therapy** [1, 2]	33
PIK3CA Q546E mutation<br>**Prognostic significance:** None<br>**Diagnostic significance:** None	None	**alpelisib + hormone therapy** [1, 2]	31
PIK3CA Q546R mutation<br>**Prognostic significance:** None<br>**Diagnostic significance:** None	None	**alpelisib + hormone therapy** [1, 2]	31

Public data sources included in relevant therapies: FDA1, NCCN, **EMA**2, ESMO
Public data sources included in prognostic and diagnostic significance: NCCN, ESMO

Disclaimer: The data presented here is from a curated knowledgebase of publicly available information, but may not be exhaustive. The data version is 2021.11(003).The content of this report has not been evaluated or approved by the FDA or other regulatory agencies.

Relevant Biomarkers (continued)

Genomic Alteration	Relevant Therapies (In this cancer type)	Relevant Therapies (In other cancer type)	Clinical Trials
PIK3CA C420R mutation<br>**Prognostic significance:** None<br>**Diagnostic significance:** None	None	**alpelisib + hormone therapy** [1, 2]	31
PIK3CA H1047 mutation<br>**Prognostic significance:** None<br>**Diagnostic significance:** None	None	alpelisib + hormone therapy	36
PIK3CA activating mutation<br>**Prognostic significance:** None<br>**Diagnostic significance:** None	None	alpelisib + hormone therapy	34
PIK3CA E545 mutation<br>**Prognostic significance:** None<br>**Diagnostic significance:** None	None	alpelisib + hormone therapy	33
PIK3CA E542 mutation<br>**Prognostic significance:** None<br>**Diagnostic significance:** None	None	alpelisib + hormone therapy	32
PIK3CA exon 20 mutation<br>**Prognostic significance:** None<br>**Diagnostic significance:** None	None	alpelisib + hormone therapy	31
PIK3CA exon 9 mutation<br>**Prognostic significance:** None<br>**Diagnostic significance:** None	None	alpelisib + hormone therapy	31
PIK3CA mutation<br>**Prognostic significance:** None<br>**Diagnostic significance:** None	None	None	31
PIK3CA mutation status<br>**Prognostic significance:** None<br>**Diagnostic significance:** None	None	None	14
PIK3CA amplification<br>**Prognostic significance:** None<br>**Diagnostic significance:** None	None	None	12
PIK3CA aberration<br>**Prognostic significance:** None<br>**Diagnostic significance:** None	None	None	10

Public data sources included in relevant therapies: FDA1, NCCN, **EMA**2, ESMO
Public data sources included in prognostic and diagnostic significance: NCCN, ESMO

Relevant Therapy Details

Current FDA Information

● In this cancer type ○ In other cancer type ◐ In this cancer type and other cancer types

FDA information is current as of 2021-10-13. For the most up-to-date information, search www.fda.gov.

PIK3CA E542K mutation

○ alpelisib + fulvestrant

Cancer type: Breast Cancer **Label as of:** 2021-07-20 **Variant class:** PIK3CA E542K mutation

Other criteria: ERBB2 negative, Hormone receptor positive

Indications and usage:
PIQRAY® is a kinase inhibitor indicated in combination with fulvestrant for the treatment of postmenopausal women, and men, with hormone receptor (HR)- positive, human epidermal growth factor receptor 2 (HER2)-negative, PIK3CA-mutated, advanced or metastatic breast cancer as detected by an FDA-approved test following progression on or after an endocrine-based regimen.

Reference:

https://www.accessdata.fda.gov/drugsatfda_docs/label/2021/212526Orig1s004lbl.pdf

PIK3CA E545K mutation

○ alpelisib + fulvestrant

Cancer type: Breast Cancer **Label as of:** 2021-07-20 **Variant class:** PIK3CA E545K mutation

Other criteria: ERBB2 negative, Hormone receptor positive

Indications and usage:
PIQRAY® is a kinase inhibitor indicated in combination with fulvestrant for the treatment of postmenopausal women, and men, with hormone receptor (HR)- positive, human epidermal growth factor receptor 2 (HER2)-negative, PIK3CA-mutated, advanced or metastatic breast cancer as detected by an FDA-approved test following progression on or after an endocrine-based regimen.

Reference:

https://www.accessdata.fda.gov/drugsatfda_docs/label/2021/212526Orig1s004lbl.pdf

PIK3CA H1047L mutation

○ alpelisib + fulvestrant

Cancer type: Breast Cancer **Label as of:** 2021-07-20 **Variant class:** PIK3CA H1047L mutation

Other criteria: ERBB2 negative, Hormone receptor positive

Indications and usage:
PIQRAY® is a kinase inhibitor indicated in combination with fulvestrant for the treatment of postmenopausal women, and men, with hormone receptor (HR)- positive, human epidermal growth factor receptor 2 (HER2)-negative, PIK3CA-mutated, advanced or metastatic breast cancer as detected by an FDA-approved test following progression on or after an endocrine-based regimen.

Reference:

https://www.accessdata.fda.gov/drugsatfda_docs/label/2021/212526Orig1s004lbl.pdf

Disclaimer: The data presented here is from a curated knowledgebase of publicly available information, but may not be exhaustive. The data version is 2021.11(003).

PIK3CA H1047R mutation

○ alpelisib + fulvestrant

Cancer type: Breast Cancer **Label as of:** 2021-07-20 **Variant class:** PIK3CA H1047R mutation

Other criteria: ERBB2 negative, Hormone receptor positive

Indications and usage:
PIQRAY® is a kinase inhibitor indicated in combination with fulvestrant for the treatment of postmenopausal women, and men, with hormone receptor (HR)- positive, human epidermal growth factor receptor 2 (HER2)-negative, PIK3CA-mutated, advanced or metastatic breast cancer as detected by an FDA-approved test following progression on or after an endocrine-based regimen.

Reference:

https://www.accessdata.fda.gov/drugsatfda_docs/label/2021/212526Orig1s004lbl.pdf

PIK3CA H1047Y mutation

○ alpelisib + fulvestrant

Cancer type: Breast Cancer **Label as of:** 2021-07-20 **Variant class:** PIK3CA H1047Y mutation

Other criteria: ERBB2 negative, Hormone receptor positive

Indications and usage:
PIQRAY® is a kinase inhibitor indicated in combination with fulvestrant for the treatment of postmenopausal women, and men, with hormone receptor (HR)- positive, human epidermal growth factor receptor 2 (HER2)-negative, PIK3CA-mutated, advanced or metastatic breast cancer as detected by an FDA-approved test following progression on or after an endocrine-based regimen.

Reference:

https://www.accessdata.fda.gov/drugsatfda_docs/label/2021/212526Orig1s004lbl.pdf

PIK3CA E545A mutation

○ alpelisib + fulvestrant

Cancer type: Breast Cancer **Label as of:** 2021-07-20 **Variant class:** PIK3CA E545A mutation

Other criteria: ERBB2 negative, Hormone receptor positive

Indications and usage:
PIQRAY® is a kinase inhibitor indicated in combination with fulvestrant for the treatment of postmenopausal women, and men, with hormone receptor (HR)- positive, human epidermal growth factor receptor 2 (HER2)-negative, PIK3CA-mutated, advanced or metastatic breast cancer as detected by an FDA-approved test following progression on or after an endocrine-based regimen.

Reference:

https://www.accessdata.fda.gov/drugsatfda_docs/label/2021/212526Orig1s004lbl.pdf

Disclaimer: The data presented here is from a curated knowledgebase of publicly available information, but may not be exhaustive. The data version is 2021.11(003).

PIK3CA E545D [c.1635G>T]

alpelisib + fulvestrant

Cancer type: Breast Cancer **Label as of:** 2021-07-20 **Variant class:** PIK3CA E545D [c.1635G>T]

Other criteria: ERBB2 negative, Hormone receptor positive

Indications and usage:
PIQRAY® is a kinase inhibitor indicated in combination with fulvestrant for the treatment of postmenopausal women, and men, with hormone receptor (HR)- positive, human epidermal growth factor receptor 2 (HER2)-negative, PIK3CA-mutated, advanced or metastatic breast cancer as detected by an FDA-approved test following progression on or after an endocrine-based regimen.

Reference:

https://www.accessdata.fda.gov/drugsatfda_docs/label/2021/212526Orig1s004lbl.pdf

PIK3CA E545G mutation

alpelisib + fulvestrant

Cancer type: Breast Cancer **Label as of:** 2021-07-20 **Variant class:** PIK3CA E545G mutation

Other criteria: ERBB2 negative, Hormone receptor positive

Indications and usage:
PIQRAY® is a kinase inhibitor indicated in combination with fulvestrant for the treatment of postmenopausal women, and men, with hormone receptor (HR)- positive, human epidermal growth factor receptor 2 (HER2)-negative, PIK3CA-mutated, advanced or metastatic breast cancer as detected by an FDA-approved test following progression on or after an endocrine-based regimen.

Reference:

https://www.accessdata.fda.gov/drugsatfda_docs/label/2021/212526Orig1s004lbl.pdf

PIK3CA Q546E mutation

alpelisib + fulvestrant

Cancer type: Breast Cancer **Label as of:** 2021-07-20 **Variant class:** PIK3CA Q546E mutation

Other criteria: ERBB2 negative, Hormone receptor positive

Indications and usage:
PIQRAY® is a kinase inhibitor indicated in combination with fulvestrant for the treatment of postmenopausal women, and men, with hormone receptor (HR)- positive, human epidermal growth factor receptor 2 (HER2)-negative, PIK3CA-mutated, advanced or metastatic breast cancer as detected by an FDA-approved test following progression on or after an endocrine-based regimen.

Reference:

https://www.accessdata.fda.gov/drugsatfda_docs/label/2021/212526Orig1s004lbl.pdf

Disclaimer: The data presented here is from a curated knowledgebase of publicly available information, but may not be exhaustive. The data version is 2021.11(003).

PIK3CA Q546R mutation

○ alpelisib + fulvestrant

Cancer type: Breast Cancer **Label as of:** 2021-07-20 **Variant class:** PIK3CA Q546R mutation

Other criteria: ERBB2 negative, Hormone receptor positive

Indications and usage:
PIQRAY® is a kinase inhibitor indicated in combination with fulvestrant for the treatment of postmenopausal women, and men, with hormone receptor (HR)- positive, human epidermal growth factor receptor 2 (HER2)-negative, PIK3CA-mutated, advanced or metastatic breast cancer as detected by an FDA-approved test following progression on or after an endocrine-based regimen.

Reference:

https://www.accessdata.fda.gov/drugsatfda_docs/label/2021/212526Orig1s004lbl.pdf

PIK3CA C420R mutation

○ alpelisib + fulvestrant

Cancer type: Breast Cancer **Label as of:** 2021-07-20 **Variant class:** PIK3CA C420R mutation

Other criteria: ERBB2 negative, Hormone receptor positive

Indications and usage:
PIQRAY® is a kinase inhibitor indicated in combination with fulvestrant for the treatment of postmenopausal women, and men, with hormone receptor (HR)- positive, human epidermal growth factor receptor 2 (HER2)-negative, PIK3CA-mutated, advanced or metastatic breast cancer as detected by an FDA-approved test following progression on or after an endocrine-based regimen.

Reference:

https://www.accessdata.fda.gov/drugsatfda_docs/label/2021/212526Orig1s004lbl.pdf

Disclaimer: The data presented here is from a curated knowledgebase of publicly available information, but may not be exhaustive. The data version is 2021.11(003).

Current NCCN Information

● In this cancer type ○ In other cancer type ◐ In this cancer type and other cancer types

NCCN information is current as of 2021-10-01. For the most up-to-date information, search www.nccn.org.
For NCCN International Adaptations & Translations, search www.nccn.org/global/international_adaptations.aspx.

PIK3CA E542K mutation

○ alpelisib + fulvestrant

Cancer type: Breast Cancer **Variant class:** PIK3CA activating mutation

Other criteria: ERBB2 negative, Hormone receptor positive

NCCN Recommendation category: 1

Population segment (Line of therapy):
- Stage IV; Invasive, Recurrent, Unresectable, Local, Regional (Second-line therapy); Preferred intervention

Reference: NCCN Guidelines® - NCCN-Breast Cancer [Version 8.2021]

PIK3CA E545K mutation

○ alpelisib + fulvestrant

Cancer type: Breast Cancer **Variant class:** PIK3CA activating mutation

Other criteria: ERBB2 negative, Hormone receptor positive

NCCN Recommendation category: 1

Population segment (Line of therapy):
- Stage IV; Invasive, Recurrent, Unresectable, Local, Regional (Second-line therapy); Preferred intervention

Reference: NCCN Guidelines® - NCCN-Breast Cancer [Version 8.2021]

PIK3CA H1047L mutation

○ alpelisib + fulvestrant

Cancer type: Breast Cancer **Variant class:** PIK3CA activating mutation

Other criteria: ERBB2 negative, Hormone receptor positive

NCCN Recommendation category: 1

Population segment (Line of therapy):
- Stage IV; Invasive, Recurrent, Unresectable, Local, Regional (Second-line therapy); Preferred intervention

Reference: NCCN Guidelines® - NCCN-Breast Cancer [Version 8.2021]

Disclaimer: The data presented here is from a curated knowledgebase of publicly available information, but may not be exhaustive. The data version is 2021.11(003).

PIK3CA H1047R mutation

○ alpelisib + fulvestrant

Cancer type: Breast Cancer **Variant class:** PIK3CA activating mutation

Other criteria: ERBB2 negative, Hormone receptor positive

NCCN Recommendation category: 1

Population segment (Line of therapy):

- Stage IV; Invasive, Recurrent, Unresectable, Local, Regional (Second-line therapy); Preferred intervention

Reference: NCCN Guidelines® - NCCN-Breast Cancer [Version 8.2021]

PIK3CA H1047Y mutation

○ alpelisib + fulvestrant

Cancer type: Breast Cancer **Variant class:** PIK3CA activating mutation

Other criteria: ERBB2 negative, Hormone receptor positive

NCCN Recommendation category: 1

Population segment (Line of therapy):

- Stage IV; Invasive, Recurrent, Unresectable, Local, Regional (Second-line therapy); Preferred intervention

Reference: NCCN Guidelines® - NCCN-Breast Cancer [Version 8.2021]

PIK3CA H1047 mutation

○ alpelisib + fulvestrant

Cancer type: Breast Cancer **Variant class:** PIK3CA activating mutation

Other criteria: ERBB2 negative, Hormone receptor positive

NCCN Recommendation category: 1

Population segment (Line of therapy):

- Stage IV; Invasive, Recurrent, Unresectable, Local, Regional (Second-line therapy); Preferred intervention

Reference: NCCN Guidelines® - NCCN-Breast Cancer [Version 8.2021]

Disclaimer: The data presented here is from a curated knowledgebase of publicly available information, but may not be exhaustive. The data version is 2021.11(003).

PIK3CA activating mutation

○ alpelisib + fulvestrant

Cancer type: Breast Cancer **Variant class:** PIK3CA activating mutation

Other criteria: ERBB2 negative, Hormone receptor positive

NCCN Recommendation category: 1

Population segment (Line of therapy):

- Stage IV; Invasive, Recurrent, Unresectable, Local, Regional (Second-line therapy); Preferred intervention

Reference: NCCN Guidelines® - NCCN-Breast Cancer [Version 8.2021]

Disclaimer: The data presented here is from a curated knowledgebase of publicly available information, but may not be exhaustive. The data version is 2021.11(003).

Current EMA Information

● In this cancer type ○ In other cancer type ◐ In this cancer type and other cancer types

EMA information is current as of 2021-10-13. For the most up-to-date information, search www.ema.europa.eu/ema.

PIK3CA E542K mutation

○ alpelisib + fulvestrant

Cancer type: Breast Cancer **Label as of:** 2021-10-11 **Variant class:** PIK3CA E542K mutation

Other criteria: ERBB2 negative, Hormone receptor positive

Reference:

https://www.ema.europa.eu/en/documents/product-information/piqray-epar-product-information_en.pdf

PIK3CA E545K mutation

○ alpelisib + fulvestrant

Cancer type: Breast Cancer **Label as of:** 2021-10-11 **Variant class:** PIK3CA E545K mutation

Other criteria: ERBB2 negative, Hormone receptor positive

Reference:

https://www.ema.europa.eu/en/documents/product-information/piqray-epar-product-information_en.pdf

PIK3CA H1047L mutation

○ alpelisib + fulvestrant

Cancer type: Breast Cancer **Label as of:** 2021-10-11 **Variant class:** PIK3CA H1047L mutation

Other criteria: ERBB2 negative, Hormone receptor positive

Reference:

https://www.ema.europa.eu/en/documents/product-information/piqray-epar-product-information_en.pdf

PIK3CA H1047R mutation

○ alpelisib + fulvestrant

Cancer type: Breast Cancer **Label as of:** 2021-10-11 **Variant class:** PIK3CA H1047R mutation

Other criteria: ERBB2 negative, Hormone receptor positive

Reference:

https://www.ema.europa.eu/en/documents/product-information/piqray-epar-product-information_en.pdf

Disclaimer: The data presented here is from a curated knowledgebase of publicly available information, but may not be exhaustive. The data version is 2021.11(003).

PIK3CA H1047Y mutation

alpelisib + fulvestrant

Cancer type: Breast Cancer **Label as of:** 2021-10-11 **Variant class:** PIK3CA H1047Y mutation

Other criteria: ERBB2 negative, Hormone receptor positive

Reference:

https://www.ema.europa.eu/en/documents/product-information/piqray-epar-product-information_en.pdf

PIK3CA E545A mutation

alpelisib + fulvestrant

Cancer type: Breast Cancer **Label as of:** 2021-10-11 **Variant class:** PIK3CA E545A mutation

Other criteria: ERBB2 negative, Hormone receptor positive

Reference:

https://www.ema.europa.eu/en/documents/product-information/piqray-epar-product-information_en.pdf

PIK3CA E545D [c.1635G>T]

alpelisib + fulvestrant

Cancer type: Breast Cancer **Label as of:** 2021-10-11 **Variant class:** PIK3CA E545D [c.1635G>T]

Other criteria: ERBB2 negative, Hormone receptor positive

Reference:

https://www.ema.europa.eu/en/documents/product-information/piqray-epar-product-information_en.pdf

PIK3CA E545G mutation

alpelisib + fulvestrant

Cancer type: Breast Cancer **Label as of:** 2021-10-11 **Variant class:** PIK3CA E545G mutation

Other criteria: ERBB2 negative, Hormone receptor positive

Reference:

https://www.ema.europa.eu/en/documents/product-information/piqray-epar-product-information_en.pdf

Disclaimer: The data presented here is from a curated knowledgebase of publicly available information, but may not be exhaustive. The data version is 2021.11(003).

PIK3CA Q546E mutation

alpelisib + fulvestrant

Cancer type: Breast Cancer **Label as of:** 2021-10-11 **Variant class:** PIK3CA Q546E mutation

Other criteria: ERBB2 negative, Hormone receptor positive

Reference:

https://www.ema.europa.eu/en/documents/product-information/piqray-epar-product-information_en.pdf

PIK3CA Q546R mutation

alpelisib + fulvestrant

Cancer type: Breast Cancer **Label as of:** 2021-10-11 **Variant class:** PIK3CA Q546R mutation

Other criteria: ERBB2 negative, Hormone receptor positive

Reference:

https://www.ema.europa.eu/en/documents/product-information/piqray-epar-product-information_en.pdf

PIK3CA C420R mutation

alpelisib + fulvestrant

Cancer type: Breast Cancer **Label as of:** 2021-10-11 **Variant class:** PIK3CA C420R mutation

Other criteria: ERBB2 negative, Hormone receptor positive

Reference:

https://www.ema.europa.eu/en/documents/product-information/piqray-epar-product-information_en.pdf

Disclaimer: The data presented here is from a curated knowledgebase of publicly available information, but may not be exhaustive. The data version is 2021.11(003).

Current ESMO Information

● In this cancer type ○ In other cancer type ◐ In this cancer type and other cancer types

ESMO information is current as of 2021-10-01. For the most up-to-date information, search www.esmo.org.

PIK3CA E542K mutation

○ alpelisib + fulvestrant

Cancer type: Breast Cancer **Variant class:** PIK3CA exon 9 mutation

Other criteria: ERBB2 negative, ER positive

ESMO Level of Evidence/Grade of Recommendation: I / B

Population segment (Line of therapy):
- Luminal A, Luminal B; Advanced (Line of therapy not specified); ESMO-MCBS v1.1 score: 3

Reference: ESMO Clinical Practice Guidelines - ESMO-ESO-ESMO Advanced Breast Cancer [Annals of Oncology (2020), doi: https://doi.org/10.1016/j.annonc.2020.09.010 (ABC 5)]

PIK3CA E545K mutation

○ alpelisib + fulvestrant

Cancer type: Breast Cancer **Variant class:** PIK3CA exon 9 mutation

Other criteria: ERBB2 negative, ER positive

ESMO Level of Evidence/Grade of Recommendation: I / B

Population segment (Line of therapy):
- Luminal A, Luminal B; Advanced (Line of therapy not specified); ESMO-MCBS v1.1 score: 3

Reference: ESMO Clinical Practice Guidelines - ESMO-ESO-ESMO Advanced Breast Cancer [Annals of Oncology (2020), doi: https://doi.org/10.1016/j.annonc.2020.09.010 (ABC 5)]

PIK3CA H1047L mutation

○ alpelisib + fulvestrant

Cancer type: Breast Cancer **Variant class:** PIK3CA exon 20 mutation

Other criteria: ERBB2 negative, ER positive

ESMO Level of Evidence/Grade of Recommendation: I / B

Population segment (Line of therapy):
- Luminal A, Luminal B; Advanced (Line of therapy not specified); ESMO-MCBS v1.1 score: 3

Reference: ESMO Clinical Practice Guidelines - ESMO-ESO-ESMO Advanced Breast Cancer [Annals of Oncology (2020), doi: https://doi.org/10.1016/j.annonc.2020.09.010 (ABC 5)]

Disclaimer: The data presented here is from a curated knowledgebase of publicly available information, but may not be exhaustive. The data version is 2021.11(003).

PIK3CA H1047R mutation

○ alpelisib + fulvestrant

Cancer type: Breast Cancer **Variant class:** PIK3CA exon 20 mutation

Other criteria: ERBB2 negative, ER positive

ESMO Level of Evidence/Grade of Recommendation: I / B

Population segment (Line of therapy):
- Luminal A, Luminal B; Advanced (Line of therapy not specified); ESMO-MCBS v1.1 score: 3

Reference: ESMO Clinical Practice Guidelines - ESMO-ESO-ESMO Advanced Breast Cancer [Annals of Oncology (2020), doi: https://doi.org/10.1016/j.annonc.2020.09.010 (ABC 5)]

PIK3CA H1047Y mutation

○ alpelisib + fulvestrant

Cancer type: Breast Cancer **Variant class:** PIK3CA exon 20 mutation

Other criteria: ERBB2 negative, ER positive

ESMO Level of Evidence/Grade of Recommendation: I / B

Population segment (Line of therapy):
- Luminal A, Luminal B; Advanced (Line of therapy not specified); ESMO-MCBS v1.1 score: 3

Reference: ESMO Clinical Practice Guidelines - ESMO-ESO-ESMO Advanced Breast Cancer [Annals of Oncology (2020), doi: https://doi.org/10.1016/j.annonc.2020.09.010 (ABC 5)]

PIK3CA E545A mutation

○ alpelisib + fulvestrant

Cancer type: Breast Cancer **Variant class:** PIK3CA exon 9 mutation

Other criteria: ERBB2 negative, ER positive

ESMO Level of Evidence/Grade of Recommendation: I / B

Population segment (Line of therapy):
- Luminal A, Luminal B; Advanced (Line of therapy not specified); ESMO-MCBS v1.1 score: 3

Reference: ESMO Clinical Practice Guidelines - ESMO-ESO-ESMO Advanced Breast Cancer [Annals of Oncology (2020), doi: https://doi.org/10.1016/j.annonc.2020.09.010 (ABC 5)]

Disclaimer: The data presented here is from a curated knowledgebase of publicly available information, but may not be exhaustive. The data version is 2021.11(003).

PIK3CA E545D [c.1635G>T]

alpelisib + fulvestrant

Cancer type: Breast Cancer **Variant class:** PIK3CA exon 9 mutation

Other criteria: ERBB2 negative, ER positive

ESMO Level of Evidence/Grade of Recommendation: I / B

Population segment (Line of therapy):
- Luminal A, Luminal B; Advanced (Line of therapy not specified); ESMO-MCBS v1.1 score: 3

Reference: ESMO Clinical Practice Guidelines - ESMO-ESO-ESMO Advanced Breast Cancer [Annals of Oncology (2020), doi: https://doi.org/10.1016/j.annonc.2020.09.010 (ABC 5)]

PIK3CA E545G mutation

alpelisib + fulvestrant

Cancer type: Breast Cancer **Variant class:** PIK3CA exon 9 mutation

Other criteria: ERBB2 negative, ER positive

ESMO Level of Evidence/Grade of Recommendation: I / B

Population segment (Line of therapy):
- Luminal A, Luminal B; Advanced (Line of therapy not specified); ESMO-MCBS v1.1 score: 3

Reference: ESMO Clinical Practice Guidelines - ESMO-ESO-ESMO Advanced Breast Cancer [Annals of Oncology (2020), doi: https://doi.org/10.1016/j.annonc.2020.09.010 (ABC 5)]

PIK3CA Q546E mutation

alpelisib + fulvestrant

Cancer type: Breast Cancer **Variant class:** PIK3CA exon 9 mutation

Other criteria: ERBB2 negative, ER positive

ESMO Level of Evidence/Grade of Recommendation: I / B

Population segment (Line of therapy):
- Luminal A, Luminal B; Advanced (Line of therapy not specified); ESMO-MCBS v1.1 score: 3

Reference: ESMO Clinical Practice Guidelines - ESMO-ESO-ESMO Advanced Breast Cancer [Annals of Oncology (2020), doi: https://doi.org/10.1016/j.annonc.2020.09.010 (ABC 5)]

PIK3CA Q546R mutation

alpelisib + fulvestrant

Cancer type: Breast Cancer **Variant class:** PIK3CA exon 9 mutation

Other criteria: ERBB2 negative, ER positive

ESMO Level of Evidence/Grade of Recommendation: I / B

Population segment (Line of therapy):
- Luminal A, Luminal B; Advanced (Line of therapy not specified); ESMO-MCBS v1.1 score: 3

Reference: ESMO Clinical Practice Guidelines - ESMO-ESO-ESMO Advanced Breast Cancer [Annals of Oncology (2020), doi: https://doi.org/10.1016/j.annonc.2020.09.010 (ABC 5)]

PIK3CA H1047 mutation

alpelisib + fulvestrant

Cancer type: Breast Cancer **Variant class:** PIK3CA exon 20 mutation

Other criteria: ERBB2 negative, ER positive

ESMO Level of Evidence/Grade of Recommendation: I / B

Population segment (Line of therapy):
- Luminal A, Luminal B; Advanced (Line of therapy not specified); ESMO-MCBS v1.1 score: 3

Reference: ESMO Clinical Practice Guidelines - ESMO-ESO-ESMO Advanced Breast Cancer [Annals of Oncology (2020), doi: https://doi.org/10.1016/j.annonc.2020.09.010 (ABC 5)]

PIK3CA E545 mutation

alpelisib + fulvestrant

Cancer type: Breast Cancer **Variant class:** PIK3CA exon 9 mutation

Other criteria: ERBB2 negative, ER positive

ESMO Level of Evidence/Grade of Recommendation: I / B

Population segment (Line of therapy):
- Luminal A, Luminal B; Advanced (Line of therapy not specified); ESMO-MCBS v1.1 score: 3

Reference: ESMO Clinical Practice Guidelines - ESMO-ESO-ESMO Advanced Breast Cancer [Annals of Oncology (2020), doi: https://doi.org/10.1016/j.annonc.2020.09.010 (ABC 5)]

Disclaimer: The data presented here is from a curated knowledgebase of publicly available information, but may not be exhaustive. The data version is 2021.11(003).

PIK3CA E542 mutation

○ alpelisib + fulvestrant

Cancer type: Breast Cancer **Variant class:** PIK3CA exon 9 mutation

Other criteria: ERBB2 negative, ER positive

ESMO Level of Evidence/Grade of Recommendation: I / B

Population segment (Line of therapy):

- Luminal A, Luminal B; Advanced (Line of therapy not specified); ESMO-MCBS v1.1 score: 3

Reference: ESMO Clinical Practice Guidelines - ESMO-ESO-ESMO Advanced Breast Cancer [Annals of Oncology (2020), doi: https://doi.org/10.1016/j.annonc.2020.09.010 (ABC 5)]

PIK3CA exon 20 mutation

○ alpelisib + fulvestrant

Cancer type: Breast Cancer **Variant class:** PIK3CA exon 20 mutation

Other criteria: ERBB2 negative, ER positive

ESMO Level of Evidence/Grade of Recommendation: I / B

Population segment (Line of therapy):

- Luminal A, Luminal B; Advanced (Line of therapy not specified); ESMO-MCBS v1.1 score: 3

Reference: ESMO Clinical Practice Guidelines - ESMO-ESO-ESMO Advanced Breast Cancer [Annals of Oncology (2020), doi: https://doi.org/10.1016/j.annonc.2020.09.010 (ABC 5)]

PIK3CA exon 9 mutation

○ alpelisib + fulvestrant

Cancer type: Breast Cancer **Variant class:** PIK3CA exon 9 mutation

Other criteria: ERBB2 negative, ER positive

ESMO Level of Evidence/Grade of Recommendation: I / B

Population segment (Line of therapy):

- Luminal A, Luminal B; Advanced (Line of therapy not specified); ESMO-MCBS v1.1 score: 3

Reference: ESMO Clinical Practice Guidelines - ESMO-ESO-ESMO Advanced Breast Cancer [Annals of Oncology (2020), doi: https://doi.org/10.1016/j.annonc.2020.09.010 (ABC 5)]

Disclaimer: The data presented here is from a curated knowledgebase of publicly available information, but may not be exhaustive. The data version is 2021.11(003).

Current Clinical Trials Information

Clinical Trials information is current as of 2021-10-01. For the most up-to-date information regarding a particular trial, search www.clinicaltrials.gov by NCT ID or search local clinical trials authority website by local identifier listed in 'Other identifiers'.

PIK3CA E542K mutation

NCT04586335
Open Label, Phase Ib Study to Evaluate the Safety, Tolerability, Pharmacokinetics and Clinical Activity of CYH33, an Oral a-specific PI3K Inhibitor in Combination With Olaparib, an Oral PARP Inhibitor in Patients With Advanced Solid Tumors.

Cancer type: Breast Cancer, Endometrial Carcinoma, Ovarian Cancer, Prostate Cancer, Unspecified Solid Tumor

Variant class: PIK3CA E542K mutation

Other identifiers: CTR20202339, CYH33-G102

Population segments: BRCA, Second line, Stage III, Stage IV

Phase: I

Therapies: HH-CYH33, olaparib

Locations: Australia, United States

US State: TX

Contact: Dr. Jason Sudia [908-380-1329; jason.sudia@haihepharma.com]

NCT04317105
A Phase I/II Biomarker Driven Combination Trial of Copanlisib and Immune Checkpoint Inhibitors in Patients With Advanced Solid Tumors

Cancer type: Unspecified Solid Tumor

Variant class: PIK3CA E542 mutation

Other identifiers: (BACON), 10221, 2019-1046, NCI10221, NCI-2020-01917

Population segments: Second line, Stage III, Stage IV

Phase: I/II

Therapies: copanlisib, nivolumab, ipilimumab

Location: United States

US States: MA, TX

Contact: Multiple contacts: See www.clinicaltrials.gov for complete list of contacts.

NCT04632992
MyTACTIC: An Open-Label Phase II Study Evaluating Targeted Therapies in Patients Who Have Advanced Solid Tumors With Genomic Alterations or Protein Expression Patterns Predictive of Response

Cancer type: Unspecified Solid Tumor

Variant class: PIK3CA activating mutation

Other identifiers: ML42439, MyTACTIC

Population segments: ALK, EGFR, HER2 negative, HER2 positive, Line of therapy N/A, Stage III, Stage IV

Phase: II

Therapies: inavolisib, atezolizumab + ipatasertib

Location: United States

US States: AK, AZ, CA, CT, FL, LA, MD, MI, MO, MT, NE, NJ, NY, OH, OR, TN, TX, WA

Contact: Reference Study ID Number: ML42439 [888-662-6728; global-roche-genentech-trials@gene.com]

Disclaimer: The data presented here is from a curated knowledgebase of publicly available information, but may not be exhaustive. The data version is 2021.11(003).

PIK3CA E542K mutation (continued)

NCT03673787
Ice-CAP: A Phase I Trial of Ipatasertib in Combination With Atezolizumab in Patients With Advanced Solid Tumours With PI3K Pathway Hyperactivation.

Cancer type: Unspecified Solid Tumor

Variant class: PIK3CA activating mutation

Other identifiers: CCR4720, EudraCT Number: 2017-003005-18, IceCAP, Ice-CAP, IRAS ID 233461

Population segments: Hormone refractory, Second line, Stage III, Stage IV

Phase: I/II

Therapies: ipatasertib, atezolizumab

Location: United Kingdom

NCT03239015
Efficacy and Safety of Targeted Precision Therapy in Refractory Tumor With Druggable Molecular Event

Cancer type: Unspecified Solid Tumor

Variant class: PIK3CA mutation

Other identifier: HETIAN64

Population segments: (N/A), Second line

Phase: II

Therapy: everolimus

Location: China

NCT04589845
Tumor-Agnostic Precision Immunooncology and Somatic Targeting Rational for You (TAPISTRY) Phase II Platform Trial

Cancer type: Unspecified Solid Tumor

Variant class: PIK3CA mutation

Other identifiers: BO41932, EudraCT Number: 2020-001847-16, TAPISTRY

Population segments: ALK, First line, HER2 positive, Second line, Stage III, Stage IV

Phase: II

Therapy: inavolisib

Locations: Australia, Belgium, Brazil, Canada, China, Germany, Israel, Italy, New Zealand, Poland, Portugal, Republic of Korea, Spain, Taiwan, United States

US States: AL, AZ, CA, DE, FL, GA, ID, IL, IN, MD, ME, MI, MN, MO, MT, NJ, NM, NV, NY, OH, PA, SC, TN, TX, WA, WI

Contact: Reference Study ID Number: BO41932 [888-662-6728; Global-Roche-Genentech-Trials@gene.com]

NCT03842228
A Phase Ib Biomarker-Driven Combination Trial of Copanlisib, Olaparib, and Durvalumab (MEDI4736) in Patients With Advanced Solid Tumors

Cancer type: Unspecified Solid Tumor

Variant class: PIK3CA mutation

Other identifiers: 10217, 19-628, NCI10217, NCI-2019-00601

Population segments: Line of therapy N/A, Stage III, Stage IV

Phase: I

Therapies: copanlisib, olaparib, durvalumab

Location: United States

US States: MA, TX

Contact: Multiple contacts: See www.clinicaltrials.gov for complete list of contacts.

Disclaimer: The data presented here is from a curated knowledgebase of publicly available information, but may not be exhaustive. The data version is 2021.11(003).

PIK3CA E542K mutation (continued)

NCT03006172

A Phase I, Open-Label, Dose-Escalation Study Evaluating the Safety, Tolerability, and Pharmacokinetics of Inavolisib as a Single Agent in Patients With Locally Advanced or Metastatic PIK3CA-Mutant Solid Tumors and in Combination With Endocrine and Targeted Therapies in Patients With Locally Advanced or Metastatic PIK3CA-Mutant Breast Cancer

Cancer type: Breast Cancer, Unspecified Solid Tumor

Variant class: PIK3CA mutation

Other inclusion criteria: ERBB2 negative, Hormone receptor positive

Other identifiers: 16-1556, 16-521, EudraCT Number: 2016-003022-17, GO39374, NCI-2017-00262

Population segments: Estrogen receptor positive, HER2 negative, Progesterone receptor positive, Second line, Stage III, Stage IV

Phase: I

Therapy: inavolisib

Locations: Canada, France, Spain, United Kingdom, United States

US States: MA, NY, TN

Contact: Reference Study ID Number: GO39374 [888-662-6728; global-roche-genentech-trials@gene.com]

No NCT ID - see other identifier(s)

A Phase Ib, open-label, dose-escalation and dose-expansion study evaluating the safety, tolerability, pharmacokinetics, and preliminary antitumor activity of inavolisib in combination with paclitaxel and with or without targeted therapies in patients with locally advanced or metastatic solid tumors

Cancer type: Unspecified Solid Tumor

Variant class: PIK3CA mutation

Other identifiers: CO42800, EudraCT number: 2020-005057-24, ISRCTN45319897

Population segments: Estrogen receptor positive, First line, HER2 positive, Progesterone receptor positive, Second line, Stage I, Stage II, Stage III, Stage IV

Phase: I

Therapies: inavolisib, chemotherapy, trastuzumab, pertuzumab

Locations: Canada, Denmark, France, Republic of Korea, Spain, United States

US State:

NCT03065062

Phase I Study of the CDK4/6 Inhibitor Palbociclib (PD-0332991) in Combination With the PI3K/mTOR Inhibitor Gedatolisib (PF-05212384) for Patients With Advanced Squamous Cell Lung, Pancreatic, Head & Neck and Other Solid Tumors

Cancer type: Endometrial Carcinoma, Head and Neck Cancer, Non-Small Cell Lung Cancer, Pancreatic Cancer, Unspecified Solid Tumor

Variant class: PIK3CA mutation

Other identifiers: 16-499, NCI-2017-00434

Population segments: Second line, Squamous Cell, Stage III, Stage IV

Phase: I

Therapies: palbociclib, gedatolisib

Location: United States

US State: MA

Contact: Dr. Geoffrey Shapiro [617-632-4942; Geoffrey_Shapiro@dfci.harvard.edu]

PIK3CA E542K mutation (continued)

NCT04192981
A Phase I Study With Expansion Cohort of Concurrent GDC-0084 With Radiation Therapy for Patients With Solid Tumor Brain Metastases or Leptomeningeal Metastases Harboring PI3K Pathway Mutations

Cancer type: Unspecified Solid Tumor

Variant class: PIK3CA mutation

Other identifiers: 19-359, NCI-2019-08530

Population segments: (N/A), CNS mets, Line of therapy N/A

Phase: I

Therapies: paxalisib, radiation therapy

Location: United States

US States: NJ, NY

Contact: Dr. T. Jonathan Yang [212-639-8157; yangt@mskcc.org]

NCT03297606
Canadian Profiling and Targeted Agent Utilization Trial (CAPTUR): A Phase II Basket Trial

Cancer type: Unspecified Solid Tumor

Variant class: PIK3CA aberration

Other identifiers: CA209-9DL, CAPTUR, ESR-17-12831, ML39800, PM1, WI233446

Population segments: Aggressive, Diffuse large B-cell lymphoma (DLBCL), Extranodal marginal zone B-cell lymphoma (MALT), First line, Follicular lymphoma (FL), Indolent, Lymphoblastic lymphoma (LBL), Mantle cell lymphoma (MCL), Other subtype, Second line, Stage III, Stage IV, Waldenstrom's macroglobulinemia (WM)

Phase: II

Therapy: temsirolimus

Location: Canada

No NCT ID - see other identifier(s)
Phase I/II Study of TAS-117 In Combination With TAS-120 In Patients With Advanced Solid Tumors

Cancer type: Unspecified Solid Tumor

Variant class: PIK3CA aberration

Other identifier: JapicCTI-194864

Population segments: FGFR, First line, Squamous Cell, Stage III, Stage IV

Phase: I/II

Therapies: TAS-117, futibatinib

Location: Japan

NCT04591431
The Rome Trial From Histology to Target: the Road to Personalize Target Therapy and Immunotherapy

Cancer type: Biliary Tract Carcinoma, Breast Cancer, Colorectal Cancer, Esophageal Cancer, Gastric Cancer, Gastroesophageal Junction Adenocarcinoma, Hepatocellular Carcinoma, Liver Cancer, Non-Small Cell Lung Cancer, Pancreatic Cancer, Unspecified Solid Tumor

Variant class: PI3K aberration

Other identifiers: EudraCT Number: 2018-002190-21, MAR-BAS-18-005, ROME

Population segments: Second line, Stage IV

Phase: II

Therapy: ipatasertib

Location: Italy

Disclaimer: The data presented here is from a curated knowledgebase of publicly available information, but may not be exhaustive. The data version is 2021.11(003).

PIK3CA E542K mutation (continued)

NCT03155620
NCI-COG Pediatric MATCH (Molecular Analysis for Therapy Choice) Screening Protocol

Cancer type: Unspecified Solid Tumor

Variant class: PI3K/AKT/MTOR mutation

Other identifiers: 17-729, 2017-0625, 20170841, APEC1621SC, COGAPEC1621, COGAPEC1621SC, N 55017 SC, NCI-2017-01251, NCI-COG Pediatric MATCH, Pediatric MATCH

Population segments: ALK, Aggressive, BRAF, FGFR, HRAS, Indolent, KRAS, NRAS, Pediatric or Adolescent, Second line, Stage III, Stage IV

Phase: II

Therapy: samotolisib

Locations: Puerto Rico, United States

US States: AK, AL, AR, AZ, CA, CO, CT, DC, DE, FL, GA, HI, IA, ID, IL, IN, KY, LA, MA, MD, ME, MI, MN, MO, MS, NC, NE, NH, NJ, NV, NY, OH, OK, OR, PA, RI, SC, SD, TN, TX, UT, VA, VT, WA, WI

Contact: Multiple contacts: See www.clinicaltrials.gov for complete list of contacts.

NCT03213678
NCI-COG Pediatric MATCH (Molecular Analysis for Therapy Choice)- Phase II Subprotocol of LY3023414 in Patients With Solid Tumors

Cancer type: Unspecified Solid Tumor

Variant class: PI3K/AKT/MTOR mutation

Other identifiers: 17-742, APEC 1621 D, APEC 1621-D, APEC1621 D, APEC1621D, NCI-2017-01249, NCI-COG Pediatric MATCH, OCR16978, Pediatric MATCH

Population segments: Aggressive, Indolent, Pediatric or Adolescent, Second line, Stage III, Stage IV

Phase: II

Therapy: samotolisib

Locations: Puerto Rico, United States

US States: AK, AL, AR, AZ, CA, CO, CT, DC, DE, FL, GA, IA, ID, IL, IN, KY, LA, MA, MD, ME, MI, MN, MO, MS, NC, ND, NE, NJ, NY, OH, OK, OR, PA, SC, SD, TN, TX, UT, VA, VT, WA, WI

Contact: Multiple contacts: See www.clinicaltrials.gov for complete list of contacts.

No NCT ID - see other identifier(s)
BCT 1901 (CAPTURE): A Phase II Randomised Study To Evaluate Alpelisib Plus Fulvestrant Versus Capecitabine In Oestrogen Receptor Positive, HER2-Negative Advanced Breast Cancer Patients With PIK3CA Mutant Circulating DNA.

Cancer type: Breast Cancer

Variant class: PIK3CA E542K mutation

Other inclusion criteria: ERBB2 negative, ER positive

Other identifiers: ACTRN12619001117101, BCT 1901, BCT 1901 (CAPTURE), CAPTURE

Population segments: Estrogen receptor positive, HER2 negative, Line of therapy N/A, Stage III, Stage IV

Phase: II

Therapies: alpelisib, hormone therapy

Location: Australia

PIK3CA E542K mutation (continued)

NCT04216472
A Phase-II, Two-Cohort Trial of Neoadjuvant Nab-Paclitaxel and Alpelisib in Anthracycline Refractory Triple Negative Breast Cancer With PIK3CA or PTEN Alterations

Cancer type: Triple Negative Breast Cancer

Variant class: PIK3CA activating mutation

Other identifiers: 2019-0752, NCI-2019-08495

Population segments: HER2 negative, Neoadjuvant, Stage I, Stage II, Stage III, Triple receptor negative

Phase: II

Therapies: alpelisib, chemotherapy

Location: United States

US State: TX

Contact: Senthilkumar Damodaran [713-792-2817; sdamodaran@mdanderson.org]

NCT05025735
Alpelisib, Fulvestrant and Dapagliflozin for the Treatment of HR+, HER2 -, PIK3CA Mutant Metastatic Breast Cancer

Cancer type: Breast Cancer

Variant class: PIK3CA activating mutation

Other inclusion criteria: ERBB2 negative, Hormone receptor positive

Other identifier: CBYL719A0US03T

Population segments: Estrogen receptor positive, HER2 negative, Progesterone receptor positive, Second line, Stage III, Stage IV

Phase: II

Therapies: alpelisib, hormone therapy, dapagliflozin

Location: United States

US State: MO

Contact: Kelley Aldrich [816-932-2677; slci1research@saint-lukes.org]

NCT04251533
A Phase III, Multicenter, Randomized, Double-blind, Placebo-controlled Study to Assess the Efficacy and Safety of Alpelisib (BYL719) in Combination With Nab-paclitaxel in Patients With Advanced Triple Negative Breast Cancer With Either Phosphoinositide-3-kinase Catalytic Subunit Alpha (PIK3CA) Mutation or Phosphatase and Tensin Homolog Protein (PTEN) Loss Without PIK3CA Mutation

Cancer type: Triple Negative Breast Cancer

Variant class: PIK3CA mutation

Other identifiers: CBYL719H12301, CBYL719H12301 53027, CTR20201097, CTRI/2020/09/027887, DOH-27-012021-7110, EPIK-B3, EudraCT Number: 2019-002637-11, NCI-2020-02353, NMRR-20-236-53027

Population segments: HER2 negative, Second line, Stage III, Stage IV, Triple receptor negative

Phase: III

Therapies: alpelisib, chemotherapy

Locations: Australia, Austria, Brazil, Bulgaria, China, Colombia, Croatia, France, Germany, Hungary, India, Israel, Italy, Malaysia, Mexico, Norway, Peru, Poland, Republic of Korea, Russian Federation, Slovakia, Slovenia, Spain, Switzerland, Taiwan, Turkey, United Kingdom, United States

US States: CA, FL, IL, IN, KS, LA, MD, MN, MO, NY, OH, TN, TX, VA, WA

Contact: Novartis Pharmaceuticals [888-669-6682; novartis.email@novartis.com]

Disclaimer: The data presented here is from a curated knowledgebase of publicly available information, but may not be exhaustive. The data version is 2021.11(003).

PIK3CA E542K mutation (continued)

NCT04191499
[truncated: 677,916 more chars]
