# Supplementary material for: Potential Targeted Therapies in Ovarian Cancer
Source: Pharmaceuticals (Basel). 2022 Oct 26;15(11):1324. doi: 10.3390/ph15111324 (PMC9697427; doi:10.3390/ph15111324)

Table S1. Proportion of classified variant for 128 HGSC patients

Clinvar Classification	Proportion
Benign	40 (3%)
Benign / Likely Benign	67 (5%)
Likely Benign	25 (2%)
VUS	991 (73%)
Likely Pathogenic	26 (2%)
Likely pathogenic / Pathogenic	19 (1%)
Pathogenic	181 (13%)

**HGSC – High grade serous ovariancancer; VUS – variants of uncertain significance.*

Table S2. Pathogenic or likely pathogenic mutations per patient (N = 128)

	Number of patients (%)	Number of patients with druggable targets (%)
None	10 (8%)	0
1 mutation	64 (50%)	1 (1%)
2 mutations	35 (27%)	18 (14%)
3 mutations	12 (9%)	4 (3%)
4 mutations	3 (3%)	1 (1%)
≥ 5 mutations	3 (3%)	3 (2%)

Table S3. All pathogenic and likely pathogenic mutations

Table S4. Clinical trials evaluating PARP inhibitors in platinum-resistant patients

PARP inhibitor	Trial	Analysis population	No. of patients	Results
Olaparib	Fong et al., 2010 (24)	*BRCA1/2* mutated ovarian cancer	50<br>- 13 platinum-sensitive<br>- 24 platinum-resistant<br>- 13 platinum-refractory	RECIST response<br>- 6 (46.2%)<br>- 8 (33.5%)<br>- 0 (0%)
Olaparib	Kaufmann et al., 2015 (25)	PROC with germline and somatic *BRCA* mutation	193 platinum-resistant	Tumor response rate 31,1 (95% CI, 24,6-38,1)<br>Median PFS 7 months<br>Median OS 16,6 months
Niraparib	QUADRA, 2018 (26)	Relapsed ovarian cancer with PARP-naïve germline or somatic *BRCA* mutation	55<br>- 18 platinum-sensitive<br>- 21 platinum-resistant<br>- 16 platinum-refractory	 ORR 39%<br>ORR 33%<br>ORR 19%
Olaparib	BAROCCO, 2019 (27)	High-grade PROC of whom 86% (109/123) are *BRCAwt*	123 platinum-resistant	Median PFS<br>- Paclitaxel arm 3,1 months<br>- Olaparib-cediranib arm 5,7 months<br>- Cediranib arm 3,8 months<br>HR for PFS in paclitaxel vs. olaparib-cediranib arm was 0,76 (90% CI: 0,49-1,17).<br><br>Median PFS in the *gBRCAwt* patients (n=109)<br>- Paclitaxel arm 2,1 months<br>- Olaparib-cediranib arm 5,8 months<br>- Cediranib arm 3,8 months<br>HR for PFS in the paclitaxel vs. olaparib-cediranib arm was 0,63 (95% CI: 0,36-1,10).
Rucaparib	ARIEL4, 2021 (28)	*BRCA1/2* mutated high grade recurrent EOC.	345<br>- 176 platinum-resistant	Median PFS<br>- Rucaparib arm 7,4 months (95% CI, 7,3-9,1)<br>- Chemotherapy arm 5,7 months (95% CI, 5,5-7,3)<br>HR 0,64 (95% CI, 0,49-0,84)
Olaparib	NRG-GY005 (NCT02502266)	Recurrent platinum-resistant or -refractory HGSC or germline *BRCA* mutant ovarian cancer	680	Estimated study completion June 2023.
Olaparib	OCTOVA (NCT03117933)	*BRCA1/2* mutated PROC	132	Estimated study completion Nov 2021. Results pending.

Figure S1. Kaplan-Meier curve for overall survival in patient groups with *BRCA1/2* mutation (upper curve) compared with the BRCAwt patients (lower curve).

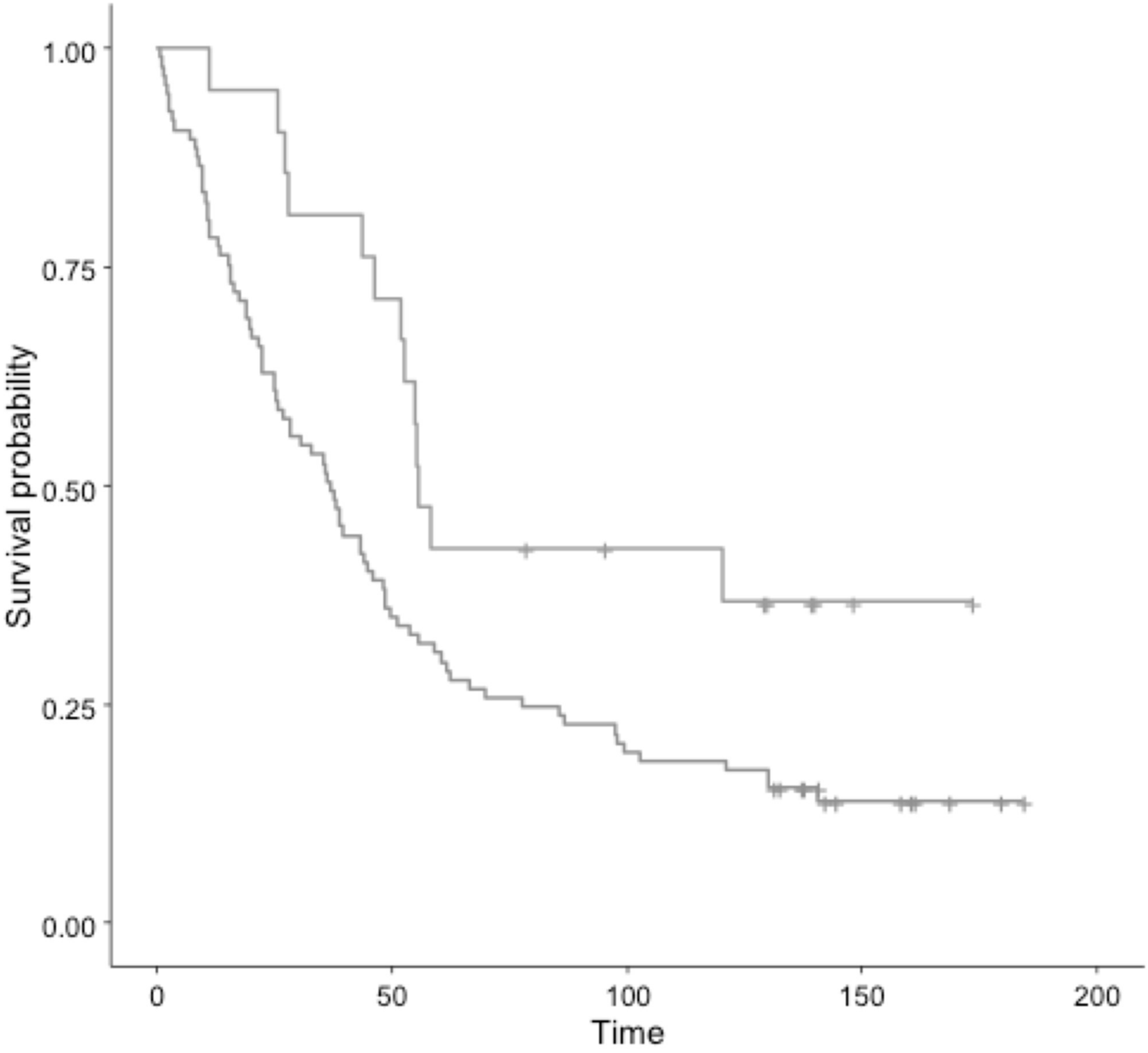

Figure S2. Mutations at the gene level in the groups with druggable targets

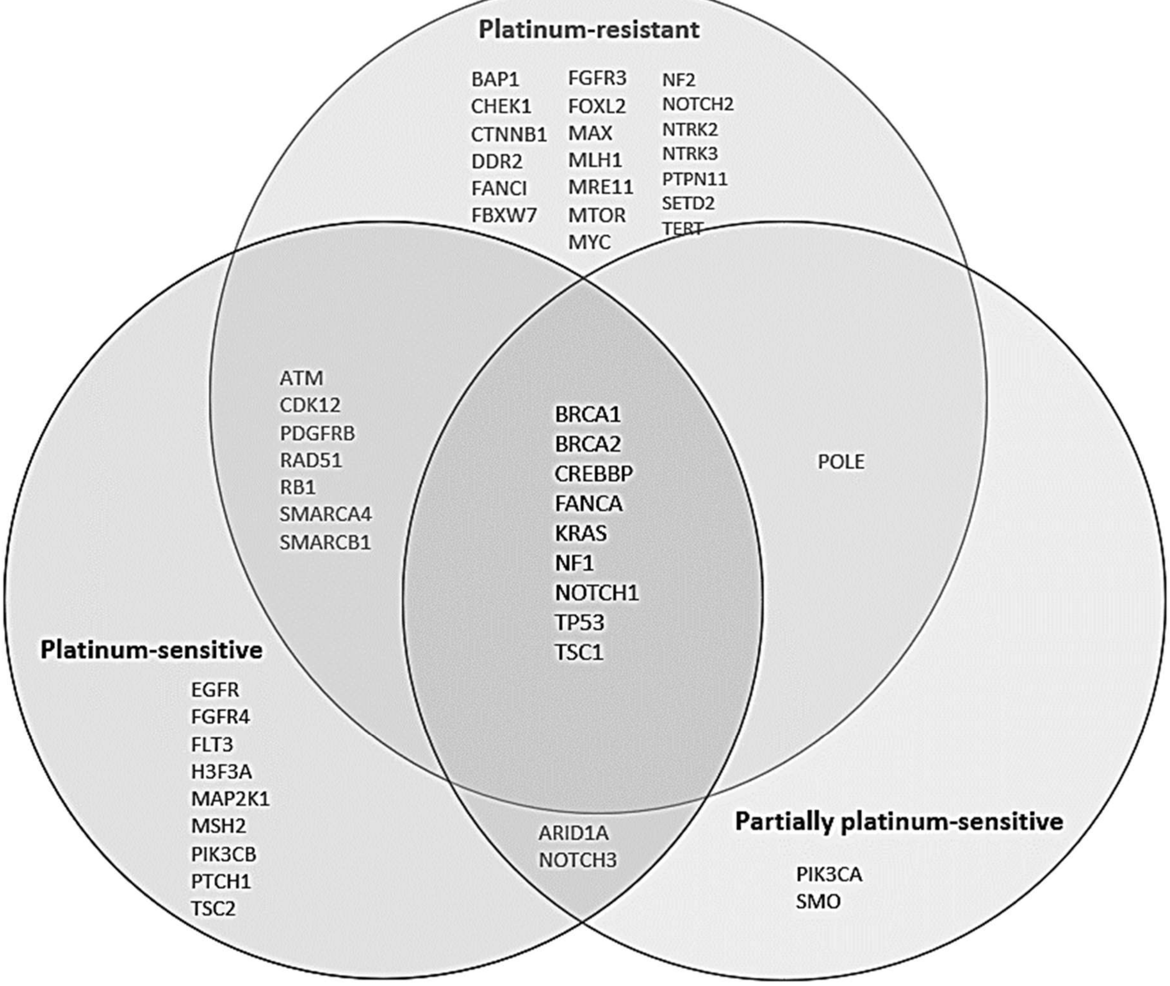

Supplement: Supplementary file 1 [file pharmaceuticals-15-01324-s001.zip › Supplementary material 1.pdf]
