# Supplementary material for: Potential Targeted Therapies in Ovarian Cancer
Source: Pharmaceuticals (Basel). 2022 Oct 26;15(11):1324. doi: 10.3390/ph15111324 (PMC9697427; doi:10.3390/ph15111324)
Supplement: Supplementary file 1 [file pharmaceuticals-15-01324-s001.zip › Supplementary material Table S3 .pdf]

SampleName	Genes	Amino Acid Change	Variant effect
600829	TP53	p.Phe338LeufsTer8	frameshiftDeletion
600829	BRCA1	p.Leu1216PhefsTer2	frameshiftDeletion
600830	TP53	p.Arg282Trp	missense
600832	TP53	p.Ile195SerfsTer52	frameshiftDeletion
600832	NF1	p.Val1753PhefsTer12	frameshiftDeletion
600832	NF2	p.Glu106GlyfsTer24	frameshiftInsertion
600833	TP53	p.Ser303AlafsTer42	frameshiftDeletion
600838	TP53	p.?	splice site
600840	TP53	p.Tyr220Cys	missense
600844	MAP2K1	p.Gln56ArgfsTer8	frameshiftDeletion
600844	TSC2	p.Glu1476Ter	nonsense
600846	NOTCH3	p.Gln1868Ter	nonsense
600846	SMARCB1	p.Gln198Ter	nonsense
600847	MLH1	p.Arg659Ter	nonsense
600847	NOTCH1	p.Gln1282Ter	nonsense
600851	BRCA2	p.Lys2909GlnfsTer16	frameshiftDeletion
600851	TP53	p.Tyr220Cys	missense
600852	MYC	p.Gln50Ter	nonsense
600852	POLE	p.Gln2217Ter	nonsense
600852	TP53	p.Gln144Ter	nonsense
600853	TP53	p.Arg213Ter	nonsense
600853	BRCA1	p.?	splice site
600854	TP53	p.Glu285Lys	missense
600856	TP53	p.?	splice site
600857	TP53	p.Arg248Trp	missense
600859	CREBBP	p.Gln2357Ter	nonsense
600859	TP53	p.Phe212SerfsTer3	frameshiftDeletion
600862	TP53	p.Val218GlyfsTer4	frameshiftInsertion
600863	TP53	p.?	splice site
600868	TP53	p.Arg335SerfsTer9	frameshiftDeletion
600870	TP53	p.?	splice site
600870	BRCA1	p.Asp1305AlafsTer2	frameshiftDeletion
600871	TP53	p.Ser241Cys	missense
600871	NF1	p.Glu19Ter	nonsense
600873	NOTCH1	p.Gln2398AsnfsTer109	frameshiftBlockSubstitution
600873	TP53	p.Ser241Cys	missense
600874	TSC1	p.?	splice site
600874	CREBBP	p.Lys2102Ter	nonsense
600874	TP53	p.Phe338LeufsTer7	frameshiftDeletion
600877	TP53	p.Arg337Cys	missense
600882	TP53	p.Ile195Thr	missense
600884	TP53	p.Arg175His	missense
600885	FBXW7	p.Arg479Gln	missense
600885	TP53	p.Arg273His	missense
600886	FGFR4	p.Trp655Ter	nonsense
600886	TP53	p.Arg273Leu	missense
600886	BRCA1	p.Arg1699Gln	missense
600887	TP53	p.Cys238Trp	missense
600909	NOTCH1	p.Leu1481AlafsTer90	frameshiftDeletion

600909	KRAS	p.Leu168Ter	nonsense
600909	CREBBP	p.Arg589MetfsTer10	frameshiftDeletion
600909	TP53	p.?	splice site
600912	SMO	p.Gln228Ter	nonsense
600912	TP53	p.Leu265Pro	missense
600913	TP53	p.Arg248Gln	missense
600917	TP53	p.Cys176Tyr	missense
600917	BRCA1	p.Ala1708Glu	missense
600918	TP53	p.Glu285Lys	missense
600921	ATM	p.Glu1751Ter	nonsense
600921	SMARCA4	p.Gln638Ter	nonsense
600926	TP53	p.Arg333ValfsTer12	frameshiftDeletion
600927	TP53	p.Ser215Ile	missense
600927	BRCA1	p.Glu1046Ter	nonsense
600929	TP53	p.Tyr234Cys	missense
600929	BRCA1	p.Gly1738Glu	missense
600932	TP53	p.Ser241Phe	missense
600933	TP53	p.Glu56LysfsTer67	frameshiftDeletion
600944	TP53	p.Ser94Ter	nonsense
600956	TP53	p.Tyr234Cys	missense
600956	BRCA1	p.Gln494Ter	nonsense
600957	TP53	p.His179Gln	missense
600957	CDK12	p.Ser301CysfsTer5	frameshiftDeletion
600962	NOTCH2	p.Trp865Ter	nonsense
600962	TSC1	p.?	splice site
600962	CHEK1	p.Gln318Ter	nonsense
600962	NTRK3	p.Gln159Ter	nonsense
600962	NF2	p.Phe507ThrfsTer7	frameshiftDeletion
600963	TP53	p.Val216Met	missense
600964	TP53	p.Cys141Tyr	missense
600967	TP53	p.Arg248Gln	missense
600968	TP53	p.Arg248Gln	missense
600970	TP53	p.Ser127Phe	missense
600971	TP53	p.Ser241Phe	missense
600973	TP53	p.Phe270Ser	missense
600973	BRCA1	p.Glu1134Ter	nonsense
600975	TP53	p.Val172LeufsTer2	frameshiftDeletion
600975	BRCA1	p.Gln1756ProfsTer74	frameshiftInsertion
600979	TP53	p.Glu224Ter	nonsense
600979	BRCA1	p.?	splice site
600979	SMARCA4	p.Glu1113Ter	nonsense
600981	TP53	p.Leu130SerfsTer40	frameshiftDeletion
600982	KRAS	p.Gly12Val	missense
600982	TP53	p.Arg196Ter	nonsense
600984	TP53	p.Glu286GlyfsTer19	frameshiftDeletion
600985	RB1	p.?	splice site
600985	TP53	p.Thr102AsnfsTer47	frameshiftInsertion
600989	TSC1	p.Lys121Asn	missense
600989	CREBBP	p.Arg1786His	missense
600989	TP53	p.Arg290AlafsTer55	frameshiftDeletion

600990	TP53	p.Met237Ile	missense
600991	BRCA2	p.Ser2219Ter	nonsense
600991	TP53	p.Ser261ArgfsTer2	frameshiftDeletion
600993	TP53	p.Arg273His	missense
600994	TP53	p.Asp42LysfsTer3	frameshiftInsertion
600996	NOTCH1	p.Gln1282Ter	nonsense
600996	RAD51	p.Arg194Ter	nonsense
600996	TP53	p.His168Arg	missense
600996	NF1	p.Trp696Ter	nonsense
600997	TP53	p.Asp259Tyr	missense
600999	NF1	p.?	splice site
600999	NF1	p.Trp696Ter	nonsense
601000	TP53	p.Arg175His	missense
601001	TP53	p.Arg248Gln	missense
601002	TP53	p.Arg175His	missense
601005	RB1	p.Leu550Ter	nonsense
601005	TP53	p.Arg248Gln	missense
601005	NF1	p.Leu2669Ter	nonsense
601007	TP53	p.Arg306Ter	nonsense
601009	TP53	p.Gln104Ter	nonsense
601010	TP53	p.His179Arg	missense
601010	NF1	p.Ser402Ter	nonsense
601015	TP53	p.Asp228LeufsTer20	frameshiftInsertion
601015	BRCA1	p.Arg1699Gln	missense
601017	TP53	p.Tyr163Cys	missense
601018	BRCA2	p.Trp2626Cys	missense
601018	TP53	p.Val157Phe	missense
601021	BRCA1	p.Glu23ValfsTer17	frameshiftDeletion
601022	TP53	p.?	splice site
601118	TP53	p.Arg273His	missense
601121	TP53	p.?	splice site
600776	TP53	p.Glu286Ter	nonsense
600778	TP53	p.Glu224Ter	nonsense
600780	ARID1A	p.Gln1127Ter	nonsense
600780	CREBBP	p.Asn1789ThrfsTer10	frameshiftDeletion
600784	TP53	p.Asp281Asn	missense
600804	KRAS	p.Gly12Arg	missense
600806	TP53	p.Tyr205Cys	missense
600809	PIK3CA	p.Glu542Lys	missense
600809	FANCA	p.Glu288Ter	nonsense
600809	TP53	p.?	splice site
600811	PDGFRB	p.Gln599Ter	nonsense
600811	CDK12	p.Gln602Ter	nonsense
600814	TP53	p.Gly279Glu	missense
600817	TP53	p.Arg342Pro	missense
600817	BRCA1	p.Gln1756ProfsTer74	frameshiftInsertion
600818	TP53	p.His193Arg	missense
600819	TP53	p.Arg175His	missense
600821	TP53	p.Cys141Tyr	missense
600821	NF1	p.Trp696Ter	nonsense

600824	TP53	p.Gly244Cys	missense
600828	NOTCH1	p.[Thr2375=;Gln2376Ter]	synonymous, nonsense
600828	POLE	p.Trp775Ter	nonsense
600828	TP53	p.Arg175His	missense
600831	TP53	p.?	splice site
600837	BAP1	p.Arg60Ter	nonsense
600837	TERT	p.Arg8Ter	nonsense
600848	BRCA2	p.Ser1741ThrfsTer35	frameshiftDeletion
600848	TP53	p.Ser149IlefsTer32	frameshiftInsertion
600875	TP53	p.Val216Met	missense
600875	SMARCB1	p.Trp321Ter	nonsense
600879	DDR2	p.Trp132Ter	nonsense
600879	TP53	p.His179Gln	missense
600880	MTOR	p.Trp1490Ter	nonsense
600880	NOTCH2,N	p.Gln143Ter	nonsense
600880	CTNNB1	p.Trp338Ter	nonsense
600880	SETD2	p.Gln2362Ter	nonsense
600880	BAP1	p.Gln277Ter	nonsense
600880	FGFR3	p.Trp685Ter	nonsense
600880	NTRK2	p.Gln316Ter	nonsense
600880	TSC1	p.?	splice site
600880	PTPN11	p.Gln506Ter	nonsense
600880	POLE	p.Trp410Ter	nonsense
600880	BRCA2	p.Trp3106Ter	nonsense
600880	LOC10050(	p.Arg33Ter	missense
600880	RAD51	p.Glu259Lys	missense
600880	FANCI	p.Gln625Ter	nonsense
600880	CREBBP	p.Gln207Ter	nonsense
600880	FANCA	p.Trp410Ter	nonsense
600880	TP53	p.Arg213Gln	missense
600880	NF1	p.Gln1255Ter	nonsense
600881	FOXL2	p.Trp98Ter	nonsense
600881	TP53	p.Leu264TyrfsTer81	frameshiftDeletion
600910	TP53	p.Tyr220Cys	missense
600931	SLX4	p.Trp1514Ter	nonsense
600931	TP53	p.Leu93ArgfsTer30	frameshiftDeletion
600935	ARID1A	p.Trp1973Ter	nonsense
600935	H3F3A,H3F	p.Gln20Ter	nonsense
600935	MSH2	p.Gln324Ter	nonsense
600935	MSH2	p.Trp345Ter	nonsense
600935	MSH2	p.Gln413Ter	nonsense
600935	PIK3CB	p.Arg149Ter	nonsense
600935	PDGFRB	p.Arg987Trp	missense
600935	EGFR	p.Ala289Thr	missense
600935	PTCH1	p.Gln1020Ter	nonsense
600935	PTCH1	p.Gln242Ter	nonsense
600935	NOTCH1	p.Gln2395Ter	nonsense
600935	ATM	p.Arg2598Ter	nonsense
600935	FLT3	p.Asp835Asn	missense
600935	CREBBP	p.Gln1259Ter	nonsense

600935	FANCA,ZNI	p.?, p.?	splice site, splice site
600935	TP53	p.Glu294Ter	nonsense
600935	BRCA1	p.?	splice site
600960	TP53	p.Gln192Ter	nonsense
601019	MRE11	p.Trp210Ter	nonsense
601019	CREBBP	p.Arg1103Ter	nonsense
601019	TP53	p.?	splice site
600781	CREBBP	p.?	splice site
600781	FANCA	p.Gln930Ter	nonsense
600781	TP53	p.Glu286Lys	missense
600781	BRCA1	p.Asn609IlefsTer3	frameshiftDeletion
600782	ARID1A	p.Gln1499Ter	nonsense
600782	TP53	p.His179Gln	missense
600782	NF1	p.Glu648Ter	nonsense
600782	NOTCH3	p.Glu1572AspfsTer16	frameshiftInsertion
600783	TP53	p.Arg175His	missense
600813	TP53	p.Trp91Ter	nonsense
600822	TP53	p.Gly245Cys	missense
600837	TP53	p.Pro177Arg	missense
601006	TP53	p.Pro153AlafsTer28	frameshiftInsertion
600811	TP53	p.Pro151Ser	missense
600812	TP53	p.Lys132Arg	missense
600827	TP53	p.Leu252del	nonframeshiftDeletion
600836	TP53	p.Met160_Ala161del	nonframeshiftDeletion
600876	TP53	p.Arg267Pro	missense
600977	TP53	p.?	splice site
600823	TP53	p.Leu265_Gly266del	nonframeshiftDeletion

Paste to varsome	Pathogenicity
chr17-7574011-TCG-T	P
chr17-41243899-ATA-A	P
chr17-7577094-G-A	LP/P
chr17-7578266-TA-T	P
chr17-29653253-AC-A	P
chr22-30035153-T-TG	P
chr17-7577031-TC-T	P
chr17-7577156-C-A	LP
chr17-7578190-T-C	P
chr15-66727447-AC-A	P
chr16-2134649-G-T	P
chr19-15276663-G-A	P
chr22-24145573-C-T	P
chr3-37090086-C-T	P
chr9-139401225-G-A	P
chr13-32950896-GTGAAGAA-G	P
chr17-7578190-T-C	P
chr8-128750611-C-T	P
chr12-133202239-G-A	P
chr17-7578500-G-A	P
chr17-7578212-G-A	P
chr17-41222944-C-T	P
chr17-7577085-C-T	LP/P
chr17-7574034-C-T	P
chr17-7577539-G-A	P
chr16-3777979-G-A	LP
chr17-7578212-GAA-G	P
chr17-7578198-C-CA	P
chr17-7576928-T-C	LP/P
chr17-7574021-CACGC-C	P
chr17-7577496-GACCTG-G	LP
chr17-41243633-GT-G	P
chr17-7577559-G-C	LP
chr17-29422382-G-T	P
chr9-139390997-CTG-CATT	P
chr17-7577559-G-C	LP
chr9-135796749-C-T	P
chr16-3778744-T-A	P
chr17-7574012-CG-C	P
chr17-7574018-G-A	LP/P
chr17-7578265-A-G	P
chr17-7578406-C-T	P
chr4-153247366-C-T	LP
chr17-7577120-C-T	P
chr5-176523308-G-A	P
chr17-7577120-C-A	P
chr17-41215947-C-T	P
chr17-7577567-A-C	P
chr9-139399881-GTTCTTCCAGGGGTCATTGAAGTTGAGGG-G	P

chr12-25368442-A-T	P
chr16-3830772-TGTTCGTGCCAGCCTTTCCT-T	P
chr17-7577156-C-A	LP
chr7-128845188-C-T	P
chr17-7577144-A-G	LP/P
chr17-7577538-C-T	P
chr17-7578403-C-T	LP/P
chr17-41215920-G-T	P
chr17-7577085-C-T	LP/P
chr11-108172448-G-T	P
chr19-11113804-C-T	P
chr17-7574029-CG-C	P
chr17-7578205-C-A	P
chr17-41244412-C-A	P
chr17-7577580-T-C	LP/P
chr17-41209133-C-T	LP/P
chr17-7577559-G-A	LP
chr17-7579520-TC-T	P
chr17-7579406-G-T	P
chr17-7577580-T-C	LP/P
chr17-41246068-G-A	P
chr17-7578393-A-T	P
chr17-37619224-ATC-A	LP
chr1-120491634-C-T	P
chr9-135796749-C-T	P
chr11-125514014-C-T	P
chr15-88680782-G-A	P
chr22-30074252-TGTCT-T	P
chr17-7578203-C-T	LP
chr17-7578508-C-T	P
chr17-7577538-C-T	P
chr17-7577538-C-T	P
chr17-7578550-G-A	LP
chr17-7577559-G-A	LP
chr17-7577129-A-G	LP/P
chr17-41244148-C-A	P
chr17-7578415-AC-A	P
chr17-41209079-T-TG	P
chr17-7578179-C-A	P
chr17-41215969-C-A	P
chr19-11138581-G-T	P
chr17-7578541-AG-A	P
chr12-25398284-C-A	P
chr17-7578263-G-A	P
chr17-7577079-CTT-C	P
chr13-48941740-G-A	LP/P
chr17-7579382-G-GT	P
chr9-135800974-C-G	P
chr16-3779691-C-T	LP
chr17-7577069-CG-C	P

chr17-7577570-C-T	P
chr13-32915148-C-A	P
chr17-7577153-CCA-C	P
chr17-7577120-C-T	P
chr17-7579563-C-CTT	P
chr9-139401225-G-A	P
chr15-41020955-C-T	P
chr17-7578427-T-C	P
chr17-29553538-G-A	P
chr17-7577506-C-A	LP
chr17-29550461-G-A	LP
chr17-29553538-G-A	P
chr17-7578406-C-T	P
chr17-7577538-C-T	P
chr17-7578406-C-T	P
chr13-48955532-TTAG-T	P
chr17-7577538-C-T	P
chr17-29685533-T-G	P
chr17-7577022-G-A	P
chr17-7579377-G-A	P
chr17-7578394-T-C	LP
chr17-29528448-C-G	P
chr17-7577599-C-CAA	P
chr17-41215947-C-T	P
chr17-7578442-T-C	P
chr13-32936732-G-C	P
chr17-7578461-C-A	LP
chr17-41276044-ACT-A	P
chr17-7577610-T-G	LP
chr17-7577120-C-T	P
chr17-7576852-C-T	P
chr17-7577082-C-CA	P
chr17-7578179-C-A	P
chr1-27097790-C-T	P
chr16-3779683-TG-T	P
chr17-7577097-C-T	P
chr12-25398285-C-G	P
chr17-7578235-T-C	LP
chr3-178936082-G-A	LP/P
chr16-89865605-C-A	P
chr17-7577610-T-C	P
chr5-149505020-G-A	P
chr17-37627889-C-T	LP
chr17-7577102-C-T	LP/P
chr17-7574002-C-G	LP/P
chr17-41209079-T-TG	P
chr17-7578271-T-C	LP/P
chr17-7578406-C-T	P
chr17-7578508-C-T	P
chr17-29553538-G-A	P

chr17-7577551-C-A	LP
chr9-139391065-GG-AA	P
chr12-133242032-C-T	P
chr17-7578406-C-T	P
chr17-7578555-C-T	LP/P
chr3-52442567-G-A	P
chr5-1295083-G-A	P
chr13-32913710-TTAAG-T	P
chr17-7578485-A-AT	P
chr17-7578203-C-T	LP
chr22-24167579-G-A	P
chr1-162724623-G-A	P
chr17-7578393-A-T	P
chr1-11217209-C-T	P
chr1-120539944-G-A	P
chr3-41268775-G-A	P
chr3-47087991-G-A	P
chr3-52439883-G-A	P
chr4-1808296-G-A	P
chr9-87342661-C-T	P
chr9-135796749-C-T	P
chr12-112926896-C-T	P
chr12-133250291-C-T	P
chr13-32968887-G-A	P
chr14-65560500-G-A	P
chr15-41021830-G-A	LP
chr15-89833495-C-T	P
chr16-3900477-G-A	P
chr16-89857940-C-T	P
chr17-7578211-C-T	P
chr17-29562683-C-T	P
chr3-138665271-C-T	P
chr17-7577147-AG-A	P
chr17-7578190-T-C	P
chr16-3639097-C-T	P
chr17-7579408-CA-C	P
chr1-27106307-G-A	P
chr1-226252110-C-T	P
chr2-47643462-C-T	P
chr2-47643526-G-A	P
chr2-47657041-C-T	P
chr3-138461576-G-A	LP
chr5-149497359-G-A	P
chr7-55221821-G-A	LP
chr9-98220405-G-A	P
chr9-98244253-G-A	P
chr9-139391008-G-A	P
chr11-108203492-C-T	P
chr13-28592642-C-T	P
chr16-3801731-G-A	P

chr16-89805698-C-T	P
chr17-7577058-C-A	P
chr17-41209153-C-T	P
chr17-7578275-G-A	P
chr11-94209485-C-T	P
chr16-3808917-G-A	P
chr17-7576852-C-T	P
chr16-3929832-C-T	LP
chr16-89828421-G-A	P
chr17-7577082-C-T	P
chr17-41245721-AT-A	P
chr1-27101213-C-T	P
chr17-7578393-A-T	P
chr17-29552209-G-T	P
chr19-15284899-C-CG	P
chr17-7578406-C-T	P
chr17-7579414-C-T	P
chr17-7577548-C-A	P
chr17-7578400-G-C	LP/P
chr17-7578474-C-CG	P
chr17-7578479-G-A	LP/P
chr17-7578535-T-C	P
chr17-7577525-GAGG-G	P
chr17-7578444-GATGGCC-G	P
chr17-7577138-C-G	P
chr17-7577599-CAGAGCCAACCTAGGAGATAACACAGGC-C	P
chr17-7577138-CGTCCCA-C	P
